# Prediction of induction motor faults using machine learning

**DOI:** 10.1016/j.heliyon.2024.e41493

**Published:** 2024-12-25

**Authors:** Ademola Abdulkareem, Tochukwu Anyim, Olawale Popoola, John Abubakar, Agbetuyi Ayoade

**Affiliations:** a Electrical and Information Engineering Department, Covenant University, P.M.B 1023, Ota, 112212, Ogun State, Nigeria; b Electrical Engineering Department, Centre for Energy and Electric Power, Faculty of Engineering and the Built Environment, Tshwane University of Technology, Nigeria; c Department of Computer Science and Engineering, University of Bologna, BO, Italy

**Keywords:** Artificial neural network classifier, Decision tree classifier, Random Forest classifier, k-NN classifier, Induction motors, Predictive maintenance

## Abstract

Unplanned downtime in industrial sectors presents significant challenges, impacting both production efficiency and profitability. To tackle this issue, companies are actively working towards optimizing their operations and reducing disruptions that hinder their ability to meet customer demands and financial goals. Predictive maintenance, utilizing advanced technologies like data analytics, machine learning, and IoT devices, offers real-time equipment data monitoring and analysis. This research study centers on the development of a versatile machine-learning model for predicting faults in induction motors within industrial environments. Implementing such a model can enable proactive maintenance, ultimately leading to decreased downtime in industrial operations. The study involved the acquisition of a dataset comprising healthy and faulty conditions of four 3-phase induction motors, along with relevant features for fault prediction. Multiple machine learning algorithms were trained using this dataset, exhibiting promising performance. The Random Forest (RF) model achieved the highest accuracy at 0.91, closely followed by the Artificial Neural Network (ANN) and k-nearest Neighbors (k-NN) models, both achieving an accuracy of 0.9. Meanwhile, the Decision Tree (DT) model showed the lowest accuracy at 0.89. Further model evaluation was carried out using a confusion matrix, which provided a detailed breakdown of the models' performance for each class, revealing the number of correctly and incorrectly classified induction motor conditions. The results from the confusion matrix indicate that the models effectively classified the various states and conditions of the induction motors. To enhance model performance in future work, potential avenues include refining the ANN and RF models, exploring transfer learning or ensemble methods, and incorporating diverse datasets to improve generalization.

## 1 Introduction

In industrial settings, the role of induction motors, particularly three-phase induction motors, is of paramount importance. These motors serve as critical components that are responsible for supplying power to a wide array of machinery and equipment [1]. Industries such as manufacturing, the oil and gas sector, mining, and transportation heavily rely on these motors. Their versatility is evident in their ability to power pumps, compressors, conveyors, fans, and a multitude of other industrial equipment. This versatility arises from their unique capability to efficiently convert electrical energy into mechanical energy, making them indispensable in the industrial landscape [2,3]. The prevalence of induction motors in industrial and commercial applications can be attributed to several key advantages. Firstly, these motors are renowned for their durability, which allows them to withstand the demanding conditions of industrial operations. They are also highly reliable, ensuring continuous operation of critical machinery [4]. Additionally, their affordability makes them an attractive choice for various applications. Systems that require both intermittent and constant motion, such as pumps, fans, compressors, conveyors, and machine tools, often employ these motors.

**Fig. 1:**
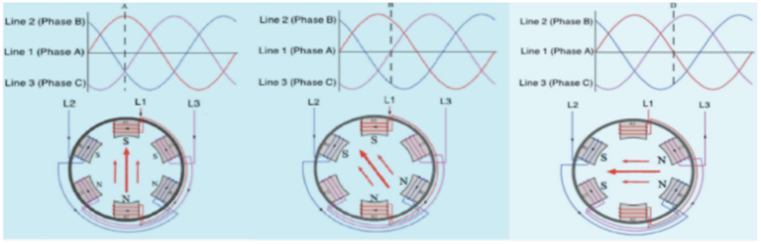
Rotating Magnetic Field generated in the Stator by AC supply [6].

To understand how induction motors operate, it's essential to grasp the principle of electromagnetic induction, which serves as the foundation of their functionality [[5], [6], [7]]. These motors consist of two primary components: a revolving rotor and a stationary stator. The stator contains a set of windings that are energized with alternating current (AC). This AC current creates a magnetic field that rotates, as visually represented in Fig. 1 [8]. The interaction of this rotating magnetic field with the rotor windings induces a current within the rotor. This interaction between the stator and rotor magnetic fields results in the generation of torque, enabling the motor to rotate [9]. provides a comprehensive explanation of this operating principle. While induction motors offer numerous advantages, they are not without their limitations. For instance, their speed control capabilities are somewhat constrained when compared to other types of motors [10]. This limitation can pose challenges in applications where precise speed control is required. Additionally, induction motors may exhibit a lower power factor at light loads, which can affect the overall efficiency of the system [11]. Moreover, these motors are susceptible to electrical and mechanical faults. If these issues are not promptly addressed through proper maintenance and monitoring [12], they can lead to compromised motor performance or even motor failure, potentially causing costly disruptions in industrial operations.

Induction motors can experience various types of faults, leading to reduced efficiency, decreased production, and higher maintenance expenses [13]. Common faults seen or experienced in induction motor is bearing faults, stator winding fault, voltage imbalance, rotor fault, shaft misalignment, overheating, etc. The impact of faults in induction motors can be significant, leading to reduced motor performance and decreased production efficiency.

Various research has been carried out on how to identify or detect these faults in induction motors. Traditionally, faults in induction motors were detected through direct observations such as visual inspection, listening for abnormal sounds, and touch for unusual vibration or temperature. However, these methods have limitations and are not always reliable in detecting faults.

Over time, the field of fault detection in induction motors has witnessed significant advancements [14], with researchers exploring novel approaches and methodologies. One prominent avenue of research involves the application of machine learning techniques in fault detection for induction motors. Machine learning and AI-based methods have emerged as powerful tools, showcasing their ability to outperform traditional techniques in terms of accuracy, particularly when dealing with scenarios involving multiple faults or complex fault patterns. This ongoing evolution in research reflects the growing recognition that harnessing the capabilities of machine learning and AI offers a promising avenue for enhancing the accuracy and effectiveness of fault detection in induction motors. As such, the field continues to be a dynamic and evolving area of study, driven by the ever-increasing demand for improved motor reliability and operational efficiency in industrial contexts.

The objective of this paper is to employ machine learning techniques for the prediction of induction motor faults, with the aim of improving operational efficiency. Through the utilization of an innovative machine learning framework tailored specifically for induction motor fault prediction, this research seeks to overcome the constraints of conventional fault prediction methods which include the lack adaptability and scalability of the models. By addressing these limitations, we aim to enhance our understanding of induction motor performance, ultimately leading to more efficient and reliable industrial processes.

Gangsar and Tiwara (2017) embarked on a similar journey, utilizing multiclass support vector machine (MSVM) methods with a radial basis function kernel (RBF) and a one-versus-one (OVO) multiclass technique. Their research focused on eliminating redundant information and mitigating dimensionality challenges by deriving three statistical characteristics (r, v, and j) from time domain vibration and current signals. The proposed diagnostic approach exhibited commendable performance, especially under consistent speed and load conditions [15].

Khanjani and M Ezoji (2021) introduced a novel approach for electrical fault detection in three-phase induction motors. They harnessed deep neural networks to extract information from thermal images of these motors, employing SIFT-based key point matching and transfer learning to enhance model accuracy. Impressively, their method achieved a detection accuracy of 98.5 % with a rapid processing speed of 0.2 s [16]. Gonçalves, Fruett et al. (2021) proposed a classification and detection model for centrifugal pumps using Markov parameters derived from vibration data. Their approach incorporated Canonical Correlation Analysis (CCA) and distance-based criteria, along with the minimal volume ellipsoids technique, resulting in high accuracy in both speed and fault detection. In the aspect of bearing fault diagnosis [17]. Siyu Shao, Ruqiang Yan et al. (2020) conducted an analysis based on Convolutional Neural Networks (CNN). Their focus was on identifying bearing faults, and their approach showcased high accuracy, rapid processing, and time-saving benefits [18].

Sunal, C. E., Dyo, V. et al. (2020) reviewed fault detection in centrifugal pump induction motors, emphasizing the utilization of motor current signature analysis and machine learning techniques. Their research identified CNN, RF, and MLP as the most successful methods for classifying faults in centrifugal pumps, consistently achieving success rates exceeding 95 % [19]. Additionally, Kavana and M. Neethi (2018) developed a predictive model for induction motors employing machine learning. Their study introduced an accelerated artificial neural network model for detecting common electrical faults. The proposed model reported an impressive accuracy of 98.5 % in detecting induction motor faults [20].

Cunha, R. G. C. et al. (2021) adopted a methodology that integrated the discrete wavelet transform (DWT) for multiresolution analysis (MRA), statistical features, and machine learning techniques. Their results demonstrated an exceptional classification accuracy of 99.23 % in distinguishing between normal and defective conditions [21]. Toma, R. N. et al. (2020) proposed a hybrid data-driven approach for bearing fault diagnosis in induction motors, incorporating statistical features, genetic algorithms (GA), and machine learning models. Their approach yielded a remarkably high accuracy rate of over 99.5 % in bearing fault diagnosis [22].

Kavana, V., & Neethi, M. (2018) introduced a forward artificial neural network model trained using three-phase voltages and currents as input variables. Their model exhibited a high accuracy rate of 98.5 % in detecting and classifying faults [23]. Abid, F. B. et al. (2019) developed a novel deep learning architecture named Deep-SincNet for diagnosing faults in induction motors. This architecture achieved an outstanding accuracy rate of 99.93 % and proved to be a cost-effective solution for induction motor diagnosis [24].

Collectively, these studies underscore the application of machine learning in the domain of induction motor fault detection and analysis. However, several gaps have been identified in the existing literature. These gaps include a lack of generalization, where models are often tailored to specific individual motors, limiting their practicality in industrial settings with multiple motors. This limitation necessitates extensive training for each unique motor condition, hindering widespread implementation. Additionally, scalability challenges arise when implementing these models in scenarios involving numerous induction motors, and the substantial data and processing demands required for accurate results using machine learning algorithms pose practical barriers. Efforts to address these gaps will undoubtedly contribute to the development of more effective and practical fault prediction models for induction motors, thereby enhancing their applicability and utility in diverse industrial settings.

## 2 Materials and methods

In this section, the material and method deployed in this research will be explored. It explores the process involved in gathering and analyzing data from four unique induction motors, each exemplifying diverse operational scenarios, training, testing and the machine learning model used. In this study, a four three-phase induction motor was used. These motors were selected based on their specifications which can be seen in Table 1. From Tables 1 and it can be noted that the motor was chosen to represent a range of power and speed commonly used in industries.

**Table 1:** Inductor motor specifications.

Motor Tag	Motor 1	Motor 2	Motor 3	Motor 4
**Power (kW)**	0.75	2.2	4.0	2.2
**Voltage (V)**	3 phase 415V	3 phase 415V	3 phase 415V	3 phase 415V
**Speed (rpm)**	1720	1180	2855	1700

Various operating condition simulations were simulated in real-time. The real-time simulation was done to create a robust dataset. Cases like normal operating conditions, load imbalance, and bearing faults were simulated in real-time. The aim of these cases was to capture the diverse range of motor behaviour and fault scenarios. The vibration and temperature sensors recorded data for each simulated condition, yielding between 85,000 and 100,000 data entries per condition. This substantial number of data entries ensured a robust and representative dataset suitable for thorough analysis. By introducing and recording data from these simulated conditions, a comprehensive understanding of the motor's behaviour and fault patterns could be attained.

**Fig. 2:**
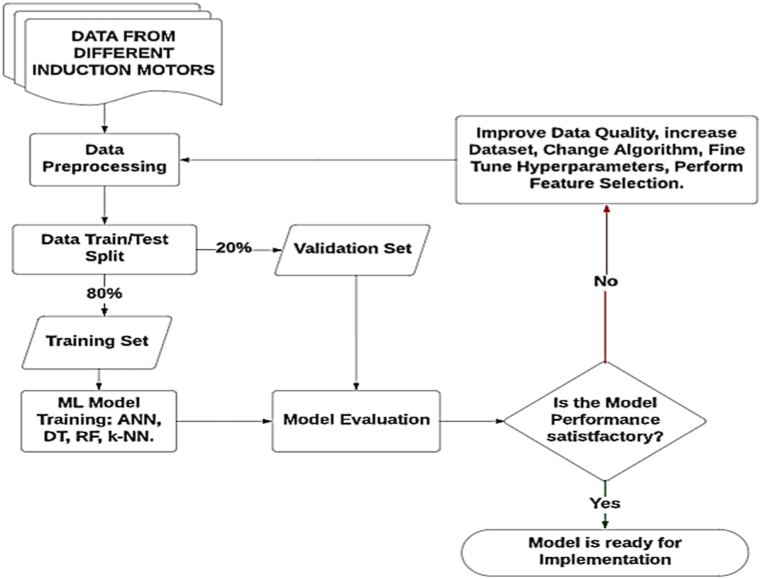
Flowchart of the Machine Learning Model training.

The flowchart of the entire system can be visualized in Fig. 2. Each of the steps will be discussed in the following section.

**Fig. 3:**
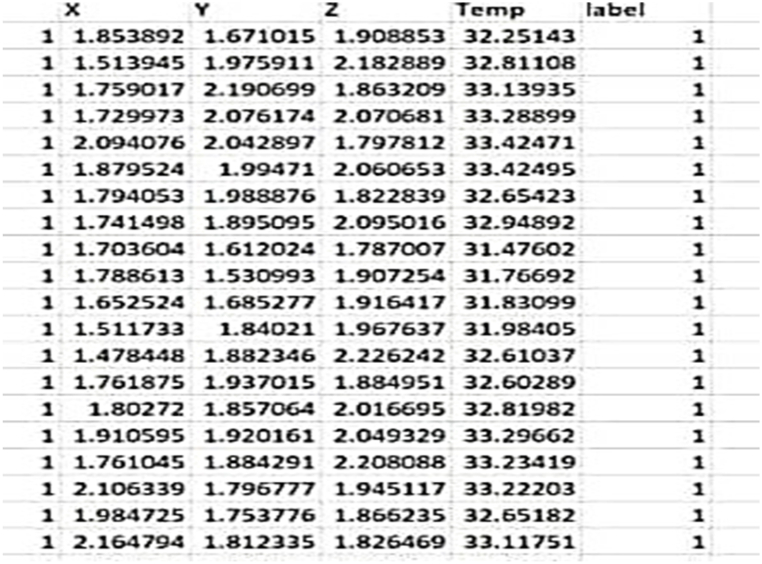
Data sample obtained.

**Data Collection:** The initial step in our methodology was the careful selection of four different induction motors, each with its unique specifications. We made sure to include motors that represented both normal and faulty conditions. This selection was crucial to cover a wide range of operating scenarios, which is essential for training a reliable machine learning model. During data collection, we ran these motors under various conditions namely healthy, load imbalance, drive end faulty bearing, and opposite drive end faulty bearing. Throughout these tests, we continuously monitored the motors, and our sensors recorded data related to vibration and temperature at regular intervals. This process provided us with a comprehensive dataset comprising of more than 1,600,000 data entries that was used to train and test our machine learning model. Fig. 3 shows a sample of the dataset set obtained.

**Data Preprocessing:** Data preprocessing is a crucial step in any machine learning project. It's all about making sure the data we use is of high quality and suitable for building predictive models. In our study, we took the raw data collected from the four different induction motors and subjected them to various preprocessing techniques to enhance the reliability of our dataset.

Fig. 3 depicts raw data points. The labels shown in the figure are assigned to individual data points for clarity in illustrating how the raw values vary under different conditions (Healthy, Load Imbalance, Drive-end Bearing Fault, and Opposite Drive-end Bearing Fault). This figure aims to provide a visual representation of the data prior to the application of feature extraction methods.

1. **Data Cleaning**: We first addressed any missing or inconsistent sensor readings. These missing values or inconsistencies could introduce bias into our predictions. We handled missing data by filling in the gaps through imputation and in cases where data couldn't be reasonably estimated, we removed those samples to maintain the integrity of our dataset.
2. **Outlier Detection**: Outliers are data points that significantly deviate from the majority of the data. They can distort the learning process of our machine learning models. To identify and deal with outliers, we employed methods like the Z-score. Outliers were either corrected to ensure that our models could learn effectively.
3. **Feature Extraction**: To represent fault patterns in induction motors effectively, we carefully selected informative and relevant features from the vibration data. These features included statistical and time-domain measures like mean, standard deviation, Root Mean Square (RMS), and variance, as well as frequency-domain features obtained through Fourier Transform.
4. **Normalization and Scaling**: It's crucial to ensure that all the features we use have a similar scale and range. If not, features with larger magnitudes can dominate certain machine learning algorithms. We applied normalization and scaling techniques to prevent this issue, ensuring that our models would perform well. We used a Min-Max normalization technique in this study.

**Data Splitting:** After preprocessing, we divided our dataset into two parts: the training set of 80 per cent and the test set of 20 per cent. The training set was used to train our machine learning models, allowing them to learn from the data. The test set was reserved for evaluating how well our models could perform on new, unseen data. By following these preprocessing steps, we ensured that our models could effectively capture complex patterns related to induction motor faults, ultimately leading to improved predictive performance.

**Machine Learning Algorithms Selection and Training**: In this phase of our study, we chose and trained four distinct machine learning algorithms to work with our data. These algorithms were selected for their effectiveness in classification tasks and their past success in predictive maintenance applications. The selected machine learning algorithms offer diverse capabilities suited for fault prediction in induction motors. Decision Trees provide transparency and interpretable rules, while Random Forests aggregate predictions from multiple trees for improved accuracy and generalization. k-Nearest Neighbors leverages proximity for prediction, and Artificial Neural Networks can model complex relationships in the data. These algorithms have demonstrated success in predictive maintenance applications and are suitable for handling the complexity of induction motor fault prediction tasks.

Here's an overview of each algorithm.

1. **Decision Tree**: The Decision Tree algorithm aims to create a tree-like structure that optimizes information gain or minimizes impurity at each level. Impurity is measured using two methods: Entropy and the Gini index. Entropy quantifies the impurity of a node's class distribution, while the Gini index measures the probability of misclassifying a randomly chosen element from a node. The mathematical equation for these two methods can be seen in equation (1) and equation (2).

(1) $$\text{Entropy}\;\left(S\right)=\sum_{i=1}^{c}-p_{i}\;\log_{2}p_{i}$$

(2) $$\text{Gini}\;\text{index}\;\left(G\right)=1-\sum_{i=1}^{c}{\left(p_{i}\right)}^{2}$$

The Decision Tree Classifier (DecisionTreeClassifier) was used with its default hyperparameters, including the criterion set to 'gini' for measuring split quality, and the splitter set to 'best', which selects the optimal split at each node. The tree's max_depth was left as None, allowing it to grow until all leaves are pure or contain fewer than the minimum min_samples_split, which defaults to 2. Each leaf node requires at least 1 sample (min_samples_leaf = 1), and no random_state was specified, leaving randomness uncontrolled. Without further tuning, the tree was allowed to grow fully without depth restrictions.

2. **Random Forest**: Random Forest utilizes a technique called bagging to create multiple data subsets for training separate decision tree models. These decision trees are the fundamental models within the Random Forest. Each tree offers its prediction, and the final prediction is determined through majority voting. This ensemble approach enhances the model's accuracy and generalization. Equation (3) shows a mathematical model as regards the output.

(3) $$Y=\text{Majority}\;\text{Vote}\;\left(h_{1}\left(x\right),h_{2}\left(x\right),\ldots ,h_{n}\left(x\right)\right)$$

The Random Forest Classifier (RandomForestClassifier) was implemented with its default hyperparameters, including ′n_estimators' set to 100, meaning the model builds 100 decision trees, and ′criterion' set to ′'gini'′ to measure split quality using Gini impurity. With no restriction on ′max_depth', each tree was allowed to grow fully, requiring at least 2 samples to split a node (′min_samples_split = 2′) and 1 sample at each leaf node (′min_samples_leaf = 1′). Bootstrap sampling was enabled (′bootstrap = True'), allowing random sampling with replacement to improve robustness. Without hyperparameter tuning, the model efficiently produced satisfactory results for fault classification.

3. **k-Nearest Neighbors (k-NN):** The k-Nearest Neighbors algorithm makes predictions by calculating the distance between a new data point and existing data points. It commonly employs the Euclidean distance as the default metric, measuring the straight-line distance between points. Alternatively, the Manhattan distance, which sums the absolute differences in Cartesian coordinates, is used for high-dimensional data. The choice of distance metric can influence the algorithm's performance and the mathematical equations can be seen in equation (4) and equation 5

(4) $$\text{Euclidean}\;\text{distance}\;d\left(x,y\right)=\sqrt{\sum_{i=1}^{n}{\left(x_{i}-y_{i}\right)}^{2}}$$

(5) $$\text{Manhattan}\;\text{distance}\;d\left(x,y\right)=\sum_{i=1}^{n}\left|x_{i}-y_{i}\right|$$

The k-Nearest Neighbors Classifier (KNeighborsClassifier) was implemented with its default hyperparameters, including n_neighbors set to 5, meaning the classification of a point was determined by the majority vote of its 5 nearest neighbors, with all neighbors treated equally (weights = 'uniform'). The algorithm was set to 'auto', allowing scikit-learn to choose the most efficient algorithm based on the dataset size, and the leaf_size was 30 for BallTree or KDTree structures. The model used the Minkowski distance metric with p = 2, corresponding to Euclidean distance. These default settings provided reasonable performance without applying additional distance weighting or neighbor selection modifications.

4. **Artificial Neural Network (ANN):** Artificial Neural Networks consist of layers of interconnected neurons. Each neuron takes inputs, multiplies them by corresponding weights, adds a bias term, and passes the result through an activation function to introduce non-linearity and generate an output. This process is repeated across all neurons in the network, allowing ANNs to model complex relationships and make predictions based on input data. Equation 6 represents the mathematicsal equation of the output

(6) $$Y=f\left(b+\sum_{i=1}^{n}W_{i}X_{i}\right)$$

This ANN model employed several key hyperparameters effectively. The input layer used 5 features, and the first hidden layer had 100 neurons with the ReLU activation for non-linearity and efficiency. The output layer, matching the number of unique target classes, utilized softmax for multi-class classification. The Adam optimizer was selected to adjust learning rates dynamically, while sparse_categorical_crossentropy served as the loss function for integer-encoded labels. The model was trained over 42 epochs with a batch size of 5000, balancing computational efficiency and generalization, with performance monitored using accuracy.

To train these algorithms, we used the data we collected, which included vibration and temperature readings from the induction motors operating under different conditions. We utilized the Jupyter Notebook Integrated Development Environment (IDE) for tasks such as data preprocessing, algorithm training, and model evaluation. Our careful selection and training of these machine learning algorithms were driven by the aim to equip ourselves with the necessary tools for a comprehensive analysis and prediction of induction motor behavior and potential faults. It was imperative to ensure that the data we used was well-prepared for training the machine learning models. As part of this preparation, we divided the dataset into two subsets: a training set and a test set. This separation allowed us to accurately gauge the models' performance.

Each of the four machine learning algorithms underwent a training process using the training dataset. During this phase, the models absorbed the underlying patterns and relationships between the vibration and temperature data and the corresponding motor conditions (healthy or faulty).

**Model Evaluation:** Following the training phase, we evaluated the models using various performance metrics, including accuracy, precision, recall, and the F1-score. These metrics provided a comprehensive assessment of how well the models could predict motor behavior and detect faults.

1. **Accuracy:** We used the accuracy metric, as indicated by equation (7), to determine how well our models performed. It quantifies the proportion of correctly predicted cases within the testing dataset, considering both true positives (TP) and true negatives (TN), relative to all cases. A higher accuracy score signifies a greater level of correctness in the model's predictions.

(7) $$Accuracy=\frac{TP+TN}{\left(TP+TN+FP+FN\right)}$$

2. **Precision**: Precision, represented by equation (8), is another crucial metric. It calculates the percentage of accurate positive predictions generated by the model out of all the positive predictions it made. Precision is particularly valuable when false positives may have significant consequences.

(8) $$Precision=\frac{TP}{\left(TP+FP\right)}$$

3. **Recall:** Recall, also known as sensitivity or true positive rate, helps evaluate how well our models predict positive instances correctly. It measures the proportion of true positive predictions relative to the total actual positive instances in the testing dataset, as illustrated in equation (9).

(9) $$Recall=\frac{TP}{\left(TP+FN\right)}$$

4. **F1-Score**: The F1-score, an amalgamation of precision and recall, offers a holistic assessment of the predictive maintenance models. It balances precision and recall by taking their harmonic average. This metric is particularly useful when both positive and negative classes hold importance, as it provides an overall evaluation of model effectiveness. Equation (10) demonstrates the calculation of the F1-score.

(10) $$F1\;score=2\times \frac{\left(Precision\times Recall\right)}{\left(Precision+Recall\right)}$$

Another essential performance metric we utilized is the confusion matrix, which provides a detailed breakdown of our models' performance by categorizing predictions into true positives (TP), true negatives (TN), false positives (FP), and false negatives (FN). The confusion matrix was generated to evaluate the models' performance by comparing the true labels with the predicted labels for each classification task. In this study, the labels 0, 1, 2, 3 represent four distinct motor conditions: Label 0 corresponds to a healthy motor condition (normal operation), Label 1 represents load imbalance, Label 2 indicates a drive-end bearing fault, and Label 3 refers to an opposite drive-end bearing fault. The matrix provides a breakdown of correct and incorrect classifications for each motor condition. For example, if a true "Load imbalance" (Label 1) condition is misclassified as "Drive-end bearing fault" (Label 2), this misclassification will appear as an off-diagonal entry, while the diagonal entries represent correct classifications.

In addition to the confusion matrix, a classification report was generated to provide a more detailed performance analysis. In this report, the labels 1, 2, 3, 4 correspond to the same motor conditions, but the numbering starts from 1 instead of 0 for consistency with reporting tools.

Label 1: Healthy motor condition (same as Label 0 in the confusion matrix).
Label 2: Load imbalance (same as Label 1 in the confusion matrix).
Label 3: Drive-end bearing fault (same as Label 2 in the confusion matrix).
Label 4: Opposite drive-end bearing fault (same as Label 3 in the confusion matrix).

## 3 Result and discussion

In this section, we delve into the outcomes of our extensive analysis and the insights gained from the application of machine learning techniques to predict induction motor behavior and faults. The culmination of our research efforts has led to a wealth of valuable findings that shed light on the effectiveness of these models in industrial settings. We will present a detailed examination of the results, discussing accuracy, precision, recall, F1-score, and the confusion matrix to provide a comprehensive assessment of the predictive maintenance models.

The dataset used in this study consists of a total of 3,600,000 samples, which were collected from induction motors under four distinct operating conditions: Healthy Condition, Load Imbalance, Drive-end Bearing Fault, and Opposite Drive-end Bearing Fault. Each condition is represented by an equal number of samples, with 900,000 samples assigned to each condition. To ensure robust model training and evaluation, the dataset was split into 80 % training data and 20 % testing data. This corresponds to a total of 2,880,000 samples for training, distributed evenly across all four conditions, with 720,000 samples allocated to each: Healthy Condition, Load Imbalance, Drive-end Bearing Fault, and Opposite Drive-end Bearing Fault. Similarly, the testing set consists of 720,000 samples, with 180,000 samples representing each motor condition.

The dataset underwent comprehensive preprocessing to ensure its quality for machine learning model development. Initially, noise reduction techniques were applied to eliminate irrelevant data points and improve signal accuracy. Outliers were then identified and handled to prevent extreme values from distorting model performance. Following this, normalization and standardization were employed to scale and transform the data, ensuring uniformity across features and preventing any single feature from dominating.

### 3.1 Random Forest (RF) model

**Fig. 4:**
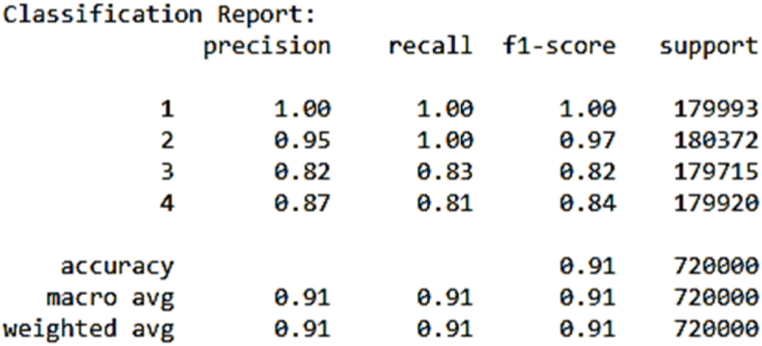
Random Forest model summary.

The Random Forest Classifier model leverages a combination of decision trees, offering superior robustness and accuracy compared to individual trees. As depicted in Fig. 4, the model's classification results following training were impressive. It achieved an impressive accuracy score of 0.91, signifying that it accurately classified 91 % of instances. This model's strength lies in its ability to aggregate predictions from multiple decision trees, resulting in heightened accuracy and enhanced generalization.

**Fig. 5:**
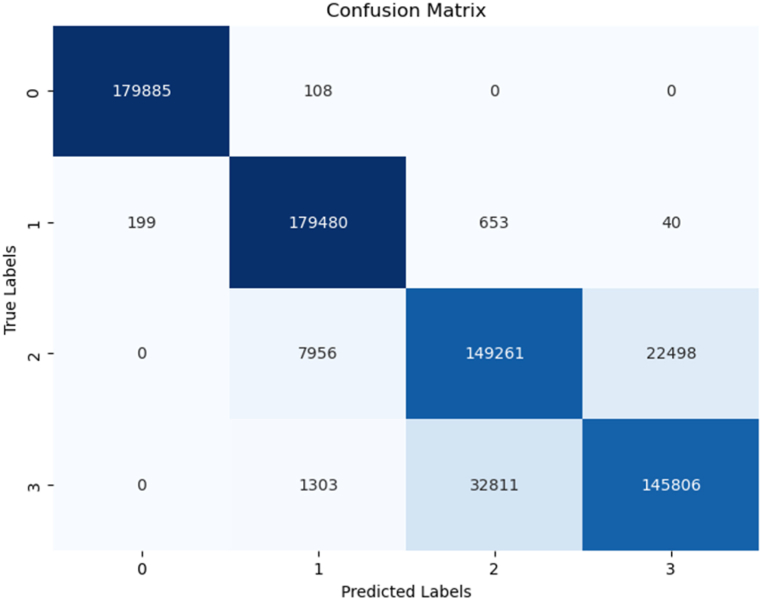
Random Forest confusion matrix.

Upon closer examination of Fig. 4, it becomes evident that the precision, recall, and F1-score for various samples exhibit near-perfect fits, ranging from 0.87 to 1.0. These metrics underscore the model's exceptional predictive performance. Fig. 5, the confusion matrix, offers a detailed insight into the prediction analysis. The diagonal elements represent accurate classifications, while the non-diagonal elements indicate instances of misclassification. Together, these findings provide a comprehensive view of the Random Forest Classifier model's effectiveness and its potential in real-world applications.

### 3.2 Artificial neural network (ANN) model

The Artificial Neural Network (ANN) model exhibited an exceptional performance, achieving an accuracy score of 0.9, which implies that it accurately classified 91 % of instances after completing 40 epochs of training. This noteworthy accomplishment underscores the model's proficiency in effectively categorizing the samples with a remarkable degree of precision. Furthermore, the ANN model excelled in capturing the intricate underlying patterns inherent in the dataset.

**Fig. 6:**
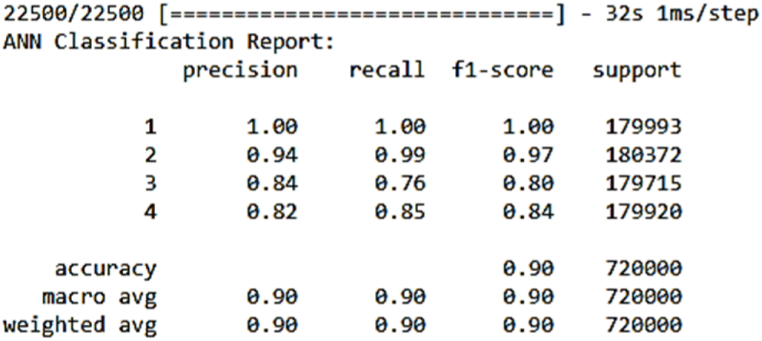
Ann's model summary.

**Fig. 7:**
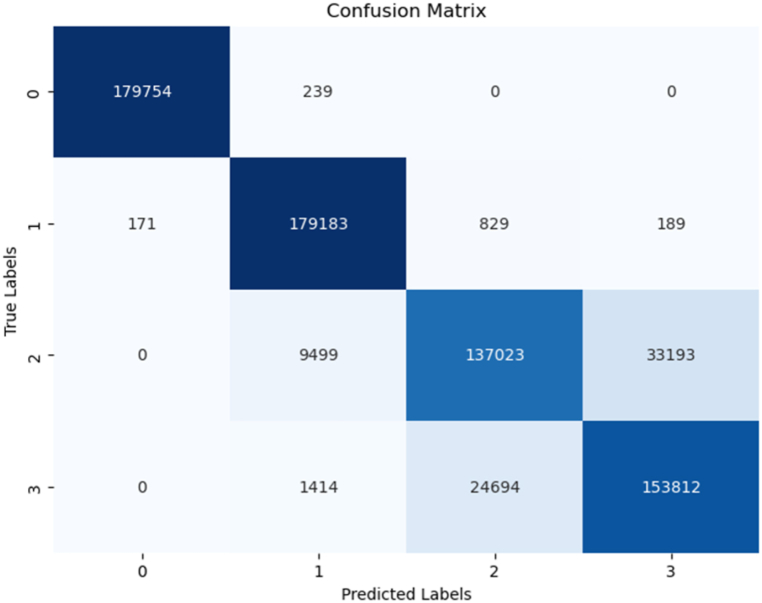
Ann's model confusion matrix.

The attainment of such a high accuracy score is a testament to the ANN model's formidable capabilities in comprehending intricate data relationships and making precise predictions, particularly when applied to the initial dataset. As depicted in Fig. 6, which provides a summary of the model after training, an in-depth review of precision, recall, and F1-score for each instance reveals impressive results, with scores ranging from 0.76 to 1.0. This diverse range of high scores highlights the model's ability to provide near-perfect fits across various instances. In Fig. 7, the confusion matrix offers a more detailed breakdown of the model's predictions, providing valuable insights into its performance. The ANN model's ability to achieve such accuracy, coupled with its consistent near-perfect fits across instances, positions it as a powerful tool for intricate pattern recognition and precise prediction in real-world applications.

#### 3.2.1 K-nearest Neighbor (k-NN) model

**Fig. 8:**
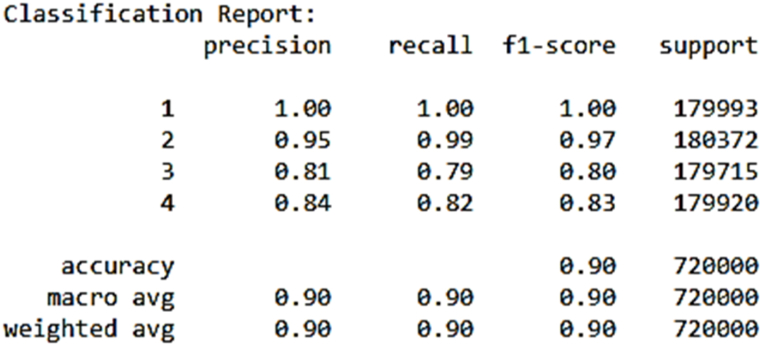
KNN's model summary.

**Fig. 9:**
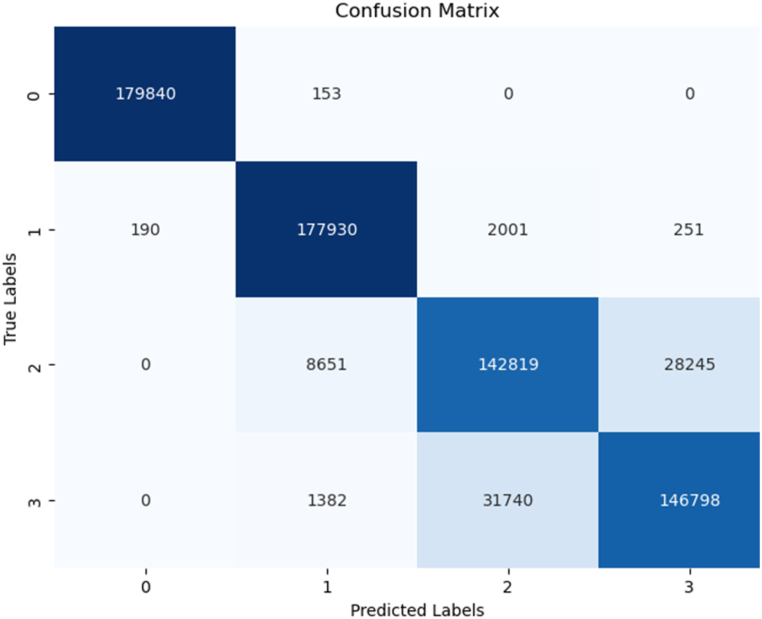
KNN's model confusion matrix.

The k-Nearest Neighbors (k-NN) model demonstrated an impressive accuracy rate of 90 %. The summary of the model is seen in Fig. 8 and the confusion matrix in Fig. 9. Notably, it exhibited high precision and recall values for both Class 1 and Class 2, highlighting its capability to effectively classify instances in these classes. While Classes 3 and 4 displayed slightly lower precision and recall scores, it's worth emphasizing that the F1 scores for all classes remained consistently above 0.80. This well-rounded performance underscores the k-NN model's effectiveness in accurately categorizing instances across multiple classes.

### 3.3 Decision tree (DT) model

The Decision Tree model delivered a commendable performance, achieving an accuracy score of 0.89, which corresponds to an accuracy rate of 89 %. While this score is slightly lower compared to the other models, it is important to note that Decision Trees are valued for their interpretability and simplicity. Although its predictive performance was marginally lower than that of the Random Forest (RF), Artificial Neural Network (ANN), and k-Nearest Neighbors (k-NN) models, it still demonstrated effectiveness in classification tasks.

**Fig. 10:**
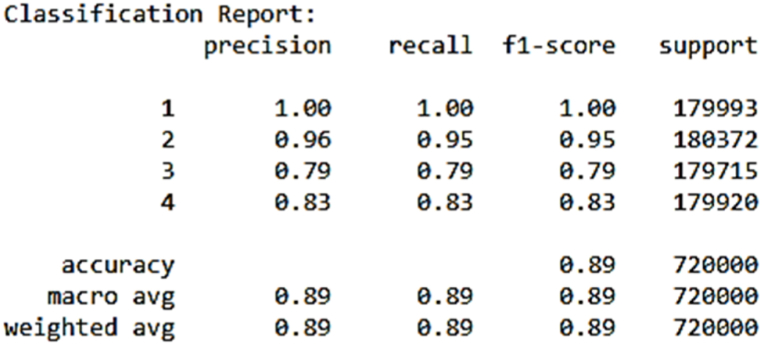
Decision tree model summary.

Fig. 10 presents a summary of the model's performance, offering insights into various metrics such as precision, recall, and F1-score. While the Decision Tree model didn't achieve the highest accuracy, it still showcased respectable performance across these key metrics.

**Fig. 11:**
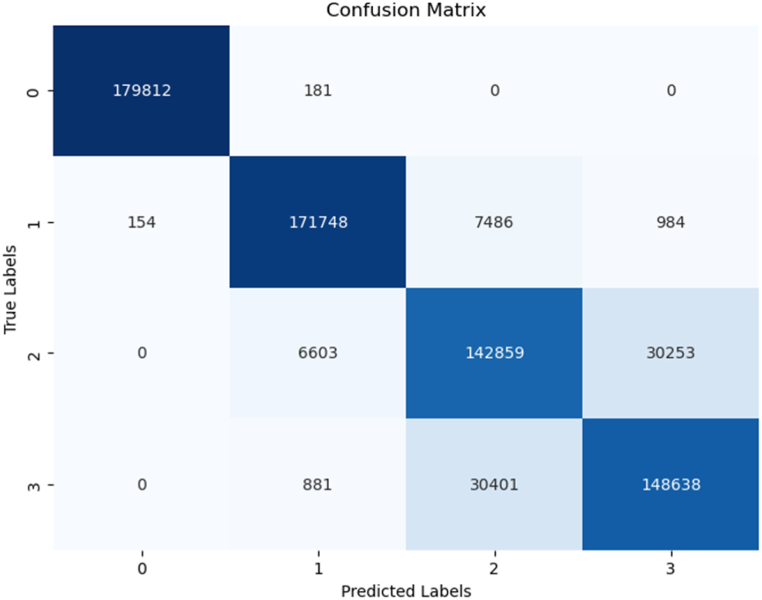
Decision tree confusion matrix.

Fig. 11, depicting the confusion matrix, provides a detailed breakdown of the model's predictions. Here, the diagonal elements represent instances correctly classified by the model, while the non-diagonal elements signify instances where misclassification occurred. This insight into the confusion matrix allows for a deeper understanding of the model's strengths and areas where it may benefit from further refinement.

Table 2, shows the summary of the results obtained from the developed machine learning models. The ANN and RF models were benchmark models in literature as presented in Table 3. The benchmark models primarily focused on the fault analysis and diagnosis of individual induction motors, whereas the developed models aimed to predict faults for multiple induction motors with varying specifications.

**Table 2:** Showing the performance of all the models.

ML Algorithm	Accuracy
RF classifier	**91 %**
ANN classifier	**90 %**
K-NN classifier	**90 %**
DT classifier	**89 %**

**Table 3:** Benchmarking with existing works.

Reference	ML Algorithm used	Component considered	Accuracy
Kavana and M Neethi (2018)	ANN classifier	Fault Analysis on 1 Induction Motor	97.5 %
Patel and VK Giri (2016)	RF classifier	classification of the mechanical fault on 1 induction motor	99.58 %
Wu et al. (2015)	ANN-Based Multi-classification	Bearing Fault Diagnosis on 1 Induction motor	94 %
Developed Model	ANN classifier	Fault prediction for 1 Induction Motor	98 %
Developed Model	ANN classifier	Fault prediction for 4 different IMs	90 %
Developed Model	RF classifier	Fault prediction for 4 different IMs	91 %

In the study conducted by Kavana and M Neethi (2018), an ANN classifier achieved an impressive accuracy of 97.5 % for fault analysis on a single induction motor. Patel and VK Giri (2016) utilized an RF classifier to achieve an outstanding accuracy of 99.58 % for the classification of mechanical faults on a single induction motor. Wu et al. (2015) employed an ANN-based multi-classification approach, achieving an accuracy of 94 % for bearing fault diagnosis on a single induction motor. When comparing these benchmark accuracies with the results of the developed ANN model, it becomes evident that the performance of the developed model for fault prediction on a single induction motor (98 %) is comparable and even slightly higher than the benchmark accuracies reported in the referenced works.

However, a noteworthy observation arose when the developed ANN model was applied to predict faults for four different induction motors, each with distinct specifications. In this scenario, the accuracy decreased to 90 %. Similarly, the developed RF model achieved an accuracy of 91 % for predicting faults in these four different induction motors. It is crucial to emphasize that the benchmark models in the referenced works were specifically tailored for fault analysis or diagnosis on individual induction motors. In contrast, the developed models were engineered to address a more intricate challenge, involving the prediction of faults for multiple motors with diverse specifications. Despite the heightened complexity associated with predicting faults for this broader scope, the developed ANN and RF models still managed to achieve commendable accuracy levels. The performance metrics for each machine learning model are as follows: Decision Tree: Accuracy = 89 % - Artificial Neural Network (ANN): Accuracy = 90 % - k-Nearest Neighbors (k-NN): Accuracy = 90 % - Random Forest (RF): Accuracy = 91 % Random Forest outperformed others due to its ability to aggregate predictions from multiple decision trees, resulting in enhanced accuracy and generalization. However, it may be computationally expensive and could overfit noisy data. Decision Trees are interpretable but may lack predictive accuracy for complex datasets. ANN can model complex relationships but requires careful tuning of hyperparameters and may suffer from overfitting. k-NN is intuitive but sensitive to the choice of distance metric and may not perform well with high-dimensional data.

The process of hyperparameter tuning for the machine learning models involved careful selection and optimization of key parameters that significantly influence model performance. For instance, in the Random Forest classifier, critical hyperparameters such as the number of trees, maximum depth of each tree, and minimum samples required to split a node were fine-tuned. Adjustments to these parameters were performed to prevent overfitting while maximizing the model's accuracy.

Similarly, for the k-Nearest Neighbors (k-NN) algorithm, the optimal value of k (the number of neighbors) was selected after testing various values to balance bias and variance. The distance metric used for k-NN, such as Euclidean or Manhattan distance, was also a crucial hyperparameter that was evaluated and optimized for this specific dataset.

In the case of the Artificial Neural Network (ANN), hyperparameters such as the number of hidden layers, neurons per layer, learning rate, and the activation function were adjusted through grid search and cross-validation techniques. This ensured that the models learned effectively from the training data and generalized well to the unseen test data. By fine-tuning these hyperparameters, the models achieved the desired balance between accuracy and computational efficiency, enabling reliable predictions across multiple fault conditions.

Traditional fault prediction models often face limitations in adapting to diverse industrial settings or motor conditions. They may require extensive customization or retraining for each unique scenario, leading to inefficiencies and delays in implementation. In contrast, our machine learning-based approach demonstrates high adaptability and scalability. By training on a diverse dataset encompassing various motor specifications and operating conditions, our model generalizes well to different industrial environments. For instance, our developed ANN classifier achieved an accuracy of 98 % in predicting faults for a single induction motor and 90 % for four different induction motors, while our RF classifier achieved 91 % accuracy for the same four induction motors. This adaptability ensures broader applicability and effectiveness across a range of settings, enhancing its practical utility in real-world scenarios.

### 3.4 Limitation of the study

The limitation of the study lies in its focus on a limited number of fault conditions, such as healthy, load imbalance, drive end faulty bearing, and opposite drive end faulty bearing. This restriction restricts the representation of fault types that induction motors may encounter in real-world industrial settings. By confining the analysis to only a few fault conditions, the study may overlook other critical fault types such as rotor bar faults, stator winding faults, insulation degradation, or other mechanical issues that can affect motor performance. Consequently, the predictive model's ability to generalize to a broader range of fault scenarios may be compromised, limiting its practical utility in real-world applications where diverse fault types are prevalent.

## 4 Conclusion

In this study, we trained multiple machine learning algorithms using a comprehensive dataset. The Random Forest model emerged as the top performer with an impressive accuracy of 0.91. It was closely followed by the Artificial Neural Network (ANN) and k-Nearest Neighbors (k-NN) models, both achieving a commendable accuracy of 0.9. The Decision Tree model, while slightly lower in accuracy at 0.89, still demonstrated promising results. Overall, our machine learning models exhibited strong potential in effectively predicting faults in induction motors, aligning with the project's objectives. These trained models hold significant promise for implementation in industrial settings to enable proactive maintenance practices and minimize downtime.

The key novelty of this work lies in the application of machine learning models for fault prediction across multiple induction motors with varying specifications, which contrasts with existing literature that typically focuses on single motor conditions. While previous studies often design machine learning models to detect faults in specific motors or operating environments, our approach introduces a more generalized framework capable of handling diverse motor configurations and multiple fault types. This makes our model highly adaptable and flexible across a wider range of industrial scenarios. Additionally, our study compares the performance of various machine learning algorithms, including Random Forest, Artificial Neural Networks, and k-Nearest Neighbors, offering valuable insights into the most effective models for fault prediction with an emphasis on operational efficiency. Our model demonstrated high accuracy across different fault conditions while maintaining scalability, addressing a key limitation of many existing fault detection models that struggle with generalization and scalability in real-world industrial environments. This research significantly advances the field of predictive maintenance by demonstrating the effectiveness of machine learning techniques in fault prediction for induction motors. By leveraging data analytics and advanced algorithms, industries can transition from reactive to proactive maintenance strategies, leading to substantial cost savings, increased equipment reliability, and enhanced operational efficiency. Furthermore, the study underscores the importance of data-driven approaches in optimizing maintenance schedules, reducing unplanned downtime, and maximizing asset utilization. In the broader context of operational optimization, the research highlights the transformative potential of predictive maintenance in driving competitiveness and sustainability across industries.

For future endeavours, we recommend a heightened focus on refining the Random Forest (RF) and Artificial Neural Network (ANN) models. Exploring additional techniques, such as transfer learning or ensemble methods, can potentially yield further enhancements in performance. Moreover, we encourage the utilization of more diverse and extensive datasets to bolster the models' generalization capabilities, a critical aspect of their robustness in various real-world scenarios.

## CRediT authorship contribution statement

**Ademola Abdulkareem:** Supervision, Resources, Methodology. **Tochukwu Anyim:** Writing – original draft, Data curation. **Olawale Popoola:** Supervision. **John Abubakar:** Writing – review & editing, Writing – original draft. **Agbetuyi Ayoade:** Conceptualization.

## Data availability statement

The authors do not have permission to share data.

## Declaration of competing interest

The authors declare the following financial interests/personal relationships which may be considered as potential competing interests: Ademola Abdulkareem reports financial support was provided by 10.13039/501100006335Covenant University. Ademola Abdulkareem reports a relationship with Covenant University that includes: employment. No conflict.
