# Supplementary material for: Prediction of induction motor faults using machine learning
Source: Heliyon. 2024 Dec 25;11(1):e41493. doi: 10.1016/j.heliyon.2024.e41493 (PMC11759633; doi:10.1016/j.heliyon.2024.e41493)
Supplement: Multimedia component 1 [file mmc1.pdf]

X	Y	Z	Temp
3.229891	3.434481	3.405443	35.20355
3.223416	3.405906	3.406006	35.12813
3.242016	3.357787	3.442684	35.14378
3.328463	3.281363	3.429537	35.13695
3.39239	3.350104	3.381537	35.32211
3.281734	3.418509	3.423262	35.32096
3.334484	3.432998	3.441337	35.50753
3.254554	3.413993	3.443502	35.29591
3.297695	3.291741	3.314217	34.84018
3.280373	3.220034	3.350196	34.72417
3.142743	3.209453	3.343938	34.38637
3.208692	3.254944	3.455779	34.87465
3.21129	3.396875	3.352737	34.96537
3.28227	3.425181	3.404846	35.29645
3.305902	3.433335	3.360018	35.26793
3.371067	3.356507	3.41813	35.3695
3.244243	3.412848	3.369567	35.10917
3.401092	3.446342	3.34919	35.48086
3.309532	3.406108	3.414856	35.33625
3.306604	3.341478	3.361041	35.07082
3.23385	3.176498	3.454432	34.75517
3.319265	3.230211	3.377018	34.89013
3.265154	3.325101	3.371971	34.96827
3.294643	3.452003	3.399714	35.37094
3.181471	3.359809	3.456342	35.04567
3.290312	3.38279	3.444457	35.30796
3.211125	3.248542	3.395536	34.73422
3.369458	3.345252	3.42258	35.3511
3.229355	3.435492	3.442974	35.28666
3.371933	3.412915	3.403124	35.46194
3.228653	3.342152	3.42686	35.04577
3.352177	3.251507	3.286986	34.81179
3.19038	3.219226	3.367384	34.56318
3.253028	3.283587	3.347366	34.79716
3.107109	3.369042	3.442087	34.87207
3.187328	3.498303	3.403192	35.24511
3.366159	3.464134	3.4279	35.6155
3.385461	3.340804	3.35509	35.22878
3.356137	3.371198	3.412963	35.35768
3.284868	3.467841	3.399594	35.38394
3.293818	3.462247	3.415265	35.42554
3.383646	3.381779	3.319043	35.23559
3.371232	3.201231	3.32259	34.82137
3.197721	3.177778	3.44161	34.65092
3.259875	3.350441	3.397412	35.06777
3.251049	3.486442	3.387914	35.32511
3.275671	3.438525	3.379388	35.25553
3.261071	3.343904	3.462361	35.19813
3.219415	3.297133	3.407285	34.88431

3.17281	3.288911	3.462464	34.88508
3.408557	3.465617	3.397275	35.64449
3.265195	3.449847	3.434721	35.37838
3.395112	3.347139	3.380292	35.31885
3.300417	3.159784	3.358534	34.65448
3.358488	3.150619	3.345899	34.73379
3.235912	3.275567	3.376455	34.8058
3.292787	3.436166	3.413372	35.36211
3.187163	3.453217	3.374819	35.08411
3.326854	3.446814	3.367435	35.35944
3.27897	3.367492	3.348508	35.03987
3.324421	3.331908	3.398554	35.17089
3.31811	3.432324	3.374426	35.32392
3.373088	3.459821	3.47324	35.72037
3.234675	3.346465	3.352055	34.90478
3.321369	3.157628	3.334065	34.64207
3.216693	3.172926	3.374495	34.53503
3.329617	3.304681	3.371698	35.06399
3.174253	3.483476	3.4744	35.33982
3.222673	3.46218	3.344808	35.11574
3.218343	3.353339	3.445821	35.08915
3.268206	3.335615	3.377001	35.00893
3.320709	3.34296	3.476241	35.35683
3.198092	3.375444	3.405614	35.00528
3.262927	3.432796	3.442564	35.35328
3.352013	3.368907	3.383361	35.27892
3.36983	3.22118	3.350929	34.9239
3.29522	3.13222	3.395076	34.66274
3.274145	3.209319	3.407148	34.81166
3.186008	3.341343	3.404147	34.90107
3.309408	3.461169	3.397599	35.41865
3.288951	3.405367	3.363513	35.17734
3.24812	3.380027	3.405648	35.12478
3.194009	3.381375	3.445958	35.09754
3.363602	3.461843	3.377956	35.49568
3.218013	3.448769	3.43438	35.27209
3.34694	3.391619	3.382713	35.31607
3.224983	3.221315	3.478969	34.88744
3.268618	3.207701	3.30003	34.56179
3.298602	3.23203	3.37961	34.85459
3.415569	3.399975	3.304413	35.3132
3.243254	3.423429	3.434704	35.27259
3.241439	3.378612	3.362558	35.01284
3.16551	3.321192	3.431515	34.87203
3.365045	3.396404	3.418931	35.44533
3.254183	3.343971	3.458576	35.17493
3.154745	3.30832	3.437995	34.83451
3.300335	3.353204	3.392416	35.15137
3.368097	3.351789	3.375978	35.26051
3.374284	3.295448	3.31432	35.016

3.273114	3.229132	3.377138	34.7871
3.214507	3.243555	3.384384	34.70633
3.14695	3.355833	3.399322	34.83679
3.374325	3.431381	3.342915	35.37588
3.329989	3.401593	3.355465	35.24123
3.279135	3.382992	3.393473	35.17246
3.168686	3.38771	3.45653	35.07914
3.372923	3.510771	3.388255	35.64558
3.345496	3.415274	3.433016	35.47465
3.310934	3.328471	3.349736	35.02712
3.233438	3.186877	3.412741	34.68579
3.340423	3.21653	3.400737	34.95835
3.269319	3.334604	3.370743	34.99547
3.390493	3.415611	3.364792	35.42459
3.151405	3.384273	3.401555	34.91361
3.203	3.330964	3.405716	34.91896
3.231541	3.337973	3.387045	34.95587
3.310315	3.323281	3.434158	35.19904
3.239707	3.354822	3.412127	35.06543
3.237892	3.422418	3.496447	35.39367
3.196484	3.385081	3.484307	35.19492
3.409836	3.283452	3.405392	35.26667
3.449842	3.230211	3.332957	35.07932
3.304583	3.232502	3.40014	34.91359
3.193968	3.348419	3.376541	34.87358
3.246388	3.430437	3.362763	35.13745
3.386121	3.426394	3.326835	35.35561
3.412393	3.431651	3.352532	35.48075
3.159488	3.400178	3.453375	35.07939
3.21859	3.398762	3.35243	34.98479
3.294602	3.395056	3.415879	35.28166
3.384884	3.363246	3.343171	35.25053
3.301242	3.204669	3.385953	34.8144
3.149755	3.138151	3.384726	34.33497
3.273939	3.34788	3.450408	35.20882
3.25707	3.483207	3.395843	35.34855
3.396927	3.463056	3.399799	35.61897
3.266556	3.348689	3.438063	35.16745
3.211579	3.342219	3.467664	35.09781
3.162458	3.359607	3.430083	34.94623
3.326607	3.399908	3.376268	35.27564
3.219332	3.37787	3.398623	35.04174
3.2249	3.319912	3.4264	34.98792
3.259998	3.234996	3.399168	34.81942
3.399401	3.258179	3.330161	35.02406
3.37878	3.241196	3.316587	34.91215
3.382203	3.347206	3.412605	35.36143
3.212981	3.411028	3.427423	35.16335
3.315306	3.462449	3.35792	35.34757
3.281899	3.402873	3.390233	35.2149

3.298066	3.414802	3.373147	35.23898
3.200855	3.438255	3.441098	35.22628
3.228365	3.418913	3.431447	35.22303
3.224323	3.305826	3.472302	35.05623
3.371397	3.245374	3.384487	35.05362
3.334773	3.214845	3.392774	34.92489
3.24313	3.345184	3.399714	35.02469
3.345991	3.412713	3.445548	35.49754
3.284662	3.430977	3.452523	35.41861
3.379687	3.384205	3.349531	35.29891
3.348754	3.338243	3.343921	35.11849
3.197391	3.391619	3.449249	35.13454
3.13004	3.406108	3.443843	35.00712
3.248244	3.372007	3.413048	35.12369
3.209805	3.275769	3.422785	34.85047
3.37317	3.248879	3.344143	34.97694
3.265154	3.191662	3.427406	34.79768
3.290807	3.301379	3.381724	34.99382
3.255544	3.327864	3.397395	35.00889
3.329164	3.42754	3.365611	35.31835
3.278393	3.409276	3.420176	35.28671
3.33568	3.421339	3.353623	35.29283
3.20333	3.38771	3.404642	35.04143
3.315223	3.434683	3.372994	35.31964
3.19603	3.417026	3.382526	35.04121
3.340959	3.395325	3.396781	35.34187
3.200319	3.197997	3.403414	34.61729
3.235665	3.157628	3.36486	34.52199
3.315058	3.268895	3.373284	34.95736
3.462916	3.44796	3.404437	35.74041
3.274557	3.439401	3.436886	35.38074
3.298025	3.364122	3.353129	35.08428
3.276083	3.291741	3.334236	34.83669
3.296045	3.407928	3.406893	35.29332
3.221023	3.407254	3.323596	34.94563
3.162087	3.368705	3.468875	35.05014
3.104016	3.314385	3.442428	34.74653
3.302026	3.257977	3.396576	34.95592
3.333865	3.243757	3.384043	34.96704
3.343104	3.271995	3.400277	35.0845
3.287054	3.272197	3.472558	35.12043
3.211249	3.357989	3.38921	34.96
3.367231	3.413589	3.380326	35.40327
3.402618	3.442299	3.324056	35.42039
3.294767	3.399504	3.354578	35.1577
3.190833	3.426326	3.311643	34.89517
3.286972	3.412511	3.396662	35.26112
3.262638	3.433403	3.376797	35.21016
3.276166	3.342286	3.404505	35.10108
3.183162	3.144149	3.427695	34.51511

3.211661	3.195233	3.416595	34.66487
3.307098	3.338647	3.424268	35.20398
3.428313	3.436975	3.427576	35.69132
3.226962	3.395864	3.415145	35.13391
3.253152	3.361426	3.450323	35.1928
3.236778	3.330627	3.429725	35.0446
3.333288	3.431718	3.288265	35.16737
3.196979	3.363381	3.413577	34.99387
3.219003	3.307242	3.347144	34.77399
3.167407	3.237624	3.505314	34.85481
3.283631	3.209858	3.294403	34.58703
3.350569	3.245779	3.444525	35.14026
3.411279	3.370727	3.334389	35.30541
3.244079	3.327999	3.519842	35.25189
3.311594	3.431516	3.347263	35.2485
3.40699	3.407658	3.399987	35.52024
3.439614	3.390743	3.279603	35.29133
3.270804	3.348756	3.376524	35.04231
3.136309	3.38306	3.421011	34.92049
3.244491	3.380162	3.361689	35.021
3.219992	3.338378	3.404437	34.96954
3.312089	3.250564	3.374938	34.91439
3.218384	3.191864	3.471774	34.79287
3.214878	3.333391	3.39482	34.92642
3.210176	3.390271	3.411718	35.07748
3.4312	3.397078	3.360239	35.46313
3.266762	3.364189	3.460639	35.25117
3.302562	3.386901	3.349599	35.13629
3.272	3.38279	3.473752	35.33197
3.335433	3.448566	3.36972	35.38703
3.164025	3.2848	3.396798	34.71327
3.220529	3.245846	3.339232	34.62577
3.148971	3.264784	3.417396	34.68163
3.299221	3.245442	3.360018	34.84242
3.273279	3.331369	3.399305	35.05952
3.324503	3.318564	3.374154	35.08853
3.326565	3.337434	3.426178	35.24808
3.374243	3.391349	3.421847	35.46077
3.36154	3.420059	3.375859	35.39521
3.399855	3.374433	3.378519	35.38504
3.215827	3.37996	3.387368	35.01403
3.212857	3.410219	3.361791	35.01778
3.150827	3.337636	3.367128	34.73507
3.235624	3.271725	3.367674	34.77757
3.271382	3.228122	3.382833	34.79356
3.253111	3.238163	3.406552	34.82743
3.261401	3.424507	3.430628	35.30572
3.319059	3.421676	3.395894	35.34966
3.308707	3.35125	3.407029	35.19736
3.386863	3.33029	3.324687	35.14237

3.208609	3.339995	3.367947	34.86838
3.236119	3.400447	3.371681	35.06891
3.270186	3.361224	3.48001	35.29453
3.151982	3.284328	3.38904	34.66894
3.245233	3.249553	3.411582	34.84611
3.196855	3.274017	3.380207	34.7252
3.261442	3.224011	3.424967	34.85497
3.278269	3.319979	3.415538	35.08102
3.380718	3.388586	3.462805	35.55846
3.280785	3.384205	3.379422	35.148
3.47533	3.450925	3.386294	35.73437
3.334567	3.399571	3.392501	35.32781
3.383976	3.424372	3.334185	35.36257
3.259091	3.371131	3.36602	35.04265
3.270845	3.385486	3.402374	35.17925
3.181512	3.299896	3.359677	34.70335
3.224447	3.251844	3.328421	34.62381
3.21661	3.227313	3.418675	34.7504
3.288663	3.425652	3.404488	35.31068
3.320379	3.416285	3.456052	35.47231
3.323307	3.391551	3.356215	35.2063
3.270722	3.353743	3.48845	35.2978
3.366778	3.402806	3.375449	35.36804
3.272124	3.394449	3.436545	35.27638
3.264453	3.426933	3.322726	35.08173
3.223539	3.265862	3.462549	34.9458
3.219992	3.258718	3.36411	34.70715
3.236943	3.245981	3.388323	34.76931
3.254101	3.243285	3.397599	34.82122
3.303304	3.315059	3.454552	35.21033
3.230386	3.324899	3.463691	35.09237
3.407815	3.364392	3.370863	35.36374
3.475908	3.462854	3.342301	35.66551
3.407279	3.429629	3.326068	35.40727
3.299262	3.414802	3.42316	35.35096
3.309614	3.360415	3.37591	35.15133
3.241439	3.312364	3.383276	34.91327
3.35226	3.30185	3.310108	34.97262
3.112017	3.2354	3.465277	34.64126
3.157756	3.296257	3.393166	34.71668
3.186008	3.330964	3.440228	34.95728
3.26668	3.469863	3.447066	35.4524
3.237191	3.380229	3.422137	35.13738
3.410826	3.329684	3.438608	35.44257
3.172356	3.393034	3.487035	35.16552
3.361375	3.422485	3.343256	35.32885
3.372181	3.442838	3.414054	35.55182
3.282476	3.343432	3.372772	35.04799
3.169057	3.231693	3.352924	34.5122
3.156271	3.217406	3.40101	34.55815

3.213765	3.274825	3.380258	34.76406
3.314234	3.305018	3.403124	35.0998
3.297942	3.387575	3.479805	35.41241
3.27897	3.399706	3.439819	35.31
3.303964	3.327056	3.371033	35.05536
3.416229	3.412241	3.392075	35.53317
3.423075	3.421541	3.352259	35.48141
3.295509	3.362842	3.439154	35.2641
3.243955	3.339658	3.406807	35.02992
3.214053	3.278667	3.369498	34.74957
3.352136	3.317553	3.342438	35.07739
3.245316	3.267479	3.401095	34.86256
3.195576	3.318968	3.460298	34.99586
3.136804	3.391147	3.46867	35.04348
3.362076	3.432257	3.393865	35.46243
3.331473	3.374972	3.394615	35.27188
3.318317	3.346734	3.429673	35.25802
3.216445	3.451464	3.461151	35.33311
3.294024	3.464741	3.421489	35.44506
3.252121	3.380768	3.418931	35.16419
3.365087	3.281228	3.342148	35.02564
3.120431	3.190179	3.427764	34.47874
3.169552	3.264582	3.415333	34.72168
3.205145	3.282171	3.395553	34.79473
3.297365	3.384205	3.422904	35.27934
3.215703	3.399032	3.418624	35.12382
3.324173	3.371266	3.449027	35.3668
3.237521	3.403143	3.437841	35.22255
3.40996	3.485161	3.390847	35.67624
3.33036	3.452543	3.394411	35.43863
3.39437	3.336356	3.36752	35.26572
3.251585	3.232704	3.347059	34.68206
3.258802	3.229604	3.319657	34.63114
3.182585	3.313914	3.388733	34.79989
3.318647	3.379218	3.29101	35.02654
3.168686	3.397684	3.48163	35.15584
3.17879	3.448095	3.437091	35.19078
3.317203	3.348824	3.481971	35.37452
3.396844	3.401323	3.369839	35.41828
3.312625	3.430437	3.421062	35.40979
3.269732	3.400784	3.466232	35.34992
3.205516	3.388182	3.441541	35.12793
3.23781	3.264379	3.368492	34.76807
3.368015	3.183035	3.328165	34.78674
3.244738	3.201636	3.400362	34.71571
3.210465	3.296526	3.415572	34.88153
3.086941	3.348621	3.454705	34.81091
3.350528	3.450453	3.365969	35.41597
3.309037	3.381442	3.405102	35.25989
3.346238	3.369311	3.43941	35.38974

3.208114	3.462652	3.427542	35.26586
3.342197	3.450656	3.360666	35.38659
3.316254	3.370457	3.351015	35.13337
3.442954	3.242409	3.355618	35.14049
3.252781	3.201366	3.452915	34.84763
3.230839	3.260673	3.35422	34.71351
3.217765	3.342421	3.385169	34.93137
3.322111	3.400515	3.353112	35.2165
3.197968	3.363785	3.360359	34.88054
3.249935	3.374635	3.395997	35.09585
3.165262	3.353137	3.474979	35.03639
3.363189	3.39418	3.453682	35.51241
3.422951	3.474243	3.415009	35.73361
3.373253	3.373692	3.464254	35.51273
3.227334	3.272062	3.397685	34.8258
3.247419	3.182361	3.34948	34.56815
3.214012	3.220708	3.332821	34.54252
3.329617	3.302929	3.356403	35.02671
3.16188	3.353541	3.442343	34.95851
3.163819	3.46346	3.434158	35.18523
3.264288	3.369783	3.409604	35.14638
3.307882	3.352328	3.382611	35.14452
3.318069	3.414128	3.451397	35.45236
3.247625	3.454564	3.38597	35.24366
3.241439	3.426192	3.45948	35.32884
3.290766	3.293898	3.313706	34.82862
3.416064	3.241061	3.363633	35.09627
3.248492	3.281632	3.351219	34.79139
3.21162	3.344915	3.397975	34.95139
3.249275	3.353069	3.417447	35.09415
3.354116	3.384879	3.370931	35.29126
3.191163	3.351722	3.374836	34.87094
3.270763	3.386699	3.3836	35.14067
3.18873	3.420328	3.488433	35.26407
3.325328	3.462786	3.425308	35.51759
3.26136	3.339928	3.444389	35.15076
3.439366	3.325236	3.395962	35.402
3.22721	3.180205	3.449658	34.73831
3.274104	3.164367	3.327773	34.53969
3.219704	3.240185	3.435454	34.822
3.262308	3.31789	3.358807	34.91749
3.195453	3.356507	3.430202	35.01186
3.223581	3.410691	3.3511	35.01888
3.189761	3.346195	3.423791	34.96285
3.310315	3.426663	3.381861	35.31076
3.314935	3.455373	3.456001	35.54577
3.312584	3.458406	3.36793	35.35467
3.249729	3.312835	3.390983	34.94929
3.283713	3.232367	3.355329	34.76967
3.355436	3.243218	3.301173	34.83181

3.342733	3.323281	3.349974	35.08584
3.161056	3.383599	3.34624	34.81228
3.228942	3.337636	3.384657	34.94423
3.28194	3.32436	3.422222	35.11324
3.214754	3.415813	3.41269	35.14547
3.349497	3.433403	3.390165	35.42934
3.250884	3.424844	3.522553	35.48448
3.205805	3.351048	3.417737	34.9953
3.304253	3.27058	3.391069	34.9763
3.360921	3.24315	3.372176	34.99893
3.225024	3.227313	3.374341	34.67184
3.28858	3.260875	3.413764	34.97044
3.215538	3.269366	3.449641	34.90773
3.379274	3.377466	3.373881	35.33652
3.275135	3.39573	3.364536	35.12829
3.29951	3.362505	3.41535	35.22006
3.130618	3.367222	3.431788	34.89698
3.231293	3.410624	3.43663	35.22264
3.313038	3.44122	3.328097	35.23097
3.447656	3.3003	3.351509	35.26839
3.224529	3.217878	3.326443	34.54538
3.324008	3.240657	3.40355	34.98136
3.297819	3.385621	3.404915	35.24409
3.368634	3.424709	3.375859	35.42089
3.24016	3.363111	3.35405	34.95754
3.147569	3.250361	3.390983	34.58926
3.109872	3.384745	3.431362	34.889
3.306892	3.439401	3.366242	35.29697
3.277527	3.481589	3.443366	35.49367
3.252327	3.377601	3.383685	35.08064
3.267793	3.231019	3.467306	34.97678
3.271382	3.278802	3.376762	34.89111
3.443367	3.271927	3.447202	35.40623
3.32607	3.249553	3.329734	34.8439
3.235459	3.262425	3.395366	34.81743
3.232943	3.34633	3.36145	34.92124
3.303675	3.410961	3.378655	35.25488
3.314564	3.456519	3.319827	35.24968
3.320049	3.396875	3.418215	35.3464
3.089498	3.366548	3.420449	34.78079
3.277816	3.404423	3.438472	35.31485
3.314564	3.345858	3.402868	35.18928
3.402701	3.227852	3.378024	35.06963
3.192483	3.205814	3.418624	34.65051
3.318399	3.307242	3.374	35.05009
3.268536	3.386834	3.427832	35.23282
3.429385	3.434279	3.244732	35.28792
3.166417	3.287495	3.458917	34.86024
3.287178	3.342623	3.333963	34.97163
3.215497	3.452745	3.447953	35.30497

3.279754	3.50794	3.336845	35.32322
3.232077	3.418509	3.443025	35.25558
3.320668	3.279408	3.488246	35.24402
3.256864	3.27961	3.420807	34.95745
3.388059	3.22091	3.393661	35.05662
3.324379	3.254877	3.385868	34.9746
3.33135	3.244835	3.384657	34.96524
3.277403	3.332649	3.350554	34.96473
3.212445	3.411904	3.369447	35.03731
3.231334	3.374164	3.382389	35.02438
3.271464	3.414802	3.354578	35.14019
3.178873	3.382521	3.408598	34.98525
3.269814	3.424237	3.410849	35.28027
3.234304	3.407321	3.449249	35.2496
3.208733	3.226571	3.378212	34.64306
3.371892	3.220978	3.331917	34.88639
3.285693	3.301109	3.410934	35.04592
3.333824	3.456182	3.378161	35.41863
3.352177	3.369581	3.34387	35.19439
3.299303	3.249755	3.389619	34.91677
3.236572	3.261144	3.326904	34.66735
3.318894	3.384205	3.475218	35.44082
3.160643	3.522362	3.411752	35.25809
3.26103	3.391282	3.396048	35.15663
3.21991	3.274623	3.442189	34.91249
3.219085	3.26721	3.423518	34.85365
3.273114	3.2385	3.36631	34.78391
3.297736	3.296661	3.352174	34.93403
3.238676	3.276847	3.439018	34.95146
3.312336	3.326719	3.385663	35.10493
3.317244	3.342354	3.368868	35.11312
3.402165	3.360415	3.363496	35.32658
3.271464	3.370996	3.372841	35.08433
3.195659	3.366818	3.414054	34.99955
3.149425	3.483678	3.46289	35.26079
3.348548	3.466628	3.382134	35.48236
3.317822	3.312094	3.454228	35.23488
3.228447	3.181957	3.40708	34.65174
3.216693	3.248609	3.449095	34.86367
3.400474	3.404828	3.379798	35.45565
3.425756	3.412443	3.414446	35.60337
3.251502	3.34195	3.35954	34.94807
3.224117	3.274084	3.407916	34.84556
3.179203	3.41487	3.371664	34.97594
3.354528	3.521419	3.394394	35.64206
3.246512	3.46191	3.410576	35.3111
3.123112	3.296257	3.417925	34.69506
3.140393	3.179598	3.41801	34.47792
3.332133	3.252383	3.40899	35.03667
3.251007	3.301715	3.393302	34.93284

3.289116	3.254405	3.441729	35.01862
3.247048	3.239039	3.434277	34.87672
3.287797	3.40813	3.410593	35.28381
3.412063	3.398291	3.334202	35.36699
3.430293	3.385081	3.388596	35.49692
3.091313	3.370727	3.473854	34.91068
3.280002	3.408939	3.367282	35.17382
3.198711	3.442703	3.495015	35.34922
3.349744	3.42235	3.318276	35.2485
3.282517	3.192807	3.402647	34.78402
3.2428	3.22118	3.455984	34.87585
3.163654	3.368031	3.458235	35.02883
3.406371	3.461236	3.379593	35.59146
3.346651	3.372614	3.392825	35.29599
3.369376	3.257775	3.377018	35.05999
3.161303	3.289248	3.407523	34.7405
3.244573	3.562529	3.368237	35.43431
3.270928	3.51313	3.404915	35.46412
3.211001	3.373692	3.347331	34.90222
3.15854	3.244431	3.469165	34.77125
3.197886	3.252114	3.446111	34.82368
3.27266	3.351115	3.43704	35.18386
3.398329	3.335547	3.4648	35.48534
3.316213	3.22765	3.430697	34.99524
3.308047	3.281295	3.471552	35.18404
3.281404	3.351654	3.363565	35.04349
3.345867	3.328538	3.384998	35.18078
3.35391	3.40503	3.351714	35.29285
3.164025	3.38744	3.445548	35.04434
3.194958	3.406782	3.381912	35.01512
3.27134	3.309601	3.384828	34.97601
3.361581	3.312701	3.3105	35.01759
3.234221	3.269838	3.438336	34.9249
3.323967	3.375781	3.412554	35.29646
3.273774	3.443242	3.359336	35.21784
3.462339	3.361022	3.331473	35.38947
3.383193	3.330897	3.372926	35.24116
3.250265	3.328538	3.394206	34.99185
3.224117	3.400784	3.369055	35.03766
3.278146	3.470065	3.434431	35.45028
3.223374	3.314655	3.326529	34.75468
3.256781	3.241735	3.400123	34.82921
3.137671	3.258179	3.434039	34.67887
3.189843	3.370929	3.44207	35.05709
3.196236	3.339591	3.428036	34.97185
3.25307	3.296998	3.392723	34.92576
3.293117	3.282643	3.449777	35.10672
3.46968	3.377533	3.371971	35.53019
3.263587	3.410556	3.438608	35.29744
3.326359	3.392764	3.33444	35.16801

3.272	3.315262	3.395826	35.01389
3.248327	3.379151	3.349037	34.99951
3.219415	3.396606	3.405665	35.0983
3.236407	3.306837	3.386891	34.89809
3.282641	3.218484	3.384384	34.8005
3.20498	3.319642	3.391887	34.86829
3.373418	3.433403	3.464118	35.64337
3.321451	3.456586	3.326102	35.27861
3.33964	3.353002	3.41431	35.28476
3.351971	3.298211	3.278221	34.8943
3.356962	3.391551	3.467869	35.52407
3.220034	3.46555	3.401709	35.24177
3.268495	3.365874	3.441644	35.2171
3.189802	3.254338	3.40072	34.71161
3.219621	3.221719	3.327961	34.54637
3.141919	3.266199	3.45537	34.75234
3.280249	3.421811	3.462054	35.40976
3.135072	3.276308	3.545402	34.95636
3.3589	3.29875	3.346172	35.05923
3.338155	3.351789	3.446401	35.34904
3.451533	3.390675	3.371528	35.51828
3.292498	3.394314	3.474127	35.40282
3.286064	3.381914	3.354493	35.10001
3.232365	3.312364	3.404096	34.93896
3.370861	3.407524	3.341125	35.31222
3.217147	3.346802	3.400686	34.97353
3.160231	3.280756	3.350605	34.59512
3.237315	3.278802	3.477537	35.03699
3.272619	3.410961	3.360018	35.14621
3.424766	3.423092	3.37238	35.5325
3.253812	3.375444	3.423995	35.16732
3.258802	3.297335	3.404488	34.96477
3.344877	3.381712	3.371988	35.26644
3.332216	3.488059	3.386652	35.50339
3.307593	3.445534	3.450613	35.49642
3.222013	3.254338	3.379934	34.73659
3.164231	3.169085	3.494248	34.67378
3.281156	3.258112	3.373403	34.8599
3.247213	3.387979	3.484358	35.31231
3.316296	3.402739	3.409689	35.33237
3.138083	3.304276	3.486199	34.89464
3.26404	3.278802	3.412537	34.95329
3.367396	3.388249	3.377274	35.34155
3.442624	3.372007	3.4028	35.52636
3.1957	3.388316	3.474536	35.17892
3.289488	3.394651	3.466232	35.37971
3.23649	3.369514	3.362746	34.98253
3.378615	3.307848	3.323101	35.07179
3.23418	3.273545	3.357699	34.75658
3.100634	3.237422	3.475218	34.64253

3.105294	3.379555	3.433834	34.87305
3.320131	3.402941	3.417516	35.35832
3.371933	3.440816	3.412264	35.54294
3.394947	3.342017	3.402613	35.3561
3.203536	3.317149	3.44398	34.9736
3.314935	3.474917	3.380497	35.4234
3.370572	3.473502	3.400157	35.58497
3.331515	3.302794	3.300747	34.90885
3.164767	3.129862	3.375722	34.32998
3.195205	3.221719	3.370794	34.58665
3.286972	3.3466	3.418198	35.16408
3.284703	3.469593	3.359285	35.29926
3.236902	3.316812	3.433561	35.02304
3.16683	3.204871	3.405324	34.56326
3.256452	3.236344	3.451295	34.92861
3.324008	3.436772	3.38418	35.36788
3.303799	3.488126	3.433203	35.54319
3.225643	3.446949	3.386618	35.18035
3.270392	3.266132	3.448311	35.01771
3.231499	3.214508	3.298734	34.49266
3.38344	3.221315	3.315189	34.8758
3.216693	3.234052	3.404676	34.7347
3.194009	3.441827	3.405119	35.14043
3.146662	3.429494	3.40118	35.00131
3.403938	3.449712	3.341892	35.47849
3.401298	3.328403	3.378433	35.28735
3.288085	3.376118	3.421574	35.23845
3.181842	3.387103	3.440365	35.07123
3.429303	3.471885	3.443229	35.80406
3.283961	3.318092	3.387249	35.02748
3.360096	3.170567	3.292732	34.66467
3.083312	3.157493	3.377513	34.21619
3.116554	3.307377	3.395621	34.65626
3.188029	3.443377	3.417993	35.1589
3.280496	3.358933	3.361962	35.05391
3.309614	3.230211	3.445838	35.01952
3.277527	3.343297	3.401504	35.0997
3.232737	3.419722	3.455251	35.28642
3.426292	3.411163	3.328097	35.41291
3.354281	3.352261	3.386021	35.25329
3.223704	3.362774	3.390574	35.00069
3.301737	3.273882	3.40442	35.00722
3.26103	3.261886	3.370061	34.81683
3.283054	3.304748	3.284258	34.77109
3.273485	3.285069	3.40899	34.9799
3.157344	3.385621	3.414873	34.95867
3.253853	3.364459	3.399987	35.09089
3.377089	3.405569	3.325199	35.28674
3.410248	3.388047	3.344484	35.36311
3.373005	3.387575	3.403022	35.40864

3.21195	3.472289	3.434721	35.31102
3.357292	3.499112	3.395485	35.60171
3.267299	3.303939	3.365372	34.91225
3.318028	3.145025	3.359796	34.66347
3.177058	3.196244	3.417328	34.59301
3.18807	3.312903	3.395758	34.82504
3.111976	3.437177	3.47428	35.10212
3.282435	3.376792	3.363241	35.10001
3.234634	3.25272	3.451482	34.91712
3.288168	3.318901	3.403823	35.07469
3.239377	3.390473	3.446111	35.21699
3.358323	3.407389	3.43438	35.48844
3.425426	3.427405	3.408581	35.62254
3.375934	3.361224	3.433016	35.42301
3.245275	3.22583	3.359984	34.68149
3.304211	3.223404	3.33369	34.74757
3.231334	3.252451	3.431651	34.86595
3.246264	3.44924	3.416765	35.29639
3.227829	3.447286	3.460775	35.34804
3.220694	3.356776	3.365645	34.92647
3.345537	3.342286	3.434448	35.31826
3.320874	3.383666	3.411258	35.3041
3.303015	3.414263	3.416424	35.34326
3.281363	3.480039	3.449777	35.51269
3.30714	3.386295	3.371204	35.19223
3.238552	3.196986	3.351577	34.58533
3.358117	3.221584	3.299655	34.78704
3.247419	3.269703	3.404829	34.88019
3.279012	3.43967	3.43409	35.38496
3.153632	3.429427	3.437637	35.09613
3.297777	3.251709	3.393115	34.92535
3.283878	3.23048	3.365747	34.78868
3.295798	3.309803	3.441951	35.15486
3.254266	3.449375	3.476327	35.44443
3.351806	3.456991	3.462941	35.64512
3.294478	3.396606	3.348167	35.13671
3.408186	3.223202	3.352055	35.01466
3.279672	3.209319	3.377837	34.75964
3.202505	3.27058	3.385339	34.74127
3.110945	3.364931	3.385953	34.74871
3.288415	3.493855	3.338141	35.31419
3.265855	3.391484	3.393797	35.1627
3.331185	3.359944	3.367674	35.17946
3.209187	3.347813	3.499517	35.17446
3.298025	3.444186	3.448686	35.46833
3.490838	3.456249	3.409757	35.83123
3.336835	3.406108	3.339999	35.23225
3.333865	3.194088	3.408495	34.9119
3.234098	3.142262	3.376677	34.5108
3.251915	3.349767	3.389346	35.03125

3.345909	3.494259	3.400839	35.57792
3.320833	3.428887	3.44918	35.48584
3.146868	3.314857	3.434175	34.82322
3.257648	3.235872	3.428241	34.87978
3.345455	3.310611	3.373523	35.11558
3.263298	3.411028	3.476804	35.38137
3.213682	3.416352	3.412895	35.14475
3.260411	3.41278	3.49377	35.41599
3.283837	3.260605	3.301991	34.71505
3.43784	3.181889	3.321549	34.92246
3.227499	3.285608	3.389824	34.8386
3.214301	3.366953	3.414992	35.04266
3.195535	3.431179	3.424882	35.1637
3.337165	3.455575	3.309903	35.27534
3.326813	3.372883	3.401709	35.27263
3.295509	3.389799	3.333912	35.0929
3.243171	3.391349	3.487512	35.31774
3.34661	3.469728	3.350503	35.41573
3.273609	3.400919	3.42848	35.27613
3.37383	3.245779	3.369839	35.0278
3.263834	3.147451	3.396048	34.62954
3.241109	3.257438	3.378979	34.78304
3.303304	3.402941	3.38921	35.25962
3.314646	3.463528	3.365628	35.36534
3.231293	3.308455	3.419101	34.96088
3.274722	3.284597	3.382833	34.92437
3.174996	3.257842	3.466164	34.83001
3.2972	3.365066	3.385953	35.15632
3.286518	3.425383	3.43397	35.36987
3.360509	3.4115	3.425717	35.48327
3.352507	3.283587	3.421949	35.1778
3.358034	3.188022	3.344194	34.81087
3.291591	3.266266	3.352447	34.85472
3.277774	3.350778	3.379064	35.06753
3.215414	3.42107	3.327074	34.97118
3.1592	3.41642	3.409689	35.01874
3.309614	3.347745	3.369669	35.10998
3.292869	3.406176	3.414054	35.2982
3.292622	3.460428	3.372517	35.32547
3.217147	3.446545	3.396338	35.18215
3.31411	3.37632	3.417993	35.28798
3.168026	3.22246	3.432078	34.66285
3.372634	3.17697	3.395826	34.93154
3.301036	3.248137	3.372295	34.87914
3.322771	3.476265	3.423722	35.53801
3.306026	3.447016	3.382065	35.34633
3.324008	3.333795	3.465107	35.31966
3.179161	3.269097	3.37857	34.67217
3.276372	3.257505	3.351748	34.80075
3.190833	3.341006	3.427218	34.96134

3.274599	3.458743	3.41946	35.38502
3.195865	3.376725	3.456734	35.115
3.402948	3.308455	3.349156	35.18331
3.375645	3.182698	3.420517	35.00464
3.267835	3.24807	3.368612	34.79833
3.22123	3.311353	3.431225	34.97173
3.361292	3.449847	3.388801	35.48811
3.319265	3.416622	3.354988	35.2496
3.338485	3.398695	3.323868	35.18437
3.156024	3.349969	3.399083	34.84329
3.260493	3.39081	3.376063	35.11072
3.289735	3.456654	3.468892	35.52166
3.304211	3.388519	3.440245	35.34167
3.275011	3.23567	3.408069	34.87319
3.242181	3.126896	3.371528	34.48362
3.252987	3.194964	3.39303	34.70312
3.404474	3.43374	3.365423	35.49619
3.237974	3.460495	3.398691	35.26335
3.204526	3.36082	3.379712	34.93072
3.300417	3.361292	3.393865	35.1724
3.198587	3.306635	3.417192	34.8812
3.310604	3.353676	3.362763	35.11001
3.156601	3.379825	3.415009	34.94467
3.248327	3.386699	3.429861	35.19277
3.130205	3.323686	3.503933	34.95864
3.451409	3.322271	3.343324	35.30674
3.320997	3.201501	3.359063	34.79187
3.40402	3.291202	3.357989	35.16724
3.209723	3.368166	3.483403	35.18491
3.411651	3.473772	3.356079	35.579
3.311511	3.407321	3.327228	35.1516
3.365499	3.356574	3.321908	35.14705
3.163324	3.403817	3.420056	35.02287
3.269361	3.496416	3.381025	35.3719
3.239212	3.427337	3.425359	35.25186
3.348012	3.303603	3.375364	35.10987
3.247625	3.168074	3.412741	34.6757
3.236201	3.180609	3.404232	34.65952
3.289652	3.30704	3.442974	35.13761
3.330277	3.497022	3.429179	35.61175
3.289652	3.415004	3.384708	35.2463
3.258225	3.344241	3.434908	35.1326
3.230509	3.238163	3.394189	34.75097
3.252698	3.297402	3.420244	34.98602
3.222013	3.40995	3.322232	34.9507
3.253317	3.420598	3.455779	35.33449
3.296788	3.411972	3.437909	35.37161
3.213022	3.269097	3.39644	34.7853
3.333082	3.201568	3.339624	34.77593
3.372675	3.261953	3.378433	35.07944

3.331473	3.41487	3.476122	35.53737
3.26037	3.44486	3.455779	35.40297
3.366571	3.446073	3.356403	35.42055
3.305614	3.35253	3.323255	35.01019
3.322523	3.367627	3.415128	35.2811
3.180481	3.450925	3.353402	35.01765
3.220199	3.438457	3.419408	35.22159
3.170047	3.363111	3.343972	34.78218
3.273403	3.197727	3.405068	34.78014
3.21393	3.215991	3.41917	34.72086
3.408475	3.368974	3.43779	35.52157
3.260122	3.457328	3.448822	35.41448
3.341743	3.43812	3.38036	35.40126
3.373707	3.323349	3.381315	35.22226
3.333494	3.329347	3.355107	35.09012
3.201557	3.304883	3.460076	34.97765
3.230138	3.398156	3.391665	35.09452
3.156148	3.363179	3.412946	34.90276
3.306191	3.383801	3.358739	35.15744
3.318605	3.205006	3.441337	34.97422
3.260865	3.210397	3.457416	34.8949
3.255255	3.229267	3.431379	34.86696
3.279713	3.349835	3.376626	35.06437
3.322152	3.46501	3.437381	35.54191
3.346073	3.452543	3.380752	35.44312
3.335845	3.349228	3.440501	35.32548
3.261731	3.36857	3.350998	35.00997
3.36587	3.486307	3.387573	35.57517
3.28924	3.438862	3.392569	35.31476
3.298644	3.424574	3.382816	35.28275
3.136639	3.208106	3.457911	34.61931
3.26103	3.154191	3.325591	34.48407
3.292045	3.269973	3.484835	35.15333
3.339887	3.461776	3.367674	35.42118
3.234469	3.451397	3.478117	35.40948
3.255709	3.427674	3.392859	35.2176
3.27629	3.283182	3.441592	35.0532
3.389668	3.33703	3.348013	35.21425
3.188854	3.346802	3.437568	34.99232
3.169098	3.364392	3.387164	34.87735
3.197061	3.393775	3.388613	35.00593
3.316791	3.300974	3.375432	35.036
3.334402	3.183103	3.405375	34.88222
3.3191	3.236681	3.403431	34.96168
3.17017	3.294167	3.458098	34.88125
3.342073	3.398897	3.393899	35.34581
3.473969	3.42754	3.331252	35.55988
3.423817	3.391888	3.40367	35.53061
3.319348	3.33184	3.323255	34.99498
3.223828	3.395191	3.392484	35.07603

3.199453	3.469593	3.377223	35.15206
3.370077	3.417161	3.392961	35.44494
3.215373	3.306568	3.438046	34.96337
3.082693	3.155067	3.370061	34.19324
3.269361	3.255551	3.421284	34.93321
3.403773	3.429494	3.338686	35.42691
3.342815	3.423024	3.451772	35.52675
3.283425	3.37814	3.370658	35.12134
3.214466	3.238905	3.473666	34.89131
3.257441	3.310611	3.310773	34.78588
3.449471	3.334738	3.357699	35.3612
3.271052	3.353474	3.394189	35.0918
3.226509	3.415476	3.451568	35.25546
3.112182	3.287293	3.394002	34.59924
3.343929	3.204601	3.35006	34.8291
3.265443	3.241937	3.363496	34.7685
3.268453	3.327797	3.415435	35.07643
3.196855	3.437109	3.42142	35.17199
3.447161	3.421541	3.351816	35.53311
3.412558	3.361292	3.344484	35.30965
3.469886	3.352463	3.303475	35.32603
3.277981	3.344308	3.444474	35.19687
3.178914	3.400919	3.361672	34.92295
3.284745	3.340467	3.418419	35.14629
3.35028	3.283856	3.30963	34.9279
3.214837	3.172657	3.404011	34.59493
3.205021	3.327729	3.391342	34.88487
3.165963	3.464	3.544959	35.4334
3.453513	3.515084	3.451431	35.96941
3.399071	3.374096	3.403482	35.43718
3.40732	3.30859	3.411718	35.32997
3.289859	3.312633	3.426468	35.1142
3.340753	3.318429	3.35538	35.08272
3.315636	3.30057	3.355908	34.98989
3.289652	3.354485	3.366071	35.0732
3.166871	3.335278	3.479652	35.01107
3.144187	3.215047	3.420022	34.56814
3.342155	3.277454	3.368697	35.0253
3.245027	3.262155	3.460025	34.97916
3.280909	3.394517	3.403448	35.22336
3.223828	3.4177	3.497129	35.35409
3.382409	3.424574	3.338004	35.36794
3.361375	3.39108	3.365679	35.30921
3.448027	3.438457	3.377564	35.6283
3.27101	3.426866	3.367469	35.19377
3.242841	3.381038	3.393405	35.08867
3.183987	3.251372	3.312188	34.4988
3.329989	3.186674	3.379286	34.82333
3.143032	3.21006	3.39569	34.5015
3.235294	3.380229	3.431174	35.15299

3.271299	3.441625	3.410576	35.32095
3.351559	3.407928	3.418147	35.43933
3.293117	3.364392	3.433885	35.25074
3.428767	3.333862	3.381298	35.36562
3.308583	3.342152	3.41963	35.20475
3.27134	3.357989	3.412809	35.14302
3.208815	3.347341	3.41269	34.98274
3.327473	3.311016	3.436477	35.21481
3.25707	3.247059	3.367111	34.7693
3.241191	3.22583	3.382765	34.72238
3.195947	3.274893	3.449232	34.87608
3.285941	3.358259	3.444201	35.24419
3.313161	3.434414	3.372926	35.31439
3.344589	3.434818	3.348917	35.3315
3.403484	3.374298	3.430151	35.50559
3.363602	3.388451	3.324772	35.21888
3.382615	3.367559	3.35081	35.27171
3.388307	3.389395	3.297302	35.21489
3.327596	3.333256	3.351236	35.07731
3.220034	3.224011	3.384845	34.67668
3.214136	3.186742	3.443025	34.70951
3.213352	3.389934	3.418283	35.09804
3.344877	3.452677	3.366924	35.41056
3.22057	3.42909	3.495902	35.36919
3.245027	3.380229	3.430492	35.17278
3.301737	3.324629	3.316161	34.92519
3.418002	3.41743	3.469011	35.71664
3.217147	3.419722	3.408922	35.15101
3.336464	3.313577	3.402851	35.16654
3.185307	3.192201	3.404147	34.57339
3.368386	3.253462	3.347485	34.98381
3.229066	3.248609	3.431447	34.85214
3.260163	3.354417	3.376114	35.03052
3.194916	3.392158	3.469864	35.17539
3.227911	3.414263	3.375569	35.08967
3.379274	3.41588	3.432265	35.5482
3.513769	3.46555	3.308727	35.68078
3.26338	3.326853	3.452676	35.14471
3.31114	3.425316	3.324227	35.18358
3.288663	3.35718	3.392075	35.13379
3.36818	3.30704	3.317645	35.03527
3.284703	3.243218	3.378263	34.84571
3.112883	3.204601	3.391887	34.41531
3.16584	3.255686	3.416834	34.69739
3.228571	3.464	3.455234	35.3741
3.322688	3.479635	3.43825	35.57697
3.279589	3.357585	3.437841	35.21492
3.325452	3.310679	3.400191	35.1303
3.194834	3.404558	3.458235	35.17689
3.460978	3.46656	3.363223	35.68672

3.336876	3.37504	3.483471	35.47815
3.297489	3.273612	3.357699	34.89517
3.209599	3.19429	3.426877	34.68078
3.290024	3.227582	3.363513	34.7909
3.21859	3.243083	3.451687	34.8614
3.269278	3.399504	3.432316	35.27196
3.129298	3.376522	3.492901	35.04808
3.287879	3.425046	3.392757	35.28198
3.361292	3.468852	3.39939	35.55283
3.421714	3.442231	3.442752	35.72157
3.319348	3.447218	3.371732	35.35331
3.357869	3.392764	3.398503	35.37701
3.248327	3.292887	3.371698	34.86042
3.34133	3.269501	3.339505	34.94227
3.189885	3.206286	3.350247	34.49633
3.263422	3.271321	3.384998	34.87536
3.187575	3.433807	3.400123	35.0979
3.252492	3.399975	3.417465	35.2038
3.234469	3.345926	3.430407	35.07449
3.353827	3.362842	3.431856	35.37567
3.260782	3.387238	3.454279	35.27458
3.280455	3.494327	3.392279	35.41621
3.317657	3.36729	3.431992	35.3066
3.351559	3.289719	3.325011	34.97715
3.252987	3.185394	3.388204	34.67164
3.209723	3.211947	3.368203	34.59136
3.176151	3.272264	3.43397	34.79367
3.198298	3.417228	3.314797	34.8985
3.229025	3.334401	3.434584	35.04652
3.297489	3.379286	3.370249	35.1537
3.270681	3.402334	3.452454	35.32525
3.279094	3.42417	3.390642	35.25623
3.414084	3.401189	3.408393	35.53999
3.332752	3.395999	3.372789	35.27293
3.329246	3.351654	3.362081	35.14487
3.300005	3.245913	3.374273	34.87634
3.338361	3.205612	3.35567	34.83141
3.198752	3.248946	3.36556	34.6425
3.324256	3.372209	3.377888	35.21347
3.153261	3.442568	3.417891	35.08087
3.221683	3.384003	3.381042	35.02185
3.235376	3.380229	3.493429	35.28931
3.318894	3.385958	3.389227	35.25661
3.321451	3.408063	3.490292	35.53155
3.362323	3.389462	3.358739	35.29257
3.261566	3.271658	3.484494	35.08962
3.329494	3.26566	3.357835	34.94807
3.297283	3.178924	3.38152	34.73974
3.333865	3.276375	3.323357	34.90566
3.12307	3.308185	3.444389	34.77893

3.183533	3.391753	3.366702	34.92401
3.288044	3.421676	3.350333	35.1822
3.334855	3.396875	3.41182	35.3648
3.329411	3.455575	3.410439	35.47824
3.325823	3.420194	3.457365	35.49564
3.278847	3.414128	3.379405	35.20916
3.400226	3.383936	3.383975	35.41856
3.340052	3.283317	3.296245	34.87508
3.234922	3.202108	3.434158	34.76918
3.312089	3.238433	3.384026	34.90774
3.175078	3.300435	3.414873	34.81116
3.333577	3.407658	3.402323	35.36481
3.217394	3.401121	3.410559	35.11445
3.167654	3.319103	3.434226	34.87808
3.216033	3.364122	3.389091	34.98362
3.327308	3.441086	3.438881	35.50415
3.274846	3.452543	3.39453	35.31749
3.306521	3.39883	3.430952	35.34895
3.246306	3.226908	3.4146	34.80554
3.317739	3.162615	3.376524	34.73789
3.393751	3.230413	3.343034	34.97914
3.315223	3.28116	3.391751	35.02492
3.208485	3.373759	3.405034	35.02305
3.240325	3.346398	3.381383	34.98113
3.150044	3.416285	3.423791	35.02926
3.39606	3.466358	3.352754	35.52142
3.267381	3.438323	3.45121	35.39402
3.24115	3.415678	3.414429	35.2067
3.290601	3.376927	3.431703	35.26787
3.366035	3.320788	3.369174	35.17333
3.364138	3.272534	3.385203	35.09871
3.290354	3.283115	3.366225	34.91899
3.191658	3.277386	3.445685	34.8644
3.216693	3.385149	3.354971	34.95643
3.32141	3.398291	3.376268	35.26074
3.268041	3.36884	3.37504	35.07694
3.188565	3.374905	3.428991	35.03439
3.191369	3.393236	3.415401	35.05089
3.365664	3.434751	3.436733	35.56948
3.326772	3.356035	3.38007	35.18837
3.290807	3.283991	3.388528	34.97067
3.246141	3.24524	3.393166	34.79839
3.33436	3.236141	3.417038	35.02362
3.313615	3.231963	3.304771	34.72361
3.400061	3.311016	3.353675	35.19247
3.111646	3.344915	3.390165	34.71569
3.280991	3.362437	3.389807	35.12355
3.207454	3.419722	3.394956	35.09927
3.345826	3.471885	3.420329	35.57143
3.290477	3.440344	3.406586	35.35136

3.305407	3.385621	3.438864	35.33493
3.215126	3.292213	3.40899	34.8679
3.383729	3.298481	3.393661	35.21679
3.345455	3.322473	3.401572	35.20286
3.252245	3.342758	3.303952	34.8299
3.202711	3.320114	3.418283	34.92209
3.209682	3.397415	3.378587	35.01956
3.366736	3.388519	3.356266	35.29475
3.326318	3.35772	3.377359	35.18514
3.164273	3.393169	3.502245	35.1814
3.287962	3.445534	3.416697	35.37932
3.271217	3.387575	3.458115	35.30653
3.360426	3.288978	3.346939	35.04288
3.3151	3.191055	3.379133	34.80001
3.234221	3.214171	3.436869	34.79996
3.272371	3.272467	3.407489	34.94662
3.245192	3.26694	3.400004	34.85873
3.371108	3.323416	3.372517	35.19748
3.25109	3.404963	3.399373	35.17208
3.225272	3.365335	3.36365	34.95084
3.240738	3.441557	3.351151	35.12401
3.369541	3.470604	3.352038	35.47115
3.215249	3.399773	3.427065	35.14291
3.348754	3.313712	3.368066	35.11764
3.240862	3.213834	3.401624	34.73667
3.393462	3.261144	3.339078	35.03706
3.427364	3.295111	3.378587	35.27188
3.348796	3.32126	3.375381	35.15023
3.253729	3.353878	3.419119	35.10932
3.235912	3.361426	3.353402	34.94315
3.184193	3.326921	3.408717	34.87556
3.329576	3.445062	3.354834	35.33401
3.204444	3.432796	3.457433	35.25791
3.183368	3.40685	3.474672	35.19278
3.258184	3.370727	3.48579	35.3017
3.358199	3.336828	3.400532	35.25984
3.285528	3.212284	3.337254	34.69019
3.28825	3.188427	3.39407	34.76822
3.242058	3.239511	3.467391	34.93926
3.276455	3.310948	3.333417	34.87772
3.376429	3.38616	3.350605	35.29841
3.286765	3.386429	3.339846	35.07939
3.310975	3.364257	3.411769	35.24113
3.231169	3.449308	3.463555	35.36585
3.341949	3.463258	3.426195	35.55691
3.315925	3.423429	3.383413	35.31934
3.331143	3.28116	3.425393	35.13331
3.191823	3.241196	3.419033	34.72733
3.308212	3.306905	3.395707	35.07454
3.325493	3.342556	3.382406	35.16121

3.417672	3.38461	3.363309	35.41299
3.176646	3.338715	3.434294	34.94077
3.245976	3.361561	3.363735	34.98805
3.253358	3.385216	3.417874	35.17432
3.322276	3.464067	3.326648	35.29797
3.189555	3.398358	3.459326	35.15418
3.247213	3.383397	3.495288	35.32619
3.133381	3.285811	3.458712	34.78387
3.373294	3.274488	3.401828	35.15936
3.376016	3.237759	3.37932	35.03577
3.295674	3.197997	3.442837	34.91203
3.279135	3.277723	3.387965	34.93021
3.266556	3.332245	3.422785	35.09808
3.341784	3.457732	3.30709	35.284
3.388348	3.392158	3.357835	35.3534
3.186255	3.380431	3.399731	34.97743
3.142455	3.410624	3.434175	35.023
3.344011	3.447353	3.388289	35.44375
3.291055	3.312027	3.340732	34.928
3.289158	3.2416	3.33635	34.76026
3.219786	3.206758	3.434516	34.74703
3.24845	3.384745	3.404829	35.13403
3.327885	3.380701	3.318276	35.10961
3.345249	3.340332	3.348696	35.12583
3.257235	3.35462	3.40014	35.0771
3.236407	3.377466	3.421199	35.12757
3.223086	3.455575	3.389227	35.19933
3.347063	3.458338	3.405034	35.51106
3.22156	3.401728	3.344347	34.98009
3.285652	3.31452	3.457365	35.1767
3.210424	3.245779	3.37059	34.67209
3.301036	3.210532	3.381861	34.81782
3.246017	3.221045	3.397395	34.75446
3.410248	3.272264	3.425649	35.28741
3.267092	3.34013	3.456871	35.19103
3.358405	3.389327	3.359216	35.28475
3.362901	3.357315	3.363786	35.23457
3.341207	3.385486	3.359898	35.24023
3.227705	3.39081	3.348218	34.97812
3.234139	3.45881	3.445207	35.353
3.175697	3.366548	3.428446	34.98678
3.364056	3.273612	3.334986	34.99107
3.308047	3.205208	3.414054	34.89191
3.362282	3.333121	3.386209	35.22934
3.336464	3.349565	3.492133	35.44048
3.290725	3.402739	3.341397	35.12711
3.322441	3.325505	3.364946	35.07907
3.301531	3.387912	3.332889	35.09971
3.148765	3.426596	3.405733	35.00953
3.248533	3.436772	3.391529	35.2189

3.284126	3.384812	3.374017	35.14481
3.218755	3.320047	3.39939	34.91571
3.257648	3.327123	3.391205	34.99833
3.179739	3.223	3.455131	34.74006
3.249811	3.211475	3.358296	34.65633
3.252245	3.175285	3.426041	34.73066
3.38641	3.355833	3.399032	35.35982
3.302685	3.404895	3.423995	35.33861
3.324173	3.434885	3.397497	35.39323
3.312749	3.350711	3.369976	35.12399
3.303758	3.376186	3.298803	35.00439
3.258266	3.458675	3.399134	35.30471
3.293323	3.438659	3.333639	35.19438
3.259586	3.307107	3.418334	35.01813
3.229148	3.194155	3.31079	34.46938
3.29918	3.263031	3.420807	35.01374
3.416187	3.406917	3.353623	35.43735
3.323266	3.412511	3.390574	35.32718
3.315058	3.324966	3.381111	35.09709
3.253235	3.273073	3.39939	34.88838
3.385667	3.380499	3.35006	35.30504
3.250183	3.47667	3.34844	35.21552
3.175903	3.430033	3.472371	35.22212
3.176481	3.386295	3.378229	34.92186
3.191039	3.276241	3.414583	34.79253
3.297654	3.267816	3.39997	34.97529
3.290725	3.252653	3.38921	34.90345
3.221766	3.220236	3.4456	34.80508
3.241109	3.243487	3.367725	34.72792
3.415857	3.351385	3.403806	35.42493
3.30747	3.403615	3.351509	35.18775
3.246306	3.335682	3.364383	34.93359
3.234098	3.344443	3.362183	34.92125
3.333989	3.478354	3.358944	35.42545
3.343558	3.457125	3.309852	35.29259
3.351188	3.34788	3.300883	35.05076
3.102242	3.242072	3.439495	34.5781
3.252451	3.266064	3.354561	34.77331
3.27167	3.365133	3.453051	35.24737
3.410826	3.446545	3.341807	35.48644
3.278063	3.351452	3.418556	35.156
3.247584	3.225965	3.378382	34.72707
3.253771	3.351115	3.415828	35.09617
3.361045	3.482331	3.331661	35.43365
3.204609	3.418104	3.441592	35.1915
3.253729	3.323551	3.379201	34.9557
3.087848	3.246587	3.44091	34.55959
3.177223	3.287765	3.389568	34.73281
3.343063	3.344443	3.384981	35.20939
3.270845	3.33521	3.416902	35.10108

3.169304	3.290191	3.433732	34.81738
3.285611	3.341006	3.397412	35.10342
3.341372	3.320653	3.345507	35.06734
3.455822	3.398762	3.345285	35.48796
3.229643	3.379488	3.445958	35.17134
3.189225	3.389462	3.315735	34.81999
3.221601	3.385351	3.402647	35.07186
3.305201	3.380027	3.265399	34.9429
3.255132	3.294976	3.38834	34.91627
3.249151	3.300031	3.422427	34.98878
3.198958	3.41743	3.522093	35.35371
3.404227	3.46218	3.439751	35.72039
3.498839	3.424709	3.399543	35.75742
3.301778	3.319575	3.369703	35.03131
3.277651	3.355967	3.384453	35.09039
3.285776	3.451397	3.439614	35.43748
3.352961	3.44796	3.418948	35.53169
3.2607	3.293156	3.3474	34.83493
3.302892	3.225898	3.410763	34.91868
3.039635	3.24962	3.435863	34.44975
3.265525	3.356574	3.425496	35.15495
3.189349	3.303535	3.452846	34.93219
3.22919	3.252114	3.380087	34.74776
3.18209	3.3003	3.490036	34.99057
3.394658	3.366616	3.38655	35.37414
3.301737	3.405906	3.422648	35.3358
3.378821	3.414667	3.320953	35.30114
3.212115	3.322136	3.410917	34.93096
3.361663	3.357989	3.392262	35.29561
3.198298	3.388788	3.366702	34.94982
3.262927	3.31762	3.34121	34.87977
3.148435	3.24679	3.377274	34.55336
3.222055	3.307511	3.376779	34.84606
3.276083	3.415611	3.405034	35.2624
3.37482	3.429764	3.380633	35.45591
3.318317	3.389597	3.422085	35.33516
3.371149	3.334941	3.358944	35.19309
3.288168	3.448903	3.373727	35.29317
3.277403	3.467234	3.449794	35.47606
3.270928	3.368907	3.32259	34.9687
3.166046	3.226369	3.407916	34.61423
3.272413	3.242881	3.355516	34.76835
3.268825	3.340534	3.454637	35.19082
3.186709	3.391282	3.433698	35.07643
3.138124	3.306905	3.459633	34.84238
3.324008	3.293561	3.384453	35.05529
3.300252	3.299155	3.377615	35.00062
3.379522	3.372883	3.426161	35.44137
3.323472	3.434683	3.418471	35.43712
3.234593	3.41925	3.451158	35.28049

3.332174	3.355765	3.365628	35.16802
3.431571	3.318699	3.34474	35.25864
3.204856	3.243689	3.353146	34.6172
3.251915	3.263975	3.403602	34.87481
3.153425	3.328403	3.372312	34.7319
3.292003	3.409545	3.439973	35.36036
3.368262	3.420935	3.372858	35.40526
3.299963	3.355361	3.45803	35.29876
3.183451	3.304411	3.440092	34.89332
3.345661	3.374972	3.366566	35.24156
3.389379	3.487116	3.440655	35.74443
3.375398	3.424035	3.419766	35.53023
3.156766	3.209184	3.43646	34.61877
3.1592	3.17232	3.306288	34.25882
3.204526	3.320316	3.386703	34.85744
3.31444	3.461573	3.335617	35.29499
3.183946	3.361696	3.483812	35.11528
3.186379	3.316475	3.381094	34.79708
3.261854	3.276106	3.449931	35.02439
3.4637	3.37996	3.344143	35.46157
3.337866	3.417498	3.409808	35.41208
3.165221	3.343163	3.397787	34.84568
3.231705	3.343095	3.425649	35.05186
3.26602	3.362639	3.332462	34.96585
3.452976	3.293561	3.277437	35.1033
3.265649	3.270782	3.365321	34.83602
3.192689	3.342017	3.384316	34.87379
3.13466	3.400447	3.430611	34.9759
3.420518	3.435559	3.316673	35.42865
3.269732	3.391753	3.440109	35.27304
3.296086	3.323821	3.356215	34.99866
3.201227	3.368637	3.511367	35.22851
3.327225	3.534493	3.361945	35.53999
3.454997	3.376253	3.379252	35.51121
3.403237	3.235804	3.332735	34.98915
3.152353	3.12447	3.470376	34.49804
3.198628	3.235804	3.414191	34.71984
3.182585	3.457799	3.389773	35.11682
3.260205	3.368772	3.411718	35.13987
3.187451	3.289248	3.402937	34.78765
3.148188	3.281969	3.495646	34.88861
3.19801	3.345926	3.423057	34.97869
3.369995	3.444388	3.43143	35.58842
3.312419	3.494933	3.408717	35.52338
3.238511	3.376657	3.443434	35.17903
3.254761	3.300165	3.360495	34.86591
3.283012	3.230952	3.392365	34.84603
3.405423	3.216665	3.302384	34.8857
3.29155	3.331099	3.374017	35.04358
3.122081	3.437581	3.449385	35.07066

3.233933	3.430033	3.378706	35.14419
3.342898	3.379218	3.426246	35.37532
3.340382	3.353811	3.369652	35.19049
3.31246	3.353878	3.429332	35.26009
3.220941	3.471885	3.415401	35.28755
3.272454	3.439468	3.445429	35.39497
3.306274	3.227245	3.25365	34.58545
3.317533	3.136803	3.415811	34.7669
3.199164	3.220236	3.38302	34.6188
3.245605	3.440142	3.407387	35.25454
3.254802	3.376186	3.389534	35.09575
3.313285	3.345791	3.402101	35.18466
3.144929	3.258179	3.42836	34.68232
3.297612	3.344241	3.36677	35.06973
3.201763	3.415341	3.503115	35.31377
3.289611	3.477209	3.359421	35.32694
3.337577	3.344915	3.461185	35.36507
3.469515	3.308185	3.349241	35.32847
3.2753	3.209184	3.453409	34.91505
3.331473	3.26876	3.317952	34.87196
3.234922	3.326921	3.427559	35.0277
3.293571	3.418644	3.346053	35.17829
3.26305	3.405367	3.377956	35.15228
3.235252	3.388788	3.400959	35.10554
3.258307	3.339254	3.406552	35.05987
3.31976	3.375242	3.405256	35.27012
3.25109	3.467639	3.426877	35.36929
3.281198	3.442164	3.426519	35.37864
3.266309	3.271321	3.417482	34.95271
3.205475	3.137949	3.386618	34.46052
3.380677	3.217608	3.344058	34.92478
3.260287	3.380634	3.397702	35.13533
3.257441	3.399975	3.393166	35.16149
3.211991	3.302592	3.437654	34.94642
3.263628	3.313577	3.373267	34.94256
3.244037	3.334536	3.39569	34.99459
3.306109	3.404289	3.404369	35.30185
3.138619	3.366481	3.485227	35.02972
3.208444	3.425316	3.356812	35.03025
3.2534	3.328673	3.430765	35.07895
3.432396	3.353339	3.346172	35.33933
3.294643	3.247666	3.427491	34.98483
3.249316	3.223471	3.34358	34.6493
3.240903	3.288574	3.380548	34.85411
3.299262	3.384138	3.386073	35.2028
3.213311	3.433942	3.383123	35.1173
3.289199	3.415476	3.386755	35.25081
3.153261	3.35563	3.419494	34.89426
3.288704	3.406243	3.422375	35.30744
3.34727	3.461169	3.423995	35.55917

3.394782	3.427472	3.425786	35.5933
3.289488	3.253664	3.412059	34.95292
3.280331	3.209319	3.339437	34.67711
3.309738	3.260201	3.396286	34.97701
3.333247	3.433942	3.316928	35.23482
3.17879	3.375242	3.383361	34.91396
3.13565	3.235872	3.441047	34.64099
3.252905	3.278802	3.421352	34.94822
3.238346	3.363111	3.379457	35.00913
3.300005	3.41278	3.429332	35.36166
3.209104	3.466156	3.453256	35.33192
3.208692	3.374905	3.437654	35.09734
3.227499	3.219495	3.339709	34.58443
3.486837	3.281497	3.334867	35.27656
3.256328	3.309196	3.396048	34.96684
3.253111	3.434548	3.384453	35.20857
3.280703	3.419385	3.354612	35.17049
3.358776	3.384879	3.352788	35.26178
3.295592	3.378881	3.36943	35.14688
3.248285	3.344039	3.339675	34.90217
3.139691	3.37349	3.440092	34.94869
3.27299	3.420194	3.436835	35.3352
3.250801	3.389327	3.411786	35.1644
3.346898	3.215923	3.363189	34.88907
3.292045	3.156078	3.421762	34.76633
3.299798	3.293224	3.386158	35.00534
3.30351	3.405839	3.43426	35.36492
3.346321	3.457193	3.374699	35.4406
3.219415	3.282037	3.440382	34.92367
3.232613	3.275769	3.365952	34.77606
3.112471	3.305557	3.42229	34.70167
3.25307	3.417093	3.385186	35.17191
3.294478	3.47977	3.487905	35.62416
3.310852	3.38434	3.454142	35.37744
3.268701	3.198131	3.407234	34.77547
3.344465	3.179463	3.343375	34.76069
3.270103	3.293898	3.436562	35.0521
3.355271	3.414735	3.325557	35.25985
3.235376	3.435896	3.397889	35.20212
3.26437	3.392832	3.339488	35.04363
3.341578	3.40038	3.404642	35.37146
3.23187	3.40375	3.359404	35.03999
3.302685	3.417835	3.440109	35.40214
3.228695	3.467841	3.487853	35.4541
3.275753	3.369379	3.445753	35.24962
3.23649	3.204332	3.446111	34.80361
3.398164	3.146845	3.352106	34.82588
3.218054	3.243689	3.453392	34.86529
3.422333	3.397212	3.332275	35.38288
3.294973	3.349161	3.457979	35.27418

3.356096	3.379083	3.318685	35.16866
3.270845	3.322001	3.385254	35.00298
3.303098	3.421676	3.412622	35.35134
3.149796	3.442299	3.421182	35.07991
3.257771	3.421811	3.408666	35.24386
3.214754	3.328066	3.451141	35.03767
3.282971	3.315329	3.399936	35.04701
3.270186	3.236141	3.388937	34.82183
3.300005	3.283519	3.380292	34.97174
3.26635	3.323416	3.398691	35.02563
3.330731	3.336288	3.332599	35.05004
3.284456	3.348824	3.398691	35.12079
3.278476	3.399032	3.344671	35.09937
3.311305	3.405097	3.428923	35.36868
3.250801	3.479096	3.402851	35.34117
3.275135	3.491429	3.383498	35.37903
3.263628	3.391888	3.489559	35.36813
3.272495	3.333458	3.44398	35.16007
3.162623	3.208914	3.465141	34.69371
3.391689	3.225358	3.33444	34.94478
3.258184	3.253057	3.423722	34.90865
3.377377	3.32746	3.360171	35.19304
3.293117	3.349161	3.46717	35.29022
3.328875	3.366009	3.374495	35.20259
3.284662	3.303737	3.278562	34.75994
3.307098	3.358731	3.414463	35.22646
3.176109	3.412511	3.427866	35.08692
3.24944	3.389462	3.440194	35.22384
3.194463	3.365133	3.425786	35.0189
3.367066	3.381644	3.445548	35.47569
3.384265	3.287226	3.426655	35.2655
3.337124	3.285137	3.426945	35.15848
3.283383	3.245442	3.419494	34.93785
3.3118	3.370861	3.364809	35.15468
3.246677	3.396336	3.413304	35.17403
3.29456	3.370457	3.337322	35.05599
3.267464	3.37632	3.43102	35.21446
3.257689	3.412511	3.375177	35.1501
3.336546	3.472559	3.378024	35.46009
3.289529	3.417767	3.42345	35.33679
3.290395	3.360281	3.441882	35.25328
3.175367	3.269501	3.426331	34.76921
3.303593	3.200221	3.323596	34.67344
3.372304	3.329145	3.384436	35.23869
3.35725	3.414667	3.373864	35.36967
3.251956	3.380903	3.460281	35.25455
3.310274	3.314251	3.398623	35.10149
3.213806	3.234726	3.345183	34.59976
3.326524	3.305826	3.38568	35.0903
3.155694	3.423631	3.445753	35.10571

3.230633	3.492709	3.438182	35.4041
3.176893	3.391686	3.457092	35.10701
3.31213	3.227852	3.396917	34.91288
3.332546	3.200962	3.401214	34.90812
3.320008	3.264986	3.408376	35.03637
3.234758	3.390001	3.421267	35.15152
3.370077	3.451262	3.32114	35.36245
3.43718	3.427809	3.364877	35.55355
3.353704	3.297267	3.272987	34.88458
3.160024	3.341747	3.406637	34.85058
3.248574	3.447758	3.387232	35.23361
3.288291	3.456654	3.409058	35.38765
3.311099	3.327932	3.348218	35.02299
3.305779	3.19712	3.397804	34.83373
3.109171	3.198671	3.425956	34.46873
3.305531	3.357922	3.357102	35.09582
3.387193	3.455777	3.453835	35.69994
3.399236	3.433268	3.357238	35.46581
3.198587	3.281295	3.485944	34.97614
3.284043	3.330358	3.366378	35.00884
3.272371	3.334604	3.38834	35.04063
3.338815	3.391416	3.415214	35.36894
3.149796	3.350172	3.395366	34.82198
3.205722	3.357383	3.445975	35.07073
3.16716	3.295178	3.422188	34.79835
3.333041	3.215249	3.398128	34.9337
3.212156	3.194357	3.400942	34.62981
3.355931	3.304276	3.388545	35.15749
3.209846	3.391753	3.439137	35.13996
3.378037	3.493248	3.303066	35.43215
3.410454	3.42235	3.371289	35.49719
3.36917	3.361494	3.262841	35.03667
3.181883	3.399032	3.439887	35.09636
3.24779	3.470335	3.358108	35.21758
3.209517	3.394651	3.407575	35.07655
3.304088	3.325101	3.365611	35.0395
3.247749	3.18344	3.419374	34.72408
3.114285	3.19921	3.49672	34.63584
3.302438	3.362033	3.382935	35.15454
3.314728	3.366885	3.457212	35.35446
3.337495	3.365807	3.347536	35.16205
3.212321	3.311285	3.474025	35.04569
3.268206	3.368233	3.394002	35.11744
3.257194	3.378275	3.42952	35.19299
3.369747	3.380768	3.388477	35.35483
3.148394	3.382992	3.398981	34.8986
3.240944	3.43347	3.477691	35.3835
3.169469	3.292887	3.401231	34.75256
3.38047	3.192201	3.352873	34.88805
3.310522	3.202647	3.407421	34.87721

3.272124	3.28945	3.363582	34.8872
3.323472	3.384812	3.469966	35.44068
3.430829	3.467234	3.408734	35.72179
3.401587	3.398762	3.347536	35.37427
3.393957	3.343904	3.295904	35.12471
3.183533	3.405097	3.405716	35.03851
3.206093	3.453823	3.384112	35.14715
3.172604	3.394988	3.365935	34.90551
3.303552	3.256562	3.40355	34.97141
3.243831	3.201299	3.398503	34.70892
3.162128	3.292146	3.484034	34.91596
3.23616	3.404963	3.444406	35.23791
3.393297	3.40348	3.381247	35.44018
3.35358	3.327527	3.416919	35.26524
3.338196	3.358124	3.341875	35.1344
3.242388	3.282373	3.445037	34.98483
3.186668	3.342017	3.364315	34.81688
3.300458	3.425113	3.389415	35.30233
3.285817	3.433268	3.344467	35.18985
3.2574	3.313105	3.422171	35.03486
3.217683	3.232974	3.36972	34.65807
3.305284	3.210734	3.42345	34.9185
3.269938	3.358124	3.38996	35.09028
3.281693	3.409613	3.397122	35.24425
3.284043	3.422957	3.390046	35.2631
3.440232	3.413724	3.422051	35.65446
3.327266	3.373894	3.346036	35.15408
3.39074	3.367155	3.353129	35.29366
3.245687	3.407995	3.335805	35.02788
3.22123	3.406311	3.400686	35.1126
3.126576	3.251103	3.432385	34.63551
3.27134	3.230615	3.343103	34.71204
3.262679	3.223876	3.452983	34.91865
3.274475	3.387642	3.408478	35.20525
3.265484	3.466897	3.52373	35.61095
3.372469	3.357787	3.406961	35.35094
3.43252	3.275365	3.369788	35.22073
3.336216	3.343365	3.387386	35.19732
3.239336	3.382318	3.408257	35.11628
3.227416	3.386834	3.386499	35.05251
3.234386	3.325371	3.365969	34.88845
3.26635	3.367357	3.324551	34.95959
3.320709	3.26384	3.399117	35.01515
3.178996	3.21006	3.401913	34.59376
3.253565	3.190112	3.385049	34.67632
3.223374	3.344039	3.387317	34.95187
3.338732	3.44176	3.449982	35.55488
3.358446	3.462719	3.349855	35.42487
3.407031	3.375242	3.380684	35.40723
3.284909	3.370861	3.359012	35.0832

3.351064	3.455306	3.315684	35.31778
3.297984	3.451936	3.396986	35.37213
3.270928	3.363111	3.34815	35.01192
3.098984	3.236074	3.406006	34.48462
3.135567	3.202647	3.335259	34.33681
3.221972	3.306837	3.451568	35.00796
3.320338	3.490485	3.375296	35.45788
3.215167	3.385216	3.476156	35.21825
3.395483	3.356102	3.369498	35.31566
3.263298	3.289854	3.369447	34.88161
3.371191	3.391753	3.355925	35.31082
3.278311	3.368705	3.422137	35.2021
3.188317	3.358191	3.357648	34.84128
3.090571	3.32746	3.443025	34.74702
3.277238	3.320451	3.325881	34.88373
3.255833	3.250429	3.448345	34.9516
3.20997	3.285069	3.377342	34.77179
3.148064	3.301176	3.487768	34.91312
3.27596	3.396404	3.460434	35.34128
3.492529	3.455441	3.341977	35.68494
3.441758	3.419722	3.354118	35.52235
3.369005	3.360281	3.377615	35.28465
3.264412	3.32874	3.430458	35.1025
3.271093	3.449106	3.324005	35.14754
3.363066	3.393573	3.398571	35.39029
3.356426	3.278936	3.373625	35.07053
3.067845	3.181822	3.404778	34.2952
3.265484	3.286754	3.409024	34.96616
3.302026	3.438862	3.416493	35.39504
3.283713	3.479972	3.475985	35.57499
3.210465	3.321597	3.397003	34.89575
3.29522	3.251912	3.430969	35.00298
3.317451	3.388316	3.423995	35.33464
3.419528	3.404019	3.353197	35.43738
3.261731	3.300233	3.413236	34.99664
3.227292	3.317553	3.35879	34.84014
3.190338	3.293022	3.398077	34.79159
3.241521	3.252788	3.351901	34.71456
3.208197	3.278128	3.394002	34.78916
3.219621	3.295717	3.425769	34.92208
3.167242	3.358394	3.439563	34.97477
3.350487	3.442029	3.422972	35.52211
3.381089	3.409074	3.378996	35.4208
3.502963	3.393304	3.340784	35.56926
3.321327	3.394247	3.42345	35.3549
3.247955	3.398088	3.342148	35.02505
3.299633	3.395325	3.410354	35.28117
3.266597	3.296863	3.324141	34.80508
3.224364	3.174746	3.381298	34.57066
3.217477	3.268221	3.383702	34.76527

3.168768	3.401728	3.497811	35.20025
3.32372	3.482937	3.438932	35.58794
3.368304	3.411702	3.407029	35.45989
3.242635	3.315733	3.372159	34.89895
3.33733	3.333727	3.420926	35.25203
3.31609	3.391753	3.404079	35.29563
3.278434	3.431583	3.379934	35.24758
3.265855	3.348554	3.348627	34.97004
3.293571	3.295246	3.424422	35.07982
3.171985	3.239039	3.336759	34.49932
3.323513	3.229335	3.412298	34.97465
3.167448	3.302457	3.444389	34.86345
3.222632	3.351317	3.437858	35.07669
3.184564	3.424439	3.480351	35.24628
3.359725	3.442838	3.397463	35.4883
3.398329	3.407524	3.387999	35.4748
3.465391	3.420059	3.339641	35.54311
3.291467	3.339928	3.467136	35.26634
3.390451	3.441288	3.351509	35.45161
3.255503	3.305826	3.410303	34.98884
3.279713	3.219091	3.323851	34.66305
3.188524	3.229132	3.435249	34.7292
3.229478	3.366548	3.369839	34.97623
3.224983	3.454767	3.48695	35.41542
3.30516	3.448297	3.383685	35.35078
3.276042	3.410017	3.471961	35.39644
3.335185	3.364661	3.373386	35.21102
3.323802	3.348621	3.434516	35.28473
3.255874	3.392697	3.401709	35.16083
3.254884	3.311825	3.412622	35.00567
3.174047	3.236209	3.397139	34.62968
3.277568	3.247801	3.305674	34.68139
3.176975	3.186135	3.43854	34.61711
3.170459	3.295448	3.400754	34.75928
3.160272	3.358394	3.473445	35.03362
3.293571	3.445938	3.331644	35.20647
3.27299	3.407995	3.394547	35.21605
3.388637	3.438188	3.376916	35.49642
3.233809	3.412982	3.423467	35.20452
3.432767	3.445331	3.357631	35.56637
3.281033	3.370861	3.388835	35.13994
3.430004	3.354957	3.368782	35.38708
3.287632	3.223539	3.362678	34.775
3.270227	3.158167	3.316911	34.4939
3.145053	3.195166	3.392774	34.46697
3.314275	3.467234	3.387113	35.41962
3.180852	3.469391	3.481561	35.33911
3.277774	3.415813	3.411241	35.28011
3.225808	3.335345	3.408188	34.98383
3.401711	3.403278	3.359626	35.41086

3.350899	3.418846	3.394411	35.40985
3.275011	3.362774	3.458371	35.26115
3.204774	3.318766	3.351918	34.77852
3.186008	3.2385	3.441865	34.75865
3.273856	3.233378	3.266439	34.55593
3.311388	3.199142	3.378996	34.80928
3.135072	3.291404	3.412929	34.69968
3.146249	3.340871	3.459599	34.93436
3.279465	3.456721	3.395366	35.33856
3.337619	3.420598	3.360222	35.30988
3.37581	3.392697	3.437091	35.50048
3.295468	3.440883	3.394053	35.33604
3.304335	3.43657	3.360904	35.27351
3.343846	3.39883	3.366702	35.29006
3.375315	3.283924	3.352464	35.07646
3.214053	3.20541	3.373847	34.59887
3.278022	3.172117	3.410439	34.74598
3.173222	3.361965	3.433203	34.98175
3.301201	3.41743	3.359336	35.22137
3.219167	3.420059	3.445071	35.23522
3.198628	3.360348	3.488876	35.15552
3.220776	3.331167	3.397616	34.94057
3.375769	3.463191	3.457007	35.6981
3.328669	3.410961	3.381315	35.31536
3.370366	3.318699	3.431737	35.31505
3.18609	3.192942	3.414259	34.59883
3.324833	3.247194	3.373983	34.93281
3.304871	3.272062	3.289527	34.75884
3.247955	3.275702	3.359898	34.79623
3.187575	3.309803	3.435266	34.90358
3.215126	3.326786	3.41356	34.9535
3.307263	3.446208	3.461355	35.52066
3.384141	3.468717	3.329325	35.44928
3.378986	3.431583	3.476207	35.67801
3.299922	3.388249	3.376063	35.19134
3.254554	3.361426	3.439904	35.17308
3.362406	3.358259	3.312018	35.12234
3.431117	3.305624	3.37313	35.29115
3.167366	3.191122	3.375262	34.46863
3.247543	3.25737	3.389364	34.81967
3.196566	3.413859	3.405443	35.08557
3.274805	3.387373	3.355465	35.08945
3.250884	3.334671	3.404556	35.02925
3.233974	3.386429	3.434226	35.17034
3.149755	3.406917	3.438114	35.03947
3.373748	3.454767	3.440092	35.63827
3.286518	3.401526	3.455319	35.36438
3.387771	3.291606	3.366378	35.15093
3.192442	3.214778	3.440945	34.71883
3.338155	3.287698	3.359626	35.01911

3.26305	3.239309	3.348372	34.72444
3.302026	3.283856	3.37313	34.96124
3.144476	3.312364	3.444525	34.83517
3.260328	3.391484	3.423381	35.21531
3.263133	3.474243	3.434908	35.42763
3.382161	3.452677	3.358671	35.47405
3.384554	3.442434	3.440518	35.63586
3.253152	3.391619	3.444389	35.24585
3.249028	3.354013	3.386755	35.02856
3.322523	3.310611	3.361194	35.03847
3.390245	3.235939	3.356778	35.01361
3.24148	3.24032	3.409467	34.81309
3.202051	3.267412	3.429247	34.82937
3.240573	3.460158	3.3969	35.26438
3.289446	3.433942	3.381162	35.27951
3.263504	3.363381	3.433016	35.18186
3.257483	3.378073	3.472422	35.287
3.176274	3.400784	3.440501	35.08927
3.309779	3.494866	3.418283	35.53838
3.363231	3.362572	3.407677	35.34277
3.334113	3.261616	3.35289	34.93851
3.210919	3.183103	3.408444	34.6189
3.366489	3.210869	3.387778	34.97463
3.224034	3.22893	3.331849	34.58029
3.324338	3.348082	3.370402	35.14452
3.176893	3.387238	3.422853	35.02241
3.198505	3.367155	3.408734	34.99487
3.210176	3.420598	3.417209	35.1558
3.323225	3.460697	3.350469	35.34476
3.281816	3.407187	3.453136	35.36171
3.40534	3.407389	3.365764	35.44121
3.220075	3.269501	3.462822	34.94677
3.323967	3.327123	3.323886	34.99615
3.35824	3.202512	3.359609	34.87671
3.258019	3.329751	3.344927	34.90369
3.180605	3.340467	3.407626	34.89495
3.25938	3.416757	3.402783	35.22346
3.26037	3.359607	3.39482	35.08323
3.313367	3.413993	3.384248	35.29494
3.29786	3.420733	3.438455	35.39431
3.151735	3.369985	3.488194	35.07255
3.289529	3.370861	3.379133	35.1373
3.270722	3.284867	3.416987	34.9909
3.342444	3.23695	3.331883	34.85685
3.184358	3.228795	3.415811	34.67684
3.288869	3.206016	3.397531	34.8156
3.241233	3.258516	3.334372	34.68812
3.3443	3.375714	3.346547	35.19643
3.151611	3.399032	3.445975	35.04348
3.294725	3.461843	3.423211	35.44402

3.200196	3.427742	3.386362	35.08214
3.323637	3.437514	3.309562	35.20551
3.324627	3.341208	3.39262	35.17871
3.301984	3.35617	3.367197	35.10631
3.241315	3.28116	3.413031	34.90984
3.277651	3.26721	3.365884	34.85568
3.324008	3.268019	3.311387	34.83965
3.386781	3.326921	3.305811	35.09354
3.126947	3.376792	3.42819	34.90201
3.222261	3.410017	3.395366	35.11132
3.237603	3.361898	3.390233	35.02842
3.320338	3.44951	3.390659	35.40188
3.295303	3.425316	3.417106	35.35205
3.171284	3.372209	3.48961	35.12326
3.216652	3.266469	3.417294	34.83309
3.341248	3.217473	3.429196	35.02445
3.324874	3.21161	3.341227	34.78345
3.276207	3.195975	3.393319	34.75675
3.294024	3.248677	3.36261	34.8438
3.232242	3.310814	3.365884	34.85174
3.354116	3.414937	3.345234	35.3008
3.328463	3.377466	3.414242	35.31367
3.31015	3.467774	3.386755	35.411
3.193803	3.455845	3.372431	35.09916
3.29786	3.407187	3.413833	35.31084
3.397092	3.411702	3.422716	35.55715
3.314564	3.289787	3.386277	35.03038
3.208238	3.276443	3.407643	34.8154
3.28194	3.251372	3.381997	34.86567
3.263587	3.292213	3.35879	34.86409
3.370283	3.360483	3.369311	35.26972
3.175986	3.362033	3.397327	34.90948
3.206877	3.377331	3.420585	35.06135
3.171243	3.375175	3.386772	34.90477
3.279424	3.425787	3.397003	35.2744
3.277733	3.428011	3.492355	35.48408
3.34529	3.432459	3.459872	35.57051
3.161179	3.318968	3.446316	34.89006
3.317451	3.229267	3.390574	34.91374
3.412187	3.240994	3.403329	35.17445
3.339722	3.235467	3.354288	34.89665
3.243295	3.247733	3.420449	34.85729
3.24911	3.386227	3.314575	34.94134
3.236655	3.3621	3.362746	34.96668
3.358735	3.354417	3.310909	35.10349
3.230922	3.413387	3.449095	35.25513
3.153508	3.440816	3.404693	35.04872
3.327266	3.413589	3.384095	35.32412
3.371149	3.34195	3.370726	35.23418
3.309738	3.253866	3.394803	34.95992

3.236572	3.289585	3.411855	34.91531
3.246347	3.315059	3.397344	34.96067
3.281198	3.345521	3.429315	35.17341
3.412434	3.369244	3.336248	35.30876
3.244079	3.341343	3.368697	34.95054
3.221518	3.343702	3.434056	35.04929
3.107563	3.425046	3.38875	34.8789
3.290189	3.460899	3.363309	35.30104
3.218136	3.351519	3.443911	35.08054
3.275094	3.283924	3.418829	35.00242
3.240738	3.271321	3.443724	34.95418
3.352796	3.267749	3.35983	35.00796
3.310109	3.283924	3.407285	35.05375
3.408104	3.288169	3.359199	35.17218
3.247873	3.328269	3.445139	35.09741
3.220199	3.415004	3.354237	35.02778
3.264576	3.375444	3.387454	35.11095
3.299922	3.423968	3.39378	35.3082
3.18972	3.370727	3.400191	34.96479
3.248038	3.408467	3.420312	35.21886
3.183451	3.303266	3.45803	34.93004
3.407361	3.229267	3.384521	35.09712
3.349167	3.276915	3.381844	35.0682
3.259792	3.359472	3.38077	35.05095
3.326895	3.407861	3.43143	35.41429
3.227705	3.346263	3.369839	34.92799
3.346403	3.333458	3.341619	35.09785
3.267958	3.398493	3.393865	35.18277
3.234304	3.461169	3.439546	35.34614
3.176316	3.450049	3.395332	35.09832
3.294602	3.408669	3.37666	35.22567
3.188194	3.287428	3.45653	34.9025
3.226921	3.308185	3.357085	34.81511
3.165634	3.234187	3.469028	34.76407
3.324379	3.223876	3.377973	34.88955
3.264453	3.262088	3.43779	34.97287
3.33502	3.334806	3.37313	35.14481
3.340794	3.363111	3.425291	35.3334
3.311718	3.442568	3.380701	35.34607
3.229808	3.38279	3.430014	35.14406
3.285734	3.448229	3.380087	35.30028
3.198834	3.454901	3.385373	35.1364
3.26037	3.477815	3.366105	35.27894
3.250141	3.256966	3.453256	34.96419
3.284992	3.211947	3.418351	34.86563
3.370325	3.234524	3.380855	35.01961
3.364014	3.363852	3.400959	35.33259
3.324916	3.349969	3.437909	35.29754
3.252657	3.356709	3.392552	35.05507
3.278311	3.323821	3.34861	34.94315

3.244862	3.429359	3.419835	35.25656
3.253647	3.490013	3.322982	35.19661
3.132763	3.450588	3.476855	35.18253
3.179038	3.365403	3.450272	35.03931
3.193597	3.296459	3.438591	34.89483
3.303015	3.23385	3.396184	34.90446
3.255544	3.230278	3.373335	34.74287
3.271299	3.205882	3.468875	34.9329
3.210176	3.321664	3.349326	34.791
3.384801	3.391888	3.389756	35.41486
3.329246	3.392899	3.352566	35.21425
3.323472	3.36392	3.392637	35.22588
3.222137	3.341276	3.406006	34.984
3.314852	3.450251	3.375842	35.3591
3.246842	3.481994	3.345694	35.21386
3.373294	3.352193	3.374	35.26844
3.124844	3.209453	3.447322	34.57331
3.305655	3.223876	3.415691	34.93108
3.240738	3.418509	3.420534	35.22534
3.399401	3.464269	3.415759	35.66194
3.304789	3.321125	3.417601	35.14603
3.312048	3.28918	3.365406	34.97791
3.187493	3.367357	3.398418	34.94868
3.356343	3.522362	3.384981	35.62751
3.183203	3.452947	3.448567	35.23613
3.165757	3.345858	3.390455	34.83672
3.14662	3.268558	3.47428	34.80914
3.263999	3.301918	3.414992	35.00912
3.400721	3.315599	3.41934	35.34754
3.328256	3.304748	3.442309	35.21557
3.289859	3.267682	3.477605	35.12773
3.329164	3.381712	3.381656	35.25322
3.311965	3.422889	3.402323	35.35086
3.375892	3.394719	3.308181	35.22318
3.159323	3.343904	3.399032	34.83713
3.124431	3.368098	3.353129	34.71335
3.314646	3.428213	3.392348	35.34655
3.266721	3.376051	3.348491	35.03177
3.278022	3.283452	3.391205	34.94739
3.202794	3.213093	3.365918	34.57371
3.303675	3.334132	3.41651	35.16965
3.249688	3.397415	3.422853	35.20385
3.45974	3.449577	3.321021	35.55459
3.268742	3.323281	3.436494	35.11324
3.299798	3.317014	3.382253	35.04883
3.238098	3.472154	3.428855	35.35508
3.333371	3.496146	3.376848	35.50216
3.201887	3.313779	3.392228	34.84945
3.257359	3.242544	3.396321	34.82393
3.136516	3.238096	3.477145	34.72669

3.289281	3.308859	3.395639	35.03727
3.246553	3.273949	3.384043	34.84213
3.381295	3.268086	3.380633	35.11651
3.252575	3.344039	3.472149	35.20124
3.40567	3.393775	3.42952	35.55158
3.348631	3.41123	3.395144	35.38984
3.358488	3.350643	3.340119	35.15857
3.145136	3.313712	3.355448	34.64477
3.253358	3.431853	3.39823	35.23335
3.177924	3.426663	3.345558	34.94185
3.293901	3.255686	3.420721	34.98594
3.280496	3.232367	3.326273	34.69909
3.211001	3.208443	3.424149	34.70883
3.353539	3.363718	3.400532	35.30846
3.285776	3.370524	3.377547	35.12489
3.385296	3.361022	3.408598	35.38965
3.359601	3.307444	3.379661	35.15301
3.312543	3.407928	3.458525	35.44231
3.219827	3.491024	3.387164	35.26522
3.279135	3.419115	3.478424	35.43723
3.170913	3.360752	3.34196	34.77451
3.223251	3.243555	3.393865	34.74618
3.214425	3.279408	3.437142	34.89993
3.228489	3.290932	3.457894	35.00126
3.234881	3.24962	3.468739	34.94862
3.290684	3.285137	3.391955	34.9804
3.255627	3.348689	3.480743	35.23688
3.457637	3.400245	3.330502	35.46284
3.248986	3.295178	3.458422	35.05653
3.293158	3.338782	3.32571	34.95826
3.211785	3.410826	3.409689	35.12151
3.340877	3.457328	3.363496	35.40449
3.241027	3.355361	3.356556	34.94797
3.219704	3.267682	3.351185	34.69785
3.208527	3.264379	3.394598	34.76113
3.328586	3.367559	3.449931	35.37032
3.366943	3.419317	3.397275	35.45224
3.27398	3.344645	3.412673	35.11932
3.271794	3.253327	3.402988	34.89366
3.358034	3.306096	3.370249	35.12605
3.298644	3.510434	3.375978	35.45556
3.294767	3.427203	3.402715	35.32354
3.194298	3.27159	3.40899	34.77725
3.207743	3.264851	3.334935	34.62997
3.268618	3.3003	3.445889	35.08326
3.28792	3.353474	3.39985	35.14107
3.231169	3.293291	3.458439	35.01348
3.104387	3.264312	3.433698	34.61875
3.342362	3.37039	3.428224	35.35916
3.368345	3.352733	3.339641	35.18365

3.423817	3.372075	3.422785	35.52908
3.259545	3.398965	3.40766	35.19557
3.300417	3.335951	3.381162	35.08921
3.2647	3.418239	3.390677	35.21186
3.400432	3.360685	3.318565	35.22513
3.176109	3.258988	3.377888	34.6419
3.106161	3.262897	3.387027	34.51747
3.147775	3.373962	3.405733	34.89226
3.33766	3.447623	3.424899	35.51051
3.409341	3.463797	3.408768	35.66736
3.308789	3.334536	3.413713	35.17561
3.19537	3.304748	3.434175	34.90718
3.387894	3.439805	3.325625	35.38617
3.358034	3.458541	3.434772	35.60053
3.341289	3.328808	3.33028	35.0517
3.099314	3.175824	3.431924	34.41026
3.176687	3.201838	3.363547	34.48683
3.277073	3.383936	3.464288	35.32488
3.288951	3.373153	3.398844	35.18416
3.222137	3.316812	3.496788	35.12902
3.187245	3.278936	3.418488	34.79866
3.308707	3.360618	3.351151	35.09565
3.47265	3.461439	3.380343	35.73848
3.339475	3.40503	3.382065	35.32766
3.149219	3.338108	3.438881	34.8895
3.284085	3.357787	3.399049	35.14036
3.213641	3.359809	3.360171	34.90571
3.378285	3.299289	3.33901	35.08714
3.170913	3.245374	3.363582	34.56948
3.1194	3.321192	3.38875	34.67767
3.167778	3.384205	3.474758	35.10935
3.437304	3.423765	3.307635	35.4198
3.346073	3.292752	3.393592	35.12177
3.326813	3.276847	3.384487	35.02495
3.261607	3.403143	3.505911	35.42408
3.389008	3.563877	3.359268	35.7335
3.399113	3.391484	3.404693	35.47794
3.344919	3.230346	3.365253	34.92079
3.089952	3.174004	3.390387	34.29498
3.185513	3.324225	3.406279	34.86721
3.170253	3.418846	3.377888	34.97868
3.315058	3.345993	3.415572	35.21844
3.259586	3.286889	3.420193	34.97798
3.204279	3.307309	3.483556	35.04025
3.307387	3.364661	3.37886	35.1622
3.422168	3.373962	3.444133	35.57629
3.337041	3.402671	3.360086	35.26912
3.246182	3.335749	3.378229	34.96375
3.21628	3.342421	3.39748	34.95505
3.246429	3.310005	3.356949	34.86145

3.333206	3.242746	3.31877	34.82065
3.222343	3.342421	3.435931	35.05239
3.181636	3.390945	3.454501	35.11009
3.266474	3.395864	3.38771	35.16032
3.385585	3.41487	3.390711	35.46892
3.420353	3.377466	3.365611	35.40827
3.274929	3.38744	3.417652	35.22587
3.215909	3.458136	3.488672	35.40671
3.402659	3.445062	3.433681	35.66625
3.212857	3.219091	3.357596	34.59064
3.310975	3.139431	3.396986	34.71714
3.133629	3.248744	3.406006	34.58809
3.185554	3.401054	3.39557	35.0119
3.222467	3.403008	3.403687	35.11465
3.271093	3.348015	3.433425	35.16575
3.216652	3.295852	3.438319	34.94333
3.325411	3.389866	3.423211	35.35372
3.278434	3.390069	3.46144	35.33504
3.369953	3.419789	3.425462	35.52149
3.385131	3.368435	3.39823	35.38283
3.334072	3.393573	3.394053	35.31701
3.293034	3.288304	3.374222	34.95369
3.295427	3.238568	3.299928	34.68769
3.261318	3.272332	3.395366	34.89564
3.261195	3.362033	3.314541	34.91478
3.214095	3.4053	3.498954	35.30968
3.226303	3.444388	3.345234	35.0857
3.323307	3.371872	3.494879	35.4665
3.352755	3.330156	3.374085	35.17551
3.323967	3.395056	3.431856	35.38082
3.287054	3.513062	3.407984	35.50595
3.307965	3.336423	3.404846	35.15854
3.204361	3.156482	3.346939	34.41184
3.356467	3.169152	3.310636	34.69279
3.217683	3.271658	3.429861	34.87418
3.300376	3.477141	3.358995	35.3494
3.182667	3.274556	3.431856	34.80831
3.306521	3.246318	3.376507	34.89636
3.301531	3.255416	3.355908	34.8603
3.350858	3.399841	3.428633	35.44304
3.183657	3.466763	3.506047	35.39304
3.31477	3.451734	3.402715	35.42093
3.317657	3.33548	3.393319	35.15246
3.454667	3.227178	3.32675	35.06967
3.246759	3.199075	3.352839	34.6106
3.216033	3.268019	3.39436	34.78498
3.159818	3.457125	3.384436	35.05389
3.283342	3.438525	3.314661	35.13075
3.231705	3.405637	3.391	35.11285
3.327679	3.35125	3.429725	35.28848

3.140228	3.313981	3.478083	34.9028
3.344341	3.427809	3.393166	35.41239
3.416971	3.52398	3.397804	35.79167
3.346321	3.339726	3.391665	35.22082
3.205145	3.205477	3.375228	34.58256
3.206135	3.15911	3.379798	34.49332
3.284497	3.338647	3.353214	34.99917
3.357498	3.532135	3.366702	35.61143
3.165097	3.379286	3.430611	34.99619
3.134907	3.209319	3.399032	34.48942
3.25274	3.283721	3.42183	34.95966
3.289776	3.379892	3.387027	35.17486
3.300829	3.441355	3.414804	35.39418
3.234139	3.465819	3.419238	35.31153
3.228447	3.285608	3.49493	35.07052
3.36884	3.176565	3.285792	34.68173
3.43087	3.201905	3.334372	34.97903
3.223044	3.308455	3.416152	34.93639
3.163448	3.432122	3.412605	35.06875
3.276166	3.431651	3.399236	35.28498
3.390781	3.413387	3.322453	35.32777
3.312543	3.362774	3.356352	35.12013
3.294602	3.365537	3.369021	35.11464
3.156271	3.428079	3.447066	35.11957
3.298726	3.491968	3.458866	35.59662
3.309656	3.370592	3.371971	35.16507
3.387069	3.187146	3.406296	35.00825
3.162169	3.113822	3.392228	34.32532
3.266845	3.242813	3.392757	34.83747
3.296086	3.429359	3.438677	35.40978
3.304046	3.415206	3.38389	35.27643
3.163695	3.298818	3.453256	34.86667
3.11544	3.339254	3.390046	34.71134
3.19339	3.389664	3.458456	35.14165
3.345744	3.470806	3.342028	35.39766
3.297654	3.30677	3.472149	35.21832
3.211702	3.354754	3.370931	34.91395
3.208815	3.294167	3.476531	35.00607
3.381832	3.275836	3.272935	34.89911
3.460689	3.29565	3.320833	35.21964
3.2355	3.249283	3.415828	34.83352
3.152724	3.355226	3.344347	34.72787
3.269361	3.356911	3.439137	35.19391
3.321987	3.434144	3.337936	35.25658
3.330154	3.446814	3.420807	35.48337
3.326565	3.441625	3.414037	35.44937
3.124761	3.409074	3.471143	35.06176
3.272124	3.455238	3.442616	35.42259
3.239171	3.354754	3.380207	34.9943
3.424848	3.232637	3.428429	35.23875

3.160024	3.166389	3.408035	34.47015
3.264576	3.280891	3.369788	34.86555
3.250636	3.332447	3.419971	35.05755
3.287054	3.360213	3.344006	35.03179
3.176027	3.311825	3.419306	34.84784
3.13466	3.358933	3.397412	34.81252
3.172026	3.386092	3.481868	35.13832
3.332669	3.368166	3.37903	35.22553
3.284703	3.352328	3.43484	35.20804
3.261524	3.404221	3.383975	35.1596
3.300953	3.366076	3.442564	35.29054
3.340959	3.35408	3.358193	35.16728
3.411156	3.273073	3.324994	35.07104
3.288869	3.189842	3.356897	34.69137
3.200938	3.337299	3.367162	34.844
3.254719	3.417093	3.454961	35.32811
3.325534	3.428079	3.3644	35.30895
3.302149	3.392427	3.307874	35.05623
3.3118	3.366279	3.455046	35.342
3.093499	3.407861	3.478714	35.0073
3.290436	3.470874	3.43796	35.48664
3.260658	3.346465	3.324653	34.90168
3.406124	3.304411	3.426246	35.34999
3.200855	3.207499	3.367862	34.56149
3.290931	3.249822	3.422444	34.97039
3.283713	3.4239	3.42258	35.33559
3.33069	3.395325	3.351236	35.21981
3.14262	3.295785	3.413901	34.72789
3.233726	3.266334	3.379474	34.78743
3.145218	3.264177	3.475593	34.79936
3.297035	3.399975	3.3474	35.14799
3.312543	3.475456	3.449504	35.57025
3.363478	3.36365	3.325522	35.16601
3.343929	3.18964	3.406756	34.92037
3.28825	3.177644	3.301156	34.54145
3.336959	3.267345	3.35243	34.95625
3.304995	3.407591	3.352652	35.19354
3.13202	3.473502	3.419289	35.10513
3.309037	3.429561	3.388869	35.32962
3.329782	3.332986	3.338448	35.05353
3.265195	3.430505	3.339147	35.12708
3.282641	3.436031	3.414395	35.34187
3.210671	3.389732	3.483284	35.23388
3.222632	3.38461	3.388937	35.04252
3.278311	3.142464	3.324346	34.49349
3.328215	3.19766	3.402715	34.89471
3.241975	3.3034	3.412008	34.95768
3.283136	3.431718	3.433629	35.37558
3.273361	3.378207	3.415828	35.19826
3.351641	3.359809	3.352021	35.18967

3.177883	3.270243	3.386891	34.69008
3.285817	3.358865	3.424149	35.20139
3.230097	3.384408	3.455251	35.20342
3.224942	3.385553	3.379474	35.02893
3.303923	3.362437	3.494606	35.40288
3.421178	3.346061	3.341824	35.28937
3.345661	3.135725	3.405409	34.80331
3.352755	3.234322	3.292494	34.78751
3.28293	3.241061	3.438728	34.96934
3.324668	3.444725	3.304447	35.21235
3.270144	3.447825	3.344893	35.18834
3.199371	3.418307	3.416561	35.12575
3.298932	3.369783	3.375501	35.14757
3.258184	3.480578	3.400839	35.35616
3.268165	3.443849	3.381383	35.25512
3.243212	3.385486	3.469898	35.26649
3.208032	3.297133	3.473973	35.00525
3.143362	3.147923	3.343051	34.25123
3.401752	3.223606	3.391665	35.0881
3.259421	3.384475	3.369498	35.08016
3.297695	3.450116	3.427286	35.43378
3.263752	3.334401	3.393575	35.03278
3.303057	3.340534	3.389295	35.12279
3.27332	3.34478	3.357579	34.99769
3.288126	3.341747	3.446026	35.21685
3.136309	3.364729	3.466283	34.97941
3.264412	3.367896	3.367094	35.04956
3.274929	3.365335	3.515596	35.39171
3.39936	3.324292	3.356113	35.22531
3.359106	3.14826	3.452659	34.96345
3.313862	3.23722	3.390796	34.92377
3.222714	3.281632	3.44103	34.93142
3.361828	3.383734	3.355584	35.27206
3.302727	3.440681	3.358057	35.27276
3.307882	3.4053	3.335805	35.158
3.118245	3.327932	3.394462	34.70237
3.256946	3.450925	3.364417	35.20896
3.30747	3.492305	3.456342	35.61096
3.326524	3.377938	3.367299	35.2078
3.240697	3.217339	3.41078	34.76399
3.222261	3.142599	3.369311	34.46955
3.354941	3.315868	3.402664	35.21155
3.376387	3.423159	3.323596	35.32016
3.238964	3.404019	3.390608	35.12433
3.192565	3.352463	3.4353	35.00785
3.306439	3.319373	3.333417	34.96171
3.238346	3.277791	3.389841	34.84526
3.299262	3.391349	3.414634	35.28103
3.151033	3.433133	3.527737	35.29559
3.169263	3.376118	3.415214	34.9647

3.257029	3.323416	3.429861	35.07341
3.456358	3.22738	3.361808	35.15048
3.25241	3.202714	3.378689	34.68745
3.258472	3.312566	3.343904	34.86486
3.26833	3.378949	3.43409	35.22881
3.43318	3.491024	3.322845	35.59113
3.339846	3.388384	3.379746	35.287
3.323802	3.330088	3.34271	35.04344
3.199453	3.399571	3.405375	35.06049
3.230179	3.498572	3.385067	35.29978
3.252575	3.412713	3.397804	35.18884
3.282682	3.245779	3.369413	34.82754
3.214713	3.114361	3.375978	34.40587
3.233149	3.248946	3.430645	34.86005
3.300376	3.424507	3.393183	35.30906
3.360302	3.42545	3.413082	35.48569
3.296334	3.390877	3.336828	35.10344
3.215744	3.263234	3.370743	34.72224
3.154374	3.265727	3.408325	34.67567
3.271794	3.366548	3.410235	35.1571
3.239459	3.398223	3.445156	35.23203
3.206794	3.404423	3.442002	35.16726
3.209805	3.370794	3.469489	35.16041
3.286642	3.251035	3.403687	34.92264
3.448275	3.242746	3.308761	35.05039
3.334897	3.238231	3.37047	34.92753
3.270474	3.365874	3.389756	35.10796
3.307882	3.447084	3.38464	35.35617
3.39804	3.398156	3.358551	35.38928
3.363808	3.414802	3.311233	35.24735
3.323018	3.36857	3.37354	35.1933
3.102119	3.443781	3.351373	34.82622
3.281239	3.442636	3.458985	35.45076
3.234593	3.337502	3.356829	34.89544
3.233149	3.182361	3.460042	34.77873
3.198422	3.182092	3.397139	34.56463
3.333824	3.329886	3.417055	35.22749
3.313615	3.445264	3.44623	35.49941
3.351765	3.410422	3.357972	35.31364
3.258349	3.321125	3.456001	35.12845
3.29951	3.344982	3.309665	34.95062
3.212486	3.377601	3.504155	35.25696
3.219167	3.396875	3.36203	35.00292
3.234139	3.398021	3.445838	35.22144
3.288374	3.384475	3.403414	35.21765
3.210053	3.259729	3.421676	34.81351
3.340836	3.252788	3.422239	35.08556
3.275836	3.193077	3.44855	34.87038
3.388307	3.301918	3.355346	35.15053
3.301489	3.3652	3.404778	35.20716

3.285899	3.386092	3.331047	35.05752
3.354735	3.408669	3.392961	35.39282
3.276001	3.364661	3.280285	34.878
3.355106	3.404693	3.397616	35.39511
3.25608	3.460832	3.362627	35.22481
3.249151	3.454362	3.468892	35.4279
3.174913	3.239107	3.342779	34.51903
3.254926	3.204062	3.365901	34.66793
3.154374	3.195975	3.471876	34.66211
3.370531	3.417633	3.386635	35.43313
3.267092	3.420733	3.524889	35.51604
3.365912	3.405637	3.359216	35.33683
3.331102	3.355226	3.41992	35.28322
3.355188	3.350104	3.33369	35.13612
3.203248	3.327595	3.459463	35.02967
3.206135	3.399975	3.39615	35.05581
3.141754	3.424439	3.419852	35.02035
3.338155	3.372411	3.346018	35.17461
3.222632	3.245307	3.430287	34.82831
3.294684	3.229672	3.426502	34.94341
3.26602	3.23513	3.437381	34.91645
3.260328	3.373962	3.374478	35.07005
3.342609	3.424237	3.361859	35.33233
3.344382	3.413185	3.420824	35.44098
3.333247	3.420463	3.383566	35.35107
3.267958	3.379555	3.357153	35.06108
3.283383	3.443107	3.411786	35.35326
3.275011	3.463326	3.412417	35.38055
3.233314	3.384677	3.427474	35.1503
3.140434	3.178385	3.456922	34.56045
3.320379	3.219765	3.368629	34.85137
3.225684	3.327595	3.439563	35.03522
3.331226	3.43684	3.377138	35.36841
3.284745	3.399841	3.479805	35.41037
3.315388	3.336288	3.428241	35.22564
3.332298	3.328875	3.346257	35.06712
3.431612	3.382925	3.381042	35.47857
3.096963	3.327595	3.39864	34.66423
3.229148	3.326516	3.388818	34.92946
3.102036	3.363313	3.470665	34.91095
3.343393	3.34296	3.355175	35.14169
3.266432	3.194762	3.420602	34.79238
3.259091	3.224078	3.439478	34.88171
3.21063	3.238433	3.429793	34.78595
3.362488	3.406378	3.373727	35.3627
3.363066	3.426124	3.392024	35.44716
3.419198	3.420598	3.313024	35.38506
3.279135	3.382992	3.381537	35.14636
3.2103	3.389866	3.398776	35.04856
3.297777	3.472019	3.320612	35.24858

3.323843	3.400515	3.400208	35.32328
3.246924	3.318092	3.420926	35.02013
3.16452	3.131681	3.415333	34.42004
3.254389	3.236748	3.406262	34.8265
3.320173	3.398425	3.378024	35.26217
3.337619	3.397145	3.437432	35.42744
3.259792	3.372883	3.436579	35.20232
3.302026	3.335075	3.424353	35.18526
3.278805	3.320316	3.382117	35.00984
3.401505	3.360146	3.383856	35.36907
3.183286	3.35125	3.419494	34.95034
3.10381	3.385081	3.416407	34.84378
3.209022	3.364526	3.426672	35.05135
3.338155	3.303535	3.325199	34.97846
3.196772	3.182563	3.396781	34.56128
3.285074	3.241061	3.393507	34.87514
3.160272	3.315868	3.475559	34.94525
3.354776	3.440479	3.37134	35.41519
3.406866	3.429966	3.325795	35.40651
3.45974	3.371198	3.385408	35.52399
3.292498	3.348082	3.390728	35.11934
3.225478	3.419722	3.36573	35.07477
3.199866	3.443647	3.336964	35.00818
3.366077	3.397549	3.273055	35.13109
3.180275	3.241129	3.461372	34.79452
3.133299	3.166389	3.412144	34.4207
3.27596	3.314857	3.447066	35.13372
3.379357	3.46656	3.366651	35.51573
3.327143	3.393236	3.405221	35.32554
3.274434	3.352328	3.448055	35.21448
3.271093	3.331706	3.436886	35.13766
3.281074	3.378746	3.38904	35.15772
3.433468	3.363852	3.328609	35.32626
3.224158	3.293561	3.456342	34.99415
3.098159	3.316407	3.367384	34.57403
3.115069	3.320586	3.436818	34.77199
3.360344	3.245172	3.37961	35.01835
3.251956	3.261144	3.384794	34.82758
3.274516	3.244566	3.432333	34.94463
3.257029	3.329347	3.427406	35.08101
3.39903	3.440951	3.425632	35.63173
3.427447	3.451599	3.322317	35.49122
3.471	3.399571	3.423722	35.69444
3.276908	3.389193	3.412127	35.22194
3.245275	3.4053	3.354902	35.06285
3.213393	3.378814	3.41963	35.07676
3.309367	3.33858	3.331917	35.00684
3.253152	3.183237	3.413628	34.72288
3.161674	3.224752	3.475031	34.7479
3.207867	3.406984	3.464987	35.22547

3.428437	3.433807	3.417993	35.66371
3.35358	3.334806	3.38834	35.21866
3.398246	3.348689	3.439291	35.45812
3.236655	3.336625	3.434942	35.06885
3.296994	3.419722	3.385885	35.27524
3.346115	3.371198	3.360649	35.22136
3.232159	3.30832	3.392842	34.90506
3.180852	3.3034	3.445753	34.89781
3.186255	3.321799	3.326341	34.68872
3.361746	3.207432	3.392348	34.96673
3.257648	3.272534	3.312683	34.70724
3.250141	3.323618	3.506542	35.22648
3.13866	3.392967	3.401283	34.90416
3.359766	3.415341	3.392484	35.41737
3.375934	3.419857	3.353743	35.37788
3.416806	3.392427	3.373045	35.44949
3.307263	3.409411	3.434925	35.3824
3.196154	3.372277	3.3622	34.89917
3.249316	3.372816	3.422103	35.14761
3.314481	3.296526	3.291283	34.8372
3.179698	3.200827	3.433902	34.64505
3.267175	3.340871	3.4013	35.07131
3.169304	3.444792	3.484102	35.26562
3.381254	3.447825	3.357989	35.45996
3.442294	3.362572	3.426468	35.55676
3.383193	3.325977	3.406347	35.30349
3.256699	3.311285	3.474758	35.14434
3.331803	3.453419	3.303458	35.2448
3.271547	3.296324	3.420193	35.02477
3.330112	3.316003	3.337476	35.01499
3.173552	3.258449	3.423825	34.73559
3.163984	3.280419	3.396457	34.70286
3.200567	3.279947	3.445071	34.88814
3.260493	3.317486	3.411735	35.02838
3.305449	3.376118	3.409536	35.2501
3.300417	3.422755	3.459872	35.45116
3.291426	3.40685	3.402442	35.27113
3.376759	3.419991	3.335515	35.34011
3.417012	3.42727	3.302929	35.3728
3.263339	3.36055	3.381656	35.063
3.294313	3.282508	3.307141	34.79712
3.248327	3.225628	3.349855	34.66557
3.318275	3.236815	3.348423	34.83987
3.216734	3.320518	3.38846	34.88842
3.308459	3.440007	3.368765	35.30724
3.17347	3.414128	3.493446	35.2281
3.331432	3.382723	3.404846	35.31111
3.27497	3.359607	3.427491	35.1866
3.413012	3.398291	3.397122	35.50666
3.283342	3.380566	3.48429	35.37496

3.307676	3.389597	3.434567	35.33919
3.191617	3.21963	3.393575	34.62405
3.289034	3.207297	3.337049	34.6865
3.136722	3.247194	3.387386	34.55075
3.229602	3.285069	3.41506	34.89721
3.173511	3.386295	3.427406	35.02291
3.25575	3.398291	3.425223	35.22421
3.374037	3.361628	3.396849	35.34066
3.438459	3.385351	3.412946	35.56862
3.394246	3.42235	3.373028	35.46555
3.35259	3.407861	3.415333	35.43528
3.336051	3.40011	3.365611	35.27343
3.331927	3.278532	3.320134	34.89909
3.173511	3.27058	3.300542	34.49242
3.105748	3.185259	3.406841	34.39012
3.261648	3.267614	3.402442	34.90152
3.211166	3.379218	3.377496	34.98063
3.315306	3.445871	3.423398	35.45451
3.303881	3.372748	3.413031	35.24695
3.270227	3.361831	3.477537	35.29054
3.203289	3.407254	3.364042	34.99529
3.458091	3.435627	3.411377	35.71807
3.296788	3.418441	3.325915	35.14085
3.320544	3.308724	3.391069	35.09535
3.22457	3.17825	3.36174	34.536
3.303799	3.228526	3.399714	34.90225
3.258514	3.275567	3.361109	34.82167
3.31048	3.338243	3.437364	35.23913
3.089416	3.339456	3.438762	34.76141
3.309944	3.40476	3.390983	35.282
3.288044	3.403615	3.407285	35.26725
3.428725	3.467706	3.37313	35.64036
3.262886	3.400515	3.429725	35.25452
3.374037	3.374298	3.350333	35.26664
3.273691	3.287158	3.431242	35.03358
3.364963	3.305422	3.298598	34.98304
3.177883	3.25117	3.393319	34.66243
3.265566	3.310948	3.368117	34.92979
3.107357	3.274151	3.450391	34.68326
3.194298	3.473637	3.41385	35.22972
3.300087	3.445938	3.496771	35.58183
3.331473	3.37659	3.410644	35.31047
3.309119	3.380634	3.376507	35.19577
3.317244	3.388856	3.396679	35.27563
3.367355	3.420126	3.410081	35.48291
3.354982	3.34815	3.382065	35.23719
3.247131	3.209251	3.383089	34.69982
3.136062	3.182226	3.429469	34.49926
3.282889	3.254742	3.36527	34.83853
3.229231	3.306163	3.374307	34.8534

3.263917	3.396404	3.428292	35.24465
3.203	3.345589	3.449044	35.0457
3.202959	3.4115	3.446708	35.18463
3.26734	3.404154	3.412963	35.23557
3.513934	3.469324	3.33398	35.74462
3.240532	3.375175	3.37163	35.02318
3.339763	3.353609	3.302349	35.04152
3.286147	3.275836	3.450272	35.07767
3.364839	3.292617	3.361211	35.0917
3.249358	3.277521	3.342114	34.76438
3.276785	3.310679	3.360973	34.93811
3.178831	3.360213	3.416373	34.95338
3.253441	3.38461	3.410013	35.15598
3.238593	3.34505	3.431907	35.08488
3.330978	3.356372	3.392228	35.2249
3.301242	3.420194	3.414839	35.34888
3.268618	3.430572	3.449931	35.37698
3.310975	3.381307	3.392296	35.23583
3.329535	3.235804	3.390165	34.95357
3.28792	3.191662	3.350401	34.67907
3.261937	3.255753	3.339999	34.73966
3.134742	3.271456	3.381793	34.58724
3.215662	3.409478	3.342847	34.98086
3.292086	3.440007	3.417209	35.37737
3.289776	3.357248	3.379934	35.10983
3.25674	3.389529	3.424746	35.20617
3.330442	3.433807	3.397872	35.40541
3.32141	3.432122	3.419903	35.43015
3.413341	3.368772	3.301088	35.23282
3.323348	3.243757	3.31391	34.79067
3.233561	3.21161	3.383924	34.67713
3.317904	3.326449	3.399646	35.14709
3.191947	3.359404	3.373523	34.88658
3.326813	3.341478	3.340647	35.07042
3.236366	3.382655	3.396576	35.08498
3.192318	3.362572	3.449521	35.06052
3.241769	3.372075	3.384572	35.04741
3.33832	3.412039	3.348764	35.26764
3.250719	3.399504	3.4508	35.27179
3.384265	3.392158	3.402783	35.44277
3.266515	3.228526	3.465192	34.96391
3.325369	3.189909	3.358193	34.77418
3.358818	3.174476	3.405921	34.91794
3.321286	3.300098	3.381366	35.05689
3.170088	3.347543	3.449232	34.97841
3.247502	3.446949	3.363888	35.17845
3.203536	3.389934	3.44207	35.12859
3.361787	3.431785	3.332565	35.32671
3.245398	3.446747	3.456939	35.37689
3.302108	3.437109	3.393592	35.34131

3.267051	3.338041	3.440279	35.15009
3.357333	3.275702	3.305044	34.91546
3.342485	3.247935	3.381179	34.98876
3.259669	3.33029	3.346257	34.91139
3.25575	3.369446	3.415742	35.1404
3.252698	3.391147	3.384998	35.11395
3.327638	3.386497	3.366924	35.22813
3.269113	3.391012	3.3489	35.07061
3.266762	3.382318	3.439683	35.24498
3.149219	3.472963	3.464731	35.24094
3.277774	3.413387	3.379252	35.20485
3.355106	3.343365	3.440228	35.35418
3.33601	3.261886	3.387062	35.01798
3.219291	3.20049	3.479328	34.83024
3.261896	3.243083	3.340647	34.71328
3.236077	3.270175	3.398895	34.84345
3.321864	3.376186	3.355721	35.16846
3.267422	3.37167	3.449078	35.24369
3.219497	3.386632	3.395639	35.05474
3.195906	3.38771	3.38418	34.98045
3.298932	3.454767	3.377138	35.33699
3.228777	3.422013	3.420926	35.2077
3.362117	3.384745	3.368748	35.30369
3.291756	3.303063	3.363326	34.95934
3.273898	3.239309	3.403005	34.86764
3.353332	3.327662	3.335549	35.08705
3.422992	3.389058	3.377427	35.46523
3.234922	3.345521	3.38904	34.98414
3.265855	3.319036	3.37591	34.96515
3.169799	3.321597	3.368902	34.74537
3.338443	3.377736	3.387914	35.27851
3.243501	3.473839	3.419971	35.35115
3.206671	3.424979	3.464186	35.26045
3.158911	3.340602	3.477333	35.00024
3.380677	3.28325	3.33664	35.05211
3.331267	3.191325	3.428616	34.94417
3.330401	3.245913	3.359472	34.91045
3.247296	3.218686	3.462225	34.89387
3.171696	3.33211	3.33352	34.69513
3.35424	3.411163	3.35729	35.31918
3.334484	3.42235	3.341397	35.26569
3.259421	3.38434	3.378365	35.09926
3.128514	3.444051	3.410235	35.01326
3.316296	3.475591	3.354357	35.37068
3.289199	3.413589	3.348235	35.16245
3.300293	3.311892	3.419425	35.12
3.164025	3.171578	3.437295	34.55424
3.280331	3.280284	3.366156	34.89073
3.331721	3.34168	3.396917	35.20465
3.41594	3.429696	3.357801	35.49576

3.317121	3.384879	3.463555	35.41291
3.153384	3.353137	3.358585	34.75588
3.213063	3.380027	3.426894	35.09458
3.360344	3.510164	3.315002	35.45655
3.191328	3.442231	3.490854	35.32295
3.244037	3.391551	3.403465	35.13628
3.152972	3.25636	3.450664	34.7447
3.302809	3.266671	3.38343	34.94789
3.379274	3.246385	3.371135	35.04386
3.395483	3.245846	3.383958	35.10617
3.275135	3.295178	3.420397	35.03056
3.269649	3.376388	3.434908	35.22789
3.306026	3.44176	3.347314	35.25884
3.412434	3.402402	3.374614	35.46517
3.198793	3.392697	3.376251	34.98033
3.113213	3.453823	3.442957	35.07273
3.308542	3.463123	3.404215	35.4355
3.351518	3.322136	3.416083	35.24711
3.292251	3.234322	3.370863	34.82658
3.247337	3.238905	3.410286	34.82459
3.237768	3.338445	3.412741	35.02672
3.283961	3.401256	3.314268	35.04975
3.397669	3.362639	3.342369	35.27541
3.264741	3.345791	3.457212	35.19902
3.160973	3.354417	3.392365	34.84915
3.291591	3.421272	3.394274	35.28516
3.320461	3.512793	3.446657	35.66299
3.198463	3.414802	3.329939	34.92667
3.26404	3.267749	3.449232	35.00937
3.13965	3.211273	3.455677	34.62794
3.365293	3.273478	3.38875	35.11105
3.2428	3.244363	3.370095	34.73872
3.342856	3.294504	3.431634	35.20175
3.277156	3.315868	3.403857	35.04405
3.352631	3.351182	3.383157	35.24106
3.298396	3.353474	3.408461	35.18281
3.303263	3.37349	3.355687	35.12181
3.151446	3.470739	3.424626	35.15324
3.24247	3.508142	3.374154	35.32372
3.265484	3.356372	3.415128	35.13175
3.267464	3.271051	3.363241	34.83603
3.364097	3.221247	3.44502	35.11727
3.305655	3.411769	3.414719	35.33985
3.312501	3.416891	3.444184	35.43046
3.35824	3.405367	3.355584	35.31153
3.259545	3.279813	3.394667	34.90659
3.296086	3.345858	3.333008	34.9961
3.161798	3.496955	3.379798	35.13518
3.186173	3.478152	3.395775	35.1823
3.246966	3.404154	3.379235	35.11725

3.189637	3.275836	3.429043	34.8202
3.298891	3.282239	3.382611	34.97158
3.261978	3.298615	3.421403	35.0115
3.299551	3.274421	3.45728	35.11922
3.164891	3.266064	3.38875	34.6566
3.434582	3.373894	3.378979	35.46081
3.306975	3.358933	3.358722	35.10473
3.389791	3.355293	3.370487	35.30361
3.258885	3.338849	3.428719	35.10872
3.288374	3.394314	3.404829	35.24226
3.241851	3.447016	3.458235	35.37256
3.348631	3.425113	3.305657	35.22451
3.224405	3.192403	3.415964	34.68517
3.245068	3.223539	3.362217	34.68091
3.209104	3.296054	3.441456	34.93413
3.404062	3.479163	3.430355	35.73662
3.298561	3.389799	3.406074	35.25738
3.406949	3.304883	3.435471	35.373
3.2249	3.273343	3.358108	34.73674
3.336505	3.484555	3.384657	35.50074
3.207949	3.501673	3.369771	35.22449
3.228612	3.376725	3.413901	35.09294
3.086116	3.240792	3.391205	34.43443
3.173635	3.235804	3.423194	34.68487
3.334938	3.323888	3.375449	35.12583
3.287384	3.258179	3.439069	35.01727
3.315595	3.305355	3.407285	35.11262
3.24445	3.34323	3.47012	35.17727
3.434623	3.408871	3.351356	35.47698
3.480032	3.483207	3.322368	35.67545
3.367685	3.333727	3.363786	35.19345
3.206423	3.250361	3.437568	34.81984
3.243625	3.418913	3.370811	35.1238
3.28693	3.480781	3.253548	35.09736
3.332009	3.322742	3.39985	35.17028
3.126659	3.201366	3.373113	34.39731
3.176604	3.168546	3.461372	34.62777
3.218301	3.362168	3.372755	34.94858
3.379852	3.410758	3.440501	35.55628
3.266432	3.39391	3.431157	35.25097
3.408557	3.290393	3.440467	35.35576
3.180976	3.337165	3.461458	35.00626
3.376594	3.547635	3.331064	35.60916
3.198257	3.406647	3.445361	35.16079
3.245811	3.367222	3.342574	34.95379
3.132639	3.224213	3.44664	34.62114
3.187245	3.357315	3.417311	34.96749
3.295592	3.312296	3.473939	35.22982
3.311347	3.281026	3.385476	35.00243
3.287095	3.224145	3.403346	34.86409

3.322853	3.327527	3.435676	35.23906
3.334278	3.422418	3.405801	35.40623
3.447244	3.395123	3.390642	35.56043
3.281115	3.373962	3.381298	35.13042
3.229066	3.398156	3.400754	35.11205
3.312625	3.449442	3.441064	35.49509
3.296086	3.399234	3.337169	35.12192
3.316048	3.249081	3.364059	34.89602
3.208609	3.22246	3.402374	34.68664
3.160932	3.367761	3.480504	35.07099
3.187369	3.427405	3.393507	35.06898
3.475743	3.38899	3.355942	35.53345
3.276867	3.282373	3.403602	34.96961
3.303923	3.289517	3.409484	35.05727
3.299427	3.45443	3.401436	35.39047
3.36554	3.508614	3.425087	35.70526
3.210259	3.351452	3.353214	34.86483
3.288209	3.221921	3.411326	34.87911
3.123277	3.242881	3.421148	34.58574
3.288333	3.397684	3.390011	35.21713
3.214218	3.378679	3.423995	35.08781
3.287343	3.310679	3.354834	34.94778
3.292333	3.278532	3.471535	35.1436
3.353662	3.320451	3.413304	35.24204
3.316584	3.342556	3.412468	35.20747
3.382409	3.390136	3.331064	35.27745
3.254266	3.345993	3.427559	35.11171
3.384636	3.399706	3.333571	35.30873
3.169675	3.33676	3.439273	34.93215
3.251791	3.281565	3.299996	34.68644
3.251172	3.275365	3.377905	34.8419
3.235954	3.352193	3.391512	35.00639
3.295179	3.450453	3.41963	35.41228
3.335804	3.409276	3.35908	35.27865
3.324503	3.334604	3.392075	35.1628
3.353291	3.355496	3.355107	35.1906
3.301819	3.380566	3.394104	35.21814
3.24412	3.425113	3.471961	35.35964
3.331391	3.373085	3.445139	35.37806
3.145795	3.184653	3.423177	34.51209
3.281033	3.179194	3.347604	34.63063
3.211537	3.355833	3.435386	35.0569
3.198587	3.402739	3.379883	35.00978
3.158292	3.303737	3.459053	34.87829
3.31576	3.306703	3.375637	35.04672
3.265319	3.329953	3.386414	35.01082
3.388884	3.398088	3.355925	35.36337
3.308501	3.39108	3.457433	35.39424
3.303222	3.413589	3.367793	35.23589
3.333659	3.322068	3.419766	35.21597

3.410001	3.343163	3.317645	35.20572
3.215868	3.253192	3.408171	34.7824
3.248945	3.240926	3.331593	34.66044
3.150044	3.306837	3.408785	34.75711
3.298396	3.407591	3.383549	35.24667
3.334814	3.445399	3.391512	35.42641
3.328834	3.364863	3.464646	35.39714
3.260163	3.386564	3.412622	35.18066
3.363272	3.401458	3.46601	35.55546
3.330318	3.541435	3.401641	35.64874
3.313986	3.370524	3.387863	35.20914
3.174006	3.203321	3.4124	34.59104
3.105377	3.204871	3.413082	34.44584
3.289281	3.25737	3.307789	34.73256
3.258926	3.407052	3.424694	35.24916
3.195453	3.335143	3.458644	35.02734
3.206217	3.27988	3.430816	34.86917
3.310109	3.318766	3.420875	35.15967
3.420848	3.446005	3.351765	35.52895
3.347971	3.400582	3.396048	35.36709
3.211496	3.41305	3.378757	35.0581
3.292787	3.372816	3.445361	35.29353
3.247131	3.33083	3.382952	34.96539
3.427034	3.351317	3.335123	35.29902
3.204526	3.279678	3.329478	34.64343
3.144146	3.228863	3.424609	34.60829
3.160643	3.334267	3.348491	34.70841
3.326359	3.407187	3.401095	35.34531
3.258266	3.42545	3.386959	35.20543
3.362571	3.367627	3.403209	35.34261
3.220405	3.310342	3.4685	35.04922
3.409258	3.454901	3.354425	35.52888
3.444233	3.489407	3.418266	35.82043
3.363684	3.346061	3.364707	35.21369
3.170212	3.178857	3.45225	34.61639
3.194751	3.254944	3.335685	34.58153
3.237108	3.328066	3.416561	35.01093
3.320544	3.454227	3.320867	35.26002
3.165634	3.294437	3.493634	34.94963
3.166582	3.229537	3.429469	34.66946
3.228901	3.310881	3.449948	35.02841
3.442294	3.344713	3.343597	35.33648
3.335515	3.428213	3.466556	35.55447
3.246388	3.415341	3.416765	35.22253
3.258307	3.34505	3.433271	35.13097
3.305614	3.322742	3.322914	34.94431
3.411403	3.294639	3.301173	35.06665
3.212115	3.304883	3.376848	34.81873
3.139898	3.36547	3.411752	34.86963
3.213682	3.385216	3.470989	35.20371

3.393833	3.455171	3.311762	35.40244
3.327679	3.369311	3.38846	35.23774
3.296953	3.335615	3.412127	35.14861
3.191823	3.385216	3.463572	35.13968
3.341784	3.522699	3.438949	35.71443
3.361622	3.35772	3.34624	35.19429
3.399855	3.193616	3.374955	34.98182
3.144434	3.183642	3.41772	34.49497
3.217848	3.292685	3.358705	34.76492
3.188606	3.433201	3.386857	35.06982
3.220034	3.354754	3.346837	34.87948
3.166747	3.280756	3.397599	34.71214
3.221931	3.302996	3.428036	34.94801
3.205475	3.344915	3.464578	35.0836
3.398989	3.418307	3.398827	35.5235
3.3549	3.419183	3.430628	35.49854
3.296705	3.295246	3.458661	35.16155
3.248904	3.204332	3.337271	34.59274
3.29056	3.171848	3.378468	34.70289
3.320544	3.27341	3.342745	34.91244
3.295262	3.40375	3.354152	35.16713
3.180687	3.428483	3.434158	35.14562
3.207454	3.416285	3.305964	34.89715
3.34133	3.35253	3.440365	35.3444
3.332752	3.356237	3.315223	35.06008
3.274145	3.353137	3.450459	35.22088
3.218343	3.470335	3.488723	35.43882
3.239047	3.440412	3.417123	35.26208
3.292168	3.173263	3.299519	34.53686
3.314605	3.164906	3.345507	34.66821
3.218796	3.268221	3.395229	34.79336
3.235129	3.469458	3.426058	35.33657
3.233685	3.43502	3.393865	35.1877
3.348424	3.265323	3.349173	34.96978
3.25839	3.266064	3.393473	34.87139
3.291014	3.326988	3.430901	35.15781
3.175697	3.37504	3.414515	34.97488
3.277857	3.438053	3.402374	35.30954
3.289446	3.43502	3.455234	35.44385
3.505603	3.253394	3.359557	35.31013
3.255462	3.195099	3.42009	34.76801
3.274475	3.244229	3.393643	34.85919
3.262556	3.37787	3.400123	35.13955
3.303799	3.413185	3.342114	35.18011
3.235087	3.374096	3.353197	34.96861
3.183574	3.406647	3.418743	35.07048
3.189266	3.380768	3.395894	34.97636
3.338402	3.40995	3.393183	35.36039
3.293818	3.44769	3.453409	35.47713
3.334154	3.424507	3.419511	35.44051

3.271423	3.223471	3.472831	34.98029
3.196937	3.171241	3.345643	34.42505
3.322359	3.280149	3.397275	35.0504
3.327596	3.412578	3.314183	35.16974
3.234675	3.369514	3.372431	34.99974
3.220364	3.257303	3.369635	34.71695
3.279548	3.27961	3.379388	34.91648
3.26635	3.370592	3.364877	35.05485
3.306274	3.448095	3.407063	35.4039
3.18708	3.409748	3.495339	35.25243
3.213847	3.367424	3.408137	35.02771
3.294354	3.312296	3.406893	35.08049
3.43784	3.274623	3.338533	35.16239
3.379811	3.25892	3.409553	35.15646
3.253482	3.258449	3.359898	34.77058
3.218178	3.338647	3.437705	35.03891
3.345744	3.433335	3.324823	35.27809
3.270887	3.356237	3.361126	35.02518
3.341702	3.392899	3.386567	35.31585
3.220446	3.359607	3.484562	35.19218
3.255585	3.411365	3.36527	35.12133
3.326813	3.427944	3.483949	35.57288
3.404474	3.409613	3.353555	35.41748
3.280744	3.171039	3.454159	34.84518
3.307676	3.21316	3.289629	34.63639
3.295468	3.325101	3.440314	35.18401
3.343764	3.444792	3.304754	35.25493
3.226756	3.395393	3.359353	35.01042
3.12307	3.251035	3.411923	34.58295
3.223539	3.240252	3.371016	34.68962
3.233396	3.313779	3.417055	34.97265
3.307511	3.416824	3.380224	35.27953
3.254224	3.420733	3.486472	35.40389
3.221395	3.354148	3.418215	35.03722
3.233768	3.192268	3.378178	34.62272
3.433716	3.280419	3.321225	35.1282
3.247997	3.358259	3.445429	35.1639
3.21991	3.410489	3.384998	35.08454
3.291673	3.3652	3.376541	35.12394
3.380264	3.385418	3.359233	35.32404
3.306397	3.367963	3.356232	35.11777
3.281569	3.373625	3.359216	35.08238
3.113089	3.392293	3.437057	34.925
3.27963	3.443781	3.423109	35.37129
3.198958	3.373153	3.407097	35.0054
3.366118	3.24315	3.403261	35.07827
3.290065	3.167265	3.410934	34.76279
3.308872	3.35718	3.424967	35.24992
3.274104	3.401728	3.431498	35.28558
3.344506	3.391147	3.377138	35.29753

3.167613	3.346869	3.407353	34.87994
3.259009	3.250631	3.325028	34.68932
3.148559	3.323686	3.442547	34.86454
3.22057	3.443107	3.460895	35.32329
3.300211	3.432796	3.423126	35.39231
3.273361	3.373153	3.455796	35.27461
3.257648	3.217608	3.389568	34.75526
3.322565	3.188022	3.427968	34.9165
3.288003	3.31917	3.358176	34.9751
3.369087	3.407726	3.3622	35.35487
3.244656	3.405232	3.362354	35.07764
3.279053	3.400784	3.407762	35.24244
3.314151	3.396538	3.374017	35.23611
3.284085	3.430707	3.32675	35.14172
3.295262	3.437581	3.444747	35.43923
3.23352	3.455373	3.390779	35.2251
3.268206	3.391686	3.450613	35.29253
3.171861	3.248811	3.383702	34.62307
3.342155	3.199749	3.384981	34.89098
3.212898	3.228256	3.379729	34.65917
3.42955	3.402604	3.379968	35.51475
3.258637	3.374096	3.451499	35.23508
3.352219	3.377601	3.353743	35.23361
3.272289	3.277993	3.375995	34.88965
3.295427	3.307983	3.383344	35.02191
3.085085	3.381307	3.432947	34.83074
3.239171	3.475659	3.428224	35.36371
3.17912	3.410152	3.427201	35.0869
3.366159	3.31762	3.379661	35.18961
3.286724	3.194357	3.453222	34.9072
3.28726	3.236276	3.418402	34.9239
3.278764	3.317283	3.419101	35.084
3.331102	3.369109	3.327228	35.11088
3.302026	3.430235	3.406074	35.35339
3.323555	3.454901	3.381861	35.40146
3.240078	3.333323	3.415691	35.02702
3.286518	3.428618	3.341466	35.17465
3.225808	3.492642	3.395502	35.30007
3.271258	3.432527	3.437023	35.3588
3.284497	3.322473	3.447526	35.17005
3.14596	3.208443	3.412758	34.54169
3.361705	3.239039	3.400106	35.05273
3.377583	3.350037	3.376387	35.27832
3.370572	3.377938	3.389432	35.35253
3.276166	3.346128	3.423398	35.15079
3.286394	3.353204	3.41385	35.16775
3.253111	3.310274	3.34017	34.83996
3.327638	3.374231	3.385305	35.24151
3.152724	3.4208	3.46526	35.13569
3.193432	3.399571	3.379661	34.99109

3.219992	3.351856	3.397599	34.98406
3.330566	3.270917	3.37811	35.00624
3.228035	3.214643	3.385595	34.67533
3.377583	3.269703	3.393251	35.13952
3.181595	3.35772	3.468398	35.06774
3.368757	3.471885	3.337493	35.44042
3.374655	3.389529	3.385936	35.37916
3.424312	3.340265	3.307652	35.20883
3.201845	3.356844	3.427815	35.02136
3.242553	3.482668	3.378775	35.27829
3.199659	3.467369	3.405051	35.2085
3.322812	3.358798	3.306885	35.02571
3.253111	3.243689	3.446299	34.92644
3.128556	3.195166	3.451687	34.55973
3.289446	3.295717	3.397412	35.01277
3.34397	3.439468	3.397958	35.44756
3.355518	3.428146	3.38609	35.4221
3.182461	3.352867	3.510702	35.15153
3.228324	3.316812	3.358142	34.83936
3.230633	3.273949	3.397958	34.83774
3.354157	3.345858	3.3622	35.18693
3.199206	3.454093	3.473394	35.32793
3.221436	3.425518	3.434874	35.22982
3.171779	3.235467	3.387317	34.60162
3.39606	3.183979	3.355653	34.91024
3.233685	3.223067	3.397207	34.73151
3.273114	3.367087	3.381094	35.09744
3.32339	3.4115	3.415538	35.37983
3.395071	3.473502	3.389091	35.61434
3.363354	3.427405	3.334662	35.32515
3.401958	3.327056	3.310858	35.13807
3.177512	3.407119	3.39158	34.99885
3.229561	3.459012	3.375432	35.19084
3.185719	3.375512	3.352993	34.86329
3.297612	3.215991	3.387249	34.83405
3.264288	3.191055	3.420585	34.77955
3.133546	3.310409	3.47353	34.87043
3.260122	3.374029	3.408052	35.14316
3.395112	3.427742	3.40292	35.5446
3.359478	3.289989	3.4028	35.16517
3.364674	3.331638	3.366702	35.18868
3.229066	3.306298	3.425223	34.96468
3.22457	3.332245	3.360154	34.8693
3.290766	3.435964	3.41801	35.3674
3.267299	3.388384	3.403141	35.17951
3.222632	3.350643	3.428633	35.05505
3.255379	3.256292	3.374478	34.8019
3.31378	3.181485	3.392757	34.80599
3.299097	3.279476	3.369925	34.93824
3.262473	3.413993	3.400873	35.22

3.255503	3.410287	3.417123	35.23219
3.41495	3.39856	3.38094	35.4761
3.324256	3.358865	3.320867	35.05959
3.403154	3.419317	3.391069	35.51785
3.253853	3.461102	3.356352	35.20681
3.203825	3.423968	3.390864	35.09167
3.151033	3.275836	3.352038	34.56738
3.294973	3.187146	3.384691	34.75961
3.202381	3.238433	3.432197	34.77317
3.25076	3.374501	3.376336	35.05436
3.252946	3.478894	3.461662	35.47403
3.397504	3.371401	3.338089	35.28485
3.437386	3.327123	3.446503	35.51232
3.399442	3.274825	3.308317	35.01279
3.263092	3.296931	3.44664	35.06544
3.239006	3.420396	3.30136	34.96506
3.239995	3.371603	3.425905	35.13289
3.34694	3.406243	3.358944	35.29607
3.232984	3.282508	3.417345	34.904
3.204939	3.195301	3.392637	34.59793
3.241274	3.226235	3.482516	34.94158
3.312419	3.372142	3.422802	35.28566
3.352425	3.448566	3.4397	35.57723
3.337742	3.446949	3.351287	35.34824
3.357663	3.347745	3.362405	35.19917
3.281363	3.330695	3.321686	34.90598
3.392349	3.400178	3.313382	35.28248
3.265814	3.461102	3.42981	35.39361
3.275341	3.359876	3.357852	35.03572
3.144764	3.178655	3.40812	34.46379
3.286023	3.233985	3.319299	34.69946
3.228653	3.296324	3.41963	34.92974
3.330896	3.474446	3.373506	35.44198
3.207867	3.376994	3.529186	35.30028
3.354363	3.375175	3.394411	35.32193
3.291178	3.292685	3.401214	35.01824
3.388389	3.355496	3.358858	35.27556
3.229685	3.31324	3.395911	34.91712
3.193803	3.343702	3.367538	34.84321
3.123483	3.375309	3.373591	34.77179
3.273031	3.28608	3.339999	34.83024
3.214507	3.238972	3.389023	34.70645
3.228612	3.237759	3.425717	34.81489
3.149012	3.361089	3.452932	34.97003
3.333288	3.419924	3.463009	35.52371
3.492281	3.50794	3.405733	35.93863
3.410413	3.445601	3.336845	35.47262
3.355188	3.325573	3.378451	35.18036
3.252946	3.396673	3.409621	35.18042
3.292622	3.462449	3.337868	35.25412

3.374985	3.411432	3.301719	35.24361
3.325534	3.203118	3.432453	34.96582
3.057081	3.212958	3.458252	34.45669
3.25241	3.315868	3.470444	35.13555
3.301201	3.471076	3.391955	35.41002
3.302685	3.422957	3.445207	35.42449
3.275052	3.332784	3.416288	35.10363
3.264205	3.307242	3.452301	35.10281
3.292704	3.331436	3.32687	34.94374
3.40101	3.402536	3.335873	35.35576
3.215002	3.346263	3.434652	35.04194
3.208362	3.344174	3.401845	34.95111
3.197721	3.267614	3.341943	34.62942
3.366489	3.152304	3.35729	34.77988
3.252987	3.306837	3.362627	34.88129
3.238139	3.351452	3.441371	35.11858
3.172068	3.448769	3.471825	35.25351
3.366324	3.455575	3.36706	35.4641
3.351188	3.435762	3.383413	35.42343
3.432973	3.407524	3.373676	35.51923
3.327514	3.349498	3.438131	35.30267
3.351023	3.331908	3.323203	35.06429
3.31543	3.319642	3.40471	35.13787
3.290189	3.265188	3.323391	34.78576
3.232778	3.190651	3.366941	34.59244
3.203454	3.265795	3.348082	34.6514
3.175656	3.439131	3.480811	35.25993
3.332587	3.464943	3.441337	35.57324
3.339021	3.42107	3.418198	35.44076
3.363849	3.306837	3.383617	35.16963
3.308624	3.298952	3.465891	35.21153
3.329411	3.408265	3.369601	35.28547
3.295715	3.335682	3.37857	35.07267
3.279053	3.309533	3.325522	34.86304
3.24944	3.296594	3.337152	34.79542
3.143486	3.21161	3.43675	34.59567
3.327679	3.324697	3.348235	35.05221
3.194339	3.278397	3.464868	34.91442
3.22853	3.388586	3.364042	35.00967
3.208444	3.411635	3.454824	35.21467
3.294354	3.429427	3.345626	35.20265
3.356508	3.365605	3.351339	35.2115
3.486631	3.451666	3.367793	35.72024
3.272825	3.466493	3.3858	35.32449
3.335433	3.432527	3.366327	35.34453
3.253482	3.277184	3.376149	34.84709
3.327885	3.172589	3.344382	34.7116
3.2063	3.214643	3.367776	34.58883
3.238098	3.371064	3.423245	35.12174
3.254554	3.475861	3.443775	35.4318

3.293447	3.412106	3.373591	35.22395
3.297118	3.3652	3.476923	35.35537
3.368592	3.305422	3.341346	35.08446
3.347682	3.364257	3.442428	35.38845
3.29852	3.384205	3.409706	35.253
3.276413	3.318631	3.437381	35.12178
3.31246	3.271658	3.393763	35.0025
3.257854	3.255618	3.316366	34.67875
3.222096	3.252451	3.387079	34.74827
3.109213	3.280419	3.379252	34.54546
3.170212	3.358259	3.43143	34.96318
3.264412	3.435492	3.411803	35.29516
3.332257	3.449847	3.400532	35.45027
3.361622	3.426933	3.44016	35.55104
3.248038	3.411432	3.333639	35.0358
3.34397	3.404289	3.386209	35.34493
3.351023	3.411837	3.335941	35.26693
3.296086	3.30057	3.37516	34.98924
3.267175	3.199007	3.404301	34.76764
3.273568	3.191662	3.406534	34.77044
3.150745	3.360887	3.365474	34.78212
3.26734	3.483813	3.365474	35.30592
3.211455	3.416622	3.436835	35.19282
3.257235	3.344106	3.408103	35.07153
3.189596	3.307377	3.389756	34.80317
3.402948	3.414937	3.432657	35.59877
3.338485	3.483342	3.36116	35.45103
3.380264	3.281093	3.383617	35.14922
3.221436	3.216125	3.419033	34.73727
3.339268	3.272197	3.376694	35.02498
3.292251	3.25737	3.377206	34.89085
3.260947	3.301311	3.357494	34.87538
3.092674	3.293561	3.425087	34.63826
3.219704	3.382521	3.375228	35.00156
3.278352	3.438592	3.487581	35.49813
3.443697	3.428213	3.267053	35.35476
3.347723	3.351924	3.439273	35.35467
3.309944	3.425113	3.364588	35.26878
3.259751	3.359539	3.466079	35.23756
3.376387	3.384138	3.294455	35.1711
3.304418	3.256899	3.378041	34.91826
3.173222	3.184046	3.35006	34.41085
3.212486	3.283384	3.416902	34.86012
3.182048	3.464876	3.429537	35.21808
3.300871	3.457328	3.435181	35.47376
3.298314	3.359741	3.440655	35.26673
3.242553	3.312027	3.472916	35.111
3.237686	3.374972	3.444133	35.17507
3.448068	3.48907	3.371835	35.72655
3.28891	3.387373	3.473394	35.37819

3.347723	3.296931	3.336299	35.00922
3.260081	3.186203	3.463163	34.85285
3.349043	3.238972	3.317201	34.8436
3.296416	3.244903	3.358739	34.83232
3.346816	3.354283	3.358466	35.18113
3.096303	3.338647	3.499977	34.90857
3.245769	3.402604	3.379729	35.11233
3.234593	3.418037	3.427747	35.22664
3.364386	3.445129	3.3622	35.42639
3.316873	3.427203	3.390301	35.34473
3.337289	3.38461	3.428906	35.38066
3.228035	3.355159	3.435863	35.09255
3.38278	3.293696	3.352038	35.11323
3.295757	3.197727	3.388852	34.79356
3.224983	3.213767	3.380633	34.65589
3.187328	3.347274	3.405938	34.92084
3.214796	3.487789	3.423586	35.32679
3.365582	3.450656	3.336214	35.38426
3.290807	3.37039	3.433425	35.25779
3.281857	3.396606	3.440501	35.31103
3.289982	3.394247	3.455319	35.35604
3.304748	3.383464	3.363565	35.1641
3.388554	3.288708	3.41078	35.24341
3.402618	3.183372	3.349531	34.90987
3.252038	3.202849	3.432385	34.80435
3.304583	3.224684	3.359626	34.8079
3.233726	3.261347	3.385612	34.78995
3.251296	3.345589	3.388392	35.01867
3.272701	3.360011	3.394939	35.11134
3.254884	3.457934	3.441081	35.38743
3.225189	3.429898	3.397122	35.16505
3.37049	3.493585	3.412758	35.65626
3.222962	3.454767	3.445412	35.32016
3.345455	3.346195	3.415759	35.28576
3.266515	3.267884	3.384726	34.87401
3.31943	3.237894	3.390659	34.93712
3.300005	3.242679	3.330962	34.77455
3.354611	3.287293	3.378979	35.09654
3.132061	3.387036	3.401692	34.87765
3.301861	3.440277	3.346053	35.24373
3.16452	3.360415	3.403448	34.89426
3.347393	3.390743	3.362831	35.27167
3.260081	3.364661	3.463828	35.24456
3.192236	3.474985	3.48949	35.39357
3.231623	3.329616	3.453801	35.08376
3.34199	3.226504	3.375228	34.9278
3.29522	3.207634	3.409416	34.85902
3.297736	3.207769	3.349565	34.73393
3.250966	3.233985	3.412912	34.82751
3.184853	3.408737	3.378416	34.98965

3.325534	3.382049	3.35584	35.18957
3.315265	3.403952	3.340511	35.18149
3.291673	3.420868	3.455643	35.41866
3.221271	3.441692	3.407916	35.20587
3.318358	3.400919	3.380752	35.26962
3.371067	3.32591	3.377496	35.21373
3.346527	3.355226	3.372244	35.21269
3.194586	3.254405	3.445583	34.82032
3.345826	3.286215	3.352856	35.01784
3.234345	3.265458	3.405443	34.84366
3.334072	3.357248	3.347877	35.13659
3.247708	3.405906	3.402237	35.17301
3.178048	3.375646	3.400123	34.94988
3.190091	3.4115	3.386073	35.02389
3.328669	3.424574	3.343904	35.26332
3.265278	3.375444	3.463572	35.27895
3.314893	3.376657	3.369993	35.18546
3.189225	3.225156	3.46971	34.7974
3.275382	3.219158	3.36631	34.74657
3.395731	3.247598	3.367742	35.07508
3.372098	3.253529	3.350691	34.99908
3.244326	3.3528	3.424268	35.09766
3.235459	3.397886	3.353828	35.02282
3.298355	3.358731	3.366907	35.10334
3.315925	3.48907	3.346053	35.38119
3.262886	3.474648	3.421472	35.39859
3.177759	3.437581	3.499704	35.30246
3.273155	3.371198	3.404778	35.15832
3.415857	3.307983	3.406995	35.33699
3.330236	3.294235	3.31258	34.91321
3.235129	3.236141	3.421676	34.81676
3.291055	3.274286	3.397395	34.96938
3.171119	3.345319	3.414668	34.90021
3.4206	3.412106	3.323493	35.39246
3.26503	3.396471	3.401709	35.1891
3.274392	3.38589	3.381588	35.14244
3.14794	3.379555	3.402033	34.89676
3.302974	3.44459	3.388545	35.34852
3.22391	3.416352	3.377683	35.09012
3.265938	3.265727	3.423722	34.95331
3.156148	3.198738	3.438591	34.59924
3.361911	3.202849	3.387454	34.94637
3.295344	3.239915	3.385953	34.87858
3.372098	3.331032	3.380838	35.2345
3.275135	3.411769	3.392791	35.22515
3.229437	3.347206	3.372977	34.9407
3.174377	3.355226	3.349787	34.78712
3.280043	3.42727	3.368151	35.2159
3.246677	3.475793	3.418829	35.35987
3.235294	3.44331	3.429793	35.28792

3.20865	3.319036	3.414736	34.92496
3.302273	3.283317	3.436426	35.09902
3.431241	3.315599	3.382458	35.33362
3.2501	3.308724	3.384794	34.92757
3.331267	3.323281	3.419016	35.21175
3.282847	3.362505	3.394121	35.13719
3.295344	3.395325	3.363428	35.16917
3.353992	3.367694	3.353214	35.21467
3.308006	3.38124	3.46439	35.38685
3.043594	3.431381	3.400277	34.77807
3.283878	3.375646	3.442087	35.27308
3.237232	3.332043	3.39025	34.96236
3.229561	3.258988	3.422699	34.85679
3.19702	3.200962	3.369379	34.54213
3.278723	3.227043	3.452932	34.96055
3.292251	3.259797	3.362814	34.86469
3.390781	3.343297	3.402783	35.35017
3.279754	3.390136	3.420858	35.24932
3.312625	3.340669	3.345899	35.04911
3.186296	3.364729	3.399305	34.94225
3.243996	3.484622	3.35006	35.22293
3.218343	3.442231	3.395417	35.17332
3.294684	3.327392	3.354766	35.00023
3.189184	3.224684	3.468346	34.79329
3.278888	3.287226	3.371357	34.91413
3.259545	3.355428	3.412076	35.11002
3.447697	3.4053	3.372244	35.54344
3.351394	3.350374	3.440314	35.36158
3.279589	3.333256	3.376268	35.02706
3.224405	3.341141	3.421267	35.02203
3.270227	3.461708	3.349531	35.22903
3.235005	3.499988	3.415077	35.37905
3.256575	3.372546	3.365866	35.03991
3.173758	3.340602	3.483897	35.04706
3.267299	3.272938	3.403005	34.92675
3.327225	3.227043	3.394939	34.9398
3.219786	3.196918	3.3762	34.59799
3.349249	3.265188	3.360973	34.9971
3.263834	3.315936	3.460827	35.13965
3.391689	3.461236	3.36943	35.53713
3.414249	3.429629	3.398043	35.57991
3.326112	3.389799	3.318412	35.12593
3.128762	3.357113	3.449777	34.91015
3.258184	3.43684	3.350759	35.15099
3.2395	3.34788	3.43143	35.09201
3.270268	3.302996	3.316195	34.80914
3.176481	3.214441	3.418283	34.63363
3.259669	3.287832	3.425513	34.99186
3.280496	3.385081	3.406057	35.20753
3.369623	3.361965	3.418147	35.37832

3.320833	3.340467	3.407575	35.20149
3.338732	3.342421	3.432879	35.30024
3.254018	3.342084	3.3873	35.01458
3.288993	3.423765	3.400123	35.29773
3.306026	3.500662	3.367674	35.43217
3.244285	3.387305	3.368492	35.05105
3.198216	3.276375	3.39262	34.76049
3.230674	3.172791	3.412622	34.64869
3.298107	3.214778	3.371425	34.79788
3.26371	3.252114	3.393388	34.85233
3.278146	3.303063	3.409331	35.03018
3.288704	3.341208	3.388409	35.09094
3.329741	3.379151	3.392978	35.27365
3.290601	3.331032	3.370044	35.03267
3.257936	3.352261	3.360785	34.98742
3.162911	3.491901	3.406534	35.18503
3.182626	3.45106	3.446094	35.22534
3.289405	3.355698	3.400481	35.15056
3.2534	3.263031	3.358313	34.77696
3.220075	3.262223	3.34994	34.684
3.344218	3.324225	3.401487	35.2038
3.212527	3.361292	3.437449	35.07551
3.411403	3.332784	3.388664	35.3414
3.320956	3.288372	3.380991	35.0297
3.350322	3.426326	3.428156	35.49875
3.306604	3.461843	3.37238	35.35884
3.333329	3.408871	3.394871	35.35063
3.15025	3.417161	3.416902	35.01657
3.335721	3.341073	3.381707	35.17881
3.146909	3.237422	3.468329	34.72866
3.222178	3.226841	3.404284	34.73007
3.218714	3.223337	3.473394	34.86596
3.313656	3.321192	3.381673	35.087
3.34298	3.336086	3.422307	35.27256
3.322152	3.404491	3.36411	35.24934
3.280331	3.346263	3.430287	35.17526
3.356178	3.310477	3.36515	35.12042
3.239129	3.416689	3.384947	35.14002
3.28392	3.440277	3.321243	35.15024
3.233891	3.329886	3.426996	35.0307
3.233933	3.281632	3.384163	34.83159
3.244079	3.269771	3.385135	34.82997
3.226715	3.361224	3.405511	35.03655
3.329947	3.428416	3.460707	35.52994
3.272536	3.390136	3.412196	35.2146
3.266185	3.325303	3.423126	35.08283
3.344341	3.360415	3.348713	35.1678
3.415445	3.410017	3.412179	35.57055
3.191617	3.408871	3.450272	35.16188
3.306191	3.379623	3.353385	35.13659

3.140475	3.245644	3.461799	34.71829
3.25043	3.335682	3.311625	34.82724
3.181182	3.247868	3.463009	34.81482
3.212527	3.206353	3.371135	34.59167
3.146785	3.228054	3.477196	34.7273
3.354281	3.353946	3.379286	35.24225
3.355559	3.386429	3.423927	35.4137
3.484486	3.425652	3.406824	35.74402
3.256122	3.367357	3.418846	35.14343
3.326813	3.356574	3.350469	35.12491
3.25208	3.413185	3.333486	35.04814
3.302273	3.481994	3.273123	35.17637
3.229561	3.34943	3.409058	35.02474
3.19038	3.241735	3.356062	34.58765
3.197968	3.225426	3.394206	34.65199
3.263133	3.351856	3.400123	35.08392
3.393751	3.38616	3.412332	35.47128
3.253606	3.32591	3.406415	35.0201
3.27563	3.296796	3.387488	34.96321
3.292416	3.357652	3.39673	35.15321
3.440438	3.475389	3.344399	35.61994
3.271093	3.423024	3.474621	35.41988
3.145136	3.387777	3.38077	34.86211
3.174831	3.271456	3.421216	34.76112
3.303222	3.306163	3.329001	34.91613
3.235665	3.216732	3.397685	34.72302
3.268907	3.22893	3.410439	34.85029
3.123524	3.256764	3.418556	34.61098
3.359354	3.351789	3.391137	35.27454
3.386657	3.394112	3.391154	35.42684
3.3841	3.376051	3.416288	35.43672
3.240655	3.349363	3.428582	35.09155
3.254596	3.393977	3.381383	35.11638
3.295674	3.465078	3.400208	35.40287
3.418373	3.402671	3.27829	35.2681
3.198381	3.246453	3.418743	34.75254
3.107068	3.230548	3.377001	34.42678
3.167984	3.399234	3.433817	35.05314
3.319966	3.406311	3.388818	35.30257
3.369046	3.392023	3.427951	35.46423
3.287178	3.262492	3.446657	35.04284
3.195288	3.300435	3.458371	34.95048
3.37449	3.421541	3.387522	35.45227
3.440645	3.51778	3.432947	35.90674
3.230262	3.348487	3.390114	34.98278
3.189761	3.221315	3.396576	34.63024
3.218673	3.246048	3.459599	34.88537
3.391276	3.379825	3.371681	35.36311
3.282724	3.319777	3.377513	35.00716
3.249976	3.239511	3.37325	34.7507

3.120513	3.286215	3.470512	34.78242
3.328792	3.382049	3.358603	35.20273
3.434706	3.378949	3.387658	35.49111
3.397751	3.427203	3.38979	35.52048
3.191287	3.32719	3.455711	34.99443
3.334855	3.416217	3.433016	35.45344
3.251709	3.364055	3.341756	34.95797
3.3264	3.232974	3.337748	34.8259
3.10018	3.249351	3.388306	34.47756
3.150497	3.337299	3.378041	34.75748
3.173552	3.413993	3.42333	35.07466
3.367891	3.437581	3.357852	35.40804
3.359065	3.340534	3.440092	35.35635
3.280826	3.337299	3.357153	34.99681
3.208692	3.403884	3.452181	35.19249
3.364097	3.506188	3.408512	35.66056
3.394741	3.314925	3.365133	35.21444
3.322565	3.218349	3.359199	34.83244
3.08558	3.20878	3.407489	34.39886
3.213641	3.392158	3.41165	35.08903
3.185513	3.379353	3.394479	34.96197
3.238758	3.269299	3.420926	34.89557
3.257936	3.258584	3.480078	35.04343
3.18873	3.413859	3.480879	35.2334
3.281693	3.42909	3.452369	35.40766
3.423322	3.409411	3.336896	35.42182
3.33469	3.345589	3.410559	35.24952
3.241728	3.358798	3.394684	35.0404
3.290519	3.351385	3.381435	35.10191
3.294519	3.290865	3.355005	34.92051
3.356962	3.242476	3.327688	34.89151
3.195618	3.260403	3.340596	34.60611
3.250801	3.404087	3.427269	35.23054
3.212692	3.438727	3.416987	35.20047
3.320255	3.388451	3.373386	35.2304
3.313904	3.308859	3.393985	35.0875
3.296416	3.412982	3.475713	35.45568
3.231705	3.518588	3.401504	35.38283
3.405959	3.49992	3.419238	35.76185
3.268536	3.268693	3.402988	34.92013
3.293199	3.212014	3.366855	34.77111
3.150126	3.268693	3.467391	34.80203
3.209517	3.396067	3.390182	35.04161
3.119853	3.282913	3.395962	34.61072
3.272578	3.255146	3.320833	34.71969
3.24412	3.282778	3.515323	35.1432
3.343723	3.39108	3.342165	35.21919
3.234675	3.408737	3.455387	35.26693
3.297076	3.434818	3.375927	35.28666
3.365994	3.347678	3.418658	35.34026

3.388059	3.410961	3.374921	35.43125
3.292003	3.32719	3.402937	35.09927
3.269155	3.253327	3.308249	34.68071
3.239913	3.248542	3.396321	34.79889
3.128721	3.407187	3.379371	34.8656
3.314192	3.441355	3.393319	35.37642
3.200278	3.390136	3.473871	35.19145
3.238882	3.277589	3.365099	34.79188
3.371397	3.323821	3.446503	35.36079
3.317657	3.541907	3.389244	35.59497
3.291137	3.498303	3.463555	35.60413
3.330483	3.243016	3.344416	34.87136
3.185183	3.148934	3.467033	34.61602
3.338608	3.237287	3.310995	34.80352
3.259504	3.322068	3.398674	35.00767
3.310852	3.352328	3.40309	35.19579
3.078198	3.291202	3.402459	34.55196
3.246512	3.283587	3.446367	34.99941
3.242099	3.382184	3.359677	35.01579
3.393751	3.43347	3.454842	35.6677
3.186255	3.376927	3.453188	35.08667
3.328421	3.351789	3.415879	35.261
3.308748	3.385688	3.423262	35.30826
3.45541	3.311353	3.31241	35.22401
3.382409	3.246183	3.3474	34.99837
3.127772	3.202916	3.430014	34.52757
3.11742	3.346802	3.359472	34.66532
3.363726	3.470335	3.425632	35.61878
3.314399	3.426461	3.360359	35.27222
3.295262	3.358124	3.402561	35.17322
3.175285	3.27004	3.433885	34.78672
3.321905	3.419789	3.43334	35.43364
3.403732	3.542244	3.398827	35.8049
3.324833	3.422485	3.310244	35.17675
3.284868	3.154865	3.430969	34.76812
3.224282	3.193279	3.380957	34.61026
3.268453	3.313779	3.427406	35.07195
3.304706	3.436099	3.328745	35.20297
3.346445	3.307579	3.457809	35.29543
3.096633	3.278465	3.382168	34.52005
3.268412	3.275162	3.448567	35.03369
3.288869	3.423563	3.407847	35.3139
3.307305	3.399975	3.38609	35.25506
3.172686	3.434953	3.404778	35.07803
3.278104	3.410287	3.399049	35.24209
3.287095	3.328201	3.357426	34.99122
3.402536	3.291	3.302759	35.04277
3.248079	3.229267	3.39985	34.78232
3.267464	3.245442	3.342659	34.73502
3.162004	3.34296	3.414634	34.87505

3.392885	3.420328	3.323067	35.3489
3.260535	3.418374	3.36486	35.14659
3.298685	3.370322	3.373727	35.14433
3.170005	3.403615	3.525486	35.2676
3.378986	3.464808	3.344808	35.46331
3.302727	3.416959	3.452778	35.42802
3.404268	3.301042	3.370402	35.21644
3.27864	3.180542	3.436699	34.82318
3.244408	3.300839	3.415009	34.96396
3.232695	3.439468	3.436903	35.28938
3.245563	3.437446	3.36822	35.1629
3.244986	3.231761	3.341909	34.6543
3.18873	3.279813	3.440245	34.85141
3.238923	3.307714	3.431992	35.00414
3.320997	3.378612	3.461389	35.40295
3.363726	3.448903	3.377905	35.46754
3.224282	3.366818	3.485381	35.21812
3.322895	3.280486	3.307959	34.85699
3.319142	3.210599	3.371289	34.83444
3.308006	3.240522	3.343307	34.81433
3.332092	3.363852	3.389705	35.23817
3.304995	3.436301	3.347877	35.24588
3.163695	3.378746	3.3371	34.78745
3.363808	3.383397	3.360495	35.2864
3.211166	3.336491	3.406279	34.95014
3.258184	3.379892	3.397207	35.12803
3.206506	3.462449	3.448891	35.30858
3.324998	3.419452	3.477674	35.53662
3.230633	3.26384	3.384367	34.78592
3.335804	3.159245	3.435539	34.89908
3.262267	3.286956	3.445293	35.03888
3.364221	3.419857	3.403124	35.46025
3.278269	3.397886	3.398776	35.21474
3.213558	3.307646	3.405784	34.89121
3.240862	3.30367	3.358261	34.8383
3.285116	3.359337	3.373165	35.0894
3.222549	3.376657	3.413781	35.07928
3.279672	3.435088	3.461475	35.43627
3.230716	3.428483	3.465959	35.32457
3.444109	3.358259	3.419442	35.53594
3.372222	3.267547	3.374648	35.0824
3.310439	3.24989	3.335259	34.82254
3.258225	3.240657	3.4161	34.86495
3.288291	3.392427	3.390029	35.20559
3.305284	3.410624	3.40824	35.32236
3.312543	3.434885	3.353334	35.27122
3.207042	3.350441	3.451499	35.07052
3.201185	3.394584	3.446094	35.14242
3.318399	3.491227	3.376353	35.45758
3.274392	3.470941	3.427269	35.42833

3.311347	3.299828	3.401214	35.07797
3.207372	3.170028	3.427354	34.6239
3.279713	3.207634	3.392126	34.7873
3.357374	3.404356	3.379593	35.35992
3.332463	3.477883	3.439154	35.59649
3.195205	3.326314	3.385834	34.84827
3.275011	3.202782	3.354323	34.68374
3.172562	3.25737	3.360444	34.59246
3.302438	3.469054	3.349326	35.31508
3.201515	3.442838	3.503865	35.375
3.168851	3.377196	3.441388	35.02339
3.222013	3.271927	3.495015	35.02672
3.36851	3.263503	3.329905	34.96759
3.36187	3.253866	3.417857	35.12433
3.327844	3.275702	3.373403	35.00046
3.209104	3.336491	3.419084	34.97363
3.334484	3.399167	3.368595	35.27447
3.390616	3.390473	3.403806	35.45521
3.327143	3.369514	3.353487	35.16053
3.163365	3.377264	3.387249	34.89315
3.205351	3.410691	3.389142	35.06221
3.271217	3.440816	3.443366	35.3907
3.370655	3.354822	3.379798	35.28109
3.337165	3.15446	3.406603	34.82831
3.198711	3.151562	3.416032	34.53982
3.266556	3.347341	3.397821	35.0765
3.359106	3.415678	3.378587	35.38627
3.378738	3.373355	3.354374	35.2837
3.174501	3.253125	3.441047	34.76368
3.219003	3.335278	3.391478	34.93225
3.265649	3.363785	3.399867	35.11495
3.317822	3.369918	3.319145	35.06593
3.184853	3.381375	3.439069	35.06246
3.202464	3.409074	3.479669	35.25033
3.165963	3.295717	3.480282	34.92396
3.489436	3.320384	3.324141	35.34382
3.332133	3.266469	3.333912	34.90329
3.360962	3.291809	3.411667	35.19179
3.25307	3.353204	3.412673	35.09231
3.353332	3.353002	3.361604	35.19944
3.307552	3.411769	3.358585	35.22124
3.427529	3.363246	3.293517	35.23521
3.211909	3.388788	3.456888	35.1768
3.161385	3.448027	3.372738	35.01184
3.29489	3.461978	3.454688	35.51351
3.291962	3.314722	3.376046	35.01311
3.344094	3.150686	3.382475	34.78245
3.169799	3.202175	3.463282	34.6906
3.239377	3.375579	3.445838	35.18382
3.397751	3.463797	3.42657	35.68094

3.285734	3.373827	3.366583	35.10804
3.269608	3.208914	3.360853	34.69961
3.201392	3.241398	3.383208	34.67036
3.224199	3.424844	3.443502	35.25325
3.29984	3.557946	3.378587	35.56778
3.290354	3.464539	3.371749	35.32782
3.299345	3.235265	3.419766	34.9511
3.201722	3.189572	3.384964	34.56158
3.295592	3.230885	3.373096	34.83125
3.332793	3.368772	3.380429	35.23018
3.424807	3.466695	3.419835	35.73172
3.257153	3.445466	3.430492	35.34196
3.379646	3.342084	3.39037	35.29602
3.313821	3.331706	3.356011	35.05423
3.311429	3.411095	3.371476	35.25644
3.188854	3.440749	3.370863	35.05189
3.20498	3.470335	3.456137	35.33834
3.12703	3.258853	3.409825	34.60412
3.324586	3.166052	3.354237	34.71164
3.280868	3.201568	3.439342	34.87981
3.354075	3.424305	3.415606	35.47509
3.270887	3.473974	3.433544	35.44101
3.344382	3.371131	3.420943	35.34928
3.339186	3.237422	3.329734	34.84606
3.381048	3.286956	3.359353	35.1107
3.17314	3.413319	3.434772	35.0973
3.237933	3.494259	3.405801	35.35264
3.20036	3.434211	3.46642	35.27172
3.274021	3.303198	3.307431	34.79862
3.3337	3.134983	3.352805	34.6605
3.17281	3.187146	3.411599	34.5513
3.210094	3.337569	3.473649	35.09748
3.271258	3.465819	3.33294	35.20399
3.407526	3.448566	3.349395	35.50024
3.328751	3.36729	3.343785	35.13796
3.344712	3.320316	3.400191	35.1935
3.294808	3.459349	3.411667	35.4135
3.315388	3.465145	3.3142	35.25804
3.308583	3.404963	3.440774	35.38835
3.25274	3.264514	3.43131	34.93839
3.108883	3.095895	3.389023	34.16258
3.329287	3.177172	3.340801	34.71685
3.354528	3.395932	3.420056	35.42376
3.319018	3.457799	3.40783	35.45467
3.214631	3.353474	3.485517	35.16813
3.371438	3.32409	3.369516	35.19311
3.279135	3.308185	3.377086	34.97304
3.421508	3.384812	3.323459	35.33468
3.160561	3.425248	3.465959	35.16408
3.148889	3.3559	3.395417	34.83264

3.145713	3.331571	3.428719	34.84531
3.267175	3.28298	3.347655	34.8274
3.308501	3.194829	3.458047	34.96641
3.272619	3.29592	3.391307	34.96306
3.218549	3.304209	3.380957	34.84031
3.421508	3.410556	3.395297	35.54808
3.440562	3.402132	3.329376	35.42716
3.314522	3.361561	3.382867	35.17979
3.283095	3.419789	3.397275	35.26991
3.248739	3.430909	3.446094	35.32585
3.304789	3.462247	3.353999	35.31556
3.342527	3.450858	3.36897	35.40591
3.271382	3.24187	3.425155	34.91618
3.12604	3.200692	3.440518	34.54189
3.242924	3.236883	3.412434	34.81522
3.314893	3.391686	3.348985	35.17238
3.35523	3.344578	3.393251	35.25438
3.235211	3.356911	3.43322	35.10629
3.284868	3.28453	3.390864	34.96397
3.283301	3.253933	3.358125	34.82204
3.364303	3.320316	3.331661	35.08647
3.254554	3.446882	3.451295	35.38487
3.247007	3.479433	3.44826	35.43291
3.152848	3.335143	3.383481	34.7698
3.282064	3.22765	3.32953	34.69932
3.226798	3.226302	3.449999	34.83896
3.215043	3.334738	3.43484	35.01724
3.241068	3.403413	3.409246	35.16836
3.430499	3.440412	3.38227	35.60454
3.436974	3.341006	3.364519	35.3625
3.457183	3.339254	3.383975	35.44541
3.260823	3.409411	3.421267	35.25097
3.246017	3.423496	3.351049	35.09584
3.222343	3.378746	3.346428	34.9361
3.250966	3.315936	3.397548	34.97313
3.252245	3.228256	3.411326	34.81432
3.187493	3.275567	3.466965	34.89785
3.202051	3.442097	3.494401	35.35386
3.412558	3.483342	3.443383	35.79283
3.320833	3.351654	3.396457	35.20164
3.266515	3.28271	3.336589	34.80117
3.325369	3.227785	3.467579	35.09621
3.254719	3.341613	3.373864	34.98569
3.340341	3.439603	3.378792	35.398
3.335474	3.412376	3.370726	35.31018
3.269484	3.317351	3.414941	35.05476
3.116513	3.200827	3.365832	34.35802
3.294602	3.161537	3.390029	34.71446
3.229107	3.376455	3.409945	35.08478
3.262143	3.496753	3.433629	35.47189

3.225354	3.429224	3.416424	35.20614
3.364427	3.385014	3.382952	35.34039
3.357168	3.310072	3.300133	34.97952
3.370984	3.392225	3.40205	35.41227
3.282889	3.414196	3.39794	35.25868
3.276661	3.390743	3.354988	35.09984
3.157426	3.185327	3.397804	34.48351
3.219951	3.222393	3.268997	34.41962
3.161633	3.251035	3.480351	34.81692
3.302026	3.441423	3.375722	35.31148
3.262184	3.470671	3.495288	35.54979
3.340959	3.380229	3.373113	35.2571
3.33832	3.293493	3.475713	35.28601
3.476691	3.34296	3.315274	35.34593
3.31609	3.311285	3.451755	35.22392
3.263463	3.358191	3.365201	35.02213
3.241398	3.337636	3.411514	35.0302
3.349497	3.323012	3.390387	35.18841
3.294066	3.284867	3.335464	34.86367
3.215291	3.211138	3.399509	34.67023
3.148188	3.245374	3.448635	34.70578
3.245646	3.397145	3.468875	35.29507
3.292952	3.464876	3.437108	35.47717
3.348878	3.433875	3.37313	35.39176
3.359271	3.349228	3.395297	35.27786
3.282517	3.397954	3.444747	35.3247
3.392019	3.470941	3.324227	35.46022
3.353209	3.427742	3.420056	35.49044
3.254059	3.339591	3.324977	34.87292
3.172315	3.145901	3.437091	34.51577
3.262102	3.204197	3.377513	34.70931
3.136846	3.401728	3.443434	35.01153
3.260906	3.51441	3.376797	35.38352
3.21892	3.39054	3.505024	35.30123
3.267051	3.342623	3.447595	35.17611
3.251997	3.293493	3.344603	34.81053
3.465473	3.310948	3.400259	35.43725
3.319554	3.349026	3.392262	35.18392
3.294478	3.374635	3.446708	35.30416
3.160231	3.340332	3.376268	34.78152
3.341702	3.232435	3.340443	34.86407
3.303799	3.262358	3.302435	34.7635
3.211867	3.248137	3.411667	34.77024
3.172769	3.395999	3.441882	35.07416
3.294189	3.423226	3.502364	35.5315
3.390451	3.457597	3.412076	35.61972
3.425921	3.366885	3.357375	35.37929
3.328957	3.343028	3.419766	35.25152
3.348466	3.436031	3.409689	35.47553
3.307305	3.437109	3.429469	35.43113

3.297819	3.324427	3.28835	34.85536
3.398329	3.163558	3.357767	34.87517
3.083105	3.15446	3.34815	34.1449
3.205145	3.281902	3.469847	34.95661
3.238346	3.487789	3.402527	35.33224
3.316626	3.488666	3.443451	35.59483
3.273774	3.376927	3.374188	35.1053
3.312584	3.297604	3.440501	35.16172
3.23583	3.349632	3.387164	34.99101
3.410248	3.381981	3.352652	35.3677
3.273361	3.323955	3.402442	35.05034
3.319843	3.343432	3.38256	35.15111
3.246306	3.231019	3.372568	34.72261
3.400886	3.234591	3.327415	34.96972
3.194545	3.248811	3.380957	34.66667
3.26866	3.340939	3.354834	34.97309
3.144764	3.378746	3.459752	35.01427
3.371438	3.475996	3.376916	35.54149
3.26004	3.412174	3.477213	35.37764
3.489394	3.395123	3.321856	35.50218
3.329782	3.40348	3.452932	35.45805
3.355601	3.426663	3.310261	35.25321
3.252946	3.401256	3.459275	35.29903
3.26371	3.26411	3.356505	34.79791
3.167077	3.125009	3.364536	34.29996
3.225272	3.185394	3.363888	34.55786
3.179244	3.40658	3.370061	34.9544
3.236696	3.451195	3.485398	35.42983
3.263463	3.426394	3.376745	35.19652
3.253028	3.328471	3.444065	35.10678
3.345207	3.387036	3.389005	35.31602
3.239665	3.355563	3.422648	35.08997
3.281239	3.366346	3.371067	35.09166
3.3337	3.355024	3.420227	35.28913
3.394493	3.354754	3.383361	35.34087
3.201969	3.223404	3.367299	34.59748
3.313904	3.215115	3.318924	34.71835
3.182708	3.288372	3.422444	34.81803
3.236696	3.383734	3.366208	35.02165
3.265855	3.43219	3.457024	35.38998
3.291261	3.451195	3.400328	35.36312
3.263133	3.386901	3.377427	35.11093
3.372552	3.380634	3.368339	35.31663
3.285074	3.419452	3.423944	35.33182
3.477557	3.425652	3.378502	35.66693
3.224942	3.321529	3.371272	34.87099
3.22622	3.170365	3.336708	34.46763
3.321492	3.213834	3.302401	34.69601
3.26536	3.375175	3.397463	35.13397
3.211991	3.499044	3.383344	35.25727

3.220529	3.429292	3.433817	35.23377
3.185925	3.352261	3.442138	35.00784
3.3622	3.341208	3.399253	35.27538
3.362736	3.340197	3.417192	35.31357
3.195741	3.334738	3.420397	34.94345
3.254307	3.338378	3.418607	35.07557
3.276785	3.348824	3.387045	35.07854
3.37317	3.269906	3.367043	35.073
3.266597	3.194896	3.390711	34.72767
3.232324	3.249081	3.413168	34.82032
3.207661	3.365133	3.463333	35.12988
3.27134	3.434683	3.386482	35.25317
3.23319	3.499786	3.361058	35.25651
3.345909	3.4053	3.427082	35.44076
3.206836	3.356237	3.358671	34.87974
3.356838	3.396471	3.394394	35.37387
3.370118	3.447151	3.335464	35.38487
3.369005	3.367424	3.396252	35.34103
3.227086	3.223539	3.396525	34.71661
3.236284	3.126424	3.355942	34.43561
3.246306	3.282845	3.354476	34.79638
3.358488	3.449577	3.360563	35.41964
3.19405	3.451127	3.433544	35.22302
3.096056	3.404356	3.392348	34.81635
3.184317	3.301918	3.409007	34.82178
3.292869	3.367963	3.411104	35.20819
3.325122	3.39883	3.433885	35.39604
3.236325	3.369648	3.471075	35.21936
3.161221	3.330358	3.456137	34.93654
3.344836	3.300031	3.311046	34.95446
3.413878	3.223741	3.404062	35.14202
3.316956	3.248542	3.351253	34.86882
3.130783	3.277656	3.421284	34.6785
3.190132	3.366009	3.35729	34.86156
3.315553	3.431583	3.404284	35.382
3.400309	3.454362	3.306442	35.4032
3.351476	3.410489	3.435335	35.48234
3.163035	3.422283	3.404096	35.02772
3.307222	3.46656	3.404556	35.44087
3.367603	3.411769	3.385851	35.41219
3.378037	3.289854	3.344279	35.07749
3.206135	3.103443	3.427576	34.47607
3.25542	3.264312	3.451295	34.98751
3.243872	3.410556	3.445276	35.26891
3.34727	3.429898	3.414941	35.47098
3.204609	3.396404	3.403175	35.06003
3.177512	3.310611	3.511845	35.0508
3.107852	3.33521	3.40899	34.72733
3.391606	3.430505	3.371681	35.47466
3.276661	3.381307	3.44814	35.28291

3.318275	3.310948	3.333912	34.97026
3.232613	3.222326	3.440638	34.82251
3.303139	3.233783	3.315547	34.72825
3.357622	3.1798	3.387317	34.88629
3.387482	3.311892	3.303424	35.05699
3.135402	3.347611	3.447936	34.89987
3.242181	3.471952	3.373045	35.24152
3.238593	3.472559	3.458474	35.42182
3.413671	3.452003	3.302793	35.41928
3.322359	3.405232	3.410149	35.35209
3.173181	3.372277	3.46896	35.0824
3.336299	3.352059	3.37741	35.1947
3.375934	3.262695	3.327483	34.97676
3.341702	3.201097	3.353811	34.82477
3.205475	3.205006	3.407916	34.65373
3.218095	3.360348	3.413355	35.03294
3.21694	3.417767	3.385953	35.09605
3.419446	3.411298	3.364741	35.47837
3.236201	3.366548	3.482516	35.23733
3.272042	3.445129	3.389295	35.2837
3.157839	3.40813	3.461099	35.11007
3.338691	3.39081	3.392245	35.31711
3.3151	3.276173	3.391955	35.0142
3.271175	3.26903	3.373403	34.86195
3.214672	3.251777	3.401419	34.76192
3.412145	3.209184	3.389773	35.07515
3.251379	3.25272	3.370487	34.77661
3.319142	3.311151	3.345268	34.99743
3.112058	3.356372	3.424763	34.8173
3.190215	3.444118	3.381435	35.08535
3.293034	3.467167	3.351287	35.29468
3.356962	3.428079	3.409212	35.47567
3.295262	3.407726	3.440024	35.36361
3.301283	3.416352	3.454296	35.42686
3.234139	3.359202	3.442957	35.13025
3.371067	3.269973	3.335805	35.00024
3.381873	3.173196	3.312563	34.7614
3.36187	3.267816	3.383225	35.07911
3.22688	3.372142	3.434652	35.12451
3.187245	3.396067	3.421284	35.06092
3.316956	3.36055	3.328796	35.06465
3.29918	3.372209	3.38846	35.18175
3.181017	3.405839	3.486762	35.21186
3.230056	3.440681	3.412349	35.23257
3.286106	3.386429	3.462464	35.34609
3.291343	3.293426	3.357375	34.92435
3.264205	3.267479	3.406875	34.91651
3.207908	3.256494	3.419988	34.79805
3.37416	3.253327	3.389824	35.08873
3.242882	3.323281	3.396542	34.96932

3.295179	3.301513	3.381042	35.00218
3.257441	3.354148	3.382185	35.03726
3.20333	3.407389	3.403874	35.08279
3.276661	3.458406	3.369311	35.27913
3.344135	3.480309	3.347809	35.42756
3.259091	3.395797	3.453256	35.28737
3.262803	3.396875	3.382679	35.1435
3.248739	3.275095	3.422103	34.93264
3.326112	3.286013	3.438728	35.16208
3.407609	3.283721	3.320765	35.07732
3.236407	3.335951	3.355789	34.89374
3.265113	3.398358	3.418061	35.22917
3.261854	3.40503	3.382662	35.15922
3.276083	3.367357	3.390318	35.1247
3.273279	3.406378	3.347468	35.11019
3.207661	3.393371	3.434567	35.12872
3.154787	3.436301	3.487768	35.22332
3.261524	3.351924	3.437398	35.16206
3.231953	3.271927	3.402459	34.84605
3.280002	3.24005	3.39823	34.87217
3.232943	3.27031	3.407421	34.85553
3.302438	3.223471	3.416646	34.92525
3.218879	3.343432	3.362183	34.88575
3.3589	3.329616	3.328507	35.0881
3.279754	3.358663	3.416203	35.17032
3.308542	3.479163	3.38568	35.43004
3.205516	3.423765	3.367333	35.04347
3.333494	3.472761	3.376473	35.45047
3.246058	3.416285	3.437585	35.2694
3.285157	3.327325	3.444594	35.17569
3.238387	3.321125	3.417055	34.99963
3.362117	3.24315	3.388886	35.03809
3.356549	3.250025	3.326358	34.9042
3.378161	3.393977	3.387573	35.40014
3.240779	3.429494	3.394411	35.19233
3.247131	3.375309	3.371374	35.03734
3.199288	3.364796	3.404284	34.9817
3.224364	3.419654	3.369311	35.08002
3.229396	3.440883	3.443298	35.29925
3.18774	3.459147	3.476327	35.32032
3.18308	3.261751	3.482994	34.89304
3.261648	3.201299	3.416152	34.78648
3.319678	3.242207	3.412826	34.99557
3.273279	3.258718	3.406875	34.9172
3.320131	3.215856	3.434107	34.98547
3.274392	3.389058	3.351884	35.08441
3.369252	3.339254	3.323374	35.12059
3.322977	3.374164	3.351714	35.15771
3.292704	3.413656	3.4109	35.3073
3.148971	3.422081	3.368561	34.91881

3.331391	3.459619	3.394547	35.45666
3.283301	3.37996	3.324551	35.02422
3.292787	3.313779	3.390114	35.04361
3.218879	3.279004	3.43779	34.9102
3.388843	3.252383	3.41165	35.1665
3.295592	3.317553	3.377018	35.02936
3.371149	3.416487	3.354732	35.36221
3.285074	3.393304	3.42142	35.26912
3.242594	3.351452	3.355738	34.94106
3.131649	3.360213	3.373864	34.75724
3.273898	3.461506	3.371766	35.28524
3.16617	3.440749	3.464936	35.20801
3.249316	3.464741	3.396645	35.29296
3.147693	3.274421	3.485313	34.84843
3.264123	3.196783	3.39187	34.72892
3.418992	3.226706	3.396866	35.14395
3.292869	3.259864	3.405648	34.95986
3.305985	3.294774	3.445071	35.15109
3.280744	3.400717	3.379746	35.18472
3.321822	3.330223	3.376132	35.11249
3.369582	3.424305	3.308727	35.27527
3.325122	3.420935	3.439342	35.45631
3.121338	3.444455	3.377069	34.92592
3.288745	3.390473	3.400396	35.22498
3.293942	3.245846	3.37209	34.85816
3.253936	3.272534	3.403124	34.89691
3.231953	3.29073	3.4013	34.88464
3.287838	3.317149	3.427013	35.12085
3.29555	3.363583	3.367248	35.10856
3.39074	3.395528	3.332667	35.31096
3.272083	3.368637	3.414122	35.1708
3.290725	3.348824	3.316587	34.95495
3.184647	3.446005	3.429179	35.18171
3.238139	3.47667	3.343648	35.17871
3.161221	3.380297	3.425223	34.97814
3.25175	3.294437	3.335651	34.79247
3.174377	3.17825	3.487291	34.7008
3.293323	3.234861	3.36943	34.82697
3.268453	3.259123	3.433681	34.96615
3.404474	3.242881	3.323851	34.98791
3.317863	3.354889	3.480197	35.38535
3.282559	3.332245	3.377564	35.03418
3.291508	3.348621	3.377973	35.09046
3.285198	3.441423	3.35954	35.23929
3.247914	3.462315	3.386073	35.26147
3.271382	3.460091	3.341125	35.20963
3.212734	3.337636	3.420773	34.98777
3.246677	3.23412	3.416169	34.82555
3.327679	3.25919	3.37932	34.97693
3.277568	3.342758	3.399936	35.09518

3.335226	3.3652	3.407353	35.28657
3.275877	3.340062	3.382816	35.04815
3.268453	3.349498	3.375654	35.03689
3.305944	3.36702	3.352106	35.1057
3.271093	3.44641	3.34653	35.1909
3.150827	3.438727	3.472064	35.18562
3.23616	3.438794	3.433749	35.28859
3.145301	3.361628	3.456325	34.97051
3.267051	3.239242	3.374017	34.78913
3.205062	3.159919	3.439751	34.62385
3.251626	3.233985	3.462412	34.93721
3.16617	3.279341	3.427269	34.77266
3.429798	3.385081	3.362541	35.43886
3.320626	3.410354	3.364434	35.25953
3.404804	3.326719	3.425922	35.39518
3.285528	3.346802	3.359898	35.03388
3.299097	3.418037	3.402255	35.31196
3.195164	3.411837	3.37209	35.00515
3.347599	3.307511	3.3511	35.06445
3.151157	3.243824	3.393422	34.58814
3.227994	3.240387	3.423995	34.81552
3.213393	3.335682	3.452318	35.05392
3.380718	3.425248	3.424029	35.55384
3.351971	3.349969	3.42067	35.319
3.331721	3.30987	3.393916	35.12852
3.203413	3.319777	3.479652	35.05709
3.40068	3.446477	3.351611	35.48555
3.253441	3.409882	3.407643	35.20606
3.204774	3.403682	3.35954	34.98088
3.165757	3.315396	3.484085	34.97485
3.225107	3.290326	3.407984	34.8834
3.280868	3.248205	3.41315	34.92452
3.256617	3.218686	3.385595	34.74668
3.282146	3.319575	3.441371	35.14511
3.25839	3.35745	3.42877	35.14843
3.323555	3.385755	3.42304	35.3403
3.420188	3.400784	3.343785	35.41117
3.455492	3.348217	3.328592	35.34019
3.174047	3.400447	3.472388	35.1534
3.291261	3.436099	3.410286	35.35188
3.225849	3.387305	3.407984	35.0971
3.293901	3.249485	3.278511	34.66139
3.210506	3.215452	3.447134	34.77335
3.246677	3.346532	3.40517	35.04733
3.191246	3.406513	3.426792	35.10456
3.44349	3.421609	3.333213	35.48455
3.281445	3.304007	3.431362	35.08764
3.356756	3.342152	3.36677	35.1945
3.250801	3.403008	3.435454	35.24608
3.305779	3.454025	3.351833	35.295

3.244532	3.366885	3.357699	34.98333
3.291591	3.343634	3.37857	35.08104
3.157096	3.324292	3.386891	34.76282
3.133917	3.254742	3.4892	34.78377
3.171408	3.270445	3.395621	34.69545
3.25674	3.239511	3.480129	34.99922
3.308666	3.388384	3.417857	35.30215
3.40134	3.359539	3.470717	35.55733
3.322771	3.354822	3.375023	35.16593
3.395854	3.358865	3.339147	35.25614
3.325617	3.37322	3.390182	35.24554
3.289199	3.461236	3.412144	35.40641
3.251379	3.403547	3.332514	35.0234
3.291137	3.266334	3.368697	34.88941
3.290725	3.252585	3.360427	34.84036
3.240779	3.308455	3.372039	34.87871
3.319018	3.379825	3.393643	35.25312
3.245481	3.445264	3.398776	35.24664
3.290849	3.292415	3.391683	34.99608
3.400267	3.356372	3.340187	35.26262
3.392431	3.436705	3.427218	35.61148
3.258802	3.44614	3.403465	35.28794
3.31543	3.390945	3.410456	35.30636
3.181677	3.280621	3.374154	34.69322
3.23616	3.308994	3.420193	34.97509
3.160272	3.299424	3.444662	34.84172
3.142249	3.286889	3.382065	34.638
3.093458	3.21653	3.437295	34.49822
3.273403	3.289248	3.404761	34.97961
3.345455	3.346734	3.514045	35.50187
3.441923	3.412646	3.335668	35.46689
3.209846	3.363987	3.471552	35.15012
3.277073	3.350778	3.316229	34.92859
3.38641	3.402469	3.319947	35.28886
3.407155	3.402267	3.273055	35.23124
3.197762	3.316879	3.398708	34.86138
3.177883	3.274017	3.369703	34.66075
3.258349	3.27058	3.406483	34.90963
3.312501	3.436031	3.369243	35.30843
3.352796	3.458878	3.415879	35.54849
3.211702	3.350441	3.533108	35.25917
3.216982	3.291	3.406057	34.86289
3.385131	3.345252	3.374495	35.28022
3.398865	3.500729	3.37088	35.64236
3.255915	3.408939	3.462242	35.32881
3.203495	3.293493	3.381622	34.78541
3.168314	3.244903	3.503933	34.86969
3.357745	3.347543	3.293466	35.04815
3.239171	3.261886	3.393592	34.82049
3.226674	3.253192	3.418266	34.8281

3.081126	3.291269	3.448754	34.65975
3.306521	3.372748	3.434806	35.30034
3.334278	3.393169	3.386158	35.29931
3.343021	3.398291	3.398367	35.35633
3.164974	3.318968	3.49493	35.00467
3.378243	3.366885	3.430066	35.43399
3.351353	3.395999	3.339761	35.24137
3.399855	3.317283	3.305316	35.09998
3.20498	3.243757	3.392143	34.7029
3.061288	3.282576	3.388954	34.46659
3.223416	3.388114	3.424541	35.12975
3.33931	3.457328	3.368015	35.41094
3.300582	3.411163	3.432726	35.36681
3.295715	3.298346	3.407165	35.05355
3.199659	3.373085	3.426348	35.04889
3.349827	3.463191	3.421403	35.56351
3.457431	3.485633	3.39835	35.79749
3.294395	3.324764	3.368083	35.02297
3.195246	3.172859	3.376882	34.4932
3.208444	3.249553	3.35185	34.63504
3.283796	3.35435	3.404812	35.14481
3.240697	3.300772	3.372176	34.86203
3.162417	3.30832	3.449709	34.8769
3.132061	3.386227	3.430475	34.93883
3.319389	3.410354	3.439904	35.42186
3.444686	3.420665	3.393166	35.61621
3.292086	3.388114	3.434243	35.30114
3.195618	3.359202	3.38834	34.92657
3.321039	3.375175	3.458371	35.38892
3.295715	3.282845	3.320833	34.83086
3.413506	3.257505	3.311063	35.01167
3.196731	3.221652	3.370385	34.58894
3.232324	3.42107	3.389432	35.14453
3.171573	3.467976	3.432726	35.20892
3.35061	3.447084	3.405528	35.49529
3.230963	3.325034	3.360257	34.86773
3.284291	3.340669	3.406824	35.12038
3.214012	3.49527	3.51546	35.54235
3.440892	3.516162	3.3954	35.82163
3.280744	3.342623	3.394973	35.09098
3.342815	3.216462	3.379133	34.91619
3.258761	3.217069	3.376831	34.72867
3.242264	3.288911	3.37163	34.83832
3.142991	3.288304	3.410098	34.70402
3.272083	3.301985	3.362149	34.91139
3.165716	3.331773	3.507343	35.06144
3.290065	3.388182	3.347212	35.10655
3.235376	3.342354	3.437773	35.08477
3.302644	3.392225	3.455575	35.37987
3.3549	3.383329	3.437364	35.43487

3.322771	3.431651	3.447083	35.49153
3.368221	3.354417	3.374767	35.26388
3.331143	3.249351	3.354101	34.90784
3.249935	3.21006	3.396116	34.73621
3.212692	3.294639	3.381844	34.80852
3.376016	3.502616	3.396849	35.65331
3.18906	3.402132	3.392024	35.01417
3.267175	3.368435	3.393047	35.11354
3.209352	3.300502	3.396969	34.84711
3.238263	3.46063	3.403346	35.27445
3.249976	3.505851	3.427815	35.45247
3.317863	3.386429	3.411582	35.30427
3.23418	3.180272	3.352038	34.54022
3.377089	3.161402	3.268366	34.6285
3.308377	3.236007	3.409894	34.95088
3.288663	3.34478	3.354783	35.02512
3.144104	3.242813	3.386755	34.55593
3.294313	3.286552	3.348917	34.89732
3.224612	3.392023	3.413645	35.11709
3.301036	3.444523	3.435403	35.44661
3.230303	3.378679	3.481084	35.24783
3.295674	3.381173	3.48047	35.3949
3.26536	3.351452	3.463214	35.22588
3.4783	3.423092	3.361467	35.6257
3.415486	3.213295	3.353282	35.01165
3.221065	3.151091	3.357477	34.45962
3.186874	3.273478	3.34965	34.63538
3.225808	3.388451	3.393388	35.06759
3.317327	3.495944	3.378911	35.47114
3.233561	3.389934	3.426996	35.16129
3.178996	3.294033	3.40442	34.78287
3.268123	3.36675	3.415128	35.16022
3.370118	3.512119	3.43247	35.73909
3.282476	3.446275	3.452028	35.44621
3.287178	3.269569	3.47353	35.11708
3.237562	3.198199	3.359489	34.60312
3.307263	3.264447	3.397122	34.98271
3.391441	3.426731	3.331729	35.37868
3.321657	3.333188	3.42229	35.21956
3.114822	3.316812	3.378792	34.6363
3.272248	3.275028	3.423518	34.987
3.268536	3.350306	3.396218	35.08381
3.32009	3.409208	3.390318	35.31246
3.218673	3.40685	3.439819	35.19376
3.292746	3.36702	3.515869	35.43496
3.271134	3.41615	3.397804	35.23695
3.40435	3.275836	3.348423	35.11344
3.313862	3.256023	3.426246	35.04241
3.332381	3.306366	3.380565	35.0931
3.217889	3.340467	3.420261	35.00411

3.326153	3.410893	3.287174	35.10384
3.362035	3.36237	3.395502	35.31309
3.240243	3.350306	3.308317	34.82971
3.153302	3.387777	3.464305	35.06265
3.265649	3.482668	3.393797	35.36165
3.33898	3.416083	3.513226	35.63758
3.352549	3.264245	3.329189	34.93274
3.273609	3.176228	3.469165	34.87374
3.214425	3.301783	3.3895	34.84467
3.288745	3.391282	3.399612	35.22503
3.306769	3.32719	3.366344	35.05153
3.324792	3.26566	3.349071	34.91862
3.122658	3.247935	3.427542	34.60943
3.18972	3.384542	3.405443	35.00649
3.282682	3.439468	3.420107	35.36197
3.304624	3.442029	3.444866	35.46969
3.143156	3.32254	3.433476	34.83038
3.336175	3.344915	3.439273	35.31409
3.250925	3.387036	3.419784	35.17715
3.508243	3.28143	3.320441	35.29167
3.327761	3.268895	3.438182	35.12706
3.244491	3.325101	3.37209	34.92334
3.291756	3.389327	3.447117	35.33123
3.350033	3.450588	3.305538	35.28303
3.184647	3.342084	3.399987	34.89062
3.271093	3.372546	3.357358	35.05305
3.208032	3.393775	3.469284	35.20634
3.255709	3.51751	3.423739	35.48159
3.286889	3.322742	3.408257	35.08999
3.279012	3.238028	3.415128	34.90254
3.284662	3.211138	3.353351	34.72099
3.287549	3.259123	3.457689	35.06041
3.260081	3.349093	3.399049	35.06885
3.33436	3.285608	3.429179	35.15835
3.245646	3.316407	3.358995	34.87822
3.274805	3.287495	3.389551	34.94558
3.2395	3.36082	3.412196	35.07824
3.241604	3.45416	3.448908	35.36725
3.254307	3.449173	3.431651	35.34638
3.274104	3.371131	3.381503	35.10934
3.327266	3.31816	3.38447	35.11625
3.298272	3.26694	3.40928	34.99509
3.301489	3.306298	3.427627	35.12832
3.266185	3.382453	3.384981	35.12439
3.424807	3.440681	3.434311	35.70649
3.239954	3.402873	3.375842	35.0917
3.285734	3.344039	3.396934	35.10928
3.214136	3.318429	3.420807	34.9489
3.294602	3.387777	3.341466	35.10302
3.248368	3.502683	3.40338	35.38859

3.249688	3.437446	3.421762	35.28901
3.164932	3.186944	3.476395	34.67533
3.343351	3.18627	3.369976	34.83131
3.30714	3.32901	3.425087	35.18479
3.389709	3.435627	3.421148	35.58989
3.280331	3.374164	3.391052	35.15048
3.252575	3.258112	3.411121	34.87988
3.231912	3.250833	3.337987	34.65884
3.40435	3.320855	3.365952	35.25022
3.201309	3.423496	3.413764	35.13522
3.18543	3.453756	3.447271	35.23994
3.220281	3.371468	3.473649	35.19389
3.306109	3.250766	3.309613	34.7589
3.411692	3.217204	3.414719	35.14625
3.228736	3.249216	3.405921	34.79692
3.182461	3.362505	3.447936	35.03535
3.261277	3.415004	3.368015	35.14774
3.448728	3.423698	3.36793	35.57649
3.340506	3.390675	3.374716	35.28245
3.234593	3.302255	3.316366	34.72987
3.208568	3.34505	3.430083	35.01523
3.324874	3.503762	3.369362	35.48386
3.306109	3.432055	3.39407	35.34005
3.277238	3.242409	3.401095	34.87755
3.137753	3.129322	3.415009	34.35564
3.2501	3.224482	3.433425	34.8497
3.38843	3.42235	3.37313	35.45306
3.43285	3.476198	3.381503	35.68626
3.200196	3.29255	3.510498	35.05796
3.31609	3.335008	3.411803	35.18843
3.238304	3.321394	3.382679	34.92486
3.390616	3.461506	3.329137	35.44726
3.2355	3.406176	3.468125	35.29099
3.170624	3.43812	3.404386	35.07959
3.134701	3.316677	3.538173	35.02802
3.336464	3.25454	3.299877	34.81225
3.359849	3.299289	3.427491	35.24032
3.322194	3.262762	3.398213	35.01407
3.170706	3.334401	3.445975	34.9439
3.304211	3.373018	3.370402	35.15504
3.440232	3.447892	3.306493	35.47647
3.36752	3.400447	3.371357	35.35555
3.129711	3.367155	3.450886	34.93661
3.230881	3.4764	3.42466	35.33941
3.277238	3.487048	3.436341	35.48961
3.31444	3.400784	3.383481	35.26672
3.34962	3.217676	3.452932	35.09511
3.116925	3.183507	3.460162	34.52733
3.274599	3.306029	3.414515	35.04025
3.290642	3.374433	3.462395	35.32963

3.416765	3.375916	3.372858	35.41288
3.239995	3.209925	3.425632	34.77873
3.304294	3.240252	3.37591	34.87692
3.298932	3.339726	3.386823	35.10659
3.37878	3.435829	3.326818	35.36015
3.242676	3.431044	3.438728	35.29678
3.270062	3.393236	3.434652	35.26507
3.205516	3.295178	3.367538	34.76272
3.329122	3.267075	3.38227	35.00378
3.358488	3.307444	3.393166	35.18011
3.294313	3.39391	3.386345	35.21394
3.237026	3.372951	3.473786	35.23405
3.407279	3.365942	3.339556	35.2975
3.310357	3.288574	3.366054	34.9743
3.394493	3.36392	3.302895	35.18494
3.166541	3.404828	3.395485	34.97839
3.114698	3.470537	3.396866	35.01173
3.168438	3.423429	3.372568	34.9731
3.254307	3.224617	3.353334	34.68405
3.245068	3.221517	3.426604	34.81729
3.229148	3.316677	3.45109	35.04413
3.222055	3.461439	3.445361	35.33266
3.392307	3.438659	3.429878	35.6213
3.381832	3.335412	3.370181	35.24206
3.254926	3.203253	3.317952	34.56131
3.263999	3.283047	3.358261	34.8438
3.26371	3.401863	3.376319	35.14248
3.301531	3.477546	3.367674	35.37179
3.20865	3.410893	3.408666	35.11256
3.289652	3.283721	3.397071	34.98624
3.176934	3.15601	3.372039	34.40572
3.272784	3.224078	3.378178	34.7776
3.335639	3.350576	3.404232	35.24867
3.34595	3.497292	3.426638	35.64106
3.268907	3.41952	3.36897	35.17639
3.398824	3.339321	3.383003	35.3158
3.329246	3.350711	3.331235	35.07535
3.319059	3.399436	3.415811	35.34458
3.192772	3.472356	3.393524	35.17914
3.275465	3.375646	3.379542	35.11791
3.102902	3.216665	3.386755	34.40864
3.233273	3.208577	3.364042	34.62639
3.278929	3.255281	3.469983	35.06004
3.300252	3.447825	3.384299	35.34036
3.258967	3.422552	3.439461	35.31544
3.277774	3.358259	3.384436	35.09563
3.394163	3.324697	3.361928	35.22754
3.371809	3.294774	3.339556	35.0643
3.216322	3.363179	3.412417	35.0332
3.193927	3.417498	3.373045	35.01691

3.282641	3.394382	3.394138	35.20649
3.19702	3.374029	3.351577	34.88167
3.294066	3.238028	3.385118	34.86983
3.210795	3.21781	3.413509	34.7056
3.151776	3.27058	3.440177	34.75025
3.234593	3.387508	3.424404	35.15257
3.342114	3.406243	3.418829	35.41648
3.346692	3.412511	3.366395	35.32553
3.388018	3.389462	3.379064	35.39321
3.297901	3.437109	3.451755	35.4593
3.369294	3.431785	3.343103	35.36617
3.296251	3.399504	3.455114	35.3808
3.28194	3.392293	3.388477	35.18801
3.194628	3.225493	3.397599	34.65226
3.290147	3.197053	3.333093	34.65788
3.17083	3.337434	3.430952	34.91795
3.307635	3.494529	3.392365	35.47627
3.201185	3.361965	3.47723	35.13918
3.355312	3.282104	3.421148	35.17894
3.295921	3.229941	3.355789	34.79206
3.385832	3.35125	3.368151	35.281
3.293983	3.387845	3.403602	35.23769
3.225024	3.439468	3.343614	35.0686
3.147651	3.358731	3.412485	34.87345
3.227581	3.248744	3.325522	34.61754
3.216899	3.214575	3.374767	34.62715
3.224075	3.286282	3.41078	34.87841
3.21393	3.432122	3.467511	35.29922
3.338237	3.423496	3.442138	35.49671
3.46337	3.4177	3.371272	35.6027
3.403979	3.361089	3.339812	35.28023
3.312749	3.375242	3.350946	35.13602
3.236119	3.408265	3.393933	35.13467
3.308501	3.425046	3.402596	35.34859
3.231004	3.334267	3.342148	34.84841
3.322771	3.204534	3.369328	34.82482
3.080548	3.186405	3.43143	34.39128
3.232324	3.313914	3.385476	34.90154
3.241521	3.47923	3.478424	35.48644
3.321575	3.422148	3.454688	35.48477
3.268206	3.296594	3.480231	35.14935
3.273815	3.263773	3.419698	34.95746
3.270309	3.240792	3.423722	34.90834
3.36154	3.422889	3.355942	35.35784
3.305944	3.418307	3.382168	35.28359
3.31477	3.410489	3.426587	35.38293
3.239913	3.29747	3.340647	34.78414
3.249316	3.228054	3.344757	34.66189
3.14926	3.253731	3.378792	34.57367
3.255462	3.408063	3.417243	35.2275

3.198752	3.469324	3.439887	35.28697
3.323266	3.429898	3.383498	35.34973
3.346898	3.347071	3.353743	35.15521
3.433015	3.334671	3.379729	35.37325
3.32372	3.460158	3.425359	35.50844
3.297819	3.431448	3.342011	35.20675
3.20865	3.319305	3.393166	34.87838
3.196277	3.208375	3.349599	34.51346
3.235995	3.170567	3.391342	34.60892
3.251214	3.333054	3.395417	35.00644
3.234716	3.450319	3.47278	35.39599
3.271464	3.442973	3.399577	35.3002
3.359643	3.347274	3.441456	35.37534
3.286889	3.255551	3.352242	34.82056
3.346156	3.264716	3.396593	35.0672
3.299427	3.40658	3.400259	35.28326
3.298272	3.445331	3.474127	35.52701
3.334237	3.365874	3.342318	35.14366
3.400391	3.262492	3.307976	34.98715
3.137011	3.177172	3.382816	34.38826
3.258143	3.26101	3.394598	34.86226
3.175944	3.315059	3.412605	34.84008
3.24181	3.483207	3.33751	35.18761
3.252946	3.386564	3.380752	35.09518
3.40167	3.39391	3.38169	35.43853
3.253235	3.347004	3.387164	35.02332
3.415899	3.413454	3.368288	35.48308
3.296788	3.413859	3.411565	35.31813
3.352342	3.388586	3.35526	35.26122
3.195659	3.244026	3.409058	34.7201
3.144558	3.154191	3.281223	34.13234
3.287673	3.25582	3.379064	34.88152
3.269649	3.458204	3.336708	35.19206
3.193102	3.485565	3.521667	35.48897
3.225065	3.409478	3.397531	35.12101
3.259215	3.313375	3.493719	35.19588
3.446872	3.324292	3.36058	35.33898
3.32207	3.299896	3.418198	35.1387
3.227375	3.401391	3.421011	35.15973
3.221395	3.396943	3.375023	35.03635
3.301572	3.297133	3.379661	35.00356
3.352961	3.258988	3.352515	34.97316
3.275424	3.201299	3.381503	34.74083
3.1665	3.292415	3.402459	34.74772
3.185265	3.382723	3.527089	35.25879
3.369335	3.478894	3.358739	35.50348
3.309697	3.417296	3.384879	35.29552
3.394699	3.325168	3.357852	35.22083
3.237686	3.374703	3.478697	35.25006
3.340093	3.465078	3.363871	35.42054

3.379976	3.423698	3.399782	35.4958
3.364922	3.286484	3.34387	35.04054
3.136309	3.162413	3.322112	34.2217
3.214919	3.222663	3.377632	34.64678
3.24878	3.411837	3.395775	35.17419
3.270804	3.492979	3.396321	35.40099
3.191617	3.422485	3.412264	35.10853
3.148188	3.294774	3.49834	34.92251
3.208692	3.339726	3.382117	34.89896
3.406908	3.333121	3.430168	35.42306
3.281816	3.369446	3.414191	35.19401
3.29918	3.397549	3.427747	35.32308
3.214177	3.288708	3.388596	34.81356
3.305449	3.18809	3.415487	34.85192
3.350074	3.217878	3.322607	34.81154
3.269196	3.268221	3.350469	34.8057
3.145424	3.412915	3.44947	35.06795
3.234345	3.489205	3.473803	35.48245
3.339475	3.44641	3.457297	35.58267
3.338361	3.394584	3.305521	35.13499
3.307635	3.369177	3.45433	35.33766
3.207166	3.428753	3.356198	35.03363
3.30219	3.399639	3.494265	35.4797
3.35857	3.313577	3.281359	34.9492
3.283713	3.137342	3.429878	34.72489
3.183987	3.188292	3.377223	34.50307
3.137258	3.382184	3.387437	34.84723
3.248079	3.502414	3.444747	35.47783
3.3443	3.424507	3.38007	35.37644
3.253977	3.3435	3.383634	35.00956
3.291756	3.355091	3.412144	35.17988
3.160973	3.358057	3.490019	35.07066
3.315017	3.349565	3.368561	35.12335
3.294684	3.382655	3.495492	35.42883
3.427282	3.390136	3.406739	35.54107
3.265731	3.143542	3.397344	34.62798
3.400721	3.203051	3.29633	34.83241
3.221353	3.251507	3.429878	34.83818
3.292581	3.409545	3.309596	35.07651
3.184812	3.414128	3.44623	35.14965
3.228695	3.446545	3.386976	35.18693
3.267587	3.358057	3.399594	35.10606
3.340794	3.415004	3.357972	35.29967
3.276991	3.389058	3.467374	35.34265
3.395153	3.444455	3.366514	35.50163
3.241686	3.332717	3.448004	35.09987
3.254348	3.159717	3.367879	34.57402
3.331762	3.180137	3.246199	34.52187
3.25641	3.292078	3.444321	35.03515
3.196442	3.470132	3.383106	35.15952

3.254554	3.432729	3.428957	35.30507
3.300582	3.39108	3.367725	35.18074
3.293653	3.355765	3.382117	35.11983
3.298272	3.337839	3.311864	34.9371
3.170005	3.326112	3.528709	35.10516
3.288291	3.370592	3.463436	35.31836
3.240119	3.32564	3.456785	35.10017
3.415651	3.293359	3.397736	35.28431
3.297736	3.197323	3.398026	34.81707
3.324709	3.245307	3.341278	34.85689
3.231499	3.318362	3.414804	34.9736
3.306315	3.425113	3.340852	35.20894
3.193927	3.450116	3.346564	35.03033
3.305284	3.41123	3.400072	35.30583
3.236366	3.34451	3.411786	35.03483
3.249523	3.380297	3.403482	35.1237
3.324874	3.43374	3.386448	35.3681
3.403113	3.428079	3.446316	35.65774
3.251915	3.236748	3.339232	34.67451
3.272536	3.128379	3.39482	34.60418
3.273196	3.186742	3.379269	34.69924
3.361952	3.4208	3.344399	35.32893
3.172356	3.465954	3.41992	35.17821
3.166747	3.343567	3.355994	34.75851
3.275506	3.33366	3.387778	35.04419
3.263092	3.393977	3.41506	35.20861
3.337536	3.383127	3.422	35.36285
3.252987	3.322203	3.475559	35.16185
3.230303	3.280015	3.441456	34.94541
3.311388	3.294774	3.422103	35.11268
3.406536	3.276038	3.35301	35.12869
3.318193	3.206893	3.364741	34.80994
3.29687	3.290798	3.403073	35.03062
3.249481	3.351654	3.400311	35.05404
3.376305	3.429494	3.401368	35.50391
3.307511	3.467504	3.307925	35.23225
3.341289	3.416824	3.426314	35.45419
3.177305	3.444118	3.407592	35.11432
3.245027	3.403817	3.402868	35.16396
3.293447	3.434414	3.427968	35.39165
3.346362	3.329616	3.293448	34.98401
3.242635	3.142734	3.39719	34.57536
3.255709	3.176902	3.399049	34.68274
3.277981	3.32719	3.46526	35.20489
3.356137	3.459147	3.346837	35.4054
3.254431	3.379151	3.389278	35.10086
3.153384	3.365403	3.388733	34.84863
3.236448	3.343297	3.439973	35.09399
3.298932	3.435425	3.423109	35.39522
3.253812	3.39364	3.415401	35.18832

3.222673	3.291	3.416356	34.89786
3.254843	3.319844	3.456478	35.11903
3.286889	3.297133	3.340153	34.88505
3.485105	3.253192	3.390318	35.33213
3.303387	3.247666	3.350009	34.83451
3.264164	3.283317	3.427644	34.99648
3.261854	3.364459	3.360052	35.02106
3.336216	3.444994	3.412008	35.47341
3.316419	3.459282	3.332462	35.28741
3.373129	3.420463	3.379542	35.42949
3.103026	3.383936	3.460145	34.9352
3.268536	3.429022	3.393115	35.24916
3.25872	3.386834	3.417038	35.18775
3.344754	3.313442	3.374699	35.12281
3.210671	3.211475	3.398554	34.65877
3.335226	3.226639	3.408018	34.98501
3.237562	3.419924	3.393763	35.16295
3.366242	3.425046	3.359694	35.38104
3.142702	3.3683	3.38065	34.81393
3.316749	3.39108	3.377086	35.23657
3.08789	3.342219	3.416765	34.71601
3.280455	3.438188	3.398435	35.3069
3.272495	3.378881	3.445276	35.26224
3.306727	3.307174	3.355823	34.98467
3.309985	3.226908	3.449641	35.02143
3.255627	3.238096	3.345439	34.69914
3.341578	3.270445	3.443383	35.17204
3.332339	3.344713	3.260164	34.91357
3.230592	3.319979	3.405733	34.95532
3.258679	3.385486	3.391648	35.12919
3.358653	3.451195	3.406057	35.52302
3.32805	3.466695	3.359268	35.38767
3.293406	3.422889	3.410013	35.32709
3.213393	3.399099	3.450886	35.18947
3.18308	3.324697	3.430407	34.91569
3.246099	3.290596	3.395485	34.90256
3.426539	3.241937	3.350503	35.09238
3.186998	3.211812	3.434107	34.68549
3.319224	3.355361	3.368356	35.14478
3.28792	3.432931	3.44548	35.41461
3.368386	3.417296	3.359285	35.36789
3.257111	3.394584	3.398145	35.15987
3.295633	3.378544	3.379525	35.16831
3.227994	3.461439	3.463282	35.38483
3.281693	3.442299	3.355516	35.22474
3.209269	3.285541	3.497965	35.03507
3.325493	3.248609	3.412196	35.02091
3.301036	3.256225	3.495288	35.16579
3.332752	3.219697	3.36411	34.8684
3.338691	3.252922	3.421062	35.07859

3.335268	3.282778	3.388272	35.06469
3.277197	3.4177	3.369021	35.19065
3.266927	3.438659	3.382935	35.24446
3.225272	3.443647	3.421693	35.24903
3.276042	3.484824	3.367606	35.33182
3.316873	3.413926	3.402237	35.3418
3.256039	3.369783	3.428378	35.1694
3.282765	3.348891	3.410644	35.14338
3.15821	3.246048	3.451619	34.7357
3.366324	3.212688	3.349736	34.89506
3.268907	3.22246	3.387317	34.78558
3.329246	3.368907	3.405256	35.27701
3.313532	3.439333	3.414225	35.41627
3.301902	3.408669	3.377837	35.24421
3.242882	3.345184	3.367742	34.95423
3.335639	3.438525	3.404574	35.44174
3.166953	3.439468	3.489951	35.26162
3.250512	3.35125	3.423245	35.10556
3.197308	3.265323	3.420738	34.79582
3.271382	3.221113	3.443246	34.91035
3.333453	3.283519	3.378331	35.0406
3.324421	3.248205	3.40367	34.99903
3.250801	3.302726	3.393234	34.93445
3.341372	3.354215	3.377513	35.21073
3.290601	3.344308	3.380309	35.08415
3.416517	3.432931	3.367947	35.52628
3.224323	3.431448	3.370675	35.10871
3.263422	3.45234	3.377103	35.25395
3.17912	3.426866	3.404915	35.07471
3.302273	3.409613	3.382816	35.25797
3.258472	3.338715	3.381844	35.00502
3.214383	3.220034	3.403397	34.6962
3.266515	3.214373	3.402578	34.79603
3.337701	3.355765	3.38655	35.22586
3.313244	3.422148	3.368987	35.27913
3.228983	3.308455	3.394871	34.90284
3.284085	3.335075	3.401948	35.09703
3.211826	3.369918	3.401283	35.01375
3.338938	3.416689	3.413491	35.42071
3.248492	3.432931	3.389278	35.20548
3.164273	3.28453	3.465806	34.86413
3.16155	3.236411	3.39262	34.59291
3.346898	3.242342	3.432726	35.09891
3.266762	3.239848	3.406534	34.86093
3.303098	3.289517	3.387096	35.0065
3.243542	3.350037	3.476327	35.20374
3.345991	3.394112	3.388664	35.33247
3.343104	3.403076	3.329666	35.21673
3.395731	3.418644	3.362524	35.43772
3.234593	3.42909	3.381503	35.14968

3.255627	3.436233	3.36631	35.17808
3.096468	3.356237	3.431788	34.79828
3.352384	3.347543	3.358739	35.17917
3.278022	3.314992	3.451704	35.14866
3.186173	3.23513	3.455029	34.78043
3.22886	3.292685	3.424677	34.93327
3.370407	3.419857	3.403482	35.47456
3.344135	3.39108	3.410013	35.36846
3.282435	3.354485	3.357238	35.0381
3.191081	3.317283	3.437108	34.93163
3.215043	3.44176	3.389483	35.15209
3.327102	3.418778	3.369106	35.30233
3.262143	3.367761	3.400805	35.11803
3.167613	3.288102	3.496584	34.94656
3.198422	3.194357	3.381162	34.55652
3.311718	3.266266	3.414873	35.03525
3.342856	3.270647	3.329342	34.92588
3.32306	3.348285	3.396576	35.1994
3.219456	3.327123	3.432316	35.00472
3.337247	3.409882	3.405665	35.38501
3.284538	3.445938	3.362405	35.25399
3.420765	3.406445	3.338396	35.41303
3.223746	3.400447	3.377103	35.05371
3.207166	3.383262	3.396917	35.0232
3.195081	3.273949	3.404556	34.77443
3.324462	3.298548	3.324192	34.93541
3.211043	3.227785	3.4146	34.73034
3.320544	3.29902	3.389705	35.07114
3.235005	3.3497	3.499346	35.23468
3.425013	3.444658	3.366855	35.56812
3.462834	3.342152	3.366446	35.42577
3.309985	3.360213	3.394053	35.19138
3.232654	3.388721	3.442616	35.19081
3.255998	3.39883	3.394598	35.15896
3.191039	3.416824	3.43334	35.14098
3.30681	3.311555	3.351304	34.98454
3.162334	3.174072	3.43264	34.54581
3.130329	3.157763	3.481033	34.54598
3.276166	3.250564	3.45907	35.01982
3.326483	3.352935	3.4146	35.25647
3.318647	3.424642	3.431583	35.43329
3.299798	3.382521	3.372585	35.17094
3.326524	3.318699	3.389517	35.12684
3.321327	3.405434	3.399577	35.32716
3.383069	3.458204	3.342847	35.45351
3.268536	3.350845	3.363991	35.01451
3.310357	3.325708	3.356011	35.03354
3.179533	3.271995	3.441951	34.81793
3.300664	3.287698	3.411496	35.05056
3.241315	3.308522	3.406483	34.95535

3.346568	3.343634	3.44473	35.34595
3.213063	3.372075	3.46364	35.15754
3.307965	3.374164	3.402289	35.23548
3.316914	3.363987	3.332275	35.07969
3.419693	3.451666	3.439137	35.72988
3.24082	3.419857	3.421608	35.23082
3.232283	3.370592	3.427542	35.11739
3.196814	3.30522	3.375432	34.78291
3.238882	3.209117	3.344058	34.59613
3.14229	3.227717	3.403806	34.55624
3.247337	3.280082	3.457843	35.01864
3.181677	3.32126	3.501341	35.06023
3.376511	3.423631	3.430475	35.55519
3.411733	3.422957	3.377905	35.51578
3.413713	3.387642	3.384862	35.4581
3.355106	3.382453	3.389483	35.32869
3.302067	3.425046	3.3629	35.24771
3.24944	3.378949	3.37874	35.06646
3.353291	3.373557	3.272594	35.04965
3.222096	3.266671	3.419357	34.84995
3.111646	3.257842	3.385322	34.51468
3.277279	3.305287	3.443997	35.10896
3.278723	3.363583	3.352924	35.04044
3.266556	3.398762	3.45121	35.3057
3.248657	3.372883	3.420534	35.14288
3.22189	3.356035	3.491946	35.20367
3.314399	3.407456	3.326597	35.15683
3.462504	3.439199	3.389227	35.68709
3.253729	3.389799	3.410627	35.1693
3.201309	3.388451	3.415964	35.06339
3.145383	3.251103	3.439546	34.6923
3.289281	3.228189	3.331934	34.72154
3.220446	3.215789	3.411718	34.71837
3.255668	3.281969	3.388664	34.88971
3.218673	3.337434	3.475355	35.11967
3.320131	3.44021	3.392501	35.38511
3.339846	3.412376	3.436068	35.46263
3.488858	3.397212	3.37857	35.6296
3.282765	3.368031	3.460912	35.29516
3.356714	3.431583	3.332735	35.31555
3.289652	3.316205	3.415418	35.0974
3.362777	3.333391	3.302588	35.04815
3.256204	3.254675	3.380224	34.81273
3.133381	3.346734	3.403636	34.79666
3.159076	3.334267	3.427065	34.87682
3.351724	3.397684	3.418812	35.41874
3.284703	3.350037	3.435454	35.20438
3.348259	3.337569	3.427218	35.29809
3.295757	3.362168	3.427423	35.23751
3.332009	3.453082	3.408939	35.47518

3.388472	3.444523	3.390472	35.53956
3.28792	3.341815	3.461372	35.25011
3.219044	3.328336	3.355584	34.83867
3.219085	3.232704	3.414787	34.7591
3.298437	3.246183	3.370658	34.8656
3.208362	3.294976	3.442411	34.93223
3.269155	3.329751	3.435744	35.12665
3.204897	3.3621	3.487154	35.16929
3.312749	3.369918	3.34919	35.12054
3.342939	3.337636	3.413355	35.25628
3.440975	3.39701	3.40974	35.59261
3.299798	3.467706	3.419494	35.45981
3.293694	3.4146	3.371084	35.22446
3.294932	3.288574	3.375995	34.96231
3.341001	3.246318	3.268724	34.73606
3.228406	3.247127	3.371647	34.71668
3.217559	3.347543	3.39598	34.96576
3.194628	3.409882	3.435181	35.13767
3.299551	3.375646	3.385595	35.18382
3.334154	3.330021	3.435727	35.26934
3.373624	3.359741	3.407626	35.3592
3.322895	3.431853	3.433612	35.46278
3.36917	3.450319	3.414174	35.56185
3.279259	3.292483	3.385732	34.95787
3.29753	3.289719	3.382458	34.98463
3.196525	3.264986	3.349378	34.63732
3.119853	3.244566	3.409894	34.55733
3.21892	3.261953	3.419391	34.83277
3.189307	3.327595	3.437517	34.95119
3.28924	3.363785	3.408649	35.18574
3.311924	3.414061	3.42495	35.38094
3.262308	3.352665	3.459889	35.21458
3.272042	3.358596	3.359557	35.02943
3.424106	3.421744	3.364025	35.50983
3.308212	3.454497	3.385936	35.37594
3.36785	3.344915	3.324414	35.13218
3.293282	3.2354	3.365747	34.82001
3.278599	3.176026	3.343512	34.60943
3.195205	3.342489	3.325386	34.75145
3.308171	3.404154	3.374188	35.24006
3.153467	3.407726	3.43172	35.03537
3.258926	3.359	3.400396	35.09094
3.142455	3.325977	3.442002	34.85501
3.391153	3.469728	3.432197	35.69179
3.313038	3.472761	3.415128	35.49027
3.39672	3.382386	3.355738	35.34575
3.213393	3.26074	3.466215	34.92042
3.317945	3.291067	3.343648	34.94735
3.216074	3.25609	3.380207	34.72803
3.245481	3.247531	3.324636	34.6521

3.121751	3.259999	3.439614	34.66022
3.206093	3.353946	3.375978	34.91095
3.356178	3.391214	3.390915	35.35333
3.377625	3.40968	3.406125	35.47387
3.377047	3.369379	3.43027	35.43728
3.291426	3.388653	3.399509	35.22492
3.287838	3.448701	3.43143	35.41819
3.413341	3.355226	3.286185	35.17061
3.447986	3.219428	3.347076	35.08256
3.193225	3.153112	3.347331	34.38098
3.258761	3.287158	3.384384	34.89846
3.262886	3.437042	3.378075	35.22145
3.321121	3.433538	3.363701	35.30971
3.260617	3.31634	3.435317	35.07771
3.191864	3.275162	3.439171	34.84574
3.187905	3.403615	3.469352	35.18399
3.466669	3.519734	3.368134	35.82619
3.260081	3.464539	3.476804	35.49135
3.365293	3.269434	3.379405	35.08178
3.198999	3.235265	3.455933	34.81075
3.340629	3.279408	3.369106	35.02713
3.272908	3.273208	3.335208	34.79135
3.321121	3.318699	3.417601	35.17644
3.035139	3.317957	3.444679	34.60864
3.309491	3.327729	3.352788	35.02902
3.245769	3.397482	3.423057	35.19588
3.35523	3.397482	3.395485	35.37495
3.209475	3.384273	3.414088	35.06801
3.33568	3.417026	3.45346	35.50173
3.284126	3.399773	3.336623	35.09575
3.43982	3.245779	3.320049	35.06322
3.219704	3.19739	3.362951	34.56986
3.282311	3.205545	3.340033	34.67449
3.169799	3.386295	3.36527	34.87891
3.269567	3.465145	3.352106	35.24073
3.248657	3.393304	3.398009	35.13828
3.250348	3.283115	3.395639	34.89583
3.278311	3.394854	3.492202	35.4125
3.239624	3.469324	3.406807	35.30401
3.311058	3.406311	3.431379	35.37616
3.327679	3.31971	3.425087	35.20936
3.309284	3.207701	3.384708	34.83589
3.177718	3.242679	3.427474	34.71819
3.293364	3.33238	3.425087	35.16203
3.212404	3.300098	3.382543	34.82135
3.249853	3.259123	3.407677	34.8686
3.296128	3.357383	3.422222	35.21649
3.208857	3.425585	3.472371	35.28445
3.227292	3.420261	3.409467	35.17557
3.311223	3.364661	3.438131	35.3002

3.24082	3.34788	3.354288	34.9262
3.38942	3.403884	3.45271	35.58887
3.37548	3.342017	3.442957	35.40176
3.369335	3.238433	3.31536	34.88276
3.263669	3.199816	3.396508	34.7447
3.377377	3.40685	3.360137	35.36657
3.214589	3.46218	3.424626	35.27261
3.244367	3.403076	3.339437	35.02218
3.146084	3.277858	3.433408	34.73892
3.32009	3.373625	3.345217	35.13601
3.323183	3.551207	3.490309	35.84841
3.232613	3.484824	3.448635	35.41404
3.202546	3.298952	3.418419	34.87575
3.349579	3.223134	3.41298	35.01959
3.330483	3.240185	3.39274	34.97085
3.347558	3.307646	3.369652	35.10523
3.206959	3.304411	3.449573	34.96546
3.142042	3.249688	3.333417	34.44981
3.257318	3.339119	3.335208	34.90139
3.338897	3.40166	3.374085	35.30158
3.287343	3.456384	3.386431	35.33551
3.194133	3.358394	3.448021	35.05207
3.248698	3.391956	3.392007	35.12229
3.358034	3.450588	3.447663	35.61133
3.38542	3.385216	3.314388	35.23681
3.266432	3.231626	3.447049	34.93083
3.34463	3.211947	3.38227	34.91714
3.205351	3.337232	3.421148	34.97156
3.427076	3.440344	3.351969	35.53064
3.261689	3.363516	3.376984	35.05566
3.125916	3.30367	3.40928	34.6985
3.175408	3.301244	3.371442	34.71868
3.34232	3.472222	3.401896	35.52419
3.255627	3.507536	3.413935	35.43816
3.326937	3.346128	3.390506	35.18989
3.193019	3.204669	3.452454	34.72316
3.331308	3.204736	3.320202	34.73651
3.504448	3.359472	3.386158	35.59775
3.386203	3.313172	3.383839	35.23285
3.236201	3.291404	3.421693	34.94
3.20399	3.347408	3.394172	34.93184
3.279507	3.349161	3.341176	34.98492
3.422621	3.378342	3.343495	35.36678
3.19038	3.392562	3.460861	35.14666
3.10315	3.443175	3.484136	35.11749
3.247378	3.413387	3.437654	35.2661
3.41198	3.366211	3.381042	35.39909
3.414455	3.272399	3.36145	35.15651
3.249935	3.208038	3.443656	34.83575
3.183863	3.310409	3.424694	34.87367

3.276496	3.449038	3.389415	35.30225
3.456441	3.436166	3.326256	35.52949
3.266309	3.322944	3.411241	35.05195
3.158705	3.283856	3.394667	34.69491
3.18044	3.421474	3.435045	35.1317
3.340341	3.537189	3.407575	35.67435
3.299798	3.35927	3.357903	35.08799
3.266597	3.17023	3.413781	34.72418
3.158045	3.163693	3.387795	34.41567
3.371644	3.3065	3.439836	35.30888
3.328957	3.405906	3.345848	35.22738
3.401587	3.332919	3.410081	35.36706
3.207001	3.267951	3.366446	34.70404
3.23682	3.296594	3.370863	34.84154
3.214342	3.369581	3.348917	34.904
3.319183	3.420396	3.389091	35.33225
3.097788	3.414196	3.453546	34.97549
3.262102	3.366885	3.419101	35.15603
3.200154	3.344241	3.458866	35.058
3.441923	3.295717	3.408461	35.37037
3.419033	3.260605	3.417788	35.26393
3.262473	3.377601	3.387863	35.11197
3.302685	3.400649	3.446657	35.37888
3.351971	3.44122	3.329871	35.31999
3.366571	3.333391	3.365696	35.19446
3.347517	3.289854	3.285451	34.8821
3.120307	3.39391	3.447492	34.96714
3.194793	3.538065	3.403943	35.35003
3.312708	3.454969	3.487086	35.608
3.260246	3.204129	3.388818	34.72983
3.296375	3.135253	3.398077	34.67846
3.255091	3.231963	3.433885	34.87798
3.306274	3.424102	3.406262	35.34968
3.302727	3.319373	3.403943	35.10782
3.315636	3.307714	3.329052	34.94678
3.22721	3.312296	3.424694	34.97258
3.272248	3.401391	3.322675	35.04281
3.215497	3.376455	3.392689	35.01728
3.320503	3.428079	3.340408	35.24548
3.174625	3.345656	3.454978	34.99677
3.298973	3.373894	3.389841	35.18801
3.251214	3.273478	3.410371	34.90886
3.36488	3.232637	3.309562	34.84767
3.29918	3.23257	3.455541	35.02308
3.424642	3.404423	3.410422	35.57459
3.256658	3.415139	3.440365	35.29615
3.345661	3.404356	3.319691	35.20331
3.308831	3.324225	3.347672	35.00873
3.288828	3.378342	3.397804	35.19296
3.213888	3.510636	3.365849	35.24851

3.185389	3.492911	3.466027	35.36649
3.173635	3.342691	3.381349	34.8271
3.272165	3.175217	3.443246	34.8117
3.293859	3.216462	3.324295	34.68921
3.292952	3.313914	3.441746	35.15718
3.326937	3.391686	3.487785	35.50225
3.225107	3.266536	3.406074	34.82719
3.396473	3.298548	3.338004	35.12309
3.328751	3.331975	3.315496	34.99888
3.314728	3.387103	3.382338	35.23494
3.217889	3.449173	3.371476	35.13515
3.238098	3.394651	3.415606	35.15662
3.232159	3.424979	3.362951	35.0948
3.314275	3.324495	3.375501	35.08208
3.245893	3.300839	3.457433	35.05998
3.224777	3.225021	3.437244	34.80385
3.246718	3.332851	3.400805	35.00795
3.381089	3.442299	3.454705	35.65902
3.303881	3.423698	3.376268	35.27797
3.321245	3.333727	3.426996	35.23012
3.226509	3.316946	3.364451	34.84948
3.28425	3.502279	3.298223	35.23621
3.20865	3.530248	3.380616	35.31223
3.265154	3.444253	3.399526	35.28909
3.191246	3.218754	3.433118	34.7078
3.243996	3.211273	3.313245	34.54465
3.31778	3.276308	3.446861	35.14042
3.406619	3.468919	3.424763	35.70758
3.282022	3.293493	3.48296	35.17875
3.294024	3.271321	3.33739	34.83817
3.286972	3.200221	3.427883	34.86516
3.408104	3.348958	3.290942	35.15585
3.236985	3.427607	3.429094	35.25575
3.091643	3.508479	3.405818	35.06386
3.168397	3.374029	3.537627	35.22593
3.230633	3.276915	3.29882	34.62743
3.361375	3.250227	3.338823	34.94246
3.271712	3.242679	3.393047	34.84845
3.22556	3.422148	3.412468	35.18247
3.236284	3.428483	3.412417	35.21966
3.431984	3.42754	3.329922	35.46516
3.340134	3.357046	3.378723	35.21686
3.218714	3.332245	3.408649	34.96254
3.211826	3.342556	3.3356	34.81028
3.340217	3.503155	3.377479	35.53384
3.264288	3.393506	3.384009	35.14229
3.259669	3.217204	3.408751	34.80075
3.093788	3.16868	3.444935	34.41101
3.281734	3.296189	3.408529	35.02125
3.307882	3.468245	3.484238	35.62025

3.379934	3.440277	3.35879	35.44232
3.284662	3.213834	3.384726	34.7955
3.326648	3.272062	3.349991	34.93869
3.294932	3.349228	3.441081	35.23728
3.347888	3.419452	3.33086	35.26562
3.214301	3.41743	3.420397	35.16487
3.271505	3.343567	3.425376	35.13933
3.213105	3.239646	3.387505	34.70154
3.29056	3.283452	3.379116	34.94837
3.261978	3.302794	3.406415	34.98786
3.291014	3.439333	3.394257	35.32336
3.255255	3.429831	3.467374	35.38428
3.322482	3.355833	3.341807	35.09488
3.343805	3.370524	3.411531	35.32611
3.401463	3.411702	3.295154	35.28775
3.277073	3.404828	3.369106	35.16242
3.190297	3.474378	3.460827	35.32532
3.20234	3.39856	3.382065	35.01362
3.186998	3.194155	3.400447	34.57327
3.21129	3.136399	3.376166	34.44699
3.233768	3.311757	3.431651	35.00096
3.184358	3.415476	3.395673	35.04105
3.288209	3.372614	3.440228	35.27185
3.399525	3.311218	3.43513	35.36987
3.264412	3.321529	3.370419	34.95544
3.359024	3.394112	3.392433	35.36921
3.313244	3.425113	3.424677	35.4074
3.320503	3.339523	3.368492	35.11324
3.195824	3.326853	3.297251	34.65708
3.272289	3.285541	3.397958	34.95419
3.121792	3.310814	3.348798	34.57284
3.2826	3.335143	3.351748	34.98415
3.20102	3.242679	3.41448	34.74073
3.232365	3.346195	3.358926	34.91417
3.336299	3.357113	3.4279	35.31616
3.325658	3.408332	3.374205	35.28748
3.334897	3.415948	3.471347	35.53677
3.367685	3.444253	3.362405	35.43213
3.252533	3.391956	3.392808	35.13243
3.269979	3.393101	3.358671	35.09844
3.212898	3.35408	3.382577	34.94056
3.225107	3.255079	3.377751	34.7402
3.228653	3.231087	3.424814	34.79841
3.231004	3.257168	3.406603	34.82076
3.231705	3.389732	3.478987	35.27048
3.258514	3.392023	3.433391	35.23441
3.388967	3.304209	3.419784	35.2979
3.409382	3.379083	3.359881	35.37528
3.326854	3.313577	3.362507	35.0573
3.270887	3.295111	3.403687	34.98458

3.306191	3.389866	3.460281	35.39276
3.218673	3.448095	3.370044	35.13137
3.39272	3.335075	3.296535	35.10408
3.141919	3.235198	3.418095	34.60303
3.197226	3.280823	3.395843	34.7751
3.172604	3.31142	3.45832	34.92479
3.25641	3.390743	3.352993	35.05119
3.283466	3.400447	3.371357	35.17174
3.419033	3.377601	3.427764	35.54159
3.234634	3.353069	3.410763	35.04752
3.337206	3.395123	3.364843	35.26337
3.285899	3.453621	3.355431	35.25852
3.298437	3.425585	3.376234	35.27012
3.206341	3.270714	3.398077	34.77781
3.16353	3.209588	3.43588	34.63318
3.262432	3.283587	3.419784	34.97609
3.318399	3.455508	3.377496	35.38197
3.241274	3.445331	3.478969	35.41296
3.28693	3.393708	3.425922	35.2839
3.264865	3.231491	3.441899	34.91585
3.35424	3.31607	3.357307	35.11127
3.316048	3.383464	3.394462	35.25638
3.270103	3.410556	3.380718	35.1851
3.243295	3.429292	3.462873	35.3471
3.266309	3.223471	3.339317	34.67714
3.33535	3.165985	3.340732	34.7055
3.269361	3.266469	3.370607	34.84626
3.153137	3.38306	3.463913	35.05111
3.151982	3.485633	3.436443	35.21282
3.420188	3.497359	3.372977	35.6862
3.280579	3.354215	3.377632	35.07805
3.374655	3.324629	3.375893	35.21527
3.233149	3.381442	3.451022	35.19436
3.333412	3.45753	3.372841	35.40904
3.322317	3.347206	3.343307	35.07893
3.381502	3.263436	3.375211	35.09493
3.090199	3.179868	3.424677	34.38333
3.219662	3.298076	3.405989	34.88408
3.168686	3.435425	3.43039	35.12632
3.347063	3.487655	3.397122	35.55787
3.185595	3.386362	3.527669	35.26874
3.267546	3.286821	3.459616	35.08145
3.243377	3.273343	3.401965	34.87305
3.415404	3.406445	3.367589	35.46514
3.290312	3.413185	3.418675	35.31804
3.276661	3.38744	3.38123	35.15
3.208939	3.308994	3.385987	34.84076
3.221188	3.181687	3.36764	34.54903
3.346115	3.20669	3.293858	34.71555
3.260741	3.305624	3.413099	35.00596

3.199206	3.433672	3.413219	35.15168
3.189513	3.486576	3.436068	35.29614
3.368799	3.444321	3.360359	35.43024
3.399195	3.382655	3.318906	35.27121
3.329741	3.378073	3.35729	35.19324
3.206052	3.450925	3.44045	35.26393
3.303304	3.418981	3.435931	35.39687
3.23748	3.304546	3.361979	34.84095
3.325452	3.147451	3.345524	34.6538
3.179615	3.164974	3.383685	34.45665
3.174707	3.385284	3.393234	34.94858
3.219209	3.452475	3.524327	35.47952
3.327184	3.41305	3.382134	35.31847
3.257483	3.299491	3.474468	35.11963
3.322276	3.304142	3.391461	35.08997
3.256575	3.365268	3.429929	35.16409
3.320131	3.432864	3.363292	35.30517
3.343929	3.4115	3.353879	35.28991
3.376882	3.372344	3.391665	35.35898
3.286477	3.202377	3.342011	34.681
3.306851	3.192403	3.30726	34.62775
3.141217	3.260201	3.379388	34.57153
3.260205	3.405839	3.398367	35.19173
3.21261	3.459484	3.457161	35.33353
3.22688	3.444253	3.462736	35.34362
3.308913	3.364931	3.423808	35.26442
3.421219	3.372681	3.381366	35.43415
3.335845	3.469391	3.433357	35.57264
3.305572	3.486981	3.386703	35.44288
3.261277	3.303131	3.408052	34.99065
3.190297	3.216799	3.341909	34.50199
3.278599	3.140847	3.304498	34.44718
3.2866	3.333593	3.40089	35.09698
3.239789	3.529506	3.452915	35.53681
3.185018	3.446747	3.418385	35.16054
3.260411	3.346937	3.46601	35.21129
3.237727	3.304816	3.396167	34.91684
3.299262	3.234726	3.462549	35.0433
3.193597	3.407658	3.397207	35.04751
3.266515	3.4826	3.491588	35.57725
3.283878	3.41123	3.419408	35.3013
3.431612	3.222663	3.304856	34.9615
3.317574	3.163019	3.371971	34.72845
3.151198	3.276308	3.450749	34.78464
3.129587	3.411432	3.432504	34.99297
3.346115	3.487318	3.28494	35.30973
3.240532	3.412241	3.400737	35.16789
3.368716	3.403884	3.331934	35.27947
3.207496	3.326853	3.504564	35.13597
3.314192	3.492979	3.304498	35.29507

3.354322	3.446073	3.417669	35.52774
3.421549	3.375512	3.355397	35.38427
3.235376	3.136534	3.430219	34.61816
3.246966	3.18782	3.338737	34.5556
3.252245	3.310477	3.403602	34.97723
3.384017	3.529034	3.328524	35.57916
3.260452	3.463393	3.41801	35.36109
3.095479	3.307444	3.462071	34.75564
3.176604	3.291876	3.413372	34.7925
3.312914	3.307309	3.468193	35.24422
3.296746	3.390203	3.406279	35.25475
3.213105	3.382588	3.429349	35.10564
3.21727	3.326247	3.462873	35.06485
3.275424	3.249283	3.335532	34.74524
3.445181	3.190651	3.26134	34.826
3.253152	3.25272	3.433987	34.91936
3.102614	3.40038	3.357767	34.74638
3.231169	3.44614	3.416714	35.25649
3.356632	3.444792	3.352941	35.38844
3.400474	3.37477	3.323272	35.26631
3.314852	3.361696	3.324278	35.05268
3.154333	3.369783	3.45184	34.99829
3.276743	3.488666	3.443656	35.50806
3.324792	3.368368	3.348235	35.1414
3.397215	3.219158	3.411991	35.1129
3.189926	3.114698	3.35688	34.31064
3.303139	3.229941	3.423262	34.9554
3.294973	3.436638	3.354442	35.23906
3.349703	3.475119	3.395144	35.5319
3.119647	3.358798	3.391205	34.76582
3.243089	3.359539	3.437142	35.13784
3.173099	3.355833	3.448157	35.00077
3.276496	3.316812	3.348235	34.92304
3.318605	3.361359	3.490786	35.42428
3.345414	3.4177	3.385254	35.37533
3.24247	3.295583	3.429725	34.98041
3.344424	3.229672	3.332906	34.8475
3.329246	3.186809	3.400976	34.86943
3.366943	3.312431	3.292766	34.98995
3.108223	3.363718	3.437637	34.85313
3.224364	3.385553	3.367214	35.00086
3.360096	3.398965	3.403823	35.40707
3.292787	3.397549	3.33589	35.10822
3.274269	3.437042	3.475849	35.46016
3.273361	3.458069	3.414259	35.36947
3.256039	3.418981	3.44311	35.3092
3.310728	3.288439	3.322879	34.8804
3.356508	3.219091	3.41344	35.0269
3.21327	3.206893	3.410644	34.68087
3.29621	3.359068	3.368748	35.10341

3.284126	3.404558	3.447475	35.34863
3.36187	3.364392	3.344467	35.20554
3.25109	3.322203	3.443178	35.08689
3.279383	3.351856	3.369038	35.05148
3.14959	3.340399	3.485466	34.9972
3.243171	3.353474	3.353606	34.94208
3.185719	3.383194	3.473257	35.14309
3.39771	3.399099	3.36058	35.39506
3.259751	3.234457	3.458576	34.94762
3.362983	3.226571	3.332053	34.87944
3.278187	3.211341	3.435266	34.88641
3.280249	3.348958	3.383617	35.07892
3.182956	3.423361	3.376541	35.01338
3.283548	3.406243	3.345609	35.12829
3.180935	3.352328	3.382475	34.8666
3.243006	3.380836	3.39731	35.09713
3.319554	3.461236	3.372704	35.38654
3.339516	3.388923	3.42171	35.37922
3.33003	3.368974	3.377768	35.21876
3.207166	3.184855	3.414327	34.62739
3.334938	3.207297	3.365201	34.84845
3.320956	3.358731	3.363241	35.14475
3.312419	3.464336	3.400669	35.43887
3.236572	3.451195	3.466556	35.38835
3.285982	3.369244	3.406671	35.18623
3.213228	3.356439	3.358534	34.89386
3.287054	3.326921	3.426826	35.1401
3.204897	3.356507	3.461099	35.10008
3.144393	3.378881	3.459752	35.01375
3.222921	3.362707	3.366242	34.94562
3.428808	3.238837	3.37695	35.14839
3.248615	3.249688	3.363019	34.74761
3.301861	3.282239	3.366583	34.94302
3.229437	3.30704	3.427542	34.97219
3.363932	3.469526	3.391342	35.54247
3.319966	3.462382	3.336828	35.31149
3.392514	3.392899	3.397685	35.45128
3.256204	3.401863	3.348423	35.06506
3.246058	3.413859	3.387334	35.1542
3.207991	3.417228	3.430986	35.17378
3.364592	3.354283	3.359489	35.22224
3.307965	3.20851	3.454569	34.98755
3.164149	3.186877	3.414992	34.53919
3.306439	3.288237	3.449726	35.14797
3.372964	3.458945	3.356352	35.46257
3.319966	3.440277	3.419084	35.44303
3.242965	3.371603	3.386721	35.05369
3.136639	3.289248	3.436954	34.75093
3.276001	3.413993	3.362678	35.16606
3.301696	3.363516	3.42212	35.24185

3.24412	3.337165	3.43397	35.08423
3.191246	3.320249	3.517318	35.11388
3.16287	3.229806	3.337936	34.46177
3.392926	3.215384	3.364673	34.99179
3.308501	3.289652	3.349991	34.93747
3.237273	3.350306	3.431907	35.09349
3.227375	3.43994	3.37354	35.14021
3.410248	3.456114	3.38846	35.60813
3.320874	3.41925	3.317866	35.17769
3.366943	3.383599	3.337731	35.24391
3.111275	3.408871	3.50291	35.10129
3.16984	3.450723	3.345217	34.97604
3.29155	3.376051	3.449931	35.30789
3.269031	3.269973	3.372159	34.8566
3.250018	3.162211	3.374972	34.58551
3.226756	3.243218	3.404096	34.77548
3.248986	3.447353	3.512663	35.50793
3.401876	3.484487	3.346036	35.55909
3.335556	3.33083	3.383839	35.16071
3.343723	3.311622	3.381179	35.13074
3.225766	3.371805	3.483949	35.22914
3.261401	3.443512	3.370999	35.21688
3.256122	3.389327	3.404846	35.16086
3.271835	3.319642	3.347621	34.91769
3.23187	3.254809	3.490582	35.00115
3.261854	3.233446	3.4257	34.87811
3.32141	3.254472	3.372517	34.93803
3.370613	3.245442	3.371476	35.02361
3.309985	3.324764	3.370112	35.0615
3.228612	3.391686	3.441405	35.18581
3.380347	3.406984	3.337731	35.32436
3.273733	3.425787	3.378433	35.22134
3.328586	3.492237	3.331405	35.38377
3.232365	3.425585	3.429793	35.24275
3.267175	3.415746	3.419647	35.27517
3.193762	3.273006	3.383344	34.72309
3.379604	3.204129	3.402732	35.02128
3.192236	3.206623	3.397378	34.60527
3.316997	3.368907	3.37666	35.18769
3.32174	3.441827	3.449146	35.51604
3.347063	3.367155	3.368339	35.23141
3.337	3.334267	3.371084	35.14349
3.330318	3.328808	3.324227	35.01447
3.197721	3.416554	3.456905	35.20653
3.250719	3.443579	3.412673	35.2848
3.169057	3.329414	3.431498	34.89773
3.263009	3.315936	3.386482	34.97527
3.267752	3.240657	3.38343	34.81434
3.283878	3.215115	3.444133	34.9265
3.267257	3.318092	3.400959	35.02093

3.263463	3.347206	3.466573	35.21979
3.29254	3.372816	3.344603	35.07265
3.327885	3.396741	3.360035	35.23601
3.340959	3.381847	3.410507	35.34241
3.250677	3.383868	3.373062	35.06751
3.324916	3.460495	3.328916	35.30089
3.265401	3.423765	3.377206	35.19602
3.299922	3.402806	3.428429	35.33769
3.217188	3.272804	3.403533	34.81803
3.285734	3.185259	3.372704	34.70906
3.345455	3.293493	3.329274	34.98138
3.320874	3.417093	3.382748	35.31486
3.261978	3.413454	3.447322	35.31932
3.294024	3.365403	3.371835	35.11924
3.254389	3.326112	3.356761	34.91368
3.32673	3.408332	3.384026	35.3113
3.202959	3.391686	3.405921	35.05211
3.176068	3.412578	3.426945	35.08497
3.116678	3.32874	3.37475	34.65761
3.247625	3.266199	3.419357	34.90475
3.185637	3.250968	3.414174	34.72455
3.341949	3.217271	3.396065	34.95309
3.178996	3.309129	3.410132	34.82838
3.405217	3.330493	3.381759	35.30776
3.449182	3.413387	3.360291	35.53823
3.415569	3.44796	3.349241	35.51617
3.305902	3.405434	3.407506	35.31076
3.239624	3.418846	3.339778	35.04704
3.22721	3.378073	3.419153	35.10431
3.352054	3.376118	3.290942	35.09267
3.284992	3.268423	3.451073	35.06069
3.201969	3.23513	3.369226	34.62734
3.255049	3.220034	3.394803	34.76634
3.323802	3.355698	3.395229	35.21429
3.311511	3.457665	3.391069	35.4013
3.324297	3.381442	3.405375	35.29386
3.247131	3.355226	3.454552	35.17532
3.288291	3.347004	3.33635	34.98887
3.414661	3.428213	3.443929	35.67807
3.241109	3.421541	3.402186	35.19266
3.190833	3.315127	3.386243	34.81514
3.058937	3.219697	3.423177	34.39878
3.324544	3.268153	3.38817	35.00903
3.251296	3.295178	3.439273	35.01971
3.2866	3.337097	3.390335	35.08156
3.22556	3.372007	3.458388	35.17324
3.313038	3.37477	3.366838	35.17037
3.427612	3.421272	3.37313	35.53638
3.497726	3.443781	3.296586	35.57154
3.297984	3.374096	3.455848	35.33063

3.216074	3.408871	3.358858	35.01545
3.209228	3.363246	3.427815	35.0515
3.32739	3.39418	3.24758	34.98341
3.265979	3.236007	3.39378	34.82293
3.146208	3.270377	3.460298	34.78164
3.14064	3.291202	3.492815	34.88611
3.419487	3.434414	3.413014	35.63457
3.387894	3.368772	3.438728	35.47817
3.38146	3.342623	3.415538	35.3562
3.275918	3.341478	3.413355	35.11812
3.26037	3.377938	3.427423	35.19461
3.351848	3.435627	3.308931	35.2617
3.309656	3.3683	3.399373	35.21998
3.183409	3.26539	3.408052	34.73783
3.134948	3.199412	3.403056	34.47664
3.33535	3.226571	3.336043	34.82774
3.235376	3.29383	3.441933	34.98776
3.264865	3.379757	3.4412	35.23855
3.163901	3.344982	3.479396	35.02524
3.287962	3.385553	3.379678	35.1672
3.388101	3.449712	3.381707	35.53093
3.442294	3.429224	3.384947	35.61172
3.315883	3.421339	3.40072	35.35253
3.18807	3.336895	3.34815	34.77339
3.192112	3.282576	3.415879	34.81156
3.361705	3.322473	3.335617	35.09416
3.222467	3.281026	3.435454	34.91736
3.249811	3.291269	3.412213	34.94873
3.280125	3.359944	3.452693	35.25373
3.376099	3.409882	3.373795	35.40028
3.371356	3.344241	3.411087	35.32791
3.391235	3.394719	3.440433	35.54594
3.260535	3.350172	3.432726	35.14585
3.281528	3.423294	3.415606	35.31423
3.242181	3.33831	3.40014	35.00852
3.329081	3.267682	3.393234	35.029
3.252822	3.223808	3.40633	34.79493
3.159035	3.310072	3.436682	34.84485
3.150703	3.292078	3.436204	34.78623
3.319554	3.370187	3.415469	35.28095
3.316131	3.388586	3.445276	35.37888
3.259297	3.3497	3.352396	34.96644
3.265484	3.406917	3.437926	35.29214
3.360426	3.464067	3.37134	35.47913
3.404845	3.426124	3.329257	35.40126
3.384842	3.358798	3.335753	35.22449
3.142826	3.235063	3.356505	34.47004
3.231334	3.265929	3.357972	34.7343
3.292333	3.306298	3.385732	35.01668
3.231664	3.352935	3.379764	34.97294

3.311965	3.446477	3.397037	35.39088
3.192895	3.413387	3.427269	35.12424
3.312543	3.409545	3.446111	35.4187
3.274434	3.411298	3.428395	35.30045
3.424683	3.4084	3.420363	35.60512
3.273774	3.380297	3.417465	35.20731
3.378491	3.314318	3.395758	35.24455
3.253812	3.210936	3.401163	34.75764
3.340382	3.23695	3.315155	34.81576
3.173222	3.231896	3.399441	34.62347
3.22952	3.315194	3.372977	34.87088
3.184111	3.359135	3.499926	35.14528
3.255049	3.452812	3.421199	35.33311
3.313574	3.364594	3.4508	35.3329
3.443037	3.447151	3.357102	35.59166
3.289817	3.447286	3.435062	35.42737
3.378161	3.446005	3.369157	35.47364
3.292869	3.367424	3.347195	35.06725
3.305985	3.232098	3.37325	34.85697
3.244656	3.240252	3.361416	34.71481
3.200897	3.292078	3.347962	34.70303
3.163241	3.358731	3.369021	34.81249
3.224818	3.397549	3.430833	35.16721
3.316997	3.423024	3.397531	35.35168
3.285074	3.376657	3.419101	35.22764
3.37482	3.342421	3.393183	35.29235
3.190957	3.422822	3.401095	35.0834
3.39936	3.446679	3.36573	35.51398
3.377625	3.410489	3.370675	35.39812
3.334195	3.207701	3.364946	34.84715
3.202918	3.159987	3.365287	34.45647
3.256245	3.188561	3.367145	34.63964
3.209682	3.30185	3.394871	34.84619
3.247131	3.36082	3.432998	35.14042
3.229148	3.408871	3.464101	35.27419
3.225725	3.349363	3.471757	35.15331
3.27695	3.342893	3.35156	34.98833
3.427447	3.446208	3.423927	35.70164
3.279135	3.509423	3.38568	35.4319
3.41726	3.369042	3.389705	35.43577
3.268783	3.279408	3.395758	34.9283
3.317987	3.269299	3.351714	34.91747
3.245605	3.293561	3.336759	34.77954
3.31444	3.299828	3.388136	35.05613
3.223663	3.370794	3.421284	35.0853
3.2355	3.373962	3.346649	34.95489
3.187493	3.351991	3.429725	34.98354
3.360921	3.371198	3.322641	35.17062
3.316461	3.396943	3.500472	35.51858
3.239253	3.419452	3.459275	35.30888

3.281486	3.352867	3.431788	35.19551
3.336216	3.288506	3.345251	34.98521
3.3045	3.181148	3.404693	34.81107
3.265608	3.209858	3.321839	34.60761
3.100758	3.288978	3.460128	34.72255
3.188317	3.394854	3.392092	34.99678
3.27398	3.412578	3.422853	35.29014
3.285652	3.406108	3.403261	35.25867
3.31844	3.363516	3.429179	35.29391
3.177429	3.402806	3.419033	35.04927
3.319018	3.469189	3.367316	35.39098
3.379109	3.418104	3.401266	35.48491
3.35061	3.339389	3.324227	35.08198
3.197267	3.155134	3.421881	34.55726
3.339186	3.284193	3.318156	34.92302
3.219662	3.364257	3.440723	35.10476
3.337	3.432661	3.332411	35.27409
3.171325	3.351182	3.465755	35.0252
3.153096	3.342826	3.447884	34.92798
3.251255	3.323484	3.458985	35.12462
3.329617	3.438794	3.383805	35.38375
3.283713	3.448836	3.429469	35.40518
3.361828	3.366481	3.424746	35.38558
3.243542	3.325101	3.411599	35.00767
3.330154	3.217002	3.3607	34.84937
3.398164	3.180542	3.356488	34.90915
3.301283	3.212554	3.391614	34.84411
3.2209	3.345791	3.414071	35.0088
3.218343	3.367492	3.356284	34.92429
3.3264	3.42545	3.350878	35.27552
3.409877	3.403884	3.353334	35.41628
3.264618	3.410219	3.413918	35.24497
3.173717	3.416689	3.429827	35.09512
3.299262	3.46036	3.406961	35.41516
3.368757	3.354957	3.372244	35.26072
3.367726	3.231356	3.324926	34.88469
3.243707	3.215249	3.342114	34.61585
3.285693	3.341141	3.435948	35.18817
3.22222	3.397617	3.387948	35.0679
3.359436	3.402334	3.347212	35.2892
3.243419	3.329212	3.413304	35.02011
3.184152	3.32126	3.451431	34.95649
3.225725	3.453553	3.378706	35.17768
3.3589	3.474513	3.450527	35.6718
3.349497	3.410893	3.374478	35.34581
3.288126	3.308118	3.419664	35.08566
3.200855	3.243353	3.434499	34.78562
3.385255	3.215789	3.380684	35.01091
3.256245	3.19584	3.346172	34.60969
3.371108	3.320451	3.362882	35.16993

3.139114	3.33211	3.395178	34.75872
3.267051	3.381981	3.347348	35.04296
3.203866	3.367155	3.40662	35.00198
3.314316	3.371064	3.366446	35.16421
3.21426	3.400582	3.459872	35.21426
3.377047	3.471952	3.409945	35.61714
3.243089	3.353541	3.487154	35.23409
3.408145	3.304748	3.338601	35.16348
3.393545	3.201568	3.365849	34.9655
3.247254	3.351519	3.378502	35.00118
3.295055	3.427674	3.441882	35.41085
3.255668	3.412848	3.395962	35.19188
3.316048	3.290191	3.411531	35.08973
3.311347	3.36702	3.35156	35.11632
3.269155	3.455912	3.473939	35.48607
3.253482	3.449712	3.450545	35.38707
3.260122	3.378073	3.420056	35.17826
3.296746	3.265795	3.439273	35.05484
3.328999	3.308253	3.373267	35.07387
3.249316	3.227919	3.448771	34.88906
3.273361	3.22583	3.379133	34.78479
3.218054	3.246924	3.416373	34.79141
3.317079	3.3621	3.37516	35.1697
3.293653	3.424237	3.415657	35.34292
3.271959	3.468043	3.380633	35.31468
3.112306	3.341545	3.38921	34.70767
3.318523	3.419587	3.305862	35.14703
3.219786	3.415274	3.429571	35.19221
3.333535	3.379825	3.329854	35.14537
3.313244	3.228593	3.402578	34.92932
3.31147	3.211947	3.353521	34.78176
3.35391	3.272669	3.397412	35.10334
3.432726	3.415746	3.365406	35.51859
3.283425	3.4084	3.385049	35.21898
3.252987	3.33366	3.388119	34.99569
3.129628	3.273208	3.320919	34.44677
3.312213	3.404154	3.439018	35.39067
3.248079	3.510164	3.388818	35.37247
3.225973	3.429022	3.531898	35.45958
3.135402	3.286552	3.469421	34.81333
3.372057	3.245037	3.351714	34.98266
3.368221	3.272736	3.36515	35.06423
3.261442	3.27833	3.413355	34.94837
3.277816	3.233648	3.45196	34.97088
3.18143	3.307174	3.377291	34.75761
3.39705	3.346802	3.351287	35.25893
3.389338	3.435829	3.324551	35.37828
3.299221	3.32281	3.382475	35.06072
3.172439	3.404895	3.401845	35.00534
3.278228	3.438255	3.406398	35.31959

3.307346	3.442434	3.361808	35.2949
3.319966	3.256023	3.377683	34.94956
3.233974	3.20878	3.430952	34.77469
3.234964	3.290865	3.428241	34.95043
3.332958	3.400447	3.323476	35.17526
3.418992	3.43374	3.352379	35.49942
3.252987	3.346128	3.413628	35.07874
3.169304	3.27523	3.382117	34.67178
3.194958	3.305153	3.48226	35.01232
3.328669	3.529978	3.322044	35.44601
3.226344	3.467774	3.43172	35.32606
3.247708	3.284867	3.434363	34.97857
3.199329	3.188696	3.476787	34.75524
3.329906	3.308118	3.390898	35.11412
3.311841	3.366346	3.488246	35.41483
3.457307	3.341343	3.364724	35.40815
3.236077	3.251709	3.449249	34.91318
3.320585	3.343769	3.389005	35.16756
3.33436	3.350576	3.360427	35.15007
3.381048	3.408939	3.320202	35.29184
3.202876	3.372951	3.427286	35.05768
3.125669	3.463258	3.385118	34.99411
3.190215	3.377331	3.408052	34.99751
3.397463	3.273747	3.394138	35.19378
3.288209	3.228054	3.440655	34.95666
3.316007	3.291606	3.414873	35.10005
3.326235	3.386362	3.431379	35.36572
3.329205	3.437177	3.347604	35.30014
3.372634	3.388249	3.343614	35.27939
3.291632	3.317553	3.372227	35.01022
3.136722	3.394247	3.368134	34.83023
3.242223	3.534156	3.414327	35.46792
3.32372	3.455575	3.378553	35.39606
3.175903	3.327258	3.43218	34.90947
3.226962	3.206353	3.37811	34.63849
3.161179	3.273478	3.505314	34.9196
3.262556	3.332717	3.384163	35.0059
3.261937	3.266334	3.37782	34.84551
3.35523	3.282239	3.419494	35.17544
3.249564	3.402065	3.404079	35.1727
3.323183	3.422081	3.359626	35.28025
3.329782	3.382049	3.40916	35.31546
3.333577	3.37841	3.396048	35.28713
3.087766	3.380836	3.410627	34.78676
3.315182	3.439333	3.482329	35.56881
3.179615	3.397212	3.325028	34.83625
3.350157	3.216395	3.353692	34.87646
3.265938	3.209319	3.393712	34.76433
3.326029	3.331571	3.415555	35.21085
3.343558	3.395797	3.434908	35.43196

3.36022	3.407658	3.309682	35.22048
3.354982	3.329616	3.415555	35.26989
3.314481	3.365537	3.341619	35.09819
3.185925	3.438794	3.420994	35.15084
3.188276	3.470335	3.370181	35.11383
3.289694	3.40348	3.449368	35.36259
3.144806	3.263099	3.385459	34.59899
3.227664	3.259527	3.4183	34.8442
3.224653	3.263773	3.455933	34.92919
3.223251	3.260942	3.387437	34.77015
3.268165	3.226908	3.35156	34.71548
3.374614	3.282104	3.395911	35.16596
3.280826	3.414735	3.408871	35.27925
3.332298	3.410152	3.383685	35.32671
3.302974	3.358865	3.308437	34.98587
3.282971	3.33858	3.366037	35.02373
3.253606	3.450925	3.416271	35.31505
3.325823	3.562799	3.411155	35.70644
3.290106	3.41305	3.356949	35.18231
3.287219	3.223269	3.30443	34.64613
3.296953	3.242476	3.450545	35.02895
3.366819	3.334873	3.358261	35.18198
3.332917	3.394584	3.452437	35.44437
3.342362	3.385418	3.38759	35.30317
3.301902	3.254405	3.40899	34.97498
3.356343	3.332717	3.403397	35.25306
3.244656	3.509558	3.356471	35.29292
3.174047	3.508614	3.4803	35.40724
3.171779	3.393034	3.424626	35.02778
3.182337	3.265121	3.387437	34.68981
3.25641	3.26849	3.413798	34.91682
3.2866	3.34451	3.447475	35.22273
3.199659	3.338849	3.440245	35.00441
3.214507	3.211745	3.358398	34.57994
3.352054	3.321394	3.443929	35.30756
3.288993	3.308253	3.32837	34.88821
3.297365	3.349295	3.423347	35.20397
3.25208	3.365066	3.35243	34.98434
3.306439	3.420935	3.439563	35.41594
3.287178	3.406513	3.331115	35.10512
3.292003	3.314116	3.384504	35.03037
3.159365	3.224348	3.396252	34.56969
3.253606	3.332312	3.3895	34.99712
3.248986	3.375377	3.432044	35.17423
3.4239	3.437042	3.382594	35.58345
3.374944	3.356102	3.458661	35.46573
3.383729	3.337973	3.304703	35.10861
3.269402	3.260471	3.424132	34.95029
3.324462	3.425652	3.353146	35.27669
3.259462	3.45234	3.415623	35.32953

3.235995	3.320923	3.3526	34.853
3.056421	3.211677	3.402885	34.33137
3.184276	3.245509	3.412127	34.70516
3.31246	3.441086	3.463214	35.52489
3.320049	3.402469	3.465823	35.46274
3.22024	3.257505	3.440638	34.87239
3.269526	3.232907	3.410371	34.86019
3.395029	3.366076	3.417737	35.44197
3.370984	3.451127	3.333434	35.39103
3.243006	3.405232	3.425359	35.21182
3.203041	3.338445	3.31241	34.73137
3.294602	3.338378	3.400805	35.12475
3.223251	3.377668	3.30576	34.84679
3.346321	3.259055	3.394871	35.05141
3.127071	3.228863	3.438063	34.60038
3.195947	3.300705	3.41385	34.85515
3.274764	3.400245	3.45641	35.33826
3.417631	3.441625	3.321635	35.44645
3.340877	3.391551	3.436767	35.42087
3.338526	3.418644	3.339096	35.26139
3.233768	3.41642	3.526049	35.43627
3.313821	3.500594	3.323749	35.35302
3.234428	3.342286	3.38198	34.96054
3.293694	3.258247	3.335668	34.80509
3.083477	3.216125	3.415538	34.42793
3.192895	3.247598	3.377973	34.65389
3.215497	3.414263	3.442275	35.2084
3.317203	3.336019	3.406739	35.182
3.218755	3.231761	3.433698	34.79767
3.279053	3.304142	3.412196	35.04079
3.320915	3.416285	3.456598	35.47468
3.475289	3.427876	3.296876	35.48833
3.264123	3.368166	3.45803	35.24838
3.16188	3.35772	3.353743	34.77389
3.206258	3.352463	3.437159	35.04186
3.235046	3.414465	3.356062	35.06306
3.292003	3.290663	3.36752	34.94194
3.252533	3.265525	3.421881	34.91953
3.163943	3.308185	3.443775	34.86697
3.308789	3.331167	3.465703	35.28193
3.469226	3.497899	3.309954	35.6568
3.331597	3.3559	3.404693	35.25248
3.299056	3.331032	3.357221	35.02312
3.285033	3.389395	3.431771	35.28311
3.345331	3.505447	3.367913	35.52911
3.302809	3.374096	3.281308	34.95949
3.257153	3.287832	3.404505	34.94042
3.047182	3.193751	3.392501	34.24925
3.224034	3.287698	3.398213	34.85394
3.223581	3.384677	3.455507	35.19031

3.263917	3.382386	3.430492	35.21881
3.245893	3.277858	3.408734	34.90323
3.354363	3.286215	3.399219	35.1379
3.31345	3.338782	3.428633	35.22771
3.321204	3.417228	3.364929	35.27691
3.225065	3.453149	3.404181	35.23106
3.287302	3.392427	3.373864	35.16807
3.128061	3.292483	3.389432	34.63532
3.294602	3.296526	3.381025	34.98998
3.256122	3.288911	3.373949	34.8737
3.221518	3.439266	3.377069	35.13365
3.242016	3.409411	3.406739	35.17807
3.379068	3.36702	3.385817	35.33933
3.407774	3.391147	3.401487	35.48913
3.45541	3.376253	3.287259	35.31093
3.300005	3.373692	3.38464	35.17845
3.250224	3.405704	3.415197	35.20641
3.30549	3.445601	3.402067	35.3858
3.200649	3.238568	3.372227	34.63853
3.301118	3.199277	3.345575	34.71403
3.159282	3.291472	3.47735	34.89365
3.188482	3.453351	3.389159	35.11865
3.23781	3.404693	3.43825	35.22747
3.229272	3.299896	3.364946	34.81932
3.209599	3.229065	3.372107	34.63706
3.358694	3.339793	3.440109	35.35396
3.223869	3.400515	3.438046	35.1874
3.358199	3.443579	3.37001	35.42655
3.300252	3.402402	3.397736	35.27041
3.358529	3.277791	3.349054	35.01889
3.257029	3.217002	3.358944	34.68561
3.197267	3.302592	3.34699	34.71596
3.189885	3.311825	3.406193	34.84947
3.264989	3.471076	3.371562	35.28623
3.233067	3.418576	3.480879	35.34068
3.306232	3.394921	3.38077	35.23002
3.359354	3.373759	3.351577	35.23608
3.360962	3.41952	3.426792	35.50415
3.285611	3.444792	3.372227	35.27531
3.29254	3.405704	3.449453	35.37386
3.199866	3.342421	3.4109	34.9485
3.177305	3.177846	3.38273	34.47766
3.270062	3.230683	3.331849	34.68478
3.261071	3.312094	3.379832	34.94808
3.251832	3.477478	3.478646	35.50564
3.129381	3.392158	3.394752	34.86781
3.337371	3.240522	3.404011	35.0113
3.321327	3.266199	3.341466	34.89559
3.334897	3.385418	3.446111	35.41482
3.238717	3.408063	3.345439	35.03386

3.27365	3.421878	3.415879	35.2945
3.228406	3.373827	3.374409	34.99979
3.425385	3.252788	3.311591	35.02849
3.191163	3.257842	3.407421	34.7369
3.159241	3.308657	3.394002	34.74887
3.167984	3.385486	3.46069	35.08184
3.301778	3.448027	3.398503	35.3752
3.241563	3.380229	3.419766	35.14175
3.33601	3.397617	3.461321	35.47719
3.210465	3.348554	3.458167	35.08845
3.397586	3.408737	3.343904	35.3794
3.302603	3.419991	3.423876	35.37118
3.406289	3.396404	3.365781	35.4193
3.22985	3.136264	3.420773	34.58483
3.210795	3.186135	3.3105	34.41107
3.185843	3.373288	3.391529	34.94297
3.319018	3.456114	3.350418	35.32543
3.187286	3.387373	3.489064	35.19022
3.218466	3.295717	3.413082	34.89181
3.254348	3.277252	3.443093	34.99553
3.423652	3.331369	3.375842	35.33705
3.303716	3.400919	3.425308	35.33504
3.255544	3.412443	3.470427	35.35356
3.224818	3.351519	3.43675	35.07949
3.253235	3.260201	3.342557	34.73595
3.422827	3.20696	3.346666	34.99938
3.2607	3.259055	3.343205	34.75119
3.127236	3.326719	3.391529	34.71297
3.087931	3.417902	3.481152	35.02241
3.332546	3.451532	3.317594	35.27321
3.327266	3.345454	3.3644	35.13205
3.335103	3.355428	3.376882	35.19829
3.248203	3.421002	3.501119	35.42335
3.264247	3.457462	3.369584	35.25051
3.351146	3.465617	3.428906	35.58811
3.404309	3.312027	3.374426	35.24935
3.191823	3.152169	3.442547	34.58407
3.275465	3.254068	3.383702	34.86113
3.202381	3.393843	3.431293	35.11105
3.274062	3.488194	3.395008	35.39478
3.239624	3.343904	3.379047	34.96903
3.192937	3.266064	3.493463	34.94692
3.177223	3.227515	3.429179	34.68768
3.394988	3.471885	3.4242	35.6874
3.290684	3.443444	3.419647	35.38715
3.355518	3.423833	3.406824	35.45801
3.256204	3.312229	3.408512	35.00046
3.269608	3.176565	3.351969	34.60944
3.307263	3.278465	3.298223	34.79709
3.268453	3.353406	3.384708	35.06524

3.149714	3.42909	3.386635	34.97529
3.22688	3.413117	3.389432	35.11523
3.298809	3.332514	3.373165	35.06069
3.354528	3.312364	3.374546	35.14149
3.346197	3.407524	3.453529	35.50409
3.160189	3.475456	3.438728	35.21352
3.293364	3.406243	3.421676	35.3161
3.256864	3.308724	3.331917	34.82673
3.365953	3.163761	3.391035	34.87756
3.24016	3.217676	3.384981	34.70714
3.196566	3.390069	3.407762	35.03862
3.246512	3.475659	3.423586	35.36962
3.363932	3.399975	3.363275	35.329
3.178419	3.28763	3.409945	34.77969
3.261978	3.251709	3.403141	34.86899
3.208197	3.30805	3.433834	34.94171
3.29621	3.458743	3.415418	35.42345
3.384347	3.425989	3.410576	35.53397
3.380264	3.329347	3.370539	35.22615
3.266144	3.164435	3.451619	34.79326
3.300994	3.18472	3.302315	34.58733
3.200567	3.283991	3.414088	34.82923
3.321245	3.458945	3.374426	35.38899
3.276537	3.454632	3.442223	35.43005
3.178996	3.39391	3.400669	34.99309
3.236572	3.319642	3.453955	35.07311
3.310728	3.408198	3.402732	35.31692
3.29188	3.416217	3.420807	35.33276
3.270969	3.431516	3.441251	35.3652
3.188895	3.283182	3.410849	34.79485
3.282641	3.186809	3.359421	34.67664
3.373418	3.161402	3.317866	34.72872
3.27563	3.327123	3.403465	35.06447
3.187575	3.451464	3.370675	35.07212
3.24049	3.353137	3.429384	35.10119
3.27563	3.365268	3.435471	35.21788
3.255544	3.317351	3.363735	34.91229
3.31477	3.313577	3.43854	35.19714
3.105666	3.380566	3.5128	35.04875
3.281363	3.429224	3.394002	35.27959
3.278187	3.374096	3.454688	35.2848
3.430746	3.28298	3.329871	35.14621
3.265525	3.155741	3.365832	34.58529
3.307098	3.285945	3.391154	35.01632
3.186709	3.409545	3.419289	35.08486
3.368015	3.46838	3.299655	35.34839
3.20964	3.39883	3.370828	35.0056
3.331391	3.356507	3.329274	35.08842
3.130329	3.364661	3.42142	34.86808
3.249316	3.42417	3.409331	35.23198

3.322276	3.455238	3.429196	35.50291
3.412558	3.351654	3.378263	35.36244
3.194669	3.174476	3.422699	34.59567
3.262803	3.174611	3.323323	34.52764
3.319719	3.252788	3.40355	34.99851
3.335185	3.537391	3.35729	35.55355
3.162953	3.413656	3.376814	34.94902
3.100139	3.308185	3.447953	34.73657
3.230015	3.301244	3.326324	34.73943
3.234634	3.289922	3.422375	34.93482
3.242965	3.393169	3.348764	35.01785
3.235665	3.446073	3.483812	35.4129
3.236902	3.361292	3.436426	35.12658
3.207454	3.217271	3.336333	34.52835
3.43586	3.214643	3.306084	34.95593
3.246924	3.228998	3.360973	34.69419
3.248327	3.402604	3.441951	35.25399
3.277403	3.465819	3.382117	35.32497
3.351518	3.455575	3.425513	35.55954
3.307428	3.421811	3.265893	35.04023
3.294107	3.359741	3.472984	35.32823
3.113131	3.435088	3.416424	34.97355
3.278146	3.439333	3.417055	35.34508
3.281899	3.364459	3.376541	35.10095
3.363107	3.200086	3.428429	35.03255
3.173057	3.210464	3.397531	34.57207
3.317533	3.279947	3.404642	35.05551
3.296746	3.416891	3.457433	35.42498
3.366035	3.437177	3.362337	35.4129
3.166128	3.341208	3.428906	34.91145
3.301077	3.316407	3.361535	35.00499
3.133464	3.301042	3.417328	34.72686
3.26136	3.437042	3.365815	35.1913
3.203413	3.416959	3.460434	35.22758
3.331968	3.340467	3.441064	35.29908
3.316667	3.271523	3.421403	35.07185
3.412352	3.200288	3.388681	35.05376
3.297118	3.227717	3.416015	34.92152
3.346197	3.327056	3.332786	35.06408
3.241233	3.425181	3.424694	35.25011
3.230509	3.44614	3.405239	35.22995
3.281486	3.433875	3.453955	35.42114
3.244243	3.394314	3.301701	34.92023
3.284002	3.447892	3.430833	35.40673
3.227045	3.465752	3.412895	35.282
3.199206	3.391753	3.462805	35.16845
3.162004	3.205208	3.445975	34.64234
3.410413	3.164771	3.341346	34.86834
3.247625	3.219091	3.4124	34.78652
3.329246	3.391147	3.353623	35.21273

3.289446	3.442568	3.493105	35.54317
3.294684	3.37996	3.333997	35.06977
3.26503	3.31789	3.412741	35.04138
3.274846	3.346061	3.419494	35.13922
3.120926	3.373422	3.449283	34.9276
3.257153	3.382386	3.406534	35.15163
3.191246	3.391821	3.477605	35.18355
3.289075	3.333593	3.406756	35.11522
3.349084	3.218417	3.390523	34.95908
3.291385	3.240994	3.377564	34.85393
3.282517	3.250631	3.391836	34.88683
3.298726	3.415409	3.421114	35.34664
3.299922	3.413993	3.339147	35.16691
3.365169	3.409074	3.315292	35.24667
3.171738	3.360348	3.389756	34.87995
3.235995	3.426259	3.39407	35.17404
3.318193	3.486442	3.346649	35.38171
3.270845	3.444186	3.481612	35.4809
3.208114	3.326247	3.409553	34.92822
3.216239	3.123998	3.406807	34.4977
3.238552	3.231761	3.407319	34.78327
3.368922	3.407052	3.396645	35.42836
3.344053	3.45443	3.46451	35.62599
3.238799	3.359404	3.38493	35.01399
3.285528	3.297807	3.437585	35.09662
3.224034	3.342286	3.338669	34.8431
3.341207	3.32874	3.453631	35.32112
3.156436	3.39209	3.358739	34.84808
3.170995	3.364729	3.48949	35.10601
3.23649	3.367357	3.392978	35.04393
3.308212	3.245442	3.38464	34.91593
3.273114	3.224078	3.334236	34.68223
3.296623	3.259257	3.408223	34.97237
3.205433	3.378612	3.42507	35.0708
3.383399	3.418037	3.344705	35.37046
3.418579	3.435627	3.377069	35.55663
3.360591	3.366076	3.348218	35.21464
3.219167	3.384205	3.398554	35.05509
3.244408	3.453621	3.363786	35.18605
3.233561	3.439468	3.406603	35.22502
3.347105	3.375309	3.280984	35.0583
3.163406	3.15938	3.510344	34.68596
3.195288	3.19894	3.364247	34.52269
3.204402	3.336288	3.464254	35.06169
3.39606	3.512995	3.348559	35.61423
3.342444	3.416352	3.421762	35.44572
3.250389	3.2941	3.398623	34.92647
3.230592	3.302659	3.413014	34.93336
3.271175	3.354754	3.340426	34.9773
3.263463	3.365874	3.410644	35.1383

3.224735	3.37012	3.436153	35.11868
3.201268	3.375309	3.46763	35.14755
3.226468	3.260538	3.379542	34.75903
3.41994	3.229739	3.372585	35.09956
3.287095	3.23722	3.360103	34.79811
3.270722	3.308792	3.402613	35.01179
3.247502	3.403817	3.4626	35.29999
3.3914	3.475322	3.356215	35.5384
3.38876	3.388586	3.331644	35.28922
3.397875	3.37039	3.29604	35.1915
3.096922	3.373288	3.454432	34.88608
3.245934	3.432931	3.38771	35.19646
3.173717	3.386969	3.438608	35.04933
3.240284	3.300233	3.352652	34.81725
3.147486	3.169219	3.392774	34.41555
3.249935	3.238635	3.413918	34.83763
3.228942	3.405974	3.437449	35.20912
3.444728	3.393438	3.403533	35.57943
3.332174	3.396875	3.416152	35.3684
3.336876	3.303198	3.327824	34.98067
3.219126	3.365739	3.405443	35.02968
3.239459	3.369648	3.372193	35.00998
3.278022	3.391551	3.383003	35.16585
3.27695	3.372546	3.367043	35.08704
3.178708	3.284463	3.424626	34.8055
3.254307	3.2354	3.412076	34.83609
3.288456	3.251035	3.420858	34.96416
3.295963	3.33238	3.402954	35.11931
3.336917	3.321664	3.376319	35.12719
3.312006	3.408534	3.477691	35.48437
3.354776	3.455373	3.34653	35.3935
3.377625	3.366346	3.314081	35.17782
3.280991	3.374298	3.312478	34.98039
3.234056	3.456249	3.36145	35.16405
3.195947	3.44614	3.438199	35.22645
3.202175	3.312296	3.387505	34.83651
3.294272	3.222393	3.376814	34.81793
3.117049	3.193751	3.403602	34.42631
3.311182	3.402199	3.432453	35.36979
3.275547	3.436907	3.48533	35.48339
3.373129	3.407052	3.364315	35.36686
3.394411	3.330223	3.364315	35.24539
3.363272	3.307714	3.366668	35.13321
3.240449	3.346465	3.38846	34.99702
3.267793	3.380499	3.335753	35.01598
3.129092	3.341478	3.392228	34.75083
3.296334	3.292887	3.364519	34.94971
3.19005	3.234591	3.382134	34.62832
3.17846	3.218889	3.434772	34.68375
3.279135	3.276106	3.43866	35.03753

3.271794	3.317283	3.451278	35.13912
3.297984	3.38899	3.41564	35.27527
3.410537	3.470335	3.335259	35.52351
3.334525	3.342758	3.394956	35.20885
3.341001	3.388788	3.336231	35.19525
3.308088	3.409006	3.37579	35.254
3.288456	3.440277	3.293909	35.10039
3.290065	3.332245	3.352123	34.99496
3.213228	3.200221	3.406893	34.65799
3.19071	3.171106	3.391529	34.51148
3.204526	3.32928	3.387232	34.87819
3.309449	3.391147	3.467852	35.41924
3.263834	3.408534	3.431907	35.27891
3.294602	3.363785	3.397088	35.17219
3.300335	3.324629	3.336487	34.96657
3.412228	3.416352	3.443025	35.64483
3.223044	3.384408	3.381861	35.0275
3.170748	3.323416	3.442718	34.91284
3.184111	3.270714	3.430901	34.80097
3.318729	3.28763	3.322112	34.89445
3.200855	3.227852	3.386345	34.64642
3.293694	3.295987	3.346445	34.91119
3.158004	3.331167	3.496993	35.02061
3.380718	3.356776	3.354016	35.25099
3.418909	3.413252	3.473462	35.71922
3.49059	3.48725	3.3083	35.67662
3.323967	3.36729	3.471143	35.40601
3.342279	3.394449	3.307107	35.14673
3.196937	3.371064	3.405528	34.99299
3.404845	3.387979	3.308863	35.27325
3.217889	3.249755	3.388954	34.73728
3.076424	3.222932	3.376592	34.34222
3.205392	3.310342	3.403124	34.87343
3.289488	3.431583	3.41049	35.33858
3.390905	3.425518	3.422256	35.57282
3.355972	3.340871	3.442633	35.35588
3.224859	3.298615	3.367128	34.81164
3.301077	3.338108	3.405307	35.14817
3.380718	3.391147	3.283388	35.1717
3.266309	3.34478	3.452403	35.18972
3.157344	3.273612	3.401913	34.68538
3.192607	3.260066	3.438199	34.81223
3.280909	3.264649	3.323391	34.76428
3.279713	3.301311	3.43143	35.07811
3.279465	3.392293	3.433271	35.28055
3.12604	3.33986	3.460145	34.88914
3.350033	3.425113	3.385254	35.40164
3.349455	3.484285	3.412281	35.58888
3.467824	3.420396	3.390983	35.66145
3.260081	3.391012	3.382628	35.12462

3.261648	3.284058	3.355243	34.83427
3.281198	3.274084	3.343699	34.82996
3.337289	3.295246	3.343529	34.99853
3.176975	3.26566	3.351373	34.6004
3.215662	3.273882	3.420397	34.85393
3.169304	3.426057	3.43056	35.10756
3.273485	3.454767	3.404369	35.34089
3.340712	3.370929	3.388136	35.26907
3.310687	3.348824	3.420449	35.22573
3.227499	3.353204	3.515119	35.26042
3.364509	3.445803	3.377751	35.46214
3.248409	3.330223	3.437568	35.0863
3.325658	3.264649	3.360512	34.94332
3.189843	3.175622	3.4493	34.64579
3.171408	3.23385	3.356693	34.5303
3.149095	3.297065	3.487444	34.90568
3.227664	3.404154	3.344467	34.99901
3.258472	3.394651	3.497948	35.38124
3.325534	3.433672	3.392484	35.3826
3.309944	3.388586	3.417652	35.30494
3.434871	3.440142	3.366719	35.5795
3.438665	3.422013	3.382543	35.58276
3.255008	3.427203	3.365525	35.15526
3.280579	3.291269	3.354493	34.88979
3.279012	3.223067	3.390387	34.81571
3.338237	3.24834	3.351969	34.91648
3.246264	3.24625	3.418334	34.85591
3.222714	3.392832	3.444321	35.18179
3.135567	3.43064	3.483608	35.15981
3.323472	3.425787	3.348218	35.26404
3.319636	3.399706	3.507974	35.54798
3.372387	3.409343	3.389005	35.42424
3.271175	3.441153	3.468193	35.44564
3.301778	3.37996	3.436494	35.30943
3.220034	3.304883	3.472166	35.04449
3.266144	3.232772	3.363923	34.75092
3.133257	3.211341	3.398708	34.48952
3.234675	3.257438	3.350554	34.70681
3.096881	3.350306	3.421455	34.76361
3.21294	3.366481	3.426058	35.06285
3.345867	3.377668	3.456734	35.44509
3.304459	3.39391	3.40708	35.28147
3.298685	3.480578	3.382799	35.40528
3.337082	3.441692	3.436409	35.52144
3.432561	3.414533	3.363616	35.51166
3.394452	3.350778	3.376268	35.31657
3.24911	3.253057	3.367674	34.76624
3.248698	3.236748	3.363223	34.71994
3.1738	3.23722	3.353606	34.53615
3.226591	3.317216	3.352328	34.82374

3.318152	3.409545	3.463146	35.46821
3.18675	3.374029	3.456257	35.08813
3.25971	3.331571	3.408717	35.05087
3.356632	3.366211	3.41726	35.35726
3.348053	3.419991	3.452983	35.53422
3.252203	3.487722	3.36781	35.28647
3.308295	3.279139	3.418471	35.06378
3.229561	3.187281	3.351663	34.54463
3.339722	3.206488	3.379712	34.88888
3.265278	3.240185	3.382867	34.80667
3.27963	3.298615	3.362831	34.92202
3.10781	3.367222	3.447782	34.88208
3.218549	3.371738	3.412196	35.0563
3.339227	3.390945	3.39644	35.32775
3.348259	3.387845	3.390659	35.32808
3.270639	3.430437	3.454415	35.39091
3.379192	3.391888	3.369498	35.3583
3.281239	3.33029	3.383259	35.03948
3.369294	3.332447	3.342301	35.14718
3.287137	3.259257	3.381844	34.89394
3.210094	3.295448	3.325352	34.68106
3.129504	3.292011	3.397548	34.65519
3.266391	3.43037	3.381315	35.22161
3.214672	3.464202	3.495015	35.43114
3.295179	3.39081	3.409672	35.26007
3.164932	3.345454	3.47515	35.01924
3.329246	3.38899	3.427559	35.3697
3.411486	3.4208	3.42229	35.60759
3.327803	3.363785	3.366173	35.17719
3.341001	3.233378	3.397122	34.98855
3.262184	3.214306	3.321072	34.60817
3.244285	3.256899	3.355789	34.73809
3.209393	3.311218	3.345302	34.75765
3.283012	3.357652	3.428412	35.20193
3.109336	3.384138	3.3762	34.76587
3.2826	3.393236	3.395144	35.20609
3.298932	3.458743	3.423057	35.44611
3.305201	3.492979	3.420125	35.52827
3.268618	3.397212	3.421864	35.24265
3.328792	3.350374	3.435386	35.30138
3.238428	3.224617	3.389824	34.72912
3.418662	3.248137	3.292698	34.9623
3.266102	3.273006	3.41813	34.95736
3.306604	3.334199	3.322249	34.97007
3.14064	3.356102	3.383617	34.78924
3.276785	3.442501	3.351731	35.20617
3.181636	3.436503	3.406978	35.1058
3.324256	3.391753	3.381571	35.26426
3.244697	3.437042	3.518393	35.48853
3.353456	3.41952	3.387727	35.4023

3.294148	3.402671	3.473036	35.42232
3.362653	3.332514	3.407779	35.276
3.280249	3.142599	3.380752	34.62138
3.19504	3.165513	3.416373	34.56305
3.269979	3.242139	3.376916	34.80821
3.185142	3.264447	3.3489	34.6102
3.293983	3.421878	3.385203	35.27188
3.201433	3.424237	3.405801	35.1197
3.196154	3.386227	3.45774	35.13861
3.266309	3.48415	3.453887	35.49774
3.328916	3.486779	3.359233	35.43341
3.292251	3.389866	3.374614	35.17493
3.359313	3.346465	3.412076	35.3086
3.280125	3.275297	3.479447	35.12713
3.399855	3.287832	3.330024	35.0896
3.293488	3.266671	3.346223	34.84614
3.341166	3.333795	3.34844	35.10204
3.223044	3.297402	3.374119	34.82031
3.292622	3.389125	3.365764	35.15477
3.174666	3.404895	3.445224	35.10507
3.360591	3.420126	3.367725	35.37549
3.254513	3.43529	3.450186	35.35701
3.329617	3.385958	3.465192	35.44618
3.277651	3.391349	3.420738	35.24712
3.328133	3.299694	3.39939	35.11039
3.329617	3.192605	3.355448	34.78336
3.223622	3.154123	3.348065	34.45126
3.173923	3.262155	3.367793	34.62197
3.195865	3.318362	3.333281	34.7174
3.269113	3.416352	3.386908	35.20914
3.295385	3.358057	3.399714	35.16712
3.211166	3.372075	3.432521	35.08534
3.187121	3.462719	3.456274	35.28293
3.341454	3.445399	3.415214	35.49276
3.340794	3.424844	3.399458	35.41191
3.332381	3.345993	3.383481	35.18614
3.266762	3.256696	3.410149	34.90568
3.264741	3.278599	3.370794	34.8631
3.290189	3.326921	3.341193	34.95969
3.397586	3.356844	3.358534	35.29791
3.125091	3.335749	3.412349	34.77356
3.274887	3.34815	3.366361	35.02769
3.193514	3.407254	3.435744	35.13072
3.321616	3.462247	3.379951	35.40911
3.273196	3.420059	3.434584	35.33044
3.282394	3.362302	3.430185	35.21463
3.190957	3.377062	3.499022	35.19748
3.360839	3.30057	3.353828	35.08419
3.349992	3.214778	3.342421	34.84791
3.307016	3.252181	3.358926	34.87182

3.178955	3.260134	3.404232	34.70824
3.212692	3.360146	3.391853	34.97366
3.352342	3.437649	3.321345	35.29435
3.35523	3.362302	3.35376	35.20678
3.151528	3.403884	3.483249	35.13542
3.240078	3.447353	3.426331	35.29965
3.313945	3.378814	3.413764	35.28382
3.453265	3.34788	3.363223	35.41032
3.347435	3.261144	3.325881	34.90755
3.228736	3.237152	3.400737	34.7592
3.270103	3.364055	3.370811	35.06174
3.283837	3.423361	3.331508	35.13552
3.406701	3.375242	3.338823	35.31497
3.254761	3.343702	3.409808	35.06896
3.188812	3.356035	3.410507	34.95324
3.262968	3.42882	3.418317	35.29165
3.348218	3.488463	3.368544	35.49967
3.208568	3.381577	3.397531	35.02392
3.267299	3.270512	3.430697	34.982
3.217971	3.254675	3.440569	34.86109
3.368139	3.285945	3.41651	35.20525
3.410413	3.266064	3.336163	35.07851
3.306356	3.251575	3.395178	34.94833
3.253193	3.29383	3.393439	34.92067
3.255091	3.363785	3.372329	35.03164
3.263999	3.42107	3.387096	35.20868
3.348301	3.403413	3.325233	35.21914
3.191411	3.434751	3.462054	35.24379
3.222714	3.462652	3.435931	35.31613
3.322441	3.352935	3.402118	35.22034
3.393586	3.327662	3.383191	35.27926
3.288291	3.191325	3.357221	34.69406
3.276083	3.238096	3.370794	34.79933
3.299675	3.287698	3.387505	34.99593
3.320049	3.359404	3.331388	35.07458
3.339928	3.375579	3.357699	35.21096
3.225478	3.378881	3.384453	35.02641
3.229602	3.415678	3.447322	35.25338
3.202423	3.480578	3.385476	35.20062
3.359106	3.448162	3.409825	35.52562
3.234428	3.331369	3.426041	35.03302
3.238387	3.314116	3.454569	35.06634
3.250966	3.316677	3.44149	35.07085
3.339062	3.279408	3.435727	35.16939
3.353539	3.198738	3.354578	34.84718
3.378697	3.245374	3.385339	35.07145
3.196236	3.321664	3.439478	34.95767
3.306315	3.439401	3.335191	35.2278
3.250306	3.382116	3.419647	35.16474
3.32174	3.361763	3.361655	35.14963

3.152642	3.411904	3.415691	35.00765
3.216652	3.477815	3.505826	35.48888
3.230674	3.374298	3.396662	35.05445
3.431984	3.23412	3.375347	35.14152
3.27299	3.178857	3.395843	34.71779
3.261937	3.340467	3.331422	34.90616
3.304748	3.340332	3.431208	35.2177
3.22622	3.375512	3.319248	34.87807
3.394988	3.321057	3.365474	35.22914
3.257648	3.330627	3.347826	34.91114
3.27897	3.436638	3.482823	35.48481
3.139485	3.491564	3.387437	35.0913
3.308624	3.423361	3.448072	35.44463
3.261731	3.331638	3.419835	35.07975
3.271464	3.298076	3.379491	34.93941
3.198422	3.270243	3.508571	35.00109
3.272248	3.282913	3.357494	34.85986
3.261153	3.218147	3.367401	34.71563
3.330978	3.298278	3.382134	35.07578
3.242924	3.362976	3.441541	35.15462
3.26037	3.431718	3.377888	35.2039
3.236366	3.321192	3.351833	34.85273
3.287425	3.440344	3.353623	35.22887
3.174006	3.478152	3.423995	35.21741
3.374078	3.461439	3.394326	35.5535
3.232365	3.324629	3.444116	35.0533
3.299551	3.225224	3.402459	34.89174
3.360921	3.251507	3.417106	35.11546
3.381955	3.388451	3.405938	35.43651
3.343516	3.414735	3.364417	35.31913
3.303923	3.334469	3.321345	34.96282
3.263752	3.261616	3.331797	34.73852
3.348383	3.380768	3.31582	35.14922
3.243254	3.508075	3.38198	35.3424
3.133175	3.432594	3.509765	35.21605
3.231004	3.330223	3.431089	35.03407
3.28293	3.267075	3.341142	34.81283
3.245934	3.258516	3.424899	34.89637
3.257689	3.323349	3.459275	35.13903
3.220157	3.316407	3.482329	35.09219
3.222137	3.27341	3.330587	34.67066
3.419157	3.355967	3.381639	35.39369
3.300128	3.388384	3.356471	35.14924
3.28957	3.37969	3.3762	35.15029
3.193885	3.361898	3.409962	34.97596
3.260493	3.415072	3.362422	35.13394
3.159777	3.483072	3.443417	35.23952
3.343723	3.378949	3.385254	35.28689
3.249811	3.259729	3.427406	34.91298
3.361993	3.223202	3.359268	34.92942

3.219126	3.37942	3.492679	35.25037
3.465844	3.504638	3.39453	35.8491
3.35523	3.38589	3.393319	35.34487
3.265484	3.254742	3.383958	34.84134
3.255833	3.317418	3.381639	34.95222
3.328834	3.492911	3.361604	35.45183
3.29786	3.540828	3.428701	35.63561
3.243089	3.366009	3.399987	35.07074
3.105996	3.189033	3.489882	34.58051
3.272083	3.204871	3.39835	34.77818
3.346857	3.305759	3.454415	35.28493
3.288209	3.305961	3.393797	35.02456
3.270227	3.25791	3.428088	34.95514
3.277609	3.26903	3.391887	34.91644
3.357127	3.341141	3.363241	35.18538
3.414043	3.427405	3.38464	35.54529
3.144558	3.378746	3.378996	34.83722
3.125297	3.313712	3.428565	34.76128
3.296416	3.439131	3.386823	35.31848
3.274269	3.419183	3.392689	35.23925
3.354322	3.32281	3.304003	35.00962
3.145713	3.242342	3.427559	34.64765
3.245151	3.242544	3.375603	34.75193
3.30714	3.356304	3.40355	35.19738
3.480898	3.421272	3.392961	35.69628
3.291591	3.383936	3.385203	35.18368
3.330071	3.318227	3.376336	35.10474
3.246718	3.393371	3.447202	35.24176
3.347764	3.503897	3.354953	35.50271
3.253193	3.463258	3.403209	35.31255
3.174295	3.261953	3.422614	34.74223
3.187575	3.198738	3.389824	34.56132
3.258143	3.25609	3.419238	34.90538
3.343063	3.370187	3.413628	35.32833
3.385172	3.309398	3.40928	35.27798
3.243419	3.250631	3.413304	34.84827
3.277362	3.31607	3.372107	34.97551
3.344011	3.388856	3.375978	35.2889
3.429303	3.399302	3.353606	35.44934
3.201557	3.360011	3.416629	35.00319
3.108924	3.340197	3.345712	34.6022
3.232613	3.466695	3.483062	35.44969
3.284332	3.38434	3.363633	35.12152
3.33964	3.249351	3.352515	34.92295
3.218013	3.197929	3.414719	34.68056
3.278723	3.315531	3.431856	35.10797
3.30813	3.398695	3.388136	35.25854
3.462586	3.413993	3.309477	35.45775
3.304418	3.349093	3.435914	35.24643
3.283425	3.345319	3.409484	35.13447

3.296086	3.419183	3.406671	35.31754
3.355807	3.522565	3.383771	35.62414
3.246141	3.398223	3.329001	34.99263
3.258472	3.272669	3.388852	34.87591
3.096015	3.237085	3.454842	34.58713
3.31114	3.304276	3.386686	35.05547
3.218466	3.375646	3.407984	35.05546
3.369788	3.281632	3.412281	35.19018
3.233768	3.259797	3.422444	34.86719
3.432726	3.304007	3.378996	35.30395
3.271794	3.348891	3.425172	35.15115
3.341743	3.416083	3.338601	35.26174
3.126865	3.426124	3.409604	34.96907
3.269278	3.38899	3.355857	35.08176
3.171119	3.32874	3.41876	34.87291
3.247419	3.298481	3.374682	34.8772
3.269856	3.27496	3.392024	34.91275
3.275094	3.319777	3.374495	34.98388
3.351353	3.399369	3.452523	35.49533
3.389379	3.446208	3.367862	35.49578
3.293612	3.316879	3.401572	35.07725
3.361292	3.27678	3.32866	34.97812
3.325782	3.44951	3.401948	35.43847
3.248533	3.48907	3.441592	35.44274
3.277898	3.402132	3.448123	35.33112
3.210754	3.255079	3.382935	34.72015
3.204856	3.205882	3.366242	34.56316
3.265401	3.320384	3.433067	35.0921
3.263257	3.466763	3.413423	35.36456
3.163489	3.345387	3.433288	34.92439
3.381502	3.311285	3.358176	35.16232
3.261401	3.260471	3.430304	34.94629
3.425261	3.372411	3.339368	35.35056
3.271423	3.34195	3.452113	35.19408
3.25872	3.434346	3.344825	35.13373
3.254513	3.307444	3.420704	35.01296
3.330483	3.287765	3.346394	34.97355
3.237645	3.277858	3.404608	34.87617
3.222632	3.300502	3.353129	34.78028
3.19636	3.331032	3.489064	35.08686
3.308047	3.43684	3.410507	35.39069
3.42757	3.425652	3.398009	35.60028
3.383522	3.30677	3.414907	35.28093
3.264659	3.316205	3.36515	34.93281
3.322688	3.376522	3.393746	35.25415
3.278104	3.471682	3.374341	35.32232
3.331185	3.403682	3.394189	35.3331
3.143156	3.238837	3.31952	34.39813
3.123813	3.155067	3.418078	34.38817
3.255091	3.288506	3.374972	34.8728

3.287755	3.384408	3.454978	35.32891
3.325493	3.414263	3.422972	35.40673
3.133752	3.249485	3.405921	34.5898
3.317409	3.280554	3.419494	35.08905
3.434499	3.413387	3.400464	35.59397
3.410413	3.450319	3.364417	35.54324
3.186503	3.305355	3.395911	34.80544
3.229313	3.311555	3.385885	34.89069
3.235087	3.324629	3.394837	34.95149
3.350569	3.313307	3.380002	35.14683
3.263834	3.285069	3.400413	34.94004
3.125091	3.315666	3.495475	34.91142
3.204774	3.359202	3.372551	34.91207
3.344011	3.378207	3.390796	35.29802
3.409918	3.391821	3.357648	35.39943
3.362447	3.328134	3.432385	35.31978
3.195494	3.325708	3.478628	35.0505
3.308789	3.504773	3.328046	35.36055
3.344011	3.446073	3.397821	35.46179
3.401546	3.338647	3.318958	35.18023
3.165675	3.166322	3.448055	34.56988
3.198546	3.245913	3.341756	34.58336
3.171201	3.372007	3.437108	35.00783
3.314027	3.460495	3.372789	35.37302
3.242346	3.284058	3.518256	35.14854
3.157344	3.290663	3.460895	34.85165
3.285611	3.304479	3.457843	35.15569
3.422662	3.382453	3.356352	35.40398
3.352342	3.380903	3.380633	35.29991
3.251214	3.404423	3.410695	35.19593
3.192071	3.364189	3.488655	35.14909
3.207743	3.288574	3.285144	34.57296
3.424271	3.304479	3.334526	35.18925
3.24746	3.226639	3.360785	34.68979
3.216858	3.378814	3.393661	35.02754
3.127525	3.411567	3.435386	34.99506
3.423322	3.443579	3.356556	35.53954
3.347888	3.361561	3.387181	35.26219
3.296499	3.34815	3.402988	35.15505
3.182172	3.36082	3.411803	34.95202
3.381419	3.501336	3.382816	35.63163
3.257606	3.357854	3.404898	35.09539
3.379687	3.212756	3.249541	34.70532
3.103438	3.155471	3.423961	34.35736
3.136722	3.329819	3.398077	34.75481
3.202464	3.480241	3.430833	35.29916
3.334814	3.440142	3.363377	35.35338
3.263875	3.233109	3.42686	34.88433
3.27662	3.277589	3.434908	35.02707
3.21162	3.324023	3.467715	35.05822

3.377253	3.390069	3.364809	35.33982
3.416229	3.428955	3.418965	35.62852
3.378367	3.320788	3.421591	35.31492
3.268866	3.222124	3.377973	34.76431
3.280084	3.234794	3.336146	34.72508
3.242016	3.325168	3.344944	34.85871
3.294684	3.497629	3.398895	35.46901
3.194669	3.476265	3.469779	35.35859
3.250636	3.372277	3.372909	35.04174
3.344506	3.356102	3.434584	35.34652
3.37284	3.344713	3.395775	35.2987
3.290519	3.386901	3.367486	35.14907
3.231004	3.468111	3.433834	35.34161
3.302933	3.345589	3.42183	35.20472
3.219126	3.148058	3.361467	34.45748
3.356962	3.136668	3.345422	34.6989
3.203	3.306972	3.455234	34.97478
3.284167	3.498842	3.425428	35.50669
3.157055	3.441625	3.432657	35.1194
3.322317	3.301648	3.40442	35.11295
3.219332	3.271254	3.390165	34.79009
3.288539	3.390271	3.417192	35.26081
3.279218	3.427472	3.45757	35.41008
3.33502	3.402199	3.375859	35.29816
3.324586	3.386766	3.344194	35.17234
3.430787	3.259123	3.343853	35.12471
3.2534	3.203186	3.342693	34.61193
3.212404	3.246048	3.339283	34.60855
3.161097	3.296863	3.38638	34.71047
3.228983	3.425316	3.41622	35.20509
3.285693	3.450858	3.360853	35.26388
3.235954	3.430909	3.411837	35.22298
3.268453	3.347678	3.421284	35.13269
3.358653	3.423833	3.525759	35.72495
3.311264	3.442905	3.38418	35.35342
3.28458	3.447016	3.380139	35.29522
3.331226	3.259729	3.405375	35.04285
3.20432	3.134983	3.398281	34.47701
3.326235	3.205477	3.346803	34.78521
3.322688	3.346869	3.317525	35.02262
3.198793	3.475793	3.504274	35.44201
3.176728	3.408669	3.397395	35.01324
3.263215	3.251709	3.428838	34.92789
3.28128	3.293291	3.370112	34.9299
3.354817	3.359337	3.442957	35.39445
3.187616	3.381038	3.374495	34.92655
3.242058	3.417161	3.416561	35.21659
3.326235	3.316609	3.3873	35.1168
3.438747	3.223471	3.324636	35.02213
3.276083	3.205342	3.358057	34.69985

3.266474	3.288304	3.378911	34.90586
3.156972	3.374231	3.436682	34.98064
3.366035	3.441018	3.339351	35.37104
3.227705	3.365268	3.374085	34.97883
3.320544	3.348621	3.374563	35.1465
3.226096	3.407726	3.481306	35.30264
3.281569	3.464741	3.392007	35.35335
3.374201	3.433066	3.430952	35.57182
3.424931	3.341815	3.381571	35.37522
3.200196	3.123998	3.438267	34.53141
3.27233	3.179261	3.320612	34.55272
3.268866	3.328066	3.391887	35.02642
3.321369	3.486374	3.385595	35.47367
3.221436	3.448162	3.433561	35.27646
3.10649	3.332447	3.450476	34.80904
3.146084	3.225965	3.394206	34.53971
3.338031	3.346465	3.422631	35.28514
3.284786	3.435088	3.426246	35.37041
3.271588	3.44021	3.415674	35.32963
3.311388	3.297133	3.391324	35.05053
3.3045	3.184383	3.387999	34.78163
3.420765	3.204264	3.315138	34.92003
3.301242	3.292685	3.392689	35.0216
3.257978	3.454093	3.434635	35.37169
3.244532	3.457597	3.429622	35.33899
3.338732	3.413656	3.352515	35.28028
3.293571	3.340534	3.346206	35.00782
3.31048	3.412241	3.410218	35.34159
3.067557	3.429561	3.435471	34.90346
3.260741	3.437177	3.485534	35.45205
3.235294	3.385486	3.325267	34.93288
3.365128	3.164839	3.363496	34.81789
3.227169	3.178115	3.392433	34.60851
3.301159	3.3497	3.412639	35.18973
3.25806	3.39573	3.4412	35.2586
3.336959	3.422552	3.397054	35.39325
3.125669	3.304344	3.406688	34.69376
3.27101	3.327258	3.420056	35.09095
3.194009	3.349026	3.442769	35.01983
3.286683	3.432661	3.459616	35.44223
3.288415	3.425113	3.441337	35.38954
3.36521	3.314318	3.408734	35.24389
3.307305	3.204466	3.373557	34.8001
3.309078	3.151495	3.32941	34.5916
3.289488	3.259594	3.37076	34.87558
3.296705	3.388923	3.256089	34.92342
3.18906	3.391888	3.413986	35.03979
3.174707	3.393843	3.3607	34.89615
3.349084	3.382453	3.385203	35.30616
3.270268	3.404356	3.347945	35.10023

3.284703	3.444523	3.491605	35.53379
3.223952	3.413589	3.442138	35.22512
3.240532	3.376118	3.472848	35.24659
3.19801	3.264986	3.400328	34.75198
3.415486	3.215856	3.381042	35.07796
3.204444	3.229941	3.403823	34.69706
3.281775	3.39701	3.393985	35.21001
3.285322	3.398628	3.469438	35.38631
3.313904	3.378207	3.373403	35.19414
3.281528	3.328066	3.379337	35.02667
3.285734	3.289585	3.347314	34.88168
3.27233	3.318766	3.500813	35.25186
3.290684	3.34323	3.416424	35.16095
3.271835	3.361426	3.468398	35.27318
3.435695	3.408602	3.407916	35.60242
3.327349	3.237759	3.456257	35.09759
3.312914	3.231289	3.324533	34.76382
3.315058	3.21653	3.390966	34.88151
3.295963	3.344578	3.402527	35.14506
3.246099	3.389529	3.398623	35.12577
3.237315	3.428146	3.382611	35.156
3.209682	3.345926	3.403806	34.96212
3.285817	3.396875	3.383072	35.19469
3.31943	3.427	3.409757	35.39243
3.281981	3.40476	3.414992	35.27335
3.269072	3.340602	3.392433	35.05548
3.187328	3.258988	3.428633	34.77741
3.329452	3.224752	3.351304	34.84423
3.368922	3.312701	3.404642	35.23952
3.258184	3.404828	3.428378	35.25073
3.27101	3.354889	3.367998	35.03753
3.357168	3.39391	3.468074	35.53012
3.200237	3.305894	3.391273	34.82651
3.295303	3.27988	3.356352	34.90115
3.132185	3.406513	3.478083	35.08757
3.262556	3.431111	3.41639	35.29155
3.261607	3.330627	3.442752	35.12738
3.390823	3.247531	3.374341	35.07863
3.344424	3.165513	3.404898	34.86463
3.287178	3.273747	3.370385	34.90066
3.226179	3.382453	3.494879	35.27723
3.387853	3.458878	3.297882	35.36712
3.280496	3.412578	3.39673	35.24726
3.339887	3.410152	3.296057	35.15168
3.128308	3.358933	3.452642	34.91941
3.240037	3.46373	3.39528	35.26747
3.300664	3.454699	3.463555	35.52961
3.319307	3.243622	3.352532	34.866
3.263463	3.161537	3.440297	34.7563
3.225313	3.197592	3.355192	34.56561

3.253317	3.293224	3.407626	34.95064
3.409382	3.469795	3.354749	35.56243
3.245976	3.432998	3.386021	35.19301
3.189431	3.312835	3.451346	34.94943
3.30219	3.362302	3.39216	35.17476
3.235252	3.341141	3.432794	35.07096
3.305036	3.394314	3.334185	35.12421
3.134082	3.333323	3.44843	34.86682
3.226756	3.342556	3.448021	35.08878
3.149219	3.263436	3.408086	34.65886
3.481104	3.277791	3.360717	35.31244
3.284497	3.245913	3.370163	34.83344
3.389668	3.303737	3.336094	35.11538
3.251874	3.358394	3.457433	35.19892
3.426086	3.461641	3.33028	35.52762
3.345537	3.437851	3.355806	35.35527
3.3443	3.379218	3.323186	35.15301
3.201062	3.396943	3.429401	35.1108
3.2355	3.510501	3.386533	35.3407
3.251874	3.490485	3.447543	35.46616
3.322977	3.256764	3.333758	34.86171
3.252492	3.136534	3.413491	34.61901
3.201268	3.235265	3.46393	34.8332
3.27996	3.360887	3.388255	35.11452
3.319142	3.421137	3.401624	35.36119
3.298107	3.336154	3.406671	35.14038
3.230592	3.347071	3.484648	35.18714
3.265938	3.365066	3.394956	35.10764
3.281528	3.352126	3.38389	35.08924
3.323142	3.431785	3.343665	35.26648
3.219704	3.400447	3.449931	35.20413
3.294107	3.389934	3.476872	35.40276
3.20201	3.243892	3.379252	34.66851
3.43949	3.229469	3.342301	35.0755
3.337082	3.217271	3.437773	35.03365
3.281899	3.296796	3.402169	35.00902
3.21958	3.432459	3.436971	35.24552
3.386657	3.459686	3.371698	35.52769
3.336051	3.402402	3.366685	35.28079
3.375191	3.387777	3.354834	35.30849
3.197556	3.409343	3.436187	35.1451
3.239377	3.46036	3.384777	35.23569
3.208238	3.337906	3.404642	34.94325
3.285611	3.247868	3.414054	34.93613
3.208692	3.189101	3.397463	34.60313
3.354487	3.238972	3.45346	35.15348
3.273114	3.436907	3.425496	35.34723
3.368922	3.484015	3.379252	35.55863
3.321781	3.351317	3.36099	35.12542
3.290024	3.265727	3.369533	34.88748

3.193143	3.317283	3.440262	34.94304
3.25938	3.372277	3.431242	35.18842
3.230097	3.423765	3.382594	35.1306
3.318523	3.413791	3.421762	35.38781
3.349538	3.290056	3.43883	35.22237
3.179945	3.170096	3.410968	34.52824
3.26602	3.204399	3.351731	34.66194
3.307552	3.254877	3.394138	34.95589
3.357292	3.459754	3.400873	35.52743
3.344424	3.421339	3.37313	35.35461
3.35259	3.414196	3.410831	35.43929
3.284126	3.356507	3.290925	34.9012
3.347187	3.418172	3.427832	35.47335
3.24911	3.462247	3.356761	35.19984
3.298314	3.386025	3.427423	35.29528
3.129958	3.207162	3.424797	34.53022
3.295179	3.135051	3.346922	34.56354
3.24016	3.244094	3.375842	34.74492
3.315677	3.424776	3.395536	35.34826
3.31081	3.450656	3.455046	35.52435
3.3337	3.355293	3.365287	35.16958
3.317162	3.262762	3.406262	35.02066
3.438088	3.371266	3.34578	35.39012
3.217683	3.351048	3.43779	35.06513
3.193267	3.402132	3.398827	35.03825
3.114327	3.350643	3.401692	34.75928
3.356838	3.347139	3.357426	35.18515
3.324421	3.268558	3.462753	35.17275
3.290271	3.237018	3.407694	34.90869
3.219621	3.216867	3.434226	34.76814
3.329329	3.342489	3.42686	35.26666
3.394287	3.478489	3.440314	35.73555
3.374366	3.439131	3.300218	35.29955
3.32772	3.327056	3.380855	35.12879
3.211661	3.347948	3.369226	34.89524
3.305737	3.428079	3.318992	35.16635
3.350033	3.441423	3.393865	35.45614
3.261731	3.351317	3.405239	35.09086
3.143321	3.140038	3.462958	34.49611
3.214425	3.196783	3.357494	34.54506
3.316914	3.34943	3.437295	35.27752
3.342362	3.463528	3.40263	35.50687
3.24115	3.388384	3.448123	35.2207
3.261896	3.335008	3.430952	35.11179
3.268247	3.316138	3.403346	35.02404
3.424436	3.407591	3.390779	35.53811
3.249894	3.354552	3.441763	35.15192
3.14097	3.346061	3.419323	34.84608
3.170212	3.362168	3.441882	34.99459
3.318275	3.32254	3.33473	34.9974

3.312914	3.208982	3.435181	34.95701
3.30384	3.224684	3.359813	34.80669
3.178089	3.256023	3.449521	34.7964
3.327184	3.402334	3.333707	35.18914
3.423982	3.415476	3.357358	35.48128
3.469515	3.387845	3.303731	35.40315
3.316131	3.380903	3.379525	35.21829
3.24911	3.364257	3.410149	35.1023
3.174418	3.465887	3.350656	35.03111
3.375356	3.432122	3.392484	35.48816
3.188276	3.30677	3.418283	34.86134
3.103438	3.139297	3.420329	34.31405
3.265896	3.260538	3.425905	34.94665
3.390864	3.406108	3.438336	35.56545
3.330896	3.437244	3.419801	35.46187
3.239542	3.335345	3.386874	34.96725
3.249564	3.323753	3.418965	35.03399
3.276743	3.367761	3.396576	35.14071
3.437263	3.369244	3.356556	35.40746
3.25971	3.338647	3.409808	35.06873
3.211537	3.337839	3.386499	34.91064
3.126782	3.355765	3.392092	34.77673
3.341784	3.267412	3.392245	35.05402
3.27167	3.282508	3.377751	34.90201
3.249358	3.295246	3.445139	35.02844
3.184606	3.30805	3.399458	34.81494
3.44114	3.427876	3.40251	35.64466
3.415239	3.39364	3.321413	35.3358
3.479785	3.405165	3.327705	35.51591
3.276867	3.391416	3.423228	35.25099
3.16951	3.373422	3.403397	34.9335
3.256617	3.384071	3.41593	35.17468
3.35424	3.299896	3.349258	35.05829
3.225313	3.204062	3.393456	34.66343
3.168191	3.27961	3.405034	34.72905
3.173676	3.413791	3.495254	35.23177
3.447821	3.466291	3.442138	35.82993
3.447409	3.42262	3.422239	35.69001
3.269402	3.321731	3.412878	35.05964
3.281651	3.301783	3.433817	35.0886
3.30747	3.398291	3.378314	35.23473
3.313285	3.380027	3.382952	35.21765
3.309037	3.327258	3.337459	34.99347
3.140104	3.312431	3.473871	34.88993
3.141795	3.227313	3.392126	34.52873
3.3118	3.253731	3.460434	35.10766
3.261153	3.319238	3.406603	35.02243
3.298314	3.37322	3.436017	35.28607
3.247749	3.447892	3.427337	35.31981
3.36719	3.43064	3.423859	35.53567

3.359519	3.390001	3.321771	35.20677
3.477228	3.414465	3.348559	35.57626
3.234634	3.356102	3.412298	35.05751
3.313079	3.359202	3.326887	35.04905
3.206135	3.231626	3.347758	34.58184
3.331515	3.23257	3.333093	34.82602
3.143568	3.242072	3.370453	34.51749
3.213847	3.421676	3.371954	35.06722
3.188812	3.459012	3.541259	35.46437
3.376222	3.457665	3.368475	35.49341
3.290271	3.320451	3.468756	35.22468
3.421961	3.380027	3.392552	35.4763
3.302067	3.347274	3.45375	35.27631
3.315925	3.379083	3.484	35.44233
3.190792	3.287024	3.420858	34.82929
3.284332	3.231491	3.388392	34.84141
3.220776	3.256225	3.355584	34.68476
3.235252	3.243757	3.408598	34.80509
3.190421	3.359876	3.405324	34.95382
3.271588	3.365066	3.48533	35.31763
3.3549	3.424574	3.37695	35.39295
3.379233	3.372816	3.38568	35.35206
3.41462	3.389799	3.348508	35.3853
3.375975	3.399504	3.39291	35.41911
3.339516	3.394584	3.347962	35.23033
3.34529	3.351722	3.356437	35.16776
3.370902	3.27523	3.354459	35.05216
3.158333	3.219428	3.396969	34.55824
3.239995	3.238702	3.449368	34.89356
3.223209	3.361965	3.378877	34.97226
3.283796	3.460158	3.470717	35.52032
3.282682	3.411163	3.440109	35.34381
3.285652	3.337973	3.460758	35.23541
3.230963	3.342556	3.313723	34.80429
3.462091	3.442905	3.458661	35.84613
3.274846	3.449847	3.375177	35.26927
3.314069	3.342489	3.422785	35.22438
3.22024	3.164098	3.366037	34.50498
3.310398	3.258112	3.352941	34.8791
3.233231	3.267682	3.314609	34.64745
3.294973	3.327999	3.414054	35.13184
3.207289	3.376657	3.450067	35.12525
3.255338	3.418846	3.417311	35.25096
3.327102	3.45598	3.513226	35.69885
3.520987	3.493114	3.305129	35.74898
3.362777	3.432864	3.455046	35.59908
3.365417	3.387575	3.381162	35.34424
3.308377	3.329145	3.397463	35.12738
3.356879	3.301513	3.302588	34.96554
3.193679	3.243016	3.414873	34.72627

3.162788	3.255686	3.355533	34.55666
3.137052	3.290191	3.401351	34.67603
3.214837	3.438592	3.386567	35.13834
3.330112	3.439064	3.386499	35.39131
3.263133	3.350643	3.46526	35.22371
3.330195	3.372209	3.433732	35.34858
3.212074	3.389058	3.408785	35.07256
3.428065	3.469054	3.374614	35.64511
3.339722	3.377129	3.387761	35.27964
3.279548	3.199142	3.309835	34.58841
3.208815	3.207095	3.444201	34.74496
3.274227	3.217541	3.348764	34.70214
3.238428	3.26229	3.393848	34.82031
3.288786	3.386497	3.410098	35.23759
3.164355	3.319642	3.495612	35.00628
3.291591	3.407658	3.36336	35.18779
3.346898	3.372277	3.415828	35.3461
3.443697	3.453217	3.361535	35.61606
3.234675	3.373827	3.394138	35.05665
3.360467	3.414667	3.386976	35.40538
3.271918	3.33548	3.375637	35.01377
3.370325	3.302457	3.322266	35.04004
3.13998	3.296931	3.356403	34.59888
3.265113	3.271388	3.37828	34.86451
3.128927	3.321394	3.38273	34.68578
3.243707	3.421541	3.40546	35.2055
3.273568	3.420396	3.443417	35.3513
3.361127	3.365066	3.383617	35.29101
3.293406	3.366009	3.400549	35.182
3.302314	3.439064	3.436	35.43877
3.344011	3.421272	3.383395	35.37601
3.362365	3.283789	3.385868	35.1209
3.284126	3.220506	3.351628	34.73654
3.167654	3.251372	3.400601	34.65643
3.248285	3.319777	3.387914	34.9546
3.207124	3.349026	3.332326	34.80699
3.256658	3.347611	3.419852	35.10362
3.315636	3.348082	3.38701	35.16181
3.238923	3.396269	3.452028	35.24161
3.245728	3.4857	3.376797	35.28754
3.43186	3.444658	3.397412	35.64991
3.316997	3.37942	3.369174	35.19431
3.350322	3.292011	3.352481	35.03953
3.312254	3.227515	3.356829	34.82475
3.371562	3.286552	3.345353	35.05845
3.229231	3.30185	3.359489	34.81157
3.307511	3.389732	3.448754	35.37014
3.142496	3.404626	3.44161	35.02623
3.224282	3.411904	3.383003	35.09284
3.261978	3.356304	3.451158	35.20273

3.322688	3.408198	3.424132	35.38987
3.341413	3.396741	3.485534	35.54004
3.324049	3.371872	3.458917	35.38948
3.262143	3.333391	3.447748	35.14552
3.350569	3.208308	3.33942	34.82846
3.287384	3.143205	3.414037	34.7111
3.255709	3.236411	3.34624	34.69739
3.127277	3.288506	3.410831	34.67171
3.210754	3.458743	3.309886	35.00579
3.273114	3.42545	3.392365	35.24972
3.33964	3.437109	3.371886	35.37591
3.270598	3.456384	3.456956	35.45312
3.314522	3.412376	3.373318	35.27003
3.292086	3.378005	3.405886	35.21702
3.378532	3.365066	3.329547	35.21083
3.32871	3.268827	3.368561	34.97673
3.243254	3.229335	3.403073	34.77896
3.32706	3.286013	3.366975	35.00724
3.182791	3.339456	3.341756	34.75347
3.329081	3.343432	3.402851	35.21568
3.199536	3.38151	3.444321	35.10634
3.219827	3.41123	3.414532	35.15057
3.235211	3.416083	3.443792	35.25881
3.346321	3.436166	3.376132	35.39775
3.274227	3.384812	3.488399	35.3733
3.303428	3.355833	3.38152	35.14005
3.251997	3.226639	3.462123	34.92132
3.335556	3.224348	3.337203	34.82586
3.327638	3.214643	3.371476	34.86227
3.347517	3.209656	3.332087	34.8087
3.210465	3.294235	3.414532	34.87424
3.252657	3.419924	3.370044	35.14409
3.210011	3.410758	3.439717	35.18315
3.383358	3.496079	3.33901	35.52858
3.286271	3.445197	3.419647	35.38134
3.268783	3.405974	3.45994	35.34543
3.286147	3.407928	3.451022	35.36818
3.343516	3.329886	3.385049	35.1787
3.345702	3.249822	3.350281	34.93236
3.25542	3.250564	3.377001	34.79498
3.315058	3.280419	3.390796	35.02085
3.218466	3.346532	3.3644	34.89648
3.30582	3.360146	3.360904	35.10963
3.239006	3.34505	3.403056	35.02269
3.261153	3.431044	3.405051	35.26354
3.242264	3.431785	3.479532	35.38673
3.375645	3.438794	3.40205	35.5243
3.347476	3.380499	3.408052	35.34834
3.278476	3.302726	3.372431	34.94948
3.2534	3.230683	3.432657	34.86879

3.349744	3.205342	3.410576	34.97578
3.277939	3.271186	3.364588	34.86218
3.389956	3.290596	3.325659	35.06445
3.148641	3.320316	3.411599	34.78967
3.303881	3.370187	3.391052	35.19328
3.187039	3.333323	3.396662	34.86942
3.301654	3.475052	3.398281	35.43354
3.238799	3.460091	3.49036	35.46473
3.337866	3.433672	3.383685	35.39032
3.248244	3.323147	3.42906	35.05186
3.39305	3.267614	3.373949	35.12657
3.393916	3.288843	3.354016	35.13129
3.287302	3.34296	3.382816	35.07947
3.276372	3.419385	3.397497	35.2548
3.218178	3.374366	3.402851	35.0408
3.308006	3.347139	3.331047	35.02068
3.32574	3.401728	3.339778	35.19793
3.278558	3.436705	3.439222	35.38871
3.245481	3.441625	3.466488	35.38676
3.266309	3.344915	3.415623	35.10958
3.337495	3.332514	3.378706	35.15741
3.329494	3.26229	3.387522	35.00562
3.249894	3.18317	3.456137	34.80857
3.262102	3.253933	3.40292	34.87364
3.244491	3.285541	3.364673	34.82061
3.368056	3.401526	3.396389	35.41382
3.291838	3.459552	3.404164	35.39104
3.243006	3.401458	3.3858	35.11706
3.207991	3.376186	3.395025	35.00539
3.33634	3.47303	3.431907	35.57851
3.23451	3.437042	3.438796	35.29219
3.35358	3.371064	3.368032	35.25354
3.246429	3.265121	3.40546	34.86939
3.351806	3.271051	3.390728	35.08058
3.410826	3.312835	3.319844	35.14601
3.373294	3.359202	3.351987	35.23562
3.260081	3.372883	3.440518	35.21157
3.181595	3.375444	3.393388	34.94246
3.200855	3.367761	3.38227	34.94347
3.344218	3.43192	3.369038	35.36835
3.262473	3.47667	3.467119	35.50193
3.177223	3.40503	3.44843	35.11797
3.254018	3.304613	3.523031	35.22945
3.354116	3.270445	3.380411	35.06175
3.290766	3.231222	3.409126	34.90023
3.279342	3.217406	3.366719	34.7523
3.215909	3.185057	3.43438	34.6908
3.217971	3.292483	3.349599	34.74483
3.371768	3.37349	3.405716	35.38103
3.313945	3.453351	3.372159	35.35584

3.277527	3.414061	3.40558	35.26336
3.112801	3.405569	3.428429	34.93453
3.329452	3.480646	3.391393	35.4915
3.338072	3.415004	3.405801	35.39831
3.306562	3.338715	3.389415	35.12674
3.217641	3.190381	3.366054	34.55681
3.328174	3.219967	3.371886	34.87598
3.333865	3.336019	3.366514	35.13047
3.463782	3.421339	3.390318	35.65322
3.18308	3.321057	3.420329	34.88569
3.228282	3.305422	3.399799	34.90546
3.176316	3.333593	3.397275	34.8479
3.331762	3.502414	3.361058	35.47782
3.218466	3.431381	3.466846	35.30606
3.201845	3.397954	3.448839	35.15724
3.119936	3.282037	3.484426	34.80244
3.384141	3.33676	3.354919	35.21668
3.349785	3.238702	3.420176	35.06982
3.334855	3.232907	3.346189	34.8627
3.257483	3.241937	3.383361	34.79453
3.255544	3.350576	3.391802	35.04633
3.364963	3.379825	3.347826	35.2534
3.360261	3.402873	3.347059	35.29185
3.221188	3.352867	3.4242	35.04706
3.179038	3.463865	3.455779	35.26667
3.340753	3.466965	3.426724	35.56356
3.305325	3.36055	3.348815	35.083
3.295179	3.279273	3.396048	34.98636
3.219456	3.206286	3.424967	34.7244
3.248038	3.343769	3.417396	35.071
3.351765	3.419857	3.341807	35.29892
3.418456	3.375714	3.363172	35.39495
3.265319	3.277386	3.439768	35.01254
3.139733	3.29282	3.331934	34.53584
3.202918	3.401054	3.44045	35.14801
3.350198	3.504368	3.340187	35.47677
3.197845	3.375107	3.364042	34.91309
3.277073	3.281902	3.468278	35.11047
3.191039	3.290528	3.501904	35.01473
3.320008	3.298413	3.377257	35.04142
3.330277	3.274354	3.429656	35.12585
3.422539	3.237354	3.415197	35.21508
3.257276	3.289989	3.481561	35.11391
3.344382	3.405367	3.3393	35.24561
3.3264	3.385688	3.375091	35.24152
3.391812	3.384879	3.326205	35.27589
3.21162	3.382857	3.399441	35.03757
3.107645	3.475861	3.44959	35.12325
3.22721	3.353811	3.383668	34.97365
3.383729	3.261279	3.366156	35.07529

3.280826	3.239309	3.384453	34.84222
3.301324	3.285137	3.388255	34.99558
3.276578	3.392562	3.424609	35.25589
3.295921	3.394382	3.320475	35.07444
3.372428	3.325775	3.337066	35.128
3.25806	3.324427	3.382662	34.97466
3.139485	3.415341	3.4626	35.08898
3.280496	3.519734	3.356147	35.39285
3.288621	3.415746	3.406824	35.29403
3.2063	3.289315	3.321618	34.65119
3.278022	3.20204	3.452028	34.90236
3.172315	3.273006	3.470307	34.86636
3.245316	3.293089	3.392416	34.89959
3.250801	3.26876	3.41726	34.91271
3.342197	3.25299	3.408598	35.05915
3.295179	3.367424	3.385749	35.15661
3.31477	3.402267	3.417652	35.34541
3.319018	3.38306	3.365901	35.19953
3.284951	3.403952	3.313024	35.05508
3.168108	3.41433	3.415282	35.04589
3.29023	3.473906	3.371016	35.34643
3.297324	3.323349	3.423757	35.14803
3.250595	3.213565	3.392774	34.73801
3.313821	3.251372	3.413099	35.0034
3.340794	3.384003	3.414856	35.35627
3.338031	3.436907	3.417857	35.47248
3.370201	3.402536	3.341619	35.30095
3.249688	3.265121	3.440655	34.95348
3.354693	3.352261	3.37088	35.22108
3.261648	3.487587	3.389483	35.35423
3.188977	3.46993	3.392433	35.16315
3.136516	3.318025	3.451823	34.84611
3.181883	3.245105	3.386891	34.64386
3.262968	3.300233	3.470785	35.12519
3.285446	3.347813	3.420329	35.16806
3.228571	3.272669	3.449487	34.94312
3.241934	3.266401	3.393013	34.83514
3.398618	3.285743	3.379133	35.18972
3.322647	3.416217	3.38077	35.31249
3.273733	3.383599	3.410507	35.19922
3.259957	3.364729	3.410166	35.12709
3.26635	3.338243	3.35156	34.95499
3.26602	3.458473	3.426792	35.38171
3.352672	3.416891	3.321652	35.25035
3.187121	3.222124	3.430731	34.70092
3.235459	3.204466	3.370931	34.63724
3.1486	3.343971	3.461185	34.94974
3.406371	3.466089	3.399185	35.64492
3.333206	3.416083	3.341056	35.24844
3.394328	3.328201	3.403738	35.327

3.192524	3.292348	3.42096	34.84494
3.348301	3.463258	3.382543	35.47534
3.223457	3.486374	3.397599	35.28581
3.261236	3.366413	3.349531	35.00097
3.040047	3.213497	3.470597	34.44761
3.178625	3.234928	3.361331	34.55858
3.340011	3.339052	3.403823	35.23213
3.316007	3.277454	3.386823	35.00776
3.211579	3.266401	3.444662	34.8817
3.237851	3.290596	3.455319	35.01537
3.325369	3.399099	3.335327	35.18164
3.476196	3.407793	3.313979	35.4838
3.243584	3.372748	3.370897	35.02294
3.169016	3.323551	3.390745	34.79569
3.242346	3.427876	3.404488	35.21425
3.260865	3.462854	3.385595	35.28992
3.354322	3.344645	3.325932	35.10532
3.209764	3.199681	3.420397	34.67877
3.19038	3.280554	3.453068	34.88468
3.318729	3.423024	3.457485	35.48657
3.412104	3.480983	3.297422	35.46748
3.263463	3.336086	3.395025	35.03901
3.36121	3.267277	3.344177	34.99109
3.27864	3.307511	3.444321	35.11751
3.378944	3.515219	3.319657	35.51846
3.336423	3.44331	3.385885	35.41305
3.208609	3.272736	3.394956	34.78036
3.101088	3.20824	3.406961	34.43044
3.220116	3.267547	3.418539	34.84575
3.284662	3.444051	3.4242	35.38527
3.259174	3.336221	3.455541	35.16226
3.170871	3.247733	3.431788	34.7237
3.330236	3.283991	3.423927	35.13431
3.334113	3.343432	3.398947	35.21815
3.319224	3.408467	3.41078	35.35369
3.204485	3.41305	3.451499	35.20184
3.268247	3.391619	3.359063	35.09227
3.168356	3.365605	3.426127	34.96359
3.293323	3.342286	3.337237	34.99149
3.289817	3.301783	3.393985	35.01935
3.234716	3.375242	3.369857	35.00673
3.19537	3.41797	3.50898	35.31837
3.33403	3.455036	3.388016	35.43812
3.436025	3.359944	3.454006	35.59753
3.39272	3.329347	3.384777	35.28452
3.304088	3.307174	3.429725	35.1405
3.298437	3.495135	3.387096	35.44597
3.304376	3.465482	3.44236	35.51496
3.196937	3.252316	3.345234	34.60145
3.291632	3.232974	3.384572	34.85226

3.105624	3.255281	3.425649	34.5841
3.201515	3.392427	3.391478	35.01899
3.216404	3.324764	3.438063	35.00545
3.22391	3.285069	3.402613	34.85754
3.176316	3.269299	3.458525	34.84124
3.381502	3.412646	3.410763	35.49898
3.322812	3.416487	3.446964	35.4582
3.38278	3.369311	3.348559	35.27098
3.223828	3.338715	3.446111	35.0698
3.360591	3.372614	3.382952	35.30489
3.221972	3.293426	3.385578	34.83432
3.269814	3.278128	3.307857	34.73553
3.16254	3.283721	3.440927	34.80417
3.272825	3.337973	3.309903	34.87746
3.229148	3.397819	3.443229	35.20438
3.359601	3.440614	3.386362	35.45889
3.349002	3.361696	3.445429	35.3923
3.381873	3.385486	3.369925	35.35109
3.31147	3.437851	3.367879	35.30717
3.302562	3.414263	3.421949	35.35435
3.258596	3.355496	3.357494	34.98874
3.163159	3.19429	3.361774	34.43686
3.233644	3.254338	3.343597	34.68256
3.255379	3.320855	3.430475	35.06554
3.191328	3.38616	3.442411	35.09439
3.28161	3.262695	3.444389	35.02615
3.305696	3.306433	3.376046	35.02501
3.2534	3.293493	3.395076	34.92397
3.38278	3.321057	3.338413	35.14327
3.296169	3.417902	3.409348	35.32077
3.276125	3.425518	3.360802	35.18743
3.256781	3.342152	3.377427	34.99918
3.448605	3.34788	3.344177	35.35848
3.218178	3.205006	3.384333	34.62994
3.256493	3.256899	3.322862	34.69279
3.097252	3.312229	3.442002	34.72609
3.274722	3.41123	3.413781	35.26897
3.246966	3.442097	3.415197	35.27887
3.327968	3.404154	3.481715	35.5185
3.244986	3.339186	3.363633	34.93673
3.384554	3.408602	3.43588	35.55174
3.32805	3.524654	3.398145	35.59944
3.351146	3.438929	3.441865	35.55809
3.186915	3.220034	3.371732	34.56688
3.128102	3.159987	3.343393	34.24498
3.275753	3.295111	3.325932	34.82518
3.265773	3.435425	3.387778	35.24545
3.228983	3.408063	3.420926	35.17765
3.197721	3.327527	3.414736	34.91963
3.301242	3.281497	3.414054	35.04386

3.343145	3.337299	3.341619	35.09912
3.332752	3.353474	3.397531	35.23404
3.183286	3.432998	3.453887	35.20432
3.25839	3.412713	3.387846	35.17978
3.243872	3.308118	3.297626	34.72201
3.426622	3.226908	3.344416	35.04638
3.153302	3.262627	3.36868	34.57985
3.105336	3.318699	3.394002	34.65295
3.183863	3.437851	3.444662	35.19603
3.33036	3.439873	3.358807	35.33306
3.308624	3.350306	3.374205	35.12333
3.35725	3.360887	3.391154	35.28988
3.212239	3.435155	3.497044	35.36673
3.402453	3.480376	3.337868	35.5335
3.36055	3.443916	3.425547	35.55387
3.367644	3.333391	3.386703	35.24274
3.221271	3.119146	3.398895	34.48079
3.287714	3.235063	3.379133	34.83636
3.156436	3.408939	3.386158	34.94488
3.337866	3.497427	3.359336	35.47649
3.155446	3.298413	3.493787	34.93638
3.241934	3.297267	3.370675	34.85379
3.229643	3.328808	3.484597	35.14501
3.426416	3.415274	3.352805	35.4762
3.309491	3.423024	3.452727	35.45597
3.262597	3.420665	3.363735	35.15365
3.2063	3.309129	3.493293	35.06994
3.258225	3.286687	3.253787	34.61066
3.447202	3.244498	3.329325	35.09685
3.256245	3.253664	3.39644	34.84607
3.150167	3.393438	3.446793	35.02988
3.1738	3.420733	3.442633	35.13215
3.333618	3.417902	3.308454	35.18203
3.364551	3.351519	3.359063	35.21518
3.305325	3.367694	3.469421	35.36236
3.191823	3.433201	3.386362	35.07577
3.310439	3.452677	3.389909	35.38552
3.346197	3.373962	3.335685	35.17299
3.40468	3.180946	3.352873	34.91638
3.175903	3.163356	3.374085	34.424
3.282394	3.302255	3.395758	35.00802
3.18906	3.461843	3.459872	35.29312
3.282022	3.458878	3.318617	35.18103
3.172769	3.226167	3.407506	34.62759
3.219291	3.285069	3.422068	34.88998
3.160189	3.313981	3.46451	34.91678
3.39804	3.458675	3.347826	35.49817
3.280167	3.44924	3.451039	35.44548
3.399401	3.387575	3.395076	35.44899
3.27134	3.24342	3.346121	34.74664

3.269856	3.236007	3.333827	34.7003
3.244697	3.269299	3.321703	34.69157
3.266267	3.383397	3.37088	35.0958
3.160272	3.419789	3.385663	34.97592
3.250595	3.404558	3.375245	35.11735
3.313038	3.39209	3.459207	35.41025
3.346197	3.396067	3.328524	35.20567
3.344919	3.372951	3.412622	35.33624
3.194215	3.420261	3.4626	35.21943
3.280084	3.412713	3.382424	35.21537
3.290106	3.217743	3.378792	34.80297
3.342444	3.178318	3.311591	34.68426
3.226426	3.276375	3.412008	34.86457
3.239088	3.400245	3.392092	35.11959
3.280125	3.409815	3.469983	35.4006
3.316832	3.386632	3.333963	35.13272
3.144269	3.231087	3.403891	34.56812
3.282476	3.377803	3.40355	35.19046
3.182626	3.413859	3.491792	35.24392
3.298066	3.436166	3.370675	35.28029
3.334154	3.393034	3.419016	35.3706
3.380677	3.29875	3.415947	35.25944
3.251337	3.198738	3.367265	34.65142
3.326689	3.257707	3.357682	34.92421
3.224942	3.304411	3.4109	34.92021
3.322276	3.436233	3.375671	35.3443
3.246471	3.422081	3.444457	35.298
3.183162	3.397482	3.401913	35.01273
3.218219	3.39108	3.402323	35.07629
3.261813	3.397684	3.391938	35.16335
3.285074	3.417228	3.416782	35.31129
3.301077	3.47667	3.459616	35.56995
3.194958	3.346734	3.427542	34.98359
3.286229	3.17542	3.36307	34.66756
3.387276	3.188494	3.357716	34.90542
3.248492	3.362033	3.415606	35.10802
3.165015	3.433133	3.388255	35.02114
3.234758	3.351856	3.409058	35.04141
3.282476	3.300165	3.402664	35.01874
3.26371	3.294976	3.341875	34.83342
3.29023	3.305422	3.443383	35.13624
3.086982	3.42754	3.44814	34.96922
3.256658	3.48105	3.440092	35.43969
3.29885	3.347274	3.37782	35.10323
3.425302	3.205545	3.391069	35.0988
3.254183	3.203523	3.376268	34.6878
3.293653	3.246722	3.422495	34.96968
3.260205	3.389934	3.403329	35.1678
3.388059	3.438996	3.315837	35.36336
3.235335	3.377601	3.395366	35.06903

3.296623	3.327325	3.365901	35.02867
3.182626	3.409074	3.430816	35.10011
3.246677	3.448701	3.34728	35.14416
3.33535	3.403008	3.43941	35.43962
3.376552	3.386092	3.375432	35.35282
3.302479	3.17205	3.420602	34.82154
3.292787	3.159313	3.350486	34.61916
3.312419	3.304883	3.409416	35.1093
3.34298	3.487183	3.364127	35.47575
3.17182	3.39108	3.363292	34.88946
3.136351	3.330156	3.449641	34.8675
3.250059	3.27159	3.388392	34.85415
3.261854	3.324562	3.356812	34.92672
3.259462	3.418778	3.453324	35.33858
3.212445	3.403817	3.393439	35.07209
3.19273	3.356507	3.478833	35.11226
3.210795	3.221989	3.334781	34.54257
3.452894	3.158234	3.361058	34.99005
3.251502	3.319507	3.363309	34.90724
3.248492	3.44331	3.429264	35.31562
3.280331	3.476939	3.423381	35.44593
3.330154	3.426192	3.356676	35.29803
3.31246	3.366683	3.339368	35.09135
3.293777	3.364863	3.388664	35.15432
3.156436	3.439805	3.443997	35.13887
3.281775	3.454969	3.353623	35.24849
3.229808	3.359539	3.41651	35.06368
3.354735	3.219832	3.396934	34.98855
3.150456	3.157897	3.454773	34.53287
3.332546	3.242746	3.399509	34.99577
3.291178	3.45079	3.425632	35.41739
3.363396	3.393304	3.358824	35.3035
3.232489	3.341815	3.446094	35.09548
3.220776	3.337771	3.348031	34.84657
3.119606	3.277454	3.389295	34.58366
3.248698	3.428955	3.360444	35.13418
3.220611	3.413656	3.447936	35.23064
3.283342	3.368166	3.439734	35.2504
3.272866	3.285406	3.331235	34.80924
3.320008	3.1736	3.402613	34.82392
3.357622	3.231019	3.3393	34.89329
3.30417	3.337097	3.380497	35.09847
3.212527	3.39209	3.382253	35.02216
3.296293	3.427068	3.378297	35.27318
3.328669	3.420935	3.37342	35.31991
3.237974	3.336625	3.36116	34.91039
3.30318	3.450993	3.433953	35.46227
3.138124	3.496012	3.461867	35.26081
3.195288	3.355563	3.494316	35.14964
3.249646	3.282104	3.408734	34.92072

3.382698	3.187214	3.346513	34.86811
3.255214	3.21626	3.388136	34.74386
3.325658	3.368166	3.419903	35.29957
3.272371	3.397954	3.42183	35.2524
3.362571	3.378814	3.330229	35.20748
3.281115	3.32099	3.359711	34.96737
3.256864	3.288237	3.388119	34.90483
3.130865	3.344241	3.427695	34.83832
3.263463	3.484824	3.431634	35.44433
3.202175	3.437312	3.407097	35.15274
3.313615	3.320721	3.410081	35.148
3.279878	3.191729	3.457143	34.89506
3.335226	3.200692	3.387437	34.88326
3.254596	3.306366	3.418948	35.00694
3.30318	3.444792	3.372517	35.31436
3.306727	3.453958	3.33589	35.26207
3.29621	3.393573	3.329461	35.09296
3.180151	3.319373	3.384384	34.797
3.271505	3.394247	3.366173	35.12069
3.256493	3.49062	3.441814	35.46403
3.262556	3.426933	3.434994	35.32309
3.196195	3.289652	3.426877	34.86001
3.242346	3.186203	3.393422	34.66155
3.289941	3.207432	3.429673	34.89133
3.428065	3.388721	3.32646	35.36413
3.324833	3.482331	3.476463	35.67112
3.269897	3.341882	3.352106	34.97189
3.255668	3.313442	3.446725	35.0855
3.223993	3.331167	3.30576	34.74672
3.331803	3.308657	3.438591	35.22375
3.177347	3.45726	3.424967	35.18115
3.182956	3.426866	3.456871	35.19672
3.240532	3.265053	3.433953	34.91865
3.332752	3.220708	3.340119	34.81815
3.232242	3.216597	3.415879	34.75503
3.307387	3.324427	3.392211	35.10341
3.235087	3.42572	3.449862	35.29289
3.414661	3.489205	3.293875	35.4833
3.439902	3.393371	3.386499	35.53148
3.312089	3.361898	3.260727	34.9081
3.190875	3.361157	3.442002	35.03782
3.217477	3.505581	3.369362	35.25298
3.192442	3.453419	3.424967	35.20576
3.280208	3.290528	3.346325	34.8695
3.155941	3.148395	3.409075	34.42415
3.205268	3.232637	3.396508	34.68876
3.269278	3.418576	3.415469	35.27683
3.337289	3.516027	3.456478	35.72834
3.338031	3.396606	3.406466	35.35944
3.260535	3.259864	3.410371	34.89948

3.196855	3.321529	3.388954	34.84824
3.26305	3.413859	3.414463	35.25069
3.269856	3.398695	3.395297	35.1905
3.205516	3.350037	3.38701	34.92527
3.192153	3.311218	3.43438	34.91474
3.234593	3.225965	3.432453	34.8169
3.409093	3.214171	3.368833	35.03359
3.272413	3.231222	3.37516	34.78581
3.279259	3.359068	3.393371	35.12019
3.283301	3.403817	3.443639	35.33682
3.363602	3.448499	3.374631	35.45923
3.354487	3.397415	3.343921	35.26042
3.37614	3.420059	3.35717	35.38627
3.116471	3.413454	3.45196	35.01126
3.284497	3.506323	3.430287	35.5344
3.163489	3.379286	3.360444	34.83923
3.250636	3.201366	3.408461	34.74573
3.114698	3.159043	3.341943	34.21043
3.284621	3.25299	3.487853	35.10656
3.225643	3.414465	3.377956	35.09038
3.434912	3.409478	3.420312	35.62973
3.315553	3.341276	3.448567	35.28136
3.363478	3.367559	3.382526	35.29922
3.273856	3.371468	3.418675	35.19083
3.210465	3.321866	3.348491	34.79025
3.235912	3.333795	3.422648	35.03415
3.289034	3.380836	3.33942	35.07119
3.211991	3.353474	3.425376	35.03084
3.321492	3.315801	3.397599	35.12718
3.278063	3.205208	3.462992	34.93336
3.289405	3.276578	3.373949	34.91951
3.291962	3.313779	3.458695	35.19178
3.298479	3.366481	3.471961	35.3503
3.328298	3.414533	3.377291	35.31356
3.354405	3.345319	3.306408	35.06428
3.279135	3.379151	3.410013	35.20023
3.243254	3.433403	3.333963	35.0741
3.183616	3.434346	3.461458	35.22455
3.180069	3.273814	3.405443	34.74324
3.264288	3.268827	3.389364	34.88135
3.187245	3.239174	3.369925	34.60551
3.322647	3.354215	3.390455	35.19808
3.256988	3.410624	3.48654	35.38798
3.396349	3.388519	3.369857	35.38923
3.352466	3.344713	3.454347	35.38224
3.374449	3.33831	3.373608	35.23974
3.231994	3.301648	3.468551	35.05567
3.251049	3.344376	3.355056	34.94258
3.112388	3.376455	3.453324	34.9244
3.338278	3.358596	3.331508	35.11294

3.280043	3.212891	3.475627	34.98212
3.183451	3.271793	3.474741	34.89776
3.193762	3.290528	3.45878	34.92638
3.377418	3.421339	3.402596	35.4912
3.338278	3.424507	3.401624	35.41041
3.445181	3.421676	3.356266	35.53881
3.318523	3.367155	3.421659	35.2856
3.258349	3.356304	3.355039	34.98459
3.316997	3.386901	3.297541	35.05402
3.314893	3.402604	3.36203	35.22479
3.23253	3.340669	3.377257	34.94253
3.128473	3.181013	3.428565	34.47803
3.21661	3.218147	3.386499	34.65999
3.260617	3.40193	3.422631	35.23714
3.341702	3.495068	3.451141	35.68049
3.251214	3.415139	3.483471	35.37851
3.275382	3.36365	3.420858	35.18184
3.315636	3.334806	3.388579	35.1362
3.457266	3.373085	3.429111	35.61827
3.244615	3.351385	3.429946	35.10761
3.202505	3.294572	3.363036	34.74496
3.17912	3.27833	3.463163	34.87727
3.295427	3.29073	3.308335	34.82014
3.286477	3.220304	3.396713	34.83983
3.233561	3.254742	3.400481	34.80766
3.114987	3.267345	3.46069	34.70759
3.395978	3.432122	3.368509	35.48083
3.379274	3.438996	3.395434	35.51821
3.482672	3.450993	3.37059	35.71623
3.317079	3.36884	3.365287	35.16285
3.2899	3.391012	3.428633	35.29043
3.254513	3.408939	3.294574	34.95908
3.378491	3.387373	3.368407	35.3445
3.134577	3.285743	3.408939	34.67749
3.100015	3.18472	3.381912	34.32188
3.244202	3.277117	3.408529	34.89746
3.279878	3.392967	3.401504	35.21346
3.388018	3.383936	3.442701	35.52029
3.18209	3.348689	3.473308	35.05981
3.280538	3.356439	3.426468	35.18962
3.292251	3.411298	3.374819	35.22225
3.452317	3.403952	3.34873	35.49917
3.244615	3.332649	3.42038	35.04572
3.117379	3.323012	3.326972	34.54213
3.188565	3.289045	3.40974	34.80453
3.365705	3.245846	3.353504	34.97445
3.273898	3.28608	3.357989	34.87148
3.263669	3.265929	3.404147	34.90599
3.158004	3.313375	3.38684	34.74082
3.308624	3.410961	3.448737	35.41897

3.426044	3.472895	3.336435	35.5656
3.487373	3.390743	3.421898	35.70695
3.293117	3.391821	3.384708	35.20318
3.287508	3.383262	3.370948	35.1421
3.264783	3.418778	3.361928	35.15035
3.36154	3.355496	3.295785	35.07891
3.257813	3.205545	3.419784	34.79532
3.087725	3.265795	3.4397	34.59868
3.135526	3.359202	3.485262	35.00711
3.317121	3.44331	3.428002	35.46294
3.334732	3.410489	3.423859	35.42062
3.240037	3.331638	3.436886	35.06959
3.182667	3.282373	3.403192	34.76272
3.329782	3.367627	3.397804	35.25909
3.394741	3.384947	3.38568	35.41251
3.248244	3.365133	3.434226	35.15497
3.103067	3.261077	3.377291	34.48543
3.250224	3.225358	3.357852	34.68662
3.327679	3.251912	3.414327	35.03757
3.293612	3.356574	3.320543	34.98686
3.303222	3.359135	3.440927	35.27674
3.132969	3.409006	3.427201	34.98347
3.368881	3.46063	3.399185	35.55099
3.340671	3.39883	3.313927	35.16771
3.458792	3.391214	3.4013	35.60044
3.246058	3.400649	3.418692	35.19389
3.318152	3.34013	3.37446	35.12247
3.292704	3.267614	3.319128	34.78724
3.335391	3.253866	3.321839	34.85646
3.100057	3.225156	3.434789	34.52603
3.223292	3.354687	3.402715	35.00865
3.176975	3.464471	3.469165	35.29276
3.327514	3.437379	3.339573	35.27932
3.393338	3.327662	3.434107	35.39007
3.293034	3.342421	3.378723	35.08188
3.233933	3.397819	3.493105	35.32391
3.34133	3.469256	3.360768	35.4256
3.292787	3.282104	3.463691	35.13525
3.346692	3.282441	3.316792	34.93262
3.168397	3.210262	3.400413	34.56774
3.165427	3.221315	3.372176	34.52366
3.295055	3.299222	3.410269	35.06081
3.205021	3.338041	3.39557	34.91667
3.278434	3.374298	3.410559	35.18928
3.289529	3.428146	3.479941	35.48303
3.335804	3.477276	3.411514	35.54202
3.445181	3.418441	3.35717	35.53371
3.406041	3.469054	3.376882	35.60191
3.238098	3.350845	3.438881	35.11172
3.3151	3.293224	3.33826	34.93406

3.273568	3.252451	3.395911	34.88014
3.332711	3.19921	3.279756	34.63904
3.183451	3.256427	3.367043	34.62864
3.228571	3.422283	3.39482	35.15075
3.183121	3.446612	3.474468	35.27874
3.288044	3.401863	3.418164	35.2872
3.294189	3.365268	3.43027	35.24709
3.316873	3.440951	3.397122	35.38971
3.269031	3.431179	3.466556	35.41556
3.357044	3.414196	3.432095	35.49553
3.306604	3.235265	3.377768	34.87513
3.275094	3.211543	3.365815	34.72821
3.18143	3.266199	3.314251	34.53014
3.196071	3.226908	3.402596	34.66943
3.158375	3.316475	3.400072	34.77735
3.207908	3.2972	3.444389	34.94043
3.284621	3.398021	3.438182	35.3151
3.333783	3.437244	3.400976	35.42701
3.262473	3.485026	3.407916	35.39074
3.343145	3.441086	3.402647	35.45954
3.43652	3.401997	3.376234	35.5205
3.330318	3.37996	3.345541	35.17294
3.262597	3.294976	3.325642	34.79548
3.258349	3.220304	3.357972	34.6936
3.243006	3.227313	3.397344	34.76147
3.186915	3.315464	3.371817	34.77576
3.299138	3.408804	3.450681	35.39776
3.153467	3.402334	3.4892	35.14928
3.237933	3.335345	3.411855	35.01836
3.269856	3.353676	3.396167	35.09395
3.349084	3.402334	3.42183	35.42974
3.280455	3.510232	3.449862	35.57692
3.382491	3.355226	3.461117	35.48569
3.2249	3.224954	3.431515	34.79145
3.34562	3.237489	3.338328	34.87907
3.171284	3.228863	3.348934	34.50215
3.313986	3.257438	3.387931	34.96199
3.114657	3.364122	3.422989	34.83605
3.235376	3.39883	3.420534	35.17058
3.247419	3.420935	3.378979	35.15438
3.402907	3.4301	3.377547	35.51132
3.371892	3.373153	3.39985	35.36773
3.372923	3.417498	3.362593	35.38549
3.345661	3.358394	3.377666	35.22958
3.395112	3.341613	3.344671	35.22887
3.310315	3.246183	3.342557	34.83012
3.1632	3.258651	3.382117	34.62218
3.24115	3.262829	3.357716	34.74842
3.214548	3.453351	3.390182	35.17789
3.206093	3.450251	3.426945	35.23301

3.311264	3.386295	3.394871	35.253
3.195164	3.330021	3.510276	35.12842
3.235706	3.412915	3.461662	35.29204
3.428354	3.490148	3.415674	35.78166
3.295303	3.406445	3.414174	35.30437
3.314935	3.259527	3.417311	35.03288
3.288456	3.188292	3.389023	34.75733
3.270062	3.249822	3.419084	34.91741
3.232283	3.247935	3.364451	34.71119
3.290147	3.350441	3.446265	35.24081
3.136763	3.376118	3.424763	34.91451
3.307346	3.373692	3.358841	35.13808
3.314399	3.399234	3.428071	35.36075
3.363932	3.484892	3.415862	35.6297
3.243831	3.456991	3.443639	35.36678
3.306933	3.361696	3.412741	35.22882
3.291591	3.25737	3.391819	34.92137
3.395442	3.268019	3.315752	35.00541
3.27629	3.231222	3.37163	34.78657
3.288209	3.292146	3.343785	34.88498
3.111728	3.321597	3.416765	34.72304
3.239253	3.477276	3.36411	35.22722
3.189555	3.406243	3.423194	35.0924
3.315636	3.368637	3.386141	35.20486
3.234056	3.404221	3.467016	35.28113
3.309656	3.42572	3.43397	35.4212
3.281981	3.393101	3.429384	35.27932
3.418621	3.306163	3.348849	35.2119
3.300541	3.207432	3.399185	34.84784
3.203165	3.168343	3.416902	34.58816
3.269814	3.254405	3.39598	34.87636
3.193803	3.2972	3.378297	34.76505
3.328504	3.4177	3.418351	35.41073
3.242965	3.41123	3.444491	35.26668
3.20201	3.434751	3.404164	35.14037
3.244903	3.436772	3.451772	35.3427
3.401546	3.420328	3.343921	35.41344
3.273815	3.36729	3.426536	35.19879
3.340052	3.339254	3.305112	35.0168
3.287838	3.236681	3.435607	34.96367
3.338856	3.278667	3.311966	34.89668
3.31844	3.269164	3.36058	34.93756
3.306604	3.323551	3.33618	34.97725
3.190297	3.340804	3.419221	34.94223
3.259421	3.412241	3.453392	35.32435
3.185059	3.400043	3.424268	35.07136
3.314687	3.464943	3.431174	35.51187
3.266845	3.423159	3.479174	35.42084
3.269732	3.380836	3.464868	35.30331
3.258431	3.365268	3.418948	35.14413

3.334897	3.246453	3.431566	35.07912
3.314027	3.223134	3.366941	34.84116
3.258225	3.23695	3.380974	34.78003
3.212445	3.225763	3.399577	34.69613
3.229355	3.310544	3.366378	34.84591
3.349497	3.413859	3.384726	35.3747
3.265773	3.402671	3.416493	35.23661
3.247254	3.421407	3.39939	35.19969
3.278393	3.430977	3.40662	35.30452
3.387482	3.440075	3.421813	35.5962
3.385461	3.360213	3.363292	35.28916
3.30483	3.327729	3.322897	34.95346
3.237108	3.2817	3.428224	34.93504
3.357168	3.274219	3.398981	35.11728
3.259751	3.298211	3.319606	34.78313
3.408557	3.350845	3.327705	35.24136
3.149714	3.339186	3.394121	34.79506
3.237067	3.368705	3.398435	35.06007
3.149755	3.395999	3.337442	34.79544
3.303552	3.443916	3.429878	35.4387
3.201763	3.443781	3.448839	35.25727
3.340258	3.386901	3.415487	35.36282
3.186049	3.313442	3.44855	34.93725
3.39074	3.201029	3.348372	34.91997
3.365788	3.215654	3.406074	35.02357
3.293777	3.25919	3.377155	34.89806
3.247048	3.29255	3.377615	34.86983
3.207124	3.385014	3.385237	35.0014
3.287962	3.397819	3.350776	35.13082
3.367396	3.363313	3.28378	35.08256
3.178337	3.42107	3.456581	35.17331
3.29221	3.457867	3.438063	35.4623
3.322111	3.417767	3.41697	35.39387
3.361169	3.315262	3.341466	35.09001
3.320338	3.238702	3.36602	34.88699
3.277774	3.248811	3.426195	34.94761
3.219044	3.291809	3.423586	34.9075
3.290849	3.373018	3.371476	35.12816
3.302149	3.362976	3.404574	35.20329
3.267711	3.370524	3.4264	35.19222
3.168686	3.369514	3.424285	34.96883
3.141011	3.441692	3.419425	35.05553
3.355188	3.517038	3.410439	35.66902
3.231252	3.382521	3.417396	35.11903
3.293983	3.283115	3.396662	34.99349
3.257895	3.238433	3.449095	34.93152
3.34929	3.263301	3.385612	35.04694
3.315388	3.252451	3.395639	34.971
3.417961	3.312094	3.373454	35.27723
3.227292	3.339523	3.434772	35.05434

3.266556	3.407187	3.3629	35.131
3.221601	3.369985	3.380275	34.98934
3.323637	3.389664	3.359472	35.21002
3.248698	3.481791	3.445293	35.43528
3.210341	3.420598	3.488928	35.313
3.238923	3.327662	3.420585	35.02282
3.439655	3.286484	3.323545	35.15952
3.282229	3.232637	3.394274	34.85218
3.276908	3.344308	3.383498	35.06118
3.290601	3.367896	3.410934	35.20271
3.220034	3.391551	3.352242	34.97177
3.361416	3.369109	3.325727	35.17389
3.237026	3.392495	3.386891	35.08676
3.286353	3.426192	3.412605	35.32455
3.165634	3.438794	3.453477	35.1775
3.31345	3.368233	3.409075	35.24935
3.242099	3.331638	3.43941	35.07962
3.27897	3.287698	3.389756	34.95558
3.187658	3.247127	3.472285	34.84765
3.314234	3.227111	3.354374	34.82282
3.259957	3.220102	3.433408	34.86164
3.361581	3.297065	3.382662	35.14121
3.330566	3.40658	3.453614	35.46803
3.244408	3.460832	3.376319	35.22923
3.277527	3.385216	3.408035	35.20565
3.310027	3.398291	3.365219	35.21168
3.199948	3.453756	3.408768	35.18749
3.351724	3.449577	3.379252	35.44571
3.199866	3.272736	3.450118	34.88187
3.315966	3.239376	3.372909	34.89397
3.366901	3.252788	3.434789	35.17001
3.374037	3.368098	3.390915	35.34183
3.317121	3.393573	3.373642	35.2353
3.285569	3.357652	3.386431	35.11572
3.252657	3.306029	3.38065	34.91821
3.365417	3.383531	3.328183	35.21954
3.228076	3.477343	3.414787	35.31375
3.109707	3.456991	3.502245	35.20164
3.26305	3.378949	3.418061	35.18221
3.294066	3.25791	3.459838	35.0767
3.292663	3.24497	3.38817	34.88862
3.240449	3.213295	3.44652	34.83277
3.175614	3.276308	3.441473	34.81775
3.222384	3.310342	3.298087	34.68089
3.42225	3.359674	3.37932	35.40349
3.316502	3.404423	3.32837	35.15867
3.262638	3.369379	3.358313	35.02972
3.183203	3.329212	3.389483	34.83634
3.298066	3.465415	3.372858	35.34902
3.209063	3.51104	3.327518	35.15501

3.355023	3.345117	3.366378	35.19634
3.204774	3.22765	3.416424	34.72033
3.366943	3.252451	3.399032	35.09117
3.261153	3.355361	3.40546	35.09893
3.49954	3.390675	3.389074	35.66163
3.321327	3.369716	3.46318	35.38813
3.279878	3.294572	3.3895	34.97204
3.277939	3.311825	3.374631	34.97301
3.380594	3.464	3.401368	35.58875
3.258019	3.496079	3.445276	35.48687
3.218549	3.349632	3.371596	34.91917
3.099974	3.232435	3.520985	34.73027
3.313615	3.300233	3.364997	35.00461
3.370325	3.291404	3.479788	35.36035
3.248574	3.30549	3.390387	34.92939
3.241109	3.219091	3.439001	34.83044
3.305531	3.274084	3.407165	35.02196
3.420394	3.374703	3.325795	35.31524
3.413383	3.441894	3.33219	35.46083
3.156849	3.364998	3.35804	34.7882
3.187864	3.368368	3.356539	34.86012
3.284373	3.467841	3.47162	35.54036
3.270351	3.40685	3.32687	35.05977
3.32805	3.243622	3.33959	34.85682
3.138825	3.212891	3.428105	34.56938
3.253565	3.305826	3.396849	34.95518
3.288085	3.372007	3.372056	35.12118
3.503623	3.397752	3.321703	35.53871
3.276908	3.32746	3.434431	35.13572
3.293777	3.328875	3.349667	34.99034
3.299427	3.415678	3.403056	35.30927
3.392349	3.516095	3.349667	35.61532
3.176439	3.427472	3.398691	35.05656
3.187699	3.285811	3.372022	34.71307
3.135113	3.24497	3.488672	34.76386
3.271877	3.282441	3.371357	34.88833
3.373913	3.221247	3.386294	35.01032
3.340877	3.242544	3.381179	34.97346
3.227664	3.240657	3.431703	34.83224
3.315718	3.365942	3.346036	35.11144
3.375315	3.432594	3.389892	35.48343
3.42423	3.379623	3.332411	35.34886
3.139073	3.354552	3.433561	34.89164
3.103768	3.42909	3.335054	34.76202
3.282682	3.500257	3.449504	35.55919
3.283713	3.410691	3.3526	35.15366
3.299303	3.251642	3.375313	34.88961
3.229726	3.22893	3.429997	34.80738
3.232407	3.343028	3.367879	34.92691
3.295303	3.405771	3.442411	35.36465

3.437056	3.450723	3.339522	35.54794
3.265979	3.254742	3.409143	34.8975
3.255338	3.343095	3.373284	34.98902
3.290477	3.435896	3.451414	35.43967
3.353868	3.500123	3.395502	35.59647
3.200855	3.394314	3.34092	34.91111
3.212527	3.303603	3.42154	34.91457
3.127978	3.261886	3.430151	34.65727
3.304418	3.289787	3.427269	35.09783
3.219044	3.255079	3.441797	34.867
3.332051	3.256225	3.393678	35.01141
3.241851	3.288372	3.494111	35.10409
3.40699	3.351856	3.405631	35.41056
3.313862	3.386295	3.421335	35.31656
3.289158	3.393438	3.366702	35.15868
3.170542	3.34478	3.423739	34.91761
3.247584	3.511647	3.412895	35.42728
3.129958	3.420396	3.393234	34.92751
3.304046	3.266132	3.370641	34.92145
3.303923	3.212149	3.419153	34.90922
3.211455	3.343028	3.408188	34.96924
3.270557	3.460765	3.404232	35.34731
3.362282	3.385688	3.355942	35.27811
3.401711	3.320855	3.375586	35.26552
3.36088	3.311285	3.31952	35.03269
3.217641	3.436435	3.391052	35.14956
3.284909	3.47667	3.362337	35.32186
3.307181	3.420868	3.403465	35.33847
3.203949	3.246048	3.408188	34.74075
3.2866	3.225089	3.354186	34.75756
3.183574	3.321057	3.46335	34.98085
3.241728	3.337502	3.384402	34.97134
3.267092	3.28453	3.449794	35.05397
3.279713	3.296863	3.389278	34.9762
3.252368	3.338041	3.361723	34.94619
3.484899	3.343297	3.359336	35.46097
3.241109	3.364392	3.450749	35.17388
3.317368	3.401526	3.331388	35.16083
3.227581	3.345117	3.395792	34.98197
3.310975	3.39364	3.346649	35.16298
3.249523	3.27368	3.324175	34.71711
3.195865	3.269164	3.371357	34.69308
3.200773	3.285541	3.3511	34.69532
3.315471	3.389664	3.376575	35.22956
3.295385	3.440479	3.388801	35.3235
3.363478	3.356237	3.441268	35.40292
3.332876	3.300165	3.372977	35.06403
3.343269	3.419115	3.385049	35.37329
3.277609	3.487655	3.388477	35.38708
3.267835	3.415611	3.451636	35.34627

3.216074	3.323079	3.361535	34.83369
3.154085	3.237287	3.347638	34.48013
3.200402	3.235333	3.386209	34.66149
3.285569	3.357652	3.399236	35.14372
3.23451	3.286754	3.481919	35.05783
3.140516	3.237961	3.369652	34.50007
3.36121	3.339591	3.452591	35.38631
3.294808	3.451869	3.369993	35.30601
3.414538	3.43037	3.37782	35.53794
3.313821	3.322271	3.371084	35.06656
3.293818	3.293224	3.361791	34.93898
3.235417	3.364257	3.381452	35.0096
3.333535	3.453553	3.343887	35.3373
3.180646	3.277117	3.375756	34.68681
3.171985	3.237422	3.358125	34.5425
3.207372	3.165715	3.383975	34.51961
3.330318	3.401391	3.413355	35.3681
3.243955	3.434953	3.442428	35.31621
3.331391	3.398291	3.43172	35.40383
3.342527	3.301379	3.392143	35.1297
3.370531	3.466089	3.46509	35.71066
3.298602	3.520071	3.379798	35.4849
3.287013	3.390203	3.34844	35.10698
3.174996	3.313105	3.434363	34.88131
3.18708	3.314385	3.40558	34.8476
3.225148	3.289382	3.388528	34.83888
3.272495	3.299626	3.362934	34.90885
3.236737	3.227852	3.473871	34.91629
3.279713	3.285945	3.396167	34.96739
3.398906	3.354013	3.453051	35.5013
3.369252	3.409882	3.343188	35.31837
3.417548	3.350239	3.387437	35.39032
3.237397	3.370861	3.429997	35.13453
3.273485	3.428079	3.416782	35.30968
3.247131	3.453217	3.357017	35.17632
3.387771	3.358596	3.333758	35.22609
3.100593	3.242746	3.404505	34.49945
3.197432	3.304479	3.400481	34.83742
3.147115	3.295178	3.408308	34.72416
3.335721	3.449645	3.411514	35.48142
3.261153	3.384475	3.409808	35.1721
3.387564	3.318092	3.402732	35.2879
3.235995	3.278936	3.400328	34.86556
3.366448	3.447218	3.396321	35.51008
3.280373	3.461439	3.347076	35.24525
3.365623	3.363516	3.342847	35.20829
3.119111	3.18809	3.432129	34.48083
3.211909	3.274151	3.350486	34.69342
3.24845	3.388384	3.404625	35.14154
3.294066	3.400245	3.418061	35.29661

3.267175	3.267749	3.461372	35.04277
3.281321	3.268423	3.383958	34.90589
3.321905	3.300705	3.420107	35.14429
3.421301	3.356641	3.356471	35.34481
3.348548	3.420261	3.426127	35.47717
3.232035	3.398628	3.414753	35.15019
3.301489	3.33366	3.462191	35.26374
3.217394	3.2941	3.312836	34.66671
3.373088	3.272871	3.346325	35.034
3.218879	3.217136	3.41049	34.7152
3.12703	3.383531	3.417737	34.89407
3.23583	3.423024	3.418897	35.2209
3.378243	3.446949	3.342182	35.41689
3.275135	3.358663	3.392007	35.1073
3.32108	3.343702	3.369379	35.12558
3.221807	3.3652	3.426724	35.0809
3.32042	3.475928	3.367452	35.40908
3.320503	3.351587	3.362882	35.12735
3.369417	3.250159	3.341551	34.96586
3.165304	3.142666	3.369908	34.34644
3.242511	3.35125	3.371135	34.97411
3.155735	3.412915	3.461458	35.11671
3.313904	3.441288	3.355738	35.29346
3.23484	3.221517	3.515852	34.9901
3.298025	3.309803	3.43866	35.15253
3.34727	3.36857	3.44282	35.39784
3.436809	3.445466	3.385612	35.6367
3.315883	3.388182	3.400771	35.28013
3.240449	3.357854	3.402851	35.0534
3.226468	3.318901	3.480334	35.10708
3.233644	3.34168	3.379116	34.95124
3.37647	3.256157	3.324295	34.95667
3.252492	3.270647	3.4471	34.98579
3.180564	3.388384	3.395894	34.97399
3.20135	3.38461	3.451977	35.13383
3.421838	3.441355	3.369157	35.55899
3.346816	3.334199	3.357153	35.13434
3.293571	3.367627	3.436954	35.26551
3.228489	3.417902	3.377086	35.10221
3.395277	3.494461	3.450749	35.79546
3.263422	3.332717	3.337237	34.90517
3.363189	3.164367	3.339437	34.76001
3.075187	3.195301	3.497266	34.54299
3.180605	3.367896	3.380411	34.89542
3.244408	3.423294	3.458576	35.32702
3.254843	3.372142	3.353692	35.00862
3.285198	3.228863	3.412025	34.88923
3.280703	3.362168	3.396235	35.13639
3.232943	3.343365	3.449709	35.10777
3.396762	3.396336	3.321004	35.30039

3.359643	3.412241	3.370948	35.36322
3.245687	3.352193	3.391273	35.02715
3.235087	3.237624	3.361535	34.6884
3.278764	3.257438	3.408308	34.92952
3.291921	3.295246	3.30489	34.81482
3.291467	3.352867	3.366497	35.07456
3.188029	3.41925	3.457024	35.19149
3.279672	3.341141	3.400481	35.09744
3.324503	3.362774	3.437176	35.32303
3.39107	3.331099	3.319776	35.1426
3.312543	3.383801	3.403823	35.26992
3.183286	3.47667	3.394973	35.17099
3.310563	3.454969	3.446947	35.51553
3.250842	3.19119	3.412809	34.73343
3.331143	3.165648	3.353419	34.72331
3.218054	3.230076	3.39794	34.71425
3.234015	3.405502	3.332821	34.99038
3.208073	3.415746	3.450732	35.2139
3.297612	3.369379	3.378962	35.15136
3.232902	3.251372	3.379729	34.75347
3.269031	3.333054	3.430697	35.12256
3.26701	3.379083	3.441797	35.24307
3.345042	3.439738	3.462481	35.59159
3.402948	3.416959	3.338618	35.39754
3.407815	3.274084	3.375995	35.17748
3.24049	3.211812	3.388733	34.70324
3.242511	3.229604	3.369089	34.70361
3.175367	3.304681	3.366429	34.71514
3.254596	3.446005	3.380633	35.22852
3.266309	3.444658	3.469574	35.44569
3.224488	3.388721	3.406824	35.09468
3.313244	3.312162	3.430748	35.17367
3.38942	3.397819	3.431089	35.52832
3.303923	3.465078	3.390233	35.39909
3.275465	3.461641	3.453938	35.46865
3.307676	3.322203	3.430833	35.18364
3.229767	3.134646	3.411531	34.5609
3.32607	3.213025	3.369788	34.85162
3.278146	3.38124	3.33514	35.0389
3.268412	3.486779	3.432265	35.46081
3.119482	3.36675	3.38389	34.76685
3.28293	3.294572	3.42981	35.06686
3.293158	3.305085	3.394121	35.03417
3.27266	3.348958	3.439359	35.18422
3.228324	3.37814	3.421574	35.11219
3.260947	3.425989	3.443929	35.33705
3.295138	3.370727	3.382458	35.15655
3.356962	3.222393	3.317866	34.82611
3.326895	3.280149	3.37475	35.01106
3.209517	3.282441	3.391342	34.79567

3.152436	3.372479	3.404846	34.89727
3.301531	3.465145	3.359813	35.32748
3.19768	3.387171	3.384521	34.98389
3.333989	3.38151	3.41651	35.33955
3.187781	3.353002	3.46734	35.06864
3.324709	3.430774	3.373165	35.33221
3.40996	3.447623	3.505178	35.84417
3.410537	3.407658	3.342114	35.40144
3.301324	3.166119	3.44664	34.86299
3.27167	3.191796	3.324278	34.5867
3.274557	3.348958	3.439956	35.18967
3.328421	3.498303	3.370181	35.48147
3.289776	3.424507	3.443281	35.39544
3.139898	3.305759	3.454961	34.83354
3.254596	3.23257	3.427286	34.86379
3.393998	3.328471	3.40251	35.32418
3.270722	3.430977	3.394377	35.26097
3.210341	3.408467	3.432726	35.16357
3.24049	3.412713	3.459957	35.29834
3.248657	3.235198	3.301565	34.58162
3.441882	3.172859	3.278	34.81631
3.283713	3.226908	3.427576	34.91572
3.217765	3.386227	3.411599	35.08497
3.190586	3.421744	3.406398	35.09183
3.356343	3.45443	3.342148	35.38528
3.314976	3.403884	3.389364	35.28754
3.298437	3.412915	3.339471	35.16201
3.225395	3.412309	3.522621	35.40148
3.353374	3.40995	3.355857	35.3115
3.296994	3.399975	3.445003	35.36134
3.41561	3.295313	3.391683	35.27525
3.293653	3.151832	3.37163	34.65093
3.203866	3.267008	3.405852	34.78129
3.275506	3.421609	3.43247	35.33425
3.308377	3.413926	3.411104	35.34261
3.250183	3.409141	3.354902	35.08198
3.248615	3.263166	3.418266	34.8979
3.159076	3.303333	3.454057	34.8682
3.321822	3.370524	3.392331	35.23605
3.297612	3.425046	3.457979	35.4459
3.261731	3.39054	3.440638	35.25405
3.312254	3.348621	3.441064	35.2738
3.31114	3.252518	3.397463	34.96585
3.317368	3.222258	3.302469	34.70556
3.290477	3.339523	3.397275	35.11052
3.171449	3.432527	3.423416	35.11077
3.233644	3.432527	3.401334	35.19849
3.374078	3.408467	3.347945	35.33623
3.246223	3.410893	3.445889	35.27613
3.30417	3.498168	3.369584	35.42684

3.193555	3.425652	3.442275	35.18532
3.318729	3.38124	3.503115	35.49498
3.260947	3.369109	3.428906	35.17981
3.380883	3.206758	3.430748	35.09108
3.304665	3.166995	3.399356	34.76881
3.316254	3.356978	3.4493	35.31883
3.275547	3.385486	3.404915	35.19509
3.340176	3.395258	3.376046	35.29466
3.204485	3.33056	3.396321	34.90078
3.272495	3.260807	3.38418	34.87042
3.257895	3.338849	3.438728	35.12844
3.236036	3.448971	3.429247	35.30073
3.296664	3.44021	3.480947	35.52721
3.385378	3.382992	3.407864	35.43627
3.334402	3.232435	3.362081	34.89543
3.319925	3.14489	3.343273	34.63119
3.27629	3.294909	3.422222	35.03648
3.215621	3.385216	3.366242	34.97888
3.267257	3.442029	3.356966	35.19575
3.18044	3.389529	3.403278	34.99237
3.264618	3.364122	3.354971	35.01525
3.24412	3.386025	3.376933	35.06635
3.289281	3.438457	3.417192	35.36781
3.263092	3.453419	3.392961	35.29027
3.358653	3.389597	3.420943	35.42087
3.198051	3.204938	3.366361	34.54648
3.336258	3.190112	3.327773	34.73191
3.33403	3.292483	3.395349	35.09868
3.298602	3.451195	3.395826	35.36933
3.275259	3.406647	3.381298	35.18909
3.327968	3.391484	3.439887	35.39932
3.211785	3.305422	3.321157	34.6974
3.354405	3.312566	3.35139	35.09102
3.201268	3.341208	3.464919	35.06704
3.233685	3.45389	3.467715	35.39047
3.172892	3.42545	3.467511	35.19488
3.376594	3.256629	3.356522	35.02845
3.351023	3.194559	3.373881	34.87475
3.364056	3.195975	3.406671	34.97805
3.177883	3.319844	3.469983	34.98026
3.315223	3.448769	3.289441	35.16772
3.29786	3.421002	3.361467	35.22654
3.334319	3.437851	3.327722	35.26932
3.168232	3.326314	3.402715	34.8262
3.248698	3.473772	3.409194	35.3388
3.285776	3.487385	3.404028	35.43836
3.358653	3.382857	3.334645	35.21741
3.340299	3.194357	3.440041	34.99554
3.242635	3.16403	3.372721	34.56843
3.24944	3.284328	3.41801	34.94542

3.35259	3.423361	3.325164	35.272
3.404144	3.457732	3.428446	35.68576
3.208238	3.378207	3.38273	34.98346
3.27299	3.290596	3.443792	35.06701
3.250677	3.294437	3.389432	34.90774
3.303345	3.370592	3.376183	35.16048
3.213641	3.40375	3.435522	35.16658
3.252162	3.413993	3.409143	35.21554
3.169263	3.258179	3.422716	34.72319
3.439077	3.210734	3.282297	34.9024
3.297695	3.197525	3.400532	34.8229
3.338443	3.322608	3.338601	35.05011
3.215868	3.443916	3.448345	35.28733
3.401917	3.477478	3.348423	35.54907
3.345661	3.385081	3.374887	35.28187
3.359931	3.364189	3.317082	35.14098
3.200897	3.386699	3.444457	35.12097
3.238634	3.467302	3.408444	35.301
3.218301	3.396269	3.425018	35.13745
3.282352	3.204534	3.45757	34.92941
3.284827	3.164232	3.374614	34.66527
3.379398	3.208577	3.437023	35.10554
3.253812	3.430303	3.407779	35.25183
3.376635	3.480983	3.445003	35.71265
3.366283	3.389395	3.372738	35.3317
3.318069	3.283519	3.413355	35.08355
3.202299	3.270377	3.401283	34.77524
3.243171	3.39728	3.396866	35.13248
3.180894	3.441692	3.407575	35.11683
3.308831	3.399032	3.387931	35.26036
3.299675	3.300098	3.454432	35.16941
3.182791	3.205073	3.432998	34.65913
3.276001	3.211947	3.392211	34.7888
3.286271	3.306905	3.389142	35.0122
3.294478	3.423698	3.415487	35.34317
3.2574	3.464808	3.369481	35.25138
3.315512	3.351385	3.433271	35.26992
3.291756	3.355091	3.286679	34.9055
3.364097	3.405367	3.423808	35.47353
3.185843	3.491496	3.368544	35.15121
3.274351	3.441288	3.457757	35.43006
3.121173	3.283384	3.380343	34.58048
3.300293	3.149338	3.371886	34.66056
3.277609	3.243555	3.424217	34.93143
3.35094	3.396269	3.38817	35.34692
3.332092	3.470739	3.478015	35.66504
3.377171	3.399908	3.348849	35.32626
3.282105	3.293763	3.435539	35.07582
3.409176	3.343432	3.263659	35.08645
3.206506	3.32564	3.462191	35.03849

3.170954	3.331436	3.306578	34.63312
3.105996	3.371805	3.454552	34.90294
3.317657	3.374366	3.417788	35.29101
3.321451	3.225561	3.403397	34.94242
3.257235	3.187079	3.411241	34.73499
3.190462	3.239107	3.522894	34.94692
3.316626	3.397078	3.385476	35.26776
3.409217	3.429224	3.382816	35.53473
3.343723	3.385553	3.36353	35.25382
3.308212	3.330762	3.395417	35.12608
3.246471	3.388653	3.379269	35.08235
3.320626	3.490957	3.282143	35.25584
3.307016	3.481589	3.361058	35.37816
3.243295	3.348419	3.353947	34.93204
3.176811	3.172522	3.449317	34.61055
3.263917	3.197457	3.347979	34.63396
3.299551	3.315059	3.454705	35.20245
3.310604	3.480915	3.352259	35.36529
3.216157	3.40813	3.46983	35.25669
3.270969	3.349565	3.437432	35.17763
3.305325	3.333323	3.418709	35.1763
3.392019	3.33184	3.357426	35.22863
3.256287	3.323821	3.374341	34.95126
3.175614	3.384475	3.460622	35.09616
3.113873	3.397684	3.348303	34.74441
3.316873	3.298413	3.418266	35.12425
3.283548	3.274556	3.274231	34.68422
3.254472	3.263436	3.423995	34.92382
3.223539	3.370322	3.363155	34.95688
3.373583	3.369244	3.439512	35.44962
3.441758	3.439536	3.317866	35.4864
3.49257	3.35745	3.377973	35.54946
3.253894	3.351654	3.363019	34.98213
3.221972	3.393775	3.388596	35.06037
3.212981	3.458878	3.411701	35.2336
3.241109	3.369514	3.303833	34.8638
3.258596	3.188022	3.423109	34.76599
3.151363	3.213699	3.428855	34.6002
3.210341	3.316407	3.502927	35.11577
3.35259	3.49682	3.372295	35.53571
3.351724	3.420194	3.454501	35.54601
3.25806	3.341141	3.400532	35.05029
3.305284	3.281228	3.42877	35.08429
3.280331	3.251372	3.350691	34.79369
3.348672	3.357787	3.355329	35.18599
3.304954	3.41278	3.367555	35.23739
3.240532	3.356304	3.411991	35.07018
3.119936	3.254203	3.360086	34.46966
3.347228	3.217406	3.400669	34.975
3.263133	3.236883	3.416117	34.86747

3.250265	3.368974	3.352652	34.9894
3.173016	3.391282	3.483335	35.15504
3.383234	3.455508	3.366327	35.49932
3.400515	3.375107	3.392961	35.41954
3.348053	3.407456	3.305453	35.18419
3.274516	3.372816	3.422989	35.20465
3.261937	3.395999	3.248313	34.84585
3.179574	3.325303	3.378775	34.79644
3.251007	3.237152	3.304361	34.59715
3.147651	3.175891	3.395792	34.4371
3.206465	3.312498	3.376114	34.82142
3.20168	3.441894	3.481902	35.32527
3.321162	3.448769	3.4493	35.53029
3.42955	3.362033	3.394939	35.45877
3.361499	3.331301	3.388664	35.22902
3.333577	3.345521	3.432521	35.29497
3.282146	3.352261	3.384964	35.09323
3.253771	3.323349	3.391	34.98116
3.254802	3.323551	3.36261	34.92177
3.357168	3.285676	3.348644	35.03226
3.188317	3.191122	3.412417	34.5957
3.285569	3.27988	3.32675	34.81513
3.248945	3.310881	3.45994	35.0941
3.241769	3.421407	3.362968	35.10804
3.234263	3.406176	3.430492	35.20599
3.368922	3.364324	3.33028	35.18979
3.343764	3.330897	3.315087	35.02845
3.410908	3.442366	3.332582	35.45731
3.281486	3.4146	3.39141	35.24221
3.311553	3.428955	3.375296	35.30412
3.181842	3.291404	3.356079	34.67763
3.215043	3.180676	3.340391	34.47379
3.216074	3.260538	3.401266	34.78381
3.270969	3.40348	3.431106	35.2817
3.232613	3.450656	3.434294	35.30796
3.383976	3.376253	3.383822	35.36589
3.275671	3.315599	3.419357	35.07411
3.385337	3.362168	3.377888	35.32509
3.352342	3.396269	3.423722	35.42774
3.24944	3.338512	3.403806	35.03285
3.204609	3.292415	3.358313	34.73452
3.266597	3.272332	3.443059	35.01148
3.270392	3.230009	3.374955	34.77829
3.295344	3.254068	3.396986	34.93365
3.148559	3.310072	3.463436	34.88044
3.280414	3.346398	3.377069	35.05936
3.327926	3.422283	3.431447	35.44812
3.316461	3.439064	3.317969	35.21159
3.429591	3.359809	3.369021	35.39731
3.305201	3.400784	3.360103	35.1954

3.256287	3.422485	3.374631	35.16766
3.282146	3.385418	3.294813	34.9686
3.321327	3.299222	3.362132	35.013
3.142578	3.190853	3.406552	34.48226
3.223416	3.240859	3.412895	34.78226
3.215126	3.369177	3.466232	35.16138
3.279424	3.511242	3.415128	35.50091
3.23517	3.459686	3.408086	35.27599
3.267134	3.325775	3.482448	35.21567
3.28062	3.323416	3.349309	34.94885
3.455863	3.379151	3.43513	35.64163
3.285982	3.394584	3.404301	35.23646
3.322111	3.319103	3.383907	35.10581
3.202051	3.239174	3.393985	34.6905
3.299056	3.230346	3.375773	34.84351
3.254967	3.262627	3.373693	34.81313
3.279589	3.330223	3.342148	34.94582
3.175532	3.372614	3.467391	35.08485
3.27134	3.381307	3.39557	35.15632
3.30714	3.424844	3.495083	35.54743
3.479578	3.421946	3.294489	35.47952
3.325452	3.364796	3.431225	35.31651
3.295509	3.436435	3.39673	35.33226
3.248698	3.395123	3.412673	35.17442
3.251214	3.307848	3.328455	34.80489
3.267422	3.238905	3.34919	34.73491
3.201557	3.210801	3.354578	34.5412
3.111192	3.227852	3.427815	34.54103
3.199247	3.492237	3.454501	35.37012
3.333164	3.511984	3.419289	35.62915
3.265896	3.376051	3.397616	35.13739
3.292251	3.324764	3.423398	35.13925
3.21958	3.356439	3.452062	35.11228
3.369747	3.4826	3.376694	35.55175
3.323885	3.4115	3.431532	35.41589
3.302026	3.32874	3.321498	34.94648
3.175903	3.199075	3.462617	34.69572
3.33898	3.199749	3.293926	34.68491
3.159365	3.271254	3.399322	34.67898
3.293571	3.389597	3.32912	35.07774
3.144517	3.352193	3.495663	35.03419
3.229355	3.408939	3.373778	35.07727
3.26305	3.419789	3.427832	35.29289
3.413053	3.394112	3.397361	35.49814
3.358282	3.420396	3.452181	35.55572
3.359189	3.413656	3.40824	35.44688
3.22655	3.330762	3.35908	34.86804
3.284951	3.225763	3.335805	34.71523
3.262143	3.254877	3.291522	34.63218
3.194256	3.250361	3.386652	34.68189

3.162829	3.363852	3.402442	34.89588
3.216734	3.47485	3.442974	35.34513
3.277774	3.432796	3.402306	35.29771
3.352342	3.377938	3.402851	35.34201
3.30351	3.353137	3.429179	35.23856
3.16188	3.4208	3.489081	35.2078
3.268453	3.432324	3.397616	35.26605
3.255173	3.241668	3.41315	34.85404
3.371314	3.245307	3.363854	35.00818
3.216074	3.221045	3.39569	34.68525
3.2395	3.279273	3.397395	34.86755
3.21892	3.370727	3.380974	34.98662
3.252905	3.412241	3.4161	35.22854
3.192565	3.409411	3.357153	34.96149
3.349579	3.379623	3.423381	35.38455
3.221188	3.440479	3.355482	35.08838
3.384017	3.430505	3.381622	35.47981
3.332216	3.43684	3.394394	35.40831
3.390286	3.36702	3.398981	35.39265
3.243501	3.276375	3.340579	34.74571
3.256039	3.231896	3.370539	34.74138
3.214631	3.227043	3.387437	34.67716
3.310604	3.342489	3.376592	35.11579
3.198752	3.420126	3.47353	35.25295
3.152889	3.421272	3.393729	34.98065
3.180151	3.399302	3.494657	35.21294
3.272042	3.429898	3.388272	35.24815
3.282064	3.379825	3.453392	35.30297
3.264453	3.404895	3.365474	35.12702
3.186585	3.311353	3.521939	35.09434
3.31213	3.23877	3.311711	34.75042
3.338402	3.193953	3.372721	34.84329
3.275052	3.209521	3.36527	34.7225
3.213352	3.302861	3.405443	34.87955
3.19273	3.351789	3.424814	34.98381
3.337536	3.383127	3.393354	35.30021
3.348837	3.393708	3.377615	35.31364
3.335391	3.445399	3.432879	35.51813
3.181595	3.441423	3.391614	35.08287
3.299675	3.440951	3.372841	35.299
3.343475	3.356372	3.411445	35.29425
3.452317	3.310679	3.404028	35.41613
3.215951	3.254136	3.383293	34.73024
3.283961	3.223337	3.362269	34.76563
3.20865	3.27833	3.403806	34.81204
3.285198	3.378073	3.348661	35.07697
3.174295	3.386901	3.396645	34.95868
3.222384	3.375916	3.431703	35.11648
3.135155	3.363246	3.472695	34.98766
3.329246	3.389395	3.361109	35.22527

3.328669	3.431044	3.476105	35.56657
3.346445	3.387777	3.398657	35.34146
3.194215	3.284395	3.449317	34.89326
3.271918	3.193818	3.402118	34.76189
3.347641	3.246655	3.396099	35.02987
3.36917	3.265727	3.324431	34.96193
3.1486	3.305153	3.37354	34.67319
3.223044	3.362505	3.412059	35.04564
3.304335	3.34195	3.402391	35.15732
3.352796	3.462112	3.345064	35.40071
3.338031	3.466965	3.386823	35.47035
3.216033	3.42572	3.482584	35.32278
3.254266	3.376455	3.39008	35.09636
3.357498	3.275162	3.355329	35.02461
3.353745	3.296257	3.354698	35.06115
3.231458	3.275028	3.436562	34.92633
3.273856	3.30704	3.404301	35.0185
3.211043	3.383329	3.411053	35.06274
3.376057	3.402536	3.383583	35.40553
3.227169	3.350037	3.402578	35.00666
3.241728	3.360483	3.418556	35.09628
3.165221	3.394854	3.440586	35.05232
3.307511	3.418711	3.361604	35.24293
3.319059	3.407524	3.431174	35.39586
3.293694	3.270984	3.407217	34.98941
3.185018	3.149136	3.398554	34.46635
3.338732	3.200692	3.427593	34.97875
3.29023	3.268086	3.398486	34.95641
3.358653	3.356439	3.348627	35.19022
3.131319	3.428281	3.420193	35.00668
3.121586	3.421137	3.429725	34.99062
3.243707	3.417026	3.365491	35.10822
3.303387	3.448229	3.421693	35.42987
3.305861	3.447151	3.409962	35.40727
3.254018	3.362976	3.433766	35.16188
3.208857	3.315194	3.402954	34.89124
3.375851	3.261751	3.371749	35.07132
3.413383	3.26256	3.371152	35.15386
3.248079	3.319575	3.340664	34.85038
3.244037	3.336828	3.363292	34.92875
3.182337	3.372411	3.387351	34.92425
3.285404	3.417902	3.40251	35.28228
3.256905	3.4208	3.378979	35.17483
3.224694	3.405434	3.484716	35.30202
3.157137	3.417093	3.427184	35.05397
3.29221	3.388856	3.421881	35.276
3.249275	3.327595	3.393592	34.98628
3.293529	3.214845	3.420738	34.89585
3.214672	3.172791	3.437227	34.6675
3.315347	3.21963	3.449914	35.01783

3.277486	3.25764	3.354306	34.80908
3.340918	3.325842	3.318958	35.01964
3.255915	3.379825	3.406279	35.14276
3.245233	3.384745	3.422222	35.16502
3.157096	3.453756	3.387437	35.04713
3.314192	3.522902	3.386294	35.53939
3.271877	3.431381	3.430901	35.34426
3.318152	3.356709	3.405784	35.22723
3.269278	3.199884	3.379064	34.71897
3.349455	3.228863	3.40367	35.01148
3.361457	3.286484	3.370863	35.09199
3.387564	3.368233	3.359472	35.30295
3.234675	3.362639	3.426672	35.10333
3.228695	3.361494	3.377223	34.97961
3.248863	3.338782	3.418215	35.06369
3.232819	3.366211	3.318685	34.87093
3.24049	3.417296	3.438319	35.26104
3.170542	3.417565	3.446503	35.12656
3.17846	3.317283	3.456871	34.94725
3.257689	3.255079	3.391069	34.84058
3.32042	3.210667	3.383822	34.86479
3.249605	3.272332	3.373523	34.82226
3.292828	3.299559	3.429759	35.0993
3.24779	3.313712	3.425649	35.02277
3.348631	3.390271	3.38181	35.31485
3.318729	3.367492	3.367094	35.16746
3.268495	3.384205	3.361808	35.0826
3.105583	3.382453	3.416305	34.84168
3.338732	3.470874	3.394939	35.49818
3.247337	3.372546	3.380139	35.05092
3.348383	3.293291	3.365065	35.06561
3.211249	3.216665	3.442616	34.76774
3.328875	3.321799	3.382065	35.12247
3.291426	3.361696	3.414446	35.19863
3.369211	3.417498	3.354561	35.35981
3.266969	3.354013	3.428241	35.15851
3.224447	3.337771	3.361246	34.8835
3.135608	3.322877	3.467374	34.88874
3.274269	3.494731	3.384726	35.38704
3.260658	3.466763	3.481919	35.50867
3.222013	3.365537	3.433578	35.09708
3.16353	3.247868	3.457297	34.76373
3.282435	3.198334	3.451499	34.90275
3.363932	3.223067	3.382406	34.98397
3.354528	3.235265	3.356761	34.934
3.288786	3.303333	3.403874	35.04211
3.265608	3.368166	3.413202	35.1536
3.304376	3.446814	3.398111	35.37737
3.28693	3.380768	3.373813	35.14165
3.325246	3.404963	3.397412	35.32996

3.158127	3.444725	3.426331	35.11469
3.252038	3.396067	3.400259	35.15664
3.286023	3.261481	3.37741	34.88667
3.26503	3.232165	3.359847	34.73825
3.183492	3.228863	3.397872	34.63587
3.325204	3.396673	3.409945	35.33915
3.303387	3.445264	3.473922	35.5376
3.396597	3.388249	3.352208	35.35059
3.340341	3.313846	3.385527	35.13772
3.235995	3.341073	3.395025	34.98984
3.227705	3.448836	3.417806	35.25719
3.245852	3.511916	3.464118	35.5361
3.142166	3.384003	3.383839	34.85407
3.258184	3.256966	3.368561	34.79657
3.197556	3.288237	3.484102	34.98504
3.245646	3.254944	3.441098	34.92335
3.228035	3.242679	3.393371	34.75365
3.323926	3.281497	3.410422	35.08552
3.317698	3.333188	3.402937	35.16857
3.299056	3.389395	3.332667	35.09705
3.248244	3.388721	3.412537	35.15912
3.244161	3.399571	3.365116	35.07022
3.222261	3.429157	3.407626	35.17999
3.267092	3.470469	3.370863	35.28798
3.199288	3.331571	3.39291	34.88417
3.280909	3.202175	3.391052	34.77563
3.31778	3.192875	3.419425	34.89797
3.265731	3.372277	3.344808	35.01329
3.343681	3.379218	3.414054	35.35037
3.309449	3.403952	3.367538	35.22787
3.27864	3.33366	3.424029	35.13032
3.249605	3.354687	3.352191	34.95571
3.381584	3.455171	3.439427	35.65484
3.16683	3.45288	3.40974	35.11527
3.259215	3.390338	3.37475	35.10402
3.129958	3.29437	3.456547	34.79036
3.233438	3.334132	3.390387	34.95893
3.220487	3.235265	3.459872	34.86636
3.295674	3.261481	3.374836	34.90215
3.167366	3.184113	3.466096	34.65194
3.413465	3.363246	3.337902	35.30151
3.338691	3.350239	3.426792	35.30394
3.414331	3.380836	3.358483	35.38688
3.258844	3.333054	3.421949	35.08115
3.214012	3.347611	3.387727	34.94011
3.228324	3.485835	3.420005	35.34427
3.375398	3.404154	3.305112	35.23602
3.242717	3.291674	3.450613	35.01808
3.206588	3.228324	3.369413	34.62296
3.226426	3.227313	3.452778	34.84644

3.321781	3.401054	3.363445	35.23955
3.391854	3.410624	3.366719	35.42088
3.284662	3.335547	3.444611	35.19262
3.25938	3.262021	3.371391	34.81642
3.261236	3.389327	3.3762	35.1094
3.294602	3.486913	3.345558	35.32876
3.245769	3.457058	3.473257	35.43594
3.079765	3.344713	3.439956	34.75441
3.137877	3.287158	3.508315	34.90512
3.327885	3.309398	3.325915	34.97039
3.202876	3.23048	3.407506	34.70287
3.308006	3.245374	3.429315	35.01303
3.154415	3.317823	3.465806	34.91538
3.374531	3.391956	3.410831	35.43864
3.402041	3.37504	3.357051	35.3442
3.460359	3.313846	3.390728	35.41155
3.197638	3.32564	3.458542	35.01112
3.27299	3.396538	3.330399	35.05071
3.19038	3.459417	3.420858	35.20538
3.402041	3.375242	3.352396	35.33446
3.115358	3.234254	3.406552	34.51764
3.142826	3.24652	3.417447	34.62836
3.213558	3.361696	3.410081	35.0188
3.383729	3.445668	3.386686	35.52341
3.347558	3.353137	3.364656	35.19378
3.362406	3.265795	3.388801	35.08805
3.196607	3.32874	3.41622	34.92309
3.362571	3.49554	3.382492	35.57703
3.345661	3.449375	3.310705	35.28211
3.249358	3.334064	3.394752	35.00314
3.196484	3.227245	3.43675	34.74576
3.20465	3.32719	3.432606	34.97312
3.276537	3.346802	3.442019	35.1938
3.258885	3.305287	3.433629	35.04606
3.29885	3.236681	3.422034	34.95807
3.230386	3.325573	3.474331	35.11711
3.326029	3.385081	3.406705	35.30852
3.413589	3.421946	3.331303	35.41572
3.432602	3.385823	3.367231	35.45687
3.119358	3.334469	3.426468	34.7891
3.282724	3.404895	3.476531	35.40984
3.256946	3.408871	3.364536	35.11725
3.374078	3.274556	3.309954	34.96031
3.164685	3.190314	3.375177	34.46081
3.165799	3.283654	3.490428	34.9194
3.186008	3.439064	3.469301	35.25725
3.493972	3.465954	3.376268	35.78608
3.301984	3.301176	3.412485	35.08509
3.371438	3.343769	3.356607	35.20792
3.257854	3.381981	3.431703	35.20731

3.374325	3.455912	3.347178	35.43885
3.23418	3.362909	3.370539	34.98008
3.276207	3.294235	3.33751	34.84958
3.133959	3.239376	3.384231	34.52071
3.230015	3.292752	3.444747	34.97983
3.265525	3.332717	3.410507	35.07001
3.262391	3.246048	3.386362	34.82082
3.303964	3.260471	3.409945	34.99484
3.239459	3.337165	3.432044	35.06983
3.295798	3.416689	3.440365	35.38513
3.322688	3.389193	3.344399	35.17395
3.386286	3.32928	3.368168	35.23398
3.274929	3.375983	3.424916	35.2167
3.233273	3.380162	3.375347	35.02634
3.277527	3.33521	3.382816	35.04115
3.367149	3.276578	3.349923	35.03699
3.224653	3.283991	3.42125	34.89756
3.256163	3.403278	3.444321	35.27778
3.240325	3.424844	3.391461	35.17471
3.329411	3.367896	3.393439	35.24932
3.377666	3.288843	3.339641	35.06432
3.348259	3.364257	3.412196	35.3236
3.256823	3.464876	3.443298	35.41169
3.397586	3.458473	3.420193	35.65499
3.168644	3.306366	3.382117	34.73843
3.288786	3.21343	3.338618	34.7028
3.217971	3.261212	3.44161	34.87766
3.195205	3.418509	3.451431	35.19333
3.094447	3.32126	3.46821	34.79702
3.272949	3.249957	3.339164	34.74924
3.275506	3.274556	3.51459	35.19226
3.411857	3.379083	3.342165	35.34195
3.23253	3.349969	3.483403	35.19499
3.301943	3.409748	3.351424	35.18889
3.364056	3.339389	3.368714	35.20867
3.340299	3.334132	3.345047	35.09347
3.203701	3.311757	3.410013	34.88789
3.255792	3.276915	3.352669	34.80021
3.206217	3.315194	3.459326	35.00875
3.240202	3.382453	3.364059	35.02182
3.356426	3.433942	3.373676	35.40961
3.188317	3.342017	3.43675	34.97889
3.316254	3.303131	3.363667	35.01381
3.286023	3.33238	3.377479	35.04187
3.417796	3.473569	3.379934	35.64416
3.261236	3.448229	3.459053	35.4194
3.284703	3.32874	3.356147	34.98437
3.183327	3.156482	3.404437	34.49158
3.333907	3.269366	3.361433	34.97369
3.247543	3.401054	3.444798	35.25511

3.25109	3.458069	3.407097	35.30511
3.134371	3.29875	3.431293	34.75437
3.22222	3.303737	3.393166	34.87401
3.386575	3.371064	3.430424	35.46213
3.358323	3.366144	3.404488	35.33288
3.251709	3.410287	3.436136	35.26547
3.287302	3.376522	3.42686	35.24918
3.268453	3.343634	3.396031	35.06863
3.417713	3.371266	3.287122	35.21729
3.250018	3.303063	3.375518	34.89473
3.126576	3.255551	3.430355	34.6408
3.104181	3.35927	3.399646	34.75149
3.255998	3.384408	3.434721	35.21516
3.332298	3.455036	3.369584	35.39403
3.275836	3.342286	3.449112	35.1979
3.182502	3.307646	3.45612	34.93337
3.364427	3.394045	3.424098	35.45012
3.456647	3.525463	3.388528	35.8614
3.313038	3.349565	3.375637	35.1345
3.19339	3.186337	3.38464	34.53558
3.186461	3.181418	3.407506	34.55968
3.280538	3.318025	3.394684	35.0361
3.251461	3.482128	3.382748	35.30528
3.244161	3.342152	3.445497	35.12044
3.137299	3.304748	3.388801	34.68096
3.269484	3.338378	3.459735	35.1987
3.303634	3.37969	3.424677	35.28705
3.394906	3.407321	3.416782	35.52981
3.216899	3.379151	3.364536	34.96468
3.320296	3.300772	3.505297	35.32721
3.32706	3.333795	3.341466	35.05595
3.40864	3.299828	3.327449	35.12942
3.183987	3.236007	3.339488	34.5249
3.22057	3.258044	3.41049	34.80836
3.094282	3.406041	3.38123	34.79185
3.294932	3.455441	3.430492	35.44639
3.339186	3.437716	3.39835	35.43412
3.364056	3.379353	3.376677	35.31348
3.197886	3.376253	3.453801	35.11197
3.315595	3.472761	3.353282	35.36061
3.417837	3.410961	3.434704	35.62711
3.378944	3.336221	3.26724	35.0124
3.180811	3.123527	3.402851	34.41054
3.140558	3.218012	3.367452	34.45173
3.298314	3.416217	3.440638	35.3902
3.305614	3.427742	3.381759	35.30261
3.190792	3.287024	3.419698	34.82675
3.16918	3.269501	3.489917	34.89473
3.183533	3.335075	3.46144	35.00724
3.457183	3.415139	3.467391	35.79377

3.34661	3.486105	3.350486	35.4515
3.247131	3.396336	3.462259	35.28208
3.357952	3.299963	3.340119	35.04657
3.276496	3.269636	3.456615	35.05688
3.372923	3.286889	3.307908	34.98028
3.232283	3.355361	3.409331	35.04426
3.260493	3.408804	3.377138	35.15242
3.153673	3.376927	3.407558	34.91563
3.368221	3.388114	3.402033	35.3972
3.266391	3.353811	3.402647	35.10084
3.313656	3.390945	3.422819	35.32952
3.168314	3.438457	3.411445	35.09071
3.371644	3.474446	3.451158	35.70091
3.326483	3.276645	3.384981	35.02487
3.368015	3.158504	3.355738	34.79339
3.272536	3.211408	3.406807	34.81196
3.280373	3.358731	3.38684	35.10761
3.169923	3.441086	3.441592	35.1659
3.226633	3.335547	3.377206	34.91832
3.152931	3.242005	3.361314	34.51783
3.250884	3.232367	3.431344	34.86411
3.21859	3.380701	3.438608	35.13375
3.350033	3.490687	3.474945	35.74118
3.310274	3.461371	3.412775	35.45417
3.418827	3.330493	3.330399	35.2252
3.355271	3.177711	3.365747	34.82941
3.221766	3.262829	3.397003	34.79195
3.236696	3.315666	3.385254	34.91445
3.164437	3.443849	3.383532	35.03298
3.318193	3.430909	3.381366	35.33619
3.250801	3.360281	3.373318	35.01676
3.252575	3.33056	3.401965	35.01829
3.235706	3.378342	3.456956	35.20615
3.265484	3.458069	3.379116	35.27539
3.29489	3.467639	3.391819	35.38841
3.340341	3.347948	3.428309	35.30585
3.24478	3.166456	3.415401	34.67176
3.266144	3.225156	3.372295	34.75258
3.332669	3.367087	3.3526	35.16537
3.283177	3.472491	3.430952	35.45898
3.188152	3.358865	3.382458	34.89665
3.337124	3.292213	3.415811	35.1496
3.191411	3.334267	3.41431	34.91963
3.347929	3.390271	3.348696	35.2409
3.246966	3.363987	3.480879	35.25169
3.26404	3.412106	3.463981	35.35731
3.230179	3.345184	3.431293	35.06543
3.344877	3.250361	3.342642	34.91503
3.401175	3.239646	3.452932	35.25589
3.30714	3.287091	3.347212	34.92282

3.180399	3.345791	3.441882	34.98105
3.21727	3.433403	3.314081	34.97379
3.368922	3.410354	3.330007	35.28986
3.317327	3.356102	3.342165	35.08497
3.218673	3.393371	3.407575	35.09377
3.273815	3.427405	3.370658	35.20806
3.285528	3.454632	3.479652	35.53156
3.427241	3.394382	3.354357	35.43571
3.353951	3.211004	3.362422	34.89206
3.204897	3.164367	3.409484	34.56703
3.253853	3.274488	3.43218	34.96454
3.291302	3.404626	3.362354	35.17833
3.41961	3.405906	3.335958	35.40399
3.143651	3.209588	3.409672	34.5324
3.198298	3.243892	3.366531	34.63258
3.234799	3.312701	3.446776	35.03835
3.345414	3.442636	3.351577	35.35621
3.192772	3.401323	3.430952	35.10565
3.33898	3.420261	3.422375	35.44804
3.251544	3.284665	3.394667	34.89971
3.467742	3.276982	3.348457	35.25464
3.380264	3.295852	3.422699	35.26697
3.268618	3.37969	3.373045	35.09757
3.2355	3.38151	3.387317	35.06033
3.315388	3.403817	3.333025	35.16509
3.219456	3.344915	3.364383	34.89507
3.291385	3.383734	3.2743	34.94026
3.192071	3.384408	3.495612	35.20852
3.152972	3.459754	3.435659	35.15668
3.27563	3.373288	3.397275	35.15189
3.311017	3.230076	3.346786	34.80568
3.284208	3.174678	3.404846	34.75288
3.270474	3.299155	3.438949	35.06963
3.224158	3.437581	3.437244	35.26733
3.318152	3.46063	3.413628	35.47164
3.345744	3.331975	3.385032	35.1881
3.250595	3.199816	3.369038	34.65604
3.160148	3.272467	3.379388	34.63975
3.20997	3.441288	3.457297	35.28826
3.285487	3.493653	3.507616	35.67796
3.247089	3.419452	3.392961	35.181
3.337124	3.182159	3.400413	34.87526
3.230015	3.114024	3.357801	34.39884
3.29951	3.256966	3.41003	34.97763
3.297901	3.370659	3.339147	35.06773
3.373377	3.502616	3.428974	35.71779
3.258967	3.439536	3.34005	35.13518
3.230097	3.307714	3.417243	34.95258
3.282105	3.378477	3.354527	35.08391
3.29687	3.468852	3.426178	35.47053

3.179079	3.502279	3.383549	35.19281
3.307841	3.362639	3.412332	35.23197
3.114244	3.233783	3.463572	34.63887
3.368015	3.146912	3.377666	34.81599
3.327555	3.230413	3.448976	35.06606
3.394534	3.445062	3.381776	35.53498
3.291756	3.41278	3.439614	35.36611
3.326978	3.337299	3.421762	35.23903
3.258885	3.226976	3.393865	34.78785
3.375439	3.320316	3.356676	35.16553
3.166788	3.444051	3.434226	35.14942
3.090117	3.446814	3.44444	35.01013
3.254678	3.38306	3.481289	35.31116
3.318729	3.2354	3.35405	34.85007
3.382986	3.20359	3.424422	35.07492
3.278311	3.269906	3.375705	34.8845
3.217518	3.30495	3.43617	34.96042
3.360179	3.427944	3.33444	35.3189
3.384677	3.430909	3.343938	35.39973
3.335515	3.344376	3.346394	35.10835
3.229025	3.317418	3.376677	34.88275
3.267464	3.435627	3.458235	35.40366
3.260205	3.51286	3.381452	35.38877
3.314275	3.393371	3.413185	35.31511
3.272825	3.250496	3.445429	34.98253
3.119111	3.155336	3.46642	34.48419
3.306521	3.211677	3.370249	34.80692
3.268866	3.436301	3.401658	35.28448
3.319554	3.472761	3.370181	35.40622
3.213063	3.345387	3.426792	35.0186
3.27398	3.261616	3.410286	34.93252
3.26536	3.265592	3.379269	34.85454
3.33036	3.376253	3.343512	35.16049
3.186049	3.442501	3.430287	35.17954
3.227746	3.46063	3.453597	35.36134
3.13433	3.276038	3.433203	34.70879
3.380058	3.229132	3.427201	35.13045
3.327803	3.220169	3.343563	34.81368
3.341825	3.32409	3.398435	35.1916
3.278805	3.442029	3.383839	35.27978
3.391524	3.390338	3.366361	35.37501
3.385708	3.397684	3.352396	35.34782
3.458338	3.387642	3.326614	35.42831
3.200732	3.412376	3.396525	35.07194
3.212486	3.483813	3.409621	35.2825
3.254389	3.458338	3.444184	35.39401
3.251791	3.273478	3.326324	34.72633
3.299592	3.175689	3.385953	34.74741
3.144682	3.191325	3.393865	34.46014
3.204361	3.409478	3.422716	35.13081

3.389338	3.491227	3.429315	35.72853
3.368056	3.362505	3.367862	35.26611
3.26569	3.344174	3.343989	34.94996
3.26767	3.21653	3.411582	34.82296
3.309862	3.348891	3.381179	35.1382
3.308006	3.468447	3.382202	35.39783
3.269196	3.478354	3.411445	35.39857
3.231376	3.372614	3.465737	35.20335
3.235335	3.196514	3.379968	34.63935
3.331515	3.199951	3.356335	34.80551
3.360591	3.272534	3.411701	35.1489
3.27134	3.419183	3.392143	35.23165
3.262597	3.390743	3.450204	35.2773
3.412187	3.404019	3.302247	35.30991
3.295963	3.314925	3.380889	35.03289
3.3443	3.399975	3.344876	35.24583
3.218136	3.48105	3.458576	35.39587
3.214136	3.524115	3.379337	35.30802
3.115523	3.344106	3.404011	34.75267
3.237108	3.262492	3.37886	34.78508
3.187121	3.18317	3.476173	34.71511
3.264824	3.321866	3.393439	35.00742
3.227251	3.441894	3.457092	35.32693
3.316461	3.411702	3.322914	35.16257
3.463411	3.346734	3.377768	35.46181
3.327638	3.234457	3.397599	34.96273
3.229355	3.288978	3.425087	34.92714
3.231994	3.425855	3.372789	35.11787
3.268907	3.479365	3.439853	35.46227
3.289817	3.439536	3.3378	35.19772
3.379439	3.249822	3.412076	35.14127
3.176811	3.184046	3.410576	34.55103
3.270845	3.221719	3.380087	34.77238
3.239336	3.389934	3.469438	35.26673
3.347311	3.418307	3.350554	35.30492
3.344053	3.393371	3.376797	35.30065
3.371644	3.319844	3.354169	35.15072
3.275176	3.361965	3.407302	35.14806
3.365912	3.476265	3.304975	35.37267
3.273691	3.398628	3.422273	35.25773
3.309697	3.352328	3.343921	35.06388
3.088591	3.191864	3.409808	34.37353
3.17116	3.201703	3.345081	34.43406
3.228571	3.33029	3.422853	35.01088
3.281115	3.47667	3.43397	35.47021
3.216115	3.444725	3.453801	35.30158
3.370366	3.342691	3.451448	35.41062
3.356591	3.280554	3.356488	35.03695
3.442954	3.346869	3.422563	35.51533
3.231334	3.364324	3.419988	35.08509

3.179904	3.352126	3.413491	34.93174
3.195453	3.347476	3.393166	34.91112
3.260122	3.315396	3.376933	34.94689
3.300128	3.236546	3.380105	34.86888
3.189142	3.169893	3.408717	34.54298
3.181718	3.277049	3.45948	34.87209
3.33568	3.402469	3.434721	35.42891
3.416146	3.470065	3.365406	35.60112
3.313285	3.357113	3.380343	35.16184
3.367108	3.37996	3.351628	35.2667
3.292869	3.407321	3.403141	35.27684
3.358199	3.463191	3.336828	35.39687
3.399442	3.423496	3.392211	35.52137
3.255915	3.333727	3.427764	35.08894
3.012002	3.159987	3.347468	34
3.255049	3.211475	3.41876	34.80001
3.267175	3.413319	3.396508	35.21926
3.27167	3.484285	3.443298	35.48661
3.196937	3.350576	3.381111	34.89478
3.3005	3.322877	3.476241	35.26872
3.332876	3.332514	3.353964	35.0932
3.344547	3.335547	3.413236	35.25497
3.262391	3.338782	3.385595	35.02194
3.237438	3.356574	3.425257	35.09301
3.164025	3.332784	3.400686	34.82671
3.290271	3.288102	3.320254	34.82919
3.27864	3.224752	3.410149	34.8618
3.240697	3.270445	3.405187	34.8679
3.151941	3.391214	3.4796	35.10063
3.303964	3.478152	3.398486	35.44582
3.425921	3.399639	3.359216	35.45495
3.516862	3.36702	3.281802	35.4132
3.307841	3.351452	3.374887	35.12562
3.267299	3.389125	3.321618	35.00285
3.307181	3.403615	3.410968	35.31715
3.314275	3.2879	3.288469	34.81173
3.276661	3.174207	3.399322	34.72326
3.111151	3.20514	3.425786	34.48684
3.144269	3.38771	3.464527	35.04323
3.349414	3.493248	3.387488	35.55417
3.280167	3.451127	3.443792	35.43376
3.23253	3.342556	3.405187	35.00773
3.28359	3.325303	3.419715	35.11343
3.292787	3.374905	3.436	35.27763
3.402494	3.356911	3.328387	35.24286
3.261566	3.350374	3.433271	35.14974
3.344795	3.328808	3.385152	35.17936
3.227911	3.225763	3.344671	34.60989
3.349208	3.258381	3.323067	34.89923
3.143692	3.262627	3.418948	34.66876

3.283425	3.394314	3.369294	35.15373
3.188812	3.38434	3.520302	35.25524
3.3841	3.456519	3.315564	35.39242
3.347187	3.370727	3.3991	35.30676
3.410578	3.375175	3.295716	35.22903
3.310728	3.388653	3.426536	35.32623
3.275795	3.431179	3.400328	35.28552
3.236613	3.307579	3.371766	34.86709
3.327638	3.185124	3.361399	34.77568
3.209145	3.204332	3.347263	34.52765
3.230839	3.262425	3.450323	34.92751
3.258472	3.482533	3.426041	35.41618
3.31609	3.433133	3.460093	35.50861
3.336546	3.366548	3.450272	35.38626
3.279465	3.277184	3.360632	34.86998
3.320626	3.328269	3.39794	35.15329
3.309202	3.356102	3.397224	35.18761
3.254678	3.371872	3.425428	35.16454
3.214548	3.310207	3.39141	34.86754
3.28095	3.256494	3.311864	34.72133
3.158251	3.20514	3.396713	34.52626
3.243666	3.274623	3.437244	34.95363
3.149384	3.3466	3.499841	35.04174
3.249729	3.44331	3.375739	35.20128
3.311759	3.428618	3.38771	35.33098
3.396019	3.436099	3.413099	35.58712
3.282187	3.382116	3.360939	35.10607
3.419487	3.395056	3.382662	35.48213
3.301118	3.433201	3.371272	35.28178
3.388595	3.40011	3.399458	35.46236
3.239583	3.22428	3.380343	34.71018
3.275094	3.14954	3.379184	34.62185
3.130453	3.274286	3.365747	34.54896
3.296251	3.444658	3.384248	35.32457
3.167819	3.434346	3.478509	35.2273
3.269649	3.452947	3.422614	35.36842
3.182791	3.361696	3.461713	35.06443
3.357622	3.373355	3.412008	35.36356
3.321162	3.384408	3.399646	35.28096
3.348796	3.300031	3.395843	35.14856
3.191534	3.287967	3.459411	34.91728
3.252368	3.234187	3.383344	34.76636
3.243831	3.251979	3.382662	34.78511
3.27299	3.269703	3.315701	34.7412
3.144104	3.31762	3.449385	34.85648
3.176151	3.378814	3.391614	34.93405
3.318894	3.440547	3.390046	35.37777
3.415486	3.435155	3.351987	35.49399
3.374614	3.377399	3.441933	35.475
3.307181	3.408871	3.350793	35.19705

3.269608	3.422283	3.410166	35.27406
3.406124	3.422283	3.338124	35.41505
3.433881	3.2447	3.303151	35.01092
3.170789	3.135455	3.385391	34.37653
3.243501	3.217339	3.442599	34.83971
3.186833	3.39701	3.405528	35.02763
3.284909	3.450453	3.365969	35.27247
3.235211	3.371805	3.41622	35.10169
3.247749	3.32254	3.456342	35.10911
3.160561	3.328201	3.468346	34.95707
3.406413	3.439536	3.341483	35.46075
3.270598	3.359472	3.445293	35.21568
3.401917	3.301513	3.355789	35.18038
3.197061	3.278128	3.473325	34.93828
3.2282	3.234726	3.290516	34.51169
3.314399	3.189101	3.357716	34.74737
3.319719	3.307242	3.346513	34.99286
3.127731	3.304479	3.43131	34.75241
3.244697	3.415678	3.401436	35.18604
3.248038	3.449308	3.414054	35.29449
3.43619	3.43529	3.418419	35.68484
3.355642	3.398425	3.390233	35.36643
3.309449	3.374298	3.472149	35.39179
3.296458	3.418981	3.37579	35.25037
3.376016	3.355024	3.3629	35.2563
3.336835	3.178183	3.359506	34.77648
3.241109	3.154191	3.412963	34.63157
3.217064	3.285474	3.370709	34.77368
3.226385	3.421339	3.399936	35.1551
3.364221	3.398358	3.338652	35.27225
3.234922	3.369783	3.43559	35.13899
3.279218	3.383666	3.376779	35.13761
3.172439	3.355428	3.456393	35.01645
3.310192	3.39573	3.366873	35.21006
3.39272	3.352126	3.455268	35.48849
3.311264	3.287226	3.365355	34.97181
3.203577	3.217339	3.395502	34.64941
3.32871	3.237152	3.419306	35.01844
3.240449	3.347543	3.387573	34.99744
3.281445	3.339321	3.369737	35.0301
3.150085	3.352059	3.363888	34.75791
3.239418	3.363179	3.462822	35.19393
3.274104	3.452003	3.341005	35.19764
3.335969	3.369244	3.394939	35.26989
3.28924	3.414465	3.380087	35.23411
3.273361	3.425181	3.406927	35.28152
3.234469	3.308522	3.425649	34.98229
3.341372	3.246116	3.327074	34.86403
3.391524	3.17387	3.372653	34.91539
3.216445	3.265862	3.377427	34.74414

3.175367	3.422889	3.41078	35.07063
3.251255	3.439873	3.383907	35.21496
3.321864	3.378005	3.426587	35.32741
3.332339	3.409613	3.371357	35.29866
3.254348	3.365942	3.464118	35.23546
3.156271	3.382655	3.422103	34.96565
3.271175	3.336962	3.422989	35.11894
3.292168	3.263436	3.352328	34.84953
3.340506	3.229469	3.424694	35.03922
3.23385	3.258314	3.339573	34.68291
3.287632	3.213699	3.440365	34.92337
3.194174	3.303468	3.346325	34.70965
3.297283	3.368974	3.368066	35.12593
3.174955	3.405502	3.416629	35.0445
3.300912	3.482196	3.439291	35.53722
3.157632	3.423429	3.417328	35.04735
3.33436	3.406243	3.340119	35.2274
3.301201	3.377129	3.394479	35.21009
3.37878	3.364998	3.340596	35.23538
3.29188	3.295313	3.396576	35.01538
3.319059	3.243083	3.29749	34.74391
3.27134	3.168748	3.4109	34.72501
3.312006	3.378612	3.297899	35.02576
3.172274	3.4239	3.412809	35.07052
3.27497	3.430235	3.372721	35.22128
3.277279	3.394112	3.43305	35.27927
3.215579	3.423159	3.352174	35.031
3.275052	3.384812	3.456478	35.3053
3.211455	3.350711	3.485057	35.15414
3.187575	3.319642	3.466982	34.99445
3.333824	3.251575	3.384913	34.98595
3.356549	3.275095	3.392825	35.10438
3.290931	3.21316	3.38285	34.80363
3.266762	3.304411	3.415077	35.02081
3.151858	3.333121	3.411786	34.82512
3.322977	3.402873	3.388818	35.30163
3.294395	3.443377	3.384026	35.31723
3.334154	3.469998	3.37874	35.45083
3.185348	3.391282	3.418164	35.03949
3.290766	3.398762	3.353368	35.14468
3.29819	3.332312	3.414259	35.14876
3.416022	3.354687	3.35792	35.33216
3.225849	3.242476	3.442974	34.8569
3.321616	3.228256	3.32305	34.77297
3.23649	3.225021	3.406193	34.76156
3.339475	3.412039	3.358824	35.29216
3.240655	3.408669	3.455387	35.27986
3.155653	3.382857	3.429401	34.9807
3.160066	3.363111	3.395008	34.87195
3.24049	3.448095	3.421062	35.29065

3.2866	3.491294	3.496447	35.65081
3.262556	3.34323	3.457911	35.19017
3.217229	3.285608	3.444883	34.93655
3.289034	3.195368	3.401231	34.80077
3.413053	3.302052	3.301974	35.08822
3.285734	3.243622	3.398776	34.89371
3.223704	3.343769	3.393575	34.96569
3.217394	3.336693	3.374341	34.89436
3.2972	3.318833	3.36943	35.01908
3.294808	3.42262	3.346377	35.1904
3.352672	3.457328	3.455933	35.63242
3.190545	3.461439	3.468534	35.31442
3.294602	3.425652	3.428923	35.3771
3.278723	3.306163	3.440638	35.10669
3.370861	3.289382	3.380224	35.13937
3.233314	3.242611	3.377342	34.72999
3.262267	3.270108	3.443775	34.99872
3.246512	3.383329	3.407575	35.13269
3.365953	3.41487	3.332207	35.29805
3.291591	3.355091	3.362678	35.07134
3.250018	3.333121	3.425564	35.0699
3.116719	3.359	3.388443	34.75382
3.224859	3.43192	3.409774	35.19641
3.253894	3.453351	3.435812	35.36372
3.259751	3.348217	3.43334	35.14121
3.216899	3.196918	3.441064	34.73352
3.384141	3.206286	3.403482	35.03755
3.285074	3.227852	3.430492	34.92714
3.383193	3.342623	3.359421	35.23727
3.251214	3.362437	3.396167	35.07234
3.225436	3.337839	3.324585	34.80564
3.212857	3.373355	3.363513	34.94093
3.288415	3.455845	3.406603	35.38078
3.263752	3.460293	3.403261	35.32927
3.24214	3.385149	3.415401	35.14423
3.216404	3.270377	3.426587	34.86142
3.317079	3.256966	3.410712	35.01754
3.413383	3.284934	3.362627	35.18415
3.251544	3.296391	3.392552	34.92073
3.319307	3.348217	3.395025	35.18766
3.28825	3.40476	3.354169	35.15405
3.354033	3.371333	3.346649	35.20836
3.355848	3.348352	3.32189	35.10793
3.291921	3.430977	3.443502	35.41476
3.049946	3.410152	3.411616	34.77033
3.272454	3.394854	3.435454	35.2756
3.234758	3.302389	3.355925	34.81704
3.239212	3.182968	3.402255	34.66694
3.192401	3.201097	3.376473	34.54783
3.256946	3.266738	3.441814	34.97542

3.290106	3.291337	3.39262	34.99415
3.346445	3.405367	3.389142	35.35912
3.26338	3.385823	3.423859	35.21065
3.325493	3.347678	3.305316	35.00383
3.187864	3.396673	3.412349	35.04406
3.270763	3.49372	3.302162	35.19661
3.24148	3.451397	3.394888	35.2428
3.274887	3.359539	3.319759	34.95068
3.187245	3.238635	3.405034	34.68111
3.282641	3.25272	3.376319	34.85773
3.264865	3.289989	3.439018	35.03747
3.421136	3.380027	3.335259	35.34921
3.329411	3.364998	3.41078	35.28091
3.280125	3.379757	3.374205	35.12542
3.288539	3.374096	3.39644	35.18006
3.272083	3.371333	3.352106	35.04108
3.229891	3.446342	3.407353	35.23366
3.265855	3.41588	3.433442	35.30275
3.177182	3.401863	3.444611	35.1026
3.138743	3.224887	3.438472	34.6181
3.304005	3.235333	3.36411	34.83973
3.186874	3.206488	3.415538	34.63297
3.31477	3.238635	3.32143	34.77716
3.240614	3.331369	3.46601	35.13396
3.394163	3.410017	3.373915	35.44034
3.346445	3.377331	3.378723	35.27502
3.448357	3.421609	3.347553	35.52655
3.191039	3.385216	3.475576	35.16422
3.278599	3.44331	3.323459	35.15009
3.110697	3.380566	3.429759	34.87816
3.359024	3.307916	3.317935	35.01779
3.198463	3.233783	3.424149	34.73683
3.238964	3.27058	3.434704	34.92895
3.263917	3.289517	3.355653	34.85206
3.357539	3.298885	3.371408	35.11174
3.34331	3.363179	3.369652	35.21738
3.331391	3.340602	3.386755	35.17934
3.20102	3.376927	3.394121	34.98979
3.277939	3.437851	3.397122	35.29779
3.303469	3.455777	3.334048	35.25489
3.241604	3.381914	3.418078	35.14184
3.238758	3.262695	3.425905	34.89202
3.208362	3.216732	3.407012	34.68371
3.286559	3.231222	3.356386	34.7757
3.273568	3.270782	3.390779	34.90901
3.285858	3.301648	3.375569	34.97012
3.326813	3.331167	3.448362	35.28342
3.330195	3.399436	3.37504	35.27977
3.307511	3.362909	3.329257	35.05017
3.384306	3.436907	3.416902	35.57159

3.175285	3.458743	3.429401	35.18958
3.159076	3.446949	3.428378	35.1261
3.237974	3.341343	3.391069	34.98611
3.33069	3.246116	3.359233	34.911
3.211537	3.253125	3.364519	34.67732
3.305036	3.351452	3.441814	35.26584
3.250925	3.404693	3.455183	35.29318
3.404887	3.356237	3.367179	35.33145
3.365128	3.312566	3.377888	35.17242
3.347517	3.378949	3.40205	35.33192
3.271052	3.359674	3.401402	35.12113
3.251832	3.415409	3.384299	35.16358
3.110532	3.38434	3.415896	34.85574
3.307263	3.38124	3.354561	35.14505
3.193927	3.238905	3.474894	34.84908
3.196772	3.253866	3.416765	34.76091
3.187121	3.242611	3.441251	34.76874
3.337124	3.316138	3.390659	35.14692
3.317409	3.301918	3.436682	35.17335
3.418909	3.417026	3.337851	35.43091
3.244491	3.342556	3.442752	35.11604
3.272784	3.373422	3.347655	35.03745
3.372799	3.427135	3.345763	35.36949
3.315347	3.395797	3.374563	35.2383
3.247873	3.320047	3.406944	34.9959
3.213971	3.227043	3.383259	34.66659
3.252121	3.268019	3.391273	34.85715
3.262597	3.318092	3.349582	34.89839
3.354817	3.382116	3.491349	35.55009
3.221436	3.386969	3.430219	35.13534
3.28128	3.325708	3.368697	34.9977
3.294272	3.347611	3.40974	35.16376
3.48395	3.459552	3.381554	35.76172
3.256122	3.414263	3.452113	35.31876
3.172439	3.364863	3.374	34.8569
3.175037	3.197794	3.430168	34.62006
3.266102	3.258449	3.351168	34.77909
3.167819	3.248003	3.45878	34.77665
3.251379	3.256696	3.372704	34.79016
3.130123	3.28116	3.497112	34.85055
3.349332	3.363852	3.391938	35.28076
3.495292	3.432998	3.392433	35.75224
3.435489	3.378477	3.350264	35.41002
3.259751	3.331638	3.429247	35.096
3.258555	3.392225	3.42495	35.21648
3.301654	3.400986	3.387778	35.2486
3.394617	3.411432	3.288572	35.25779
3.249523	3.242274	3.343324	34.69031
3.054854	3.20696	3.443246	34.40589
3.256864	3.378746	3.385527	35.0971

3.297447	3.389193	3.406534	35.25463
3.389008	3.452812	3.38077	35.53764
3.236572	3.281497	3.461099	35.00532
3.185307	3.301379	3.394973	34.79208
3.380759	3.439873	3.405375	35.54511
3.412723	3.483881	3.398503	35.69622
3.263092	3.373894	3.397275	35.1258
3.20465	3.23048	3.37932	34.6451
3.204815	3.249283	3.377632	34.68289
3.343351	3.260268	3.41119	35.08326
3.261071	3.279206	3.347434	34.80531
3.221312	3.300368	3.448379	34.9854
3.138495	3.301176	3.41992	34.74382
3.326235	3.381105	3.421403	35.33242
3.410908	3.400515	3.323272	35.34543
3.444604	3.361292	3.382134	35.46206
3.212775	3.334401	3.39661	34.92794
3.314151	3.446814	3.444542	35.50028
3.259504	3.405434	3.30651	34.98843
3.340794	3.280082	3.345626	34.97762
3.102737	3.198401	3.400174	34.39769
3.182007	3.351924	3.426263	34.96382
3.148889	3.416959	3.440706	35.0652
3.389998	3.432729	3.328183	35.38089
3.334773	3.343163	3.48313	35.4031
3.334649	3.322675	3.335276	35.03469
3.202134	3.428887	3.490701	35.31706
3.370655	3.527687	3.372704	35.6436
3.362612	3.391484	3.41165	35.41333
3.357209	3.244835	3.348099	34.94184
3.096633	3.175217	3.366259	34.25948
3.245687	3.263908	3.416305	34.88883
3.302438	3.297065	3.39008	35.02809
3.217765	3.319777	3.43438	34.98947
3.302026	3.32254	3.454569	35.22393
3.223168	3.400717	3.472609	35.26189
3.313574	3.418846	3.340187	35.20965
3.38311	3.366211	3.361382	35.29296
3.312625	3.338243	3.394803	35.15075
3.215043	3.409545	3.3954	35.09458
3.349703	3.445197	3.426519	35.53508
3.285404	3.295246	3.36943	34.94171
3.343764	3.216867	3.285537	34.71446
3.119028	3.247801	3.488587	34.73469
3.283755	3.395932	3.35185	35.11984
3.2176	3.454025	3.47781	35.37766
3.309367	3.457867	3.397872	35.41193
3.344259	3.292213	3.4146	35.16256
3.306191	3.386362	3.415197	35.2865
3.271877	3.451195	3.438199	35.40355

3.38876	3.448095	3.465533	35.71215
3.283177	3.285406	3.388869	34.95783
3.355312	3.173196	3.339573	34.76239
3.143156	3.27186	3.393865	34.63293
3.134618	3.287024	3.430083	34.72662
3.15788	3.249553	3.378195	34.58207
3.213105	3.295583	3.393149	34.8362
3.279672	3.385216	3.481834	35.37173
3.243336	3.434683	3.416169	35.25685
3.231458	3.411702	3.46497	35.28734
3.353291	3.390945	3.41356	35.39595
3.391565	3.33029	3.361331	35.23279
3.293859	3.451734	3.396866	35.36241
3.32706	3.341747	3.328302	35.04455
3.267381	3.266738	3.364059	34.8282
3.270804	3.190246	3.353606	34.64556
3.21261	3.38744	3.415947	35.08586
3.291178	3.440007	3.447936	35.44258
3.180316	3.410893	3.378161	34.98389
3.286436	3.252181	3.392348	34.8999
3.313326	3.286417	3.35451	34.95083
3.365623	3.45598	3.437824	35.6182
3.270433	3.521217	3.444559	35.56742
3.372758	3.335412	3.413287	35.31648
3.226921	3.231356	3.389636	34.71828
3.309284	3.254338	3.337731	34.83515
3.201515	3.303603	3.408291	34.86151
3.250389	3.311825	3.380531	34.92566
3.070526	3.275971	3.444116	34.59298
3.235541	3.348824	3.371067	34.95341
3.268247	3.377601	3.400345	35.15189
3.354652	3.353811	3.412826	35.31611
3.250842	3.368974	3.405375	35.10596
3.301077	3.353474	3.446111	35.271
3.379934	3.394449	3.388136	35.40628
3.421013	3.352463	3.322214	35.26013
3.302273	3.267008	3.356079	34.88765
3.154085	3.237287	3.334696	34.45183
3.252616	3.348015	3.342966	34.92753
3.212898	3.452138	3.376251	35.14116
3.304748	3.454227	3.366037	35.32425
3.210506	3.369783	3.444116	35.10424
3.179863	3.302592	3.427832	34.85468
3.403732	3.405165	3.42258	35.55707
3.360179	3.555857	3.391734	35.72392
3.311017	3.383599	3.408308	35.27595
3.298809	3.187079	3.438591	34.88572
3.205557	3.173196	3.40089	34.56899
3.350651	3.285945	3.398009	35.12655
3.29654	3.414398	3.381042	35.25202

3.26866	3.336693	3.450084	35.1721
3.129092	3.27988	3.382065	34.5939
3.315471	3.336962	3.379252	35.12016
3.274186	3.404019	3.367674	35.1512
3.368221	3.399369	3.40292	35.42375
3.134165	3.390069	3.451875	34.99862
3.297406	3.386564	3.424984	35.28914
3.313656	3.350711	3.380429	35.14883
3.410372	3.282778	3.317611	35.0744
3.301572	3.215182	3.316468	34.68616
3.220281	3.245846	3.368441	34.6891
3.075022	3.37969	3.468074	34.88202
3.374408	3.514006	3.29795	35.45842
3.240532	3.419789	3.445412	35.28209
3.274681	3.358057	3.330365	34.97018
3.167036	3.365739	3.481612	35.08234
3.358735	3.537728	3.38996	35.67724
3.389214	3.461102	3.436852	35.67886
3.400556	3.275432	3.38331	35.18055
3.263133	3.130401	3.434857	34.67559
3.209104	3.220573	3.412673	34.70612
3.300788	3.384273	3.361791	35.15333
3.21925	3.335951	3.43941	35.03909
3.31778	3.331369	3.373813	35.10109
3.127566	3.334401	3.516909	35.00468
3.258514	3.418172	3.401061	35.22089
3.331597	3.428685	3.449249	35.50908
3.320131	3.358663	3.427065	35.28237
3.202381	3.30987	3.437909	34.94188
3.325534	3.381847	3.459667	35.41618
3.307181	3.345387	3.329649	35.01198
3.465102	3.260336	3.260914	35.02103
3.258019	3.162345	3.366446	34.58466
3.228241	3.310477	3.357494	34.8239
3.198175	3.367963	3.391273	34.95774
3.264865	3.433335	3.304703	35.05722
3.235789	3.407187	3.437091	35.22596
3.280496	3.327932	3.382185	35.03034
3.212445	3.419857	3.508997	35.35987
3.281899	3.515825	3.429725	35.54827
3.280043	3.382992	3.436272	35.26804
3.349909	3.227178	3.451005	35.11231
3.285157	3.18445	3.337237	34.62847
3.299097	3.248879	3.399202	34.93536
3.212404	3.372883	3.353334	34.91664
3.223828	3.277252	3.382867	34.79708
3.238304	3.282306	3.403209	34.88428
3.258555	3.325303	3.397753	35.01066
3.253358	3.381173	3.42715	35.18576
3.322152	3.391686	3.365474	35.22432

3.360096	3.383936	3.398793	35.36321
3.259132	3.389327	3.41622	35.19232
3.38975	3.437312	3.374273	35.49116
3.350033	3.377399	3.333707	35.18457
3.364097	3.21006	3.317525	34.814
3.345661	3.235467	3.323681	34.84271
3.322895	3.336962	3.425786	35.23816
3.169923	3.40995	3.410013	35.02875
3.304954	3.379555	3.377121	35.18565
3.193267	3.315868	3.367469	34.78103
3.241645	3.384408	3.446674	35.20991
3.240119	3.531124	3.407387	35.44151
3.343764	3.493248	3.498681	35.78498
3.186132	3.276712	3.361194	34.66607
3.351064	3.224348	3.388681	34.97235
3.275671	3.280554	3.432197	35.02555
3.358446	3.433875	3.402868	35.47772
3.227499	3.355091	3.441456	35.10346
3.216775	3.289315	3.31079	34.65042
3.202051	3.293898	3.411462	34.8484
3.306645	3.37969	3.334781	35.09705
3.207413	3.38926	3.48533	35.2302
3.255709	3.442838	3.382679	35.2285
3.216198	3.354013	3.544226	35.30113
3.452069	3.294504	3.275885	35.09999
3.388637	3.193144	3.417874	35.05012
3.274145	3.247464	3.387505	34.85212
3.265401	3.340332	3.410149	35.08561
3.28425	3.457934	3.3607	35.27586
3.336917	3.434953	3.303202	35.21504
3.317203	3.313375	3.378314	35.07032
3.166293	3.30832	3.372926	34.71747
3.241027	3.358461	3.441098	35.13963
3.312501	3.498707	3.412332	35.53972
3.246141	3.428685	3.414668	35.24658
3.359189	3.225628	3.377103	34.9676
3.1811	3.175891	3.462054	34.65515
3.322441	3.249148	3.386141	34.95844
3.365045	3.429494	3.404539	35.48623
3.34661	3.443781	3.387232	35.43931
3.207784	3.250766	3.432538	34.8127
3.283136	3.272399	3.345558	34.83458
3.259297	3.344039	3.402323	35.06325
3.30351	3.456654	3.343529	35.27763
3.182708	3.478422	3.468346	35.33402
3.139691	3.375916	3.439546	34.9528
3.225189	3.279745	3.457416	34.96854
3.35226	3.314183	3.405256	35.20767
3.352631	3.25737	3.363036	34.99191
3.413589	3.287361	3.423722	35.32351

3.205186	3.364729	3.434107	35.05967
3.341495	3.404828	3.345763	35.25225
3.422456	3.376725	3.339078	35.35322
3.33931	3.362168	3.33132	35.12259
3.131072	3.409141	3.409621	34.94117
3.200525	3.467908	3.401896	35.20467
3.299262	3.483813	3.470887	35.60625
3.356467	3.33238	3.362473	35.1631
3.330401	3.140442	3.452932	34.88418
3.196772	3.199075	3.39644	34.59664
3.304335	3.347813	3.384726	35.13151
3.348342	3.385553	3.397275	35.33772
3.353085	3.345454	3.373113	35.20756
3.168809	3.227245	3.379951	34.56103
3.294643	3.358394	3.377138	35.11686
3.28095	3.462382	3.422921	35.41444
3.308006	3.413319	3.304788	35.10798
3.179656	3.386497	3.388511	34.95173
3.241191	3.278734	3.475781	35.04148
3.164066	3.275028	3.372568	34.63901
3.498014	3.318227	3.409484	35.5445
3.325823	3.243487	3.363565	34.90408
3.332133	3.31944	3.385459	35.13186
3.277403	3.346061	3.409399	35.12274
3.362035	3.364392	3.332684	35.18014
3.316832	3.388249	3.350469	35.17235
3.325658	3.386766	3.333639	35.15161
3.250183	3.414667	3.446725	35.29487
3.1957	3.460158	3.418675	35.21386
3.312006	3.446949	3.46746	35.54601
3.311223	3.250361	3.364519	34.88927
3.31576	3.122044	3.371016	34.63279
3.156189	3.223741	3.458593	34.69775
3.225107	3.404491	3.410695	35.13899
3.409258	3.45598	3.363462	35.551
3.300087	3.297402	3.386635	35.01616
3.278723	3.23257	3.327688	34.69875
3.124679	3.289585	3.447799	34.74923
3.249729	3.405974	3.453597	35.28989
3.293942	3.51906	3.404983	35.52757
3.294395	3.434481	3.420653	35.37787
3.339722	3.26721	3.43854	35.15031
3.222673	3.213632	3.392774	34.6771
3.259339	3.230278	3.425359	34.86494
3.313945	3.378746	3.375211	35.19936
3.323183	3.452273	3.359216	35.34538
3.2753	3.375107	3.374989	35.10641
3.413548	3.35718	3.382287	35.38549
3.293571	3.378342	3.351304	35.10164
3.326854	3.407052	3.361382	35.25925

3.205846	3.469593	3.456734	35.33991
3.191246	3.427674	3.451636	35.20517
3.199123	3.223539	3.461304	34.79712
3.307841	3.17852	3.380411	34.75952
3.291508	3.249418	3.458047	35.04863
3.367314	3.460765	3.404761	35.56006
3.255091	3.36055	3.466641	35.23081
3.325452	3.303535	3.310909	34.91943
3.361457	3.225965	3.397327	35.01752
3.318729	3.352396	3.384862	35.17331
3.297942	3.389327	3.389824	35.21946
3.268371	3.410422	3.374426	35.16725
3.216817	3.395056	3.453051	35.19285
3.320874	3.361696	3.367827	35.16108
3.30417	3.182968	3.409041	34.82383
3.208857	3.257505	3.429605	34.82337
3.261195	3.307579	3.472967	35.14215
3.237273	3.4053	3.387454	35.11654
3.385131	3.400649	3.384845	35.424
3.287755	3.35098	3.328097	34.97834
3.334567	3.363987	3.407984	35.28385
3.30318	3.413589	3.348303	35.19318
3.355766	3.485094	3.320526	35.4038
3.274599	3.442973	3.455387	35.4291
3.261524	3.375512	3.376882	35.08131
3.155653	3.132018	3.416152	34.40318
3.338237	3.214845	3.315905	34.76437
3.331803	3.333323	3.379593	35.14867
3.298314	3.422957	3.363377	35.23598
3.26037	3.340669	3.472285	35.21122
3.339763	3.306433	3.391734	35.13382
3.27134	3.238433	3.314456	34.66649
3.43219	3.311218	3.369243	35.29722
3.175491	3.403076	3.38343	34.96776
3.191039	3.459282	3.453085	35.27701
3.149837	3.384542	3.456513	35.03096
3.271877	3.241735	3.36781	34.79156
3.322523	3.183844	3.402374	34.8513
3.270722	3.238096	3.452557	34.9664
3.206258	3.359337	3.425411	35.0312
3.351311	3.372883	3.341994	35.19562
3.437593	3.444994	3.375586	35.61546
3.311347	3.391821	3.35995	35.1889
3.277651	3.336895	3.406739	35.09742
3.27101	3.41278	3.427593	35.29445
3.309119	3.471817	3.350691	35.33872
3.316584	3.444186	3.407626	35.41913
3.262638	3.290259	3.447526	35.0518
3.109213	3.168411	3.403278	34.35305
3.285941	3.261616	3.378519	34.88921

3.366613	3.428079	3.402732	35.48261
3.384347	3.405165	3.362678	35.38369
3.219497	3.340804	3.478765	35.1363
3.299468	3.309331	3.417328	35.10801
3.264288	3.226504	3.389636	34.78939
3.296705	3.364526	3.37192	35.12337
3.234922	3.408871	3.438728	35.23133
3.209888	3.423226	3.399731	35.1227
3.117214	3.360887	3.384384	34.75015
3.291921	3.224482	3.381503	34.82761
3.22952	3.243622	3.408922	34.79296
3.235871	3.312027	3.42183	34.98467
3.212362	3.344376	3.451363	35.06859
3.319224	3.446747	3.388681	35.38907
3.516409	3.40321	3.360154	35.66269
3.387606	3.337165	3.312137	35.13158
3.259957	3.40166	3.451755	35.2988
3.250389	3.464336	3.350128	35.1927
3.261689	3.39546	3.409825	35.19734
3.257565	3.310544	3.311063	34.78664
3.288786	3.211543	3.428019	34.89418
3.127236	3.211677	3.437142	34.56114
3.270227	3.383262	3.402255	35.17277
3.355889	3.450993	3.421489	35.55028
3.325081	3.3683	3.410644	35.27836
3.236943	3.334604	3.401504	34.99194
3.305944	3.245644	3.408751	34.96414
3.275836	3.342826	3.365764	35.01681
3.350322	3.45726	3.365696	35.4298
3.221147	3.386901	3.389278	35.04503
3.278063	3.396741	3.423057	35.26488
3.143321	3.291	3.369635	34.62215
3.293447	3.225628	3.341892	34.74683
3.260493	3.286687	3.359353	34.84647
3.255132	3.384071	3.437995	35.21969
3.20432	3.445803	3.446725	35.26266
3.426086	3.397212	3.357869	35.44706
3.360096	3.310207	3.346359	35.08731
3.385378	3.372748	3.382935	35.35935
3.265278	3.454834	3.387164	35.28547
3.191452	3.405502	3.35035	34.93563
3.163901	3.332245	3.372772	34.76422
3.248945	3.267479	3.358534	34.77743
3.15025	3.217136	3.459428	34.67214
3.266721	3.330021	3.418351	35.08388
3.187946	3.407456	3.503473	35.2671
3.387853	3.402132	3.432675	35.53779
3.414744	3.361831	3.471467	35.5933
3.278104	3.309938	3.33618	34.88516
3.331762	3.215384	3.404096	34.94425

3.285734	3.370592	3.364025	35.09538
3.25043	3.461439	3.40014	35.29582
3.227746	3.399975	3.35185	35.0062
3.308666	3.273814	3.378775	34.96614
3.131113	3.184922	3.372568	34.3699
3.272	3.239915	3.394803	34.84688
3.20069	3.378679	3.445293	35.1048
3.275011	3.467908	3.396048	35.35477
3.301654	3.435559	3.459735	35.48157
3.350239	3.369244	3.439887	35.39939
3.332546	3.327662	3.418078	35.22207
3.427777	3.418711	3.332019	35.44124
3.285982	3.414937	3.435931	35.35014
3.356632	3.384003	3.330314	35.20603
3.153343	3.220708	3.445548	34.65637
3.214095	3.214036	3.306885	34.47139
3.162375	3.255686	3.426451	34.71084
3.278146	3.433672	3.37579	35.24246
3.253565	3.460225	3.506201	35.53196
3.337495	3.372681	3.415128	35.32489
3.288663	3.312027	3.397599	35.04713
3.495911	3.374703	3.39216	35.62552
3.291921	3.356439	3.384862	35.12352
3.199783	3.308994	3.42229	34.90013
3.235459	3.320518	3.327364	34.79576
3.328792	3.257168	3.361535	34.93605
3.327514	3.228998	3.331149	34.80521
3.230798	3.232435	3.413355	34.78099
3.142826	3.34195	3.415947	34.83377
3.284621	3.437649	3.464595	35.45951
3.379934	3.46063	3.337373	35.43999
3.367768	3.399369	3.386345	35.38651
3.401216	3.347341	3.383498	35.33965
3.301324	3.378005	3.461935	35.3598
3.297076	3.478624	3.363888	35.35613
3.323307	3.454901	3.39598	35.43179
3.362158	3.233041	3.335822	34.90003
3.136021	3.114361	3.426792	34.3449
3.281857	3.22246	3.426519	34.89962
3.198381	3.423833	3.393729	35.08574
3.292375	3.510029	3.446537	35.59527
3.192648	3.406513	3.409706	35.07026
3.261442	3.307714	3.495833	35.19299
3.224529	3.320316	3.377103	34.88019
3.446089	3.339321	3.451499	35.56896
3.28128	3.388788	3.393967	35.19091
3.313945	3.422081	3.406245	35.36199
3.180564	3.261077	3.360103	34.61732
3.372593	3.211004	3.323493	34.84769
3.238511	3.22455	3.365577	34.67613

3.193638	3.291404	3.339726	34.66767
3.205475	3.40193	3.459752	35.19773
3.333865	3.494664	3.396713	35.54344
3.363107	3.476939	3.452659	35.69097
3.499829	3.368772	3.271316	35.35685
3.291508	3.361022	3.472217	35.32368
3.281816	3.458001	3.368714	35.28821
3.285404	3.451599	3.441729	35.44173
3.263793	3.266671	3.333707	34.75384
3.222055	3.121033	3.389142	34.4653
3.153797	3.208914	3.366753	34.45925
3.176852	3.353878	3.460298	35.03125
3.242264	3.519262	3.47544	35.56908
3.294602	3.406243	3.384998	35.2386
3.23253	3.311016	3.462481	35.06405
3.299757	3.301985	3.403346	35.062
3.247172	3.326449	3.43663	35.07329
3.351559	3.376522	3.368492	35.26206
3.327761	3.391956	3.403141	35.31954
3.326483	3.387845	3.352617	35.19727
3.203124	3.164165	3.364178	34.46363
3.381378	3.225763	3.295017	34.83691
3.197927	3.247598	3.418829	34.75424
3.251049	3.393843	3.314183	34.96137
3.146744	3.432527	3.510396	35.24696
3.329246	3.48105	3.342045	35.38402
3.261153	3.367492	3.389619	35.09081
3.316956	3.387103	3.407847	35.29559
3.287879	3.43219	3.45919	35.44288
3.459245	3.422013	3.32525	35.50247
3.249481	3.283991	3.364536	34.82783
3.265154	3.102702	3.352259	34.43881
3.202959	3.14981	3.264188	34.21322
3.211826	3.341613	3.390506	34.92829
3.201144	3.458541	3.382355	35.14281
3.223292	3.452947	3.44444	35.31478
3.245275	3.365942	3.411377	35.10028
3.344135	3.337704	3.399253	35.22821
3.371727	3.370659	3.444116	35.45872
3.295509	3.372411	3.408103	35.21712
3.219951	3.364459	3.41078	35.04035
3.292086	3.332717	3.356096	35.0091
3.424395	3.238905	3.340869	35.05999
3.295592	3.198671	3.361433	34.7353
3.236201	3.207701	3.397889	34.7049
3.198628	3.278128	3.383481	34.74523
3.224075	3.399099	3.412127	35.12807
3.327803	3.474176	3.396065	35.48396
3.329329	3.441018	3.414054	35.45413
3.25175	3.354417	3.364178	34.98602

3.381873	3.404963	3.405119	35.47065
3.307057	3.461641	3.325915	35.25777
3.393586	3.403413	3.407558	35.4982
3.261978	3.277049	3.409808	34.93898
3.21793	3.15136	3.342369	34.42032
3.262308	3.203995	3.331695	34.60913
3.355931	3.363313	3.352958	35.20877
3.209929	3.414398	3.45375	35.22161
3.138867	3.409545	3.396099	34.92953
3.154622	3.333997	3.456547	34.93096
3.302355	3.367694	3.394752	35.19258
3.322771	3.343634	3.449726	35.30483
3.366695	3.357922	3.401828	35.32739
3.154745	3.349835	3.484767	35.02758
3.362818	3.352867	3.312819	35.11321
3.38278	3.191122	3.362968	34.91282
3.289405	3.226976	3.33664	34.72945
3.138537	3.300165	3.427678	34.75867
3.17182	3.400784	3.348644	34.87866
3.34727	3.478826	3.37383	35.48808
3.374738	3.405367	3.30472	35.23637
3.365953	3.389664	3.39112	35.37176
3.130205	3.393169	3.448413	34.98918
3.259669	3.424507	3.365952	35.16049
3.41231	3.420126	3.449283	35.66695
3.414826	3.309129	3.355601	35.22485
3.184399	3.146912	3.373113	34.4045
3.261483	3.218215	3.406279	34.80152
3.23649	3.40995	3.426246	35.20982
3.35923	3.473435	3.38568	35.52836
3.214053	3.359404	3.375705	34.9397
3.186544	3.338782	3.448021	34.99259
3.109048	3.331099	3.445156	34.80005
3.370036	3.387845	3.348576	35.28368
3.274516	3.373692	3.434431	35.23159
3.31811	3.334806	3.359933	35.07897
3.253647	3.29228	3.444662	35.03029
3.339763	3.187955	3.3629	34.81167
3.388678	3.218147	3.375518	35.01226
3.400845	3.307242	3.314456	35.10017
3.122452	3.348824	3.463009	34.90716
3.214218	3.474109	3.383378	35.20768
3.364221	3.469324	3.34271	35.43631
3.40897	3.399167	3.337629	35.36964
3.313945	3.397145	3.382065	35.25459
3.173429	3.386699	3.462992	35.10143
3.252492	3.413387	3.401965	35.19924
3.390988	3.294707	3.348235	35.12507
3.384347	3.189775	3.365986	34.9199
3.20168	3.205342	3.398145	34.62481

3.211208	3.359809	3.416834	35.0243
3.203165	3.432729	3.431225	35.19765
3.348672	3.415072	3.354766	35.31003
3.273155	3.352193	3.428309	35.16821
3.229808	3.364661	3.476105	35.20521
3.205145	3.437986	3.471467	35.30148
3.299221	3.411095	3.414242	35.32326
3.280002	3.310814	3.40355	35.03855
3.249481	3.283924	3.4375	34.98725
3.1957	3.246924	3.415691	34.74103
3.390328	3.192942	3.38389	34.97906
3.264989	3.296459	3.337459	34.8298
3.320833	3.324562	3.386567	35.12077
3.224612	3.382049	3.422665	35.115
3.243707	3.434683	3.438063	35.30554
3.256245	3.434751	3.371681	35.18794
3.348012	3.416824	3.372858	35.35199
3.252657	3.406311	3.442275	35.27227
3.254596	3.379151	3.384367	35.09048
3.255709	3.343095	3.401692	35.05196
3.330112	3.224482	3.342352	34.82551
3.384801	3.177172	3.366514	34.89449
3.276826	3.250496	3.341619	34.76427
3.251461	3.427	3.431362	35.29104
3.291715	3.425383	3.388681	35.28219
3.265649	3.340602	3.443792	35.16031
3.288415	3.415409	3.318071	35.09875
3.192937	3.432122	3.5394	35.41052
3.208032	3.404019	3.421216	35.12362
3.271629	3.334064	3.505979	35.29508
3.221725	3.274084	3.394803	34.81166
3.318028	3.280082	3.418061	35.08624
3.278269	3.24679	3.38065	34.84467
3.321162	3.198266	3.388528	34.84959
3.245275	3.310409	3.359762	34.86597
3.349992	3.342286	3.36486	35.17583
3.285899	3.413993	3.447799	35.37385
3.323018	3.46063	3.389415	35.42933
3.187204	3.37841	3.412622	35.00328
3.306521	3.407658	3.327432	35.14187
3.247131	3.385688	3.437295	35.2042
3.312254	3.404356	3.37886	35.25965
3.229685	3.234996	3.46023	34.88666
3.402989	3.241668	3.343938	35.02593
3.263958	3.222595	3.384248	34.76834
3.383358	3.429427	3.378075	35.46825
3.272701	3.413117	3.438472	35.32268
3.226633	3.339523	3.358261	34.88559
3.115729	3.328269	3.41477	34.74202
3.230179	3.354754	3.384794	34.98467

3.223374	3.477074	3.457229	35.39569
3.231293	3.435829	3.417652	35.23626
3.146002	3.270445	3.464663	34.79088
3.268825	3.216395	3.457843	34.92636
3.447202	3.23695	3.373795	35.1776
3.278682	3.269703	3.404983	34.9489
3.330277	3.3559	3.430134	35.30523
3.216775	3.36237	3.433561	35.07866
3.305531	3.389732	3.339027	35.12586
3.364922	3.389462	3.376473	35.33703
3.358034	3.439536	3.349309	35.37207
3.168933	3.370322	3.414446	34.94963
3.260081	3.385284	3.374136	35.09352
3.254678	3.346937	3.426877	35.11318
3.272371	3.289045	3.408734	34.9856
3.206629	3.230143	3.370249	34.62886
3.284951	3.25892	3.43692	35.00887
3.270804	3.386227	3.355925	35.07921
3.420105	3.430572	3.3622	35.5164
3.324379	3.384677	3.393439	35.27501
3.253152	3.35563	3.397258	35.06408
3.178501	3.377331	3.402766	34.96033
3.234139	3.430505	3.450221	35.30206
3.219456	3.394045	3.369703	35.01414
3.268371	3.302592	3.371323	34.92466
3.155859	3.225089	3.479805	34.74636
3.283012	3.240926	3.401146	34.88704
3.318317	3.238298	3.367793	34.88556
3.370984	3.34788	3.411803	35.33662
3.304583	3.377938	3.435079	35.30804
3.238016	3.341006	3.344842	34.88437
3.288704	3.377533	3.397003	35.18917
3.375026	3.440075	3.30356	35.31037
3.277197	3.464471	3.442019	35.45257
3.268701	3.397347	3.31582	35.01122
3.176563	3.276982	3.47694	34.89886
3.263999	3.2972	3.376302	34.9142
3.317244	3.237691	3.444321	35.04925
3.283301	3.26256	3.348013	34.81879
3.317863	3.31324	3.453546	35.23598
3.304954	3.425316	3.412008	35.36201
3.311718	3.405097	3.399441	35.30511
3.291096	3.388249	3.372312	35.16384
3.25938	3.376253	3.388477	35.1036
3.122204	3.375377	3.450869	34.93814
3.205887	3.440547	3.404096	35.16137
3.176604	3.345858	3.501358	35.10296
3.255173	3.234861	3.335736	34.66986
3.186296	3.171039	3.438455	34.6043
3.293034	3.257977	3.403789	34.95203

3.165221	3.345184	3.47619	35.02156
3.473474	3.444455	3.354152	35.64587
3.309903	3.338782	3.393098	35.14224
3.408805	3.37787	3.373727	35.40165
3.326112	3.420194	3.408257	35.38887
3.337619	3.459956	3.368066	35.4131
3.159901	3.424844	3.356795	34.92303
3.346775	3.321125	3.333775	35.05454
3.17182	3.254675	3.444866	34.76956
3.244903	3.241735	3.374136	34.74641
3.183904	3.307579	3.406449	34.82767
3.343558	3.358259	3.410968	35.29751
3.347929	3.432459	3.394803	35.43399
3.313161	3.365807	3.407097	35.23908
3.254224	3.37787	3.406688	35.13568
3.236902	3.388316	3.414225	35.13713
3.230963	3.430168	3.363854	35.10551
3.245605	3.407187	3.369976	35.10066
3.13367	3.366481	3.427815	34.89334
3.129051	3.237826	3.392552	34.52478
3.243047	3.212554	3.328575	34.5789
3.230303	3.234052	3.388068	34.72815
3.31081	3.318294	3.46705	35.26115
3.234345	3.396336	3.468346	35.26743
3.306521	3.421002	3.433135	35.40221
3.399236	3.363852	3.274487	35.13304
3.404309	3.391686	3.37504	35.4249
3.223416	3.417363	3.352873	35.03699
3.306686	3.428416	3.427269	35.40595
3.230386	3.416015	3.381264	35.11137
3.317863	3.300705	3.339931	34.96012
3.206794	3.239848	3.38626	34.68546
3.199618	3.299155	3.428719	34.89231
3.170706	3.302726	3.42582	34.83055
3.366613	3.382925	3.363036	35.29705
3.30648	3.35462	3.405921	35.19743
3.410124	3.35563	3.375245	35.35922
3.220776	3.327662	3.454142	35.05652
3.271423	3.427674	3.361587	35.18358
3.280538	3.395325	3.324039	35.05066
3.26668	3.444321	3.366208	35.21971
3.148971	3.307511	3.439751	34.82395
3.20201	3.214575	3.390165	34.62827
3.221642	3.220708	3.422103	34.75445
3.34496	3.281228	3.3644	35.03029
3.324049	3.290056	3.452778	35.19714
3.27563	3.329077	3.387863	35.03462
3.288333	3.302861	3.389074	35.00772
3.308171	3.345117	3.358858	35.07743
3.411403	3.479837	3.393712	35.67402

3.24812	3.416622	3.39216	35.17531
3.247419	3.360011	3.357852	34.97495
3.27398	3.318227	3.38198	34.99442
3.319018	3.268625	3.321362	34.85188
3.26136	3.307377	3.410439	35.00533
3.329741	3.378207	3.385936	35.25618
3.177223	3.343163	3.425598	34.93275
3.376387	3.389934	3.354834	35.31582
3.356714	3.348621	3.401231	35.28392
3.350899	3.387305	3.386482	35.32354
3.239047	3.37841	3.455029	35.20939
3.272949	3.37322	3.389773	35.12947
3.227581	3.373085	3.425121	35.10726
3.336587	3.378073	3.335123	35.15974
3.169964	3.252451	3.424149	34.71533
3.104016	3.266401	3.421676	34.59621
3.149755	3.301783	3.469745	34.87873
3.296911	3.349767	3.406057	35.1662
3.333989	3.325708	3.411855	35.20734
3.354322	3.389597	3.404113	35.37459
3.226591	3.368435	3.442428	35.13278
3.371397	3.434414	3.373318	35.4426
3.407237	3.413319	3.359609	35.44487
3.311841	3.366683	3.377155	35.17263
3.247708	3.279071	3.351151	34.78393
3.235087	3.183237	3.404011	34.66235
3.333865	3.336288	3.327603	35.04597
3.293942	3.369446	3.404505	35.19934
3.314027	3.405097	3.40621	35.32496
3.182131	3.413993	3.418624	35.08312
3.293117	3.382251	3.39835	35.21208
3.418249	3.366009	3.424336	35.50704
3.445058	3.447623	3.401641	35.69451
3.242223	3.421878	3.445037	35.28954
3.304253	3.359944	3.402988	35.19779
3.202711	3.262829	3.421216	34.80323
3.250512	3.282306	3.360239	34.81701
3.149879	3.237018	3.380019	34.54115
3.117049	3.226908	3.413764	34.52105
3.091395	3.291741	3.436903	34.65733
3.291797	3.33831	3.444457	35.21393
3.36785	3.464336	3.457075	35.68344
3.438665	3.410556	3.414191	35.62691
3.212074	3.378005	3.40575	35.04175
3.321492	3.412174	3.404079	35.3521
3.397009	3.442434	3.348474	35.46182
3.343599	3.381038	3.409808	35.34488
3.161262	3.271927	3.33739	34.54917
3.198628	3.22273	3.403209	34.66723
3.273526	3.326853	3.36706	34.97967

3.277073	3.355765	3.374546	35.06702
3.329659	3.412174	3.442206	35.45334
3.189926	3.334267	3.465652	35.02866
3.27963	3.343634	3.359489	35.01316
3.348383	3.379016	3.365884	35.25487
3.409258	3.467976	3.412485	35.68444
3.260782	3.429427	3.445617	35.3479
3.242511	3.315127	3.444133	35.05475
3.239047	3.282037	3.38609	34.84787
3.332298	3.279813	3.367111	35.00543
3.11445	3.255012	3.422631	34.59621
3.229726	3.260807	3.443059	34.90565
3.11973	3.283587	3.419698	34.66383
3.304665	3.377399	3.372653	35.17053
3.362241	3.383936	3.373062	35.31163
3.349579	3.351856	3.41888	35.31398
3.236613	3.377399	3.458081	35.20853
3.333123	3.480241	3.346479	35.40042
3.364633	3.372344	3.350793	35.24281
3.441634	3.357517	3.320816	35.31322
3.131608	3.255012	3.392075	34.56691
3.110697	3.256292	3.382799	34.5037
3.163324	3.349228	3.400481	34.86069
3.313079	3.451801	3.394274	35.39892
3.273939	3.345926	3.445224	35.19321
3.217724	3.318766	3.449658	35.02058
3.177553	3.362033	3.457774	35.0451
3.445553	3.485768	3.360154	35.68828
3.396267	3.444455	3.430986	35.64506
3.253358	3.332245	3.363445	34.93945
3.240367	3.257236	3.445821	34.92715
3.213022	3.251103	3.399816	34.75333
3.350981	3.256292	3.324738	34.90219
3.246842	3.27213	3.411445	34.8987
3.209682	3.312633	3.430338	34.94733
3.143816	3.321462	3.440979	34.84587
3.321864	3.396471	3.405256	35.32114
3.43252	3.401526	3.384743	35.52933
3.323018	3.36884	3.42686	35.31049
3.180605	3.342354	3.459207	35.01187
3.392101	3.450925	3.468756	35.73269
3.280579	3.333727	3.332769	34.93514
3.386616	3.20076	3.301923	34.80879
3.175656	3.199479	3.393592	34.54512
3.139361	3.363516	3.377939	34.79024
3.216239	3.402873	3.370198	35.0275
3.345826	3.400447	3.337066	35.23312
3.285239	3.352935	3.50216	35.35776
3.310522	3.351519	3.353146	35.08408
3.225849	3.40968	3.456393	35.25189

3.389255	3.462517	3.38609	35.57104
3.439655	3.377736	3.442002	35.61812
3.315058	3.282576	3.371545	34.98347
3.220157	3.254809	3.402732	34.78342
3.238016	3.26849	3.411735	34.87208
3.282022	3.273612	3.382662	34.91594
3.261689	3.252181	3.393729	34.84881
3.191782	3.324764	3.465703	35.01205
3.172686	3.389934	3.415214	35.0024
3.354405	3.438659	3.385408	35.44116
3.424147	3.361157	3.404096	35.46506
3.310192	3.35098	3.428514	35.247
3.204732	3.426529	3.371698	35.05734
3.302108	3.431718	3.450118	35.45313
3.291715	3.365403	3.389636	35.15311
3.383852	3.261616	3.310636	34.95488
3.176811	3.208106	3.418266	34.62046
3.225519	3.385418	3.382321	35.03613
3.146249	3.373692	3.420244	34.92007
3.302108	3.420261	3.35156	35.21254
3.267876	3.338917	3.414446	35.09732
3.277857	3.345589	3.432248	35.17267
3.306892	3.487789	3.395894	35.46763
3.433551	3.489205	3.445958	35.85719
3.301242	3.326314	3.383089	35.07415
3.426209	3.278667	3.427133	35.33955
3.215497	3.278195	3.416799	34.85513
3.202464	3.317283	3.384504	34.84148
3.143651	3.290326	3.409672	34.70896
3.222426	3.257573	3.410661	34.81176
3.239583	3.339119	3.467562	35.15204
3.243419	3.436031	3.431788	35.29413
3.213806	3.377331	3.467988	35.18017
3.401216	3.36884	3.40396	35.43142
3.339433	3.417228	3.354527	35.29402
3.37548	3.389529	3.399202	35.40998
3.339145	3.293426	3.350963	35.01486
3.261442	3.244229	3.355243	34.74671
3.304583	3.25892	3.304464	34.76214
3.293529	3.380903	3.422631	35.26313
3.270722	3.506458	3.425359	35.49379
3.234056	3.372142	3.396917	35.05769
3.329494	3.295717	3.403977	35.1147
3.220364	3.305018	3.436886	34.96836
3.289199	3.457125	3.37886	35.32463
3.232118	3.44176	3.487632	35.40407
3.316337	3.388586	3.412417	35.30748
3.268165	3.234591	3.386192	34.80802
3.345001	3.253394	3.351168	34.94058
3.309532	3.328336	3.396645	35.12635

3.237892	3.312296	3.373335	34.88363
3.153261	3.274488	3.410405	34.69694
3.279053	3.30859	3.372193	34.96304
3.25307	3.412106	3.375876	35.14065
3.301778	3.420935	3.459139	35.44855
3.259875	3.383734	3.421608	35.19349
3.297118	3.353204	3.428565	35.22339
3.375439	3.359472	3.459326	35.47564
3.458421	3.383262	3.384214	35.54487
3.360839	3.237085	3.347536	34.9316
3.189967	3.183642	3.351714	34.4502
3.202258	3.309803	3.374426	34.80264
3.354693	3.461978	3.328319	35.36794
3.326483	3.479365	3.357153	35.40733
3.170253	3.358461	3.410968	34.91897
3.199329	3.324832	3.427695	34.94559
3.311511	3.469256	3.415691	35.4805
3.328875	3.489744	3.415964	35.56387
3.323142	3.420396	3.373983	35.30787
3.29753	3.203118	3.498749	35.04956
3.192772	3.176835	3.333366	34.40132
3.374861	3.275769	3.346189	35.04392
3.359271	3.325371	3.376319	35.18418
3.165551	3.2941	3.407796	34.761
3.197886	3.24497	3.393507	34.69302
3.325988	3.359	3.375023	35.18211
3.296004	3.486172	3.37666	35.39822
3.338897	3.423092	3.386362	35.37529
3.11676	3.387508	3.517659	35.09882
3.321864	3.350374	3.383958	35.17376
3.297447	3.408871	3.440245	35.37139
3.471536	3.360011	3.367947	35.48713
3.300417	3.239848	3.404147	34.92931
3.286972	3.18162	3.3452	34.64366
3.133835	3.369311	3.40292	34.84545
3.395401	3.466493	3.374682	35.56823
3.22886	3.438727	3.364656	35.12138
3.22523	3.353946	3.339829	34.87375
3.112595	3.336895	3.496703	34.9332
3.298272	3.518049	3.415555	35.55795
3.371809	3.476198	3.431413	35.66192
3.282105	3.35563	3.404215	35.14261
3.292663	3.240657	3.440979	34.99467
3.284167	3.257033	3.361996	34.83918
3.307016	3.252248	3.421148	35.00804
3.351559	3.249822	3.383225	35.01721
3.337825	3.288641	3.372346	35.04827
3.112677	3.282845	3.45005	34.71316
3.314399	3.4177	3.35006	35.23054
3.269361	3.37659	3.427764	35.21207

3.316749	3.415274	3.395093	35.32885
3.159694	3.314925	3.426741	34.83516
3.32607	3.381375	3.442905	35.37967
3.26305	3.385688	3.359898	35.06976
3.508325	3.277993	3.339692	35.32644
3.263752	3.214306	3.398009	34.77985
3.306604	3.260336	3.369226	34.91128
3.236531	3.402739	3.376541	35.08545
3.394205	3.448432	3.287037	35.33445
3.24911	3.351856	3.368748	34.98464
3.280538	3.290393	3.370794	34.92344
3.189101	3.442097	3.476395	35.28616
3.290766	3.514747	3.412878	35.52846
3.276743	3.407524	3.497675	35.44875
3.367644	3.28608	3.407455	35.18466
3.295303	3.195907	3.374887	34.75805
3.330483	3.255281	3.421199	35.0661
3.314811	3.39573	3.375313	35.23862
3.277197	3.307848	3.4028	35.02429
3.226674	3.270512	3.313518	34.63691
3.190668	3.266873	3.411479	34.76444
3.193473	3.336558	3.42142	34.9447
3.27167	3.412174	3.417447	35.27238
3.298973	3.470132	3.379405	35.37564
3.248162	3.393169	3.464254	35.28177
3.366695	3.329145	3.420585	35.30547
3.297035	3.247127	3.387488	34.9014
3.37746	3.254607	3.352993	35.0182
3.340382	3.337771	3.400208	35.22224
3.262019	3.376994	3.365133	35.05994
3.302974	3.444051	3.347553	35.2577
3.322606	3.348352	3.403806	35.21437
3.230427	3.304209	3.365628	34.83277
3.244491	3.389327	3.387727	35.09799
3.170954	3.499786	3.477333	35.37468
3.264494	3.442434	3.457297	35.41001
3.225849	3.232974	3.392024	34.7247
3.261813	3.183103	3.389074	34.68783
3.292704	3.278734	3.405443	35.00032
3.424106	3.384273	3.439683	35.59334
3.241851	3.377466	3.422358	35.14201
3.316584	3.325168	3.316536	34.95966
3.284291	3.293022	3.401828	35.00526
3.321451	3.3435	3.353402	35.09101
3.191039	3.354687	3.414225	34.96329
3.225725	3.441018	3.402306	35.20187
3.227169	3.388451	3.467102	35.23177
3.368386	3.326921	3.330706	35.10776
3.391977	3.261414	3.446111	35.26847
3.252863	3.240859	3.339334	34.68579

3.304129	3.329751	3.464373	35.26574
3.310769	3.446005	3.333554	35.24841
3.362241	3.442501	3.335873	35.35837
3.253111	3.349498	3.377086	35.00647
3.239006	3.280149	3.35613	34.77814
3.236448	3.390203	3.435232	35.1862
3.284043	3.502347	3.418675	35.49932
3.257771	3.455912	3.456956	35.42403
3.234428	3.319507	3.432743	35.02174
3.080012	3.153719	3.476139	34.4164
3.356178	3.234322	3.418471	35.07049
3.37515	3.409208	3.42495	35.5086
3.371974	3.507468	3.444065	35.75833
3.246512	3.285608	3.431379	34.97105
3.326235	3.257236	3.406603	35.02917
3.236943	3.320923	3.375023	34.90411
3.382203	3.416217	3.301719	35.26986
3.171531	3.463528	3.479311	35.30098
3.127071	3.400582	3.397327	34.88681
3.238552	3.311151	3.44502	35.03933
3.338567	3.265255	3.305998	34.85367
3.385667	3.233041	3.412946	35.1201
3.310934	3.297133	3.428582	35.13102
3.228406	3.341073	3.461577	35.11879
3.388472	3.375781	3.315564	35.22542
3.357044	3.36702	3.315155	35.13664
3.401587	3.366346	3.328302	35.26132
3.156931	3.390473	3.397344	34.93004
3.210176	3.430033	3.359148	35.04947
3.296788	3.483409	3.414583	35.47682
3.289611	3.345319	3.384163	35.09263
3.2826	3.202242	3.393251	34.78428
3.142207	3.179868	3.418692	34.48397
3.283219	3.315801	3.419221	35.09076
3.362983	3.384542	3.395093	35.36276
3.290436	3.307781	3.378996	35.00104
3.207001	3.244768	3.438199	34.81025
3.355023	3.370727	3.446896	35.42842
3.232984	3.453688	3.433305	35.31324
3.39404	3.445938	3.356693	35.48096
3.202093	3.333121	3.418675	34.95003
3.144269	3.348285	3.436341	34.89538
3.241563	3.341276	3.439751	35.10027
3.365252	3.38151	3.429043	35.43533
3.362901	3.290056	3.332428	35.01891
3.31411	3.25427	3.397412	34.97606
3.217435	3.312633	3.430014	34.96358
3.388183	3.335817	3.33872	35.18803
3.395153	3.410354	3.343836	35.37746
3.413177	3.40011	3.387522	35.49001

3.253111	3.369716	3.420738	35.14614
3.250966	3.436705	3.33473	35.09986
3.283755	3.485094	3.435181	35.49705
3.317904	3.394988	3.368527	35.22892
3.223209	3.225561	3.441337	34.81055
3.125462	3.240252	3.41813	34.57817
3.267505	3.275365	3.390063	34.90421
3.383193	3.415813	3.433612	35.55957
3.310439	3.365672	3.352055	35.11247
3.198587	3.267412	3.431839	34.82746
3.258472	3.316205	3.405392	35.00729
3.246594	3.368368	3.413287	35.11265
3.361911	3.386025	3.417516	35.41269
3.226962	3.452947	3.409416	35.24621
3.166293	3.411702	3.497522	35.21602
3.183904	3.292483	3.393661	34.76669
3.442583	3.280217	3.356062	35.22333
3.27365	3.249283	3.394735	34.87083
3.311965	3.369648	3.383617	35.19352
3.309779	3.371198	3.446367	35.32935
3.418538	3.416285	3.321311	35.39231
3.33535	3.353743	3.370198	35.18053
3.346156	3.372411	3.322163	35.13994
3.178254	3.361965	3.414395	34.95162
3.266639	3.462045	3.378382	35.28501
3.158498	3.402739	3.42067	35.01131
3.327225	3.280958	3.396662	35.06147
3.264412	3.172117	3.401214	34.69604
3.147115	3.292483	3.461372	34.83431
3.283342	3.456519	3.4412	35.44682
3.394988	3.41797	3.447543	35.62055
3.326772	3.32591	3.400259	35.16665
3.266597	3.224819	3.34624	34.69585
3.191658	3.314453	3.464169	34.98588
3.219951	3.4084	3.374904	35.05799
3.336381	3.472222	3.398759	35.50434
3.263009	3.421878	3.42466	35.29044
3.251049	3.233041	3.429247	34.86135
3.206629	3.17178	3.365901	34.49172
3.321616	3.22583	3.40546	34.94789
3.301201	3.386564	3.397207	35.23669
3.303675	3.467167	3.411036	35.44861
3.291096	3.426933	3.334458	35.16565
3.429385	3.35098	3.384333	35.41104
3.292704	3.367087	3.344944	35.06123
3.395442	3.372479	3.35804	35.32633
3.178584	3.455912	3.424217	35.17927
3.199907	3.419857	3.392211	35.07706
3.17479	3.249216	3.395349	34.65583
3.277981	3.212486	3.380207	34.76806

3.216858	3.243892	3.44855	34.85252
3.277403	3.436638	3.422563	35.3496
3.216198	3.408332	3.489951	35.30122
3.396019	3.378005	3.368203	35.3619
3.444439	3.269097	3.407387	35.31531
3.410083	3.324292	3.395434	35.33474
3.26602	3.367357	3.425649	35.17995
3.257235	3.445871	3.3489	35.1646
3.262638	3.434346	3.393661	35.2491
3.339103	3.371198	3.280558	35.03088
3.249069	3.18344	3.452386	34.79916
3.191617	3.240252	3.361092	34.59811
3.250224	3.316475	3.470188	35.13154
3.33036	3.413859	3.369754	35.30011
3.401546	3.444186	3.412673	35.61596
3.336258	3.37996	3.389875	35.28288
3.355436	3.315059	3.39985	35.20471
3.291261	3.384273	3.402868	35.22233
3.344341	3.455036	3.331252	35.33654
3.265855	3.404154	3.453801	35.32163
3.275382	3.309466	3.406807	35.03262
3.094942	3.155606	3.373489	34.2287
3.302149	3.21343	3.330348	34.71394
3.235789	3.366481	3.426877	35.11461
3.255709	3.448769	3.44311	35.37362
3.227334	3.357585	3.446503	35.11959
3.366035	3.290461	3.410405	35.19717
3.358117	3.244229	3.307243	34.85316
3.439119	3.355967	3.375091	35.42302
3.219951	3.413252	3.464714	35.265
3.196855	3.441153	3.322794	34.96515
3.154745	3.357922	3.458525	34.98787
3.288126	3.274084	3.346155	34.85048
3.224117	3.178183	3.382458	34.58017
3.193514	3.271658	3.434448	34.83136
3.13833	3.386092	3.505161	35.11557
3.419281	3.423361	3.400942	35.58355
3.414744	3.401323	3.323459	35.35599
3.359313	3.336895	3.401948	35.26552
3.341166	3.39364	3.395894	35.33669
3.271052	3.465145	3.437841	35.43147
3.341372	3.41433	3.3378	35.25535
3.363519	3.406984	3.345029	35.30353
3.267175	3.251305	3.421131	34.91881
3.093169	3.198603	3.447322	34.48031
3.299798	3.247733	3.371493	34.87379
3.268412	3.442434	3.41813	35.33292
3.290725	3.443579	3.387658	35.31758
3.231169	3.315059	3.444474	35.03054
3.241769	3.263975	3.39719	34.8386

3.369129	3.233513	3.354288	34.95669
3.395401	3.404626	3.396645	35.48096
3.208568	3.442366	3.45375	35.2798
3.255709	3.42855	3.441132	35.32508
3.181636	3.258988	3.38609	34.67192
3.393833	3.250496	3.329223	34.99304
3.213641	3.248609	3.408188	34.76754
3.195865	3.337097	3.469097	35.05537
3.231747	3.396134	3.400686	35.11334
3.35989	3.402806	3.354084	35.30625
3.434664	3.384003	3.344535	35.40777
3.509769	3.406041	3.397753	35.73658
3.307387	3.444321	3.475184	35.54705
3.318935	3.438457	3.346496	35.27806
3.307923	3.327325	3.375023	35.07333
3.321451	3.234187	3.348576	34.8414
3.13037	3.194964	3.355465	34.35283
3.122204	3.26539	3.379116	34.5407
3.179368	3.424574	3.479055	35.23237
3.340134	3.464673	3.438063	35.58199
3.275094	3.313846	3.427013	35.08576
3.3224	3.261212	3.380429	34.97223
3.286559	3.262492	3.443809	35.03526
3.327885	3.351317	3.33577	35.08362
3.343681	3.41797	3.412144	35.43094
3.294313	3.40038	3.379678	35.21351
3.292952	3.247329	3.360035	34.83288
3.2753	3.198805	3.367469	34.70442
3.270804	3.265458	3.390387	34.89046
3.214383	3.299694	3.40546	34.87491
3.2647	3.471345	3.434499	35.42383
3.226096	3.469458	3.449914	35.36899
3.353827	3.368907	3.360239	35.23232
3.363313	3.317823	3.34121	35.09974
3.447574	3.415543	3.407813	35.64335
3.215456	3.461978	3.416834	35.25702
3.305737	3.361494	3.418283	35.23788
3.241893	3.279004	3.437909	34.96079
3.299633	3.193683	3.30547	34.61085
3.131443	3.248137	3.418743	34.60984
3.24779	3.378005	3.422324	35.1561
3.217312	3.483207	3.555702	35.61118
3.307346	3.385486	3.339437	35.12144
3.318564	3.320181	3.500795	35.35603
3.365128	3.288574	3.425138	35.22328
3.297076	3.311622	3.443911	35.16592
3.346816	3.440007	3.359626	35.37113
3.290684	3.411163	3.453068	35.38965
3.373624	3.339254	3.321754	35.12661
3.296953	3.231222	3.335822	34.75345

3.083889	3.214912	3.377615	34.34325
3.205021	3.298413	3.397207	34.83359
3.153755	3.422889	3.453614	35.11704
3.313409	3.489811	3.377905	35.44696
3.337165	3.374164	3.367248	35.22271
3.309367	3.328201	3.38389	35.0978
3.278022	3.43347	3.437926	35.37763
3.397875	3.493922	3.346104	35.57112
3.295921	3.444253	3.458252	35.4848
3.29155	3.281026	3.4124	35.01802
3.264288	3.139836	3.357579	34.52975
3.245275	3.182631	3.407063	34.68997
3.242181	3.417228	3.37753	35.13165
3.282889	3.486981	3.537968	35.72406
3.172356	3.409748	3.393729	34.99802
3.306109	3.281969	3.43397	35.09909
3.308954	3.333188	3.382679	35.10515
3.43153	3.311488	3.435266	35.44075
3.326565	3.420531	3.431515	35.44147
3.290024	3.373288	3.430969	35.25705
3.254761	3.317014	3.391052	34.96958
3.366448	3.25582	3.311574	34.9062
3.292168	3.198671	3.382543	34.77398
3.237645	3.266266	3.365628	34.76557
3.040666	3.353743	3.389705	34.57876
3.215909	3.454362	3.390335	35.18341
3.347806	3.496348	3.374904	35.52992
3.393586	3.366346	3.392586	35.38441
3.282806	3.33521	3.463197	35.22847
3.335721	3.432324	3.386942	35.38981
3.382615	3.458675	3.39823	35.57467
3.422498	3.367896	3.307294	35.2645
3.20531	3.176768	3.477264	34.74327
3.16353	3.174678	3.333707	34.3334
3.272495	3.299491	3.444184	35.08624
3.227292	3.447758	3.329683	35.06122
3.320874	3.458473	3.412485	35.47038
3.168232	3.329077	3.438455	34.9104
3.211455	3.3034	3.411241	34.88926
3.295262	3.352328	3.439614	35.24157
3.407072	3.404289	3.352328	35.40883
3.193885	3.330088	3.430816	34.952
3.332752	3.338917	3.364622	35.13023
3.276908	3.293089	3.394411	34.97304
3.418084	3.255281	3.285809	34.96159
3.240779	3.211543	3.334662	34.58504
3.241356	3.275836	3.350691	34.76195
3.167242	3.380768	3.43131	35.00565
3.246429	3.400986	3.345149	35.03461
3.292086	3.38279	3.357511	35.1217

3.378903	3.441692	3.337271	35.3961
3.264865	3.396336	3.485875	35.37251
3.307305	3.491968	3.350861	35.37919
3.347228	3.41123	3.474007	35.55924
3.358117	3.331975	3.316519	35.06533
3.250801	3.17023	3.379781	34.61528
3.190875	3.183911	3.3511	34.45143
3.189761	3.322271	3.42021	34.90269
3.225808	3.457462	3.409808	35.25442
3.256328	3.410017	3.428224	35.25768
3.264535	3.344106	3.459752	35.20044
3.209929	3.3497	3.466232	35.10743
3.261442	3.357989	3.445395	35.19264
3.417548	3.371266	3.359012	35.37414
3.265071	3.410961	3.46439	35.35795
3.30549	3.364729	3.361211	35.1196
3.309985	3.23513	3.425172	34.9859
3.374738	3.258314	3.336538	34.98437
3.240202	3.237759	3.370453	34.71938
3.286765	3.340062	3.351219	35.00286
3.070567	3.412174	3.467647	34.94238
3.305284	3.477209	3.344944	35.32956
3.233685	3.394786	3.358875	35.0232
3.277444	3.397617	3.36145	35.13072
3.261854	3.426192	3.433681	35.31707
3.372634	3.447353	3.429878	35.59729
3.241563	3.36729	3.360853	34.98462
3.336216	3.211543	3.357545	34.84379
3.233891	3.170635	3.427065	34.68259
3.302232	3.380431	3.376132	35.17945
3.198628	3.445331	3.414446	35.1786
3.229973	3.456991	3.413577	35.27074
3.284126	3.360415	3.436682	35.22849
3.288415	3.360415	3.385203	35.1253
3.321822	3.371738	3.45566	35.37719
3.30714	3.353137	3.429861	35.24799
3.269031	3.399773	3.404096	35.21029
3.358075	3.320451	3.393115	35.20754
3.402907	3.228189	3.352242	35.01444
3.288333	3.197457	3.394667	34.78945
3.286106	3.249081	3.375586	34.85575
3.196277	3.285743	3.410644	34.81615
3.277279	3.415409	3.378843	35.2073
3.303964	3.433807	3.427815	35.41298
3.262102	3.433605	3.405119	35.27136
3.23649	3.387171	3.392825	35.08692
3.354116	3.389395	3.372994	35.30565
3.208073	3.420126	3.369788	35.04647
3.400968	3.404423	3.400004	35.50004
3.210878	3.283047	3.453597	34.93611

3.26503	3.201771	3.331354	34.60947
3.317451	3.220236	3.380019	34.87091
3.410413	3.405839	3.365679	35.44873
3.208857	3.414263	3.433987	35.17576
3.242635	3.42727	3.382406	35.16527
3.161592	3.321125	3.413219	34.8233
3.335556	3.399571	3.440433	35.4348
3.307841	3.388114	3.448072	35.36584
3.25707	3.328808	3.487973	35.21237
3.233809	3.340871	3.379184	34.94998
3.363396	3.283721	3.4028	35.16003
3.358983	3.210734	3.307158	34.78162
3.320956	3.207499	3.350691	34.78658
3.188854	3.252585	3.411735	34.72979
3.141547	3.375579	3.362405	34.78743
3.312584	3.452745	3.39673	35.40527
3.355353	3.444455	3.375722	35.43473
3.322895	3.441018	3.43675	35.48969
3.197144	3.433268	3.391819	35.09949
3.259132	3.412039	3.447799	35.31104
3.357168	3.417767	3.34624	35.31586
3.342362	3.339523	3.388068	35.20385
3.20465	3.163221	3.431225	34.61153
3.221683	3.236411	3.425717	34.79679
3.237273	3.372816	3.367469	35.0018
3.384389	3.481791	3.367384	35.56164
3.207702	3.346937	3.381776	34.91182
3.1267	3.325977	3.435624	34.80661
3.199123	3.34633	3.444798	35.02955
3.325905	3.480039	3.377427	35.45188
3.221848	3.374298	3.431703	35.11177
3.287343	3.350845	3.43588	35.21285
3.182255	3.238163	3.507292	34.89279
3.362282	3.20541	3.271776	34.69981
3.367809	3.225965	3.368254	34.96783
3.361622	3.313038	3.388068	35.18805
3.206176	3.343028	3.43409	35.01434
3.278805	3.423226	3.31079	35.07891
3.21694	3.431785	3.414992	35.19021
3.463535	3.446005	3.317952	35.54836
3.230551	3.406176	3.449504	35.23945
3.172191	3.369851	3.45346	35.04104
3.277692	3.373894	3.403653	35.17167
3.398081	3.315733	3.347348	35.18463
3.349208	3.190448	3.376268	34.86701
3.237768	3.178992	3.429179	34.71397
3.206258	3.298144	3.40633	34.85566
3.245027	3.422957	3.40558	35.21174
3.436355	3.447623	3.30327	35.46035
3.248822	3.358124	3.467562	35.21381

3.207413	3.381442	3.405051	35.03755
3.132309	3.391484	3.45375	35.00176
3.339021	3.445466	3.361825	35.37083
3.251791	3.339254	3.455097	35.15178
3.262679	3.323618	3.473036	35.18062
3.194834	3.230413	3.394411	34.65649
3.457142	3.231828	3.355277	35.14763
3.303057	3.276375	3.35081	34.89832
3.371026	3.354957	3.427013	35.38545
3.233438	3.361561	3.391904	35.02223
3.260865	3.38899	3.4146	35.19182
3.30648	3.472693	3.33589	35.3025
3.359065	3.481387	3.407336	35.59275
3.194586	3.43192	3.365474	35.03334
3.288828	3.394988	3.449931	35.34335
3.177677	3.309533	3.428378	34.86628
3.394246	3.221045	3.387437	35.05684
3.420394	3.193683	3.388289	35.05605
3.346321	3.289787	3.420449	35.17455
3.286229	3.414937	3.387437	35.24463
3.269814	3.431785	3.34965	35.16295
3.386863	3.329953	3.365628	35.23117
3.354363	3.37841	3.363445	35.26129
3.162128	3.408804	3.45774	35.11357
3.180852	3.458541	3.406125	35.15041
3.280496	3.370659	3.508077	35.39909
3.255503	3.279139	3.384572	34.87421
3.28062	3.234996	3.459923	34.99738
3.216404	3.235333	3.374358	34.67057
3.257771	3.254472	3.400362	34.85975
3.274805	3.347071	3.341397	34.97056
3.350775	3.37969	3.411855	35.3621
3.262308	3.414802	3.369021	35.15175
3.284332	3.406378	3.377274	35.19954
3.270021	3.363381	3.334526	34.98073
3.3808	3.440075	3.345746	35.41525
3.181471	3.404289	3.45832	35.14727
3.273691	3.386632	3.4412	35.27289
3.225931	3.28453	3.426894	34.91388
3.326359	3.270782	3.392774	35.02882
3.332092	3.211408	3.401146	34.92982
3.428148	3.40995	3.370317	35.50664
3.25938	3.430774	3.494657	35.45502
3.334979	3.40375	3.337049	35.21659
3.249729	3.322136	3.39262	34.97321
3.348466	3.380566	3.355687	35.23614
3.24944	3.429494	3.411309	35.24822
3.173882	3.454969	3.454074	35.23222
3.210259	3.293898	3.45121	34.95327
3.277321	3.224348	3.411872	34.8618

3.310975	3.20696	3.429997	34.93701
3.28128	3.227582	3.414651	34.88361
3.307676	3.35617	3.451005	35.30203
3.254183	3.327999	3.410712	35.03533
3.403855	3.37167	3.340903	35.30548
3.389627	3.428618	3.374887	35.47322
3.340506	3.439199	3.35983	35.35601
3.25905	3.458271	3.394172	35.29469
3.290312	3.454767	3.40737	35.38425
3.228035	3.367829	3.37354	34.98396
3.305861	3.29875	3.325284	34.89756
3.151735	3.221854	3.444065	34.65211
3.263257	3.257842	3.357648	34.78571
3.255338	3.333188	3.417584	35.06423
3.343145	3.449038	3.418283	35.51113
3.273526	3.417633	3.441712	35.34144
3.32739	3.30495	3.435676	35.19961
3.212775	3.350037	3.406671	34.98414
3.370283	3.43064	3.344552	35.369
3.164108	3.461641	3.394291	35.0947
3.234386	3.383734	3.452915	35.20621
3.186338	3.223269	3.403602	34.64239
3.206959	3.214778	3.441081	34.75088
3.333288	3.276578	3.45005	35.1819
3.400597	3.317014	3.387812	35.28142
3.301366	3.353878	3.429997	35.23728
3.314646	3.35435	3.373113	35.14296
3.321286	3.332851	3.362712	35.08772
3.401505	3.398156	3.329837	35.33406
3.295303	3.451532	3.329444	35.21768
3.1705	3.406715	3.353214	34.89873
3.219497	3.356709	3.424217	35.0518
3.237645	3.307107	3.359063	34.84053
3.317616	3.271725	3.356284	34.93196
3.250389	3.257842	3.405324	34.86183
3.224488	3.334401	3.439359	35.04704
3.198628	3.391888	3.379798	34.98595
3.442212	3.420665	3.398912	35.62336
3.315966	3.349363	3.402459	35.19911
3.300994	3.355091	3.415418	35.20724
3.227004	3.383666	3.366566	35.00109
3.270186	3.459012	3.401504	35.3367
3.217312	3.413387	3.352174	35.01342
3.270722	3.277049	3.445821	35.03686
3.13932	3.216125	3.46439	34.65688
3.237933	3.264649	3.428241	34.89959
3.293364	3.279813	3.423739	35.04413
3.351311	3.317486	3.407353	35.2174
3.333659	3.371603	3.401624	35.28461
3.350322	3.373827	3.397548	35.317

3.281651	3.38616	3.430151	35.2651
3.367396	3.458204	3.31993	35.36912
3.277651	3.400582	3.387931	35.19556
3.327514	3.343971	3.290021	34.96669
3.238923	3.219158	3.33751	34.60386
3.274145	3.255416	3.321311	34.72475
3.232984	3.330425	3.446435	35.0724
3.333041	3.397752	3.380821	35.29495
3.283961	3.352598	3.456785	35.255
3.280167	3.370524	3.401078	35.16408
3.280703	3.291606	3.491332	35.19004
3.341207	3.384812	3.366259	35.25267
3.292993	3.409141	3.436733	35.35455
3.25307	3.377736	3.368475	35.0493
3.231829	3.345252	3.442223	35.09309
3.186791	3.296863	3.394394	34.78418
3.25905	3.233985	3.355312	34.71923
3.222962	3.194694	3.424694	34.70612
3.300788	3.311151	3.404215	35.0862
3.237273	3.340467	3.481101	35.17955
3.372387	3.430909	3.357903	35.40339
3.419858	3.383262	3.393541	35.48094
3.34397	3.427405	3.353146	35.32318
3.267051	3.409478	3.496771	35.42985
3.329824	3.415004	3.381452	35.32703
3.243171	3.325168	3.383941	34.94652
3.334113	3.283384	3.300695	34.87197
3.138289	3.241263	3.414856	34.60128
3.161922	3.313038	3.426996	34.83646
3.13268	3.372816	3.397958	34.83974
3.314522	3.3652	3.388954	35.20106
3.237273	3.390203	3.452301	35.22533
3.395401	3.366211	3.437347	35.48596
3.222838	3.397752	3.426877	35.15467
3.344589	3.45726	3.388375	35.46686
3.37515	3.39364	3.339488	35.28766
3.332051	3.362707	3.407899	35.27536
3.128556	3.264986	3.425087	34.65424
3.21327	3.230076	3.419084	34.75003
3.151652	3.21188	3.405307	34.54536
3.26569	3.370727	3.367469	35.05937
3.196937	3.364122	3.475832	35.13155
3.28128	3.399032	3.46746	35.37403
3.325905	3.354957	3.440842	35.31702
3.418992	3.403143	3.371135	35.47352
3.34463	3.449712	3.426041	35.53282
3.300912	3.420463	3.358244	35.22499
3.288621	3.314655	3.437313	35.13963
3.253235	3.271388	3.293721	34.65362
3.321864	3.256494	3.358176	34.91208

3.229355	3.302861	3.368816	34.83445
3.250018	3.301715	3.416765	34.98198
3.181306	3.387036	3.440569	35.07036
3.379522	3.355024	3.418556	35.38568
3.342155	3.373557	3.411599	35.32928
3.353002	3.356372	3.339505	35.15776
3.233149	3.44021	3.527822	35.49082
3.321822	3.481859	3.382679	35.45841
3.212981	3.338782	3.460162	35.07695
3.376759	3.262155	3.320987	34.96318
3.132886	3.174678	3.437159	34.49262
3.177223	3.203792	3.366566	34.49887
3.162169	3.284193	3.399731	34.7143
3.262184	3.347476	3.387624	35.04493
3.177182	3.392495	3.466829	35.1307
3.351394	3.395123	3.422904	35.42137
3.20201	3.3466	3.38759	34.91135
3.381213	3.419722	3.407558	35.50681
3.360632	3.45389	3.391938	35.50237
3.350404	3.450925	3.39187	35.47337
3.284002	3.314655	3.360717	34.96203
3.224323	3.199681	3.38684	34.63722
3.295344	3.288574	3.33178	34.86652
3.302273	3.348689	3.354783	35.06344
3.246264	3.44924	3.418965	35.3012
3.170129	3.351115	3.413168	34.90744
3.275753	3.332851	3.398623	35.06668
3.357787	3.375781	3.368168	35.27335
3.345744	3.442905	3.406807	35.4783
3.282517	3.489205	3.479737	35.60077
3.224034	3.39081	3.451056	35.19499
3.243501	3.297672	3.383617	34.8864
3.344589	3.212149	3.361024	34.87103
3.27464	3.253327	3.381486	34.85286
3.130742	3.226639	3.396338	34.51229
3.115317	3.296594	3.441933	34.73125
3.286188	3.399975	3.392552	35.22301
3.253977	3.403615	3.388545	35.15177
3.372098	3.365874	3.394956	35.34157
3.273238	3.367559	3.412144	35.16665
3.330154	3.453756	3.412673	35.48077
3.396803	3.456923	3.363377	35.52564
3.451162	3.322136	3.362149	35.34707
3.255792	3.236209	3.369226	34.7474
3.215414	3.233917	3.331371	34.5713
3.178172	3.372748	3.372193	34.88273
3.343269	3.415139	3.342983	35.2726
3.269155	3.346465	3.471279	35.24091
3.171325	3.33676	3.449095	34.95723
3.229231	3.314385	3.405187	34.93891

3.420848	3.395932	3.403465	35.53251
3.308501	3.417228	3.428309	35.38773
3.298644	3.439468	3.39378	35.3393
3.218714	3.251777	3.459769	34.89836
3.163283	3.245711	3.337714	34.49696
3.399195	3.231491	3.373608	35.06026
3.252286	3.291	3.397548	34.92149
3.203165	3.286484	3.410883	34.83335
3.080507	3.294841	3.450272	34.66953
3.299963	3.426798	3.39187	35.3103
3.345785	3.407928	3.342182	35.26058
3.289323	3.341006	3.429588	35.1819
3.192359	3.377736	3.39835	34.98187
3.310563	3.474513	3.382611	35.41758
3.332711	3.386429	3.359285	35.22237
3.430829	3.30549	3.372789	35.28947
3.237768	3.169085	3.402459	34.63387
3.178172	3.277049	3.401896	34.73841
3.232407	3.349093	3.363019	34.92954
3.324998	3.432594	3.321754	35.22439
3.267134	3.35408	3.434022	35.17166
3.210011	3.338782	3.415469	34.97272
3.154374	3.378477	3.494998	35.11177
3.470092	3.499988	3.319725	35.68463
3.377625	3.398156	3.461645	35.57008
3.33568	3.337906	3.413713	35.24178
3.27266	3.282913	3.406824	34.96864
3.189431	3.236815	3.349275	34.55997
3.316997	3.218282	3.308113	34.7084
3.281651	3.238298	3.369584	34.8093
3.170706	3.29383	3.449027	34.86185
3.20832	3.368907	3.415708	35.03542
3.342362	3.399234	3.390506	35.33976
3.33502	3.390069	3.403533	35.33215
3.266102	3.358731	3.450459	35.21552
3.136268	3.410758	3.460827	35.06805
3.369995	3.471413	3.409808	35.60024
3.346403	3.352598	3.338601	35.1331
3.396143	3.238568	3.307772	34.92509
3.279424	3.184653	3.390233	34.73227
3.130453	3.381779	3.377479	34.80969
3.239336	3.38899	3.427065	35.172
3.371108	3.439064	3.318838	35.333
3.193432	3.32126	3.44427	34.96113
3.275382	3.367963	3.424967	35.20026
3.254678	3.397212	3.483693	35.34737
3.352714	3.467908	3.407029	35.54871
3.376387	3.388384	3.37504	35.35662
3.382368	3.284126	3.422103	35.24462
3.257813	3.245577	3.413355	34.86881

3.285528	3.285608	3.33855	34.85337
3.268247	3.206825	3.349326	34.66686
3.308748	3.24807	3.333162	34.81028
3.203577	3.334604	3.450817	35.02681
3.160726	3.378073	3.425376	34.97253
3.288374	3.388384	3.443298	35.31341
3.334484	3.384205	3.350247	35.20163
3.264576	3.330493	3.453392	35.15685
3.264948	3.443647	3.457638	35.4144
3.377625	3.35563	3.389346	35.31898
3.329576	3.237624	3.370658	34.91498
3.405464	3.221854	3.292238	34.87495
3.241851	3.319507	3.428633	35.02899
3.248986	3.45908	3.349241	35.17619
3.301283	3.374703	3.361757	35.13341
3.331886	3.387373	3.394462	35.29956
3.191369	3.354283	3.342693	34.8067
3.2899	3.448836	3.395894	35.34529
3.169923	3.479096	3.496908	35.36999
3.401381	3.44122	3.379798	35.53722
3.208073	3.232233	3.406466	34.71579
3.401587	3.237759	3.407779	35.15393
3.310604	3.261212	3.41784	35.02825
3.305366	3.240387	3.34358	34.80886
3.269031	3.231356	3.42686	34.89177
3.252905	3.257505	3.365901	34.78038
3.242429	3.369244	3.409621	35.09744
3.255255	3.507468	3.414088	35.43753
3.149796	3.425383	3.498153	35.21124
3.273238	3.369446	3.462054	35.27992
3.273815	3.329414	3.375313	35.00395
3.292292	3.425652	3.493293	35.51281
3.290519	3.361898	3.361876	35.08213
3.322111	3.286889	3.42836	35.13257
3.295592	3.214845	3.305095	34.64747
3.328834	3.37969	3.324499	35.12309
3.185183	3.471008	3.379388	35.12868
3.253812	3.40321	3.378365	35.12826
3.307305	3.352463	3.396031	35.17289
3.197432	3.426596	3.397224	35.09735
3.318069	3.470402	3.421131	35.50924
3.20931	3.458541	3.452778	35.31467
3.299386	3.387373	3.405102	35.25176
3.168603	3.251844	3.42154	34.70532
3.362406	3.250968	3.383344	35.0437
3.264659	3.282643	3.422239	34.98426
3.345537	3.253731	3.343137	34.92492
3.230138	3.250564	3.40176	34.79384
3.292333	3.294707	3.377496	34.97332
3.224447	3.376792	3.366617	34.98058

3.304583	3.38926	3.407626	35.27277
3.211867	3.373018	3.414668	35.0499
3.195989	3.336221	3.432061	34.97274
3.325575	3.41615	3.439734	35.44769
3.471206	3.429831	3.365628	35.63403
3.301118	3.210869	3.359233	34.76925
3.272207	3.190516	3.379286	34.70537
3.266185	3.240657	3.415555	34.88117
3.273155	3.390743	3.354527	35.09116
3.280208	3.455238	3.324005	35.18088
3.206423	3.358933	3.427286	35.03478
3.236572	3.27651	3.366992	34.78861
3.221642	3.460023	3.417857	35.26851
3.320915	3.534965	3.398708	35.60762
3.311223	3.391214	3.429929	35.34034
3.304995	3.198131	3.474349	35.00161
3.182791	3.258112	3.376882	34.6524
3.406701	3.279813	3.37047	35.17549
3.340134	3.369311	3.372994	35.23116
3.303758	3.281834	3.427764	35.08008
3.234881	3.276241	3.372482	34.79633
3.30017	3.268827	3.433834	35.05706
3.297447	3.381375	3.341602	35.09554
3.298272	3.400245	3.414327	35.29765
3.074114	3.387373	3.484017	34.9317
3.24379	3.418778	3.483966	35.37132
3.235335	3.374096	3.365491	34.99603
3.362488	3.313644	3.385732	35.18616
3.225849	3.208106	3.356625	34.5929
3.331886	3.27496	3.407575	35.08241
3.210506	3.348352	3.462481	35.09754
3.368139	3.457597	3.280779	35.2838
3.230798	3.36884	3.345285	34.93043
3.291426	3.353069	3.340306	35.01764
3.083683	3.323484	3.454432	34.74821
3.240944	3.565831	3.427695	35.56362
3.316584	3.484892	3.501137	35.71264
3.363849	3.305085	3.35983	35.11378
3.286188	3.133029	3.427883	34.7165
3.26536	3.244633	3.402186	34.85882
3.335639	3.401728	3.448822	35.45804
3.35692	3.368166	3.351321	35.21796
3.324173	3.28271	3.354306	34.966
3.211537	3.254944	3.411377	34.78377
3.315388	3.406243	3.353282	35.2147
3.306274	3.402536	3.40824	35.30684
3.295303	3.454969	3.422495	35.42868
3.220405	3.36365	3.433953	35.09025
3.233025	3.363179	3.468704	35.19282
3.18675	3.293089	3.432248	34.85862

3.510511	3.278262	3.377768	35.41507
3.21162	3.253731	3.441064	34.84622
3.31015	3.368166	3.342762	35.09697
3.287137	3.398762	3.470802	35.39355
3.416022	3.467976	3.312546	35.48068
3.317327	3.311285	3.353197	35.01109
3.303015	3.31324	3.408871	35.10582
3.167778	3.474243	3.417294	35.18059
3.317079	3.532876	3.437091	35.6786
3.240408	3.351924	3.414054	35.06484
3.29023	3.186405	3.433425	34.85419
3.316131	3.218754	3.403124	34.91531
3.303263	3.307646	3.472831	35.234
3.329865	3.392293	3.392024	35.30057
3.340588	3.303737	3.401743	35.15162
3.280991	3.26229	3.351765	34.82136
3.316997	3.318092	3.382321	35.08895
3.197061	3.394314	3.40205	35.03649
3.249234	3.439266	3.338601	35.11014
3.274846	3.398425	3.406006	35.22424
3.271134	3.316205	3.440314	35.11134
3.34595	3.354417	3.388204	35.24456
3.242223	3.331571	3.423262	35.04443
3.327473	3.297807	3.352975	35.00332
3.318069	3.264784	3.40928	35.03367
3.259957	3.337636	3.358534	34.95493
3.308171	3.382049	3.359489	35.15958
3.414125	3.378746	3.366259	35.39886
3.241521	3.360281	3.356335	34.95932
3.317327	3.379892	3.341619	35.13581
3.175449	3.487992	3.430083	35.25539
3.18143	3.435964	3.476668	35.25657
3.236284	3.335749	3.346053	34.87174
3.24911	3.182159	3.456461	34.80536
3.304706	3.202242	3.419101	34.88916
3.363107	3.350913	3.450817	35.41135
3.253276	3.41487	3.484972	35.38571
3.336505	3.295583	3.310636	34.92562
3.353044	3.272871	3.436289	35.1869
3.382409	3.353069	3.312137	35.155
3.276743	3.314116	3.408922	35.05039
3.232077	3.415813	3.352737	35.05224
3.236242	3.461573	3.443707	35.36036
3.370737	3.429359	3.333878	35.34385
3.319471	3.312498	3.435863	35.19921
3.223787	3.298211	3.339778	34.7486
3.29588	3.246722	3.430338	34.9917
3.359808	3.374231	3.394939	35.33293
3.352755	3.406513	3.356284	35.30356
3.291838	3.389597	3.417993	35.26831

3.323307	3.312431	3.366429	35.05561
3.200897	3.309129	3.36261	34.77235
3.329865	3.432594	3.280626	35.14509
3.260658	3.494866	3.454142	35.50938
3.259751	3.333391	3.429605	35.10062
3.095973	3.190044	3.408529	34.3829
3.335804	3.263975	3.390762	35.03019
3.319389	3.407119	3.409757	35.34886
3.33535	3.458878	3.481988	35.65491
3.279259	3.366885	3.434175	35.22652
3.323596	3.243487	3.402596	34.98456
3.332793	3.2817	3.341619	34.9549
3.425467	3.351991	3.322163	35.26873
3.143362	3.435694	3.439819	35.09215
3.222426	3.460293	3.380701	35.18956
3.178213	3.30987	3.469421	34.95794
3.34463	3.257505	3.306766	34.85165
3.349868	3.24989	3.399663	35.0496
3.222178	3.302457	3.442752	34.97955
3.144971	3.480107	3.473922	35.26737
3.395483	3.450049	3.423586	35.63939
3.410578	3.394517	3.334867	35.35695
3.354776	3.295448	3.294472	34.92993
3.200196	3.319642	3.41622	34.91104
3.203248	3.370861	3.344347	34.87255
3.340629	3.519869	3.394343	35.60817
3.31576	3.382049	3.336418	35.12572
3.163241	3.180002	3.466709	34.63527
3.198628	3.165445	3.405375	34.54669
3.279053	3.353272	3.502313	35.3453
3.381873	3.47923	3.429878	35.6872
3.34166	3.401054	3.362678	35.28135
3.211496	3.202242	3.439768	34.73051
3.276001	3.264312	3.364673	34.84309
3.273155	3.341545	3.354152	34.98276
3.365705	3.407321	3.392211	35.41222
3.243089	3.437177	3.40662	35.24088
3.222508	3.35772	3.390165	34.98613
3.153261	3.249283	3.389278	34.59562
3.385048	3.236074	3.358176	35.0056
3.169428	3.334334	3.408393	34.85877
3.302809	3.402671	3.417055	35.31884
3.245605	3.402199	3.434772	35.23145
3.421508	3.38771	3.333093	35.36208
3.468525	3.353743	3.338277	35.40196
3.376924	3.367424	3.342489	35.24077
3.172356	3.413117	3.427269	35.07874
3.220364	3.472963	3.416169	35.29032
3.196525	3.366076	3.370863	34.90537
3.267587	3.178183	3.346581	34.59678

3.190875	3.163289	3.412605	34.54083
3.182337	3.316879	3.461236	34.96439
3.20997	3.507738	3.454569	35.42761
3.386203	3.437581	3.426195	35.59754
3.310315	3.300368	3.433902	35.14837
3.277486	3.295246	3.393251	34.97648
3.247131	3.317553	3.415674	35.00792
3.28858	3.372277	3.370743	35.11998
3.315471	3.422418	3.351134	35.24555
3.305078	3.376859	3.366344	35.15645
3.283466	3.267951	3.374801	34.88952
3.243006	3.226504	3.371238	34.70261
3.294478	3.254405	3.433271	35.01185
3.234139	3.346398	3.361791	34.92475
3.397545	3.396134	3.430679	35.54151
3.276455	3.394921	3.422972	35.2572
3.357787	3.345454	3.425393	35.33217
3.392307	3.370861	3.282433	35.1506
3.370407	3.44459	3.497931	35.7352
3.211249	3.470335	3.359745	35.14125
3.254059	3.43529	3.410644	35.26954
3.18242	3.247868	3.418675	34.72058
3.246099	3.200692	3.334236	34.57201
3.130288	3.27213	3.457365	34.74424
3.244986	3.46346	3.345831	35.16957
3.205928	3.401256	3.520865	35.3309
3.343186	3.361359	3.340204	35.14873
3.361457	3.256023	3.387914	35.06267
3.407815	3.339456	3.357818	35.28069
3.234716	3.372344	3.447867	35.17099
3.28825	3.381173	3.35596	35.10638
3.23451	3.352261	3.38123	34.9809
3.324833	3.326516	3.370385	35.0984
3.279135	3.28116	3.372926	34.90484
3.16951	3.263031	3.410098	34.70675
3.274186	3.293359	3.415316	35.01339
3.261483	3.389462	3.364673	35.08503
3.361127	3.34141	3.413918	35.30554
3.284373	3.405839	3.388937	35.22396
3.301407	3.379488	3.416765	35.26444
3.313986	3.406378	3.323374	35.14652
3.397545	3.457597	3.338601	35.47456
3.264164	3.428887	3.431583	35.32343
3.249605	3.388384	3.355942	35.0376
3.169634	3.189505	3.427337	34.58393
3.305449	3.210667	3.317457	34.68692
3.247873	3.340399	3.46098	35.15858
3.283755	3.445534	3.406944	35.34879
3.235005	3.386901	3.469966	35.25178
3.302603	3.341276	3.37579	35.09389

3.296004	3.238972	3.382134	34.8696
3.443325	3.274556	3.458303	35.43616
3.299345	3.356844	3.422154	35.22219
3.308996	3.418441	3.458405	35.45728
3.281816	3.377129	3.391614	35.16144
3.367066	3.239848	3.331167	34.91547
3.310109	3.209656	3.357852	34.78324
3.163984	3.318025	3.412554	34.8203
3.135031	3.428011	3.469284	35.12157
3.339763	3.457665	3.416629	35.51898
3.365582	3.431718	3.432879	35.55424
3.431489	3.347274	3.355243	35.34392
3.312708	3.299424	3.416629	35.11377
3.298767	3.425585	3.366208	35.24891
3.342939	3.450049	3.378775	35.42649
3.3337	3.384003	3.346291	35.19082
3.155405	3.237422	3.397804	34.59302
3.112595	3.18654	3.378519	34.34595
3.276413	3.349295	3.380889	35.0653
3.236284	3.456788	3.426314	35.31195
3.292333	3.43839	3.436375	35.41629
3.201474	3.361359	3.465874	35.11365
3.180192	3.227919	3.405938	34.64423
3.285899	3.214778	3.379474	34.78878
3.445594	3.422215	3.486148	35.82492
3.247337	3.447758	3.416561	35.29504
3.279672	3.345589	3.444201	35.20277
3.297241	3.253799	3.328779	34.78806
3.394741	3.190988	3.31033	34.82357
3.247997	3.270377	3.334321	34.72874
3.213105	3.416217	3.437244	35.19644
3.221518	3.46191	3.43027	35.29951
3.333206	3.443849	3.371357	35.37542
3.385172	3.344241	3.388528	35.30879
3.419775	3.406445	3.39112	35.52616
3.300211	3.470806	3.471262	35.5807
3.387441	3.491361	3.323527	35.49333
3.313532	3.323147	3.40205	35.13557
3.304748	3.171578	3.329871	34.62706
3.214837	3.142329	3.381452	34.47927
3.122534	3.27159	3.354783	34.50177
3.197061	3.474917	3.446913	35.31087
3.239707	3.478287	3.396235	35.30067
3.252781	3.385958	3.496038	35.34561
3.296251	3.338984	3.430151	35.19386
3.311347	3.296661	3.4722	35.22627
3.288374	3.419789	3.385749	35.25624
3.391812	3.381779	3.426024	35.4874
3.254761	3.350576	3.419511	35.10521
3.252657	3.301446	3.36527	34.87455

3.300788	3.221854	3.365167	34.80553
3.352714	3.224954	3.341312	34.87369
3.229478	3.291741	3.328097	34.72135
3.250595	3.393236	3.424814	35.20099
3.170459	3.460293	3.449914	35.22727
3.370902	3.414465	3.355448	35.35881
3.338072	3.375646	3.386465	35.26996
3.409465	3.43192	3.406074	35.59203
3.331721	3.457193	3.411121	35.48832
3.350115	3.362707	3.358313	35.20643
3.243254	3.350778	3.397395	35.03212
3.316832	3.205477	3.313245	34.69126
3.134618	3.163761	3.403533	34.399
3.268288	3.343837	3.346581	34.96057
3.115317	3.404019	3.436682	34.95469
3.228695	3.411769	3.348713	35.0272
3.300829	3.358528	3.469284	35.33219
3.32739	3.353609	3.355039	35.12968
3.291921	3.270849	3.44768	35.07372
3.296499	3.37349	3.391989	35.18641
3.262514	3.401795	3.444474	35.28876
3.386245	3.38151	3.347127	35.3021
3.274475	3.289113	3.329819	34.81777
3.236325	3.176498	3.366719	34.56877
3.264576	3.282643	3.398213	34.93154
3.169428	3.4146	3.470256	35.16958
3.27464	3.431988	3.377751	35.2354
3.305655	3.450723	3.42524	35.44804
3.309738	3.32746	3.407097	35.14774
3.324751	3.370457	3.442837	35.35275
3.419322	3.428618	3.360563	35.50684
3.266391	3.428753	3.379815	35.21479
3.442748	3.298413	3.304839	35.15147
3.239418	3.207769	3.457075	34.84151
3.312873	3.229874	3.315888	34.74173
3.24812	3.276241	3.365406	34.80981
3.3118	3.458608	3.374375	35.36749
3.137423	3.437716	3.454961	35.1167
3.219662	3.365605	3.456461	35.14213
3.227705	3.356507	3.404011	35.02512
3.328463	3.329549	3.437671	35.26011
3.263917	3.35435	3.433016	35.16302
3.283425	3.364931	3.470717	35.31126
3.285652	3.349969	3.461372	35.26298
3.35325	3.279004	3.425513	35.1772
3.315512	3.197794	3.353334	34.75924
3.282682	3.185933	3.361194	34.67869
3.209269	3.309129	3.381315	34.83156
3.234551	3.483274	3.414463	35.34017
3.312089	3.488329	3.334594	35.34612

3.346651	3.379151	3.405307	35.33759
3.371273	3.37814	3.426996	35.43665
3.336381	3.390675	3.455643	35.45041
3.411238	3.48725	3.384657	35.67007
3.382698	3.440749	3.345285	35.41986
3.375274	3.224078	3.359557	34.96101
3.230509	3.128716	3.396031	34.51566
3.260617	3.205545	3.426195	34.81547
3.267464	3.420665	3.31952	35.0676
3.357333	3.448432	3.41917	35.54277
3.114121	3.345319	3.431703	34.81282
3.215002	3.333593	3.437722	35.02095
3.205227	3.377466	3.394326	35.00062
3.291797	3.353811	3.426792	35.2092
3.21727	3.335008	3.447271	35.04989
3.327349	3.385621	3.432862	35.36978
3.220157	3.356844	3.448908	35.10753
3.440686	3.263705	3.322726	35.11017
3.348383	3.178587	3.293261	34.65775
3.321699	3.211138	3.38609	34.87358
3.117502	3.344241	3.380838	34.70662
3.245027	3.44641	3.375501	35.19725
3.270309	3.458069	3.40812	35.34937
3.387729	3.418374	3.32646	35.34077
3.181718	3.375983	3.429605	35.02311
3.307346	3.399571	3.412264	35.3115
3.304046	3.424776	3.498562	35.54813
3.353002	3.350576	3.31432	35.09001
3.30813	3.165445	3.380906	34.73265
3.244532	3.148664	3.371272	34.5358
3.235046	3.296796	3.425428	34.95743
3.24016	3.474041	3.35717	35.20695
3.240078	3.426057	3.436	35.27422
3.24478	3.330088	3.424592	35.04969
3.184317	3.384003	3.425018	35.0363
3.163241	3.31479	3.40633	34.79799
3.360096	3.402739	3.364929	35.33027
3.260782	3.396538	3.408291	35.19435
3.330442	3.370996	3.397122	35.26641
3.305036	3.237085	3.421898	34.97219
3.369747	3.184653	3.283303	34.69595
3.279919	3.181283	3.346394	34.63011
3.380718	3.307309	3.394479	35.2313
3.147486	3.396673	3.497317	35.14158
3.347888	3.488463	3.314575	35.38093
3.253565	3.395662	3.38418	35.12393
3.371562	3.408939	3.339965	35.31431
3.281239	3.399302	3.447884	35.33172
3.280084	3.485094	3.440706	35.50111
3.226096	3.376792	3.430969	35.12491

3.380718	3.214306	3.342966	34.91527
3.319884	3.19584	3.342028	34.7398
3.34463	3.282171	3.373454	35.05143
3.245852	3.428213	3.388323	35.18731
3.20036	3.459552	3.413287	35.21095
3.221188	3.383464	3.334713	34.91827
3.312625	3.315194	3.388272	35.08606
3.22156	3.377736	3.409467	35.07003
3.217765	3.378949	3.412059	35.07006
3.223416	3.304883	3.4109	34.91791
3.317863	3.331301	3.441951	35.25013
3.323348	3.227717	3.35243	34.83983
3.32339	3.169691	3.408649	34.83597
3.284703	3.212284	3.322948	34.6571
3.212404	3.251305	3.449948	34.86205
3.345084	3.447151	3.389602	35.44852
3.297901	3.454901	3.398418	35.38157
3.194751	3.450116	3.377206	35.09914
3.25542	3.362976	3.380633	35.04875
3.319307	3.381442	3.373403	35.21303
3.26668	3.394988	3.362217	35.10311
3.360756	3.379016	3.464919	35.4985
3.261813	3.280015	3.406961	34.93888
3.359643	3.165782	3.364536	34.81024
3.320791	3.229874	3.355925	34.8466
3.372057	3.412309	3.417055	35.49135
3.290395	3.432527	3.434516	35.39516
3.304005	3.387103	3.343051	35.12557
3.17116	3.343634	3.43247	34.93555
3.358694	3.406647	3.374068	35.35574
3.264824	3.390473	3.451499	35.28442
3.166747	3.353946	3.460076	35.00882
3.176646	3.343702	3.43588	34.95515
3.388513	3.325034	3.404164	35.30829
3.357457	3.244903	3.385186	35.02364
3.388843	3.241533	3.343512	34.99377
3.231087	3.216462	3.394701	34.7059
3.197968	3.332312	3.351168	34.79162
3.388554	3.424035	3.371903	35.45433
3.349208	3.425518	3.325301	35.26961
3.281404	3.425383	3.380906	35.24264
3.192895	3.415813	3.335395	34.92864
3.294437	3.43347	3.412622	35.35819
3.318317	3.44122	3.328967	35.24442
3.337825	3.309601	3.404284	35.16395
3.202711	3.199479	3.453324	34.73491
3.247419	3.199277	3.398384	34.71209
3.30813	3.349363	3.369021	35.10885
3.436025	3.42882	3.41107	35.65426
3.258349	3.384071	3.430697	35.21076

3.176109	3.350778	3.440416	34.97937
3.203619	3.310679	3.39453	34.85149
3.357993	3.415341	3.332752	35.28287
3.151982	3.356641	3.425138	34.90602
3.239006	3.383329	3.428719	35.16252
3.134866	3.293089	3.51991	34.93686
3.388265	3.265458	3.284445	34.91566
3.482507	3.209656	3.400004	35.25242
3.387523	3.286215	3.374836	35.1571
3.208156	3.325101	3.45225	35.01918
3.249151	3.410422	3.354442	35.08152
3.311718	3.421811	3.351987	35.23788
3.469226	3.466358	3.304805	35.57657
3.184523	3.396201	3.399987	35.00869
3.140929	3.442501	3.464459	35.1556
3.227251	3.419317	3.418419	35.19299
3.350981	3.352935	3.390523	35.2574
3.324792	3.252585	3.389108	34.97758
3.234098	3.169759	3.412281	34.64879
3.227994	3.318699	3.399083	34.93229
3.325988	3.428416	3.363292	35.30825
3.394493	3.456519	3.367384	35.52847
3.239212	3.363785	3.406739	35.07216
3.17945	3.31607	3.352055	34.71754
3.246677	3.345724	3.448754	35.14087
3.37614	3.447555	3.332053	35.39147
3.31576	3.349093	3.418965	35.23417
3.215497	3.317418	3.377888	34.85581
3.155158	3.193077	3.440245	34.58831
3.330483	3.244094	3.373591	34.93752
3.350528	3.292685	3.41194	35.17148
3.381543	3.338445	3.405256	35.32476
3.229602	3.354754	3.354902	34.91804
3.327431	3.388249	3.415265	35.33723
3.306521	3.427742	3.326733	35.18426
3.34364	3.437177	3.388613	35.42139
3.159983	3.447084	3.358261	34.97505
3.175573	3.370727	3.452181	35.04755
3.166788	3.353339	3.368083	34.80641
3.306109	3.247666	3.372176	34.88894
3.352219	3.212891	3.412059	35.00094
3.322029	3.286215	3.415862	35.10359
3.247625	3.421811	3.429332	35.26686
3.305201	3.424911	3.400055	35.33553
3.420188	3.356844	3.347229	35.32261
3.372304	3.344241	3.358568	35.21513
3.113337	3.369918	3.399117	34.79364
3.248904	3.430505	3.382816	35.18695
3.266804	3.363246	3.451687	35.22962
3.183492	3.29255	3.382441	34.7414

3.293529	3.227111	3.421762	34.92491
3.240037	3.219091	3.418419	34.78309
3.254431	3.224145	3.457416	34.9109
3.319966	3.323147	3.364315	35.06712
3.325947	3.397752	3.456785	35.44556
3.259669	3.420868	3.396457	35.21924
3.35325	3.410826	3.41593	35.44452
3.330483	3.391282	3.326614	35.15667
3.388513	3.401054	3.317355	35.28469
3.187286	3.435357	3.396576	35.0929
3.26569	3.36055	3.411087	35.1325
3.163819	3.286889	3.396372	34.71646
3.233067	3.193683	3.35613	34.57607
3.260452	3.227515	3.443383	34.90075
3.366324	3.393236	3.376609	35.34865
3.272825	3.387305	3.466368	35.32751
3.408392	3.398897	3.305743	35.29806
3.333989	3.365335	3.430151	35.33401
3.367809	3.376118	3.320629	35.19205
3.230881	3.420059	3.434857	35.2385
3.138454	3.426596	3.351492	34.86836
3.18275	3.31324	3.413918	34.85385
3.153178	3.259864	3.361331	34.55746
3.249894	3.303805	3.429537	35.01421
3.274516	3.20723	3.415606	34.8264
3.261854	3.279273	3.415469	34.95596
3.308624	3.323484	3.41801	35.16047
3.412682	3.395662	3.389756	35.48408
3.307965	3.413926	3.346462	35.20035
3.360055	3.430572	3.354374	35.36797
3.267422	3.403008	3.3607	35.11895
3.293653	3.425989	3.353964	35.21184
3.228117	3.405165	3.385527	35.092
3.3297	3.372075	3.424217	35.3264
3.148724	3.242342	3.436	34.67269
3.263587	3.237354	3.346377	34.71698
3.261978	3.258449	3.427269	34.93649
3.37746	3.445264	3.410098	35.56002
3.280455	3.407995	3.455711	35.36613
3.351146	3.372479	3.38007	35.27764
3.244532	3.311757	3.419766	34.99851
3.339804	3.381981	3.3275	35.15865
3.206506	3.473839	3.443298	35.32126
3.271175	3.420531	3.41622	35.28689
3.09919	3.235602	3.360291	34.38407
3.19834	3.246385	3.374614	34.6558
3.24313	3.273478	3.405869	34.88134
3.340753	3.299559	3.408939	35.15858
3.270268	3.348824	3.413901	35.12302
3.300541	3.350374	3.404028	35.17102

3.333618	3.355967	3.429742	35.31182
3.432272	3.41433	3.300457	35.37247
3.316131	3.429292	3.358381	35.27787
3.191576	3.379083	3.397412	34.98105
3.259421	3.401054	3.410303	35.20565
3.205846	3.359741	3.367862	34.90534
3.260411	3.320721	3.380292	34.96651
3.251007	3.26539	3.381094	34.8267
3.169799	3.326719	3.397531	34.81917
3.230674	3.34451	3.442155	35.08879
3.474959	3.405771	3.373267	35.60632
3.318605	3.399975	3.392348	35.29346
3.299056	3.358663	3.395434	35.16711
3.268577	3.357922	3.361928	35.02556
3.328751	3.388316	3.427491	35.367
3.28726	3.406243	3.378178	35.20763
3.290601	3.305692	3.407421	35.05899
3.0836	3.26539	3.419766	34.54518
3.209145	3.239242	3.403056	34.726
3.197638	3.252316	3.38771	34.69587
3.279383	3.309129	3.423808	35.07781
3.279589	3.397886	3.417584	35.25875
3.365994	3.356439	3.424814	35.37288
3.244656	3.407928	3.402988	35.1724
3.377047	3.456721	3.376285	35.51023
3.333659	3.417228	3.421284	35.42738
3.267381	3.343028	3.322709	34.90461
3.21228	3.248677	3.412963	34.77515
3.22391	3.214845	3.381929	34.65874
3.294973	3.304276	3.398196	35.04529
3.302108	3.37942	3.396389	35.22126
3.204485	3.375983	3.430679	35.07525
3.326607	3.435088	3.420653	35.44964
3.306727	3.384812	3.490906	35.44985
3.425302	3.421811	3.374034	35.53449
3.320338	3.440749	3.445702	35.50308
3.224405	3.382925	3.454432	35.18594
3.250265	3.362774	3.410695	35.10278
3.199164	3.277656	3.394019	34.76842
3.288786	3.190112	3.351645	34.6803
3.234716	3.187483	3.361365	34.57756
3.18576	3.258449	3.401436	34.71332
3.138867	3.328269	3.422631	34.80981
3.36686	3.457867	3.379133	35.49668
3.244986	3.362909	3.429315	35.13225
3.38047	3.328471	3.355312	35.19138
3.239624	3.399841	3.460554	35.2696
3.356591	3.436368	3.345694	35.35408
3.334814	3.414667	3.361552	35.29369
3.392142	3.306972	3.287242	35.02104

3.143073	3.248205	3.441524	34.68524
3.232489	3.299626	3.302042	34.6882
3.155653	3.28325	3.430407	34.76507
3.315182	3.400515	3.394121	35.29103
3.231376	3.382655	3.475372	35.24638
3.304088	3.371198	3.447612	35.31963
3.274475	3.342084	3.378723	35.04056
3.38542	3.442231	3.415418	35.58243
3.298231	3.436031	3.398571	35.34136
3.287879	3.427203	3.466692	35.44838
3.213187	3.370659	3.397548	35.01019
3.178254	3.180744	3.372721	34.46418
3.335185	3.22118	3.330297	34.80302
3.227664	3.262088	3.40899	34.82944
3.239336	3.329684	3.427013	35.0422
3.225684	3.334401	3.439751	35.05051
3.336629	3.366279	3.316775	35.09391
3.407609	3.357989	3.363616	35.33344
3.34265	3.405232	3.357017	35.28027
3.220364	3.527821	3.473939	35.53662
3.3118	3.456856	3.384708	35.38626
3.276661	3.302929	3.335498	34.86518
3.370201	3.258314	3.344995	34.99294
3.180151	3.234389	3.410303	34.66783
3.168768	3.299289	3.408615	34.78117
3.204567	3.363448	3.443434	35.07591
3.304253	3.404828	3.382628	35.25142
3.184152	3.369918	3.362405	34.86822
3.316956	3.336962	3.418624	35.20951
3.239624	3.367357	3.409842	35.08766
3.35127	3.449308	3.407506	35.50592
3.368221	3.426394	3.354203	35.37631
3.359931	3.34815	3.37059	35.22291
3.234593	3.265727	3.430986	34.90065
3.231458	3.198131	3.349224	34.56717
3.149837	3.226571	3.395911	34.55297
3.307676	3.374366	3.339573	35.09814
3.185183	3.327056	3.444611	34.95651
3.228983	3.388856	3.410695	35.11327
3.274887	3.357854	3.431788	35.19199
3.430416	3.314385	3.356539	35.27249
3.319018	3.424979	3.424814	35.42003
3.331143	3.435559	3.399066	35.41338
3.283631	3.336962	3.430424	35.16244
3.252451	3.254338	3.307908	34.64564
3.352879	3.281497	3.363343	35.04588
3.249564	3.363583	3.385271	35.04741
3.301737	3.391888	3.415231	35.28892
3.186544	3.388182	3.42229	35.04435
3.307635	3.361494	3.371425	35.13956

3.253977	3.3466	3.3511	34.9452
3.300293	3.4301	3.414992	35.36881
3.193019	3.422485	3.452096	35.1987
3.406124	3.407861	3.381878	35.47919
3.267464	3.330425	3.453477	35.1632
3.379976	3.277656	3.372994	35.11785
3.293777	3.201771	3.449385	34.93045
3.187905	3.183035	3.332343	34.402
3.192772	3.253057	3.430543	34.78052
3.282105	3.374501	3.36706	35.10263
3.235335	3.408063	3.42686	35.20451
3.274269	3.449712	3.389619	35.2993
3.183286	3.35563	3.484375	35.10181
3.357457	3.388721	3.381724	35.33057
3.412063	3.477276	3.417379	35.72162
3.444027	3.445871	3.370146	35.61955
3.310604	3.258651	3.387727	34.9568
3.246388	3.177172	3.344637	34.54396
3.27996	3.248744	3.325096	34.73116
3.343723	3.35462	3.33178	35.11675
3.20201	3.439603	3.390983	35.12216
3.128803	3.350845	3.470393	34.94162
3.270268	3.354687	3.405392	35.11724
3.289776	3.402132	3.439154	35.33748
3.339186	3.478826	3.372994	35.46857
3.237273	3.41743	3.494469	35.37709
3.248244	3.372614	3.447458	35.20027
3.228159	3.255214	3.425291	34.85113
3.428767	3.256157	3.356062	35.1405
3.236655	3.189842	3.385356	34.63943
3.163035	3.191055	3.351185	34.40636
3.172892	3.283115	3.39216	34.71884
3.369623	3.432324	3.398759	35.48979
3.257854	3.474109	3.364946	35.26279
3.32772	3.339456	3.405597	35.21001
3.197721	3.3466	3.435965	35.00776
3.318729	3.369109	3.420039	35.28678
3.347393	3.457462	3.392365	35.48216
3.486095	3.379083	3.396252	35.62258
3.292498	3.257505	3.359421	34.8528
3.224447	3.206421	3.343393	34.55722
3.272908	3.308185	3.393712	34.99577
3.335639	3.413319	3.400191	35.37704
3.2753	3.410287	3.411121	35.26236
3.138949	3.30704	3.438114	34.79742
3.11511	3.347408	3.437517	34.83227
3.387111	3.448229	3.431106	35.63355
3.306851	3.439468	3.517455	35.6277
3.253152	3.381847	3.360086	35.04012
3.189142	3.247801	3.49302	34.89771

3.259462	3.311488	3.322232	34.81728
3.400556	3.267614	3.339897	35.06851
3.350734	3.215452	3.35954	34.88845
3.109831	3.235737	3.413236	34.52342
3.213435	3.364324	3.354237	34.90216
3.328256	3.385081	3.365474	35.22322
3.315471	3.41123	3.357716	35.23548
3.280496	3.393034	3.35879	35.12155
3.12538	3.392967	3.483608	35.05515
3.279012	3.477411	3.435522	35.47062
3.328298	3.35745	3.338328	35.10352
3.467948	3.270445	3.368441	35.2845
3.24746	3.210464	3.368765	34.67187
3.299427	3.29592	3.374273	34.98444
3.298437	3.418509	3.384998	35.27381
3.394864	3.425989	3.29529	35.30486
3.167654	3.302861	3.400873	34.76962
3.224034	3.328134	3.405324	34.95791
3.170088	3.408871	3.504479	35.23334
3.321162	3.453958	3.38684	35.40505
3.374078	3.348621	3.453085	35.43528
3.283177	3.287226	3.422529	35.03542
3.20931	3.274691	3.434635	34.87294
3.350322	3.288237	3.358568	35.04459
3.292251	3.220169	3.381162	34.81815
3.360385	3.286754	3.343853	35.03117
3.121998	3.299694	3.419221	34.70297
3.208609	3.345521	3.374955	34.89579
3.346486	3.380027	3.422512	35.37677
3.258143	3.413117	3.392569	35.19046
3.222673	3.371468	3.464305	35.17869
3.248162	3.411095	3.494265	35.3866
3.321039	3.369379	3.354936	35.15005
3.395277	3.265188	3.364707	35.10592
3.394823	3.257303	3.362627	35.08313
3.187987	3.303468	3.388801	34.78901
3.279672	3.371468	3.326256	35.00144
3.353332	3.41797	3.408188	35.44339
3.354693	3.379083	3.365082	35.26706
3.269155	3.333391	3.361126	34.97143
3.227169	3.418172	3.411599	35.17539
3.153508	3.516836	3.480487	35.38072
3.307305	3.463123	3.443997	35.51979
3.206217	3.270647	3.445838	34.88183
3.3264	3.24807	3.38464	34.96145
3.235747	3.287158	3.451158	34.99415
3.355312	3.294504	3.385459	35.12801
3.260328	3.247666	3.403755	34.85788
3.255049	3.237152	3.320663	34.64164
3.169304	3.339119	3.412008	34.87687

3.311511	3.420126	3.353334	35.23669
3.149672	3.384408	3.429793	34.97187
3.220611	3.409882	3.410507	35.14053
3.282889	3.391821	3.46601	35.3586
3.35725	3.477478	3.412008	35.59045
3.283177	3.299761	3.382833	34.97602
3.262226	3.241735	3.376541	34.78955
3.316543	3.272669	3.348013	34.91359
3.30813	3.413185	3.360222	35.22918
3.274351	3.436638	3.37325	35.23509
3.275259	3.363313	3.365355	35.05946
3.299922	3.286889	3.359489	34.93344
3.186626	3.373355	3.382662	34.92544
3.234922	3.521554	3.414463	35.42469
3.243666	3.521284	3.504905	35.641
3.17215	3.329212	3.449368	34.94313
3.176852	3.185596	3.457433	34.65698
3.35259	3.275499	3.419084	35.15403
3.264494	3.369985	3.363974	35.04749
3.334443	3.362033	3.397344	35.25604
3.210713	3.235804	3.423859	34.76741
3.349208	3.292617	3.395229	35.1319
3.287673	3.362707	3.33219	35.01275
3.371521	3.407793	3.379883	35.39901
3.254431	3.40503	3.394479	35.16883
3.217353	3.377062	3.415487	35.07252
3.241769	3.396471	3.458184	35.26174
3.388348	3.406176	3.357989	35.38439
3.39903	3.265323	3.394411	35.17938
3.28128	3.271523	3.426928	35.00655
3.279878	3.261279	3.3954	34.91213
3.372346	3.405097	3.365696	35.36389
3.330442	3.372075	3.324977	35.111
3.245192	3.359202	3.37961	35.01589
3.105047	3.344241	3.406193	34.73483
3.208485	3.46218	3.357358	35.11215
3.270557	3.532741	3.416765	35.53212
3.29122	3.374972	3.398674	35.19272
3.19603	3.224684	3.529851	34.94277
3.114121	3.203253	3.365406	34.35716
3.418456	3.321125	3.451551	35.46885
3.337825	3.393304	3.334031	35.19337
3.273856	3.257101	3.397446	34.8943
3.230798	3.240252	3.40662	34.78336
3.299097	3.381914	3.422989	35.2783
3.311553	3.410085	3.35879	35.22675
3.413259	3.389058	3.361876	35.40994
3.135938	3.359068	3.458252	34.94865
3.176357	3.407658	3.42495	35.07048
3.288621	3.451599	3.372295	35.29693

3.366035	3.261751	3.341074	34.98277
3.193885	3.247396	3.369038	34.63607
3.24746	3.241466	3.419289	34.85015
3.184152	3.401391	3.424831	35.07356
3.412063	3.45261	3.33045	35.47758
3.342155	3.364122	3.403806	35.29161
3.360137	3.27004	3.341875	34.98976
3.203784	3.343971	3.415145	34.96974
3.261112	3.524519	3.345439	35.3375
3.178254	3.490822	3.426741	35.26041
3.31048	3.344174	3.379695	35.12599
3.186049	3.181687	3.501171	34.76419
3.311553	3.280689	3.439478	35.12024
3.301531	3.412174	3.466283	35.44448
3.348837	3.353069	3.418488	35.31415
3.302397	3.234861	3.335071	34.77168
3.237645	3.250429	3.428701	34.86887
3.23319	3.346195	3.382867	34.96832
3.266886	3.407052	3.405989	35.22566
3.318729	3.463865	3.333025	35.30371
3.243955	3.393977	3.480828	35.31058
3.23583	3.342556	3.34728	34.88832
3.241398	3.252316	3.386397	34.78869
3.393503	3.286148	3.375432	35.17133
3.246553	3.319103	3.445702	35.07571
3.275877	3.407726	3.390523	35.21297
3.341207	3.436975	3.34549	35.32132
3.438706	3.359607	3.37857	35.43769
3.319348	3.315464	3.304481	34.91811
3.367025	3.367829	3.357358	35.25252
3.130329	3.480578	3.40292	35.08111
3.265979	3.480713	3.4397	35.45848
3.1778	3.269906	3.350196	34.60892
3.245852	3.166726	3.393251	34.62625
3.223168	3.266334	3.422938	34.85939
3.295633	3.408737	3.469847	35.43186
3.213558	3.418711	3.481851	35.30044
3.373459	3.314183	3.371681	35.1806
3.324998	3.287428	3.445293	35.17709
3.358364	3.380364	3.410695	35.37764
3.267629	3.382251	3.442752	35.25344
3.211331	3.387845	3.409553	35.06996
3.268123	3.407995	3.416578	35.25358
3.328009	3.392764	3.363786	35.23579
3.233396	3.295448	3.411803	34.92108
3.288539	3.290663	3.340306	34.87485
3.286848	3.315262	3.494248	35.26159
3.347888	3.394584	3.361859	35.27903
3.352095	3.360685	3.421352	35.3442
3.331844	3.419654	3.318685	35.20435

3.314893	3.361628	3.423722	35.27009
3.271918	3.3683	3.363991	35.06008
3.365045	3.421339	3.404761	35.46888
3.281445	3.460091	3.446367	35.46179
3.230179	3.335345	3.397412	34.96982
3.128185	3.168478	3.425496	34.44328
3.291715	3.23722	3.335054	34.75344
3.162582	3.260066	3.442957	34.75697
3.326235	3.402402	3.387045	35.30385
3.207454	3.264582	3.471126	34.92658
3.410991	3.280891	3.337186	35.11444
3.39738	3.319305	3.389159	35.28234
3.379934	3.451936	3.377888	35.50958
3.267134	3.405434	3.433544	35.28292
3.215291	3.368031	3.381997	34.97503
3.149549	3.343904	3.444747	34.91572
3.367932	3.439401	3.304379	35.29517
3.256575	3.381577	3.470017	35.28742
3.198422	3.256899	3.351373	34.62814
3.280909	3.288574	3.456632	35.10798
3.32574	3.352935	3.350486	35.11464
3.365953	3.397212	3.410166	35.42992
3.318193	3.416083	3.386516	35.31502
3.299592	3.336625	3.375978	35.07754
3.305944	3.34168	3.344688	35.03406
3.35358	3.449106	3.34196	35.36719
3.289652	3.447488	3.408666	35.36973
3.270763	3.304613	3.375586	34.94363
3.17714	3.15756	3.428514	34.53306
3.299633	3.227111	3.342011	34.76386
3.260617	3.339928	3.396645	35.04473
3.288209	3.408063	3.412213	35.28811
3.206671	3.304816	3.440382	34.94562
3.273361	3.258584	3.392296	34.8852
3.347558	3.29383	3.323255	34.97355
3.43586	3.357113	3.359813	35.38499
3.198463	3.406647	3.43854	35.14633
3.196154	3.460225	3.404812	35.18469
3.218219	3.336828	3.349974	34.84317
3.34496	3.315262	3.340221	35.05184
3.182543	3.260201	3.355789	34.6103
3.201062	3.283182	3.454006	34.91583
3.126246	3.40166	3.475031	35.0573
3.413795	3.433672	3.381042	35.55059
3.378202	3.386362	3.364997	35.3342
3.41429	3.353676	3.303543	35.20725
3.319265	3.351654	3.461117	35.33961
3.23983	3.441153	3.326102	35.06637
3.370655	3.467976	3.389551	35.54987
3.342114	3.394651	3.350742	35.24224

3.237026	3.199075	3.379405	34.64741
3.143568	3.235063	3.452574	34.68174
3.268453	3.370524	3.434107	35.2107
3.28458	3.453082	3.470307	35.50567
3.261442	3.3497	3.407643	35.09195
3.247749	3.19921	3.485193	34.9025
3.271423	3.272804	3.39378	34.9153
3.34628	3.29592	3.378723	35.09663
3.397545	3.460293	3.420721	35.66004
3.205846	3.413387	3.426331	35.15051
3.192648	3.388721	3.411121	35.03445
3.204196	3.219967	3.358739	34.57611
3.380429	3.327932	3.335464	35.14671
3.16716	3.318092	3.403943	34.80856
3.237645	3.416285	3.424814	35.22307
3.232654	3.44924	3.480197	35.40534
3.369294	3.383127	3.323408	35.2167
3.451533	3.375309	3.435266	35.62406
3.444315	3.401391	3.409331	35.6086
3.294684	3.39883	3.404164	35.26448
3.336752	3.42107	3.393183	35.3811
3.259833	3.344982	3.357852	34.96923
3.338897	3.206488	3.333127	34.7852
3.234634	3.207432	3.35272	34.6021
3.07065	3.295852	3.484562	34.72517
3.27497	3.437514	3.402783	35.30294
3.325328	3.389462	3.401027	35.30415
3.290106	3.265862	3.42258	35.00396
3.262267	3.283721	3.404369	34.94231
3.286023	3.378746	3.452557	35.30745
3.283878	3.413926	3.301002	35.04826
3.380718	3.389934	3.347775	35.30985
3.233809	3.36055	3.402118	35.04317
3.250677	3.286215	3.389227	34.88931
3.252781	3.254068	3.35983	34.75932
3.289034	3.277993	3.32276	34.80985
3.244161	3.32746	3.408461	35.00732
3.237727	3.386092	3.468415	35.25257
3.184193	3.420733	3.436426	35.1413
3.37614	3.359	3.32953	35.19229
3.332051	3.366076	3.405034	35.27647
3.448935	3.45598	3.480351	35.89338
3.320379	3.405232	3.400396	35.32643
3.340547	3.380566	3.446998	35.4185
3.272413	3.316879	3.356471	34.93226
3.347187	3.160189	3.346718	34.7318
3.251874	3.195301	3.350333	34.60805
3.209228	3.352733	3.416987	35.00483
3.138784	3.43684	3.480675	35.17399
3.328463	3.405771	3.353555	35.24285

3.311388	3.275904	3.48047	35.19906
3.381213	3.237489	3.357187	34.99815
3.266597	3.272736	3.486012	35.1063
3.335969	3.459821	3.383958	35.44395
3.301448	3.465617	3.42877	35.47913
3.367396	3.393101	3.324005	35.23567
3.201268	3.249283	3.360035	34.63665
3.187451	3.227111	3.367725	34.57477
3.25905	3.295987	3.416083	34.98772
3.184028	3.37841	3.441951	35.06047
3.287343	3.461641	3.392075	35.35934
3.301902	3.383127	3.396048	35.22817
3.26103	3.359202	3.444866	35.19323
3.301943	3.415948	3.394939	35.29761
3.451987	3.446275	3.380309	35.66006
3.291962	3.410556	3.432794	35.34678
3.293199	3.318766	3.362286	34.99456
3.268577	3.170433	3.424132	34.75158
3.31444	3.178115	3.309426	34.61784
3.189843	3.410758	3.370794	34.98832
3.261442	3.491361	3.426587	35.44317
3.167201	3.391753	3.464101	35.10129
3.265278	3.383127	3.414327	35.18806
3.234015	3.214912	3.42362	34.77215
3.42291	3.308185	3.394257	35.325
3.266845	3.445938	3.484648	35.48262
3.309078	3.42545	3.371152	35.28198
3.258266	3.308253	3.41223	35.0044
3.354033	3.19402	3.31154	34.74383
3.268412	3.185394	3.329274	34.5765
3.201309	3.289922	3.373898	34.75593
3.153384	3.432257	3.482448	35.19977
3.240284	3.517645	3.452659	35.51139
3.337371	3.444994	3.391324	35.4307
3.32673	3.345387	3.4013	35.21142
3.264288	3.365807	3.426178	35.17393
3.30582	3.463932	3.444866	35.52021
3.387812	3.459484	3.4013	35.59451
3.405052	3.371603	3.330212	35.28457
3.217353	3.220102	3.382134	34.65634
3.191823	3.166861	3.44398	34.61933
3.322565	3.257977	3.370999	34.9449
3.247749	3.442636	3.401709	35.25227
3.324421	3.428685	3.386107	35.35531
3.124844	3.347476	3.494793	34.97895
3.247502	3.297874	3.392552	34.91513
3.305449	3.32874	3.434772	35.20168
3.377625	3.405771	3.370794	35.38806
3.231788	3.420328	3.398742	35.16209
3.389709	3.311488	3.406824	35.2871

3.275877	3.297874	3.392825	34.97778
3.40963	3.222865	3.324619	34.95709
3.209805	3.187483	3.352515	34.50373
3.231128	3.335951	3.435539	35.0566
3.141547	3.373288	3.405716	34.87713
3.243295	3.453419	3.458013	35.38923
3.369211	3.461708	3.360291	35.46902
3.417425	3.381307	3.382748	35.44774
3.281816	3.412578	3.401027	35.25954
3.278146	3.445668	3.381776	35.28178
3.309656	3.424911	3.424081	35.39781
3.342114	3.313779	3.334781	35.03048
3.2501	3.163154	3.350998	34.53533
3.20135	3.148732	3.348235	34.39114
3.244986	3.398897	3.415623	35.181
3.233108	3.489609	3.439546	35.40572
3.217559	3.357046	3.382134	34.95627
3.280826	3.302322	3.413423	35.04338
3.239336	3.238163	3.478952	34.95564
3.336876	3.326314	3.414327	35.22039
3.411032	3.449173	3.400532	35.62106
3.269319	3.418711	3.373813	35.18611
3.381749	3.349026	3.385476	35.30509
3.299303	3.220776	3.399782	34.87562
3.334649	3.141184	3.329257	34.62463
3.258225	3.250698	3.315905	34.6678
3.299345	3.392832	3.43409	35.327
3.113378	3.435694	3.426058	34.99649
3.327968	3.401593	3.355772	35.23748
3.268742	3.351182	3.357716	35.00198
3.313161	3.387238	3.394803	35.25906
3.217064	3.48105	3.399168	35.26361
3.376222	3.458338	3.454432	35.68285
3.238346	3.335615	3.399987	34.9939
3.342155	3.193414	3.328848	34.75437
3.21958	3.17205	3.381861	34.55553
3.3297	3.297267	3.346121	34.99202
3.178584	3.430437	3.452795	35.18606
3.255297	3.468111	3.376319	35.26896
3.241686	3.367492	3.452523	35.1858
3.282022	3.310611	3.336913	34.8968
3.275918	3.296189	3.431447	35.05865
3.301737	3.413724	3.40824	35.32138
3.270186	3.440749	3.465072	35.43577
3.371521	3.408467	3.376097	35.39221
3.413053	3.199884	3.326955	34.91942
3.306645	3.162008	3.393729	34.74992
3.261153	3.268423	3.403209	34.90389
3.162499	3.346734	3.433885	34.92648
3.292622	3.466897	3.390847	35.3797

3.343021	3.445466	3.399373	35.46169
3.22919	3.40166	3.437364	35.20004
3.196484	3.337973	3.448584	35.01378
3.354487	3.475187	3.389364	35.52987
3.258885	3.457597	3.428224	35.36732
3.374325	3.352396	3.329018	35.17276
3.247131	3.206825	3.486421	34.92049
3.302355	3.17077	3.338021	34.63788
3.288993	3.248272	3.375228	34.85951
3.405258	3.456586	3.376831	35.57282
3.179244	3.435694	3.426178	35.14079
3.246636	3.37632	3.354629	35.00186
3.134412	3.328066	3.431788	34.81965
3.369788	3.390675	3.394615	35.39001
3.290849	3.331099	3.415077	35.13184
3.210424	3.408871	3.525213	35.36689
3.234922	3.384879	3.430833	35.1616
3.376759	3.296796	3.3607	35.12578
3.418538	3.194559	3.305095	34.87197
3.306892	3.190381	3.39864	34.82325
3.178337	3.289854	3.38846	34.73739
3.162252	3.39081	3.383344	34.9118
3.362612	3.472761	3.281359	35.30615
3.345785	3.414667	3.394053	35.38875
3.234015	3.395393	3.369021	35.04744
3.21826	3.376657	3.428378	35.10181
3.303758	3.440075	3.41269	35.39316
3.365829	3.424237	3.389705	35.444
3.297654	3.273747	3.435266	35.06545
3.168026	3.132962	3.397668	34.39188
3.279053	3.217002	3.432248	34.89408
3.256988	3.390743	3.357852	35.06308
3.40435	3.440412	3.454313	35.7049
3.187204	3.406108	3.367674	34.96556
3.121462	3.312296	3.455455	34.8086
3.25043	3.352126	3.369481	34.98972
3.330525	3.343634	3.343051	35.08851
3.198092	3.358528	3.42686	35.01475
3.28759	3.403345	3.416885	35.28666
3.251502	3.289315	3.436409	35.00107
3.421136	3.257573	3.33901	35.08962
3.507748	3.229537	3.35908	35.26161
3.348919	3.250631	3.413014	35.07835
3.204485	3.405232	3.452386	35.18668
3.2534	3.476939	3.307516	35.13365
3.309821	3.411769	3.403687	35.32483
3.40996	3.39701	3.301565	35.28822
3.195618	3.400582	3.503882	35.26973
3.163489	3.467504	3.365679	35.04359
3.254513	3.418913	3.513362	35.45935

3.38942	3.312229	3.331235	35.12278
3.305242	3.140308	3.404898	34.72383
3.219539	3.212554	3.388409	34.65834
3.25839	3.343904	3.439001	35.14118
3.291261	3.4671	3.366156	35.32317
3.398123	3.400852	3.334372	35.34248
3.255627	3.311016	3.44236	35.07056
3.191906	3.355967	3.39528	34.92656
3.199206	3.356439	3.443519	35.04904
3.3078	3.375377	3.380667	35.19049
3.211372	3.350306	3.427337	35.02685
3.314357	3.318025	3.388545	35.09664
3.222467	3.221921	3.465072	34.85288
3.421054	3.282508	3.331832	35.12827
3.283054	3.265592	3.406449	34.95267
3.38575	3.415678	3.369174	35.42395
3.233809	3.415543	3.432538	35.22995
3.317162	3.401997	3.335037	35.16939
3.304541	3.377331	3.362678	35.1483
3.288993	3.409343	3.342557	35.1403
3.151528	3.465482	3.361041	35.00287
3.260287	3.423631	3.444406	35.3315
3.137341	3.32254	3.422768	34.79424
3.360385	3.157763	3.395877	34.86286
3.366448	3.192201	3.412383	34.98752
3.228571	3.323147	3.444542	35.04269
3.304046	3.473772	3.394308	35.42729
3.24845	3.408602	3.457348	35.30105
3.350651	3.353474	3.352652	35.17503
3.351023	3.386092	3.338737	35.21675
3.198587	3.423698	3.434942	35.17602
3.140186	3.334401	3.450613	34.8873
3.252451	3.354889	3.434925	35.1433
3.197226	3.329616	3.430679	34.95798
3.345249	3.260673	3.353623	34.9624
3.296581	3.184248	3.402255	34.7952
3.285693	3.253125	3.421676	34.96448
3.290972	3.325034	3.365764	35.01101
3.33964	3.347206	3.36145	35.15649
3.30516	3.462449	3.354834	35.31864
3.314893	3.412713	3.434806	35.40604
3.282229	3.423563	3.346377	35.16496
3.329906	3.460967	3.378041	35.42026
3.210836	3.461034	3.349002	35.09652
3.323348	3.38124	3.404761	35.29
3.180399	3.25117	3.434005	34.7569
3.28392	3.160323	3.376814	34.65955
3.325328	3.224887	3.398367	34.93843
3.463865	3.438794	3.377751	35.66408
3.261854	3.371603	3.489456	35.31966

3.303552	3.375714	3.363718	35.14487
3.262597	3.320249	3.355329	34.91567
3.321039	3.444321	3.399867	35.4122
3.164767	3.398291	3.389074	34.94619
3.134206	3.345387	3.4242	34.84048
3.130989	3.321125	3.45005	34.83692
3.325782	3.317755	3.36955	35.07949
3.347847	3.231896	3.390182	34.9851
3.311058	3.281902	3.338737	34.9015
3.258019	3.294437	3.46185	35.08216
3.273939	3.375849	3.396218	35.15148
3.403113	3.425855	3.419101	35.59336
3.427529	3.440681	3.278187	35.37102
3.308996	3.33703	3.386073	35.12107
3.247832	3.398965	3.378519	35.10623
3.289116	3.454969	3.409484	35.3867
3.259091	3.43529	3.384367	35.22308
3.276867	3.24962	3.349991	34.78075
3.155034	3.168411	3.436835	34.52664
3.232489	3.239915	3.413457	34.80127
3.274764	3.371131	3.40928	35.17153
3.424725	3.497764	3.417942	35.79534
3.253977	3.387036	3.430304	35.20683
3.315141	3.332514	3.394274	35.14257
3.261153	3.359944	3.430424	35.16354
3.335639	3.457125	3.361399	35.388
3.133257	3.357315	3.416493	34.84764
3.207001	3.327932	3.372721	34.84893
3.149755	3.321462	3.502705	34.99384
3.282105	3.276982	3.357324	34.86808
3.387853	3.241735	3.446145	35.21649
3.366489	3.31169	3.401231	35.22453
3.266102	3.364392	3.498903	35.33384
3.273073	3.45753	3.363138	35.25587
3.432355	3.418509	3.395553	35.58975
3.400803	3.459552	3.31391	35.43196
3.227086	3.413724	3.344603	35.01897
3.113832	3.399571	3.453682	34.97889
3.296581	3.41433	3.347212	35.17798
3.259421	3.366144	3.39719	35.10063
3.267464	3.24962	3.422563	34.91889
3.175697	3.177105	3.470648	34.66479
3.238593	3.261481	3.408939	34.8519
3.275135	3.381038	3.427491	35.23383
3.487002	3.415746	3.395144	35.70231
3.296375	3.341073	3.415401	35.16645
3.298685	3.366413	3.366429	35.11982
3.26404	3.410354	3.425649	35.26965
3.315471	3.440749	3.348968	35.2809
3.179285	3.361696	3.40355	34.92957

3.231376	3.300907	3.332138	34.75438
3.218879	3.287226	3.403482	34.85315
3.298561	3.238568	3.386891	34.88472
3.32141	3.308859	3.440024	35.20459
3.380429	3.291741	3.381298	35.1678
3.25575	3.308657	3.388937	34.94884
3.275836	3.408871	3.366702	35.1633
3.362571	3.429292	3.365355	35.39468
3.406825	3.407658	3.360444	35.43341
3.244079	3.420868	3.325744	35.03051
3.132969	3.438794	3.38921	34.96553
3.161592	3.384003	3.407404	34.94809
3.224818	3.331436	3.388733	34.93057
3.292292	3.24962	3.424967	34.97844
3.212857	3.242207	3.417106	34.77133
3.254059	3.375444	3.422989	35.16567
3.238181	3.398965	3.399458	35.13092
3.379769	3.381375	3.388579	35.37829
3.345042	3.351924	3.420738	35.30827
3.237108	3.326314	3.388937	34.94669
3.265236	3.39263	3.445565	35.27706
3.318234	3.475726	3.418897	35.51636
3.159612	3.325438	3.417652	34.8381
3.250554	3.310544	3.333025	34.81934
3.161922	3.265458	3.484426	34.858
3.263958	3.255753	3.408393	34.89365
3.195164	3.346398	3.419306	34.9653
3.303758	3.324832	3.434039	35.18783
3.31015	3.355361	3.386431	35.16446
3.440603	3.410017	3.365287	35.52303
3.316461	3.382655	3.370931	35.20405
3.381584	3.42855	3.37504	35.45582
3.22057	3.380027	3.38464	35.01859
3.209187	3.366009	3.351219	34.88995
3.151487	3.275499	3.358449	34.58165
3.227292	3.276038	3.448021	34.94449
3.261896	3.290124	3.404505	34.9558
3.272536	3.35462	3.429673	35.17515
3.275052	3.437379	3.450869	35.40798
3.331556	3.400312	3.416049	35.37434
3.260658	3.365335	3.410371	35.1304
3.367025	3.383262	3.318259	35.20077
3.235706	3.371603	3.383089	35.02987
3.186915	3.441355	3.445429	35.21204
3.239129	3.385149	3.372022	35.04278
3.192565	3.251372	3.3777	34.66082
3.213146	3.190516	3.342148	34.495
3.267092	3.221652	3.380156	34.76418
3.257441	3.297133	3.468619	35.10159
3.19405	3.350509	3.415555	34.96365

3.403031	3.378544	3.403261	35.45508
3.306604	3.421811	3.383003	35.29453
3.427364	3.477478	3.360188	35.63045
3.330896	3.407119	3.381025	35.31119
3.328504	3.391551	3.381298	35.27252
3.242429	3.358596	3.336589	34.91444
3.305655	3.304883	3.362968	34.99293
3.153797	3.29538	3.417601	34.75954
3.226344	3.245644	3.401965	34.77523
3.187039	3.245307	3.430355	34.75062
3.330483	3.415274	3.375296	35.3156
3.293653	3.396336	3.475031	35.41174
3.328751	3.405704	3.451022	35.45648
3.245852	3.339254	3.458013	35.14517
3.360055	3.434211	3.367589	35.40482
3.265608	3.376792	3.462464	35.28019
3.294849	3.40503	3.382577	35.23119
3.203289	3.28116	3.40783	34.8153
3.207083	3.221921	3.37134	34.61426
3.286477	3.299155	3.360836	34.93381
3.276083	3.253596	3.411514	34.92227
3.296045	3.282441	3.445429	35.10317
3.143238	3.346937	3.44664	34.9127
3.311058	3.337973	3.389074	35.1342
3.463287	3.414061	3.3644	35.57954
3.439655	3.420733	3.361808	35.53678
3.273444	3.417026	3.442411	35.34146
3.27662	3.417565	3.399577	35.25592
3.217518	3.305759	3.331337	34.73294
3.306727	3.261616	3.293534	34.74882
3.217559	3.25737	3.403943	34.78598
3.176563	3.278465	3.409058	34.75365
3.160478	3.30704	3.414549	34.79297
3.313161	3.44304	3.358517	35.30174
3.232118	3.390945	3.425018	35.15602
3.349414	3.342017	3.363837	35.17174
3.174088	3.363246	3.496976	35.1259
3.338773	3.412578	3.384384	35.34771
3.342568	3.402402	3.391137	35.34852
3.341083	3.351452	3.369635	35.18683
3.134	3.185057	3.430577	34.50336
3.199123	3.217204	3.368816	34.58102
3.142991	3.22091	3.418419	34.57484
3.329782	3.374231	3.394547	35.26641
3.293117	3.412039	3.460315	35.41273
3.289075	3.3621	3.471347	35.31881
3.292333	3.3466	3.419784	35.17928
3.43153	3.419857	3.426877	35.65939
3.386121	3.40321	3.350077	35.35574
3.314564	3.349632	3.406671	35.20585

3.239336	3.330088	3.364451	34.90627
3.208032	3.243083	3.376404	34.67368
3.361127	3.273006	3.311046	34.93099
3.243707	3.273073	3.386175	34.83865
3.240573	3.335345	3.378655	34.95153
3.181512	3.350306	3.426604	34.95995
3.316873	3.447151	3.426928	35.46845
3.355477	3.361628	3.380752	35.26487
3.28458	3.404558	3.440177	35.33366
3.202134	3.471885	3.395502	35.2029
3.381667	3.391147	3.455421	35.54999
3.2355	3.318564	3.405989	34.96351
3.28161	3.236074	3.343512	34.74733
3.209888	3.213901	3.4124	34.69264
3.126823	3.27186	3.421455	34.65754
3.201103	3.271523	3.375654	34.71909
3.31976	3.374164	3.392348	35.23953
3.30219	3.423496	3.412963	35.35408
3.366613	3.389732	3.417038	35.43003
3.226839	3.428281	3.43131	35.23988
3.381584	3.465482	3.34445	35.46969
3.420642	3.413319	3.360291	35.47567
3.414909	3.348082	3.342352	35.28124
3.255379	3.307107	3.371289	34.90605
3.225024	3.258449	3.320117	34.62135
3.256039	3.269906	3.386567	34.85955
3.293488	3.385284	3.359677	35.13495
3.216198	3.375579	3.465055	35.17516
3.153838	3.416959	3.423381	35.03814
3.301902	3.343702	3.437654	35.23294
3.386987	3.410152	3.417311	35.51984
3.306439	3.464404	3.435454	35.50201
3.245646	3.405771	3.42524	35.21851
3.300788	3.318294	3.436	35.17133
3.18873	3.246857	3.393729	34.67761
3.381749	3.156549	3.313996	34.72786
3.222055	3.249688	3.458303	34.89789
3.228695	3.297065	3.419289	34.9307
3.130123	3.305287	3.398844	34.68841
3.345455	3.431381	3.358671	35.3472
3.26866	3.365268	3.404761	35.13548
3.319801	3.407456	3.358449	35.2383
3.190916	3.412174	3.521888	35.32418
3.428395	3.473502	3.344075	35.58877
3.332174	3.366144	3.328421	35.10935
3.437634	3.258247	3.288572	35.01687
3.247048	3.226774	3.411803	34.80075
3.202093	3.26074	3.377018	34.70065
3.273609	3.370794	3.401658	35.1516
3.282682	3.367896	3.353538	35.05987

3.201433	3.366009	3.422682	35.02928
3.207784	3.346465	3.440706	35.03984
3.191287	3.367963	3.463674	35.101
3.376882	3.429898	3.433135	35.57553
3.412599	3.460765	3.430014	35.71431
3.240944	3.352126	3.387846	35.00914
3.256122	3.270243	3.384862	34.85674
3.251007	3.196312	3.443724	34.8126
3.30417	3.222797	3.312376	34.69954
3.293076	3.30367	3.383839	35.00841
3.148229	3.351722	3.400328	34.8328
3.145177	3.375309	3.419221	34.91902
3.305531	3.410354	3.458849	35.43299
3.290147	3.33831	3.375978	35.06057
3.308459	3.38461	3.403261	35.26153
3.225272	3.431381	3.438404	35.25875
3.310852	3.370794	3.4397	35.31624
3.310274	3.310948	3.296944	34.87192
3.435489	3.213228	3.374136	35.10085
3.255338	3.241263	3.353743	34.7236
3.30714	3.348285	3.355107	35.0739
3.152271	3.400717	3.377138	34.89807
3.332133	3.390473	3.385987	35.28835
3.190875	3.359	3.387454	34.91382
3.248698	3.399167	3.397054	35.1491
3.20168	3.438255	3.501784	35.36079
3.302149	3.459956	3.424967	35.45997
3.34463	3.380162	3.403414	35.33124
3.385708	3.307916	3.415896	35.29037
3.252038	3.192066	3.438472	34.79409
3.26136	3.1829	3.372295	34.64971
3.201062	3.244498	3.382662	34.67522
3.218095	3.330425	3.321908	34.76752
3.254802	3.37504	3.363309	35.03589
3.185389	3.384273	3.390864	34.96455
3.232572	3.398965	3.417055	35.15713
3.35824	3.398223	3.364929	35.31634
3.295921	3.43374	3.493225	35.53829
3.284456	3.437918	3.352123	35.21379
3.39437	3.299155	3.445702	35.35534
3.278847	3.300098	3.371698	34.94294
3.385461	3.230009	3.362354	35.00238
3.298809	3.3034	3.385936	35.02495
3.263958	3.409141	3.372585	35.15078
3.277444	3.336828	3.360052	34.99472
3.280496	3.287226	3.370607	34.91601
3.23484	3.311285	3.346445	34.81594
3.316172	3.4733	3.369925	35.39945
3.213517	3.561653	3.479788	35.60842
3.325039	3.499516	3.422716	35.59162

3.250306	3.313914	3.482107	35.15218
3.342114	3.339658	3.414463	35.26132
3.240408	3.256292	3.523849	35.09581
3.303222	3.216799	3.377479	34.82672
3.171036	3.150956	3.428224	34.50464
3.250471	3.291472	3.319094	34.74698
3.215043	3.343769	3.385442	34.92896
3.309656	3.421272	3.405648	35.34954
3.136103	3.270849	3.458098	34.75576
3.196236	3.304546	3.393865	34.82048
3.396225	3.4301	3.478577	35.71765
3.426952	3.44176	3.377069	35.58836
3.382038	3.333593	3.397821	35.29897
3.263545	3.252114	3.407114	34.88199
3.311429	3.365807	3.359216	35.13059
3.383028	3.393708	3.361433	35.35302
3.324091	3.37787	3.332036	35.12522
3.164891	3.234322	3.438677	34.69636
3.231334	3.311353	3.407506	34.94195
3.229726	3.452543	3.39528	35.22046
3.301861	3.422822	3.40205	35.32802
3.264412	3.337367	3.396866	35.04791
3.219827	3.281767	3.507292	35.07031
3.151033	3.262088	3.390097	34.62054
3.461473	3.291269	3.347826	35.2708
3.254018	3.274151	3.36261	34.81202
3.258885	3.238298	3.366173	34.75205
3.215002	3.344645	3.427474	35.02271
3.325411	3.429831	3.351645	35.28462
3.317739	3.477074	3.408649	35.49581
3.290601	3.377331	3.383481	35.1633
3.194545	3.335615	3.475832	35.06398
3.229808	3.399167	3.416629	35.1506
3.276455	3.372277	3.384777	35.12415
3.421013	3.315464	3.41356	35.37898
3.356013	3.227515	3.398145	35.01079
3.316543	3.291135	3.409689	35.08885
3.272165	3.373759	3.381452	35.11074
3.399401	3.449712	3.332445	35.44791
3.340547	3.407389	3.344876	35.25384
3.25307	3.322068	3.413713	35.02649
3.087106	3.340939	3.402391	34.68006
3.278682	3.485363	3.373062	35.3507
3.325328	3.467302	3.476804	35.64008
3.288209	3.285271	3.381741	34.95295
3.296829	3.171039	3.464101	34.9021
3.269814	3.286484	3.362831	34.87402
3.369995	3.250968	3.459275	35.22634
3.320503	3.295583	3.325113	34.92228
3.24115	3.302592	3.399918	34.92767

3.169634	3.386429	3.394684	34.94316
3.345372	3.398358	3.394956	35.35416
3.271959	3.38434	3.429997	35.23959
3.244697	3.419587	3.396713	35.18426
3.226591	3.389732	3.42836	35.14859
3.243089	3.406243	3.462054	35.29446
3.243419	3.299424	3.420466	34.97063
3.426251	3.254068	3.353487	35.1248
3.274764	3.246722	3.347417	34.76418
3.320997	3.388451	3.398776	35.28754
3.35692	3.415476	3.345677	35.30908
3.406825	3.416959	3.388869	35.51591
3.229478	3.263368	3.31582	34.63246
3.283755	3.358326	3.387454	35.11546
3.158375	3.503627	3.432351	35.25721
3.293571	3.517375	3.335736	35.37164
3.184358	3.28918	3.436289	34.85368
3.247914	3.171241	3.363377	34.57531
3.32805	3.24187	3.419494	35.02772
3.29984	3.303468	3.38077	35.01605
3.23187	3.289517	3.492491	35.08122
3.303593	3.310814	3.344143	34.96023
3.278352	3.324292	3.399594	35.05577
3.310728	3.367492	3.304344	35.01274
3.275382	3.369177	3.427082	35.20754
3.217477	3.398021	3.375654	35.03152
3.199082	3.374164	3.382475	34.95404
3.304376	3.393775	3.450323	35.37556
3.301902	3.362639	3.343853	35.06923
3.311223	3.237961	3.386499	34.91022
3.245027	3.236141	3.345422	34.67165
3.336134	3.310881	3.393933	35.14042
3.447037	3.467639	3.338124	35.6037
3.26404	3.370996	3.418061	35.16699
3.314399	3.365942	3.401061	35.22888
3.187451	3.312162	3.361314	34.74674
3.282435	3.44021	3.357682	35.22653
3.26305	3.484487	3.38568	35.3422
3.207124	3.393371	3.416493	35.08802
3.05704	3.219563	3.447663	34.44788
3.29155	3.201299	3.349326	34.70573
3.270021	3.292078	3.467715	35.11607
3.363231	3.38306	3.393916	35.35748
3.227994	3.323416	3.432606	35.01592
3.318688	3.266738	3.367128	34.94712
3.399319	3.312094	3.340238	35.16382
3.414166	3.372344	3.354698	35.35967
3.208238	3.338984	3.336708	34.79705
3.184894	3.355226	3.371681	34.858
3.154539	3.435762	3.390165	35.00815

3.344382	3.423429	3.371954	35.35652
3.403072	3.283384	3.392433	35.22339
3.203825	3.213834	3.447748	34.75654
3.210011	3.224348	3.418471	34.72903
3.332587	3.3621	3.369652	35.19157
3.403113	3.396269	3.346104	35.36903
3.2972	3.347408	3.37504	35.09384
3.258019	3.274219	3.451704	35.01576
3.22853	3.388182	3.350077	34.97824
3.375398	3.53045	3.313092	35.52966
3.315223	3.457462	3.380616	35.38612
3.245275	3.304344	3.504888	35.17007
3.151611	3.153921	3.397685	34.40185
3.328256	3.263099	3.423518	35.0834
3.423281	3.409074	3.376575	35.50777
3.321039	3.319373	3.407097	35.15477
3.217559	3.273141	3.441627	34.90288
3.307841	3.335412	3.445139	35.24417
3.318977	3.372748	3.344484	35.13006
3.414043	3.403008	3.307635	35.32354
3.119606	3.377129	3.444764	34.92294
3.160726	3.388047	3.367862	34.86856
3.207619	3.42545	3.380684	35.08095
3.336546	3.359472	3.350128	35.15179
3.190957	3.281497	3.376558	34.72069
3.289446	3.247801	3.408325	34.93185
3.152972	3.331301	3.478731	34.96997
3.477887	3.454497	3.379474	35.73286
3.411898	3.397212	3.343103	35.38374
3.335391	3.282845	3.345081	34.97065
3.208774	3.292954	3.471467	34.99225
3.192359	3.495944	3.360359	35.15729
3.336505	3.441692	3.464527	35.58167
3.320255	3.352463	3.314592	35.02312
3.245357	3.248677	3.473854	34.98065
3.181141	3.238365	3.443656	34.75163
3.304706	3.294302	3.441933	35.14041
3.355642	3.35462	3.417942	35.33123
3.308872	3.325438	3.342506	35.00017
3.22952	3.23358	3.450272	34.86143
3.278847	3.365807	3.397821	35.14376
3.317945	3.321866	3.34179	35.01064
3.341743	3.448297	3.311199	35.27227
3.21327	3.373894	3.484631	35.20788
3.156849	3.384947	3.427474	34.98367
3.162705	3.346398	3.345848	34.73367
3.4312	3.275904	3.345285	35.16544
3.170913	3.256023	3.412451	34.69964
3.258307	3.307579	3.44311	35.07055
3.254884	3.462584	3.482601	35.48839

3.446419	3.401997	3.328677	35.43815
3.406	3.356035	3.350469	35.2969
3.391812	3.279341	3.374136	35.14991
3.163654	3.410422	3.478987	35.16691
3.309367	3.547635	3.376149	35.56074
3.225107	3.411702	3.368748	35.06302
3.308996	3.207769	3.353248	34.76661
3.144682	3.169826	3.382935	34.38923
3.197144	3.258314	3.481067	34.91206
3.23715	3.422755	3.426604	35.24005
3.391771	3.334873	3.440859	35.41718
3.259462	3.267547	3.386959	34.86274
3.331432	3.245374	3.336691	34.86171
3.328834	3.335345	3.416902	35.22818
3.304748	3.426057	3.384436	35.30288
3.298355	3.442568	3.349429	35.24846
3.28161	3.348621	3.371493	35.05464
3.260122	3.29073	3.349071	34.83202
3.233933	3.317014	3.403295	34.95081
3.272743	3.292213	3.381503	34.93379
3.199989	3.382386	3.364809	34.93537
3.263587	3.334671	3.395297	35.03678
3.314605	3.36729	3.421625	35.27725
3.333618	3.347611	3.41888	35.26979
3.336134	3.296728	3.354169	35.02251
3.357415	3.404423	3.422563	35.45413
3.259009	3.477343	3.413236	35.378
3.218178	3.394314	3.42715	35.13756
3.209352	3.226639	3.410354	34.71485
3.292127	3.275499	3.337936	34.84435
3.170706	3.291943	3.421199	34.79687
3.281651	3.480646	3.412809	35.4338
3.209393	3.347611	3.46023	35.08856
3.342609	3.266401	3.384998	35.03777
3.362612	3.241466	3.439018	35.14511
3.468154	3.38616	3.363854	35.52797
3.312171	3.446679	3.453068	35.51431
3.325658	3.446073	3.370522	35.36196
3.192772	3.338243	3.418488	34.94044
3.355065	3.258112	3.276107	34.80875
3.317327	3.28918	3.363019	34.98423
3.104428	3.325842	3.41003	34.70164
3.142372	3.417363	3.436767	35.04322
3.284002	3.460899	3.361791	35.28419
3.420724	3.384677	3.385169	35.46762
3.45508	3.351048	3.344671	35.38065
3.261483	3.267345	3.420517	34.9401
3.253936	3.394247	3.34433	35.0345
3.3662	3.507671	3.346922	35.53371
3.307511	3.428416	3.376984	35.29779

3.238181	3.219293	3.358654	34.64877
3.181265	3.155673	3.430287	34.54183
3.253482	3.305355	3.407421	34.97708
3.241439	3.436907	3.475082	35.38639
3.311676	3.390203	3.400277	35.27427
3.162994	3.271254	3.487581	34.87992
3.281321	3.258516	3.366429	34.84589
3.281651	3.363381	3.428582	35.21185
3.51047	3.454632	3.337254	35.71208
3.21261	3.352396	3.455507	35.09573
3.202835	3.294302	3.399918	34.82575
3.257153	3.204062	3.430952	34.81506
3.354858	3.359539	3.281154	35.04115
3.238057	3.324966	3.342438	34.84413
3.201845	3.297133	3.379678	34.78551
3.097829	3.315868	3.455319	34.76443
3.347187	3.424237	3.439427	35.51197
3.386451	3.392158	3.312546	35.25021
3.410413	3.407052	3.456871	35.6508
3.248409	3.380768	3.368833	35.04652
3.333659	3.442703	3.389773	35.41418
3.357292	3.411904	3.344467	35.29943
3.366778	3.351115	3.271554	35.02779
3.156106	3.232772	3.408853	34.60854
3.111398	3.217002	3.395707	34.44754
3.276578	3.257505	3.416237	34.94223
3.227664	3.391349	3.409689	35.11364
3.310192	3.37349	3.451892	35.34735
3.154457	3.314722	3.432879	34.83669
3.226055	3.315127	3.403891	34.93076
3.325039	3.388182	3.415811	35.33304
3.47995	3.379555	3.38314	35.5815
3.244821	3.362707	3.47162	35.22396
3.249481	3.329414	3.331235	34.85434
3.331432	3.287293	3.304464	34.8829
3.41297	3.307848	3.411531	35.3403
3.26602	3.293426	3.324721	34.79756
3.212981	3.247531	3.441524	34.83664
3.1592	3.349969	3.380343	34.80925
3.275712	3.444658	3.444866	35.41222
3.381089	3.432998	3.387164	35.49098
3.402165	3.324697	3.372448	35.26805
3.272042	3.334873	3.464322	35.20666
3.297819	3.518251	3.394615	35.51161
3.294519	3.483342	3.528965	35.72185
3.33469	3.317688	3.272646	34.88691
3.252492	3.183237	3.388392	34.66625
3.101129	3.222056	3.362303	34.36308
3.173923	3.380094	3.393985	34.93716
3.284703	3.511242	3.454415	35.59837

3.266804	3.348689	3.469233	35.23615
3.251131	3.247801	3.455234	34.95064
3.174748	3.219428	3.39216	34.58362
3.326772	3.348015	3.40621	35.228
3.40134	3.534426	3.417038	35.8224
3.270639	3.442568	3.404352	35.30796
3.245646	3.198603	3.382253	34.67146
3.310728	3.176228	3.290106	34.56334
3.323307	3.234052	3.397753	34.95271
3.273031	3.392158	3.335481	35.05234
3.296623	3.454834	3.474127	35.54419
3.185802	3.440344	3.395911	35.09911
3.353827	3.337973	3.35156	35.1457
3.307387	3.349363	3.386925	35.14638
3.33568	3.39856	3.402459	35.34981
3.253565	3.414667	3.429878	35.26542
3.326978	3.40813	3.390796	35.3262
3.216322	3.204062	3.425717	34.71432
3.348383	3.115507	3.306817	34.54944
3.159571	3.30832	3.444235	34.85871
3.318564	3.441018	3.401197	35.40247
3.133546	3.476804	3.452199	35.18766
3.231376	3.341478	3.342148	34.86499
3.320874	3.265188	3.402118	35.02502
3.272619	3.298076	3.396184	34.97844
3.214053	3.366009	3.456137	35.13004
3.307181	3.463865	3.387471	35.39752
3.285322	3.383936	3.448771	35.30898
3.42757	3.290191	3.300354	35.09049
3.289817	3.16713	3.37209	34.67701
3.244573	3.239713	3.311608	34.60453
3.132268	3.35408	3.396116	34.79384
3.222921	3.462719	3.392961	35.22276
3.266762	3.478826	3.411377	35.39413
3.34562	3.350104	3.387641	35.23318
3.229313	3.308994	3.439819	35.00304
3.376305	3.420328	3.415214	35.51415
3.407815	3.509153	3.350691	35.6362
3.260163	3.410758	3.440143	35.29375
3.31279	3.248811	3.315871	34.78292
3.232159	3.129525	3.394206	34.51704
3.29555	3.217473	3.37001	34.79509
3.246182	3.409208	3.435727	35.25013
3.328463	3.480848	3.383498	35.47251
3.097417	3.407928	3.420176	34.888
3.251832	3.242544	3.385203	34.78753
3.235665	3.269097	3.382611	34.80457
3.308047	3.338445	3.378723	35.10601
3.235087	3.435694	3.420329	35.25012
3.344424	3.417161	3.485722	35.59169

3.328586	3.37942	3.429315	35.35117
3.404062	3.232772	3.325318	34.9681
3.294849	3.164704	3.34474	34.62289
3.304954	3.247464	3.361672	34.863
3.142661	3.370255	3.380957	34.81879
3.284497	3.430977	3.365014	35.22689
3.303387	3.396606	3.343665	35.14634
3.332133	3.350104	3.387164	35.20264
3.215662	3.370929	3.373727	34.96409
3.287384	3.448903	3.395076	35.33814
3.327473	3.42909	3.42171	35.44073
3.438665	3.349363	3.383208	35.42534
3.301531	3.197727	3.33294	34.68392
3.219332	3.140375	3.354766	34.42647
3.265896	3.337771	3.430407	35.12539
3.289034	3.464943	3.373966	35.33066
3.262391	3.421811	3.432265	35.30557
3.273279	3.362235	3.363991	35.04979
3.177635	3.243555	3.468807	34.81031
3.182832	3.289652	3.396713	34.76483
3.294272	3.401391	3.442957	35.35401
3.264906	3.48132	3.36943	35.30379
3.333041	3.324832	3.412946	35.20574
3.291467	3.25299	3.380974	34.8878
3.366077	3.196581	3.404488	34.97903
3.288044	3.281632	3.358807	34.89448
3.350157	3.437581	3.396116	35.45293
3.168974	3.421339	3.39794	35.02519
3.365252	3.395393	3.335208	35.26049
3.207331	3.309061	3.433544	34.94139
3.37878	3.368031	3.334219	35.22807
3.22985	3.427405	3.436545	35.256
3.295798	3.481926	3.425376	35.49502
3.215208	3.353204	3.429588	35.0465
3.383028	3.193549	3.311216	34.80549
3.289776	3.129727	3.421676	34.70356
3.320833	3.323079	3.391393	35.12808
3.217559	3.413656	3.428036	35.18045
3.284456	3.433807	3.324789	35.14502
3.190916	3.342354	3.364195	34.82664
3.32673	3.269636	3.380275	34.99979
3.179656	3.250496	3.419818	34.72278
3.277733	3.370053	3.469097	35.30648
3.240119	3.437446	3.465942	35.3647
3.373294	3.397078	3.405102	35.43461
3.367685	3.227582	3.334986	34.89835
3.311635	3.109239	3.42877	34.72207
3.253482	3.221382	3.397395	34.77152
3.183327	3.429561	3.452096	35.19298
3.303593	3.513669	3.332138	35.37759

3.272578	3.417296	3.38921	35.22382
3.197226	3.369918	3.388596	34.95408
3.241521	3.358731	3.42524	35.10662
3.363272	3.465887	3.379303	35.50674
3.276083	3.452543	3.430543	35.39895
3.283796	3.397482	3.411104	35.2529
3.211001	3.196716	3.399936	34.63024
3.315718	3.180946	3.347707	34.71054
3.386946	3.286889	3.30813	35.01143
3.333329	3.456317	3.459889	35.59656
3.248368	3.443579	3.390779	35.23178
3.293406	3.350643	3.425632	35.20325
3.19273	3.288708	3.37504	34.73702
3.356302	3.312701	3.350878	35.09435
3.201928	3.372951	3.422427	35.04498
3.205186	3.433605	3.476105	35.30213
3.174377	3.421272	3.47162	35.19798
3.372346	3.302659	3.380957	35.17325
3.380635	3.175554	3.402237	34.95996
3.371314	3.203253	3.342028	34.86848
3.196401	3.272197	3.453085	34.87961
3.254472	3.461978	3.336009	35.16559
3.312914	3.419587	3.297558	35.11661
3.360302	3.358731	3.301019	35.09472
3.198834	3.377399	3.403789	35.00718
3.239129	3.388721	3.368561	35.04302
3.270268	3.494394	3.411582	35.43629
3.322523	3.429494	3.349838	35.27361
3.318482	3.231896	3.450272	35.05229
3.156395	3.195099	3.432453	34.5784
3.257359	3.275836	3.40783	34.9219
3.330772	3.357248	3.393149	35.22838
3.433592	3.466628	3.4146	35.73933
3.174748	3.356978	3.42142	34.94841
3.251337	3.329616	3.452659	35.12438
3.233603	3.26721	3.398571	34.83084
3.316254	3.332177	3.336708	35.01838
3.210259	3.357315	3.428514	35.04232
3.268041	3.426596	3.433885	35.33193
3.171449	3.348958	3.413355	34.90602
3.434829	3.254472	3.33739	35.10924
3.339351	3.232435	3.414463	35.0208
3.380223	3.286282	3.405187	35.20765
3.196071	3.35617	3.467136	35.09325
3.277197	3.475119	3.345848	35.26554
3.329865	3.384205	3.322521	35.13089
3.422827	3.359404	3.271418	35.1682
3.195824	3.367222	3.441797	35.06146
3.169717	3.520071	3.442002	35.33907
3.219869	3.489137	3.492491	35.49151

3.298396	3.334401	3.358603	35.03207
3.316461	3.146643	3.386635	34.72227
3.244202	3.199951	3.415811	34.74464
3.253235	3.371131	3.442002	35.19601
3.344011	3.459215	3.384572	35.46156
3.361457	3.400784	3.33942	35.27319
3.257029	3.32901	3.401982	35.02468
3.201845	3.306029	3.353197	34.74706
3.292292	3.245172	3.425769	34.97047
3.357292	3.408871	3.375569	35.36082
3.249811	3.409748	3.486063	35.36932
3.289199	3.372748	3.373932	35.12933
3.210671	3.265727	3.425359	34.83603
3.343063	3.244566	3.366702	34.951
3.361457	3.239039	3.435607	35.12983
3.363148	3.338041	3.403755	35.28037
3.222714	3.450521	3.445071	35.30959
3.36521	3.415543	3.38389	35.41092
3.277692	3.35718	3.347212	35.01169
3.297447	3.367492	3.377547	35.14378
3.170954	3.407861	3.34844	34.89179
3.257483	3.445803	3.414736	35.30897
3.114987	3.319642	3.391052	34.66966
3.279837	3.145295	3.364468	34.59076
3.260906	3.189438	3.455302	34.84453
3.382203	3.324764	3.416714	35.32134
3.268123	3.445466	3.414327	35.33061
3.307717	3.460023	3.406978	35.43295
3.360261	3.342691	3.348849	35.16415
3.339928	3.311757	3.335958	35.02385
3.155075	3.273141	3.365901	34.60064
3.237026	3.434009	3.406552	35.22054
3.238593	3.40166	3.450664	35.24969
3.286188	3.415139	3.404232	35.28171
3.348094	3.247531	3.417055	35.0786
3.225354	3.164502	3.41622	34.62679
3.232943	3.239915	3.387283	34.74502
3.280579	3.285069	3.40824	34.99377
3.309738	3.44769	3.43172	35.46451
3.304459	3.450251	3.340204	35.25843
3.362488	3.334604	3.410013	35.28509
3.299138	3.390608	3.318514	35.06894
3.36253	3.417363	3.38169	35.40423
3.215167	3.48934	3.354169	35.17919
3.31114	3.332447	3.382134	35.10712
3.179038	3.219697	3.378092	34.56283
3.237521	3.19557	3.367469	34.61473
3.374861	3.310342	3.43559	35.31503
3.333371	3.508682	3.395212	35.56973
3.294725	3.414398	3.49065	35.48774

3.35226	3.356102	3.419255	35.32995
3.255833	3.302726	3.433135	35.03271
3.40633	3.395932	3.344126	35.371
3.197185	3.384542	3.441388	35.10142
3.10814	3.348756	3.391819	34.72004
3.140475	3.378881	3.42865	34.93717
3.32607	3.354552	3.352038	35.1223
3.306439	3.197525	3.480879	35.01773
3.279135	3.258314	3.379525	34.86931
3.15025	3.229604	3.515682	34.82243
3.349002	3.412646	3.358858	35.3144
3.445264	3.474109	3.403278	35.75646
3.336835	3.440614	3.295034	35.20938
3.318234	3.394045	3.364741	35.2193
3.218013	3.39054	3.415896	35.10434
3.286559	3.443647	3.275118	35.06251
3.306727	3.403345	3.361604	35.20762
3.258596	3.283789	3.344535	34.80358
3.099232	3.114091	3.463572	34.34429
3.247708	3.231356	3.367197	34.71467
3.314728	3.392697	3.48076	35.4624
3.350899	3.452273	3.327859	35.33742
3.219951	3.335075	3.503541	35.17895
3.329122	3.341478	3.469693	35.35767
3.2607	3.389799	3.4161	35.19651
3.416187	3.420261	3.358279	35.47671
3.226591	3.316542	3.414975	34.95926
3.202423	3.327729	3.371493	34.83579
3.088673	3.348352	3.431362	34.76306
3.324874	3.265525	3.336964	34.89203
3.30813	3.160391	3.350742	34.65563
3.267257	3.239107	3.414446	34.8777
3.213435	3.324832	3.452642	35.03099
3.348713	3.389529	3.419306	35.39541
3.465019	3.440749	3.327586	35.56118
3.490384	3.421541	3.371903	35.67156
3.259132	3.353811	3.328114	34.92197
3.204196	3.349902	3.434107	35.02508
3.297035	3.431044	3.366821	35.2584
3.351518	3.399099	3.36943	35.3134
3.277774	3.213362	3.388102	34.78679
3.080548	3.169826	3.468074	34.43516
3.199247	3.348554	3.43779	35.01936
3.424601	3.46218	3.447475	35.78184
3.370448	3.412915	3.387778	35.42513
3.276083	3.368705	3.426519	35.20681
3.259957	3.324697	3.409075	35.03716
3.272371	3.358731	3.453273	35.23539
3.387853	3.372883	3.303014	35.19028
3.279672	3.337097	3.422512	35.13677

3.237933	3.332919	3.415145	35.02025
3.146497	3.299828	3.347894	34.60086
3.34694	3.263975	3.401436	35.07788
3.207537	3.276915	3.363377	34.7181
3.266309	3.342893	3.430475	35.13764
3.180027	3.346532	3.410917	34.91414
3.475289	3.40375	3.451977	35.77475
3.345084	3.402199	3.385391	35.34101
3.454502	3.395056	3.34844	35.48386
3.303222	3.360752	3.389415	35.16762
3.178007	3.36237	3.343307	34.79651
3.188565	3.35563	3.399253	34.9272
3.212239	3.249553	3.293841	34.51648
3.213765	3.186135	3.378621	34.56653
3.202051	3.301513	3.409706	34.86121
3.204361	3.478759	3.478373	35.40403
3.418126	3.431718	3.45196	35.71087
3.413878	3.393977	3.376319	35.45363
3.294808	3.321799	3.382679	35.04931
3.285652	3.339995	3.42715	35.16633
3.332009	3.390338	3.381588	35.27816
3.277073	3.290663	3.406824	34.99524
3.287384	3.297402	3.305743	34.81148
3.307882	3.271186	3.500864	35.22567
3.14394	3.242207	3.34919	34.4721
3.335061	3.224482	3.429179	35.02621
3.232737	3.2972	3.429742	34.9627
3.20597	3.373962	3.422461	35.0561
3.219374	3.422822	3.405597	35.15539
3.361004	3.427405	3.344535	35.3416
3.370366	3.380431	3.388801	35.35615
3.453224	3.430707	3.351441	35.56559
3.277032	3.406378	3.452591	35.34828
3.311883	3.392697	3.310364	35.08355
3.1559	3.321664	3.390438	34.76221
3.320296	3.276847	3.321771	34.87355
3.165469	3.248609	3.398691	34.64143
3.21727	3.367492	3.405443	35.02945
3.23286	3.397415	3.461901	35.25245
3.40167	3.376859	3.425854	35.49783
3.338113	3.391619	3.45832	35.46211
3.434293	3.430572	3.358176	35.53863
3.293406	3.406176	3.453068	35.38469
3.276702	3.395325	3.394735	35.19687
3.227911	3.294707	3.412605	34.90921
3.206093	3.272264	3.36261	34.70309
3.254348	3.283384	3.384248	34.88026
3.167448	3.237961	3.335481	34.48424
3.228612	3.238028	3.456069	34.88185
3.245522	3.301244	3.431379	35.00308

3.288291	3.320586	3.383225	35.0336
3.303304	3.452138	3.41813	35.43045
3.377625	3.45106	3.42142	35.59781
3.301902	3.424709	3.384043	35.29286
3.466174	3.404558	3.285468	35.39246
3.302314	3.374635	3.421318	35.26577
3.316832	3.370053	3.355192	35.14289
3.205433	3.248407	3.384453	34.69725
3.28392	3.171646	3.341159	34.60634
3.157839	3.267682	3.423228	34.72011
3.301201	3.432257	3.415128	35.37581
3.213847	3.446882	3.51043	35.42517
3.365375	3.34195	3.400055	35.28569
3.230138	3.346465	3.412963	35.02805
3.400267	3.396134	3.401146	35.48288
3.330896	3.46656	3.46173	35.61767
3.318605	3.362168	3.390182	35.20604
3.194545	3.292617	3.364093	34.72559
3.204279	3.233446	3.400822	34.6978
3.258555	3.312296	3.377427	34.93777
3.228901	3.230952	3.390438	34.72348
3.20267	3.291067	3.466266	34.96341
3.264865	3.344713	3.39407	35.05885
3.319513	3.383531	3.368015	35.20627
3.318812	3.40658	3.387045	35.29675
3.398741	3.402806	3.43438	35.56681
3.321286	3.478691	3.372892	35.42891
3.347022	3.414263	3.377479	35.35433
3.361911	3.345454	3.32646	35.12484
3.359189	3.276038	3.328779	34.97216
3.18477	3.256629	3.398094	34.69987
3.26136	3.276982	3.363496	34.83621
3.182213	3.316273	3.42449	34.88243
3.286106	3.463865	3.359199	35.28961
3.213228	3.377129	3.437108	35.11093
3.285734	3.365605	3.442428	35.25593
3.222426	3.371064	3.379542	34.9919
3.346362	3.391416	3.405801	35.36486
3.289941	3.418981	3.422836	35.339
3.348796	3.278869	3.337868	34.9755
3.190008	3.195705	3.388562	34.55725
3.297901	3.182968	3.341602	34.66264
3.255503	3.25892	3.341892	34.73666
3.227664	3.320586	3.41801	34.97709
3.18011	3.386429	3.438728	35.06239
3.236077	3.393977	3.427201	35.17608
3.350322	3.406176	3.425104	35.448
3.449677	3.418239	3.375927	35.58412
3.351518	3.399099	3.400686	35.38175
3.333412	3.394314	3.408257	35.34825

3.295674	3.358663	3.360291	35.08286
3.304583	3.275365	3.342574	34.88144
3.35692	3.29228	3.34445	35.03699
3.171449	3.235872	3.383907	34.59432
3.218136	3.265121	3.385527	34.76392
3.244491	3.395932	3.429725	35.20427
3.303057	3.465617	3.419971	35.46341
3.278805	3.375242	3.409194	35.18917
3.316172	3.382049	3.433408	35.33872
3.225931	3.386362	3.366378	35.00423
3.437428	3.416285	3.383617	35.56987
3.374408	3.401526	3.382543	35.39743
3.297406	3.245981	3.375859	34.87428
3.218714	3.158639	3.394172	34.55123
3.238882	3.28143	3.409894	34.89824
3.249234	3.262964	3.363565	34.77918
3.257441	3.350104	3.392007	35.04989
3.192318	3.408804	3.454978	35.17355
3.198834	3.404828	3.424746	35.113
3.291921	3.406311	3.398418	35.26223
3.361705	3.412646	3.367179	35.36038
3.338443	3.407389	3.422222	35.41838
3.319554	3.370929	3.39262	35.23261
3.255668	3.245577	3.423535	34.88638
3.327926	3.240994	3.28748	34.73684
3.337124	3.249553	3.369174	34.95433
3.27596	3.29538	3.320356	34.81403
3.183244	3.310409	3.391273	34.79923
3.226798	3.398425	3.432521	35.17715
3.270681	3.409613	3.464118	35.36668
3.387812	3.368031	3.339965	35.26039
3.308253	3.411567	3.491673	35.51337
3.276455	3.428753	3.352856	35.17785
3.292622	3.383868	3.454006	35.33625
3.32574	3.326921	3.349599	35.05582
3.331762	3.181485	3.424217	34.91412
3.183368	3.193212	3.400805	34.56405
3.201515	3.266334	3.407353	34.77796
3.233025	3.319912	3.395502	34.93812
3.279837	3.355428	3.412946	35.1563
3.240738	3.39209	3.414344	35.15403
3.301778	3.347341	3.423722	35.21017
3.175078	3.420665	3.453597	35.15877
3.441346	3.48105	3.353265	35.6537
3.36818	3.432122	3.381844	35.4492
3.408104	3.331301	3.322351	35.18592
3.273733	3.23385	3.417516	34.88707
3.350693	3.236411	3.342625	34.8972
3.23484	3.296324	3.340852	34.77099
3.343104	3.340534	3.329837	35.08034

3.135443	3.373692	3.438199	34.9357
3.193679	3.385688	3.359881	34.91802
3.213063	3.402402	3.414054	35.11543
3.313697	3.403817	3.366583	35.23478
3.324709	3.419048	3.428633	35.42786
3.264741	3.424305	3.464373	35.38638
3.197185	3.337097	3.428787	34.97011
3.356673	3.240455	3.369226	34.97729
3.312831	3.198536	3.327296	34.69805
3.247007	3.256225	3.373403	34.78109
3.13899	3.241398	3.363138	34.49001
3.182708	3.325438	3.452523	34.96486
3.319307	3.480713	3.330774	35.3369
3.330937	3.382723	3.361177	35.21453
3.286724	3.4146	3.412332	35.29942
3.267546	3.407726	3.451295	35.32765
3.312708	3.411028	3.400259	35.32203
3.355931	3.389732	3.382253	35.3306
3.342114	3.242679	3.341739	34.89021
3.226055	3.209993	3.406756	34.70711
3.352631	3.255214	3.413764	35.09813
3.278104	3.347611	3.381383	35.0664
3.327308	3.375309	3.31923	35.09865
3.203371	3.361898	3.400106	34.97515
3.168974	3.369851	3.428105	34.97855
3.13466	3.371266	3.419221	34.88718
3.366736	3.487857	3.413696	35.63758
3.289694	3.418441	3.358261	35.19607
3.353662	3.391956	3.399714	35.36869
3.180852	3.246924	3.440228	34.76222
3.315718	3.213093	3.401078	34.89755
3.352837	3.179531	3.329461	34.74872
3.24445	3.244363	3.366088	34.73356
3.216198	3.319238	3.389892	34.88758
3.244079	3.418037	3.471006	35.34199
3.296664	3.459215	3.417106	35.42916
3.341537	3.394314	3.310432	35.15209
3.262844	3.406243	3.43293	35.27397
3.209805	3.42417	3.429571	35.18984
3.30252	3.436705	3.440655	35.44424
3.300788	3.365403	3.293875	34.96354
3.339846	3.255281	3.350759	34.93253
3.227705	3.223337	3.369584	34.65861
3.287219	3.267075	3.402954	34.95738
3.288168	3.405637	3.391802	35.23808
3.336093	3.36547	3.374085	35.2163
3.261607	3.356911	3.413781	35.12151
3.214095	3.368637	3.464169	35.15344
3.190751	3.396471	3.420466	35.06768
3.373335	3.476535	3.371084	35.53406

3.301819	3.397145	3.414532	35.29907
3.281981	3.349767	3.380087	35.07676
3.218961	3.212486	3.429929	34.74773
3.370861	3.19402	3.367145	34.90223
3.280496	3.160391	3.374836	34.64789
3.351353	3.304209	3.358517	35.08166
3.140104	3.314588	3.378451	34.68598
3.296458	3.386092	3.340408	35.10108
3.196937	3.327392	3.461117	35.01905
3.278476	3.429764	3.392893	35.27203
3.272454	3.439131	3.424933	35.34942
3.264164	3.444388	3.450732	35.3992
3.255915	3.342219	3.463623	35.18593
3.382574	3.302659	3.353504	35.13558
3.372304	3.277656	3.38181	35.12035
3.24247	3.3065	3.389636	34.91662
3.315058	3.386564	3.3991	35.27114
3.280167	3.345454	3.379678	35.06246
3.3005	3.352126	3.338124	35.03064
3.292003	3.300907	3.350077	34.92619
3.250512	3.421002	3.478987	35.37999
3.128762	3.440277	3.450886	35.09444
3.265401	3.409545	3.428497	35.27709
3.347352	3.270445	3.446589	35.19167
3.341495	3.283789	3.379167	35.06061
3.229272	3.267816	3.474263	34.98822
3.281115	3.261684	3.41917	34.9677
3.214342	3.251103	3.392586	34.74041
3.289735	3.360281	3.354851	35.06152
3.212362	3.371805	3.413781	35.04639
3.251337	3.396875	3.322862	34.98762
3.155529	3.355698	3.388477	34.83154
3.257359	3.441625	3.342097	35.14071
3.278805	3.441827	3.502023	35.53778
3.334567	3.40348	3.357153	35.25906
3.260988	3.255079	3.422495	34.91652
3.255049	3.247801	3.383259	34.80181
3.418992	3.298144	3.412144	35.33359
3.391441	3.399639	3.333349	35.32298
3.289859	3.365739	3.401845	35.17649
3.278599	3.367963	3.412161	35.17929
3.154663	3.362909	3.373591	34.81286
3.298726	3.448162	3.358313	35.28093
3.300087	3.509288	3.434039	35.58318
3.172439	3.376725	3.505928	35.17135
3.217971	3.263503	3.404045	34.80052
3.340836	3.242544	3.432811	35.08628
3.309821	3.260471	3.397599	34.98065
3.259256	3.232435	3.385152	34.78155
3.261277	3.217271	3.436562	34.86523

3.181883	3.262762	3.377138	34.66114
3.416806	3.413724	3.329308	35.40041
3.324132	3.409208	3.402527	35.34799
3.320626	3.439064	3.383208	35.36337
3.098695	3.365537	3.472336	34.91216
3.244202	3.443107	3.383702	35.20616
3.357292	3.460158	3.407234	35.54222
3.313986	3.360146	3.411531	35.2382
3.195164	3.205073	3.423995	34.6665
3.269938	3.217339	3.419238	34.84643
3.299963	3.349767	3.382185	35.12067
3.398865	3.434144	3.404574	35.57043
3.340836	3.380297	3.426451	35.37361
3.204155	3.30057	3.428088	34.90394
3.152931	3.346869	3.41049	34.85469
3.299881	3.502751	3.371698	35.4321
3.228736	3.476804	3.4663	35.42666
3.260658	3.344713	3.374102	35.00598
3.130082	3.240657	3.514863	34.8007
3.271134	3.276915	3.383361	34.90088
3.413919	3.314318	3.43322	35.40395
3.370448	3.282037	3.333434	35.02008
3.260205	3.238433	3.428599	34.89175
3.249234	3.354215	3.380497	35.01576
3.31312	3.374635	3.407148	35.25841
3.402329	3.426057	3.316826	35.36843
3.257483	3.40193	3.399509	35.17972
3.117049	3.366818	3.407148	34.81254
3.332092	3.428753	3.427883	35.46359
3.377171	3.388721	3.377751	35.365
3.294354	3.270108	3.361791	34.8896
3.202423	3.193212	3.385936	34.5732
3.22787	3.353743	3.456956	35.13522
3.294189	3.432931	3.427423	35.38883
3.468525	3.37167	3.333315	35.43031
3.28858	3.298009	3.418624	35.06227
3.215868	3.327932	3.384026	34.89304
3.209187	3.391619	3.435727	35.13076
3.342444	3.476602	3.423381	35.58102
3.178996	3.394517	3.412963	35.0213
3.243707	3.295448	3.404642	34.92797
3.160024	3.26074	3.457229	34.78406
3.385832	3.353406	3.382168	35.31637
3.29188	3.291943	3.44386	35.11141
3.387771	3.255079	3.415691	35.17889
3.281445	3.263301	3.484562	35.11497
3.249234	3.379825	3.352072	35.00961
3.361375	3.401526	3.422512	35.45634
3.349043	3.396673	3.30136	35.15382
3.222962	3.3466	3.376063	34.93196

3.200979	3.455036	3.428429	35.23554
3.209846	3.402806	3.407762	35.09552
3.369335	3.309331	3.426093	35.27996
3.258844	3.225291	3.407165	34.81316
3.221312	3.255012	3.425223	34.83557
3.355601	3.386632	3.39025	35.34059
3.300664	3.391214	3.365491	35.17633
3.360509	3.391012	3.356812	35.28778
3.34562	3.339321	3.381844	35.19692
3.214053	3.414533	3.394803	35.10202
3.238634	3.483409	3.38227	35.27899
3.335309	3.405232	3.42333	35.40923
3.169428	3.353204	3.402851	34.88792
3.278888	3.293628	3.403619	34.99868
3.143032	3.30832	3.467374	34.87314
3.319801	3.27961	3.377223	34.99978
3.295055	3.205679	3.40928	34.85409
3.356797	3.255012	3.392007	35.05921
3.308336	3.33393	3.441303	35.23362
3.367561	3.409343	3.374341	35.38162
3.30483	3.347071	3.404932	35.17516
3.284332	3.397684	3.371221	35.16729
3.177594	3.385284	3.402664	34.97552
3.222921	3.519734	3.462054	35.49854
3.223952	3.381375	3.404011	35.07129
3.309821	3.254944	3.335191	34.83209
3.299345	3.253327	3.422921	34.9975
3.354817	3.33393	3.431362	35.31353
3.331308	3.457867	3.422853	35.51454
3.405175	3.409545	3.309818	35.32322
3.292828	3.299289	3.429162	35.09741
3.322152	3.330425	3.306595	34.96159
3.222096	3.421474	3.417601	35.18464
3.144434	3.518454	3.412537	35.21581
3.247749	3.340602	3.387232	34.99748
3.173016	3.269703	3.455251	34.82775
3.255627	3.258718	3.376592	34.81237
3.264082	3.340871	3.412127	35.08822
3.32838	3.307848	3.403056	35.13678
3.18275	3.249081	3.41356	34.71277
3.399896	3.304411	3.425786	35.33536
3.308913	3.362639	3.373335	35.14903
3.316543	3.398291	3.413014	35.33046
3.293942	3.35718	3.406688	35.17729
3.283878	3.400986	3.391751	35.21842
3.218631	3.411904	3.343017	34.99304
3.334608	3.352261	3.384436	35.2068
3.168273	3.275162	3.442138	34.80064
3.238593	3.267143	3.417533	34.88307
3.162252	3.366279	3.38314	34.85771

3.449347	3.415072	3.410149	35.65131
3.380553	3.432527	3.393388	35.50238
3.406	3.315868	3.364434	35.2396
3.254967	3.296998	3.399594	34.94494
3.333164	3.429494	3.424149	35.45939
3.246759	3.478354	3.396099	35.31594
3.281445	3.414398	3.356147	35.16457
3.112801	3.194425	3.441797	34.50202
3.187658	3.242679	3.379269	34.63451
3.317038	3.354215	3.473121	35.3666
3.307635	3.426866	3.39424	35.33241
3.257524	3.278599	3.499159	35.12803
3.272743	3.229537	3.387437	34.8097
3.296086	3.248137	3.424541	34.98257
3.430952	3.385621	3.299041	35.3037
3.214919	3.387575	3.405324	35.06797
3.149425	3.379016	3.360836	34.80874
3.285033	3.329819	3.390114	35.06173
3.229107	3.358933	3.358807	34.93464
3.288498	3.274421	3.337714	34.83357
3.192689	3.245779	3.37828	34.65013
3.210218	3.304748	3.420517	34.90978
3.157344	3.370929	3.438881	34.97904
3.465886	3.456317	3.297916	35.53224
3.318193	3.362168	3.433561	35.3
3.414208	3.324158	3.370266	35.28843
3.191658	3.392764	3.509066	35.25532
3.362158	3.517847	3.352447	35.55921
3.277651	3.404423	3.363189	35.14986
3.271258	3.264447	3.329751	34.75665
3.115853	3.225426	3.426519	34.54308
3.20931	3.318497	3.448004	34.99797
3.240655	3.403008	3.416714	35.18291
3.360509	3.322271	3.356744	35.1373
3.22886	3.268895	3.452096	34.9412
3.294272	3.265121	3.434107	35.03666
3.318358	3.37996	3.426246	35.32327
3.400515	3.41487	3.397139	35.51563
3.251709	3.384542	3.42715	35.18952
3.212032	3.333188	3.390472	34.91024
3.263174	3.34195	3.402374	35.06727
3.259009	3.374635	3.410985	35.14847
3.32141	3.297267	3.329751	34.93809
3.232654	3.26903	3.426587	34.89401
3.255049	3.34633	3.395553	35.04416
3.179491	3.411567	3.439222	35.11709
3.460276	3.43994	3.325659	35.54483
3.445965	3.382116	3.441251	35.63986
3.357993	3.332043	3.371391	35.1852
3.249811	3.441288	3.424404	35.30346

3.334443	3.467437	3.439137	35.57794
3.248574	3.371064	3.321038	34.92114
3.271959	3.187416	3.374341	34.68724
3.11643	3.221652	3.395383	34.468
3.160107	3.324225	3.421744	34.84548
3.194174	3.408737	3.416527	35.09338
3.256864	3.240387	3.416919	34.86318
3.217435	3.251642	3.385953	34.73385
3.40402	3.320047	3.416288	35.35781
3.234345	3.35253	3.41119	35.04664
3.392514	3.403278	3.3895	35.45607
3.290354	3.402941	3.366276	35.18114
3.361911	3.333458	3.367128	35.18754
3.225478	3.279947	3.400532	34.84522
3.292622	3.305826	3.379457	35.00255
3.174542	3.289585	3.352413	34.64967
3.260493	3.367222	3.385936	35.08073
3.258101	3.374096	3.443877	35.21724
3.237191	3.410219	3.397344	35.14874
3.329947	3.373692	3.398912	35.27514
3.398535	3.320249	3.382406	35.27216
3.325452	3.364324	3.392706	35.23125
3.234098	3.489137	3.428139	35.3819
3.254307	3.390945	3.461782	35.28494
3.222508	3.196918	3.340818	34.52656
3.292952	3.236276	3.362746	34.81464
3.254389	3.302052	3.41119	34.98009
3.226591	3.466156	3.462736	35.39089
3.137382	3.427	3.418198	35.01278
3.328628	3.264919	3.406347	35.05064
3.292746	3.246116	3.385851	34.88623
3.349497	3.345252	3.409126	35.27803
3.295633	3.367761	3.432163	35.25984
3.395071	3.494866	3.393985	35.67176
3.241315	3.318766	3.418965	35.00505
3.412145	3.214845	3.295085	34.88046
3.191823	3.227111	3.39939	34.65358
3.165056	3.328066	3.364025	34.73848
3.157179	3.391282	3.414344	34.96953
3.289611	3.449442	3.393712	35.34121
3.373954	3.379825	3.391665	35.36894
3.348919	3.373018	3.457331	35.4429
3.201969	3.328606	3.443298	34.99373
3.379192	3.394045	3.349122	35.31845
3.330896	3.42727	3.407404	35.41295
3.380058	3.370187	3.333758	35.23458
3.215909	3.174678	3.413133	34.62164
3.13268	3.169961	3.381827	34.36085
3.247378	3.355833	3.378519	35.01092
3.310728	3.424776	3.38343	35.31096

3.231334	3.413926	3.433834	35.22384
3.169428	3.31479	3.447595	34.90176
3.258472	3.308185	3.388852	34.95358
3.36785	3.368907	3.405477	35.36192
3.3589	3.401458	3.355874	35.30505
3.1778	3.318227	3.441524	34.91431
3.221065	3.354013	3.358671	34.90599
3.242388	3.307309	3.450596	35.05151
3.388843	3.269636	3.290703	34.93974
3.271052	3.265997	3.351049	34.80616
3.077579	3.260066	3.384043	34.44225
3.153838	3.346061	3.368475	34.76303
3.352507	3.449914	3.348184	35.38022
3.297406	3.439603	3.409263	35.37075
3.361581	3.428213	3.429878	35.53126
3.226055	3.388047	3.499994	35.30038
3.372758	3.444994	3.334833	35.38455
3.386162	3.422957	3.414276	35.5394
3.362695	3.361831	3.367128	35.2513
3.230881	3.179733	3.393643	34.62281
3.200979	3.231424	3.325915	34.52235
3.161344	3.321057	3.396252	34.78551
3.330731	3.436031	3.408188	35.43346
3.215786	3.395123	3.426485	35.13265
3.139114	3.259662	3.45682	34.73508
3.256575	3.329684	3.430151	35.08676
3.402165	3.409343	3.408444	35.53187
3.320709	3.41952	3.401419	35.36063
3.259669	3.432527	3.434567	35.32808
3.207042	3.353878	3.462549	35.1022
3.243254	3.26694	3.285127	34.60327
3.463411	3.236478	3.353368	35.16734
3.257359	3.30185	3.378706	34.9151
3.208527	3.369783	3.382134	34.96437
3.11643	3.388182	3.412963	34.87062
3.361911	3.401526	3.343819	35.28542
3.311965	3.356776	3.367844	35.13088
3.293282	3.377668	3.352737	35.10267
3.201227	3.387777	3.405119	35.03802
3.37482	3.48597	3.444406	35.71829
3.28726	3.405232	3.391052	35.23357
3.42856	3.221854	3.335753	35.02062
3.229066	3.135523	3.402186	34.54085
3.157385	3.296998	3.42761	34.79281
3.20036	3.432594	3.391069	35.10341
3.322895	3.446545	3.39407	35.40844
3.187369	3.312431	3.38036	34.7888
3.205805	3.202175	3.391904	34.61325
3.156024	3.257573	3.454569	34.76257
3.340506	3.411028	3.363496	35.30242

3.367644	3.466021	3.439614	35.64849
3.389915	3.399167	3.405102	35.47552
3.282146	3.230346	3.382816	34.82193
3.296004	3.159987	3.303475	34.52486
3.335515	3.259931	3.350912	34.93357
3.301201	3.390877	3.320407	35.07818
3.175779	3.456586	3.423262	35.17252
3.239707	3.439468	3.394138	35.2112
3.327431	3.368166	3.424677	35.31389
3.316213	3.38306	3.399577	35.26704
3.280002	3.4115	3.412076	35.27738
3.212074	3.390271	3.390574	35.03539
3.230798	3.379488	3.428258	35.13516
3.282517	3.271927	3.376046	34.89887
3.340341	3.128379	3.384026	34.72885
3.228324	3.272601	3.421557	34.88135
3.202876	3.406782	3.3762	35.01995
3.221353	3.433605	3.393729	35.15734
3.33403	3.414465	3.378655	35.32893
3.186008	3.226032	3.360427	34.55329
3.261277	3.307579	3.383208	34.94604
3.198422	3.368637	3.4508	35.08993
3.303675	3.416622	3.382679	35.27607
3.356302	3.406445	3.437722	35.48927
3.398411	3.3497	3.352123	35.27007
3.296086	3.147317	3.426041	34.76537
3.281651	3.19894	3.309682	34.59223
3.216817	3.353272	3.421062	35.03152
3.302603	3.407726	3.376797	35.2414
3.272413	3.410422	3.358944	35.14223
3.234056	3.429292	3.361399	35.10499
3.230881	3.368098	3.417447	35.08679
3.295839	3.438323	3.406415	35.35829
3.276042	3.45571	3.433357	35.41194
3.262762	3.406243	3.455472	35.32308
3.317739	3.244094	3.417055	35.0047
3.247419	3.136668	3.375432	34.52498
3.374779	3.198805	3.304583	34.78444
3.317863	3.425113	3.35214	35.25888
3.17714	3.464876	3.404778	35.1532
3.217518	3.372614	3.42686	35.08803
3.2753	3.328606	3.339556	34.92723
3.238057	3.2416	3.362354	34.70538
3.335433	3.31634	3.364383	35.0862
3.136021	3.394854	3.48799	35.09213
3.215084	3.487655	3.455234	35.39633
3.271588	3.299357	3.393439	34.97298
3.417425	3.181081	3.364877	34.9708
3.231169	3.155539	3.380497	34.54179
3.286765	3.247329	3.422563	34.95609

3.211702	3.444186	3.423347	35.22415
3.397504	3.452812	3.34312	35.47389
3.220322	3.403615	3.358944	35.01343
3.333371	3.275634	3.325846	34.9084
3.162334	3.38616	3.414736	34.97046
3.225766	3.480578	3.393166	35.26849
3.330896	3.435088	3.39291	35.39835
3.39239	3.260268	3.363241	35.08564
3.176027	3.139027	3.377342	34.37819
3.277898	3.160458	3.438404	34.78137
3.304624	3.384475	3.411803	35.27153
3.38146	3.496146	3.393882	35.64457
3.185967	3.380229	3.336231	34.83749
3.128432	3.307781	3.378246	34.64512
3.224942	3.306298	3.413014	34.92896
3.270763	3.345252	3.371545	35.02367
3.228489	3.360887	3.429401	35.09193
3.218961	3.392764	3.401351	35.07947
3.291591	3.289045	3.466505	35.15396
3.353497	3.256494	3.308045	34.87163
3.445718	3.240589	3.382679	35.20173
3.263215	3.25737	3.3629	34.79607
3.252203	3.38899	3.457894	35.26756
3.285528	3.402267	3.335191	35.10114
3.35226	3.368503	3.3526	35.21131
3.315553	3.418509	3.34699	35.22812
3.317863	3.44331	3.465669	35.54694
3.143651	3.44924	3.482329	35.21537
3.281363	3.415611	3.385476	35.23118
3.280249	3.365133	3.368919	35.08215
3.34929	3.19584	3.430355	34.99726
3.177924	3.177846	3.389892	34.49468
3.379852	3.2416	3.40546	35.10973
3.244945	3.471211	3.442633	35.39812
3.417507	3.442366	3.294744	35.389
3.151817	3.306635	3.384555	34.70755
3.339928	3.271523	3.346053	34.95794
3.168768	3.244026	3.438097	34.7248
3.23682	3.380701	3.326801	34.92911
3.189307	3.462921	3.512408	35.41091
3.349744	3.411837	3.455796	35.52624
3.312171	3.243757	3.404113	34.96349
3.402165	3.229806	3.37209	35.05975
3.267835	3.222595	3.385595	34.77976
3.333494	3.422889	3.405869	35.40569
3.256369	3.46036	3.391342	35.28721
3.246842	3.441423	3.391342	35.22496
3.261648	3.387575	3.343734	35.03547
3.244821	3.347813	3.366924	34.96243
3.208857	3.443444	3.386823	35.13643

3.252327	3.443916	3.430083	35.32713
3.204732	3.386227	3.432589	35.10237
3.168232	3.163626	3.435539	34.54221
3.361746	3.182226	3.350861	34.82089
3.264741	3.235602	3.386618	34.80368
3.349785	3.410961	3.393405	35.38798
3.303881	3.442434	3.468398	35.52041
3.28293	3.364392	3.365696	35.07934
3.262391	3.332717	3.356744	34.94558
3.289735	3.320518	3.418266	35.11324
3.135732	3.367087	3.441081	34.92819
3.231087	3.378207	3.399577	35.07027
3.201103	3.351991	3.423381	34.99943
3.323183	3.284597	3.393456	35.05358
3.382698	3.231154	3.403755	35.08938
3.297489	3.218754	3.417447	34.90586
3.294478	3.291067	3.371732	34.95744
3.306851	3.413859	3.434976	35.39133
3.341248	3.432594	3.393371	35.41654
3.342898	3.378814	3.341704	35.18955
3.160354	3.341747	3.336231	34.69733
3.229767	3.463797	3.416203	35.29092
3.194958	3.50794	3.380497	35.23324
3.25307	3.448634	3.438745	35.35801
3.236407	3.26721	3.393729	34.82638
3.189967	3.15938	3.381503	34.46228
3.305242	3.251777	3.398264	34.95308
3.400267	3.375512	3.353896	35.33445
3.325493	3.416824	3.495066	35.56999
3.214631	3.372007	3.370811	34.95782
3.276908	3.341208	3.4744	35.25319
3.217023	3.349565	3.332309	34.82978
3.350693	3.342219	3.442957	35.34799
3.093169	3.348352	3.405716	34.7168
3.14596	3.40193	3.472899	35.09634
3.246842	3.339523	3.427968	35.08222
3.324421	3.264514	3.380906	34.98492
3.269608	3.203455	3.350554	34.66515
3.281239	3.266334	3.398077	34.93202
3.233768	3.319507	3.478901	35.12124
3.385378	3.445129	3.344757	35.43415
3.408475	3.450993	3.342216	35.49192
3.376305	3.382453	3.341517	35.27016
3.218301	3.358798	3.427406	35.06072
3.253317	3.425585	3.307124	35.02031
3.179656	3.413454	3.422921	35.08593
3.349785	3.391956	3.273072	35.08327
3.189184	3.195975	3.518478	34.84014
3.1705	3.240926	3.392177	34.62139
3.252451	3.320653	3.445156	35.0908

3.367273	3.394651	3.422427	35.45402
3.304871	3.348217	3.400413	35.16787
3.287673	3.297402	3.346121	34.90041
3.192648	3.302996	3.434721	34.89859
3.241521	3.360483	3.418829	35.09643
3.296499	3.398291	3.369908	35.19235
3.238428	3.370255	3.427866	35.1308
3.223209	3.401323	3.481306	35.28232
3.2574	3.207836	3.422375	34.8051
3.409795	3.270445	3.341943	35.09938
3.267752	3.277319	3.412946	34.95906
3.299716	3.335749	3.397139	35.12217
3.272784	3.433403	3.404778	35.29353
3.385502	3.422687	3.34271	35.38087
3.361663	3.361157	3.320884	35.14645
3.412063	3.395999	3.303134	35.29404
3.128886	3.369311	3.371084	34.76501
3.203165	3.410354	3.400464	35.08145
3.189967	3.36547	3.362166	34.87068
3.254719	3.219495	3.381042	34.73434
3.141052	3.169624	3.360274	34.33129
3.320461	3.307983	3.46335	35.25161
3.255668	3.425652	3.467647	35.37664
3.434211	3.46036	3.408188	35.71296
3.374243	3.36237	3.402868	35.35589
3.336051	3.288372	3.421693	35.15172
3.274557	3.326719	3.409553	35.07455
3.233768	3.383127	3.384453	35.05382
3.1665	3.404626	3.401487	34.99098
3.276702	3.377803	3.390728	35.14979
3.163283	3.224819	3.417311	34.62534
3.248368	3.186877	3.388255	34.66489
3.2826	3.259123	3.422665	34.97299
3.324008	3.359539	3.39794	35.22907
3.293364	3.409343	3.367572	35.20456
3.349538	3.461506	3.329666	35.35858
3.335928	3.326584	3.381741	35.14765
3.36022	3.378949	3.302196	35.14133
3.250925	3.384475	3.394291	35.1158
3.253441	3.438727	3.310654	35.05704
3.30615	3.365133	3.42836	35.26878
3.209888	3.223067	3.381452	34.64501
3.259504	3.166658	3.359915	34.58306
3.239542	3.25427	3.433067	34.89097
3.301407	3.471008	3.448618	35.53424
3.270928	3.43064	3.431106	35.34101
3.296829	3.344241	3.409467	35.16139
3.334195	3.31607	3.338107	35.02544
3.393338	3.369042	3.404966	35.41683
3.179533	3.369311	3.394684	34.92738

3.205928	3.347476	3.44357	35.04426
3.19504	3.336693	3.4279	34.9626
3.273568	3.276982	3.325574	34.77997
3.191823	3.236276	3.411872	34.70091
3.233644	3.268221	3.421966	34.8843
3.146373	3.296257	3.521479	34.97238
3.380718	3.429831	3.356215	35.41556
3.344589	3.381038	3.456939	35.45011
3.499375	3.452947	3.273259	35.54418
3.369211	3.347408	3.43218	35.37627
3.266226	3.4208	3.329478	35.08697
3.238923	3.421339	3.374887	35.12774
3.361705	3.432055	3.326324	35.31348
3.157632	3.242544	3.363923	34.53499
3.119358	3.165041	3.387727	34.33386
3.253936	3.223876	3.358296	34.69246
3.24445	3.389664	3.412059	35.15185
3.322194	3.433335	3.394002	35.37787
3.220694	3.384812	3.438864	35.1479
3.275052	3.349228	3.417362	35.14194
3.293447	3.366616	3.385322	35.15012
3.451492	3.419722	3.380002	35.60024
3.220281	3.35098	3.459906	35.11903
3.104263	3.312498	3.34624	34.5326
3.170335	3.242139	3.497453	34.8539
3.336216	3.250564	3.285724	34.77206
3.262927	3.26256	3.446998	34.9907
3.287838	3.314183	3.397258	35.0493
3.16683	3.334806	3.505911	35.06738
3.307717	3.422957	3.368203	35.2671
3.425055	3.431111	3.360922	35.52561
3.497767	3.425113	3.415828	35.79157
3.308459	3.363111	3.394206	35.19472
3.315842	3.401795	3.408376	35.32644
3.244408	3.372479	3.357528	34.99492
3.405588	3.367222	3.300951	35.21218
3.25208	3.201568	3.372789	34.67132
3.034438	3.235939	3.457143	34.455
3.17879	3.346937	3.392961	34.87306
3.31778	3.392764	3.392484	35.27618
3.339722	3.377938	3.421182	35.3545
3.317533	3.354889	3.450186	35.319
3.232984	3.322675	3.466232	35.09874
3.291096	3.434346	3.39453	35.31323
3.457637	3.394988	3.314763	35.41692
3.257689	3.333795	3.465055	35.17451
3.176439	3.283384	3.430219	34.81041
3.220735	3.287158	3.346735	34.73297
3.327349	3.249755	3.361194	34.91594
3.276455	3.318227	3.349378	34.92854

3.256905	3.312027	3.526595	35.25977
3.114863	3.399706	3.444338	34.96101
3.338155	3.381307	3.410166	35.33435
3.354528	3.437042	3.36515	35.3936
3.495457	3.413656	3.413764	35.75695
3.309078	3.381914	3.35567	35.15291
3.274475	3.369716	3.409007	35.16721
3.281899	3.333054	3.393985	35.07042
3.350074	3.313712	3.3009	34.97365
3.170088	3.210532	3.381656	34.53101
3.217765	3.319777	3.410849	34.93801
3.140475	3.425585	3.467391	35.12403
3.328504	3.453553	3.378161	35.40124
3.307676	3.368637	3.416936	35.25479
3.358818	3.338243	3.42067	35.30833
3.243914	3.348621	3.466778	35.18058
3.400226	3.436368	3.342847	35.44328
3.290271	3.304546	3.408939	35.05909
3.335226	3.264649	3.304703	34.8422
3.107975	3.170433	3.433834	34.42159
3.16254	3.250698	3.363854	34.56341
3.222632	3.271119	3.424558	34.87223
3.209558	3.413387	3.370112	35.03569
3.272248	3.383666	3.418471	35.21354
3.269072	3.437244	3.425428	35.33897
3.289941	3.454699	3.445702	35.46712
3.381419	3.407658	3.387454	35.43692
3.435159	3.425518	3.379917	35.57701
3.233396	3.345184	3.422307	35.05281
3.292333	3.301042	3.370624	34.97214
3.28029	3.256562	3.363104	34.83209
3.322359	3.239174	3.256208	34.6523
3.255255	3.264447	3.356215	34.77952
3.197515	3.390069	3.470376	35.17762
3.20168	3.453082	3.444201	35.26729
3.309449	3.368772	3.372448	35.16168
3.365375	3.330088	3.429725	35.32464
3.357457	3.433066	3.384862	35.43441
3.266185	3.411837	3.518921	35.48156
3.383687	3.381173	3.385612	35.37993
3.238964	3.233378	3.43409	34.84625
3.242429	3.206421	3.322914	34.55175
3.172727	3.225695	3.366975	34.53783
3.204114	3.275904	3.361655	34.70463
3.082363	3.34168	3.392228	34.64909
3.222756	3.375916	3.368748	34.97962
3.310687	3.400582	3.432998	35.36636
3.312419	3.471952	3.412196	35.48073
3.307965	3.474446	3.397258	35.44378
3.347352	3.434616	3.425717	35.50505

3.376717	3.396134	3.376933	35.37843
3.392926	3.362842	3.361672	35.30769
3.254554	3.292011	3.353334	34.83197
3.220776	3.214441	3.331388	34.54047
3.200938	3.268558	3.358125	34.67391
3.225601	3.34633	3.425632	35.04554
3.289735	3.436301	3.433476	35.3997
3.222673	3.362572	3.461986	35.15416
3.248657	3.330762	3.457962	35.13262
3.396844	3.365605	3.42009	35.45006
3.351064	3.429629	3.473854	35.60753
3.240367	3.475187	3.433152	35.37607
3.327143	3.319575	3.415691	35.18735
3.222426	3.209319	3.428736	34.74576
3.338237	3.209993	3.362746	34.85619
3.249811	3.219832	3.351356	34.65943
3.265773	3.326112	3.422085	35.08142
3.104181	3.350509	3.36203	34.65007
3.242553	3.348554	3.399458	35.03024
3.230221	3.43684	3.355414	35.10002
3.36253	3.467639	3.383413	35.51794
3.266432	3.43839	3.490786	35.47864
3.348837	3.414128	3.40355	35.41501
3.336216	3.34168	3.409194	35.24133
3.37383	3.291337	3.318634	35.01545
3.281486	3.257573	3.382799	34.87999
3.220446	3.286148	3.308846	34.64727
3.088137	3.314588	3.408871	34.63886
3.277733	3.419654	3.364758	35.18678
3.24214	3.453419	3.459309	35.38954
3.300541	3.375512	3.34728	35.1019
3.167201	3.35772	3.523713	35.15723
3.311347	3.421205	3.429673	35.40563
3.418002	3.452475	3.445872	35.74268
3.355023	3.377331	3.36781	35.26992
3.28128	3.201097	3.397463	34.7881
3.256946	3.174072	3.360922	34.59588
3.262432	3.231559	3.375245	34.76491
3.243955	3.315396	3.349702	34.85199
3.317079	3.386834	3.412468	35.30538
3.110821	3.38899	3.440638	34.92064
3.284002	3.380431	3.364877	35.11497
3.30681	3.419183	3.399577	35.32547
3.301654	3.494057	3.369703	35.41261
3.254266	3.416487	3.385885	35.17473
3.308501	3.346465	3.442206	35.26337
3.239872	3.329616	3.440382	35.07246
3.406289	3.26566	3.263813	34.91039
3.279589	3.22738	3.380752	34.80534
3.318317	3.295246	3.353845	34.9796

3.134165	3.34943	3.437773	34.87892
3.24944	3.424642	3.3526	35.10922
3.184606	3.39728	3.41697	35.04837
3.344877	3.355496	3.397395	35.26468
3.231912	3.395864	3.474076	35.27361
3.364674	3.422283	3.404386	35.46931
3.301984	3.459686	3.444133	35.50093
3.379068	3.320249	3.393439	35.25371
3.293282	3.242948	3.37047	34.84684
3.196607	3.168748	3.421813	34.58544
3.247708	3.237018	3.369157	34.73134
3.203825	3.351182	3.398895	34.95007
3.321987	3.405097	3.398009	35.32443
3.284085	3.348891	3.397753	35.11807
3.196277	3.337367	3.421131	34.95197
3.222013	3.455441	3.439683	35.30703
3.348094	3.475389	3.348917	35.42789
3.282724	3.374972	3.420125	35.22105
3.326648	3.344847	3.333554	35.06191
3.309862	3.282508	3.414122	35.06507
3.289158	3.286417	3.375995	34.94496
3.288168	3.326719	3.354374	34.98365
3.395318	3.361224	3.397736	35.38825
3.148476	3.353069	3.425956	34.89233
3.299138	3.352193	3.361791	35.07957
3.183162	3.379151	3.42038	35.01303
3.329617	3.400245	3.436289	35.41422
3.285363	3.425518	3.422631	35.34284
3.279342	3.360415	3.462753	35.27505
3.198587	3.336423	3.458439	35.03655
3.360014	3.283587	3.382679	35.10834
3.370366	3.175689	3.365679	34.85785
3.317203	3.273949	3.386345	35.00166
3.200855	3.27368	3.406534	34.79079
3.205681	3.326584	3.349804	34.79298
3.347228	3.402671	3.368271	35.30929
3.319348	3.373759	3.414174	35.28548
3.202588	3.446208	3.398571	35.15446
3.275176	3.452273	3.435931	35.40816
3.260328	3.408737	3.415947	35.23678
3.418868	3.334604	3.339027	35.25315
3.330071	3.195705	3.391597	34.87018
3.220075	3.251979	3.3991	34.76911
3.261648	3.310948	3.368851	34.92283
3.260741	3.377196	3.366344	35.06023
3.41429	3.368637	3.415128	35.48399
3.243625	3.393169	3.40367	35.13936
3.139774	3.405839	3.436409	35.01156
3.212734	3.380566	3.408649	35.05513
3.353126	3.461169	3.387727	35.49266

3.187534	3.385553	3.417226	35.02969
3.333371	3.310005	3.42761	35.20611
3.207578	3.264447	3.402476	34.77643
3.367561	3.204669	3.339249	34.85729
3.364056	3.184518	3.344535	34.81712
3.29687	3.277858	3.382526	34.95739
3.246058	3.333795	3.416782	35.04351
3.218343	3.448836	3.354169	35.09756
3.208857	3.406715	3.383225	35.04824
3.372098	3.40348	3.301667	35.2198
3.178996	3.434481	3.51621	35.33448
3.189184	3.453621	3.371135	35.08136
3.226962	3.338041	3.492202	35.17597
3.379522	3.31816	3.338737	35.13051
3.289116	3.236748	3.383276	34.85218
3.355394	3.281632	3.343989	35.00936
3.238098	3.346128	3.426093	35.07344
3.314027	3.410758	3.362678	35.24214
3.373954	3.374029	3.34549	35.25528
3.228447	3.354215	3.37238	34.95256
3.170047	3.368637	3.398503	34.91351
3.241356	3.476535	3.458439	35.43648
3.332174	3.489879	3.396099	35.52794
3.268	3.309331	3.433698	35.07499
3.354446	3.276915	3.416629	35.15582
3.175243	3.224145	3.396986	34.60558
3.328586	3.247261	3.378195	34.95037
3.292828	3.249822	3.426724	34.9839
3.377089	3.326719	3.354561	35.17851
3.213682	3.374568	3.41003	35.04711
3.283837	3.381307	3.391955	35.17574
3.285817	3.367492	3.395263	35.15709
3.327638	3.429224	3.389756	35.3715
3.195205	3.482398	3.457979	35.34737
3.294024	3.433201	3.385067	35.29644
3.207578	3.2848	3.467033	34.96211
3.375645	3.238365	3.35717	34.98785
3.400226	3.288439	3.371681	35.18284
3.363148	3.359876	3.384112	35.28516
3.286394	3.399706	3.401214	35.24182
3.260823	3.315464	3.444457	35.09624
3.248162	3.284058	3.28349	34.64786
3.376264	3.366346	3.410763	35.38627
3.229107	3.507603	3.36174	35.26617
3.163901	3.429359	3.416782	35.07283
3.265649	3.305961	3.492884	35.19191
3.27864	3.291202	3.413764	35.01502
3.321822	3.28453	3.459139	35.19409
3.274062	3.276038	3.434772	35.01779
3.187245	3.246655	3.482738	34.86857

3.340258	3.319777	3.361041	35.09696
3.364427	3.342286	3.407916	35.30155
3.314069	3.410287	3.326256	35.16155
3.175243	3.345117	3.388272	34.85107
3.232407	3.364459	3.402715	35.04996
3.306232	3.417161	3.359745	35.23268
3.322688	3.402671	3.318003	35.1457
3.371314	3.284261	3.439035	35.25777
3.14394	3.239578	3.417038	34.61472
3.257895	3.25272	3.402374	34.86059
3.239748	3.385418	3.36926	35.03868
3.437799	3.453958	3.404386	35.69849
3.222508	3.389529	3.427849	35.1381
3.267257	3.29255	3.436272	35.0423
3.263463	3.40348	3.364605	35.11986
3.40897	3.482735	3.336299	35.54948
3.175738	3.445062	3.448771	35.20301
3.195123	3.353272	3.400362	34.93881
3.107563	3.263301	3.494998	34.75753
3.320214	3.303063	3.372704	35.04208
3.336216	3.276915	3.408461	35.09809
3.369871	3.26411	3.379133	35.07955
3.215167	3.284261	3.47012	34.98428
3.308459	3.362302	3.39112	35.1862
3.385378	3.423024	3.371238	35.44372
3.426209	3.412848	3.313177	35.38378
3.169057	3.351654	3.392007	34.86
3.132268	3.4115	3.399305	34.92638
3.332381	3.462854	3.400549	35.47902
3.25043	3.383666	3.319111	34.94854
3.329411	3.19611	3.383532	34.85198
3.129009	3.143205	3.514164	34.58372
3.185925	3.363313	3.399987	34.93984
3.422333	3.358394	3.397224	35.44002
3.410331	3.43374	3.318855	35.40716
3.282229	3.314183	3.425445	35.09867
3.251709	3.322271	3.412332	35.02094
3.247914	3.423631	3.434107	35.28192
3.387606	3.517241	3.283985	35.46382
3.259174	3.437042	3.351441	35.15509
3.18675	3.266536	3.431293	34.79847
3.159612	3.295852	3.362064	34.65184
3.329741	3.211341	3.395707	34.91264
3.279919	3.240185	3.370931	34.81258
3.351146	3.228795	3.399731	35.00642
3.198628	3.301109	3.41622	34.86708
3.37647	3.385688	3.39644	35.3977
3.334195	3.344645	3.3873	35.19551
3.393957	3.355226	3.333844	35.23244
3.20333	3.336895	3.378809	34.87381

3.179656	3.478287	3.426451	35.23543
3.189101	3.425922	3.364264	35.00558
3.360632	3.27341	3.337442	34.98851
3.2355	3.233243	3.411036	34.78797
3.240284	3.301042	3.413014	34.95102
3.261236	3.448027	3.448686	35.39628
3.377212	3.389866	3.32438	35.25088
3.454502	3.302659	3.362968	35.31358
3.257194	3.288102	3.338891	34.79761
3.229973	3.412106	3.437654	35.22524
3.309078	3.518049	3.357255	35.45409
3.302562	3.401863	3.390165	35.25772
3.202299	3.2972	3.350401	34.72263
3.262803	3.258516	3.442462	34.97167
3.160478	3.262964	3.438404	34.74875
3.299963	3.266064	3.404198	34.98576
3.24779	3.223	3.426587	34.82645
3.237727	3.275769	3.393865	34.84829
3.300623	3.368233	3.373454	35.1434
3.324916	3.349565	3.445821	35.31395
3.30813	3.381847	3.356266	35.152
3.385378	3.340939	3.331388	35.17706
3.25142	3.383531	3.372789	35.0678
3.362695	3.462652	3.331371	35.39359
3.196855	3.287158	3.37134	34.73455
3.266969	3.24005	3.323408	34.68004
3.206712	3.260875	3.416544	34.79748
3.284415	3.370592	3.391683	35.15297
3.298561	3.381779	3.414719	35.25875
3.436603	3.3683	3.376387	35.44733
3.318069	3.263705	3.386294	34.98104
3.3662	3.394921	3.403909	35.41177
3.286889	3.493585	3.43102	35.51338
3.216775	3.398897	3.378433	35.03798
3.149714	3.320316	3.408547	34.78534
3.181471	3.207634	3.37944	34.54472
3.269567	3.333391	3.479873	35.23201
3.236778	3.376859	3.430543	35.14749
3.223952	3.290259	3.509202	35.10207
3.296128	3.289585	3.412127	35.04615
3.376181	3.353474	3.438881	35.41943
3.311718	3.429359	3.363923	35.28049
3.367644	3.374231	3.424626	35.41498
3.270598	3.367829	3.416629	35.17127
3.303716	3.368233	3.354629	35.109
3.352549	3.442568	3.400959	35.47966
3.345414	3.392225	3.37163	35.28983
3.134247	3.223269	3.354152	34.42034
3.280909	3.233176	3.402101	34.86758
3.209269	3.343365	3.43779	35.02993

3.317409	3.478422	3.376455	35.42764
3.225643	3.400784	3.427832	35.16953
3.298809	3.300839	3.436954	35.13092
3.26668	3.307781	3.392552	34.97874
3.351518	3.486172	3.395656	35.56116
3.306356	3.47795	3.461236	35.58783
3.324998	3.315194	3.293738	34.90639
3.114492	3.170298	3.413645	34.3914
3.17413	3.27678	3.3378	34.58881
3.253276	3.370929	3.429878	35.16914
3.220529	3.424035	3.376677	35.09732
3.19438	3.279273	3.450204	34.88436
3.254101	3.337569	3.457075	35.15747
3.383316	3.342017	3.419562	35.36773
3.504036	3.402334	3.414463	35.75248
3.332381	3.382386	3.431788	35.37136
3.217724	3.351991	3.438506	35.06885
3.256823	3.401795	3.351117	35.07216
3.293199	3.352733	3.381025	35.10982
3.375892	3.291337	3.369174	35.13048
3.213022	3.196447	3.440638	34.72308
3.179368	3.303131	3.379252	34.74854
3.300252	3.395662	3.400669	35.26208
3.365912	3.420328	3.375245	35.40401
3.27695	3.348419	3.39719	35.10021
3.303057	3.30832	3.387795	35.04906
3.236737	3.399841	3.355107	35.03269
3.405299	3.489272	3.367384	35.62373
3.364674	3.382116	3.374341	35.31577
3.23583	3.169893	3.357665	34.53344
3.142455	3.173802	3.328728	34.27451
3.201144	3.301379	3.414088	34.86851
3.27101	3.441355	3.404761	35.30701
3.301407	3.325708	3.370675	35.04604
3.17149	3.275297	3.442292	34.80831
3.257895	3.371266	3.444866	35.21276
3.339516	3.420733	3.382287	35.36258
3.33931	3.41123	3.357204	35.28649
3.287467	3.377601	3.375739	35.14011
3.256864	3.342556	3.43588	35.12807
3.253152	3.370861	3.414191	35.13442
3.332999	3.375175	3.344006	35.16498
3.330483	3.278869	3.373386	35.01312
3.233438	3.244835	3.378314	34.73725
3.124473	3.354822	3.449368	34.89487
3.305407	3.406108	3.406824	35.30966
3.377831	3.446949	3.430765	35.60971
3.310769	3.351654	3.360154	35.10025
3.296705	3.344982	3.423467	35.19335
3.269361	3.52243	3.390455	35.44941

3.327844	3.477815	3.455097	35.6211
3.27398	3.311151	3.311336	34.82446
3.299138	3.18964	3.424677	34.86161
3.060463	3.242679	3.386431	34.37202
3.263174	3.405704	3.425649	35.25759
3.190421	3.313442	3.438523	34.92488
3.253235	3.326382	3.411462	35.03136
3.194793	3.291943	3.46601	34.94754
3.285652	3.401189	3.411309	35.26551
3.300087	3.424776	3.432197	35.39434
3.414703	3.404356	3.394257	35.51736
3.241975	3.387508	3.429264	35.17934
3.384595	3.349498	3.361945	35.26089
3.314976	3.3528	3.354561	35.09972
3.311841	3.283047	3.342352	34.91363
3.213228	3.274219	3.420329	34.84919
3.254183	3.389125	3.342847	35.0206
3.227416	3.39236	3.406347	35.108
3.342362	3.397212	3.352532	35.25229
3.302026	3.364055	3.476599	35.36289
3.379728	3.350037	3.31096	35.13993
3.291261	3.407321	3.45005	35.37591
3.273444	3.497427	3.424592	35.47832
3.295303	3.423698	3.383157	35.27427
3.211414	3.173128	3.371988	34.51844
3.247873	3.172859	3.334935	34.51655
3.227251	3.298211	3.382952	34.85059
3.150373	3.41925	3.441865	35.076
3.19603	3.349093	3.436494	35.01067
3.269402	3.286484	3.346718	34.83788
3.25076	3.283519	3.445992	35.00773
3.34463	3.3652	3.35833	35.19993
3.289158	3.427472	3.46971	35.45837
3.34232	3.467639	3.359336	35.42109
3.312419	3.345319	3.402135	35.18181
3.403979	3.29747	3.310159	35.07626
3.320214	3.194559	3.398759	34.86178
3.308459	3.227919	3.297558	34.68772
3.1015	3.339658	3.410149	34.72571
3.285281	3.409882	3.372107	35.19798
3.206093	3.428955	3.384879	35.09445
3.252987	3.411702	3.422307	35.24112
3.24977	3.347274	3.412673	35.07212
3.390781	3.390608	3.370266	35.38252
3.326194	3.482061	3.397889	35.50168
3.324586	3.436099	3.416373	35.43807
3.217806	3.228459	3.378706	34.66811
3.174542	3.160593	3.343887	34.34895
3.300335	3.263031	3.364042	34.89213
3.266804	3.408804	3.381929	35.1767

3.266391	3.43967	3.412315	35.30974
3.110862	3.2323	3.462071	34.62495
3.246801	3.312229	3.408785	34.98049
3.299633	3.373759	3.421267	35.25788
3.357044	3.43374	3.410917	35.49196
3.2103	3.348285	3.445429	35.05965
3.2355	3.380027	3.345149	34.96488
3.339062	3.326853	3.407251	35.21087
3.427735	3.349902	3.376694	35.38837
3.22189	3.249957	3.391137	34.75124
3.257565	3.222932	3.35966	34.70132
3.16551	3.333323	3.402732	34.83561
3.25872	3.371738	3.38169	35.07744
3.258225	3.431246	3.34624	35.12897
3.284085	3.410489	3.44502	35.35614
3.145218	3.303805	3.431907	34.79049
3.355271	3.460091	3.389483	35.49883
3.303593	3.497494	3.472763	35.64974
3.395194	3.421676	3.396849	35.51824
3.229973	3.34296	3.12	34.37937
3.195659	3.173128	3.326665	34.38487
3.258885	3.252922	3.339471	34.72564
3.311511	3.412039	3.358449	35.23019
3.18708	3.409141	3.396986	35.03602
3.159323	3.253664	3.445736	34.74192
3.22457	3.3065	3.440297	34.98826
3.324462	3.337367	3.40517	35.19739
3.357085	3.366683	3.394956	35.3105
3.177264	3.370524	3.471364	35.09276
3.245522	3.41123	3.397531	35.16958
3.31811	3.338445	3.289083	34.93199
3.422827	3.270714	3.391052	35.23586
3.242676	3.277723	3.350537	34.76863
3.19071	3.285271	3.414719	34.81185
3.199701	3.37841	3.392859	34.98739
3.385048	3.39418	3.32866	35.28681
3.305036	3.426326	3.391188	35.31887
3.327596	3.361898	3.355397	35.14904
3.170624	3.35125	3.432862	34.95189
3.295509	3.491968	3.389756	35.43845
3.366489	3.421407	3.409757	35.48311
3.352425	3.261212	3.368134	35.01101
3.214012	3.165378	3.377138	34.51844
3.209599	3.291674	3.390301	34.81376
3.233603	3.407254	3.355925	35.04384
3.356797	3.436301	3.364946	35.39649
3.168108	3.244835	3.404505	34.65166
3.163324	3.277521	3.453682	34.82022
3.178048	3.397212	3.490906	35.19557
3.332298	3.386834	3.370794	35.24753

3.32805	3.399436	3.477622	35.49941
3.297241	3.398628	3.404898	35.27123
3.228942	3.305826	3.45196	35.02185
3.3662	3.221584	3.296398	34.7976
3.467783	3.255416	3.376797	35.26955
3.228241	3.268153	3.431703	34.89363
3.197803	3.376388	3.428258	35.05623
3.267092	3.423563	3.393098	35.23403
3.399937	3.412039	3.28939	35.27254
3.365582	3.387036	3.409041	35.40439
3.269814	3.368166	3.317611	34.95376
3.16287	3.393843	3.426058	35.0132
3.301036	3.449779	3.434022	35.45508
3.347558	3.405771	3.351424	35.27995
3.412434	3.199277	3.408325	35.09469
3.206465	3.158706	3.369089	34.46974
3.268536	3.311285	3.406603	35.02118
3.218673	3.422081	3.378945	35.09395
3.314357	3.410624	3.44814	35.42946
3.141135	3.340265	3.433698	34.8652
3.151982	3.257303	3.358006	34.54197
3.174996	3.306366	3.459667	34.92191
3.310068	3.419115	3.327841	35.17558
3.316461	3.421609	3.501068	35.57383
3.372964	3.44277	3.396031	35.51397
3.230963	3.213834	3.407626	34.72814
3.32075	3.203523	3.321294	34.71315
3.388389	3.224954	3.310943	34.8853
3.328339	3.251372	3.348917	34.89479
3.135897	3.431179	3.39141	34.96009
3.272	3.410691	3.371152	35.16862
3.258596	3.394045	3.346922	35.04992
3.312213	3.394719	3.342386	35.15872
3.293694	3.38926	3.450476	35.34266
3.162911	3.455508	3.493293	35.29517
3.248409	3.389327	3.434652	35.20918
3.321946	3.256966	3.321567	34.83324
3.334649	3.209117	3.370436	34.86325
3.193308	3.276173	3.425581	34.82139
3.327555	3.375175	3.386618	35.24626
3.256864	3.413993	3.410149	35.22802
3.359973	3.375175	3.323272	35.17862
3.160973	3.243218	3.427286	34.68234
3.253606	3.284665	3.377496	34.86667
3.152106	3.346869	3.483198	35.01189
3.237603	3.435222	3.385049	35.17744
3.360426	3.415409	3.457416	35.56096
3.33502	3.321057	3.333162	35.02734
3.26602	3.124066	3.443025	34.68591
3.324421	3.244566	3.37354	34.92519

3.208609	3.338243	3.404915	34.94539
3.358488	3.44796	3.329598	35.34838
3.212898	3.434885	3.362882	35.0742
3.218838	3.315733	3.330911	34.7567
3.200979	3.359607	3.36972	34.89846
3.26569	3.445399	3.396798	35.2868
3.227994	3.445264	3.4257	35.26728
3.274145	3.402132	3.413509	35.24722
3.191988	3.24962	3.452591	34.8195
3.257648	3.152978	3.404898	34.64745
3.414373	3.257033	3.357068	35.11314
3.311924	3.456451	3.382253	35.38027
3.276743	3.436233	3.409808	35.31938
3.236119	3.359068	3.420824	35.08588
3.282187	3.254809	3.317952	34.73367
3.36055	3.24187	3.366566	34.98305
3.285528	3.402065	3.395775	35.23318
3.111605	3.407928	3.474775	35.03842
3.251502	3.391349	3.390301	35.12337
3.335639	3.319373	3.419954	35.21481
3.351229	3.234726	3.362269	34.93765
3.242553	3.188157	3.38568	34.64935
3.310728	3.267816	3.447458	35.10774
3.238717	3.40038	3.422904	35.18646
3.425385	3.461573	3.374068	35.62169
3.257565	3.426192	3.353095	35.13146
3.31048	3.361561	3.393575	35.19437
3.110697	3.386497	3.387846	34.79947
3.259215	3.459484	3.364673	35.23319
3.252286	3.460832	3.449914	35.4074
3.251379	3.279273	3.385254	34.86697
3.208444	3.148327	3.403585	34.52681
3.328504	3.159447	3.406603	34.82028
3.305572	3.389125	3.416629	35.29433
3.425096	3.482937	3.352652	35.62095
3.258307	3.37942	3.436545	35.2133
3.13367	3.348689	3.390591	34.77303
3.233149	3.25636	3.352396	34.70514
3.315182	3.327729	3.393439	35.13036
3.192937	3.398223	3.455779	35.15352
3.223869	3.444321	3.409945	35.22174
3.146373	3.347543	3.537712	35.12004
3.409588	3.263503	3.361791	35.12716
3.429591	3.21653	3.371425	35.08924
3.251832	3.290056	3.364826	34.84687
3.263628	3.406445	3.472302	35.36222
3.243377	3.432931	3.372278	35.15712
3.370696	3.389193	3.315343	35.21539
3.340836	3.373827	3.331388	35.15158
3.215043	3.402536	3.397753	35.0844

3.145837	3.459686	3.42865	35.1256
3.3045	3.448297	3.393592	35.371
3.240078	3.339591	3.397071	35.00001
3.23187	3.223	3.417652	34.7721
3.130948	3.1612	3.461884	34.51298
3.320874	3.321529	3.385339	35.11154
3.326235	3.451329	3.416834	35.47599
3.392472	3.433133	3.360973	35.45889
3.27563	3.31944	3.462003	35.17568
3.275259	3.310611	3.322232	34.84991
3.229767	3.254068	3.385663	34.76549
3.313574	3.379825	3.317832	35.07543
3.144476	3.407456	3.435045	35.02239
3.211001	3.409074	3.410712	35.1182
3.220116	3.258651	3.412485	34.81306
3.364427	3.226504	3.401266	35.03381
3.353291	3.241466	3.39823	35.03554
3.397586	3.312633	3.382935	35.25459
3.292457	3.365133	3.394837	35.16552
3.295097	3.436031	3.334116	35.19355
3.332257	3.400245	3.349599	35.23041
3.41363	3.415678	3.299877	35.33338
3.260988	3.41588	3.377444	35.16965
3.097417	3.477478	3.471484	35.1523
3.252905	3.348217	3.446998	35.1561
3.302562	3.24524	3.434635	35.01246
3.222756	3.186944	3.407046	34.65012
3.199041	3.271388	3.437756	34.85009
3.255915	3.406041	3.406875	35.2014
3.230798	3.394988	3.41813	35.14691
3.439779	3.385621	3.343273	35.41973
3.274557	3.325101	3.403346	35.05744
3.274104	3.389597	3.427423	35.25014
3.190256	3.320518	3.392978	34.84039
3.275052	3.360146	3.42345	35.17913
3.135237	3.43994	3.388409	34.97124
3.299386	3.367896	3.459326	35.32774
3.206217	3.293763	3.428582	34.89465
3.408351	3.214036	3.332616	34.95247
3.249481	3.258247	3.406552	34.86342
3.410619	3.388316	3.465345	35.62881
3.311099	3.394517	3.405119	35.29303
3.318894	3.380701	3.372227	35.20793
3.304418	3.36675	3.375927	35.15386
3.326318	3.42909	3.40546	35.40266
3.263545	3.457799	3.40928	35.33653
3.223993	3.444118	3.41385	35.23011
3.213517	3.331369	3.42079	34.97581
3.287962	3.211543	3.420261	34.87541
3.295798	3.233243	3.433766	34.96954

3.269072	3.337569	3.419016	35.10698
3.335103	3.431516	3.451482	35.52782
3.36851	3.403952	3.402937	35.43444
3.301984	3.361696	3.426468	35.24801
3.393173	3.308387	3.321021	35.10026
3.316337	3.2292	3.400942	34.93383
3.152436	3.328538	3.415691	34.8249
3.242924	3.446612	3.505382	35.47713
3.174295	3.429494	3.367197	34.98742
3.306975	3.266469	3.378706	34.94623
3.209228	3.20878	3.375296	34.59886
3.243377	3.228189	3.433493	34.84325
3.172521	3.395056	3.394257	34.96741
3.454461	3.453014	3.418743	35.76426
3.349538	3.4208	3.430253	35.48953
3.423075	3.409141	3.369839	35.49274
3.249358	3.327392	3.379184	34.95451
3.301407	3.412376	3.365764	35.22483
3.227705	3.457732	3.376524	35.18637
3.264164	3.405839	3.331252	35.05362
3.16287	3.161132	3.412059	34.47368
3.236613	3.203118	3.371954	34.63906
3.266144	3.306905	3.46971	35.14438
3.448027	3.479837	3.334458	35.62453
3.370407	3.446679	3.467528	35.67328
3.331515	3.323955	3.4028	35.1783
3.265938	3.323955	3.434925	35.10515
3.325947	3.296661	3.405324	35.11195
3.328545	3.351654	3.448618	35.33257
3.146208	3.364863	3.430219	34.92248
3.129546	3.371872	3.5143	35.08525
3.210053	3.314722	3.366702	34.81355
3.349744	3.217136	3.359131	34.88907
3.25175	3.19429	3.3629	34.63305
3.295592	3.264716	3.4109	34.98791
3.180564	3.384475	3.394973	34.96343
3.458421	3.462247	3.320305	35.57784
3.457802	3.42909	3.346735	35.56178
3.382368	3.347611	3.305947	35.12944
3.241934	3.361965	3.471347	35.21542
3.288828	3.45416	3.33294	35.21691
3.259297	3.439266	3.495288	35.47479
3.27101	3.305422	3.317457	34.81883
3.146538	3.090301	3.46098	34.39005
3.220776	3.217406	3.429179	34.76081
3.180729	3.409141	3.404096	35.03768
3.328504	3.454362	3.424831	35.50507
3.266969	3.350037	3.375501	35.03448
3.386121	3.295785	3.40442	35.23965
3.199536	3.3466	3.434499	35.00852

3.351641	3.384879	3.367947	35.27933
3.160849	3.355698	3.412826	34.89642
3.190586	3.322136	3.365014	34.7835
3.126329	3.299761	3.402459	34.67594
3.211702	3.261751	3.413696	34.80409
3.357292	3.247598	3.362098	34.97868
3.305407	3.232772	3.388664	34.89089
3.307635	3.301109	3.360035	34.9826
3.219291	3.390406	3.421096	35.11821
3.371809	3.415678	3.285895	35.21135
3.464112	3.433066	3.376319	35.64897
3.40435	3.445534	3.345712	35.47861
3.187946	3.396673	3.457826	35.14369
3.299138	3.424979	3.360427	35.23576
3.262679	3.362572	3.342233	34.97977
3.254389	3.210464	3.391734	34.73725
3.12505	3.173465	3.400754	34.39322
3.23517	3.26694	3.506388	35.06946
3.209475	3.400312	3.370573	35.00792
3.406619	3.375377	3.41269	35.47662
3.223457	3.336828	3.460844	35.09708
3.405876	3.364998	3.376387	35.37291
3.241521	3.387305	3.453324	35.23052
3.313986	3.361359	3.356011	35.11944
3.192483	3.304209	3.362882	34.74378
3.230963	3.35927	3.336623	34.89092
3.198175	3.324023	3.381929	34.84121
3.232077	3.326988	3.434039	35.03579
3.213311	3.27988	3.436971	34.89815
3.304459	3.249014	3.406483	34.9633
3.30648	3.316609	3.378979	35.0554
3.235912	3.425855	3.450255	35.29585
3.344053	3.417767	3.321959	35.23408
3.444027	3.318429	3.309477	35.20818
3.333659	3.442029	3.388392	35.40969
3.244408	3.369177	3.358858	34.99061
3.257648	3.367559	3.417311	35.14385
3.235211	3.35408	3.350196	34.91854
3.296086	3.25609	3.381315	34.90543
3.133587	3.249148	3.392007	34.55827
3.28392	3.396741	3.461423	35.36159
3.194091	3.453756	3.453614	35.27275
3.410619	3.40375	3.40517	35.53097
3.387894	3.350711	3.438063	35.43722
3.331391	3.320384	3.394991	35.15314
3.278311	3.321664	3.411241	35.0754
3.314522	3.370053	3.410849	35.25955
3.223292	3.312094	3.387164	34.8815
3.295179	3.334267	3.360359	35.02858
3.173511	3.250025	3.38568	34.63366

3.115935	3.26566	3.43409	34.6478
3.167407	3.316475	3.408632	34.81582
3.278599	3.337097	3.445617	35.18495
3.324049	3.409478	3.440279	35.43096
3.360261	3.4239	3.419221	35.49564
3.282765	3.3652	3.404693	35.16603
3.396432	3.353204	3.357562	35.2853
3.351353	3.397078	3.401214	35.37812
3.275753	3.413656	3.332019	35.09773
3.215868	3.329684	3.420466	34.97656
3.182791	3.231491	3.390489	34.62394
3.220322	3.230143	3.408393	34.74222
3.296004	3.403952	3.391069	35.24993
3.27398	3.48725	3.482721	35.58435
3.256122	3.437986	3.419238	35.29874
3.311017	3.406378	3.473513	35.46836
3.406165	3.347611	3.382117	35.34805
3.345249	3.408669	3.409553	35.40836
3.214919	3.397347	3.450869	35.18894
3.262721	3.304007	3.464663	35.11952
3.187163	3.24187	3.386874	34.64829
3.32607	3.240118	3.352038	34.87205
3.241851	3.228391	3.358534	34.67643
3.227829	3.331436	3.44236	35.05442
3.083229	3.356641	3.450886	34.81197
3.366118	3.385351	3.350333	35.2735
3.351064	3.419385	3.436153	35.50268
3.437593	3.43064	3.344194	35.51541
3.308583	3.427742	3.480675	35.52542
3.371479	3.439805	3.383498	35.47683
3.293406	3.396336	3.416237	35.28263
3.414538	3.306905	3.292357	35.08105
3.137052	3.19247	3.420807	34.50488
3.170954	3.246385	3.341619	34.52375
3.206341	3.343702	3.438523	35.02587
3.290601	3.471008	3.422785	35.45411
3.325287	3.431583	3.428156	35.45549
3.235954	3.335749	3.505024	35.21866
3.21294	3.370322	3.46526	35.15698
3.38909	3.366481	3.399646	35.39031
3.413878	3.468245	3.377018	35.61757
3.256039	3.404423	3.433834	35.25708
3.238181	3.210262	3.350622	34.61146
3.165963	3.223	3.449385	34.69737
3.309161	3.228526	3.289714	34.67343
3.314564	3.273208	3.383583	34.98823
3.174047	3.323079	3.470785	34.9807
3.18143	3.360281	3.447117	35.02644
3.331762	3.42882	3.430355	35.46842
3.427076	3.466089	3.406074	35.70526

3.324173	3.463865	3.408393	35.48044
3.214012	3.413656	3.462873	35.24887
3.310274	3.381712	3.400055	35.25215
3.279713	3.316879	3.358568	34.95282
3.367025	3.289113	3.334372	35.03012
3.223251	3.235198	3.337646	34.60496
3.160066	3.242611	3.400191	34.61978
3.13136	3.384273	3.412809	34.89439
3.339145	3.466628	3.395366	35.49073
3.26602	3.380836	3.377291	35.10368
3.278723	3.335008	3.476241	35.24763
3.239294	3.429629	3.454142	35.32
3.361581	3.472154	3.479924	35.7368
3.395978	3.400717	3.395621	35.47144
3.372923	3.316677	3.367179	35.17504
3.183781	3.171915	3.445412	34.61593
3.205805	3.254405	3.390029	34.72337
3.206052	3.265188	3.387368	34.74167
3.309738	3.351856	3.326187	35.02415
3.224323	3.324158	3.47324	35.09837
3.189019	3.383734	3.403874	34.99976
3.283301	3.394517	3.46601	35.3654
3.451863	3.466493	3.377018	35.69681
3.313161	3.468111	3.465328	35.59015
3.24082	3.410017	3.398452	35.15866
3.342815	3.338108	3.431089	35.29582
3.24845	3.261616	3.363701	34.77482
3.347929	3.266132	3.344501	34.96025
3.296293	3.242544	3.365628	34.84195
3.248492	3.388586	3.412127	35.15848
3.20997	3.450184	3.432265	35.25298
3.299963	3.378881	3.421796	35.27096
3.276661	3.313172	3.379269	34.9833
3.257854	3.361426	3.402323	35.09811
3.198381	3.41642	3.519228	35.34397
3.310027	3.463797	3.363258	35.35065
3.317739	3.284463	3.418078	35.09522
3.345661	3.132827	3.342745	34.65994
3.21129	3.200692	3.456325	34.76288
3.212898	3.236344	3.345882	34.60284
3.155075	3.339928	3.420738	34.86661
3.350198	3.44924	3.331167	35.33648
3.186668	3.383262	3.387914	34.95868
3.319678	3.452206	3.373608	35.36904
3.252121	3.388316	3.397003	35.13275
3.325782	3.421205	3.416237	35.40781
3.402866	3.399032	3.385271	35.46018
3.390451	3.270917	3.382799	35.14746
3.251007	3.238635	3.380889	34.76775
3.238469	3.2261	3.366446	34.68133

3.26569	3.283384	3.343375	34.81568
3.261731	3.344713	3.377496	35.01575
3.204815	3.435492	3.405597	35.15126
3.125669	3.395999	3.492202	35.0812
3.272165	3.382116	3.441592	35.26053
3.33036	3.370322	3.403721	35.27918
3.314151	3.444927	3.44132	35.48911
3.262556	3.464134	3.477196	35.49674
3.25109	3.379083	3.465976	35.26114
3.232077	3.188696	3.32068	34.48547
3.365705	3.156347	3.338465	34.74585
3.233933	3.239039	3.361382	34.68863
3.271959	3.299828	3.379116	34.94351
3.227416	3.368435	3.383208	35.00508
3.312171	3.394921	3.380889	35.24327
3.307346	3.454969	3.327978	35.24833
3.313161	3.444253	3.415043	35.42801
3.15722	3.409815	3.432316	35.04945
3.293117	3.402739	3.406279	35.27422
3.288539	3.352126	3.363292	35.05952
3.374779	3.262021	3.421898	35.17923
3.275382	3.234591	3.368339	34.78476
3.316832	3.227448	3.374904	34.87414
3.258349	3.276915	3.432879	34.9812
3.327679	3.42727	3.353845	35.28879
3.169387	3.377668	3.423586	34.98667
3.256204	3.356439	3.406398	35.09251
3.168232	3.352733	3.472831	35.0373
3.274599	3.43967	3.393575	35.28671
3.337412	3.453553	3.511197	35.71165
3.378821	3.396538	3.371425	35.37187
3.297118	3.261279	3.419818	35.00323
3.285528	3.193953	3.347604	34.67274
3.290189	3.223943	3.412144	34.88965
3.322936	3.27988	3.313927	34.8688
3.199824	3.363246	3.397719	34.96512
3.124596	3.369244	3.387658	34.79173
3.246182	3.310409	3.38048	34.91326
3.260287	3.410422	3.36058	35.1193
3.321492	3.445197	3.399373	35.41402
3.269031	3.410556	3.408342	35.24316
3.285239	3.354013	3.426127	35.19385
3.199453	3.268288	3.389295	34.73823
3.461308	3.306635	3.339096	35.28495
3.252533	3.319575	3.408802	35.00913
3.307181	3.371401	3.324619	35.05787
3.297695	3.327056	3.44386	35.20091
3.339722	3.416352	3.393848	35.37873
3.198793	3.372546	3.410201	35.0105
3.280744	3.397482	3.35451	35.12246

3.150745	3.429966	3.476787	35.17661
3.27695	3.403682	3.404232	35.23646
3.19071	3.355698	3.490974	35.13262
3.353085	3.294774	3.414054	35.18627
3.333865	3.198536	3.407626	34.91972
3.29753	3.171376	3.436153	34.84325
3.269814	3.266401	3.428224	34.97311
3.315512	3.35563	3.402255	35.21138
3.273403	3.421474	3.381844	35.21865
3.268907	3.402806	3.36631	35.13402
3.199824	3.395393	3.362405	34.9582
3.254513	3.454227	3.452096	35.4026
3.324214	3.457597	3.410968	35.47245
3.301077	3.325303	3.421489	35.15555
3.299881	3.263436	3.401896	34.9748
3.24878	3.185798	3.411326	34.71389
3.272413	3.269703	3.379013	34.8784
3.382368	3.373759	3.347212	35.27686
3.226674	3.399504	3.399987	35.10809
3.250389	3.381307	3.410627	35.14343
3.305325	3.390203	3.441047	35.34954
3.243707	3.374164	3.373335	35.03164
3.262473	3.459215	3.373335	35.25867
3.17314	3.462112	3.452846	35.24353
3.244326	3.397684	3.495833	35.35231
3.172068	3.256427	3.378911	34.6297
3.393833	3.205882	3.389432	35.02714
3.262226	3.204264	3.400328	34.75962
3.388142	3.200288	3.399936	35.02543
3.199123	3.34633	3.422103	34.97992
3.377996	3.314049	3.349446	35.1416
3.34628	3.355496	3.398571	35.27031
3.338897	3.379825	3.373881	35.25338
3.208073	3.423092	3.415333	35.15256
3.270557	3.522902	3.433698	35.54763
3.283837	3.459147	3.423739	35.41547
3.392679	3.348824	3.360239	35.27337
3.277774	3.175352	3.407626	34.74636
3.246759	3.266603	3.38273	34.82364
3.271505	3.342623	3.462003	35.21736
3.308459	3.440075	3.352396	35.27159
3.296458	3.347745	3.378178	35.09982
3.28425	3.303872	3.392842	35.00924
3.230963	3.286619	3.473854	35.03215
3.277692	3.458945	3.379951	35.30583
3.287302	3.504099	3.436699	35.54969
3.251997	3.421137	3.376404	35.15921
3.288993	3.339389	3.476241	35.27966
3.163159	3.230817	3.413918	34.63077
3.36554	3.229806	3.371886	34.97921

3.364839	3.27651	3.380292	35.0982
3.318317	3.289315	3.360922	34.9821
3.274351	3.320586	3.426741	35.09828
3.357498	3.374231	3.405852	35.35174
3.277981	3.394584	3.33826	35.07454
3.326689	3.373894	3.256839	34.95776
3.129051	3.407995	3.463862	35.05286
3.261277	3.442636	3.387351	35.25045
3.219786	3.404356	3.378519	35.05669
3.288415	3.242476	3.326529	34.73907
3.220281	3.242005	3.400345	34.75047
3.376965	3.335951	3.36602	35.22349
3.224983	3.371468	3.469591	35.1953
3.37647	3.355496	3.318071	35.16029
3.383605	3.289787	3.355789	35.11469
3.294932	3.32254	3.347536	34.97435
3.218796	3.389664	3.422768	35.11916
3.240655	3.492237	3.45815	35.46865
3.195288	3.436975	3.398043	35.11714
3.282806	3.326988	3.39644	35.06451
3.287095	3.250429	3.399799	34.91381
3.148765	3.237489	3.477315	34.75252
3.304335	3.246385	3.342301	34.81693
3.253482	3.268558	3.396576	34.8729
3.315141	3.328066	3.344484	35.02395
3.325534	3.333795	3.38568	35.1493
3.294395	3.393236	3.384163	35.20788
3.25707	3.358394	3.340323	34.95419
3.372387	3.413522	3.356284	35.36182
3.190668	3.462517	3.474195	35.32943
3.293612	3.393438	3.343665	35.11804
3.193019	3.207769	3.389074	34.59133
3.315636	3.172117	3.350247	34.69661
3.304294	3.312566	3.36307	35.00698
3.345661	3.406378	3.380173	35.34
3.312708	3.430303	3.376473	35.31216
3.396762	3.333054	3.415128	35.36784
3.254719	3.337636	3.352532	34.93035
3.389338	3.451599	3.383276	35.54119
3.221683	3.419317	3.416493	35.1766
3.157179	3.428213	3.458388	35.14661
3.125669	3.24679	3.391683	34.53509
3.274681	3.268693	3.390131	34.90546
3.323555	3.224684	3.478714	35.10981
3.336835	3.217608	3.441473	35.04194
3.163365	3.232637	3.49672	34.81627
3.327555	3.322203	3.383907	35.12449
3.404928	3.384003	3.393848	35.45058
3.431736	3.423833	3.353794	35.50872
3.277981	3.368031	3.411036	35.17563

3.200237	3.340871	3.317184	34.74098
3.274764	3.467976	3.375739	35.30997
3.394617	3.488666	3.336163	35.53076
3.25542	3.360955	3.433135	35.15914
3.160643	3.229672	3.335123	34.45045
3.254224	3.223471	3.450067	34.8929
3.338897	3.380499	3.3622	35.22931
3.3297	3.437446	3.392143	35.39921
3.230303	3.307781	3.48949	35.11117
3.243914	3.279408	3.386431	34.85352
3.286518	3.373085	3.409297	35.20155
3.428148	3.479163	3.315138	35.53733
3.27563	3.470671	3.513073	35.61808
3.103397	3.397617	3.427354	34.89423
3.089251	3.230952	3.479788	34.61348
3.303675	3.361089	3.335822	35.05215
3.305242	3.239781	3.463146	35.06874
3.283878	3.27523	3.438779	35.04625
3.207413	3.217002	3.439018	34.75222
3.384719	3.307781	3.430662	35.32021
3.434582	3.362909	3.321174	35.31037
3.431695	3.383329	3.387215	35.49314
3.214053	3.360213	3.419664	35.0376
3.191122	3.370861	3.334389	34.82426
3.288663	3.441962	3.411326	35.3613
3.412599	3.42235	3.350247	35.45587
3.275176	3.26721	3.342898	34.80001
3.129587	3.189977	3.465055	34.57987
3.188647	3.368435	3.455592	35.07859
3.465556	3.422889	3.393763	35.66802
3.383605	3.38279	3.412468	35.44202
3.281775	3.274893	3.374068	34.8994
3.22391	3.335143	3.441951	35.05307
3.266886	3.403413	3.401504	35.20789
3.412599	3.467234	3.37076	35.59888
3.309903	3.344308	3.423245	35.22026
3.145053	3.279273	3.410064	34.68871
3.1811	3.29747	3.406074	34.79861
3.340217	3.298548	3.431907	35.20542
3.264288	3.33703	3.433152	35.12625
3.264948	3.259864	3.382389	34.84794
3.153302	3.298818	3.501068	34.9485
3.427777	3.367357	3.397344	35.47179
3.341289	3.398156	3.378519	35.30884
3.392225	3.386227	3.350111	35.33202
3.280125	3.32409	3.427747	35.12077
3.246388	3.376657	3.439018	35.18659
3.189802	3.394247	3.336077	34.8762
3.397133	3.26256	3.322777	35.01254
3.19405	3.239646	3.42038	34.73176

3.170789	3.397752	3.420875	35.02773
3.187451	3.469189	3.470376	35.32864
3.44877	3.397078	3.352379	35.48436
3.445965	3.327258	3.420159	35.47377
3.282022	3.360078	3.425138	35.19791
3.29122	3.406243	3.448993	35.37115
3.364303	3.471345	3.401231	35.56889
3.324462	3.383734	3.390165	35.26597
3.255132	3.246857	3.364519	34.75895
3.150332	3.270445	3.369362	34.59194
3.143486	3.286417	3.377751	34.63024
3.292045	3.323349	3.459394	35.21442
3.230179	3.278667	3.416441	34.88749
3.285074	3.323012	3.382134	35.02949
3.282146	3.360281	3.390983	35.12394
3.374985	3.380297	3.431276	35.45884
3.348919	3.374568	3.307022	35.11759
3.398329	3.348621	3.362558	35.29035
3.256452	3.372748	3.42449	35.16828
3.347558	3.434683	3.320748	35.27609
3.229808	3.312768	3.375023	34.87068
3.317904	3.219765	3.287549	34.66865
3.167613	3.244431	3.395553	34.63012
3.214796	3.458069	3.388409	35.18487
3.250966	3.40503	3.468739	35.32365
3.399071	3.374366	3.364451	35.35241
3.292045	3.291269	3.331456	34.86449
3.409795	3.395528	3.388681	35.47513
3.309326	3.489946	3.404846	35.49725
3.363231	3.426461	3.425223	35.52086
3.203577	3.335547	3.356232	34.82203
3.240408	3.175083	3.405511	34.65943
3.191039	3.30859	3.392024	34.81393
3.251915	3.397684	3.400123	35.15961
3.160396	3.277252	3.481152	34.8733
3.304129	3.222393	3.376797	34.83945
3.315471	3.281834	3.458593	35.17311
3.309532	3.34168	3.369771	35.09676
3.391111	3.404154	3.506201	35.71013
3.298561	3.452543	3.334099	35.2372
3.330978	3.419722	3.437381	35.46218
3.392637	3.41588	3.275254	35.23407
3.361911	3.276915	3.350759	35.02809
3.209682	3.242409	3.344688	34.60646
3.206382	3.28325	3.34092	34.68032
3.184441	3.439603	3.42906	35.167
3.363643	3.4671	3.332275	35.40737
3.277568	3.403076	3.460213	35.3589
3.26103	3.339523	3.406466	35.06622
3.272949	3.372479	3.41315	35.17897

3.391317	3.51933	3.370607	35.66593
3.281775	3.458473	3.440979	35.44719
3.336464	3.313172	3.320475	34.98551
3.168191	3.165378	3.40471	34.47853
3.335474	3.249283	3.325113	34.85377
3.214177	3.420935	3.395093	35.11693
3.243047	3.328606	3.393575	34.97483
3.207743	3.25454	3.430219	34.81579
3.213393	3.319912	3.436818	34.98554
3.348136	3.358933	3.388954	35.26086
3.494261	3.421811	3.395144	35.73145
3.224323	3.425585	3.449862	35.26905
3.279878	3.38097	3.432248	35.25446
3.34199	3.302794	3.375449	35.09512
3.347764	3.298615	3.33901	35.01892
3.23649	3.295852	3.320407	34.72886
3.140681	3.282239	3.453341	34.78027
3.126659	3.355833	3.325915	34.63189
3.241521	3.468717	3.395621	35.28237
3.368221	3.430437	3.361876	35.40194
3.309284	3.351115	3.352941	35.08005
3.308459	3.327662	3.45728	35.25513
3.185719	3.457799	3.366514	35.07281
3.473309	3.46373	3.363223	35.7075
3.358611	3.400178	3.308693	35.19844
3.261854	3.141521	3.400805	34.62265
3.201144	3.233648	3.331508	34.53981
3.290395	3.291269	3.444713	35.10856
3.253358	3.478894	3.326187	35.17867
3.262968	3.253731	3.394138	34.85589
3.147899	3.243487	3.445412	34.69398
3.26668	3.288911	3.438677	35.03834
3.310893	3.397145	3.40945	35.3078
3.376181	3.427337	3.371272	35.43311
3.188276	3.337636	3.372312	34.82831
3.386863	3.34323	3.414719	35.36755
3.293529	3.336288	3.400396	35.11694
3.393173	3.308657	3.285417	35.02299
3.169263	3.213969	3.305879	34.37101
3.272454	3.287698	3.34046	34.83353
3.128061	3.347206	3.403141	34.78497
3.299963	3.4053	3.322862	35.11238
3.278269	3.428753	3.431106	35.35294
3.380141	3.376994	3.308454	35.1943
3.270681	3.396269	3.465891	35.34137
3.382409	3.551409	3.322249	35.61085
3.363808	3.41487	3.473837	35.60308
3.370861	3.287832	3.324005	35.01304
3.234469	3.155134	3.350213	34.48189
3.156849	3.215317	3.401777	34.55652

3.254926	3.404895	3.47544	35.34667
3.229685	3.420665	3.381315	35.12012
3.254513	3.267749	3.360342	34.79415
3.246966	3.254944	3.462958	34.97404
3.224942	3.368974	3.411786	35.06334
3.229891	3.400515	3.438199	35.2009
3.39338	3.469256	3.346735	35.50873
3.261195	3.407187	3.455319	35.32138
3.376057	3.351856	3.306953	35.12712
3.349332	3.214912	3.406193	34.98622
3.400144	3.272804	3.331115	35.05976
3.20597	3.354417	3.415009	34.99707
3.268288	3.421878	3.383413	35.21178
3.173181	3.371198	3.356369	34.83382
3.290972	3.34296	3.353061	35.02243
3.281033	3.312903	3.3873	35.00984
3.325246	3.340939	3.380548	35.15307
3.309491	3.490553	3.406893	35.50341
3.342238	3.475322	3.417055	35.56394
3.297571	3.31971	3.422409	35.13767
3.323101	3.191729	3.345081	34.74452
3.27897	3.223269	3.38551	34.8054
3.310728	3.358124	3.390642	35.18098
3.1486	3.429831	3.432947	35.07575
3.163778	3.348554	3.399032	34.85704
3.269608	3.274825	3.366225	34.8555
3.273939	3.328403	3.457689	35.18215
3.277486	3.423833	3.472865	35.43178
3.277073	3.407793	3.407728	35.25336
3.232778	3.435425	3.339164	35.06698
3.412847	3.342421	3.406398	35.40441
3.348094	3.306298	3.289885	34.92902
3.315553	3.226032	3.376251	34.87119
3.338691	3.256831	3.417959	35.08035
3.117131	3.40321	3.375057	34.82213
3.356261	3.401728	3.343563	35.27295
3.243047	3.400717	3.371204	35.0836
3.243996	3.362235	3.431157	35.13263
3.249688	3.365335	3.414736	35.11595
3.310728	3.385688	3.407779	35.27873
3.283136	3.45571	3.438847	35.43946
3.411444	3.370457	3.384589	35.41496
3.237851	3.182631	3.47324	34.81846
3.324668	3.177846	3.342165	34.71121
3.398659	3.217069	3.370658	35.0211
3.299345	3.450319	3.401845	35.3822
3.231087	3.421744	3.420653	35.21157
3.22886	3.290326	3.351714	34.76855
3.220817	3.331773	3.398554	34.94403
3.391235	3.394584	3.386806	35.42838

3.264288	3.344308	3.420585	35.11469
3.173923	3.387508	3.436477	35.0463
3.25971	3.359741	3.453068	35.20946
3.347228	3.294841	3.337646	35.00651
3.401216	3.289585	3.379218	35.20399
3.295921	3.264716	3.331235	34.81441
3.269897	3.346465	3.431208	35.1549
3.188152	3.403143	3.329342	34.87732
3.365417	3.367492	3.259908	35.03516
3.308295	3.349228	3.426655	35.23496
3.299221	3.387777	3.43588	35.31959
3.109543	3.376725	3.433203	34.87477
3.357498	3.461708	3.340119	35.39929
3.390121	3.437514	3.417465	35.58687
3.336505	3.227245	3.40442	34.98127
3.216445	3.151495	3.467681	34.6914
3.324132	3.250294	3.420449	35.03966
3.276991	3.364526	3.353896	35.04084
3.40864	3.456991	3.37504	35.57718
3.103562	3.323079	3.412144	34.69832
3.220858	3.175487	3.340119	34.47456
3.18275	3.332649	3.447339	34.96939
3.31609	3.432998	3.363718	35.29756
3.186296	3.478624	3.464459	35.3338
3.346527	3.479365	3.459599	35.6752
3.229272	3.235467	3.466437	34.90037
3.468649	3.186	3.352515	35.06654
3.404639	3.266334	3.366907	35.13371
3.30681	3.340736	3.359421	35.06611
3.246347	3.396808	3.379184	35.09972
3.247296	3.424372	3.355107	35.10942
3.311676	3.343567	3.324687	35.00698
3.36187	3.333525	3.304293	35.05019
3.222508	3.453756	3.501341	35.43927
3.206052	3.437312	3.406398	35.15969
3.304253	3.41797	3.476736	35.48596
3.358075	3.262358	3.416851	35.13241
3.266886	3.171578	3.438813	34.7825
3.246759	3.259999	3.457161	34.97197
3.271134	3.371805	3.443997	35.24099
3.286147	3.443781	3.4124	35.36212
3.301778	3.333391	3.352379	35.02364
3.24944	3.233378	3.421267	34.84112
3.20832	3.292483	3.38771	34.80707
3.108099	3.420665	3.421813	34.94279
3.340671	3.440479	3.329649	35.29317
3.213723	3.41615	3.431839	35.18583
3.30417	3.336558	3.367998	35.06995
3.288374	3.189572	3.453614	34.90121
3.369706	3.314385	3.343478	35.11116

3.340629	3.323079	3.403278	35.19736
3.401793	3.404423	3.42836	35.56386
3.25806	3.386699	3.437654	35.2311
3.31943	3.344376	3.300849	34.97358
3.263339	3.344915	3.337254	34.93171
3.300994	3.402671	3.399799	35.27713
3.21393	3.488935	3.367606	35.20498
3.24779	3.442434	3.49302	35.4516
3.135691	3.344713	3.389261	34.76585
3.361086	3.1612	3.427013	34.94
3.327968	3.193077	3.329154	34.72328
3.333989	3.358596	3.451073	35.36503
3.321946	3.46036	3.459053	35.57869
3.25938	3.403008	3.389688	35.16475
3.298809	3.294841	3.375432	34.98326
3.328504	3.284665	3.314047	34.8917
3.215621	3.335143	3.427406	35.00313
3.124638	3.428685	3.396508	34.94116
3.273031	3.459282	3.475389	35.50508
3.274929	3.386632	3.423944	35.23786
3.26338	3.22583	3.374222	34.75222
3.251131	3.213228	3.407302	34.77021
3.208156	3.23567	3.429673	34.77423
3.282765	3.433403	3.38875	35.28031
3.398741	3.429224	3.381435	35.5088
3.326483	3.423968	3.358279	35.28865
3.275547	3.305557	3.339044	34.87625
3.22391	3.308387	3.437432	34.98468
3.303057	3.42855	3.339709	35.20683
3.191081	3.456519	3.418061	35.19446
3.288869	3.415746	3.426741	35.33812
3.251007	3.192807	3.442002	34.80117
3.362901	3.154595	3.347894	34.7565
3.275341	3.337367	3.501341	35.30028
3.466545	3.495405	3.422768	35.89219
3.308583	3.394314	3.48949	35.47159
3.249234	3.279947	3.410507	34.91898
3.289116	3.191122	3.360973	34.70363
3.346115	3.291741	3.393729	35.11994
3.214466	3.442838	3.434652	35.25197
3.207083	3.537661	3.400055	35.36752
3.136639	3.355428	3.514266	35.06472
3.310522	3.212958	3.299553	34.66387
3.371108	3.137679	3.373881	34.79429
3.332834	3.292483	3.394002	35.09312
3.260328	3.459821	3.514437	35.56387
3.338691	3.474378	3.401317	35.5197
3.371521	3.410624	3.281257	35.18952
3.403443	3.32436	3.29558	35.10201
3.16254	3.332649	3.346445	34.70455

3.1665	3.410556	3.416782	35.0374
3.289034	3.457665	3.440041	35.45924
3.189926	3.340467	3.40014	34.89896
3.292622	3.213362	3.350247	34.73647
3.14596	3.150012	3.466641	34.53175
3.22024	3.408871	3.417055	35.15183
3.315265	3.463595	3.402851	35.44825
3.428272	3.418711	3.417192	35.62858
3.236119	3.318631	3.423739	35.00383
3.261277	3.295313	3.374	34.89908
3.271588	3.258247	3.398895	34.89502
3.3264	3.406984	3.372363	35.28213
3.174006	3.412309	3.339437	34.8885
3.213435	3.337704	3.489882	35.14058
3.226798	3.253799	3.341449	34.66171
3.304294	3.190246	3.383003	34.78308
3.285941	3.288574	3.387999	34.9689
3.324132	3.377129	3.408649	35.29123
3.277692	3.391686	3.404079	35.21151
3.37449	3.399032	3.334918	35.28801
3.328545	3.322203	3.374904	35.10697
3.388802	3.396673	3.342045	35.32974
3.249523	3.450521	3.380019	35.22596
3.158457	3.419924	3.393865	34.99018
3.220157	3.364594	3.461372	35.15174
3.225395	3.254472	3.415606	34.82229
3.266515	3.146845	3.317355	34.46199
3.245976	3.195638	3.422222	34.7531
3.350651	3.463797	3.466829	35.66598
3.293818	3.412376	3.436358	35.36261
3.405505	3.340399	3.404164	35.37905
3.376099	3.327123	3.301804	35.06186
3.335556	3.241466	3.389705	34.97811
3.24779	3.379353	3.42686	35.16897
3.265566	3.470671	3.406671	35.36339
3.231417	3.446949	3.405375	35.234
3.308501	3.332043	3.36087	35.05396
3.167861	3.178655	3.417925	34.53574
3.233231	3.164974	3.38684	34.5808
3.2063	3.296054	3.432589	34.9086
3.347806	3.417498	3.396304	35.40428
3.313574	3.435762	3.409041	35.39722
3.502922	3.354148	3.346462	35.49596
3.253688	3.298683	3.408529	34.96537
3.386162	3.413185	3.346104	35.36895
3.209228	3.42727	3.376541	35.07939
3.325575	3.445264	3.392501	35.40807
3.173511	3.236815	3.441541	34.72693
3.179574	3.231222	3.383481	34.60099
3.244862	3.238702	3.430202	34.86229

3.359395	3.501066	3.408734	35.63956
3.231788	3.473232	3.487291	35.47143
3.340836	3.330021	3.356352	35.11037
3.281198	3.27058	3.404761	34.95583
3.374944	3.273949	3.363309	35.07756
3.265649	3.329414	3.370607	34.9758
3.186833	3.381914	3.429861	35.04783
3.204856	3.410287	3.371971	35.02269
3.251956	3.317957	3.399373	34.98371
3.250677	3.232704	3.332394	34.648
3.270186	3.210397	3.433681	34.86338
3.227457	3.395191	3.477367	35.26959
3.301283	3.43502	3.483829	35.53227
3.484734	3.499381	3.377291	35.84121
3.398783	3.354754	3.32126	35.21444
3.410578	3.294639	3.320066	35.10616
3.253936	3.352463	3.40928	35.08516
3.245316	3.44486	3.386908	35.21944
3.269237	3.376118	3.333349	35.0043
3.314234	3.247059	3.385186	34.93383
3.091354	3.151091	3.412588	34.29648
3.18477	3.2416	3.40471	34.68147
3.264494	3.424305	3.407353	35.26114
3.302355	3.527956	3.490991	35.75351
3.23682	3.384542	3.387249	35.0697
3.304418	3.303805	3.48417	35.25292
3.234056	3.313779	3.371204	34.87382
3.400927	3.356776	3.32883	35.24011
3.257235	3.380027	3.406944	35.14754
3.300953	3.383868	3.431225	35.30465
3.167984	3.320653	3.34815	34.69395
3.253111	3.218484	3.376882	34.71952
3.175449	3.285945	3.430321	34.81407
3.285569	3.315464	3.378229	35.00552
3.199989	3.414465	3.497266	35.29519
3.27662	3.493518	3.385595	35.39143
3.387399	3.415409	3.433425	35.56748
3.464483	3.40375	3.313655	35.44863
3.318729	3.353069	3.411462	35.23295
3.216693	3.448836	3.312018	35.00177
3.238469	3.412915	3.495424	35.37192
3.195411	3.309938	3.334253	34.70011
3.311759	3.166322	3.361518	34.7001
3.227127	3.209521	3.431362	34.76223
3.15887	3.414533	3.461645	35.12752
3.157426	3.457867	3.507838	35.32014
3.393586	3.423092	3.375432	35.47099
3.274764	3.328808	3.443775	35.15441
3.335969	3.348487	3.386703	35.20649
3.299551	3.348419	3.447322	35.25926

3.363973	3.364594	3.399867	35.33174
3.220075	3.35462	3.393916	34.98223
3.382038	3.348824	3.353879	35.23619
3.156684	3.209184	3.429264	34.60286
3.28062	3.249216	3.285485	34.64701
3.176027	3.280015	3.41061	34.75926
3.251956	3.411769	3.390915	35.17036
3.302974	3.455777	3.438046	35.48124
3.345166	3.445399	3.375859	35.41481
3.30351	3.364998	3.473001	35.36033
3.383275	3.418644	3.366719	35.41966
3.352466	3.394045	3.316639	35.18897
3.398411	3.426596	3.377069	35.49278
3.209475	3.339658	3.395639	34.9301
3.188194	3.136803	3.403823	34.45785
3.266061	3.207027	3.309835	34.57616
3.255709	3.30987	3.432248	35.04612
3.234593	3.489744	3.391256	35.30366
3.25076	3.416554	3.463009	35.33587
3.293777	3.355428	3.432726	35.23004
3.388925	3.331571	3.413645	35.34422
3.311965	3.350913	3.39025	35.16706
3.186461	3.304209	3.381707	34.77178
3.266185	3.364931	3.430543	35.18571
3.224653	3.352867	3.428156	35.06328
3.366943	3.221315	3.348423	34.9124
3.287879	3.252653	3.308607	34.72096
3.185018	3.271388	3.395724	34.7275
3.121132	3.342017	3.412809	34.77961
3.320503	3.436705	3.338004	35.25909
3.292746	3.45416	3.369192	35.30476
3.335309	3.382588	3.430355	35.37508
3.286807	3.382857	3.378024	35.15516
3.330855	3.361696	3.451823	35.3666
3.394576	3.415678	3.307158	35.30763
3.42889	3.355024	3.372022	35.39188
3.205433	3.234861	3.38198	34.66222
3.182626	3.164232	3.347741	34.38301
3.172645	3.276645	3.436204	34.80047
3.278517	3.504907	3.37944	35.40703
3.220364	3.469324	3.492065	35.44834
3.191287	3.393236	3.431907	35.0868
3.253688	3.295448	3.465482	35.08284
3.396762	3.325303	3.418436	35.35813
3.306645	3.367559	3.453494	35.33013
3.226138	3.393438	3.369021	35.02594
3.24445	3.280082	3.460076	35.01721
3.24511	3.222663	3.338737	34.62774
3.423735	3.189168	3.325676	34.91655
3.285157	3.261953	3.352805	34.832

3.218879	3.34788	3.436767	35.05858
3.18642	3.413589	3.377291	35.00123
3.341001	3.43529	3.42067	35.48159
3.37317	3.453756	3.335958	35.40707
3.339928	3.379083	3.373795	35.25383
3.148146	3.37841	3.489746	35.08652
3.27431	3.435896	3.345302	35.17226
3.343888	3.395258	3.352396	35.25106
3.355848	3.213565	3.334526	34.8408
3.175573	3.101489	3.361058	34.2595
3.175944	3.227178	3.367469	34.54919
3.186338	3.456586	3.442564	35.23782
3.356178	3.513736	3.346411	35.52394
3.161385	3.411904	3.440024	35.07999
3.276042	3.345724	3.40812	35.11623
3.156024	3.355496	3.506337	35.08992
3.363561	3.441153	3.371681	35.43662
3.388472	3.383464	3.423739	35.47878
3.308459	3.309196	3.373011	35.03046
3.310315	3.276173	3.412298	35.04822
3.273485	3.215519	3.340255	34.67749
3.304418	3.220776	3.339897	34.75585
3.295385	3.298211	3.357068	34.94298
3.13334	3.356237	3.415367	34.843
3.108264	3.406782	3.434226	34.93994
3.386987	3.410826	3.372176	35.42261
3.407114	3.401121	3.37429	35.45003
3.317574	3.432998	3.405392	35.39194
3.255338	3.374838	3.465192	35.25943
3.286807	3.404289	3.447458	35.35387
3.292787	3.305018	3.294386	34.81511
3.398741	3.187214	3.337373	34.8832
3.201144	3.1798	3.427423	34.6318
3.241109	3.347274	3.389142	35.00172
3.18906	3.404626	3.408802	35.05631
3.374243	3.425181	3.337646	35.35062
3.244656	3.390136	3.417925	35.16616
3.273403	3.35718	3.414463	35.14938
3.247749	3.38097	3.487649	35.30535
3.246677	3.384071	3.433203	35.19072
3.229643	3.322608	3.486609	35.13585
3.36121	3.291067	3.478032	35.33584
3.319925	3.23513	3.404232	34.96184
3.336711	3.217743	3.38921	34.92768
3.304294	3.301042	3.334372	34.91902
3.266309	3.342758	3.417533	35.10904
3.241274	3.41487	3.36261	35.09188
3.306026	3.451869	3.42258	35.44554
3.214425	3.408804	3.434107	35.17626
3.33766	3.4115	3.371067	35.31379

3.284786	3.388923	3.429588	35.27677
3.366736	3.4177	3.40621	35.46779
3.320008	3.363246	3.440655	35.32184
3.299015	3.251979	3.295239	34.71461
3.241975	3.169287	3.354306	34.53821
3.301861	3.317418	3.309971	34.89615
3.177099	3.4733	3.401913	35.16527
3.172934	3.432796	3.334867	34.92097
3.287054	3.395999	3.426451	35.29034
3.232159	3.323214	3.38834	34.92778
3.289529	3.347408	3.474246	35.29401
3.2428	3.435896	3.442769	35.3165
3.303469	3.291809	3.501887	35.26336
3.199082	3.283991	3.394343	34.7828
3.444439	3.230413	3.390847	35.19455
3.316296	3.246655	3.365082	34.89349
3.321657	3.338108	3.360291	35.09473
3.26338	3.349835	3.405733	35.09231
3.329782	3.344443	3.349855	35.10353
3.239995	3.425383	3.394189	35.18114
3.318688	3.474311	3.365065	35.39653
3.211496	3.410085	3.474263	35.26047
3.260741	3.437042	3.336026	35.12481
3.186132	3.34323	3.457502	35.02214
3.43718	3.303468	3.33219	35.21016
3.312378	3.287698	3.456683	35.17499
3.29786	3.22246	3.353146	34.77416
3.25806	3.272938	3.418743	34.94097
3.326277	3.429292	3.389346	35.36778
3.270309	3.420598	3.384299	35.21534
3.231747	3.353137	3.389705	34.9953
3.149219	3.383127	3.413969	34.93347
3.230922	3.413522	3.423671	35.19983
3.322482	3.479163	3.405187	35.50318
3.265154	3.333997	3.474485	35.2119
3.234881	3.215047	3.383907	34.6875
3.241068	3.225358	3.442684	34.85211
3.309161	3.250227	3.323937	34.79572
3.399195	3.236141	3.33045	34.97605
3.20964	3.325438	3.332053	34.76031
3.220199	3.353676	3.437313	35.07534
3.296953	3.411298	3.383924	35.25244
3.288869	3.479028	3.391665	35.39981
3.315718	3.480713	3.344893	35.35993
3.218136	3.412039	3.43438	35.19204
3.219415	3.372883	3.421335	35.08069
3.208898	3.342219	3.399799	34.94353
3.409052	3.288237	3.371545	35.2014
3.243336	3.228256	3.345712	34.65135
3.383605	3.237354	3.383907	35.06151

3.256617	3.366009	3.404147	35.10942
3.377501	3.420935	3.378433	35.43766
3.309738	3.370187	3.340238	35.09497
3.277857	3.382386	3.426996	35.24165
3.148971	3.422283	3.381588	34.94775
3.256493	3.459215	3.472967	35.46347
3.223251	3.429157	3.462191	35.30148
3.345579	3.291067	3.350503	35.02277
3.209558	3.177913	3.436272	34.66543
3.293818	3.246992	3.363223	34.84101
3.309326	3.256023	3.449794	35.08399
3.32574	3.349026	3.370863	35.15066
3.232613	3.353743	3.445088	35.11964
3.265195	3.357113	3.302469	34.88637
3.197061	3.421407	3.446094	35.19206
3.311676	3.462719	3.32421	35.26651
3.281981	3.465954	3.400379	35.37521
3.273485	3.409343	3.361587	35.148
3.318977	3.300368	3.425035	35.14792
3.207454	3.302929	3.369788	34.78883
3.300623	3.248272	3.391256	34.91999
3.37779	3.3466	3.373591	35.26514
3.333783	3.339928	3.382185	35.17311
3.234675	3.392023	3.418266	35.1492
3.288869	3.352396	3.385868	35.11021
3.2282	3.327392	3.385749	34.92259
3.287879	3.400515	3.381929	35.20466
3.211702	3.365874	3.478356	35.17319
3.242305	3.465684	3.458849	35.41572
3.18741	3.342691	3.44664	35.00001
3.290601	3.239578	3.378246	34.85061
3.181265	3.238702	3.487956	34.84951
3.359973	3.241735	3.380087	35.01106
3.257194	3.291876	3.485398	35.12625
3.339268	3.412646	3.34861	35.2707
3.361004	3.38616	3.399526	35.37166
3.421796	3.399773	3.318565	35.35733
3.262143	3.411972	3.375927	35.16031
3.300087	3.447555	3.349531	35.26338
3.16551	3.385621	3.428378	35.00606
3.309119	3.325101	3.350401	35.01724
3.28891	3.277252	3.355925	34.88049
3.160231	3.206353	3.41344	34.56982
3.324874	3.298278	3.355039	35.00318
3.314069	3.338041	3.44577	35.26492
3.37812	3.445129	3.360717	35.45318
3.308006	3.38151	3.454961	35.36682
3.238304	3.342556	3.410166	35.03125
3.224859	3.393506	3.373608	35.03332
3.386368	3.419385	3.374887	35.4459

3.286477	3.421339	3.4412	35.37675
3.240408	3.31634	3.400942	34.95835
3.088137	3.173196	3.388255	34.28458
3.305696	3.180407	3.417584	34.84025
3.303345	3.250968	3.360239	34.86401
3.308831	3.326382	3.390932	35.10805
3.24878	3.393371	3.404898	35.15376
3.383852	3.382588	3.40517	35.42616
3.301819	3.399504	3.354493	35.17293
3.431076	3.419654	3.351969	35.49414
3.209022	3.428146	3.39453	35.12019
3.177429	3.392967	3.418044	35.02559
3.204237	3.349565	3.383089	34.91286
3.321492	3.321597	3.388681	35.12035
3.294024	3.274825	3.359677	34.89457
3.266886	3.279341	3.430151	34.99921
3.187905	3.357383	3.459752	35.06189
3.354033	3.360078	3.315888	35.11648
3.408805	3.397145	3.390796	35.48112
3.275506	3.329077	3.325011	34.89691
3.284868	3.346532	3.441661	35.21065
3.256617	3.458608	3.393388	35.28839
3.302933	3.462989	3.37088	35.35003
3.310769	3.391416	3.316997	35.09282
3.166871	3.278128	3.486302	34.90064
3.154415	3.192538	3.417038	34.53476
3.263875	3.256831	3.440501	34.96604
3.342898	3.326651	3.38331	35.16647
3.301448	3.309466	3.405324	35.08638
3.252162	3.345117	3.395911	35.03598
3.320214	3.353609	3.425496	35.26807
3.285734	3.429359	3.376626	35.25145
3.412434	3.471278	3.368885	35.60326
3.274269	3.422755	3.425905	35.3197
3.31378	3.360213	3.354425	35.11302
3.129793	3.243959	3.386431	34.52643
3.323967	3.314049	3.352447	35.03002
3.266597	3.287293	3.397003	34.94348
3.245769	3.39209	3.372909	35.07442
3.244037	3.347678	3.466368	35.17789
3.37482	3.368974	3.390864	35.34535
3.357168	3.317486	3.376114	35.16189
3.431159	3.398762	3.334645	35.41075
3.254719	3.429157	3.441115	35.32421
3.201598	3.388653	3.365901	34.95498
3.192194	3.375849	3.40413	34.99002
3.234139	3.25892	3.370709	34.75296
3.250512	3.215721	3.424933	34.81287
3.18675	3.234322	3.432521	34.7307
3.18477	3.277049	3.478424	34.92019

3.387936	3.352463	3.418522	35.39841
3.391771	3.395864	3.35879	35.37108
3.284703	3.376725	3.419221	35.22724
3.352879	3.334199	3.38036	35.19835
3.288663	3.377264	3.400277	35.19565
3.406289	3.432392	3.345694	35.45407
3.361086	3.399099	3.347178	35.28566
3.333412	3.273141	3.379047	35.01938
3.181842	3.239781	3.438131	34.74418
3.286312	3.290528	3.347348	34.88508
3.273815	3.332649	3.421403	35.11181
3.300087	3.361763	3.383361	35.14974
3.233974	3.338647	3.424797	35.04523
3.306769	3.345926	3.42154	35.21321
3.319513	3.340871	3.34271	35.05764
3.350198	3.438862	3.398299	35.46059
3.258514	3.431044	3.419494	35.28935
3.243171	3.346465	3.384248	34.99376
3.176233	3.276982	3.348713	34.61772
3.223292	3.280689	3.364877	34.76409
3.195081	3.245913	3.371357	34.64052
3.231623	3.242139	3.447731	34.87919
3.201598	3.361292	3.441916	35.06138
3.311965	3.40658	3.4412	35.40021
3.433674	3.419048	3.389756	35.58113
3.452152	3.394382	3.369771	35.5239
3.323266	3.378814	3.390131	35.25252
3.317657	3.44176	3.403755	35.4077
3.297406	3.407726	3.395758	35.2715
3.329576	3.364863	3.299058	35.03665
3.323761	3.237894	3.377018	34.91676
3.125174	3.233176	3.390029	34.50062
3.225601	3.321934	3.474468	35.09899
3.348094	3.430572	3.417123	35.47903
3.300953	3.363246	3.447066	35.29419
3.252038	3.35435	3.403482	35.07246
3.296994	3.353002	3.429452	35.22461
3.278764	3.429022	3.369038	35.21888
3.415569	3.457058	3.377069	35.59692
3.281363	3.384879	3.37591	35.14305
3.226096	3.30859	3.410507	34.93102
3.187451	3.236681	3.365952	34.59182
3.269691	3.228863	3.373045	34.77008
3.214342	3.287226	3.409638	34.85669
3.271959	3.334469	3.374478	35.00912
3.192854	3.315733	3.476531	35.01833
3.337949	3.402334	3.387386	35.33006
3.335268	3.350845	3.402527	35.24471
3.38509	3.356102	3.405477	35.37161
3.284909	3.445938	3.454961	35.45721

3.381543	3.461708	3.393234	35.56803
3.286724	3.310342	3.39794	35.03995
3.329865	3.274084	3.265535	34.76545
3.261813	3.248879	3.404693	34.86583
3.255091	3.381981	3.381315	35.09108
3.197556	3.39391	3.4892	35.22728
3.294973	3.416689	3.356215	35.19931
3.279177	3.316205	3.459752	35.17144
3.390864	3.311555	3.326409	35.11391
3.362447	3.387575	3.424404	35.43232
3.320791	3.481522	3.40824	35.51132
3.278599	3.335615	3.373284	35.02353
3.300541	3.238972	3.3644	34.84074
3.270144	3.288911	3.299689	34.74197
3.133505	3.249755	3.426741	34.63537
3.195576	3.29073	3.427201	34.86173
3.170748	3.316138	3.459957	34.93463
3.263958	3.350374	3.387027	35.05384
3.296169	3.348958	3.380667	35.10728
3.301159	3.342758	3.377888	35.09856
3.206712	3.339591	3.410559	34.95654
3.397545	3.434953	3.389807	35.53702
3.320874	3.403615	3.484852	35.50867
3.358611	3.38744	3.358261	35.27899
3.336423	3.224819	3.386618	34.93685
3.29753	3.188359	3.384009	34.76636
3.140516	3.405906	3.382441	34.89531
3.31844	3.433268	3.35538	35.28506
3.211909	3.404828	3.429725	35.15248
3.274392	3.337097	3.413304	35.10509
3.186379	3.359202	3.386567	34.90249
3.395442	3.44176	3.483659	35.75254
3.378243	3.481589	3.425496	35.67484
3.355106	3.376253	3.399987	35.3381
3.193349	3.214306	3.446998	34.73302
3.307758	3.228661	3.373335	34.85352
3.284208	3.26256	3.424285	34.98757
3.24082	3.27806	3.351577	34.76758
3.085869	3.239242	3.444542	34.54714
3.199866	3.351452	3.418829	34.98559
3.293653	3.415476	3.434226	35.36437
3.378615	3.462315	3.379184	35.53222
3.330277	3.354417	3.45346	35.353
3.290106	3.354957	3.432061	35.21953
3.274186	3.427742	3.462907	35.41134
3.423116	3.404895	3.244152	35.20868
3.335721	3.242005	3.412826	35.03021
3.164561	3.179463	3.315633	34.3066
3.265113	3.214238	3.418505	34.8275
3.22057	3.401256	3.359881	35.01087

3.33898	3.380634	3.336077	35.17266
3.254059	3.373962	3.49863	35.32784
3.262143	3.314116	3.414054	35.02969
3.139403	3.433807	3.448839	35.09909
3.478217	3.553835	3.346428	35.87855
3.30384	3.440075	3.469438	35.51744
3.35457	3.293426	3.33826	35.02082
3.243914	3.195368	3.485585	34.88657
3.366819	3.26721	3.331695	34.97592
3.286147	3.2261	3.342847	34.73398
3.265443	3.237691	3.382065	34.79982
3.090199	3.260875	3.442411	34.59926
3.245893	3.376927	3.429929	35.16623
3.269196	3.396538	3.394547	35.1827
3.402824	3.408669	3.348269	35.40025
3.248327	3.365403	3.423245	35.13173
3.242305	3.376455	3.38921	35.0683
3.305119	3.448634	3.426809	35.44573
3.39738	3.364324	3.326614	35.24401
3.274351	3.261414	3.381366	34.86965
3.258679	3.214778	3.349258	34.66318
3.2063	3.343365	3.444798	35.03876
3.274475	3.446545	3.418215	35.35536
3.320214	3.374501	3.339573	35.12585
3.257813	3.263908	3.444406	34.9768
3.246553	3.303198	3.45225	35.05525
3.202299	3.481589	3.425837	35.29082
3.362076	3.50949	3.396389	35.63685
3.377171	3.374838	3.360018	35.29586
3.365375	3.232233	3.362098	34.96276
3.225684	3.259864	3.423995	34.85306
3.34463	3.298481	3.402101	35.14974
3.209888	3.30987	3.387624	34.84833
3.252863	3.264986	3.370999	34.8078
3.173099	3.331234	3.465055	34.98393
3.234098	3.359809	3.41049	35.06049
3.194339	3.335412	3.424728	34.95133
3.307717	3.38306	3.370453	35.18477
3.229272	3.375916	3.491264	35.2618
3.456028	3.456923	3.411036	35.75938
3.282352	3.405097	3.38418	35.20751
3.343846	3.237961	3.291965	34.77483
3.342485	3.230683	3.34196	34.86527
3.269897	3.361696	3.375467	35.06631
3.216157	3.456654	3.367316	35.13862
3.283012	3.441894	3.346939	35.20799
3.198917	3.341141	3.42067	34.96499
3.284208	3.370255	3.380411	35.12714
3.274186	3.453486	3.456751	35.45418
3.196896	3.432257	3.501358	35.33628

3.263215	3.327999	3.330399	34.87945
3.268536	3.175891	3.415145	34.74378
3.283301	3.239107	3.390438	34.86028
3.239707	3.352463	3.397668	35.02865
3.296251	3.312027	3.399646	35.0682
3.174047	3.286215	3.400413	34.74619
3.295344	3.293022	3.382662	34.98752
3.222549	3.410354	3.38227	35.08405
3.31943	3.418778	3.433766	35.42695
3.193927	3.413319	3.38198	35.02731
3.265649	3.274151	3.382884	34.8818
3.285239	3.362168	3.381435	35.11394
3.373294	3.40193	3.333366	35.28834
3.30582	3.293426	3.417652	35.08783
3.325534	3.220102	3.337032	34.79429
3.26338	3.251372	3.383532	34.82844
3.376635	3.420598	3.364042	35.40355
3.197308	3.450925	3.320134	34.9817
3.186173	3.405097	3.389892	35.00968
3.270062	3.311757	3.331593	34.86152
3.291921	3.420598	3.40662	35.31141
3.294973	3.535572	3.427951	35.61616
3.254266	3.446545	3.443656	35.3668
3.187121	3.202782	3.412775	34.61936
3.311099	3.189775	3.399185	34.83232
3.385915	3.293291	3.437517	35.30613
3.278599	3.394382	3.34786	35.09645
3.264535	3.277184	3.457706	35.04962
3.163283	3.243555	3.349787	34.51865
3.294148	3.312768	3.403329	35.07328
3.304706	3.471548	3.331525	35.28657
3.270763	3.415543	3.490582	35.4377
3.131896	3.356439	3.394053	34.79367
3.274186	3.333391	3.425172	35.12249
3.299963	3.421339	3.437227	35.39755
3.380141	3.39418	3.331951	35.28327
3.248244	3.230076	3.406961	34.8
3.276537	3.189909	3.407506	34.77523
3.232448	3.355091	3.414446	35.05521
3.423529	3.399099	3.326921	35.37791
3.308212	3.369985	3.37591	35.1692
3.215786	3.340399	3.382406	34.91658
3.092798	3.344713	3.396713	34.68834
3.293736	3.529034	3.382952	35.50075
3.288498	3.482128	3.442616	35.5172
3.28693	3.384542	3.343103	35.08275
3.187451	3.212688	3.471416	34.76998
3.315017	3.227852	3.37782	34.87743
3.432932	3.324966	3.416834	35.43298
3.398246	3.406984	3.392842	35.48403

3.21925	3.289382	3.440569	34.93978
3.173222	3.262762	3.352583	34.5885
3.254472	3.283384	3.375177	34.86069
3.255462	3.388923	3.392484	35.1315
3.226055	3.462449	3.414941	35.27709
3.117791	3.411702	3.473036	35.0564
3.214548	3.351452	3.385476	34.94476
3.315883	3.330762	3.409894	35.17451
3.375934	3.284193	3.338192	35.0472
3.224529	3.281902	3.425769	34.9026
3.300953	3.380903	3.405273	35.24141
3.290312	3.432729	3.438949	35.40512
3.413135	3.466493	3.30327	35.45084
3.339763	3.324764	3.363752	35.11271
3.235789	3.312701	3.418846	34.97944
3.157137	3.396875	3.382134	34.91123
3.341207	3.500325	3.420619	35.62415
3.240697	3.352665	3.324158	34.8705
3.259297	3.174274	3.410473	34.70982
3.121998	3.237354	3.457485	34.65032
3.330195	3.348082	3.430969	35.28978
3.309037	3.472559	3.374767	35.39281
3.269526	3.304142	3.427423	35.05326
3.254678	3.179329	3.364264	34.60972
3.283672	3.235804	3.3371	34.73723
3.213558	3.345926	3.388494	34.93711
3.254142	3.449645	3.420176	35.32196
3.161056	3.463393	3.395093	35.09361
3.260535	3.362302	3.421557	35.14795
3.21063	3.283317	3.36822	34.74945
3.389544	3.291943	3.389841	35.20686
3.339681	3.272467	3.420346	35.12193
3.386492	3.406513	3.376677	35.42194
3.338815	3.397617	3.474962	35.51316
3.356797	3.389799	3.32571	35.20899
3.275011	3.305355	3.403755	35.01615
3.258266	3.347543	3.409484	35.08432
3.214919	3.489542	3.440245	35.36732
3.232695	3.513871	3.37504	35.31681
3.21694	3.393775	3.515443	35.32676
3.273403	3.183035	3.430424	34.80346
3.284126	3.172589	3.372312	34.67698
3.23385	3.282171	3.408598	34.88603
3.345579	3.486307	3.452727	35.67327
3.261731	3.307242	3.44161	35.07401
3.333535	3.247396	3.318037	34.82993
3.37779	3.251305	3.349395	35.00383
3.311388	3.366818	3.383958	35.18681
3.141547	3.446342	3.451414	35.13682
3.250224	3.481454	3.42142	35.38567

3.194586	3.331503	3.474536	35.05224
3.291756	3.232435	3.302264	34.67136
3.254719	3.212958	3.430032	34.82718
3.269361	3.285069	3.388664	34.92643
3.189555	3.379218	3.405869	34.99542
3.373335	3.408198	3.393251	35.4331
3.387812	3.400784	3.414856	35.49579
3.390658	3.319103	3.391	35.27122
3.221642	3.353743	3.395724	34.98769
3.228282	3.433201	3.351475	35.07921
3.280208	3.519936	3.443519	35.58373
3.244408	3.439873	3.381656	35.19507
3.193844	3.137949	3.34815	34.35096
3.197432	3.183305	3.432947	34.64343
3.347105	3.310948	3.404761	35.18824
3.408145	3.487722	3.434499	35.77333
3.354116	3.386227	3.414259	35.38896
3.274021	3.215182	3.378723	34.76205
3.333288	3.305557	3.368697	35.06737
3.357745	3.386362	3.360325	35.27925
3.372799	3.443984	3.328575	35.36875
3.138124	3.350374	3.359626	34.71874
3.197102	3.324427	3.45346	34.99618
3.238016	3.318362	3.353692	34.85421
3.349744	3.319103	3.368151	35.13178
3.298025	3.280891	3.371033	34.94142
3.28161	3.282576	3.462532	35.1093
3.148559	3.311016	3.377257	34.69405
3.445223	3.452071	3.402118	35.70564
3.33865	3.433403	3.365133	35.35088
3.337495	3.348419	3.399509	35.23768
3.247337	3.344376	3.444542	35.13016
3.261648	3.462989	3.344467	35.20199
3.303345	3.484285	3.408035	35.47876
3.285858	3.403952	3.366037	35.173
3.154374	3.188157	3.461253	34.62178
3.200773	3.232839	3.385271	34.6548
3.232077	3.292348	3.472422	35.04398
3.434871	3.390001	3.447202	35.64586
3.323802	3.277521	3.492952	35.25704
3.359313	3.288641	3.412622	35.18334
3.249316	3.316475	3.438591	35.06046
3.371892	3.352867	3.339897	35.19226
3.269649	3.417767	3.402306	35.24708
3.220487	3.464741	3.379951	35.19341
3.137506	3.39391	3.505178	35.1309
3.242882	3.364459	3.29899	34.84603
3.358859	3.284126	3.346053	35.0269
3.277114	3.239848	3.380343	34.8263
3.282889	3.393438	3.416765	35.25445

3.223127	3.386362	3.416902	35.10858
3.360096	3.359876	3.349122	35.20197
3.418579	3.341545	3.34682	35.28474
3.270557	3.355496	3.362951	35.02682
3.166995	3.350441	3.388187	34.84448
3.301366	3.491968	3.385391	35.44171
3.260865	3.408265	3.40575	35.21462
3.286312	3.249822	3.337885	34.77537
3.146373	3.145295	3.437807	34.45928
3.197638	3.341208	3.521956	35.18384
3.255915	3.479163	3.436579	35.42626
3.417218	3.448499	3.39112	35.61253
3.310027	3.240724	3.428787	35.00612
3.387152	3.19611	3.362252	34.93172
3.233149	3.252316	3.422171	34.84889
3.329947	3.390001	3.309886	35.11612
3.250677	3.475052	3.40367	35.33385
3.294519	3.360348	3.33323	35.02484
3.251007	3.139566	3.389278	34.56944
3.264329	3.169961	3.326324	34.52737
3.22589	3.396134	3.488655	35.29291
3.331638	3.495338	3.442633	35.64046
3.324503	3.432864	3.476105	35.56144
3.315265	3.381914	3.366583	35.19031
3.333907	3.288506	3.374136	35.04333
3.394411	3.353339	3.336555	35.23523
3.224447	3.400784	3.338874	34.97238
3.241521	3.406311	3.39025	35.13415
3.182172	3.321664	3.374546	34.78491
3.163406	3.180407	3.382117	34.45152
3.272207	3.193346	3.368697	34.6884
3.236778	3.349093	3.443997	35.11619
3.28693	3.488598	3.456342	35.55794
3.149714	3.406176	3.465124	35.09682
3.349909	3.336491	3.375228	35.18564
3.378573	3.224011	3.373318	34.99817
3.363684	3.331301	3.352873	35.15553
3.23983	3.393438	3.482465	35.30396
3.329824	3.438862	3.406756	35.43453
3.180316	3.350643	3.40558	34.91209
3.332463	3.179868	3.256276	34.54485
3.165592	3.146508	3.39141	34.4025
3.13235	3.277791	3.431242	34.704
3.131896	3.380836	3.418897	34.90136
3.289529	3.470739	3.403465	35.40893
3.375769	3.432527	3.361655	35.42253
3.417053	3.301311	3.41506	35.34265
3.276207	3.346937	3.366497	35.02822
3.318935	3.461506	3.409518	35.46628
3.386245	3.442366	3.443161	35.64519

3.286724	3.345252	3.338601	34.98653
3.197226	3.203927	3.428378	34.67808
3.164561	3.160593	3.430458	34.51644
3.209187	3.277184	3.466368	34.94752
3.313202	3.485768	3.378843	35.43972
3.261442	3.419452	3.488518	35.42135
3.207702	3.356035	3.404215	34.98079
3.29555	3.28298	3.443639	35.09935
3.417548	3.344308	3.359131	35.31545
3.308954	3.38899	3.347877	35.15108
3.236696	3.369311	3.379951	35.02017
3.264164	3.335075	3.421403	35.09601
3.195288	3.313914	3.314064	34.66438
3.3914	3.208443	3.316775	34.86853
3.255091	3.228593	3.378229	34.7489
3.232489	3.440681	3.486898	35.40092
3.138165	3.395056	3.435539	34.98256
3.403608	3.433942	3.39378	35.55675
3.345455	3.397684	3.384231	35.32941
3.345661	3.371738	3.35376	35.20648
3.262679	3.40476	3.459531	35.32854
3.309985	3.469795	3.362627	35.36229
3.32075	3.368772	3.404284	35.25601
3.375274	3.259729	3.290788	34.88859
3.087848	3.115978	3.386431	34.15483
3.240862	3.22583	3.347417	34.64436
3.148229	3.47303	3.452932	35.21312
3.280496	3.503627	3.439683	35.5403
3.204237	3.373018	3.458866	35.12986
3.306397	3.302726	3.413304	35.09992
3.185513	3.240994	3.451687	34.7845
3.344053	3.333391	3.373676	35.16266
3.352095	3.454362	3.417925	35.54156
3.277156	3.422081	3.402527	35.27341
3.284703	3.316542	3.403005	35.06017
3.228695	3.184653	3.354834	34.54392
3.321451	3.160323	3.342693	34.66701
3.280208	3.369109	3.383003	35.12155
3.150538	3.453553	3.474195	35.22207
3.288333	3.466493	3.44161	35.48044
3.353662	3.424439	3.413833	35.4706
3.392926	3.349228	3.362831	35.28046
3.340794	3.369244	3.396662	35.28421
3.229355	3.401256	3.422375	35.16674
3.295757	3.402199	3.386959	35.23657
3.245976	3.220843	3.32687	34.5997
3.309985	3.180609	3.343171	34.68734
3.241191	3.20669	3.385254	34.68597
3.191081	3.377803	3.416714	35.01938
3.121544	3.458675	3.449999	35.11696

3.319884	3.412106	3.350759	35.23183
3.221023	3.335345	3.427542	35.01569
3.286188	3.25582	3.415265	34.95744
3.239459	3.358596	3.444184	35.14324
3.29918	3.395056	3.427832	35.31781
3.339763	3.402402	3.349258	35.2508
3.426168	3.330223	3.428701	35.45564
3.245398	3.221517	3.40014	34.76014
3.239006	3.180609	3.323732	34.48961
3.179574	3.281497	3.380207	34.70377
3.200484	3.472289	3.410507	35.23299
3.237438	3.49554	3.464851	35.48349
3.25674	3.462854	3.46821	35.46157
3.314357	3.348217	3.381588	35.14745
3.432808	3.383127	3.373932	35.46608
3.303552	3.467234	3.422853	35.47433
3.267092	3.452003	3.407421	35.32754
3.30017	3.320721	3.414122	35.12744
3.1705	3.171915	3.397599	34.48233
3.322895	3.17542	3.338942	34.69498
3.286559	3.269636	3.378365	34.90777
3.268577	3.481522	3.457143	35.50408
3.147981	3.399639	3.431362	35.00491
3.271588	3.316609	3.475832	35.19089
3.282435	3.36857	3.400413	35.16331
3.279548	3.317216	3.438114	35.12714
3.187905	3.394921	3.470938	35.16845
3.241686	3.414735	3.426246	35.23165
3.284538	3.38926	3.391665	35.19403
3.379398	3.319036	3.376592	35.21494
3.349373	3.183776	3.406875	34.91972
3.187946	3.195975	3.360171	34.49124
3.167654	3.295717	3.419016	34.79367
3.270227	3.442568	3.309136	35.09883
3.236696	3.440681	3.422904	35.27017
3.354322	3.426326	3.388886	35.42162
3.203784	3.326584	3.464663	35.04001
3.385791	3.429898	3.365525	35.44716
3.377583	3.465684	3.454347	35.70171
3.39239	3.400515	3.371886	35.41124
3.301036	3.273343	3.424865	35.04922
3.23748	3.177307	3.2876	34.40004
3.250265	3.218956	3.392041	34.74747
3.3337	3.410691	3.320612	35.19302
3.218755	3.488261	3.47249	35.44342
3.06269	3.382453	3.459207	34.8417
3.280991	3.330627	3.472149	35.23406
3.33502	3.34788	3.378655	35.18548
3.277362	3.290596	3.39748	34.97529
3.197102	3.409882	3.443997	35.16236

3.213723	3.435964	3.439427	35.24575
3.272165	3.28453	3.345285	34.83652
3.494426	3.161806	3.316178	34.99054
3.23286	3.166928	3.44149	34.70377
3.21195	3.320114	3.364485	34.82464
3.155323	3.45106	3.445412	35.16414
3.37746	3.459282	3.340562	35.43861
3.331102	3.440412	3.359336	35.33702
3.308666	3.351722	3.391597	35.16455
3.185967	3.398965	3.481169	35.19542
3.268577	3.496483	3.356676	35.31709
3.290972	3.385486	3.426348	35.27569
3.423281	3.27213	3.394871	35.2483
3.278888	3.148597	3.376063	34.62126
3.270722	3.239781	3.381383	34.81444
3.329535	3.358326	3.357426	35.14991
3.324874	3.447084	3.3873	35.39914
3.285858	3.365403	3.36394	35.08411
3.159694	3.342489	3.471876	34.99414
3.138825	3.310072	3.403278	34.7276
3.339475	3.341815	3.450664	35.33943
3.278517	3.365403	3.441882	35.23851
3.328586	3.380027	3.47174	35.44528
3.279177	3.316609	3.413236	35.0706
3.330566	3.222797	3.361706	34.86514
3.337577	3.204601	3.279893	34.66177
3.296623	3.242005	3.367793	34.84623
3.104593	3.427607	3.383208	34.86588
3.242635	3.49372	3.387317	35.32132
3.386451	3.441894	3.386755	35.52126
3.322194	3.389327	3.344518	35.17342
3.280744	3.410287	3.477418	35.41924
3.186296	3.447623	3.400345	35.1258
3.244491	3.434144	3.47694	35.39109
3.295097	3.285878	3.283218	34.75388
3.410908	3.170096	3.425513	35.06512
3.216239	3.235063	3.378297	34.67823
3.269155	3.329886	3.40783	35.0659
3.299716	3.456384	3.380787	35.35022
3.377872	3.467639	3.336572	35.44906
3.220817	3.258853	3.391273	34.76865
3.252987	3.36237	3.37313	35.0257
3.219662	3.356709	3.429656	35.06405
3.242181	3.332312	3.39482	34.98377
3.235005	3.383397	3.459463	35.22115
3.377913	3.39883	3.412315	35.46431
3.298149	3.256629	3.428565	35.01445
3.35028	3.231559	3.310517	34.81547
3.258184	3.218349	3.445531	34.88044
3.26569	3.371131	3.362013	35.04832

3.239459	3.362505	3.376694	35.0042
3.252616	3.419991	3.326102	35.04805
3.273733	3.353002	3.413696	35.13929
3.262226	3.433875	3.302895	35.04868
3.290601	3.433807	3.475286	35.48757
3.269897	3.447151	3.378229	35.25923
3.393751	3.360415	3.356318	35.29248
3.233438	3.172387	3.403465	34.63382
3.336752	3.197457	3.360427	34.82046
3.382409	3.25737	3.394462	35.12575
3.328504	3.468515	3.35717	35.38806
3.279507	3.424035	3.467852	35.42568
3.282971	3.394786	3.357631	35.12826
3.235087	3.286215	3.363275	34.79846
3.272207	3.320114	3.326477	34.8733
3.151982	3.345184	3.457416	34.95155
3.196566	3.404423	3.45566	35.17476
3.161138	3.333054	3.436903	34.90019
3.312254	3.270984	3.328796	34.85851
3.258555	3.163356	3.415691	34.69573
3.440232	3.21963	3.38655	35.15237
3.182378	3.276645	3.468619	34.89264
3.331432	3.430977	3.327142	35.24671
3.30516	3.466897	3.399168	35.42532
3.350074	3.432729	3.366446	35.37726
3.212775	3.398291	3.353555	34.9735
3.225313	3.404221	3.400004	35.11547
3.231829	3.443714	3.319043	35.03903
3.387688	3.379488	3.376336	35.36471
3.31147	3.208375	3.3836	34.83972
3.254761	3.138286	3.376387	34.54666
3.297365	3.249822	3.410644	34.95866
3.329329	3.44951	3.375449	35.38828
3.29621	3.466291	3.379133	35.3606
3.223787	3.370592	3.413287	35.06764
3.206135	3.310881	3.413526	34.89898
3.227746	3.404626	3.388528	35.09658
3.241356	3.397078	3.344075	35.01262
3.263793	3.309803	3.359285	34.90409
3.216115	3.322338	3.522144	35.18339
3.1161	3.255349	3.422222	34.59966
3.431489	3.212217	3.407489	35.16283
3.379604	3.24834	3.353197	35.00963
3.302974	3.30495	3.384384	35.03405
3.248203	3.403615	3.38464	35.1306
3.311305	3.394988	3.409757	35.30466
3.340753	3.420463	3.302264	35.18969
3.410702	3.425855	3.416731	35.60477
3.081497	3.433268	3.407643	34.88119
3.228612	3.408939	3.432197	35.2034

3.223209	3.399436	3.43218	35.17077
3.289034	3.24524	3.348542	34.79461
3.190462	3.184585	3.409143	34.57893
3.313656	3.220236	3.428787	34.96926
3.249605	3.438525	3.457604	35.36957
3.409918	3.489946	3.342881	35.58172
3.285652	3.379892	3.389432	35.1711
3.352837	3.309331	3.313143	34.99688
3.161221	3.326988	3.445429	34.90575
3.263545	3.434077	3.353163	35.16193
3.195453	3.406513	3.414105	35.08602
3.252616	3.285608	3.331661	34.76633
3.242181	3.24834	3.492764	35.01432
3.291632	3.263503	3.349514	34.84236
3.32273	3.234794	3.447884	35.0627
3.34661	3.316407	3.354698	35.08961
3.290725	3.324764	3.443519	35.17991
3.255132	3.389732	3.430884	35.21652
3.393421	3.439266	3.346462	35.44264
3.326937	3.398695	3.380019	35.28191
3.363396	3.458743	3.36972	35.47044
3.236077	3.388653	3.442479	35.19785
3.204815	3.41305	3.417652	35.12854
3.21925	3.294437	3.3895	34.83916
3.303634	3.250429	3.34624	34.83285
3.149219	3.169893	3.39378	34.42301
3.286724	3.272467	3.412213	34.98834
3.249853	3.424035	3.494793	35.41975
3.404639	3.423159	3.359762	35.46104
3.303139	3.36547	3.418334	35.241
3.408598	3.33831	3.384981	35.33929
3.23781	3.419924	3.472371	35.33539
3.263298	3.443444	3.354612	35.18505
3.08723	3.363381	3.483215	34.90616
3.291508	3.321529	3.40367	35.08741
3.260535	3.304883	3.444747	35.0731
3.30813	3.225426	3.441132	34.99552
3.305655	3.240589	3.413304	34.96241
3.310934	3.262964	3.475781	35.15951
3.320585	3.374703	3.332531	35.11171
3.321492	3.431179	3.434652	35.46052
3.265154	3.442501	3.333912	35.14177
3.27464	3.393169	3.362763	35.11773
3.378944	3.453014	3.347059	35.44235
3.250925	3.395797	3.386345	35.12319
3.275011	3.366818	3.448055	35.24743
3.165304	3.259257	3.420107	34.71119
3.293199	3.219765	3.386158	34.83027
3.263875	3.237961	3.393865	34.82279
3.311635	3.380297	3.399168	35.25009

3.289281	3.424102	3.387727	35.27198
3.413589	3.408804	3.412025	35.56351
3.282724	3.342084	3.407097	35.12064
3.370118	3.357383	3.389074	35.3058
3.195453	3.410152	3.443792	35.15889
3.176687	3.418374	3.382117	35.00096
3.165386	3.291067	3.388306	34.71139
3.283095	3.242544	3.416083	34.92343
3.325122	3.194627	3.38597	34.84469
3.284992	3.207162	3.452659	34.93019
3.192524	3.324764	3.439512	34.9564
3.342815	3.368031	3.387249	35.26539
3.351023	3.379555	3.395656	35.32692
3.445099	3.445803	3.381008	35.6455
3.288291	3.396471	3.381929	35.19672
3.245027	3.403817	3.371613	35.09561
3.32372	3.438323	3.363837	35.32615
3.342197	3.38926	3.343665	35.21515
3.233149	3.325505	3.401436	34.9636
3.227292	3.227919	3.381707	34.69424
3.254926	3.21781	3.405597	34.7848
3.330236	3.364324	3.393319	35.24305
3.349125	3.396673	3.427457	35.42975
3.238304	3.395932	3.390506	35.10498
3.31444	3.343095	3.409143	35.19669
3.247543	3.350778	3.382065	35.00798
3.393256	3.361628	3.411735	35.41524
3.304954	3.442838	3.419084	35.4158
3.181801	3.332447	3.429793	34.9285
3.128226	3.250564	3.406142	34.58055
3.307222	3.243555	3.410439	34.96606
3.256658	3.201771	3.380207	34.69799
3.309738	3.291809	3.400123	35.05452
3.213682	3.314992	3.460469	35.02713
3.361127	3.380836	3.362337	35.27896
3.470381	3.433672	3.312086	35.52353
3.438294	3.456788	3.358261	35.60489
3.277238	3.412106	3.395195	35.23575
3.260947	3.385081	3.379798	35.10735
3.222055	3.329684	3.380087	34.90179
3.367025	3.331436	3.317321	35.08538
3.2607	3.264716	3.459974	35.01892
3.162953	3.215923	3.446913	34.6699
3.18308	3.288574	3.459463	34.90023
3.390946	3.378477	3.388937	35.39718
3.325204	3.40476	3.440996	35.42474
3.304088	3.394112	3.391324	35.24665
3.227334	3.338849	3.4796	35.15099
3.287467	3.360078	3.362081	35.07192
3.394699	3.429629	3.351987	35.43644

3.258307	3.360281	3.4146	35.12345
3.185472	3.287293	3.425445	34.82827
3.162045	3.196379	3.376711	34.47166
3.295674	3.268086	3.372721	34.91197
3.264205	3.243218	3.368885	34.78038
3.314564	3.356237	3.390779	35.18554
3.191782	3.410893	3.486216	35.24526
3.396473	3.438457	3.364946	35.48797
3.357828	3.409343	3.323255	35.24862
3.490467	3.420598	3.338601	35.59685
3.25241	3.411298	3.414856	35.22268
3.214136	3.353339	3.377547	34.93064
3.182791	3.325236	3.412264	34.87656
3.288951	3.313577	3.334935	34.91411
3.19339	3.262695	3.446026	34.8368
3.196112	3.290326	3.431634	34.87171
3.130205	3.31971	3.475696	34.88819
3.415775	3.440479	3.415742	35.64568
3.381832	3.414263	3.371016	35.41632
3.295179	3.373759	3.407097	35.21715
3.254967	3.378073	3.428565	35.18559
3.296416	3.378746	3.413764	35.24534
3.354363	3.424507	3.317866	35.26242
3.333865	3.276847	3.360836	34.98865
3.131649	3.24315	3.418965	34.59987
3.156684	3.217271	3.401641	34.56014
3.291343	3.273949	3.399373	34.9736
3.287137	3.253125	3.415094	34.95324
3.354735	3.374164	3.415947	35.36763
3.210341	3.331369	3.436903	35.0041
3.321245	3.421878	3.390779	35.3437
3.308872	3.438255	3.351509	35.26657
3.427117	3.479904	3.391683	35.70409
3.259462	3.438323	3.365423	35.1891
3.26734	3.307781	3.329137	34.8415
3.258349	3.242139	3.373011	34.77424
3.359313	3.332717	3.290072	35.01173
3.186255	3.313307	3.405597	34.84347
3.234263	3.328403	3.376728	34.91834
3.174501	3.385958	3.482141	35.14403
3.315388	3.386295	3.361604	35.18927
3.406536	3.362976	3.395502	35.41173
3.402	3.384947	3.436289	35.53905
3.252781	3.381847	3.388238	35.10088
3.319801	3.403884	3.358858	35.23138
3.270309	3.408198	3.391052	35.20299
3.303675	3.251507	3.356966	34.85875
3.222797	3.259931	3.387181	34.76638
3.090364	3.248272	3.452096	34.59324
3.240408	3.244498	3.398964	34.79691

3.302026	3.346937	3.44398	35.25412
3.308377	3.410691	3.419511	35.35392
3.287962	3.344174	3.382083	35.08196
3.310645	3.384475	3.415742	35.29331
3.363107	3.446208	3.337595	35.37214
3.449347	3.417161	3.313842	35.44527
3.309243	3.339591	3.377274	35.10797
3.29984	3.265188	3.338533	34.83997
3.205928	3.253192	3.386635	34.71356
3.289859	3.32436	3.382509	35.04372
3.240367	3.377062	3.392757	35.07315
3.299716	3.399369	3.424558	35.32126
3.141589	3.39108	3.488041	35.09616
3.289776	3.399841	3.434482	35.32226
3.352013	3.360281	3.325045	35.13252
3.424725	3.395123	3.412059	35.55802
3.243212	3.390675	3.398435	35.12156
3.316378	3.335345	3.432453	35.23495
3.249193	3.273949	3.391853	34.86498
3.302232	3.212217	3.311353	34.66993
3.110409	3.269097	3.343	34.44404
3.221601	3.293022	3.395553	34.85444
3.155281	3.315801	3.489149	34.9639
3.301489	3.429696	3.391972	35.3202
3.367603	3.434818	3.422358	35.54243
3.399772	3.358933	3.387727	35.3711
3.321039	3.372344	3.431106	35.32311
3.341825	3.433403	3.353555	35.3325
3.328999	3.385621	3.358057	35.2098
3.3622	3.299828	3.259618	34.87953
3.220075	3.200355	3.327279	34.49915
3.109625	3.248137	3.362303	34.4387
3.275547	3.353676	3.40546	35.12672
3.219044	3.375646	3.379116	34.99359
3.283095	3.429359	3.438455	35.38089
3.21393	3.418576	3.439495	35.20833
3.240408	3.357585	3.453205	35.16283
3.269814	3.406715	3.406893	35.2333
3.494715	3.478489	3.424217	35.91997
3.295138	3.378207	3.398844	35.20874
3.274722	3.287361	3.411172	34.99239
3.251296	3.19247	3.404829	34.71978
3.305119	3.190785	3.369157	34.75578
3.220776	3.24315	3.365423	34.67769
3.247337	3.322675	3.379388	34.94022
3.156106	3.340467	3.452164	34.93877
3.314069	3.439401	3.360836	35.30084
3.309862	3.326719	3.432998	35.20303
3.434994	3.367963	3.337442	35.3579
3.311511	3.443984	3.46983	35.54362

3.326937	3.409276	3.362217	35.26612
3.320626	3.348082	3.407916	35.21844
3.393875	3.277319	3.303816	34.99622
3.253606	3.205949	3.355056	34.64546
3.202794	3.331369	3.371408	34.84437
3.119193	3.321125	3.393729	34.68796
3.247007	3.375377	3.395366	35.08969
3.275712	3.368503	3.42183	35.1953
3.301984	3.336828	3.36232	35.05335
3.317616	3.412713	3.427542	35.39611
3.297324	3.439536	3.45919	35.4796
3.332669	3.411567	3.344279	35.24444
3.330112	3.336356	3.403295	35.20343
3.24247	3.279139	3.354288	34.77948
3.222302	3.195503	3.417533	34.69078
3.315141	3.245711	3.365082	34.8889
3.177924	3.304007	3.418948	34.83411
3.236943	3.304748	3.446026	35.02401
3.181636	3.404828	3.436494	35.10108
3.235459	3.3621	3.375143	34.99117
3.28726	3.330897	3.402664	35.0964
3.405629	3.43064	3.391802	35.54963
3.301613	3.446949	3.396389	35.36786
3.372469	3.396606	3.351032	35.31353
3.31378	3.275432	3.437858	35.11007
3.344919	3.23048	3.334645	34.85415
3.26899	3.276915	3.342898	34.8077
3.289199	3.356304	3.370453	35.08577
3.246017	3.375377	3.394684	35.08603
3.236119	3.368637	3.390046	35.0395
3.20234	3.355361	3.393729	34.94466
3.338361	3.423563	3.395621	35.3954
3.301324	3.448903	3.384726	35.34599
3.309738	3.44149	3.403687	35.38965
3.226385	3.316879	3.417055	34.9641
3.328009	3.331234	3.419766	35.22365
3.288786	3.305355	3.404863	35.0487
3.285281	3.220641	3.287992	34.60019
3.204939	3.202849	3.434721	34.70646
3.196484	3.328066	3.399117	34.88394
3.285652	3.415476	3.415896	35.30678
3.302685	3.4146	3.360154	35.22022
3.339145	3.369311	3.452846	35.40361
3.295757	3.368166	3.428122	35.25216
3.360344	3.426192	3.412758	35.4867
3.450997	3.467167	3.298973	35.52572
3.41759	3.301244	3.364008	35.23203
3.215126	3.218619	3.374887	34.63238
3.296169	3.233041	3.411718	34.92169
3.202505	3.378679	3.305214	34.80244

3.340712	3.451329	3.328165	35.31375
3.16716	3.331706	3.386294	34.79973
3.178873	3.298683	3.394138	34.77029
3.159158	3.37659	3.421079	34.95646
3.358323	3.486981	3.374938	35.53251
3.256617	3.456586	3.498545	35.51393
3.356838	3.365605	3.364519	35.24104
3.184152	3.261549	3.478765	34.88569
3.389503	3.25764	3.328592	34.99781
3.333989	3.27368	3.402186	35.07242
3.320833	3.23412	3.309238	34.75388
3.150827	3.235872	3.406006	34.59755
3.228447	3.370255	3.339692	34.91615
3.270474	3.428011	3.42952	35.3308
3.389132	3.404221	3.337731	35.33753
3.19438	3.310746	3.410968	34.86738
3.239954	3.346869	3.404556	35.03202
3.329576	3.481994	3.435266	35.59066
3.357869	3.350711	3.31964	35.11258
3.353126	3.214373	3.283797	34.72568
3.277279	3.137612	3.4338	34.71998
3.242223	3.334671	3.398759	34.99763
3.254596	3.44277	3.401214	35.26645
3.289776	3.456991	3.346172	35.25411
3.258225	3.293965	3.398708	34.94349
3.206547	3.33056	3.436443	34.99303
3.327308	3.451329	3.435948	35.52014
3.398123	3.488194	3.343938	35.5544
3.31147	3.386227	3.342062	35.13782
3.306686	3.246453	3.413491	34.9779
3.25905	3.277858	3.364127	34.83445
3.354776	3.260336	3.395962	35.07509
3.264783	3.251305	3.340732	34.73776
3.372016	3.226032	3.375637	34.99333
3.123813	3.27341	3.403158	34.61434
3.288869	3.386227	3.388409	35.18975
3.24544	3.422822	3.404505	35.21
3.335433	3.418037	3.344688	35.26553
3.171201	3.364189	3.449777	35.01844
3.329824	3.440883	3.444611	35.52174
3.251791	3.350845	3.371766	34.9949
3.446212	3.277184	3.32675	35.16054
3.317987	3.226639	3.34092	34.80058
3.327308	3.291741	3.429605	35.15727
3.289075	3.448095	3.383907	35.31565
3.272371	3.390203	3.344262	35.06582
3.213146	3.319777	3.340937	34.77502
3.294932	3.334806	3.346973	34.99994
3.196442	3.419857	3.466897	35.23281
3.290477	3.482196	3.408308	35.44665

3.277156	3.366953	3.448822	35.2541
3.329452	3.252383	3.344808	34.89045
3.24746	3.25144	3.369925	34.76401
3.337041	3.274758	3.409842	35.0982
3.267835	3.272736	3.434226	34.99576
3.221725	3.263301	3.348576	34.68699
3.223539	3.32874	3.394956	34.93549
3.332999	3.365739	3.370658	35.20263
3.223292	3.392967	3.416919	35.12343
3.169304	3.390473	3.402459	34.96829
3.258225	3.3683	3.400464	35.10989
3.298479	3.409748	3.40517	35.29885
3.37016	3.393573	3.393848	35.39548
3.344877	3.239174	3.391529	34.99747
3.271175	3.218686	3.357375	34.7168
3.277279	3.272669	3.350605	34.8334
3.438129	3.399099	3.403806	35.57798
3.316791	3.433066	3.391035	35.35898
3.34199	3.334738	3.366412	35.14521
3.207083	3.291404	3.408444	34.84735
3.33766	3.410422	3.358057	35.28298
3.263875	3.549185	3.424967	35.5714
3.218095	3.423833	3.436545	35.22248
3.09519	3.241331	3.470785	34.62948
3.35325	3.210667	3.338993	34.83855
3.332876	3.290663	3.436869	35.18297
3.405464	3.349902	3.392842	35.37498
3.243625	3.275904	3.413219	34.9038
3.181718	3.287698	3.373744	34.70789
3.28957	3.366211	3.336265	35.03348
3.379811	3.423496	3.336845	35.35736
3.207661	3.422283	3.382304	35.07766
3.132556	3.372681	3.423671	34.89541
3.259339	3.37814	3.414242	35.16398
3.320585	3.409613	3.306357	35.13081
3.420807	3.276578	3.352379	35.1597
3.174955	3.212756	3.371135	34.52351
3.202835	3.240657	3.400055	34.70873
3.257606	3.382453	3.387505	35.11115
3.449636	3.450521	3.343461	35.58362
3.303716	3.378477	3.418061	35.27011
3.189678	3.293022	3.327074	34.63488
3.176398	3.313375	3.46439	34.95063
3.379439	3.6	3.367128	35.80876
3.320049	3.451464	3.466164	35.57063
3.267587	3.316003	3.449249	35.12269
3.170253	3.17825	3.428378	34.56295
3.282394	3.264784	3.43218	35.00573
3.354446	3.40038	3.422921	35.43958
3.391194	3.331032	3.413236	35.3471

3.225065	3.223202	3.377632	34.67014
3.36884	3.300165	3.443911	35.2978
3.30549	3.397212	3.335685	35.13482
3.420353	3.412915	3.339368	35.4284
3.229355	3.371738	3.384316	35.01896
3.101995	3.389058	3.427354	34.87244
3.238428	3.419317	3.493634	35.38192
3.365087	3.379623	3.339965	35.23604
3.359395	3.217608	3.36602	34.92627
3.312914	3.249485	3.40089	34.97059
3.176027	3.313442	3.485159	34.99539
3.341372	3.4115	3.288862	35.14214
3.469721	3.375175	3.336435	35.44741
3.282352	3.335817	3.413168	35.1194
3.122369	3.340467	3.434226	34.82576
3.262267	3.522025	3.397412	35.44823
3.331061	3.514949	3.42877	35.65177
3.304129	3.34013	3.322249	34.97763
3.237645	3.220978	3.484153	34.92573
3.096221	3.227785	3.425973	34.50412
3.332876	3.333997	3.408922	35.21662
3.28957	3.337367	3.387982	35.0835
3.40534	3.313442	3.339147	35.17755
3.199659	3.293426	3.465465	34.96023
3.309284	3.337367	3.357648	35.06027
3.344506	3.356641	3.325727	35.10964
3.341289	3.406513	3.363087	35.29337
3.052255	3.380836	3.488365	34.87911
3.272536	3.425316	3.379866	35.22083
3.162252	3.367492	3.440655	34.98614
3.364344	3.284867	3.321021	34.98578
3.394947	3.189505	3.439734	35.10376
3.272784	3.31169	3.396457	35.00917
3.280496	3.429292	3.480419	35.46682
3.381914	3.451734	3.336862	35.42375
3.325782	3.32254	3.387249	35.12866
3.39041	3.320047	3.277079	35.02361
3.142207	3.37477	3.411599	34.89468
3.203124	3.532269	3.429196	35.4108
3.223704	3.393236	3.425786	35.14431
3.198381	3.243487	3.384981	34.67222
3.27167	3.215923	3.386021	34.77449
3.256452	3.278667	3.485722	35.09645
3.275424	3.431246	3.433834	35.35813
3.333412	3.302929	3.431362	35.19893
3.360756	3.28453	3.361246	35.06516
3.248533	3.251507	3.429043	34.89579
3.2866	3.305826	3.313979	34.8462
3.288621	3.359539	3.451073	35.26788
3.320997	3.438053	3.36868	35.3302

3.246966	3.386227	3.394581	35.11161
3.281775	3.316542	3.295972	34.8197
3.195205	3.246116	3.433834	34.77786
3.269031	3.256899	3.39262	34.87275
3.299633	3.36082	3.434124	35.2577
3.349703	3.424979	3.433408	35.50593
3.297612	3.400784	3.396576	35.25856
3.421013	3.343634	3.456342	35.53414
3.268866	3.30367	3.362132	34.908
3.289323	3.358798	3.368885	35.08806
3.185637	3.497764	3.363445	35.15332
3.2866	3.448162	3.402647	35.35136
3.10847	3.212756	3.337987	34.30563
3.204774	3.169556	3.414736	34.58959
3.306067	3.315666	3.429656	35.16325
3.335928	3.440614	3.444065	35.5333
3.327803	3.373085	3.41049	35.29444
3.27266	3.215654	3.382065	34.76741
3.363684	3.243218	3.406142	35.0794
3.3768	3.363852	3.2987	35.13693
3.279012	3.401391	3.383089	35.18972
3.193927	3.420598	3.37163	35.02059
3.265484	3.390877	3.427013	35.2332
3.260122	3.288843	3.299911	34.72039
3.279424	3.221652	3.420466	34.8793
3.22024	3.282576	3.400004	34.83835
3.16353	3.353406	3.473325	35.02958
3.225436	3.41305	3.416424	35.17095
3.40167	3.399234	3.336896	35.35222
3.334319	3.334738	3.330297	35.04946
3.383811	3.324225	3.403602	35.29501
3.255462	3.354822	3.448686	35.17983
3.356879	3.502279	3.382594	35.57955
3.273444	3.449308	3.357733	35.22688
3.30582	3.24679	3.356011	34.85104
3.150951	3.144014	3.427815	34.44464
3.260122	3.290865	3.425854	35.00023
3.186296	3.397482	3.478424	35.1869
3.354116	3.471817	3.383003	35.50778
3.268907	3.278936	3.511794	35.18129
3.340836	3.281902	3.41888	35.14188
3.314893	3.280217	3.400686	35.04168
3.429303	3.4115	3.32143	35.40565
3.276248	3.404423	3.430543	35.29408
3.220281	3.411904	3.282075	34.86337
3.157385	3.321125	3.416714	34.82174
3.212734	3.335682	3.332667	34.79082
3.268701	3.320384	3.362797	34.94565
3.246966	3.303737	3.416407	34.97895
3.187616	3.371198	3.424336	35.01403

3.274227	3.333054	3.44091	35.15626
3.445429	3.404558	3.336777	35.45929
3.412187	3.400784	3.368748	35.44826
3.301201	3.351789	3.421676	35.21415
3.204444	3.400312	3.380565	35.01877
3.33469	3.479904	3.415333	35.55369
3.210919	3.352261	3.395894	34.96137
3.306191	3.187281	3.37782	34.76941
3.098531	3.254472	3.420483	34.55552
3.172356	3.316812	3.432385	34.87932
3.230509	3.486037	3.438762	35.39051
3.27629	3.382925	3.42009	35.2243
3.237686	3.233176	3.377069	34.71832
3.281486	3.230413	3.362405	34.776
3.288869	3.33393	3.478628	35.27268
3.35424	3.418239	3.334372	35.28454
3.344589	3.442703	3.371204	35.39748
3.330236	3.32874	3.42611	35.23694
3.274722	3.22118	3.368407	34.75414
3.295344	3.265795	3.322948	34.79739
3.187987	3.33056	3.367469	34.80161
3.263257	3.482668	3.408529	35.38864
3.21294	3.432122	3.45757	35.27531
3.298602	3.387979	3.365355	35.16445
3.349002	3.342623	3.392569	35.23499
3.385007	3.360685	3.348031	35.25583
3.298809	3.409882	3.383771	35.25307
3.282146	3.400986	3.377018	35.18241
3.28825	3.345858	3.398486	35.12215
3.210836	3.202512	3.306834	34.43896
3.287508	3.162547	3.385612	34.6915
3.214878	3.335412	3.412349	34.96917
3.228901	3.486105	3.484443	35.48703
3.21892	3.414802	3.479652	35.2988
3.358735	3.327325	3.394138	35.22625
3.309367	3.240859	3.358892	34.85213
3.315595	3.296122	3.398776	35.07382
3.286394	3.351317	3.447322	35.23683
3.3078	3.446679	3.402237	35.39359
3.249151	3.389193	3.385391	35.10278
3.432272	3.23695	3.285741	34.95238
3.180192	3.181553	3.391683	34.51166
3.20531	3.288911	3.39291	34.80405
3.159653	3.371738	3.410354	34.92348
3.236366	3.425787	3.419698	35.22987
3.265278	3.422889	3.350571	35.13559
3.403031	3.3466	3.422921	35.42821
3.264164	3.361359	3.374478	35.05087
3.388843	3.409141	3.432061	35.55394
3.354652	3.451397	3.342352	35.3754

3.334195	3.466358	3.338976	35.356
3.269773	3.194357	3.420056	34.79761
3.150827	3.138286	3.370794	34.30714
3.290849	3.277723	3.410405	35.0049
3.305944	3.511445	3.330365	35.37399
3.191741	3.45288	3.544533	35.46452
3.199989	3.412713	3.424183	35.13153
3.293323	3.25737	3.437841	35.0258
3.379357	3.28271	3.351731	35.08105
3.334649	3.412646	3.440808	35.46222
3.247419	3.423496	3.429861	35.27125
3.2282	3.394786	3.372312	35.04059
3.263587	3.230952	3.322879	34.6516
3.355848	3.199412	3.326682	34.7927
3.251296	3.268356	3.368544	34.80637
3.162334	3.385553	3.409689	34.9581
3.186173	3.447488	3.478697	35.29658
3.366035	3.444523	3.373625	35.45365
3.290147	3.358798	3.37504	35.10332
3.404392	3.356911	3.35272	35.30022
3.221601	3.396134	3.491059	35.28879
3.319966	3.476804	3.376933	35.43073
3.388307	3.418913	3.388409	35.47868
3.429674	3.28325	3.337459	35.16105
3.170129	3.128177	3.405034	34.40212
3.21892	3.200625	3.332957	34.50963
3.176769	3.354148	3.411462	34.92487
3.324709	3.451127	3.431242	35.50372
3.17182	3.423024	3.451227	35.15162
3.190916	3.298683	3.448021	34.91446
3.159406	3.250698	3.389483	34.6126
3.339969	3.366616	3.415964	35.31887
3.27266	3.467167	3.394462	35.34454
3.304005	3.449106	3.41772	35.42445
3.285941	3.271321	3.404403	34.96704
3.295757	3.134916	3.35567	34.58364
3.370448	3.210734	3.280779	34.749
3.275712	3.297739	3.354834	34.89404
3.214796	3.465752	3.413253	35.256
3.214012	3.454362	3.410917	35.22427
3.351146	3.366279	3.397003	35.30111
3.312295	3.348082	3.295375	34.95411
3.331721	3.393977	3.426672	35.38408
3.197102	3.444658	3.393217	35.12736
3.292498	3.411163	3.419835	35.32094
3.322152	3.28116	3.329734	34.90446
3.298685	3.166861	3.368151	34.68719
3.235706	3.203658	3.401828	34.70358
3.14794	3.381173	3.428173	34.95746
3.22721	3.49217	3.436682	35.39215

3.338773	3.436638	3.384691	35.40099
3.203454	3.276982	3.363036	34.70857
3.273279	3.196851	3.370334	34.70199
3.182337	3.344713	3.430458	34.95795
3.319678	3.435896	3.398981	35.38886
3.323802	3.472693	3.43455	35.55613
3.443284	3.33986	3.361808	35.36786
3.242429	3.15008	3.433902	34.67126
3.346527	3.147384	3.270037	34.53466
3.22457	3.273478	3.387641	34.80089
3.302026	3.44304	3.342847	35.24312
3.200154	3.409343	3.424677	35.12561
3.177223	3.427809	3.370726	34.99786
3.25905	3.311622	3.3777	34.93797
3.330154	3.401323	3.339078	35.20517
3.295427	3.466224	3.430816	35.47176
3.386698	3.47667	3.430679	35.69391
3.216734	3.288169	3.392569	34.82666
3.307428	3.134377	3.31657	34.52248
3.325287	3.161604	3.328984	34.64822
3.294973	3.324225	3.373182	35.03421
3.20531	3.492305	3.411326	35.28911
3.214507	3.385351	3.366907	34.97819
3.27629	3.351924	3.404625	35.12268
3.239624	3.317216	3.364042	34.87785
3.300664	3.336423	3.43854	35.21626
3.137712	3.447758	3.463606	35.15819
3.241686	3.453486	3.395468	35.24909
3.261689	3.323484	3.427457	35.07849
3.435695	3.203253	3.311267	34.942
3.278434	3.155269	3.368134	34.61752
3.243707	3.242881	3.398844	34.80033
3.228282	3.410152	3.427815	35.19575
3.389874	3.486576	3.312222	35.46347
3.306191	3.392967	3.385049	35.23502
3.264618	3.309398	3.399884	34.9938
3.226055	3.38306	3.407421	35.08703
3.282806	3.454901	3.38418	35.31742
3.341908	3.473974	3.475576	35.68824
3.338155	3.360955	3.366907	35.19524
3.217518	3.181216	3.422648	34.66026
3.330896	3.192133	3.381452	34.84199
3.290807	3.323214	3.393797	35.06797
3.364757	3.53638	3.399663	35.70867
3.264618	3.399167	3.39835	35.18675
3.144228	3.334671	3.443042	34.88017
3.19273	3.302187	3.361859	34.73767
3.269196	3.305557	3.38198	34.95625
3.298561	3.384475	3.470239	35.38606
3.242635	3.391147	3.472371	35.28301

3.203206	3.352867	3.502449	35.17885
3.28858	3.263773	3.295563	34.71829
3.47133	3.246048	3.335924	35.16744
3.278847	3.230076	3.36706	34.77967
3.178708	3.400986	3.383089	34.96948
3.288333	3.435492	3.366429	35.24824
3.383605	3.416554	3.336435	35.34958
3.357869	3.386429	3.312205	35.17444
3.316337	3.361561	3.462344	35.35756
3.132226	3.410624	3.420875	34.97154
3.262886	3.417296	3.41431	35.25751
3.298809	3.320653	3.400294	35.09407
3.346403	3.188427	3.389909	34.88629
3.182915	3.124942	3.387982	34.38572
3.367726	3.224213	3.394956	35.02221
3.241233	3.458945	3.422222	35.31854
3.379604	3.507805	3.36336	35.59927
3.154292	3.318766	3.461526	34.90782
3.271423	3.335817	3.362541	34.98479
3.114739	3.332447	3.437227	34.7981
3.248533	3.356304	3.336026	34.92155
3.23385	3.414802	3.484443	35.34193
3.258885	3.420328	3.391342	35.20516
3.223003	3.25892	3.446708	34.8948
3.310852	3.232502	3.379815	34.88285
3.32306	3.194762	3.398981	34.86893
3.361127	3.330964	3.335412	35.11102
3.245151	3.430977	3.421028	35.26334
3.270845	3.423631	3.334355	35.11392
3.323307	3.381779	3.400942	35.28274
3.275671	3.39081	3.329018	35.04103
3.192937	3.403615	3.436494	35.12314
3.256039	3.448903	3.40812	35.29812
3.230922	3.379623	3.459207	35.20341
3.307552	3.252316	3.443246	35.05768
3.391441	3.223606	3.292596	34.8489
3.239995	3.270512	3.383685	34.81949
3.340093	3.390338	3.356352	35.24065
3.299551	3.486172	3.512527	35.7031
3.291426	3.408602	3.313791	35.08109
3.244862	3.336019	3.390796	34.98893
3.257565	3.313105	3.397685	34.98167
3.064793	3.335615	3.4914	34.81427
3.2355	3.426933	3.439001	35.27269
3.189101	3.369177	3.421472	35.00659
3.262143	3.286148	3.427815	34.99862
3.241191	3.247127	3.409962	34.82843
3.303222	3.206151	3.470461	35.00677
3.217188	3.380836	3.384589	35.01285
3.392266	3.43967	3.392706	35.54213

3.321286	3.453621	3.358108	35.34176
3.413671	3.339052	3.345575	35.26583
3.199247	3.357652	3.337169	34.81922
3.222178	3.406108	3.412383	35.13981
3.137299	3.504234	3.370658	35.07753
3.310893	3.349565	3.30576	34.977
3.184317	3.176768	3.378092	34.48049
3.269814	3.174746	3.38684	34.68217
3.2607	3.277656	3.447816	35.02063
3.386533	3.492709	3.319367	35.4852
3.342774	3.447623	3.436613	35.54731
3.251709	3.35408	3.42495	35.11809
3.254224	3.292954	3.413833	34.96561
3.218384	3.323214	3.338857	34.78945
3.233231	3.363381	3.448362	35.14922
3.21991	3.390945	3.378706	35.02804
3.13301	3.367559	3.486557	35.02272
3.138867	3.239309	3.35156	34.45985
3.37515	3.212082	3.392842	35.0073
3.269897	3.207769	3.413526	34.81292
3.277527	3.304479	3.385817	34.9805
3.264659	3.390338	3.429742	35.23618
3.347764	3.444186	3.351645	35.36489
3.378408	3.403547	3.393729	35.43507
3.430004	3.402132	3.297149	35.3336
3.141589	3.358596	3.500949	35.05335
3.281321	3.449914	3.279603	35.07457
3.226179	3.408467	3.499585	35.34441
3.318069	3.352126	3.335685	35.06373
3.230015	3.082079	3.418965	34.46274
3.23983	3.230211	3.391614	34.74834
3.20564	3.342556	3.451295	35.04976
3.409135	3.454699	3.408734	35.64693
3.268825	3.355765	3.369788	35.03858
3.309367	3.31971	3.39141	35.09567
3.251915	3.320181	3.365491	34.91439
3.24049	3.362707	3.384862	35.02476
3.313244	3.400178	3.330382	35.14666
3.248986	3.393304	3.402732	35.14933
3.19306	3.38279	3.43854	35.08234
3.231252	3.246116	3.429315	34.8468
3.331844	3.197525	3.346394	34.77919
3.349909	3.26694	3.39407	35.07475
3.299345	3.339591	3.433288	35.20881
3.250636	3.457867	3.450886	35.39943
3.343145	3.446342	3.338141	35.32997
3.326607	3.330897	3.335924	35.0365
3.401216	3.380566	3.429384	35.51266
3.260906	3.475996	3.36527	35.2743
3.200608	3.431381	3.445889	35.22118

3.163571	3.277858	3.339402	34.57159
3.324132	3.224819	3.402715	34.94517
3.165881	3.198873	3.369021	34.46868
3.313821	3.34478	3.421949	35.22702
3.299221	3.456384	3.442769	35.48468
3.387812	3.445466	3.403278	35.56818
3.344506	3.355833	3.369243	35.20304
3.325411	3.331773	3.373267	35.11746
3.255214	3.345117	3.426792	35.11019
3.232613	3.378275	3.394632	35.06294
3.150538	3.397347	3.419971	34.98058
3.292952	3.351519	3.373932	35.09112
3.261648	3.265323	3.442155	34.98336
3.227375	3.226571	3.415214	34.76475
3.147074	3.288978	3.46144	34.82671
3.303263	3.353676	3.441592	35.26634
3.286477	3.3683	3.478969	35.34335
3.332587	3.431381	3.322504	35.23997
3.271464	3.358528	3.451175	35.22837
3.305861	3.398762	3.282348	35.02238
3.374366	3.365672	3.340153	35.22624
3.285858	3.464471	3.356062	35.28353
3.295798	3.38124	3.389688	35.19679
3.200154	3.194223	3.354101	34.50083
3.269443	3.188022	3.442411	34.83192
3.263422	3.307714	3.393575	34.9737
3.30483	3.474513	3.407302	35.45903
3.211208	3.38899	3.43779	35.13395
3.284827	3.311825	3.393712	35.0298
3.319554	3.295111	3.353129	34.98044
3.358323	3.407658	3.42362	35.4655
3.23418	3.393236	3.445412	35.21014
3.223828	3.375646	3.377683	35.00092
3.155818	3.281902	3.438677	34.78057
3.340753	3.301176	3.319026	34.96549
3.162623	3.23722	3.397992	34.60877
3.318688	3.320249	3.368629	35.06742
3.127318	3.31917	3.517301	34.97169
3.301407	3.416487	3.33028	35.15622
3.422621	3.394382	3.408529	35.54408
3.504819	3.432998	3.35231	35.68534
3.334237	3.330156	3.433749	35.26549
3.290519	3.382925	3.292101	34.97552
3.246759	3.347206	3.39823	35.03381
3.399772	3.371064	3.323118	35.25634
3.266144	3.262762	3.403823	34.90376
3.058731	3.215452	3.431975	34.40829
3.260122	3.254338	3.431924	34.93362
3.306439	3.435694	3.439546	35.44818
3.313739	3.507064	3.383907	35.49854

3.267752	3.378949	3.434857	35.22923
3.259504	3.285271	3.426741	34.98858
3.283672	3.354822	3.435948	35.21367
3.466009	3.402132	3.346377	35.51999
3.230922	3.303535	3.422495	34.95673
3.220322	3.275297	3.315769	34.63841
3.167613	3.225021	3.458081	34.72441
3.350569	3.244296	3.346308	34.92223
3.2826	3.273275	3.408052	34.97199
3.233933	3.354148	3.435488	35.10241
3.182378	3.377062	3.434175	35.03691
3.312501	3.443781	3.412878	35.4208
3.370696	3.42909	3.370811	35.42394
3.53435	3.423159	3.397395	35.82699
3.320709	3.379892	3.403977	35.27957
3.293199	3.368031	3.378894	35.13862
3.323101	3.368974	3.391137	35.23284
3.345991	3.25919	3.362763	34.98077
3.295839	3.174881	3.33826	34.63314
3.12307	3.20979	3.446776	34.56897
3.138702	3.360955	3.465328	34.9743
3.340877	3.487116	3.431225	35.61774
3.320214	3.364931	3.403244	35.24416
3.366324	3.341613	3.436699	35.36717
3.27266	3.32901	3.456871	35.17889
3.345084	3.460158	3.395349	35.48953
3.35758	3.369918	3.350537	35.22152
3.309862	3.278869	3.384163	34.99159
3.134	3.279139	3.445838	34.74248
3.215497	3.22246	3.322931	34.52797
3.337701	3.226908	3.38701	34.94507
3.230386	3.332447	3.351628	34.86381
3.242841	3.33986	3.496976	35.22511
3.181141	3.349093	3.385544	34.86669
3.28029	3.399571	3.391717	35.2074
3.361911	3.458675	3.376848	35.48263
3.473557	3.388788	3.372022	35.5634
3.260328	3.372344	3.389636	35.09966
3.303716	3.394314	3.385732	35.23405
3.281115	3.31479	3.409894	35.06355
3.325204	3.263773	3.269184	34.74069
3.236696	3.210599	3.380889	34.67514
3.213558	3.358461	3.395417	34.97966
3.263545	3.408737	3.453034	35.32492
3.307016	3.408063	3.389773	35.28017
3.273361	3.375579	3.446793	35.26023
3.346445	3.360078	3.408103	35.30154
3.256534	3.375309	3.456069	35.24312
3.335804	3.506458	3.308181	35.37987
3.281569	3.277049	3.438472	35.04451

3.309491	3.247396	3.371835	34.895
3.248863	3.203995	3.397599	34.72385
3.179491	3.304142	3.363036	34.71556
3.18708	3.310679	3.481612	35.00576
3.216157	3.380903	3.413833	35.07469
3.281363	3.370457	3.457297	35.28949
3.277114	3.461371	3.404369	35.36327
3.294643	3.474715	3.434516	35.49671
3.336629	3.428685	3.3895	35.38943
3.416022	3.395056	3.336094	35.37272
3.371026	3.298076	3.358517	35.11127
3.245769	3.288708	3.309937	34.71063
3.279342	3.245442	3.435096	34.96313
3.325287	3.302052	3.342506	34.98493
3.232489	3.302524	3.376114	34.85652
3.333659	3.422215	3.355363	35.29413
3.15524	3.40038	3.443264	35.04843
3.311635	3.368166	3.409348	35.24583
3.182172	3.397886	3.478577	35.1791
3.363148	3.450184	3.39453	35.50543
3.28726	3.439401	3.410166	35.35009
3.421631	3.290596	3.404147	35.30536
3.223581	3.184113	3.446094	34.73113
3.316956	3.210127	3.325778	34.7291
3.21426	3.195436	3.388528	34.60962
3.294725	3.263638	3.391938	34.94219
3.116183	3.371198	3.392228	34.7876
3.186585	3.433403	3.37828	35.04708
3.30549	3.446949	3.382628	35.34624
3.386822	3.466965	3.408103	35.62358
3.31877	3.454227	3.393797	35.41563
3.329329	3.433335	3.411872	35.43255
3.28029	3.323214	3.376745	35.00768
3.407402	3.339052	3.401777	35.37503
3.288786	3.265188	3.301565	34.73496
3.232654	3.196514	3.289902	34.43652
3.224075	3.220641	3.386823	34.68248
3.212981	3.368031	3.390523	34.98862
3.237273	3.453823	3.4899	35.44668
3.293447	3.431718	3.37475	35.26937
3.240985	3.375377	3.512612	35.33292
3.325163	3.407524	3.386976	35.31256
3.461596	3.437042	3.444048	35.80027
3.298561	3.384408	3.377837	35.18384
3.302892	3.259055	3.38788	34.94115
3.277073	3.195773	3.408649	34.79172
3.282847	3.220439	3.369021	34.77163
3.268247	3.220169	3.34844	34.6941
3.299345	3.339321	3.371545	35.0732
3.084425	3.368368	3.480606	34.90523

3.257318	3.363381	3.415947	35.13101
3.261442	3.460158	3.428719	35.3796
3.313986	3.502616	3.412741	35.55241
3.285899	3.433807	3.42761	35.37303
3.372964	3.37012	3.422904	35.41386
3.226179	3.334604	3.403022	34.97172
3.356013	3.315464	3.334116	35.0631
3.254472	3.289315	3.381247	34.88693
3.288828	3.290191	3.300406	34.78719
3.16188	3.326449	3.428241	34.86843
3.245605	3.448971	3.400959	35.25979
3.282847	3.405637	3.408427	35.2628
3.285817	3.389193	3.365611	35.1397
3.252533	3.382655	3.465669	35.27143
3.289817	3.412713	3.365491	35.19962
3.282971	3.418104	3.418061	35.31141
3.371768	3.391214	3.367742	35.33675
3.358818	3.17178	3.374631	34.84362
3.239459	3.207903	3.420875	34.76273
3.259957	3.280352	3.407711	34.9372
3.235087	3.297672	3.389619	34.88113
3.302562	3.369514	3.398776	35.20581
3.274145	3.30057	3.38834	34.97008
3.177017	3.339321	3.405801	34.8806
3.171243	3.421811	3.439836	35.1228
3.372057	3.466089	3.360086	35.48437
3.285734	3.43192	3.39985	35.30784
3.365417	3.3528	3.359336	35.22047
3.309037	3.25582	3.435454	35.05155
3.327555	3.282441	3.377888	35.02438
3.2866	3.300435	3.377496	34.97331
3.427282	3.390001	3.37475	35.47082
3.175285	3.375175	3.44311	35.03681
3.275011	3.412241	3.359694	35.15354
3.189555	3.37194	3.445139	35.06538
3.337165	3.413387	3.412895	35.4083
3.272	3.434414	3.463146	35.42167
3.278723	3.388451	3.481033	35.37498
3.199412	3.327123	3.49256	35.09263
3.357127	3.245037	3.389278	35.03216
3.403773	3.180609	3.341994	34.88987
3.338196	3.26101	3.394598	35.03732
3.217641	3.333458	3.334253	34.80016
3.17281	3.337704	3.35804	34.76342
3.223787	3.447488	3.364588	35.1293
3.374201	3.377938	3.392569	35.36733
3.209104	3.452543	3.42599	35.24252
3.241728	3.412848	3.397054	35.16378
3.289364	3.376927	3.372585	35.13589
3.404309	3.315666	3.339266	35.18042

3.300912	3.28325	3.386618	34.98697
3.210836	3.244566	3.373795	34.67735
3.251379	3.295044	3.410985	34.95773
3.236407	3.398762	3.355994	35.03155
3.379357	3.385149	3.367179	35.33885
3.274434	3.378746	3.367248	35.09554
3.166376	3.389866	3.41651	34.99129
3.178955	3.422552	3.374375	34.99813
3.33469	3.445197	3.400618	35.44561
3.23286	3.358798	3.4109	35.05647
3.303139	3.304411	3.41813	35.10703
3.246429	3.241533	3.416646	34.84227
3.36653	3.225898	3.357648	34.9417
3.357663	3.221854	3.385135	34.97357
3.360055	3.292415	3.37516	35.11129
3.240614	3.31452	3.446435	35.0543
3.237315	3.403615	3.346683	35.02378
3.217394	3.363381	3.406705	35.02349
3.386286	3.415072	3.305709	35.28501
3.222591	3.425383	3.472984	35.31539
3.245027	3.443175	3.35937	35.1549
3.251832	3.344376	3.437159	35.12384
3.415156	3.300907	3.323391	35.13715
3.318935	3.256225	3.377905	34.94823
3.243419	3.277723	3.381759	34.83853
3.241686	3.317081	3.437926	35.04364
3.259957	3.320114	3.383805	34.97187
3.395483	3.368772	3.311131	35.21573
3.265608	3.367222	3.422563	35.17201
3.195741	3.379623	3.404591	35.00704
3.225436	3.414667	3.419852	35.18199
3.332628	3.456788	3.390591	35.44452
3.263504	3.357854	3.423057	35.148
3.290065	3.264649	3.341261	34.82339
3.181141	3.228122	3.489473	34.82942
3.345909	3.288843	3.366156	35.05286
3.253771	3.250833	3.377052	34.79207
3.384059	3.309668	3.360171	35.16874
3.249399	3.322675	3.438677	35.07438
3.261195	3.387103	3.376677	35.10549
3.172109	3.35408	3.388443	34.86419
3.340464	3.478017	3.36556	35.45334
3.190586	3.499112	3.439018	35.33235
3.290312	3.44277	3.408853	35.36126
3.201227	3.293224	3.437517	34.9021
3.368056	3.231896	3.390796	35.03064
3.44382	3.269838	3.394274	35.2869
3.267711	3.342489	3.397821	35.06841
3.263958	3.427135	3.426945	35.309
3.279094	3.364459	3.39112	35.1267

3.356632	3.323753	3.324687	35.06196
3.338443	3.358326	3.364929	35.1858
3.321286	3.434683	3.424831	35.44625
3.16518	3.454093	3.455524	35.21444
3.262886	3.405367	3.44814	35.3054
3.229973	3.319507	3.439819	35.02747
3.236284	3.243689	3.393456	34.77408
3.246388	3.229604	3.418419	34.81997
3.257194	3.232435	3.428429	34.87168
3.2793	3.335817	3.365253	35.00794
3.375934	3.366413	3.425837	35.41866
3.286436	3.410961	3.394019	35.25078
3.318688	3.407726	3.379269	35.28198
3.175779	3.329212	3.394837	34.83181
3.319348	3.442905	3.334151	35.26169
3.218508	3.465347	3.367333	35.16281
3.333329	3.353339	3.376353	35.18869
3.25175	3.229874	3.44311	34.88628
3.277362	3.232502	3.371084	34.79053
3.360344	3.298548	3.424541	35.23333
3.449512	3.38279	3.379593	35.51425
3.349414	3.382318	3.440979	35.42856
3.297365	3.380903	3.414054	35.25277
3.247378	3.321664	3.381452	34.94261
3.352013	3.416487	3.403687	35.42742
3.273196	3.504301	3.414463	35.47066
3.132474	3.423631	3.499517	35.17251
3.164809	3.315599	3.481834	34.9683
3.293447	3.276982	3.350793	34.8786
3.270722	3.266064	3.421199	34.95899
3.25076	3.241735	3.326409	34.65484
3.26701	3.235804	3.507838	35.07417
3.21228	3.325371	3.330894	34.7634
3.421178	3.388316	3.354561	35.40964
3.365499	3.397415	3.348576	35.29468
3.290271	3.329751	3.409757	35.116
3.165757	3.402065	3.387982	34.95422
3.328545	3.43347	3.453341	35.52182
3.294725	3.470469	3.366514	35.3389
3.341949	3.322944	3.357426	35.09968
3.184606	3.176228	3.428514	34.59021
3.281321	3.314385	3.3917	35.02333
3.27662	3.410826	3.405716	35.2546
3.476568	3.424574	3.324823	35.54502
3.338031	3.283519	3.419579	35.14082
3.287425	3.323079	3.337527	34.93723
3.285899	3.382992	3.443178	35.29595
3.320668	3.500325	3.295034	35.3046
3.253729	3.398493	3.389432	35.14196
3.229602	3.337502	3.373795	34.92163

3.144104	3.263571	3.457229	34.75544
3.275712	3.295583	3.421148	35.03435
3.284786	3.256831	3.415828	34.95781
3.33601	3.199749	3.414327	34.94172
3.28359	3.240522	3.460298	35.01678
3.298066	3.360955	3.397412	35.17428
3.325287	3.408669	3.377155	35.29385
3.377253	3.419452	3.363582	35.4014
3.213476	3.342691	3.392433	34.93847
3.125174	3.404895	3.362183	34.81525
3.333824	3.470941	3.413833	35.52891
3.23682	3.38461	3.387027	35.06937
3.275753	3.267345	3.393251	34.91168
3.205186	3.23412	3.410013	34.72136
3.245769	3.31789	3.416373	35.00721
3.293942	3.361359	3.395434	35.16182
3.423157	3.38124	3.397207	35.49175
3.328628	3.315059	3.404761	35.15682
3.291426	3.336491	3.364809	35.03496
3.271382	3.418981	3.434294	35.32348
3.319224	3.477613	3.358381	35.39031
3.194256	3.439873	3.393797	35.11194
3.25542	3.347813	3.35052	34.94974
3.228365	3.253394	3.421267	34.83881
3.254472	3.224752	3.432112	34.85698
3.253853	3.214238	3.455984	34.88484
3.414867	3.247194	3.333741	35.04169
3.286889	3.266266	3.483966	35.13205
3.290601	3.394314	3.374819	35.1815
3.309449	3.358124	3.418897	35.23997
3.372057	3.327595	3.305385	35.06189
3.238222	3.355496	3.375654	34.98389
3.163283	3.412174	3.422716	35.04688
3.174336	3.398695	3.475661	35.15735
3.249193	3.337839	3.396866	35.01566
3.320296	3.277049	3.348559	34.93257
3.254472	3.250361	3.42686	34.9015
3.333412	3.396943	3.421727	35.38345
3.20564	3.387171	3.446913	35.13774
3.376222	3.392427	3.310841	35.22471
3.335391	3.323955	3.381724	35.14069
3.267629	3.413185	3.401811	35.23156
3.251172	3.403076	3.432675	35.24096
3.32706	3.441625	3.415623	35.45392
3.160643	3.407995	3.395178	34.97174
3.264865	3.309264	3.348031	34.88065
3.122905	3.250901	3.480606	34.7325
3.237356	3.288843	3.424268	34.94255
3.165799	3.297267	3.447475	34.85524
3.331597	3.26411	3.410422	35.06428

3.329782	3.329077	3.427133	35.23892
3.413878	3.406378	3.378229	35.48493
3.256575	3.371872	3.451158	35.22496
3.341578	3.390945	3.375978	35.28815
3.22886	3.382251	3.42258	35.12455
3.246553	3.387845	3.393251	35.11134
3.209888	3.309331	3.372636	34.81438
3.256204	3.239983	3.408461	34.84235
3.266102	3.272601	3.411053	34.941
3.254637	3.308859	3.407609	34.98768
3.359973	3.413185	3.404488	35.43935
3.296004	3.372951	3.378809	35.15532
3.28161	3.351182	3.400413	35.12349
3.373129	3.368907	3.346513	35.24452
3.405258	3.444253	3.368816	35.52832
3.183368	3.406041	3.412844	35.0558
3.271382	3.363583	3.4161	35.16254
3.198999	3.26539	3.438455	34.83841
3.229643	3.257168	3.333724	34.65841
3.214796	3.234861	3.446589	34.82398
3.223663	3.25582	3.456393	34.91064
3.1307	3.266738	3.413781	34.63804
3.396349	3.314722	3.385186	35.26137
3.297984	3.424979	3.433681	35.39342
3.458792	3.431785	3.34919	35.5752
3.263545	3.333862	3.367606	34.97436
3.334773	3.319844	3.307039	34.96702
3.242759	3.338378	3.302332	34.79604
3.313821	3.457328	3.374631	35.36967
3.194256	3.289585	3.412963	34.8252
3.237686	3.238433	3.341056	34.65106
3.210094	3.250631	3.40783	34.76342
3.310274	3.389664	3.397821	35.26466
3.35292	3.366616	3.416697	35.34879
3.307428	3.38744	3.438182	35.34183
3.293117	3.303805	3.39378	35.03054
3.28792	3.4084	3.429401	35.3258
3.276372	3.485363	3.405733	35.4171
3.307346	3.431381	3.454501	35.47343
3.203784	3.284934	3.420721	34.85283
3.228901	3.252922	3.419494	34.83507
3.239171	3.338041	3.379593	34.95641
3.259916	3.367222	3.450886	35.2215
3.303304	3.297402	3.464919	35.19438
3.192565	3.224145	3.378246	34.60248
3.365912	3.280689	3.434925	35.22916
3.468814	3.37349	3.272509	35.30195
3.376965	3.370457	3.383481	35.33714
3.208444	3.450521	3.430611	35.24676
3.312666	3.380364	3.443519	35.34948

3.242429	3.36547	3.336214	34.92866
3.369046	3.267749	3.310858	34.9364
3.120183	3.253057	3.386141	34.52468
3.149384	3.297941	3.434516	34.79248
3.161262	3.340265	3.454364	34.95441
3.29918	3.426731	3.380616	35.28383
3.299757	3.34323	3.41298	35.17326
3.399277	3.277521	3.343239	35.09469
3.143486	3.289517	3.465959	34.82992
3.349043	3.499786	3.34844	35.48227
3.422168	3.477209	3.428974	35.76892
3.303881	3.31762	3.323868	34.93141
3.141424	3.244903	3.416373	34.61941
3.178254	3.291404	3.409416	34.78643
3.219951	3.410826	3.43056	35.18501
3.337206	3.448499	3.417942	35.49622
3.281569	3.275702	3.452591	35.07244
3.228489	3.283519	3.510907	35.10098
3.302397	3.306029	3.372585	35.00934
3.421095	3.354957	3.399305	35.43435
3.379109	3.429359	3.422512	35.55599
3.245976	3.379892	3.44855	35.21361
3.251214	3.313914	3.373795	34.91731
3.245275	3.289517	3.361723	34.82457
3.355106	3.326988	3.296518	35.0041
3.216817	3.29747	3.426041	34.92038
3.212197	3.438862	3.389892	35.14043
3.164314	3.474109	3.468056	35.28372
3.428725	3.425046	3.334816	35.46328
3.385832	3.352463	3.447612	35.45742
3.294272	3.369177	3.37741	35.14022
3.209723	3.448971	3.461389	35.31347
3.313202	3.480376	3.42333	35.52522
3.263463	3.304411	3.41651	35.01672
3.327514	3.142329	3.365798	34.69145
3.104552	3.237759	3.439597	34.57394
3.192236	3.37659	3.403278	34.98987
3.225725	3.469728	3.410388	35.28233
3.283796	3.319036	3.466982	35.20354
3.261689	3.263301	3.418829	34.92801
3.337412	3.340736	3.449453	35.32992
3.23748	3.399841	3.459139	35.26181
3.390616	3.394921	3.405307	35.46822
3.285033	3.389462	3.373045	35.15484
3.367685	3.346937	3.417328	35.33943
3.243955	3.296796	3.302571	34.70824
3.297159	3.248946	3.321754	34.7619
3.160066	3.237894	3.357034	34.51508
3.257483	3.350441	3.368612	34.99956
3.19038	3.41925	3.4877	35.26372

3.188647	3.440681	3.368066	35.04518
3.346115	3.376522	3.42686	35.3778
3.389956	3.338175	3.359557	35.24263
3.272866	3.396471	3.44664	35.3045
3.229355	3.553161	3.433357	35.52295
3.25208	3.392899	3.516977	35.40504
3.216363	3.171241	3.370334	34.52152
3.348919	3.163289	3.328268	34.70202
3.197267	3.271995	3.40986	34.78653
3.270928	3.448971	3.409416	35.33366
3.130824	3.39081	3.458934	35.00838
3.281651	3.258381	3.297336	34.69522
3.236861	3.31014	3.41801	34.97436
3.300871	3.365537	3.376677	35.14509
3.222632	3.396201	3.449948	35.20129
3.376181	3.458541	3.365423	35.48856
3.289735	3.339995	3.4028	35.12201
3.44679	3.236546	3.306152	35.02788
3.33832	3.215654	3.395775	34.94098
3.206547	3.250698	3.321549	34.56713
3.194751	3.373692	3.408802	35.00111
3.298561	3.405434	3.395894	35.26932
3.257978	3.457193	3.36249	35.2207
3.313079	3.348217	3.418539	35.22546
3.17912	3.318092	3.450067	34.93558
3.305861	3.375714	3.347638	35.11476
3.421796	3.489946	3.421779	35.78023
3.316956	3.433875	3.443434	35.4757
3.281857	3.194964	3.343785	34.65857
3.271794	3.15318	3.391734	34.65004
3.268618	3.311218	3.369072	34.93914
3.355807	3.486172	3.379661	35.53556
3.200402	3.447623	3.418812	35.19703
3.126411	3.274017	3.456393	34.73776
3.242594	3.223404	3.423006	34.80814
3.321369	3.343769	3.429179	35.25713
3.303345	3.441018	3.452642	35.48169
3.246594	3.479298	3.392893	35.31064
3.329494	3.377062	3.474349	35.44648
3.294808	3.225965	3.311404	34.68387
3.393751	3.200018	3.321771	34.86617
3.234922	3.249822	3.343682	34.67567
3.225808	3.467774	3.40471	35.26582
3.282064	3.527687	3.410252	35.53198
3.35725	3.430168	3.299877	35.24177
3.298066	3.345724	3.319077	34.96967
3.251255	3.323618	3.342915	34.87109
3.133051	3.380431	3.488945	35.05618
3.270969	3.449712	3.38921	35.29119
3.221147	3.300907	3.40014	34.88072

3.415321	3.259729	3.348031	35.10135
3.222096	3.164704	3.432385	34.65546
3.249069	3.26566	3.396917	34.85766
3.211826	3.283587	3.408103	34.83988
3.320915	3.348285	3.359063	35.11268
3.16918	3.35253	3.447271	34.98304
3.232819	3.35772	3.368595	34.9615
3.161674	3.281767	3.455848	34.83063
3.276001	3.258044	3.391938	34.88901
3.351394	3.379218	3.425786	35.39289
3.349909	3.414398	3.421625	35.45748
3.239336	3.32746	3.446589	35.08014
3.335763	3.269838	3.359626	34.97483
3.292086	3.197525	3.372022	34.74829
3.294849	3.299694	3.322521	34.86951
3.170253	3.443984	3.393712	35.06825
3.192607	3.464269	3.396576	35.16776
3.348136	3.43657	3.380565	35.41229
3.28825	3.354754	3.403414	35.15238
3.309078	3.389597	3.417396	35.3047
3.276372	3.389934	3.424899	35.25032
3.274599	3.42545	3.46896	35.42047
3.21628	3.324495	3.417192	34.95895
3.4531	3.278128	3.371187	35.27484
3.236531	3.256494	3.383498	34.78085
3.315883	3.313914	3.391597	35.09766
3.276702	3.485565	3.438745	35.49045
3.330195	3.408871	3.426263	35.41242
3.27629	3.346398	3.339215	34.96756
3.302685	3.336693	3.433663	35.2106
3.085415	3.352396	3.472967	34.85576
3.270309	3.455508	3.432385	35.39683
3.235706	3.486846	3.419562	35.36165
3.386863	3.348352	3.38568	35.31525
3.237892	3.222663	3.394735	34.73441
3.313945	3.189303	3.38094	34.79761
3.217064	3.240185	3.365014	34.66219
3.33436	3.513332	3.342676	35.46718
3.23418	3.482061	3.372977	35.24598
3.365623	3.433605	3.339147	35.35348
3.135072	3.343297	3.396525	34.77729
3.228076	3.345589	3.376865	34.94269
3.221023	3.40476	3.453546	35.22435
3.291467	3.399099	3.387113	35.22074
3.301324	3.249148	3.388664	34.91778
3.324627	3.198873	3.375108	34.82914
3.305779	3.27341	3.429588	35.07007
3.324792	3.327258	3.346922	35.04862
3.229025	3.425518	3.414395	35.20163
3.232159	3.388856	3.387641	35.0698

3.237974	3.223943	3.39598	34.74012
3.249028	3.270849	3.345081	34.75556
3.256864	3.371064	3.392365	35.09525
3.270351	3.455171	3.414753	35.35763
3.199701	3.44176	3.473513	35.3023
3.160726	3.204197	3.403141	34.54366
3.39371	3.141655	3.383958	34.87445
3.247254	3.201231	3.390506	34.69877
3.294808	3.361831	3.373318	35.11638
3.30747	3.507738	3.364622	35.44413
3.324173	3.469256	3.340374	35.34348
3.312048	3.37194	3.310244	35.03826
3.364138	3.369042	3.384657	35.30856
3.131773	3.388451	3.445838	34.97666
3.270887	3.379892	3.364264	35.08377
3.204856	3.37996	3.417993	35.05701
3.312048	3.319642	3.374341	35.06406
3.356178	3.193886	3.424694	34.99567
3.242388	3.262964	3.406893	34.85896
3.21793	3.337973	3.476531	35.1218
3.36286	3.410219	3.360632	35.34328
3.293612	3.385149	3.379815	35.17897
3.36686	3.356641	3.342148	35.19444
3.169717	3.314318	3.411496	34.82242
3.245275	3.383868	3.406875	35.12964
3.274846	3.423361	3.395639	35.2561
3.253152	3.368031	3.411462	35.12226
3.226633	3.350845	3.424012	35.05413
3.276125	3.210532	3.420875	34.84866
3.296251	3.244161	3.382134	34.88149
3.339969	3.336423	3.391324	35.19896
3.326235	3.392832	3.415265	35.34463
3.261937	3.424574	3.416032	35.27512
3.350404	3.362707	3.374682	35.24286
3.22057	3.378275	3.371493	34.98601
3.34133	3.395932	3.393456	35.33673
3.240697	3.415072	3.396781	35.16579
3.231211	3.438794	3.457161	35.32896
3.175161	3.279543	3.407762	34.75011
3.345785	3.210599	3.376268	34.90359
3.290106	3.230278	3.396457	34.86902
3.316543	3.274084	3.436153	35.10944
3.282517	3.351048	3.437909	35.20718
3.354817	3.416622	3.406824	35.44071
3.371851	3.366144	3.350844	35.24515
3.355353	3.306568	3.367725	35.1157
3.208362	3.291067	3.393234	34.81614
3.252203	3.375512	3.34358	34.9881
3.199371	3.44486	3.444815	35.2456
3.325452	3.469054	3.368629	35.40762

3.270392	3.244161	3.481902	35.04312
3.206671	3.141925	3.409894	34.52273
3.244738	3.265323	3.44843	34.9601
3.316213	3.350576	3.377274	35.14723
3.340134	3.417026	3.416646	35.43096
3.311594	3.378342	3.370641	35.18334
3.211661	3.358259	3.418675	35.02593
3.276248	3.430842	3.304924	35.07715
3.331927	3.444994	3.440774	35.52694
3.244573	3.45416	3.345456	35.14751
3.241191	3.427876	3.467579	35.3497
3.191823	3.275365	3.427491	34.82055
3.323802	3.176161	3.384726	34.7987
3.361622	3.319238	3.397071	35.22129
3.299922	3.385958	3.409075	35.25852
3.270557	3.376657	3.403056	35.1608
3.343021	3.428483	3.393439	35.41158
3.285487	3.215856	3.319316	34.65868
3.429096	3.228526	3.302605	34.9639
3.104181	3.327056	3.440876	34.7712
3.25773	3.494798	3.370607	35.32015
3.191369	3.467908	3.44161	35.2715
3.301902	3.30185	3.359353	34.97019
3.238593	3.176565	3.351765	34.54117
3.30417	3.267277	3.404642	34.99858
3.151157	3.382723	3.485466	35.09318
3.38975	3.45106	3.362286	35.49501
3.424353	3.391282	3.421762	35.57002
3.359148	3.324495	3.359523	35.14527
3.243501	3.354283	3.454603	35.16543
3.229396	3.40685	3.381059	35.08872
3.310687	3.407591	3.399066	35.30748
3.35061	3.387238	3.349736	35.2424
3.167077	3.240589	3.516517	34.88507
3.201969	3.200962	3.38967	34.59732
3.2647	3.276915	3.430611	34.99014
3.398535	3.437244	3.406057	35.57973
3.36286	3.42882	3.4124	35.49716
3.234304	3.348689	3.446111	35.11452
3.263587	3.314588	3.370044	34.93763
3.281569	3.263436	3.343393	34.80681
3.324462	3.334941	3.331866	35.03178
3.223168	3.395595	3.465209	35.23451
3.197638	3.402132	3.384231	35.01589
3.202876	3.275567	3.398912	34.78267
3.367355	3.230211	3.326341	34.88447
3.165716	3.174341	3.394479	34.47035
3.283796	3.373288	3.426536	35.23373
3.238387	3.450521	3.451704	35.35837
3.431282	3.455441	3.318429	35.49951

3.458049	3.381105	3.342199	35.44746
3.345455	3.34478	3.33294	35.10155
3.215208	3.394719	3.4375	35.15459
3.225725	3.470132	3.391683	35.24231
3.207372	3.395258	3.413253	35.08561
3.258307	3.292685	3.333861	34.79906
3.201598	3.1242	3.383208	34.41452
3.173923	3.231828	3.485176	34.81235
3.283425	3.447016	3.444611	35.43368
3.342403	3.473704	3.434857	35.59969
3.322812	3.363381	3.396389	35.23147
3.334567	3.349565	3.407558	35.25138
3.311718	3.322271	3.444679	35.2229
3.291673	3.280554	3.383157	34.9533
3.335804	3.342691	3.381844	35.18282
3.248904	3.4208	3.370811	35.13947
3.222879	3.339793	3.426451	35.02709
3.235459	3.240589	3.382816	34.74223
3.306562	3.158369	3.409587	34.77646
3.215208	3.301581	3.40176	34.87275
3.278764	3.360213	3.419425	35.17859
3.258349	3.423698	3.453341	35.34694
3.355312	3.428753	3.369294	35.38624
3.406825	3.350239	3.310995	35.19971
3.397628	3.364931	3.41992	35.44992
3.243584	3.456991	3.411923	35.29689
3.225436	3.436638	3.455438	35.30785
3.246594	3.303939	3.333349	34.79694
3.24115	3.223471	3.364929	34.67813
3.144888	3.268221	3.364946	34.56551
3.278558	3.339995	3.4744	35.25415
3.167943	3.389395	3.492133	35.15906
3.346651	3.379555	3.389483	35.30387
3.40101	3.340197	3.464118	35.49988
3.415527	3.387238	3.356062	35.3982
3.339186	3.216125	3.423944	35.00551
3.265896	3.335615	3.365389	34.97849
3.249605	3.392158	3.416373	35.17801
3.354116	3.458069	3.302196	35.301
3.230056	3.2447	3.388323	34.75145
3.123978	3.144823	3.409416	34.34718
3.245192	3.198805	3.435471	34.78729
3.23748	3.381105	3.407847	35.10868
3.409547	3.501942	3.398537	35.72885
3.30483	3.422148	3.412605	35.35612
3.285817	3.383531	3.464544	35.34367
3.353374	3.356844	3.313859	35.10352
3.320915	3.385755	3.380548	35.2416
3.242924	3.400245	3.364127	35.06683
3.206382	3.29592	3.38493	34.80427

3.153261	3.245509	3.431651	34.68003
3.355312	3.328808	3.377291	35.18517
3.232407	3.326449	3.436272	35.04022
3.290189	3.387305	3.362882	35.13917
3.23781	3.402132	3.514795	35.38926
3.300747	3.419317	3.35642	35.21813
3.383687	3.354889	3.418846	35.39513
3.454337	3.429494	3.420397	35.71617
3.290849	3.275836	3.445344	35.07718
3.336216	3.356507	3.334321	35.11001
3.24445	3.383599	3.437926	35.19515
3.347599	3.373557	3.361126	35.23081
3.278476	3.212486	3.346513	34.69546
3.103768	3.225763	3.432777	34.53108
3.122369	3.250968	3.4264	34.61293
3.295138	3.399302	3.396167	35.24901
3.310852	3.46373	3.448294	35.53826
3.425426	3.46528	3.325164	35.52295
3.327803	3.3652	3.452267	35.36855
3.282806	3.42417	3.405733	35.29735
3.331061	3.350643	3.325301	35.06619
3.338691	3.375444	3.395349	35.2903
3.200443	3.310881	3.328592	34.70079
3.207784	3.189842	3.393388	34.59385
3.260576	3.288102	3.344211	34.81664
3.227375	3.372277	3.442428	35.14289
3.298644	3.419452	3.472234	35.46709
3.17912	3.36675	3.490019	35.12936
3.323926	3.363583	3.385544	35.21063
3.30813	3.409748	3.437654	35.39099
3.480073	3.399841	3.381639	35.62285
3.305325	3.330627	3.452113	35.24346
3.264453	3.283789	3.364042	34.85905
3.279589	3.288304	3.4626	35.11756
3.3516	3.312027	3.321618	35.0186
3.274434	3.22738	3.375518	34.78262
3.215208	3.273073	3.418368	34.84673
3.10216	3.312162	3.43409	34.71937
3.333453	3.495607	3.398026	35.54747
3.364014	3.459552	3.387386	35.51219
3.440397	3.405502	3.424541	35.64228
3.31609	3.398762	3.386004	35.27143
3.350404	3.438525	3.392348	35.44729
3.350528	3.399639	3.316229	35.19607
3.350651	3.377196	3.349685	35.22042
3.140805	3.27651	3.375637	34.59809
3.13998	3.165715	3.387232	34.37935
3.269897	3.273006	3.410763	34.94955
3.251049	3.434683	3.35908	35.14887
3.293818	3.42855	3.420994	35.36438

3.164355	3.356709	3.471671	35.03499
3.246471	3.329077	3.390745	34.97716
3.33964	3.438053	3.396713	35.43227
3.436644	3.395191	3.379593	35.51323
3.270681	3.307511	3.412468	35.03045
3.210836	3.299963	3.368885	34.78776
3.284745	3.277049	3.407899	34.98459
3.357292	3.250294	3.321822	34.8965
3.203866	3.220843	3.36897	34.59968
3.199866	3.248744	3.391597	34.70143
3.131237	3.321799	3.393319	34.71487
3.267958	3.441288	3.408223	35.30776
3.376511	3.467167	3.39453	35.5718
3.42922	3.396673	3.344603	35.42372
3.305572	3.389125	3.424558	35.31167
3.329782	3.416217	3.352686	35.26668
3.316708	3.391416	3.412196	35.31399
3.390864	3.34815	3.268894	35.06817
3.17479	3.225156	3.389091	34.58953
3.093375	3.205342	3.364383	34.31413
3.237191	3.324292	3.435335	35.04391
3.228076	3.411163	3.404761	35.14709
3.32009	3.386295	3.379798	35.23934
3.220735	3.323955	3.479174	35.10306
3.204691	3.321731	3.43513	34.96679
3.314852	3.373018	3.384998	35.21022
3.474217	3.400245	3.390387	35.63005
3.276207	3.351789	3.429401	35.17639
3.250801	3.374703	3.358551	35.016
3.274516	3.257775	3.398009	34.89845
3.367108	3.277521	3.328643	34.99242
3.296499	3.29538	3.36249	34.95109
3.195081	3.303198	3.431362	34.89701
3.143445	3.355226	3.39141	34.8105
3.316419	3.42107	3.384896	35.31851
3.400185	3.468784	3.42142	35.68591
3.405629	3.420935	3.373591	35.48858
3.292704	3.382655	3.42836	35.27769
3.356508	3.359741	3.379525	35.26032
3.267381	3.326516	3.392757	35.02169
3.314976	3.291943	3.360171	34.9789
3.228035	3.181687	3.34815	34.52138
3.179203	3.313509	3.403482	34.82387
3.092921	3.459215	3.439614	35.03283
3.283177	3.439873	3.402851	35.3262
3.296829	3.416352	3.436818	35.3789
3.244945	3.341545	3.44577	35.12142
3.213971	3.358731	3.436971	35.07203
3.338113	3.413993	3.458917	35.51235
3.379439	3.402402	3.409689	35.46972

3.376552	3.290461	3.336418	35.05838
3.193267	3.234861	3.425769	34.73137
3.264618	3.238096	3.293244	34.60466
3.308088	3.202916	3.399646	34.85548
3.232572	3.325708	3.322777	34.79076
3.288745	3.338445	3.416902	35.14729
3.16551	3.415543	3.427559	35.06971
3.315347	3.411432	3.3725	35.26798
3.364138	3.347678	3.379337	35.25021
3.398288	3.455036	3.359489	35.51626
3.263257	3.415678	3.42906	35.28704
3.448852	3.311757	3.381059	35.36068
3.264453	3.236209	3.370794	34.76977
3.363891	3.228459	3.312427	34.84263
3.194504	3.235265	3.402988	34.68514
3.273155	3.404558	3.401896	35.22497
3.146868	3.435357	3.432061	35.08211
3.263504	3.43657	3.333571	35.12445
3.207661	3.370524	3.444389	35.10024
3.348259	3.35563	3.354459	35.17847
3.247502	3.419317	3.485193	35.3833
3.318317	3.496214	3.372295	35.45943
3.301778	3.26229	3.491775	35.17299
3.307016	3.271321	3.350009	34.89418
3.272908	3.218484	3.393933	34.8001
3.275217	3.18964	3.382952	34.71806
3.207413	3.293022	3.412963	34.86149
3.203083	3.354957	3.41448	34.99078
3.189513	3.374703	3.366378	34.89909
3.313491	3.388586	3.376404	35.2225
3.34628	3.456586	3.40251	35.5
3.374531	3.448162	3.414992	35.57065
3.389833	3.444994	3.404369	35.57396
3.359148	3.401593	3.357238	35.30887
3.408062	3.322608	3.341807	35.20937
3.26437	3.238905	3.412144	34.86591
3.264494	3.243824	3.344211	34.72838
3.159942	3.244026	3.374682	34.56682
3.357745	3.404154	3.381162	35.36372
3.221147	3.419789	3.45832	35.26793
3.195865	3.34505	3.409194	34.94177
3.239459	3.342826	3.437654	35.09447
3.312089	3.412174	3.412059	35.34899
3.24115	3.419857	3.45387	35.30209
3.353332	3.37039	3.484579	35.50639
3.222302	3.200355	3.440007	34.75054
3.379852	3.220102	3.350094	34.94163
3.341083	3.2261	3.372909	34.91986
3.353992	3.275634	3.361808	35.03214
3.173676	3.356574	3.37059	34.83402

3.241604	3.381375	3.390029	35.07932
3.233396	3.413656	3.336453	35.0148
3.400144	3.468987	3.36249	35.55739
3.278476	3.391484	3.394547	35.19194
3.298602	3.416689	3.46746	35.45052
3.327679	3.336154	3.382594	35.1524
3.372139	3.276712	3.380224	35.11446
3.322771	3.25582	3.349923	34.89455
3.25905	3.237759	3.343512	34.70168
3.161427	3.280958	3.406142	34.71963
3.188359	3.43684	3.331712	34.95664
3.299633	3.407995	3.356062	35.19015
3.309491	3.357248	3.380906	35.15506
3.219044	3.377938	3.474758	35.20775
3.154622	3.390945	3.373113	34.87304
3.331102	3.438255	3.475696	35.58676
3.297406	3.418778	3.396116	35.29646
3.298561	3.273343	3.395161	34.97885
3.210713	3.194088	3.40824	34.64202
3.259421	3.253596	3.403329	34.86793
3.251049	3.27833	3.34196	34.76951
3.382574	3.388923	3.349906	35.31636
3.0909	3.396471	3.414992	34.83736
3.299798	3.367829	3.353231	35.09649
3.240408	3.427944	3.458525	35.32833
3.388265	3.469256	3.344262	35.49214
3.225148	3.3652	3.437295	35.11132
3.306933	3.393371	3.368885	35.20218
3.190751	3.30704	3.457007	34.95202
3.397916	3.28325	3.29899	35.00748
3.31147	3.217339	3.41165	34.92067
3.352013	3.285743	3.342728	35.00819
3.274351	3.372075	3.423245	35.20323
3.204279	3.432257	3.398964	35.1285
3.205846	3.365672	3.338141	34.85331
3.350404	3.352935	3.424541	35.33052
3.163241	3.432729	3.445293	35.14111
3.279919	3.398897	3.456734	35.3473
3.24478	3.420261	3.417925	35.2323
3.353868	3.27058	3.374205	35.04793
3.280084	3.152708	3.353078	34.5826
3.254472	3.206488	3.447185	34.85
3.241686	3.240926	3.412264	34.82098
3.220776	3.313375	3.400191	34.90729
3.364551	3.382857	3.38834	35.34773
3.282476	3.363448	3.399782	35.15082
3.193019	3.409815	3.336708	34.91866
3.192854	3.434077	3.45387	35.22757
3.372016	3.502077	3.37354	35.5924
3.308789	3.373759	3.413901	35.26179

3.31477	3.3435	3.396389	35.1704
3.266639	3.27523	3.346701	34.80719
3.386203	3.261549	3.378723	35.10877
3.343475	3.306163	3.338823	35.02564
3.369541	3.344982	3.430321	35.36762
3.208857	3.375579	3.386482	34.98727
3.316048	3.375377	3.430611	35.31775
3.227499	3.319912	3.344876	34.81532
3.269237	3.376792	3.400174	35.15191
3.207908	3.496551	3.397293	35.27339
3.212404	3.453958	3.475696	35.36153
3.125627	3.364931	3.45878	34.94008
3.352466	3.234996	3.329137	34.86849
3.343021	3.185731	3.393933	34.8818
3.386533	3.24315	3.35862	35.02529
3.251461	3.302457	3.411872	34.97606
3.254431	3.347071	3.36943	34.98731
3.381832	3.324292	3.332548	35.13544
3.353868	3.359135	3.312052	35.10566
3.18209	3.43037	3.440041	35.16568
3.257111	3.493653	3.38123	35.33952
3.311099	3.428079	3.444184	35.45186
3.328504	3.317014	3.390097	35.12876
3.372593	3.255753	3.408103	35.13058
3.223952	3.319642	3.39528	34.9172
3.291715	3.350845	3.392757	35.1281
3.306397	3.355428	3.370317	35.12116
3.410784	3.395393	3.377973	35.45358
3.271547	3.370322	3.392501	35.12603
3.212115	3.374231	3.392484	35.00458
3.25142	3.386362	3.390932	35.11367
3.339969	3.437244	3.370931	35.37484
3.160396	3.358394	3.40517	34.88458
3.301366	3.383127	3.393712	35.22189
3.167283	3.234726	3.466778	34.76393
3.314275	3.234591	3.362541	34.85713
3.414373	3.216395	3.424268	35.17123
3.333206	3.270782	3.38314	35.02272
3.266432	3.310477	3.468329	35.1498
3.262349	3.429696	3.347144	35.13657
3.329576	3.386295	3.344194	35.18222
3.407444	3.402267	3.256191	35.19499
3.195205	3.437783	3.447919	35.2278
3.138908	3.426933	3.415964	35.01108
3.286353	3.344376	3.427695	35.17864
3.316667	3.267479	3.342097	34.88958
3.339268	3.192538	3.412332	34.92871
3.260122	3.257573	3.419374	34.91325
3.207372	3.342219	3.452028	35.05441
3.364179	3.370861	3.351151	35.23936

3.388802	3.402334	3.372329	35.40834
3.251585	3.370322	3.422921	35.1489
3.212734	3.365605	3.367606	34.93266
3.247007	3.41433	3.439768	35.27197
3.362488	3.483342	3.375347	35.53455
3.226385	3.365807	3.423791	35.08582
3.264123	3.294235	3.389551	34.93696
3.134825	3.170904	3.473104	34.56722
3.313574	3.174813	3.346939	34.69076
3.288085	3.243016	3.46971	35.05265
3.406743	3.302929	3.365355	35.21494
3.212981	3.330964	3.444474	35.02555
3.336093	3.345454	3.35376	35.12808
3.313986	3.369109	3.365952	35.15813
3.303015	3.444118	3.324943	35.20849
3.205887	3.475052	3.431242	35.2962
3.182832	3.491429	3.457143	35.33823
3.137134	3.405097	3.39349	34.91031
3.329081	3.215923	3.360205	34.84358
3.302768	3.210262	3.447509	34.96458
3.284456	3.312566	3.37695	34.99395
3.22655	3.347071	3.440228	35.08116
3.309367	3.349498	3.372312	35.11905
3.388802	3.330627	3.352788	35.2088
3.296705	3.348082	3.354698	35.04975
3.176192	3.388519	3.354306	34.87377
3.247502	3.433066	3.405239	35.23852
3.26701	3.445938	3.425001	35.35255
3.22391	3.367761	3.435931	35.11124
3.320668	3.309061	3.401828	35.11988
3.165922	3.204332	3.44814	34.65373
3.247625	3.245509	3.412878	34.84534
3.283466	3.332514	3.440927	35.17532
3.358529	3.363111	3.443502	35.41201
3.259421	3.351722	3.409416	35.09583
3.303428	3.313914	3.397344	35.08299
3.262638	3.347678	3.35301	34.97067
3.324668	3.465617	3.330689	35.31542
3.150126	3.448634	3.382748	35.01043
3.293076	3.349026	3.374836	35.08791
3.198999	3.246048	3.39112	34.6926
3.269031	3.228728	3.362814	34.74597
3.327473	3.349969	3.402391	35.22545
3.42889	3.418778	3.386772	35.56356
3.260947	3.344241	3.407711	35.07908
3.367726	3.330021	3.35613	35.16869
3.288456	3.33703	3.365423	35.03099
3.381543	3.440479	3.374648	35.48096
3.207124	3.448769	3.379133	35.12747
3.137547	3.407726	3.449726	35.03994

3.224075	3.324292	3.397753	34.93305
3.199659	3.30677	3.371135	34.78313
3.273155	3.276982	3.426604	35.00001
3.249894	3.259864	3.431992	34.92349
3.184234	3.228593	3.463197	34.77976
3.376676	3.364594	3.353487	35.25809
3.411444	3.39364	3.391853	35.48154
3.331968	3.399908	3.380275	35.29613
3.345909	3.345454	3.382696	35.21283
3.230262	3.376792	3.418556	35.10688
3.316997	3.444455	3.38771	35.37707
3.307387	3.478354	3.267223	35.1667
3.29522	3.3497	3.422921	35.19923
3.118575	3.250698	3.363565	34.46663
3.261277	3.217406	3.410985	34.80959
3.243955	3.389934	3.384129	35.09027
3.413012	3.419587	3.401027	35.56177
3.22688	3.315531	3.442957	35.01887
3.344877	3.290124	3.415657	35.16165
3.261236	3.375579	3.41402	35.16204
3.400556	3.4888	3.318293	35.50497
3.226962	3.424439	3.435744	35.24145
3.166376	3.386632	3.385135	34.9156
3.101871	3.255079	3.478032	34.69
3.28227	3.352126	3.343989	35.0036
3.282229	3.293965	3.434789	35.07489
3.275094	3.219495	3.393985	34.8072
3.23352	3.262088	3.431975	34.89251
3.28726	3.301581	3.399799	35.02603
3.466917	3.365133	3.388392	35.53294
3.388265	3.396606	3.361672	35.37134
3.225395	3.37349	3.412264	35.07525
3.222921	3.403682	3.368203	35.03951
3.332876	3.452745	3.431379	35.52541
3.254431	3.434953	3.312427	35.05483
3.33601	3.307444	3.388545	35.12085
3.114368	3.215856	3.41801	34.5003
3.190215	3.288641	3.417328	34.82384
3.304995	3.403817	3.432743	35.36043
3.417878	3.389664	3.377138	35.45474
3.272784	3.269366	3.394121	34.91151
3.256946	3.321664	3.420926	35.04986
3.26338	3.398762	3.398094	35.1826
3.386698	3.471885	3.300695	35.39919
3.24313	3.384542	3.419425	35.15387
3.249193	3.342017	3.37811	34.98378
3.152271	3.323686	3.344757	34.6588
3.240037	3.285608	3.370266	34.82325
3.192277	3.313442	3.395707	34.83531
3.304995	3.253933	3.399646	34.96028

3.212816	3.31816	3.479106	35.07292
3.451945	3.348824	3.387045	35.46159
3.34232	3.364796	3.432709	35.35665
3.44679	3.389125	3.304992	35.35901
3.243171	3.343365	3.388613	34.99653
3.175326	3.407861	3.362371	34.93181
3.236943	3.434144	3.410013	35.22823
3.271175	3.366616	3.324823	34.96912
3.239047	3.248609	3.3858	34.77414
3.200773	3.245307	3.393882	34.7009
3.215249	3.394247	3.426655	35.12993
3.308872	3.4053	3.399577	35.29962
3.455204	3.440749	3.375722	35.64498
3.278599	3.336423	3.402186	35.08851
3.27266	3.35125	3.39557	35.09347
3.310192	3.449779	3.416714	35.43725
3.373583	3.373422	3.408308	35.39052
3.250059	3.296391	3.354016	34.83321
3.253853	3.278465	3.356539	34.80782
3.076135	3.294302	3.435948	34.62747
3.258679	3.298009	3.365764	34.88129
3.213063	3.250361	3.458439	34.88
3.260535	3.287361	3.385544	34.90531
3.256575	3.330223	3.458849	35.15069
3.408805	3.468717	3.366702	35.58495
3.354817	3.405974	3.449163	35.51001
3.376924	3.348689	3.405392	35.33736
3.240573	3.330897	3.37059	34.92417
3.286765	3.386429	3.41003	35.23287
3.157756	3.402334	3.340732	34.83399
3.29056	3.287495	3.349736	34.89296
3.231582	3.253529	3.359472	34.711
3.197391	3.307107	3.416561	34.87824
3.242181	3.429629	3.417737	35.2467
3.314687	3.391753	3.380275	35.24051
3.400391	3.322271	3.434635	35.39486
3.317409	3.3497	3.327006	35.038
3.324833	3.433403	3.438182	35.48041
3.291137	3.508682	3.361535	35.40373
3.344795	3.364526	3.426263	35.34737
3.218136	3.291809	3.37516	34.79961
3.319884	3.26721	3.375449	34.96896
3.132103	3.318631	3.43866	34.80899
3.280455	3.279476	3.355243	34.86537
3.222426	3.241398	3.455251	34.8739
3.219539	3.290596	3.402954	34.86081
3.234386	3.335951	3.402868	34.99228
3.34331	3.354013	3.385697	35.23243
3.317038	3.371198	3.433288	35.31663
3.305119	3.387171	3.330502	35.10071

3.277486	3.375983	3.412213	35.19451
3.375563	3.406647	3.353862	35.34844
3.215456	3.374501	3.372721	34.96925
3.233685	3.247666	3.339147	34.65833
3.26833	3.277925	3.408922	34.95285
3.220322	3.372209	3.394684	35.02291
3.267464	3.431583	3.465737	35.41123
3.297489	3.400582	3.392348	35.2486
3.270804	3.322203	3.455779	35.15756
3.382574	3.388316	3.397787	35.41974
3.356962	3.475119	3.384794	35.52514
3.289158	3.44304	3.4183	35.37999
3.257895	3.345926	3.386772	35.0303
3.1705	3.244498	3.408529	34.66496
3.294478	3.261953	3.356966	34.86148
3.274557	3.345454	3.385544	35.06302
3.197432	3.27523	3.425496	34.82816
3.211661	3.317486	3.455592	35.0175
3.288044	3.296189	3.429332	35.08054
3.250265	3.349295	3.346053	34.93194
3.342444	3.359809	3.403738	35.28266
3.251832	3.397415	3.411172	35.183
3.308872	3.353676	3.376745	35.1368
3.347022	3.386901	3.370948	35.28021
3.345207	3.350037	3.334986	35.11698
3.226426	3.269771	3.37001	34.75829
3.175903	3.257842	3.356897	34.59304
3.146043	3.346937	3.377973	34.76867
3.310439	3.471143	3.383344	35.41154
3.246471	3.45753	3.467596	35.42613
3.292457	3.374433	3.379883	35.15316
3.312501	3.32901	3.440314	35.22981
3.398618	3.443444	3.417908	35.61938
3.333494	3.461034	3.442769	35.5698
3.307552	3.32874	3.369993	35.06462
3.211661	3.2354	3.440927	34.80592
3.194091	3.230548	3.334594	34.52435
3.271918	3.311892	3.414719	35.04766
3.269691	3.376725	3.356403	35.05704
3.166128	3.284934	3.473854	34.88668
3.231705	3.244431	3.412417	34.80716
3.36917	3.331975	3.415589	35.30615
3.34661	3.467234	3.400413	35.51942
3.349909	3.369379	3.421352	35.35843
3.254678	3.402739	3.454211	35.29498
3.29588	3.367761	3.425035	35.24479
3.310563	3.378814	3.329666	35.09252
3.432231	3.31142	3.322982	35.19659
3.138083	3.243757	3.404454	34.58353
3.204402	3.272871	3.379729	34.73816

3.18972	3.351924	3.3873	34.89548
3.310645	3.453082	3.41356	35.43857
3.257895	3.39856	3.426741	35.23281
3.332999	3.408467	3.432487	35.43128
3.228983	3.327864	3.402459	34.96188
3.372428	3.487183	3.414463	35.65023
3.317739	3.4702	3.455848	35.58399
3.341207	3.306366	3.32733	34.99599
3.17446	3.172185	3.430492	34.56351
3.175532	3.250631	3.401078	34.67308
3.311223	3.327999	3.371835	35.07505
3.221931	3.395797	3.369328	35.02257
3.183533	3.256427	3.432061	34.771
3.230963	3.289719	3.422222	34.92601
3.29221	3.34195	3.458115	35.25266
3.467989	3.358461	3.335242	35.40447
3.336093	3.343837	3.424132	35.27844
3.21096	3.408265	3.402971	35.09941
3.289158	3.372951	3.380429	35.14389
3.301654	3.301513	3.295034	34.82826
3.426952	3.257101	3.318872	35.05727
3.161056	3.200827	3.394684	34.51852
3.120884	3.33521	3.392211	34.71914
3.239913	3.384205	3.445003	35.20203
3.355188	3.468852	3.341534	35.41296
3.229726	3.335412	3.414992	35.00742
3.351518	3.347678	3.401095	35.27019
3.188606	3.369581	3.487632	35.15107
3.413094	3.504705	3.368083	35.67605
3.369211	3.445803	3.363275	35.44076
3.381873	3.238568	3.33942	34.96309
3.192648	3.142195	3.361467	34.38675
3.226509	3.226908	3.338652	34.59616
3.182213	3.459821	3.383003	35.10563
3.262762	3.390406	3.325778	35.00483
3.181677	3.261751	3.472712	34.86748
3.206588	3.289382	3.367111	34.75145
3.308501	3.374635	3.472064	35.39027
3.362117	3.401593	3.371903	35.34744
3.299551	3.378477	3.444048	35.31783
3.251131	3.401256	3.457826	35.29189
3.251049	3.324292	3.417669	35.03559
3.344754	3.286821	3.360222	35.03293
3.301407	3.271119	3.322112	34.82046
3.255627	3.287832	3.34358	34.80385
3.11676	3.399975	3.404164	34.87789
3.2282	3.369042	3.473018	35.20452
3.367974	3.437581	3.418539	35.54093
3.328628	3.374366	3.323391	35.10857
3.34331	3.345521	3.471126	35.40067

3.211208	3.472289	3.38447	35.1995
3.321781	3.466021	3.469233	35.61297
3.321121	3.311892	3.386021	35.0925
3.345537	3.20386	3.379695	34.89581
3.135526	3.201568	3.431634	34.54512
3.25971	3.36392	3.366992	35.03036
3.217848	3.460562	3.466556	35.36789
3.217683	3.344443	3.351015	34.86092
3.196607	3.237759	3.400191	34.68908
3.246182	3.31324	3.442275	35.05458
3.230097	3.383868	3.453256	35.19787
3.430169	3.463595	3.373471	35.63527
3.268123	3.446275	3.410951	35.32499
3.375068	3.317755	3.406824	35.26878
3.307263	3.17333	3.320595	34.6161
3.324709	3.207701	3.354715	34.80403
3.258307	3.293156	3.342438	34.81885
3.288044	3.398156	3.350486	35.1311
3.196772	3.408534	3.43559	35.14031
3.267299	3.435492	3.387232	35.24774
3.323596	3.379892	3.415998	35.31217
3.307965	3.336558	3.349667	35.03817
3.30417	3.386834	3.419801	35.29318
3.241851	3.439738	3.501341	35.45091
3.245728	3.418441	3.385442	35.15936
3.262721	3.200086	3.31229	34.55905
3.256823	3.210734	3.339437	34.6288
3.256452	3.286484	3.434635	35.00182
3.199206	3.436772	3.452864	35.24515
3.206918	3.420463	3.432589	35.18202
3.285116	3.309331	3.377427	34.98937
3.238181	3.278195	3.33132	34.71781
3.274104	3.368166	3.448413	35.24918
3.190875	3.406715	3.397258	35.03961
3.254554	3.426663	3.38568	35.19717
3.335391	3.405367	3.397531	35.35329
3.438459	3.271456	3.323545	35.12404
3.285074	3.223	3.419442	34.89236
3.31279	3.279745	3.354578	34.93522
3.253853	3.316205	3.412127	35.01192
3.29089	3.407591	3.365952	35.19178
3.206176	3.370255	3.372977	34.94024
3.183409	3.392899	3.444747	35.09692
3.231293	3.410758	3.372551	35.08281
3.315265	3.403884	3.408461	35.32994
3.309573	3.427742	3.398503	35.34788
3.386327	3.420396	3.351901	35.39776
3.221848	3.246385	3.477742	34.93273
3.318317	3.175083	3.305657	34.61144
3.362777	3.229874	3.363633	34.95527

3.270557	3.354552	3.343324	34.98184
3.215456	3.390945	3.447373	35.16847
3.220858	3.285474	3.397634	34.84086
3.278434	3.305961	3.381025	34.97525
3.318935	3.42107	3.381861	35.31737
3.31576	3.431853	3.451909	35.48719
3.155405	3.34633	3.469915	34.98888
3.254967	3.37504	3.388596	35.09155
3.282724	3.299626	3.439751	35.0992
3.458421	3.30859	3.310517	35.22041
3.268618	3.238568	3.428395	34.91
3.280744	3.259325	3.362217	34.83719
3.253441	3.307309	3.37475	34.90982
3.276331	3.367087	3.370999	35.0824
3.277898	3.366683	3.347758	35.03412
3.228117	3.392764	3.402732	35.10251
3.142702	3.380297	3.412042	34.90882
3.320379	3.414263	3.349855	35.23565
3.281445	3.427809	3.459889	35.42076
3.365334	3.395797	3.38036	35.36029
3.213476	3.271793	3.436221	34.87918
3.245934	3.190583	3.282041	34.43541
3.352219	3.24524	3.391324	35.02634
3.355023	3.352935	3.340306	35.15642
3.167696	3.394719	3.442019	35.06057
3.250801	3.329212	3.41003	35.0291
3.216445	3.269366	3.392211	34.78413
3.27266	3.331975	3.405358	35.07273
3.314811	3.433605	3.391938	35.3578
3.15058	3.423092	3.529101	35.27562
3.228901	3.431246	3.401777	35.18629
3.326235	3.282171	3.357392	34.97608
3.426209	3.200625	3.357443	35.01649
3.246718	3.256427	3.419204	34.88106
3.286229	3.307646	3.411087	35.06172
3.287054	3.388788	3.373113	35.15793
3.40699	3.421744	3.33323	35.40506
3.309202	3.350441	3.360274	35.09443
3.245728	3.371131	3.443639	35.18317
3.12637	3.385553	3.447731	34.96264
3.224983	3.441625	3.371613	35.13445
3.378367	3.458608	3.43143	35.63783
3.33799	3.346667	3.366566	35.16289
3.175862	3.145766	3.45109	34.55384
3.359024	3.275432	3.37741	35.07682
3.268783	3.391956	3.454705	35.30333
3.353992	3.465819	3.347144	35.41598
3.204897	3.334334	3.453546	35.03508
3.187121	3.279476	3.396321	34.75109
3.12736	3.236344	3.352259	34.42973

3.261896	3.451464	3.409382	35.31929
3.222632	3.460832	3.502296	35.4571
3.240614	3.423563	3.465277	35.33397
3.218631	3.266603	3.422921	34.85002
3.31114	3.160593	3.346854	34.65415
3.45475	3.241668	3.308215	35.061
3.260906	3.359539	3.399799	35.09514
3.241356	3.40658	3.410405	35.17846
3.30252	3.450723	3.369788	35.31992
3.323142	3.351519	3.321157	35.04173
3.320791	3.369109	3.361211	35.16265
3.27864	3.461439	3.443724	35.45282
3.12769	3.458473	3.436613	35.10068
3.292003	3.367627	3.355584	35.08414
3.29885	3.209386	3.412127	34.87672
3.274475	3.188022	3.396798	34.74317
3.221518	3.25454	3.388681	34.75508
3.317079	3.394719	3.40367	35.30338
3.280579	3.423765	3.376132	35.22686
3.354735	3.36729	3.412878	35.34588
3.15458	3.309803	3.378178	34.70658
3.302438	3.295987	3.372926	34.98822
3.12274	3.380701	3.380906	34.79796
3.21925	3.425316	3.406739	35.16307
3.231211	3.37969	3.407779	35.09172
3.262226	3.339995	3.460844	35.18878
3.210259	3.243757	3.433885	34.80573
3.325163	3.211543	3.342148	34.78594
3.321657	3.248946	3.397685	34.98152
3.376429	3.423092	3.308317	35.2867
3.263999	3.453958	3.462481	35.44546
3.254843	3.425113	3.329495	35.07154
3.221023	3.339254	3.331917	34.81512
3.267793	3.434818	3.359063	35.18574
3.198999	3.509355	3.37504	35.23324
3.256039	3.434548	3.473053	35.40873
3.182461	3.315194	3.479583	35.0011
3.37317	3.160323	3.40367	34.91346
3.339804	3.192066	3.396321	34.89384
3.264205	3.34013	3.398623	35.05734
3.343475	3.409411	3.376387	35.33357
3.268247	3.349295	3.386772	35.06031
3.330978	3.374972	3.389364	35.25931
3.251379	3.333525	3.337015	34.88012
3.281528	3.322877	3.409945	35.08225
3.060834	3.394921	3.415947	34.7703
3.261483	3.384205	3.424814	35.20505
3.255915	3.37012	3.461372	35.24202
3.306109	3.355765	3.368407	35.11709
3.231046	3.190246	3.43351	34.73335

3.302355	3.251912	3.397685	34.9458
3.293488	3.323079	3.432675	35.15856
3.364262	3.376118	3.359796	35.26994
3.328669	3.376994	3.399867	35.28165
3.382945	3.399099	3.342284	35.32276
3.164932	3.369042	3.407779	34.9235
3.296334	3.459349	3.333724	35.24639
3.20597	3.493248	3.402732	35.27382
3.300252	3.401863	3.362422	35.192
3.16353	3.248744	3.419664	34.68335
3.272701	3.194425	3.367725	34.68972
3.330566	3.250159	3.378843	34.96245
3.362158	3.430842	3.373949	35.41596
3.318853	3.384677	3.463708	35.4166
3.227086	3.352665	3.373062	34.94768
3.304624	3.318294	3.377837	35.05252
3.226756	3.283317	3.358892	34.76432
3.256617	3.388653	3.391188	35.1306
3.083765	3.380364	3.532034	35.04248
3.183739	3.390877	3.413048	35.0239
3.249481	3.309398	3.411531	34.98616
3.287425	3.272534	3.377564	34.91425
3.211208	3.1829	3.397003	34.59406
3.268453	3.288304	3.37325	34.89781
3.194875	3.409545	3.500165	35.27958
3.472113	3.475254	3.312018	35.61811
3.338278	3.40995	3.38198	35.33562
3.422374	3.329684	3.33207	35.23485
3.208485	3.3652	3.393985	34.98017
3.285899	3.463191	3.345371	35.25744
3.142084	3.469728	3.428036	35.13801
3.324379	3.300839	3.397872	35.10137
3.157962	3.16585	3.461304	34.58096
3.304954	3.183372	3.423057	34.85708
3.287838	3.368098	3.364809	35.09624
3.407196	3.430505	3.452505	35.68551
3.363313	3.394921	3.359745	35.30887
3.230097	3.312027	3.421267	34.97081
3.208568	3.33521	3.37088	34.86424
3.321864	3.313644	3.366173	35.05455
3.227622	3.36675	3.382372	35.00002
3.20432	3.417498	3.408257	35.11664
3.181554	3.338243	3.490565	35.07353
3.331927	3.286552	3.411053	35.11545
3.34331	3.253731	3.385629	35.01298
3.248822	3.223202	3.435727	34.84914
3.247997	3.36082	3.412963	35.0985
3.2899	3.396673	3.422972	35.29043
3.344919	3.461102	3.375995	35.44891
3.384306	3.400919	3.323527	35.2887

3.384182	3.383666	3.390523	35.39721
3.136681	3.38151	3.420704	34.91724
3.319307	3.461573	3.408683	35.46542
3.185059	3.388586	3.36602	34.91893
3.223086	3.258044	3.435198	34.8679
3.165304	3.166389	3.402664	34.46995
3.284167	3.265053	3.457143	35.06479
3.251997	3.454093	3.412127	35.30939
3.401876	3.462382	3.466079	35.77327
3.282641	3.342623	3.439904	35.19339
3.328669	3.31607	3.360973	35.06336
3.201227	3.307714	3.39731	34.84586
3.296788	3.365739	3.345831	35.06915
3.182585	3.384677	3.418266	35.01922
3.214961	3.423698	3.389568	35.1126
3.250884	3.232502	3.391069	34.77632
3.33168	3.210869	3.374222	34.86886
3.274434	3.234187	3.498681	35.06684
3.367149	3.380836	3.379934	35.33061
3.295963	3.370322	3.460486	35.3281
3.325823	3.445803	3.375381	35.37235
3.339145	3.395595	3.404863	35.35616
3.317079	3.352733	3.279023	34.93898
3.206753	3.371198	3.406159	35.01613
3.157179	3.457058	3.362746	35.00054
3.161757	3.422889	3.417447	35.05545
3.175367	3.238096	3.39823	34.63908
3.283796	3.162952	3.359302	34.62673
3.233067	3.237354	3.459906	34.89851
3.371809	3.436705	3.393729	35.49315
3.233891	3.436166	3.451278	35.31621
3.357869	3.390877	3.321072	35.20355
3.329246	3.263234	3.423381	35.08556
3.32673	3.39418	3.369805	35.24925
3.239624	3.294437	3.42865	34.96933
3.304665	3.37632	3.356471	35.13278
3.140228	3.379083	3.423876	34.92663
3.298726	3.328201	3.2891	34.86724
3.186956	3.240994	3.486353	34.86347
3.266185	3.244026	3.388068	34.82842
3.144641	3.250025	3.410371	34.62452
3.2972	3.387171	3.405221	35.2468
3.319018	3.437986	3.412008	35.42047
3.464648	3.436099	3.363582	35.62892
3.275094	3.335547	3.371817	35.01251
3.322276	3.346802	3.372193	35.14112
3.209805	3.416487	3.314371	34.92111
3.250306	3.462719	3.411326	35.32281
3.16551	3.388856	3.369038	34.88337
3.187575	3.138218	3.397156	34.44501

3.244161	3.23203	3.378195	34.73244
3.311099	3.352733	3.402527	35.19599
3.374738	3.450386	3.457263	35.66841
3.299221	3.391012	3.414515	35.27994
3.263669	3.31944	3.435659	35.09191
3.359271	3.345319	3.340391	35.14924
3.446872	3.371064	3.409126	35.54742
3.187575	3.338378	3.449112	34.99634
3.204402	3.395258	3.411599	35.07549
3.229685	3.34478	3.398981	34.9928
3.298107	3.256696	3.356403	34.8567
3.28825	3.211475	3.362558	34.74971
3.23583	3.282171	3.470324	35.02534
3.110491	3.335884	3.43409	34.78947
3.383605	3.411567	3.386073	35.44723
3.411815	3.464471	3.347826	35.54097
3.459039	3.350239	3.351969	35.40349
3.25773	3.390473	3.388068	35.13019
3.2793	3.398897	3.436272	35.3012
3.291797	3.410961	3.296722	35.04974
3.393545	3.401323	3.3223	35.3071
3.175862	3.237557	3.430407	34.70934
3.050936	3.222595	3.424882	34.39135
3.173552	3.329212	3.470648	34.99273
3.302314	3.432055	3.419511	35.38738
3.350858	3.4239	3.43322	35.50569
3.372923	3.288102	3.440365	35.2726
3.206217	3.313846	3.442479	34.96896
3.384224	3.390271	3.37666	35.38142
3.335845	3.39418	3.391052	35.31565
3.258101	3.269097	3.374853	34.83667
3.123524	3.283182	3.394684	34.61654
3.222797	3.237422	3.299672	34.52579
3.307882	3.245779	3.326187	34.78812
3.324916	3.306972	3.338601	34.98634
3.253441	3.361763	3.523167	35.35347
3.143857	3.451195	3.38314	35.00318
3.34661	3.460293	3.45387	35.62114
3.476568	3.429157	3.327245	35.56034
3.428065	3.384003	3.352839	35.4115
3.206753	3.348419	3.438199	35.03638
3.249028	3.373692	3.363923	35.02166
3.284126	3.36055	3.383242	35.11192
3.281074	3.290056	3.336759	34.84944
3.144806	3.204534	3.377751	34.45406
3.169057	3.285069	3.415265	34.76525
3.176769	3.414128	3.459769	35.16167
3.365169	3.475726	3.3489	35.46592
3.204444	3.383868	3.458439	35.15311
3.360962	3.359539	3.369993	35.24877

3.22853	3.353002	3.494538	35.21722
3.347971	3.423361	3.372721	35.36589
3.294354	3.301783	3.410695	35.06581
3.298602	3.249688	3.302298	34.72413
3.151982	3.262695	3.406722	34.6603
3.234263	3.240926	3.34017	34.64709
3.202175	3.290663	3.426178	34.87377
3.277857	3.341141	3.395127	35.08176
3.264164	3.349093	3.44398	35.17604
3.166087	3.424911	3.470734	35.18587
3.356591	3.42754	3.365474	35.37803
3.450873	3.399436	3.378723	35.55173
3.36253	3.413993	3.355243	35.33902
3.234634	3.391619	3.424012	35.16079
3.24247	3.368772	3.393524	35.0613
3.182131	3.304546	3.359813	34.71517
3.377996	3.21781	3.280489	34.78035
3.1632	3.218282	3.445565	34.67265
3.233438	3.427742	3.42952	35.24922
3.144476	3.434414	3.437585	35.0869
3.357952	3.407321	3.35995	35.32471
3.309491	3.379825	3.441507	35.33696
3.298231	3.316475	3.37475	35.02781
3.225766	3.364122	3.447373	35.13236
3.389833	3.38461	3.405733	35.44489
3.206671	3.302592	3.420329	34.8969
3.337247	3.271186	3.3371	34.93176
3.155116	3.293291	3.367111	34.64744
3.128061	3.245307	3.406228	34.56888
3.180811	3.302389	3.344279	34.6736
3.251832	3.35253	3.404915	35.07116
3.275052	3.394382	3.39794	35.19821
3.316667	3.428685	3.448873	35.47561
3.260906	3.403884	3.395366	35.18242
3.402041	3.408332	3.35688	35.41663
3.43021	3.449779	3.371613	35.60109
3.267216	3.351856	3.368833	35.02442
3.287302	3.290393	3.387317	34.97436
3.226138	3.244633	3.396065	34.75966
3.247749	3.229537	3.363155	34.70194
3.253936	3.409074	3.358415	35.09772
3.183698	3.473165	3.47544	35.3402
3.208733	3.458271	3.391938	35.17977
3.322895	3.332514	3.451551	35.28478
3.410619	3.385014	3.409808	35.50014
3.277733	3.392697	3.466624	35.35059
3.251131	3.434144	3.400191	35.23777
3.298932	3.281902	3.433152	35.08146
3.233644	3.223741	3.381639	34.69884
3.363354	3.211273	3.366583	34.92231

3.266597	3.199277	3.353231	34.65529
3.263834	3.322944	3.411258	35.04658
3.095561	3.337502	3.415742	34.72023
3.299798	3.43374	3.389312	35.31953
3.288003	3.445197	3.396031	35.33348
3.336381	3.459282	3.390233	35.4574
3.287425	3.433335	3.410831	35.33864
3.402247	3.412578	3.406824	35.53558
3.328256	3.34141	3.375978	35.15069
3.426127	3.32564	3.310295	35.18659
3.246223	3.233109	3.407967	34.80441
3.1486	3.227515	3.356062	34.46519
3.128267	3.299424	3.413287	34.70312
3.288621	3.413859	3.398895	35.27256
3.264948	3.460225	3.431992	35.39457
3.220116	3.378207	3.444525	35.14458
3.197308	3.338243	3.485944	35.09788
3.393586	3.391686	3.410559	35.47912
3.366489	3.463191	3.440297	35.64127
3.251791	3.415139	3.386772	35.16831
3.312955	3.215991	3.453546	35.01258
3.221807	3.160054	3.384862	34.54073
3.343723	3.224482	3.264256	34.68449
3.339433	3.28271	3.378314	35.05187
3.150662	3.37787	3.416782	34.93128
3.177429	3.39364	3.461526	35.12215
3.314564	3.388856	3.422409	35.32604
3.304459	3.417498	3.409672	35.33872
3.366901	3.450858	3.34196	35.40015
3.188111	3.410422	3.494179	35.25362
3.296829	3.386429	3.386772	35.20402
3.321204	3.278802	3.371374	34.98828
3.430334	3.253933	3.363019	35.15428
3.225354	3.224213	3.43438	34.79708
3.273361	3.299828	3.359626	34.90395
3.131072	3.350172	3.417243	34.82888
3.382038	3.440749	3.385885	35.5072
3.224777	3.401189	3.339692	34.97577
3.234593	3.389058	3.447407	35.20626
3.210589	3.363718	3.454893	35.11473
3.326153	3.451195	3.474263	35.60111
3.294395	3.414128	3.381724	35.24823
3.438954	3.32564	3.393933	35.39755
3.29089	3.17232	3.385339	34.71967
3.262019	3.196918	3.431583	34.81146
3.260205	3.243689	3.370385	34.77594
3.267793	3.337367	3.376609	35.011
3.245316	3.370255	3.394479	35.07285
3.142991	3.36729	3.451022	34.96625
3.205268	3.450453	3.404556	35.18269

3.380099	3.472626	3.362627	35.52181
3.332216	3.431111	3.397207	35.40194
3.262803	3.39054	3.345098	35.04746
3.349249	3.287293	3.438387	35.21473
3.298767	3.27368	3.352958	34.88775
3.376429	3.25946	3.368203	35.05982
3.336959	3.300637	3.367435	35.06187
3.244615	3.308253	3.410849	34.97152
3.185348	3.38744	3.385322	34.95927
3.312006	3.340871	3.397548	35.16115
3.268701	3.393034	3.392535	35.16955
3.287797	3.444455	3.422239	35.38872
3.188482	3.360213	3.485244	35.12509
3.260328	3.418913	3.465004	35.36631
3.223622	3.280958	3.426331	34.89979
3.325617	3.223471	3.400413	34.94044
3.298809	3.224011	3.448908	34.98904
3.351641	3.227448	3.363974	34.92636
3.213435	3.254338	3.427337	34.82149
3.286724	3.413454	3.29454	35.03932
3.163489	3.368368	3.411036	34.92599
3.30318	3.365672	3.329427	35.04711
3.22589	3.394382	3.461935	35.23065
3.316254	3.438255	3.34121	35.2602
3.323018	3.383464	3.473922	35.44539
3.409506	3.349767	3.302793	35.1866
3.318729	3.282576	3.421864	35.10154
3.272949	3.303737	3.35301	34.89713
3.29588	3.277319	3.363036	34.91143
3.314399	3.305287	3.334713	34.95115
3.284951	3.395864	3.346104	35.10974
3.149507	3.387912	3.408188	34.93193
3.289158	3.378005	3.38077	35.15569
3.244243	3.395123	3.485176	35.32323
3.286765	3.461641	3.392706	35.35946
3.226138	3.37969	3.481561	35.24198
3.258885	3.354417	3.417891	35.11909
3.175573	3.233311	3.469148	34.78415
3.431406	3.256696	3.354237	35.14346
3.312213	3.269366	3.419681	35.05363
3.320503	3.261751	3.356232	34.91635
3.303428	3.308792	3.406006	35.09073
3.351146	3.377736	3.408035	35.35029
3.288869	3.363516	3.394394	35.15317
3.322853	3.451599	3.366617	35.35937
3.212816	3.476804	3.450459	35.3572
3.254101	3.415004	3.411667	35.22751
3.294684	3.330762	3.383293	35.06998
3.383894	3.245509	3.338806	34.98135
3.34995	3.244094	3.400771	35.03953

3.276702	3.268221	3.365747	34.85552
3.26833	3.331369	3.424609	35.10403
3.342527	3.37349	3.393848	35.29113
3.25542	3.371872	3.373011	35.05154
3.284868	3.351924	3.392569	35.11508
3.112512	3.342826	3.389551	34.71167
3.241645	3.436031	3.427832	35.2816
3.292498	3.478961	3.453563	35.54296
3.295262	3.325505	3.389227	35.07273
3.313038	3.239781	3.437722	35.03018
3.259627	3.222258	3.430799	34.85993
3.25241	3.282441	3.384248	34.87395
3.33766	3.283047	3.335054	34.95413
3.261607	3.334806	3.403278	35.0502
3.195947	3.386564	3.38007	34.96905
3.281198	3.362235	3.427133	35.20519
3.242305	3.406782	3.374358	35.10214
3.267546	3.475119	3.37354	35.30499
3.280084	3.43347	3.402937	35.30562
3.280125	3.399302	3.406603	35.23901
3.172769	3.255483	3.369703	34.60903
3.435489	3.289248	3.363582	35.24401
3.314316	3.301648	3.356471	34.99059
3.370943	3.388653	3.363155	35.31931
3.317904	3.350643	3.414617	35.23274
3.346156	3.346937	3.366514	35.18122
3.2647	3.351452	3.359268	34.99712
3.358776	3.383734	3.363701	35.28314
3.15058	3.439199	3.413236	35.05746
3.233726	3.460832	3.427133	35.31699
3.185389	3.315127	3.457894	34.95992
3.2972	3.277184	3.372568	34.93486
3.30813	3.213497	3.423109	34.93002
3.281486	3.186674	3.412264	34.78938
3.192401	3.224011	3.497198	34.86195
3.306191	3.346465	3.34283	35.041
3.306274	3.385823	3.432061	35.32238
3.340588	3.422215	3.324499	35.24179
3.249316	3.35435	3.454501	35.17807
3.293653	3.433403	3.378843	35.28246
3.291838	3.464134	3.415606	35.42609
3.317822	3.454699	3.397685	35.42308
3.326112	3.340602	3.367316	35.12529
3.167283	3.1798	3.384572	34.46405
3.274557	3.233311	3.31964	34.67366
3.399401	3.271523	3.419289	35.24815
3.431406	3.444321	3.306476	35.44932
3.235129	3.332245	3.461304	35.11359
3.306604	3.379757	3.375313	35.18574
3.205145	3.383801	3.408359	35.04498

3.30483	3.450588	3.371937	35.32938
3.231004	3.448297	3.459002	35.35332
3.223704	3.418037	3.409825	35.16364
3.089704	3.285878	3.410439	34.58294
3.30681	3.226302	3.360461	34.81813
3.243171	3.19739	3.44473	34.80002
3.334443	3.241129	3.410968	35.02144
3.17747	3.299761	3.465055	34.92466
3.366406	3.350306	3.372431	35.24582
3.40963	3.355226	3.338618	35.27715
3.364303	3.39263	3.40899	35.41372
3.179698	3.377533	3.409945	34.97909
3.236407	3.485431	3.425035	35.37206
3.222384	3.505379	3.38863	35.30541
3.359354	3.407187	3.346871	35.29889
3.359436	3.257101	3.341943	34.96008
3.221147	3.171241	3.452369	34.71138
3.271794	3.314655	3.440433	35.10966
3.341578	3.430437	3.384981	35.3942
3.34661	3.346937	3.34965	35.14534
3.284703	3.310409	3.38771	35.01331
3.22886	3.352733	3.455643	35.1323
3.25575	3.449645	3.362678	35.19974
3.302644	3.45571	3.379951	35.35332
3.267917	3.40375	3.399526	35.20656
3.250348	3.309129	3.493838	35.16746
3.161097	3.276915	3.403704	34.70473
3.309738	3.276645	3.380684	34.97885
3.384141	3.29875	3.373267	35.17368
3.267464	3.248542	3.425905	34.92384
3.203866	3.325236	3.436426	34.97549
3.334608	3.362302	3.410439	35.28563
3.315058	3.35718	3.351697	35.10322
3.384059	3.387036	3.351918	35.31988
3.178831	3.403076	3.369498	34.9446
3.21859	3.47795	3.424149	35.3148
3.223498	3.381847	3.307448	34.86016
3.33135	3.308994	3.35717	35.04544
3.239088	3.254338	3.440774	34.90698
3.305861	3.316946	3.43102	35.16859
3.233149	3.336828	3.447748	35.08963
3.398081	3.392697	3.333861	35.32344
3.38476	3.342556	3.437705	35.41175
3.308542	3.335884	3.325079	34.98419
3.250059	3.36702	3.465533	35.23153
3.267134	3.488598	3.355908	35.29501
3.295921	3.407995	3.375211	35.22391
3.286848	3.365133	3.352737	35.06119
3.228365	3.312566	3.463282	35.06008
3.169345	3.251305	3.393985	34.64551

3.288621	3.235535	3.403602	34.89289
3.319265	3.226302	3.430765	34.99911
3.304706	3.297941	3.379951	35.01282
3.212569	3.342691	3.463026	35.09086
3.319307	3.427337	3.414088	35.40237
3.339846	3.396741	3.370658	35.2854
3.353951	3.318025	3.380974	35.16667
3.187864	3.431718	3.44681	35.19606
3.235789	3.437851	3.364383	35.13402
3.181141	3.310881	3.39569	34.80532
3.339969	3.25609	3.302622	34.82931
3.262391	3.218686	3.460571	34.92327
3.276578	3.386497	3.376592	35.13762
3.272701	3.382184	3.460503	35.30321
3.345001	3.350172	3.375978	35.20647
3.402824	3.253394	3.375842	35.12098
3.354281	3.396741	3.385663	35.34978
3.266144	3.434279	3.391461	35.25181
3.244986	3.447421	3.36619	35.17901
3.300871	3.365807	3.354442	35.09706
3.305655	3.285945	3.395042	35.02166
3.231499	3.33548	3.416851	35.01551
3.148971	3.230143	3.485671	34.75518
3.23781	3.23695	3.384845	34.74385
3.351641	3.251575	3.447339	35.16143
3.312955	3.355361	3.396508	35.19263
3.331143	3.345724	3.385936	35.18821
3.321162	3.370322	3.412008	35.27719
3.210053	3.356641	3.415538	35.01202
3.372634	3.393169	3.328319	35.2567
3.3151	3.448229	3.449504	35.51631
3.300664	3.391956	3.377496	35.2042
3.170665	3.285811	3.346956	34.62101
3.317822	3.255349	3.407762	35.00917
3.153261	3.402873	3.438063	35.03818
3.331515	3.440412	3.408512	35.44546
3.223127	3.399302	3.443434	35.1949
3.403814	3.322675	3.397616	35.32227
3.247502	3.283654	3.396866	34.89347
3.43153	3.404626	3.387931	35.54091
3.360467	3.487048	3.45387	35.70995
3.228818	3.365874	3.378041	34.99125
3.223209	3.252114	3.454023	34.89636
3.282394	3.290056	3.310909	34.7958
3.281445	3.304276	3.42877	35.08256
3.221395	3.277723	3.402715	34.8362
3.143775	3.256494	3.46584	34.75807
3.310439	3.295313	3.451278	35.17559
3.461266	3.373962	3.362064	35.48232
3.361911	3.37969	3.409194	35.38064

3.295798	3.384475	3.400413	35.22732
3.267629	3.329684	3.373182	34.98635
3.292581	3.360078	3.385322	35.13393
3.360839	3.414061	3.337373	35.29639
3.253688	3.304209	3.368561	34.89005
3.065412	3.224348	3.376063	34.32008
3.249811	3.25892	3.425291	34.90659
3.220157	3.406311	3.393456	35.09444
3.386822	3.419587	3.397872	35.4976
3.283177	3.37504	3.408308	35.19635
3.30615	3.286552	3.4109	35.05875
3.270144	3.397415	3.396048	35.18997
3.500076	3.491901	3.370181	35.84285
3.296293	3.394854	3.42333	35.30121
3.265773	3.27988	3.402459	34.9374
3.120843	3.243622	3.40442	34.54546
3.318729	3.288102	3.385749	35.03465
3.202093	3.378679	3.367197	34.93709
3.256204	3.326382	3.431293	35.08122
3.174006	3.259257	3.478236	34.85734
3.390451	3.338849	3.454773	35.45341
3.300376	3.453284	3.371289	35.32411
3.473474	3.411432	3.361945	35.5907
3.246017	3.336086	3.387505	34.98441
3.253771	3.312296	3.346939	34.86063
3.261813	3.38151	3.416373	35.18142
3.297901	3.372277	3.327688	35.04621
3.274021	3.279004	3.38418	34.91355
3.200237	3.261212	3.371545	34.68566
3.179285	3.334806	3.515664	35.11594
3.323018	3.449308	3.461372	35.56193
3.376882	3.43219	3.38036	35.46513
3.289982	3.295717	3.422972	35.06984
3.296705	3.301042	3.460162	35.17751
3.285157	3.462045	3.355874	35.27628
3.383935	3.476602	3.407438	35.63689
3.354363	3.354957	3.33311	35.14366
3.270186	3.224415	3.40633	34.83422
3.048585	3.239848	3.369021	34.30178
3.305119	3.359	3.395161	35.18051
3.217971	3.320653	3.424336	34.96987
3.241274	3.303063	3.353828	34.82818
3.192648	3.307916	3.444525	34.93079
3.320255	3.34141	3.418829	35.2269
3.307841	3.355496	3.411462	35.21445
3.372139	3.387508	3.395775	35.39076
3.228942	3.344915	3.458712	35.1221
3.379976	3.35435	3.311182	35.15039
3.260823	3.27368	3.39378	34.89404
3.266721	3.319575	3.27696	34.75183

3.309779	3.288708	3.352788	34.94432
3.185843	3.380836	3.32762	34.81972
3.232695	3.425989	3.443605	35.27456
3.326277	3.458271	3.352447	35.35046
3.32207	3.335412	3.475849	35.34245
3.401793	3.318833	3.340426	35.18438
3.292127	3.354754	3.381435	35.11279
3.264288	3.451464	3.414259	35.33519
3.296458	3.355765	3.318941	34.98781
3.280868	3.154797	3.364468	34.6138
3.306274	3.209251	3.32646	34.70532
3.209558	3.273882	3.423381	34.8471
3.212445	3.407456	3.407899	35.11167
3.220611	3.297065	3.455353	34.99189
3.315801	3.306635	3.380855	35.05807
3.222797	3.290798	3.414054	34.89265
3.398453	3.357787	3.39187	35.37476
3.243666	3.36547	3.445907	35.17124
3.346238	3.452475	3.389483	35.46243
3.305325	3.362437	3.399185	35.19728
3.391441	3.338243	3.331951	35.18566
3.285652	3.230413	3.400464	34.86833
3.305737	3.216058	3.363974	34.80107
3.108223	3.348487	3.384726	34.70412
3.263669	3.459484	3.363155	35.23962
3.2501	3.404019	3.383685	35.13354
3.229437	3.372681	3.459275	35.18513
3.253152	3.295785	3.3526	34.83555
3.330731	3.391888	3.418215	35.35885
3.29687	3.500257	3.408478	35.5005
3.382615	3.437918	3.399867	35.53285
3.241027	3.164906	3.414941	34.65915
3.289611	3.18937	3.402596	34.7919
3.27431	3.287765	3.34815	34.85455
3.269361	3.427	3.35966	35.17338
3.220735	3.334671	3.378161	34.90559
3.212445	3.230683	3.374529	34.65212
3.242181	3.284058	3.527464	35.16831
3.307511	3.400649	3.350742	35.17968
3.327926	3.407793	3.44473	35.44549
3.224818	3.434548	3.426451	35.23854
3.262514	3.377533	3.444594	35.23597
3.306067	3.255686	3.257095	34.65472
3.425467	3.232502	3.403056	35.18432
3.249151	3.257707	3.356676	34.75244
3.16353	3.283452	3.414054	34.74698
3.19471	3.42417	3.384367	35.05797
3.352507	3.408804	3.359557	35.31519
3.334525	3.405569	3.370931	35.29367
3.305572	3.349498	3.453563	35.28843

3.178625	3.350778	3.474553	35.05952
3.374985	3.468717	3.3607	35.49787
3.386327	3.470671	3.406961	35.62811
3.411321	3.252585	3.322163	35.02041
3.239665	3.135994	3.441115	34.65019
3.33898	3.254068	3.337254	34.89845
3.23748	3.315733	3.412485	34.97586
3.365499	3.44176	3.3858	35.47306
3.115482	3.307377	3.482943	34.84487
3.161056	3.307579	3.430287	34.82983
3.209846	3.334469	3.41049	34.95204
3.358859	3.388653	3.396593	35.36601
3.293859	3.416217	3.459735	35.42222
3.309697	3.430909	3.423654	35.41008
3.259132	3.391686	3.434976	35.23849
3.353621	3.268153	3.296671	34.87253
3.407237	3.26074	3.289748	34.95843
3.241563	3.231559	3.409007	34.79311
3.173264	3.420665	3.407626	35.05427
3.234758	3.421676	3.480197	35.34966
3.316461	3.467167	3.308829	35.25306
3.384512	3.352328	3.328387	35.19351
3.269856	3.343702	3.451295	35.19269
3.185678	3.407524	3.469216	35.18737
3.313862	3.487048	3.427269	35.54986
3.280125	3.331234	3.369174	35.0083
3.413053	3.163491	3.336708	34.86117
3.202258	3.19894	3.359933	34.5285
3.26701	3.285945	3.422222	34.99659
3.24049	3.442501	3.411309	35.25709
3.301201	3.327056	3.395093	35.10193
3.252327	3.280554	3.406125	34.91749
3.201928	3.246048	3.420517	34.76329
3.174006	3.343432	3.449317	34.97817
3.276702	3.487385	3.407353	35.42578
3.318523	3.454093	3.442002	35.52021
3.297324	3.363785	3.365236	35.10848
3.250719	3.22455	3.399407	34.77681
3.306109	3.274151	3.324619	34.84286
3.271629	3.255214	3.346257	34.77336
3.3443	3.428685	3.344143	35.30701
3.1778	3.425787	3.417447	35.09687
3.29819	3.396134	3.399918	35.25697
3.211537	3.330223	3.347297	34.80826
3.244862	3.384138	3.443997	35.2105
3.218838	3.444725	3.45653	35.31349
3.336381	3.457799	3.474212	35.6378
3.203413	3.316273	3.463742	35.01463
3.35259	3.183709	3.325761	34.74922
3.397092	3.241802	3.395434	35.12594

3.278929	3.419857	3.37209	35.20587
3.267917	3.468987	3.50013	35.56923
3.237768	3.382521	3.352856	34.99214
3.277486	3.2292	3.383924	34.81165
3.317451	3.310679	3.350281	35.00366
3.250183	3.357113	3.433595	35.14029
3.102077	3.432122	3.409058	34.92679
3.280826	3.420733	3.39453	35.261
3.315388	3.26876	3.411394	35.04112
3.348796	3.164974	3.355925	34.76592
3.268948	3.192066	3.40442	34.7566
3.278311	3.325168	3.421131	35.10469
3.238016	3.464876	3.432248	35.3464
3.433716	3.47667	3.317542	35.54931
3.264783	3.352261	3.347059	34.97237
3.346486	3.318294	3.285997	34.94323
3.081456	3.374972	3.40916	34.75694
3.218961	3.486442	3.350946	35.1741
3.229107	3.401526	3.438455	35.20196
3.292003	3.334671	3.379934	35.06532
3.22589	3.11025	3.404846	34.48445
3.329906	3.18344	3.401845	34.86541
3.307098	3.385688	3.439359	35.33985
3.407072	3.429629	3.38655	35.53909
3.235582	3.379083	3.424882	35.13736
3.191039	3.282441	3.415128	34.80728
3.174088	3.265188	3.356676	34.60465
3.283219	3.377668	3.371016	35.12064
3.210094	3.415206	3.438608	35.19063
3.2176	3.461169	3.467255	35.3702
3.17846	3.3065	3.504291	35.02737
3.376676	3.198266	3.422972	35.04631
3.469144	3.247194	3.332565	35.15781
3.259998	3.285137	3.375296	34.87687
3.285611	3.380364	3.375705	35.14202
3.245976	3.407119	3.39719	35.16084
3.335721	3.389058	3.33381	35.179
3.333041	3.380566	3.350895	35.19193
3.252863	3.444253	3.459258	35.39284
3.086694	3.493248	3.34312	34.88262
3.265443	3.407793	3.447543	35.315
3.230757	3.346869	3.369038	34.93424
3.228901	3.182159	3.437517	34.71973
3.180976	3.155876	3.39644	34.46762
3.266969	3.295852	3.481476	35.14774
3.27134	3.427068	3.360768	35.18028
3.423611	3.418644	3.423722	35.63252
3.2826	3.316812	3.43484	35.12578
3.300912	3.284193	3.364656	34.94101
3.189307	3.278667	3.398026	34.75784

3.308212	3.42107	3.360427	35.24705
3.152394	3.469728	3.451278	35.21139
3.24911	3.412241	3.418061	35.22454
3.255338	3.241129	3.450135	34.9341
3.40765	3.215047	3.419136	35.14235
3.266185	3.263975	3.421148	34.94439
3.391482	3.402671	3.339624	35.34342
3.275259	3.373962	3.394496	35.14647
3.278847	3.400852	3.390046	35.20339
3.304211	3.373962	3.405716	35.23432
3.239624	3.360078	3.314166	34.86252
3.233108	3.444186	3.444542	35.31731
3.13367	3.479163	3.384913	35.04594
3.1811	3.435222	3.451414	35.199
3.22787	3.270108	3.430628	34.89475
3.27864	3.153045	3.372926	34.62359
3.259462	3.283452	3.397872	34.92138
3.327019	3.368368	3.444474	35.35673
3.264494	3.430572	3.46098	35.39212
3.329411	3.402604	3.305862	35.1337
3.3549	3.216193	3.360376	34.901
3.288539	3.233917	3.344876	34.76075
3.184276	3.357854	3.459684	35.05484
3.296293	3.535572	3.34283	35.4329
3.171779	3.460562	3.411445	35.14663
3.322441	3.26411	3.34711	34.9058
3.172769	3.192268	3.41385	34.56733
3.308913	3.242813	3.37695	34.8949
3.204361	3.344241	3.416237	34.97398
3.332463	3.442434	3.400601	35.43466
3.341495	3.391214	3.381741	35.30116
3.369252	3.278465	3.358313	35.06406
3.212775	3.330088	3.361041	34.84073
3.312708	3.47175	3.429946	35.51974
3.184358	3.47977	3.417652	35.22971
3.205557	3.386092	3.383225	34.99593
3.202835	3.205342	3.410831	34.65507
3.222921	3.18344	3.470512	34.78161
3.32871	3.312903	3.471075	35.2973
3.335226	3.455238	3.427235	35.52695
3.297283	3.41615	3.419971	35.3426
3.293406	3.364931	3.429469	35.24289
3.255091	3.274151	3.426058	34.95312
3.352342	3.304613	3.3511	35.06849
3.1778	3.366144	3.356079	34.83224
3.119152	3.384542	3.420602	34.88532
3.207537	3.39391	3.471075	35.20947
3.199577	3.375781	3.377496	34.94777
3.34529	3.213025	3.381469	34.91919
3.252203	3.188022	3.372738	34.64185

3.297447	3.273006	3.405324	34.9979
3.216899	3.386025	3.366787	34.98464
3.452399	3.461641	3.379133	35.69199
3.310893	3.423833	3.381162	35.3043
3.385708	3.408737	3.385322	35.44399
3.279754	3.393304	3.402971	35.21713
3.305737	3.424574	3.359557	35.2474
3.205887	3.421811	3.401487	35.1147
3.27897	3.384071	3.368356	35.11953
3.167737	3.214306	3.479805	34.74876
3.265566	3.210262	3.423637	34.83102
3.180894	3.348487	3.44814	35.00171
3.393256	3.41487	3.401828	35.51001
3.327308	3.350239	3.441865	35.31201
3.378161	3.362842	3.429793	35.42437
3.233479	3.260942	3.429111	34.88365
3.328133	3.33029	3.354169	35.07841
3.146868	3.406715	3.386891	34.9207
3.242965	3.460697	3.393933	35.2643
3.133422	3.397212	3.452369	35.0137
3.255379	3.25427	3.362473	34.77123
3.317121	3.188224	3.358944	34.75409
3.274392	3.257303	3.373454	34.84345
3.305737	3.396538	3.457979	35.40132
3.246306	3.426663	3.392501	35.19404
3.35094	3.389934	3.390182	35.33747
3.462339	3.35927	3.265279	35.24088
3.387317	3.349093	3.399015	35.34702
3.180481	3.438929	3.426263	35.15075
3.283301	3.502751	3.416254	35.49328
3.225313	3.364594	3.397855	35.02411
3.241769	3.239174	3.343324	34.66657
3.154828	3.157965	3.471484	34.57912
3.230221	3.319642	3.486489	35.13037
3.210713	3.474985	3.434652	35.31406
3.389915	3.443377	3.379815	35.5169
3.270062	3.346128	3.49215	35.28779
3.442253	3.342084	3.342352	35.32792
3.222178	3.226302	3.436562	34.79948
3.284538	3.368637	3.339283	35.03438
3.223044	3.38097	3.398912	35.05727
3.293117	3.368098	3.39037	35.16368
3.176274	3.272736	3.351697	34.61505
3.233396	3.217676	3.380616	34.6828
3.24911	3.244903	3.424899	34.87355
3.277114	3.378342	3.376268	35.12025
3.332752	3.427203	3.444406	35.49778
3.316337	3.389664	3.392416	35.26609
3.379646	3.374703	3.373881	35.33129
3.365004	3.346734	3.383157	35.25839

3.402494	3.504638	3.326597	35.562
3.24115	3.407658	3.382884	35.12018
3.255544	3.353204	3.378212	35.02236
3.202258	3.239444	3.409007	34.7244
3.207784	3.194829	3.327756	34.46123
3.253771	3.198266	3.410269	34.74976
3.264205	3.414802	3.463606	35.36275
3.160437	3.405367	3.48163	35.1546
3.420064	3.360146	3.38418	35.41037
3.26734	3.377601	3.431293	35.21758
3.37713	3.413454	3.386414	35.43794
3.250018	3.302659	3.428173	35.00899
3.279837	3.383464	3.414122	35.22018
3.235912	3.307714	3.350333	34.81898
3.318482	3.32126	3.373864	35.08062
3.198133	3.33393	3.404369	34.91186
3.219621	3.258651	3.350895	34.67729
3.234098	3.239713	3.432606	34.84622
3.278311	3.334469	3.435744	35.15698
3.355518	3.397954	3.417311	35.42435
3.265113	3.395325	3.434908	35.25938
3.32306	3.375579	3.404232	35.27584
3.401752	3.381847	3.32409	35.28637
3.336959	3.426394	3.345541	35.28901
3.254719	3.382184	3.395758	35.1223
3.210465	3.331032	3.374478	34.86712
3.199453	3.303737	3.386226	34.80904
3.300376	3.285069	3.346786	34.90267
3.208939	3.262897	3.388886	34.74629
3.316956	3.372816	3.409007	35.26688
3.150703	3.41615	3.464373	35.11916
3.31943	3.38926	3.368322	35.21929
3.426044	3.360752	3.370794	35.3955
3.351848	3.275904	3.406637	35.12607
3.209888	3.27186	3.400942	34.79433
3.312666	3.415678	3.42582	35.38801
3.207166	3.426596	3.410098	35.14679
3.351683	3.310544	3.315223	35.00156
3.177264	3.27341	3.388272	34.69867
3.145053	3.206353	3.415742	34.54166
3.159942	3.329212	3.434942	34.88488
3.267257	3.415746	3.4412	35.32248
3.363973	3.468245	3.369021	35.49095
3.448027	3.365807	3.392791	35.50273
3.217105	3.340534	3.400191	34.95866
3.370325	3.37942	3.369635	35.31194
3.346362	3.448903	3.388818	35.45343
3.364798	3.357315	3.352396	35.21381
3.170129	3.226976	3.369379	34.54021
3.183162	3.193279	3.328507	34.40564

3.184276	3.315464	3.416493	34.86768
3.316461	3.473232	3.354971	35.36723
3.290477	3.468111	3.463964	35.53756
3.184935	3.330964	3.413236	34.8959
3.288209	3.326314	3.444457	35.17985
3.422786	3.365942	3.383413	35.42732
3.337371	3.378544	3.364417	35.22655
3.213063	3.304546	3.42183	34.91844
3.190751	3.315262	3.454023	34.96348
3.233479	3.318766	3.394394	34.93418
3.3662	3.218889	3.379047	34.97244
3.193638	3.211677	3.380889	34.58334
3.149466	3.303063	3.460162	34.85994
3.083972	3.367896	3.432726	34.7985
3.414826	3.490822	3.398912	35.7169
3.365169	3.451936	3.420653	35.57081
3.400845	3.409478	3.392365	35.49412
3.280744	3.378207	3.459326	35.30953
3.380388	3.467437	3.364929	35.51613
3.309326	3.376388	3.441746	35.32961
3.375315	3.271254	3.345643	35.03384
3.101789	3.132827	3.406807	34.26672
3.219291	3.235804	3.330314	34.5816
3.175408	3.397752	3.426058	35.04916
3.329411	3.500325	3.38343	35.51703
3.316254	3.396875	3.405494	35.31028
3.281033	3.383868	3.494128	35.39864
3.202381	3.369042	3.455097	35.10887
3.440562	3.394988	3.355192	35.468
3.346115	3.310072	3.413082	35.20235
3.347187	3.33548	3.348235	35.11845
3.191988	3.29747	3.478032	34.97978
3.224859	3.226369	3.382526	34.68732
3.330483	3.248003	3.30269	34.79102
3.252987	3.255618	3.366719	34.77823
3.132886	3.352126	3.443519	34.89458
3.210548	3.432931	3.467869	35.29437
3.370407	3.473367	3.37475	35.52875
3.477393	3.402065	3.425564	35.7179
3.313202	3.443107	3.333741	35.2478
3.173099	3.396673	3.457689	35.11093
3.2753	3.447151	3.394991	35.3077
3.27662	3.340602	3.378075	35.04059
3.356756	3.220304	3.342915	34.87587
3.196772	3.194829	3.393319	34.58053
3.128143	3.35718	3.404284	34.80946
3.193143	3.428753	3.440518	35.18736
3.355188	3.449982	3.394257	35.48699
3.227457	3.337502	3.437295	35.0558
3.297736	3.325371	3.390233	35.08004

3.311429	3.34141	3.443383	35.2613
3.354322	3.387642	3.426519	35.41932
3.308047	3.335075	3.344757	35.02437
3.354982	3.308522	3.407575	35.20631
3.235376	3.230346	3.361177	34.67233
3.27167	3.218754	3.386107	34.78087
3.181966	3.302052	3.39719	34.79109
3.22754	3.368368	3.424694	35.09593
3.236531	3.472559	3.443161	35.38382
3.219456	3.451666	3.473257	35.36661
3.313079	3.413117	3.426877	35.38562
3.436974	3.352598	3.342421	35.33952
3.305902	3.395056	3.468943	35.42242
3.245728	3.416487	3.328063	35.02961
3.278847	3.331436	3.439973	35.16077
3.194545	3.236141	3.323476	34.51327
3.333783	3.174139	3.324005	34.68332
3.245893	3.324495	3.37504	34.93153
3.165922	3.466493	3.439359	35.20783
3.234469	3.444927	3.427082	35.28372
3.285776	3.369177	3.400072	35.1712
3.300417	3.370524	3.381861	35.16634
3.298685	3.37841	3.439291	35.30539
3.192854	3.407389	3.375927	34.99876
3.294643	3.37167	3.439956	35.28326
3.237026	3.283384	3.3378	34.7408
3.400515	3.252451	3.385595	35.1352
3.284951	3.233446	3.381162	34.83122
3.257813	3.300839	3.367316	34.88898
3.160313	3.300637	3.379781	34.70258
3.311553	3.365605	3.352021	35.11468
3.175862	3.409006	3.461185	35.15158
3.370077	3.466426	3.316434	35.38532
3.236242	3.358596	3.519433	35.30076
3.289652	3.460562	3.378382	35.33209
3.422044	3.421744	3.412537	35.61141
3.402907	3.414196	3.357562	35.43284
3.240738	3.256023	3.396474	34.81739
3.271134	3.190246	3.31432	34.56036
3.212404	3.205882	3.37932	34.60827
3.298107	3.376657	3.364059	35.13577
3.238841	3.459349	3.398674	35.2627
3.161468	3.376455	3.427201	34.9746
3.193349	3.332312	3.465414	35.03135
3.252781	3.331234	3.415316	35.04941
3.326359	3.424439	3.424183	35.43353
3.244697	3.388182	3.454569	35.24211
3.254183	3.274151	3.428787	34.9571
3.250801	3.24625	3.376711	34.77481
3.424972	3.261144	3.319248	35.0626

3.295179	3.247598	3.39835	34.92213
3.262597	3.325303	3.322879	34.85576
3.202711	3.384003	3.445293	35.12087
3.275217	3.38616	3.327228	35.02595
3.319265	3.415543	3.400396	35.34654
3.347393	3.456317	3.420261	35.54066
3.187575	3.37477	3.444883	35.06668
3.260081	3.362235	3.395093	35.08894
3.295262	3.331706	3.371357	35.04721
3.415569	3.320586	3.391614	35.33028
3.221312	3.234996	3.386959	34.70812
3.263092	3.232907	3.423126	34.87401
3.252863	3.409748	3.385544	35.15618
3.340464	3.444455	3.374563	35.39964
3.226756	3.401863	3.35156	35.00753
3.26767	3.349295	3.452846	35.20354
3.223209	3.342286	3.414634	35.00742
3.271093	3.403615	3.455234	35.33503
3.319059	3.407254	3.425155	35.38211
3.405711	3.293022	3.393371	35.25229
3.281033	3.195368	3.460775	34.91349
3.273609	3.213969	3.370198	34.73985
3.3224	3.248811	3.414787	35.02025
3.318234	3.34296	3.330536	35.03279
3.246512	3.437918	3.426655	35.2938
3.139526	3.43192	3.358006	34.8966
3.285652	3.402402	3.446384	35.34487
3.327266	3.482398	3.335208	35.36768
3.327968	3.378814	3.407438	35.30065
3.23253	3.382386	3.417328	35.12139
3.274434	3.337569	3.444065	35.17348
3.205021	3.26256	3.340289	34.63071
3.421714	3.24652	3.38227	35.16132
3.231087	3.247464	3.376779	34.7345
3.250018	3.336086	3.328848	34.86488
3.305614	3.402941	3.389688	35.26571
3.371727	3.399841	3.306067	35.22065
3.241439	3.393236	3.410644	35.14998
3.276083	3.377196	3.435113	35.24417
3.165881	3.396875	3.50013	35.1884
3.300252	3.386564	3.372159	35.17984
3.231788	3.292685	3.498562	35.10124
3.316708	3.24989	3.375177	34.92354
3.325493	3.162076	3.458337	34.93258
3.366283	3.226571	3.37666	34.98421
3.269938	3.298952	3.485125	35.169
3.347641	3.349093	3.338806	35.12859
3.222673	3.351654	3.372909	34.93549
3.331721	3.377331	3.34728	35.17406
3.188359	3.424035	3.404011	35.08675

3.289488	3.505581	3.387454	35.45002
3.281734	3.435762	3.391529	35.28929
3.339887	3.386295	3.413543	35.35643
3.312089	3.317688	3.367538	35.045
3.310769	3.22428	3.414532	34.94061
3.263298	3.24497	3.423791	34.9023
3.302314	3.350643	3.410081	35.18873
3.236284	3.443984	3.359915	35.13875
3.215662	3.376725	3.408223	35.0522
3.3078	3.328875	3.38759	35.10394
3.201103	3.348756	3.444321	35.03814
3.291838	3.418711	3.428719	35.35543
3.266185	3.465078	3.422853	35.3879
3.251667	3.355765	3.46439	35.20794
3.168603	3.26256	3.420585	34.72667
3.402536	3.215182	3.373864	35.03246
3.282765	3.268288	3.343375	34.82
3.425467	3.268693	3.412144	35.28334
3.257978	3.33858	3.425649	35.09943
3.301366	3.340467	3.401709	35.14609
3.251172	3.352126	3.32143	34.88627
3.361251	3.425855	3.365338	35.38424
3.161427	3.46656	3.376336	35.06033
3.262226	3.431381	3.377683	35.20677
3.198876	3.31762	3.379474	34.82337
3.334484	3.291606	3.346513	34.99096
3.328091	3.334806	3.403329	35.1957
3.349538	3.258381	3.369839	35.00223
3.249028	3.339456	3.433425	35.09878
3.344795	3.423765	3.39748	35.41398
3.288539	3.412511	3.418402	35.31209
3.321575	3.424305	3.323732	35.2031
3.180894	3.329347	3.456598	34.97835
3.215538	3.384677	3.397258	35.04535
3.271382	3.421407	3.36278	35.17239
3.29852	3.42572	3.387215	35.29461
3.2866	3.235872	3.498408	35.09653
3.286353	3.187753	3.412673	34.80328
3.259916	3.249418	3.391	34.83292
3.384595	3.288439	3.372363	35.15015
3.338856	3.360415	3.403585	35.2758
3.259586	3.350778	3.395962	35.06471
3.318358	3.340467	3.394121	35.16666
3.306686	3.401323	3.331934	35.13822
3.328628	3.446545	3.339846	35.3024
3.289281	3.460899	3.386823	35.35048
3.224612	3.364796	3.415776	35.06221
3.139444	3.217136	3.388102	34.49253
3.372469	3.245172	3.349309	34.9786
3.22688	3.246655	3.425854	34.83085

3.322276	3.321529	3.340988	35.01762
3.254802	3.376725	3.482124	35.29941
3.360715	3.44304	3.387522	35.46916
3.368799	3.376927	3.409962	35.39134
3.446914	3.391214	3.377496	35.52241
3.181347	3.382925	3.441337	35.06314
3.256122	3.452003	3.368509	35.21846
3.146909	3.361022	3.422989	34.89981
3.309821	3.303872	3.419101	35.12259
3.251502	3.16403	3.406398	34.66146
3.261401	3.186068	3.477554	34.88691
3.197803	3.28608	3.406807	34.81183
3.362282	3.38434	3.40546	35.38345
3.30351	3.412443	3.374154	35.24792
3.363231	3.331301	3.394411	35.24538
3.278517	3.349767	3.3777	35.06396
3.23418	3.443377	3.366719	35.1477
3.341001	3.46036	3.307669	35.2893
3.309078	3.394584	3.414719	35.30975
3.264824	3.306635	3.420585	35.03347
3.176027	3.208577	3.421403	34.62664
3.26404	3.296998	3.383481	34.92954
3.329947	3.370727	3.390847	35.25101
3.305119	3.347678	3.445548	35.26594
3.235789	3.350037	3.454211	35.13842
3.290354	3.370255	3.425683	35.23958
3.327844	3.351924	3.350367	35.11677
3.377583	3.389934	3.425104	35.4721
3.277733	3.458204	3.409553	35.36903
3.14031	3.423765	3.437091	35.05342
3.165799	3.277993	3.408325	34.72747
3.302108	3.253125	3.362746	34.8715
3.285817	3.180137	3.394803	34.74637
3.2826	3.292011	3.442275	35.0878
3.2209	3.33083	3.425376	35.00081
3.341207	3.33238	3.369839	35.14584
3.450089	3.348756	3.377547	35.43661
3.421219	3.346802	3.335685	35.27766
3.261896	3.405232	3.452642	35.31279
3.258225	3.464606	3.330416	35.16732
3.322152	3.440681	3.436903	35.48766
3.368056	3.376792	3.322317	35.19775
3.230427	3.235198	3.464936	34.89902
3.126452	3.302389	3.416203	34.71201
3.215827	3.300705	3.416373	34.90415
3.431117	3.403817	3.360649	35.47858
3.331638	3.335615	3.394053	35.18494
3.28425	3.347948	3.397958	35.11682
3.249688	3.330358	3.452676	35.12243
3.237521	3.356507	3.412946	35.06612

3.394864	3.427742	3.324772	35.37317
3.277816	3.399369	3.409808	35.24111
3.23385	3.381173	3.436835	35.16428
3.136722	3.265121	3.406006	34.63067
3.327514	3.225224	3.402937	34.95394
3.204732	3.277858	3.428378	34.85617
3.301448	3.304479	3.364673	34.98658
3.159942	3.319507	3.500608	35.00726
3.38843	3.372344	3.352396	35.29836
3.327308	3.357585	3.400618	35.23787
3.41528	3.385755	3.323868	35.32402
3.30747	3.402873	3.433817	35.36613
3.280826	3.449645	3.338737	35.20222
3.227045	3.283856	3.453665	34.97338
3.345744	3.260134	3.2891	34.8212
3.209517	3.255888	3.401913	34.76072
3.2395	3.390473	3.401555	35.11982
3.246842	3.428146	3.473922	35.37651
3.325699	3.408265	3.442581	35.43695
3.437098	3.325168	3.387931	35.37933
3.387317	3.375512	3.388477	35.38175
3.284868	3.416285	3.381025	35.23058
3.28957	3.415948	3.42969	35.34655
3.245934	3.344241	3.390591	35.00881
3.275052	3.321057	3.385186	35.00997
3.248863	3.300772	3.383089	34.90375
3.159859	3.225156	3.420858	34.62635
3.217353	3.256292	3.481186	34.9521
3.252286	3.285541	3.429725	34.97991
3.295921	3.345993	3.432265	35.21309
3.286518	3.402469	3.365474	35.16997
3.351683	3.348285	3.459633	35.39989
3.266556	3.346465	3.328097	34.92211
3.393792	3.441355	3.340187	35.4343
3.352714	3.427337	3.362746	35.36315
3.291467	3.294639	3.390779	35.00032
3.239336	3.22455	3.31935	34.57684
3.289982	3.224819	3.409689	34.88575
3.240738	3.407726	3.358688	35.06651
3.310192	3.449779	3.385067	35.36805
3.186915	3.393775	3.463691	35.14793
3.346527	3.295785	3.401146	35.14591
3.228076	3.34323	3.430355	35.05451
3.469804	3.477141	3.431617	35.87873
3.281074	3.437244	3.442752	35.40311
3.31844	3.322136	3.352464	35.03565
3.231912	3.248137	3.461645	34.92337
3.223539	3.332447	3.336231	34.81517
3.197102	3.270714	3.469216	34.91317
3.222797	3.231896	3.386362	34.70329

3.161468	3.282508	3.494998	34.91742
3.334938	3.360887	3.411394	35.28534
3.363726	3.420665	3.406398	35.4681
3.353374	3.491227	3.375484	35.53216
3.390163	3.359809	3.440501	35.46741
3.305572	3.364526	3.421011	35.25012
3.354281	3.43529	3.356676	35.37069
3.42988	3.428079	3.383771	35.5795
3.295592	3.295044	3.349258	34.91943
3.188441	3.177576	3.364178	34.46085
3.29654	3.25454	3.396235	34.93566
3.215909	3.310679	3.402169	34.89508
3.328298	3.409141	3.369567	35.28488
3.151157	3.346802	3.465414	34.97077
3.301036	3.362976	3.37857	35.14399
3.2607	3.355967	3.411207	35.11183
3.4133	3.505986	3.359745	35.66107
3.286147	3.462045	3.500369	35.59444
3.291302	3.350711	3.321618	34.97134
3.185059	3.265121	3.459735	34.85387
3.262226	3.284395	3.251382	34.60914
3.158292	3.190448	3.44427	34.59822
3.189967	3.255618	3.399782	34.71272
3.126947	3.293628	3.453341	34.77515
3.293859	3.384879	3.439819	35.31014
3.409918	3.369985	3.389125	35.42051
3.406536	3.331032	3.467511	35.49935
3.350198	3.371603	3.427832	35.37809
3.326689	3.419722	3.407148	35.38668
3.374944	3.524856	3.380821	35.66454
3.413218	3.412982	3.30032	35.32755
3.372222	3.318766	3.370539	35.18542
3.118781	3.170096	3.36365	34.291
3.267175	3.319777	3.371954	34.96101
3.254224	3.420059	3.382935	35.176
3.321162	3.385755	3.445975	35.38522
3.255338	3.304748	3.412809	34.9916
3.264741	3.303131	3.437978	35.06366
3.293777	3.37322	3.36793	35.12725
3.465308	3.491901	3.419647	35.875
3.231953	3.363313	3.413764	35.07062
3.354611	3.293628	3.351458	35.05021
3.197803	3.270175	3.386823	34.73335
3.240325	3.370861	3.37903	35.02948
3.172686	3.306231	3.347502	34.67128
3.249028	3.289585	3.351833	34.81129
3.175738	3.334941	3.457416	34.9811
3.274846	3.389664	3.376114	35.13971
3.343764	3.389799	3.447458	35.44674
3.423859	3.347611	3.431362	35.49443

3.286807	3.352665	3.461509	35.2717
3.273238	3.405637	3.378161	35.1756
3.339062	3.376455	3.366719	35.23071
3.385378	3.237422	3.332684	34.95352
3.259009	3.240252	3.390915	34.81071
3.151776	3.301042	3.37446	34.67316
3.226839	3.364661	3.457962	35.15904
3.276207	3.491564	3.359131	35.32839
3.343351	3.360281	3.402237	35.28239
3.285446	3.300368	3.370675	34.95572
3.312996	3.373894	3.422529	35.29015
3.224199	3.505514	3.369243	35.26727
3.45211	3.411028	3.374426	35.57039
3.304995	3.242207	3.414105	34.96626
3.298767	3.229739	3.399185	34.89275
3.256039	3.262223	3.415606	34.90625
3.323555	3.313509	3.362678	35.05031
3.22123	3.290596	3.453477	34.97499
3.253977	3.313442	3.385339	34.94756
3.126081	3.314992	3.508435	34.94045
3.300417	3.470671	3.388272	35.39937
3.288621	3.414128	3.484443	35.46023
3.369706	3.395258	3.418675	35.45246
3.28326	3.330964	3.426451	35.13982
3.318275	3.413656	3.409808	35.36084
3.312996	3.349632	3.369737	35.12165
3.3808	3.254338	3.308062	34.92666
3.244367	3.138825	3.323749	34.41
3.265773	3.293224	3.352157	34.85658
3.153797	3.390877	3.441865	35.02144
3.24379	3.399504	3.364383	35.06766
3.280167	3.407187	3.454159	35.36034
3.290972	3.376253	3.37695	35.14748
3.354735	3.444658	3.473922	35.64857
3.308088	3.522362	3.425666	35.61096
3.330772	3.378814	3.426706	35.34892
3.330483	3.268288	3.388255	35.0225
3.300747	3.234052	3.368407	34.8392
3.187781	3.24962	3.429861	34.7606
3.308377	3.309735	3.352174	34.98589
3.196855	3.282306	3.456871	34.91098
3.207372	3.313846	3.398861	34.8761
3.228447	3.374568	3.40824	35.07549
3.2534	3.379016	3.45225	35.23602
3.225725	3.375781	3.450664	35.16496
3.410578	3.418509	3.439018	35.63717
3.298891	3.39883	3.359285	35.17553
3.391482	3.422081	3.380752	35.47581
3.32937	3.28918	3.338396	34.95672
3.353621	3.174072	3.409416	34.91334

3.230221	3.298211	3.355653	34.79738
3.28759	3.376725	3.393439	35.17717
3.230509	3.4146	3.386209	35.11936
3.266474	3.373085	3.336435	34.99838
3.227664	3.358191	3.40471	35.03024
3.286724	3.412982	3.360836	35.18327
3.327184	3.506188	3.39262	35.54508
3.288663	3.466763	3.439137	35.47635
3.238428	3.343297	3.433885	35.08501
3.24779	3.248474	3.392433	34.80747
3.308212	3.224145	3.328251	34.74604
3.276537	3.332245	3.37221	35.0093
3.184935	3.338378	3.424285	34.93628
3.200567	3.262695	3.374682	34.69648
3.270351	3.374298	3.381042	35.10706
3.289364	3.402536	3.365133	35.1756
3.33135	3.389732	3.457996	35.44249
3.20964	3.396471	3.463146	35.20233
3.290106	3.391619	3.415248	35.26294
3.391647	3.387979	3.375859	35.39089
3.411362	3.335951	3.36822	35.30352
3.283012	3.232165	3.374614	34.80986
3.340547	3.234794	3.334099	34.85283
3.19438	3.298144	3.363002	34.73493
3.331886	3.419317	3.324465	35.21635
3.183533	3.375983	3.398981	34.96011
3.21426	3.369042	3.420312	35.05877
3.176852	3.30859	3.386976	34.77187
3.279012	3.470537	3.418061	35.41741
3.324214	3.479635	3.478356	35.66801
3.391153	3.377601	3.412673	35.44762
3.180069	3.265862	3.482653	34.8947
3.299675	3.216395	3.346394	34.7501
3.315058	3.24652	3.40072	34.96842
3.271629	3.31479	3.333741	34.87628
3.220694	3.230211	3.414924	34.75746
3.191947	3.266873	3.389653	34.71951
3.258514	3.421811	3.376524	35.17519
3.36653	3.475119	3.313996	35.39124
3.328174	3.374501	3.420193	35.31956
3.263422	3.335749	3.399987	35.04903
3.26503	3.324899	3.38435	34.99463
3.384059	3.423968	3.416646	35.54219
3.47133	3.373153	3.309971	35.38864
3.314852	3.210127	3.405716	34.89931
3.312708	3.202108	3.40442	34.87425
3.234593	3.348891	3.394394	35.00249
3.33733	3.437042	3.356011	35.336
3.223746	3.433066	3.422171	35.22359
3.193885	3.362168	3.431788	35.02428

3.141506	3.356507	3.397395	34.82215
3.359354	3.52216	3.404079	35.67542
3.296293	3.4826	3.527311	35.72049
3.391441	3.396673	3.311404	35.2685
3.174955	3.203725	3.453273	34.68338
3.341908	3.190246	3.28893	34.65961
3.396308	3.264312	3.367316	35.11196
3.264329	3.366346	3.355755	35.0212
3.135485	3.237287	3.406466	34.5681
3.220157	3.312633	3.396372	34.89596
3.235871	3.330493	3.351015	34.87019
3.355394	3.368098	3.357903	35.22887
3.242594	3.37632	3.426587	35.15038
3.184234	3.402065	3.489695	35.21706
3.289116	3.442299	3.409604	35.35926
3.320833	3.379151	3.39262	35.25338
3.354157	3.281363	3.310602	34.93305
3.271423	3.177509	3.376524	34.66917
3.272207	3.313577	3.393592	35.00577
3.269814	3.376927	3.340579	35.02314
3.351311	3.446814	3.386908	35.45551
3.279053	3.373422	3.404625	35.17574
3.215538	3.291067	3.434175	34.92137
3.150085	3.395595	3.417379	34.97009
3.355023	3.527417	3.385339	35.63646
3.339681	3.437851	3.405938	35.45209
3.309449	3.324158	3.369516	35.0577
3.180192	3.248744	3.462685	34.81387
3.346445	3.278936	3.387863	35.07984
3.3297	3.347543	3.359285	35.13075
3.373335	3.347408	3.347331	35.19974
3.135196	3.228054	3.440638	34.62201
3.275795	3.298413	3.360512	34.90812
3.203825	3.349969	3.436818	35.03034
3.317203	3.451666	3.413236	35.44911
3.235417	3.427203	3.470461	35.3419
3.29984	3.410354	3.449095	35.39921
3.237686	3.293022	3.424268	34.95241
3.410784	3.244633	3.3895	35.1491
3.328751	3.244768	3.324158	34.82711
3.264824	3.318092	3.321942	34.84281
3.260493	3.419789	3.40517	35.23774
3.281775	3.392225	3.326767	35.05255
3.391936	3.372816	3.285826	35.16148
3.363478	3.32874	3.325096	35.08874
3.234098	3.410489	3.488979	35.34296
3.134948	3.470874	3.418624	35.10433
3.292292	3.426192	3.4161	35.34519
3.376594	3.286282	3.388187	35.16254
3.302314	3.162345	3.345592	34.63592

3.19005	3.240859	3.515886	34.93452
3.277321	3.394988	3.37504	35.15442
3.291962	3.38926	3.380053	35.18487
3.288126	3.259931	3.308539	34.73727
3.26602	3.249755	3.372858	34.80733
3.222797	3.37787	3.373949	34.99536
3.188936	3.432661	3.38655	35.06869
3.280868	3.488531	3.37047	35.35674
3.181059	3.38097	3.451278	35.07997
3.273485	3.313914	3.442002	35.11517
3.225313	3.248677	3.370317	34.7104
3.325493	3.249216	3.399731	34.99498
3.408104	3.277925	3.371562	35.17681
3.308913	3.416015	3.375654	35.27083
3.26767	3.405367	3.40708	35.22608
3.304789	3.36365	3.309682	35.00303
3.24082	3.394719	3.396627	35.12122
3.309243	3.421541	3.365474	35.26138
3.256245	3.44304	3.405239	35.27945
3.139114	3.4671	3.461782	35.19957
3.284208	3.323618	3.400959	35.07008
3.355889	3.19894	3.463845	35.09171
3.281404	3.233715	3.319725	34.6897
3.224323	3.302996	3.436443	34.97163
3.2753	3.382521	3.422631	35.22681
3.241893	3.343971	3.338072	34.88453
3.378532	3.273141	3.369447	35.09706
3.269608	3.272197	3.369839	34.85765
3.266762	3.374366	3.404505	35.15067
3.126246	3.446949	3.392228	34.97526
3.259009	3.411298	3.438455	35.28872
3.213682	3.428011	3.409842	35.16357
3.294272	3.33548	3.499312	35.33311
3.240202	3.227582	3.453733	34.87924
3.40035	3.319575	3.339283	35.18035
3.280785	3.287024	3.403891	34.98899
3.386286	3.40995	3.354016	35.37945
3.289652	3.365942	3.438591	35.25684
3.217889	3.385621	3.348372	34.94564
3.27398	3.359	3.364332	35.04499
3.255503	3.39236	3.383634	35.11975
3.212527	3.430774	3.439614	35.2322
3.26136	3.475793	3.454228	35.46939
3.16683	3.299289	3.443997	34.85431
3.367066	3.193616	3.449845	35.07389
3.333371	3.273478	3.427013	35.12492
3.270681	3.394517	3.407643	35.21016
3.331597	3.355765	3.396304	35.23384
3.247172	3.33548	3.403261	35.02006
3.300706	3.277252	3.372585	34.94272

3.277486	3.322675	3.347485	34.93638
3.193019	3.366144	3.454927	35.08168
3.134825	3.435627	3.382083	34.94707
3.3118	3.428955	3.443298	35.45337
3.284167	3.308455	3.429588	35.09944
3.291055	3.218484	3.330075	34.70013
3.231046	3.243083	3.45653	34.89923
3.239665	3.367155	3.443451	35.16081
3.266267	3.412376	3.365628	35.14769
3.414703	3.411163	3.35792	35.45278
3.36022	3.345926	3.363837	35.20391
3.372923	3.362909	3.389023	35.32391
3.176852	3.399504	3.428906	35.06238
3.29654	3.496753	3.39939	35.47224
3.197226	3.464808	3.40442	35.1962
3.24412	3.374433	3.395894	35.08247
3.217477	3.161806	3.448567	34.67441
3.362035	3.18445	3.359063	34.84432
3.285941	3.36365	3.441456	35.24998
3.434871	3.456182	3.390762	35.66715
3.287962	3.379151	3.426587	35.25578
3.239047	3.300368	3.375927	34.86574
3.244862	3.207027	3.374682	34.67161
3.228983	3.340265	3.349463	34.87311
3.238469	3.46555	3.435624	35.35625
3.156395	3.461304	3.435727	35.16771
3.186214	3.368233	3.474604	35.1144
3.245934	3.190718	3.370163	34.62841
3.360096	3.176902	3.379013	34.8672
3.234139	3.285743	3.382253	34.83686
3.309284	3.41123	3.404846	35.32502
3.280208	3.413387	3.405307	35.26715
3.390493	3.46373	3.309136	35.40811
3.402	3.312835	3.362712	35.22046
3.368964	3.385149	3.342489	35.26212
3.126741	3.400515	3.417192	34.92939
3.299386	3.455845	3.425854	35.44688
3.159158	3.416891	3.410166	35.02073
3.292375	3.212823	3.337646	34.7072
3.167407	3.139903	3.413287	34.43986
3.258225	3.321057	3.440586	35.09432
3.219704	3.439266	3.422103	35.22816
3.341825	3.417633	3.404488	35.4094
3.252781	3.288574	3.424404	34.97599
3.373913	3.209925	3.352805	34.91232
3.240037	3.276375	3.388733	34.84344
3.227581	3.461034	3.343631	35.12139
3.197927	3.470267	3.318276	35.02128
3.2249	3.412578	3.435215	35.20984
3.193225	3.209723	3.381929	34.58043

3.310687	3.149136	3.407234	34.76015
3.256617	3.259257	3.402817	34.87306
3.364922	3.386497	3.443383	35.47687
3.33403	3.41123	3.443724	35.46415
3.394576	3.391349	3.407711	35.47432
3.342856	3.319305	3.356266	35.09117
3.358941	3.32281	3.335259	35.08807
3.271134	3.446208	3.411735	35.33314
3.178337	3.443107	3.357989	35.00589
3.177099	3.356709	3.422495	34.95531
3.212569	3.271658	3.39748	34.79218
3.249523	3.172589	3.395758	34.65258
3.255008	3.260134	3.390029	34.84349
3.350115	3.463663	3.414873	35.55089
3.247089	3.358933	3.375808	35.01114
3.403237	3.353743	3.403806	35.40249
3.375563	3.278128	3.371476	35.10591
3.329411	3.255551	3.367589	34.94711
3.243171	3.361022	3.402664	35.06587
3.276867	3.452071	3.467715	35.48092
3.225065	3.445331	3.415811	35.23939
3.315595	3.341613	3.292238	34.94032
3.184729	3.145699	3.467596	34.60918
3.218508	3.24625	3.386073	34.72466
3.188482	3.312903	3.462958	34.9729
3.282476	3.44277	3.327432	35.16607
3.369087	3.400447	3.417737	35.46041
3.492694	3.386766	3.307243	35.45916
3.23253	3.299694	3.403278	34.90982
3.349455	3.431179	3.357989	35.35402
3.228777	3.434009	3.330229	35.0356
3.31444	3.42882	3.377615	35.31521
3.183409	3.266873	3.38655	34.69405
3.153467	3.193953	3.400055	34.49864
3.250265	3.277858	3.420449	34.93841
3.356013	3.424372	3.453239	35.56177
3.329081	3.467032	3.444764	35.57763
3.210011	3.338715	3.460417	35.07087
3.284745	3.25737	3.412963	34.95263
3.386946	3.210599	3.363377	34.96541
3.312254	3.364189	3.391444	35.19933
3.235789	3.435357	3.451278	35.31859
3.197432	3.427944	3.397736	35.10141
3.251461	3.318227	3.317798	34.80482
3.328133	3.204534	3.38314	34.86675
3.251956	3.196514	3.384248	34.68505
3.234922	3.325168	3.504683	35.19252
3.141135	3.462247	3.408649	35.07718
3.41561	3.458069	3.407012	35.6647
3.42922	3.409882	3.344364	35.45209

3.396267	3.357383	3.322999	35.21849
3.256204	3.339456	3.401351	35.04434
3.303428	3.439873	3.346394	35.24702
3.244615	3.381038	3.384453	35.07297
3.300087	3.277993	3.367401	34.93165
3.10447	3.195436	3.397599	34.38936
3.192029	3.247935	3.440177	34.78876
3.223457	3.429764	3.403823	35.17562
3.312171	3.500999	3.439018	35.60237
3.327926	3.339591	3.437995	35.28161
3.302892	3.288237	3.457433	35.15707
3.208774	3.269501	3.471757	34.9416
3.353745	3.358259	3.298598	35.07406
3.337784	3.384273	3.440774	35.40696
3.353374	3.426394	3.319776	35.26856
3.220487	3.264379	3.387113	34.77091
3.257854	3.248744	3.3238	34.67998
3.181265	3.205882	3.395144	34.57478
3.301201	3.349632	3.347672	35.0476
3.233025	3.447016	3.436716	35.3062
3.224199	3.42572	3.408188	35.17794
3.359106	3.388316	3.387795	35.34657
3.45475	3.374096	3.307789	35.34967
3.32108	3.374703	3.380156	35.21694
3.212569	3.417565	3.380224	35.07352
3.21261	3.408534	3.435198	35.17408
3.199329	3.238702	3.351168	34.58989
3.306975	3.216193	3.335617	34.74206
3.249316	3.228459	3.429043	34.8471
3.229767	3.420396	3.426263	35.218
3.109584	3.481589	3.50129	35.25307
3.380883	3.37349	3.359933	35.30084
3.288374	3.283924	3.474758	35.15377
3.318028	3.33858	3.383089	35.13768
3.279713	3.294033	3.403209	35.00048
3.346032	3.380027	3.394667	35.31488
3.228324	3.38616	3.362337	35.00018
3.423116	3.328471	3.351765	35.27689
3.147569	3.221517	3.352941	34.44299
3.253894	3.234996	3.313348	34.6184
3.145837	3.278667	3.399714	34.66646
3.237603	3.452745	3.392689	35.23246
3.301737	3.434346	3.408717	35.36753
3.356013	3.406647	3.395962	35.39776
3.275506	3.365874	3.434363	35.21651
3.374449	3.405569	3.40251	35.45003
3.344341	3.443849	3.365679	35.38736
3.384554	3.442703	3.385936	35.51709
3.222549	3.266671	3.351168	34.70183
3.152601	3.14361	3.373949	34.32956

3.261071	3.285271	3.332002	34.78483
3.276578	3.362505	3.397736	35.13139
3.204567	3.461573	3.48226	35.3754
3.196731	3.401189	3.435812	35.12464
3.250842	3.302457	3.421557	34.99589
3.376882	3.310207	3.357068	35.14744
3.314151	3.364998	3.449334	35.33184
3.167201	3.369244	3.435863	34.99031
3.254472	3.409478	3.4375	35.27273
3.230386	3.363718	3.372022	34.97679
3.355683	3.241061	3.312205	34.85176
3.265731	3.219293	3.368646	34.73087
3.082528	3.235265	3.403397	34.44116
3.153096	3.350374	3.357648	34.74716
3.324874	3.438323	3.396713	35.40057
3.272413	3.400312	3.333963	35.0655
3.39173	3.400649	3.424063	35.5242
3.270928	3.382925	3.38921	35.14504
3.357828	3.449712	3.326682	35.34439
3.439407	3.431718	3.350469	35.53546
3.362323	3.358798	3.326341	35.15466
3.221642	3.203995	3.419698	34.71264
3.163283	3.231019	3.293397	34.36792
3.095932	3.336828	3.458644	34.81339
3.319471	3.490418	3.347741	35.39558
3.225354	3.412511	3.470393	35.28761
3.183739	3.347476	3.423876	34.95266
3.196566	3.327729	3.465465	35.02848
3.365664	3.416015	3.389074	35.42428
3.316048	3.34168	3.395553	35.16739
3.235252	3.353676	3.392109	35.0094
3.222384	3.349498	3.43941	35.07557
3.260576	3.2385	3.32392	34.66379
3.432107	3.186405	3.295426	34.86267
3.211826	3.228728	3.402937	34.70861
3.158746	3.314049	3.464766	34.91433
3.22057	3.413185	3.435249	35.20177
3.395813	3.467302	3.293602	35.39359
3.420023	3.390001	3.42304	35.56055
3.315595	3.414937	3.359063	35.2468
3.167325	3.343297	3.443366	34.95025
3.34166	3.436099	3.344262	35.31771
3.319224	3.382318	3.344399	35.15134
3.389297	3.256764	3.381042	35.11014
3.167696	3.152506	3.361399	34.35458
3.252657	3.291337	3.415077	34.96137
3.263339	3.464876	3.39025	35.30994
3.282889	3.4239	3.383822	35.24902
3.154663	3.342421	3.376132	34.77362
3.228241	3.352598	3.424404	35.06234

3.195576	3.37194	3.485074	35.16588
3.365004	3.38151	3.342847	35.24629
3.369458	3.316542	3.467647	35.38688
3.350569	3.33858	3.398912	35.24345
3.248368	3.286215	3.447612	35.01193
3.265608	3.212217	3.361655	34.69984
3.364963	3.225628	3.300064	34.81175
3.279919	3.316812	3.388323	35.01819
3.132309	3.405771	3.417175	34.95302
3.199907	3.424035	3.458388	35.23092
3.360839	3.44021	3.36897	35.42267
3.384842	3.368368	3.373795	35.32861
3.312048	3.416824	3.457229	35.45784
3.215084	3.439805	3.370181	35.1057
3.269938	3.422889	3.410763	35.27741
3.298231	3.320114	3.333844	34.94632
3.342362	3.159987	3.390864	34.81734
3.202134	3.210532	3.367077	34.56921
3.262391	3.363313	3.436818	35.1876
3.256245	3.451936	3.419221	35.32948
3.349785	3.420531	3.359199	35.3341
3.202546	3.365672	3.446179	35.08236
3.281528	3.393843	3.407438	35.23196
3.202381	3.382521	3.452233	35.13208
3.282724	3.359068	3.440859	35.23162
3.2972	3.346061	3.376609	35.09432
3.441758	3.345387	3.521002	35.72474
3.226633	3.236546	3.375091	34.69719
3.330896	3.183035	3.375927	34.81001
3.240532	3.262627	3.395775	34.82986
3.240779	3.340399	3.44091	35.09918
3.201598	3.424102	3.38227	35.0683
3.209888	3.462315	3.35908	35.11928
3.21393	3.441827	3.428787	35.23576
3.316048	3.432459	3.320543	35.20188
3.287013	3.379825	3.424814	35.2513
3.309243	3.428281	3.383822	35.31624
3.341825	3.290798	3.418897	35.16354
3.282352	3.17852	3.361928	34.66336
3.332133	3.156078	3.315888	34.62247
3.293364	3.290056	3.387181	34.98658
3.255668	3.48442	3.378706	35.31066
3.255214	3.395932	3.402118	35.16735
3.251874	3.366616	3.448754	35.19792
3.248986	3.358259	3.348337	34.95374
3.268288	3.427472	3.478236	35.43138
3.155323	3.381375	3.418215	34.95227
3.209599	3.304546	3.482107	35.04268
3.255544	3.344713	3.40355	35.0592
3.421384	3.297604	3.386345	35.28122

3.258514	3.217069	3.39262	34.76265
3.280868	3.236074	3.386976	34.84076
3.233273	3.34633	3.440024	35.09379
3.315595	3.389934	3.287924	35.03655
3.196319	3.389058	3.403175	35.02584
3.355931	3.442434	3.374426	35.42874
3.237521	3.379151	3.426587	35.14547
3.290519	3.426933	3.398895	35.3053
3.316172	3.401189	3.455779	35.4295
3.443202	3.366953	3.345081	35.39035
3.293777	3.187753	3.383481	34.75567
3.301531	3.159245	3.304992	34.53864
3.291591	3.240792	3.468329	35.05243
3.352755	3.464336	3.302128	35.31159
3.223251	3.39883	3.39482	35.08783
3.123442	3.329819	3.396866	34.72312
3.260658	3.33056	3.479941	35.20649
3.229437	3.4301	3.413509	35.21061
3.319059	3.39418	3.376063	35.24616
3.220858	3.320384	3.494998	35.13012
3.164561	3.296054	3.410763	34.7696
3.15091	3.27523	3.396372	34.66273
3.425055	3.243353	3.286611	34.9525
3.259009	3.209925	3.377615	34.7153
3.275588	3.310342	3.390506	34.99934
3.221147	3.294572	3.382458	34.8282
3.320874	3.422148	3.3452	35.2438
3.317409	3.494596	3.369857	35.44858
3.313326	3.442231	3.437654	35.47339
3.169016	3.417498	3.440655	35.11028
3.291014	3.379488	3.378996	35.15912
3.28029	3.33986	3.464783	35.23661
3.373624	3.350711	3.349599	35.21255
3.308624	3.184113	3.421062	34.86237
3.287962	3.157493	3.333008	34.56641
3.30483	3.311353	3.456359	35.20951
3.346857	3.457395	3.356761	35.40298
3.280125	3.334738	3.352396	34.97927
3.216982	3.360281	3.417038	35.03841
3.130783	3.390203	3.376455	34.82659
3.244037	3.421339	3.468125	35.34282
3.309985	3.442973	3.400259	35.38593
3.278805	3.297672	3.475286	35.16407
3.229643	3.263031	3.404147	34.82524
3.264865	3.236209	3.363701	34.75516
3.372263	3.206421	3.383242	34.96761
3.325658	3.326651	3.340374	35.03487
3.195906	3.296189	3.423671	34.86667
3.209682	3.341478	3.34832	34.83105
3.318894	3.399841	3.384794	35.27727

3.274557	3.486172	3.396201	35.39405
3.311676	3.435627	3.43941	35.45918
3.219044	3.428483	3.428463	35.21705
3.186915	3.357787	3.437398	35.01173
3.262638	3.320316	3.421148	35.05984
3.414744	3.279206	3.359626	35.16803
3.22952	3.235333	3.37134	34.69265
3.321699	3.318968	3.426314	35.19735
3.276496	3.429898	3.402988	35.29007
3.396432	3.366009	3.343375	35.28228
3.276083	3.321529	3.357903	34.95359
3.263917	3.378746	3.36955	35.07758
3.111605	3.397145	3.453801	34.96898
3.265814	3.502886	3.31079	35.2247
3.19438	3.306905	3.460844	34.96805
3.245481	3.2323	3.370999	34.72018
3.231128	3.228122	3.441183	34.83313
3.327761	3.188966	3.379525	34.82399
3.268206	3.311757	3.458439	35.13485
3.308006	3.364796	3.412127	35.2366
3.265731	3.360955	3.404096	35.11819
3.340712	3.399099	3.31316	35.16671
3.230716	3.40321	3.438711	35.20972
3.282105	3.46036	3.380207	35.31914
3.257978	3.456923	3.357494	35.20919
3.284167	3.354013	3.37516	35.08004
3.328463	3.32099	3.427815	35.21984
3.266927	3.2292	3.409621	34.84476
3.285363	3.24342	3.385322	34.86303
3.384512	3.309331	3.334253	35.11231
3.363726	3.386969	3.373949	35.32345
3.27464	3.411972	3.461031	35.37374
3.287632	3.372748	3.393524	35.16875
3.238387	3.342421	3.369311	34.94179
3.319059	3.440951	3.388255	35.3751
3.188441	3.463865	3.489763	35.36155
3.210919	3.412915	3.450084	35.21252
3.157055	3.25764	3.409536	34.66649
3.285239	3.253259	3.394394	34.90412
3.219951	3.260673	3.49406	34.99551
3.384842	3.212419	3.376012	34.99243
3.221848	3.319979	3.506269	35.15605
3.350115	3.366885	3.352174	35.20215
3.384224	3.39364	3.367111	35.36791
3.399566	3.450386	3.33797	35.46183
3.244821	3.445871	3.402851	35.25544
3.273485	3.451397	3.342932	35.19917
3.173717	3.383666	3.409194	34.97778
3.343269	3.380094	3.367469	35.24951
3.318317	3.277117	3.38921	35.01729

3.220446	3.271186	3.518001	35.07194
3.257153	3.264312	3.419715	34.92224
3.345702	3.366683	3.416134	35.33192
3.319719	3.432661	3.399526	35.38307
3.276083	3.355361	3.445293	35.21868
3.215538	3.30212	3.426468	34.92868
3.22754	3.381038	3.373113	35.01083
3.325782	3.454901	3.380752	35.40391
3.27233	3.49399	3.445889	35.51494
3.227952	3.353676	3.443843	35.10657
3.127525	3.195638	3.373199	34.38686
3.313409	3.213901	3.381997	34.85254
3.284415	3.29538	3.358858	34.91672
3.274887	3.300435	3.417038	35.03417
3.222384	3.368435	3.429878	35.09614
3.298685	3.400784	3.443792	35.36416
3.324709	3.383329	3.295034	35.05759
3.396102	3.384879	3.35983	35.3588
3.227457	3.399099	3.364519	35.03136
3.203	3.432864	3.421693	35.17674
3.216817	3.398291	3.399987	35.08388
3.342238	3.335682	3.332002	35.07257
3.184606	3.253596	3.396304	34.68896
3.291715	3.342893	3.396849	35.11966
3.215373	3.358057	3.467255	35.13984
3.363107	3.388114	3.333179	35.23544
3.312831	3.312094	3.394956	35.09435
3.392596	3.335075	3.348286	35.21698
3.257441	3.359404	3.422563	35.13705
3.261236	3.39054	3.303645	34.95339
3.161757	3.422148	3.424677	35.06964
3.338031	3.385553	3.339641	35.18914
3.182585	3.235333	3.43027	34.71888
3.121792	3.221247	3.405733	34.50148
3.247914	3.2848	3.47012	35.05707
3.342238	3.319912	3.373676	35.12922
3.285734	3.387845	3.37857	35.16491
3.242635	3.405165	3.406858	35.1704
3.286848	3.383734	3.408666	35.22417
3.283342	3.413454	3.361672	35.17873
3.431241	3.433403	3.356761	35.53505
3.273774	3.407995	3.396798	35.22269
3.195947	3.369514	3.332394	34.8275
3.109171	3.283047	3.400822	34.59829
3.32108	3.284126	3.329939	34.90904
3.25707	3.299491	3.402715	34.96182
3.311759	3.355563	3.39864	35.19512
3.251956	3.400178	3.411172	35.18931
3.39371	3.344982	3.335344	35.21278
3.357952	3.331975	3.38493	35.21457

3.400185	3.391147	3.347007	35.3534
3.247337	3.425113	3.420329	35.25377
3.221972	3.391619	3.383242	35.04395
3.161468	3.363785	3.405989	34.90051
3.321492	3.3003	3.348269	34.9854
3.25274	3.242274	3.395843	34.81219
3.145919	3.231087	3.482192	34.74296
3.15359	3.274556	3.457024	34.79976
3.387564	3.312296	3.382816	35.23167
3.354858	3.399706	3.382083	35.3497
3.288085	3.373692	3.424814	35.24023
3.271588	3.395056	3.442087	35.28865
3.277527	3.45288	3.33664	35.19749
3.391111	3.424709	3.328131	35.36567
3.377377	3.406917	3.35804	35.36213
3.198175	3.312162	3.389346	34.83149
3.191988	3.256899	3.406415	34.73444
3.32706	3.312768	3.402306	35.14301
3.256905	3.352396	3.375586	35.01782
3.340093	3.379892	3.362064	35.2303
3.169263	3.299694	3.433749	34.8381
3.335804	3.309129	3.401078	35.15149
3.294313	3.433538	3.409945	35.35221
3.398246	3.45106	3.37915	35.55047
3.280208	3.436907	3.419971	35.35066
3.230509	3.361359	3.426741	35.09157
3.212775	3.298278	3.401896	34.86051
3.331432	3.25737	3.328234	34.86944
3.181677	3.223471	3.39141	34.60598
3.198051	3.264716	3.420466	34.79552
3.192895	3.298144	3.44577	34.91268
3.335515	3.353811	3.391665	35.22799
3.446584	3.378275	3.405904	35.55551
3.393998	3.38589	3.374801	35.38915
3.294519	3.391686	3.417106	35.2768
3.279342	3.448836	3.356556	35.23617
3.320585	3.410152	3.41772	35.37552
3.36587	3.349093	3.29379	35.07001
3.195741	3.228526	3.401385	34.6696
3.118987	3.237354	3.41888	34.55932
3.27299	3.369985	3.440109	35.23256
3.297365	3.424035	3.418829	35.35753
3.331844	3.353339	3.400532	35.23832
3.274392	3.314588	3.42154	35.07388
3.289075	3.359472	3.449794	35.26593
3.352136	3.421272	3.315957	35.2463
3.431406	3.391484	3.362763	35.45687
3.227581	3.387845	3.382799	35.04699
3.256946	3.296594	3.398895	34.94686
3.220817	3.224011	3.399953	34.71143

3.320296	3.255214	3.374136	34.94076
3.226674	3.296054	3.37059	34.81758
3.240284	3.327932	3.439222	35.06714
3.156065	3.356035	3.487018	35.04894
3.368345	3.406108	3.398196	35.42843
3.325493	3.365066	3.411923	35.27498
3.436809	3.383194	3.367793	35.46155
3.25208	3.395528	3.40708	35.17046
3.371397	3.448701	3.389841	35.50998
3.276743	3.31452	3.344194	34.90973
3.303923	3.250564	3.354698	34.85227
3.129917	3.234996	3.376797	34.48603
3.21261	3.354754	3.342813	34.85444
3.204526	3.393708	3.457024	35.17171
3.309284	3.4208	3.408052	35.35295
3.351724	3.356978	3.463436	35.42731
3.357993	3.367087	3.392501	35.308
3.36022	3.49217	3.438881	35.68784
3.330401	3.424574	3.407847	35.40694
3.306191	3.362774	3.415265	35.23507
3.339887	3.330762	3.346445	35.08825
3.29522	3.286484	3.33369	34.86585
3.176893	3.23513	3.425018	34.69451
3.25872	3.277252	3.366719	34.83807
3.189472	3.243892	3.410098	34.70855
3.270433	3.356372	3.403329	35.11677
3.261772	3.387642	3.459889	35.2899
3.275382	3.383734	3.443911	35.27618
3.282146	3.359472	3.383157	35.10505
3.449182	3.44021	3.376336	35.63198
3.296416	3.453014	3.436562	35.45761
3.381708	3.408669	3.354698	35.36813
3.27963	3.220573	3.411121	34.85695
3.284909	3.217878	3.299758	34.61907
3.239047	3.336356	3.375569	34.94365
3.29155	3.45571	3.371357	35.31027
3.258307	3.394517	3.416595	35.20268
3.291302	3.32564	3.391955	35.07033
3.225107	3.372007	3.430151	35.1105
3.386245	3.451936	3.40176	35.57558
3.373583	3.479904	3.405631	35.61752
3.291838	3.338647	3.432265	35.1881
3.273485	3.27213	3.417584	34.97039
3.304913	3.255686	3.39598	34.95591
3.266886	3.25892	3.346138	34.77084
3.248038	3.270175	3.380906	34.83026
3.078775	3.262762	3.413918	34.51609
3.170377	3.332649	3.392501	34.82241
3.303428	3.380634	3.418061	35.2742
3.318028	3.394045	3.407234	35.31178

3.353704	3.395528	3.430969	35.44494
3.230427	3.358933	3.407711	35.04447
3.265979	3.458541	3.396457	35.31543
3.41462	3.437379	3.351509	35.49591
3.338031	3.267749	3.327824	34.90568
3.201309	3.186	3.315769	34.40155
3.307676	3.268153	3.410286	35.02051
3.217064	3.425046	3.38568	35.11165
3.301902	3.426663	3.321873	35.16117
3.196442	3.37632	3.451295	35.10348
3.204774	3.311083	3.458797	34.99545
3.290642	3.406647	3.444491	35.36092
3.48028	3.498033	3.384026	35.84325
3.300376	3.406243	3.447748	35.38845
3.37581	3.28453	3.32883	35.02719
3.240037	3.186337	3.477759	34.84123
3.321162	3.276308	3.32687	34.88542
3.303222	3.252383	3.347621	34.83924
3.264247	3.223	3.37637	34.75262
3.156354	3.291943	3.403346	34.72644
3.234428	3.416824	3.378024	35.11489
3.256493	3.404087	3.406603	35.19779
3.395236	3.39418	3.35156	35.35916
3.279053	3.328538	3.436613	35.14754
3.227292	3.368368	3.458252	35.16877
3.309119	3.438188	3.466999	35.51953
3.412682	3.343163	3.307584	35.18958
3.337949	3.194425	3.372909	34.84374
3.243625	3.186	3.340579	34.54835
3.222549	3.352733	3.411872	35.02278
3.261195	3.462719	3.364178	35.24352
3.311635	3.381375	3.388221	35.22851
3.253317	3.314251	3.393251	34.96519
3.267134	3.346465	3.41726	35.11835
3.285239	3.437649	3.425291	35.37492
3.379976	3.500729	3.398213	35.66082
3.381914	3.380027	3.375432	35.35129
3.334402	3.258718	3.345916	34.91755
3.227127	3.221315	3.416441	34.75539
3.369005	3.288169	3.389568	35.15309
3.195411	3.292954	3.454978	34.92697
3.291467	3.278734	3.341602	34.858
3.162994	3.327729	3.458934	34.94078
3.23649	3.367357	3.366992	34.9871
3.194339	3.357383	3.392211	34.92826
3.294231	3.36702	3.342011	35.05801
3.254266	3.400717	3.510702	35.4132
3.415074	3.44796	3.43172	35.69545
3.237191	3.343837	3.392143	34.9922
3.410826	3.237152	3.346377	35.03853

3.372634	3.19894	3.384095	34.95393
3.258885	3.392225	3.389159	35.13893
3.213476	3.464336	3.364809	35.14408
3.260741	3.410489	3.387471	35.17924
3.291838	3.358596	3.408529	35.17982
3.3264	3.367492	3.396849	35.24931
3.315347	3.449577	3.432879	35.48344
3.292333	3.489744	3.45566	35.57077
3.277816	3.333795	3.417447	35.11442
3.256781	3.254136	3.356369	34.76065
3.412517	3.218821	3.343086	34.99494
3.266886	3.238568	3.428105	34.90558
3.287714	3.266132	3.409672	34.97109
3.231252	3.302996	3.38759	34.87994
3.245687	3.302929	3.376865	34.88791
3.331597	3.400784	3.419306	35.38259
3.270103	3.455441	3.416373	35.36122
3.179574	3.414667	3.363974	34.9595
3.278228	3.313509	3.344961	34.91244
3.26037	3.355293	3.44386	35.18104
3.373047	3.45598	3.407234	35.56753
3.28425	3.362909	3.325113	34.99024
3.301407	3.203523	3.35243	34.73894
3.301861	3.184922	3.329393	34.64888
3.372387	3.338849	3.350827	35.18659
3.186874	3.419048	3.387164	35.03575
3.257359	3.42417	3.358944	35.13938
3.184111	3.348487	3.460434	35.03563
3.316584	3.396201	3.392757	35.28168
3.32871	3.4702	3.449641	35.59441
3.320997	3.385755	3.467869	35.43274
3.230138	3.349295	3.40442	35.01556
3.332051	3.25582	3.391802	35.00642
3.324751	3.294302	3.392228	35.07554
3.314893	3.295044	3.367384	35.00128
3.230716	3.284193	3.44236	34.95743
3.1957	3.257438	3.306817	34.52593
3.325246	3.311353	3.370181	35.0657
3.331762	3.377736	3.358875	35.20039
3.270103	3.381712	3.481851	35.34318
3.124019	3.362976	3.387607	34.77665
3.245687	3.419587	3.440655	35.28252
3.405876	3.486644	3.391819	35.67268
3.363066	3.37942	3.365491	35.287
3.259833	3.223808	3.428667	34.85911
3.326978	3.265458	3.359336	34.9454
3.208815	3.324832	3.417328	34.94366
3.401835	3.415274	3.323118	35.35753
3.219167	3.33703	3.399168	34.95326
3.189678	3.356641	3.436699	35.01374

3.182378	3.314925	3.371323	34.76358
3.314728	3.42417	3.379729	35.31029
3.278599	3.456519	3.461304	35.48042
3.337577	3.37039	3.466096	35.43152
3.160148	3.248542	3.457416	34.75807
3.349373	3.257033	3.312103	34.87267
3.398906	3.348352	3.400464	35.37392
3.333618	3.380634	3.358654	35.2103
3.242058	3.250429	3.395843	34.80667
3.197638	3.279071	3.339607	34.64919
3.281486	3.345656	3.351577	35.00434
3.36818	3.375377	3.338345	35.22998
3.212404	3.400245	3.444065	35.1749
3.126205	3.422755	3.415145	34.97238
3.297942	3.380229	3.405375	35.23358
3.39837	3.348554	3.431992	35.44213
3.346445	3.295515	3.35688	35.04834
3.236242	3.225561	3.435659	34.82664
3.239748	3.32436	3.430407	35.03888
3.261772	3.390743	3.420397	35.21032
3.356302	3.443714	3.358244	35.39696
3.281981	3.389664	3.346649	35.09088
3.196112	3.368705	3.419408	35.01638
3.137794	3.409882	3.425104	34.99135
3.304995	3.513399	3.352856	35.42537
3.288498	3.383599	3.445821	35.30874
3.283961	3.310274	3.367657	34.96754
3.21694	3.184046	3.422989	34.66594
3.298025	3.240926	3.420397	34.96197
3.390534	3.378207	3.460963	35.5532
3.381378	3.315127	3.327364	35.10307
3.20036	3.231761	3.401163	34.68629
3.247378	3.321125	3.374222	34.92562
3.319389	3.396673	3.367384	35.23336
3.388554	3.419317	3.344791	35.38472
3.231458	3.43502	3.392143	35.17907
3.105294	3.367963	3.52003	35.03619
3.26635	3.41123	3.377734	35.17184
3.440356	3.331638	3.381503	35.38655
3.357745	3.272332	3.317235	34.93565
3.226962	3.258651	3.395417	34.7907
3.276578	3.294235	3.396389	34.97915
3.230881	3.316946	3.403209	34.9438
3.474258	3.411769	3.333622	35.53121
3.285487	3.397954	3.396269	35.22519
3.302809	3.389664	3.387573	35.22592
3.104923	3.452677	3.425973	35.01495
3.293983	3.54002	3.425513	35.61839
3.246182	3.38616	3.431651	35.19082
3.310563	3.233446	3.40205	34.93291

3.142084	3.190718	3.499858	34.68492
3.389833	3.265997	3.368816	35.10477
3.282889	3.438727	3.427201	35.37631
3.370613	3.33029	3.380497	35.22888
3.301118	3.253125	3.380701	34.90861
3.196236	3.296189	3.376387	34.76399
3.195865	3.331571	3.343597	34.76884
3.317863	3.431718	3.403414	35.38545
3.20234	3.46036	3.408171	35.20586
3.25142	3.395393	3.465345	35.29614
3.173552	3.248407	3.441337	34.75192
3.403526	3.228661	3.429861	35.18656
3.418703	3.291606	3.402783	35.29819
3.250842	3.406176	3.440024	35.26309
3.314564	3.41925	3.418351	35.38363
3.346403	3.371468	3.375705	35.25551
3.354322	3.357989	3.372329	35.23597
3.330896	3.346263	3.341568	35.09182
3.180399	3.38124	3.439887	35.05421
3.106985	3.503492	3.420926	35.11955
3.304871	3.475187	3.40824	35.46265
3.243625	3.239848	3.412144	34.8226
3.22222	3.158032	3.356318	34.47479
3.229808	3.266671	3.493856	35.02974
3.286353	3.421609	3.441882	35.37856
3.288168	3.433605	3.444048	35.4135
3.399814	3.263436	3.371971	35.12789
3.245646	3.213834	3.416356	34.77935
3.312666	3.344915	3.418283	35.21678
3.196112	3.363246	3.386755	34.93303
3.305078	3.475659	3.37429	35.38989
3.173305	3.461708	3.366992	35.05526
3.262143	3.329953	3.444065	35.12995
3.22622	3.250968	3.407916	34.79961
3.404433	3.2354	3.413168	35.16678
3.277321	3.306837	3.420107	35.0602
3.388472	3.452812	3.438728	35.66321
3.315388	3.374029	3.391802	35.22849
3.321162	3.375377	3.349105	35.15069
3.280496	3.33184	3.369021	35.01011
3.331844	3.410758	3.357153	35.26902
3.234428	3.459147	3.39985	35.25518
3.178089	3.456991	3.421472	35.17454
3.223581	3.266469	3.445054	34.90895
3.287632	3.16713	3.398213	34.72935
3.278311	3.239174	3.39835	34.86682
3.250966	3.333458	3.410985	35.04083
3.350816	3.38771	3.483761	35.53698
3.241356	3.335817	3.394667	34.98929
3.374325	3.246453	3.387386	35.06872

3.377913	3.322405	3.344177	35.14818
3.323018	3.408265	3.368612	35.26933
3.236902	3.417767	3.439478	35.25676
3.254059	3.469661	3.418471	35.36182
3.203701	3.340736	3.388954	34.90521
3.331267	3.259257	3.270753	34.74751
3.212156	3.229065	3.441337	34.79405
3.26668	3.265458	3.423995	34.95494
3.233561	3.35098	3.387982	34.99079
3.438912	3.390001	3.387027	35.5231
3.29456	3.366548	3.412929	35.21278
3.372634	3.362168	3.410235	35.36804
3.242882	3.344241	3.349122	34.91145
3.345042	3.424574	3.384504	35.38791
3.188854	3.501268	3.427747	35.30863
3.219539	3.446679	3.392757	35.17984
3.189142	3.203995	3.44855	34.70467
3.219539	3.199344	3.386772	34.62587
3.358075	3.290259	3.462753	35.2938
3.35956	3.436907	3.404215	35.48973
3.256575	3.399975	3.442616	35.26773
3.303634	3.284732	3.340664	34.89567
3.261896	3.271254	3.384453	34.87068
3.368015	3.306366	3.335805	35.07315
3.179945	3.369716	3.339488	34.80846
3.11478	3.4084	3.405392	34.89467
3.245976	3.463595	3.386141	35.26018
3.240573	3.331706	3.443161	35.08464
3.334649	3.290056	3.377138	35.0549
3.271175	3.231289	3.381213	34.79649
3.285116	3.277521	3.435266	35.04629
3.201392	3.418172	3.404574	35.10366
3.429591	3.440816	3.423995	35.69468
3.307098	3.393101	3.391597	35.25162
3.395112	3.34323	3.341653	35.22581
3.199618	3.343163	3.424132	34.97852
3.338691	3.446747	3.285485	35.20597
3.221601	3.468919	3.394274	35.2363
3.259462	3.274556	3.390711	34.88627
3.178378	3.162278	3.482789	34.66477
3.20597	3.297537	3.418948	34.88129
3.281198	3.368435	3.402237	35.1643
3.420147	3.396404	3.428684	35.58716
3.232943	3.342826	3.430628	35.06486
3.295715	3.26074	3.392637	34.93955
3.288333	3.304883	3.382458	34.99767
3.342733	3.401593	3.375125	35.31209
3.23286	3.365268	3.34994	34.93731
3.132309	3.367627	3.416373	34.86785
3.199371	3.414533	3.394274	35.06876

3.258101	3.320384	3.357085	34.90997
3.316048	3.308657	3.404147	35.11397
3.208197	3.280149	3.394667	34.79504
3.289116	3.295246	3.453597	35.13388
3.299386	3.377803	3.392927	35.20421
3.470794	3.390069	3.366924	35.549
3.323307	3.409882	3.39291	35.32663
3.35824	3.416689	3.311404	35.23967
3.274475	3.372142	3.440706	35.24183
3.307057	3.443714	3.417328	35.41848
3.269155	3.452543	3.405511	35.32906
3.285569	3.261144	3.325659	34.77177
3.136887	3.163895	3.491741	34.59715
3.261648	3.290798	3.406483	34.96106
3.236943	3.355428	3.398009	35.02984
3.404639	3.423631	3.400072	35.55022
3.239006	3.314318	3.408478	34.96734
3.404227	3.253799	3.412826	35.20581
3.222261	3.29747	3.378178	34.82761
3.320296	3.367357	3.343512	35.11903
3.163654	3.410556	3.356096	34.89846
3.282641	3.457597	3.418607	35.39824
3.126246	3.34788	3.381861	34.73594
3.251461	3.187214	3.373199	34.63947
3.325246	3.297807	3.363513	35.02149
3.277733	3.319507	3.427337	35.10462
3.332711	3.459349	3.438319	35.55467
3.339928	3.454699	3.402732	35.48247
3.371892	3.368368	3.345763	35.23899
3.426127	3.352665	3.29529	35.21288
3.297984	3.386834	3.352106	35.13162
3.228241	3.444321	3.415128	35.24264
3.278434	3.422822	3.398776	35.26963
3.210878	3.348554	3.375381	34.90832
3.233479	3.152708	3.381179	34.54214
3.214012	3.279071	3.433612	34.89057
3.184441	3.388653	3.500523	35.21186
3.265319	3.426596	3.377427	35.20251
3.37746	3.379286	3.381793	35.35383
3.231829	3.29437	3.394462	34.87737
3.374325	3.294976	3.413713	35.23241
3.243419	3.328538	3.406193	35.00309
3.295427	3.40503	3.366787	35.19792
3.22024	3.450116	3.364127	35.12628
3.290271	3.363583	3.353999	35.06804
3.206671	3.224415	3.377973	34.63331
3.250512	3.205545	3.382253	34.69728
3.202093	3.305624	3.441081	34.9389
3.319018	3.424844	3.403533	35.3732
3.333865	3.444792	3.468687	35.59178

3.420435	3.404221	3.375313	35.48817
3.378573	3.304209	3.406415	35.24593
3.40633	3.413185	3.348508	35.41831
3.32009	3.475254	3.389415	35.45491
3.253894	3.37841	3.420397	35.16612
3.227086	3.345252	3.399441	34.98916
3.191617	3.2292	3.371766	34.59728
3.242594	3.225224	3.396576	34.75432
3.261071	3.314251	3.374154	34.94038
3.237974	3.419924	3.500676	35.39765
3.153384	3.400852	3.421744	34.99835
3.406784	3.35927	3.472507	35.57257
3.283631	3.339523	3.372227	35.04077
3.400391	3.313577	3.383754	35.26457
3.284827	3.383666	3.456103	35.32335
3.32273	3.40503	3.420176	35.37438
3.216033	3.357517	3.379474	34.94814
3.35791	3.303468	3.337783	35.04904
3.199577	3.224145	3.464663	34.80679
3.215703	3.261751	3.331951	34.63407
3.185059	3.290461	3.452915	34.89437
3.290972	3.360146	3.413372	35.1919
3.359931	3.454093	3.453904	35.63679
3.373294	3.411298	3.411292	35.47924
3.31312	3.326516	3.373028	35.07857
3.393256	3.405434	3.38493	35.45242
3.285487	3.399032	3.371067	35.17243
3.331597	3.412915	3.352737	35.26354
3.136186	3.322203	3.397344	34.73538
3.157674	3.223876	3.346053	34.45518
3.255668	3.256899	3.41963	34.9026
3.254843	3.372007	3.402459	35.11497
3.29852	3.391619	3.476736	35.4158
3.215456	3.373288	3.403602	35.03413
3.28924	3.339052	3.4397	35.19956
3.371273	3.331571	3.350605	35.16776
3.319348	3.268356	3.387454	34.99654
3.192978	3.310274	3.460366	34.97131
3.33568	3.44796	3.426229	35.50982
3.230674	3.394786	3.435659	35.18453
3.334154	3.258853	3.339761	34.90385
3.210548	3.192875	3.398895	34.61857
3.142661	3.255955	3.424609	34.66429
3.208692	3.351991	3.449862	35.07393
3.348012	3.436503	3.441473	35.54507
3.388142	3.415072	3.371374	35.43267
3.389791	3.377264	3.408308	35.43436
3.252533	3.360281	3.484426	35.26352
3.314357	3.417902	3.330962	35.18913
3.386987	3.401795	3.414122	35.49459

3.388554	3.338984	3.292579	35.09487
3.218136	3.180339	3.395775	34.60093
3.19801	3.262021	3.299519	34.52504
3.148146	3.388653	3.429486	34.97714
3.338732	3.489811	3.385459	35.51886
3.284456	3.375916	3.456001	35.30536
3.173965	3.303737	3.492491	34.98569
3.273568	3.362505	3.395621	35.12018
3.430664	3.33056	3.402851	35.40968
3.312584	3.365268	3.357631	35.12847
3.216033	3.385958	3.462753	35.19246
3.181389	3.342691	3.425649	34.94094
3.182048	3.276915	3.374205	34.68604
3.385172	3.207027	3.376677	34.98281
3.236902	3.23695	3.43131	34.84348
3.200196	3.342286	3.412008	34.95135
3.122947	3.416487	3.471944	35.07575
3.442171	3.484015	3.363718	35.68485
3.34496	3.435829	3.371613	35.38415
3.32306	3.37632	3.329751	35.11458
3.245522	3.396134	3.410849	35.16569
3.376676	3.462315	3.418266	35.61345
3.254389	3.392495	3.333298	35.00753
3.348094	3.152101	3.315769	34.64842
3.1048	3.123324	3.390506	34.21688
3.187781	3.291337	3.457092	34.91137
3.211166	3.484083	3.412809	35.28718
3.297447	3.447151	3.390591	35.34651
3.263958	3.373422	3.426741	35.19109
3.273856	3.390203	3.468534	35.34083
3.231623	3.394045	3.483608	35.28984
3.384017	3.369716	3.390046	35.36529
3.344589	3.334671	3.391427	35.20545
3.206382	3.348756	3.360103	34.86552
3.242924	3.267479	3.426502	34.91289
3.234758	3.259797	3.357852	34.72811
3.329205	3.218282	3.291812	34.69944
3.281569	3.261144	3.38493	34.89264
3.139197	3.403817	3.432197	34.99666
3.259297	3.441018	3.467323	35.41747
3.328421	3.455171	3.409553	35.47325
3.469804	3.395528	3.335753	35.49061
3.319471	3.395123	3.375654	35.24823
3.216693	3.402402	3.374836	35.0376
3.314646	3.399975	3.448703	35.40804
3.232778	3.272399	3.303407	34.63228
3.347435	3.165041	3.397923	34.85493
3.21195	3.21188	3.382543	34.62744
3.165963	3.38899	3.377888	34.90401
3.18609	3.40685	3.408461	35.05394

3.316337	3.39701	3.393047	35.28354
3.239954	3.347678	3.422972	35.07406
3.322936	3.35563	3.399594	35.2218
3.254554	3.328403	3.407711	35.03047
3.365045	3.380903	3.373864	35.31288
3.32937	3.375579	3.374358	35.2243
3.408763	3.387171	3.38036	35.4364
3.215167	3.23385	3.375705	34.66757
3.267958	3.20231	3.334935	34.62488
3.158705	3.241466	3.414804	34.64625
3.27299	3.35098	3.35035	34.99471
3.243707	3.438727	3.479447	35.40488
3.269278	3.465415	3.349923	35.23591
3.290065	3.34788	3.433493	35.2071
3.340547	3.405704	3.398281	35.36694
3.281239	3.448027	3.42952	35.39811
3.308624	3.39236	3.420738	35.31706
3.337412	3.280756	3.352464	34.98665
3.198834	3.168074	3.427201	34.60062
3.336587	3.178857	3.324022	34.69981
3.302355	3.416083	3.413304	35.33897
3.283548	3.467437	3.367964	35.31099
3.23418	3.413319	3.438063	35.23798
3.263133	3.352126	3.48122	35.26186
3.280909	3.369042	3.336623	35.02151
3.302438	3.355159	3.424149	35.22964
3.160231	3.3559	3.457365	34.99291
3.234881	3.353272	3.445889	35.12532
3.292704	3.369985	3.412281	35.21482
3.360302	3.225156	3.312222	34.82712
3.293241	3.132153	3.406262	34.68273
3.191576	3.232637	3.341295	34.53808
3.154044	3.26566	3.485534	34.84364
3.277692	3.485633	3.272577	35.12938
3.237397	3.433942	3.425496	35.26263
3.361457	3.456586	3.397719	35.52271
3.231334	3.355428	3.41697	35.05903
3.332628	3.363785	3.410797	35.28532
3.40633	3.413319	3.341756	35.40384
3.455121	3.414735	3.407779	35.65802
3.298314	3.222393	3.413918	34.90791
3.222343	3.125885	3.334304	34.35662
3.255214	3.255079	3.378792	34.80832
3.316461	3.432796	3.361246	35.29252
3.298355	3.422215	3.428224	35.37626
3.087395	3.371805	3.444866	34.84108
3.2282	3.311622	3.435045	34.99591
3.342898	3.378814	3.391529	35.29851
3.309491	3.365066	3.380633	35.17156
3.237191	3.33393	3.465891	35.13181

3.24214	3.332919	3.427337	35.05611
3.254018	3.347341	3.310926	34.85905
3.439077	3.198199	3.321089	34.95982
3.269567	3.226706	3.364536	34.74649
3.20865	3.268625	3.392689	34.7665
3.172109	3.314857	3.358466	34.71286
3.349208	3.417296	3.500659	35.63511
3.324544	3.456923	3.315292	35.26247
3.371026	3.361965	3.357443	35.24864
3.195618	3.431583	3.485671	35.2977
3.311223	3.440075	3.382799	35.34412
3.321575	3.390473	3.428036	35.35721
3.398246	3.286754	3.365065	35.16036
3.312543	3.15911	3.373199	34.71159
3.246966	3.244363	3.389142	34.78948
3.305407	3.410691	3.427542	35.36499
3.362447	3.457934	3.364247	35.45462
3.243377	3.321057	3.354169	34.87287
3.247832	3.315059	3.419869	35.01317
3.15722	3.345252	3.459804	34.96837
3.336093	3.380903	3.357443	35.21366
3.279218	3.359135	3.493924	35.34014
3.359271	3.337367	3.389568	35.23939
3.278805	3.302929	3.508997	35.24929
3.34265	3.258988	3.268246	34.76633
3.300912	3.183642	3.365287	34.7225
3.298726	3.274286	3.281683	34.73311
3.143651	3.34815	3.407012	34.82959
3.143197	3.420059	3.459053	35.09966
3.317244	3.459417	3.359199	35.34798
3.294684	3.420396	3.387846	35.27595
3.293983	3.406984	3.428991	35.33507
3.249316	3.429831	3.404079	35.23288
3.343104	3.368233	3.447271	35.39772
3.203083	3.282373	3.363377	34.72029
3.393132	3.203725	3.395366	35.03387
3.247131	3.198873	3.409604	34.73511
3.324421	3.406378	3.351219	35.23023
3.310975	3.453351	3.400447	35.41121
3.355766	3.410624	3.370743	35.35076
3.20234	3.392427	3.462464	35.17603
3.264618	3.445129	3.335259	35.14929
3.171696	3.406917	3.58	35.39773
3.263504	3.430505	3.367384	35.18513
3.16683	3.296391	3.463146	34.88985
3.326318	3.284328	3.396252	35.06596
3.310769	3.294909	3.388051	35.03716
3.385708	3.144419	3.424882	34.95248
3.238263	3.287158	3.39054	34.86709
3.292003	3.329886	3.428633	35.16136

3.228282	3.381847	3.34653	34.95609
3.300912	3.444186	3.355874	35.27168
3.225107	3.384677	3.354561	34.9729
3.274434	3.427203	3.385476	35.24137
3.299427	3.445399	3.400805	35.36934
3.255915	3.435492	3.428019	35.31204
3.395566	3.327595	3.385271	35.28799
3.247625	3.219765	3.373454	34.70282
3.301448	3.176431	3.335054	34.64179
3.389462	3.31607	3.378382	35.23438
3.345826	3.466493	3.363769	35.43595
3.27497	3.381577	3.42761	35.23491
3.239624	3.348082	3.419647	35.06695
3.243089	3.369109	3.361467	34.99328
3.259174	3.389597	3.418556	35.1981
3.201309	3.459484	3.427013	35.24289
3.227787	3.335075	3.511436	35.21335
3.148518	3.256899	3.367725	34.55477
3.385956	3.28298	3.422239	35.25026
3.244202	3.215519	3.397872	34.73945
3.422291	3.273208	3.354033	35.15919
3.196236	3.265121	3.42969	34.81261
3.335433	3.337232	3.322999	35.04139
3.297654	3.38926	3.431191	35.30915
3.338938	3.407658	3.314132	35.18368
3.263793	3.436975	3.402203	35.27605
3.258266	3.399302	3.420107	35.22074
3.235747	3.396741	3.403346	35.12924
3.398453	3.385755	3.417055	35.491
3.365623	3.249485	3.366804	35.01132
3.286972	3.216125	3.449573	34.94737
3.264618	3.258853	3.430645	34.95053
3.35725	3.438929	3.348985	35.36832
3.272536	3.39054	3.385868	35.15791
3.277651	3.336221	3.358841	34.9912
3.168232	3.347476	3.484341	35.05098
3.243584	3.462112	3.363377	35.20192
3.269031	3.449914	3.442155	35.40317
3.240037	3.403884	3.387317	35.11919
3.313326	3.313105	3.451823	35.222
3.142661	3.174678	3.442138	34.52489
3.368716	3.276038	3.412417	35.1759
3.39903	3.272938	3.39794	35.20375
3.333288	3.291135	3.360444	35.01778
3.206258	3.335817	3.436289	35.00356
3.291178	3.373153	3.381997	35.15219
3.284373	3.444927	3.38875	35.30903
3.368097	3.460428	3.328779	35.39487
3.214837	3.399706	3.424831	35.13698
3.201309	3.445197	3.434243	35.22746

3.223333	3.301648	3.394803	34.87545
3.321162	3.29383	3.363189	35.00316
3.172521	3.276173	3.410218	34.74234
3.363808	3.290191	3.390301	35.14775
3.263669	3.376725	3.44207	35.23121
3.381172	3.460765	3.344109	35.45772
3.351394	3.359068	3.396576	35.28495
3.36917	3.357315	3.347519	35.21271
3.194463	3.378612	3.392706	34.97604
3.251956	3.457462	3.407575	35.30672
3.143486	3.408939	3.417089	34.9842
3.298066	3.351789	3.381639	35.11975
3.241233	3.218754	3.435317	34.82192
3.126741	3.21626	3.455455	34.61013
3.307882	3.214306	3.381162	34.83951
3.305531	3.309264	3.456478	35.20674
3.352837	3.440075	3.389346	35.44944
3.298767	3.397549	3.428036	35.32281
3.258431	3.375714	3.366208	35.05164
3.263545	3.442703	3.353692	35.18195
3.422869	3.444118	3.351356	35.52835
3.272207	3.412309	3.42038	35.28026
3.260246	3.373692	3.427866	35.18603
3.140063	3.25791	3.365065	34.53267
3.345001	3.216597	3.363019	34.88602
3.25905	3.308859	3.371204	34.91772
3.265443	3.371266	3.402084	35.13571
3.253523	3.394786	3.408359	35.1748
3.382656	3.398762	3.39262	35.43147
3.282064	3.335278	3.381503	35.04834
3.392761	3.380364	3.341483	35.3015
3.196855	3.418913	3.448498	35.19141
3.151652	3.429696	3.435164	35.08698
3.16984	3.322068	3.434704	34.89039
3.282641	3.231154	3.425445	34.918
3.259916	3.231087	3.385254	34.78027
3.272371	3.249688	3.406466	34.89457
3.196154	3.280149	3.456888	34.90477
3.362942	3.364257	3.303833	35.11873
3.431489	3.375714	3.409484	35.52473
3.304005	3.391349	3.325727	35.09697
3.319389	3.387979	3.397958	35.2812
3.286064	3.443107	3.307124	35.13025
3.297654	3.417161	3.39864	35.29898
3.330071	3.416487	3.291471	35.13403
3.225725	3.295448	3.461389	35.01274
3.160891	3.264177	3.388613	34.64342
3.28392	3.264514	3.438898	35.02317
3.37383	3.340265	3.421489	35.34737
3.313244	3.353406	3.37209	35.13559

3.261813	3.3435	3.445156	35.16124
3.295674	3.348958	3.40234	35.15359
3.271918	3.361965	3.352669	35.02146
3.393833	3.421878	3.356676	35.42786
3.259256	3.407254	3.40928	35.21661
3.32339	3.402604	3.382611	35.28837
3.072011	3.250564	3.463231	34.58246
3.298107	3.263436	3.369584	34.90026
3.237067	3.193077	3.406415	34.69345
3.234634	3.320653	3.397122	34.9468
3.25208	3.299828	3.470734	35.10039
3.436149	3.361224	3.349122	35.37123
3.364592	3.327729	3.366753	35.18006
3.444398	3.433875	3.329325	35.50485
3.274062	3.419857	3.395962	35.24743
3.221518	3.424574	3.341875	35.02456
3.195411	3.32409	3.364946	34.79818
3.265525	3.270782	3.386465	34.88199
3.210011	3.33056	3.410763	34.94445
3.229148	3.298885	3.434908	34.96983
3.215868	3.353878	3.425172	35.03976
3.395153	3.38899	3.434925	35.52994
3.382491	3.402402	3.363923	35.37631
3.308954	3.346398	3.374409	35.11596
3.344547	3.344443	3.411735	35.27114
3.268742	3.386699	3.399816	35.17171
3.359849	3.379825	3.341824	35.22909
3.32937	3.38151	3.398503	35.29007
3.283425	3.32409	3.41813	35.10695
3.069248	3.190179	3.38256	34.26795
3.256988	3.194492	3.405767	34.73869
3.255462	3.267749	3.394326	34.87054
3.251915	3.372748	3.422836	35.15474
3.247997	3.410826	3.435693	35.25756
3.305614	3.367761	3.433476	35.28454
3.379316	3.324697	3.285485	35.02791
3.391606	3.432527	3.395962	35.53219
3.295757	3.444792	3.375705	35.3051
3.324256	3.422485	3.387641	35.34474
3.195494	3.318564	3.338379	34.72818
3.23616	3.27961	3.32305	34.6984
3.202959	3.330088	3.410286	34.92695
3.269484	3.406041	3.36249	35.13401
3.226426	3.386901	3.497061	35.29227
3.314357	3.400515	3.421813	35.34978
3.360467	3.361561	3.395894	35.30875
3.486012	3.394045	3.369174	35.5959
3.316749	3.380431	3.471876	35.42057
3.263834	3.388316	3.352447	35.06093
3.259421	3.361224	3.449521	35.20432

3.299345	3.353676	3.392501	35.15042
3.257153	3.241398	3.420755	34.87441
3.170212	3.217541	3.411241	34.6113
3.13169	3.220034	3.48654	34.69718
3.282806	3.36082	3.439291	35.2322
3.274227	3.381577	3.43247	35.24391
3.292746	3.390136	3.382065	35.1929
3.340299	3.340265	3.427082	35.28628
3.248492	3.383801	3.419101	35.16326
3.40134	3.470132	3.378775	35.59812
3.368304	3.40968	3.393064	35.42493
3.401835	3.336558	3.347468	35.23864
3.170665	3.224887	3.387931	34.57738
3.267299	3.286282	3.358176	34.8579
3.133711	3.380162	3.411872	34.88849
3.34133	3.434144	3.380207	35.39132
3.178254	3.378544	3.442547	35.04944
3.311553	3.342556	3.413304	35.19829
3.267381	3.386834	3.43027	35.23563
3.426251	3.433605	3.38684	35.59036
3.303716	3.397684	3.425172	35.32767
3.276702	3.358259	3.391342	35.10839
3.186791	3.302187	3.436767	34.88849
3.171738	3.302929	3.309153	34.57812
3.279053	3.237759	3.360069	34.78163
3.224694	3.223943	3.369157	34.65242
3.187039	3.278465	3.463418	34.89544
3.174418	3.378679	3.419766	34.99153
3.35758	3.424911	3.416356	35.48572
3.31147	3.356304	3.364673	35.12183
3.331061	3.284126	3.394752	35.07261
3.345991	3.429359	3.405852	35.44713
3.330112	3.467908	3.392211	35.46688
3.307965	3.369851	3.330484	35.06902
3.374944	3.277252	3.34121	35.03645
3.079847	3.211206	3.360427	34.28871
3.258967	3.295448	3.424831	35.00549
3.20898	3.383464	3.430697	35.10148
3.29122	3.456991	3.370607	35.31071
3.243831	3.324764	3.435948	35.06081
3.284745	3.323551	3.410354	35.09166
3.260988	3.410219	3.432061	35.2767
3.390039	3.473906	3.382833	35.59054
3.277279	3.376051	3.409194	35.1876
3.348754	3.323012	3.386431	35.17814
3.215414	3.276578	3.369447	34.74786
3.350198	3.246385	3.34283	34.91838
3.124926	3.254607	3.381059	34.52733
3.26635	3.298076	3.372585	34.91313
3.17945	3.337636	3.463691	35.00884

3.307428	3.37659	3.415487	35.26847
3.240655	3.382116	3.423194	35.15139
3.433015	3.353002	3.37313	35.3989
3.30714	3.384003	3.378689	35.20358
3.34995	3.448364	3.411957	35.5107
3.279424	3.373759	3.365491	35.09171
3.263504	3.260403	3.365611	34.80927
3.320585	3.19274	3.302349	34.64778
3.252368	3.2817	3.424422	34.9601
3.184688	3.419789	3.399867	35.06037
3.224447	3.389327	3.406944	35.09618
3.362406	3.389529	3.39112	35.36371
3.271794	3.336356	3.410013	35.09059
3.332092	3.421002	3.4508	35.49676
3.270928	3.456384	3.372772	35.26974
3.324998	3.404491	3.433902	35.40818
3.332834	3.322742	3.347093	35.05671
3.392472	3.294841	3.418948	35.28325
3.176975	3.252181	3.395144	34.66665
3.236985	3.226908	3.351833	34.64789
3.202546	3.236681	3.372397	34.63892
3.187823	3.271119	3.403823	34.75076
3.204155	3.408198	3.412554	35.10534
3.304005	3.427944	3.405784	35.35207
3.214342	3.368098	3.418198	35.05227
3.351394	3.347408	3.376728	35.21605
3.31477	3.451734	3.485534	35.60204
3.449388	3.508479	3.424217	35.88643
3.269938	3.319575	3.391734	35.00986
3.248574	3.215654	3.289663	34.51267
3.314728	3.210667	3.384282	34.85335
3.29588	3.381779	3.309153	35.02203
3.220157	3.364931	3.398623	35.01525
3.174583	3.389125	3.391614	34.95317
3.267546	3.323955	3.454688	35.15188
3.334938	3.408534	3.376114	35.31239
3.354693	3.475861	3.418931	35.59646
3.262844	3.364594	3.427133	35.17021
3.208362	3.340467	3.442616	35.03216
3.31279	3.294167	3.355994	34.96985
3.344424	3.278869	3.373199	35.0432
3.260493	3.252922	3.400959	34.86362
3.183038	3.223404	3.402868	34.63387
3.202835	3.226504	3.422785	34.72749
3.371356	3.372479	3.358108	35.2738
3.271877	3.390675	3.395639	35.17813
3.313615	3.360348	3.424404	35.26599
3.127401	3.382116	3.404556	34.86296
3.309573	3.408063	3.386686	35.27901
3.361334	3.430774	3.456086	35.59363

3.457596	3.373018	3.35006	35.44598
3.231747	3.22145	3.440774	34.819
3.29122	3.256764	3.369516	34.87046
3.242841	3.358596	3.388801	35.02952
3.297736	3.446073	3.340801	35.2359
3.237851	3.331167	3.386004	34.95251
3.150868	3.335008	3.410218	34.82365
3.119111	3.373692	3.478015	34.98705
3.33036	3.460495	3.408393	35.4866
3.34298	3.445668	3.417447	35.50157
3.34562	3.359674	3.417788	35.32003
3.249316	3.236411	3.428923	34.86423
3.287632	3.261414	3.360154	34.85231
3.383894	3.313442	3.367265	35.19214
3.317121	3.270243	3.356403	34.9279
3.133629	3.262964	3.380275	34.56292
3.190008	3.328066	3.412758	34.89962
3.329205	3.369244	3.416476	35.30219
3.259132	3.408265	3.379133	35.15263
3.245522	3.403952	3.42275	35.20882
3.16918	3.385149	3.475849	35.11687
3.225849	3.411972	3.441933	35.22528
3.332834	3.303333	3.366497	35.0567
3.371644	3.278465	3.383225	35.12377
3.201185	3.22583	3.427559	34.73285
3.204237	3.293628	3.41992	34.87108
3.311924	3.427068	3.362746	35.27336
3.379481	3.410152	3.340937	35.33641
3.29951	3.395797	3.399526	35.25826
3.212775	3.374029	3.475917	35.18803
3.21096	3.491766	3.414054	35.30625
3.332504	3.477613	3.450186	35.62012
3.271959	3.361292	3.373045	35.06464
3.301819	3.287158	3.374665	34.97137
3.235211	3.225291	3.407813	34.7629
3.364715	3.275904	3.362286	35.05722
3.303057	3.254675	3.425769	35.01479
3.283136	3.250968	3.34005	34.77567
3.185513	3.294976	3.428991	34.85292
3.197762	3.411095	3.410149	35.09244
3.188936	3.382521	3.399441	34.98723
3.282847	3.401054	3.341926	35.10735
3.208774	3.35745	3.467733	35.12513
3.305696	3.408332	3.395621	35.29066
3.305861	3.402402	3.41535	35.32119
3.302273	3.237354	3.363377	34.83876
3.35325	3.224954	3.351492	34.89713
3.305944	3.280554	3.338959	34.88786
3.214754	3.405165	3.393098	35.07934
3.303387	3.395797	3.307993	35.06657

3.286972	3.348015	3.365304	35.05151
3.233891	3.332649	3.309	34.7787
3.270021	3.422552	3.42258	35.30269
3.188235	3.506727	3.443502	35.35367
3.251997	3.400582	3.423995	35.21833
3.210671	3.283384	3.399936	34.81905
3.333659	3.221787	3.360086	34.86615
3.20036	3.262088	3.474962	34.914
3.319471	3.345791	3.327296	35.0346
3.256204	3.263571	3.388409	34.85008
3.321575	3.277925	3.278119	34.78325
3.268536	3.317418	3.414327	35.05149
3.302232	3.377062	3.297558	35.00025
3.180976	3.368435	3.39262	34.9241
3.187328	3.46656	3.364605	35.09131
3.230056	3.393304	3.387965	35.07564
3.360137	3.380431	3.362405	35.27606
3.278104	3.284126	3.418317	35.00833
3.29254	3.275634	3.40263	34.98702
3.253606	3.370727	3.420807	35.14958
3.351353	3.413522	3.425428	35.46703
3.330813	3.45598	3.332497	35.31174
3.300417	3.272467	3.340869	34.86226
3.138042	3.27186	3.377615	34.58621
3.224158	3.402873	3.420261	35.15429
3.244491	3.533685	3.479873	35.61518
3.275176	3.4301	3.400345	35.28185
3.166995	3.235804	3.420875	34.66528
3.241315	3.159313	3.458388	34.74256
3.396432	3.292954	3.404761	35.25676
3.371603	3.377399	3.356761	35.28216
3.29555	3.318429	3.399799	35.081
3.245275	3.273343	3.359677	34.78472
3.244862	3.315531	3.439018	35.04958
3.342527	3.3621	3.317048	35.09827
3.365788	3.394517	3.385732	35.37023
3.099479	3.456991	3.384453	34.92168
3.186544	3.422957	3.462873	35.20914
3.28957	3.355563	3.330843	34.99833
3.303469	3.285676	3.35833	34.93601
3.227911	3.20979	3.382952	34.65867
3.30648	3.288574	3.46867	35.19023
3.228489	3.437177	3.437193	35.27581
3.420229	3.467437	3.371272	35.61713
3.361169	3.385081	3.412554	35.39815
3.398081	3.293561	3.308062	35.05022
3.132309	3.359674	3.43559	34.89249
3.260328	3.522497	3.33369	35.30567
3.178172	3.511984	3.50557	35.47889
3.344382	3.301379	3.280046	34.88862

3.167407	3.121707	3.440109	34.45873
3.246141	3.231222	3.425001	34.83736
3.327308	3.414533	3.470512	35.51525
3.404845	3.374501	3.382782	35.40542
3.30351	3.267816	3.380429	34.94537
3.264659	3.24679	3.415538	34.8912
3.239253	3.395191	3.349582	35.01594
3.286642	3.427742	3.372977	35.24192
3.176563	3.424035	3.349446	34.94163
3.227004	3.385958	3.472098	35.23688
3.225725	3.323753	3.456052	35.06297
3.308295	3.255416	3.444952	35.06982
3.363602	3.275702	3.360563	35.05058
3.2322	3.239174	3.41165	34.79507
3.253235	3.389462	3.391188	35.12497
3.316956	3.411432	3.411241	35.35622
3.356384	3.410691	3.346649	35.29957
3.313945	3.33056	3.370181	35.08299
3.4206	3.401795	3.352856	35.43412
3.110739	3.43064	3.463828	35.06226
3.255668	3.468111	3.449317	35.4294
3.180605	3.29383	3.399083	34.77428
3.20931	3.215991	3.420056	34.71269
3.194009	3.191729	3.413048	34.61085
3.269649	3.28918	3.493173	35.16459
3.234593	3.418576	3.369839	35.10119
3.413795	3.297807	3.450476	35.40531
3.259297	3.213767	3.355533	34.67604
3.369294	3.293359	3.386345	35.15802
3.255833	3.377399	3.360785	35.03779
3.252451	3.374366	3.39274	35.09364
3.178543	3.457597	3.329274	34.97524
3.318234	3.398897	3.367538	35.23603
3.24812	3.333391	3.386635	34.98121
3.286559	3.282845	3.393166	34.96902
3.202835	3.248744	3.458439	34.8541
3.344135	3.382992	3.394121	35.31602
3.31015	3.351182	3.441405	35.27554
3.326318	3.401189	3.442292	35.42219
3.307428	3.3621	3.374665	35.14752
3.251915	3.350104	3.307789	34.85364
3.244945	3.367357	3.386209	35.04762
3.24511	3.527013	3.358517	35.33656
3.189843	3.410961	3.487581	35.24416
3.163901	3.277184	3.447867	34.80803
3.257111	3.226504	3.395349	34.78619
3.278929	3.245442	3.383072	34.84846
3.318358	3.414061	3.435539	35.41817
3.271423	3.36547	3.448618	35.23787
3.325204	3.236411	3.353606	34.86548

3.34595	3.286889	3.343768	34.99971
3.369417	3.339793	3.381759	35.24981
3.218631	3.410489	3.483693	35.29758
3.293117	3.486307	3.336777	35.30498
3.220116	3.365133	3.477009	35.18702
3.321327	3.268625	3.284394	34.77609
3.284868	3.210667	3.469625	34.97468
3.185018	3.347948	3.413372	34.93352
3.175779	3.411837	3.49065	35.22203
3.388018	3.468987	3.418897	35.65422
3.348136	3.353541	3.362678	35.19161
3.422786	3.349632	3.389295	35.40451
3.179203	3.298144	3.445361	34.88185
3.260906	3.38434	3.355448	35.05239
3.277321	3.457058	3.329444	35.19044
3.333742	3.421407	3.365406	35.31451
3.133835	3.22765	3.456001	34.65174
3.157715	3.250025	3.429946	34.69592
3.261854	3.353676	3.462498	35.22151
3.352714	3.483139	3.426519	35.62464
3.35391	3.410017	3.44623	35.51045
3.194586	3.208443	3.446793	34.72246
3.264906	3.187753	3.378229	34.68105
3.315925	3.322742	3.387505	35.10811
3.40864	3.415341	3.374392	35.48468
3.256823	3.480983	3.425973	35.40903
3.190957	3.357517	3.386823	34.90938
3.250925	3.201568	3.354425	34.62863
3.392349	3.275095	3.346274	35.08087
3.232077	3.356844	3.436085	35.10555
3.277238	3.400784	3.410644	35.24477
3.198133	3.412511	3.426331	35.13173
3.371686	3.356439	3.342642	35.20563
3.436025	3.348958	3.325983	35.29354
3.403196	3.402536	3.378041	35.45276
3.203371	3.412578	3.422495	35.13494
3.303428	3.421205	3.383361	35.28704
3.208815	3.327729	3.460076	35.04348
3.30252	3.250429	3.349667	34.83791
3.21294	3.18344	3.349702	34.49559
3.099438	3.315666	3.472132	34.80427
3.128185	3.429359	3.440041	35.04559
3.292457	3.356372	3.417771	35.19652
3.305737	3.247464	3.43056	35.01536
3.258225	3.299761	3.393388	34.94453
3.21063	3.403952	3.447595	35.18684
3.373913	3.444186	3.343495	35.40425
3.392307	3.364392	3.353282	35.29138
3.239129	3.345252	3.384112	34.98197
3.150002	3.321192	3.440433	34.85762

3.276826	3.268827	3.308539	34.73201
3.326153	3.26074	3.330774	34.87082
3.26833	3.306635	3.370607	34.93185
3.297612	3.37841	3.427474	35.2772
3.192236	3.368233	3.413594	34.99415
3.365128	3.366481	3.378041	35.29066
3.378573	3.3621	3.363718	35.27916
3.41693	3.419722	3.438591	35.65278
3.254348	3.385081	3.416851	35.17395
3.312749	3.391956	3.406023	35.29302
3.237108	3.327056	3.362831	34.89122
3.320131	3.200355	3.367947	34.80689
3.094819	3.185866	3.425291	34.40789
3.234593	3.384879	3.440638	35.18232
3.164932	3.411163	3.494418	35.20507
3.287054	3.435425	3.414054	35.34945
3.333824	3.278936	3.444321	35.1757
3.377955	3.269232	3.36058	35.06786
3.227169	3.341613	3.43866	35.06714
3.323307	3.417026	3.345353	35.23826
3.315223	3.437109	3.422273	35.43271
3.36253	3.356776	3.326597	35.15125
3.164314	3.193481	3.373864	34.46405
3.206794	3.266806	3.336504	34.6356
3.279424	3.358798	3.421881	35.18231
3.271299	3.455575	3.41651	35.36443
3.297406	3.449375	3.447816	35.47643
3.284085	3.37814	3.407234	35.20277
3.243377	3.315801	3.441746	35.05289
3.386739	3.399504	3.385391	35.42621
3.389874	3.390338	3.380497	35.40232
3.301201	3.413926	3.396389	35.29474
3.300541	3.343567	3.370266	35.0823
3.269732	3.19766	3.418556	34.80146
3.298314	3.250968	3.286867	34.69255
3.247254	3.374703	3.38609	35.06847
3.248244	3.460293	3.459207	35.4177
3.187245	3.395056	3.463282	35.15055
3.27563	3.293156	3.358313	34.89145
3.243666	3.21653	3.414873	34.77767
3.407402	3.328403	3.383992	35.31285
3.222384	3.427472	3.501137	35.38107
3.347641	3.464606	3.398486	35.51171
3.32339	3.317688	3.409894	35.16234
3.350734	3.121235	3.285537	34.52058
3.192153	3.172589	3.348508	34.4238
3.212898	3.305759	3.425581	34.92893
3.079559	3.441557	3.401027	34.88061
3.326235	3.487857	3.413219	35.54796
3.341083	3.487183	3.371016	35.48667

3.385708	3.373962	3.354988	35.30161
3.292663	3.382857	3.397395	35.21033
3.34595	3.430977	3.405852	35.45058
3.403031	3.428146	3.397003	35.54986
3.352672	3.367559	3.33473	35.17107
3.219374	3.172859	3.373949	34.53955
3.152394	3.161065	3.358125	34.33268
3.291178	3.353474	3.408103	35.16624
3.227746	3.447151	3.398554	35.2115
3.299716	3.39573	3.399049	35.25752
3.142414	3.34505	3.495629	35.0139
3.177883	3.270512	3.471876	34.87652
3.312584	3.335615	3.404505	35.16613
3.32174	3.435762	3.4264	35.45303
3.260081	3.43502	3.370658	35.19468
3.320503	3.34505	3.489814	35.39063
3.261277	3.24315	3.352106	34.73713
3.373253	3.145834	3.307567	34.67179
3.277486	3.26411	3.295648	34.69495
3.229478	3.413589	3.469489	35.29701
3.175408	3.438188	3.420176	35.12473
3.376387	3.449038	3.341619	35.41617
3.266639	3.382925	3.384572	35.12552
3.35424	3.427809	3.354783	35.3501
3.276207	3.447218	3.456342	35.444
3.410784	3.412646	3.395093	35.52875
3.30318	3.320788	3.366838	35.03077
3.320461	3.248205	3.369447	34.91554
3.241769	3.174207	3.392092	34.63115
3.211579	3.253596	3.338396	34.62131
3.123524	3.426192	3.449658	35.0495
3.252368	3.42417	3.343495	35.09468
3.20564	3.343095	3.466096	35.0833
3.247584	3.31634	3.349872	34.86236
3.22655	3.292348	3.490701	35.07186
3.299881	3.382386	3.396252	35.22258
3.389915	3.373288	3.363019	35.3269
3.323967	3.38589	3.370334	35.22624
3.343269	3.336558	3.377598	35.17645
3.315595	3.218552	3.396048	34.89822
3.262597	3.185596	3.352242	34.61446
3.246017	3.280756	3.3142	34.70311
3.328669	3.359202	3.422444	35.29211
3.107769	3.409882	3.472643	35.02965
3.320668	3.437244	3.357187	35.30258
3.299427	3.37167	3.418812	35.24749
3.284291	3.450453	3.407438	35.3618
3.257235	3.433403	3.393337	35.23451
3.328916	3.36392	3.404079	35.26281
3.253647	3.346195	3.428855	35.11363

3.342939	3.250833	3.32421	34.87151
3.257936	3.185259	3.435386	34.78535
3.286889	3.357517	3.333844	35.00331
3.144311	3.384138	3.450323	35.00445
3.218301	3.402334	3.377683	35.0472
3.280084	3.385418	3.434908	35.27045
3.256287	3.317351	3.343785	34.87029
3.218755	3.272736	3.431038	34.88146
3.270722	3.374905	3.426996	35.20969
3.296293	3.404423	3.407319	35.28713
3.435819	3.407928	3.408853	35.60327
3.375934	3.286687	3.334594	35.04478
3.318564	3.190651	3.398435	34.84892
3.214136	3.282171	3.406279	34.83784
3.162623	3.352463	3.421267	34.91169
3.311841	3.410017	3.38846	35.29212
3.316502	3.375849	3.366975	35.18061
3.268082	3.363718	3.414327	35.15174
3.302644	3.37194	3.382321	35.17531
3.336464	3.423833	3.350401	35.29295
3.218879	3.434144	3.43854	35.25111
3.391524	3.354283	3.322249	35.1997
3.255627	3.215047	3.479993	34.94299
3.313945	3.240792	3.330757	34.80047
3.340464	3.346532	3.389688	35.21857
3.271918	3.474917	3.395366	35.36184
3.197102	3.400717	3.440655	35.13501
3.249935	3.384745	3.450118	35.23631
3.201185	3.353743	3.344552	34.83105
3.323142	3.319238	3.386209	35.11339
3.194586	3.347543	3.412844	34.95241
3.23187	3.396741	3.47087	35.26842
3.303222	3.393101	3.427695	35.32208
3.333824	3.307646	3.388937	35.11737
3.351188	3.18344	3.364809	34.83096
3.287178	3.198131	3.364383	34.72217
3.221106	3.310544	3.44282	34.99504
3.232448	3.444186	3.385135	35.18595
3.361457	3.440681	3.32866	35.33691
3.334814	3.400784	3.372022	35.28622
3.31877	3.37322	3.359387	35.16323
3.312254	3.37996	3.445975	35.35307
3.345372	3.478691	3.405733	35.5534
3.366242	3.378544	3.311745	35.1745
3.321534	3.170972	3.370044	34.75029
3.188111	3.140981	3.414088	34.48925
3.266185	3.263031	3.445293	34.99513
3.241893	3.424979	3.330638	35.04543
3.434046	3.446545	3.423995	35.71695
3.130783	3.353541	3.402425	34.80321

3.22721	3.284395	3.427542	34.9178
3.221023	3.372681	3.447953	35.14197
3.302768	3.353204	3.374358	35.1172
3.182007	3.34168	3.45034	34.99407
3.276661	3.420463	3.435198	35.34024
3.230633	3.305624	3.432061	34.98159
3.467205	3.269771	3.321737	35.17927
3.369665	3.190448	3.351321	34.85719
3.357333	3.198064	3.393302	34.93869
3.22457	3.389125	3.366446	35.00745
3.284126	3.451532	3.440774	35.4367
3.261896	3.452408	3.370709	35.23678
3.424188	3.407591	3.295512	35.32924
3.177965	3.409545	3.464305	35.16419
3.252657	3.375849	3.376558	35.06195
3.32174	3.364998	3.528931	35.52251
3.36088	3.303468	3.297745	34.96798
3.292622	3.128244	3.412025	34.68543
3.257029	3.215452	3.382543	34.73383
3.242717	3.334267	3.406125	35.01394
3.257648	3.43037	3.372789	35.18385
3.243171	3.392225	3.382543	35.0901
3.259751	3.330425	3.490582	35.22748
3.14031	3.401323	3.40621	34.93682
3.177223	3.37969	3.439205	35.04238
3.319595	3.413859	3.402596	35.34839
3.23385	3.359539	3.409672	35.05757
3.31312	3.363448	3.447185	35.3215
3.268371	3.239646	3.416032	34.88478
3.334608	3.239376	3.318821	34.81645
3.305696	3.214104	3.341807	34.74823
3.346486	3.293696	3.386823	35.10993
3.255091	3.409613	3.45005	35.30182
3.349414	3.44176	3.301906	35.25442
3.297695	3.396201	3.453136	35.37241
3.338773	3.427203	3.304907	35.20588
3.222261	3.426731	3.453102	35.27414
3.240325	3.472424	3.4397	35.38425
3.172562	3.336625	3.463555	34.99127
3.395071	3.228998	3.415879	35.13823
3.305572	3.202647	3.345763	34.73155
3.409258	3.315531	3.417601	35.36226
3.282724	3.442231	3.420875	35.36978
3.297365	3.392427	3.379798	35.20305
3.345991	3.280284	3.333554	34.96302
3.320626	3.348756	3.349787	35.09279
3.208485	3.413252	3.406074	35.11169
3.185884	3.399773	3.450886	35.13079
3.243419	3.377803	3.457638	35.22333
3.254554	3.331099	3.447254	35.12283

3.349579	3.288102	3.349974	35.02388
3.222673	3.178452	3.446043	34.71665
3.250512	3.230548	3.379269	34.74543
3.239047	3.291202	3.393456	34.88403
3.37049	3.395325	3.391	35.3938
3.342238	3.491361	3.366583	35.48864
3.24379	3.423092	3.381707	35.15713
3.229973	3.402402	3.375518	35.06813
3.344795	3.424776	3.360239	35.33475
3.170047	3.437379	3.326921	34.9073
3.307305	3.358663	3.39262	35.179
3.1705	3.214238	3.471006	34.73541
3.27497	3.135455	3.364247	34.55812
3.344754	3.259931	3.405938	35.0741
3.379976	3.463932	3.391802	35.56633
3.296499	3.420935	3.414463	35.33931
3.237974	3.344443	3.33855	34.87804
3.214713	3.312296	3.393712	34.8775
3.39437	3.406311	3.371204	35.42675
3.21261	3.49372	3.378314	35.23598
3.089622	3.317081	3.439478	34.7145
3.179368	3.295987	3.390114	34.75667
3.293612	3.32254	3.446248	35.18733
3.322647	3.273343	3.400413	35.04301
3.285116	3.260942	3.395349	34.92274
3.190627	3.226639	3.447407	34.75493
3.285693	3.319575	3.28274	34.80596
3.425096	3.40166	3.44652	35.64848
3.326112	3.458204	3.336896	35.31594
3.266144	3.391012	3.418539	35.2164
3.259174	3.442838	3.314592	35.08718
3.345042	3.466156	3.369089	35.44513
3.24445	3.426663	3.343375	35.08256
3.313945	3.353609	3.387522	35.17131
3.146125	3.19274	3.481152	34.65728
3.265525	3.207095	3.382048	34.73306
3.25938	3.313914	3.392774	34.97667
3.470794	3.389934	3.433953	35.69529
3.256328	3.336288	3.427065	35.09391
3.282064	3.329684	3.384811	35.04335
3.259215	3.355159	3.394462	35.07019
3.333412	3.465752	3.383958	35.45133
3.18708	3.410893	3.425717	35.10268
3.190627	3.385014	3.416765	35.03427
3.063556	3.28945	3.474553	34.67377
3.341702	3.290865	3.372789	35.06259
3.375604	3.258651	3.463572	35.2648
3.252905	3.319103	3.361672	34.90584
3.195906	3.252788	3.458115	34.84708
3.318523	3.347206	3.336299	35.05531

3.414249	3.427607	3.331661	35.43033
3.4093	3.431988	3.354203	35.47838
3.148724	3.363179	3.329137	34.70325
3.236737	3.376927	3.378161	35.03299
3.24214	3.426461	3.344876	35.08035
3.308996	3.377399	3.371749	35.17802
3.329741	3.289517	3.331456	34.94309
3.176975	3.204466	3.396781	34.56588
3.199948	3.305624	3.398145	34.84032
3.301118	3.414465	3.389841	35.28142
3.399566	3.432055	3.455592	35.67896
3.241851	3.353339	3.374	34.9835
3.329782	3.316542	3.393797	35.13861
3.284621	3.341006	3.438677	35.19149
3.34331	3.489609	3.36926	35.49301
3.168933	3.344578	3.441985	34.95355
3.206052	3.299896	3.360086	34.75791
3.16951	3.228661	3.413236	34.63845
3.253936	3.229267	3.378075	34.74751
3.345785	3.237422	3.394581	35.0023
3.285404	3.351789	3.375842	35.07938
3.224818	3.355967	3.462617	35.14579
3.384017	3.440883	3.388068	35.5166
3.378903	3.434818	3.381741	35.47832
3.412888	3.375714	3.32068	35.28985
3.228282	3.383329	3.384316	35.04197
3.143486	3.421878	3.369481	34.90839
3.240738	3.33831	3.395417	34.99503
3.257276	3.29282	3.317252	34.76078
3.267464	3.219293	3.363223	34.7228
3.210053	3.178318	3.449982	34.69737
3.255008	3.419924	3.419766	35.25796
3.381667	3.414735	3.448072	35.5855
3.437221	3.394786	3.372653	35.49843
3.277774	3.348958	3.387045	35.081
3.32009	3.396538	3.360939	35.2205
3.282682	3.431381	3.488331	35.49348
3.277197	3.411904	3.372789	35.18622
3.219167	3.307646	3.357443	34.79776
3.246801	3.230143	3.411582	34.8071
3.136846	3.236613	3.465072	34.69777
3.281445	3.204129	3.384367	34.76646
3.265979	3.323618	3.455097	35.14861
3.34232	3.362976	3.421966	35.32918
3.257565	3.348689	3.421148	35.1108
3.416146	3.423563	3.367657	35.50435
3.331721	3.391686	3.389568	35.29793
3.398453	3.459349	3.290584	35.37537
3.19867	3.411769	3.346377	34.95643
3.300953	3.375781	3.380241	35.17547

3.155735	3.358731	3.315633	34.67932
3.213146	3.234659	3.391802	34.70012
3.219456	3.209993	3.352123	34.5732
3.331061	3.279408	3.424967	35.12837
3.293818	3.433538	3.424353	35.38264
3.361334	3.375242	3.392143	35.33236
3.313574	3.285339	3.433067	35.12081
3.377996	3.374298	3.345746	35.26527
3.292993	3.406176	3.436085	35.34665
3.213682	3.431448	3.342693	35.02425
3.250925	3.352665	3.466778	35.20476
3.157467	3.306972	3.338345	34.61959
3.255792	3.286619	3.43056	34.99176
3.293859	3.218619	3.404011	34.86825
3.170954	3.25636	3.458985	34.80223
3.196772	3.324832	3.478356	35.05078
3.393833	3.409006	3.402101	35.49904
3.326978	3.365739	3.395485	35.24376
3.358323	3.419115	3.42877	35.50182
3.326813	3.421272	3.35596	35.2784
3.325493	3.413185	3.376251	35.3022
3.249481	3.38306	3.324261	34.9564
3.327514	3.35408	3.376728	35.17841
3.134206	3.265188	3.388801	34.58769
3.274599	3.274017	3.350622	34.83052
3.166541	3.300772	3.433698	34.8344
3.318812	3.416689	3.385527	35.31554
3.323967	3.386227	3.501358	35.5135
3.332793	3.317553	3.390182	35.1395
3.295427	3.377803	3.436341	35.29048
3.362365	3.430437	3.367589	35.40162
3.260576	3.376118	3.430014	35.19675
3.310975	3.351587	3.295529	34.95922
3.08855	3.237354	3.398827	34.4489
3.174748	3.263638	3.389688	34.6749
3.175367	3.324764	3.388886	34.80817
3.254967	3.290393	3.426468	34.98926
3.207207	3.313442	3.425223	34.9325
3.262597	3.340467	3.397054	35.05113
3.392225	3.403952	3.394667	35.46822
3.457719	3.434346	3.341653	35.56198
3.34595	3.428955	3.366378	35.35983
3.220941	3.365066	3.374017	34.96345
3.296086	3.373085	3.363991	35.12339
3.217848	3.328943	3.387863	34.90797
3.331968	3.286552	3.315564	34.90673
3.195081	3.274556	3.395093	34.75506
3.151487	3.291539	3.42952	34.77215
3.219291	3.362033	3.443707	35.10561
3.338237	3.442636	3.383975	35.41137

3.285404	3.331301	3.411923	35.11348
3.31213	3.310881	3.351287	34.99467
3.279383	3.39701	3.448362	35.32369
3.372304	3.423226	3.381503	35.43802
3.34929	3.423226	3.370505	35.36364
3.276042	3.305557	3.394002	34.99751
3.12505	3.202782	3.403414	34.46315
3.197762	3.2354	3.380497	34.64337
3.141382	3.274354	3.414873	34.68044
3.275052	3.341006	3.358995	34.99632
3.228406	3.367896	3.440825	35.13206
3.286106	3.375444	3.449641	35.29403
3.294313	3.42909	3.40546	35.33267
3.363437	3.428281	3.392348	35.45339
3.324874	3.41487	3.39216	35.33932
3.371521	3.360213	3.328524	35.18265
3.205928	3.310274	3.34179	34.74032
3.229643	3.254809	3.315769	34.61399
3.33667	3.258112	3.310466	34.84366
3.21096	3.303939	3.352993	34.76197
3.20597	3.357248	3.416117	35.00568
3.260163	3.420598	3.420363	35.27202
3.356838	3.382992	3.444935	35.45492
3.394658	3.372681	3.343648	35.29358
3.342073	3.389462	3.436	35.41724
3.229107	3.428213	3.433016	35.24842
3.300541	3.391821	3.437108	35.334
3.246347	3.283991	3.388136	34.87259
3.371438	3.173667	3.359336	34.8419
3.129216	3.18654	3.403414	34.43674
3.16254	3.265727	3.419033	34.71694
3.234221	3.336019	3.399901	34.98558
3.278022	3.412848	3.397122	35.2433
3.226921	3.385149	3.390029	35.05546
3.388802	3.354754	3.379013	35.31891
3.231829	3.398965	3.453818	35.23591
3.395854	3.482196	3.32259	35.48964
3.42757	3.40476	3.406398	35.57293
3.333412	3.370255	3.309545	35.07976
3.243047	3.24679	3.373523	34.75206
3.197185	3.25272	3.268621	34.43533
3.217641	3.311353	3.407711	34.91246
3.29819	3.355496	3.301531	34.95294
3.196649	3.372614	3.483539	35.16634
3.200525	3.426663	3.406381	35.12428
3.291261	3.372479	3.446793	35.29259
3.398205	3.383194	3.353112	35.34503
3.302438	3.402604	3.410831	35.30427
3.276207	3.428618	3.42686	35.33885
3.234634	3.306366	3.431771	34.99133

3.26037	3.251844	3.367367	34.78753
3.331597	3.192538	3.315974	34.70121
3.214548	3.183911	3.420875	34.65579
3.23616	3.262897	3.388664	34.80534
3.213352	3.291674	3.468466	34.9929
3.341413	3.459686	3.357852	35.39847
3.311223	3.398762	3.374426	35.23547
3.372964	3.380499	3.362473	35.30441
3.207124	3.456317	3.434277	35.26457
3.327596	3.443984	3.375381	35.37225
3.325246	3.373827	3.352123	35.16283
3.412558	3.261077	3.329751	35.05828
3.174295	3.202377	3.372159	34.5016
3.21826	3.335547	3.425496	35.00561
3.256617	3.381779	3.385408	35.10293
3.300912	3.376522	3.385237	35.18792
3.180976	3.368435	3.42258	34.98962
3.238511	3.342623	3.45595	35.13197
3.190668	3.403884	3.430492	35.10564
3.389173	3.433403	3.408598	35.55641
3.320008	3.427405	3.433408	35.4463
3.360137	3.357113	3.436818	35.3878
3.221023	3.277858	3.397275	34.82378
3.24049	3.190988	3.394701	34.67075
3.279383	3.171915	3.368015	34.65574
3.282641	3.290056	3.334458	34.84784
3.179326	3.308994	3.423245	34.85748
3.208733	3.346937	3.422699	35.00357
3.314976	3.407052	3.443502	35.41286
3.312996	3.345656	3.356761	35.08458
3.317409	3.40685	3.41385	35.35289
3.253441	3.467706	3.414174	35.3468
3.267051	3.388856	3.404778	35.18358
3.290106	3.247194	3.28806	34.66896
3.282064	3.224954	3.333486	34.70208
3.248492	3.266199	3.342642	34.73888
3.165963	3.389732	3.406483	34.96816
3.212445	3.402739	3.419289	35.12626
3.292663	3.392427	3.358449	35.14609
3.240944	3.3559	3.398401	35.04047
3.31279	3.431785	3.484852	35.55259
3.232324	3.4177	3.440706	35.24928
3.291467	3.423496	3.401368	35.30527
3.32108	3.378746	3.408035	35.28675
3.432107	3.327258	3.379815	35.35524
3.277279	3.16275	3.436886	34.78171
3.302108	3.17387	3.295563	34.55127
3.238304	3.173533	3.434959	34.71584
3.293364	3.31634	3.36822	35.0026
3.146827	3.373894	3.415418	34.91122

3.178213	3.464067	3.370522	35.07887
3.228159	3.365605	3.441251	35.12744
3.312378	3.363044	3.367265	35.14422
3.323183	3.419857	3.441064	35.45348
3.38942	3.453082	3.415469	35.61501
3.26569	3.382049	3.367589	35.08439
3.24082	3.159313	3.298632	34.39212
3.362653	3.251642	3.316792	34.90017
3.275259	3.342421	3.3858	35.05848
3.224034	3.451329	3.395161	35.2051
3.245934	3.315127	3.372517	34.90562
3.257978	3.307781	3.382594	34.93793
3.285611	3.364863	3.345081	35.04115
3.31213	3.418037	3.453529	35.45259
3.152477	3.448971	3.52639	35.33043
3.259669	3.411432	3.423876	35.25857
3.255049	3.342152	3.454006	35.16285
3.416517	3.336693	3.339556	35.25374
3.288126	3.224617	3.43675	34.94042
3.227787	3.192201	3.396713	34.65003
3.217889	3.293156	3.414583	34.88823
3.343393	3.338782	3.277011	34.96162
3.173057	3.34296	3.382065	34.828
3.26767	3.39391	3.362149	35.10276
3.189225	3.325303	3.422631	34.91345
3.281445	3.412376	3.353675	35.15474
3.352755	3.476602	3.42906	35.61599
3.41429	3.359068	3.36486	35.35313
3.265319	3.232098	3.393575	34.81249
3.284208	3.23412	3.349514	34.76186
3.284291	3.316812	3.399509	35.05221
3.378243	3.439603	3.364178	35.44893
3.172727	3.41642	3.369413	34.96025
3.188029	3.271456	3.451431	34.85606
3.263174	3.33238	3.383344	35.00473
3.246883	3.449577	3.431362	35.3304
3.345207	3.468852	3.408871	35.53838
3.200237	3.375781	3.492219	35.20009
3.176934	3.2848	3.423262	34.79938
3.274351	3.288372	3.396969	34.96272
3.430251	3.283924	3.389125	35.27677
3.243666	3.207095	3.394615	34.71273
3.230262	3.19557	3.389551	34.64715
3.263257	3.294909	3.449249	35.06709
3.381873	3.400043	3.350776	35.34105
3.239047	3.432459	3.412247	35.23403
3.271134	3.380566	3.394547	35.15201
3.182502	3.334671	3.486268	35.0584
3.234304	3.399773	3.393439	35.11105
3.356178	3.415543	3.406875	35.44144

3.402701	3.38151	3.322538	35.28431
3.253853	3.241937	3.419238	34.86505
3.259132	3.186809	3.362013	34.6309
3.275052	3.291539	3.340647	34.84802
3.363189	3.36884	3.360563	35.25336
3.250183	3.394988	3.377751	35.101
3.143816	3.366076	3.39673	34.84667
3.263422	3.389664	3.388818	35.14251
3.282146	3.489542	3.410422	35.44912
3.286394	3.467639	3.495902	35.59744
3.247584	3.372816	3.431651	35.1647
3.317244	3.319305	3.463504	35.26967
3.247955	3.309398	3.420329	35.00207
3.429055	3.247464	3.350827	35.11067
3.248079	3.20514	3.396593	34.72244
3.207331	3.254675	3.397804	34.7443
3.271547	3.335951	3.452028	35.18105
3.337784	3.391484	3.349736	35.22364
3.316914	3.390675	3.3607	35.20021
3.245976	3.391484	3.345643	35.01392
3.11544	3.384542	3.522485	35.10001
3.308418	3.507671	3.411855	35.54935
3.286188	3.39209	3.401368	35.22505
3.395566	3.301581	3.370044	35.19781
3.23616	3.209925	3.390301	34.69308
3.350899	3.269636	3.422154	35.14423
3.285322	3.37322	3.376353	35.12718
3.365087	3.375512	3.363906	35.27941
3.164891	3.340534	3.361126	34.75904
3.286312	3.331234	3.376933	35.0388
3.119565	3.451801	3.437807	35.07093
3.246058	3.517645	3.472643	35.56772
3.295921	3.461641	3.439222	35.48121
3.307428	3.255753	3.399731	34.96977
3.243584	3.268423	3.457024	34.98315
3.28227	3.311825	3.435215	35.11497
3.290065	3.319575	3.392007	35.05447
3.348177	3.275567	3.287344	34.85644
3.194504	3.228189	3.38834	34.63763
3.261648	3.307107	3.381861	34.94288
3.310027	3.386969	3.380633	35.22063
3.286271	3.41743	3.399526	35.27661
3.201722	3.433133	3.389227	35.10354
3.249811	3.403952	3.450596	35.27909
3.21294	3.413791	3.469369	35.26103
3.23484	3.253327	3.340528	34.67625
3.377583	3.269299	3.34121	35.02483
3.198628	3.273141	3.364195	34.69215
3.337206	3.404289	3.413099	35.38894
3.290024	3.44277	3.453409	35.45807

3.402206	3.401997	3.336845	35.35932
3.302067	3.283384	3.327961	34.86152
3.298314	3.420126	3.407626	35.32656
3.107687	3.463393	3.432862	35.05949
3.285239	3.459552	3.398435	35.36408
3.182131	3.276645	3.416714	34.77859
3.301737	3.269906	3.403465	34.99644
3.187493	3.229065	3.454842	34.76964
3.309862	3.325371	3.411701	35.15351
3.310027	3.257438	3.436136	35.05875
3.264989	3.266536	3.392075	34.8838
3.260782	3.325505	3.422972	35.07112
3.340547	3.400447	3.322436	35.18958
3.158168	3.406108	3.454569	35.09208
3.21426	3.432594	3.335225	35.01168
3.208197	3.432594	3.446111	35.24091
3.304748	3.418104	3.421966	35.36757
3.3443	3.316677	3.383702	35.14858
3.339392	3.236209	3.419766	35.04074
3.282394	3.292078	3.427764	35.05576
3.318482	3.303198	3.339283	34.9655
3.333247	3.365066	3.347434	35.15091
3.321822	3.378746	3.377257	35.22106
3.309408	3.346869	3.40633	35.18778
3.218714	3.400312	3.354169	34.99225
3.353209	3.522228	3.399441	35.65198
3.152683	3.477613	3.462395	35.25357
3.220735	3.380297	3.393643	35.03923
3.178295	3.173196	3.460162	34.63899
3.255173	3.283519	3.420892	34.96249
3.195329	3.31479	3.497385	35.06728
3.325452	3.348689	3.404829	35.22357
3.223581	3.269569	3.462208	34.95325
3.352342	3.318901	3.307567	35.00453
3.290189	3.351048	3.35804	35.04929
3.425962	3.451464	3.391648	35.6393
3.17945	3.402941	3.372858	34.95301
3.177553	3.31014	3.323272	34.63748
3.148971	3.339456	3.368492	34.73798
3.326442	3.43994	3.480214	35.59014
3.22853	3.330223	3.346121	34.84285
3.326153	3.202782	3.400942	34.89752
3.265814	3.236815	3.387437	34.81046
3.315058	3.354687	3.392365	35.1867
3.357745	3.38461	3.379542	35.31744
3.300912	3.356709	3.403124	35.18371
3.286724	3.345656	3.35671	35.02701
3.209269	3.454699	3.351304	35.08427
3.275712	3.507738	3.405784	35.4647
3.253235	3.459417	3.424473	35.35074

3.150126	3.351856	3.426536	34.89455
3.183533	3.26074	3.44577	34.81041
3.254596	3.243689	3.369447	34.76162
3.25043	3.259797	3.384862	34.82145
3.28924	3.267412	3.37782	34.90757
3.192524	3.228189	3.437364	34.74051
3.382409	3.31452	3.319111	35.08595
3.322853	3.350778	3.351373	35.10555
3.393627	3.363852	3.395843	35.38616
3.185925	3.363246	3.423739	34.99163
3.242759	3.40321	3.359813	35.06352
3.174872	3.46656	3.399714	35.14085
3.393627	3.451329	3.365491	35.51109
3.230468	3.285271	3.449027	34.97382
3.275382	3.245307	3.385544	34.84582
3.297035	3.237961	3.421762	34.95631
3.381378	3.335547	3.340306	35.17603
3.347764	3.407793	3.392296	35.37421
3.266102	3.34943	3.403619	35.09275
3.21096	3.291674	3.390932	34.81812
3.199494	3.418644	3.349002	34.97901
3.255668	3.497899	3.446572	35.48854
3.273774	3.474513	3.417601	35.41364
3.182172	3.353002	3.424677	34.96307
3.104717	3.23075	3.470376	34.62628
3.29555	3.286754	3.386908	34.98354
3.250842	3.401593	3.418061	35.20504
3.270969	3.292011	3.403602	34.97779
3.299386	3.235333	3.394172	34.89537
3.280043	3.337299	3.418215	35.12863
3.323225	3.317149	3.332394	34.99132
3.412104	3.418441	3.464578	35.69626
3.119688	3.388182	3.414122	34.88028
3.310192	3.421744	3.346803	35.22306
3.186833	3.344039	3.372977	34.8406
3.359643	3.245374	3.361808	34.97832
3.197721	3.255753	3.385254	34.6982
3.256328	3.350441	3.472984	35.22528
3.246718	3.41642	3.398912	35.18657
3.407609	3.377938	3.363258	35.37628
3.331638	3.326247	3.387931	35.15107
3.382945	3.305692	3.322334	35.07486
3.182172	3.301513	3.433016	34.86871
3.201515	3.483342	3.408274	35.25453
3.239665	3.470941	3.359472	35.20412
3.266061	3.324697	3.342421	34.90474
3.175903	3.190853	3.391273	34.52172
3.181182	3.254472	3.403465	34.69905
3.222591	3.394382	3.441951	35.17973
3.347022	3.44769	3.350077	35.3675

3.287384	3.201838	3.430969	34.87634
3.267216	3.259325	3.387573	34.86305
3.296004	3.322136	3.392092	35.07325
3.291921	3.367087	3.332292	35.03185
3.371232	3.426866	3.383549	35.4481
3.135815	3.429561	3.434107	35.04974
3.152229	3.342084	3.39598	34.81096
3.188317	3.256562	3.390847	34.69163
3.357292	3.235939	3.345183	34.91619
3.238428	3.172252	3.398077	34.63266
3.283631	3.371603	3.435403	35.24908
3.256369	3.406445	3.436017	35.267
3.47067	3.410152	3.307738	35.46322
3.391565	3.37477	3.371766	35.35288
3.335928	3.37632	3.402596	35.30202
3.250183	3.394719	3.371374	35.08646
3.348961	3.487048	3.362269	35.48447
3.16551	3.374972	3.384947	34.8878
3.31976	3.244363	3.363565	34.89274
3.164479	3.202782	3.483335	34.72415
3.187286	3.270243	3.415555	34.77333
3.236036	3.415543	3.454347	35.28252
3.333865	3.388451	3.412434	35.34555
3.301159	3.235467	3.406705	34.92695
3.418662	3.257573	3.354919	35.119
3.243831	3.3621	3.437517	35.14589
3.310728	3.379757	3.378451	35.20162
3.336216	3.426529	3.410883	35.43057
3.261112	3.387103	3.378996	35.11038
3.246429	3.324158	3.336981	34.84874
3.261566	3.232839	3.482738	35.00089
3.191493	3.287024	3.34491	34.66474
3.303387	3.30859	3.393388	35.0626
3.292251	3.398358	3.442087	35.34106
3.226303	3.358124	3.367674	34.94612
3.359313	3.355698	3.372039	35.24124
3.344341	3.381307	3.287122	35.0788
3.377583	3.381577	3.392143	35.38175
3.210795	3.388451	3.410439	35.07205
3.210548	3.447286	3.44664	35.27934
3.207042	3.287967	3.339232	34.68838
3.295344	3.21781	3.303424	34.64976
3.13899	3.278262	3.440791	34.74044
3.272743	3.440547	3.452795	35.41407
3.201639	3.418374	3.500932	35.31536
3.34265	3.307242	3.371084	35.09674
3.420353	3.237759	3.428906	35.24117
3.34397	3.279476	3.375177	35.04786
3.266474	3.387912	3.404028	35.17861
3.257111	3.42909	3.42495	35.29394

3.247337	3.444118	3.363172	35.17033
3.374037	3.256629	3.34919	35.00682
3.232572	3.186135	3.37446	34.59856
3.129587	3.283115	3.404778	34.65173
3.171243	3.322271	3.453051	34.93402
3.28425	3.461843	3.396321	35.36231
3.369129	3.444186	3.451346	35.62964
3.311347	3.311488	3.414515	35.13255
3.245893	3.33393	3.409024	35.02648
3.357457	3.429966	3.350145	35.35171
3.384471	3.442299	3.417516	35.58509
3.258143	3.426933	3.364451	35.15918
3.18807	3.239444	3.382185	34.63471
3.213146	3.141049	3.358261	34.42206
3.291096	3.35253	3.3917	35.12812
3.2647	3.450251	3.385339	35.27019
3.311759	3.443849	3.403329	35.39844
3.120678	3.357383	3.434226	34.85906
3.280084	3.205679	3.436221	34.88027
3.372717	3.312768	3.360461	35.15135
3.463823	3.406311	3.420125	35.68562
3.231623	3.395797	3.434243	35.18572
3.28458	3.39054	3.470239	35.36875
3.235789	3.268356	3.410422	34.86404
3.336299	3.214912	3.296876	34.71866
3.206011	3.282508	3.373932	34.75007
3.169717	3.361831	3.496226	35.11161
3.086817	3.443175	3.463026	35.0356
3.341042	3.445736	3.44161	35.55032
3.362241	3.390203	3.377768	35.33563
3.43982	3.362505	3.372039	35.43218
3.217023	3.414398	3.454893	35.23963
3.310728	3.434077	3.371954	35.3062
3.339804	3.412443	3.437637	35.46612
3.419858	3.3034	3.248142	34.98833
3.082116	3.15446	3.44623	34.35722
3.123483	3.20386	3.348423	34.34182
3.281198	3.382453	3.394803	35.1787
3.298149	3.513332	3.395604	35.50373
3.284868	3.426731	3.472967	35.45449
3.169923	3.293089	3.451329	34.86355
3.203577	3.225021	3.435454	34.75358
3.404103	3.342421	3.39528	35.36098
3.365705	3.422283	3.383771	35.42648
3.244037	3.386969	3.433834	35.19267
3.285776	3.349902	3.393388	35.11443
3.233149	3.194896	3.377069	34.62469
3.380965	3.207297	3.384913	34.99221
3.239583	3.32254	3.420994	35.01395
3.259215	3.375714	3.386362	35.09743

3.159365	3.407928	3.428872	35.04248
3.352961	3.38434	3.37446	35.29528
3.357539	3.37969	3.430066	35.41672
3.358941	3.435559	3.349974	35.36682
3.22457	3.438996	3.472166	35.3477
3.342444	3.478624	3.405597	35.54655
3.310398	3.312835	3.415282	35.1351
3.307016	3.2199	3.33057	34.73922
3.200938	3.139701	3.370181	34.41848
3.085085	3.2817	3.434652	34.61665
3.138743	3.443175	3.355465	34.91394
3.300912	3.414398	3.396798	35.29603
3.267917	3.381375	3.393592	35.14465
3.292911	3.249418	3.449641	35.03331
3.259256	3.352935	3.464919	35.2195
3.341042	3.402739	3.389756	35.34289
3.391936	3.427674	3.439973	35.61854
3.289859	3.350778	3.386703	35.11066
3.265979	3.256629	3.404079	34.89055
3.259339	3.19119	3.343938	34.6014
3.313945	3.265592	3.352532	34.90232
3.216074	3.362707	3.382867	34.967
3.189225	3.447623	3.460554	35.26387
3.172356	3.392764	3.463555	35.11358
3.320668	3.371872	3.358585	35.16268
3.345455	3.318968	3.409877	35.21335
3.285281	3.417498	3.370385	35.21087
3.225313	3.435896	3.371084	35.12149
3.315471	3.391416	3.463282	35.42301
3.256163	3.274825	3.417857	34.939
3.372469	3.197794	3.377223	34.93603
3.234634	3.240589	3.361962	34.69482
3.295303	3.392562	3.458951	35.37194
3.19038	3.44641	3.408666	35.15028
3.300458	3.408939	3.407558	35.30663
3.212321	3.293224	3.411394	34.86923
3.305985	3.229132	3.42877	34.9719
3.284126	3.3435	3.421813	35.15899
3.420765	3.445129	3.432726	35.70391
3.289116	3.488935	3.449027	35.54746
3.400556	3.28271	3.273072	34.95539
3.33766	3.110857	3.408785	34.73881
3.136804	3.154865	3.323698	34.20974
3.119606	3.321799	3.478628	34.876
3.200732	3.46528	3.341346	35.06696
3.312213	3.415746	3.441473	35.42139
3.311965	3.403076	3.374358	35.24637
3.234593	3.307646	3.436971	35.00541
3.308171	3.419183	3.406824	35.3443
3.383976	3.484352	3.342011	35.51085

3.298149	3.429966	3.415487	35.3649
3.304294	3.208173	3.409672	34.8806
3.260906	3.115978	3.333724	34.41802
3.284208	3.247194	3.383361	34.86447
3.304624	3.423968	3.374853	35.27709
3.409423	3.451532	3.433169	35.69407
3.065824	3.326853	3.418948	34.63893
3.277073	3.259797	3.435198	34.98979
3.244945	3.288574	3.442138	34.99764
3.316007	3.40348	3.336623	35.17358
3.243089	3.434683	3.471347	35.37697
3.273691	3.410219	3.374597	35.17882
3.286271	3.293965	3.363565	34.92797
3.379563	3.180609	3.300082	34.74527
3.247543	3.141453	3.387454	34.562
3.265278	3.284732	3.324619	34.77671
3.183121	3.449442	3.378314	35.07466
3.262679	3.486779	3.401078	35.38007
3.240985	3.464876	3.33444	35.139
3.334195	3.355967	3.395826	35.23892
3.1884	3.347678	3.517438	35.1679
3.252657	3.47795	3.406193	35.35003
3.266886	3.447758	3.464919	35.44355
3.415321	3.274354	3.367793	35.17655
3.300376	3.117933	3.421199	34.6999
3.248904	3.20049	3.413014	34.74998
3.244161	3.395797	3.404693	35.14852
3.262473	3.494057	3.42038	35.43775
3.276702	3.375377	3.391478	35.14612
3.22589	3.305759	3.419784	34.94466
3.173635	3.299828	3.376745	34.7233
3.347971	3.314183	3.431907	35.25657
3.356096	3.394854	3.410627	35.40421
3.217188	3.39364	3.410763	35.09809
3.354982	3.338849	3.393183	35.24116
3.305614	3.307107	3.347894	34.96474
3.350322	3.175554	3.400362	34.88957
3.355559	3.239444	3.338891	34.90631
3.240655	3.346532	3.428855	35.08595
3.1592	3.396471	3.3334	34.80829
3.348796	3.414802	3.388647	35.38381
3.28792	3.406782	3.411121	35.28229
3.323101	3.402941	3.366924	35.25417
3.241068	3.435088	3.440092	35.30509
3.265608	3.463123	3.452727	35.44769
3.236036	3.33238	3.364127	34.90335
3.317038	3.200423	3.378382	34.8231
3.270433	3.165243	3.405648	34.70387
3.30252	3.286215	3.382168	34.98724
3.233933	3.379218	3.406705	35.09429

3.246182	3.437649	3.340511	35.1041
3.332752	3.291876	3.440979	35.19434
3.260163	3.371401	3.32675	34.95971
3.217188	3.349295	3.49834	35.19263
3.267092	3.382318	3.40014	35.15923
3.295592	3.409208	3.450749	35.39103
3.318688	3.37167	3.357153	35.15477
3.353621	3.300098	3.416493	35.20441
3.301778	3.265255	3.427951	35.0399
3.300623	3.226167	3.376473	34.83932
3.327514	3.280958	3.369038	35.00169
3.373789	3.372681	3.38077	35.32913
3.141259	3.404491	3.4493	35.04005
3.301819	3.375849	3.355107	35.12255
3.293859	3.385486	3.365969	35.14997
3.29588	3.474648	3.387351	35.39613
3.237315	3.428281	3.358671	35.10394
3.3045	3.373625	3.451056	35.33337
3.212362	3.328808	3.390165	34.90071
3.417713	3.249688	3.397531	35.19287
3.276743	3.255079	3.426365	34.95944
3.34929	3.351856	3.317235	35.09107
3.282229	3.370861	3.415009	35.19979
3.225931	3.360078	3.361194	34.93542
3.183946	3.3559	3.419766	34.96255
3.313244	3.319844	3.288009	34.87832
3.198216	3.312027	3.450647	34.96534
3.241191	3.372614	3.373898	35.02398
3.268453	3.38306	3.495885	35.37321
3.356673	3.439873	3.396167	35.4723
3.366118	3.293628	3.371903	35.12008
3.296251	3.169152	3.426468	34.81441
3.238717	3.201703	3.362763	34.62047
3.193432	3.330156	3.402357	34.88892
3.290395	3.453688	3.367452	35.29478
3.314234	3.469458	3.340971	35.32349
3.203206	3.420935	3.44311	35.19794
3.199659	3.365268	3.384384	34.94002
3.343186	3.402469	3.363445	35.28946
3.260865	3.365268	3.380701	35.06582
3.339763	3.361561	3.34196	35.14553
3.235995	3.206893	3.46439	34.8481
3.34929	3.245307	3.33045	34.88696
3.358488	3.316677	3.426536	35.27328
3.403154	3.45571	3.321294	35.44485
3.232943	3.377736	3.433016	35.14642
3.294024	3.350441	3.303816	34.93777
3.159942	3.28945	3.4338	34.79543
3.39606	3.340871	3.375501	35.29674
3.140516	3.370457	3.459121	34.98547

3.167489	3.359607	3.480214	35.06686
3.19702	3.369177	3.493105	35.18056
3.397298	3.271051	3.346394	35.08311
3.355724	3.148058	3.404437	34.85016
3.317409	3.204938	3.371135	34.81794
3.190091	3.272534	3.434107	34.82504
3.348424	3.430505	3.335805	35.30178
3.381378	3.508142	3.285281	35.43313
3.373212	3.414061	3.361314	35.37581
3.237273	3.419452	3.3474	35.05989
3.231252	3.408939	3.450255	35.24866
3.279548	3.490553	3.366361	35.34929
3.298644	3.354754	3.337936	35.03192
3.317574	3.15628	3.416919	34.81201
3.162623	3.114294	3.403363	34.35169
3.286518	3.236815	3.388255	34.85753
3.297654	3.458541	3.310159	35.19598
3.432396	3.395528	3.413423	35.57866
3.155611	3.371064	3.3858	34.85947
3.178213	3.298413	3.427951	34.8422
3.261153	3.375309	3.434789	35.20669
3.340753	3.41797	3.345712	35.27925
3.222838	3.353002	3.488604	35.1918
3.234716	3.339658	3.399322	34.99335
3.203	3.295111	3.427968	34.88922
3.396308	3.242207	3.413969	35.16565
3.369995	3.269164	3.369993	35.07089
3.343475	3.265188	3.437381	35.15156
3.223746	3.359472	3.326119	34.85261
3.440438	3.409074	3.386243	35.56643
3.297819	3.408669	3.249455	34.95453
3.469597	3.396067	3.260982	35.32783
3.161138	3.444118	3.433544	35.13572
3.195205	3.476265	3.384026	35.17223
3.275135	3.406243	3.436545	35.30875
3.287302	3.299694	3.332326	34.87444
3.324916	3.153584	3.444338	34.88213
3.257029	3.201434	3.428241	34.80311
3.259174	3.372479	3.488996	35.31471
3.336876	3.421811	3.400618	35.39925
3.364262	3.379892	3.376336	35.31436
3.254307	3.312701	3.468551	35.12863
3.150951	3.3652	3.347604	34.75293
3.30318	3.409074	3.37562	35.24304
3.315512	3.375983	3.397054	35.24452
3.28062	3.336895	3.381469	35.04865
3.261483	3.339726	3.482465	35.23385
3.162788	3.257842	3.390506	34.63786
3.358859	3.205882	3.400464	34.97478
3.298437	3.243757	3.396031	34.91578

3.354198	3.332043	3.368339	35.17023
3.216858	3.367761	3.426314	35.07478
3.344836	3.442029	3.328455	35.30306
3.333659	3.421137	3.405648	35.40174
3.306191	3.415746	3.295512	35.08903
3.135196	3.40995	3.406262	34.94461
3.259833	3.459147	3.334389	35.16758
3.171366	3.306635	3.430049	34.84979
3.273774	3.269906	3.380889	34.88592
3.345001	3.250833	3.389688	35.01921
3.334773	3.40813	3.398486	35.36007
3.251131	3.433875	3.41622	35.27224
3.328421	3.381712	3.382816	35.25414
3.307635	3.321731	3.426468	35.17297
3.380141	3.345589	3.288879	35.08282
3.20597	3.357787	3.437637	35.05392
3.17945	3.381307	3.408325	34.98326
3.26136	3.340265	3.421011	35.10037
3.248327	3.34505	3.396798	35.02938
3.304913	3.300165	3.375654	35.00874
3.266927	3.182294	3.464305	34.86177
3.251791	3.273814	3.409672	34.90933
3.297612	3.343095	3.420295	35.18428
3.3622	3.401189	3.385305	35.37604
3.298272	3.42545	3.372721	35.26178
3.360715	3.406715	3.362217	35.33439
3.299386	3.33211	3.406057	35.13299
3.379109	3.437581	3.339897	35.3933
3.223374	3.428955	3.341363	35.03708
3.323472	3.411769	3.408717	35.36569
3.130742	3.261818	3.425018	34.65195
3.232077	3.199075	3.341397	34.55347
3.282146	3.318631	3.476173	35.21915
3.349455	3.503492	3.381025	35.56253
3.235335	3.406445	3.474485	35.30513
3.327968	3.369918	3.408734	35.28403
3.31114	3.335278	3.343665	35.02919
3.355147	3.34943	3.333792	35.13478
3.20865	3.401256	3.317969	34.89315
3.123936	3.331436	3.443315	34.82932
3.101211	3.275634	3.329393	34.40846
3.275052	3.270647	3.404659	34.94231
3.290684	3.268019	3.465823	35.1045
3.279135	3.249014	3.414617	34.92571
3.183121	3.265458	3.452523	34.8346
3.278929	3.33521	3.449982	35.1911
3.439943	3.47148	3.396645	35.72457
3.350569	3.475861	3.315718	35.36173
3.328215	3.400986	3.392279	35.31653
3.245027	3.393708	3.380224	35.09233

3.284126	3.45234	3.352583	35.24561
3.243212	3.383127	3.329359	34.95399
3.302685	3.294639	3.363155	34.96445
3.146043	3.159852	3.449607	34.51619
3.185595	3.172454	3.397804	34.51696
3.343929	3.420935	3.39216	35.39426
3.326565	3.439468	3.441541	35.50481
3.265525	3.373692	3.379047	35.09081
3.314357	3.283789	3.463981	35.18673
3.268288	3.371401	3.411326	35.16243
3.406908	3.455238	3.395229	35.61371
3.183657	3.418239	3.439887	35.14225
3.268907	3.354148	3.403482	35.1089
3.098531	3.253394	3.386959	34.47985
3.246801	3.309398	3.331439	34.80516
3.256946	3.259257	3.396031	34.85894
3.319554	3.311285	3.366463	35.04497
3.18906	3.328134	3.489354	35.06519
3.36884	3.353676	3.371681	35.25687
3.396638	3.398291	3.360376	35.3905
3.467205	3.439333	3.34358	35.59784
3.2607	3.39263	3.367248	35.09587
3.216899	3.417161	3.37811	35.07749
3.280661	3.387508	3.408188	35.21785
3.239088	3.361898	3.291982	34.81681
3.253977	3.255281	3.401965	34.85673
3.178955	3.239781	3.422239	34.70311
3.147775	3.294639	3.480078	34.88138
3.259174	3.418778	3.433766	35.29518
3.369376	3.386227	3.384436	35.35711
3.264412	3.326988	3.403687	35.04013
3.326359	3.379016	3.384981	35.24847
3.306356	3.383397	3.445821	35.34735
3.397463	3.44486	3.329461	35.42654
3.241645	3.302996	3.398452	34.92643
3.262803	3.281497	3.375944	34.87646
3.197762	3.26256	3.397889	34.7408
3.308171	3.2416	3.332838	34.79416
3.235046	3.226302	3.401692	34.75136
3.289281	3.405097	3.41165	35.28274
3.148435	3.304074	3.436255	34.80762
3.407361	3.372411	3.409723	35.46527
3.362447	3.396201	3.354152	35.29755
3.458297	3.418307	3.343256	35.53167
3.311965	3.386362	3.374239	35.20956
3.213352	3.401121	3.318634	34.90459
3.164231	3.343634	3.436153	34.92845
3.19537	3.350845	3.293431	34.70021
3.222302	3.204871	3.368612	34.60429
3.219003	3.234457	3.438881	34.81544

3.229561	3.387305	3.462123	35.22361
3.33964	3.410691	3.41344	35.40901
3.406495	3.368166	3.352464	35.32887
3.317079	3.379623	3.40251	35.26783
3.295468	3.382992	3.399151	35.2206
3.283383	3.363313	3.437295	35.23455
3.277279	3.441153	3.378314	35.26244
3.229437	3.315666	3.361552	34.84674
3.285404	3.252922	3.391989	34.89849
3.105253	3.252181	3.414515	34.55216
3.300417	3.237557	3.379457	34.87031
3.209558	3.269569	3.38742	34.75903
3.272454	3.342556	3.417669	35.12234
3.204237	3.392158	3.451892	35.15647
3.318812	3.423968	3.404556	35.37307
3.328999	3.394247	3.403073	35.32711
3.397215	3.445331	3.320509	35.40745
3.287797	3.37504	3.439018	35.27361
3.350115	3.361628	3.274948	35.02177
3.154745	3.262897	3.381315	34.61122
3.190297	3.304681	3.35954	34.73273
3.142125	3.249822	3.379798	34.55171
3.258555	3.300974	3.377018	34.91211
3.248162	3.38279	3.434772	35.1946
3.292704	3.40476	3.4412	35.35411
3.315512	3.38461	3.409689	35.29101
3.431076	3.40375	3.387505	35.53707
3.318275	3.401526	3.389705	35.29034
3.263298	3.353878	3.42686	35.14717
3.2322	3.348015	3.430952	35.07529
3.171284	3.287226	3.400413	34.74236
3.309408	3.240118	3.393456	34.92618
3.201845	3.291606	3.418709	34.85878
3.179656	3.21963	3.423791	34.66397
3.206423	3.291943	3.434635	34.90436
3.344589	3.376186	3.395639	35.30545
3.290065	3.362168	3.422512	35.21433
3.34331	3.43839	3.401163	35.45077
3.307016	3.41278	3.390029	35.29104
3.388101	3.468717	3.326136	35.45096
3.353291	3.351856	3.334594	35.13778
3.320668	3.263503	3.33207	34.8677
3.198422	3.262964	3.371204	34.68477
3.208485	3.266536	3.306476	34.57304
3.170005	3.285002	3.390097	34.71214
3.29819	3.440007	3.373437	35.295
3.231582	3.399234	3.456802	35.24248
3.300747	3.378679	3.444935	35.32283
3.24247	3.379555	3.445361	35.19823
3.33102	3.432324	3.437705	35.49053

3.310769	3.403817	3.450408	35.41169
3.327266	3.358057	3.421744	35.28501
3.158663	3.285608	3.438387	34.79427
3.197061	3.207903	3.339556	34.49218
3.316914	3.214643	3.343529	34.77771
3.218549	3.263705	3.374154	34.73686
3.201474	3.316205	3.453972	34.98888
3.255585	3.381981	3.40558	35.14523
3.371026	3.394921	3.374273	35.35751
3.441181	3.347071	3.359182	35.37329
3.364427	3.388653	3.469148	35.53685
3.254678	3.428483	3.397463	35.22718
3.307552	3.414465	3.373932	35.2607
3.303716	3.381105	3.378297	35.1889
3.391111	3.19557	3.356198	34.92596
3.150167	3.315127	3.36411	34.67781
3.234469	3.296257	3.413355	34.92859
3.234386	3.364729	3.451636	35.16186
3.263999	3.452745	3.385408	35.27426
3.207784	3.388384	3.391597	35.02412
3.360179	3.352463	3.443946	35.3933
3.221601	3.403143	3.466556	35.25053
3.401958	3.445399	3.357085	35.49795
3.344919	3.349902	3.417925	35.29743
3.337289	3.357989	3.364195	35.18093
3.18675	3.262358	3.42067	34.7661
3.167902	3.165513	3.335054	34.32587
3.236985	3.24834	3.394138	34.78728
3.250265	3.321462	3.331917	34.84016
3.172562	3.327595	3.519501	35.09386
3.263958	3.472761	3.353896	35.24903
3.32108	3.35772	3.440382	35.3115
3.475083	3.40193	3.307857	35.45515
3.331432	3.41615	3.421557	35.42075
3.284497	3.433807	3.344757	35.18878
3.284126	3.373827	3.385135	35.1451
3.308418	3.247935	3.312666	34.76444
3.30516	3.219563	3.328217	34.72927
3.243501	3.28298	3.412622	34.9177
3.159736	3.346937	3.414941	34.87945
3.228324	3.319103	3.494947	35.14354
3.31778	3.369446	3.388255	35.21594
3.248574	3.34815	3.432743	35.11531
3.341166	3.378005	3.400959	35.31358
3.23781	3.461708	3.482192	35.44824
3.335804	3.448499	3.385578	35.42237
3.347764	3.399975	3.385203	35.3416
3.368964	3.304816	3.393115	35.19716
3.157591	3.144756	3.401982	34.40429
3.241686	3.18472	3.367367	34.59989

3.153425	3.279004	3.450459	34.79477
3.244408	3.33393	3.335259	34.86192
3.19405	3.381981	3.429264	35.06245
3.2176	3.382992	3.437244	35.13362
3.204939	3.382588	3.443246	35.11817
3.38509	3.44277	3.400277	35.54977
3.341908	3.456991	3.408444	35.5043
3.384059	3.407254	3.362081	35.38632
3.235541	3.281834	3.393592	34.85617
3.267711	3.186	3.348849	34.6191
3.331515	3.240792	3.303287	34.77881
3.305366	3.39108	3.381452	35.22122
3.173923	3.373759	3.391324	34.91749
3.208733	3.351519	3.409331	34.98436
3.283961	3.350172	3.423961	35.17791
3.274021	3.44122	3.399322	35.3014
3.319884	3.432931	3.449249	35.49275
3.211166	3.386901	3.481237	35.2243
3.244367	3.35718	3.404642	35.06441
3.275052	3.321462	3.373454	34.9852
3.321822	3.263773	3.412997	35.04779
3.228365	3.225358	3.38771	34.70411
3.127483	3.212419	3.444815	34.58008
3.182626	3.316609	3.413014	34.85898
3.326895	3.401997	3.364741	35.25563
3.261896	3.40321	3.423876	35.24546
3.333783	3.385216	3.427866	35.37204
3.205103	3.349902	3.487495	35.14381
3.332917	3.485565	3.393729	35.51494
3.391689	3.405839	3.356147	35.38693
3.466587	3.310544	3.383225	35.40154
3.200484	3.191122	3.379593	34.55052
3.299633	3.221989	3.340255	34.74882
3.258514	3.339186	3.362405	34.96363
3.365705	3.400986	3.371374	35.3528
3.154993	3.366279	3.38418	34.84411
3.131979	3.350509	3.459872	34.92482
3.218219	3.372277	3.427082	35.08931
3.361581	3.40321	3.396372	35.40331
3.30747	3.402873	3.467033	35.43877
3.26569	3.40321	3.382475	35.16322
3.194586	3.303737	3.526646	35.10548
3.242099	3.334334	3.282212	34.74175
3.391153	3.190651	3.391188	34.99181
3.231788	3.18499	3.363155	34.56962
3.17479	3.248744	3.403141	34.67184
3.202711	3.338041	3.382816	34.88373
3.319513	3.422418	3.350946	35.25398
3.31279	3.416824	3.399168	35.3325
3.284745	3.375983	3.454893	35.30372

3.160066	3.393371	3.458593	35.07718
3.341825	3.473704	3.390046	35.50043
3.306975	3.40476	3.361399	35.2108
3.443573	3.277791	3.391972	35.29872
3.150126	3.182698	3.39424	34.45401
3.289446	3.269906	3.405034	34.97299
3.2647	3.385418	3.378075	35.11253
3.333288	3.375579	3.322027	35.11843
3.23286	3.30495	3.36515	34.83866
3.191864	3.289989	3.331627	34.64298
3.191988	3.418509	3.489746	35.27009
3.325988	3.497831	3.342966	35.41561
3.294684	3.393438	3.457979	35.37037
3.336299	3.332986	3.412878	35.23055
3.237356	3.322675	3.426792	35.02206
3.309862	3.219226	3.400191	34.89622
3.289446	3.190112	3.354578	34.68815
3.350981	3.237152	3.316468	34.84225
3.092426	3.329953	3.432521	34.73356
3.232778	3.360685	3.378297	34.98912
3.246058	3.368166	3.390847	35.06196
3.265113	3.409276	3.337049	35.07589
3.292003	3.388653	3.418419	35.26754
3.264205	3.47303	3.426519	35.40898
3.274516	3.417498	3.392569	35.23584
3.319554	3.293898	3.357238	34.98678
3.394411	3.251912	3.384248	35.11772
3.243666	3.306029	3.39748	34.93535
3.326483	3.385688	3.384112	35.26143
3.29221	3.392225	3.369243	35.16826
3.363189	3.354283	3.328217	35.15079
3.278805	3.315396	3.354186	34.93801
3.223663	3.402739	3.377564	35.05954
3.173923	3.489677	3.438881	35.27498
3.251667	3.373827	3.405051	35.11767
3.312378	3.314251	3.404693	35.11937
3.34529	3.318833	3.429861	35.2564
3.226055	3.305018	3.476736	35.06795
3.348012	3.236478	3.384879	34.98389
3.235087	3.181957	3.397071	34.64437
3.277238	3.307646	3.386345	34.98796
3.209929	3.357787	3.395161	34.96969
3.212239	3.406715	3.358944	35.00254
3.167201	3.423698	3.421813	35.07867
3.265401	3.378342	3.434772	35.22257
3.263298	3.408198	3.418112	35.24683
3.287797	3.438255	3.428701	35.38929
3.256452	3.322203	3.348917	34.89248
3.292251	3.199007	3.392757	34.79723
3.369458	3.191459	3.336163	34.8258

3.33469	3.35435	3.347536	35.13086
3.24779	3.412848	3.323613	35.01643
3.28891	3.410826	3.356863	35.17465
3.297984	3.333593	3.417038	35.15718
3.220364	3.4239	3.355516	35.05039
3.34133	3.49062	3.428036	35.61942
3.162169	3.42235	3.496993	35.22913
3.219085	3.353204	3.413236	35.01922
3.191122	3.255079	3.428088	34.77596
3.31312	3.266536	3.419221	35.04842
3.234386	3.283182	3.396593	34.86316
3.392679	3.205545	3.393729	35.03327
3.228406	3.228189	3.453597	34.85448
3.336134	3.327056	3.359881	35.10132
3.19071	3.393101	3.398759	35.01276
3.364468	3.39883	3.370743	35.344
3.107852	3.367155	3.371749	34.71575
3.224653	3.38899	3.424012	35.13322
3.26899	3.447421	3.387045	35.27711
3.421673	3.392967	3.394837	35.50896
3.242553	3.260807	3.398623	34.83652
3.263752	3.252855	3.374085	34.81183
3.279589	3.268221	3.395962	34.92791
3.33436	3.420328	3.341739	35.26174
3.262226	3.392967	3.381997	35.1322
3.229643	3.35563	3.368629	34.95006
3.278476	3.258584	3.40176	34.91708
3.209558	3.401795	3.380616	35.03331
3.219415	3.510771	3.407285	35.3515
3.293736	3.385418	3.359677	35.13579
3.160602	3.287563	3.479532	34.89276
3.167861	3.240589	3.425496	34.68774
3.398659	3.332851	3.408615	35.3573
3.255668	3.316475	3.408598	35.00875
3.333618	3.229132	3.36822	34.89991
3.220652	3.270377	3.415811	34.84715
3.357127	3.355428	3.41992	35.34057
3.301283	3.346667	3.332053	35.00714
3.341825	3.401997	3.401709	35.36913
3.056009	3.378073	3.412264	34.71485
3.270845	3.437918	3.437227	35.37013
3.226096	3.405771	3.452727	35.23587
3.372593	3.297604	3.357153	35.11068
3.212404	3.218686	3.408171	34.69936
3.291385	3.280352	3.434022	35.06346
3.305861	3.405906	3.433391	35.36831
3.39606	3.444792	3.353078	35.47497
3.322317	3.324158	3.321976	34.98188
3.342032	3.335615	3.343938	35.09807
3.111233	3.399099	3.418692	34.89566

3.264824	3.501133	3.351015	35.30667
3.297241	3.493855	3.396866	35.46192
3.262349	3.329549	3.371255	34.97029
3.246223	3.215856	3.436426	34.82892
3.265896	3.232637	3.375756	34.77596
3.343351	3.360415	3.408734	35.29689
3.336464	3.303063	3.390711	35.117
3.280538	3.214643	3.398554	34.81849
3.210754	3.277117	3.408888	34.8251
3.33898	3.367963	3.41535	35.31831
3.270474	3.366009	3.362064	35.0477
3.361622	3.394854	3.380752	35.35097
3.115688	3.362302	3.481919	34.9632
3.191328	3.370255	3.375177	34.91258
3.261112	3.371872	3.403124	35.12984
3.326112	3.258651	3.361382	34.9331
3.16287	3.295852	3.383157	34.70509
3.293447	3.316744	3.388613	35.04826
3.26635	3.380836	3.40708	35.16954
3.40897	3.398088	3.313365	35.31422
3.317822	3.308859	3.354016	35.00866
3.381089	3.323281	3.337373	35.14216
3.249151	3.403143	3.427406	35.22516
3.26602	3.485565	3.375091	35.32789
3.134289	3.426866	3.437364	35.04763
3.317987	3.327056	3.325199	34.98579
3.219951	3.24342	3.415026	34.78495
3.139073	3.25117	3.464646	34.73354
3.313945	3.34943	3.434857	35.26569
3.308748	3.31452	3.454824	35.22165
3.316048	3.298144	3.339624	34.94988
3.268082	3.29902	3.420602	35.02398
3.237356	3.366616	3.371817	34.99793
3.237026	3.35745	3.386908	35.01016
3.311965	3.374635	3.369447	35.17344
3.26004	3.441018	3.410081	35.29392
3.263009	3.382116	3.452505	35.26438
3.193927	3.285811	3.348184	34.67456
3.403237	3.250429	3.344893	35.04772
3.25307	3.310611	3.417379	35.00945
3.290354	3.412915	3.413372	35.30595
3.31147	3.404558	3.406466	35.31875
3.39639	3.387103	3.35983	35.3643
3.312543	3.322203	3.359626	35.03856
3.390575	3.365066	3.330894	35.24011
3.160973	3.475524	3.429605	35.19543
3.259504	3.433133	3.432095	35.32364
3.157179	3.30859	3.396389	34.74943
3.262556	3.212351	3.41315	34.80607
3.213105	3.213093	3.419784	34.71406

3.296293	3.396606	3.523781	35.52472
3.167902	3.404289	3.48683	35.17994
3.38179	3.266064	3.423791	35.20755
3.352136	3.318564	3.425581	35.26142
3.294313	3.367559	3.386021	35.15561
3.263793	3.39364	3.427559	35.23674
3.214383	3.421407	3.378263	35.0816
3.2972	3.436368	3.380087	35.29942
3.345455	3.385958	3.323613	35.1712
3.207083	3.284126	3.444457	34.91018
3.258266	3.237018	3.330843	34.67064
3.249069	3.370255	3.474877	35.25687
3.354116	3.477613	3.406347	35.57151
3.368427	3.342354	3.384981	35.26029
3.34727	3.354148	3.347246	35.15729
3.326854	3.302996	3.436409	35.19577
3.265401	3.324764	3.362763	34.94793
3.324751	3.473569	3.373403	35.4264
3.260081	3.520341	3.433135	35.51789
3.24944	3.339186	3.378041	34.97798
3.131608	3.171039	3.408871	34.42001
3.306439	3.218417	3.375449	34.83286
3.21859	3.312498	3.456342	35.02339
3.286807	3.41487	3.407506	35.28964
3.219869	3.291	3.477759	35.026
3.293571	3.287698	3.370999	34.94649
3.399113	3.24342	3.377069	35.09374
3.446625	3.37787	3.319827	35.36648
3.203495	3.374231	3.445702	35.10211
3.218879	3.44951	3.417669	35.23907
3.194669	3.370861	3.436204	35.05467
3.251214	3.320721	3.324619	34.82465
3.254967	3.248205	3.42611	34.89622
3.154663	3.263301	3.434311	34.72782
3.153714	3.432122	3.456888	35.1443
3.421466	3.399504	3.378706	35.48753
3.368221	3.37814	3.384828	35.33776
3.360014	3.358798	3.396866	35.30384
3.336175	3.34296	3.402391	35.22916
3.249564	3.436975	3.388886	35.21581
3.357539	3.491024	3.347638	35.47993
3.359808	3.347948	3.372312	35.22597
3.131072	3.218686	3.413577	34.53332
3.097541	3.209993	3.453256	34.52776
3.289364	3.28325	3.41622	35.02645
3.26103	3.445399	3.42686	35.34235
3.330236	3.369851	3.464101	35.40992
3.210795	3.228998	3.493719	34.90547
3.273403	3.248542	3.39569	34.87075
3.360137	3.389664	3.310295	35.1823

3.353456	3.388114	3.399987	35.36043
3.221931	3.409882	3.422171	35.16893
3.215744	3.322405	3.435744	34.99378
3.256122	3.259392	3.348917	34.7544
3.4093	3.293224	3.381179	35.23392
3.172026	3.298413	3.347212	34.65211
3.275506	3.375579	3.404028	35.1714
3.184234	3.432864	3.46185	35.22352
3.35061	3.376859	3.403261	35.33676
3.396968	3.369042	3.400481	35.41496
3.450955	3.343297	3.378638	35.42896
3.22721	3.338849	3.468466	35.12637
3.344877	3.457799	3.347877	35.38011
3.259833	3.432729	3.39864	35.25032
3.363148	3.240926	3.300269	34.84169
3.14695	3.178722	3.379593	34.40633
3.082776	3.309331	3.446793	34.69857
3.174996	3.415813	3.483812	35.21406
3.294684	3.433605	3.432538	35.40258
3.29155	3.271725	3.476514	35.13788
3.308459	3.245981	3.388204	34.92544
3.252946	3.349093	3.476804	35.22329
3.348961	3.390271	3.358944	35.26557
3.317079	3.42572	3.396389	35.35525
3.295344	3.441827	3.370112	35.28548
3.263545	3.247868	3.352737	34.75379
3.27662	3.311892	3.389483	35.00275
3.265443	3.351317	3.360717	35.00162
3.243955	3.363785	3.36353	34.98805
3.246883	3.422889	3.42495	35.25802
3.222384	3.328808	3.444201	35.0408
3.370861	3.370053	3.347314	35.24381
3.350404	3.397954	3.32189	35.20449
3.441428	3.433066	3.401965	35.65544
3.24511	3.425787	3.440365	35.29418
3.270392	3.401054	3.450647	35.31787
3.275795	3.276173	3.393626	34.9319
3.314728	3.182294	3.296347	34.599
3.120225	3.288708	3.445156	34.73179
3.226633	3.413656	3.426724	35.19742
3.178295	3.43994	3.44132	35.18111
3.314151	3.359135	3.376609	35.15998
3.316048	3.264986	3.444866	35.10751
3.401546	3.3559	3.409825	35.41667
3.335061	3.389597	3.442888	35.41727
3.355642	3.395325	3.3718	35.31935
3.26701	3.386497	3.386294	35.13791
3.362241	3.301244	3.39794	35.1852
3.186132	3.253125	3.351219	34.59268
3.1778	3.269434	3.440791	34.806

3.263999	3.321597	3.434175	35.09411
3.197762	3.378881	3.368203	34.93026
3.295262	3.41615	3.381929	35.25499
3.227705	3.413589	3.411326	35.16594
3.269361	3.363785	3.426945	35.18228
3.2899	3.376994	3.396252	35.18896
3.460483	3.444388	3.398759	35.71486
3.279342	3.44122	3.398895	35.31211
3.296334	3.367829	3.385885	35.16032
3.257895	3.196918	3.46098	34.86673
3.314357	3.25144	3.304174	34.76652
3.251214	3.315733	3.40974	34.99989
3.213517	3.4239	3.394138	35.11988
3.213105	3.393438	3.461526	35.19973
3.219539	3.29437	3.38921	34.83901
3.317987	3.270984	3.407489	35.04313
3.396597	3.347813	3.378024	35.31862
3.289611	3.350509	3.430543	35.2054
3.303222	3.423429	3.415418	35.36155
3.274681	3.414196	3.446111	35.34607
3.357457	3.281969	3.394803	35.12573
3.276578	3.189977	3.339846	34.6275
3.186791	3.286282	3.350605	34.66529
3.128679	3.318025	3.423995	34.76811
3.241975	3.442905	3.355192	35.1385
3.361787	3.448634	3.41726	35.54877
3.3118	3.407861	3.418965	35.35402
3.277156	3.369514	3.39008	35.13124
3.258926	3.381644	3.402868	35.14587
3.416682	3.397212	3.373676	35.46106
3.32009	3.425922	3.404028	35.37899
3.219951	3.264851	3.33589	34.65875
3.234922	3.153584	3.372909	34.52913
3.291096	3.302389	3.39216	35.01948
3.283054	3.431516	3.362183	35.21872
3.313285	3.420059	3.361791	35.25892
3.142702	3.396875	3.41431	34.95003
3.249688	3.216193	3.401487	34.76083
3.274269	3.284193	3.423518	35.01146
3.382615	3.403682	3.399049	35.45619
3.264082	3.460023	3.412963	35.35062
3.429385	3.388249	3.406944	35.54199
3.26037	3.226706	3.355806	34.70728
3.403154	3.145834	3.308369	34.73893
3.221271	3.25636	3.405921	34.79622
3.229932	3.380094	3.409808	35.09425
3.129793	3.460697	3.359762	34.94208
3.26536	3.474513	3.335617	35.21596
3.316131	3.371603	3.387982	35.21645
3.339804	3.363516	3.326546	35.11618

3.256245	3.434144	3.4781	35.41933
3.277321	3.43839	3.421267	35.35042
3.34529	3.383936	3.43571	35.41156
3.390616	3.302389	3.335566	35.11335
3.242429	3.131075	3.326921	34.39575
3.22556	3.164165	3.38834	34.56554
3.266762	3.421878	3.418743	35.2857
3.27596	3.439536	3.387164	35.27537
3.212775	3.382521	3.411889	35.06658
3.248203	3.329212	3.421131	35.04769
3.255132	3.292685	3.439836	35.02387
3.39041	3.363381	3.408939	35.40674
3.417796	3.469795	3.415811	35.71436
3.24812	3.41615	3.402851	35.19766
3.393462	3.315329	3.393729	35.27507
3.301696	3.185327	3.393456	34.7895
3.358364	3.178992	3.377274	34.86418
3.254307	3.289989	3.368185	34.85948
3.289158	3.358798	3.408564	35.17447
3.120967	3.396538	3.392262	34.85355
3.286188	3.457732	3.381554	35.32526
3.25773	3.37659	3.408581	35.14469
3.401711	3.465887	3.344535	35.51477
3.248698	3.498236	3.425717	35.42843
3.358158	3.411567	3.390182	35.40056
3.300912	3.310072	3.432402	35.14575
3.33535	3.249418	3.356676	34.92282
3.236943	3.178318	3.429179	34.71069
3.262927	3.279813	3.330877	34.77449
3.223457	3.37504	3.431242	35.11591
3.250842	3.416554	3.405648	35.21061
3.25641	3.348958	3.424353	35.11587
3.271712	3.343365	3.391	35.06416
3.331102	3.328943	3.428071	35.24357
3.323225	3.360618	3.423774	35.28621
3.394906	3.440075	3.449504	35.673
3.372304	3.430235	3.382031	35.4545
3.362447	3.339321	3.319316	35.09698
3.273691	3.18654	3.415742	34.77965
3.278228	3.18445	3.329802	34.59706
3.200608	3.289719	3.383498	34.77495
3.310439	3.338782	3.336691	35.02007
3.194009	3.410489	3.412485	35.08801
3.278476	3.401863	3.384009	35.19159
3.271547	3.346465	3.380429	35.04746
3.316337	3.378814	3.382389	35.22044
3.207619	3.404558	3.426076	35.13453
3.409341	3.438996	3.427559	35.65421
3.226839	3.342017	3.427491	35.04288
3.305242	3.252046	3.302759	34.74482

3.273444	3.216125	3.400959	34.81147
3.357003	3.310207	3.387027	35.16948
3.192565	3.381307	3.439614	35.08037
3.241356	3.393304	3.349122	35.01541
3.216775	3.366953	3.433561	35.08868
3.320049	3.299289	3.396167	35.08478
3.278311	3.299694	3.409842	35.02429
3.165386	3.389732	3.469625	35.10498
3.301902	3.456047	3.411241	35.42086
3.382079	3.383262	3.351577	35.30655
3.365128	3.223471	3.338891	34.89231
3.237067	3.146238	3.397429	34.57137
3.247708	3.239578	3.393678	34.79056
3.190545	3.420463	3.360291	34.98811
3.340753	3.429224	3.366702	35.34977
3.336258	3.437783	3.331661	35.28203
3.264205	3.372883	3.339982	35.00073
3.137712	3.314049	3.479566	34.90069
3.338237	3.427	3.305009	35.20449
3.312625	3.414937	3.439222	35.4156
3.347435	3.320451	3.341261	35.07087
3.263587	3.14206	3.408137	34.64365
3.353868	3.242072	3.397958	35.03753
3.274269	3.375444	3.372909	35.10034
3.442872	3.469661	3.472797	35.89353
3.141094	3.325371	3.403209	34.76587
3.258926	3.306231	3.414736	35.0069
3.147198	3.328875	3.353248	34.67763
3.381213	3.349363	3.399987	35.33639
3.261112	3.402604	3.428787	35.25316
3.266886	3.378612	3.495083	35.3583
3.250719	3.355833	3.464254	35.20571
3.401051	3.244431	3.309153	34.95167
3.389503	3.183911	3.339624	34.8607
3.314275	3.229267	3.369021	34.85966
3.192772	3.373422	3.432794	35.04866
3.170954	3.467841	3.37371	35.07822
3.368675	3.471548	3.276005	35.30504
3.39639	3.417026	3.37886	35.47135
3.198917	3.445331	3.43218	35.21801
3.242346	3.421946	3.35098	35.08427
3.312213	3.39418	3.442991	35.37755
3.336093	3.330088	3.305998	34.99003
3.284951	3.184248	3.429537	34.82942
3.155281	3.233446	3.416919	34.62585
3.246553	3.321057	3.436971	35.06089
3.284827	3.398358	3.294574	35.00223
3.382656	3.395528	3.406415	35.45456
3.233025	3.3435	3.416578	35.03579
3.138702	3.405771	3.426724	34.98788

3.242264	3.364931	3.411991	35.09283
3.32306	3.339928	3.331593	35.03902
3.21261	3.291404	3.437841	34.92372
3.285899	3.383531	3.431771	35.27218
3.263628	3.33083	3.429401	35.10305
3.426457	3.246587	3.338891	35.07697
3.433716	3.20696	3.294744	34.90965
3.347146	3.258247	3.387164	35.03459
3.230056	3.350643	3.409212	35.02881
3.281528	3.44176	3.30356	35.10958
3.265525	3.373625	3.377649	35.08761
3.399772	3.400717	3.284513	35.23676
3.197845	3.427944	3.485313	35.29383
3.169139	3.442097	3.362388	34.99319
3.298025	3.379218	3.480538	35.39591
3.393503	3.258247	3.35347	35.06229
3.280043	3.199547	3.38077	34.7455
3.207331	3.262964	3.404028	34.77604
3.250141	3.391888	3.420875	35.18843
3.269691	3.409748	3.405085	35.23571
3.374985	3.399436	3.300627	35.21499
3.264741	3.261212	3.406193	34.90249
3.259504	3.349767	3.392211	35.05411
3.227292	3.404221	3.464663	35.2612
3.280249	3.388316	3.39141	35.18203
3.24247	3.367424	3.390506	35.05175
3.298809	3.301176	3.383498	35.01475
3.232984	3.235198	3.490019	34.95946
3.391936	3.255416	3.345524	35.03529
3.270433	3.259729	3.394394	34.88589
3.384265	3.442568	3.335174	35.40516
3.265731	3.455575	3.479669	35.49037
3.294189	3.437918	3.364195	35.26147
3.2209	3.399302	3.356812	35.0006
3.336876	3.381847	3.366702	35.23768
3.140599	3.466628	3.460912	35.19988
3.282105	3.469661	3.43409	35.45731
3.155323	3.252788	3.467391	34.77861
3.334897	3.164771	3.384845	34.79832
3.411114	3.235535	3.394615	35.14111
3.285652	3.421811	3.440723	35.37493
3.366077	3.440883	3.472899	35.66288
3.212569	3.369783	3.450886	35.12356
3.358983	3.324225	3.356846	35.13846
3.348342	3.413454	3.362814	35.32338
3.198051	3.465684	3.475491	35.35534
3.113708	3.355698	3.462617	34.90222
3.227251	3.285541	3.380292	34.81706
3.25142	3.34323	3.466505	35.18461
3.338732	3.249553	3.370726	34.96124

3.308624	3.233311	3.467664	35.07187
3.27167	3.286552	3.393848	34.94606
3.263215	3.317486	3.346854	34.89245
3.383687	3.387912	3.35792	35.33411
3.301407	3.472087	3.374222	35.3739
3.326029	3.421205	3.397497	35.36738
3.195741	3.422418	3.334781	34.94796
3.358818	3.449577	3.344211	35.3846
3.221312	3.431785	3.374085	35.11032
3.335556	3.359135	3.404983	35.26884
3.145094	3.214575	3.491656	34.72575
3.260452	3.207971	3.406944	34.77832
3.297159	3.279004	3.422648	35.04827
3.474753	3.420935	3.376251	35.64556
3.255833	3.347543	3.488604	35.25202
3.286683	3.355967	3.429997	35.20974
3.260122	3.283991	3.343529	34.80516
3.329947	3.427876	3.400396	35.39687
3.146744	3.4301	3.406688	35.01486
3.150951	3.392158	3.414856	34.95895
3.153219	3.325505	3.48637	34.97454
3.332587	3.296998	3.364588	35.03813
3.369294	3.202377	3.404505	34.99877
3.281363	3.252518	3.401351	34.90923
3.271175	3.298818	3.505024	35.21492
3.271093	3.342623	3.344467	34.95943
3.440438	3.407052	3.370181	35.52688
3.421549	3.428887	3.339147	35.46546
3.291137	3.380229	3.346735	35.09046
3.234469	3.434548	3.412673	35.22952
3.261813	3.446612	3.417908	35.32714
3.284085	3.416959	3.347894	35.15789
3.268206	3.25299	3.352328	34.77429
3.15557	3.131007	3.433681	34.43912
3.227127	3.247059	3.407643	34.79245
3.323266	3.402267	3.365815	35.25064
3.421343	3.441894	3.446179	35.72752
3.233067	3.353878	3.360103	34.93507
3.297612	3.306703	3.413713	35.0903
3.220446	3.326584	3.510771	35.17727
3.351971	3.438727	3.285963	35.21852
3.169139	3.309331	3.455029	34.90545
3.208156	3.351654	3.342983	34.8383
3.21892	3.255079	3.4397	34.86215
3.296705	3.208106	3.294369	34.61171
3.324544	3.196783	3.435317	34.95606
3.42225	3.327729	3.429946	35.44434
3.253111	3.325505	3.452505	35.11893
3.433427	3.468043	3.412417	35.73729
3.376099	3.408804	3.355397	35.35769

3.451863	3.38434	3.365611	35.49221
3.226921	3.423833	3.370368	35.09706
3.128102	3.432998	3.426178	35.02305
3.233438	3.405637	3.420585	35.18134
3.236077	3.345926	3.320543	34.83776
3.282435	3.186203	3.400754	34.76525
3.194133	3.204736	3.385271	34.57882
3.268825	3.371266	3.432146	35.20884
3.189926	3.443579	3.426928	35.18303
3.395236	3.364392	3.366429	35.32654
3.273526	3.360618	3.447322	35.22902
3.292292	3.380229	3.355107	35.11129
3.305119	3.414802	3.392092	35.29583
3.311511	3.374298	3.388068	35.21243
3.192112	3.356978	3.409604	34.96054
3.24016	3.306096	3.34005	34.80224
3.172315	3.287293	3.399594	34.74297
3.252368	3.196783	3.35526	34.62315
3.271794	3.31452	3.420039	35.06477
3.30813	3.325236	3.447202	35.22706
3.308913	3.313846	3.36602	35.02633
3.435778	3.420868	3.409007	35.63181
3.313244	3.395393	3.412144	35.315
3.35259	3.375512	3.342983	35.20632
3.25076	3.386362	3.397821	35.12729
3.232984	3.397886	3.365628	35.04321
3.191287	3.331638	3.355243	34.78445
3.197886	3.230143	3.423518	34.72623
3.27563	3.217541	3.378996	34.77132
3.335639	3.360078	3.421216	35.30659
3.245398	3.482061	3.442292	35.42209
3.332628	3.355159	3.364605	35.16544
3.246429	3.373085	3.456274	35.21661
3.353497	3.380903	3.303407	35.13355
3.278682	3.400447	3.446776	35.32621
3.230922	3.427944	3.296603	34.95349
3.217559	3.293965	3.469199	35.00872
3.162334	3.283519	3.368561	34.64503
3.273774	3.260605	3.411394	34.93229
3.289941	3.220573	3.386397	34.82543
3.281074	3.282913	3.43438	35.0473
3.167819	3.327392	3.407285	34.83765
3.433303	3.39054	3.416015	35.57541
3.391936	3.437918	3.376933	35.50308
3.425467	3.435492	3.36336	35.54142
3.274227	3.386699	3.42391	35.2364
3.328669	3.390743	3.3777	35.26324
3.252657	3.316812	3.377138	34.93411
3.379109	3.319036	3.319793	35.0901
3.087354	3.226369	3.424029	34.47738

3.199907	3.284126	3.31773	34.61736
3.157467	3.323686	3.49522	34.9992
3.277651	3.452475	3.405256	35.34693
3.288374	3.410489	3.516773	35.52243
3.333618	3.347341	3.454364	35.3468
3.173676	3.386699	3.42599	35.02106
3.341454	3.402199	3.380411	35.32218
3.28293	3.451464	3.414668	35.37685
3.250306	3.344645	3.386004	35.00923
3.15656	3.198064	3.383344	34.47785
3.157014	3.221719	3.415811	34.60157
3.280826	3.272534	3.3275	34.79033
3.302685	3.288035	3.400959	35.03267
3.287054	3.3652	3.421966	35.21318
3.273403	3.367963	3.443588	35.23665
3.307181	3.430168	3.418061	35.39073
3.447367	3.420935	3.351151	35.53078
3.47566	3.364729	3.284872	35.3248
3.261978	3.350643	3.402613	35.08419
3.245605	3.369177	3.362081	35.00027
3.266391	3.322271	3.332173	34.87775
3.300788	3.285743	3.37325	34.96292
3.229437	3.244431	3.36752	34.70401
3.179821	3.25454	3.430151	34.75458
3.121379	3.384947	3.454177	34.9645
3.37647	3.448499	3.448839	35.64965
3.254926	3.351722	3.386073	35.03495
3.339021	3.325168	3.420602	35.2363
3.244243	3.360483	3.471058	35.2166
3.348796	3.477141	3.422802	35.59482
3.270474	3.382857	3.413986	35.19809
3.403484	3.256899	3.333707	35.03795
3.121751	3.19402	3.41506	34.46224
3.168892	3.303333	3.397872	34.7668
3.167201	3.262223	3.374341	34.62174
3.291673	3.339321	3.379661	35.07417
3.261607	3.365672	3.427542	35.17076
3.316502	3.375512	3.48961	35.44805
3.253729	3.396471	3.396048	35.15201
3.46271	3.474985	3.345234	35.66959
3.354735	3.425518	3.375995	35.39256
3.322853	3.397482	3.328796	35.15832
3.251544	3.279678	3.428531	34.96286
3.200402	3.25582	3.327978	34.57895
3.326772	3.271995	3.360291	34.96134
3.257854	3.311285	3.357034	34.88942
3.249316	3.371064	3.452727	35.21075
3.211785	3.424035	3.390796	35.10908
3.303057	3.353878	3.413509	35.20492
3.342155	3.336356	3.385544	35.19095

3.318523	3.379825	3.406893	35.28102
3.205186	3.4146	3.458508	35.22209
3.358323	3.398695	3.427525	35.45444
3.207784	3.291741	3.436903	34.91185
3.330318	3.193279	3.329274	34.72913
3.219415	3.232704	3.441933	34.81918
3.1592	3.239578	3.392433	34.59428
3.182543	3.310881	3.451227	34.92983
3.291385	3.429292	3.348031	35.20112
3.245563	3.369648	3.371204	35.02116
3.392225	3.430168	3.356659	35.44243
3.273815	3.399369	3.483744	35.39405
3.389173	3.404019	3.352907	35.37037
3.430334	3.409006	3.405034	35.58528
3.372675	3.345252	3.354919	35.21018
3.248203	3.260605	3.393166	34.83651
3.225148	3.264312	3.3511	34.7022
3.138495	3.317553	3.402255	34.741
3.322317	3.359876	3.351782	35.12517
3.254018	3.409748	3.422853	35.24029
3.120307	3.395325	3.468602	35.0164
3.28227	3.344915	3.403278	35.11749
3.393503	3.411769	3.424609	35.55359
3.306274	3.393101	3.396048	35.25955
3.271505	3.425383	3.447629	35.36691
3.217641	3.304479	3.409808	34.90201
3.16452	3.216732	3.418147	34.61219
3.377996	3.158908	3.343034	34.78832
3.239253	3.291202	3.444406	34.9959
3.216734	3.301109	3.405528	34.8833
3.131608	3.33184	3.392416	34.73567
3.320585	3.375377	3.408939	35.28027
3.292292	3.402199	3.3858	35.22646
3.329865	3.439738	3.413696	35.45172
3.201845	3.423024	3.372448	35.04501
3.424024	3.448566	3.403022	35.65359
3.309944	3.37841	3.362695	35.16251
3.415197	3.237624	3.344876	35.04584
3.28095	3.222326	3.35908	34.74987
3.21129	3.306635	3.395093	34.86066
3.21826	3.367155	3.385118	34.98643
3.323431	3.399975	3.385049	35.28805
3.171201	3.40193	3.413628	35.02192
3.261483	3.344308	3.400072	35.06369
3.186132	3.347543	3.461048	35.03933
3.337536	3.449442	3.437517	35.54181
3.426952	3.443107	3.440314	35.72961
3.293282	3.402536	3.409058	35.28022
3.25241	3.246183	3.422768	34.8789
3.268123	3.150417	3.309971	34.45717

3.23583	3.212351	3.40517	34.73018
3.2972	3.313712	3.363837	34.99565
3.165716	3.356844	3.415828	34.91613
3.165386	3.387979	3.418675	34.98973
3.277032	3.381173	3.466164	35.32285
3.290271	3.388856	3.387675	35.19696
3.296086	3.428887	3.452846	35.43974
3.299922	3.45079	3.40662	35.39493
3.347393	3.345724	3.408512	35.27312
3.299097	3.249351	3.322982	34.76971
3.399855	3.264042	3.359131	35.10123
3.226921	3.30522	3.3873	34.8747
3.269526	3.399773	3.361535	35.1183
3.192895	3.344106	3.409075	34.93295
3.322152	3.348958	3.365884	35.13177
3.263587	3.376455	3.371084	35.0752
3.282105	3.457125	3.360427	35.26881
3.204114	3.382386	3.488177	35.21418
3.316749	3.426933	3.385663	35.33373
3.22985	3.35408	3.439359	35.1018
3.43054	3.315801	3.388136	35.34495
3.310563	3.155808	3.474468	34.9215
3.275588	3.166658	3.358415	34.61495
3.218384	3.221854	3.43102	34.76933
3.25806	3.356507	3.392791	35.06696
3.17813	3.396808	3.412741	35.02393
3.252327	3.412915	3.377069	35.1434
3.195576	3.353743	3.421335	34.9867
3.344465	3.443781	3.406944	35.47772
3.378862	3.473906	3.398827	35.60107
3.318234	3.440951	3.41622	35.43445
3.358776	3.313644	3.405733	35.22178
3.279094	3.226437	3.363019	34.76341
3.337082	3.250496	3.335327	34.88228
3.334195	3.322675	3.359268	35.08616
3.3005	3.380701	3.397582	35.22316
3.183863	3.338445	3.433749	34.95478
3.269608	3.453958	3.39852	35.31786
3.247419	3.406647	3.431651	35.23832
3.319307	3.46993	3.416902	35.50166
3.234469	3.422889	3.489354	35.37171
3.211001	3.361965	3.427764	35.05247
3.257813	3.343837	3.463896	35.19421
3.365252	3.33083	3.392143	35.2438
3.322647	3.188966	3.390046	34.83582
3.288745	3.181418	3.373864	34.70978
3.20399	3.255888	3.387027	34.71608
3.258431	3.355496	3.356949	34.98718
3.254843	3.428887	3.321379	35.06205
3.28458	3.379892	3.360222	35.10487

3.202711	3.399369	3.458576	35.18352
3.28161	3.418711	3.420466	35.31501
3.357003	3.445668	3.424268	35.54715
3.412393	3.369177	3.375245	35.3938
3.329659	3.219967	3.409467	34.96141
3.248409	3.217541	3.322249	34.5877
3.345001	3.315464	3.3511	35.07616
3.340506	3.374635	3.339692	35.17078
3.316172	3.40321	3.371306	35.24919
3.128886	3.310207	3.3622	34.61633
3.285569	3.334334	3.374307	35.03821
3.293241	3.454295	3.372312	35.31296
3.321822	3.441827	3.448072	35.51387
3.227169	3.409882	3.460417	35.26402
3.237108	3.309331	3.47665	35.10137
3.198175	3.342893	3.344876	34.80145
3.434046	3.201434	3.415657	35.1627
3.205557	3.186135	3.426502	34.65329
3.264989	3.225358	3.403857	34.81951
3.258472	3.291404	3.421335	34.98792
3.287054	3.443647	3.382526	35.29848
3.306191	3.393775	3.378178	35.22176
3.324297	3.366346	3.338686	35.11501
3.236985	3.378005	3.431362	35.15224
3.300953	3.46865	3.430748	35.48901
3.255214	3.440075	3.438472	35.34339
3.434211	3.286215	3.315019	35.12838
3.326524	3.213362	3.380599	34.87699
3.232365	3.290259	3.432675	34.95312
3.28957	3.368637	3.407967	35.19558
3.37515	3.359135	3.327483	35.18595
3.266185	3.320721	3.423211	35.073
3.24746	3.344039	3.354527	34.93284
3.138	3.419048	3.476105	35.12337
3.266969	3.480511	3.419852	35.4168
3.311594	3.472154	3.42836	35.51472
3.247048	3.33366	3.377376	34.95921
3.333412	3.30704	3.467579	35.28712
3.22189	3.298885	3.38389	34.84239
3.302067	3.267075	3.35422	34.88328
3.32706	3.193279	3.394547	34.86474
3.236242	3.255618	3.362763	34.73296
3.240408	3.315127	3.417311	34.99149
3.359313	3.394045	3.373506	35.3283
3.292292	3.39391	3.363974	35.1606
3.272825	3.407591	3.357767	35.13437
3.161179	3.424642	3.465891	35.16396
3.26136	3.457597	3.474468	35.47386
3.286889	3.294909	3.361604	34.9271
3.40068	3.229267	3.338072	34.98094

3.305531	3.21653	3.415674	34.91471
3.371974	3.393843	3.398827	35.41092
3.292911	3.38926	3.412724	35.25839
3.367685	3.359202	3.320236	35.15392
3.292704	3.299357	3.39644	35.02573
3.247502	3.389799	3.373659	35.07484
3.207908	3.466291	3.448294	35.31874
3.266762	3.466426	3.409536	35.36299
3.226921	3.395662	3.465055	35.24252
3.264948	3.267884	3.412468	34.93125
3.288085	3.264514	3.433953	35.02147
3.251709	3.275634	3.449453	35.00013
3.259174	3.265592	3.425104	34.94125
3.263587	3.240185	3.345643	34.72157
3.292003	3.324225	3.423859	35.13854
3.228035	3.4208	3.415128	35.19075
3.267917	3.401863	3.417192	35.24106
3.255957	3.398493	3.395349	35.15977
3.306026	3.368233	3.379167	35.16771
3.292168	3.418711	3.41003	35.31528
3.341413	3.423429	3.394326	35.39895
3.207289	3.243083	3.412963	34.75201
3.375191	3.273882	3.330092	35.00531
3.33766	3.270512	3.421284	35.11529
3.363272	3.421002	3.354493	35.35433
3.295468	3.357315	3.407131	35.1819
3.354487	3.372951	3.351765	35.22408
3.257235	3.316744	3.336112	34.85426
3.358694	3.443579	3.386311	35.46328
3.193597	3.507131	3.446793	35.37348
3.229313	3.444253	3.420193	35.25591
3.177264	3.288506	3.403482	34.76495
3.286848	3.225493	3.422103	34.90751
3.287673	3.296054	3.460486	35.14756
3.289982	3.284934	3.409467	35.01672
3.205021	3.225358	3.47058	34.83429
3.289323	3.337097	3.320543	34.93489
3.320668	3.399908	3.429179	35.37836
3.324132	3.404356	3.373523	35.27395
3.236861	3.37322	3.403994	35.08165
3.252616	3.377803	3.405921	35.13034
3.285116	3.449645	3.379116	35.2999
3.348342	3.451599	3.413918	35.51855
3.362241	3.37659	3.363019	35.2736
3.199824	3.197457	3.416305	34.64322
3.246058	3.218417	3.343171	34.63022
3.350528	3.321057	3.425581	35.26336
3.459122	3.408467	3.355465	35.53866
3.263174	3.36729	3.40292	35.12388
3.292416	3.279745	3.436221	35.0692

3.17446	3.328673	3.339897	34.7076
3.32739	3.4733	3.360171	35.40265
3.178048	3.486913	3.421079	35.23903
3.187823	3.356574	3.437858	35.01207
3.064711	3.244229	3.41859	34.45502
3.27963	3.28325	3.38609	34.93928
3.279094	3.342893	3.461833	35.23417
3.331432	3.298885	3.356829	35.02276
3.219786	3.226706	3.399219	34.71347
3.249358	3.277454	3.403073	34.89754
3.372057	3.328471	3.304651	35.0622
3.406454	3.393843	3.416834	35.5257
3.148518	3.393977	3.407353	34.9412
3.18774	3.410017	3.389278	35.02252
3.194751	3.420328	3.349719	34.97389
3.390163	3.404019	3.365304	35.39964
3.364138	3.265525	3.436289	35.1951
3.197886	3.204466	3.465192	34.76121
3.235912	3.300502	3.375654	34.85858
3.363767	3.404019	3.356897	35.32353
3.394576	3.420194	3.407438	35.5368
3.269732	3.328538	3.374767	34.99191
3.293241	3.264514	3.353555	34.85692
3.27134	3.413926	3.390574	35.21672
3.394947	3.531393	3.363394	35.68447
3.294272	3.379083	3.39864	35.20831
3.212197	3.272534	3.38834	34.7733
3.142084	3.226841	3.377206	34.4957
3.25806	3.29073	3.410712	34.96231
3.366118	3.337636	3.400259	35.27833
3.305325	3.293291	3.373659	34.99024
3.204402	3.283384	3.458576	34.93358
3.344218	3.381577	3.420858	35.37158
3.340093	3.394854	3.378911	35.29986
3.438624	3.396808	3.324363	35.40032
3.149631	3.367492	3.399322	34.86815
3.215456	3.334671	3.407012	34.95714
3.195453	3.423226	3.423381	35.14285
3.33667	3.391956	3.321004	35.1594
3.317038	3.198738	3.369993	34.80107
3.292581	3.241735	3.417993	34.94658
3.171985	3.356709	3.463555	35.03392
3.413506	3.394314	3.322197	35.3352
3.433963	3.330223	3.333878	35.26532
3.338732	3.270512	3.363786	34.99189
3.237438	3.371198	3.489269	35.26498
3.248657	3.480376	3.329819	35.17957
3.370696	3.467639	3.3858	35.54102
3.305531	3.347274	3.350537	35.05818
3.222343	3.267547	3.485244	34.9965

3.108759	3.267277	3.408717	34.58016
3.277568	3.309668	3.4124	35.05008
3.322317	3.318766	3.460162	35.27228
3.316749	3.303603	3.342318	34.96924
3.16617	3.292078	3.464271	34.88143
3.312914	3.385486	3.364792	35.18906
3.350033	3.348015	3.380701	35.22308
3.363478	3.348015	3.351509	35.18865
3.21261	3.373827	3.460366	35.15322
3.232778	3.432998	3.367384	35.12339
3.202876	3.421744	3.379525	35.05994
3.337082	3.25609	3.311404	34.8422
3.145589	3.236546	3.437773	34.65704
3.257689	3.294976	3.403448	34.9549
3.263092	3.38616	3.412127	35.1851
3.347764	3.429359	3.338192	35.30305
3.459534	3.324764	3.377223	35.40409
3.376552	3.306298	3.337919	35.09629
3.224735	3.394921	3.455029	35.2142
3.239665	3.518049	3.378928	35.34969
3.211867	3.396269	3.419255	35.11077
3.287137	3.277454	3.341193	34.84484
3.236861	3.202849	3.465465	34.8435
3.137134	3.325573	3.517182	35.00689
3.259875	3.368503	3.405801	35.12562
3.377542	3.29902	3.463282	35.35669
3.293034	3.250159	3.396747	34.91953
3.307057	3.304344	3.451414	35.18824
3.31778	3.302187	3.401487	35.09779
3.258266	3.385149	3.400191	35.14623
3.339969	3.468919	3.324005	35.34149
3.282435	3.361157	3.409314	35.16657
3.291137	3.314049	3.311949	34.86966
3.179161	3.254405	3.381025	34.64541
3.315553	3.255955	3.366105	34.91444
3.199412	3.408534	3.381383	35.02754
3.301489	3.428955	3.406756	35.35091
3.25806	3.395325	3.4161	35.20283
3.473763	3.309061	3.388886	35.42638
3.324998	3.266469	3.33028	34.87974
3.388389	3.371603	3.374682	35.34538
3.319018	3.480646	3.404352	35.49702
3.225272	3.388586	3.342045	34.95444
3.221312	3.194829	3.381707	34.6088
3.270309	3.225561	3.324482	34.65801
3.222797	3.280284	3.398213	34.83502
3.319884	3.426731	3.411531	35.39671
3.161056	3.419857	3.460639	35.14174
3.324379	3.245577	3.437381	35.06692
3.385708	3.2879	3.405648	35.22419

3.46803	3.355361	3.381793	35.49958
3.346362	3.369648	3.453494	35.42155
3.287384	3.39108	3.325028	35.05851
3.241315	3.361292	3.443366	35.15141
3.393008	3.33548	3.294182	35.10045
3.288869	3.266266	3.400549	34.95396
3.144764	3.29538	3.359387	34.61248
3.234675	3.320653	3.462753	35.09041
3.27365	3.418239	3.393916	35.23852
3.327349	3.402065	3.44473	35.4317
3.323183	3.415611	3.399305	35.35287
3.323431	3.315868	3.385339	35.10475
3.281156	3.360146	3.390932	35.12136
3.444769	3.4733	3.312836	35.55582
3.312584	3.494394	3.446248	35.60464
3.295179	3.25791	3.326205	34.78691
3.185389	3.057683	3.378775	34.22391
3.288498	3.257977	3.425445	34.98946
3.160231	3.415948	3.400686	35.00028
3.290766	3.50666	3.414276	35.51383
3.125339	3.297739	3.504069	34.89156
3.349002	3.252922	3.385442	35.02324
3.258885	3.299963	3.398367	34.95731
3.484033	3.430909	3.412878	35.76776
3.303428	3.470402	3.459411	35.56093
3.283713	3.403008	3.420858	35.28613
3.198711	3.291943	3.38389	34.77652
3.3622	3.273275	3.306953	34.92497
3.268288	3.276578	3.372568	34.87031
3.19834	3.328066	3.401896	34.89408
3.067639	3.412511	3.461526	34.92333
3.351724	3.45416	3.395434	35.49112
3.403691	3.394854	3.3873	35.45728
3.338278	3.348082	3.380497	35.19708
3.240614	3.354754	3.50707	35.27489
3.310522	3.438592	3.371664	35.31499
3.336835	3.447623	3.383771	35.41876
3.348259	3.306972	3.315206	34.98622
3.129422	3.150821	3.289714	34.11044
3.103645	3.166591	3.383839	34.29439
3.254472	3.327999	3.388426	34.98723
3.249605	3.489137	3.467255	35.50136
3.277403	3.38097	3.378911	35.13241
3.261442	3.23358	3.428514	34.88366
3.293241	3.290932	3.427747	35.07694
3.25175	3.380768	3.357801	35.0297
3.460441	3.427203	3.361535	35.59579
3.242058	3.405165	3.406688	35.16877
3.332546	3.317014	3.394053	35.14625
3.310645	3.193818	3.300542	34.62445

3.382038	3.217676	3.342182	34.92381
3.210713	3.349093	3.34815	34.84959
3.243295	3.395056	3.396576	35.12725
3.201185	3.420531	3.406483	35.11254
3.319348	3.422957	3.35347	35.26032
3.278888	3.323281	3.417908	35.09478
3.447285	3.379488	3.386772	35.51786
3.240449	3.350104	3.413833	35.06046
3.362241	3.464606	3.44253	35.63996
3.272825	3.445736	3.420176	35.35427
3.313367	3.186877	3.334867	34.69029
3.166541	3.15965	3.323596	34.28501
3.244202	3.281026	3.405869	34.90019
3.151446	3.475187	3.42067	35.15432
3.245316	3.436301	3.406654	35.24391
3.294478	3.322742	3.440109	35.17624
3.290354	3.232704	3.34624	34.76505
3.349909	3.335008	3.454552	35.35587
3.336917	3.419587	3.359694	35.30498
3.325782	3.484689	3.461594	35.64584
3.366242	3.405367	3.369652	35.35979
3.389668	3.162884	3.360035	34.85971
3.285033	3.163626	3.34965	34.60981
3.238593	3.32591	3.387437	34.94577
3.199824	3.417026	3.393234	35.07293
3.270804	3.465954	3.421284	35.39649
3.303469	3.407119	3.416288	35.32833
3.316254	3.325034	3.426195	35.19845
3.243377	3.367424	3.400549	35.0757
3.418703	3.467032	3.377342	35.62618
3.228777	3.471885	3.463487	35.40984
3.410743	3.389125	3.36868	35.41946
3.248986	3.132894	3.435522	34.65156
3.347971	3.173533	3.350946	34.77194
3.245728	3.306433	3.393319	34.93165
3.331185	3.455912	3.435181	35.53696
3.209269	3.416959	3.431515	35.17715
3.227375	3.326045	3.409058	34.96882
3.180522	3.282913	3.503814	34.97925
3.388884	3.37349	3.382117	35.36685
3.34628	3.388182	3.468346	35.49438
3.307263	3.417633	3.435198	35.40097
3.300829	3.388249	3.42345	35.29695
3.367891	3.254675	3.383958	35.06514
3.315512	3.237557	3.338055	34.81278
3.285858	3.248677	3.30975	34.71035
3.170789	3.308387	3.414941	34.81932
3.180275	3.453958	3.436699	35.20599
3.280579	3.459012	3.382253	35.31733
3.324503	3.433672	3.40043	35.39772

3.331267	3.405097	3.384299	35.31474
3.170871	3.417565	3.531301	35.31272
3.396184	3.438188	3.371903	35.50196
3.407609	3.431988	3.427576	35.63514
3.344341	3.322001	3.335907	35.05579
3.220735	3.130333	3.388153	34.48059
3.218961	3.190988	3.345439	34.51594
3.217435	3.312364	3.326563	34.73676
3.331308	3.435492	3.417038	35.4529
3.142991	3.353272	3.402954	34.83047
3.209846	3.285406	3.447936	34.92663
3.255833	3.316744	3.372517	34.9308
3.311594	3.447892	3.418266	35.43959
3.222178	3.419048	3.40928	35.16132
3.312089	3.422552	3.432044	35.41539
3.254059	3.336895	3.437227	35.1125
3.348342	3.245913	3.309221	34.83979
3.335804	3.235939	3.311625	34.79582
3.313202	3.298615	3.376132	35.02452
3.149714	3.381375	3.39748	34.89466
3.248368	3.437446	3.383958	35.20345
3.210053	3.39236	3.379729	35.01182
3.411486	3.390203	3.377615	35.44298
3.272042	3.354687	3.449163	35.21684
3.271382	3.411365	3.366804	35.15923
3.312996	3.435492	3.450749	35.48657
3.342485	3.399302	3.382816	35.32336
3.314605	3.200894	3.341346	34.73782
3.215208	3.210599	3.398367	34.66637
3.184853	3.322338	3.423876	34.90013
3.223869	3.468582	3.372363	35.19261
3.258431	3.435694	3.433953	35.33096
3.279383	3.318025	3.417601	35.08369
3.189678	3.191796	3.461117	34.70664
3.147321	3.31762	3.416765	34.79219
3.31114	3.467841	3.415487	35.47614
3.29918	3.439738	3.390506	35.3339
3.335928	3.331975	3.391887	35.18162
3.316626	3.194829	3.382236	34.81839
3.292911	3.14644	3.34167	34.572
3.2428	3.314183	3.416083	34.99197
3.399484	3.51104	3.357494	35.63699
3.194256	3.47303	3.426655	35.25631
3.36884	3.426057	3.348781	35.36507
3.258967	3.352193	3.415759	35.10974
3.344259	3.415274	3.351577	35.29385
3.198834	3.421878	3.437108	35.17732
3.328009	3.408804	3.452966	35.46589
3.174088	3.274556	3.430969	34.78761
3.356261	3.169489	3.303083	34.67656

3.300912	3.189438	3.378024	34.76303
3.350693	3.432392	3.414515	35.48299
3.240944	3.454564	3.47469	35.42307
3.202093	3.414128	3.410166	35.10858
3.199907	3.37996	3.376711	34.95591
3.32838	3.329482	3.392978	35.16205
3.204774	3.390608	3.438643	35.12528
3.25542	3.428483	3.497129	35.44676
3.194091	3.333525	3.353606	34.79113
3.383852	3.334806	3.432589	35.38163
3.320049	3.228256	3.3526	34.83417
3.297777	3.18445	3.426297	34.85083
3.264535	3.263436	3.365542	34.818
3.196442	3.354754	3.362422	34.86197
3.375068	3.4795	3.438182	35.69107
3.23649	3.416554	3.386345	35.13701
3.229313	3.396606	3.391188	35.08828
3.223251	3.398156	3.434294	35.17268
3.346486	3.487655	3.40899	35.58256
3.28891	3.4177	3.439273	35.3699
3.31778	3.365537	3.401572	35.23651
3.237892	3.250901	3.398776	34.805
3.325823	3.166793	3.36515	34.73983
3.3695	3.250159	3.313774	34.9053
3.355353	3.396875	3.429588	35.44848
3.25043	3.358865	3.398571	35.06808
3.333247	3.383868	3.389278	35.28354
3.184729	3.284934	3.363309	34.68561
3.371314	3.26849	3.361058	35.05276
3.215373	3.339658	3.423739	35.00445
3.196442	3.446679	3.523167	35.41452
3.168603	3.458608	3.467374	35.25772
3.412888	3.313105	3.32762	35.16811
3.361787	3.180137	3.437108	35.00502
3.391111	3.20824	3.349582	34.9392
3.199742	3.329145	3.511043	35.13819
3.277321	3.445534	3.317952	35.14011
3.339433	3.398291	3.277284	35.08369
3.32871	3.396067	3.347672	35.20931
3.197886	3.344982	3.415521	34.95988
3.227416	3.43657	3.39777	35.18592
3.256328	3.448836	3.396048	35.27221
3.367438	3.390473	3.338107	35.26085
3.354693	3.210464	3.472916	35.13413
3.156972	3.211677	3.404096	34.5539
3.281857	3.321529	3.419511	35.10095
3.303964	3.424642	3.389074	35.30822
3.403154	3.457193	3.474741	35.78366
3.189101	3.352328	3.415009	34.95561
3.215249	3.245711	3.410218	34.76916

3.265236	3.254877	3.387317	34.84844
3.361457	3.406176	3.361467	35.33319
3.23253	3.432055	3.477622	35.36186
3.288085	3.432527	3.378519	35.26766
3.185348	3.249688	3.420466	34.73488
3.379563	3.232974	3.312018	34.88589
3.380759	3.169893	3.405545	34.95508
3.312873	3.334873	3.381111	35.11398
3.217559	3.432931	3.491178	35.36068
3.441428	3.473839	3.351441	35.63412
3.307717	3.380229	3.401351	35.24615
3.369129	3.381914	3.311063	35.18669
3.215909	3.422013	3.437108	35.21495
3.178048	3.506323	3.452301	35.34975
3.194875	3.422957	3.420585	35.13488
3.260988	3.180407	3.428497	34.76635
3.274805	3.174341	3.401112	34.72341
3.351146	3.285137	3.454961	35.25041
3.265484	3.439805	3.426314	35.33867
3.363437	3.458204	3.42715	35.59494
3.376099	3.37969	3.35035	35.28298
3.282352	3.316407	3.436613	35.12823
3.18477	3.310611	3.427286	34.88176
3.262019	3.349767	3.366225	35.00279
3.184482	3.384475	3.400839	34.98482
3.281981	3.422283	3.422358	35.32778
3.344382	3.310746	3.424063	35.22405
3.171449	3.194627	3.442309	34.63184
3.275547	3.255686	3.387795	34.8738
3.284043	3.236411	3.41078	34.90049
3.317121	3.448027	3.386226	35.3819
3.292498	3.428955	3.364877	35.23966
3.36884	3.38306	3.42906	35.4466
3.293571	3.384745	3.232199	34.85518
3.360261	3.399369	3.389125	35.37618
3.129711	3.470065	3.357034	34.95642
3.291426	3.411702	3.439956	35.36377
3.15821	3.256696	3.396116	34.63761
3.258926	3.161132	3.326136	34.49584
3.279713	3.255618	3.372517	34.84935
3.31444	3.401458	3.415435	35.33808
3.293158	3.461978	3.519092	35.65056
3.322317	3.396673	3.349787	35.20128
3.289817	3.27159	3.428855	35.02958
3.411692	3.3559	3.262705	35.11713
3.221436	3.349228	3.447663	35.09095
3.126494	3.395056	3.375927	34.82667
3.100386	3.421744	3.471416	35.03676
3.321451	3.387845	3.409058	35.30969
3.284868	3.222797	3.45878	34.9775

3.267381	3.21498	3.386618	34.76435
3.154622	3.257101	3.464629	34.78047
3.309119	3.390069	3.375057	35.21323
3.394246	3.45288	3.380633	35.54894
3.356797	3.389193	3.289851	35.12925
3.318564	3.361359	3.37209	35.16462
3.242511	3.347274	3.35301	34.92577
3.319018	3.451127	3.316281	35.23987
3.295303	3.450723	3.421625	35.4175
3.276042	3.32564	3.360768	34.96876
3.119523	3.156684	3.463009	34.48058
3.250801	3.187214	3.347178	34.58112
3.226962	3.381038	3.502177	35.29181
3.3443	3.503627	3.370181	35.52784
3.228653	3.39728	3.4449	35.20577
3.321204	3.304276	3.426604	35.16477
3.275753	3.331234	3.376677	35.01515
3.409217	3.369514	3.345712	35.32301
3.215249	3.336962	3.401965	34.95066
3.206382	3.358057	3.408223	34.99109
3.061164	3.348015	3.422989	34.68385
3.322029	3.281093	3.356471	34.96251
3.320833	3.265795	3.35729	34.92823
3.275465	3.266064	3.453665	35.04036
3.217229	3.349902	3.423927	35.03132
3.327638	3.378949	3.451158	35.39583
3.453966	3.43657	3.273583	35.40978
3.436149	3.416015	3.386482	35.57275
3.235665	3.393101	3.351492	35.0077
3.190668	3.39236	3.379116	34.96809
3.314646	3.444118	3.367316	35.32659
3.225313	3.39856	3.345916	34.98481
3.249275	3.245509	3.381366	34.78003
3.125627	3.169085	3.425223	34.43842
3.200113	3.339591	3.448805	35.02574
3.433345	3.461236	3.406671	35.70966
3.320874	3.430842	3.459616	35.51302
3.263752	3.409208	3.38227	35.17165
3.299633	3.299087	3.427269	35.10771
3.26767	3.339658	3.401163	35.06944
3.377212	3.369244	3.356812	35.2767
3.278187	3.356102	3.39129	35.10681
3.192483	3.371603	3.465874	35.11639
3.168273	3.291809	3.360171	34.65779
3.370325	3.239511	3.388545	35.04733
3.250925	3.231222	3.375432	34.73942
3.259875	3.319305	3.410371	35.02802
3.165551	3.36729	3.458985	35.033
3.43553	3.474783	3.409126	35.74943
3.366365	3.432122	3.387914	35.4585

3.43153	3.402941	3.34462	35.44252
3.315306	3.394517	3.403056	35.29772
3.184069	3.409074	3.331644	34.88639
3.154003	3.397145	3.398912	34.94166
3.228942	3.27833	3.328302	34.6913
3.183616	3.169489	3.392501	34.49455
3.197638	3.231087	3.393302	34.66168
3.200897	3.445197	3.508435	35.3888
3.329576	3.49527	3.411786	35.56835
3.421466	3.399369	3.363241	35.45342
3.379192	3.307107	3.414804	35.27197
3.305119	3.312566	3.441405	35.18009
3.26767	3.36729	3.399594	35.12644
3.269196	3.374838	3.475951	35.31326
3.266185	3.378544	3.3356	35.00786
3.338443	3.234322	3.441405	35.08186
3.122245	3.209453	3.396986	34.45755
3.301654	3.250766	3.415964	34.98173
3.22156	3.306837	3.471194	35.04998
3.25109	3.383936	3.402255	35.1324
3.289611	3.355091	3.390642	35.12817
3.408598	3.397482	3.366378	35.42801
3.357415	3.38771	3.367401	35.29695
3.383852	3.456519	3.277301	35.3082
3.246966	3.401997	3.458525	35.28593
3.340464	3.398695	3.357972	35.26328
3.18077	3.264851	3.34902	34.60178
3.200113	3.17825	3.322112	34.39586
3.157096	3.257775	3.445497	34.74552
3.230344	3.422687	3.396116	35.15835
3.227251	3.442029	3.498221	35.41717
3.322895	3.37167	3.380514	35.21506
3.291921	3.332514	3.457706	35.2305
3.429591	3.413185	3.315087	35.39609
3.360096	3.414465	3.406534	35.4469
3.283548	3.348689	3.394547	35.10945
3.173923	3.246722	3.381008	34.61712
3.179079	3.220034	3.436204	34.69074
3.281074	3.264716	3.31686	34.75051
3.21694	3.246924	3.401504	34.75646
3.23385	3.310342	3.403039	34.93547
3.227292	3.288574	3.449351	34.97481
3.296416	3.420531	3.364042	35.22798
3.308377	3.386295	3.409689	35.27909
3.39305	3.424776	3.317542	35.3469
3.317533	3.448297	3.368015	35.34357
3.351064	3.399908	3.342011	35.25421
3.379192	3.404828	3.323749	35.28655
3.310439	3.309061	3.347826	34.97942
3.143733	3.24005	3.386414	34.54833

3.229767	3.207836	3.362541	34.61382
3.184399	3.288304	3.39407	34.75953
3.301201	3.447084	3.34965	35.26504
3.224447	3.392697	3.464118	35.22858
3.265236	3.349363	3.435522	35.16048
3.2501	3.335278	3.429332	35.08304
3.286642	3.375444	3.436903	35.26735
3.339763	3.419654	3.417925	35.43869
3.374696	3.333997	3.402988	35.2951
3.186956	3.261886	3.377069	34.67017
3.232613	3.233917	3.385169	34.72656
3.330483	3.302726	3.339164	34.99046
3.249316	3.277521	3.375722	34.83779
3.188441	3.32874	3.419903	34.91329
3.23418	3.339995	3.40251	34.99989
3.333948	3.42754	3.434721	35.47995
3.498798	3.453756	3.360359	35.73516
3.346898	3.394517	3.434141	35.43478
3.26701	3.388653	3.36486	35.09575
3.294354	3.333727	3.407558	35.12881
3.333907	3.316273	3.341346	35.03234
3.384512	3.290326	3.378587	35.1677
3.141382	3.268288	3.373523	34.57675
3.089168	3.258449	3.426519	34.55694
3.264205	3.409411	3.439137	35.29745
3.306851	3.464404	3.413781	35.45552
3.235376	3.355159	3.371903	34.96873
3.320049	3.342758	3.463759	35.32765
3.207661	3.374568	3.470785	35.1668
3.417218	3.443242	3.361246	35.53571
3.336051	3.379757	3.404437	35.31383
3.342238	3.25582	3.363599	34.96702
3.231293	3.168141	3.379047	34.56645
3.268371	3.217271	3.394002	34.78767
3.186255	3.281767	3.376882	34.7117
3.287797	3.362707	3.361859	35.07791
3.130247	3.390271	3.482192	35.0568
3.236902	3.406984	3.354169	35.04662
3.316461	3.41487	3.45387	35.45587
3.420972	3.409208	3.300474	35.33659
3.351105	3.399773	3.401027	35.38306
3.282146	3.414196	3.384794	35.2283
3.222467	3.31142	3.467102	35.05303
3.332051	3.277858	3.299996	34.85385
3.311635	3.226504	3.378792	34.86921
3.237067	3.253057	3.358227	34.71924
3.207413	3.348217	3.424626	35.0077
3.240697	3.448229	3.462549	35.38212
3.320833	3.400582	3.40101	35.31859
3.281363	3.355496	3.427883	35.19245

3.283219	3.38926	3.432743	35.28097
3.258143	3.401526	3.463436	35.32008
3.328586	3.444388	3.366412	35.35569
3.277486	3.354552	3.388835	35.09652
3.38179	3.21626	3.365611	34.97141
3.161963	3.158773	3.422154	34.48861
3.25307	3.180946	3.36752	34.61686
3.206877	3.313442	3.418283	34.9166
3.242924	3.366413	3.354817	34.97248
3.215621	3.377466	3.4375	35.11776
3.26635	3.41588	3.454211	35.34925
3.203784	3.394247	3.490565	35.24462
3.410991	3.44924	3.369635	35.55355
3.330071	3.413522	3.389892	35.34278
3.376552	3.365739	3.372619	35.30216
3.256163	3.297739	3.403056	34.95675
3.275465	3.276847	3.34138	34.81839
3.248986	3.270917	3.328865	34.72015
3.30516	3.315666	3.362405	35.0142
3.180192	3.369177	3.411172	34.96458
3.178419	3.409141	3.443383	35.11854
3.236448	3.396134	3.415026	35.15498
3.274516	3.392967	3.402033	35.20289
3.318688	3.371738	3.413764	35.27872
3.229932	3.425787	3.494538	35.37946
3.218755	3.369783	3.39598	35.01702
3.243047	3.252316	3.389619	34.79935
3.390781	3.172522	3.365219	34.89456
3.25674	3.212284	3.409825	34.78594
3.222178	3.264042	3.366497	34.72879
3.20465	3.331773	3.397736	34.90689
3.357539	3.414465	3.35231	35.32273
3.285734	3.358259	3.370607	35.0828
3.326318	3.438255	3.36087	35.3252
3.226385	3.456788	3.402732	35.23873
3.295757	3.449106	3.41801	35.40705
3.353167	3.382655	3.31536	35.1628
3.398576	3.294167	3.361058	35.16853
3.257895	3.263638	3.37105	34.81597
3.289611	3.262762	3.367964	34.87666
3.238758	3.271119	3.389756	34.83138
3.293488	3.380027	3.398281	35.20788
3.268618	3.437514	3.43941	35.36915
3.174295	3.382184	3.439001	35.04099
3.145301	3.359876	3.449999	34.95285
3.279424	3.433066	3.458115	35.42396
3.287755	3.429561	3.468824	35.45793
3.243089	3.454025	3.372039	35.2021
3.190668	3.282306	3.456206	34.896
3.252657	3.175554	3.338277	34.54022

3.378862	3.198064	3.352856	34.89732
3.273444	3.231828	3.344467	34.72228
3.14827	3.310814	3.380139	34.69928
3.234304	3.370794	3.415009	35.09484
3.2753	3.401391	3.370828	35.15479
3.359643	3.425989	3.345166	35.33691
3.31411	3.426394	3.439836	35.44525
3.174872	3.431516	3.441865	35.15639
3.293323	3.366144	3.414429	35.21247
3.298396	3.337636	3.345729	35.01099
3.409052	3.314116	3.384708	35.28678
3.231417	3.245711	3.385527	34.75052
3.284291	3.302996	3.384436	34.98903
3.281816	3.338849	3.398486	35.09275
3.344465	3.385823	3.350622	35.22781
3.145466	3.390877	3.415623	34.94583
3.270351	3.390945	3.389023	35.16091
3.132103	3.373759	3.457485	34.97072
3.2793	3.439738	3.402647	35.31698
3.360179	3.399773	3.45682	35.52492
3.366242	3.37787	3.374	35.30916
3.209764	3.218012	3.4508	34.78534
3.274681	3.215317	3.354237	34.71024
3.309903	3.231626	3.423859	34.97518
3.357869	3.324697	3.344757	35.11062
3.119028	3.315733	3.404625	34.69964
3.1738	3.387036	3.357938	34.87324
3.306356	3.344174	3.389926	35.13934
3.266474	3.448432	3.382321	35.26349
3.292003	3.46063	3.449095	35.49202
3.299015	3.44149	3.400822	35.35993
3.248533	3.303063	3.409553	34.96591
3.380594	3.241196	3.318003	34.91921
3.40996	3.25764	3.357972	35.10679
3.244697	3.331167	3.374955	34.94332
3.313821	3.346802	3.350622	35.07546
3.272124	3.368705	3.384043	35.10526
3.33102	3.360078	3.387863	35.22355
3.240985	3.386564	3.409126	35.13108
3.298685	3.425383	3.451363	35.43451
3.098283	3.423765	3.50985	35.12063
3.259339	3.405637	3.39453	35.181
3.205351	3.305018	3.424422	34.90827
3.362695	3.264784	3.398981	35.10873
3.220776	3.243016	3.426911	34.81186
3.316337	3.219226	3.439154	34.99558
3.264082	3.272534	3.43704	34.99326
3.352384	3.349632	3.326187	35.11255
3.217641	3.448701	3.418675	35.23679
3.338856	3.401997	3.396031	35.35022

3.12802	3.395662	3.403346	34.89129
3.271299	3.443242	3.425513	35.35715
3.257813	3.43064	3.415401	35.27798
3.366984	3.356574	3.331985	35.17234
3.339268	3.228526	3.348167	34.86709
3.281115	3.240657	3.383225	34.84312
3.308295	3.302592	3.39482	35.06335
3.340712	3.343702	3.345473	35.11623
3.265236	3.357046	3.330894	34.94848
3.256617	3.327325	3.397804	35.01095
3.264205	3.36237	3.379252	35.06362
3.233974	3.429427	3.361808	35.106
3.310769	3.468111	3.409962	35.46384
3.203742	3.427203	3.469557	35.27065
3.147569	3.289854	3.472695	34.85431
3.187823	3.276106	3.420039	34.79713
3.427447	3.246924	3.44623	35.31461
3.267793	3.162682	3.34965	34.57004
3.257978	3.21188	3.450988	34.87777
3.243295	3.344645	3.406978	35.03976
3.369706	3.351519	3.384453	35.28197
3.315265	3.471008	3.305589	35.25176
3.328916	3.393977	3.374273	35.26336
3.146167	3.418037	3.421216	35.01899
3.257936	3.457462	3.392075	35.2859
3.246223	3.437581	3.436426	35.3138
3.340547	3.357989	3.354698	35.16729
3.28194	3.175015	3.404574	34.74806
3.32273	3.223876	3.370351	34.86927
3.290024	3.314116	3.465601	35.20338
3.351353	3.404087	3.361587	35.30679
3.306067	3.306905	3.390046	35.05747
3.218631	3.371064	3.35729	34.93494
3.117296	3.342421	3.44577	34.84419
3.290271	3.478691	3.359131	35.33099
3.271918	3.47485	3.457143	35.49679
3.230221	3.400784	3.380292	35.07558
3.193184	3.270445	3.508435	34.98978
3.220322	3.287698	3.402783	34.85581
3.357787	3.214306	3.391427	34.97109
3.332917	3.269232	3.365969	34.98115
3.281775	3.28635	3.370709	34.91711
3.258926	3.299155	3.401419	34.9623
3.337289	3.399706	3.364792	35.27346
3.290684	3.353339	3.347212	35.0317
3.352879	3.334199	3.404028	35.25011
3.106614	3.463123	3.473104	35.14456
3.2574	3.494259	3.444406	35.47964
3.303057	3.373422	3.376933	35.16768
3.313574	3.272534	3.314678	34.83391

3.243584	3.215182	3.387368	34.71439
3.266556	3.353272	3.357699	35.00173
3.276826	3.385958	3.492628	35.39073
3.410248	3.429561	3.301463	35.35981
3.309119	3.304209	3.41049	35.10296
3.306356	3.36082	3.35214	35.09311
3.249729	3.397347	3.445139	35.25253
3.265608	3.481252	3.389346	35.34873
3.261401	3.411298	3.398503	35.20658
3.258967	3.30495	3.384947	34.93905
3.198422	3.307714	3.414174	34.8766
3.262473	3.242813	3.465277	34.9865
3.282435	3.229874	3.375518	34.80557
3.366819	3.218889	3.422989	35.06989
3.30615	3.31917	3.350333	34.99763
3.284332	3.327864	3.409194	35.09765
3.323678	3.414398	3.413509	35.38236
3.193679	3.404693	3.354851	34.94858
3.308418	3.360483	3.372448	35.1413
3.265896	3.446005	3.452113	35.40955
3.315306	3.421878	3.347962	35.23707
3.25307	3.266469	3.412963	34.90326
3.335845	3.243824	3.310995	34.81177
3.20201	3.235265	3.429264	34.75902
3.317574	3.404693	3.399032	35.31613
3.338938	3.360887	3.415145	35.3023
3.33799	3.350845	3.365082	35.16878
3.325534	3.266469	3.349258	34.92242
3.357085	3.416083	3.377683	35.38076
3.213847	3.507468	3.442002	35.40802
3.258101	3.446208	3.462003	35.41457
3.137671	3.358057	3.38227	34.78407
3.27134	3.2879	3.442275	35.05418
3.239665	3.322944	3.41992	35.01267
3.279672	3.25892	3.420125	34.96059
3.246636	3.233243	3.434959	34.86464
3.281198	3.269299	3.408052	34.96023
3.308583	3.358731	3.409877	35.21968
3.308088	3.420194	3.292801	35.09698
3.284868	3.307579	3.422512	35.08358
3.214342	3.362168	3.380701	34.9573
3.323183	3.411163	3.376882	35.29411
3.312089	3.477276	3.345712	35.34626
3.312089	3.345454	3.40367	35.18473
3.23781	3.236613	3.406262	34.78995
3.291137	3.226841	3.384026	34.83657
3.338938	3.357383	3.371698	35.19962
3.360797	3.39701	3.405239	35.40743
3.273733	3.374164	3.478577	35.32745
3.283342	3.344308	3.407217	35.12712

3.219167	3.342152	3.370334	34.90141
3.383564	3.47303	3.367248	35.54038
3.231788	3.430033	3.409655	35.20718
3.168191	3.377601	3.406944	34.94751
3.142166	3.267816	3.411258	34.65995
3.274475	3.310477	3.36099	34.93265
3.196566	3.281632	3.424422	34.83792
3.34595	3.290393	3.428514	35.1927
3.18675	3.236883	3.452437	34.77986
3.386822	3.323012	3.404863	35.3017
3.418002	3.348689	3.339061	35.28214
3.405876	3.346802	3.410951	35.4087
3.258266	3.397482	3.432726	35.24435
3.200814	3.413319	3.419494	35.12441
3.213435	3.414196	3.363036	35.03046
3.357952	3.437649	3.320578	35.30493
3.292787	3.261751	3.432555	35.02265
3.151405	3.200692	3.415128	34.54183
3.237521	3.26876	3.44282	34.93956
3.415239	3.470335	3.363701	35.59599
3.359519	3.409141	3.359523	35.33119
3.32207	3.383464	3.42067	35.32686
3.208032	3.31789	3.448754	34.99549
3.259545	3.440883	3.381707	35.23049
3.360261	3.47977	3.386755	35.54682
3.287467	3.379892	3.388068	35.17208
3.207124	3.290393	3.457229	34.95191
3.081744	3.241398	3.423671	34.49719
3.320874	3.351182	3.42703	35.26756
3.265278	3.382184	3.382048	35.11541
3.278517	3.290596	3.442462	35.07618
3.154787	3.311285	3.468807	34.90846
3.307635	3.340736	3.376728	35.10576
3.389214	3.328471	3.352669	35.20472
3.43751	3.399436	3.368527	35.50021
3.147569	3.366211	3.467784	35.01056
3.23616	3.370457	3.364724	34.9882
3.278805	3.36237	3.398554	35.13775
3.42258	3.291606	3.301497	35.08517
3.231623	3.210532	3.435198	34.78266
3.19273	3.33521	3.393575	34.87924
3.173346	3.364729	3.478083	35.08621
3.475578	3.458541	3.337817	35.64555
3.352219	3.379083	3.453392	35.45477
3.367231	3.3466	3.358057	35.20808
3.259009	3.395797	3.398912	35.16835
3.343186	3.477411	3.366668	35.46039
3.306933	3.367829	3.346308	35.09695
3.293406	3.262223	3.344876	34.83329
3.116389	3.242544	3.365201	34.4476

3.114203	3.287765	3.439973	34.70522
3.310893	3.354148	3.356471	35.09791
3.254843	3.27496	3.437159	34.97862
3.272908	3.243016	3.375194	34.81276
3.167984	3.321934	3.48961	35.0061
3.292168	3.42262	3.385936	35.27114
3.347929	3.402536	3.30547	35.17319
3.429426	3.342152	3.429128	35.48978
3.218879	3.330425	3.410439	34.96284
3.251007	3.404693	3.40367	35.18071
3.26004	3.376725	3.387283	35.10346
3.403196	3.261818	3.340579	35.0631
3.208815	3.251507	3.373335	34.68711
3.222921	3.298144	3.366156	34.80424
3.285446	3.39263	3.464936	35.36362
3.394122	3.40995	3.354578	35.39781
3.36521	3.368031	3.440842	35.43157
3.350899	3.375175	3.356761	35.23202
3.304459	3.428955	3.441337	35.43302
3.314192	3.460023	3.427542	35.49208
3.217806	3.347476	3.35937	34.8861
3.308171	3.237287	3.369379	34.86463
3.20531	3.249418	3.34474	34.61234
3.146332	3.330425	3.463708	34.92068
3.161509	3.313712	3.4161	34.81321
3.297654	3.281565	3.372295	34.94484
3.274145	3.274017	3.490616	35.13567
3.376594	3.355226	3.366907	35.26677
3.282889	3.343163	3.477605	35.27755
3.309738	3.37841	3.369925	35.17787
3.361787	3.392832	3.421693	35.43644
3.304789	3.452745	3.372227	35.33464
3.216198	3.353339	3.389517	34.96133
3.293488	3.276241	3.356931	34.89049
3.292746	3.257236	3.412895	34.96969
3.23983	3.402132	3.350946	35.03537
3.32805	3.414533	3.378706	35.31611
3.212197	3.412309	3.400532	35.10563
3.339103	3.267884	3.358296	34.97495
3.25839	3.247868	3.345712	34.72715
3.443903	3.42417	3.403823	35.64547
3.293199	3.508277	3.417652	35.53007
3.377006	3.392899	3.377888	35.37407
3.213558	3.170702	3.409945	34.60083
3.295468	3.208577	3.288009	34.59613
3.140681	3.326853	3.437568	34.84335
3.307387	3.464067	3.376933	35.37537
3.121833	3.334469	3.483556	34.91935
3.248203	3.290798	3.336572	34.77877
3.341619	3.268221	3.441814	35.16383

3.372016	3.302929	3.336572	35.07606
3.266886	3.39573	3.480879	35.36467
3.308377	3.484689	3.394752	35.4616
3.279837	3.315127	3.434141	35.11452
3.410166	3.320047	3.283166	35.08013
3.263009	3.220371	3.382799	34.75823
3.180729	3.229874	3.368083	34.56689
3.238428	3.403076	3.402596	35.14731
3.244697	3.461102	3.425291	35.33755
3.328421	3.469728	3.348167	35.37084
3.332711	3.355024	3.428292	35.3046
3.301489	3.335884	3.473053	35.29236
3.318399	3.365066	3.392024	35.21595
3.462091	3.4764	3.375586	35.73771
3.299427	3.430437	3.389295	35.31146
3.337454	3.16558	3.307635	34.63684
3.226715	3.174004	3.413645	34.64492
3.30747	3.298683	3.380429	35.02153
3.275094	3.463595	3.420125	35.39817
3.293117	3.377196	3.383685	35.16896
3.109955	3.240657	3.475832	34.67133
3.280084	3.3003	3.355925	34.91159
3.28359	3.36884	3.398367	35.16196
3.383358	3.448701	3.375432	35.50462
3.21195	3.421474	3.411172	35.1484
3.357663	3.408063	3.447595	35.51737
3.249935	3.2447	3.35376	34.71934
3.44382	3.27523	3.340187	35.18041
3.153096	3.259931	3.313979	34.45388
3.271712	3.291472	3.391052	34.95079
3.119028	3.41925	3.397958	34.91143
3.269319	3.452947	3.392927	35.30278
3.313326	3.440479	3.394667	35.37556
3.397916	3.355361	3.358671	35.29568
3.272042	3.34478	3.418675	35.1285
3.290849	3.491092	3.397889	35.44413
3.347888	3.447218	3.45832	35.60508
3.376305	3.254809	3.315377	34.93386
3.236943	3.152236	3.384981	34.557
3.15557	3.233243	3.385834	34.55806
3.236077	3.389193	3.415879	35.14086
3.238676	3.422552	3.348627	35.07242
3.176852	3.290461	3.419442	34.80322
3.241233	3.210936	3.365133	34.65134
3.204237	3.277319	3.457007	34.91652
3.184894	3.439805	3.434704	35.18078
3.417383	3.48934	3.390046	35.69986
3.309326	3.501201	3.403397	35.51869
3.386987	3.226032	3.333127	34.9331
3.309573	3.215452	3.398759	34.8842

3.277939	3.276578	3.374614	34.89589
3.218508	3.324629	3.406688	34.94115
3.316131	3.422755	3.392365	35.33789
3.18543	3.332043	3.427201	34.92988
3.299015	3.372075	3.390455	35.18546
3.282352	3.324764	3.360291	34.9796
3.276001	3.397752	3.362558	35.13028
3.226509	3.484218	3.405989	35.30611
3.3151	3.470739	3.419511	35.49994
3.265525	3.218282	3.422972	34.84701
3.32174	3.142127	3.309051	34.55428
3.260205	3.220371	3.431924	34.85952
3.335556	3.401458	3.370385	35.28574
3.251997	3.458204	3.405852	35.30466
3.25938	3.31324	3.367043	34.91892
3.119812	3.236276	3.386686	34.48836
3.378615	3.256023	3.385067	35.09396
3.245233	3.407861	3.485995	35.35504
3.333577	3.477748	3.447953	35.61787
3.300458	3.469054	3.433357	35.49451
3.322359	3.283789	3.366787	34.99168
3.40402	3.171511	3.306868	34.7937
3.245522	3.227785	3.366583	34.70074
3.179698	3.371603	3.407421	34.9606
3.189019	3.451397	3.384981	35.10641
3.297654	3.448432	3.371562	35.30815
3.327679	3.387642	3.415077	35.33603
3.259421	3.310207	3.420312	35.02887
3.18242	3.426057	3.452727	35.18471
3.257153	3.499651	3.362286	35.3113
3.320874	3.465752	3.337663	35.32267
3.29753	3.157358	3.467528	34.88121
3.217105	3.146643	3.338754	34.40029
3.262803	3.287024	3.461833	35.07637
3.256658	3.433066	3.329939	35.09387
3.412104	3.475726	3.398981	35.67808
3.133959	3.303603	3.39112	34.67623
3.223044	3.253731	3.374478	34.72559
3.195576	3.284867	3.421028	34.83541
3.323513	3.432931	3.361672	35.30917
3.20399	3.473435	3.420653	35.26535
3.286765	3.435762	3.404215	35.32804
3.184441	3.247261	3.412349	34.70984
3.496158	3.275769	3.321959	35.25619
3.326359	3.272534	3.383822	35.01307
3.342073	3.309264	3.389705	35.14062
3.234881	3.420059	3.441797	35.26242
3.368675	3.403952	3.383668	35.39266
3.239336	3.382386	3.339897	34.96694
3.407732	3.398628	3.311165	35.30788

3.168026	3.44149	3.542504	35.38331
3.237974	3.464943	3.378979	35.22997
3.22886	3.434144	3.438813	35.27353
3.37515	3.300098	3.376711	35.1645
3.26767	3.168546	3.379013	34.64681
3.262927	3.190314	3.424609	34.78375
3.23418	3.34296	3.383771	34.96539
3.252946	3.44924	3.448686	35.38081
3.227457	3.354013	3.330638	34.85867
3.243625	3.266334	3.42258	34.90335
3.168933	3.287967	3.383685	34.70226
3.202794	3.384003	3.445958	35.1225
3.297241	3.427607	3.317832	35.14421
3.273733	3.411904	3.412008	35.26441
3.314234	3.395999	3.452045	35.40575
3.250842	3.2323	3.323408	34.62783
3.361457	3.224078	3.33826	34.88422
3.377501	3.286821	3.303338	34.98015
3.353662	3.376186	3.402306	35.33987
3.182915	3.401121	3.424677	35.06993
3.333535	3.381038	3.363803	35.22227
3.278393	3.338108	3.378843	35.04069
3.266515	3.402806	3.352174	35.09788
3.209187	3.416217	3.411104	35.13071
3.217848	3.465684	3.527396	35.51214
3.167489	3.423361	3.37516	34.97654
3.379563	3.231289	3.368748	35.00626
3.314151	3.18499	3.406125	34.8437
3.392802	3.355226	3.446435	35.47613
3.235294	3.446275	3.424012	35.28176
3.285776	3.415948	3.364536	35.19577
3.277197	3.274893	3.309767	34.74877
3.298602	3.191459	3.329461	34.6562
3.158581	3.272399	3.440586	34.77001
3.221972	3.486374	3.397395	35.28211
3.230798	3.497427	3.518955	35.59142
3.341207	3.361898	3.383822	35.24097
3.400474	3.164906	3.407575	34.99173
3.269319	3.180407	3.389824	34.69999
3.250018	3.327325	3.440842	35.09064
3.315595	3.469391	3.340937	35.32625
3.371232	3.435492	3.405989	35.51604
3.320791	3.386766	3.357477	35.19309
3.274351	3.290528	3.392007	34.95659
3.271877	3.380566	3.396235	35.15732
3.362323	3.434279	3.331985	35.33207
3.290436	3.411635	3.445088	35.37268
3.293282	3.339523	3.371681	35.06068
3.143321	3.143138	3.396986	34.35862
3.293488	3.172859	3.331218	34.60818

3.389462	3.359404	3.436	35.45514
3.35259	3.49399	3.455865	35.71227
3.245192	3.39573	3.433203	35.21297
3.318482	3.327864	3.424217	35.20518
3.229643	3.289652	3.415128	34.90747
3.381708	3.386025	3.310585	35.22214
3.187905	3.426394	3.47781	35.2523
3.107604	3.460158	3.430014	35.04601
3.137052	3.329819	3.493429	34.96405
3.289859	3.258449	3.365764	34.86296
3.352384	3.170163	3.406961	34.89672
3.39705	3.256157	3.363582	35.08759
3.185595	3.368166	3.436341	35.02923
3.346692	3.444051	3.387573	35.44082
3.458544	3.436301	3.348764	35.58361
3.405629	3.294639	3.352634	35.16656
3.214631	3.399436	3.376524	35.0303
3.248492	3.459349	3.416663	35.32314
3.246223	3.499651	3.35376	35.26876
3.335474	3.40038	3.378348	35.30061
3.300252	3.223674	3.433016	34.95671
3.092179	3.153247	3.446384	34.37691
3.29122	3.242544	3.378451	34.8589
3.317698	3.407321	3.439632	35.41094
3.368304	3.438659	3.402902	35.50981
3.219539	3.36675	3.492901	35.22405
3.256122	3.271793	3.408529	34.91189
3.280868	3.245913	3.357562	34.79795
3.374408	3.374501	3.373949	35.31954
3.179863	3.46063	3.468875	35.29004
3.241728	3.444118	3.452437	35.35328
3.157632	3.335951	3.442155	34.91034
3.354363	3.198603	3.388255	34.92233
3.258143	3.249216	3.400532	34.84944
3.2793	3.312364	3.443826	35.12848
3.286724	3.406176	3.389023	35.23002
3.430251	3.396741	3.388272	35.52162
3.365623	3.36082	3.34653	35.21045
3.402536	3.340399	3.313996	35.17537
3.20201	3.372748	3.444679	35.09338
3.138454	3.488194	3.359609	35.02082
3.185018	3.421744	3.398077	35.06145
3.304913	3.263099	3.384026	34.94599
3.268742	3.2261	3.404079	34.82983
3.189555	3.259392	3.427201	34.78003
3.19735	3.438727	3.484153	35.3138
3.400061	3.46993	3.396576	35.63381
3.353209	3.369109	3.427406	35.37829
3.276372	3.304681	3.314882	34.8233
3.255627	3.199816	3.400686	34.73625

3.220735	3.326112	3.404625	34.94475
3.279919	3.466493	3.445361	35.47025
3.310604	3.444253	3.381588	35.34926
3.238181	3.308792	3.463418	35.0736
3.221931	3.198805	3.423876	34.71106
3.318069	3.215721	3.411241	34.93066
3.297489	3.370592	3.412554	35.22721
3.29456	3.481387	3.414191	35.46667
3.258101	3.44769	3.437057	35.36326
3.406	3.393371	3.371954	35.42553
3.339598	3.314857	3.342966	35.04523
3.375934	3.336288	3.35185	35.19099
3.236985	3.459417	3.388204	35.23589
3.227416	3.4115	3.397412	35.13032
3.140475	3.287698	3.43322	34.74776
3.284909	3.196986	3.278102	34.52602
3.245605	3.174409	3.460008	34.7885
3.283755	3.415274	3.425087	35.32229
3.26536	3.498775	3.512135	35.65503
3.386698	3.385688	3.363087	35.34713
3.411774	3.308994	3.427593	35.37531
3.370737	3.356911	3.35688	35.23572
3.262762	3.332514	3.421472	35.0875
3.210218	3.387508	3.368885	34.97785
3.266845	3.390675	3.351032	35.06957
3.228489	3.369648	3.396576	35.03931
3.284662	3.269434	3.391665	34.93226
3.170789	3.216462	3.403278	34.59279
3.236943	3.195705	3.460145	34.81643
3.221766	3.401593	3.475764	35.26764
3.360302	3.458338	3.40558	35.54121
3.342362	3.43967	3.381588	35.40868
3.389338	3.35563	3.399373	35.36652
3.25175	3.370053	3.382935	35.06123
3.417713	3.464808	3.288333	35.4245
3.269361	3.435492	3.462464	35.41677
3.285157	3.349969	3.330638	34.976
3.114409	3.186877	3.4279	34.45865
3.274681	3.185798	3.310995	34.55112
3.219539	3.24032	3.423671	34.79617
3.318564	3.500594	3.432333	35.60084
3.174336	3.422013	3.502569	35.26719
3.393256	3.337906	3.378229	35.29009
3.300871	3.33184	3.33635	34.98321
3.411073	3.283924	3.403278	35.26578
3.312708	3.335682	3.369038	35.08898
3.169469	3.382386	3.484426	35.13021
3.143816	3.416217	3.400328	34.96419
3.347806	3.288439	3.385254	35.09789
3.261937	3.225358	3.386567	34.77503

3.203124	3.213497	3.42258	34.69923
3.17446	3.377196	3.480896	35.12206
3.382533	3.419722	3.443366	35.588
3.510305	3.454632	3.355925	35.75255
3.376346	3.404491	3.413014	35.47479
3.356013	3.353878	3.414344	35.32256
3.298685	3.38097	3.405528	35.23716
3.307965	3.45261	3.41385	35.43231
3.365747	3.396336	3.280165	35.14326
3.341825	3.194694	3.388392	34.88667
3.075475	3.149001	3.345012	34.10941
3.268742	3.262088	3.515733	35.1527
3.296458	3.465347	3.393456	35.3904
3.33003	3.479972	3.431038	35.57799
3.239294	3.336895	3.352345	34.89459
3.283837	3.23257	3.45803	34.99497
3.309985	3.323955	3.344757	35.00429
3.464772	3.347678	3.406381	35.52942
3.21294	3.356507	3.405614	34.99633
3.269567	3.378544	3.385356	35.12406
3.161303	3.26849	3.375842	34.62583
3.389008	3.218417	3.336418	34.92807
3.222096	3.218754	3.350622	34.59485
3.208156	3.33238	3.398912	34.91845
3.157055	3.414061	3.468807	35.13818
3.411527	3.464808	3.359745	35.56714
3.398205	3.383329	3.395093	35.43713
3.518223	3.412174	3.35347	35.67164
3.277238	3.356641	3.472166	35.28278
3.275918	3.429898	3.321822	35.11131
3.274516	3.367492	3.365491	35.06727
3.289446	3.23203	3.349923	34.76964
3.219497	3.183574	3.410013	34.64212
3.166541	3.244296	3.390182	34.61573
3.223209	3.403615	3.471075	35.26496
3.349868	3.463663	3.423126	35.5684
3.322152	3.415476	3.416902	35.38881
3.242181	3.287563	3.409126	34.91719
3.337784	3.319238	3.435727	35.2537
3.303345	3.368974	3.42212	35.2574
3.37317	3.350239	3.353351	35.21873
3.305614	3.378679	3.374068	35.1785
3.291467	3.336693	3.341329	34.98415
3.153425	3.208982	3.425376	34.58679
3.321245	3.224415	3.281018	34.67184
3.207908	3.262021	3.432794	34.83814
3.234056	3.371064	3.316161	34.87873
3.199577	3.38589	3.479515	35.19298
3.356673	3.457934	3.34994	35.41071
3.383028	3.35435	3.348917	35.23959

3.387647	3.393304	3.38846	35.42135
3.250719	3.394651	3.425445	35.20573
3.363396	3.40166	3.359813	35.32394
3.244821	3.300502	3.352856	34.82821
3.263504	3.179396	3.356011	34.61112
3.116141	3.243622	3.371135	34.46239
3.210878	3.352598	3.41888	35.01229
3.231912	3.454901	3.391393	35.2219
3.262267	3.384542	3.435266	35.23036
3.342527	3.373759	3.434226	35.38002
3.375274	3.341141	3.371493	35.24311
3.352013	3.342489	3.455728	35.3794
3.293942	3.37012	3.360649	35.10491
3.30615	3.32719	3.409399	35.14434
3.283177	3.367087	3.373795	35.10349
3.342114	3.306366	3.32172	34.9857
3.165427	3.186674	3.429725	34.57376
3.26635	3.339658	3.387863	35.03747
3.191081	3.308387	3.513618	35.07949
3.245192	3.394247	3.392211	35.12009
3.255049	3.429359	3.440706	35.32448
3.33469	3.423968	3.412213	35.42454
3.277774	3.412174	3.398418	35.24411
3.442294	3.425046	3.405631	35.64782
3.289323	3.429022	3.365219	35.23361
3.358446	3.389125	3.422427	35.42263
3.211084	3.280419	3.358261	34.72233
3.231211	3.165311	3.288043	34.36107
3.154952	3.271051	3.375518	34.61683
3.269814	3.408871	3.360785	35.13719
3.231664	3.450723	3.446452	35.33262
3.265443	3.385553	3.409416	35.18298
3.253235	3.352261	3.484034	35.24666
3.403649	3.404963	3.358807	35.41699
3.343434	3.379825	3.404096	35.32938
3.248986	3.325371	3.436545	35.07471
3.240202	3.283924	3.372022	34.82376
3.323802	3.298076	3.452932	35.21447
3.274764	3.268558	3.308727	34.72733
3.244161	3.206016	3.379678	34.67879
3.112801	3.271927	3.413901	34.61051
3.176192	3.368166	3.497726	35.1429
3.290436	3.428685	3.426655	35.36966
3.345991	3.442636	3.349054	35.35196
3.429096	3.386834	3.408853	35.54244
3.227787	3.44331	3.441115	35.29626
3.357292	3.456788	3.378723	35.4725
3.358941	3.422418	3.324892	35.28323
3.331844	3.341276	3.366225	35.13691
3.239459	3.182698	3.412179	34.68859

3.271753	3.202175	3.369686	34.70888
3.105748	3.360955	3.399441	34.75815
3.27695	3.505042	3.397616	35.44365
3.195618	3.408534	3.451568	35.17272
3.187823	3.353609	3.446384	35.02423
3.250224	3.333391	3.416578	35.05129
3.385007	3.391282	3.4508	35.54748
3.311676	3.450453	3.384299	35.37109
3.338815	3.36082	3.411224	35.2933
3.180522	3.244633	3.408035	34.68609
3.349744	3.243892	3.372994	34.9779
3.320255	3.236546	3.400959	34.9585
3.257978	3.31014	3.332462	34.83346
3.099108	3.298144	3.459735	34.73813
3.206052	3.370322	3.384453	34.96521
3.3005	3.446477	3.479924	35.54707
3.386657	3.491698	3.30859	35.45969
3.347063	3.416352	3.470052	35.56143
3.293034	3.444523	3.384708	35.31825
3.285611	3.37942	3.457962	35.31984
3.32739	3.344982	3.325778	35.04683
3.317904	3.270175	3.367947	34.95471
3.158663	3.156212	3.384043	34.39246
3.195205	3.227111	3.406057	34.67555
3.253729	3.431044	3.413764	35.26636
3.313574	3.434548	3.334389	35.23131
3.280208	3.342152	3.419613	35.14266
3.2209	3.371401	3.469847	35.18678
3.179038	3.369244	3.464101	35.07795
3.436768	3.438525	3.37487	35.59794
3.31576	3.360078	3.443451	35.31174
3.344011	3.309331	3.360785	35.08177
3.262432	3.20204	3.463384	34.89311
3.352466	3.247531	3.326511	34.89016
3.247254	3.261077	3.367077	34.77841
3.333659	3.342489	3.335719	35.07682
3.133422	3.285743	3.469301	34.80697
3.203784	3.401256	3.374085	35.00522
3.250348	3.483678	3.41963	35.38689
3.372181	3.464471	3.351918	35.46325
3.338113	3.397145	3.418198	35.38646
3.341743	3.409815	3.386755	35.35334
3.245728	3.374501	3.466079	35.23961
3.392431	3.294033	3.34433	35.11821
3.31976	3.193009	3.380821	34.81817
3.234139	3.202445	3.370436	34.62886
3.20564	3.370322	3.410286	35.0208
3.257648	3.447825	3.395485	35.27165
3.333907	3.410354	3.314558	35.1795
3.315017	3.37504	3.427269	35.30745

3.294354	3.400447	3.406057	35.27143
3.299221	3.393506	3.525623	35.52837
3.275753	3.468582	3.361535	35.2824
3.316708	3.312431	3.35347	35.01284
3.402701	3.224482	3.388954	35.08616
3.215621	3.2354	3.413099	34.75372
3.327143	3.186877	3.398094	34.85868
3.215909	3.343567	3.411275	34.98691
3.285858	3.32746	3.420619	35.12509
3.256122	3.383801	3.407558	35.1547
3.263752	3.422418	3.450204	35.3491
3.187781	3.452745	3.371511	35.07719
3.413424	3.453351	3.378263	35.58673
3.25175	3.462382	3.45196	35.41409
3.32042	3.346667	3.350247	35.08878
3.257318	3.276847	3.362968	34.82592
3.332793	3.236478	3.385408	34.95176
3.323678	3.235939	3.315496	34.77777
3.370366	3.320721	3.390114	35.22844
3.146002	3.433538	3.414105	35.03697
3.286353	3.413117	3.38019	35.22508
3.158952	3.375983	3.478441	35.08012
3.303716	3.436772	3.389892	35.33599
3.252327	3.372816	3.435607	35.18372
3.3005	3.42572	3.454279	35.44559
3.198628	3.326112	3.459275	35.01592
3.317574	3.268962	3.350929	34.91412
3.317368	3.198805	3.411155	34.89195
3.307676	3.232839	3.333878	34.77619
3.302108	3.262964	3.444611	35.07205
3.227787	3.408467	3.36249	35.04813
3.317698	3.338647	3.357017	35.08009
3.335515	3.374838	3.315377	35.10714
3.312006	3.4177	3.44814	35.43979
3.213517	3.430842	3.429196	35.21173
3.305036	3.392562	3.412741	35.29217
3.349043	3.374568	3.371067	35.25792
3.347063	3.353272	3.304361	35.06114
3.236036	3.212958	3.420312	34.76507
3.295344	3.260673	3.366873	34.88224
3.223663	3.229132	3.429196	34.79281
3.354281	3.456047	3.321771	35.33975
3.207949	3.41615	3.441047	35.19334
3.219167	3.377331	3.343529	34.91972
3.17912	3.372951	3.437313	35.02766
3.285033	3.447218	3.333707	35.19511
3.20432	3.439199	3.471484	35.30236
3.348672	3.400515	3.390864	35.35714
3.153879	3.251238	3.426382	34.68239
3.271753	3.239376	3.348576	34.74407

3.428684	3.244431	3.371528	35.1485
3.3622	3.269906	3.395485	35.11121
3.249729	3.345858	3.38285	35.00372
3.204402	3.332919	3.350554	34.80567
3.199329	3.34168	3.346325	34.80449
3.359725	3.420328	3.397531	35.43922
3.28425	3.477343	3.417243	35.44196
3.203454	3.434144	3.442121	35.2252
3.29555	3.377062	3.382645	35.17171
3.35259	3.307309	3.395639	35.17232
3.315883	3.305692	3.321618	34.92664
3.316626	3.25946	3.435352	35.07588
3.257524	3.264784	3.40899	34.90063
3.235665	3.332986	3.352788	34.87907
3.421673	3.428955	3.365133	35.52271
3.268288	3.368368	3.419784	35.1743
3.275712	3.342758	3.364809	35.01431
3.121421	3.387979	3.409825	34.87423
3.279837	3.438794	3.376251	35.25836
3.324668	3.428281	3.434857	35.46157
3.266927	3.321664	3.431225	35.09421
3.206794	3.199142	3.431327	34.695
3.372222	3.213969	3.404966	35.03153
3.306851	3.245779	3.392501	34.93088
3.379976	3.326853	3.382202	35.24557
3.25043	3.372614	3.440501	35.18983
3.268371	3.422755	3.367333	35.17871
3.297612	3.394584	3.335753	35.11199
3.321162	3.451666	3.367725	35.35824
3.253647	3.430977	3.346325	35.11855
3.250842	3.400852	3.427337	35.2237
3.15458	3.269636	3.453801	34.78412
3.380429	3.254001	3.334458	34.98283
3.405629	3.290191	3.4028	35.26654
3.330442	3.322944	3.408649	35.18653
3.31345	3.287967	3.395945	35.0451
3.265236	3.390203	3.367248	35.10049
3.341537	3.386295	3.359336	35.24149
3.332876	3.37039	3.346786	35.16033
3.185307	3.393034	3.453341	35.12015
3.128102	3.416959	3.390029	34.90892
3.309944	3.377803	3.416544	35.27894
3.230138	3.306163	3.375569	34.85815
3.231499	3.211071	3.389023	34.68259
3.16716	3.247059	3.462242	34.78071
3.233933	3.288439	3.447049	34.984
3.306604	3.307916	3.39262	35.06649
3.383193	3.334941	3.385408	35.2773
3.304376	3.370322	3.419255	35.25633
3.214342	3.371468	3.384436	34.9858

3.221807	3.406984	3.409553	35.13472
3.330277	3.527619	3.294062	35.38318
3.203124	3.399773	3.347741	34.94293
3.357085	3.337097	3.352993	35.15404
3.241356	3.20723	3.427695	34.78032
3.364179	3.267884	3.327159	34.9617
3.314893	3.326651	3.44398	35.2379
3.490178	3.407187	3.346513	35.58419
3.301861	3.316475	3.475423	35.25591
3.274186	3.342152	3.342983	34.96191
3.250348	3.298952	3.400396	34.94086
3.3589	3.429764	3.315837	35.2794
3.235087	3.492709	3.361876	35.24697
3.14629	3.437783	3.418249	35.05595
3.170212	3.32719	3.431992	34.89647
3.306892	3.194492	3.457416	34.96078
3.271629	3.247127	3.382867	34.83574
3.229107	3.235467	3.430628	34.8217
3.265938	3.254136	3.390029	34.85428
3.19801	3.310544	3.386482	34.82133
3.380677	3.396067	3.360444	35.35088
3.376346	3.383734	3.38597	35.37026
3.222921	3.378275	3.308522	34.85344
3.202753	3.392293	3.4493	35.14785
3.326442	3.489946	3.364673	35.44682
3.225478	3.390675	3.359268	34.99712
3.338072	3.277791	3.341193	34.95696
3.164767	3.23358	3.421011	34.65584
3.303387	3.288641	3.365406	34.95779
3.279672	3.338984	3.388801	35.06718
3.443862	3.364122	3.394326	35.49329
3.291797	3.340467	3.379934	35.07755
3.298396	3.325236	3.393643	35.08865
3.217394	3.370255	3.372721	34.96421
3.326648	3.442434	3.387778	35.3939
3.191864	3.49217	3.380821	35.1927
3.213971	3.332717	3.409007	34.95398
3.183162	3.268625	3.422375	34.77569
3.242099	3.222326	3.459207	34.88387
3.381419	3.249418	3.396713	35.11112
3.320503	3.291404	3.431975	35.14684
3.267134	3.310679	3.416834	35.03916
3.317616	3.383329	3.409962	35.29341
3.316708	3.446814	3.368339	35.33923
3.355889	3.375646	3.304515	35.12971
3.17879	3.419452	3.356676	34.95229
3.164974	3.390945	3.375108	34.90004
3.30747	3.413791	3.41917	35.35797
3.263545	3.333997	3.373062	34.98659
3.268825	3.233378	3.369362	34.77001

3.243542	3.234794	3.391665	34.76659
3.326483	3.32126	3.389688	35.13273
3.298561	3.384677	3.336555	35.09416
3.397834	3.3559	3.379866	35.34303
3.252245	3.330964	3.371886	34.95267
3.309326	3.423159	3.359131	35.25122
3.293364	3.428753	3.404164	35.32703
3.31378	3.453351	3.429793	35.48151
3.185224	3.428079	3.328285	34.92313
3.230344	3.334132	3.447185	35.07637
3.121173	3.252114	3.493446	34.75944
3.244285	3.248137	3.45878	34.94416
3.209723	3.233311	3.378161	34.65985
3.373047	3.289854	3.389773	35.16606
3.268288	3.317283	3.434925	35.0957
3.391854	3.411635	3.365338	35.42007
3.323142	3.346532	3.414736	35.23547
3.330525	3.354822	3.343239	35.11338
3.174212	3.44176	3.412758	35.1137
3.219085	3.456856	3.369089	35.14935
3.192029	3.346061	3.423586	34.96706
3.279259	3.269636	3.386021	34.90855
3.302809	3.243487	3.394547	34.92151
3.230551	3.26721	3.399799	34.82685
3.359725	3.377736	3.444491	35.44877
3.31114	3.421272	3.406756	35.35521
3.395071	3.315127	3.429605	35.3566
3.353209	3.309938	3.353419	35.0871
3.357374	3.389934	3.417192	35.41061
3.27167	3.46555	3.404915	35.3617
3.288168	3.434279	3.398077	35.31444
3.214301	3.381644	3.404915	35.05275
3.261648	3.345117	3.33647	34.92674
3.173717	3.225561	3.463759	34.75136
3.234675	3.167332	3.407217	34.63368
3.184028	3.246453	3.465414	34.82321
3.312584	3.321462	3.357153	35.03163
3.266639	3.368637	3.451346	35.2403
3.475949	3.374905	3.394206	35.58678
3.235541	3.309466	3.405051	34.94166
3.35424	3.374433	3.299246	35.11193
3.220281	3.441153	3.427559	35.24549
3.337619	3.423294	3.345984	35.28464
3.216033	3.337704	3.418198	34.9895
3.192318	3.266401	3.363718	34.66257
3.2395	3.304748	3.439546	35.01543
3.352961	3.418441	3.408171	35.44357
3.359395	3.373827	3.383089	35.30522
3.357003	3.278397	3.400464	35.1293
3.241439	3.288911	3.381707	34.85856

3.390905	3.423698	3.383072	35.48316
3.298726	3.478691	3.363565	35.35918
3.239253	3.381847	3.438336	35.18085
3.182461	3.284463	3.38771	34.73298
3.201185	3.272736	3.398486	34.77185
3.227127	3.318564	3.381469	34.89158
3.255214	3.265592	3.411752	34.90339
3.246306	3.262897	3.491929	35.05335
3.069619	3.317283	3.46555	34.72821
3.463576	3.416959	3.417038	35.70162
3.408434	3.395191	3.369089	35.42857
3.408805	3.385688	3.353334	35.37414
3.203866	3.291809	3.400277	34.82333
3.279465	3.412106	3.36822	35.18163
3.279589	3.418239	3.292562	35.02986
3.365664	3.323821	3.341807	35.1193
3.12538	3.226976	3.394956	34.49828
3.151281	3.277049	3.41385	34.70574
3.258143	3.4208	3.398367	35.21994
3.372304	3.472019	3.397889	35.58055
3.252616	3.371131	3.425837	35.1593
3.313574	3.271186	3.405869	35.03038
3.215208	3.342556	3.46526	35.10122
3.386327	3.526137	3.349787	35.62437
3.314357	3.427203	3.415845	35.39509
3.284662	3.326382	3.34728	34.95973
3.137877	3.194896	3.347246	34.35113
3.24181	3.324427	3.35804	34.88528
3.189019	3.357854	3.428991	34.99809
3.296746	3.280486	3.417038	35.03834
3.201639	3.2848	3.475338	34.96728
3.345207	3.403615	3.47058	35.53067
3.359684	3.456249	3.393251	35.50832
3.484528	3.458069	3.360512	35.71372
3.331968	3.334064	3.431924	35.26509
3.220199	3.342084	3.388801	34.9439
3.264783	3.372479	3.51014	35.37322
3.237974	3.375444	3.292323	34.84474
3.380759	3.239039	3.32675	34.93398
3.224075	3.175487	3.345166	34.49263
3.125133	3.338917	3.454756	34.87331
3.173758	3.45571	3.465652	35.25889
3.36088	3.441692	3.370658	35.4297
3.273898	3.304479	3.431089	35.07157
3.311718	3.335412	3.38007	35.11036
3.321657	3.48442	3.487444	35.69276
3.361457	3.493316	3.373983	35.55113
3.276331	3.312498	3.39557	35.01676
3.339475	3.275634	3.368544	35.01512
3.088178	3.258179	3.384299	34.46186

3.301531	3.296661	3.340715	34.91727
3.148105	3.27496	3.410405	34.6867
3.282435	3.302929	3.363036	34.93803
3.16951	3.353002	3.509935	35.12183
3.336216	3.422485	3.458985	35.52692
3.340712	3.391956	3.389278	35.31755
3.431324	3.346195	3.374341	35.38297
3.247584	3.280621	3.415282	34.92729
3.364344	3.378544	3.374836	35.30832
3.238841	3.418374	3.363582	35.09635
3.237356	3.258112	3.310227	34.62596
3.249523	3.219023	3.305282	34.55627
3.195246	3.266401	3.404556	34.75828
3.244408	3.449577	3.373659	35.1988
3.245769	3.432324	3.413986	35.25224
3.303716	3.374096	3.433544	35.29439
3.332917	3.294707	3.326955	34.95154
3.287219	3.414128	3.444457	35.36972
3.229478	3.509019	3.410763	35.37728
3.29489	3.399436	3.422017	35.3053
3.170253	3.256831	3.370453	34.60812
3.343104	3.262829	3.375484	35.01023
3.128556	3.291606	3.417055	34.69489
3.186132	3.30677	3.38713	34.78852
3.179945	3.33211	3.453051	34.97457
3.257978	3.277386	3.335123	34.76765
3.249193	3.347948	3.409433	35.06525
3.361787	3.378544	3.396662	35.35045
3.253606	3.371198	3.412537	35.13253
3.352095	3.392764	3.372022	35.30647
3.324049	3.36675	3.407762	35.26641
3.437304	3.405839	3.381435	35.54199
3.327473	3.335008	3.370709	35.12345
3.261854	3.245779	3.327978	34.69138
3.13037	3.270243	3.356556	34.51984
3.291261	3.387171	3.390523	35.20167
3.232035	3.449577	3.432044	35.29942
3.264865	3.394112	3.405187	35.19119
3.224653	3.283182	3.432794	34.92104
3.374284	3.321731	3.382816	35.22326
3.330772	3.494327	3.365406	35.46748
3.334195	3.453351	3.458849	35.5897
3.230179	3.293156	3.377155	34.83326
3.190668	3.190179	3.392808	34.55589
3.327761	3.269434	3.359285	34.9557
3.277568	3.36082	3.406415	35.14885
3.147074	3.30185	3.342233	34.59417
3.147115	3.262155	3.39037	34.61272
3.287302	3.325371	3.413918	35.10902
3.302974	3.366481	3.363667	35.1233

3.356261	3.391888	3.468892	35.52551
3.200979	3.387103	3.397327	35.01896
3.297489	3.398762	3.433288	35.33415
3.365375	3.404558	3.351628	35.31671
3.453183	3.359	3.383617	35.47906
3.238882	3.232165	3.374938	34.71407
3.169882	3.278397	3.417788	34.75798
3.184152	3.382655	3.327483	34.8197
3.295798	3.430707	3.345217	35.20772
3.29089	3.448566	3.404369	35.36539
3.229808	3.36547	3.459548	35.17077
3.17215	3.29255	3.450613	34.86567
3.36488	3.455036	3.346377	35.41453
3.339928	3.434346	3.463555	35.57097
3.404763	3.394314	3.317883	35.30665
3.17182	3.159919	3.464118	34.60444
3.194793	3.163491	3.321004	34.34953
3.381996	3.325438	3.346394	35.16859
3.278888	3.423563	3.367947	35.20482
3.153467	3.285474	3.438608	34.78309
3.18477	3.260605	3.437227	34.79414
3.246471	3.327123	3.434294	35.06812
3.393462	3.398358	3.356198	35.37456
3.351023	3.361898	3.461918	35.43322
3.158787	3.402402	3.476395	35.13306
3.287879	3.356709	3.388323	35.12284
3.315017	3.369244	3.337936	35.09941
3.458668	3.306231	3.354715	35.31245
3.2355	3.208982	3.383498	34.67469
3.198505	3.275971	3.374426	34.72045
3.230015	3.365942	3.434687	35.11788
3.413548	3.431583	3.313944	35.39874
3.21628	3.358191	3.437142	35.07627
3.275176	3.340332	3.369822	35.01879
3.121833	3.328134	3.489695	34.91892
3.316626	3.489677	3.398299	35.4983
3.416311	3.391214	3.464169	35.64502
3.328463	3.281902	3.345984	34.95541
3.186461	3.162547	3.456257	34.62502
3.253977	3.211677	3.397889	34.75246
3.283095	3.405637	3.399731	35.24433
3.301036	3.363785	3.388528	35.16754
3.15821	3.29875	3.416288	34.77369
3.099644	3.304276	3.445207	34.72094
3.249358	3.367694	3.369839	35.02221
3.28891	3.421609	3.399185	35.29078
3.291426	3.417093	3.367111	35.21626
3.236242	3.411702	3.461918	35.29112
3.260122	3.315733	3.372517	34.93797
3.377583	3.269366	3.373199	35.09493

3.444233	3.248003	3.265825	34.95916
3.25043	3.27368	3.361433	34.80057
3.231211	3.394449	3.389381	35.08376
3.269979	3.414533	3.402749	35.2417
3.331762	3.418441	3.377086	35.32923
3.290766	3.357113	3.325642	34.99297
3.274104	3.36547	3.41917	35.17933
3.107068	3.444523	3.481749	35.12378
3.305242	3.498977	3.420329	35.54192
3.301902	3.289045	3.378996	34.98514
3.35725	3.155202	3.412741	34.88728
3.223828	3.230076	3.402698	34.73728
3.293117	3.366885	3.460775	35.31499
3.32042	3.402873	3.393183	35.30559
3.320503	3.277454	3.377479	34.99715
3.134247	3.263099	3.363786	34.52851
3.268825	3.277049	3.423535	34.98397
3.220116	3.406243	3.430407	35.17501
3.281486	3.422283	3.464049	35.41786
3.286477	3.469526	3.410917	35.4159
3.426581	3.346398	3.392143	35.41197
3.314316	3.260807	3.396338	34.98846
3.369706	3.260807	3.317969	34.93821
3.296788	3.246857	3.407302	34.9436
3.288374	3.440547	3.299604	35.11325
3.237851	3.430909	3.410354	35.22388
3.161385	3.421878	3.323698	34.84741
3.295921	3.32591	3.35983	35.01077
3.19834	3.34168	3.452574	35.03467
3.260947	3.459754	3.404693	35.32509
3.311553	3.448903	3.401283	35.40457
3.226138	3.358663	3.42657	35.07574
3.190297	3.171241	3.372244	34.4687
3.366242	3.243824	3.321703	34.90166
3.280414	3.35745	3.416015	35.1687
3.27398	3.504907	3.437568	35.52423
3.281899	3.351519	3.349378	35.01325
3.285281	3.227515	3.369089	34.79257
3.225684	3.223134	3.350196	34.61135
3.326194	3.361089	3.472217	35.39967
3.177553	3.485094	3.498903	35.40416
3.230551	3.457867	3.434107	35.31881
3.286972	3.313981	3.476992	35.22132
3.384512	3.215789	3.299996	34.83284
3.362365	3.256831	3.356215	34.9971
3.31477	3.292415	3.344075	34.94428
3.201598	3.426394	3.475644	35.27751
3.38212	3.459349	3.341432	35.45085
3.268701	3.364796	3.344484	35.00272
3.342815	3.353474	3.343665	35.13825

3.156519	3.347341	3.433084	34.91298
3.213765	3.349767	3.325505	34.80821
3.298272	3.445803	3.446179	35.46693
3.322235	3.381442	3.392143	35.26042
3.237108	3.1643	3.422546	34.66589
3.258514	3.208173	3.373147	34.70062
3.280991	3.359068	3.420602	35.18353
3.413259	3.441827	3.359199	35.51948
3.284291	3.388586	3.35347	35.10849
3.217064	3.212554	3.409604	34.69928
3.212404	3.227785	3.377615	34.65244
3.254389	3.388923	3.39823	35.14172
3.238758	3.444253	3.428173	35.29401
3.228818	3.467639	3.44843	35.36772
3.174047	3.294639	3.491332	34.96344
3.264618	3.205747	3.410695	34.79077
3.456482	3.247666	3.346428	35.16147
3.322441	3.337165	3.395297	35.17094
3.268907	3.40038	3.370402	35.13767
3.298355	3.406311	3.397548	35.2744
3.365788	3.358124	3.378024	35.27379
3.318977	3.384745	3.33942	35.14521
3.342279	3.395999	3.328524	35.19696
3.134041	3.415611	3.428173	35.00238
3.188689	3.485363	3.462549	35.3496
3.202876	3.409208	3.395366	35.06717
3.25608	3.16066	3.442957	34.74405
3.174831	3.168209	3.384794	34.45569
3.357869	3.24652	3.439342	35.1465
3.232283	3.499583	3.500318	35.55862
3.416352	3.467706	3.345439	35.55275
3.302644	3.323821	3.432811	35.1805
3.327143	3.185933	3.325591	34.69806
3.202959	3.290528	3.418675	34.85878
3.221353	3.463932	3.382458	35.19902
3.19372	3.557205	3.445088	35.47952
3.33568	3.382116	3.344194	35.18644
3.21991	3.185259	3.435198	34.70178
3.274227	3.213362	3.324227	34.63934
3.292911	3.28635	3.387317	34.97778
3.292746	3.440883	3.385339	35.31104
3.324627	3.436301	3.373813	35.34553
3.320008	3.353406	3.297814	34.98795
3.306232	3.243016	3.361399	34.85547
3.26037	3.370322	3.36099	35.03268
3.245316	3.468852	3.367947	35.23044
3.258555	3.437649	3.441268	35.3515
3.257771	3.390675	3.406756	35.17159
3.264576	3.132018	3.44207	34.69806
3.322895	3.154865	3.360785	34.69779

3.251172	3.326786	3.45682	35.12693
3.309738	3.451464	3.382236	35.36455
3.287054	3.398628	3.440092	35.32592
3.306356	3.304546	3.397395	35.06902
3.249935	3.208038	3.365679	34.66523
3.435077	3.364055	3.355465	35.38895
3.101459	3.399706	3.456274	34.9578
3.23451	3.449847	3.348968	35.12375
3.12802	3.372209	3.425206	34.88781
3.331844	3.264919	3.371391	34.98123
3.235005	3.207499	3.364315	34.62842
3.306274	3.266132	3.416152	35.02585
3.215868	3.332447	3.394956	34.92682
3.352095	3.408467	3.365952	35.32754
3.367603	3.370659	3.342011	35.22642
3.416888	3.373018	3.361569	35.38213
3.301696	3.352598	3.334867	35.02717
3.209269	3.396538	3.433203	35.13618
3.202381	3.447892	3.306493	34.95633
3.327473	3.413724	3.368168	35.29004
3.202835	3.304816	3.454705	34.96855
3.175408	3.184922	3.402101	34.53135
3.237768	3.21781	3.369908	34.66924
3.312708	3.393236	3.360239	35.1956
3.283878	3.361359	3.446657	35.25183
3.235294	3.338512	3.380224	34.95034
3.273155	3.268086	3.409757	34.94371
3.272825	3.280621	3.297336	34.72456
3.351724	3.367896	3.389892	35.29036
3.228736	3.409815	3.374767	35.08
3.142661	3.424979	3.43825	35.06375
3.189142	3.368435	3.434158	35.0328
3.326978	3.27058	3.349514	34.93513
3.243171	3.242274	3.361297	34.71572
3.289859	3.31479	3.400549	35.06224
3.210795	3.38589	3.467323	35.19085
3.397628	3.407861	3.333844	35.35557
3.409588	3.3559	3.3009	35.19605
3.430664	3.326921	3.332752	35.24842
3.264453	3.386632	3.454722	35.28225
3.218631	3.45881	3.348696	35.10803
3.145342	3.421272	3.481016	35.15503
3.33535	3.404626	3.341824	35.22976
3.244079	3.24524	3.431498	34.87771
3.137464	3.231828	3.436136	34.62538
3.193019	3.364661	3.468142	35.10734
3.410166	3.475659	3.352106	35.57119
3.337949	3.389058	3.416493	35.36468
3.259215	3.244431	3.338362	34.70537
3.224694	3.153584	3.406807	34.58089

3.256452	3.357652	3.340784	34.95222
3.382244	3.460495	3.35347	35.47995
3.287879	3.483409	3.403192	35.43243
3.196442	3.313509	3.420056	34.89781
3.233603	3.152506	3.396031	34.57445
3.309078	3.182429	3.372431	34.75333
3.235747	3.409006	3.420875	35.19439
3.326194	3.524452	3.444338	35.69595
3.214961	3.461843	3.38418	35.18423
3.380182	3.37194	3.292545	35.14855
3.32574	3.289719	3.373216	35.02611
3.4093	3.365739	3.399322	35.43217
3.221395	3.423024	3.431975	35.21793
3.220652	3.403076	3.361126	35.01775
3.195205	3.262021	3.410763	34.76219
3.311058	3.20669	3.35792	34.77898
3.218961	3.286417	3.423194	34.89467
3.298066	3.447353	3.399305	35.36736
3.221931	3.529439	3.458644	35.51014
3.297819	3.367155	3.423859	35.24513
3.394658	3.263975	3.339607	35.04702
3.383811	3.223943	3.375228	35.01366
3.252451	3.308118	3.442564	35.05772
3.261772	3.441827	3.424268	35.3305
3.184812	3.405569	3.375569	34.97641
3.328628	3.288237	3.37047	35.02318
3.308212	3.20541	3.383907	34.82678
3.148146	3.18937	3.429878	34.5422
3.113749	3.322742	3.43588	34.77177
3.2793	3.47923	3.415879	35.43228
3.349827	3.468178	3.442428	35.6204
3.444192	3.383599	3.364536	35.47146
3.252575	3.289315	3.336231	34.78434
3.337454	3.376253	3.365884	35.22492
3.392225	3.506323	3.360512	35.61739
3.309656	3.429629	3.403124	35.3623
3.10649	3.230817	3.382662	34.43849
3.17512	3.130266	3.311591	34.21326
3.24346	3.191122	3.398827	34.68657
3.276991	3.437446	3.378195	35.25344
3.346692	3.515084	3.481578	35.80173
3.130453	3.339658	3.437722	34.84932
3.266886	3.33676	3.434925	35.13522
3.314481	3.344241	3.390114	35.15767
3.434417	3.361224	3.369294	35.41156
3.23253	3.351182	3.382014	34.97592
3.254266	3.347678	3.375637	35.00184
3.233561	3.286148	3.410371	34.89797
3.372717	3.289248	3.304344	34.9772
3.187987	3.191999	3.384589	34.53604

3.262886	3.274623	3.364877	34.83741
3.095025	3.3683	3.473257	34.91219
3.293942	3.426461	3.36498	35.23759
3.401505	3.374029	3.394718	35.42318
3.388018	3.377062	3.405921	35.42482
3.203742	3.442029	3.4471	35.25397
3.339722	3.51286	3.37579	35.55029
3.27233	3.392697	3.39262	35.17694
3.3768	3.290124	3.259243	34.88941
3.160684	3.205882	3.432606	34.61169
3.053163	3.222663	3.388392	34.31657
3.152312	3.338041	3.356625	34.71624
3.269155	3.488126	3.430134	35.46072
3.268412	3.449308	3.427542	35.36854
3.238181	3.331167	3.451789	35.09709
3.195535	3.277117	3.412844	34.80047
3.372057	3.282104	3.430628	35.23629
3.432685	3.382386	3.324158	35.35534
3.253936	3.384138	3.412349	35.16114
3.280002	3.36365	3.353487	35.04462
3.268288	3.250698	3.394394	34.86145
3.34562	3.245779	3.289782	34.79103
3.198505	3.241398	3.337288	34.56362
3.233933	3.378544	3.428906	35.14137
3.151033	3.450656	3.433084	35.12691
3.343351	3.404356	3.386908	35.34526
3.345166	3.359337	3.381707	35.2394
3.360344	3.329549	3.403909	35.256
3.251585	3.441355	3.369857	35.1882
3.377872	3.392697	3.445276	35.52289
3.315347	3.369109	3.359609	35.14724
3.34298	3.219495	3.359302	34.87981
3.107563	3.184181	3.40679	34.39161
3.252863	3.375107	3.377751	35.06339
3.155075	3.459215	3.378451	35.035
3.266927	3.483948	3.449249	35.48851
3.244285	3.411095	3.425308	35.22732
3.334484	3.318564	3.406483	35.18106
3.213682	3.287428	3.421983	34.88269
3.355601	3.31789	3.396986	35.20499
3.274557	3.390473	3.45907	35.32226
3.364963	3.391282	3.313569	35.20354
3.263133	3.184855	3.357562	34.62564
3.181883	3.21936	3.373454	34.55817
3.264576	3.253259	3.362644	34.7895
3.187245	3.417296	3.310517	34.86512
3.18642	3.459417	3.452301	35.26548
3.278764	3.45261	3.424353	35.39142
3.263215	3.323821	3.478304	35.19376
3.261689	3.360011	3.34595	34.98013

3.369706	3.398897	3.427695	35.48015
3.28759	3.458136	3.463708	35.50887
3.341124	3.401795	3.345694	35.24466
3.26569	3.16275	3.385476	34.64394
3.251172	3.134714	3.303543	34.3717
3.255091	3.256494	3.402596	34.8632
3.314151	3.533213	3.379525	35.54704
3.190256	3.434009	3.446913	35.20653
3.291797	3.377803	3.359472	35.11445
3.200649	3.306635	3.488399	35.04143
3.348342	3.33986	3.348781	35.13175
3.188854	3.324899	3.475781	35.02798
3.273939	3.39108	3.411855	35.21898
3.27897	3.333795	3.431038	35.14666
3.363849	3.261953	3.322931	34.93876
3.348383	3.163558	3.38036	34.81535
3.275465	3.201097	3.28939	34.53905
3.086982	3.324832	3.389295	34.61593
3.182708	3.443377	3.364997	35.03137
3.256122	3.475052	3.372159	35.27684
3.33102	3.429898	3.376336	35.35103
3.293447	3.34478	3.355994	35.03823
3.288456	3.369514	3.456393	35.30097
3.356137	3.467369	3.32143	35.36782
3.401711	3.433538	3.42686	35.62406
3.327225	3.165715	3.382543	34.77857
3.197515	3.05	3.375449	34.22636
3.256823	3.201771	3.372858	34.68228
3.188854	3.373894	3.369703	34.90315
3.377913	3.495338	3.417925	35.68763
3.109955	3.400515	3.431191	34.92329
3.161014	3.342826	3.43663	34.92069
3.243996	3.373557	3.386038	35.05873
3.339103	3.33029	3.468483	35.35239
3.249481	3.353946	3.409757	35.0797
3.26569	3.334199	3.433544	35.12399
3.254389	3.329077	3.397156	35.0085
3.394493	3.226032	3.319844	34.92047
3.385213	3.227245	3.283559	34.82348
3.245357	3.262964	3.387386	34.8228
3.147486	3.339591	3.367827	34.73357
3.233396	3.417902	3.374205	35.10664
3.256946	3.450521	3.289902	35.04512
3.407732	3.413454	3.388937	35.51038
3.214837	3.413387	3.496089	35.32273
3.260246	3.451936	3.42952	35.36075
3.275918	3.42855	3.43397	35.35362
3.360385	3.344308	3.319077	35.10285
3.293447	3.172117	3.432794	34.8286
3.24181	3.210195	3.330587	34.57544

3.212734	3.317486	3.466488	35.04367
3.283755	3.486239	3.303134	35.21079
3.315842	3.420059	3.381861	35.3084
3.245151	3.336086	3.363769	34.93061
3.163613	3.319575	3.475303	34.9601
3.287137	3.371805	3.429997	35.24537
3.338732	3.361359	3.450272	35.37969
3.360715	3.397617	3.39598	35.38833
3.306232	3.300165	3.387147	35.03676
3.273939	3.187955	3.404915	34.7596
3.308583	3.250901	3.356198	34.86648
3.285858	3.290259	3.355124	34.90051
3.361086	3.372546	3.389023	35.3191
3.169428	3.440277	3.408666	35.09105
3.276496	3.445736	3.390506	35.29741
3.288828	3.380634	3.378621	35.15602
3.320626	3.417363	3.362917	35.27154
3.166335	3.37322	3.447595	35.02277
3.322276	3.378881	3.392484	35.25565
3.22622	3.328201	3.398094	34.94703
3.425426	3.197592	3.329871	34.94785
3.303964	3.227313	3.46185	35.03584
3.32706	3.359741	3.34578	35.12212
3.288003	3.4301	3.427269	35.36878
3.239171	3.397886	3.365884	35.0573
3.294189	3.392495	3.384981	35.20759
3.331597	3.39883	3.362064	35.25313
3.180316	3.402671	3.488535	35.20728
3.27365	3.405569	3.375927	35.17147
3.269113	3.3034	3.457774	35.11711
3.297324	3.280015	3.398026	34.997
3.367396	3.277117	3.357852	35.05604
3.287879	3.230143	3.447936	34.97643
3.281156	3.270647	3.344723	34.82459
3.256946	3.270445	3.429196	34.95594
3.33898	3.390473	3.339351	35.20134
3.257029	3.432324	3.392092	35.22898
3.241356	3.423968	3.35035	35.08515
3.27996	3.413656	3.38846	35.23036
3.354776	3.469121	3.317542	35.36018
3.181512	3.416083	3.391273	35.02653
3.350198	3.374298	3.428241	35.38488
3.242016	3.283991	3.3895	34.8661
3.387441	3.151697	3.346053	34.7998
3.32372	3.176565	3.409825	34.8543
3.330112	3.376253	3.418795	35.32458
3.301036	3.431179	3.421966	35.38804
3.24977	3.353339	3.308045	34.85658
3.199371	3.304411	3.456751	34.96456
3.33733	3.435896	3.37562	35.37638

3.241893	3.521756	3.492832	35.61176
3.202258	3.385958	3.433544	35.09846
3.263834	3.290191	3.415674	34.98461
3.328091	3.28116	3.475457	35.23612
3.295303	3.287158	3.300951	34.79591
3.32207	3.206623	3.388255	34.86925
3.210589	3.247935	3.384589	34.70779
3.269484	3.289854	3.369379	34.89499
3.28458	3.334738	3.31154	34.89967
3.317533	3.392899	3.327108	35.13297
3.248492	3.443579	3.441541	35.34306
3.281734	3.435896	3.41726	35.34586
3.355931	3.42545	3.398077	35.44332
3.26767	3.423833	3.321856	35.08009
3.349332	3.35617	3.431634	35.35077
3.213888	3.312094	3.415964	34.92392
3.29489	3.226774	3.403619	34.88748
3.300211	3.302187	3.377444	35.00679
3.43285	3.407052	3.3858	35.54444
3.223993	3.384677	3.445207	35.16869
3.245522	3.380499	3.34653	34.99085
3.218136	3.299626	3.386976	34.84255
3.336794	3.368974	3.314882	35.09603
3.200649	3.419789	3.453	35.21147
3.216775	3.379892	3.444321	35.14051
3.162004	3.272062	3.392348	34.67127
3.371314	3.242544	3.42449	35.13473
3.303881	3.263031	3.34624	34.86095
3.369541	3.260471	3.399918	35.11632
3.22424	3.32901	3.422904	34.99873
3.292251	3.346061	3.361126	35.04964
3.366324	3.387036	3.299263	35.16595
3.34962	3.421474	3.316587	35.24262
3.268165	3.420868	3.373864	35.18842
3.215621	3.396201	3.392024	35.05928
3.284497	3.352396	3.375108	35.07712
3.259132	3.352193	3.326051	34.91393
3.302026	3.242207	3.425973	34.98572
3.207207	3.232098	3.384981	34.66661
3.27365	3.24989	3.349275	34.77274
3.296004	3.370794	3.400464	35.19796
3.379934	3.424102	3.361535	35.41295
3.274021	3.417228	3.414941	35.28309
3.164809	3.38771	3.413901	34.97744
3.237397	3.349969	3.392825	35.00755
3.310439	3.46865	3.394735	35.431
3.196484	3.392697	3.419562	35.06999
3.262762	3.269434	3.413713	34.93258
3.168479	3.201905	3.450067	34.65823
3.29023	3.280352	3.425564	35.04244

3.364922	3.270782	3.374904	35.07407
3.426416	3.293965	3.377956	35.26592
3.26037	3.340332	3.42154	35.09951
3.29753	3.349026	3.370402	35.08796
3.272083	3.472963	3.355312	35.27034
3.442707	3.499853	3.277778	35.53272
3.243171	3.450656	3.415128	35.28914
3.125009	3.433403	3.400072	34.96008
3.220776	3.307174	3.42345	34.94459
3.31576	3.252518	3.328097	34.82426
3.315182	3.185664	3.393729	34.82032
3.300541	3.245172	3.378979	34.88619
3.214713	3.315194	3.45878	35.02613
3.280538	3.405637	3.34196	35.1124
3.408392	3.411028	3.370112	35.46535
3.295179	3.404963	3.405597	35.28211
3.191534	3.343904	3.397071	34.90328
3.248698	3.415072	3.413901	35.22072
3.309119	3.510703	3.410507	35.55457
3.261978	3.396741	3.392893	35.16374
3.240037	3.270445	3.432862	34.92697
3.112388	3.204601	3.443246	34.52655
3.357127	3.232502	3.36336	34.94807
3.241315	3.243016	3.395076	34.78715
3.420848	3.353743	3.389415	35.40953
3.249234	3.341141	3.446367	35.13122
3.333453	3.367627	3.359404	35.18314
3.301489	3.414263	3.336367	35.16485
3.380264	3.481522	3.295154	35.39407
3.194751	3.386632	3.408564	35.02889
3.252822	3.402199	3.322862	35.00251
3.144352	3.288102	3.42495	34.73904
3.338526	3.243622	3.326324	34.85072
3.385378	3.282171	3.438932	35.28373
3.299345	3.322877	3.386789	35.07058
3.259669	3.324225	3.449504	35.12391
3.324916	3.411567	3.430134	35.41524
3.302273	3.349026	3.401845	35.16709
3.376016	3.361224	3.373591	35.29324
3.126988	3.420126	3.387624	34.90816
3.16918	3.377062	3.423313	34.98429
3.267958	3.458675	3.405784	35.34045
3.192236	3.320114	3.408001	34.87669
3.309779	3.242611	3.371835	34.88516
3.255173	3.224415	3.461713	34.92251
3.288209	3.225965	3.364536	34.78563
3.322565	3.261481	3.383276	34.97941
3.436355	3.376522	3.385714	35.48516
3.245027	3.343297	3.388954	35.00118
3.277651	3.400043	3.324772	35.05626

3.305985	3.418778	3.404829	35.33427
3.381749	3.443916	3.344194	35.42233
3.254059	3.368166	3.32658	34.93892
3.303634	3.325573	3.369243	35.04748
3.141465	3.241061	3.437517	34.65734
3.254224	3.285811	3.411121	34.94406
3.231582	3.351519	3.404011	35.02269
3.387977	3.335682	3.407916	35.3386
3.274104	3.3621	3.423722	35.18192
3.3768	3.3652	3.483079	35.54308
3.290684	3.352126	3.390915	35.12462
3.313409	3.371131	3.38077	35.19369
3.183574	3.386025	3.368697	34.91594
3.261772	3.433605	3.383276	35.22287
3.091684	3.285878	3.375859	34.51164
3.218631	3.263908	3.379798	34.74982
3.305902	3.250294	3.400277	34.95569
3.263999	3.269434	3.385322	34.8732
3.331267	3.331369	3.420602	35.2329
3.298644	3.385621	3.401641	35.23873
3.340423	3.36884	3.377632	35.2409
3.3987	3.336154	3.338737	35.2118
3.29155	3.378342	3.375893	35.151
3.251255	3.442838	3.31686	35.07482
3.286972	3.433807	3.377001	35.2647
3.272578	3.382521	3.28303	34.91557
3.270144	3.265592	3.38655	34.88093
3.216775	3.278128	3.358398	34.73007
3.171449	3.298278	3.487171	34.95662
3.219126	3.370255	3.416902	35.06461
3.452729	3.39236	3.368237	35.51738
3.289611	3.357787	3.349616	35.04434
3.358075	3.364122	3.377496	35.26888
3.279837	3.349093	3.462003	35.24973
3.323472	3.451397	3.375228	35.37911
3.245646	3.375849	3.42258	35.14726
3.315925	3.337973	3.358313	35.07757
3.155653	3.211004	3.44814	34.64586
3.188771	3.287091	3.363309	34.69916
3.176151	3.237354	3.457706	34.76923
3.309326	3.344039	3.378996	35.12164
3.312419	3.328201	3.465908	35.28383
3.376965	3.364392	3.394684	35.34837
3.313904	3.336423	3.420585	35.20595
3.394864	3.392293	3.363957	35.38133
3.300582	3.424372	3.390574	35.30351
3.287343	3.414802	3.318548	35.09613
3.138949	3.299761	3.42021	34.74235
3.193803	3.280486	3.34682	34.65967
3.269072	3.299694	3.352515	34.87873

3.248904	3.276578	3.422853	34.93789
3.305985	3.397617	3.40558	35.28963
3.182296	3.360887	3.45346	35.04353
3.38245	3.409343	3.414924	35.50293
3.368386	3.406782	3.412264	35.46076
3.34628	3.419654	3.348218	35.30051
3.253688	3.40476	3.454842	35.29862
3.334938	3.365133	3.379798	35.22553
3.163283	3.335884	3.406415	34.84439
3.299138	3.284934	3.299996	34.79735
3.134577	3.211206	3.43102	34.56278
3.139815	3.215654	3.381025	34.47463
3.238634	3.280958	3.428309	34.93694
3.262226	3.355226	3.426451	35.14688
3.342485	3.398762	3.424422	35.41316
3.384182	3.371198	3.435266	35.46779
3.287302	3.302389	3.41622	35.0638
3.389668	3.383531	3.329956	35.27646
3.354157	3.411635	3.376183	35.36135
3.3078	3.410624	3.345524	35.19071
3.223168	3.345993	3.346104	34.86557
3.218961	3.251912	3.326341	34.60741
3.267257	3.293763	3.374358	34.90956
3.293406	3.373288	3.370044	35.13121
3.257565	3.411635	3.426877	35.26098
3.261112	3.35745	3.388937	35.06727
3.285281	3.374433	3.420824	35.227
3.343516	3.341343	3.355942	35.1401
3.338485	3.421744	3.372858	35.34191
3.255833	3.43684	3.428241	35.31529
3.248822	3.375107	3.440041	35.19076
3.183162	3.349093	3.405614	34.915
3.31213	3.259392	3.369294	34.92145
3.238098	3.210936	3.36897	34.65288
3.191287	3.28298	3.454074	34.89417
3.168562	3.292752	3.411121	34.77191
3.362447	3.356574	3.384453	35.27715
3.312501	3.402873	3.368953	35.23528
3.35094	3.355698	3.411326	35.30884
3.271588	3.387373	3.428241	35.24157
3.310109	3.453014	3.409143	35.42759
3.376841	3.426057	3.293738	35.2622
3.426622	3.291067	3.370999	35.24482
3.138495	3.216058	3.389705	34.49161
3.217683	3.297537	3.336026	34.72557
3.143403	3.361224	3.406398	34.8563
3.297695	3.465145	3.389517	35.38405
3.276331	3.425316	3.458439	35.40095
3.243749	3.277184	3.414736	34.91019
3.221518	3.386699	3.458934	35.19772

3.403608	3.424507	3.405733	35.56226
3.336093	3.433875	3.403329	35.42984
3.261566	3.345926	3.368953	34.99936
3.269897	3.204399	3.438881	34.861
3.19702	3.294033	3.302605	34.59963
3.31576	3.262897	3.331832	34.85512
3.230344	3.25272	3.389346	34.77186
3.20135	3.267884	3.417925	34.80411
3.2136	3.315531	3.409536	34.91675
3.393091	3.400717	3.374478	35.41889
3.417548	3.377736	3.377069	35.42778
3.328009	3.360415	3.362473	35.16218
3.222055	3.368974	3.460775	35.16416
3.370778	3.409006	3.478987	35.61676
3.32673	3.349295	3.328097	35.05989
3.424683	3.227717	3.328165	35.00837
3.096881	3.177239	3.336691	34.19978
3.159653	3.272669	3.377683	34.63538
3.152064	3.416352	3.4013	34.98465
3.308088	3.419924	3.358244	35.2395
3.21628	3.33986	3.478782	35.12724
3.30681	3.370794	3.374614	35.16506
3.188606	3.416083	3.500676	35.28129
3.407897	3.477478	3.347366	35.55984
3.409093	3.406513	3.431975	35.59229
3.247708	3.329616	3.356625	34.90643
3.215744	3.278599	3.453324	34.93643
3.227086	3.298885	3.388119	34.863
3.305861	3.256023	3.30136	34.75181
3.288828	3.274691	3.369311	34.90398
3.117626	3.303198	3.461799	34.79419
3.249275	3.394719	3.450204	35.25687
3.313367	3.418441	3.400277	35.33972
3.400597	3.402469	3.400277	35.49555
3.305407	3.399773	3.427474	35.34097
3.237562	3.462112	3.444764	35.36674
3.324132	3.447623	3.387505	35.39915
3.339598	3.325236	3.374955	35.13788
3.331061	3.209656	3.34832	34.80821
3.191163	3.23048	3.424285	34.71394
3.206093	3.373759	3.369771	34.94071
3.231788	3.367829	3.406841	35.065
3.338361	3.406311	3.33751	35.23059
3.233438	3.290393	3.384095	34.84952
3.24878	3.398088	3.370044	35.08786
3.244491	3.518521	3.48151	35.5856
3.356137	3.489677	3.4161	35.62364
3.385791	3.357787	3.351304	35.25836
3.397834	3.27031	3.405307	35.2115
3.172604	3.254472	3.453341	34.78936

3.24247	3.314183	3.387181	34.92805
3.169923	3.275028	3.395758	34.70253
3.252822	3.283115	3.36943	34.84393
3.197845	3.352059	3.428156	35.00289
3.237562	3.415746	3.393865	35.15403
3.24082	3.374972	3.413781	35.11555
3.433262	3.351385	3.378058	35.40668
3.286436	3.375646	3.399918	35.18646
3.319389	3.458743	3.356335	35.34493
3.356508	3.329751	3.378041	35.19149
3.237438	3.212419	3.345098	34.60247
3.35824	3.216934	3.329547	34.84251
3.264948	3.362909	3.405341	35.12347
3.300912	3.474648	3.461389	35.56904
3.217806	3.389125	3.389602	35.04329
3.306645	3.305422	3.386976	35.04878
3.228282	3.30677	3.382816	34.87126
3.282064	3.48725	3.405511	35.43319
3.258802	3.48442	3.485961	35.55206
3.223704	3.376792	3.426331	35.10954
3.267299	3.246116	3.322027	34.69101
3.361004	3.238702	3.404284	35.0596
3.259833	3.310342	3.428633	35.04827
3.203	3.280419	3.397071	34.78952
3.150373	3.277723	3.401913	34.67913
3.261071	3.353204	3.393848	35.06864
3.245522	3.365133	3.385425	35.0423
3.353456	3.360618	3.391529	35.28181
3.244656	3.386901	3.433817	35.19383
3.308872	3.375107	3.42495	35.28908
3.361251	3.42855	3.417669	35.50458
3.533484	3.393573	3.360376	35.67944
3.36983	3.242072	3.387914	35.05047
3.205722	3.221921	3.346718	34.55744
3.237108	3.241263	3.350213	34.67601
3.351724	3.407119	3.36411	35.31975
3.324091	3.417228	3.358398	35.26894
3.140764	3.353946	3.421693	34.86806
3.093045	3.299694	3.491195	34.79706
3.301448	3.461236	3.35168	35.30097
3.307965	3.514612	3.430628	35.60459
3.376594	3.410624	3.430611	35.52723
3.199742	3.256764	3.499977	34.95571
3.222508	3.22738	3.336504	34.58375
3.349579	3.316744	3.383634	35.16012
3.309944	3.34168	3.380821	35.12182
3.217105	3.281902	3.399731	34.82943
3.184564	3.288035	3.395161	34.76168
3.310975	3.373422	3.370266	35.17041
3.333907	3.432998	3.342898	35.29099

3.309367	3.449375	3.380002	35.35428
3.143775	3.334604	3.476855	34.95297
3.25608	3.343297	3.413491	35.07902
3.344011	3.401526	3.353896	35.26832
3.482548	3.287293	3.331576	35.27265
3.198422	3.177441	3.357017	34.46672
3.229891	3.234457	3.342745	34.62901
3.265855	3.341073	3.421284	35.11257
3.400061	3.471008	3.289493	35.402
3.244161	3.373153	3.415487	35.1226
3.223663	3.349632	3.376541	34.94117
3.204939	3.362168	3.445889	35.07929
3.295138	3.518588	3.381179	35.4771
3.388348	3.459417	3.474962	35.75662
3.344135	3.314251	3.378792	35.13217
3.246594	3.181013	3.415674	34.70815
3.287549	3.243824	3.390847	34.88078
3.304789	3.374568	3.431174	35.29259
3.312171	3.320518	3.387982	35.09608
3.218961	3.280486	3.337527	34.69436
3.15157	3.30987	3.4493	34.85567
3.221353	3.368907	3.350588	34.92152
3.297406	3.386969	3.40101	35.23759
3.288168	3.425383	3.446793	35.40152
3.221477	3.33703	3.482721	35.14103
3.298561	3.352867	3.371084	35.1001
3.310769	3.311488	3.403755	35.10776
3.397463	3.291539	3.333827	35.10079
3.319719	3.303333	3.375074	35.04677
3.183698	3.360213	3.414856	34.9607
3.332174	3.437986	3.266524	35.13109
3.363519	3.407658	3.392825	35.40952
3.283301	3.351654	3.288742	34.88401
3.246718	3.359472	3.414122	35.09529
3.139691	3.479163	3.448839	35.19891
3.295303	3.499583	3.474468	35.63991
3.206794	3.282508	3.384794	34.77554
3.28194	3.187011	3.453801	34.88194
3.187699	3.244633	3.443502	34.77934
3.37515	3.380701	3.407029	35.40706
3.24812	3.304748	3.453188	35.06412
3.341124	3.307983	3.324022	34.99211
3.144352	3.303198	3.458917	34.84633
3.311017	3.398291	3.324602	35.12503
3.179409	3.40038	3.450323	35.11672
3.230509	3.433605	3.445548	35.29069
3.246801	3.389597	3.497112	35.34284
3.365953	3.371198	3.395093	35.34007
3.290519	3.275836	3.410405	35.00005
3.358983	3.267345	3.318139	34.92943

3.264247	3.220641	3.472712	34.95815
3.303057	3.424979	3.337783	35.19481
3.31213	3.451936	3.427695	35.47022
3.301819	3.430033	3.35717	35.24555
3.310852	3.326584	3.398009	35.12838
3.242388	3.360078	3.409774	35.07764
3.224323	3.466763	3.431225	35.31835
3.287467	3.498236	3.444542	35.55438
3.276372	3.393977	3.396235	35.19648
3.106573	3.138151	3.456598	34.39771
3.40002	3.200018	3.293875	34.81888
3.259751	3.260201	3.463146	35.01391
3.388925	3.417498	3.365628	35.42712
3.241851	3.304546	3.489763	35.12995
3.302355	3.275904	3.358517	34.91261
3.276702	3.31324	3.29708	34.80381
3.388513	3.42262	3.39985	35.51226
3.074032	3.397954	3.457229	34.89608
3.22754	3.409343	3.384777	35.09824
3.220941	3.36675	3.437466	35.10589
3.372511	3.34943	3.374972	35.2628
3.319678	3.285271	3.420449	35.10641
3.34496	3.240252	3.418044	35.05799
3.245192	3.266469	3.407029	34.87306
3.317121	3.358259	3.383003	35.17855
3.334484	3.389934	3.361467	35.23869
3.393586	3.351115	3.334458	35.22398
3.159323	3.282104	3.368782	34.63583
3.196607	3.409074	3.395502	35.05346
3.249729	3.55397	3.348235	35.38313
3.308088	3.43839	3.415333	35.40473
3.274269	3.205342	3.402578	34.79324
3.219291	3.186472	3.425803	34.68253
3.278434	3.254472	3.415487	34.93802
3.38278	3.443175	3.352157	35.44037
3.346898	3.326584	3.439887	35.29879
3.234221	3.225291	3.420346	34.78814
3.257854	3.255349	3.440245	34.94907
3.277733	3.340534	3.324499	34.92571
3.372799	3.474446	3.448908	35.69851
3.209764	3.487722	3.423671	35.31582
3.180481	3.338512	3.486796	35.06353
3.216487	3.253394	3.384009	34.73135
3.336835	3.25117	3.376575	34.97341
3.204774	3.263638	3.396508	34.75547
3.29819	3.370322	3.376063	35.14835
3.257359	3.418037	3.459821	35.34657
3.450419	3.431853	3.333707	35.52318
3.394163	3.371468	3.318702	35.2353
3.377171	3.325775	3.324312	35.11048

3.118905	3.342017	3.417584	34.78518
3.275918	3.513197	3.421881	35.51229
3.157014	3.464606	3.443911	35.19418
3.369087	3.191662	3.322249	34.79501
3.150745	3.182698	3.472439	34.62637
3.243377	3.304479	3.425803	34.99327
3.291921	3.418846	3.462805	35.43044
3.386368	3.365874	3.378246	35.33623
3.305242	3.225224	3.398179	34.89483
3.281816	3.266199	3.36058	34.85099
3.211991	3.294302	3.400549	34.84715
3.255503	3.437312	3.35804	35.16208
3.258184	3.488126	3.377768	35.32221
3.227004	3.412106	3.373932	35.07939
3.280579	3.216058	3.356011	34.72864
3.296128	3.167332	3.401504	34.75557
3.308047	3.282508	3.365253	34.95423
3.281156	3.34815	3.400157	35.1153
3.241769	3.382588	3.398367	35.10057
3.33865	3.393236	3.393967	35.32609
3.350198	3.402469	3.392211	35.3677
3.28792	3.343837	3.267121	34.82973
3.395153	3.384812	3.375722	35.39134
3.159447	3.466695	3.388716	35.08336
3.200237	3.484959	3.414668	35.26926
3.211084	3.306298	3.426877	34.92898
3.268412	3.15008	3.333622	34.50878
3.177718	3.247261	3.443349	34.76293
3.32009	3.391012	3.441388	35.38434
3.237686	3.463528	3.468278	35.42152
3.34166	3.315936	3.348184	35.06351
3.268206	3.201097	3.374426	34.70913
3.372675	3.274354	3.382986	35.11651
3.30417	3.358933	3.371698	35.12698
3.261896	3.489744	3.420926	35.42825
3.125256	3.498842	3.384504	35.06968
3.348631	3.335749	3.349378	35.12469
3.23682	3.220573	3.416152	34.77433
3.213352	3.262425	3.414395	34.8107
3.214053	3.289045	3.448891	34.94588
3.298602	3.406041	3.416186	35.31511
3.331391	3.39209	3.366037	35.24663
3.321987	3.371603	3.374136	35.19898
3.30017	3.347948	3.415214	35.18937
3.276661	3.366953	3.395076	35.13548
3.389338	3.421205	3.36602	35.43699
3.281239	3.494529	3.439427	35.52147
3.169304	3.41487	3.408444	35.03473
3.143032	3.177172	3.419511	34.48167
3.287013	3.219563	3.387505	34.81924

3.269732	3.340804	3.43825	35.15756
3.312543	3.434751	3.521036	35.63766
3.244738	3.342691	3.37857	34.97652
3.340547	3.220102	3.454688	35.08441
3.368262	3.247598	3.296433	34.85907
3.391441	3.308522	3.40413	35.27851
3.23352	3.342556	3.355397	34.90101
3.258019	3.434953	3.417379	35.29219
3.18642	3.404693	3.447526	35.13537
3.288456	3.289989	3.281649	34.74492
3.288333	3.238702	3.384998	34.8585
3.184111	3.324764	3.433118	34.92402
3.122905	3.357517	3.465618	34.93287
3.395854	3.440681	3.345353	35.44863
3.377377	3.323821	3.385868	35.24127
3.448357	3.384273	3.362644	35.4779
3.249564	3.377129	3.380633	35.06689
3.277651	3.3652	3.352174	35.03999
3.35259	3.399773	3.371681	35.32214
3.259833	3.397078	3.340511	35.04523
3.170624	3.212149	3.404352	34.58535
3.215456	3.195099	3.398571	34.63346
3.26899	3.331234	3.483676	35.23435
3.299427	3.436233	3.390301	35.32633
3.37878	3.448229	3.475985	35.71347
3.175986	3.31944	3.464049	34.96225
3.324338	3.287158	3.41315	35.10477
3.31811	3.26849	3.369362	34.95457
3.404845	3.423698	3.364826	35.47374
3.23748	3.382184	3.399322	35.09239
3.173594	3.374298	3.41535	34.97049
3.23253	3.280015	3.384538	34.82581
3.37016	3.249755	3.338072	34.959
3.220611	3.269501	3.395161	34.79998
3.250348	3.318833	3.416834	35.02029
3.13899	3.434885	3.44132	35.0841
3.457101	3.427405	3.356744	35.57845
3.391936	3.383262	3.386567	35.40463
3.42489	3.394584	3.370948	35.46729
3.27629	3.422552	3.493514	35.47153
3.281693	3.405637	3.346973	35.12589
3.24016	3.381712	3.436971	35.17955
3.329535	3.354822	3.295205	35.00618
3.169428	3.16585	3.439819	34.55905
3.117296	3.208712	3.411548	34.47695
3.186503	3.356641	3.469301	35.07809
3.311305	3.442838	3.436017	35.46672
3.354405	3.40166	3.39644	35.38438
3.214177	3.25609	3.48417	34.95123
3.248533	3.290393	3.415555	34.95133

3.359601	3.310274	3.410132	35.22584
3.399525	3.360955	3.390574	35.3812
3.257318	3.37996	3.441251	35.2226
3.239624	3.365874	3.408717	35.08196
3.238511	3.222124	3.382696	34.70826
3.384719	3.249014	3.272526	34.84587
3.285693	3.275769	3.384913	34.9336
3.263628	3.375714	3.410849	35.16063
3.190833	3.470537	3.377564	35.13601
3.333535	3.388047	3.34919	35.20564
3.465143	3.357113	3.3238	35.37027
3.407114	3.409074	3.381724	35.48367
3.24511	3.376859	3.455438	35.22015
3.350528	3.386429	3.444065	35.44674
3.195741	3.334738	3.347042	34.78303
3.288291	3.244431	3.337817	34.76776
3.173387	3.195907	3.410149	34.56855
3.126782	3.33676	3.452795	34.86792
3.121379	3.436975	3.428497	35.02212
3.299757	3.411163	3.402937	35.29986
3.29852	3.304411	3.443383	35.15215
3.309532	3.218282	3.346666	34.77638
3.221683	3.237894	3.403977	34.75249
3.297406	3.414061	3.419238	35.3367
3.40035	3.424844	3.301275	35.32744
3.365829	3.365874	3.387999	35.31264
3.19438	3.228863	3.349258	34.55337
3.246801	3.16248	3.353351	34.53179
3.279713	3.271658	3.374972	34.8898
3.256328	3.456249	3.420039	35.34088
3.340836	3.463326	3.403533	35.50507
3.256534	3.422081	3.423126	35.27336
3.341867	3.316273	3.350588	35.06996
3.343145	3.332851	3.382458	35.1787
3.36719	3.450049	3.378314	35.47852
3.256617	3.438794	3.420176	35.30364
3.285611	3.316744	3.372585	34.99607
3.206093	3.168882	3.414958	34.59149
3.304129	3.203725	3.337748	34.71323
3.153714	3.357854	3.401504	34.86078
3.25872	3.530652	3.434772	35.54104
3.167283	3.44122	3.444253	35.16624
3.256864	3.354148	3.467988	35.22363
3.306851	3.285406	3.361331	34.94938
3.3264	3.236007	3.419323	35.01092
3.244079	3.369109	3.375637	35.02643
3.344094	3.444658	3.434976	35.54013
3.297612	3.371064	3.429571	35.26572
3.393132	3.284665	3.305129	35.01353
3.230963	3.174881	3.361109	34.54124

3.094571	3.173465	3.380957	34.28328
3.059762	3.371131	3.412417	34.70822
3.276826	3.495809	3.421642	35.47573
3.318935	3.483476	3.413236	35.52246
3.36818	3.400312	3.404898	35.43005
3.184193	3.354552	3.461458	35.05132
3.341454	3.415948	3.36602	35.32078
3.379563	3.492911	3.413185	35.67556
3.347929	3.335278	3.326273	35.0716
3.193555	3.172657	3.410763	34.56315
3.158251	3.191527	3.324073	34.33764
3.272289	3.288911	3.393865	34.95261
3.267257	3.497292	3.344006	35.28827
3.278352	3.461776	3.558276	35.70343
3.132268	3.394247	3.485517	35.07718
3.278146	3.294707	3.365219	34.91544
3.288745	3.293898	3.420738	35.05827
3.32706	3.350104	3.361194	35.13476
3.209104	3.37814	3.517318	35.27953
3.29588	3.341478	3.398571	35.12944
3.269319	3.251507	3.374017	34.82091
3.442418	3.222797	3.283968	34.93974
3.194834	3.188629	3.373523	34.51944
3.269856	3.329953	3.359881	34.96272
3.111522	3.392697	3.468056	34.99024
3.316337	3.45079	3.353879	35.31549
3.313285	3.414128	3.407489	35.34588
3.406165	3.400515	3.366583	35.42977
3.225478	3.414533	3.444133	35.23488
3.404969	3.501673	3.357443	35.62838
3.343805	3.454834	3.444321	35.58219
3.420477	3.261077	3.325557	35.06642
3.183409	3.136534	3.39482	34.4271
3.112017	3.156549	3.388699	34.30137
3.295509	3.397347	3.322914	35.08536
3.300912	3.504301	3.440774	35.58881
3.257895	3.441288	3.378996	35.22184
3.144764	3.354013	3.48226	35.00941
3.202753	3.356035	3.402851	34.96698
3.410702	3.34141	3.458047	35.51046
3.314027	3.386227	3.390745	35.24987
3.208568	3.395191	3.418846	35.1003
3.341124	3.34451	3.441047	35.3279
3.225973	3.245779	3.38435	34.73619
3.412888	3.221854	3.321345	34.95484
3.229272	3.20541	3.375773	34.63637
3.270598	3.353204	3.420039	35.14675
3.16353	3.382723	3.384589	34.89963
3.367231	3.415206	3.343358	35.32597
3.265236	3.418778	3.401368	35.23759

3.35956	3.375983	3.363565	35.26761
3.221395	3.39573	3.446435	35.18987
3.406949	3.485768	3.381179	35.64984
3.330607	3.386632	3.369362	35.24025
3.340836	3.235939	3.396116	34.99159
3.255338	3.210532	3.347536	34.64282
3.147074	3.206421	3.372363	34.45137
3.186833	3.350239	3.382867	34.87579
3.312501	3.429157	3.391904	35.34295
3.222302	3.375175	3.411172	35.06979
3.236985	3.398088	3.384316	35.09327
3.202835	3.313172	3.497607	35.08064
3.343805	3.281093	3.409604	35.12632
3.351724	3.3497	3.345132	35.15268
3.273733	3.434818	3.388272	35.26261
3.330607	3.31607	3.418539	35.19349
3.320008	3.287495	3.372568	35.00729
3.29254	3.194357	3.36411	34.72505
3.27332	3.287765	3.283098	34.71013
3.259586	3.353002	3.451431	35.19087
3.154787	3.407456	3.396747	34.96119
3.361375	3.42882	3.40708	35.48228
3.280331	3.351115	3.357648	35.02702
3.284332	3.375781	3.428974	35.24569
3.221188	3.424035	3.408188	35.16768
3.291055	3.427674	3.446299	35.41176
3.275259	3.268693	3.345371	34.80884
3.406289	3.163761	3.327074	34.8259
3.262968	3.217069	3.407217	34.80431
3.264948	3.392293	3.356693	35.08134
3.147074	3.409276	3.39482	34.94409
3.308418	3.404289	3.369839	35.23139
3.152807	3.353676	3.458525	34.97435
3.271505	3.382386	3.406671	35.18331
3.226096	3.29255	3.456598	34.99674
3.299633	3.389462	3.432385	35.31653
3.265484	3.410556	3.437773	35.29976
3.437098	3.406445	3.384845	35.55032
3.37878	3.234861	3.429946	35.14619
3.304129	3.177105	3.378024	34.74309
3.222426	3.219967	3.449044	34.81346
3.171531	3.383194	3.332752	34.80481
3.307098	3.420194	3.391546	35.31075
3.263422	3.473569	3.373608	35.29273
3.277692	3.376051	3.402578	35.17404
3.317616	3.401256	3.361791	35.22727
3.30285	3.407591	3.405511	35.30444
3.244243	3.431785	3.371442	35.15468
3.344877	3.372614	3.35729	35.21441
3.247584	3.218349	3.410235	34.78007

3.311305	3.20359	3.326801	34.70469
3.339763	3.287091	3.36498	35.03301
3.281816	3.474176	3.335191	35.25027
3.215951	3.413724	3.435249	35.19285
3.242553	3.362437	3.447185	35.16497
3.193308	3.368098	3.412008	34.99273
3.318523	3.425518	3.388664	35.34108
3.23781	3.377062	3.442769	35.17692
3.258637	3.34451	3.411445	35.08279
3.18275	3.330358	3.487853	35.05297
3.324957	3.261347	3.331508	34.87114
3.368469	3.216934	3.411872	35.04491
3.31844	3.194694	3.321089	34.68835
3.173016	3.272804	3.415947	34.74858
3.224529	3.382992	3.396934	35.06062
3.35028	3.483746	3.417754	35.60148
3.311511	3.479567	3.319793	35.29333
3.215332	3.419183	3.436494	35.20616
3.252162	3.41588	3.435607	35.27754
3.284786	3.418441	3.405733	35.28915
3.464401	3.412039	3.429247	35.71936
3.34694	3.270849	3.349855	34.98011
3.228489	3.097108	3.391324	34.43182
3.252038	3.223	3.377359	34.72809
3.277857	3.431583	3.401641	35.29379
3.415074	3.466695	3.42142	35.7139
3.177058	3.322944	3.422137	34.8806
3.248904	3.362842	3.400072	35.07672
3.215579	3.402065	3.447458	35.19324
3.356797	3.407052	3.43247	35.48019
3.220817	3.383801	3.429588	35.12568
3.296128	3.338917	3.427065	35.18669
3.174377	3.288641	3.477759	34.92136
3.453183	3.253192	3.343853	35.16071
3.411568	3.266064	3.412468	35.24791
3.245563	3.27058	3.36411	34.789
3.242223	3.307579	3.371425	34.87861
3.27497	3.407995	3.429912	35.29772
3.309408	3.463326	3.346786	35.31224
3.365953	3.394651	3.366173	35.32811
3.224488	3.451397	3.402868	35.22309
3.189349	3.413926	3.423484	35.10939
3.243542	3.431988	3.455694	35.33784
3.335309	3.326651	3.368356	35.11717
3.318317	3.182429	3.363582	34.75418
3.228365	3.210936	3.434431	34.77474
3.213393	3.377736	3.440927	35.12097
3.344259	3.443714	3.402937	35.46836
3.303675	3.424439	3.372482	35.27086
3.281486	3.319575	3.374426	34.99727

3.120637	3.340467	3.447339	34.85065
3.261401	3.412915	3.416646	35.24979
3.294148	3.435964	3.47885	35.50784
3.250471	3.320788	3.361723	34.90431
3.278682	3.229806	3.477827	35.02095
3.2501	3.224887	3.349446	34.66693
3.313285	3.259797	3.43484	35.0682
3.319636	3.346869	3.334577	35.05324
3.309367	3.361898	3.472166	35.36453
3.181182	3.336828	3.377069	34.82143
3.278352	3.475254	3.416782	35.42348
3.322647	3.444051	3.384538	35.3816
3.290972	3.448297	3.393098	35.34034
3.198917	3.420531	3.397599	35.08815
3.263009	3.413926	3.422307	35.2679
3.20102	3.302524	3.40205	34.84442
3.422044	3.226841	3.305453	34.95102
3.274599	3.195907	3.451482	34.88027
3.36088	3.34013	3.357699	35.17926
3.323307	3.420328	3.439836	35.4521
3.362076	3.438188	3.325437	35.32576
3.268618	3.336693	3.411889	35.08849
3.395566	3.359876	3.373318	35.33245
3.156766	3.388788	3.495816	35.14134
3.223168	3.434414	3.403619	35.18471
3.25806	3.335749	3.468295	35.18669
3.24379	3.249755	3.397685	34.81301
3.337206	3.293898	3.44855	35.22506
3.224735	3.277521	3.41194	34.86323
3.258926	3.342286	3.380889	35.01173
3.276042	3.330695	3.425359	35.12106
3.274434	3.357922	3.383157	35.0848
3.323555	3.399234	3.404932	35.33018
3.278929	3.4208	3.36099	35.18366
3.25307	3.448903	3.435948	35.35248
3.297076	3.478085	3.308505	35.23384
3.253729	3.357922	3.426195	35.13364
3.288869	3.395797	3.374495	35.18025
3.193597	3.234457	3.432947	34.7469
3.266597	3.213093	3.349446	34.67722
3.306562	3.248003	3.441388	35.04202
3.407072	3.423698	3.383174	35.51873
3.262102	3.45598	3.482925	35.49044
3.358364	3.410017	3.396508	35.41146
3.22919	3.336288	3.391137	34.956
3.321286	3.38097	3.402306	35.27953
3.25905	3.425652	3.40662	35.25058
3.150662	3.410556	3.439427	35.05228
3.108429	3.250698	3.435624	34.60203
3.287219	3.243353	3.428019	34.96032

3.308748	3.216328	3.372295	34.82644
3.361004	3.264986	3.437159	35.18897
3.242181	3.356709	3.413679	35.07836
3.241851	3.318294	3.420466	35.00847
3.36917	3.396943	3.324175	35.24832
3.428065	3.406917	3.388681	35.53999
3.213105	3.393304	3.357903	34.97283
3.257483	3.441423	3.381707	35.22716
3.214878	3.40166	3.4124	35.11416
3.316213	3.393169	3.362593	35.20827
3.373212	3.309466	3.434857	35.3079
3.137134	3.227448	3.412997	34.56447
3.239913	3.223202	3.395349	34.74135
3.265525	3.338108	3.361433	34.97448
3.397958	3.439805	3.415418	35.60454
3.277527	3.408265	3.364195	35.16019
3.272866	3.344713	3.430117	35.15518
3.282311	3.381712	3.334116	35.0468
3.373005	3.411298	3.384777	35.42062
3.195535	3.452543	3.397412	35.15035
3.255627	3.308185	3.441268	35.06198
3.091808	3.226639	3.446759	34.53742
3.242181	3.255888	3.39864	34.82499
3.373995	3.251844	3.417925	35.14658
3.324751	3.3497	3.385186	35.18129
3.219209	3.321057	3.500932	35.14097
3.291921	3.368233	3.403943	35.19104
3.289034	3.409613	3.386874	35.23789
3.480238	3.443647	3.301838	35.54449
3.191163	3.427135	3.421301	35.13747
3.162911	3.429022	3.337424	34.89639
3.151075	3.379825	3.42715	34.95913
3.295055	3.364796	3.299877	34.9628
3.305366	3.251575	3.430083	35.02249
3.255379	3.219495	3.41622	34.81271
3.207166	3.267682	3.444389	34.87425
3.343021	3.394786	3.38655	35.32282
3.40534	3.364392	3.400259	35.42262
3.26136	3.377331	3.390796	35.11535
3.24445	3.335615	3.369481	34.94054
3.273031	3.354552	3.41772	35.14994
3.354817	3.480107	3.342404	35.43866
3.222673	3.410624	3.412144	35.15025
3.230922	3.302592	3.416424	34.94139
3.118245	3.181418	3.40621	34.40766
3.254101	3.209993	3.367538	34.68268
3.356838	3.25299	3.413901	35.10276
3.361581	3.383868	3.369294	35.3018
3.186585	3.343365	3.449914	35.00684
3.365747	3.364324	3.367674	35.26462

3.315017	3.40813	3.352737	35.21682
3.331721	3.432931	3.324482	35.24579
3.214136	3.423698	3.391529	35.11508
3.199041	3.373355	3.338601	34.85624
3.091024	3.340602	3.404556	34.69263
3.289529	3.356978	3.314456	34.9655
3.297118	3.324495	3.404761	35.10855
3.276496	3.280419	3.384095	34.92187
3.236655	3.332717	3.446043	35.08458
3.304871	3.419924	3.373574	35.26599
3.42889	3.393034	3.357972	35.44428
3.322029	3.37659	3.356556	35.17153
3.236572	3.347543	3.346786	34.89976
3.265401	3.393977	3.419715	35.22384
3.293241	3.440277	3.347792	35.22868
3.213022	3.274623	3.389483	34.78217
3.269196	3.244094	3.423876	34.91346
3.151363	3.211004	3.462873	34.6687
3.224447	3.259931	3.414617	34.82999
3.339887	3.329751	3.465124	35.34557
3.295303	3.450251	3.410763	35.39271
3.263917	3.361224	3.422785	35.15568
3.400061	3.423698	3.370385	35.47543
3.322771	3.410691	3.410985	35.36675
3.424147	3.421541	3.306561	35.38382
3.179615	3.395864	3.377888	34.94889
3.327803	3.30185	3.32172	34.94453
3.149012	3.221652	3.382543	34.51118
3.253152	3.313577	3.336094	34.83836
3.244243	3.31452	3.386226	34.93057
3.309862	3.36675	3.405528	35.2305
3.236242	3.415206	3.477605	35.33309
3.388884	3.41487	3.44473	35.59427
3.371727	3.355293	3.398571	35.32552
3.422168	3.395662	3.400942	35.52929
3.27365	3.407524	3.372244	35.16769
3.194628	3.387777	3.458439	35.14019
3.241563	3.339119	3.401777	35.01251
3.190338	3.247194	3.366463	34.62224
3.215167	3.227582	3.374136	34.65043
3.251131	3.236007	3.42906	34.86761
3.19801	3.281026	3.438472	34.87047
3.359973	3.413387	3.449385	35.53798
3.391359	3.410691	3.397736	35.48777
3.318482	3.350374	3.361876	35.11808
3.404763	3.393775	3.388681	35.46029
3.288415	3.447758	3.417328	35.38655
3.297942	3.421272	3.399509	35.3105
3.241728	3.359741	3.35185	34.94879
3.314275	3.289719	3.371545	34.99738

3.075434	3.282508	3.344075	34.39923
3.28891	3.271725	3.335668	34.8241
3.207784	3.319912	3.422699	34.9424
3.28359	3.366009	3.419988	35.20305
3.207001	3.371738	3.435028	35.08098
3.330896	3.35745	3.434925	35.32045
3.311883	3.332447	3.3873	35.12004
3.362323	3.436099	3.375978	35.43226
3.317409	3.361292	3.387232	35.19506
3.327679	3.413926	3.367384	35.28921
3.108924	3.315262	3.400259	34.66696
3.166128	3.247868	3.318156	34.46513
3.165757	3.267008	3.369601	34.61868
3.255503	3.269299	3.379678	34.84199
3.235252	3.332851	3.446725	35.0833
3.344218	3.378679	3.415145	35.35275
3.367479	3.37477	3.415128	35.39503
3.455204	3.369716	3.346001	35.42465
3.320544	3.448229	3.431805	35.4895
3.270845	3.438996	3.390216	35.26968
3.315471	3.379825	3.371204	35.1963
3.203289	3.343297	3.305299	34.72697
3.302067	3.249957	3.356028	34.8498
3.167325	3.329953	3.362831	34.74495
3.190545	3.322608	3.446179	34.96194
3.255338	3.386969	3.419784	35.18665
3.327596	3.386227	3.408683	35.31877
3.270763	3.340332	3.41078	35.09871
3.314728	3.351789	3.447049	35.29922
3.250925	3.45106	3.433698	35.34759
3.379233	3.435627	3.336845	35.38263
3.334072	3.367222	3.371152	35.2093
3.305036	3.325168	3.359012	35.02729
3.159653	3.210532	3.40101	34.55051
3.290106	3.280352	3.34919	34.87516
3.077249	3.259729	3.445821	34.57589
3.288333	3.365807	3.347758	35.05502
3.242058	3.332649	3.48492	35.18127
3.324751	3.382049	3.393047	35.26922
3.263133	3.358057	3.446861	35.19969
3.342444	3.406243	3.387931	35.34963
3.328875	3.430303	3.441592	35.48992
3.388142	3.409411	3.320202	35.30838
3.233355	3.294707	3.433902	34.96769
3.224529	3.234996	3.332616	34.59632
3.326318	3.283115	3.358995	34.98183
3.253028	3.353676	3.384589	35.03183
3.222343	3.455036	3.460639	35.3527
3.264494	3.416757	3.430151	35.29449
3.343228	3.369109	3.363445	35.2166

3.370201	3.385823	3.393712	35.37832
3.351394	3.395123	3.401283	35.37409
3.253729	3.459821	3.427832	35.36005
3.31345	3.362168	3.360904	35.13074
3.221725	3.241263	3.428036	34.81256
3.348837	3.213699	3.339641	34.83695
3.106449	3.236411	3.414446	34.52014
3.19005	3.224954	3.385067	34.61366
3.218301	3.276173	3.444338	34.91706
3.245976	3.387036	3.398554	35.1199
3.239336	3.394112	3.417533	35.16236
3.341867	3.409141	3.373318	35.32275
3.23748	3.354148	3.434959	35.10901
3.38113	3.429022	3.391461	35.49177
3.418167	3.446275	3.343529	35.50567
3.358405	3.380701	3.35792	35.26305
3.268495	3.264784	3.353845	34.80403
3.156024	3.208106	3.407643	34.55177
3.223622	3.322608	3.30443	34.72429
3.296623	3.366683	3.351833	35.08398
3.205928	3.408939	3.419562	35.12616
3.237933	3.364459	3.412076	35.08251
3.277692	3.303333	3.423859	35.06155
3.377542	3.409141	3.414327	35.49045
3.309573	3.477883	3.44091	35.55027
3.275176	3.418981	3.412093	35.28322
3.21228	3.26876	3.43056	34.85756
3.289405	3.25892	3.359233	34.84872
3.307346	3.230278	3.415742	34.94889
3.238346	3.261886	3.365406	34.75704
3.086611	3.222056	3.41651	34.44988
3.212486	3.296189	3.418198	34.89096
3.331556	3.386969	3.423501	35.36146
3.334484	3.424574	3.335736	35.25817
3.348796	3.366211	3.412622	35.32998
3.200196	3.404289	3.392211	35.04365
3.308171	3.421541	3.380821	35.29259
3.430829	3.423698	3.325181	35.44387
3.443243	3.302457	3.341329	35.24119
3.167407	3.228324	3.398094	34.6
3.223416	3.256764	3.376387	34.73721
3.233768	3.378544	3.387948	35.05144
3.284208	3.41433	3.372056	35.20525
3.217971	3.342084	3.451619	35.0764
3.18543	3.327325	3.406534	34.87437
3.173429	3.397347	3.453938	35.10492
3.43586	3.472222	3.384708	35.69116
3.309367	3.448701	3.476037	35.56282
3.286848	3.35772	3.350537	35.04017
3.1851	3.215991	3.446043	34.71658

3.261566	3.27678	3.3607	34.8301
3.373459	3.259594	3.363172	35.04262
3.272289	3.281228	3.323937	34.78288
3.150291	3.264245	3.389159	34.62158
3.199536	3.351317	3.434635	35.01914
3.329947	3.398897	3.441251	35.42284
3.400762	3.396741	3.391205	35.46355
3.299716	3.366616	3.462003	35.33152
3.173841	3.37632	3.423995	34.99436
3.310274	3.454362	3.394087	35.39797
3.380883	3.341815	3.359268	35.23012
3.365087	3.254405	3.358398	35.00252
3.217765	3.213834	3.356539	34.58757
3.150456	3.398088	3.402715	34.94428
3.268495	3.457799	3.437295	35.40862
3.370861	3.454025	3.264052	35.24536
3.230468	3.287698	3.40205	34.8764
3.273031	3.347476	3.384112	35.06097
3.187163	3.421407	3.490036	35.26651
3.357044	3.504907	3.408922	35.64323
3.327102	3.366346	3.364315	35.17719
3.433221	3.257438	3.414992	35.28191
3.204485	3.259257	3.470648	34.9074
3.337825	3.307174	3.40783	35.1664
3.266597	3.247261	3.367299	34.79098
3.257483	3.272736	3.412912	34.92651
3.108759	3.369311	3.412673	34.81195
3.163736	3.376253	3.448703	35.02614
3.245398	3.410354	3.418061	35.21229
3.27332	3.369379	3.390574	35.12364
3.266144	3.345184	3.383498	35.03956
3.350651	3.402536	3.468704	35.53612
3.252781	3.351519	3.363769	34.98105
3.389915	3.265997	3.361723	35.08944
3.302603	3.228728	3.333008	34.7542
3.222096	3.273478	3.340102	34.69152
3.25307	3.43219	3.374478	35.18151
3.299881	3.412174	3.318412	35.1175
3.291302	3.346802	3.418539	35.17474
3.239418	3.349498	3.310449	34.8308
3.274764	3.468987	3.498084	35.57973
3.192648	3.440749	3.446452	35.22549
3.285322	3.393371	3.39025	35.20164
3.231334	3.268356	3.421284	34.87806
3.350858	3.242072	3.392706	35.01946
3.248244	3.271725	3.394684	34.86423
3.25608	3.28453	3.436085	34.99991
3.27134	3.255551	3.411053	34.91517
3.28095	3.297941	3.36515	34.9285
3.290189	3.338782	3.414105	35.14507

3.258101	3.428416	3.423381	35.2912
3.202711	3.370255	3.419425	35.03423
3.288786	3.31634	3.39303	35.04684
3.259009	3.427607	3.401231	35.24298
3.397958	3.459484	3.28835	35.3697
3.334195	3.298818	3.437176	35.20436
3.291838	3.249418	3.316826	34.74052
3.255214	3.166793	3.379064	34.61585
3.32273	3.376455	3.317798	35.08801
3.205392	3.399504	3.385049	35.02888
3.208609	3.412241	3.372568	35.03648
3.264576	3.312296	3.406739	35.01503
3.19471	3.362168	3.385339	34.92451
3.341619	3.550196	3.461253	35.82298
3.186379	3.439266	3.467391	35.25433
3.191452	3.305085	3.424609	34.87843
3.243419	3.194829	3.391853	34.67933
3.365788	3.295987	3.404556	35.19593
3.290849	3.319642	3.419784	35.11708
3.237933	3.220506	3.428633	34.80392
3.216899	3.246924	3.384572	34.71934
3.30252	3.344713	3.380889	35.11237
3.338608	3.456991	3.386635	35.44939
3.279259	3.399099	3.449914	35.33138
3.18143	3.356776	3.405119	34.92693
3.235417	3.377264	3.377513	35.02943
3.329122	3.426529	3.467238	35.5383
3.399401	3.470604	3.324551	35.47633
3.346073	3.194896	3.418675	34.96262
3.34133	3.215452	3.31848	34.77809
3.191246	3.250429	3.430765	34.77192
3.346651	3.457328	3.380087	35.4534
3.261854	3.371401	3.383413	35.08732
3.17978	3.288169	3.392706	34.74615
3.150497	3.313644	3.365099	34.67745
3.283342	3.469795	3.405375	35.39751
3.304294	3.515758	3.424012	35.5846
3.276743	3.404154	3.409348	35.24822
3.157302	3.233243	3.474621	34.75601
3.227952	3.231087	3.336367	34.60346
3.459287	3.354148	3.34815	35.40423
3.287714	3.350509	3.338686	35.00038
3.211331	3.285811	3.441746	34.91723
3.232407	3.315262	3.379405	34.89139
3.293323	3.311959	3.370658	34.99826
3.347929	3.33056	3.35717	35.12885
3.300417	3.389597	3.44398	35.34389
3.094695	3.373557	3.47856	34.93456
3.301407	3.38744	3.416424	35.28108
3.310852	3.377129	3.388733	35.21863

3.393833	3.333593	3.343529	35.20604
3.242264	3.230009	3.420125	34.81556
3.260658	3.268423	3.37458	34.84019
3.255297	3.425181	3.403619	35.23478
3.422992	3.440951	3.275306	35.35539
3.317533	3.295448	3.414668	35.11134
3.214383	3.290124	3.321021	34.66933
3.090406	3.34633	3.397344	34.68803
3.259957	3.523239	3.354988	35.35306
3.279094	3.445331	3.438063	35.40621
3.211001	3.28945	3.404028	34.84198
3.168314	3.176296	3.423245	34.54321
3.421961	3.299694	3.39864	35.31394
3.348466	3.416352	3.424626	35.46516
3.367479	3.357248	3.426382	35.38132
3.214177	3.226167	3.354425	34.60207
3.22919	3.247666	3.398776	34.7789
3.214383	3.386092	3.351424	34.94568
3.300293	3.410961	3.394189	35.28146
3.210713	3.363381	3.333281	34.84831
3.205145	3.351048	3.456069	35.07769
3.247254	3.340332	3.409484	35.04447
3.424518	3.303939	3.399305	35.33027
3.450254	3.307242	3.329564	35.24126
3.223044	3.271658	3.421062	34.86666
3.28194	3.394314	3.434363	35.29277
3.314069	3.39236	3.366855	35.21113
3.403567	3.331436	3.331456	35.19621
3.365334	3.312498	3.320714	35.04769
3.239336	3.403952	3.471808	35.30257
3.070815	3.512725	3.366634	34.94191
3.305572	3.442434	3.425189	35.42962
3.245646	3.273814	3.365969	34.80032
3.206588	3.162143	3.430219	34.61121
3.164809	3.262088	3.428906	34.73554
3.288415	3.417026	3.4914	35.48133
3.328957	3.370053	3.401214	35.27005
3.330483	3.298952	3.331917	34.96636
3.254926	3.309331	3.460076	35.10408
3.288786	3.36675	3.329001	35.01706
3.210671	3.345791	3.419494	34.99829
3.216157	3.498303	3.29338	35.06802
3.167902	3.373355	3.401436	34.92555
3.317863	3.346263	3.348764	35.07906
3.272165	3.248003	3.420312	34.92071
3.317121	3.280621	3.298172	34.82325
3.257648	3.261347	3.460042	35.00503
3.383193	3.432122	3.347826	35.40764
3.351476	3.373288	3.45624	35.4467
3.287508	3.350306	3.343393	35.00977

3.268577	3.291	3.348031	34.84882
3.267216	3.32901	3.370334	34.97774
3.20234	3.452677	3.390029	35.14938
3.317162	3.538537	3.395843	35.60095
3.209269	3.327325	3.412127	34.93873
3.227457	3.159987	3.464783	34.72772
3.277279	3.219428	3.406534	34.83928
3.226468	3.383599	3.473854	35.23439
3.363396	3.426326	3.394053	35.45276
3.25241	3.292954	3.474672	35.09469
3.246718	3.272467	3.392365	34.85744
3.301242	3.29538	3.362081	34.96056
3.2826	3.31169	3.304958	34.83054
3.115234	3.374905	3.404642	34.82077
3.238428	3.473772	3.393575	35.28219
3.155859	3.391349	3.510924	35.178
3.328545	3.368435	3.315206	35.07752
3.288003	3.24625	3.396321	34.89905
3.25208	3.184046	3.485262	34.87896
3.16353	3.379151	3.416288	34.96114
3.415197	3.375377	3.368475	35.39869
3.358859	3.364729	3.351287	35.21461
3.428767	3.306298	3.393371	35.33174
3.224488	3.314992	3.373608	34.86081
3.217477	3.458945	3.41315	35.24675
3.19438	3.533348	3.383702	35.29455
3.314646	3.362707	3.380292	35.17693
3.162623	3.172252	3.422972	34.52132
3.193308	3.179396	3.4161	34.58902
3.225272	3.324495	3.461986	35.07657
3.383976	3.51441	3.405818	35.71612
3.280002	3.330762	3.431617	35.14355
3.269484	3.237422	3.363445	34.76735
3.259462	3.254338	3.423876	34.91458
3.267134	3.410219	3.357972	35.12812
3.246264	3.4084	3.389653	35.14779
3.202629	3.423631	3.366907	35.03593
3.131896	3.34633	3.459275	34.9142
3.180027	3.263031	3.349582	34.59741
3.345826	3.251372	3.348065	34.93117
3.288456	3.276915	3.378843	34.92888
3.260576	3.388788	3.418539	35.19936
3.236201	3.374501	3.3954	35.06422
3.397215	3.406647	3.35879	35.40657
3.419033	3.353474	3.3009	35.2114
3.3549	3.334132	3.349122	35.13431
3.162829	3.372142	3.423654	34.96039
3.31609	3.501336	3.429384	35.5906
3.181141	3.436907	3.40899	35.11
3.295509	3.237085	3.309477	34.70551

3.112718	3.177509	3.49377	34.57851
3.183533	3.260875	3.418829	34.75179
3.196937	3.44021	3.456189	35.25498
3.385873	3.421811	3.360444	35.41854
3.280496	3.278465	3.456342	35.08434
3.380223	3.292617	3.36174	35.12649
3.228117	3.337906	3.41194	35.00268
3.389915	3.374029	3.311182	35.21516
3.265113	3.387845	3.330553	35.01481
3.243914	3.423024	3.439546	35.28374
3.196855	3.266873	3.375467	34.69922
3.315553	3.277386	3.430014	35.10107
3.262226	3.275297	3.370112	34.84889
3.340176	3.332717	3.411326	35.23504
3.342032	3.375175	3.413918	35.33762
3.302933	3.364459	3.405102	35.20941
3.351683	3.363111	3.405938	35.31489
3.336794	3.32436	3.310909	34.98978
3.354982	3.375781	3.421676	35.38423
3.215579	3.440479	3.397275	35.16751
3.254224	3.463191	3.449726	35.41638
3.216115	3.330964	3.372568	34.87515
3.295757	3.132355	3.346172	34.55727
3.218301	3.212554	3.440791	34.77018
3.267711	3.434683	3.414941	35.30747
3.200319	3.43684	3.474519	35.29509
3.379852	3.348554	3.372568	35.27169
3.37779	3.243083	3.392723	35.0806
3.325081	3.29228	3.367384	35.01751
3.255585	3.337636	3.398367	35.03248
3.295715	3.415274	3.418283	35.33357
3.210836	3.411972	3.377479	35.0515
3.320626	3.301042	3.321481	34.92655
3.226509	3.20979	3.369226	34.62559
3.206506	3.294572	3.383737	34.79898
3.188276	3.334267	3.391137	34.8621
3.243254	3.455643	3.479532	35.44107
3.349827	3.411432	3.430407	35.47001
3.397298	3.353137	3.386618	35.35059
3.24511	3.315396	3.401572	34.96794
3.364757	3.366683	3.405256	35.3498
3.341908	3.447892	3.356966	35.37183
3.290807	3.475254	3.436	35.49275
3.166747	3.350306	3.397616	34.86427
3.205598	3.159313	3.366992	34.46459
3.257606	3.242679	3.355226	34.7349
3.298066	3.375781	3.412127	35.23889
3.305449	3.503627	3.528436	35.78896
3.160354	3.380297	3.405921	34.93403
3.246924	3.239983	3.414037	34.83425

3.387606	3.245644	3.356829	35.02918
3.43054	3.352328	3.409553	35.47167
3.211001	3.399234	3.400328	35.07397
3.238593	3.383599	3.412076	35.12581
3.198546	3.330964	3.387215	34.86876
3.29819	3.229874	3.306015	34.68803
3.212239	3.26256	3.344228	34.65511
3.182956	3.36857	3.461372	35.07908
3.092303	3.446882	3.435676	34.99589
3.323843	3.445534	3.377751	35.37262
3.291838	3.348015	3.417652	35.17663
3.413094	3.347813	3.373386	35.34455
3.203742	3.386766	3.481732	35.20885
3.312873	3.417228	3.321481	35.16367
3.344218	3.400649	3.442837	35.46135
3.341083	3.345656	3.281632	34.9817
3.128391	3.189775	3.378348	34.3872
3.213641	3.216597	3.332377	34.53175
3.141671	3.378881	3.431992	34.9471
3.322647	3.497225	3.393575	35.51765
3.338897	3.396404	3.436954	35.42757
3.14695	3.287158	3.473257	34.8483
3.250925	3.255753	3.363565	34.76711
3.370737	3.35462	3.414719	35.3572
3.368799	3.409411	3.384879	35.40752
3.265319	3.37659	3.417806	35.18146
3.227004	3.349026	3.432402	35.06931
3.199907	3.225628	3.347502	34.55454
3.401298	3.233917	3.306749	34.92396
3.20399	3.254809	3.411053	34.76626
3.216487	3.342489	3.418743	35.00215
3.129669	3.433672	3.410559	34.9938
3.418002	3.475996	3.341943	35.56684
3.387069	3.35435	3.405733	35.37267
3.358323	3.385149	3.349838	35.25493
3.247749	3.433201	3.52078	35.49202
3.322194	3.428887	3.37932	35.33604
3.286312	3.397684	3.439666	35.3213
3.394411	3.274151	3.318872	35.02339
3.089911	3.155741	3.419442	34.31849
3.192442	3.25117	3.417618	34.7474
3.179326	3.372479	3.406193	34.95902
3.31609	3.432324	3.448959	35.48249
3.18873	3.420328	3.422853	35.12066
3.334402	3.315194	3.460025	35.2906
3.178254	3.290259	3.416015	34.79835
3.369788	3.404154	3.374426	35.37533
3.318523	3.391416	3.387573	35.26412
3.323678	3.409545	3.398435	35.33879
3.276248	3.31142	3.369635	34.9575

3.226138	3.181148	3.408052	34.64705
3.341867	3.23412	3.3142	34.81073
3.25608	3.298009	3.373403	34.89231
3.227375	3.376186	3.462123	35.19451
3.233108	3.431516	3.389091	35.16834
3.349827	3.390136	3.355192	35.25896
3.41297	3.36729	3.366225	35.37121
3.345909	3.428079	3.42507	35.48618
3.18642	3.435222	3.414054	35.12894
3.298479	3.362842	3.443093	35.27921
3.266267	3.29875	3.404403	34.984
3.324957	3.173196	3.321686	34.65689
3.183327	3.251238	3.404966	34.69995
3.17149	3.424642	3.40928	35.06271
3.212486	3.436368	3.448277	35.26328
3.297118	3.401323	3.382048	35.22689
3.259586	3.293291	3.423739	34.99974
3.348342	3.294437	3.404164	35.15353
3.246429	3.338445	3.404369	35.02735
3.302562	3.426663	3.407353	35.34955
3.391029	3.39728	3.32953	35.30857
3.378161	3.338175	3.373813	35.24801
3.221642	3.239983	3.394939	34.7372
3.228076	3.179868	3.302179	34.41696
3.183574	3.283182	3.371715	34.69764
3.259462	3.41797	3.415214	35.25347
3.223209	3.469526	3.460196	35.38531
3.19339	3.430977	3.423177	35.15484
3.330607	3.34633	3.372022	35.15794
3.415569	3.403345	3.363616	35.45004
3.304129	3.462112	3.435317	35.49165
3.240697	3.511849	3.44236	35.4771
3.240862	3.325438	3.396764	34.9701
3.194999	3.125009	3.394684	34.42695
3.354157	3.155269	3.379064	34.80702
3.269361	3.310544	3.433408	35.07998
3.254307	3.472626	3.449453	35.4366
3.132804	3.384071	3.403005	34.87566
3.276042	3.368098	3.454637	35.26688
3.238511	3.357989	3.35995	34.95563
3.308377	3.267816	3.396525	34.99121
3.236531	3.383329	3.414719	35.12649
3.365458	3.434279	3.450596	35.59831
3.307181	3.396404	3.430475	35.34404
3.418332	3.303939	3.343103	35.19383
3.322111	3.18162	3.399731	34.83976
3.156519	3.202714	3.362422	34.44218
3.171944	3.290798	3.385885	34.71984
3.272825	3.427944	3.344944	35.15084
3.283095	3.449038	3.4124	35.36694

3.295262	3.416554	3.416714	35.33195
3.163901	3.329212	3.431072	34.88508
3.377831	3.366616	3.4028	35.37288
3.365087	3.488666	3.438387	35.68973
3.420972	3.408063	3.392211	35.5347
3.292045	3.244094	3.378161	34.86346
3.233396	3.11052	3.307925	34.2895
3.267752	3.229874	3.34607	34.70906
3.343475	3.433538	3.318497	35.25974
3.176481	3.488329	3.519893	35.45479
3.158168	3.387238	3.388545	34.90644
3.305036	3.358259	3.468756	35.33965
3.226798	3.357652	3.444065	35.11323
3.275547	3.328471	3.415759	35.09412
3.176068	3.365537	3.428105	34.98463
3.257895	3.402469	3.425018	35.23759
3.28858	3.313712	3.387727	35.02904
3.475578	3.265525	3.309341	35.16118
3.288786	3.151562	3.410849	34.72546
3.226962	3.287765	3.361604	34.78043
3.151941	3.383329	3.443588	35.00464
3.383646	3.50147	3.372704	35.61469
3.268701	3.442366	3.376336	35.24201
3.321534	3.359135	3.29321	34.99375
3.221436	3.357989	3.489524	35.20165
3.318894	3.474917	3.401368	35.4777
3.320544	3.418037	3.442633	35.44716
3.448687	3.276847	3.330808	35.17408
3.233891	3.131412	3.461918	34.67303
3.191988	3.191796	3.301395	34.36241
3.254678	3.293022	3.39291	34.921
3.257606	3.493451	3.409553	35.4021
3.289488	3.412713	3.36868	35.20588
3.146249	3.3528	3.4759	34.99609
3.193803	3.330627	3.420858	34.93123
3.315718	3.315531	3.417737	35.158
3.295097	3.357854	3.398964	35.1644
3.199164	3.407591	3.461577	35.2003
3.345702	3.384745	3.414378	35.36758
3.284291	3.186135	3.389142	34.74377
3.408557	3.178318	3.261886	34.72014
3.324957	3.179665	3.373932	34.78529
3.196112	3.336558	3.396184	34.89529
3.198051	3.482398	3.423603	35.27842
3.363767	3.469256	3.369106	35.4929
3.31114	3.409411	3.316502	35.1319
3.296953	3.403345	3.379866	35.22618
3.173222	3.385621	3.455916	35.08315
3.293983	3.457395	3.424353	35.43517
3.264989	3.403547	3.331184	35.05026

3.278063	3.147249	3.430424	34.73539
3.265071	3.150888	3.36336	34.56828
3.294973	3.26721	3.41344	34.99756
3.264205	3.41615	3.411326	35.25136
3.278434	3.44331	3.399663	35.31637
3.299386	3.349835	3.40928	35.1788
3.235954	3.324562	3.342421	34.83861
3.230303	3.370592	3.397804	35.04803
3.268288	3.371738	3.434226	35.21325
3.326937	3.320384	3.449777	35.26321
3.384512	3.359607	3.435471	35.44361
3.361787	3.30859	3.379047	35.15896
3.301531	3.243689	3.345439	34.81176
3.321575	3.223606	3.37354	34.87313
3.284827	3.230683	3.382441	34.82771
3.296458	3.379623	3.377768	35.16863
3.173387	3.447151	3.384777	35.0625
3.278847	3.450993	3.403465	35.34239
3.304541	3.394854	3.353061	35.16558
3.309367	3.402604	3.406927	35.31088
3.241563	3.412443	3.327773	35.01103
3.356219	3.40685	3.499653	35.6254
3.181636	3.313981	3.377615	34.77365
3.383316	3.21343	3.353197	34.94141
3.282971	3.202175	3.381759	34.75981
3.325988	3.37659	3.396235	35.26696
3.317409	3.426663	3.411053	35.39011
3.241233	3.387238	3.344569	34.99191
3.214136	3.290528	3.379405	34.79735
3.325988	3.365605	3.337356	35.11418
3.243625	3.390069	3.463759	35.26399
3.245811	3.376792	3.391563	35.08185
3.22457	3.341545	3.50748	35.21181
3.372593	3.399032	3.372568	35.3662
3.356673	3.246183	3.372295	34.99653
3.327019	3.230211	3.362695	34.87576
3.221395	3.199007	3.454773	34.7779
3.263958	3.331638	3.379405	34.9962
3.249688	3.447286	3.416782	35.29964
3.372428	3.457125	3.302997	35.34074
3.2209	3.358865	3.429725	35.07162
3.242511	3.370861	3.386823	35.0513
3.329205	3.432055	3.404488	35.41334
3.29522	3.429764	3.414532	35.35597
3.335556	3.359809	3.354306	35.15949
3.226798	3.18964	3.431276	34.71785
3.231623	3.187416	3.35422	34.55503
3.340918	3.364324	3.424422	35.33443
3.325699	3.461371	3.37579	35.40702
3.195164	3.417633	3.438557	35.16317

3.270186	3.383329	3.399049	35.16582
3.209475	3.255146	3.428139	34.81636
3.343764	3.39263	3.327432	35.19045
3.226261	3.354283	3.472882	35.16771
3.174583	3.34505	3.519484	35.13641
3.160519	3.329212	3.457962	34.93649
3.46205	3.282239	3.35717	35.27275
3.35523	3.169017	3.37608	34.8329
3.402618	3.269906	3.364946	35.13281
3.219786	3.292415	3.483744	35.04201
3.294107	3.431853	3.338396	35.19161
3.307305	3.470132	3.336435	35.29989
3.399814	3.397415	3.32837	35.32554
3.2607	3.376051	3.383771	35.09575
3.257194	3.386227	3.40737	35.16194
3.27332	3.438457	3.42345	35.34659
3.348837	3.367761	3.399594	35.30497
3.293034	3.203927	3.398367	34.82197
3.153302	3.131951	3.443792	34.45834
3.26136	3.307714	3.440569	35.07196
3.375521	3.425383	3.381878	35.45059
3.338856	3.432459	3.357085	35.33166
3.260493	3.417767	3.418948	35.26345
3.171036	3.298952	3.418266	34.8065
3.24016	3.376253	3.406603	35.1012
3.297035	3.417228	3.342489	35.17498
3.248698	3.351452	3.44282	35.14484
3.233685	3.302929	3.456615	35.03606
3.162747	3.284665	3.474076	34.87918
3.323018	3.234457	3.419136	34.99972
3.393091	3.255551	3.360768	35.07145
3.316296	3.340197	3.417533	35.21275
3.223869	3.363246	3.419903	35.06622
3.374201	3.428753	3.41697	35.53181
3.289817	3.440883	3.310227	35.14038
3.443779	3.389934	3.356607	35.46707
3.170542	3.434414	3.390387	35.04069
3.132928	3.453823	3.402664	35.02772
3.244037	3.360955	3.429043	35.1253
3.319719	3.246385	3.374546	34.92108
3.296623	3.133096	3.41315	34.70725
3.319018	3.200558	3.436426	34.95465
3.187946	3.439536	3.501836	35.33367
3.408846	3.484757	3.324414	35.52764
3.400639	3.364122	3.338754	35.27724
3.299963	3.320788	3.344893	34.97574
3.140723	3.328269	3.453546	34.88147
3.284126	3.418239	3.425104	35.32963
3.239253	3.397886	3.422665	35.18166
3.304624	3.361292	3.298769	34.97364

3.287632	3.239848	3.497999	35.10659
3.204567	3.206488	3.414583	34.66957
3.301077	3.211273	3.444679	34.9569
3.285858	3.311488	3.392842	35.02941
3.373707	3.353204	3.432606	35.39971
3.285611	3.415274	3.411872	35.29745
3.349167	3.417633	3.407916	35.43295
3.337206	3.386497	3.35156	35.21546
3.40732	3.41925	3.294079	35.31471
3.245769	3.407524	3.448976	35.27452
3.300376	3.44641	3.352856	35.26877
3.149466	3.343904	3.395741	34.80838
3.258349	3.220236	3.338465	34.65079
3.294519	3.191392	3.430679	34.86847
3.269608	3.375512	3.34046	35.01934
3.259009	3.411567	3.505451	35.43581
3.364468	3.442097	3.393047	35.48739
3.229643	3.34451	3.414122	35.02523
3.353786	3.341073	3.313569	35.0693
3.188895	3.361831	3.463487	35.08195
3.163571	3.390069	3.377683	34.90069
3.131154	3.367627	3.464714	34.97104
3.239748	3.335615	3.394888	34.98582
3.245522	3.251642	3.428156	34.88756
3.274351	3.231963	3.442701	34.93938
3.222797	3.330695	3.46069	35.08189
3.317451	3.343837	3.444406	35.28201
3.429138	3.393438	3.362763	35.45618
3.347311	3.390136	3.400259	35.35201
3.337041	3.404828	3.310108	35.16454
3.280167	3.41743	3.403192	35.27128
3.279177	3.43037	3.339232	35.15754
3.319513	3.405502	3.388392	35.29887
3.31312	3.342017	3.370181	35.10624
3.143238	3.236546	3.417942	34.60853
3.278146	3.231222	3.357648	34.76006
3.277816	3.340871	3.449163	35.19925
3.329122	3.474109	3.438182	35.5788
3.270268	3.410017	3.398418	35.22298
3.345496	3.330762	3.451005	35.32918
3.271382	3.31169	3.404215	35.02307
3.37416	3.404019	3.365832	35.3658
3.234263	3.450521	3.380139	35.19285
3.293859	3.327325	3.437841	35.17995
3.089168	3.243083	3.347093	34.34965
3.254101	3.192201	3.382816	34.67718
3.228901	3.256157	3.391273	34.78043
3.342939	3.309668	3.459411	35.29584
3.218549	3.390877	3.451005	35.18303
3.387111	3.365605	3.384504	35.35095

3.34995	3.352059	3.370675	35.20982
3.449223	3.458338	3.338396	35.58874
3.317904	3.4671	3.388255	35.42976
3.19471	3.403952	3.325028	34.88399
3.173717	3.27368	3.37811	34.66928
3.308789	3.311622	3.284377	34.84266
3.215373	3.233513	3.414651	34.75245
3.253812	3.275836	3.37313	34.83826
3.173346	3.340399	3.497266	35.07495
3.351476	3.418576	3.423535	35.47422
3.409011	3.435492	3.416152	35.62088
3.329164	3.367424	3.353282	35.15993
3.268701	3.328875	3.413901	35.07597
3.248492	3.34451	3.39216	35.01842
3.306686	3.405232	3.364997	35.21907
3.278929	3.370524	3.392978	35.14366
3.316584	3.275499	3.384521	34.99971
3.103892	3.168209	3.424046	34.38639
3.28458	3.212891	3.405989	34.83975
3.230221	3.298346	3.459411	35.02458
3.273774	3.40685	3.346513	35.11022
3.249069	3.35745	3.416373	35.10093
3.309284	3.339928	3.397446	35.15291
3.325658	3.405637	3.409041	35.35776
3.466752	3.48907	3.301224	35.61299
3.291055	3.345319	3.428224	35.19214
3.286683	3.308792	3.329939	34.88776
3.214383	3.260066	3.362354	34.69399
3.336835	3.235063	3.330894	34.83829
3.181966	3.299963	3.369174	34.72526
3.262061	3.356844	3.414412	35.12373
3.217105	3.397549	3.433544	35.15628
3.371974	3.428416	3.453597	35.6063
3.295262	3.410017	3.375296	35.22708
3.431901	3.417498	3.437091	35.67738
3.32306	3.38744	3.394121	35.27966
3.270722	3.360887	3.432044	35.19007
3.207784	3.340804	3.37354	34.88058
3.20432	3.268288	3.374972	34.71755
3.280868	3.214104	3.389023	34.79719
3.19038	3.245644	3.390915	34.67242
3.223663	3.263503	3.493856	35.00937
3.238552	3.451734	3.430168	35.31429
3.29056	3.428213	3.437637	35.39292
3.284497	3.366144	3.369839	35.09566
3.346362	3.385149	3.447612	35.44259
3.317574	3.424372	3.306322	35.15643
3.294313	3.409545	3.432044	35.34807
3.351476	3.416285	3.330979	35.2668
3.354116	3.326516	3.314746	35.04077

3.140599	3.252585	3.340988	34.46955
3.313945	3.249351	3.399185	34.96882
3.165675	3.288574	3.438148	34.81556
3.273279	3.40038	3.372909	35.15271
3.242717	3.393236	3.457348	35.25491
3.344671	3.38306	3.438881	35.41523
3.232695	3.373422	3.429861	35.12955
3.367809	3.412376	3.374409	35.38894
3.293488	3.383329	3.462736	35.35605
3.37548	3.410085	3.326256	35.29541
3.200649	3.253664	3.423262	34.78315
3.195865	3.192335	3.282194	34.33008
3.200732	3.241735	3.383293	34.66984
3.210506	3.268153	3.341142	34.65681
3.209228	3.315936	3.435676	34.96523
3.239995	3.382521	3.398145	35.09605
3.286848	3.356507	3.476736	35.31349
3.439696	3.4146	3.332053	35.45839
3.370448	3.382318	3.413781	35.41509
3.28759	3.481387	3.414583	35.45229
3.267629	3.414735	3.376132	35.17879
3.276496	3.313038	3.354101	34.92761
3.377748	3.290865	3.349787	35.09111
3.184276	3.224213	3.364519	34.55448
3.241645	3.301042	3.376524	34.8742
3.194463	3.344241	3.452505	35.03165
3.255585	3.397886	3.412008	35.19407
3.220487	3.399436	3.421369	35.14117
3.335928	3.362572	3.395621	35.2567
3.21063	3.352328	3.446367	35.07126
3.414084	3.439603	3.387249	35.57776
3.351848	3.442434	3.423705	35.52757
3.277651	3.322742	3.319418	34.87551
3.221106	3.18937	3.414191	34.66745
3.20168	3.230413	3.387232	34.65577
3.248079	3.283856	3.403619	34.90994
3.277444	3.362033	3.347212	35.02176
3.181389	3.365739	3.470393	35.08919
3.223952	3.396471	3.434789	35.17161
3.204815	3.363179	3.424149	35.03369
3.369252	3.415139	3.350554	35.34598
3.332793	3.436031	3.374239	35.36373
3.323513	3.420598	3.357972	35.27411
3.262184	3.313442	3.387505	34.97025
3.300335	3.249553	3.311608	34.74799
3.31943	3.269434	3.331405	34.87651
3.265195	3.332919	3.357307	34.95338
3.130865	3.322271	3.372585	34.66975
3.214095	3.374838	3.457143	35.15163
3.315265	3.378679	3.415487	35.29018

3.264576	3.387171	3.411872	35.19
3.347063	3.379286	3.456547	35.45084
3.239995	3.425248	3.456888	35.31795
3.29621	3.430572	3.429435	35.39249
3.331556	3.374838	3.386635	35.25431
3.376099	3.251507	3.397258	35.10525
3.154085	3.170972	3.380957	34.40797
3.207042	3.202916	3.372653	34.57548
3.227952	3.318092	3.370522	34.86842
3.248945	3.359809	3.359268	34.98094
3.256988	3.426192	3.408853	35.25213
3.25307	3.354822	3.434908	35.14447
3.196772	3.375309	3.429196	35.05367
3.407114	3.438323	3.404915	35.59835
3.327019	3.452206	3.396525	35.43521
3.397257	3.332851	3.346104	35.21753
3.282641	3.204534	3.326068	34.64246
3.264948	3.201299	3.349667	34.64831
3.272083	3.31014	3.350537	34.90383
3.330566	3.380229	3.303901	35.08301
3.196979	3.362842	3.376183	34.91092
3.188854	3.34788	3.427303	34.97223
3.272	3.385486	3.466829	35.32273
3.313244	3.404491	3.353385	35.2064
3.334732	3.402604	3.437091	35.43231
3.209805	3.43684	3.467664	35.30085
3.212527	3.343028	3.447458	35.05746
3.268618	3.292954	3.349667	34.85677
3.291632	3.187888	3.382662	34.74949
3.268165	3.232704	3.410644	34.85737
3.161056	3.250429	3.415128	34.6717
3.167572	3.316812	3.372704	34.73835
3.325452	3.399908	3.340801	35.19555
3.294313	3.423429	3.393865	35.29494
3.308377	3.395595	3.409894	35.29988
3.212321	3.418981	3.40737	35.13544
3.311017	3.450319	3.428156	35.46526
3.394328	3.416487	3.350861	35.40443
3.454832	3.335143	3.314729	35.27984
3.238717	3.179598	3.391768	34.63555
3.326772	3.241668	3.33178	34.83267
3.264659	3.34505	3.402596	35.07778
3.313945	3.408332	3.307823	35.1167
3.237273	3.332919	3.390608	34.96515
3.132433	3.351519	3.383907	34.7619
3.138165	3.361965	3.504666	35.06136
3.352136	3.480983	3.352191	35.45612
3.298932	3.429966	3.503677	35.55948
3.298767	3.375309	3.380258	35.16969
3.212898	3.267479	3.439273	34.87516

3.288993	3.190314	3.328916	34.63148
3.343929	3.224684	3.351373	34.87589
3.301489	3.218282	3.357886	34.78333
3.11511	3.291135	3.37886	34.58093
3.224117	3.362437	3.382134	34.9824
3.313491	3.408939	3.365815	35.24385
3.31213	3.420598	3.374716	35.28584
3.308253	3.39728	3.41269	35.30941
3.159571	3.401728	3.497198	35.17879
3.305366	3.437581	3.406552	35.3778
3.329947	3.358057	3.35879	35.1532
3.438417	3.288506	3.28685	35.08099
3.179368	3.262964	3.374904	34.6512
3.280249	3.307579	3.321788	34.85322
3.264247	3.393169	3.401402	35.1795
3.401546	3.44149	3.281052	35.32223
3.191081	3.274623	3.387607	34.73009
3.136598	3.357652	3.422444	34.8687
3.169304	3.448701	3.376933	35.0398
3.26569	3.404963	3.412844	35.23347
3.307841	3.363583	3.3895	35.1841
3.295138	3.312162	3.408171	35.0847
3.220529	3.272534	3.390745	34.79678
3.332587	3.240252	3.41269	35.01923
3.301654	3.222797	3.354084	34.78525
3.279135	3.258044	3.400259	34.91406
3.194174	3.358461	3.384657	34.91374
3.20865	3.369177	3.387778	34.97566
3.211579	3.387103	3.38198	35.00858
3.25109	3.40375	3.409365	35.19128
3.256328	3.4208	3.472217	35.37747
3.273733	3.474176	3.418334	35.41442
3.251709	3.354283	3.408598	35.08278
3.297654	3.266603	3.39378	34.95911
3.375563	3.260066	3.332872	34.98199
3.294973	3.370053	3.319861	35.01782
3.261524	3.362909	3.397753	35.09939
3.297489	3.447353	3.353351	35.2656
3.284043	3.319575	3.354578	34.95945
3.211579	3.361561	3.324329	34.82665
3.289281	3.472424	3.489951	35.6012
3.19273	3.454362	3.428616	35.21644
3.230344	3.389125	3.422103	35.14178
3.205805	3.232704	3.424268	34.75079
3.26899	3.272062	3.40176	34.92581
3.24478	3.269569	3.348559	34.75107
3.378986	3.25892	3.416629	35.17013
3.216074	3.220169	3.419101	34.73453
3.355065	3.332919	3.33531	35.10181
3.197597	3.383194	3.44519	35.10769

3.396844	3.448769	3.326955	35.42825
3.156189	3.382453	3.442905	35.01052
3.200443	3.363044	3.426724	35.02946
3.225478	3.419452	3.510549	35.39088
3.418249	3.405434	3.306783	35.33618
3.275341	3.249485	3.490548	35.08449
3.271835	3.202849	3.373608	34.71911
3.305449	3.232165	3.408598	34.93325
3.361993	3.443714	3.306118	35.29542
3.3264	3.40375	3.353947	35.23478
3.2063	3.334469	3.369652	34.85498
3.192895	3.288843	3.363053	34.71146
3.196277	3.431448	3.410081	35.13355
3.255214	3.533685	3.378365	35.41665
3.273568	3.447218	3.465942	35.45922
3.152889	3.23048	3.437585	34.65933
3.304665	3.200625	3.457024	34.96846
3.425591	3.288843	3.346581	35.1843
3.262432	3.275499	3.361945	34.83192
3.314069	3.260268	3.370658	34.93059
3.204774	3.307848	3.404625	34.8699
3.305078	3.328066	3.370522	35.05889
3.307346	3.361157	3.338482	35.06615
3.373294	3.396067	3.4264	35.47897
3.038563	3.333795	3.424012	34.60557
3.292869	3.400043	3.326477	35.09327
3.282394	3.477815	3.401555	35.40462
3.365994	3.309803	3.4434	35.31154
3.202629	3.213767	3.404625	34.65947
3.246017	3.217945	3.432521	34.8245
3.252203	3.368637	3.332599	34.94905
3.408475	3.451734	3.333349	35.47415
3.283878	3.389125	3.383754	35.17499
3.336546	3.270714	3.323971	34.90049
3.100469	3.349969	3.39598	34.71501
3.316708	3.566505	3.351219	35.56354
3.169799	3.561383	3.443639	35.43318
3.259627	3.341815	3.431992	35.12399
3.176233	3.219832	3.454501	34.72408
3.313038	3.276241	3.461389	35.16167
3.282889	3.352328	3.45641	35.25125
3.400845	3.334401	3.402323	35.35171
3.309614	3.249216	3.372329	34.90033
3.217683	3.300368	3.419749	34.91485
3.292581	3.368166	3.379525	35.13894
3.239212	3.415476	3.348559	35.05797
3.306315	3.382857	3.409058	35.26569
3.223787	3.361157	3.399509	35.01687
3.215868	3.417633	3.444508	35.22147
3.250306	3.301379	3.392228	34.92822

3.423281	3.263705	3.404625	35.25121
3.209022	3.266536	3.43796	34.86175
3.262102	3.378342	3.427678	35.19985
3.311676	3.436435	3.390591	35.35419
3.335845	3.382723	3.356352	35.21471
3.341289	3.266064	3.34283	34.94193
3.308913	3.340669	3.389295	35.13589
3.142702	3.503492	3.412554	35.17935
3.319719	3.488261	3.441439	35.59631
3.117255	3.344645	3.386021	34.7183
3.255957	3.189438	3.337731	34.5766
3.199453	3.211004	3.449521	34.74467
3.310398	3.386227	3.416493	35.29824
3.208898	3.337906	3.439751	35.02147
3.340506	3.304411	3.375637	35.09582
3.271258	3.245779	3.435386	34.94683
3.384966	3.380162	3.295768	35.18404
3.258967	3.341815	3.418351	35.09271
3.224818	3.426731	3.354271	35.0636
3.150002	3.38124	3.342591	34.77497
3.313697	3.414937	3.38077	35.29012
3.230262	3.337636	3.379116	34.935
3.332009	3.252855	3.35185	34.91248
3.185265	3.211947	3.466948	34.75381
3.370325	3.390203	3.425666	35.45805
3.388348	3.444051	3.427201	35.61858
3.359436	3.369716	3.351151	35.22648
3.314316	3.288304	3.398929	35.05426
3.223168	3.289382	3.431975	34.92956
3.306521	3.42572	3.409058	35.35987
3.300912	3.495944	3.427764	35.54208
3.144476	3.339523	3.368203	34.72766
3.148476	3.168276	3.434056	34.50593
3.263009	3.243218	3.343103	34.72138
3.274805	3.310005	3.397958	35.01319
3.352342	3.360011	3.45612	35.4193
3.20366	3.309398	3.43675	34.94111
3.305201	3.32409	3.408785	35.13414
3.404845	3.373692	3.363309	35.36107
3.390039	3.390203	3.391461	35.42636
3.219951	3.367424	3.413355	35.05247
3.248203	3.360415	3.44768	35.17399
3.218384	3.348689	3.36527	34.90292
3.304748	3.346532	3.307039	34.95972
3.32574	3.228122	3.409143	34.96997
3.161757	3.219563	3.418402	34.6129
3.145053	3.320923	3.379832	34.71368
3.345331	3.39546	3.435812	35.43708
3.408681	3.443175	3.385254	35.56939
3.436685	3.367222	3.407984	35.51424

3.183574	3.298144	3.392279	34.77532
3.300128	3.374838	3.341687	35.08729
3.376305	3.424574	3.380548	35.44762
3.258101	3.382992	3.338806	35.00692
3.128061	3.242342	3.492713	34.75153
3.136268	3.210599	3.369157	34.42986
3.281857	3.293022	3.468278	35.14525
3.341207	3.418374	3.352055	35.295
3.325411	3.258584	3.484153	35.1999
3.183286	3.255888	3.402255	34.7041
3.283837	3.340939	3.433885	35.17915
3.403031	3.395258	3.333298	35.33863
3.45112	3.376253	3.367589	35.47722
3.195205	3.322068	3.394684	34.85834
3.207331	3.366076	3.396116	34.98422
3.274227	3.420396	3.3607	35.17186
3.352672	3.365537	3.407984	35.32684
3.215002	3.251709	3.351253	34.65279
3.241191	3.245644	3.45682	34.92765
3.083642	3.350104	3.469574	34.83945
3.449801	3.420328	3.383668	35.60589
3.330731	3.359607	3.44855	35.3546
3.35226	3.310274	3.35509	35.08941
3.205351	3.315396	3.49834	35.09261
3.280496	3.527687	3.395877	35.49712
3.314234	3.47303	3.454961	35.58058
3.298809	3.332784	3.271622	34.83922
3.120596	3.213295	3.450135	34.57857
3.083064	3.27961	3.380838	34.48997
3.216033	3.401863	3.466505	35.23544
3.303139	3.412511	3.465465	35.44694
3.290189	3.32126	3.367538	35.00492
3.200649	3.228728	3.475849	34.84362
3.218301	3.341276	3.370522	34.89801
3.40501	3.358596	3.41813	35.4483
3.314192	3.434346	3.366753	35.303
3.247131	3.343837	3.415674	35.0654
3.220858	3.285069	3.35925	34.75604
3.277651	3.280149	3.382679	34.92071
3.36286	3.316542	3.339709	35.09267
3.221312	3.35098	3.441678	35.08142
3.277527	3.438053	3.445293	35.40267
3.231211	3.398223	3.385953	35.08452
3.38608	3.343432	3.351714	35.2285
3.422333	3.302659	3.335583	35.18334
3.399814	3.327325	3.398435	35.32548
3.19005	3.427472	3.437705	35.17164
3.331391	3.456923	3.389364	35.43942
3.245605	3.255416	3.326119	34.67285
3.262019	3.156886	3.423876	34.70706

3.125174	3.292617	3.413389	34.68169
3.209434	3.418172	3.465465	35.2544
3.206753	3.443984	3.442087	35.25386
3.312089	3.327932	3.394326	35.12598
3.270309	3.22583	3.404591	34.83378
3.370655	3.239174	3.386976	35.04389
3.278599	3.343837	3.419903	35.14346
3.285817	3.465482	3.415128	35.41482
3.317286	3.399571	3.38771	35.27954
3.435695	3.271119	3.272714	35.0061
3.170954	3.230346	3.440416	34.70473
3.18774	3.336895	3.334918	34.74374
3.144847	3.324629	3.466027	34.90983
3.275176	3.483139	3.409945	35.41883
3.330236	3.453823	3.412485	35.48068
3.247172	3.398021	3.433698	35.22339
3.305655	3.335008	3.367248	35.06817
3.38047	3.384138	3.432998	35.48301
3.29188	3.446005	3.362269	35.26989
3.292787	3.4301	3.366361	35.24604
3.194958	3.23075	3.410166	34.69195
3.118822	3.131681	3.374682	34.23121
3.312171	3.220236	3.398776	34.90038
3.247708	3.386632	3.366378	35.05244
3.275795	3.48752	3.478987	35.58075
3.155364	3.322271	3.466147	34.92793
3.324338	3.271658	3.405767	35.05473
3.35127	3.346532	3.352379	35.16061
3.370283	3.42882	3.388204	35.46048
3.205598	3.382925	3.53408	35.31899
3.331391	3.415004	3.403687	35.37908
3.237397	3.294167	3.495339	35.10971
3.376099	3.272669	3.264802	34.86186
3.301489	3.227178	3.417106	34.93229
3.107893	3.24834	3.362678	34.43617
3.170954	3.349632	3.401624	34.88076
3.319224	3.398088	3.427354	35.36724
3.292168	3.45079	3.381571	35.3232
3.413259	3.39391	3.41385	35.5342
3.223746	3.345724	3.400208	34.98456
3.329782	3.388249	3.377001	35.25869
3.419899	3.495472	3.419511	35.78321
3.389668	3.347813	3.393456	35.33721
3.229437	3.174409	3.366566	34.5488
3.196855	3.153921	3.306271	34.30089
3.23583	3.339523	3.40251	35.00246
3.331515	3.471143	3.302861	35.28162
3.236407	3.390136	3.476787	35.27684
3.112883	3.240724	3.471467	34.66834
3.217023	3.250159	3.401948	34.76468

3.384471	3.345926	3.414498	35.36773
3.348713	3.383734	3.41963	35.38344
3.252327	3.508345	3.459002	35.53126
3.228241	3.356911	3.469148	35.16962
3.246924	3.20514	3.383191	34.6906
3.460524	3.262492	3.279807	35.05705
3.238304	3.304479	3.428088	34.98717
3.192648	3.442838	3.436409	35.20809
3.137712	3.386429	3.410763	34.90852
3.359601	3.357585	3.363837	35.22806
3.306439	3.349295	3.361058	35.08759
3.292168	3.353878	3.397139	35.14531
3.176109	3.401863	3.414583	35.03459
3.388101	3.460225	3.479106	35.76691
3.280002	3.384003	3.343938	35.06824
3.414909	3.181957	3.365355	34.96825
3.246801	3.154393	3.377751	34.56746
3.170583	3.262223	3.410235	34.70763
3.257441	3.443579	3.406347	35.28567
3.296994	3.444792	3.402391	35.36616
3.187905	3.325034	3.431839	34.93011
3.215786	3.268827	3.412059	34.82491
3.1738	3.304411	3.478151	34.95544
3.385007	3.380364	3.393729	35.3988
3.404103	3.409411	3.443093	35.61203
3.223003	3.434279	3.448976	35.28324
3.26037	3.246655	3.3873	34.81978
3.350404	3.168276	3.343938	34.75044
3.296664	3.228863	3.362217	34.80539
3.345909	3.337704	3.341773	35.10638
3.184482	3.43037	3.430748	35.15059
3.215043	3.410893	3.360836	35.02195
3.327926	3.362168	3.477367	35.41708
3.312996	3.383262	3.352686	35.1579
3.310357	3.404491	3.545965	35.62123
3.206341	3.453284	3.382253	35.14245
3.299922	3.443107	3.437978	35.44671
3.290354	3.27341	3.344006	34.84918
3.411733	3.141521	3.369157	34.8812
3.198051	3.20669	3.459224	34.75339
3.267381	3.448364	3.336367	35.16483
3.235376	3.432527	3.514386	35.44951
3.322688	3.409006	3.330843	35.18763
3.246553	3.197794	3.422239	34.75912
3.251214	3.335143	3.379661	34.97656
3.156849	3.387777	3.509731	35.16974
3.329659	3.450049	3.403738	35.45204
3.254142	3.431111	3.454637	35.35679
3.416641	3.303603	3.360785	35.22807
3.287054	3.15008	3.39644	34.68692

3.328298	3.241061	3.416356	35.01963
3.230839	3.345589	3.403619	35.00724
3.253235	3.413791	3.403278	35.20461
3.168067	3.469863	3.359745	35.04579
3.26004	3.367963	3.424609	35.16593
3.196442	3.358461	3.422716	35.00193
3.277238	3.404828	3.473189	35.39039
3.336752	3.472289	3.404693	35.51828
3.322276	3.445803	3.4412	35.50853
3.344547	3.323147	3.408666	35.21786
3.243501	3.202108	3.431651	34.78246
3.319595	3.200692	3.351833	34.77122
3.317121	3.389529	3.363292	35.20382
3.26136	3.479567	3.405665	35.37145
3.229107	3.398291	3.419647	35.15375
3.280496	3.253799	3.428019	34.96846
3.238717	3.254675	3.366514	34.74451
3.261566	3.377062	3.432606	35.20665
3.252286	3.434144	3.450732	35.35082
3.229148	3.453621	3.483215	35.41385
3.28227	3.255079	3.343051	34.78933
3.414373	3.191594	3.418078	35.10346
3.242635	3.223741	3.358534	34.66798
3.282311	3.353137	3.430611	35.19533
3.28095	3.446073	3.397753	35.32374
3.383811	3.422418	3.392961	35.48647
3.31576	3.395932	3.35451	35.19564
3.314728	3.318362	3.396645	35.1159
3.224282	3.388923	3.461048	35.21325
3.247089	3.472828	3.399475	35.31196
3.261071	3.372209	3.492543	35.32603
3.358323	3.311151	3.353487	35.10108
3.301448	3.14024	3.446742	34.80689
3.304789	3.250968	3.349037	34.84267
3.317533	3.399234	3.455029	35.42656
3.337742	3.536313	3.353419	35.54832
3.260782	3.380162	3.390711	35.1201
3.255957	3.305759	3.348355	34.85421
3.145053	3.221854	3.394138	34.52832
3.261153	3.385284	3.397804	35.14762
3.279465	3.469795	3.470444	35.53133
3.280868	3.424776	3.408035	35.29947
3.308088	3.272467	3.380821	34.96641
3.262514	3.155134	3.444406	34.74921
3.365829	3.217945	3.366242	34.94156
3.297324	3.324832	3.397804	35.09452
3.206423	3.456519	3.336231	35.04906
3.233149	3.429022	3.358125	35.09526
3.354528	3.342286	3.339283	35.12981
3.291756	3.316812	3.353368	34.96763

3.340836	3.424507	3.40974	35.43375
3.189802	3.506053	3.482312	35.44049
3.187163	3.356304	3.474826	35.09088
3.238346	3.268086	3.402459	34.85163
3.383028	3.163087	3.405324	34.94468
3.25905	3.274017	3.440706	34.99352
3.32937	3.33056	3.370897	35.11829
3.287343	3.44277	3.474366	35.49804
3.36653	3.363246	3.344058	35.21233
3.239047	3.275365	3.351782	34.75826
3.314687	3.32719	3.364059	35.06385
3.246099	3.339928	3.399577	35.01939
3.229025	3.405569	3.385186	35.09413
3.180894	3.373894	3.414327	34.98333
3.315265	3.349565	3.422699	35.24228
3.309449	3.237085	3.449436	35.04206
3.262349	3.269569	3.428667	34.96468
3.306439	3.277993	3.441047	35.10659
3.299303	3.361224	3.362422	35.10106
3.306026	3.356035	3.387164	35.15852
3.255792	3.369918	3.355226	35.00918
3.166087	3.344578	3.436409	34.93513
3.262226	3.430303	3.381094	35.21187
3.311553	3.455104	3.397992	35.41093
3.262762	3.459484	3.393661	35.30434
3.227622	3.359944	3.457212	35.14879
3.169345	3.271119	3.459889	34.83296
3.357333	3.222595	3.328438	34.85048
3.386657	3.2879	3.349071	35.10254
3.310645	3.434683	3.378024	35.32063
3.270887	3.394314	3.436358	35.27297
3.32838	3.359539	3.436954	35.32395
3.269361	3.295044	3.316366	34.79014
3.346981	3.269164	3.336845	34.94807
3.158004	3.324023	3.393251	34.77812
3.205268	3.447758	3.46896	35.31764
3.163695	3.410624	3.466573	35.1403
3.284621	3.30987	3.449914	35.14798
3.315182	3.213699	3.371067	34.83208
3.350693	3.203118	3.395161	34.93928
3.198298	3.400178	3.461389	35.18179
3.355559	3.45234	3.388596	35.48058
3.403608	3.467369	3.38834	35.61796
3.342898	3.405502	3.302656	35.16252
3.232242	3.356304	3.424353	35.07908
3.249935	3.379083	3.375705	35.0612
3.279218	3.449712	3.4109	35.35666
3.341042	3.381847	3.338141	35.18433
3.201227	3.213025	3.432657	34.71609
3.240903	3.160526	3.401982	34.62096

3.260081	3.305085	3.456802	35.09891
3.370943	3.472693	3.345643	35.4648
3.348796	3.467706	3.429878	35.58967
3.243831	3.370187	3.3622	34.99887
3.290477	3.303131	3.430441	35.10346
3.23983	3.314992	3.394547	34.94015
3.3078	3.36082	3.422853	35.25091
3.228406	3.376522	3.44918	35.1692
3.239253	3.376859	3.445617	35.18586
3.145589	3.228324	3.418351	34.59659
3.3914	3.201299	3.360853	34.9493
3.263917	3.210801	3.407745	34.79384
3.261153	3.38461	3.347331	35.03577
3.257441	3.429831	3.496516	35.45279
3.375026	3.513669	3.306288	35.47727
3.432808	3.407658	3.335327	35.43531
3.43054	3.38124	3.268894	35.22729
3.142661	3.3497	3.44502	34.91393
3.273114	3.458878	3.324977	35.17545
3.214177	3.429966	3.349667	35.03734
3.300623	3.207769	3.35376	34.74942
3.215951	3.145901	3.382134	34.49101
3.265278	3.213565	3.469233	34.93732
3.222591	3.469728	3.450255	35.36266
3.381955	3.468043	3.464373	35.73835
3.319307	3.372614	3.356471	35.1567
3.282806	3.279408	3.411514	34.99342
3.261277	3.333458	3.435249	35.11644
3.2282	3.384475	3.414395	35.11007
3.347682	3.400245	3.34653	35.25743
3.278723	3.381914	3.421608	35.23073
3.277114	3.320923	3.416834	35.08339
3.291467	3.195099	3.459889	34.93378
3.255214	3.189842	3.357221	34.61848
3.306851	3.284732	3.334867	34.89003
3.265979	3.356776	3.411445	35.12566
3.203206	3.456182	3.416919	35.21774
3.338485	3.416487	3.388511	35.36465
3.350981	3.377196	3.265637	35.03735
3.399566	3.38771	3.407558	35.47694
3.2647	3.427135	3.360308	35.1649
3.206011	3.459754	3.45878	35.32323
3.136928	3.328808	3.384913	34.72427
3.269484	3.182698	3.403755	34.73583
3.156313	3.223808	3.398912	34.56765
3.318399	3.379353	3.483011	35.44617
3.302644	3.494596	3.447185	35.58539
3.380759	3.353339	3.381997	35.30475
3.373583	3.306972	3.409126	35.24699
3.404103	3.281093	3.348013	35.1235

3.280785	3.338917	3.46642	35.23921
3.202134	3.41278	3.35538	34.98591
3.16518	3.368098	3.449641	35.01352
3.334195	3.363516	3.31877	35.08691
3.27365	3.240994	3.473854	35.02572
3.191864	3.250429	3.399577	34.70507
3.154828	3.26384	3.497658	34.86789
3.347393	3.371333	3.359745	35.22248
3.342114	3.417026	3.434567	35.47448
3.480981	3.404423	3.330553	35.52314
3.351146	3.344039	3.414242	35.29017
3.289529	3.382992	3.371954	35.14813
3.330978	3.388721	3.364468	35.23493
3.338361	3.440883	3.380616	35.40046
3.221395	3.337839	3.393643	34.94782
3.16419	3.171174	3.43247	34.54316
3.270887	3.229806	3.327194	34.67449
3.244285	3.388586	3.427286	35.18243
3.34166	3.486239	3.406006	35.56239
3.215786	3.425855	3.477009	35.31034
3.260741	3.33211	3.406688	35.04986
3.308336	3.33676	3.436971	35.23034
3.422786	3.370255	3.342369	35.34699
3.290684	3.372075	3.429111	35.25177
3.143032	3.364729	3.391614	34.83082
3.187369	3.301513	3.445463	34.9073
3.333412	3.221113	3.293056	34.71756
3.246718	3.234322	3.366855	34.71824
3.25274	3.284665	3.375449	34.8603
3.177305	3.347611	3.479532	35.0606
3.328916	3.438457	3.410013	35.43879
3.434087	3.454295	3.366975	35.6093
3.534267	3.439603	3.405989	35.88156
3.325658	3.406445	3.364673	35.26251
3.285941	3.374433	3.403414	35.19037
3.223952	3.365942	3.340596	34.89886
3.389874	3.33238	3.344313	35.19644
3.265236	3.263908	3.391938	34.87829
3.086652	3.207432	3.429264	34.44588
3.170088	3.343769	3.450527	34.97299
3.345579	3.438188	3.4124	35.47986
3.342898	3.441894	3.466215	35.59978
3.26037	3.371738	3.445429	35.22043
3.222673	3.328673	3.461577	35.07913
3.333948	3.388721	3.387027	35.29076
3.412599	3.416824	3.36365	35.47309
3.289859	3.314722	3.384299	35.02656
3.167325	3.277252	3.402476	34.7164
3.225436	3.273949	3.345558	34.71179
3.332711	3.236681	3.369072	34.9163

3.2753	3.327123	3.321464	34.88443
3.25076	3.322068	3.375091	34.93698
3.145548	3.360281	3.424336	34.89815
3.37449	3.41588	3.420517	35.51205
3.356178	3.427337	3.369379	35.38523
3.453595	3.387036	3.392484	35.56066
3.264824	3.415948	3.44959	35.33595
3.344135	3.38097	3.332394	35.17662
3.277981	3.360685	3.409621	35.15647
3.318152	3.315194	3.340784	34.9943
3.146455	3.180339	3.423995	34.50589
3.183327	3.274825	3.367435	34.66946
3.143816	3.410489	3.47694	35.1192
3.346733	3.4239	3.419153	35.4659
3.377872	3.398358	3.451704	35.54933
3.363107	3.326112	3.372721	35.18633
3.236448	3.341613	3.496857	35.21471
3.349249	3.457125	3.394053	35.48917
3.29984	3.370727	3.429401	35.26948
3.315883	3.2879	3.308522	34.8591
3.167613	3.273275	3.391597	34.68455
3.202629	3.229672	3.394087	34.67121
3.31279	3.306703	3.407762	35.11047
3.212486	3.352261	3.395366	34.96364
3.296746	3.346532	3.409212	35.16566
3.292416	3.39701	3.467187	35.39336
3.374944	3.419183	3.362593	35.39359
3.412929	3.372748	3.416919	35.49392
3.448893	3.464	3.343972	35.6126
3.241604	3.368974	3.397531	35.06861
3.364963	3.352193	3.328984	35.15177
3.264453	3.247868	3.396559	34.85161
3.303923	3.248946	3.316383	34.76495
3.207372	3.246587	3.390728	34.71123
3.257524	3.405637	3.39835	35.18539
3.171573	3.421878	3.4508	35.14764
3.304459	3.375444	3.373062	35.1667
3.222013	3.348285	3.394769	34.97448
3.371974	3.349228	3.415333	35.34945
3.26404	3.395393	3.447936	35.28567
3.373129	3.388047	3.448072	35.50846
3.254802	3.288372	3.363701	34.84722
3.268082	3.239781	3.359012	34.75975
3.19339	3.209319	3.329001	34.46417
3.285487	3.24834	3.354834	34.80739
3.146992	3.353743	3.336282	34.69445
3.221395	3.373085	3.465209	35.1814
3.319142	3.451734	3.369857	35.35863
3.355106	3.382453	3.404011	35.36046
3.432767	3.428887	3.386362	35.59325

3.331844	3.455036	3.367111	35.38763
3.374243	3.413859	3.377564	35.41316
3.373459	3.397347	3.297404	35.20004
3.35127	3.248811	3.337459	34.91428
3.228076	3.195503	3.350333	34.55645
3.2136	3.217136	3.398418	34.67726
3.196442	3.4146	3.331286	34.92475
3.328916	3.448903	3.407199	35.45548
3.247914	3.400312	3.439018	35.24166
3.229643	3.375175	3.43866	35.14595
3.261566	3.379218	3.402783	35.14615
3.338567	3.427674	3.457689	35.54057
3.259132	3.442231	3.420039	35.31636
3.410207	3.376388	3.413986	35.48951
3.2063	3.219091	3.421403	34.71583
3.349703	3.254607	3.380446	35.01754
3.240779	3.21653	3.362763	34.6574
3.339557	3.320114	3.331832	35.03229
3.1088	3.293898	3.38152	34.57899
3.210135	3.393034	3.379116	35.01213
3.254967	3.433133	3.407984	35.26099
3.433922	3.453419	3.329598	35.52528
3.285941	3.39263	3.436545	35.30261
3.35259	3.396673	3.385271	35.34508
3.311718	3.383801	3.39569	35.25033
3.387482	3.31971	3.282604	35.02856
3.26668	3.27833	3.405989	34.94371
3.146084	3.220034	3.360922	34.45395
3.136722	3.271051	3.387113	34.60232
3.200773	3.432729	3.369857	35.05822
3.221518	3.419452	3.407899	35.15774
3.297241	3.331167	3.391188	35.09373
3.238222	3.326516	3.43559	35.05159
3.178337	3.35772	3.452096	35.02496
3.434747	3.494664	3.444679	35.86895
3.323307	3.435492	3.40841	35.41653
3.348383	3.254472	3.381094	35.01577
3.205145	3.192335	3.4412	34.69809
3.294519	3.255686	3.378928	34.8959
3.231417	3.287495	3.393814	34.86002
3.346073	3.372142	3.408717	35.32845
3.146249	3.333525	3.462941	34.9256
3.255585	3.392225	3.375569	35.102
3.297571	3.428887	3.4146	35.35934
3.382863	3.409411	3.361808	35.38782
3.256946	3.388653	3.403209	35.15761
3.300582	3.382049	3.417396	35.26962
3.257483	3.324023	3.406824	35.02535
3.377377	3.290596	3.29454	34.96889
3.260823	3.25117	3.377223	34.80861

3.330978	3.288843	3.375552	35.04075
3.167778	3.358057	3.359421	34.79995
3.266804	3.402334	3.394411	35.18984
3.205227	3.432324	3.429264	35.19699
3.338361	3.414398	3.386294	35.35496
3.230179	3.389799	3.437227	35.17597
3.356673	3.425989	3.471006	35.60561
3.328628	3.372883	3.415964	35.30777
3.389132	3.280486	3.425717	35.25936
3.324049	3.210801	3.368492	34.8395
3.214837	3.217002	3.41876	34.72416
3.239624	3.247935	3.383208	34.76826
3.19071	3.403615	3.343239	34.91433
3.32574	3.393101	3.405307	35.32237
3.271712	3.354283	3.393831	35.09423
3.222879	3.445534	3.416288	35.2361
3.242676	3.45416	3.371545	35.20041
3.349208	3.434818	3.409553	35.4742
3.29122	3.395056	3.357153	35.14584
3.328669	3.292617	3.399049	35.09534
3.292003	3.27058	3.345848	34.85063
3.261813	3.242881	3.3991	34.84049
3.287632	3.316273	3.341346	34.93114
3.35523	3.379555	3.383719	35.31002
3.138537	3.400582	3.393456	34.90342
3.316048	3.395797	3.428633	35.35808
3.147486	3.382857	3.402578	34.90418
3.338773	3.429764	3.377956	35.37123
3.265484	3.390743	3.467528	35.3215
3.301407	3.394854	3.436272	35.3407
3.255379	3.310477	3.437773	35.05881
3.34364	3.215721	3.358995	34.87233
3.315842	3.18472	3.415998	34.8684
3.339681	3.279341	3.364809	35.01551
3.21129	3.269299	3.443264	34.88435
3.22787	3.437379	3.303679	34.98292
3.202051	3.343702	3.378843	34.88598
3.305902	3.412309	3.336367	35.17023
3.264205	3.396336	3.433067	35.25558
3.332793	3.425787	3.388375	35.37224
3.319471	3.40503	3.380684	35.2809
3.358859	3.372546	3.3238	35.1716
3.322771	3.299559	3.384521	35.06585
3.273114	3.229065	3.408922	34.85646
3.277774	3.275297	3.390114	34.92663
3.203165	3.283654	3.387573	34.77619
3.379811	3.342354	3.359557	35.22958
3.139691	3.378746	3.453324	34.98912
3.224405	3.388451	3.409007	35.09868
3.191741	3.406647	3.426246	35.10474

3.325575	3.44459	3.37666	35.37196
3.225849	3.425383	3.463691	35.30219
3.330648	3.402739	3.420585	35.38758
3.179986	3.257236	3.489473	34.89057
3.411609	3.231896	3.341551	35.01819
3.302232	3.220236	3.428497	34.94364
3.327143	3.296459	3.382799	35.06487
3.261071	3.297807	3.404096	34.9699
3.229148	3.385823	3.374546	35.02795
3.183739	3.362842	3.36943	34.8672
3.380429	3.443377	3.318753	35.36263
3.226921	3.418307	3.481425	35.32784
3.260576	3.470604	3.415077	35.37071
3.20465	3.36237	3.427883	35.03973
3.401711	3.27961	3.35301	35.12595
3.364757	3.235265	3.363923	34.97203
3.265071	3.307781	3.426246	35.0489
3.238428	3.337165	3.431293	35.06593
3.287095	3.335345	3.378229	35.05234
3.348589	3.373153	3.348355	35.20416
3.270722	3.323686	3.38996	35.01669
3.122452	3.394921	3.407285	34.88611
3.223292	3.404693	3.439137	35.19766
3.335845	3.473637	3.349582	35.39872
3.247584	3.356911	3.377632	35.01179
3.333865	3.276847	3.394939	35.06323
3.208238	3.212958	3.417311	34.69771
3.331721	3.283856	3.393319	35.07033
3.368551	3.258314	3.366088	35.03546
3.372469	3.317688	3.351185	35.14128
3.192813	3.308522	3.415197	34.86834
3.297489	3.377938	3.371204	35.15284
3.225973	3.36237	3.37857	34.97851
3.271629	3.482196	3.392143	35.37008
3.206712	3.459686	3.37591	35.14339
3.26371	3.363448	3.433408	35.18332
3.130247	3.276308	3.445548	34.72745
3.385007	3.244498	3.387795	35.08871
3.35028	3.311083	3.361109	35.10002
3.3297	3.339995	3.368578	35.13457
3.271299	3.347948	3.348235	34.97976
3.266597	3.318429	3.384589	34.98443
3.371603	3.370727	3.345626	35.24322
3.32937	3.405367	3.357494	35.25257
3.184688	3.457665	3.419971	35.18716
3.169304	3.453823	3.456461	35.22492
3.265484	3.372816	3.472285	35.2927
3.2428	3.334334	3.392007	34.98339
3.33832	3.252922	3.434652	35.1075
3.228942	3.196514	3.444594	34.76669

3.224199	3.227313	3.443315	34.82088
3.355188	3.302996	3.333707	35.03314
3.292704	3.375646	3.432675	35.2718
3.316749	3.439603	3.329615	35.23887
3.256905	3.391282	3.382117	35.11714
3.280331	3.417498	3.337919	35.12905
3.359643	3.435155	3.362337	35.3945
3.287178	3.437783	3.368475	35.2552
3.361622	3.313981	3.41876	35.25723
3.183904	3.209117	3.408802	34.61749
3.292045	3.235872	3.390728	34.87296
3.313821	3.338984	3.431958	35.23624
3.502304	3.437851	3.422	35.84285
3.251709	3.339928	3.442155	35.12477
3.319554	3.333525	3.355942	35.0706
3.254142	3.326516	3.420517	35.05344
3.357622	3.422822	3.323323	35.27779
3.20366	3.470469	3.404505	35.22283
3.150621	3.432459	3.423518	35.0653
3.118616	3.277184	3.462907	34.74189
3.330442	3.244026	3.345371	34.87557
3.292622	3.207162	3.447867	34.93639
3.37317	3.229604	3.393882	35.04356
3.251007	3.27496	3.475576	35.05425
3.285899	3.335951	3.389295	35.07525
3.373005	3.409411	3.34005	35.31869
3.373335	3.38097	3.302077	35.17417
3.175202	3.342758	3.371476	34.80909
3.192401	3.420126	3.381741	35.03834
3.323885	3.479972	3.408188	35.51458
3.274269	3.38461	3.370948	35.1161
3.333206	3.268558	3.309784	34.85744
3.132886	3.207903	3.461389	34.61827
3.202175	3.32436	3.397395	34.88452
3.320049	3.39418	3.419732	35.34382
3.363066	3.348756	3.313365	35.10595
3.278022	3.307174	3.427013	35.07757
3.244079	3.297537	3.411531	34.94841
3.294354	3.404895	3.44357	35.3632
3.36554	3.475861	3.345763	35.46017
3.286559	3.418441	3.382748	35.24276
3.216775	3.315194	3.506388	35.13476
3.179533	3.288776	3.378502	34.71587
3.307016	3.280149	3.406534	35.0371
3.328792	3.307646	3.346513	35.01359
3.303057	3.27058	3.42038	35.03779
3.19867	3.303198	3.436221	34.91548
3.403649	3.353406	3.400686	35.39583
3.332587	3.37504	3.360222	35.19925
3.352755	3.347206	3.359353	35.18058

3.238676	3.394382	3.343529	34.99967
3.171284	3.43064	3.36249	34.97305
3.188482	3.395393	3.370044	34.9501
3.292457	3.260673	3.332156	34.80001
3.254802	3.229874	3.409075	34.81852
3.238552	3.33029	3.430083	35.04852
3.275588	3.387171	3.457365	35.31357
3.312666	3.407861	3.326529	35.15377
3.428065	3.336288	3.390165	35.38878
3.276785	3.317283	3.33514	34.89606
3.228282	3.371603	3.471552	35.20709
3.310109	3.489946	3.383174	35.45157
3.277403	3.382184	3.446981	35.28392
3.222096	3.34478	3.345285	34.85878
3.255957	3.261279	3.446367	34.97128
3.128927	3.274825	3.413918	34.65215
3.284002	3.250766	3.403602	34.9161
3.250636	3.276847	3.458866	35.02102
3.293818	3.331773	3.400686	35.10834
3.267505	3.366009	3.401163	35.12671
3.365417	3.418037	3.349991	35.3427
3.298767	3.354283	3.418215	35.20672
3.341166	3.307916	3.303543	34.94727
3.16386	3.388249	3.437091	35.02725
3.324627	3.512321	3.354868	35.47034
3.230303	3.345521	3.401555	35.00141
3.234675	3.300233	3.297882	34.68521
3.269278	3.216665	3.417516	34.83975
3.261731	3.417902	3.372431	35.16473
3.269113	3.443175	3.432692	35.36792
3.337	3.37996	3.376524	35.25531
3.28891	3.252248	3.432521	34.99331
3.369129	3.376118	3.350486	35.26022
3.311841	3.456249	3.369243	35.3512
3.280826	3.406378	3.427542	35.3018
3.191122	3.370727	3.359421	34.8787
3.214672	3.253529	3.396082	34.75408
3.271959	3.28945	3.377973	34.91831
3.260617	3.258651	3.423057	34.92475
3.162747	3.285137	3.469813	34.87089
3.287095	3.264716	3.469011	35.09641
3.411733	3.409748	3.356403	35.43987
3.358776	3.375983	3.367862	35.27529
3.353539	3.351519	3.346257	35.16309
3.230427	3.331436	3.44843	35.07338
3.282682	3.404558	3.391683	35.22346
3.286147	3.436368	3.335822	35.17844
3.434293	3.360281	3.289817	35.23542
3.11808	3.228863	3.384589	34.46378
3.19306	3.207971	3.414992	34.64854

3.167118	3.419722	3.437176	35.10339
3.302768	3.462854	3.457962	35.53981
3.251337	3.392832	3.372244	35.08676
3.365994	3.240522	3.431396	35.13378
3.143486	3.348891	3.426178	34.87276
3.429921	3.484892	3.332684	35.59211
3.363849	3.483274	3.414617	35.62326
3.365004	3.305085	3.331883	35.05518
3.144641	3.17077	3.394479	34.41645
3.168356	3.316138	3.398623	34.79527
3.211414	3.38744	3.403619	35.05628
3.322152	3.354485	3.415077	35.25144
3.161922	3.280689	3.423074	34.75715
3.28825	3.349228	3.400123	35.1331
3.348754	3.380836	3.419494	35.37689
3.484074	3.445331	3.381111	35.72992
3.283425	3.360483	3.351987	35.04189
3.204526	3.290865	3.370658	34.75794
3.251049	3.396269	3.464237	35.29482
3.275259	3.335817	3.267189	34.78466
3.328298	3.268019	3.361604	34.95885
3.227746	3.2323	3.359609	34.65649
3.121792	3.311488	3.472319	34.84443
3.327308	3.466224	3.443229	35.56863
3.418538	3.447016	3.358722	35.54133
3.281404	3.311892	3.404079	35.04513
3.302355	3.351115	3.379405	35.12277
3.294602	3.451397	3.477264	35.53911
3.390905	3.469526	3.386976	35.59191
3.304665	3.370659	3.380377	35.17268
3.360839	3.201636	3.410644	34.99209
3.086199	3.18809	3.423535	34.39006
3.267505	3.354148	3.354084	34.99781
3.1811	3.343971	3.424967	34.94161
3.263628	3.294167	3.379951	34.91473
3.152394	3.278262	3.450886	34.79182
3.299468	3.377129	3.440638	35.30725
3.300706	3.415678	3.401419	35.30849
3.428313	3.378746	3.332906	35.35695
3.22919	3.300974	3.384862	34.86505
3.341619	3.39263	3.309562	35.14668
3.252987	3.368166	3.331661	34.94768
3.242841	3.273141	3.372363	34.8067
3.207619	3.299087	3.329615	34.69293
3.210424	3.267614	3.399918	34.78398
3.204609	3.419857	3.389722	35.0819
3.294189	3.401728	3.391751	35.24259
3.2972	3.392697	3.423518	35.29889
3.348383	3.295852	3.347127	35.03198
3.254348	3.343702	3.456461	35.17008

3.214425	3.525193	3.413781	35.38633
3.34463	3.452408	3.404301	35.49117
3.236119	3.266199	3.42258	34.88664
3.339227	3.163087	3.354237	34.73717
3.192978	3.250496	3.430355	34.77496
3.166912	3.39209	3.392893	34.94568
3.21162	3.334132	3.439836	35.01936
3.268453	3.287967	3.356778	34.86105
3.258472	3.289248	3.446248	35.03768
3.350651	3.393506	3.383907	35.33093
3.284291	3.393438	3.416237	35.25636
3.332876	3.402671	3.376387	35.29565
3.322853	3.425855	3.406415	35.3901
3.429715	3.394247	3.35347	35.43889
3.316626	3.296931	3.392365	35.06382
3.273031	3.255079	3.309818	34.69645
3.097788	3.261077	3.405085	34.53467
3.257689	3.425046	3.377837	35.18333
3.259421	3.443512	3.392279	35.25909
3.227829	3.393236	3.425649	35.15303
3.213228	3.258449	3.416765	34.80691
3.340093	3.316542	3.431566	35.24375
3.332339	3.486913	3.380889	35.48855
3.390204	3.484959	3.430952	35.7203
3.241934	3.260605	3.37857	34.79088
3.206465	3.128514	3.372636	34.41147
3.25542	3.286687	3.337714	34.78806
3.273691	3.445264	3.408188	35.32892
3.219621	3.382925	3.454705	35.17607
3.184894	3.232233	3.399816	34.65056
3.240903	3.256225	3.481306	35.00371
3.349084	3.366818	3.341483	35.17636
3.357952	3.395191	3.424899	35.44022
3.19071	3.449982	3.388136	35.11391
3.251049	3.389125	3.425359	35.19418
3.312749	3.30987	3.303901	34.89018
3.449801	3.263975	3.373523	35.24178
3.205392	3.20723	3.322368	34.47134
3.181512	3.290596	3.451141	34.88303
3.166665	3.4177	3.401624	35.02023
3.375851	3.457867	3.332889	35.41521
3.317822	3.365268	3.392024	35.21513
3.27695	3.314588	3.416032	35.06743
3.125957	3.322742	3.470665	34.87454
3.32772	3.51933	3.373403	35.53297
3.410949	3.481926	3.448004	35.79632
3.341537	3.288304	3.304566	34.90743
3.183987	3.147317	3.470103	34.61658
3.325493	3.280554	3.374631	35.00862
3.189637	3.423024	3.427406	35.13849

3.379934	3.464269	3.350674	35.47704
3.169139	3.224078	3.426741	34.65715
3.133092	3.302726	3.449982	34.80114
3.184894	3.341545	3.397258	34.88401
3.339227	3.418913	3.42154	35.4438
3.29753	3.444186	3.394871	35.34957
3.323307	3.403547	3.441132	35.41823
3.210135	3.266469	3.34815	34.66764
3.387688	3.248879	3.328029	34.97345
3.404062	3.208308	3.260914	34.77376
3.222384	3.306433	3.432726	34.96677
3.130453	3.435627	3.368697	34.90824
3.255627	3.424844	3.412161	35.25344
3.320668	3.419857	3.303748	35.14769
3.367231	3.332447	3.342233	35.14253
3.249976	3.367963	3.488979	35.28469
3.109584	3.496551	3.433698	35.13798
3.319719	3.504975	3.418948	35.58368
3.317409	3.308185	3.347485	34.992
3.336216	3.136197	3.424012	34.82437
3.238799	3.197592	3.399987	34.69306
3.277857	3.341006	3.458797	35.2207
3.265071	3.477276	3.304634	35.15361
3.316254	3.308657	3.405239	35.11681
3.227457	3.253799	3.367862	34.72092
3.271588	3.253125	3.390182	34.86476
3.209558	3.390069	3.402016	35.05446
3.276496	3.461776	3.402937	35.35967
3.303139	3.427203	3.38568	35.30459
3.325122	3.342084	3.37059	35.13353
3.252987	3.296459	3.429588	35.00502
3.338072	3.288843	3.354851	35.011
3.264082	3.259325	3.424541	34.93704
3.318317	3.386632	3.33323	35.13436
3.120967	3.349498	3.450425	34.87787
3.349579	3.399975	3.32305	35.20965
3.199783	3.315464	3.408188	34.88344
3.29122	3.346802	3.396338	35.12601
3.202794	3.445601	3.470239	35.31031
3.341042	3.474311	3.451755	35.63499
3.219992	3.327325	3.442974	35.02964
3.342733	3.156415	3.428309	34.89223
3.360261	3.229335	3.360359	34.94143
3.281239	3.370659	3.361297	35.07973
3.28227	3.433403	3.468551	35.45374
3.169799	3.343297	3.458047	34.98777
3.304005	3.288506	3.336623	34.8959
3.313862	3.30805	3.397787	35.09395
3.301613	3.411365	3.421591	35.34515
3.186668	3.414802	3.403482	35.0617

3.213723	3.414735	3.397258	35.10711
3.339145	3.317149	3.455455	35.29525
3.35758	3.316812	3.327569	35.05516
3.248492	3.296526	3.471876	35.08782
3.26602	3.27651	3.325812	34.76296
3.248244	3.283991	3.369157	34.83523
3.332876	3.376455	3.375927	35.23732
3.257111	3.412106	3.404795	35.21273
3.261112	3.412039	3.399799	35.2104
3.222714	3.379892	3.365338	34.98077
3.268165	3.394584	3.39141	35.16931
3.251626	3.446814	3.402101	35.27074
3.368634	3.370457	3.432657	35.42646
3.255874	3.288776	3.39112	34.91041
3.20135	3.18445	3.386345	34.55259
3.340918	3.238096	3.347945	34.89114
3.330813	3.350172	3.315223	35.04258
3.246347	3.355698	3.411889	35.08134
3.226426	3.313577	3.328387	34.76306
3.270186	3.285878	3.403039	34.96144
3.319018	3.417498	3.322675	35.18031
3.256452	3.374635	3.434976	35.19534
3.107109	3.389664	3.477486	34.99458
3.223044	3.374501	3.393319	35.03089
3.395524	3.332582	3.473786	35.49238
3.331102	3.377331	3.368902	35.21999
3.294684	3.309331	3.37371	35.00216
3.298272	3.233985	3.425837	34.95923
3.214672	3.295852	3.414395	34.88668
3.406908	3.418037	3.381452	35.50223
3.247997	3.376186	3.359182	35.01449
3.343104	3.365537	3.395485	35.27858
3.115812	3.343365	3.371357	34.68028
3.250471	3.396808	3.389977	35.13235
3.219621	3.464471	3.45491	35.35485
3.310027	3.363381	3.408785	35.23061
3.243377	3.215519	3.409143	34.7623
3.34133	3.181755	3.379047	34.83685
3.312089	3.346398	3.394479	35.1667
3.43553	3.492777	3.385544	35.73721
3.239583	3.367694	3.388392	35.0414
3.182461	3.243218	3.358654	34.57924
3.214631	3.229874	3.367538	34.63984
3.288621	3.375849	3.408495	35.21044
3.219003	3.414263	3.418607	35.16431
3.219951	3.458136	3.466641	35.36737
3.145053	3.293022	3.416987	34.73392
3.416888	3.165985	3.365338	34.93762
3.395483	3.273612	3.399646	35.2012
3.240738	3.325303	3.383174	34.93981

3.285487	3.47822	3.377973	35.3607
3.261937	3.431314	3.398299	35.25108
3.414166	3.338378	3.306715	35.18046
3.339186	3.357585	3.324499	35.09739
3.208692	3.363987	3.429605	35.05587
3.110037	3.413319	3.386226	34.85314
3.331102	3.42909	3.418812	35.44232
3.189225	3.34168	3.439512	34.98618
3.217188	3.138353	3.36781	34.44589
3.182997	3.131007	3.420738	34.4708
3.291055	3.378073	3.403448	35.20958
3.313161	3.468852	3.418283	35.48889
3.378738	3.441288	3.408512	35.55065
3.293447	3.290461	3.404488	35.0255
3.230015	3.230346	3.361672	34.66168
3.217848	3.284732	3.372977	34.77874
3.305449	3.430303	3.363769	35.26851
3.160189	3.473974	3.449658	35.23418
3.188194	3.368705	3.407694	34.97344
3.209022	3.163424	3.418812	34.59438
3.367231	3.176902	3.269849	34.64408
3.372428	3.271388	3.383668	35.11098
3.3914	3.380836	3.345439	35.30821
3.293736	3.418846	3.460622	35.42964
3.321204	3.43812	3.277488	35.13137
3.376387	3.335412	3.380889	35.25357
3.437758	3.442973	3.285485	35.41436
3.230262	3.479163	3.4796	35.46424
3.128514	3.467504	3.462327	35.17846
3.232489	3.329819	3.34994	34.85897
3.300912	3.145429	3.442701	34.80823
3.24746	3.149271	3.383208	34.56963
3.229437	3.295178	3.432743	34.95762
3.276702	3.43037	3.417175	35.32258
3.269691	3.377331	3.43438	35.22888
3.423487	3.408871	3.360632	35.47291
3.272495	3.25582	3.387454	34.86667
3.316337	3.376118	3.401896	35.2572
3.197391	3.402941	3.42686	35.11034
3.304418	3.418374	3.419852	35.36281
3.123978	3.401458	3.381179	34.84665
3.280373	3.350778	3.330843	34.96776
3.225931	3.245172	3.484426	34.95362
3.377006	3.299357	3.389142	35.19412
3.191741	3.266873	3.450527	34.85218
3.353621	3.376455	3.378723	35.2888
3.322152	3.377129	3.460008	35.39921
3.34397	3.383936	3.33473	35.18785
3.292911	3.377196	3.384095	35.1694
3.273361	3.416352	3.385953	35.21634

3.23319	3.442434	3.389773	35.19388
3.212486	3.434481	3.477827	35.32378
3.204774	3.386429	3.457297	35.15693
3.293488	3.196244	3.462958	34.94741
3.340836	3.170567	3.328183	34.70007
3.243625	3.330358	3.437023	35.07494
3.341495	3.441894	3.451278	35.56405
3.353951	3.367357	3.423313	35.36714
3.279878	3.361292	3.364656	35.06361
3.370448	3.244296	3.33057	34.93128
3.343681	3.313914	3.386073	35.14637
3.121173	3.410489	3.406381	34.91538
3.29023	3.454362	3.423313	35.41805
3.1632	3.426461	3.413764	35.05836
3.303634	3.253596	3.316298	34.7743
3.217971	3.177509	3.387368	34.576
3.243212	3.28763	3.402715	34.90557
3.198834	3.38306	3.439819	35.09836
3.395978	3.475793	3.36793	35.57506
3.345372	3.398762	3.395502	35.35624
3.417053	3.298615	3.376916	35.25334
3.252657	3.332447	3.435727	35.09643
3.340258	3.477748	3.332838	35.38074
3.231829	3.471615	3.373403	35.21893
3.258472	3.417026	3.435454	35.29351
3.166995	3.192605	3.43409	34.5997
3.227457	3.179261	3.427218	34.68772
3.322853	3.318699	3.410166	35.16397
3.428519	3.490418	3.410985	35.77236
3.342155	3.406041	3.385953	35.34424
3.331061	3.337232	3.447936	35.30505
3.285033	3.314588	3.433476	35.12325
3.286724	3.260201	3.375023	34.88019
3.305325	3.340736	3.37383	35.09437
3.128721	3.359607	3.418164	34.84638
3.175656	3.374703	3.549648	35.26957
3.21162	3.299626	3.377274	34.80708
3.33535	3.196379	3.335003	34.75944
3.244161	3.197929	3.336913	34.56759
3.271134	3.327729	3.493617	35.25311
3.196154	3.426259	3.363701	35.02051
3.450461	3.437581	3.317935	35.50131
3.357622	3.381307	3.453716	35.47216
3.326194	3.335075	3.36764	35.11409
3.260741	3.426596	3.389977	35.21995
3.288498	3.43064	3.369038	35.2437
3.291055	3.39546	3.394684	35.22844
3.257194	3.274623	3.384998	34.86896
3.133051	3.148664	3.409348	34.37528
3.296086	3.23385	3.38759	34.87052

3.246966	3.387036	3.434226	35.20008
3.41561	3.445871	3.414225	35.6538
3.275753	3.290663	3.381042	34.93597
3.397174	3.310477	3.423057	35.33671
3.245275	3.33521	3.435164	35.08509
3.338113	3.385014	3.326955	35.16039
3.13136	3.334604	3.432998	34.82992
3.225519	3.372748	3.357238	34.95357
3.103438	3.357922	3.407506	34.7641
3.210465	3.327527	3.376814	34.86457
3.353002	3.259392	3.350196	34.96906
3.253688	3.230211	3.402732	34.80295
3.270763	3.311757	3.422785	35.06247
3.25109	3.356844	3.387846	35.04164
3.395524	3.456788	3.356079	35.50659
3.449388	3.416285	3.351987	35.52686
3.325658	3.343904	3.394479	35.19092
3.233273	3.466021	3.435727	35.34614
3.2793	3.478287	3.453205	35.51184
3.227746	3.377736	3.402237	35.06775
3.263628	3.316946	3.416441	35.04435
3.136969	3.20824	3.48949	34.68939
3.209599	3.260471	3.478373	34.93812
3.227457	3.391147	3.46069	35.22428
3.341289	3.398695	3.3622	35.27433
3.284043	3.368098	3.448004	35.26987
3.402165	3.327662	3.334253	35.191
3.243542	3.214845	3.453102	34.85731
3.29819	3.388519	3.312973	35.05017
3.252368	3.391147	3.448157	35.25135
3.301324	3.41925	3.32733	35.15563
3.213888	3.32126	3.415401	34.94273
3.227375	3.246924	3.337595	34.63952
3.253111	3.217945	3.454961	34.88908
3.300046	3.363246	3.344484	35.06788
3.319389	3.400515	3.434891	35.38938
3.269196	3.462584	3.394274	35.32653
3.358735	3.373759	3.418624	35.38134
3.407444	3.383464	3.31258	35.27718
3.28095	3.346869	3.333571	34.96644
3.211909	3.432864	3.398367	35.14521
3.222055	3.406243	3.363769	35.03352
3.211537	3.238702	3.402988	34.7299
3.254719	3.198064	3.355738	34.63214
3.184152	3.269434	3.396917	34.72395
3.309367	3.498707	3.397923	35.50136
3.168438	3.453217	3.524548	35.37059
3.398989	3.376253	3.371493	35.37176
3.34265	3.366279	3.452812	35.40457
3.349579	3.356844	3.397344	35.27779

3.267257	3.291067	3.406927	34.97488
3.333535	3.364998	3.39141	35.24756
3.158045	3.381712	3.463077	35.05707
3.328133	3.308859	3.300559	34.91431
3.162664	3.17205	3.41194	34.49684
3.111316	3.203792	3.375586	34.37447
3.175986	3.266132	3.434994	34.78213
3.231994	3.459484	3.424967	35.30552
3.392266	3.473502	3.336009	35.49213
3.370613	3.361696	3.435045	35.41685
3.250183	3.342556	3.367316	34.96352
3.361086	3.367155	3.411462	35.35638
3.325534	3.491833	3.32421	35.36048
3.296416	3.371603	3.380429	35.15682
3.184812	3.24962	3.357903	34.59674
3.169469	3.139229	3.338243	34.27879
3.207413	3.284395	3.391478	34.79564
3.332257	3.417633	3.393592	35.36464
3.278352	3.457867	3.470938	35.50389
3.217353	3.394719	3.378314	35.02985
3.300541	3.289922	3.450323	35.14006
3.425632	3.434144	3.420193	35.66312
3.369046	3.405502	3.402937	35.439
3.202876	3.318766	3.41992	34.92308
3.21925	3.285271	3.397685	34.83701
3.21661	3.284328	3.433834	34.90823
3.308707	3.266738	3.401794	35.0011
3.25773	3.22765	3.317611	34.62004
3.129463	3.226504	3.449044	34.62446
3.09985	3.351115	3.416032	34.76002
3.373418	3.444051	3.445736	35.62646
3.372016	3.451262	3.352805	35.43594
3.418662	3.386429	3.392672	35.48335
3.330195	3.375983	3.451721	35.39617
3.35923	3.443647	3.413628	35.52433
3.287549	3.412848	3.386209	35.24027
3.432437	3.360415	3.283985	35.2189
3.132226	3.16248	3.474843	34.54691
3.169964	3.181418	3.280489	34.24583
3.130948	3.287765	3.471825	34.8115
3.306109	3.488126	3.357511	35.38272
3.316997	3.420328	3.467647	35.49911
3.186008	3.364931	3.453341	35.06023
3.2176	3.349498	3.4759	35.1449
3.431736	3.366548	3.386618	35.45522
3.324173	3.315194	3.388136	35.11102
3.237108	3.34141	3.357511	34.91098
3.170542	3.360415	3.486898	35.08992
3.226715	3.293156	3.344075	34.75334
3.348631	3.212554	3.344603	34.84484

3.29984	3.249148	3.364349	34.86135
3.152766	3.298615	3.486813	34.91571
3.192772	3.402536	3.452659	35.15577
3.359189	3.396808	3.353811	35.291
3.490797	3.413117	3.387096	35.68726
3.318317	3.383801	3.330826	35.12291
3.190627	3.327595	3.466437	35.01732
3.323101	3.436907	3.354766	35.30186
3.276496	3.339052	3.411326	35.10964
3.345455	3.247261	3.292715	34.80033
3.205433	3.115372	3.396593	34.43287
3.177223	3.317216	3.427815	34.88085
3.195453	3.475793	3.470597	35.36106
3.365953	3.482668	3.391853	35.57675
3.224117	3.389732	3.40367	35.08918
3.308336	3.301918	3.404915	35.08404
3.244656	3.374366	3.446657	35.1945
3.348754	3.395528	3.359063	35.27687
3.413135	3.351654	3.3526	35.30759
3.323678	3.285406	3.380207	35.02745
3.231664	3.259055	3.362269	34.72938
3.249399	3.193818	3.333997	34.56368
3.225272	3.297335	3.366173	34.80765
3.260865	3.373894	3.363292	35.04661
3.193308	3.413926	3.457689	35.19285
3.22457	3.424709	3.454569	35.27797
3.376965	3.426326	3.41356	35.52509
3.392307	3.349295	3.407489	35.37691
3.3151	3.421946	3.435045	35.42721
3.251874	3.468784	3.39187	35.29695
3.243501	3.355293	3.437517	35.13028
3.24746	3.241196	3.313228	34.61763
3.364674	3.11827	3.30784	34.59335
3.228653	3.273141	3.383958	34.80103
3.190627	3.448162	3.453068	35.25175
3.220199	3.461641	3.427815	35.29067
3.309037	3.447892	3.432061	35.46416
3.341413	3.30704	3.342506	35.0311
3.320626	3.344308	3.470052	35.34607
3.192813	3.431314	3.387317	35.0759
3.294066	3.340669	3.434567	35.20242
3.233685	3.334738	3.35729	34.88842
3.384636	3.271927	3.430014	35.2402
3.254802	3.192538	3.387863	34.69049
3.223127	3.258381	3.370743	34.72777
3.158581	3.315262	3.400464	34.776
3.306026	3.374096	3.357801	35.13381
3.160932	3.383262	3.462736	35.06603
3.385543	3.447353	3.383941	35.52506
3.238139	3.402132	3.479072	35.31186

3.326565	3.450386	3.327074	35.27836
3.389297	3.424035	3.444184	35.61402
3.375645	3.407658	3.39939	35.45039
3.223663	3.267277	3.357682	34.71983
3.256988	3.194627	3.345984	34.60825
3.199082	3.21316	3.391734	34.6222
3.312666	3.418374	3.309136	35.13873
3.208939	3.45726	3.413355	35.22484
3.14926	3.421609	3.399867	34.98688
3.173346	3.332043	3.450869	34.95521
3.261112	3.356237	3.407285	35.10474
3.331143	3.417565	3.4065	35.39029
3.249234	3.353541	3.492491	35.25921
3.198505	3.279408	3.430475	34.85053
3.325988	3.214238	3.381145	34.87892
3.418538	3.206556	3.343563	34.98233
3.254678	3.2385	3.392348	34.80054
3.226179	3.320451	3.313314	34.74459
3.17149	3.361696	3.497266	35.11747
3.332422	3.446073	3.333162	35.29505
3.342032	3.454767	3.366583	35.40816
3.361004	3.418778	3.361928	35.36077
3.169304	3.357046	3.391393	34.87099
3.266515	3.389732	3.408717	35.19294
3.284002	3.384408	3.387658	35.17348
3.43784	3.369514	3.350946	35.39705
3.219827	3.16248	3.352669	34.47131
3.203495	3.163424	3.453733	34.65867
3.209187	3.324023	3.375722	34.85172
3.277444	3.437514	3.384862	35.26916
3.201515	3.382992	3.380429	34.97419
3.145795	3.365672	3.502194	35.08075
3.152312	3.367155	3.429452	34.93917
3.318564	3.380027	3.501904	35.48932
3.344506	3.433335	3.439001	35.52507
3.33535	3.340736	3.461372	35.35147
3.16188	3.285137	3.427354	34.77614
3.274145	3.25582	3.328933	34.74231
3.407774	3.231693	3.337203	34.99985
3.354817	3.32254	3.323851	35.05352
3.096015	3.281632	3.371152	34.50154
3.225024	3.332312	3.369567	34.89102
3.326978	3.435425	3.460025	35.53729
3.382574	3.505379	3.334833	35.53807
3.354611	3.420463	3.441268	35.52398
3.182915	3.379016	3.444935	35.06589
3.221312	3.368435	3.394411	35.01623
3.36752	3.324023	3.34549	35.13186
3.349538	3.292213	3.407012	35.15751
3.2103	3.245981	3.328097	34.57934

3.286518	3.246655	3.406057	34.91798
3.238469	3.421946	3.438728	35.26768
3.406289	3.463663	3.34653	35.52428
3.256328	3.361898	3.404352	35.10025
3.236737	3.350845	3.413713	35.05371
3.144187	3.365605	3.423586	34.90518
3.31378	3.46656	3.400191	35.44567
3.300747	3.364257	3.414804	35.2254
3.367561	3.285204	3.350691	35.05843
3.157674	3.218484	3.445276	34.66038
3.341289	3.257303	3.337527	34.91117
3.314151	3.258786	3.422273	35.0404
3.398535	3.368772	3.291453	35.17937
3.082239	3.37504	3.449163	34.84628
3.138743	3.428685	3.321703	34.80842
3.31576	3.424911	3.388357	35.33304
3.33832	3.453688	3.338345	35.33593
3.264824	3.422822	3.460298	35.3744
3.208815	3.402334	3.438591	35.15965
3.19306	3.340804	3.418283	34.94623
3.393957	3.27031	3.304344	34.98223
3.398865	3.235602	3.32675	34.96606
3.252038	3.235872	3.375057	34.7512
3.256122	3.349161	3.386959	35.03391
3.248657	3.43657	3.40471	35.24755
3.322029	3.427742	3.351015	35.27127
3.254884	3.411095	3.431327	35.26367
3.233768	3.402536	3.449163	35.23778
3.134907	3.334941	3.421744	34.8138
3.312625	3.419115	3.382799	35.30135
3.252616	3.262492	3.464373	35.006
3.301943	3.241937	3.426928	34.98704
3.162087	3.221517	3.454057	34.69586
3.32207	3.224415	3.370811	34.87001
3.2607	3.266401	3.414105	34.9223
3.345042	3.360752	3.342693	35.15691
3.20036	3.38744	3.454688	35.14379
3.221972	3.477209	3.377086	35.21766
3.269361	3.498572	3.387863	35.39157
3.316914	3.428416	3.354988	35.27025
3.285074	3.382318	3.43588	35.27671
3.283425	3.334469	3.399918	35.08982
3.246718	3.247666	3.408308	34.83807
3.298602	3.248609	3.409007	34.95513
3.377955	3.299559	3.363172	35.13985
3.250554	3.299155	3.344688	34.81993
3.235747	3.368098	3.401845	35.06332
3.252451	3.387642	3.43247	35.20956
3.288745	3.399504	3.363155	35.16328
3.25641	3.466224	3.388016	35.29285

3.232159	3.471278	3.488672	35.47098
3.150167	3.406513	3.501631	35.17839
3.303758	3.371064	3.409416	35.23509
3.261195	3.242679	3.440365	34.92893
3.286394	3.254944	3.391478	34.90395
3.214466	3.262627	3.445088	34.88069
3.31213	3.249418	3.436886	35.04745
3.243419	3.320047	3.404369	34.98053
3.396308	3.345993	3.3142	35.17443
3.243749	3.361561	3.387641	35.03546
3.293199	3.445466	3.385663	35.32276
3.180894	3.452071	3.437244	35.20441
3.294519	3.457193	3.357238	35.28913
3.257813	3.41487	3.381895	35.17022
3.299097	3.287967	3.385612	34.99112
3.275506	3.298413	3.386362	34.96401
3.328916	3.283317	3.387573	35.05045
3.343929	3.286687	3.344467	34.99638
3.337907	3.347948	3.352004	35.13366
3.234345	3.376118	3.413219	35.10266
3.231376	3.361292	3.403891	35.04335
3.284497	3.341613	3.397753	35.10306
3.212898	3.402536	3.403772	35.09288
3.26103	3.40995	3.448021	35.31111
3.160437	3.46373	3.500523	35.32355
3.176687	3.352665	3.454961	35.01657
3.232448	3.209925	3.414259	34.73735
3.319554	3.245779	3.364519	34.89747
3.247625	3.25454	3.412809	34.86494
3.300458	3.262695	3.428633	35.03291
3.201433	3.311757	3.450255	34.97093
3.352177	3.388047	3.336777	35.21926
3.301984	3.358191	3.371493	35.12013
3.277032	3.435222	3.45387	35.41416
3.180522	3.448095	3.426741	35.17193
3.334154	3.470065	3.361808	35.41395
3.193308	3.397482	3.355823	34.93412
3.32075	3.248811	3.382884	34.94688
3.187658	3.273073	3.417806	34.78525
3.368964	3.300637	3.382185	35.16412
3.282352	3.329482	3.394138	35.06393
3.370448	3.355361	3.352379	35.22186
3.349785	3.362505	3.403005	35.303
3.230509	3.371064	3.372534	34.99425
3.15557	3.358057	3.365611	34.78678
3.248657	3.470402	3.380429	35.26843
3.198958	3.381307	3.456342	35.13093
3.220776	3.405704	3.444389	35.20585
3.177058	3.306905	3.456461	34.92059
3.204444	3.214845	3.415401	34.68937

3.359024	3.261212	3.373693	35.0376
3.252987	3.257236	3.400873	34.85645
3.300335	3.298615	3.383293	35.01204
3.266144	3.393169	3.418624	35.22131
3.352507	3.417767	3.350401	35.31477
3.35725	3.419789	3.348235	35.32483
3.294148	3.459686	3.414225	35.41839
3.10216	3.419317	3.401913	34.88334
3.333659	3.412578	3.417106	35.40808
3.267505	3.336356	3.402527	35.06484
3.265236	3.226032	3.412673	34.84081
3.271505	3.232637	3.417754	34.88007
3.280744	3.301176	3.404898	35.02204
3.27662	3.343297	3.409399	35.11498
3.343599	3.345724	3.365355	35.17044
3.298314	3.369716	3.432862	35.27151
3.290684	3.406041	3.345336	35.14285
3.140846	3.411635	3.423876	34.99917
3.248162	3.514343	3.361604	35.32228
3.281198	3.439333	3.429315	35.37856
3.214878	3.358057	3.406944	35.00687
3.162911	3.250631	3.425376	34.69861
3.254884	3.19712	3.39794	34.72273
3.285116	3.189909	3.355994	34.68134
3.394411	3.239646	3.360274	35.03847
3.288993	3.300839	3.453887	35.14648
3.271464	3.408602	3.362217	35.14334
3.304748	3.382184	3.339078	35.10775
3.290519	3.402806	3.337203	35.11763
3.33964	3.455171	3.382594	35.43883
3.19669	3.495607	3.385118	35.22017
3.231623	3.381375	3.413781	35.10943
3.277156	3.311555	3.38169	34.98614
3.280455	3.224348	3.381861	34.80302
3.186296	3.298818	3.377751	34.75098
3.311305	3.333795	3.416049	35.1846
3.276001	3.353609	3.478833	35.28802
3.357539	3.37942	3.292016	35.11424
3.36851	3.290461	3.379815	35.13569
3.27963	3.360011	3.361655	35.05371
3.216115	3.444186	3.40292	35.18913
3.242965	3.46191	3.360632	35.19413
3.179533	3.405165	3.427951	35.07853
3.284332	3.33521	3.355533	34.99637
3.199618	3.228661	3.409672	34.6965
3.251214	3.229941	3.404932	34.80176
3.249151	3.237354	3.43779	34.88532
3.333206	3.325236	3.396116	35.17018
3.300541	3.314318	3.394257	35.07081
3.337495	3.398291	3.339863	35.2163

3.265773	3.329886	3.433698	35.11507
3.227251	3.377803	3.359131	34.97255
3.271423	3.441962	3.362064	35.21587
3.31943	3.421339	3.358227	35.26736
3.277444	3.300165	3.392689	34.98592
3.241686	3.248137	3.33647	34.671
3.353539	3.258853	3.374136	35.02141
3.277939	3.39209	3.384504	35.17013
3.346115	3.37787	3.373608	35.26429
3.29621	3.349835	3.404812	35.16209
3.266969	3.329684	3.378843	34.99728
3.246883	3.386092	3.360359	35.0363
3.425302	3.437177	3.341636	35.49724
3.137877	3.394517	3.43293	34.97504
3.220405	3.344174	3.34445	34.85193
3.131484	3.261953	3.436886	34.67982
3.264618	3.311825	3.367265	34.92777
3.184358	3.242544	3.466965	34.81878
3.307965	3.257842	3.463828	35.11568
3.166582	3.240859	3.461782	34.76488
3.442707	3.373085	3.39274	35.5069
3.334154	3.386632	3.369584	35.2485
3.418373	3.407254	3.356812	35.44984
3.260411	3.326314	3.395485	35.01197
3.198752	3.374838	3.320339	34.81891
3.273279	3.466291	3.35775	35.26369
3.380429	3.443916	3.264324	35.24478
3.283755	3.249148	3.461509	35.03865
3.205351	3.229537	3.359063	34.60028
3.237067	3.229469	3.451755	34.87219
3.32141	3.408804	3.393592	35.32162
3.382574	3.358596	3.332258	35.21145
3.281651	3.31607	3.493173	35.24965
3.247584	3.343837	3.345626	34.9132
3.300953	3.432796	3.430543	35.41015
3.408104	3.481657	3.325659	35.52196
3.270268	3.371872	3.475491	35.30811
3.087312	3.346802	3.445975	34.78865
3.132268	3.303535	3.466692	34.83764
3.342362	3.315464	3.377035	35.12711
3.186008	3.215721	3.410371	34.63997
3.267629	3.216732	3.46971	34.95043
3.183698	3.323416	3.451721	34.96085
3.42456	3.372411	3.381571	35.44132
3.409465	3.439603	3.33398	35.45117
3.44877	3.402604	3.355755	35.50383
3.219415	3.29282	3.441882	34.95053
3.233768	3.416554	3.325028	34.99697
3.220157	3.446612	3.383481	35.16076
3.36917	3.35125	3.320612	35.1406

3.155859	3.165985	3.387334	34.41489
3.136474	3.204736	3.442974	34.57892
3.278022	3.335345	3.419374	35.12247
3.404474	3.440614	3.457007	35.71151
3.346073	3.373422	3.322658	35.14306
3.298767	3.311825	3.430969	35.14176
3.186461	3.337906	3.43114	34.95357
3.295055	3.444051	3.381724	35.31511
3.312006	3.45234	3.393439	35.39592
3.251502	3.40658	3.29101	34.93955
3.198876	3.315329	3.450408	34.97349
3.096303	3.370659	3.41651	34.79604
3.307181	3.254944	3.425104	35.02294
3.238882	3.243622	3.381503	34.75348
3.284167	3.246048	3.426246	34.95566
3.251874	3.344039	3.428991	35.10533
3.289982	3.409074	3.392842	35.25184
3.396968	3.400717	3.343171	35.3589
3.427117	3.363044	3.388954	35.44257
3.152724	3.302929	3.44855	34.84138
3.260328	3.472491	3.400208	35.34178
3.28227	3.456451	3.356949	35.26009
3.357003	3.29902	3.323408	35.00589
3.18077	3.213497	3.385067	34.56831
3.172068	3.268693	3.427133	34.76198
3.198257	3.405839	3.409894	35.08147
3.477516	3.40503	3.366583	35.59568
3.288663	3.327258	3.443775	35.18142
3.364179	3.342286	3.371613	35.22162
3.262349	3.367559	3.456598	35.24005
3.302355	3.445399	3.339982	35.24274
3.262514	3.394112	3.361382	35.09025
3.249853	3.305355	3.37475	34.8977
3.145136	3.256225	3.421062	34.66254
3.18209	3.297065	3.388409	34.76126
3.270804	3.265323	3.367691	34.84054
3.272701	3.275297	3.405648	34.9495
3.283219	3.264447	3.449845	35.04543
3.186791	3.359135	3.471143	35.0882
3.313615	3.397415	3.396713	35.28649
3.329287	3.408467	3.29483	35.12213
3.381007	3.329616	3.383072	35.25577
3.25307	3.390608	3.39823	35.14252
3.21294	3.391282	3.354254	34.96007
3.275588	3.312768	3.374631	34.96993
3.346197	3.263571	3.342642	34.9468
3.21129	3.277184	3.395434	34.797
3.29122	3.416757	3.443451	35.38202
3.238016	3.479972	3.395758	35.29962
3.386822	3.35745	3.350145	35.25735

3.361251	3.284597	3.358347	35.06005
3.363643	3.394651	3.415435	35.43079
3.286312	3.468852	3.434772	35.46623
3.323266	3.447218	3.4161	35.4589
3.17281	3.277589	3.378587	34.67689
3.357622	3.257977	3.31611	34.90153
3.224488	3.244363	3.449095	34.87143
3.179945	3.372681	3.420653	34.99244
3.154993	3.285002	3.468108	34.84991
3.262143	3.27678	3.381111	34.876
3.274722	3.303603	3.463981	35.14339
3.436479	3.362437	3.383549	35.44989
3.243419	3.33986	3.445821	35.11451
3.3118	3.373153	3.349582	35.1264
3.359065	3.382453	3.356011	35.26415
3.294849	3.414735	3.43397	35.3648
3.23583	3.317216	3.354306	34.84826
3.279795	3.274219	3.309323	34.75201
3.268041	3.249081	3.416032	34.90469
3.231499	3.386834	3.35613	34.99503
3.361169	3.481454	3.404625	35.59156
3.188029	3.419452	3.425172	35.12228
3.383069	3.333795	3.385936	35.27568
3.236448	3.359876	3.402885	35.04915
3.428643	3.469324	3.399867	35.70219
3.283507	3.46346	3.446913	35.47486
3.370201	3.288843	3.312546	34.98875
3.188606	3.19894	3.447032	34.68912
3.269443	3.24342	3.328183	34.70326
3.163736	3.369918	3.413168	34.93458
3.261607	3.380431	3.357221	35.04925
3.136557	3.280486	3.466897	34.79707
3.246718	3.296189	3.402306	34.93106
3.366571	3.398897	3.408257	35.43078
3.358818	3.448701	3.389483	35.48168
3.262844	3.399908	3.41385	35.21839
3.292704	3.37996	3.412008	35.23604
3.317079	3.321529	3.391205	35.11607
3.41297	3.377668	3.316366	35.28487
3.241027	3.332582	3.339147	34.86008
3.128597	3.234052	3.372704	34.47213
3.258472	3.271119	3.3622	34.81424
3.245687	3.390743	3.402783	35.13662
3.373129	3.427674	3.387198	35.462
3.291508	3.367627	3.463436	35.31891
3.296416	3.294504	3.402578	35.03666
3.351559	3.375849	3.436699	35.40975
3.418909	3.527552	3.324431	35.64327
3.307263	3.443242	3.432265	35.45056
3.295427	3.224617	3.352652	34.77248

3.220487	3.2199	3.401965	34.70612
3.306397	3.266738	3.394905	34.98098
3.284291	3.420531	3.347144	35.16451
3.252987	3.310611	3.460827	35.10429
3.121297	3.2972	3.401709	34.65769
3.238057	3.306972	3.427218	34.99018
3.272619	3.361831	3.332053	34.97762
3.429591	3.388519	3.443519	35.62301
3.220776	3.408265	3.404761	35.12479
3.326483	3.351587	3.478151	35.3925
3.340299	3.387642	3.35821	35.23927
3.378944	3.339591	3.335532	35.16911
3.205145	3.209386	3.306033	34.43979
3.191864	3.268693	3.417737	34.78472
3.066237	3.36884	3.41772	34.72896
3.315471	3.446747	3.381912	35.36606
3.339268	3.42882	3.418266	35.4584
3.342032	3.353137	3.32554	35.09616
3.323761	3.323416	3.405852	35.16684
3.304005	3.463797	3.403414	35.4253
3.431489	3.524923	3.417311	35.86814
3.364344	3.314385	3.28112	34.96307
3.239872	3.223539	3.428582	34.81468
3.207331	3.191257	3.337817	34.47443
3.318358	3.352059	3.446861	35.30734
3.235211	3.397617	3.383685	35.08698
3.267793	3.290932	3.457655	35.08669
3.146249	3.299963	3.452523	34.82942
3.234758	3.326921	3.396252	34.95887
3.30285	3.360011	3.396713	35.18115
3.412805	3.450184	3.402186	35.63077
3.16254	3.413656	3.431515	35.06774
3.383894	3.359539	3.414668	35.39661
3.258266	3.273545	3.442905	34.99558
3.465473	3.322675	3.283422	35.20739
3.180935	3.200018	3.372841	34.51246
3.265979	3.273478	3.381571	34.87817
3.179533	3.395191	3.440927	35.0851
3.32904	3.420396	3.353385	35.27572
3.261442	3.397886	3.462549	35.3174
3.370283	3.376657	3.355499	35.27489
3.273485	3.406513	3.487171	35.41644
3.366406	3.465954	3.371937	35.49764
3.338113	3.425585	3.48267	35.58964
3.384677	3.245846	3.329751	34.964
3.22523	3.177509	3.325625	34.45685
3.167201	3.230952	3.43102	34.6773
3.269938	3.397886	3.351628	35.09341
3.219621	3.376994	3.394274	35.03095
3.251997	3.281497	3.416152	34.94076

3.229726	3.361426	3.450732	35.14247
3.203041	3.420396	3.423927	35.15445
3.251502	3.378005	3.442684	35.20874
3.398865	3.465887	3.369294	35.56269
3.227375	3.388182	3.447339	35.18841
3.363767	3.33366	3.304634	35.05538
3.377996	3.27159	3.398708	35.15648
3.389338	3.25144	3.337169	35.00264
3.194463	3.324832	3.414856	34.90687
3.255792	3.39728	3.406347	35.18081
3.171078	3.328673	3.374614	34.77613
3.342155	3.383531	3.338959	35.19224
3.274145	3.33858	3.394752	35.06722
3.29456	3.374635	3.415555	35.23621
3.294849	3.50019	3.439973	35.56481
3.34199	3.462719	3.4375	35.58055
3.333371	3.326719	3.421182	35.2286
3.360632	3.208443	3.370129	34.91792
3.260741	3.195166	3.413764	34.76586
3.272289	3.375444	3.391393	35.13643
3.150951	3.452408	3.476327	35.22513
3.194958	3.332245	3.36486	34.81483
3.267711	3.244094	3.351373	34.75166
3.266474	3.280486	3.384248	34.90044
3.253482	3.390136	3.430697	35.21339
3.266226	3.4177	3.409672	35.25556
3.248657	3.448297	3.396491	35.25522
3.433922	3.35435	3.34832	35.34958
3.368221	3.243757	3.308505	34.87698
3.250554	3.257168	3.35792	34.75705
3.302603	3.275904	3.395025	34.99299
3.150167	3.462584	3.393302	35.06411
3.356384	3.398291	3.37325	35.33062
3.309449	3.425383	3.391171	35.32642
3.26136	3.33211	3.366651	34.96366
3.191534	3.369783	3.442888	35.06007
3.317409	3.433807	3.379849	35.33749
3.276702	3.472963	3.45728	35.50343
3.32871	3.348015	3.339573	35.08651
3.259132	3.164569	3.457161	34.79034
3.298479	3.181148	3.400174	34.78802
3.386286	3.369514	3.307993	35.19038
3.317038	3.482398	3.426655	35.5453
3.160849	3.386901	3.435522	35.01429
3.265938	3.29282	3.35567	34.86374
3.194834	3.296189	3.40205	34.81704
3.32042	3.46993	3.424558	35.52084
3.251255	3.453082	3.416987	35.31619
3.205433	3.421744	3.476241	35.27703
3.26004	3.321866	3.449521	35.1196

3.395071	3.166726	3.320339	34.79312
3.375274	3.270917	3.382065	35.11266
3.2647	3.300637	3.319162	34.79829
3.265278	3.440614	3.428906	35.34565
3.280826	3.428887	3.364792	35.21381
3.394576	3.353272	3.338584	35.23988
3.319018	3.360415	3.376728	35.17369
3.268866	3.406647	3.442616	35.3092
3.237645	3.438525	3.389364	35.19418
3.322441	3.44459	3.45832	35.54368
3.364757	3.399302	3.313007	35.2194
3.328009	3.135253	3.371101	34.68865
3.184523	3.179868	3.404863	34.54627
3.286889	3.354417	3.406705	35.15587
3.276125	3.452745	3.389415	35.30954
3.393256	3.432257	3.382611	35.50601
3.155199	3.285474	3.447049	34.80534
3.176068	3.251912	3.406347	34.68857
3.221766	3.375714	3.426724	35.1038
3.35692	3.418172	3.3725	35.37363
3.170706	3.399773	3.461799	35.12146
3.326607	3.407726	3.397344	35.33883
3.212074	3.253529	3.375108	34.70253
3.479125	3.240589	3.326324	35.15155
3.39008	3.284058	3.378911	35.16688
3.340217	3.338041	3.351765	35.11653
3.257648	3.355833	3.411752	35.10605
3.260535	3.396134	3.325727	35.01237
3.212156	3.352665	3.389977	34.95202
3.383069	3.411432	3.293807	35.24399
3.183739	3.431179	3.502279	35.30716
3.197968	3.484218	3.406978	35.24586
3.309161	3.433807	3.441337	35.45392
3.390039	3.266738	3.390523	35.15431
3.255792	3.170433	3.355908	34.57443
3.292622	3.246116	3.425632	34.97296
3.22754	3.400986	3.404915	35.124
3.327555	3.380297	3.403329	35.29401
3.334195	3.346667	3.320663	35.05421
3.25208	3.225561	3.34046	34.65309
3.255132	3.329212	3.378792	34.97026
3.231582	3.441086	3.471211	35.36551
3.328463	3.499988	3.40413	35.55949
3.285157	3.455104	3.40928	35.37789
3.307057	3.225089	3.435045	34.97912
3.23748	3.216867	3.424967	34.78695
3.300252	3.269906	3.342813	34.86055
3.271547	3.348756	3.410593	35.11844
3.40831	3.425181	3.383413	35.5252
3.215249	3.371333	3.395366	35.0114

3.278146	3.343904	3.339863	34.96758
3.288951	3.349295	3.33248	34.98686
3.294602	3.478759	3.423603	35.4816
3.187699	3.446208	3.412127	35.15154
3.253441	3.426596	3.478714	35.39803
3.155983	3.288102	3.467971	34.85855
3.344671	3.192605	3.385032	34.88097
3.327514	3.213497	3.369464	34.8551
3.372882	3.407321	3.397003	35.43839
3.310604	3.415139	3.409143	35.34585
3.267505	3.371401	3.358244	35.04464
3.285446	3.274017	3.30136	34.74651
3.314646	3.283856	3.330178	34.89491
3.198752	3.364324	3.402749	34.97614
3.212115	3.388114	3.406875	35.06641
3.26701	3.41588	3.390438	35.21123
3.271134	3.372748	3.372858	35.08748
3.291673	3.207095	3.384572	34.79576
3.262556	3.157628	3.34844	34.54489
3.227416	3.275836	3.40355	34.84707
3.276083	3.402671	3.327978	35.0656
3.416558	3.397145	3.427542	35.57844
3.315265	3.440344	3.346325	35.27379
3.223168	3.329145	3.393234	34.93179
3.21628	3.351048	3.420636	35.02455
3.315182	3.506323	3.338413	35.40059
3.2753	3.416083	3.416288	35.28633
3.291261	3.386362	3.372704	35.16093
3.231128	3.231626	3.479549	34.9247
3.312666	3.219023	3.343171	34.77721
3.323678	3.315733	3.480282	35.31262
3.458297	3.475996	3.451158	35.89379
3.279094	3.40166	3.423177	35.27815
3.265401	3.322405	3.334236	34.88039
3.237562	3.23048	3.404915	34.77305
3.404763	3.315868	3.305964	35.10903
3.217848	3.414937	3.394172	35.10982
3.127277	3.462045	3.373659	34.96992
3.176646	3.394382	3.461117	35.12117
3.350363	3.27213	3.358193	35.00864
3.33766	3.134646	3.369908	34.70582
3.330442	3.280891	3.391205	35.05642
3.213105	3.336288	3.485176	35.12647
3.238676	3.419048	3.32733	35.01819
3.429385	3.427944	3.297473	35.3894
3.317739	3.335278	3.365321	35.09097
3.19005	3.354417	3.380497	34.88678
3.24346	3.372142	3.387914	35.05856
3.301984	3.442366	3.348218	35.2533
3.266639	3.398021	3.324823	35.02787

3.304954	3.281632	3.349787	34.91173
3.09684	3.165041	3.474059	34.47341
3.32339	3.290259	3.385442	35.04888
3.280744	3.413522	3.443229	35.35155
3.45673	3.47667	3.361399	35.69555
3.262844	3.314722	3.454842	35.12174
3.252781	3.243959	3.35156	34.71913
3.286106	3.234659	3.393319	34.86299
3.317739	3.344443	3.310312	34.99072
3.174625	3.445264	3.433817	35.16832
3.235541	3.453756	3.445617	35.34591
3.207454	3.273949	3.425513	34.84731
3.297241	3.176161	3.342233	34.6477
3.369458	3.254136	3.417601	35.14096
3.281651	3.367963	3.462123	35.29522
3.24445	3.460832	3.452318	35.39552
3.394493	3.473906	3.342574	35.51224
3.336134	3.396875	3.400532	35.34291
3.39738	3.363516	3.305811	35.19675
3.184853	3.367492	3.406944	34.96184
3.119358	3.505649	3.381878	35.06593
3.215621	3.408534	3.383089	35.06671
3.217312	3.240185	3.406466	34.75338
3.26503	3.207903	3.358193	34.68157
3.25109	3.243083	3.416629	34.85581
3.227994	3.433875	3.424609	35.23998
3.348301	3.434279	3.425632	35.5062
3.405217	3.384947	3.390881	35.44679
3.283425	3.294639	3.386482	34.97334
3.267835	3.29902	3.385732	34.94719
3.287838	3.34195	3.386209	35.08585
3.275918	3.373288	3.444662	35.25615
3.211084	3.433133	3.343512	35.02404
3.262432	3.337636	3.393933	35.03775
3.158498	3.195975	3.413082	34.54255
3.285941	3.180609	3.377155	34.70908
3.247708	3.289854	3.405477	34.92631
3.320915	3.437649	3.470631	35.55208
3.307181	3.454025	3.390165	35.3819
3.430004	3.366144	3.381247	35.43881
3.327308	3.339591	3.344876	35.07662
3.286394	3.41642	3.398657	35.27277
3.158993	3.468919	3.406875	35.12695
3.252575	3.475322	3.42154	35.37767
3.053204	3.272938	3.381997	34.41262
3.206712	3.181755	3.411991	34.61451
3.271258	3.226032	3.46289	34.96379
3.285074	3.400245	3.383805	35.20204
3.27167	3.462315	3.426451	35.40172
3.299221	3.376186	3.379116	35.1701

3.349538	3.296661	3.451892	35.26538
3.41363	3.356574	3.321771	35.25201
3.25043	3.312364	3.42998	35.03507
3.238428	3.389125	3.35538	35.01355
3.234015	3.458878	3.401521	35.25734
3.197721	3.407456	3.382253	35.02338
3.306727	3.291809	3.363104	34.96699
3.251255	3.192942	3.392808	34.69443
3.185843	3.236748	3.391256	34.64379
3.237108	3.348285	3.434022	35.09333
3.346857	3.424372	3.355175	35.3273
3.328834	3.420126	3.430697	35.44375
3.444233	3.394112	3.386976	35.54361
3.264123	3.355293	3.448754	35.19995
3.370943	3.395662	3.329888	35.26189
3.228241	3.442703	3.392757	35.19018
3.29786	3.367357	3.345643	35.07462
3.089251	3.221652	3.425956	34.47542
3.158375	3.21033	3.311455	34.35143
3.230468	3.296324	3.435744	34.96894
3.354116	3.477883	3.363019	35.47734
3.218384	3.420531	3.470734	35.29066
3.333247	3.324764	3.381042	35.13628
3.321039	3.301715	3.389346	35.07733
3.394823	3.375107	3.365815	35.34773
3.261854	3.330358	3.388306	35.00827
3.195123	3.316744	3.385885	34.82727
3.224612	3.376657	3.406534	35.06794
3.228076	3.340736	3.369311	34.91556
3.283219	3.274556	3.371425	34.89604
3.243872	3.196379	3.411684	34.72708
3.206588	3.30704	3.443724	34.95761
3.341372	3.369648	3.49186	35.49454
3.490632	3.44486	3.361791	35.70098
3.388513	3.411298	3.380565	35.44532
3.413589	3.356574	3.27771	35.15556
3.287879	3.373018	3.475849	35.34991
3.292003	3.453756	3.36822	35.30013
3.25274	3.392697	3.355329	35.05255
3.356384	3.267547	3.391955	35.08561
3	3.177172	3.445276	34.22523
3.220735	3.267749	3.363974	34.72822
3.225107	3.354822	3.420244	35.05125
3.331308	3.481118	3.475235	35.67994
3.177553	3.407052	3.44016	35.10503
3.327266	3.292617	3.475849	35.26022
3.279919	3.349093	3.348644	35.00201
3.401175	3.350306	3.389415	35.35899
3.244285	3.310544	3.390438	34.93118
3.265731	3.363246	3.376592	35.06305

3.128721	3.364998	3.382475	34.78013
3.18576	3.297807	3.335378	34.65493
3.295633	3.248811	3.332394	34.78154
3.235747	3.223269	3.433408	34.81562
3.250925	3.345521	3.430219	35.10919
3.157261	3.402132	3.446964	35.06477
3.448151	3.462517	3.32675	35.57007
3.458462	3.411567	3.318139	35.46237
3.330648	3.407861	3.375313	35.29978
3.235582	3.418441	3.420312	35.21343
3.309243	3.448903	3.423313	35.4477
3.211166	3.37787	3.26337	34.72811
3.30648	3.215654	3.360188	34.79353
3.087725	3.181889	3.401419	34.33147
3.152766	3.288506	3.439785	34.79076
3.278352	3.434144	3.414463	35.32851
3.279713	3.431111	3.424081	35.34589
3.216239	3.369379	3.414992	35.05221
3.277279	3.307714	3.413168	35.04685
3.261153	3.397819	3.416561	35.21605
3.393792	3.329549	3.30472	35.11224
3.247708	3.289113	3.393985	34.89955
3.320255	3.361628	3.38418	35.19534
3.203619	3.321731	3.35908	34.79814
3.245275	3.257505	3.35584	34.7417
3.167778	3.245913	3.322112	34.47312
3.252781	3.25609	3.41622	34.88706
3.189101	3.395325	3.453	35.13272
3.39606	3.498909	3.431771	35.7654
3.355518	3.446073	3.419971	35.53539
3.485641	3.441692	3.315155	35.58116
3.329452	3.356709	3.401419	35.2424
3.256039	3.387642	3.375654	35.09316
3.292457	3.361763	3.406074	35.18273
3.167572	3.315801	3.344467	34.67438
3.298891	3.216597	3.374222	34.80969
3.228406	3.234591	3.358466	34.66044
3.173552	3.341882	3.410013	34.88784
3.190132	3.422485	3.46144	35.21282
3.379233	3.418239	3.360359	35.39602
3.28693	3.360955	3.43056	35.22242
3.321822	3.337906	3.388733	35.15685
3.354941	3.369379	3.409467	35.34344
3.330896	3.362302	3.394411	35.24246
3.25076	3.351317	3.352396	34.95131
3.387358	3.296324	3.39303	35.21863
3.15623	3.252922	3.396304	34.62544
3.276826	3.2261	3.356966	34.74448
3.145795	3.226976	3.380889	34.51217
3.244243	3.385284	3.380821	35.0735

3.229602	3.451397	3.460912	35.36121
3.300829	3.470671	3.393848	35.41246
3.323225	3.376051	3.429878	35.33331
3.41627	3.409074	3.347485	35.42882
3.353085	3.381038	3.413901	35.37458
3.380099	3.426798	3.309341	35.30507
3.211208	3.31917	3.430594	34.96552
3.179739	3.20359	3.368271	34.50766
3.269691	3.258314	3.36781	34.82304
3.215579	3.320923	3.402391	34.91724
3.205887	3.474378	3.465942	35.3706
3.263174	3.501673	3.423126	35.46194
3.325204	3.383734	3.407779	35.30611
3.392142	3.32719	3.361552	35.22776
3.29621	3.366683	3.445975	35.28895
3.172851	3.380229	3.414378	34.97971
3.312996	3.345724	3.441268	35.26953
3.18176	3.259729	3.378229	34.65662
3.379646	3.253125	3.332599	34.97514
3.249976	3.188292	3.360291	34.61035
3.171408	3.292685	3.397889	34.74905
3.134041	3.331369	3.425087	34.81141
3.292869	3.42417	3.36145	35.22252
3.247502	3.438727	3.433288	35.31224
3.399896	3.472087	3.35908	35.55617
3.2534	3.419654	3.508997	35.44899
3.374201	3.441557	3.315957	35.33891
3.410537	3.370861	3.371357	35.38492
3.377996	3.352328	3.339846	35.20432
3.207537	3.191594	3.423808	34.66367
3.205681	3.202579	3.327807	34.47369
3.131938	3.272467	3.383617	34.58731
3.316708	3.508547	3.367248	35.47184
3.23319	3.464	3.457979	35.3902
3.201268	3.414802	3.474604	35.24916
3.184977	3.329549	3.477213	35.03281
3.310769	3.336693	3.423398	35.20583
3.320956	3.445938	3.379644	35.37133
3.252946	3.342489	3.455114	35.16141
3.195411	3.258584	3.366037	34.65731
3.24977	3.175689	3.331866	34.52018
3.414744	3.218012	3.259192	34.81458
3.230922	3.298346	3.373301	34.83781
3.161097	3.334738	3.394411	34.81086
3.18576	3.33393	3.429452	34.93965
3.31345	3.382992	3.418863	35.30303
3.433922	3.45571	3.364588	35.60681
3.399731	3.450656	3.417447	35.63658
3.196896	3.39728	3.364605	34.96073
3.262019	3.381038	3.386362	35.11521

3.308789	3.353811	3.337731	35.0516
3.393256	3.272804	3.36602	35.12102
3.241975	3.170635	3.324857	34.47676
3.161674	3.234457	3.449385	34.71304
3.23682	3.428955	3.393388	35.18025
3.364014	3.484892	3.382424	35.55675
3.201845	3.351452	3.384521	34.91489
3.270969	3.310005	3.450681	35.1201
3.169964	3.34451	3.449794	34.97273
3.348796	3.426866	3.387863	35.40847
3.3622	3.378814	3.451278	35.47138
3.337495	3.292146	3.382185	35.07673
3.15788	3.188359	3.463504	34.63481
3.287632	3.237018	3.279671	34.62295
3.291137	3.241937	3.396304	34.89643
3.305407	3.398425	3.312427	35.08643
3.144558	3.390473	3.492423	35.11091
3.125215	3.42882	3.377683	34.90155
3.25274	3.412578	3.437364	35.27542
3.351848	3.390473	3.33398	35.21773
3.321451	3.354148	3.422785	35.26602
3.296994	3.374838	3.370163	35.14271
3.260865	3.326314	3.447612	35.12695
3.32871	3.29228	3.337032	34.95907
3.368757	3.244903	3.309341	34.88249
3.228241	3.246857	3.355056	34.67945
3.155529	3.39108	3.365321	34.85828
3.214218	3.418576	3.42142	35.16943
3.336587	3.397212	3.350418	35.23504
3.245563	3.416689	3.431651	35.25622
3.266391	3.416083	3.428633	35.29385
3.115358	3.405097	3.533262	35.16834
3.306727	3.383734	3.353555	35.14713
3.235747	3.294167	3.434635	34.97335
3.377253	3.231087	3.403175	35.07606
3.224158	3.228459	3.438455	34.81266
3.276908	3.211812	3.395076	34.79675
3.272124	3.369648	3.391	35.12254
3.324668	3.407456	3.334986	35.19763
3.184399	3.381307	3.365065	34.89948
3.284909	3.445803	3.435522	35.4144
3.223457	3.454834	3.366634	35.14912
3.332628	3.399099	3.419715	35.38205
3.309491	3.385418	3.415265	35.2918
3.371603	3.380836	3.435198	35.4612
3.316832	3.27368	3.341244	34.90163
3.35391	3.200221	3.381179	34.9094
3.270227	3.220708	3.334116	34.66829
3.31943	3.293426	3.332684	34.93178
3.183781	3.413791	3.409757	35.0669

3.158416	3.399773	3.385885	34.92857
3.248863	3.380431	3.455524	35.23636
3.197886	3.388923	3.331439	34.87209
3.304541	3.382723	3.454279	35.3604
3.250719	3.431988	3.413031	35.26023
3.20168	3.331234	3.477401	35.07343
3.198298	3.207095	3.317474	34.44483
3.399442	3.195503	3.360836	34.95417
3.281528	3.254001	3.343137	34.78553
3.271299	3.283789	3.417993	34.992
3.276496	3.390136	3.374239	35.14025
3.266927	3.361898	3.390983	35.09419
3.285817	3.371266	3.350281	35.06698
3.318275	3.43064	3.419306	35.41875
3.176852	3.469661	3.441763	35.24392
3.314357	3.35718	3.382065	35.16809
3.284703	3.311083	3.409979	35.06348
3.391111	3.283721	3.352788	35.11127
3.318275	3.304613	3.437637	35.18323
3.304954	3.290663	3.354936	34.94274
3.24911	3.274488	3.41078	34.90737
3.349084	3.395393	3.337305	35.22972
3.196195	3.385351	3.427013	35.06959
3.266845	3.375175	3.345984	35.02464
3.086611	3.323551	3.467852	34.78411
3.255462	3.36884	3.388119	35.07803
3.300541	3.476198	3.500659	35.6575
3.280909	3.382655	3.414992	35.22266
3.218796	3.247059	3.449436	34.86563
3.275836	3.208308	3.404352	34.80703
3.271753	3.227111	3.390847	34.80969
3.410124	3.300031	3.288333	35.04757
3.257648	3.334941	3.372244	34.97397
3.211826	3.352261	3.36943	34.90549
3.29687	3.352261	3.366293	35.0846
3.243295	3.453688	3.391529	35.24443
3.272578	3.473637	3.41211	35.3971
3.276455	3.435896	3.463777	35.43603
3.274351	3.331369	3.427968	35.12454
3.288126	3.230143	3.43438	34.94732
3.442954	3.283115	3.303066	35.11459
3.248285	3.265862	3.433817	34.93708
3.387441	3.329212	3.360768	35.22018
3.260246	3.39391	3.461236	35.30322
3.373335	3.419385	3.38655	35.44291
3.312543	3.363313	3.357494	35.1238
3.325575	3.42909	3.405102	35.40026
3.043182	3.387373	3.472439	34.83873
3.251296	3.419048	3.411258	35.22932
3.214919	3.379825	3.393626	35.02544

3.332999	3.287226	3.43646	35.17483
3.225189	3.194829	3.399577	34.65636
3.309738	3.202916	3.467374	35.0072
3.262143	3.294976	3.403823	34.96546
3.29984	3.374501	3.398486	35.21013
3.264783	3.384879	3.350895	35.05209
3.324256	3.346869	3.322402	35.03672
3.189472	3.397954	3.368339	34.95414
3.236737	3.493046	3.40604	35.3479
3.274557	3.46838	3.346308	35.24604
3.307098	3.389058	3.35301	35.15839
3.305737	3.230211	3.418266	34.95075
3.291014	3.222797	3.379337	34.8172
3.345661	3.259055	3.37313	35.00243
3.390616	3.291943	3.354578	35.13209
3.323183	3.355496	3.379798	35.17875
3.263917	3.403008	3.392825	35.18154
3.317244	3.410893	3.403414	35.33855
3.251997	3.397819	3.347894	35.04587
3.374985	3.459686	3.395332	35.55385
3.102077	3.418576	3.425172	34.9324
3.210836	3.418981	3.503745	35.34295
3.130618	3.316609	3.417465	34.75498
3.2793	3.176633	3.389619	34.71312
3.211826	3.159245	3.411445	34.57527
3.325988	3.24187	3.452574	35.09555
3.239583	3.258516	3.419442	34.87055
3.331267	3.416487	3.325864	35.21186
3.277816	3.395528	3.377956	35.16306
3.381419	3.35563	3.329086	35.1955
3.189307	3.387036	3.408035	35.01671
3.25274	3.455912	3.334372	35.14496
3.286642	3.45416	3.39262	35.34265
3.301861	3.401526	3.370317	35.21205
3.321451	3.230683	3.442888	35.03999
3.307552	3.25299	3.363223	34.88416
3.265278	3.245644	3.404778	34.86652
3.373294	3.319036	3.348167	35.13943
3.330896	3.298548	3.40737	35.13138
3.278022	3.367559	3.367674	35.07986
3.301572	3.33986	3.402596	35.14715
3.238387	3.361022	3.366293	34.97587
3.340134	3.467841	3.39112	35.48626
3.209846	3.435762	3.418539	35.19115
3.20531	3.431785	3.470648	35.28649
3.181182	3.307579	3.476804	34.97557
3.307428	3.257101	3.406125	34.9867
3.179491	3.229469	3.412042	34.65943
3.337784	3.272467	3.3873	35.04552
3.237356	3.301783	3.425513	34.97357

3.378697	3.409074	3.346939	35.34546
3.338031	3.348756	3.377018	35.1904
3.406041	3.357585	3.318276	35.22998
3.22754	3.403952	3.416424	35.15566
3.264865	3.51569	3.355482	35.34837
3.197473	3.474783	3.446452	35.31047
3.348754	3.389327	3.342625	35.22737
3.252368	3.239107	3.426945	34.87247
3.255668	3.311083	3.455933	35.10048
3.311676	3.333997	3.432606	35.22206
3.357828	3.3621	3.382935	35.27582
3.329659	3.349363	3.384504	35.18979
3.271175	3.295785	3.430014	35.04426
3.208403	3.345993	3.424183	35.00403
3.238758	3.438323	3.330007	35.06637
3.357498	3.431179	3.384896	35.43045
3.220858	3.360955	3.405443	35.023
3.191369	3.354552	3.442616	35.0258
3.141176	3.261481	3.43218	34.68969
3.34962	3.265997	3.405716	35.09752
3.250265	3.25764	3.40899	34.86913
3.303716	3.310207	3.377632	35.03241
3.292622	3.337232	3.405869	35.12899
3.355724	3.44304	3.406262	35.49923
3.314069	3.387777	3.382065	35.23437
3.369005	3.360146	3.378979	35.28733
3.142908	3.377668	3.420056	34.92105
3.295014	3.406715	3.360563	35.1871
3.187039	3.365942	3.396508	34.94041
3.36818	3.273478	3.338737	35.008
3.29852	3.243892	3.430219	34.99102
3.253317	3.379151	3.431617	35.19102
3.239047	3.380701	3.508997	35.33242
3.403484	3.412039	3.326392	35.36122
3.370531	3.286754	3.410371	35.19883
3.350157	3.364122	3.364963	35.22416
3.247832	3.404895	3.483897	35.34965
3.214095	3.465145	3.385834	35.19318
3.324379	3.39883	3.342693	35.19499
3.263092	3.288506	3.354357	34.84521
3.186049	3.295717	3.444321	34.88924
3.214507	3.250429	3.402135	34.76018
3.310687	3.227785	3.367179	34.84455
3.296004	3.247194	3.430338	34.993
3.283219	3.31014	3.40899	35.05601
3.2322	3.40348	3.441882	35.22049
3.322194	3.469728	3.417243	35.50828
3.271052	3.403884	3.429503	35.27926
3.385585	3.347611	3.291436	35.10474
3.241893	3.417161	3.441933	35.27171

3.170088	3.399436	3.320919	34.81129
3.223663	3.283452	3.440245	34.93576
3.306851	3.285271	3.308744	34.83408
3.21195	3.245037	3.452369	34.85265
3.343599	3.412039	3.397463	35.38568
3.324916	3.382184	3.385459	35.25328
3.371974	3.36729	3.405494	35.36744
3.422704	3.307646	3.331388	35.18588
3.352837	3.405839	3.410985	35.42189
3.267587	3.531393	3.383498	35.44992
3.271464	3.429359	3.414873	35.30388
3.211208	3.340399	3.386772	34.91612
3.254101	3.271051	3.389909	34.86512
3.247172	3.298818	3.441115	35.02267
3.152312	3.255551	3.438608	34.71513
3.174872	3.23567	3.467374	34.78389
3.339722	3.2941	3.431106	35.19286
3.333041	3.32591	3.450272	35.28972
3.306975	3.437716	3.367589	35.29641
3.292828	3.373288	3.452369	35.30998
3.242058	3.334401	3.395212	34.98892
3.351683	3.421474	3.320867	35.25649
3.334649	3.467302	3.435045	35.56914
3.244037	3.416487	3.376933	35.13279
3.208815	3.290326	3.377223	34.7805
3.25608	3.228189	3.429025	34.86126
3.260122	3.382992	3.372431	35.08487
3.366159	3.368435	3.425359	35.40067
3.193885	3.368368	3.37666	34.91729
3.327019	3.282104	3.423279	35.12173
3.270227	3.363718	3.366907	35.05273
3.465927	3.460428	3.397736	35.75961
3.278888	3.451195	3.437364	35.41705
3.232324	3.372951	3.387863	35.03587
3.191617	3.255618	3.435965	34.79545
3.305902	3.353811	3.335259	35.03988
3.213228	3.290393	3.464322	34.98077
3.235087	3.257775	3.390097	34.79492
3.11511	3.269771	3.536808	34.87962
3.371356	3.32719	3.380957	35.22473
3.485023	3.409545	3.387982	35.66876
3.409423	3.437851	3.413781	35.62176
3.245481	3.367222	3.414992	35.11144
3.25938	3.343837	3.354016	34.95735
3.298107	3.404558	3.412196	35.30206
3.397586	3.464336	3.287515	35.37767
3.233933	3.268962	3.41813	34.87817
3.101912	3.245981	3.376524	34.44822
3.242388	3.330358	3.442138	35.08342
3.33568	3.410219	3.374205	35.31352

3.397751	3.42882	3.375876	35.49359
3.260205	3.335075	3.453887	35.15839
3.284126	3.327392	3.439649	35.16277
3.340588	3.410826	3.368714	35.31357
3.471825	3.483005	3.340306	35.69629
3.25872	3.40476	3.480334	35.36537
3.200567	3.243757	3.38273	34.67267
3.19306	3.231963	3.419835	34.7116
3.353456	3.328673	3.33369	35.08547
3.228035	3.314318	3.391461	34.90613
3.245976	3.2817	3.422137	34.94112
3.142537	3.284597	3.504803	34.90203
3.337784	3.401458	3.353675	35.25407
3.373789	3.388451	3.399901	35.40545
3.455987	3.423698	3.392075	35.64517
3.195824	3.288776	3.458644	34.92676
3.285941	3.342219	3.433408	35.18551
3.309367	3.439131	3.314848	35.1894
3.35989	3.337569	3.347417	35.14901
3.152106	3.231222	3.38522	34.54472
3.128679	3.258853	3.426127	34.64337
3.09985	3.408467	3.461662	34.98522
3.373047	3.466897	3.412042	35.60193
3.337866	3.392158	3.455507	35.4566
3.314687	3.293291	3.396031	35.05964
3.175573	3.336356	3.462805	34.99562
3.371232	3.532135	3.40974	35.73559
3.439366	3.489946	3.370641	35.70682
3.315223	3.300233	3.347195	34.9692
3.088261	3.229739	3.388272	34.40854
3.190792	3.271119	3.428497	34.81121
3.326194	3.348958	3.40396	35.22388
3.262226	3.327258	3.391342	35.00894
3.314811	3.283856	3.386652	35.01877
3.169552	3.300974	3.504069	34.99532
3.284703	3.408534	3.366156	35.18076
3.397999	3.420194	3.397395	35.52233
3.340423	3.386092	3.425513	35.38333
3.213393	3.34296	3.442991	35.04944
3.342733	3.377129	3.445497	35.41249
3.281074	3.308253	3.389824	35.00528
3.406784	3.262021	3.285826	34.95166
3.142166	3.258112	3.425632	34.67017
3.185967	3.424102	3.453188	35.1892
3.215084	3.475659	3.409672	35.27046
3.312708	3.402536	3.375842	35.25006
3.325204	3.27213	3.392757	35.0292
3.26503	3.372075	3.423671	35.18378
3.258472	3.47795	3.449931	35.4584
3.407897	3.483813	3.423876	35.74101

3.35193	3.279273	3.370522	35.05464
3.304046	3.181822	3.34462	34.68018
3.139609	3.200423	3.320884	34.30935
3.112512	3.341343	3.399799	34.73084
3.198422	3.338715	3.427695	34.97397
3.224529	3.306163	3.396525	34.89171
3.210011	3.344308	3.501273	35.17244
3.324462	3.373759	3.333366	35.11995
3.233067	3.379555	3.493293	35.28249
3.326442	3.394651	3.412349	35.34269
3.389503	3.363448	3.426519	35.44334
3.34628	3.380027	3.32866	35.17108
3.298396	3.267547	3.384521	34.94254
3.243831	3.262223	3.281632	34.58657
3.251667	3.246857	3.421608	34.87621
3.172274	3.393977	3.349855	34.86741
3.315471	3.502751	3.500113	35.74702
3.201309	3.464471	3.435744	35.27289
3.284208	3.343837	3.34005	34.9811
3.268577	3.312431	3.40043	35.01028
3.284126	3.502144	3.38036	35.41527
3.270433	3.522699	3.454859	35.59319
3.362653	3.376994	3.345285	35.23661
3.186915	3.133231	3.497044	34.6511
3.360179	3.228324	3.253104	34.70449
3.219745	3.277993	3.451005	34.93878
3.311635	3.404558	3.389159	35.28126
3.083394	3.277454	3.460076	34.65926
3.210836	3.313307	3.392416	34.8684
3.238346	3.358798	3.330655	34.89298
3.445058	3.441153	3.413696	35.70672
3.232159	3.429494	3.454006	35.3038
3.327266	3.384003	3.388733	35.26956
3.325782	3.335615	3.3777	35.13637
3.440768	3.322001	3.286577	35.15878
3.380924	3.237152	3.335054	34.94838
3.127772	3.206016	3.373881	34.41159
3.158457	3.317486	3.352038	34.67469
3.301902	3.412241	3.378297	35.25302
3.266061	3.437379	3.389159	35.25337
3.276743	3.401323	3.431566	35.29062
3.201268	3.303468	3.41078	34.86612
3.194793	3.379218	3.513755	35.2428
3.410619	3.578434	3.372448	35.84142
3.31213	3.445534	3.408922	35.41517
3.27101	3.177644	3.400345	34.72066
3.223251	3.124335	3.37429	34.44266
3.275465	3.223471	3.378365	34.78255
3.254266	3.408534	3.310193	34.99181
3.315925	3.305624	3.45948	35.22807

3.112058	3.255214	3.349838	34.43224
3.259009	3.307781	3.470188	35.13174
3.284373	3.434144	3.377837	35.26158
3.361499	3.397212	3.423467	35.44927
3.180399	3.414263	3.383072	35.00218
3.248162	3.389529	3.501819	35.35596
3.310192	3.374298	3.357886	35.14354
3.421301	3.294572	3.29529	35.07528
3.318193	3.185596	3.338192	34.70531
3.221642	3.286215	3.393933	34.8361
3.061824	3.360146	3.40766	34.6783
3.379769	3.440951	3.336998	35.39578
3.253441	3.420126	3.377564	35.16269
3.304088	3.367424	3.381042	35.1658
3.195782	3.368503	3.511248	35.21605
3.383522	3.492372	3.40546	35.66615
3.356384	3.455441	3.439819	35.60118
3.437634	3.37012	3.31079	35.31011
3.298355	3.180272	3.447322	34.88893
3.194669	3.193212	3.343665	34.46381
3.271382	3.349565	3.436545	35.1766
3.214218	3.399436	3.353061	34.97808
3.328751	3.286013	3.437313	35.16476
3.139774	3.277656	3.421267	34.69813
3.214919	3.309331	3.415282	34.91864
3.300417	3.362639	3.423876	35.24098
3.333742	3.409613	3.44826	35.4699
3.228076	3.409545	3.448294	35.23876
3.345496	3.355293	3.375569	35.21786
3.326813	3.277319	3.369516	34.99324
3.379934	3.296863	3.313996	35.03074
3.335433	3.29437	3.395962	35.10722
3.245233	3.367087	3.361501	34.99363
3.220281	3.430235	3.442104	35.25342
3.381213	3.44277	3.341517	35.41279
3.250348	3.337704	3.422495	35.07393
3.302314	3.344039	3.392876	35.13666
3.222426	3.396943	3.467391	35.2406
3.322688	3.514814	3.479464	35.74403
3.254018	3.385486	3.442496	35.23019
3.305119	3.214373	3.397821	34.87005
3.270516	3.18317	3.382458	34.69254
3.333288	3.274151	3.376285	35.01528
3.22424	3.442501	3.423535	35.24829
3.233438	3.335951	3.371357	34.92129
3.323183	3.26101	3.350656	34.9084
3.23517	3.265727	3.367589	34.76327
3.224818	3.33831	3.455319	35.09121
3.259833	3.460899	3.484034	35.49866
3.269031	3.488935	3.399168	35.3945

3.388554	3.368368	3.426877	35.45281
3.350074	3.239578	3.398861	35.02575
3.280538	3.234726	3.381776	34.82571
3.247007	3.255416	3.388647	34.81266
3.255338	3.433335	3.414191	35.27582
3.423364	3.463258	3.391086	35.65817
3.244491	3.367559	3.348303	34.96417
3.325617	3.322405	3.399032	35.15377
3.222962	3.351182	3.455507	35.11571
3.303634	3.416689	3.432623	35.38534
3.19071	3.504301	3.422103	35.30698
3.34397	3.395393	3.436562	35.43559
3.187204	3.144756	3.400191	34.46513
3.378078	3.226437	3.334389	34.91727
3.319224	3.311622	3.411394	35.14325
3.305449	3.465213	3.405921	35.43703
3.280826	3.397212	3.366992	35.14935
3.227127	3.262829	3.39141	34.79144
3.280538	3.26229	3.338277	34.79087
3.338897	3.367694	3.366173	35.21
3.202505	3.414937	3.454142	35.20742
3.196979	3.423159	3.420312	35.13933
3.182956	3.306298	3.435181	34.88562
3.409918	3.284395	3.349667	35.14705
3.363313	3.235737	3.415674	35.08307
3.390616	3.247531	3.380241	35.09108
3.206423	3.321731	3.445173	34.99254
3.297324	3.375107	3.333298	35.0634
3.32904	3.387036	3.37296	35.24558
3.342733	3.368974	3.356488	35.2
3.143857	3.358596	3.393371	34.82305
3.232572	3.436166	3.406381	35.21514
3.283095	3.501605	3.48122	35.6324
3.349084	3.434144	3.3474	35.33653
3.358776	3.227785	3.493497	35.22595
3.182873	3.171646	3.364536	34.43649
3.252327	3.280419	3.380241	34.86059
3.364221	3.43374	3.36278	35.40239
3.409877	3.471817	3.367265	35.59531
3.160519	3.223404	3.434243	34.65323
3.248285	3.238096	3.389278	34.77896
3.268453	3.318901	3.381878	34.98359
3.284332	3.442029	3.388494	35.30204
3.184028	3.447016	3.435744	35.19693
3.211331	3.413589	3.457757	35.23168
3.186833	3.27651	3.446913	34.85462
3.471577	3.259527	3.370539	35.27315
3.249935	3.251575	3.364707	34.75831
3.330071	3.314318	3.351304	35.04145
3.230716	3.379892	3.462805	35.21141

3.370572	3.402065	3.368544	35.35961
3.340836	3.358191	3.350469	35.15911
3.367644	3.327056	3.285537	35.00765
3.183987	3.390473	3.428156	35.05659
3.133257	3.495742	3.411445	35.13932
3.229561	3.421811	3.452864	35.27882
3.336464	3.289719	3.360973	35.02278
3.309037	3.199075	3.379252	34.80455
3.167201	3.29565	3.466863	34.89717
3.280125	3.420598	3.433544	35.34449
3.391317	3.463326	3.41651	35.64384
3.312378	3.35253	3.383771	35.15732
3.273856	3.17825	3.347945	34.61362
3.178584	3.220843	3.337373	34.4753
3.214095	3.395595	3.428906	35.13527
3.286229	3.527215	3.416782	35.55434
3.279589	3.456721	3.38759	35.32182
3.274227	3.252585	3.381622	34.85063
3.231417	3.150686	3.425445	34.63001
3.323678	3.28325	3.418198	35.10582
3.313615	3.427876	3.386635	35.33106
3.31576	3.447151	3.392348	35.3904
3.240408	3.400447	3.400618	35.14157
3.343475	3.312903	3.380906	35.13241
3.271299	3.350913	3.318259	34.92069
3.305902	3.412578	3.39968	35.30927
3.183286	3.464943	3.397872	35.15169
3.224075	3.483409	3.546357	35.60598
3.13301	3.233311	3.432998	34.61202
3.323472	3.162547	3.358688	34.71127
3.334154	3.256023	3.433135	35.10185
3.339681	3.424035	3.388869	35.38455
3.301613	3.44331	3.472302	35.52591
3.346403	3.384542	3.402118	35.34186
3.331102	3.263234	3.352634	34.9349
3.434706	3.329212	3.332411	35.26153
3.183038	3.369648	3.461594	35.0821
3.261731	3.41615	3.334696	35.07838
3.225107	3.364459	3.480487	35.20407
3.336216	3.354822	3.374273	35.1937
3.318647	3.217878	3.394189	34.89935
3.210589	3.204399	3.390728	34.626
3.262391	3.323214	3.482721	35.20029
3.310109	3.419317	3.389295	35.3105
3.381254	3.404154	3.353214	35.35402
3.316213	3.314318	3.297899	34.89436
3.263257	3.333391	3.41344	35.07293
3.210836	3.435964	3.330416	35.00105
3.351641	3.487318	3.289919	35.33271
3.26037	3.427944	3.492219	35.44567

3.244491	3.382588	3.385476	35.07833
3.156684	3.164704	3.43056	34.50842
3.295509	3.179261	3.39569	34.76759
3.316749	3.339052	3.460281	35.30473
3.278434	3.451464	3.381912	35.29538
3.278929	3.346667	3.505877	35.33838
3.368221	3.302524	3.404778	35.21603
3.254802	3.305085	3.361177	34.87825
3.419074	3.302322	3.352464	35.21239
3.166995	3.394112	3.392637	34.94972
3.179368	3.448162	3.440382	35.19938
3.138825	3.405569	3.433288	35.00207
3.332793	3.284261	3.336777	34.94991
3.292952	3.237624	3.362269	34.81654
3.292704	3.290596	3.395894	35.00537
3.17879	3.3435	3.440262	34.96898
3.406619	3.382925	3.355789	35.36869
3.461802	3.355698	3.371937	35.46514
3.329741	3.359876	3.408052	35.26446
3.288126	3.343769	3.412929	35.1489
3.24412	3.394921	3.4257	35.19245
3.310687	3.430505	3.365747	35.28473
3.34595	3.448499	3.399117	35.47417
3.197267	3.295313	3.440569	34.90468
3.140599	3.190853	3.44369	34.55914
3.268495	3.227448	3.419766	34.86654
3.298602	3.437109	3.417601	35.38614
3.346362	3.434683	3.374767	35.39161
3.277939	3.391416	3.450459	35.31289
3.293076	3.234052	3.401777	34.8954
3.282311	3.245711	3.328063	34.73615
3.347929	3.32436	3.35156	35.10303
3.250842	3.478826	3.436699	35.41469
3.28924	3.467706	3.444457	35.49131
3.134165	3.259594	3.400055	34.59998
3.33766	3.195436	3.312137	34.71242
3.253028	3.199614	3.378604	34.68184
3.21096	3.46528	3.504479	35.44608
3.24082	3.467234	3.426911	35.34602
3.419982	3.387777	3.385459	35.47341
3.438954	3.343028	3.284513	35.19629
3.419322	3.369985	3.377632	35.41594
3.19537	3.414533	3.399168	35.07071
3.225519	3.401997	3.352532	35.00724
3.192565	3.372546	3.367197	34.90284
3.254183	3.212351	3.335668	34.61832
3.154539	3.233985	3.436784	34.66885
3.21129	3.329684	3.415828	34.9564
3.195576	3.481926	3.505775	35.45167
3.435407	3.461304	3.378229	35.65212

3.370943	3.33083	3.404284	35.2828
3.324916	3.310746	3.37886	35.08263
3.342939	3.259055	3.461918	35.19064
3.270887	3.364189	3.360717	35.04167
3.312584	3.39573	3.384299	35.2534
3.315718	3.388047	3.393354	35.26326
3.275753	3.304411	3.429537	35.07209
3.183451	3.173735	3.403602	34.52775
3.263133	3.185664	3.383413	34.68394
3.231499	3.343297	3.425087	35.05062
3.244821	3.465078	3.420397	35.33581
3.312543	3.444455	3.422768	35.44399
3.358859	3.398695	3.415879	35.43014
3.387111	3.32746	3.311267	35.10738
3.419033	3.479163	3.438472	35.78711
3.21096	3.514073	3.446861	35.42678
3.290436	3.412511	3.353555	35.17443
3.161262	3.166119	3.454057	34.57291
3.273691	3.207432	3.311063	34.59642
3.189596	3.224011	3.471194	34.79895
3.283136	3.437918	3.381025	35.2741
3.254472	3.504975	3.532239	35.68874
3.264824	3.355967	3.330911	34.94525
3.390493	3.31452	3.454842	35.40045
3.496035	3.388182	3.358807	35.58233
3.314605	3.331234	3.424353	35.20437
3.255627	3.354148	3.33988	34.94077
3.242965	3.371064	3.407234	35.09737
3.350033	3.324023	3.364656	35.13553
3.300046	3.25919	3.355397	34.86419
3.133299	3.245577	3.43663	34.64741
3.15157	3.283721	3.402033	34.69513
3.243584	3.39391	3.461253	35.26682
3.338732	3.425585	3.403755	35.41842
3.35857	3.364796	3.372261	35.25999
3.383687	3.332649	3.390728	35.285
3.288333	3.398965	3.401931	35.246
3.377831	3.480713	3.34433	35.49453
3.338526	3.455373	3.393302	35.46025
3.248038	3.358191	3.36943	34.99764
3.175243	3.21498	3.423023	34.64247
3.254101	3.253057	3.414395	34.87932
3.243419	3.385149	3.4338	35.18726
3.290807	3.479028	3.482738	35.60321
3.170624	3.397617	3.464714	35.12294
3.207083	3.317418	3.490292	35.08322
3.330483	3.253394	3.351492	34.90954
3.450007	3.274286	3.471944	35.48001
3.305325	3.437918	3.396917	35.35738
3.258514	3.417363	3.465328	35.35967

3.266886	3.326988	3.35613	34.94154
3.357003	3.211677	3.315274	34.7971
3.277238	3.212284	3.339215	34.67635
3.222302	3.294572	3.400737	34.8707
3.157096	3.437379	3.475832	35.20462
3.296953	3.480174	3.394803	35.42685
3.423776	3.437581	3.433135	35.69488
3.495705	3.339254	3.297387	35.34029
3.303098	3.30677	3.471143	35.22803
3.322152	3.449779	3.286628	35.17893
3.350198	3.405839	3.485858	35.57986
3.356797	3.271051	3.255117	34.79493
3.239459	3.14981	3.3777	34.54127
3.074651	3.227987	3.410644	34.42386
3.242553	3.415004	3.467391	35.32411
3.258679	3.518858	3.433681	35.51277
3.299963	3.446545	3.385186	35.33887
3.266597	3.332851	3.448362	35.15543
3.23583	3.358124	3.455046	35.15803
3.227994	3.3683	3.399322	35.04128
3.42988	3.403413	3.402578	35.56668
3.260493	3.295785	3.471109	35.11076
3.29621	3.317216	3.39037	35.05917
3.265566	3.21471	3.371357	34.72642
3.382079	3.19429	3.353487	34.89748
3.205887	3.277184	3.384163	34.76053
3.204732	3.342623	3.370453	34.87113
3.158952	3.422485	3.479379	35.18387
3.372181	3.464134	3.298564	35.34583
3.281321	3.38151	3.483079	35.36995
3.425096	3.403143	3.338925	35.41643
3.326524	3.393708	3.477929	35.48422
3.350816	3.407793	3.353692	35.29646
3.263422	3.330021	3.437432	35.11839
3.334855	3.188427	3.347792	34.76893
3.187616	3.173465	3.35821	34.43701
3.223869	3.317486	3.410013	34.94452
3.130329	3.440412	3.393609	34.97291
3.2501	3.461978	3.468875	35.44659
3.293653	3.420328	3.420909	35.34586
3.312749	3.333188	3.426655	35.20962
3.315966	3.358259	3.390387	35.19217
3.328256	3.339793	3.418198	35.23948
3.266721	3.362033	3.456751	35.23786
3.353332	3.352193	3.366429	35.20823
3.40699	3.27341	3.3629	35.14556
3.230551	3.202242	3.410218	34.70756
3.292333	3.245105	3.354647	34.81488
3.196484	3.313038	3.425649	34.9091
3.249234	3.370524	3.358108	35.00247

3.283837	3.437581	3.453324	35.43301
3.322688	3.453756	3.410098	35.45881
3.273196	3.392562	3.378928	35.14859
3.419033	3.425989	3.36556	35.51138
3.26569	3.472491	3.413491	35.38256
3.429303	3.406782	3.400004	35.56716
3.225725	3.228324	3.321157	34.55928
3.31444	3.141521	3.353419	34.63402
3.232118	3.223404	3.334799	34.59234
3.286229	3.405771	3.41119	35.27653
3.209228	3.445736	3.410644	35.19435
3.254802	3.421002	3.413764	35.24674
3.183822	3.331571	3.441712	34.95707
3.331308	3.304074	3.437637	35.21055
3.355601	3.361561	3.422853	35.35706
3.301077	3.382588	3.436954	35.31465
3.249894	3.383599	3.44473	35.22193
3.299963	3.355833	3.380343	35.1299
3.379274	3.249553	3.343461	34.99027
3.31015	3.15938	3.382253	34.72675
3.217188	3.259931	3.433851	34.85618
3.194751	3.38151	3.399833	34.99859
3.282559	3.421744	3.382304	35.24027
3.343723	3.462517	3.344347	35.38018
3.391153	3.392023	3.447884	35.55616
3.209888	3.403547	3.414105	35.1111
3.386657	3.430505	3.419425	35.56825
3.417631	3.436638	3.33444	35.46355
3.398741	3.396471	3.319162	35.30099
3.22391	3.146912	3.382083	34.51052
3.261483	3.185798	3.395212	34.70643
3.204732	3.34013	3.373318	34.87195
3.362076	3.479433	3.348764	35.46697
3.137299	3.335817	3.433425	34.84649
3.181677	3.35408	3.421267	34.95689
3.179986	3.343095	3.428514	34.94502
3.299386	3.410152	3.425649	35.3465
3.21859	3.42107	3.428804	35.20059
3.357828	3.303063	3.446572	35.28588
3.144558	3.27806	3.521138	34.92788
3.348218	3.257707	3.262022	34.7621
3.385337	3.189438	3.374495	34.93993
3.316956	3.272669	3.315326	34.84301
3.148064	3.296054	3.446248	34.81112
3.202794	3.425989	3.332053	34.96523
3.288786	3.465347	3.39997	35.38787
3.395896	3.459888	3.341824	35.48301
3.332752	3.417633	3.383293	35.3432
3.268	3.425248	3.368833	35.18663
3.314522	3.45106	3.377479	35.36372

3.393091	3.392899	3.334389	35.31412
3.351559	3.217069	3.324192	34.81649
3.240408	3.074868	3.373352	34.36995
3.218673	3.188629	3.400004	34.62948
3.235459	3.426866	3.374614	35.13165
3.312955	3.404558	3.408444	35.32632
3.255874	3.388788	3.391	35.12886
3.194834	3.304276	3.433288	34.90304
3.12538	3.370457	3.428446	34.88529
3.3987	3.468043	3.366924	35.56186
3.244326	3.362505	3.50054	35.28567
3.29753	3.367357	3.298922	34.97173
3.233273	3.267614	3.444321	34.93105
3.366571	3.266132	3.361928	35.03913
3.324998	3.214238	3.39823	34.91412
3.393586	3.296661	3.356761	35.15367
3.120596	3.305018	3.431515	34.73844
3.283425	3.435829	3.384453	35.27766
3.22886	3.454227	3.386686	35.20346
3.420848	3.436907	3.380906	35.57279
3.303304	3.411028	3.391342	35.28196
3.249523	3.370053	3.506269	35.32607
3.250059	3.367963	3.374563	35.03466
3.414125	3.298413	3.358995	35.2073
3.363726	3.219226	3.371766	34.95185
3.262226	3.249014	3.425717	34.91301
3.19071	3.404019	3.37209	34.97831
3.196607	3.450723	3.399936	35.15424
3.304253	3.426663	3.368561	35.26841
3.258514	3.390001	3.469028	35.30792
3.222673	3.377399	3.384998	35.01822
3.18939	3.419115	3.45034	35.17956
3.269031	3.394449	3.41344	35.21908
3.296911	3.323753	3.42715	35.15543
3.272	3.241937	3.438745	34.9474
3.214177	3.245981	3.422171	34.79354
3.316626	3.227987	3.405239	34.9412
3.257978	3.339254	3.357153	34.95112
3.325617	3.323079	3.431583	35.22643
3.245027	3.434144	3.427269	35.28364
3.199329	3.432796	3.449845	35.23013
3.277032	3.466089	3.332275	35.21575
3.338155	3.430505	3.399322	35.41822
3.247213	3.361628	3.423603	35.12182
3.337289	3.368503	3.479396	35.45585
3.249935	3.341343	3.39852	35.02856
3.343145	3.230278	3.347604	34.87817
3.418373	3.229065	3.374614	35.0991
3.364757	3.323551	3.362678	35.16237
3.281775	3.436907	3.415282	35.34383

3.272949	3.439064	3.351782	35.19038
3.133752	3.375309	3.431839	34.92163
3.352837	3.387508	3.365014	35.28128
3.193102	3.37841	3.506678	35.22186
3.179326	3.451464	3.434363	35.19335
3.228447	3.304276	3.510413	35.1452
3.327349	3.248542	3.389773	34.97578
3.311017	3.229132	3.401999	34.92436
3.285074	3.217271	3.378314	34.78989
3.258514	3.237557	3.428514	34.88595
3.193968	3.348015	3.361382	34.83955
3.350074	3.406176	3.402715	35.3985
3.287095	3.449577	3.391921	35.33209
3.314976	3.475254	3.402442	35.47222
3.202505	3.407658	3.425155	35.12811
3.32372	3.434683	3.374222	35.3409
3.273155	3.37996	3.42553	35.22286
3.31213	3.322608	3.367145	35.05499
3.205021	3.195436	3.43999	34.70195
3.3987	3.224415	3.381383	35.06071
3.249316	3.260336	3.418078	34.89283
3.410867	3.423698	3.330484	35.41181
3.262143	3.426933	3.473666	35.40676
3.262019	3.382251	3.381776	35.10783
3.200938	3.346263	3.458388	35.06309
3.312378	3.388384	3.382253	35.23241
3.18873	3.462786	3.425786	35.21992
3.273403	3.392899	3.435369	35.27321
3.121998	3.255753	3.466556	34.7104
3.347929	3.217878	3.405409	34.98793
3.444563	3.260807	3.419033	35.32292
3.392307	3.285743	3.414327	35.25289
3.241274	3.333795	3.418675	35.03719
3.254183	3.343297	3.402732	35.05134
3.326442	3.341006	3.348082	35.08483
3.427241	3.434077	3.365201	35.54623
3.211372	3.474176	3.3873	35.21018
3.175243	3.417026	3.381793	34.99415
3.29188	3.416083	3.413645	35.31681
3.270969	3.319238	3.457757	35.15576
3.347641	3.305287	3.35023	35.05778
3.240985	3.239646	3.417652	34.82843
3.267381	3.259392	3.403465	34.89832
3.284291	3.347745	3.37163	35.05889
3.408186	3.416083	3.427815	35.60214
3.270557	3.375714	3.402306	35.1571
3.272289	3.354283	3.39985	35.10865
3.20498	3.36857	3.396304	34.98495
3.363767	3.425585	3.341278	35.33654
3.228406	3.451464	3.376455	35.17405

3.237191	3.267143	3.455558	34.96316
3.164767	3.228998	3.460008	34.7311
3.301654	3.229604	3.378161	34.85279
3.342032	3.250294	3.36058	34.94789
3.393297	3.298818	3.426996	35.31135
3.239872	3.364931	3.446793	35.1637
3.299303	3.356372	3.411582	35.19795
3.286106	3.43037	3.382065	35.26637
3.344919	3.449712	3.367606	35.40566
3.251007	3.429494	3.351424	35.12069
3.163283	3.412309	3.422171	35.04598
3.207083	3.324495	3.451278	35.01338
3.3516	3.287495	3.410712	35.15979
3.332381	3.250564	3.417874	35.05266
3.22688	3.298413	3.357307	34.79414
3.204856	3.306568	3.451772	34.97039
3.299427	3.398223	3.394326	35.25201
3.446212	3.407591	3.327483	35.44732
3.364468	3.378207	3.332957	35.21627
3.130494	3.389058	3.39349	34.86071
3.221271	3.382386	3.386908	35.03024
3.29155	3.453688	3.46717	35.51537
3.231458	3.395797	3.367401	35.03919
3.313986	3.231761	3.417601	34.97072
3.136763	3.187955	3.432265	34.51943
3.263339	3.270243	3.392825	34.88994
3.253482	3.298885	3.399475	34.94556
3.398906	3.393371	3.405256	35.48284
3.244285	3.372951	3.384572	35.05482
3.374449	3.330897	3.36058	35.19504
3.27134	3.383194	3.390506	35.14937
3.39272	3.464943	3.297422	35.39002
3.15124	3.454767	3.427542	35.12424
3.273073	3.393708	3.380889	35.15512
3.193927	3.27806	3.436187	34.85006
3.24445	3.279678	3.35671	34.79028
3.329906	3.295987	3.410286	35.12999
3.377707	3.342421	3.396474	35.30586
3.240408	3.364931	3.463572	35.20157
3.340299	3.401121	3.349463	35.24962
3.315306	3.35125	3.409399	35.21697
3.384925	3.358933	3.363019	35.28459
3.199041	3.429764	3.391546	35.09538
3.137506	3.425046	3.395758	34.9597
3.246759	3.37349	3.415265	35.12854
3.215786	3.303266	3.420125	34.91786
3.261318	3.217743	3.408052	34.80401
3.233438	3.176498	3.456069	34.75785
3.255379	3.237152	3.452233	34.93008
3.356549	3.340399	3.436665	35.34306

3.454791	3.425248	3.395451	35.65333
3.287673	3.361628	3.341415	35.03057
3.357869	3.366076	3.349667	35.21185
3.291137	3.423765	3.40558	35.31435
3.341248	3.446882	3.350639	35.35434
3.268783	3.424035	3.343938	35.13125
3.306892	3.341545	3.357579	35.06403
3.163241	3.239511	3.395434	34.60954
3.246264	3.282643	3.361859	34.812
3.241356	3.310746	3.428173	35.00774
3.431612	3.38589	3.432061	35.59663
3.2322	3.352665	3.46144	35.15214
3.346156	3.363381	3.344467	35.16897
3.306769	3.318833	3.413764	35.13696
3.347888	3.452071	3.385118	35.45561
3.216775	3.43219	3.359626	35.06966
3.244738	3.366683	3.405443	35.08775
3.105872	3.324158	3.376183	34.62709
3.19834	3.232098	3.385015	34.6473
3.292251	3.226167	3.377786	34.82389
3.2793	3.210464	3.407626	34.82648
3.317038	3.264986	3.404574	35.02156
3.19537	3.346398	3.384367	34.88934
3.421343	3.449308	3.354169	35.54252
3.405052	3.363246	3.349156	35.30772
3.289652	3.441692	3.389943	35.31611
3.22952	3.437042	3.399049	35.19435
3.320255	3.394921	3.361655	35.21889
3.236036	3.365672	3.353402	34.9527
3.275052	3.254742	3.401214	34.9
3.181224	3.273949	3.458934	34.86304
3.194174	3.311959	3.433288	34.9184
3.292704	3.346667	3.394121	35.12412
3.390121	3.345858	3.380906	35.30648
3.227581	3.360887	3.413355	35.05486
3.301077	3.364392	3.410218	35.21639
3.273485	3.360685	3.387386	35.09801
3.341289	3.416622	3.344484	35.27479
3.294478	3.405569	3.375091	35.21519
3.267835	3.334469	3.392109	35.03865
3.194298	3.326045	3.406006	34.88981
3.245811	3.226639	3.366071	34.69774
3.251131	3.278262	3.44814	35.00174
3.321327	3.289652	3.413969	35.10543
3.288951	3.259123	3.463828	35.0769
3.292086	3.354552	3.385663	35.12151
3.316337	3.331234	3.441337	35.2453
3.402123	3.373422	3.34769	35.32037
3.25872	3.384273	3.391256	35.12577
3.231087	3.421339	3.390677	35.14513

3.20597	3.367424	3.372636	34.93285
3.253358	3.336828	3.377973	34.98124
3.266185	3.239039	3.398299	34.83989
3.241521	3.306972	3.385203	34.90588
3.315058	3.386564	3.413423	35.30246
3.216734	3.366616	3.365935	34.93997
3.381667	3.360213	3.450204	35.47093
3.374366	3.362976	3.340579	35.22127
3.34562	3.399167	3.432606	35.4388
3.282847	3.456317	3.395707	35.34581
3.339969	3.397212	3.404846	35.36147
3.211331	3.337165	3.392689	34.92225
3.315306	3.297065	3.338874	34.94425
3.112058	3.255416	3.460997	34.67577
3.200031	3.300637	3.351731	34.72809
3.129216	3.267547	3.445821	34.70663
3.274599	3.347678	3.437841	35.18234
3.292251	3.372883	3.451278	35.30545
3.405959	3.394382	3.412537	35.5164
3.206671	3.347611	3.434959	35.02734
3.321864	3.37194	3.336146	35.11637
3.339681	3.412646	3.345064	35.26385
3.310852	3.404626	3.246369	34.96744
3.237026	3.277925	3.420721	34.9102
3.178501	3.250901	3.353265	34.5756
3.24049	3.260673	3.39673	34.82758
3.316584	3.477343	3.347485	35.36012
3.283383	3.428011	3.448822	35.40124
3.28693	3.331032	3.404949	35.10097
3.248327	3.284934	3.413901	34.93532
3.322565	3.448566	3.442138	35.51726
3.356467	3.502077	3.383276	35.57969
3.277197	3.447623	3.479396	35.49746
3.229932	3.310274	3.403056	34.9268
3.204774	3.237624	3.388016	34.68002
3.309078	3.302929	3.351509	34.97109
3.231829	3.312431	3.388136	34.90303
3.243006	3.288102	3.454961	35.02041
3.070815	3.275567	3.423467	34.54757
3.393091	3.378073	3.35983	35.33733
3.375109	3.393169	3.36972	35.35265
3.382739	3.380836	3.396457	35.40084
3.229685	3.367829	3.474246	35.2078
3.336216	3.426259	3.365832	35.33146
3.346733	3.457058	3.278716	35.2313
3.437964	3.305422	3.339437	35.23199
3.12703	3.227313	3.381912	34.4741
3.126164	3.242611	3.455319	34.66619
3.137093	3.363044	3.354971	34.73402
3.330442	3.440412	3.422222	35.4731

3.216652	3.385351	3.436989	35.13614
3.32273	3.303805	3.403619	35.11681
3.130989	3.377466	3.434772	34.92672
3.430416	3.543457	3.380752	35.82638
3.417878	3.43502	3.390711	35.58361
3.307676	3.307511	3.384998	35.05128
3.193473	3.228324	3.436477	34.74094
3.208485	3.282778	3.322249	34.64305
3.224942	3.319777	3.384009	34.89501
3.286436	3.345858	3.319213	34.94483
3.17512	3.251035	3.478287	34.8419
3.223086	3.376051	3.424285	35.10209
3.325617	3.39883	3.447049	35.42591
3.455822	3.407254	3.359609	35.53785
3.283012	3.36055	3.362678	35.06452
3.275094	3.371266	3.364946	35.07559
3.313656	3.41433	3.441439	35.42138
3.310852	3.344376	3.308658	34.9719
3.450007	3.233783	3.314405	35.04692
3.20498	3.237287	3.400959	34.70803
3.196525	3.377668	3.396747	34.98732
3.16716	3.418374	3.436392	35.09882
3.337536	3.391214	3.310364	35.13641
3.286972	3.243083	3.370112	34.83255
3.24878	3.313442	3.461662	35.1031
3.217971	3.486172	3.452778	35.39404
3.343888	3.509962	3.370811	35.54217
3.290106	3.331638	3.37163	35.03638
3.348796	3.18627	3.430611	34.97581
3.209022	3.286148	3.508094	35.05801
3.223581	3.320384	3.381656	34.88822
3.217394	3.265525	3.39303	34.77959
3.271175	3.262155	3.376114	34.85284
3.177512	3.379286	3.472012	35.11387
3.222467	3.487992	3.420193	35.33659
3.284868	3.382251	3.448822	35.30442
3.438582	3.388653	3.395349	35.53763
3.230592	3.308522	3.361842	34.83428
3.321369	3.409815	3.499329	35.55497
3.326194	3.394921	3.298837	35.0945
3.272825	3.221517	3.38996	34.79786
3.294519	3.199547	3.309818	34.622
3.2249	3.267143	3.436562	34.89474
3.25707	3.414735	3.352379	35.10376
3.250925	3.44122	3.389159	35.22867
3.270433	3.363111	3.424609	35.17804
3.244367	3.334132	3.392552	34.98757
3.303758	3.443579	3.51268	35.61949
3.227251	3.506188	3.426519	35.40068
3.292045	3.415072	3.464731	35.42668

3.220281	3.262021	3.33855	34.6591
3.361086	3.196514	3.45225	35.07241
3.298602	3.217473	3.39719	34.8612
3.18873	3.249283	3.398912	34.69425
3.19702	3.253799	3.418471	34.76503
3.272825	3.301513	3.350708	34.88696
3.207949	3.410691	3.403056	35.09832
3.336505	3.389799	3.327296	35.16809
3.202423	3.349161	3.445412	35.0443
3.318729	3.365537	3.386345	35.20529
3.332958	3.418307	3.349378	35.27096
3.448068	3.501336	3.42079	35.86043
3.376759	3.361224	3.406057	35.36586
3.280373	3.193279	3.327569	34.61617
3.226839	3.222191	3.366378	34.6472
3.312625	3.336221	3.345098	35.03763
3.193102	3.392832	3.406398	35.0341
3.146662	3.383464	3.412213	34.92478
3.27299	3.273208	3.426604	34.9914
3.259297	3.347745	3.387727	35.03943
3.309326	3.478691	3.400618	35.46339
3.289116	3.494124	3.474604	35.61474
3.171738	3.38124	3.412332	34.97501
3.255915	3.239107	3.372517	34.7612
3.395071	3.298413	3.383634	35.21952
3.268288	3.33831	3.458661	35.19358
3.166293	3.306433	3.356539	34.6775
3.164767	3.219832	3.397685	34.57476
3.279094	3.334334	3.412315	35.10717
3.256617	3.375646	3.377922	35.07314
3.309985	3.395797	3.444952	35.3805
3.22391	3.385081	3.417106	35.10795
3.333412	3.400784	3.430287	35.41057
3.366984	3.387305	3.411121	35.4126
3.529524	3.33238	3.375654	35.57037
3.338072	3.260134	3.370453	34.98233
3.235582	3.245509	3.347127	34.67521
3.238552	3.337569	3.324346	34.83321
3.382038	3.405839	3.35538	35.36415
3.304665	3.402469	3.348423	35.17237
3.179656	3.33986	3.373045	34.81592
3.179409	3.282845	3.470802	34.90448
3.311099	3.436301	3.362388	35.29096
3.295633	3.461708	3.447799	35.49948
3.382203	3.364863	3.413713	35.40247
3.184441	3.166928	3.435522	34.58484
3.203619	3.213362	3.333093	34.50433
3.364303	3.345117	3.364997	35.21361
3.295509	3.374029	3.386277	35.17293
3.183987	3.262964	3.462907	34.85375

3.208073	3.312566	3.375859	34.82453
3.299138	3.372209	3.378587	35.16007
3.327885	3.426798	3.34474	35.26829
3.273691	3.399167	3.455302	35.33114
3.124308	3.34013	3.471058	34.90981
3.298437	3.350306	3.43941	35.24365
3.277692	3.404221	3.347587	35.11539
3.46304	3.311622	3.340238	35.30214
3.305119	3.224887	3.408871	34.9172
3.252245	3.283115	3.400737	34.91113
3.254224	3.38151	3.413168	35.15781
3.368922	3.453553	3.257896	35.22663
3.248822	3.334132	3.384299	34.97926
3.208527	3.394651	3.352652	34.95428
3.130453	3.396808	3.495885	35.10149
3.325452	3.490755	3.377206	35.47383
3.384389	3.435762	3.502722	35.75694
3.314275	3.31142	3.410849	35.13079
3.190875	3.163424	3.448277	34.61914
3.275588	3.216665	3.37238	34.75485
3.353456	3.346398	3.381588	35.22897
3.324297	3.32928	3.357017	35.07404
3.106037	3.261886	3.338737	34.40938
3.182791	3.312498	3.429605	34.88663
3.303057	3.434144	3.367111	35.27899
3.2972	3.444321	3.398213	35.35645
3.320626	3.405232	3.471688	35.48288
3.138908	3.374096	3.424081	34.91329
3.255049	3.371805	3.470052	35.26279
3.308336	3.359135	3.352805	35.09521
3.484404	3.244363	3.383225	35.29578
3.239789	3.244903	3.409263	34.81897
3.221353	3.245037	3.33913	34.62558
3.270928	3.311892	3.447612	35.11742
3.38344	3.478354	3.295444	35.39473
3.285611	3.363987	3.383481	35.12321
3.20531	3.355496	3.365594	34.88992
3.159117	3.437446	3.40662	35.05783
3.263257	3.483746	3.425496	35.4281
3.339928	3.397684	3.442292	35.44429
3.334402	3.292011	3.377768	35.06002
3.209352	3.201434	3.442701	34.73047
3.317162	3.262762	3.394394	34.99471
3.286353	3.385284	3.407643	35.22424
3.3191	3.352598	3.398844	35.20514
3.186379	3.27213	3.379525	34.69668
3.17116	3.250227	3.342642	34.53484
3.180687	3.341006	3.40621	34.89321
3.258844	3.436233	3.427559	35.31906
3.259421	3.421407	3.445975	35.32817

3.241604	3.361494	3.473377	35.21811
3.24115	3.314318	3.398895	34.95107
3.355147	3.236815	3.330416	34.88113
3.420147	3.273882	3.316775	35.0745
3.259215	3.42727	3.379729	35.19567
3.232077	3.474715	3.396372	35.27647
3.328133	3.401593	3.287191	35.08787
3.336258	3.326179	3.36972	35.12119
3.279053	3.336625	3.317935	34.90569
3.258143	3.362437	3.42906	35.15943
3.136227	3.441827	3.439291	35.0888
3.242181	3.439199	3.434431	35.30413
3.295839	3.197592	3.384043	34.78293
3.309862	3.207701	3.367333	34.79916
3.205516	3.25582	3.42183	34.79538
3.442129	3.416554	3.434499	35.69202
3.276496	3.402536	3.472166	35.38152
3.334072	3.312094	3.361689	35.06805
3.168356	3.224213	3.389909	34.57519
3.313656	3.358394	3.419801	35.25173
3.112842	3.408669	3.40974	34.90053
3.197886	3.507805	3.491536	35.48218
3.237933	3.466493	3.41772	35.31799
3.393173	3.269366	3.346257	35.07011
3.247955	3.244026	3.427423	34.87462
3.272743	3.288843	3.350333	34.85825
3.295179	3.354889	3.415077	35.19334
3.322152	3.419722	3.336077	35.22134
3.303345	3.426192	3.377888	35.28579
3.272454	3.382588	3.377274	35.12154
3.343104	3.37659	3.345882	35.19428
3.213311	3.44277	3.441115	35.26343
3.158828	3.480107	3.360427	35.04948
3.304088	3.469324	3.440774	35.51926
3.199783	3.229469	3.490087	34.87448
3.312584	3.133838	3.443178	34.80944
3.361787	3.268962	3.422358	35.16701
3.23187	3.400919	3.351833	35.01724
3.336959	3.400852	3.451482	35.46483
3.216693	3.307781	3.454432	35.00474
3.319307	3.273949	3.292221	34.80043
3.260658	3.369379	3.35035	35.00798
3.322111	3.480309	3.431907	35.56331
3.096798	3.432661	3.477742	35.06662
3.214342	3.390473	3.364775	34.98437
3.198546	3.295785	3.46335	34.95833
3.27497	3.265121	3.285144	34.66869
3.274021	3.288641	3.412554	34.99667
3.303387	3.275162	3.413696	35.03391
3.280455	3.318362	3.373403	34.99012

3.35358	3.378746	3.371425	35.27776
3.317822	3.355496	3.363718	35.13186
3.375109	3.35462	3.359779	35.24661
3.161715	3.346195	3.408598	34.86829
3.25208	3.471008	3.424268	35.37312
3.212074	3.550398	3.457007	35.53084
3.296293	3.402402	3.364877	35.18989
3.212156	3.195099	3.449385	34.73737
3.239995	3.15783	3.366446	34.53537
3.285982	3.246587	3.425581	34.95935
3.349868	3.434683	3.35879	35.36434
3.325617	3.376994	3.406074	35.28855
3.218755	3.296594	3.419084	34.90749
3.280125	3.239174	3.326324	34.71328
3.235211	3.305018	3.38759	34.89302
3.24779	3.420194	3.381298	35.15864
3.169593	3.478422	3.460366	35.28788
3.171903	3.423563	3.474945	35.20485
3.203248	3.248272	3.44814	34.83145
3.320668	3.22428	3.35318	34.82809
3.2209	3.205477	3.428446	34.73339
3.319265	3.415206	3.427406	35.40487
3.228282	3.400245	3.465465	35.25642
3.430375	3.450319	3.336401	35.52562
3.357415	3.328403	3.397548	35.23318
3.392266	3.399639	3.381452	35.42997
3.165469	3.405637	3.361672	34.90387
3.273856	3.442299	3.411036	35.32902
3.101665	3.464067	3.409536	34.99679
3.261731	3.225291	3.358517	34.71309
3.147445	3.147047	3.446964	34.48548
3.248739	3.29073	3.401283	34.92131
3.268453	3.438457	3.387027	35.2563
3.39969	3.426933	3.480436	35.72236
3.301407	3.315868	3.362132	35.00584
3.312543	3.236074	3.40899	34.95816
3.202876	3.312701	3.443434	34.96124
3.299922	3.428146	3.390438	35.31003
3.278764	3.42727	3.416834	35.31956
3.238098	3.429561	3.427747	35.25951
3.245398	3.249148	3.41697	34.85737
3.3078	3.148664	3.386448	34.70735
3.291797	3.282373	3.425649	35.05048
3.277321	3.388384	3.394189	35.18185
3.320874	3.406647	3.406364	35.34366
3.344712	3.437312	3.391665	35.4307
3.353002	3.328336	3.380957	35.1871
3.326813	3.378005	3.301974	35.06573
3.205805	3.409411	3.453477	35.20109
3.121214	3.412039	3.387624	34.87784

3.227457	3.446814	3.419511	35.25596
3.149714	3.266266	3.407694	34.66528
3.225313	3.167804	3.407694	34.61528
3.199659	3.192201	3.44843	34.70161
3.281651	3.473772	3.403397	35.39819
3.188029	3.401391	3.491247	35.22728
3.437263	3.40011	3.366293	35.49626
3.284662	3.266806	3.425087	34.9996
3.368469	3.276375	3.389005	35.1249
3.281074	3.305085	3.38834	34.99511
3.301696	3.392764	3.371238	35.19454
3.145094	3.433403	3.414992	35.03663
3.320874	3.359135	3.303952	35.0158
3.177099	3.215586	3.439018	34.68283
3.273361	3.237826	3.394121	34.8438
3.190792	3.263571	3.455387	34.85351
3.342774	3.407456	3.379235	35.33399
3.352013	3.419183	3.432197	35.49566
3.341248	3.334738	3.376387	35.16541
3.284992	3.354552	3.413287	35.16641
3.286312	3.430235	3.343989	35.18326
3.256287	3.457934	3.344603	35.17951
3.269237	3.417363	3.42304	35.29064
3.144929	3.38097	3.383685	34.85315
3.179491	3.237691	3.438046	34.73428
3.274186	3.224887	3.361331	34.7456
3.246141	3.32099	3.406006	34.99213
3.325163	3.412376	3.417516	35.38995
3.247131	3.338917	3.397122	35.01407
3.339722	3.298144	3.358568	35.04307
3.380058	3.223067	3.390694	35.03735
3.369458	3.362505	3.427082	35.39868
3.189472	3.330627	3.420466	34.9209
3.281156	3.413656	3.377069	35.20807
3.196607	3.411904	3.453205	35.18584
3.326689	3.294639	3.311319	34.90358
3.210548	3.207701	3.457433	34.77901
3.220487	3.280015	3.413491	34.86279
3.138908	3.353878	3.433442	34.88954
3.349497	3.432122	3.405204	35.45943
3.353456	3.405637	3.420738	35.44413
3.440108	3.368368	3.321157	35.33436
3.241274	3.310881	3.439359	35.03231
3.31444	3.423429	3.386294	35.3224
3.209187	3.42572	3.34092	34.99801
3.245852	3.369716	3.37313	35.02616
3.163613	3.260807	3.415828	34.70152
3.179945	3.223674	3.424575	34.67516
3.267175	3.324225	3.362422	34.94989
3.289034	3.420598	3.388801	35.26613

3.360632	3.435155	3.481237	35.65668
3.255338	3.361898	3.451772	35.20178
3.273774	3.335817	3.400601	35.07316
3.383852	3.28116	3.34208	35.06638
3.433386	3.370053	3.356079	35.39971
3.129504	3.327729	3.435045	34.8153
3.276207	3.396943	3.393797	35.19727
3.22688	3.364324	3.383003	34.99447
3.283054	3.266064	3.338328	34.80473
3.215538	3.216597	3.371698	34.62189
3.211537	3.287765	3.46601	34.97502
3.101912	3.360281	3.436136	34.82853
3.406083	3.436099	3.401095	35.58288
3.340753	3.41305	3.4109	35.41105
3.466216	3.40995	3.313433	35.46549
3.234593	3.386497	3.426741	35.15547
3.279424	3.455508	3.348713	35.23379
3.291838	3.385553	3.327978	35.06261
3.29188	3.358663	3.337527	35.02478
3.149879	3.239983	3.430134	34.65723
3.164561	3.233243	3.480351	34.78442
3.224405	3.311757	3.390591	34.89069
3.354033	3.386092	3.446452	35.45889
3.383193	3.385081	3.469233	35.57026
3.19339	3.353811	3.507565	35.17064
3.276867	3.331167	3.358739	34.97821
3.305284	3.260875	3.38568	34.94555
3.3662	3.368233	3.392092	35.32756
3.188524	3.433066	3.445685	35.19799
3.208774	3.404693	3.404096	35.08928
3.273733	3.332919	3.364877	34.98861
3.364179	3.251035	3.363479	35.00428
3.246924	3.204736	3.412179	34.75311
3.248038	3.325034	3.391955	34.97439
3.160932	3.370996	3.432316	34.97268
3.420807	3.4177	3.344143	35.4503
3.452028	3.392023	3.371391	35.52201
3.367809	3.351452	3.341943	35.18472
3.253647	3.387305	3.428429	35.2026
3.278558	3.436233	3.378178	35.25418
3.276743	3.373018	3.363104	35.07901
3.330607	3.272332	3.311216	34.86314
3.164767	3.199614	3.399987	34.53558
3.08723	3.30987	3.443502	34.70229
3.187616	3.400852	3.422358	35.07455
3.316873	3.426866	3.480419	35.54107
3.328339	3.381577	3.465414	35.43429
3.347187	3.348689	3.504496	35.48905
3.211826	3.294841	3.433271	34.91953
3.369376	3.311016	3.373403	35.16851

3.354528	3.401458	3.436017	35.47075
3.265236	3.400717	3.416373	35.23091
3.201309	3.30987	3.372176	34.79579
3.269979	3.231222	3.383344	34.79839
3.353291	3.251575	3.358585	34.97095
3.278847	3.28608	3.380002	34.93044
3.261401	3.429898	3.452505	35.36535
3.204155	3.46993	3.402868	35.21916
3.375769	3.44331	3.425632	35.58602
3.457843	3.383734	3.329325	35.42461
3.415734	3.39108	3.417311	35.541
3.211867	3.4208	3.417942	35.16155
3.298767	3.38279	3.434976	35.30571
3.225684	3.257236	3.308573	34.5949
3.245852	3.14516	3.365628	34.51868
3.111069	3.206623	3.454961	34.5537
3.255462	3.407793	3.456478	35.31271
3.18477	3.474648	3.476582	35.34828
3.343929	3.411365	3.365764	35.31561
3.304005	3.308927	3.472422	35.23753
3.322812	3.349228	3.402033	35.21286
3.234056	3.32254	3.436579	35.03595
3.321369	3.415072	3.381025	35.30775
3.267546	3.366953	3.364144	35.0479
3.406413	3.342219	3.317457	35.1954
3.144599	3.262762	3.369345	34.56256
3.254389	3.219495	3.322794	34.60624
3.287755	3.241196	3.431362	34.96408
3.234304	3.403547	3.342762	35.00848
3.311759	3.418239	3.518324	35.59391
3.260782	3.448432	3.462054	35.42541
3.302355	3.355091	3.424336	35.22972
3.415569	3.406311	3.292306	35.30058
3.373913	3.397954	3.332599	35.27932
3.27897	3.393506	3.411872	35.23533
3.339021	3.324629	3.364451	35.11233
3.188317	3.134983	3.433578	34.51921
3.290849	3.184316	3.295222	34.54874
3.226674	3.33703	3.462054	35.1072
3.295097	3.519936	3.399577	35.52019
3.191411	3.417026	3.453256	35.18578
3.277197	3.325505	3.441064	35.14658
3.261648	3.363583	3.410763	35.12959
3.370283	3.361224	3.368765	35.27015
3.198133	3.380701	3.454978	35.12481
3.347929	3.384071	3.401879	35.34365
3.235005	3.341747	3.425308	35.05538
3.329164	3.261481	3.271878	34.75023
3.274681	3.180002	3.369106	34.66553
3.175532	3.264514	3.415947	34.73595

3.044419	3.319979	3.411991	34.56187
3.277692	3.404963	3.347894	35.11768
3.306521	3.467639	3.392433	35.41518
3.364014	3.394854	3.452727	35.5136
3.313038	3.430842	3.390335	35.34438
3.345042	3.385149	3.44282	35.42922
3.391153	3.449847	3.303952	35.36786
3.453678	3.389327	3.406398	35.59628
3.240408	3.204264	3.371681	34.64927
3.162829	3.148327	3.392075	34.40189
3.26767	3.259931	3.41356	34.9222
3.222632	3.510232	3.330314	35.18903
3.277568	3.488261	3.457553	35.53937
3.145053	3.372816	3.446111	34.9721
3.210795	3.295583	3.452045	34.95995
3.293323	3.357922	3.394956	35.15191
3.348342	3.339456	3.4028	35.249
3.228447	3.377264	3.395349	35.05319
3.281404	3.307174	3.440791	35.1151
3.270227	3.220034	3.387727	34.78405
3.364427	3.236344	3.336299	34.91325
3.297612	3.254675	3.370539	34.8821
3.214383	3.29875	3.458508	34.98886
3.100799	3.407389	3.381349	34.80931
3.349249	3.456991	3.44664	35.60388
3.315883	3.45881	3.2972	35.20809
3.402	3.401526	3.37088	35.43227
3.154498	3.376657	3.445429	34.99967
3.400886	3.446477	3.377683	35.54301
3.343681	3.366481	3.42686	35.35052
3.36686	3.235737	3.317883	34.87698
3.273815	3.113552	3.397105	34.57955
3.144847	3.208308	3.360853	34.42545
3.109213	3.350509	3.422427	34.79315
3.28957	3.440883	3.339488	35.20382
3.199412	3.400312	3.488587	35.24399
3.205475	3.403615	3.390506	35.04999
3.149095	3.302187	3.474007	34.88749
3.371108	3.412039	3.412213	35.47809
3.385543	3.375849	3.45184	35.51718
3.281981	3.335749	3.361109	35.0046
3.311594	3.297874	3.433885	35.14568
3.299138	3.273478	3.367606	34.92015
3.308212	3.236007	3.394206	34.91622
3.28029	3.256427	3.366651	34.83955
3.324297	3.349093	3.416288	35.24698
3.12274	3.414263	3.446162	35.01406
3.356261	3.456586	3.367742	35.44579
3.322317	3.4146	3.390659	35.32986
3.305655	3.399639	3.379252	35.23576

3.253606	3.428416	3.461526	35.36479
3.342939	3.41797	3.375381	35.34892
3.327803	3.359539	3.294949	35.01215
3.405382	3.214508	3.3297	34.94063
3.192401	3.178183	3.423739	34.60109
3.25043	3.30522	3.383754	34.91836
3.223539	3.444051	3.43085	35.26615
3.346692	3.399571	3.367742	35.30018
3.226591	3.385553	3.423245	35.12826
3.26103	3.304613	3.416697	35.01225
3.23583	3.389732	3.540168	35.4133
3.30813	3.468111	3.467443	35.58377
3.258184	3.332447	3.471893	35.1876
3.361622	3.306703	3.407643	35.217
3.343145	3.223134	3.372295	34.91654
3.32075	3.200962	3.394956	34.86864
3.218796	3.349767	3.370948	34.91859
3.215662	3.391551	3.383907	35.03145
3.230056	3.444658	3.344893	35.09375
3.275588	3.418913	3.392092	35.24024
3.246512	3.381442	3.377052	35.06182
3.353827	3.402604	3.409842	35.41449
3.340588	3.385284	3.450135	35.43577
3.361045	3.371468	3.365679	35.2656
3.395896	3.312296	3.367197	35.21573
3.264989	3.233176	3.381707	34.78817
3.267835	3.196783	3.395093	34.74408
3.330731	3.321462	3.38152	35.1246
3.333164	3.501336	3.434226	35.63853
3.184358	3.432998	3.389909	35.06676
3.277733	3.38151	3.440365	35.2687
3.243419	3.37504	3.41888	35.13253
3.269361	3.39573	3.425513	35.24901
3.259957	3.372411	3.398213	35.11775
3.306109	3.298481	3.495765	35.27033
3.17314	3.294437	3.406466	34.77542
3.421219	3.269164	3.438677	35.33311
3.341124	3.219293	3.389568	34.9415
3.380347	3.328538	3.418897	35.33031
3.217023	3.315599	3.434704	34.97942
3.238387	3.397819	3.314132	34.94227
3.292168	3.429629	3.403397	35.32465
3.393215	3.478557	3.296739	35.41938
3.235252	3.411028	3.423177	35.20276
3.286972	3.408467	3.354237	35.15951
3.232902	3.380229	3.492082	35.28096
3.360839	3.375107	3.316451	35.16546
3.319595	3.263773	3.430935	35.08215
3.256864	3.186877	3.331576	34.55953
3.242965	3.200962	3.411326	34.73433

3.296045	3.37942	3.348696	35.10371
3.295468	3.460765	3.443246	35.4871
3.168108	3.395662	3.410507	34.99462
3.141547	3.3652	3.461031	34.98041
3.251997	3.413117	3.450459	35.30361
3.344382	3.492305	3.399458	35.56728
3.241645	3.384677	3.438063	35.19167
3.284415	3.281228	3.402237	34.98063
3.159859	3.239174	3.534643	34.90583
3.418868	3.247396	3.290055	34.95535
3.3808	3.20878	3.403056	35.03477
3.384389	3.235804	3.347059	34.97926
3.231912	3.288978	3.412895	34.90607
3.281074	3.366211	3.387948	35.12792
3.208156	3.458001	3.37591	35.14287
3.36521	3.443647	3.296927	35.28221
3.224364	3.382723	3.423876	35.11858
3.239954	3.460225	3.435181	35.34688
3.259297	3.388721	3.436562	35.23584
3.411362	3.372479	3.32409	35.2869
3.352054	3.233783	3.369993	34.95428
3.221972	3.170837	3.405239	34.60923
3.202423	3.270243	3.429043	34.83592
3.335268	3.376994	3.343188	35.17213
3.312419	3.387912	3.385953	35.23956
3.29122	3.375781	3.395349	35.18722
3.106449	3.375444	3.428412	34.85472
3.226385	3.394517	3.435198	35.17355
3.345867	3.493248	3.429384	35.63804
3.296788	3.274488	3.433203	35.06067
3.303139	3.239511	3.411104	34.94974
3.245522	3.219428	3.435317	34.83277
3.293983	3.270984	3.387249	34.94638
3.356879	3.294774	3.349395	35.05316
3.256823	3.364526	3.435607	35.17543
3.176934	3.308994	3.438864	34.88641
3.304665	3.362842	3.402851	35.20473
3.267917	3.470335	3.386686	35.32409
3.322317	3.460293	3.334253	35.30643
3.231747	3.423092	3.375449	35.11711
3.226426	3.392293	3.462753	35.22904
3.15557	3.294033	3.4279	34.78299
3.433262	3.276847	3.355806	35.19502
3.254307	3.273275	3.404232	34.90176
3.344053	3.280689	3.368748	35.03663
3.247048	3.388249	3.45907	35.25724
3.380677	3.404154	3.367077	35.38307
3.303758	3.364998	3.33901	35.06786
3.321616	3.351452	3.423313	35.26164
3.151446	3.411635	3.439069	35.05557

3.258967	3.408265	3.432879	35.2698
3.245893	3.375714	3.401351	35.10108
3.289116	3.218821	3.403397	34.85698
3.338897	3.220641	3.424404	35.01576
3.201185	3.287698	3.439836	34.89499
3.277651	3.277723	3.404232	34.96254
3.276867	3.359202	3.381571	35.08945
3.393998	3.403952	3.386499	35.45423
3.300994	3.363583	3.400345	35.19285
3.257029	3.418846	3.348491	35.10416
3.262267	3.475389	3.356471	35.25671
3.372016	3.460765	3.34005	35.42883
3.275176	3.369177	3.356147	35.05197
3.288415	3.283047	3.398708	34.98564
3.200855	3.268827	3.3474	34.65086
3.31444	3.252585	3.369157	34.91131
3.371974	3.289787	3.376319	35.13415
3.400803	3.346869	3.416015	35.40883
3.242717	3.459484	3.481902	35.45348
3.350816	3.419048	3.393371	35.40784
3.214672	3.369042	3.342028	34.88848
3.299675	3.420733	3.359557	35.22574
3.20267	3.443916	3.439904	35.24001
3.210465	3.430505	3.447339	35.24399
3.072135	3.304344	3.470103	34.71537
3.299551	3.1767	3.335037	34.63819
3.282229	3.216665	3.476787	34.99769
3.377212	3.268019	3.419033	35.19141
3.218343	3.27031	3.40517	34.81868
3.303552	3.38151	3.353692	35.13562
3.3662	3.371603	3.378723	35.3057
3.404804	3.4084	3.305777	35.31106
3.161757	3.432864	3.427968	35.10027
3.242264	3.47923	3.34994	35.20709
3.201969	3.371603	3.396252	34.98488
3.326854	3.361494	3.321635	35.0727
3.327266	3.216462	3.388613	34.90292
3.205516	3.201501	3.411172	34.65328
3.229231	3.275702	3.381929	34.80346
3.32141	3.342623	3.450783	35.30196
3.353827	3.371468	3.37886	35.27864
3.240325	3.378949	3.443383	35.18789
3.266639	3.312835	3.398964	35.00372
3.2753	3.339928	3.427747	35.14485
3.359931	3.43994	3.372841	35.42857
3.256988	3.428955	3.413048	35.26735
3.264205	3.335412	3.400959	35.05213
3.125875	3.263571	3.483812	34.77371
3.256699	3.227852	3.359506	34.70985
3.331267	3.274286	3.442292	35.1555

3.313739	3.336154	3.364042	35.08134
3.234139	3.330088	3.420107	35.01661
3.336629	3.359674	3.397531	35.25607
3.29885	3.336423	3.362712	35.04647
3.372222	3.410017	3.319384	35.27311
3.240367	3.48597	3.407251	35.34301
3.203866	3.433875	3.397224	35.12734
3.156354	3.344443	3.365474	34.75843
3.273691	3.300637	3.363292	34.91446
3.310563	3.289045	3.410559	35.07311
3.312336	3.366818	3.449436	35.33208
3.249646	3.357585	3.386499	35.03716
3.305861	3.356035	3.392893	35.17069
3.395112	3.386362	3.40367	35.45575
3.37746	3.370727	3.363565	35.29525
3.263669	3.385284	3.394002	35.14481
3.208939	3.416959	3.433288	35.1803
3.291385	3.392495	3.404915	35.24505
3.2063	3.293898	3.351714	34.72703
3.232324	3.258651	3.402715	34.81839
3.186709	3.201097	3.436886	34.6675
3.158581	3.260403	3.488058	34.84759
3.240614	3.343163	3.379746	34.9711
3.347641	3.334671	3.406858	35.24587
3.323348	3.397145	3.396662	35.30707
3.368551	3.34943	3.440791	35.39808
3.324627	3.425046	3.407285	35.39411
3.353291	3.424102	3.408734	35.4579
3.324668	3.453082	3.356897	35.34532
3.30582	3.314857	3.384436	35.06205
3.131814	3.222124	3.394615	34.501
3.316213	3.287091	3.340647	34.9283
3.21228	3.322405	3.433817	34.98199
3.307635	3.415948	3.369157	35.25368
3.205186	3.344713	3.495765	35.15073
3.312378	3.317486	3.4124	35.1433
3.259627	3.353676	3.410149	35.10216
3.350404	3.422081	3.325386	35.2649
3.307511	3.401121	3.431089	35.35642
3.268742	3.373422	3.32068	34.96962
3.089333	3.284597	3.410303	34.57903
3.18209	3.258786	3.335583	34.56202
3.215662	3.245442	3.386823	34.71831
3.225065	3.24342	3.424694	34.81727
3.205186	3.306635	3.451295	34.97021
3.358405	3.372277	3.45994	35.46773
3.427942	3.366818	3.411923	35.50285
3.456853	3.413387	3.38834	35.61635
3.35989	3.387305	3.354169	35.27254
3.262968	3.381981	3.426348	35.20679

3.263339	3.463258	3.362917	35.24662
3.256946	3.350643	3.370794	35.0036
3.34496	3.26411	3.306953	34.86723
3.139815	3.231559	3.387045	34.52257
3.214136	3.296054	3.346786	34.7381
3.270392	3.385823	3.416561	35.21002
3.345084	3.363583	3.382679	35.25063
3.267092	3.328336	3.378365	34.99356
3.277321	3.334738	3.371971	35.01595
3.299262	3.379488	3.367111	35.15116
3.347517	3.475456	3.426945	35.5974
3.320131	3.403817	3.326205	35.16055
3.254678	3.2817	3.428565	34.97421
3.145424	3.230548	3.382338	34.52234
3.225766	3.238365	3.399782	34.75327
3.194091	3.264379	3.400822	34.74317
3.282187	3.326247	3.38007	35.02573
3.192648	3.380499	3.461645	35.12696
3.349868	3.425316	3.423467	35.48529
3.264824	3.388586	3.413133	35.19639
3.314728	3.354754	3.315428	35.01787
3.284002	3.364392	3.492628	35.35926
3.330813	3.419452	3.34711	35.26382
3.242346	3.347678	3.43322	35.1017
3.266845	3.261818	3.341329	34.76657
3.28458	3.250766	3.38834	34.88399
3.23253	3.370053	3.325846	34.89436
3.235706	3.375242	3.435403	35.15223
3.283837	3.424237	3.383037	35.25012
3.298437	3.324899	3.471688	35.25868
3.321657	3.38434	3.357562	35.18987
3.341207	3.384273	3.444986	35.42365
3.264123	3.477613	3.41003	35.38276
3.303634	3.409141	3.407916	35.3148
3.233396	3.261953	3.434363	34.89716
3.345785	3.250631	3.336146	34.90339
3.199536	3.215991	3.40662	34.66193
3.153962	3.262695	3.375023	34.59531
3.174212	3.270782	3.489422	34.90745
3.26371	3.351991	3.337049	34.94755
3.217724	3.375107	3.414924	35.06783
3.386492	3.404356	3.363479	35.38836
3.223746	3.371198	3.494998	35.24756
3.398865	3.453553	3.32484	35.43851
3.39107	3.425585	3.443178	35.61909
3.448233	3.407254	3.345371	35.49012
3.28924	3.225089	3.391461	34.84485
3.228653	3.16558	3.409416	34.62149
3.129381	3.360685	3.390165	34.78896
3.3337	3.4146	3.351765	35.2697

3.189637	3.429359	3.379866	35.04838
3.191823	3.320788	3.466437	35.00505
3.222838	3.352328	3.354988	34.89813
3.349909	3.424372	3.436545	35.51192
3.337	3.489811	3.355039	35.44855
3.316914	3.410893	3.395485	35.32049
3.204856	3.236141	3.448481	34.80918
3.287219	3.235872	3.370385	34.81792
3.350239	3.284193	3.316315	34.94316
3.283136	3.282643	3.330757	34.82461
3.205681	3.251238	3.445293	34.83702
3.18873	3.359135	3.445003	35.03527
3.273155	3.419587	3.464101	35.39386
3.335763	3.42107	3.314541	35.20696
3.339969	3.347543	3.445975	35.34279
3.217641	3.356102	3.395553	34.98373
3.267958	3.434818	3.466283	35.42058
3.339681	3.421541	3.252576	35.08105
3.461596	3.24342	3.411121	35.30485
3.153425	3.180811	3.334799	34.32711
3.189472	3.242813	3.405443	34.69601
3.252616	3.405569	3.381861	35.13845
3.340423	3.400312	3.364332	35.28064
3.226261	3.362505	3.414122	35.05719
3.186585	3.268423	3.387999	34.70756
3.136062	3.410826	3.503541	35.16115
3.448027	3.546692	3.364247	35.83587
3.329824	3.489677	3.45034	35.64097
3.319348	3.277117	3.377973	34.99497
3.252451	3.180272	3.426144	34.74224
3.357787	3.259325	3.333775	34.94347
3.266845	3.235535	3.391273	34.81831
3.322812	3.286013	3.358125	34.9786
3.117956	3.238433	3.414583	34.55002
3.218549	3.372142	3.402391	35.03574
3.297777	3.37814	3.389807	35.1946
3.332546	3.402604	3.387658	35.31943
3.282682	3.359135	3.436085	35.22123
3.245728	3.384205	3.395639	35.10679
3.271918	3.459888	3.362337	35.25675
3.355271	3.305085	3.344688	35.0619
3.347806	3.181822	3.330996	34.74608
3.203784	3.203388	3.435454	34.70672
3.206547	3.396067	3.384453	35.02259
3.283136	3.416959	3.404232	35.27902
3.296499	3.407321	3.322266	35.10792
3.274887	3.261077	3.391938	34.89321
3.230386	3.337299	3.448277	35.08578
3.157632	3.532135	3.436085	35.32609
3.355518	3.508345	3.400004	35.62791

3.273155	3.359202	3.409484	35.14237
3.40068	3.241129	3.356352	35.04685
3.223993	3.236883	3.435198	34.8236
3.327143	3.309398	3.389312	35.10741
3.233891	3.317823	3.385766	34.91415
3.230097	3.268895	3.319913	34.65484
3.219209	3.302052	3.39303	34.86344
3.228489	3.407119	3.362456	35.04664
3.182461	3.382857	3.460827	35.10805
3.28957	3.407052	3.436272	35.34149
3.238057	3.346128	3.43779	35.09893
3.39305	3.427742	3.434363	35.60885
3.257936	3.354417	3.370044	35.01238
3.333247	3.269232	3.361621	34.97236
3.332958	3.217541	3.301292	34.72676
3.24977	3.372479	3.376114	35.04729
3.19801	3.446275	3.388613	35.12281
3.300912	3.4208	3.369516	35.25037
3.2501	3.347139	3.422034	35.09302
3.249894	3.345858	3.394206	35.02891
3.310687	3.406513	3.467443	35.45465
3.19537	3.418981	3.424149	35.13506
3.254348	3.361494	3.395843	35.07643
3.221312	3.246318	3.409126	34.78136
3.382244	3.24497	3.376728	35.05949
3.261071	3.274825	3.392723	34.89477
3.28858	3.253529	3.394394	34.91201
3.255255	3.236276	3.362934	34.73261
3.289281	3.334064	3.393575	35.08788
3.289281	3.381981	3.405324	35.21835
3.315017	3.473569	3.429878	35.52862
3.192689	3.384475	3.371408	34.93841
3.249853	3.31479	3.35746	34.88052
3.304253	3.386295	3.387522	35.2216
3.382079	3.51131	3.438267	35.77615
3.291014	3.365066	3.462941	35.31115
3.301489	3.191325	3.313297	34.62687
3.288333	3.154325	3.390267	34.68551
3.339433	3.385553	3.295358	35.09536
3.186296	3.44331	3.409621	35.13666
3.224653	3.375107	3.364792	34.97335
3.186296	3.322001	3.410985	34.87436
3.232695	3.436907	3.408444	35.22155
3.335804	3.483813	3.420329	35.5756
3.29852	3.412511	3.497743	35.50743
3.164066	3.311151	3.431788	34.84751
3.287755	3.258516	3.419374	34.97574
3.333041	3.313779	3.354016	35.0527
3.261978	3.299155	3.403533	34.9736
3.278558	3.263975	3.370692	34.86111

3.235087	3.212351	3.341346	34.58898
3.321534	3.337569	3.337288	35.04298
3.327555	3.334604	3.33207	35.03825
3.26569	3.318968	3.395434	35.00734
3.1738	3.388788	3.364895	34.89229
3.226014	3.390136	3.464663	35.2276
3.297035	3.446005	3.392433	35.34713
3.402412	3.365403	3.39673	35.4107
3.243212	3.277252	3.389091	34.85308
3.288869	3.242544	3.405665	34.91327
3.214425	3.356844	3.446861	35.09052
3.37647	3.466156	3.380872	35.53963
3.243501	3.395662	3.364519	35.05893
3.186791	3.318901	3.355857	34.7481
3.15722	3.290461	3.444184	34.8144
3.259586	3.49554	3.433271	35.46287
3.309202	3.522295	3.480794	35.7338
3.278599	3.324225	3.368902	34.98904
3.136227	3.168478	3.45005	34.51456
3.272825	3.228728	3.405733	34.84812
3.441799	3.400245	3.381247	35.53918
3.314852	3.400717	3.377871	35.25521
3.21628	3.280823	3.439205	34.91159
3.235954	3.250833	3.394462	34.79118
3.290147	3.331032	3.413901	35.12759
3.321822	3.391619	3.3489	35.1872
3.292086	3.385958	3.44918	35.32909
3.102036	3.374501	3.478236	34.95197
3.259132	3.349093	3.419852	35.11227
3.340671	3.316273	3.359557	35.08696
3.38542	3.265795	3.349463	35.05235
3.21859	3.2416	3.427542	34.80536
3.327266	3.356102	3.415811	35.26776
3.253565	3.399706	3.435386	35.24475
3.464236	3.481118	3.256992	35.49337
3.246182	3.327527	3.426741	35.05186
3.322029	3.338175	3.357187	35.0889
3.075475	3.406176	3.444764	34.88995
3.257648	3.518791	3.398213	35.43281
3.25307	3.404423	3.420125	35.22061
3.337866	3.224752	3.399901	34.96891
3.138578	3.203321	3.441746	34.57774
3.341495	3.281363	3.417669	35.1395
3.34496	3.435425	3.425632	35.5014
3.385997	3.338512	3.383737	35.28759
3.237892	3.234052	3.350622	34.66285
3.152394	3.270917	3.378723	34.61795
3.245068	3.42107	3.355601	35.09841
3.308501	3.453217	3.381639	35.36437
3.187781	3.416352	3.373591	35.00216

3.18143	3.351789	3.442957	34.99877
3.194875	3.264649	3.438677	34.82825
3.423281	3.308387	3.451005	35.45035
3.429426	3.296324	3.281922	35.06765
3.223787	3.241196	3.411514	34.78079
3.305779	3.349632	3.357562	35.07924
3.306686	3.395528	3.42524	35.32959
3.350033	3.419587	3.34653	35.30487
3.308913	3.380162	3.311728	35.05263
3.239665	3.369446	3.422785	35.12062
3.077249	3.481859	3.442684	35.05479
3.312336	3.467032	3.453	35.55903
3.23682	3.248677	3.427133	34.85981
3.195865	3.233176	3.446537	34.77878
3.19071	3.215586	3.391819	34.60938
3.299922	3.371401	3.470256	35.36048
3.338031	3.420935	3.416169	35.43386
3.381914	3.288102	3.390472	35.18315
3.26635	3.231896	3.38406	34.79349
3.325452	3.33858	3.379593	35.14627
3.20498	3.367492	3.388051	34.96454
3.264082	3.464876	3.396815	35.32592
3.13899	3.476804	3.426195	35.1427
3.298479	3.361426	3.421011	35.22782
3.279837	3.313509	3.41963	35.07925
3.390369	3.26876	3.379883	35.13618
3.250224	3.24315	3.475423	34.98264
3.354982	3.405165	3.383481	35.36496
3.320255	3.404019	3.408836	35.34196
3.295757	3.37942	3.373113	35.15648
3.307098	3.336491	3.331644	34.99671
3.182873	3.366076	3.414463	34.97086
3.211455	3.427944	3.413236	35.16598
3.231046	3.503223	3.414924	35.37713
3.182296	3.33393	3.508929	35.10588
3.27497	3.192066	3.424183	34.81298
3.251337	3.231626	3.419818	34.83827
3.246388	3.327056	3.405869	35.00563
3.351765	3.446545	3.496243	35.69501
3.195411	3.325775	3.439632	34.96519
3.316213	3.281565	3.348099	34.93251
3.324668	3.295785	3.394803	35.08423
3.361292	3.401256	3.367657	35.33561
3.203825	3.394786	3.416373	35.08364
3.251997	3.409276	3.355994	35.08863
3.116636	3.364661	3.446452	34.89287
3.333206	3.293224	3.289578	34.86719
3.246017	3.244026	3.515545	35.06309
3.301613	3.295044	3.341056	34.91466
3.244243	3.307377	3.393712	34.93132

3.280496	3.426124	3.417192	35.32163
3.368799	3.386429	3.364332	35.31233
3.358818	3.318429	3.337936	35.08407
3.214095	3.337232	3.350674	34.83656
3.236696	3.380499	3.404829	35.09904
3.232407	3.487789	3.369516	35.24706
3.226591	3.460765	3.368475	35.17297
3.237521	3.318631	3.440245	35.04299
3.1592	3.217339	3.450391	34.67239
3.311594	3.260403	3.426724	35.04807
3.27695	3.338445	3.382372	35.04599
3.349332	3.395932	3.442547	35.46158
3.291137	3.35435	3.421813	35.19805
3.310934	3.285069	3.400072	35.04229
3.314316	3.312566	3.341432	34.98158
3.454667	3.325438	3.3474	35.32971
3.094489	3.319103	3.432061	34.71334
3.197762	3.449375	3.383805	35.11854
3.184482	3.395325	3.436409	35.08633
3.292086	3.330088	3.332582	34.95193
3.25043	3.23567	3.390318	34.78062
3.290519	3.214373	3.422904	34.89297
3.129876	3.406378	3.394172	34.89872
3.417342	3.405637	3.342557	35.41287
3.34995	3.39856	3.354715	35.27661
3.412517	3.334334	3.360359	35.28532
3.216528	3.342421	3.432879	35.033
3.328628	3.443647	3.339164	35.29457
3.232407	3.481859	3.378314	35.25333
3.273196	3.430168	3.354425	35.17725
3.161179	3.216799	3.444594	34.66287
3.229272	3.226167	3.380429	34.69194
3.209434	3.312229	3.499431	35.097
3.414991	3.486913	3.420534	35.75599
3.383729	3.313644	3.395962	35.25498
3.34232	3.159919	3.401538	34.84045
3.2647	3.252653	3.377615	34.82118
3.289941	3.389597	3.396576	35.21732
3.224859	3.419587	3.354698	35.049
3.207578	3.421744	3.403312	35.12224
3.141341	3.372277	3.460349	34.99394
3.226261	3.223808	3.345831	34.60454
3.344259	3.238163	3.362882	34.93126
3.256039	3.299559	3.38609	34.92335
3.31246	3.439738	3.444338	35.48066
3.247625	3.418778	3.419647	35.23905
3.407691	3.360146	3.308181	35.21711
3.46671	3.334469	3.354306	35.39089
3.333288	3.337502	3.382577	35.16758
3.193927	3.416487	3.426741	35.13212

3.325988	3.473232	3.445702	35.58648
3.230798	3.389732	3.392637	35.07967
3.277733	3.237152	3.319589	34.68889
3.173511	3.133366	3.449862	34.5189
3.207496	3.366076	3.506201	35.22532
3.191493	3.489811	3.402033	35.23312
3.329205	3.436503	3.410507	35.43623
3.271134	3.29902	3.435045	35.06224
3.433633	3.214912	3.37105	35.09373
3.246759	3.298683	3.449504	35.03982
3.312543	3.393101	3.39528	35.27158
3.23319	3.433605	3.377564	35.14788
3.282517	3.386766	3.366651	35.12946
3.197597	3.215384	3.388954	34.61774
3.207619	3.229267	3.385578	34.66263
3.249564	3.268625	3.416987	34.90911
3.32108	3.409343	3.332207	35.18783
3.347929	3.436368	3.43941	35.54008
3.345414	3.385486	3.35792	35.24511
3.339433	3.30522	3.418812	35.18966
3.390575	3.331503	3.339556	35.18566
3.343805	3.452273	3.426246	35.53706
3.271629	3.447555	3.427082	35.37073
3.218466	3.371872	3.424473	35.08326
3.229685	3.223067	3.404915	34.73961
3.270969	3.149406	3.374017	34.60124
3.2282	3.337636	3.472729	35.13521
3.334154	3.478691	3.423552	35.56784
3.168232	3.431044	3.466232	35.19413
3.371232	3.36729	3.399322	35.35231
3.422415	3.254136	3.405034	35.22928
3.363107	3.304411	3.331235	35.04815
3.220487	3.399436	3.408751	35.11358
3.277651	3.419385	3.367162	35.19126
3.189926	3.402334	3.44664	35.13594
3.320585	3.326988	3.246864	34.82002
3.193638	3.182496	3.428054	34.62266
3.17714	3.30212	3.397753	34.78192
3.157385	3.398762	3.491741	35.1556
3.291673	3.44769	3.412213	35.38235
3.38608	3.394719	3.438864	35.53124
3.43652	3.31169	3.393064	35.35982
3.219992	3.318294	3.415606	34.95004
3.366819	3.408332	3.371084	35.37067
3.364097	3.41952	3.337118	35.3149
3.34199	3.372883	3.380224	35.25884
3.187823	3.27341	3.406961	34.76263
3.166128	3.156819	3.402459	34.45038
3.185719	3.306568	3.411445	34.84035
3.309573	3.485296	3.44664	35.57901

3.280991	3.437514	3.425155	35.36503
3.143403	3.358865	3.459411	34.96707
3.300005	3.247127	3.38713	34.90711
3.400721	3.254001	3.344569	35.04932
3.346981	3.35772	3.342148	35.15332
3.209269	3.416487	3.458712	35.23559
3.219085	3.382857	3.44959	35.16356
3.217889	3.308792	3.298769	34.66916
3.386038	3.216665	3.378587	35.00996
3.275094	3.221719	3.335822	34.68487
3.203248	3.319912	3.415879	34.91756
3.114409	3.42909	3.402186	34.93209
3.410042	3.432796	3.372295	35.52133
3.37845	3.394517	3.31507	35.24339
3.342609	3.319575	3.353334	35.08481
3.218673	3.323416	3.458508	35.05218
3.360055	3.44331	3.374119	35.439
3.253523	3.355698	3.380429	35.02824
3.378944	3.269636	3.358926	35.06729
3.106408	3.118135	3.40089	34.23176
3.20399	3.248272	3.340323	34.59729
3.214466	3.446342	3.423381	35.23498
3.324544	3.498303	3.412724	35.56603
3.323967	3.355361	3.443178	35.31877
3.299427	3.296998	3.460622	35.17563
3.202258	3.268693	3.486216	34.9572
3.419281	3.372344	3.342591	35.34438
3.392514	3.381105	3.448004	35.53553
3.338443	3.422215	3.390983	35.38249
3.254472	3.253462	3.334048	34.70531
3.254142	3.18445	3.363036	34.61707
3.20498	3.230615	3.320748	34.51803
3.294932	3.327258	3.322027	34.92888
3.146373	3.435357	3.461526	35.14547
3.23517	3.474041	3.459343	35.41947
3.34661	3.421002	3.423654	35.46914
3.486549	3.345724	3.371681	35.49689
3.296705	3.389462	3.36145	35.155
3.211249	3.478624	3.439427	35.33363
3.247337	3.427809	3.45121	35.32719
3.205516	3.263503	3.320936	34.59154
3.316626	3.137207	3.343921	34.60859
3.255338	3.195301	3.415418	34.75796
3.153632	3.401526	3.378161	34.90505
3.16452	3.450319	3.51962	35.34491
3.350033	3.400784	3.366242	35.30686
3.284703	3.318362	3.475372	35.2224
3.315553	3.34195	3.419715	35.21974
3.276042	3.30987	3.424183	35.07295
3.375521	3.405367	3.411258	35.47106

3.291261	3.422755	3.394257	35.28765
3.384265	3.341141	3.437364	35.40682
3.248368	3.237018	3.389807	34.77794
3.220694	3.198603	3.315019	34.46986
3.171531	3.281026	3.379832	34.68433
3.227829	3.404693	3.405119	35.13319
3.269979	3.456451	3.405051	35.3384
3.263917	3.443916	3.423876	35.3389
3.274599	3.393236	3.409263	35.21947
3.399319	3.417767	3.392757	35.50977
3.322895	3.474176	3.406193	35.49538
3.377377	3.452003	3.386431	35.52282
3.24511	3.315666	3.367197	34.89336
3.186874	3.191796	3.360205	34.47984
3.321121	3.207971	3.329257	34.7411
3.272083	3.326314	3.396457	35.03962
3.209145	3.453419	3.421506	35.23472
3.208073	3.413791	3.417857	35.13774
3.28825	3.355226	3.479788	35.32043
3.281775	3.38461	3.367657	35.12532
3.316254	3.327999	3.432316	35.21832
3.153962	3.340332	3.412861	34.84783
3.294231	3.437716	3.471808	35.49645
3.259132	3.40503	3.395229	35.18075
3.411032	3.268693	3.358926	35.1354
3.311388	3.218282	3.389636	34.87441
3.169304	3.232907	3.391256	34.59922
3.172975	3.344982	3.347877	34.75747
3.331721	3.44924	3.339164	35.31357
3.227251	3.424911	3.379764	35.12069
3.330071	3.388653	3.390574	35.28989
3.200319	3.318092	3.453034	34.98843
3.333742	3.404963	3.387522	35.32691
3.409423	3.454834	3.437705	35.71121
3.414579	3.378679	3.383702	35.43786
3.222632	3.218552	3.470921	34.85866
3.253853	3.203927	3.305521	34.53325
3.186998	3.250159	3.411377	34.71964
3.3622	3.450116	3.303611	35.30439
3.206176	3.466358	3.438199	35.29303
3.108305	3.341073	3.377086	34.67138
3.200443	3.278262	3.438404	34.8696
3.303634	3.299694	3.443502	35.15328
3.291055	3.362168	3.462941	35.3049
3.243625	3.390271	3.412758	35.1529
3.223416	3.373759	3.439069	35.13013
3.333206	3.254203	3.394394	35.01108
3.487703	3.21498	3.311097	35.08101
3.225189	3.216867	3.443195	34.79994
3.199577	3.320923	3.351441	34.77083

3.210836	3.392832	3.475627	35.22428
3.372964	3.48415	3.305129	35.40567
3.4166	3.396134	3.359489	35.4275
3.299262	3.393775	3.347178	35.13882
3.109501	3.345184	3.434925	34.80947
3.293241	3.425652	3.377427	35.26151
3.311429	3.358798	3.405989	35.21754
3.42324	3.228391	3.387556	35.13657
3.201804	3.122246	3.324158	34.28156
3.2826	3.27833	3.411053	34.9896
3.285074	3.449038	3.35035	35.23558
3.342032	3.483746	3.439717	35.63147
3.150044	3.391349	3.393848	34.90925
3.217806	3.301446	3.432879	34.94619
3.16386	3.315396	3.413457	34.81625
3.29489	3.341343	3.425973	35.18691
3.341289	3.424035	3.413781	35.44255
3.266515	3.369244	3.460213	35.26075
3.219456	3.274825	3.350725	34.71193
3.324957	3.143542	3.375927	34.71066
3.317327	3.1674	3.365474	34.72329
3.34232	3.343365	3.317611	35.05808
3.131608	3.436435	3.474007	35.14283
3.205103	3.498438	3.36602	35.20299
3.254884	3.395393	3.408922	35.18033
3.316172	3.37814	3.306135	35.05185
3.233479	3.374298	3.494793	35.27518
3.236861	3.453553	3.371425	35.18611
3.231499	3.388856	3.523713	35.36593
3.352219	3.231087	3.307908	34.81297
3.380347	3.163693	3.346803	34.81216
3.197803	3.276915	3.383907	34.74171
3.279094	3.476872	3.424268	35.44502
3.278764	3.479702	3.447049	35.5003
3.317822	3.406176	3.332684	35.17483
3.249811	3.314116	3.382458	34.93362
3.265896	3.365672	3.428497	35.18223
3.129669	3.385149	3.444679	34.9623
3.273073	3.334671	3.375773	35.01483
3.199701	3.314857	3.445821	34.96423
3.349084	3.340939	3.372994	35.18868
3.230592	3.181013	3.466692	34.78473
3.369335	3.186135	3.343597	34.83015
3.252863	3.248744	3.410831	34.85939
3.278104	3.377803	3.320816	34.99997
3.207826	3.404626	3.408734	35.0972
3.335639	3.465347	3.349736	35.38048
3.190751	3.389529	3.407506	35.02416
3.271794	3.440277	3.341773	35.16862
3.275382	3.437379	3.355874	35.20097

3.32009	3.365537	3.471603	35.39471
3.251131	3.289787	3.315087	34.73598
3.260906	3.142532	3.428395	34.68312
3.279053	3.201029	3.366429	34.71522
3.331185	3.401997	3.360086	35.25483
3.250059	3.451666	3.376319	35.22154
3.219539	3.358394	3.396986	34.99602
3.290024	3.347139	3.437159	35.2134
3.183409	3.363246	3.409825	34.9557
3.25575	3.386699	3.412537	35.17112
3.156065	3.36237	3.469045	35.02349
3.135361	3.351856	3.448277	34.90981
3.206093	3.312701	3.445565	34.97293
3.360591	3.249081	3.363309	34.99179
3.29687	3.2199	3.316059	34.68529
3.311388	3.273814	3.403244	35.02561
3.160107	3.335345	3.483079	35.00392
3.356137	3.417296	3.305402	35.22327
3.340712	3.405165	3.343188	35.24564
3.365582	3.424102	3.440109	35.55339
3.130783	3.395932	3.365747	34.8157
3.292869	3.419991	3.426741	35.35616
3.206465	3.41952	3.371886	35.04621
3.335061	3.310072	3.373813	35.0923
3.186255	3.185529	3.448157	34.65711
3.258885	3.141116	3.393166	34.59856
3.29786	3.305894	3.414583	35.09097
3.33799	3.440344	3.361314	35.35626
3.33568	3.439266	3.36781	35.36306
3.221807	3.307377	3.433152	34.96851
3.143857	3.291067	3.41078	34.71345
3.358405	3.424439	3.422256	35.49939
3.198175	3.453958	3.441746	35.25617
3.194916	3.360752	3.399663	34.95319
3.163984	3.228661	3.494538	34.80416
3.310687	3.253866	3.381025	34.93186
3.418291	3.248744	3.403823	35.20583
3.326483	3.291202	3.327313	34.93059
3.254884	3.291741	3.43941	35.02033
3.253936	3.338041	3.285161	34.78219
3.326029	3.415004	3.422444	35.40837
3.344011	3.447623	3.339829	35.33836
3.401546	3.43037	3.418419	35.59832
3.107315	3.449038	3.387317	34.92769
3.244573	3.436772	3.37579	35.17582
3.27596	3.32928	3.439154	35.14795
3.250059	3.238905	3.372858	34.7487
3.198505	3.155943	3.371886	34.45241
3.254472	3.232435	3.415947	34.83843
3.228571	3.433403	3.402664	35.19222

3.495375	3.472963	3.346104	35.7385
3.28392	3.325977	3.439683	35.15929
3.340134	3.305557	3.37076	35.08685
3.171614	3.348689	3.407575	34.89315
3.317533	3.455845	3.365219	35.35396
3.152518	3.370996	3.464714	35.02513
3.24944	3.326786	3.390557	34.97823
3.193225	3.253933	3.510907	34.95917
3.404887	3.221315	3.385868	35.07727
3.284745	3.226235	3.410712	34.87962
3.414001	3.303603	3.39025	35.28673
3.297159	3.333121	3.451022	35.22867
3.246388	3.378814	3.405648	35.11834
3.302232	3.440547	3.321089	35.19054
3.288044	3.409545	3.342199	35.13788
3.260328	3.38151	3.370743	35.07839
3.206506	3.410422	3.451909	35.20141
3.210836	3.383936	3.437705	35.12189
3.244615	3.337771	3.378382	34.96508
3.311017	3.219023	3.385049	34.86519
3.21925	3.204534	3.416441	34.70147
3.309903	3.344578	3.427491	35.23013
3.291343	3.437851	3.426536	35.39143
3.351023	3.363111	3.340221	35.16974
3.340588	3.382588	3.355857	35.22371
3.33436	3.354754	3.357358	35.1525
3.13235	3.395191	3.463981	35.03233
3.276578	3.429764	3.42067	35.32863
3.159406	3.355698	3.415538	34.8992
3.254018	3.280891	3.376473	34.85708
3.20267	3.220506	3.438949	34.74936
3.300128	3.268356	3.424012	35.03446
3.225395	3.299357	3.374887	34.8314
3.308954	3.379825	3.461918	35.38042
3.270969	3.326786	3.399168	35.04414
3.402329	3.387373	3.393916	35.45242
3.282682	3.42909	3.344535	35.17401
3.247873	3.438794	3.389773	35.21803
3.222756	3.465213	3.368492	35.17434
3.228324	3.385149	3.380292	35.03724
3.224653	3.334806	3.405597	34.97445
3.222013	3.237826	3.41726	34.78211
3.217641	3.244498	3.437637	34.8317
3.395071	3.326988	3.345626	35.19889
3.340093	3.410489	3.45242	35.49481
3.331391	3.428483	3.403874	35.40896
3.267422	3.340534	3.411377	35.09315
3.288539	3.332717	3.33398	34.95298
3.31477	3.327999	3.418147	35.18408
3.184935	3.405165	3.378996	34.98329

3.133216	3.375983	3.473701	35.01348
3.165963	3.258449	3.387522	34.6396
3.28062	3.217339	3.418419	34.868
3.293488	3.206016	3.350503	34.72286
3.349909	3.263301	3.424814	35.13402
3.21195	3.345858	3.400413	34.95951
3.394246	3.392427	3.41992	35.50266
3.422704	3.441827	3.382065	35.59014
3.410289	3.417228	3.321567	35.3769
3.264824	3.372411	3.40205	35.13678
3.298644	3.410893	3.357358	35.19716
3.22024	3.344847	3.370385	34.90976
3.306562	3.360685	3.266456	34.90589
3.178708	3.295987	3.443298	34.87153
3.138124	3.251709	3.367197	34.51954
3.167077	3.291269	3.437858	34.82389
3.273733	3.401391	3.407217	35.23094
3.321245	3.418374	3.439291	35.44212
3.462132	3.361426	3.363923	35.46086
3.169016	3.321529	3.477674	34.98137
3.324586	3.355496	3.332275	35.07789
3.314399	3.460562	3.437671	35.51586
3.258926	3.426663	3.377615	35.18909
3.155199	3.231559	3.436767	34.66495
3.14794	3.230076	3.412537	34.59284
3.270351	3.287226	3.418027	34.99752
3.350528	3.295987	3.394854	35.14134
3.341825	3.383531	3.452574	35.43998
3.213311	3.348621	3.418692	35.0085
3.308996	3.341815	3.370027	35.09644
3.41363	3.381442	3.338618	35.34323
3.400144	3.476535	3.299809	35.43682
3.250059	3.31944	3.388886	34.95987
3.203289	3.350306	3.378365	34.90208
3.221601	3.348554	3.444747	35.08346
3.323431	3.33676	3.330757	35.03108
3.168232	3.257707	3.38493	34.63727
3.268618	3.320855	3.408325	35.04606
3.139361	3.343297	3.466846	34.94045
3.402371	3.418981	3.417379	35.57294
3.393297	3.379892	3.364656	35.35232
3.370778	3.389462	3.413986	35.43188
3.261978	3.351856	3.417925	35.12033
3.354033	3.352665	3.410831	35.30789
3.222467	3.359	3.355874	34.91385
3.32174	3.300772	3.413969	35.13065
3.125462	3.233378	3.378621	34.47674
3.086281	3.246992	3.405034	34.47859
3.258514	3.243487	3.387573	34.80939
3.280043	3.318699	3.431771	35.1176

3.359148	3.42262	3.395417	35.43835
3.241027	3.383397	3.446793	35.20661
3.279012	3.382251	3.411991	35.21107
3.413177	3.430842	3.37666	35.53346
3.338031	3.430168	3.379388	35.37362
3.2647	3.377331	3.320322	34.96854
3.204691	3.277521	3.391052	34.77372
3.220116	3.281295	3.382577	34.79717
3.28726	3.279678	3.397071	34.97217
3.248945	3.340669	3.364588	34.95072
3.273238	3.316138	3.43143	35.09637
3.16918	3.39108	3.415964	34.99888
3.298602	3.425046	3.419511	35.36394
3.3662	3.375646	3.358671	35.27069
3.340629	3.455441	3.436272	35.55897
3.20201	3.411298	3.452864	35.19558
3.342609	3.405165	3.397071	35.36762
3.24247	3.285204	3.428019	34.95398
3.279713	3.194964	3.346854	34.66059
3.152436	3.246587	3.391614	34.59303
3.076053	3.24524	3.422631	34.49087
3.169593	3.265188	3.403278	34.69673
3.307305	3.38899	3.393319	35.24684
3.317822	3.38151	3.434022	35.34249
3.376305	3.349026	3.367589	35.25407
3.266845	3.403143	3.459616	35.3343
3.388513	3.462112	3.363445	35.51901
3.401505	3.424574	3.378706	35.4987
3.293323	3.380027	3.337254	35.07406
3.180357	3.279408	3.361263	34.65949
3.229478	3.272601	3.321976	34.66611
3.226468	3.269164	3.431413	34.89133
3.263917	3.379016	3.372448	35.08451
3.241728	3.367694	3.467801	35.21975
3.189596	3.38616	3.430083	35.06364
3.315801	3.324562	3.468125	35.28812
3.27996	3.376388	3.398418	35.17064
3.337041	3.422485	3.394394	35.38747
3.22457	3.425518	3.434465	35.23578
3.250348	3.409006	3.413986	35.21125
3.234263	3.247531	3.382952	34.75509
3.330112	3.1643	3.276158	34.54915
3.207083	3.222932	3.395485	34.66927
3.233891	3.324158	3.393916	34.94583
3.11709	3.319912	3.440638	34.78329
3.377748	3.394517	3.35983	35.33974
3.32174	3.345184	3.378638	35.15051
3.339887	3.337636	3.377138	35.17041
3.253977	3.445736	3.498681	35.48473
3.399319	3.442029	3.355994	35.48243

3.34397	3.389866	3.337032	35.20585
3.414579	3.269366	3.270003	34.95016
3.139609	3.263908	3.389619	34.59849
3.170294	3.371198	3.380224	34.87968
3.182502	3.35772	3.403124	34.92698
3.24412	3.390203	3.375296	35.07191
3.295427	3.391012	3.437585	35.32209
3.235087	3.351317	3.436852	35.10173
3.240284	3.358326	3.493907	35.25319
3.3735	3.428416	3.428906	35.55564
3.353415	3.409208	3.374205	35.35009
3.265566	3.28453	3.406159	34.95521
3.254596	3.264042	3.456666	34.99687
3.275424	3.310746	3.414651	35.05267
3.295798	3.275971	3.350742	34.88142
3.27629	3.26229	3.373591	34.8588
3.223003	3.330627	3.452727	35.06478
3.148641	3.406176	3.451295	35.06424
3.317244	3.451936	3.404079	35.42976
3.403113	3.365066	3.406279	35.43238
3.39371	3.394449	3.398009	35.45799
3.248079	3.419722	3.457229	35.32429
3.361622	3.364661	3.374273	35.27077
3.283012	3.286889	3.354561	34.88568
3.314976	3.252316	3.281853	34.72098
3.168314	3.226235	3.411377	34.62646
3.179326	3.348621	3.354033	34.79278
3.091684	3.382655	3.401368	34.77906
3.304046	3.439738	3.380855	35.32344
3.209764	3.359876	3.495203	35.19267
3.275135	3.415948	3.411872	35.27602
3.26833	3.411298	3.451499	35.33763
3.299221	3.439131	3.46983	35.50613
3.412434	3.364459	3.333281	35.29181
3.357787	3.297739	3.398145	35.16824
3.224777	3.255079	3.340443	34.65789
3.255998	3.209993	3.415418	34.79153
3.185224	3.196918	3.30859	34.37455
3.22721	3.247598	3.401231	34.77979
3.27996	3.337704	3.482789	35.27055
3.199164	3.445871	3.490769	35.34785
3.324462	3.418644	3.367401	35.29253
3.354033	3.33393	3.397616	35.23802
3.353621	3.42262	3.400686	35.43778
3.248657	3.483881	3.41622	35.37618
3.355766	3.393506	3.370863	35.31359
3.326565	3.227313	3.432044	35.02009
3.318935	3.158639	3.320765	34.60987
3.239088	3.315329	3.388528	34.9261
3.276248	3.351722	3.376166	35.05992

3.207991	3.389125	3.395826	35.03544
3.243584	3.309668	3.417192	34.98624
3.287013	3.350576	3.322624	34.96386
3.288663	3.398965	3.431856	35.31216
3.252286	3.474446	3.458559	35.45607
3.378367	3.429898	3.459343	35.63609
3.293488	3.326382	3.407575	35.11089
3.365169	3.332986	3.402391	35.27075
3.28858	3.28763	3.443366	35.09368
3.191246	3.210262	3.321703	34.44558
3.078816	3.21936	3.427747	34.45151
3.233768	3.324629	3.336964	34.82204
3.222797	3.351182	3.496584	35.20518
3.31976	3.437446	3.330348	35.24234
3.203784	3.359472	3.473649	35.13158
3.35028	3.384812	3.420721	35.39161
3.386575	3.437109	3.373454	35.48198
3.375026	3.434211	3.301906	35.29393
3.366778	3.284934	3.37591	35.11128
3.226756	3.261953	3.374222	34.75112
3.245687	3.22428	3.382935	34.72919
3.34694	3.387845	3.389108	35.3218
3.328298	3.457125	3.431583	35.52543
3.124225	3.355361	3.443042	34.88167
3.264412	3.313509	3.412417	35.02974
3.289735	3.366548	3.401146	35.17646
3.354941	3.475591	3.402937	35.56143
3.226426	3.452947	3.488672	35.41836
3.326442	3.365066	3.423893	35.30323
3.291508	3.229267	3.447731	34.982
3.387358	3.327056	3.298018	35.07806
3.283342	3.255349	3.412349	34.9438
3.326648	3.204332	3.362968	34.81895
3.117791	3.259123	3.43102	34.63086
3.280331	3.390743	3.345029	35.08609
3.257565	3.408265	3.400004	35.19484
3.326483	3.395123	3.356607	35.22191
3.195989	3.319507	3.404761	34.87649
3.303387	3.335682	3.424882	35.19072
3.335185	3.455036	3.516892	35.72248
3.474629	3.462584	3.323118	35.62018
3.35358	3.251777	3.363428	34.98261
3.202134	3.14826	3.316127	34.32161
3.27431	3.301244	3.374819	34.94234
3.325452	3.433875	3.356147	35.30339
3.320338	3.416352	3.328984	35.19449
3.128762	3.293628	3.464646	34.80384
3.171408	3.34013	3.413713	34.88741
3.354281	3.447151	3.494657	35.69837
3.320379	3.494866	3.413423	35.55093

3.264164	3.341276	3.450323	35.17282
3.253358	3.284732	3.468278	35.0648
3.240573	3.260403	3.476719	35.00209
3.430128	3.312835	3.349923	35.254
3.294849	3.24497	3.347417	34.80428
3.215827	3.218754	3.411769	34.71486
3.143197	3.266671	3.386823	34.60627
3.333948	3.380229	3.385322	35.26846
3.322194	3.450656	3.403005	35.43544
3.324173	3.416285	3.371681	35.2961
3.184276	3.346061	3.483778	35.08174
3.317574	3.468178	3.431379	35.5257
3.334319	3.431179	3.38331	35.37629
3.446048	3.291674	3.384708	35.31861
3.320709	3.225561	3.425973	34.99017
3.201474	3.207634	3.379508	34.58861
3.300623	3.377938	3.397821	35.21791
3.372799	3.414196	3.280489	35.19845
3.242635	3.35745	3.399714	35.05043
3.283755	3.32254	3.355175	34.96661
3.13866	3.379488	3.466147	35.01653
3.324751	3.487722	3.369498	35.44881
3.349579	3.427607	3.466522	35.58382
3.321327	3.323484	3.38834	35.12337
3.267051	3.210397	3.43247	34.85388
3.314151	3.267412	3.383037	34.97346
3.332133	3.277656	3.363854	34.99323
3.328215	3.294033	3.373676	35.04196
3.137423	3.279206	3.338567	34.51553
3.130123	3.285878	3.445361	34.74769
3.313532	3.395595	3.380906	35.24776
3.265319	3.358798	3.443929	35.19968
3.259998	3.37841	3.343921	35.01223
3.227127	3.381442	3.450869	35.18085
3.290684	3.427742	3.460895	35.44302
3.268742	3.323955	3.447714	35.13925
3.446831	3.27988	3.319248	35.15138
3.248038	3.230143	3.362337	34.70211
3.292787	3.375983	3.414583	35.23315
3.345744	3.402873	3.367862	35.30559
3.372964	3.359472	3.34283	35.21547
3.195618	3.289922	3.329393	34.64616
3.28293	3.378544	3.38123	35.14426
3.199618	3.483678	3.462873	35.37052
3.31477	3.498101	3.418266	35.55633
3.184399	3.264716	3.435335	34.79818
3.349043	3.240185	3.397872	35.02266
3.28693	3.283856	3.437517	35.06903
3.318275	3.364055	3.345984	35.11279
3.237851	3.302187	3.465823	35.06369

3.296251	3.25636	3.354919	34.84866
3.227829	3.265458	3.386277	34.7875
3.30017	3.388519	3.337987	35.10921
3.182667	3.402267	3.445429	35.11727
3.25142	3.41433	3.355653	35.09768
3.246429	3.354754	3.367469	34.98232
3.339475	3.353137	3.384248	35.21895
3.377047	3.309668	3.403482	35.24812
3.314522	3.253462	3.360904	34.89536
3.240532	3.27031	3.432248	34.92642
3.318647	3.361292	3.320526	35.05189
3.426416	3.453082	3.38048	35.6194
3.281404	3.328066	3.346342	34.95424
3.313615	3.311825	3.368203	35.03697
3.182461	3.275028	3.410183	34.76149
3.303675	3.506727	3.381861	35.47132
3.298685	3.490822	3.437449	35.54719
3.27167	3.357046	3.3725	35.05353
3.103026	3.155876	3.511504	34.54879
3.369912	3.275634	3.317935	34.97101
3.309532	3.322405	3.485381	35.30743
3.391153	3.407658	3.381179	35.44448
3.254513	3.240657	3.404352	34.83114
3.275382	3.282778	3.374188	34.90293
3.295344	3.26849	3.331729	34.82249
3.386863	3.437446	3.386465	35.5118
3.19372	3.430572	3.448413	35.20987
3.184729	3.416622	3.439495	35.1402
3.198092	3.34788	3.392228	34.91573
3.360797	3.320451	3.407438	35.24482
3.352466	3.295852	3.404761	35.16695
3.280703	3.212217	3.429656	34.88156
3.205722	3.329077	3.402391	34.91352
3.351064	3.400649	3.354118	35.28231
3.369417	3.42235	3.281768	35.21168
3.332216	3.360415	3.40176	35.25729
3.181842	3.317957	3.391768	34.81375
3.22754	3.439131	3.419374	35.23904
3.296293	3.553161	3.428292	35.65826
3.319348	3.42107	3.42258	35.40732
3.268	3.186203	3.508997	34.9704
3.137341	3.18317	3.387573	34.4125
3.295757	3.303535	3.441814	35.14076
3.441676	3.429022	3.354629	35.54363
3.393751	3.311757	3.422785	35.33143
3.202588	3.250159	3.402493	34.73431
3.271093	3.345858	3.479805	35.26246
3.287343	3.419452	3.343529	35.16092
3.380635	3.377938	3.369703	35.33139
3.189637	3.37194	3.408393	34.9852

3.169758	3.391619	3.457843	35.0929
3.199453	3.393775	3.393575	35.02201
3.371727	3.352193	3.380156	35.27847
3.24214	3.231356	3.405256	34.78572
3.366571	3.193481	3.41992	35.00708
3.162953	3.310274	3.518393	35.03255
3.386616	3.461236	3.361263	35.50817
3.379728	3.400312	3.371613	35.38251
3.389791	3.348015	3.314797	35.16591
3.160107	3.319103	3.474894	34.95051
3.260782	3.538807	3.36058	35.40114
3.247296	3.503897	3.495083	35.58944
3.325988	3.427607	3.292715	35.15215
3.232118	3.184585	3.473104	34.8099
3.200237	3.212486	3.459531	34.77151
3.306109	3.362639	3.434294	35.27621
3.310604	3.374703	3.408564	35.25615
3.306521	3.294976	3.35908	34.96466
3.225313	3.25427	3.456018	34.91004
3.27864	3.328269	3.392501	35.04958
3.300128	3.373355	3.4412	35.30167
3.358529	3.429157	3.348849	35.34945
3.219827	3.37996	3.44132	35.14077
3.29221	3.394786	3.38389	35.20589
3.203206	3.336154	3.396389	34.91037
3.419817	3.321866	3.321754	35.1896
3.274227	3.258381	3.408325	34.9217
3.267464	3.317688	3.371459	34.95599
3.312213	3.373018	3.473718	35.39847
3.433716	3.401728	3.364809	35.48879
3.355147	3.314116	3.380292	35.15924
3.371809	3.302861	3.324363	35.04876
3.144434	3.376388	3.42304	34.92811
3.18077	3.491968	3.393643	35.19604
3.18308	3.408804	3.425001	35.0878
3.285611	3.251912	3.38771	34.88737
3.246182	3.24962	3.396713	34.81582
3.27365	3.339928	3.468466	35.23029
3.230757	3.454227	3.452574	35.35169
3.371356	3.344982	3.412724	35.33311
3.349785	3.216193	3.358688	34.88613
3.360591	3.204601	3.351151	34.86793
3.286559	3.307781	3.353606	34.93704
3.217641	3.405434	3.42836	35.16336
3.282105	3.44459	3.326955	35.1682
3.358983	3.390203	3.433544	35.45047
3.266927	3.250496	3.407217	34.88607
3.235046	3.223471	3.381724	34.70151
3.313574	3.254809	3.413355	35.01094
3.277362	3.425922	3.460145	35.40826

3.342444	3.436166	3.429878	35.5068
3.272289	3.410085	3.409791	35.25242
3.380594	3.369514	3.403619	35.38705
3.254266	3.332784	3.374068	34.96584
3.359395	3.366413	3.322232	35.15593
3.26103	3.486037	3.433817	35.44644
3.160643	3.386092	3.362354	34.85207
3.12373	3.180946	3.363871	34.32604
3.273114	3.234996	3.356897	34.75566
3.236985	3.308724	3.410235	34.95453
3.316873	3.460225	3.484034	35.62193
3.279919	3.407726	3.434516	35.31802
3.387647	3.243824	3.387982	35.09341
3.395318	3.283317	3.391665	35.20461
3.414744	3.379623	3.376132	35.42372
3.277857	3.371738	3.427013	35.2184
3.216157	3.409343	3.362695	35.02506
3.237438	3.348689	3.446043	35.12122
3.333164	3.302929	3.324414	34.96451
3.32706	3.27368	3.399646	35.05172
3.149219	3.287967	3.428838	34.75789
3.144146	3.412578	3.448959	35.0633
3.388142	3.450453	3.423092	35.62314
3.40468	3.415746	3.357562	35.4401
3.355724	3.367694	3.405119	35.33196
3.235871	3.346937	3.39644	35.00549
3.270516	3.380094	3.407899	35.17882
3.32871	3.473839	3.413952	35.52433
3.347105	3.430572	3.353078	35.33681
3.18609	3.200692	3.437517	34.66664
3.145259	3.122583	3.362968	34.24351
3.259627	3.317823	3.44016	35.08938
3.266267	3.492979	3.378178	35.3514
3.33733	3.43967	3.397344	35.43213
3.150868	3.25737	3.471126	34.78706
3.32937	3.223	3.376387	34.89507
3.303139	3.33184	3.41813	35.16701
3.418167	3.399369	3.358654	35.43617
3.263917	3.466493	3.418488	35.37649
3.21661	3.399436	3.375245	35.03183
3.230303	3.228593	3.366583	34.66922
3.346073	3.286417	3.286901	34.87459
3.208898	3.270512	3.383685	34.75148
3.227416	3.309466	3.401811	34.9168
3.16155	3.395864	3.530056	35.24216
3.376882	3.462382	3.43854	35.65839
3.460318	3.363381	3.419442	35.58258
3.481228	3.406984	3.354629	35.58193
3.266432	3.397819	3.50777	35.42705
3.28161	3.458204	3.315189	35.17115

3.278929	3.43994	3.404147	35.31989
3.295715	3.271119	3.32409	34.81234
3.208857	3.185192	3.41119	34.62496
3.113543	3.242611	3.464731	34.65918
3.215951	3.370727	3.398537	35.01854
3.306769	3.471211	3.487427	35.63127
3.302026	3.389934	3.401163	35.25452
3.257978	3.254742	3.415418	34.89372
3.301201	3.242207	3.407984	34.94457
3.264412	3.362505	3.41356	35.13939
3.399525	3.466426	3.304344	35.42328
3.290642	3.398291	3.434311	35.32039
3.254059	3.339995	3.353896	34.93705
3.242058	3.220641	3.354306	34.65069
3.351683	3.259729	3.367469	35.00469
3.245769	3.378073	3.3895	35.08005
3.256781	3.454834	3.43513	35.37178
3.214177	3.380162	3.453665	35.15585
3.346073	3.355159	3.343375	35.14842
3.342403	3.307107	3.352652	35.0556
3.334649	3.424102	3.459889	35.529
3.227622	3.424439	3.349582	35.05447
3.347435	3.39364	3.393251	35.34462
3.223663	3.313105	3.385203	34.88024
3.255709	3.196716	3.312086	34.5359
3.130618	3.184046	3.384112	34.39214
3.230262	3.391282	3.42142	35.14483
3.207001	3.492642	3.468483	35.41854
3.272371	3.453082	3.462481	35.46185
3.353868	3.31169	3.460025	35.3255
3.40633	3.198064	3.339147	34.92741
3.309367	3.364998	3.474059	35.37545
3.28792	3.426124	3.346649	35.1836
3.327266	3.422418	3.395417	35.36818
3.358653	3.347948	3.279074	35.01954
3.34166	3.183776	3.336896	34.74982
3.17945	3.177441	3.280711	34.25837
3.266804	3.291809	3.4028	34.96649
3.226261	3.468178	3.384794	35.22414
3.316461	3.451262	3.431498	35.48654
3.318935	3.380162	3.396116	35.25909
3.355518	3.301176	3.442275	35.2673
3.224694	3.362505	3.419153	35.06476
3.443243	3.478489	3.357767	35.66209
3.300871	3.399908	3.48047	35.44723
3.331061	3.286821	3.346325	34.9726
3.260947	3.146777	3.385851	34.59945
3.277651	3.215452	3.336299	34.6778
3.222962	3.414802	3.439836	35.22057
3.305449	3.484083	3.428701	35.52811

3.171861	3.380229	3.473036	35.10582
3.275836	3.316812	3.409808	35.05624
3.243047	3.279947	3.468261	35.03175
3.428684	3.385284	3.401641	35.52237
3.306109	3.414398	3.477827	35.4846
3.350115	3.370457	3.382918	35.27719
3.264205	3.320047	3.442496	35.10937
3.369541	3.241196	3.268758	34.78735
3.290766	3.163424	3.359199	34.64278
3.275094	3.334738	3.356403	34.97703
3.122122	3.4115	3.463759	35.04515
3.180151	3.414263	3.411531	35.06387
3.31877	3.419857	3.342557	35.22841
3.386822	3.31944	3.416015	35.31827
3.366283	3.34788	3.367947	35.23043
3.28293	3.423429	3.466573	35.42905
3.273403	3.481454	3.392467	35.37305
3.355023	3.416352	3.403857	35.43408
3.275382	3.253394	3.333145	34.74892
3.149961	3.110048	3.370607	34.24309
3.337247	3.239376	3.394615	34.98798
3.20432	3.401593	3.442019	35.15569
3.263257	3.470537	3.436886	35.42412
3.151817	3.37659	3.428224	34.95603
3.236572	3.295852	3.478032	35.07374
3.266969	3.294639	3.394343	34.95454
3.416641	3.383397	3.40014	35.48863
3.309449	3.418239	3.424609	35.38392
3.378243	3.458069	3.412468	35.59492
3.274269	3.299559	3.428224	35.05536
3.341207	3.193818	3.339283	34.776
3.294313	3.2354	3.351714	34.79157
3.273114	3.29255	3.359285	34.88675
3.206011	3.375646	3.412008	35.03702
3.234675	3.430033	3.367879	35.12213
3.319678	3.43374	3.417925	35.42557
3.40468	3.444186	3.322845	35.42638
3.329947	3.411972	3.425632	35.41728
3.257689	3.456721	3.396235	35.29284
3.287425	3.437986	3.434721	35.40105
3.377377	3.285945	3.323272	35.02156
3.25905	3.201097	3.362064	34.66208
3.168768	3.205882	3.387249	34.53018
3.225436	3.398897	3.444201	35.20075
3.22024	3.452947	3.432333	35.28163
3.259091	3.401997	3.422375	35.23339
3.26635	3.350509	3.401385	35.09077
3.238222	3.311353	3.463487	35.07944
3.200649	3.326314	3.448703	34.99766
3.39705	3.403682	3.400805	35.4916

3.302067	3.402941	3.378041	35.23249
3.347929	3.332312	3.400447	35.22733
3.326607	3.232772	3.35879	34.87192
3.371562	3.192875	3.360359	34.88641
3.242181	3.318227	3.322385	34.79456
3.26503	3.405097	3.403397	35.21166
3.143073	3.381577	3.418692	34.92697
3.284662	3.446477	3.352396	35.23355
3.255008	3.379825	3.381895	35.08745
3.346898	3.390877	3.365491	35.2767
3.332174	3.449847	3.409689	35.47011
3.286518	3.448162	3.419357	35.38773
3.263587	3.314992	3.392484	34.98759
3.330731	3.246992	3.393319	34.98755
3.262061	3.188494	3.388937	34.69987
3.262803	3.233243	3.354561	34.72418
3.252162	3.44951	3.475576	35.43849
3.240573	3.446275	3.438063	35.32403
3.284703	3.398291	3.465601	35.37583
3.290436	3.323955	3.389636	35.05968
3.280125	3.276241	3.486813	35.1453
3.259586	3.388384	3.431634	35.22495
3.25707	3.407187	3.456189	35.31427
3.408846	3.4177	3.352106	35.44156
3.405588	3.290663	3.331115	35.11072
3.235211	3.196379	3.421881	34.73044
3.330236	3.221315	3.393916	34.93162
3.187369	3.37012	3.421199	35.00427
3.353992	3.433672	3.355056	35.36298
3.247131	3.452947	3.421676	35.31713
3.255255	3.389529	3.419903	35.19233
3.184935	3.375309	3.392978	34.94858
3.331102	3.394382	3.375773	35.27231
3.259297	3.445466	3.431481	35.34882
3.405175	3.369379	3.359114	35.34318
3.177512	3.232974	3.460758	34.7693
3.311223	3.163154	3.293073	34.54232
3.376346	3.251372	3.370198	35.04632
3.318564	3.451869	3.349889	35.314
3.244615	3.440614	3.440007	35.32474
3.22886	3.361898	3.368407	34.96157
3.199164	3.333593	3.403209	34.91084
3.337784	3.352396	3.327688	35.08994
3.219992	3.332245	3.409536	34.96728
3.206341	3.389529	3.425973	35.09864
3.264576	3.360415	3.505178	35.33553
3.309532	3.334401	3.402476	35.15236
3.386492	3.220708	3.373813	35.00935
3.262968	3.152775	3.343597	34.52459
3.281198	3.22273	3.419852	34.88419

3.180192	3.435896	3.428224	35.14778
3.398246	3.459215	3.330774	35.46251
3.334278	3.458136	3.386294	35.44168
3.307057	3.422687	3.341756	35.20723
3.144723	3.377399	3.459258	35.01015
3.300211	3.434414	3.274129	35.07001
3.413135	3.41797	3.488041	35.74879
3.325864	3.318901	3.377342	35.09922
3.187163	3.142801	3.450749	34.57133
3.310975	3.238635	3.360444	34.85418
3.277486	3.303198	3.408427	35.02706
3.417507	3.46838	3.450323	35.78611
3.171696	3.368031	3.415128	34.95215
3.188977	3.271254	3.420721	34.79053
3.201515	3.339186	3.338294	34.78626
3.279837	3.401256	3.426536	35.28624
3.20102	3.411972	3.406262	35.09298
3.295344	3.394382	3.47174	35.40397
3.185637	3.333593	3.426724	34.93268
3.354405	3.230615	3.399782	35.01764
3.456152	3.212217	3.302042	34.98617
3.30219	3.210397	3.363292	34.77944
3.183657	3.341815	3.420858	34.9335
3.240367	3.49062	3.387846	35.31074
3.291137	3.415813	3.289373	35.04283
3.421219	3.360281	3.302742	35.23509
3.224777	3.426798	3.480112	35.33885
3.140887	3.439333	3.453921	35.12553
3.305655	3.413926	3.509543	35.55193
3.419487	3.339658	3.277215	35.13039
3.252286	3.169017	3.415282	34.69351
3.180069	3.202714	3.390387	34.55483
3.253441	3.349228	3.478577	35.22854
3.287755	3.500999	3.351424	35.35742
3.430375	3.390203	3.381042	35.49178
3.262226	3.328875	3.420721	35.07673
3.282146	3.328875	3.419101	35.11675
3.148806	3.364796	3.474076	35.02393
3.285693	3.387103	3.360342	35.12334
3.203371	3.361359	3.434005	35.04811
3.254348	3.353137	3.459872	35.19817
3.233149	3.249081	3.43588	34.87179
3.465267	3.219832	3.337885	35.10113
3.283878	3.309668	3.445156	35.13551
3.34995	3.375512	3.370539	35.26081
3.226014	3.413319	3.458167	35.26409
3.25806	3.435492	3.314456	35.06838
3.301242	3.3559	3.379201	35.13035
3.335433	3.425652	3.327279	35.24411
3.214754	3.432122	3.438523	35.23763

3.225808	3.486711	3.368288	35.22758
3.178172	3.347745	3.480811	35.06559
3.399649	3.227987	3.380838	35.0694
3.319389	3.135253	3.371391	34.67043
3.229025	3.304007	3.447936	35.00925
3.360839	3.399639	3.432777	35.47349
3.224818	3.400717	3.44282	35.20035
3.327308	3.379623	3.350674	35.17684
3.303675	3.327258	3.384316	35.08422
3.221312	3.44122	3.420073	35.23152
3.157179	3.399234	3.415418	34.98927
3.299592	3.326516	3.412963	35.13632
3.13598	3.339995	3.42553	34.83548
3.289116	3.366144	3.395843	35.16263
3.209434	3.252114	3.475082	34.91229
3.353044	3.272467	3.360359	35.01997
3.255874	3.222258	3.422427	34.83341
3.323555	3.326045	3.318207	34.98047
3.315595	3.380566	3.438608	35.34559
3.317409	3.426866	3.378178	35.31866
3.268165	3.407793	3.365679	35.14193
3.309573	3.458743	3.352924	35.31601
3.186544	3.441423	3.369567	35.04548
3.258637	3.429157	3.454415	35.36186
3.220817	3.345926	3.389278	34.9547
3.312666	3.195975	3.377888	34.80273
3.344795	3.230683	3.392143	34.98006
3.423941	3.381981	3.431106	35.56922
3.313161	3.402873	3.380616	35.26223
3.284992	3.326112	3.316451	34.89245
3.239418	3.310544	3.373659	34.88384
3.344135	3.394112	3.31981	35.17783
3.25274	3.385958	3.419494	35.17813
3.102119	3.390406	3.46821	34.965
3.208609	3.320181	3.446435	34.99669
3.317574	3.321394	3.433681	35.20974
3.315471	3.255955	3.354323	34.8885
3.289323	3.214845	3.433135	34.91376
3.303345	3.294909	3.446401	35.14853
3.240944	3.329347	3.396304	34.97782
3.364427	3.416554	3.367947	35.37655
3.375892	3.423698	3.334151	35.34334
3.331308	3.393101	3.349395	35.21227
3.216033	3.376522	3.384726	35.00119
3.283136	3.397482	3.401777	35.23106
3.21261	3.430235	3.315564	34.95992
3.316543	3.305287	3.337442	34.96181
3.177058	3.204332	3.41119	34.59728
3.279053	3.217204	3.451602	34.93685
3.23418	3.429898	3.424746	35.24512

3.458957	3.479298	3.419391	35.83299
3.343764	3.360955	3.424081	35.33253
3.323843	3.31452	3.399441	35.13354
3.192937	3.316273	3.31345	34.66306
3.33469	3.410556	3.418419	35.40878
3.151858	3.442434	3.383634	35.0026
3.214796	3.360887	3.416305	35.03335
3.12802	3.225021	3.45225	34.62507
3.274227	3.231019	3.412417	34.87082
3.404928	3.224078	3.472439	35.27272
3.339475	3.343702	3.388187	35.20693
3.236119	3.357113	3.444525	35.13344
3.314275	3.395662	3.398486	35.28798
3.346197	3.432324	3.432777	35.51295
3.463617	3.476737	3.209674	35.37896
3.186915	3.373692	3.417533	35.00307
3.117173	3.386699	3.377547	34.79155
3.25905	3.390473	3.413645	35.18901
3.253235	3.360618	3.364997	35.00462
3.311965	3.215452	3.385237	34.85986
3.200484	3.188696	3.363769	34.51061
3.28293	3.283654	3.397531	34.9724
3.212651	3.441962	3.397616	35.16509
3.452935	3.444994	3.366242	35.62857
3.251956	3.361763	3.460315	35.21277
3.299551	3.349228	3.319452	34.9814
3.217848	3.357383	3.452932	35.11246
3.326772	3.44331	3.298069	35.19991
3.208279	3.363313	3.418334	35.02884
3.227622	3.293561	3.400413	34.87942
3.158251	3.233446	3.483488	34.77792
3.277774	3.244768	3.369839	34.81553
3.259875	3.24315	3.445685	34.93871
3.407939	3.329684	3.386976	35.32335
3.246182	3.33366	3.476668	35.17445
3.381625	3.384138	3.409808	35.43482
3.312254	3.428618	3.3356	35.2181
3.378408	3.405704	3.358875	35.36356
3.180234	3.361224	3.357972	34.83094
3.27134	3.38616	3.387795	35.14993
3.138042	3.374703	3.353811	34.75905
3.231334	3.274151	3.352669	34.74068
3.327638	3.258988	3.323545	34.85443
3.220652	3.249688	3.450596	34.87797
3.313285	3.340939	3.403619	35.17736
3.231376	3.353204	3.415265	35.05053
3.348177	3.37477	3.325113	35.15597
3.357869	3.339119	3.404113	35.27196
3.266762	3.373557	3.383754	35.10352
3.205557	3.409208	3.419511	35.12583

3.291385	3.375377	3.381094	35.15552
3.194916	3.320855	3.449573	34.97509
3.248244	3.270108	3.399714	34.8717
3.200773	3.284867	3.419511	34.84345
3.241563	3.291674	3.48608	35.09311
3.207784	3.374366	3.393081	34.99671
3.354405	3.361224	3.414054	35.33447
3.287302	3.356102	3.368544	35.077
3.441758	3.418644	3.410149	35.64252
3.307057	3.422283	3.364929	35.25702
3.324544	3.377803	3.325045	35.11078
3.248986	3.345387	3.345575	34.91955
3.307181	3.342017	3.344893	35.03795
3.235046	3.313509	3.370948	34.87484
3.205103	3.285608	3.318906	34.63454
3.246182	3.260807	3.430117	34.91333
3.310027	3.400043	3.402732	35.29755
3.328421	3.43967	3.432606	35.48977
3.228076	3.362639	3.403482	35.03818
3.279672	3.307848	3.419562	35.06636
3.364056	3.356641	3.351816	35.20945
3.303222	3.370861	3.455097	35.33337
3.304871	3.426259	3.314251	35.15011
3.194545	3.245105	3.442616	34.79341
3.152064	3.203792	3.339982	34.38572
3.253193	3.227515	3.468824	34.94051
3.269526	3.297335	3.380207	34.93512
3.284126	3.351856	3.48417	35.31363
3.184564	3.387508	3.414787	35.02214
3.281445	3.396336	3.390097	35.19931
3.410207	3.435829	3.356266	35.49328
3.403979	3.436435	3.350895	35.46924
3.232984	3.354889	3.449931	35.13354
3.332339	3.362235	3.366429	35.18428
3.207124	3.285406	3.409962	34.83764
3.336464	3.293426	3.286491	34.86801
3.20432	3.25454	3.396082	34.73365
3.14229	3.32746	3.405187	34.77738
3.063845	3.303333	3.462105	34.67754
3.347599	3.473502	3.379474	35.4895
3.304418	3.408804	3.473786	35.45983
3.392225	3.375444	3.394326	35.40513
3.183286	3.354485	3.447066	35.01771
3.314728	3.401526	3.4109	35.32894
3.369335	3.441288	3.390591	35.49089
3.352755	3.345589	3.386635	35.23671
3.109543	3.193346	3.415725	34.43553
3.175037	3.201568	3.414344	34.59371
3.175367	3.246992	3.390318	34.64123
3.287467	3.36547	3.416987	35.20378

3.239418	3.374096	3.395553	35.0707
3.213476	3.321664	3.470273	35.06271
3.283713	3.364594	3.370266	35.09149
3.397999	3.442299	3.402596	35.58204
3.384059	3.457799	3.321243	35.40755
3.275753	3.423429	3.459872	35.3987
3.262597	3.345454	3.391836	35.05062
3.259792	3.277117	3.386738	34.8839
3.30017	3.288237	3.318617	34.84755
3.276001	3.29282	3.38771	34.95581
3.248079	3.293965	3.482601	35.10477
3.165799	3.396471	3.418829	35.00954
3.307676	3.405367	3.410098	35.32016
3.402329	3.356237	3.379968	35.35383
3.320833	3.347611	3.438267	35.28423
3.24878	3.391753	3.434499	35.21496
3.274145	3.392832	3.469693	35.34975
3.26536	3.340804	3.367316	34.99288
3.256328	3.236815	3.352379	34.71305
3.166293	3.224348	3.383481	34.55691
3.194545	3.27523	3.396014	34.75737
3.149012	3.272399	3.351714	34.55473
3.261071	3.440277	3.392024	35.25506
3.300335	3.404895	3.496157	35.49127
3.312625	3.349295	3.400464	35.1873
3.234551	3.402334	3.467869	35.27995
3.337371	3.454969	3.367913	35.40132
3.42922	3.388653	3.393081	35.5122
3.40567	3.360887	3.316298	35.23206
3.218673	3.261684	3.398503	34.78596
3.221766	3.302726	3.283439	34.63085
3.200649	3.293493	3.373318	34.76103
3.291302	3.37659	3.370129	35.13402
3.284043	3.389125	3.483829	35.3942
3.160354	3.393371	3.435454	35.02721
3.267216	3.394247	3.464987	35.3274
3.406	3.352935	3.390591	35.37786
3.330855	3.388653	3.397804	35.30742
3.226468	3.413522	3.443485	35.23342
3.370531	3.341141	3.394871	35.28386
3.21859	3.218686	3.453597	34.81223
3.374078	3.216934	3.30015	34.81286
3.205557	3.191999	3.422017	34.65631
3.275877	3.300637	3.364877	34.92271
3.133629	3.34943	3.447185	34.89833
3.334855	3.445736	3.392996	35.43048
3.23385	3.345724	3.430543	35.073
3.325947	3.39573	3.313416	35.12761
3.231293	3.375444	3.454535	35.18486
3.383852	3.444658	3.428582	35.61309

3.331803	3.370659	3.39598	35.26615
3.388018	3.250766	3.295222	34.90655
3.301737	3.234591	3.369703	34.84538
3.171366	3.25946	3.382321	34.64226
3.232737	3.322001	3.379866	34.90786
3.292457	3.408939	3.38609	35.24219
3.254967	3.425855	3.403056	35.2343
3.234304	3.416622	3.427764	35.22295
3.194009	3.361292	3.442837	35.0468
3.360467	3.399302	3.472831	35.55953
3.405464	3.484285	3.45728	35.80977
3.296045	3.375444	3.438864	35.2922
3.309656	3.264784	3.384367	34.96079
3.215084	3.209049	3.410337	34.68889
3.266639	3.166861	3.365287	34.61085
3.233479	3.271793	3.350605	34.7357
3.201474	3.353811	3.392501	34.93669
3.13932	3.384879	3.432265	34.95567
3.320626	3.403345	3.419698	35.36506
3.307346	3.371468	3.388681	35.19847
3.295385	3.437581	3.403891	35.35016
3.230468	3.435694	3.427678	35.25609
3.311058	3.385284	3.486626	35.45099
3.310975	3.311555	3.350077	34.99097
3.43619	3.258584	3.347195	35.14265
3.287302	3.231356	3.361041	34.78779
3.290147	3.328606	3.36058	35.00568
3.201763	3.343904	3.322709	34.76303
3.309326	3.378544	3.360086	35.15574
3.173222	3.383397	3.414804	34.98838
3.276001	3.359674	3.365082	35.05253
3.278352	3.41588	3.429878	35.32228
3.386286	3.475052	3.414037	35.65307
3.284538	3.404895	3.476309	35.41333
3.394658	3.337232	3.38273	35.30153
3.28825	3.200962	3.41049	34.83154
3.244656	3.17542	3.399441	34.65618
3.242016	3.18809	3.397207	34.67323
3.183533	3.282576	3.357102	34.66426
3.24082	3.338715	3.419715	35.04923
3.237191	3.374972	3.41344	35.10686
3.212569	3.339726	3.416305	34.9822
3.295921	3.371266	3.364383	35.11991
3.274887	3.462449	3.426655	35.4095
3.308171	3.434751	3.41049	35.38636
3.337454	3.377196	3.376404	35.24999
3.298767	3.227111	3.367299	34.81727
3.338897	3.261481	3.35908	34.96221
3.318853	3.302457	3.396798	35.09047
3.297901	3.390877	3.388323	35.21948

3.214713	3.370996	3.404488	35.02944
3.302727	3.335547	3.395621	35.125
3.159406	3.356507	3.419988	34.9107
3.351724	3.460225	3.344211	35.39237
3.210341	3.381847	3.494879	35.24127
3.275588	3.423159	3.461236	35.40073
3.21129	3.324562	3.429332	34.97474
3.336835	3.295852	3.409689	35.14354
3.308377	3.22765	3.456615	35.03478
3.290477	3.250698	3.340784	34.79274
3.167283	3.258584	3.452505	34.78489
3.237315	3.331301	3.361416	34.89786
3.254843	3.380431	3.35127	35.02145
3.271299	3.387575	3.345422	35.06027
3.20564	3.367357	3.414327	35.02315
3.262184	3.465954	3.409007	35.35079
3.344877	3.490081	3.475968	35.73082
3.424931	3.377803	3.305453	35.28746
3.403649	3.23048	3.376183	35.07342
3.214713	3.187079	3.405443	34.62933
3.234964	3.299087	3.373199	34.84804
3.30648	3.381173	3.358449	35.15169
3.466463	3.446342	3.321208	35.56262
3.15887	3.321799	3.427406	34.84984
3.250925	3.360752	3.380974	35.0348
3.215951	3.455643	3.483556	35.39016
3.338526	3.466291	3.402255	35.5037
3.252368	3.403008	3.443246	35.26655
3.274475	3.267143	3.416987	34.96035
3.172397	3.242274	3.392501	34.62919
3.426993	3.277656	3.371886	35.21824
3.296581	3.271523	3.401624	34.98467
3.247873	3.247127	3.39985	34.82093
3.239006	3.273208	3.388119	34.83292
3.241975	3.419115	3.395417	35.17444
3.274392	3.489542	3.377189	35.35949
3.325493	3.401863	3.386414	35.29967
3.180192	3.36392	3.480265	35.10418
3.209393	3.440614	3.425087	35.21509
3.230922	3.447218	3.446111	35.32259
3.457142	3.356304	3.417311	35.5555
3.342609	3.253799	3.393865	35.0296
3.324544	3.207836	3.427542	34.96323
3.25043	3.320451	3.358381	34.89618
3.33502	3.359741	3.35422	35.15799
3.348424	3.350643	3.393115	35.25246
3.226962	3.271456	3.372789	34.76922
3.162252	3.379218	3.446589	35.02476
3.311553	3.489744	3.412826	35.51913
3.351394	3.484352	3.488382	35.75969

3.239377	3.355226	3.367555	34.96812
3.327844	3.248946	3.452591	35.11512
3.215703	3.299963	3.373813	34.80918
3.338031	3.263368	3.409825	35.07542
3.382986	3.233109	3.307022	34.88274
3.298025	3.227852	3.381503	34.84833
3.195989	3.294707	3.410218	34.83418
3.308583	3.392697	3.35908	35.18287
3.30483	3.378746	3.395229	35.22321
3.268866	3.368637	3.391972	35.11533
3.175367	3.40348	3.433732	35.07838
3.212734	3.434885	3.472081	35.31264
3.241769	3.384071	3.34341	34.98362
3.392142	3.249688	3.351969	35.03731
3.255297	3.203051	3.383515	34.70505
3.400556	3.304074	3.314746	35.09324
3.279424	3.357046	3.365065	35.05423
3.354487	3.371872	3.318497	35.14897
3.346238	3.270512	3.392109	35.07025
3.255915	3.332514	3.311353	34.83171
3.183492	3.438323	3.466351	35.24368
3.293983	3.528428	3.386209	35.50709
3.287838	3.381914	3.470717	35.35805
3.30549	3.298009	3.411667	35.08404
3.288786	3.235535	3.477469	35.05479
3.246801	3.290528	3.417055	34.95112
3.308088	3.278465	3.35908	34.93198
3.287219	3.218821	3.381315	34.80454
3.265649	3.30057	3.398486	34.97369
3.250183	3.371131	3.341875	34.97037
3.198834	3.323618	3.382065	34.84207
3.28062	3.397549	3.376063	35.16947
3.275424	3.379083	3.398776	35.16739
3.203248	3.411163	3.414191	35.11342
3.362777	3.438457	3.415265	35.52432
3.227829	3.295111	3.474059	35.04431
3.3622	3.240522	3.366566	34.98371
3.317409	3.28325	3.418743	35.0933
3.378161	3.423496	3.394547	35.47994
3.333865	3.447353	3.400259	35.44774
3.359436	3.375916	3.393302	35.33222
3.240284	3.275836	3.336896	34.72944
3.308707	3.393977	3.358756	35.18523
3.21393	3.525934	3.458252	35.48412
3.158787	3.464	3.400754	35.10235
3.152436	3.296796	3.441456	34.81182
3.248822	3.216193	3.419818	34.79902
3.314151	3.283587	3.408512	35.06454
3.290106	3.293898	3.411104	35.04017
3.208197	3.239376	3.509543	34.95709

3.304913	3.253529	3.32438	34.79462
3.347393	3.323484	3.400106	35.2061
3.317904	3.405974	3.321362	35.1498
3.218219	3.423361	3.407933	35.15915
3.18774	3.403615	3.377427	34.9826
3.245605	3.416487	3.352174	35.08207
3.33069	3.464741	3.38875	35.45364
3.353992	3.338445	3.417857	35.29207
3.167943	3.268558	3.349923	34.58382
3.268123	3.209453	3.388937	34.75896
3.334897	3.373355	3.431651	35.35682
3.398906	3.504705	3.402067	35.71934
3.270887	3.393775	3.419579	35.23509
3.353621	3.294841	3.337015	35.01911
3.202505	3.339389	3.417925	34.963
3.340464	3.497225	3.344211	35.44866
3.257936	3.482331	3.44843	35.46352
3.17248	3.290326	3.42449	34.80441
3.135113	3.20049	3.495134	34.68072
3.305201	3.27988	3.396986	35.01166
3.333824	3.335682	3.387386	35.17529
3.325081	3.294909	3.400959	35.09668
3.23649	3.236613	3.467784	34.9216
3.248409	3.336423	3.375449	34.96401
3.387771	3.421946	3.284923	35.25783
3.451657	3.448432	3.34653	35.59019
3.25608	3.366076	3.356761	35.00477
3.184894	3.338984	3.36544	34.80883
3.241769	3.41588	3.419101	35.21871
3.383522	3.467639	3.342011	35.47331
3.327266	3.361089	3.4242	35.29701
3.113708	3.236411	3.443809	34.60023
3.255173	3.231828	3.458098	34.93081
3.330195	3.369918	3.403687	35.27787
3.417837	3.405434	3.288828	35.29601
3.260246	3.389462	3.425564	35.21548
3.270887	3.304142	3.408717	35.01533
3.222384	3.420328	3.447578	35.24832
3.404557	3.56017	3.346308	35.73106
3.30714	3.456923	3.436494	35.48946
3.2209	3.299626	3.430765	34.94435
3.084384	3.205342	3.409194	34.39246
3.36554	3.257438	3.47174	35.25801
3.354487	3.350576	3.350691	35.1728
3.334608	3.251103	3.42391	35.07191
3.245728	3.227717	3.388937	34.74993
3.365664	3.289989	3.441064	35.26238
3.318729	3.377668	3.315888	35.07774
3.412929	3.368705	3.358824	35.35803
3.1632	3.359068	3.445088	34.97948

3.181141	3.431651	3.405614	35.09112
3.185224	3.481657	3.419579	35.23995
3.362035	3.379757	3.400328	35.36167
3.327266	3.271995	3.365747	34.97435
3.179615	3.265255	3.44091	34.80109
3.196236	3.355226	3.487018	35.13502
3.437716	3.40193	3.35185	35.46964
3.397298	3.38899	3.368595	35.38958
3.26833	3.279813	3.326	34.77564
3.200567	3.31971	3.438387	34.96048
3.213806	3.532606	3.3607	35.28511
3.361045	3.524115	3.365815	35.59971
3.314151	3.350441	3.347621	35.07758
3.152477	3.216193	3.506815	34.77858
3.199989	3.257573	3.413423	34.76874
3.315553	3.413993	3.464118	35.47439
3.336959	3.394719	3.39129	35.31978
3.289364	3.30522	3.376473	34.98758
3.176975	3.252046	3.49036	34.87457
3.311841	3.330762	3.410098	35.16612
3.2972	3.340939	3.390983	35.11456
3.347063	3.414735	3.358654	35.31428
3.225808	3.375512	3.422785	35.10358
3.225436	3.332919	3.396627	34.95243
3.237108	3.309196	3.389943	34.91146
3.343681	3.341545	3.284121	34.98384
3.247419	3.225628	3.49036	34.97085
3.218673	3.445938	3.405051	35.20321
3.284167	3.45443	3.49198	35.55511
3.447903	3.411702	3.338891	35.48495
3.486672	3.320855	3.348832	35.39281
3.33964	3.373962	3.442087	35.39134
3.201103	3.484218	3.433953	35.31171
3.262927	3.495338	3.376677	35.34597
3.297901	3.290326	3.40425	35.03442
3.268618	3.234861	3.389739	34.81736
3.191122	3.21936	3.394053	34.62342
3.132474	3.290393	3.483744	34.84665
3.301778	3.426731	3.453085	35.44799
3.354487	3.289517	3.411121	35.17142
3.302479	3.237422	3.429043	34.98296
3.296829	3.38926	3.416817	35.27591
3.296169	3.415678	3.426058	35.35245
3.275465	3.399504	3.411036	35.23895
3.370283	3.350239	3.331644	35.16495
3.218549	3.334941	3.474911	35.11298
3.247131	3.39418	3.359131	35.05184
3.234758	3.273141	3.380633	34.80711
3.324132	3.257842	3.339283	34.87867
3.231417	3.300974	3.415231	34.93633

3.260493	3.389193	3.410371	35.18221
3.285157	3.374905	3.419221	35.22425
3.434211	3.407389	3.393848	35.56576
3.362035	3.334199	3.303185	35.0496
3.433798	3.377601	3.405187	35.52451
3.234634	3.468111	3.380224	35.23231
3.261153	3.420194	3.437858	35.31155
3.174047	3.335817	3.395639	34.84422
3.244821	3.248879	3.411718	34.84404
3.192029	3.225561	3.437654	34.73431
3.2395	3.347341	3.432061	35.09221
3.243377	3.391619	3.499772	35.34559
3.346857	3.265862	3.399236	35.07702
3.385213	3.255888	3.438319	35.22455
3.290684	3.389193	3.42304	35.27593
3.295633	3.350037	3.403192	35.15773
3.292663	3.353474	3.380429	35.10897
3.322029	3.42572	3.382406	35.3355
3.35923	3.408467	3.387249	35.38972
3.214342	3.360281	3.379184	34.94985
3.205433	3.257977	3.393985	34.73902
3.271217	3.228526	3.449641	34.94018
3.227127	3.344645	3.411514	35.01432
3.364839	3.373625	3.419698	35.39675
3.265525	3.411702	3.418607	35.26045
3.318564	3.353609	3.360171	35.1216
3.301448	3.343432	3.33485	35.00655
3.408887	3.415746	3.368407	35.47302
3.264453	3.483544	3.462753	35.51175
3.305614	3.426394	3.333758	35.19469
3.133134	3.186337	3.396627	34.43003
3.282724	3.273006	3.318207	34.77519
3.190421	3.280486	3.426604	34.82675
3.280167	3.442636	3.419357	35.36175
3.237315	3.372748	3.449266	35.18061
3.326895	3.208038	3.404932	34.91937
3.362901	3.284126	3.376063	35.10137
3.461802	3.366885	3.415913	35.58577
3.271052	3.357383	3.420517	35.15792
3.262226	3.455104	3.440365	35.39572
3.263009	3.37996	3.348082	35.0313
3.330937	3.243487	3.250768	34.6686
3.372057	3.253462	3.378843	35.06041
3.128844	3.293224	3.460162	34.79333
3.22222	3.364392	3.448157	35.12691
3.310192	3.454497	3.379661	35.36654
3.379646	3.393304	3.353402	35.32718
3.341207	3.355428	3.387846	35.23562
3.294066	3.338243	3.402647	35.12731
3.271299	3.385014	3.347826	35.05992

3.393462	3.445466	3.348303	35.46032
3.292251	3.448229	3.384078	35.32326
3.244408	3.244566	3.365065	34.73168
3.173882	3.202782	3.386277	34.53246
3.303345	3.31944	3.476531	35.26806
3.286848	3.508951	3.399185	35.47727
3.305407	3.432392	3.413833	35.38247
3.176233	3.255214	3.526373	34.95863
3.269814	3.316812	3.423927	35.07395
3.302974	3.336895	3.414668	35.17014
3.421549	3.42417	3.400004	35.58823
3.22721	3.332447	3.429401	35.02694
3.282105	3.344308	3.337442	34.97183
3.263834	3.252585	3.421693	34.91554
3.331968	3.269838	3.278938	34.79008
3.171573	3.290326	3.351765	34.64338
3.213352	3.299896	3.407251	34.87702
3.117049	3.345791	3.428872	34.81406
3.336093	3.443579	3.40367	35.45181
3.404474	3.371872	3.399731	35.43593
3.417672	3.393977	3.392211	35.49668
3.307882	3.354754	3.504479	35.41633
3.321987	3.448836	3.369498	35.35773
3.357663	3.408198	3.447663	35.51781
3.313904	3.391416	3.292783	35.04673
3.236242	3.190381	3.403465	34.6793
3.118616	3.225156	3.389892	34.46844
3.289075	3.317688	3.408512	35.08428
3.208403	3.444792	3.448243	35.2727
3.29221	3.423159	3.386141	35.27286
3.224405	3.184653	3.447049	34.7362
3.216817	3.260673	3.385527	34.75131
3.310687	3.364324	3.417123	35.25236
3.467659	3.411567	3.351373	35.55516
3.247378	3.426798	3.45271	35.32835
3.333412	3.413859	3.404096	35.38189
3.285611	3.232233	3.385885	34.84034
3.384719	3.260403	3.325233	34.98604
3.238841	3.270108	3.344688	34.7308
3.248492	3.389529	3.430679	35.20111
3.242264	3.45753	3.427286	35.32878
3.322152	3.393843	3.374478	35.24872
3.321864	3.370053	3.453068	35.36793
3.40002	3.376051	3.38389	35.40068
3.301572	3.343837	3.404625	35.16029
3.370325	3.439401	3.415418	35.54323
3.309903	3.384879	3.391188	35.23888
3.350239	3.201299	3.347195	34.82942
3.239377	3.174678	3.307363	34.44166
3.170005	3.332043	3.362456	34.75456

3.120719	3.432931	3.471194	35.1052
3.228447	3.456923	3.424967	35.29216
3.233768	3.35125	3.437449	35.1
3.319224	3.207364	3.370965	34.82684
3.322812	3.294774	3.397668	35.08423
3.279919	3.46218	3.468892	35.51228
3.363148	3.484757	3.373864	35.53584
3.367396	3.376792	3.397958	35.36172
3.270969	3.245374	3.364571	34.79045
3.269567	3.125211	3.319111	34.42519
3.267092	3.28635	3.417669	34.9877
3.175078	3.448027	3.416987	35.13855
3.287755	3.496955	3.439154	35.54043
3.219126	3.463865	3.421966	35.2804
3.280785	3.302794	3.392757	34.99912
3.339392	3.319575	3.417192	35.21742
3.400267	3.474311	3.428088	35.71275
3.237768	3.453486	3.3895	35.22747
3.315306	3.361628	3.357238	35.1256
3.233231	3.168748	3.481783	34.79668
3.279012	3.164704	3.318565	34.53102
3.175862	3.307377	3.416782	34.83223
3.295921	3.450858	3.389841	35.34964
3.184688	3.394988	3.382662	34.96851
3.225024	3.37659	3.414174	35.0854
3.200443	3.335278	3.444866	35.00842
3.378367	3.394584	3.400686	35.43059
3.321369	3.431785	3.446657	35.48783
3.304541	3.300098	3.373028	35.00203
3.271547	3.301446	3.442633	35.08504
3.389956	3.381173	3.362166	35.34237
3.304129	3.266671	3.355533	34.88977
3.222591	3.269569	3.348696	34.70285
3.218095	3.291876	3.431651	34.92321
3.195081	3.354822	3.321481	34.76961
3.29155	3.370659	3.424268	35.23998
3.27134	3.43064	3.378706	35.22732
3.212156	3.410893	3.454091	35.21957
3.271588	3.418644	3.441183	35.33826
3.464772	3.501268	3.359557	35.7629
3.296086	3.370727	3.394462	35.18487
3.325369	3.392899	3.369106	35.24195
3.279548	3.222865	3.449044	34.94472
3.29456	3.177576	3.317969	34.59187
3.242346	3.266671	3.390216	34.83051
3.267422	3.378005	3.356249	35.05454
3.139073	3.363044	3.41622	34.87229
3.215373	3.372479	3.347894	34.91036
3.26503	3.301581	3.433203	35.05047
3.332917	3.376253	3.385817	35.25859

3.265814	3.33211	3.414174	35.07733
3.285239	3.381644	3.432265	35.2677
3.262721	3.408602	3.441661	35.29795
3.377955	3.3466	3.372159	35.26237
3.320585	3.232772	3.367674	34.87818
3.280579	3.1643	3.318565	34.53356
3.207331	3.290932	3.444474	34.92565
3.214631	3.389462	3.352157	34.9552
3.360921	3.46528	3.431157	35.61367
3.322936	3.404558	3.37666	35.27864
3.221807	3.373557	3.480658	35.21712
3.294643	3.397684	3.416424	35.28869
3.330731	3.43347	3.377342	35.3604
3.441717	3.421811	3.364042	35.54853
3.289158	3.342893	3.36972	35.05474
3.214878	3.230683	3.427678	34.77367
3.320131	3.246318	3.413952	35.00801
3.215786	3.384071	3.410132	35.07272
3.38476	3.42754	3.357409	35.422
3.121544	3.374838	3.475849	34.99015
3.195164	3.256157	3.355192	34.62775
3.224859	3.245711	3.408836	34.78715
3.307593	3.326921	3.370385	35.06159
3.214012	3.434683	3.465482	35.30056
3.408434	3.421474	3.368833	35.48549
3.255957	3.254136	3.447339	34.95778
3.428272	3.189438	3.273839	34.81371
3.37581	3.152506	3.416493	34.93018
3.293983	3.403547	3.324346	35.09871
3.228653	3.475996	3.431992	35.34969
3.286188	3.490755	3.369993	35.3722
3.305078	3.454901	3.366975	35.3285
3.366035	3.316812	3.337373	35.09509
3.202918	3.361157	3.412025	34.99861
3.2322	3.446882	3.432248	35.29433
3.290725	3.401391	3.391819	35.23442
3.297489	3.200221	3.372295	34.76659
3.294643	3.146171	3.305657	34.49645
3.254967	3.193077	3.395212	34.7081
3.306604	3.416015	3.38048	35.27633
3.243254	3.474109	3.43322	35.38017
3.296169	3.403547	3.358125	35.17736
3.241521	3.313038	3.382748	34.91377
3.270557	3.309938	3.414191	35.03925
3.14229	3.347341	3.419084	34.85125
3.356467	3.366885	3.369379	35.25366
3.318317	3.391349	3.369839	35.22474
3.320956	3.277454	3.33739	34.91048
3.317863	3.244633	3.422904	35.01894
3.328215	3.182698	3.315172	34.67055

3.235871	3.195975	3.343648	34.55991
3.329287	3.389732	3.395894	35.30217
3.205557	3.454295	3.401794	35.18568
3.305201	3.461708	3.405733	35.42841
3.289364	3.394719	3.315974	35.051
3.279053	3.413656	3.439427	35.33983
3.18143	3.482668	3.357238	35.09753
3.367438	3.40503	3.481919	35.60718
3.185513	3.247127	3.39644	34.6771
3.360797	3.127368	3.38198	34.7669
3.330648	3.193953	3.388613	34.86109
3.34995	3.31607	3.334321	35.05162
3.275877	3.466695	3.401709	35.36639
3.242965	3.384138	3.309102	34.91136
3.137341	3.281902	3.404829	34.66615
3.333783	3.343971	3.303628	35.01016
3.214466	3.314588	3.474195	35.05798
3.203495	3.384071	3.38094	34.982
3.26734	3.388249	3.496157	35.38272
3.374284	3.376051	3.332769	35.23261
3.319925	3.203051	3.358807	34.79235
3.357292	3.168882	3.339897	34.75799
3.193803	3.207971	3.455779	34.73936
3.190668	3.398695	3.384623	34.99399
3.321864	3.465819	3.401487	35.46456
3.378285	3.449577	3.322999	35.38078
3.324338	3.369514	3.369021	35.18837
3.134206	3.389327	3.483949	35.06723
3.288126	3.480309	3.364144	35.3408
3.306975	3.41797	3.463777	35.46357
3.318935	3.341545	3.355994	35.0869
3.16984	3.145227	3.489951	34.62448
3.235871	3.238298	3.296722	34.54985
3.292581	3.356844	3.395366	35.14882
3.440356	3.513399	3.390983	35.80476
3.201309	3.421339	3.460366	35.23241
3.211661	3.303872	3.39291	34.85065
3.234551	3.283047	3.390898	34.85077
3.359766	3.365537	3.390233	35.30353
3.213476	3.36082	3.427695	35.05523
3.199123	3.391888	3.486762	35.22095
3.155199	3.374838	3.435965	34.97652
3.39837	3.232637	3.369993	35.05306
3.384306	3.296594	3.353402	35.12588
3.328834	3.289922	3.466846	35.23807
3.2063	3.354552	3.375245	34.91113
3.262597	3.470671	3.404505	35.35216
3.339062	3.449645	3.292562	35.22859
3.461596	3.365874	3.258595	35.23908
3.16584	3.401256	3.387914	34.95249

3.106243	3.452206	3.401828	34.964
3.299303	3.470537	3.426314	35.47983
3.306892	3.361359	3.29795	34.97696
3.343063	3.180676	3.437023	34.96506
3.187534	3.165648	3.366105	34.437
3.207619	3.305085	3.474212	35.02226
3.341578	3.465752	3.306135	35.299
3.445058	3.459484	3.432044	35.78693
3.256823	3.404963	3.389295	35.16258
3.203825	3.300098	3.382202	34.80185
3.254101	3.340467	3.359694	34.95085
3.317451	3.375916	3.368748	35.18671
3.280208	3.400649	3.423859	35.27987
3.261607	3.384947	3.444287	35.24953
3.153962	3.245037	3.39835	34.60771
3.386533	3.225426	3.356471	34.98183
3.25274	3.240792	3.387113	34.78986
3.376965	3.361831	3.378075	35.30645
3.225065	3.36365	3.484562	35.21112
3.388101	3.415813	3.326341	35.33572
3.332381	3.369042	3.406193	35.28621
3.344218	3.425316	3.260846	35.11731
3.17182	3.380701	3.432998	35.01921
3.212486	3.447892	3.334372	35.03939
3.165757	3.353811	3.462753	35.01221
3.309656	3.247666	3.336777	34.81928
3.355807	3.171983	3.402647	34.89875
3.299056	3.347139	3.413696	35.18184
3.273691	3.455643	3.424609	35.38752
3.358859	3.445331	3.45653	35.62103
3.28759	3.328875	3.350878	34.97946
3.383069	3.272062	3.344228	35.04947
3.176027	3.319912	3.360018	34.73587
3.159282	3.383262	3.408052	34.94283
3.237397	3.379016	3.390455	35.06589
3.22787	3.335278	3.436477	35.05005
3.277857	3.239646	3.377547	34.82136
3.284332	3.16895	3.439427	34.81624
3.285982	3.256696	3.427627	34.98594
3.334732	3.336625	3.442974	35.3009
3.421095	3.441086	3.331405	35.47421
3.300252	3.435559	3.346803	35.23154
3.395194	3.375916	3.359302	35.33606
3.302273	3.351924	3.338891	35.03576
3.310893	3.333391	3.425513	35.20351
3.272413	3.420396	3.360836	35.16818
3.347847	3.384273	3.422631	35.38929
3.106161	3.221113	3.426655	34.51275
3.249605	3.142532	3.407967	34.61373
3.258431	3.353474	3.402067	35.08143

3.377501	3.532135	3.459087	35.85721
3.276991	3.455508	3.425257	35.39586
3.374573	3.402671	3.411241	35.46306
3.23682	3.258179	3.423603	34.87287
3.39074	3.33056	3.354783	35.21725
3.177759	3.35253	3.400754	34.90008
3.30417	3.390203	3.3718	35.19558
3.058153	3.264514	3.401487	34.44764
3.247419	3.236411	3.341875	34.66972
3.278847	3.209049	3.384828	34.77254
3.346486	3.2941	3.333162	34.99346
3.258761	3.381105	3.455813	35.26011
3.286436	3.442501	3.346581	35.21602
3.363602	3.411028	3.377751	35.38411
3.4604	3.410422	3.271009	35.36103
3.320833	3.364729	3.388187	35.21215
3.19339	3.397886	3.400942	35.03385
3.252781	3.41743	3.351833	35.09908
3.25076	3.41278	3.32409	35.02382
3.24346	3.240387	3.417243	34.83457
3.208609	3.167872	3.380377	34.51916
3.166706	3.223808	3.446299	34.69401
3.252781	3.478354	3.491366	35.53745
3.394988	3.504638	3.367964	35.63605
3.267134	3.36729	3.405852	35.13895
3.364468	3.350306	3.350537	35.1937
3.272866	3.343365	3.440109	35.17408
3.383275	3.410354	3.334918	35.33198
3.244079	3.337569	3.408444	35.0292
3.267752	3.33676	3.359881	34.97301
3.16617	3.252585	3.422955	34.70472
3.316914	3.254809	3.346939	34.873
3.245811	3.267345	3.415845	34.89561
3.304253	3.298413	3.45109	35.16843
3.246883	3.350239	3.464731	35.18614
3.346321	3.376522	3.414736	35.35174
3.388678	3.401458	3.38759	35.43953
3.431159	3.385553	3.380207	35.4815
3.317657	3.434077	3.35231	35.2784
3.306892	3.389529	3.363786	35.18254
3.164644	3.394988	3.360836	34.87695
3.221023	3.322675	3.353675	34.82644
3.273155	3.237826	3.367452	34.78503
3.198505	3.220641	3.445975	34.75591
3.255833	3.398425	3.448567	35.27573
3.293406	3.461439	3.426877	35.44827
3.360137	3.34943	3.367947	35.22038
3.389214	3.327258	3.384487	35.27165
3.300335	3.323012	3.420312	35.14635
3.233231	3.394112	3.422239	35.1593

3.300087	3.398291	3.424677	35.31997
3.18741	3.290393	3.366907	34.71128
3.28726	3.212688	3.467664	34.98005
3.180399	3.245105	3.381793	34.62946
3.203413	3.244566	3.424012	34.77094
3.231829	3.381442	3.400464	35.08091
3.269443	3.372681	3.466846	35.28917
3.252863	3.396606	3.34283	35.03403
3.363231	3.381779	3.441746	35.45928
3.32108	3.391282	3.432743	35.36819
3.362901	3.412982	3.320748	35.26219
3.375769	3.305624	3.352345	35.12465
3.344671	3.359068	3.331849	35.1287
3.219374	3.302052	3.406262	34.89274
3.220858	3.297739	3.359523	34.78434
3.17413	3.243083	3.410917	34.67502
3.299056	3.399639	3.338874	35.13303
3.250512	3.46656	3.49863	35.52258
3.321699	3.378814	3.414532	35.30246
3.25542	3.374905	3.439768	35.20416
3.337536	3.367492	3.377751	35.2319
3.305902	3.368637	3.45624	35.33687
3.299056	3.359876	3.32068	35.00629
3.229932	3.256562	3.47735	34.9718
3.168974	3.216462	3.345967	34.4635
3.325782	3.253125	3.344552	34.88349
3.274929	3.30522	3.350264	34.8987
3.221807	3.333795	3.467238	35.10082
3.237603	3.404895	3.424541	35.19748
3.330195	3.381375	3.424967	35.34946
3.410207	3.415072	3.432129	35.61378
3.40996	3.436301	3.355465	35.49201
3.254843	3.399369	3.442411	35.26217
3.28293	3.383262	3.324141	35.02973
3.259957	3.303939	3.413781	35.00206
3.39074	3.284867	3.275868	34.94476
3.21859	3.228391	3.412622	34.74385
3.184193	3.258179	3.398213	34.70226
3.137299	3.387642	3.442019	34.97862
3.292952	3.510434	3.378826	35.44934
3.232695	3.426798	3.393047	35.16577
3.348878	3.384138	3.450067	35.45125
3.19834	3.371333	3.424063	35.03717
3.373872	3.383868	3.437295	35.47738
3.375934	3.430909	3.320254	35.32881
3.334154	3.288372	3.396951	35.09347
3.213476	3.216597	3.365355	34.60351
3.146084	3.166119	3.388596	34.39657
3.193555	3.254338	3.318054	34.53904
3.277197	3.319979	3.340102	34.91371

3.188441	3.436166	3.440092	35.19236
3.239336	3.377129	3.38921	35.06328
3.321699	3.413454	3.459548	35.47665
3.418002	3.498505	3.425359	35.79848
3.344877	3.449038	3.397582	35.46965
3.30681	3.431988	3.357631	35.26174
3.224199	3.293224	3.433033	34.94253
3.335226	3.268288	3.275681	34.78669
3.325246	3.222056	3.407762	34.9526
3.231788	3.293898	3.334099	34.74425
3.202794	3.313172	3.427764	34.92782
3.191947	3.439603	3.40367	35.12789
3.311676	3.419452	3.389227	35.31407
3.311718	3.334604	3.375262	35.09807
3.328916	3.344713	3.446793	35.31422
3.218673	3.403076	3.440382	35.18674
3.28693	3.465954	3.420329	35.42966
3.317203	3.35772	3.385442	35.18288
3.370407	3.218821	3.347007	34.91143
3.148889	3.125683	3.439154	34.42484
3.238511	3.226976	3.35879	34.66659
3.223251	3.292752	3.398793	34.86454
3.254472	3.38097	3.319913	34.95324
3.172356	3.349093	3.433408	34.95215
3.197308	3.390001	3.384743	34.98976
3.229066	3.374568	3.443502	35.15395
3.457761	3.462584	3.357153	35.65772
3.317987	3.472356	3.388664	35.44233
3.359973	3.384745	3.336418	35.2283
3.238634	3.318564	3.431242	35.02559
3.253441	3.2879	3.366378	34.84907
3.343393	3.299155	3.321294	34.9718
3.285157	3.293359	3.343785	34.88096
3.188771	3.339186	3.398094	34.88916
3.194133	3.409748	3.415214	35.09263
3.299427	3.365066	3.432453	35.26288
3.286023	3.376051	3.382117	35.14751
3.323555	3.389125	3.422375	35.34622
3.222426	3.430774	3.474366	35.32984
3.270845	3.455171	3.378843	35.28018
3.305737	3.270984	3.366583	34.92689
3.286312	3.249553	3.377291	34.86096
3.222055	3.189707	3.408103	34.65695
3.177718	3.323079	3.38996	34.81198
3.233974	3.34195	3.466232	35.14306
3.344506	3.427944	3.315905	35.24409
3.255008	3.340871	3.398435	35.03844
3.332546	3.421339	3.414242	35.41854
3.218343	3.465482	3.48047	35.41016
3.327803	3.432729	3.35688	35.30763

3.355023	3.374096	3.385408	35.30132
3.478465	3.267075	3.372363	35.30871
3.237438	3.22145	3.437091	34.82339
3.322441	3.30522	3.370948	35.04783
3.252492	3.306837	3.378638	34.91522
3.313574	3.365672	3.365901	35.1496
3.196566	3.389866	3.403261	35.02833
3.118286	3.385418	3.459889	34.97126
3.205887	3.376792	3.401436	35.01613
3.29786	3.410624	3.421267	35.33462
3.289859	3.402402	3.439836	35.33975
3.337825	3.374972	3.436426	35.3772
3.223828	3.261481	3.437091	34.88117
3.278682	3.191122	3.386192	34.73596
3.317739	3.240657	3.334236	34.81608
3.293818	3.29255	3.342011	34.89425
3.166046	3.361898	3.426178	34.95054
3.264948	3.463056	3.411599	35.35616
3.318564	3.402671	3.393797	35.30243
3.296705	3.375983	3.340477	35.07966
3.322029	3.432864	3.429196	35.45344
3.195411	3.396741	3.454483	35.15286
3.267092	3.400515	3.399714	35.19809
3.308748	3.339052	3.315428	34.97046
3.412434	3.23257	3.380821	35.10735
3.2355	3.283991	3.345353	34.75531
3.253853	3.336423	3.388255	35.00392
3.27596	3.384812	3.391665	35.16554
3.35956	3.355563	3.357119	35.20885
3.230386	3.344308	3.406006	35.00867
3.226426	3.394045	3.430611	35.16258
3.178996	3.417498	3.436835	35.12376
3.275424	3.416487	3.427764	35.31258
3.318317	3.409613	3.478969	35.50333
3.315718	3.334671	3.407558	35.17759
3.251337	3.188898	3.4899	34.89809
3.313367	3.20514	3.353947	34.77195
3.232365	3.188359	3.432453	34.7298
3.371727	3.295313	3.320629	35.02391
3.179244	3.331099	3.399185	34.85303
3.195906	3.48415	3.378587	35.17912
3.238923	3.395999	3.422716	35.17692
3.264576	3.431179	3.303833	35.04997
3.301448	3.426596	3.45196	35.44451
3.271794	3.451464	3.400055	35.32054
3.227169	3.348891	3.419016	35.0401
3.319636	3.219091	3.327688	34.75874
3.420642	3.246655	3.360649	35.11198
3.24911	3.297672	3.409536	34.95535
3.272207	3.3621	3.366242	35.05207

3.29221	3.355967	3.416083	35.1914
3.348301	3.358798	3.338806	35.15126
3.289694	3.362302	3.416919	35.20158
3.230179	3.395325	3.386533	35.0772
3.182255	3.442097	3.446111	35.20496
3.275341	3.416015	3.41813	35.2903
3.278269	3.319642	3.465482	35.1895
3.323926	3.293898	3.379474	35.04496
3.234758	3.197525	3.470188	34.83759
3.35692	3.205477	3.395076	34.95787
3.22952	3.242476	3.439001	34.85624
3.282394	3.303131	3.328779	34.86347
3.218714	3.358259	3.423109	35.05105
3.22024	3.37504	3.358517	34.94983
3.179904	3.344039	3.394036	34.8715
3.262597	3.406378	3.388562	35.1767
3.316791	3.459754	3.44357	35.53223
3.289982	3.448297	3.432896	35.42521
3.242429	3.335615	3.403209	35.00987
3.27134	3.209858	3.383839	34.75573
3.398618	3.240455	3.334389	34.99284
3.340877	3.348621	3.338737	35.11262
3.262803	3.387845	3.366497	35.08837
3.263422	3.326179	3.38771	35.00125
3.262597	3.309196	3.410917	35.01306
3.213352	3.33676	3.347417	34.82678
3.314646	3.446679	3.339829	35.27208
3.185307	3.421878	3.478151	35.23749
3.197102	3.339389	3.426877	34.97076
3.257029	3.300637	3.440382	35.0466
3.330896	3.290461	3.392893	35.08203
3.224199	3.244026	3.399032	34.76059
3.421095	3.256966	3.439495	35.30795
3.22123	3.267884	3.469165	34.95963
3.375191	3.353137	3.323954	35.1652
3.270186	3.356372	3.395025	35.09807
3.348136	3.365672	3.377069	35.24961
3.083477	3.407658	3.384726	34.7794
3.217806	3.403143	3.382253	35.05788
3.273815	3.466763	3.321686	35.18703
3.392472	3.354889	3.370112	35.30777
3.216363	3.243892	3.391955	34.72768
3.296623	3.238231	3.355857	34.81187
3.327431	3.373085	3.406006	35.28382
3.370696	3.431179	3.39557	35.48265
3.30648	3.421878	3.371476	35.26919
3.232902	3.299087	3.338328	34.76728
3.21793	3.319507	3.408939	34.93361
3.312254	3.469795	3.401572	35.45242
3.214218	3.484218	3.42304	35.31652

3.249564	3.344915	3.361024	34.95356
3.183616	3.226504	3.473786	34.79699
3.309284	3.224011	3.431515	34.97392
3.417425	3.301244	3.392365	35.29368
3.25575	3.261481	3.342233	34.74354
3.308583	3.253529	3.442138	35.06017
3.25641	3.386699	3.463146	35.28323
3.342362	3.422013	3.356062	35.31425
3.341784	3.44331	3.337459	35.31887
3.276042	3.377264	3.397821	35.16268
3.049781	3.334536	3.387914	34.55278
3.26103	3.4208	3.470239	35.38342
3.245605	3.424035	3.306288	34.99823
3.31279	3.292954	3.392382	35.04677
3.138289	3.195031	3.38036	34.42474
3.282311	3.239511	3.404164	34.88902
3.273279	3.379353	3.389176	35.1423
3.420229	3.434077	3.306118	35.40169
3.261195	3.305153	3.440109	35.06499
3.350734	3.283115	3.302571	34.91183
3.149425	3.424507	3.491332	35.19359
3.303098	3.541165	3.334594	35.442
3.230221	3.468447	3.485398	35.4534
3.227994	3.341747	3.363241	34.90431
3.246099	3.255146	3.476105	35.00134
3.291508	3.266536	3.421762	35.00671
3.303758	3.253125	3.383549	34.92061
3.330277	3.237287	3.374205	34.92353
3.279053	3.286619	3.368953	34.90791
3.247213	3.33676	3.445565	35.11547
3.327638	3.404963	3.31524	35.15549
3.276537	3.407321	3.338669	35.10014
3.210053	3.389462	3.457638	35.17586
3.167283	3.420463	3.399253	35.02244
3.259669	3.427876	3.46705	35.38895
3.284291	3.332919	3.445344	35.18767
3.263752	3.270984	3.37238	34.84775
3.199577	3.17387	3.375415	34.50167
3.288869	3.312364	3.420312	35.09799
3.306645	3.411904	3.468875	35.46074
3.395648	3.422889	3.289015	35.28607
3.307923	3.291067	3.391273	35.02958
3.323761	3.316812	3.321379	34.96767
3.300005	3.468987	3.394189	35.40772
3.31114	3.49089	3.344211	35.37068
3.127731	3.412174	3.437926	35.00239
3.311553	3.333121	3.403244	35.15566
3.222426	3.279139	3.459667	34.96609
3.327473	3.286484	3.451295	35.19357
3.279465	3.232165	3.45109	34.96935

3.313945	3.303333	3.393388	35.0742
3.287755	3.326247	3.385339	35.04943
3.320626	3.389866	3.391256	35.27338
3.248615	3.3652	3.402971	35.08758
3.237974	3.370053	3.310091	34.87181
3.228447	3.395797	3.399594	35.103
3.282229	3.466224	3.444406	35.47262
3.300417	3.415341	3.406313	35.31782
3.204609	3.24962	3.370675	34.66796
3.286106	3.214643	3.358193	34.7424
3.239748	3.320249	3.3607	34.87745
3.356013	3.423833	3.423006	35.49448
3.326565	3.377331	3.357153	35.18438
3.301984	3.319844	3.412963	35.12696
3.226509	3.322608	3.386635	34.91037
3.412475	3.409882	3.367265	35.46555
3.206258	3.456923	3.428497	35.25136
3.224323	3.402941	3.402169	35.11524
3.135196	3.275432	3.479976	34.81164
3.243419	3.240387	3.349446	34.68622
3.287755	3.326786	3.418453	35.12303
3.311841	3.312027	3.43854	35.18735
3.172769	3.26876	3.42304	34.75471
3.337289	3.336356	3.399475	35.21077
3.306686	3.348689	3.381383	35.13126
3.398865	3.381577	3.383225	35.40879
3.266102	3.39364	3.364741	35.10442
3.214878	3.409006	3.391631	35.0848
3.272	3.389058	3.357852	35.09223
3.392142	3.425855	3.348508	35.41499
3.284621	3.298683	3.443911	35.11039
3.177635	3.233715	3.384436	34.60429
3.245563	3.305085	3.441337	35.03335
3.351641	3.412646	3.427832	35.47101
3.4166	3.459484	3.304259	35.44525
3.287302	3.32719	3.428241	35.14432
3.23418	3.28635	3.401214	34.87974
3.278352	3.384408	3.415504	35.22202
3.390658	3.501336	3.389773	35.66705
3.296581	3.411635	3.431634	35.3567
3.133381	3.256157	3.407779	34.60764
3.128844	3.193616	3.438046	34.52714
3.318399	3.324764	3.381452	35.1047
3.284085	3.379353	3.354919	35.09102
3.280826	3.297672	3.47058	35.1582
3.187286	3.238972	3.40517	34.68223
3.320791	3.278262	3.449453	35.15695
3.395401	3.422889	3.309682	35.33073
3.43318	3.434683	3.407523	35.6531
3.198711	3.402132	3.38568	35.0214

3.206423	3.367963	3.39702	34.98834
3.217435	3.399639	3.371749	35.02643
3.333948	3.368233	3.357818	35.18208
3.219621	3.316609	3.360308	34.82462
3.177883	3.289113	3.422375	34.80894
3.100964	3.317755	3.44652	34.75617
3.451822	3.432527	3.334389	35.52922
3.398948	3.351115	3.421216	35.42543
3.341908	3.335547	3.327074	35.06078
3.191906	3.358259	3.445207	35.04075
3.281445	3.46993	3.361331	35.29734
3.416311	3.424372	3.392978	35.56185
3.332133	3.3065	3.289629	34.89399
3.148518	3.209993	3.446043	34.62346
3.147074	3.251844	3.502057	34.83432
3.273403	3.341815	3.428429	35.14632
3.303716	3.324225	3.410354	35.13462
3.356261	3.262088	3.39569	35.08157
3.199206	3.230278	3.497061	34.89024
3.319018	3.428618	3.393302	35.35908
3.354817	3.400043	3.394888	35.37835
3.374861	3.422957	3.396934	35.47676
3.167077	3.342219	3.425035	34.90727
3.215208	3.337367	3.419988	34.99087
3.254018	3.389934	3.356983	35.05292
3.425962	3.299963	3.325045	35.16234
3.153261	3.245105	3.408188	34.62784
3.221065	3.327123	3.345984	34.81944
3.178666	3.431785	3.475985	35.2399
3.382615	3.455036	3.333775	35.42576
3.402494	3.358057	3.426007	35.45885
3.309738	3.30495	3.380906	35.04124
3.234922	3.370727	3.472439	35.22164
3.332669	3.492979	3.421676	35.59173
3.322235	3.331234	3.338345	35.03297
3.289982	3.219967	3.353879	34.75309
3.165221	3.229469	3.384026	34.56696
3.066031	3.329953	3.474485	34.76761
3.220199	3.418981	3.361194	35.05169
3.282064	3.3003	3.435249	35.08939
3.27563	3.286417	3.425257	35.02311
3.287054	3.257505	3.386874	34.90093
3.248285	3.32099	3.416782	35.02038
3.368675	3.397212	3.34179	35.28634
3.363066	3.433335	3.417857	35.51942
3.248079	3.328066	3.371221	34.93577
3.204732	3.344376	3.373403	34.88142
3.270804	3.282171	3.398691	34.94517
3.284332	3.274488	3.38522	34.9285
3.232283	3.405165	3.35642	35.03746

3.290807	3.412039	3.448481	35.3818
3.257978	3.416217	3.43143	35.28186
3.338237	3.337097	3.413952	35.24613
3.329205	3.297604	3.369174	35.04209
3.341289	3.385351	3.43663	35.40792
3.283672	3.499988	3.413236	35.48145
3.3297	3.347948	3.463026	35.3585
3.278929	3.189168	3.344535	34.64113
3.31312	3.192403	3.320663	34.67077
3.109213	3.305624	3.425172	34.701
3.239294	3.499988	3.438932	35.4406
3.135897	3.393236	3.444696	34.99364
3.316914	3.297537	3.351321	34.97602
3.274805	3.261212	3.423313	34.96193
3.374696	3.334401	3.359609	35.20112
3.264783	3.393304	3.431924	35.24771
3.353868	3.463056	3.328592	35.36909
3.258844	3.347408	3.467801	35.21281
3.362571	3.225898	3.228294	34.65016
3.208362	3.217743	3.393661	34.65673
3.108924	3.276847	3.360529	34.49607
3.226138	3.431718	3.40251	35.18288
3.214383	3.46191	3.394274	35.20519
3.315017	3.428955	3.376507	35.31434
3.322317	3.332514	3.465345	35.31368
3.228901	3.361292	3.452864	35.14503
3.353415	3.422889	3.372653	35.37662
3.391812	3.511579	3.401641	35.71793
3.285198	3.434009	3.396372	35.30363
3.321946	3.138892	3.351492	34.64047
3.165799	3.145429	3.403056	34.42606
3.265195	3.30495	3.350281	34.87686
3.254183	3.434683	3.410166	35.26744
3.306727	3.403413	3.444474	35.38899
3.19504	3.247059	3.443093	34.79981
3.288786	3.266132	3.392978	34.93693
3.331597	3.404828	3.447254	35.45255
3.46106	3.461708	3.387982	35.73043
3.218508	3.361561	3.512732	35.25381
3.294189	3.353946	3.413491	35.18564
3.307428	3.276375	3.434022	35.08985
3.417631	3.290798	3.311608	35.09469
3.254761	3.321462	3.392075	34.98154
3.194339	3.297807	3.461526	34.94956
3.043924	3.363785	3.36556	34.55505
3.346568	3.374905	3.345712	35.1978
3.290147	3.434683	3.375091	35.26939
3.357787	3.381847	3.439836	35.44334
3.242676	3.38461	3.436699	35.1908
3.370077	3.434414	3.433203	35.57067

3.403773	3.48907	3.368902	35.62327
3.409547	3.374905	3.350691	35.34641
3.227416	3.128783	3.399168	34.5159
3.160437	3.197255	3.35642	34.42568
3.279919	3.319575	3.412537	35.07718
3.205433	3.503694	3.438608	35.37395
3.229643	3.359404	3.40367	35.03495
3.143032	3.229065	3.407302	34.56845
3.224364	3.248205	3.432248	34.84272
3.34265	3.376522	3.437432	35.39334
3.324998	3.434346	3.368697	35.33088
3.261401	3.465617	3.380974	35.28703
3.335598	3.332919	3.471535	35.35714
3.297447	3.244903	3.335532	34.78382
3.381089	3.236613	3.307039	34.88629
3.286023	3.265929	3.365406	34.87015
3.197185	3.415948	3.430901	35.14717
3.230551	3.40476	3.388784	35.10356
3.368304	3.389462	3.360836	35.31024
3.312171	3.385081	3.35792	35.17153
3.277156	3.357922	3.453324	35.24419
3.236737	3.363987	3.440365	35.14073
3.356673	3.470065	3.40558	35.55891
3.309985	3.387171	3.45612	35.38606
3.301366	3.219563	3.370675	34.81383
3.234799	3.193144	3.385953	34.64389
3.272454	3.322742	3.345848	34.92195
3.102077	3.505851	3.447458	35.17199
3.298932	3.410893	3.318276	35.11232
3.172727	3.217069	3.44473	34.68901
3.215126	3.231761	3.355653	34.61906
3.19438	3.291674	3.477691	34.97159
3.297736	3.479904	3.391137	35.41996
3.35857	3.485161	3.421148	35.63012
3.455616	3.352328	3.3452	35.38577
3.351559	3.155876	3.438199	34.93198
3.277898	3.217541	3.333486	34.67676
3.214383	3.349161	3.394172	34.9584
3.228571	3.448499	3.370368	35.15461
3.395937	3.41615	3.380411	35.47184
3.217889	3.344847	3.368475	34.90044
3.281651	3.320653	3.378178	35.00819
3.307882	3.377129	3.447612	35.3409
3.297035	3.457665	3.400737	35.39079
3.27266	3.435222	3.470376	35.44069
3.331721	3.327258	3.376677	35.12885
3.230056	3.172994	3.381042	34.57872
3.380347	3.155067	3.284053	34.65607
3.346321	3.368772	3.422154	35.35101
3.241398	3.48725	3.385987	35.30156

3.205186	3.407456	3.396781	35.07148
3.246718	3.323618	3.418436	35.02632
3.219704	3.274556	3.388545	34.79458
3.312543	3.328134	3.373403	35.08166
3.243831	3.405771	3.472899	35.31876
3.261401	3.432392	3.466266	35.4009
3.241769	3.375175	3.483914	35.27143
3.313285	3.2323	3.385356	34.89985
3.362612	3.192672	3.359404	34.86431
3.349249	3.21343	3.377155	34.9193
3.203454	3.327527	3.376933	34.84949
3.261277	3.447353	3.391137	35.26905
3.348507	3.389395	3.312273	35.1606
3.325782	3.344241	3.366924	35.13167
3.23385	3.380701	3.376882	35.03214
3.259009	3.429224	3.409263	35.26408
3.308418	3.419385	3.431958	35.40025
3.393751	3.41487	3.383498	35.471
3.331927	3.233109	3.462003	35.11
3.219126	3.156347	3.316724	34.37776
3.266515	3.283587	3.433698	35.01544
3.316626	3.441288	3.333912	35.25168
3.345042	3.373288	3.429588	35.37435
3.098943	3.299087	3.432453	34.68017
3.269278	3.316677	3.419306	35.06238
3.284373	3.352059	3.411462	35.15561
3.301283	3.454632	3.406074	35.40512
3.256905	3.342084	3.412741	35.07653
3.240037	3.347274	3.464032	35.16315
3.214919	3.311892	3.457979	35.01761
3.457926	3.25272	3.307022	35.08951
3.358694	3.259325	3.385612	35.05881
3.318605	3.298952	3.398776	35.08659
3.251667	3.323821	3.390029	34.97546
3.255668	3.372411	3.355994	35.01604
3.280249	3.384003	3.365338	35.11558
3.321327	3.450925	3.383634	35.39177
3.211166	3.442164	3.445429	35.26684
3.290354	3.41952	3.352174	35.18656
3.26503	3.36675	3.50586	35.35187
3.410867	3.389058	3.316127	35.30466
3.308872	3.212284	3.436289	34.95781
3.228406	3.192807	3.338413	34.52521
3.260782	3.283047	3.418078	34.96757
3.302479	3.381375	3.347485	35.11941
3.317038	3.386969	3.33294	35.13167
3.154663	3.277319	3.406176	34.69695
3.179244	3.306568	3.390949	34.78137
3.238676	3.331975	3.44091	35.07616
3.305449	3.446342	3.394121	35.36996

3.236201	3.38279	3.461713	35.22736
3.335474	3.425787	3.408052	35.42113
3.246677	3.271456	3.411718	34.89747
3.469845	3.225561	3.349463	35.14899
3.355559	3.278532	3.422103	35.17376
3.277321	3.334469	3.395434	35.06667
3.316749	3.369783	3.388119	35.21412
3.348012	3.422081	3.394615	35.41106
3.210919	3.380499	3.358244	34.94079
3.316749	3.372883	3.31374	35.05825
3.160932	3.406984	3.463026	35.11854
3.170253	3.45726	3.387846	35.08446
3.240697	3.4084	3.473973	35.32001
3.345249	3.31014	3.393064	35.15683
3.2753	3.204466	3.385339	34.75588
3.264741	3.231559	3.385135	34.79159
3.229561	3.388047	3.44623	35.19048
3.341743	3.451599	3.391205	35.45445
3.258926	3.414533	3.394854	35.20026
3.294767	3.257438	3.300354	34.72844
3.144228	3.239781	3.438881	34.66356
3.197721	3.3466	3.41963	34.97204
3.289034	3.469121	3.455728	35.5186
3.29122	3.423092	3.395076	35.29008
3.343681	3.29437	3.394684	35.12246
3.260823	3.199277	3.374085	34.68826
3.302479	3.246722	3.358841	34.84978
3.29155	3.41487	3.419784	35.32686
3.306892	3.472424	3.403192	35.44999
3.252451	3.432661	3.360768	35.15121
3.330566	3.333593	3.33739	35.05426
3.279094	3.337434	3.430969	35.15474
3.277032	3.468919	3.409945	35.39179
3.213022	3.451936	3.40517	35.20423
3.278599	3.33548	3.423126	35.13223
3.133051	3.20723	3.47012	34.63625
3.363313	3.204197	3.371425	34.91733
3.276372	3.263099	3.42345	34.96979
3.374366	3.479702	3.39702	35.59996
3.301077	3.442434	3.455779	35.48669
3.322194	3.379488	3.347314	35.15802
3.259256	3.290932	3.363428	34.86197
3.373335	3.299626	3.368066	35.14059
3.223581	3.376455	3.419681	35.09399
3.199082	3.409074	3.435795	35.14699
3.245852	3.397212	3.507838	35.38087
3.296169	3.302659	3.372994	34.98925
3.394534	3.16558	3.383225	34.92697
3.192029	3.189572	3.440501	34.66184
3.238428	3.327932	3.426724	35.03575

3.347723	3.482668	3.420056	35.59856
3.368634	3.498909	3.339965	35.50466
3.353002	3.408669	3.355329	35.30673
3.277857	3.346061	3.357699	35.01067
3.227746	3.434346	3.396184	35.17831
3.26371	3.462786	3.309153	35.12883
3.329824	3.423361	3.402732	35.39184
3.256823	3.240387	3.463777	34.96556
3.154787	3.142195	3.397872	34.38357
3.22688	3.172252	3.42877	34.67452
3.412269	3.423024	3.354442	35.46579
3.356096	3.503964	3.469148	35.7708
3.182378	3.349969	3.388136	34.87698
3.317038	3.306635	3.413099	35.13129
3.212404	3.332851	3.358313	34.83999
3.347641	3.367357	3.407217	35.31814
3.204072	3.405771	3.387164	35.04433
3.154828	3.410085	3.472626	35.13296
3.120926	3.307579	3.421352	34.72253
3.39338	3.259797	3.347263	35.05183
3.282724	3.172791	3.409808	34.75636
3.349414	3.297672	3.409126	35.1738
3.189513	3.394449	3.520797	35.27996
3.403278	3.48287	3.386755	35.64767
3.447738	3.407928	3.306766	35.40609
3.436149	3.367829	3.312973	35.30662
3.182337	3.360281	3.478032	35.09603
3.257441	3.475591	3.35833	35.25067
3.199824	3.460832	3.497811	35.39742
3.346156	3.348419	3.287719	35.01215
3.24478	3.16558	3.451414	34.74859
3.153632	3.221854	3.432538	34.63105
3.25274	3.283384	3.46289	35.04872
3.325287	3.485094	3.456751	35.63505
3.345744	3.386295	3.364656	35.26233
3.269897	3.277858	3.415128	34.9697
3.277609	3.24032	3.381298	34.8305
3.253152	3.351856	3.410235	35.08421
3.348094	3.416554	3.307465	35.20857
3.20964	3.426461	3.466624	35.27552
3.246924	3.358933	3.412349	35.09069
3.250512	3.220439	3.402237	34.77356
3.367273	3.24962	3.294676	34.85749
3.356756	3.249283	3.408853	35.08344
3.250224	3.395325	3.425973	35.20728
3.286683	3.457665	3.497266	35.57924
3.418703	3.445264	3.338942	35.4946
3.332711	3.364055	3.318992	35.08533
3.394823	3.321866	3.346428	35.1889
3.145259	3.389866	3.450238	35.01886

3.232943	3.469256	3.362064	35.19141
3.193597	3.374231	3.340971	34.85143
3.288704	3.172454	3.419852	34.79066
3.153838	3.238635	3.414412	34.62856
3.269814	3.322338	3.442138	35.12586
3.23484	3.452003	3.437517	35.32283
3.434623	3.403817	3.413764	35.6024
3.398576	3.355091	3.411565	35.41221
3.30516	3.344915	3.389364	35.13712
3.282146	3.349093	3.411053	35.14336
3.230097	3.397549	3.382935	35.07401
3.239583	3.371738	3.370317	35.01072
3.291591	3.339321	3.389892	35.09637
3.313656	3.345993	3.433834	35.25531
3.146208	3.219023	3.451568	34.65024
3.264618	3.253057	3.367947	34.80075
3.278104	3.261481	3.372755	34.85918
3.281486	3.396404	3.423057	35.27163
3.330772	3.442703	3.399509	35.42916
3.347888	3.405434	3.453119	35.50233
3.31576	3.398762	3.285332	35.05055
3.410743	3.336423	3.396065	35.3641
3.269237	3.477411	3.322982	35.20314
3.265649	3.422013	3.455524	35.364
3.102614	3.333525	3.423859	34.74471
3.249193	3.177441	3.357989	34.57988
3.233067	3.205006	3.38302	34.65963
3.289364	3.340669	3.400038	35.11663
3.31477	3.411972	3.487563	35.51952
3.313862	3.419115	3.390029	35.31987
3.36554	3.297807	3.407234	35.20522
3.405876	3.372142	3.341074	35.31131
3.243542	3.387373	3.475832	35.28431
3.232695	3.363448	3.383395	35.00613
3.157179	3.327999	3.414446	34.83137
3.297571	3.379151	3.357153	35.12495
3.301861	3.264177	3.488314	35.16973
3.198711	3.240387	3.443161	34.79339
3.241398	3.240387	3.445958	34.89286
3.340341	3.342286	3.406807	35.24645
3.308996	3.411837	3.433612	35.38862
3.325163	3.408602	3.306698	35.13936
3.335763	3.343702	3.393712	35.2109
3.26602	3.413859	3.377547	35.17645
3.402165	3.412174	3.357989	35.42772
3.237768	3.418913	3.376268	35.12293
3.298809	3.352665	3.328933	35.00802
3.145259	3.152371	3.377905	34.34132
3.215291	3.224078	3.384572	34.66586
3.218013	3.332177	3.469693	35.09435

3.307387	3.445062	3.386686	35.35514
3.250018	3.372614	3.457007	35.22503
3.378944	3.338782	3.414174	35.33932
3.326318	3.326853	3.383617	35.13132
3.440645	3.424911	3.372704	35.57191
3.253193	3.368907	3.440024	35.18673
3.168108	3.273141	3.395758	34.69443
3.161221	3.298076	3.397736	34.73823
3.283837	3.378679	3.350946	35.08031
3.273815	3.291809	3.42142	35.02254
3.2607	3.283587	3.448567	35.03524
3.149342	3.261886	3.519211	34.89875
3.367025	3.372007	3.402101	35.35951
3.33799	3.418509	3.437278	35.47463
3.342238	3.465213	3.350725	35.39678
3.36917	3.360146	3.379252	35.28829
3.248492	3.395864	3.413764	35.17797
3.373088	3.417228	3.291931	35.23073
3.314234	3.417026	3.422768	35.38771
3.260865	3.367155	3.339505	34.97985
3.148476	3.196177	3.405938	34.50545
3.25142	3.243083	3.435045	34.8968
3.219662	3.403682	3.456052	35.2245
3.314192	3.459821	3.388528	35.40632
3.19273	3.381847	3.482329	35.17532
3.35292	3.352463	3.445378	35.38056
3.321616	3.353609	3.392007	35.1979
3.40501	3.428281	3.39083	35.54099
3.280909	3.373894	3.406961	35.18594
3.186503	3.304411	3.336401	34.67324
3.220776	3.306096	3.433152	34.96345
3.314687	3.308859	3.306953	34.89889
3.260287	3.254472	3.42524	34.91966
3.212527	3.28143	3.381912	34.77942
3.141011	3.300974	3.542811	35.01762
3.294725	3.363987	3.414958	35.21198
3.439572	3.437986	3.423978	35.71028
3.390328	3.42855	3.338635	35.39533
3.286765	3.363516	3.37163	35.09879
3.290519	3.380297	3.333946	35.06128
3.287343	3.400043	3.371476	35.17959
3.288456	3.367357	3.369311	35.10582
3.233933	3.280891	3.385339	34.83254
3.058524	3.170096	3.382799	34.20111
3.267587	3.258179	3.439256	34.97438
3.215084	3.453284	3.452932	35.31614
3.317698	3.452475	3.440245	35.51102
3.248574	3.356776	3.457826	35.18903
3.276166	3.326247	3.445054	35.15467
3.257565	3.349093	3.406347	35.07931

3.482218	3.446612	3.346632	35.65326
3.266927	3.316677	3.468773	35.16541
3.274351	3.254809	3.362064	34.813
3.114492	3.211341	3.420107	34.49528
3.337206	3.256023	3.29662	34.80999
3.167819	3.266334	3.418436	34.72851
3.258431	3.295785	3.361331	34.86619
3.203124	3.320586	3.463418	35.02272
3.377996	3.440883	3.367538	35.45854
3.355724	3.467437	3.398691	35.53603
3.510593	3.432459	3.373659	35.74347
3.299262	3.436772	3.411514	35.37354
3.245151	3.351587	3.344791	34.923
3.243089	3.310207	3.365901	34.87417
3.234428	3.249216	3.360495	34.71003
3.265566	3.237894	3.392723	34.82384
3.154498	3.251709	3.419238	34.66915
3.17813	3.39546	3.454586	35.11249
3.324957	3.455777	3.388613	35.42121
3.319554	3.424911	3.392484	35.35036
3.259998	3.360752	3.440365	35.18452
3.298066	3.32254	3.414157	35.12689
3.361004	3.504975	3.412213	35.65923
3.332463	3.435222	3.351697	35.31194
3.322812	3.270377	3.360836	34.95033
3.255833	3.22765	3.349258	34.6851
3.227292	3.226571	3.407489	34.74767
3.343558	3.268019	3.400924	35.07821
3.166128	3.351452	3.398264	34.86684
3.237603	3.382318	3.422972	35.14467
3.151487	3.349363	3.426041	34.891
3.352796	3.417498	3.409416	35.44387
3.309697	3.433605	3.355329	35.26656
3.470464	3.444523	3.388204	35.7139
3.288498	3.393034	3.370607	35.16489
3.334979	3.319642	3.388289	35.14471
3.204402	3.32126	3.354971	34.78983
3.334402	3.253125	3.323715	34.85677
3.205846	3.231289	3.334168	34.55075
3.17846	3.309331	3.374409	34.74953
3.222714	3.440277	3.456052	35.3112
3.279795	3.427876	3.452846	35.4019
3.341248	3.350643	3.446657	35.35385
3.398288	3.408265	3.42154	35.54967
3.273238	3.379825	3.456052	35.28949
3.365705	3.445736	3.419511	35.55593
3.265896	3.373625	3.493361	35.34146
3.281033	3.223404	3.331303	34.69166
3.240779	3.20541	3.368561	34.64576
3.157261	3.220236	3.381827	34.52455

3.251214	3.248946	3.412878	34.8607
3.222508	3.378679	3.385135	35.02096
3.283713	3.33986	3.418556	35.143
3.285487	3.428011	3.375296	35.24505
3.374201	3.410287	3.411241	35.4789
3.269072	3.423496	3.32496	35.0892
3.426828	3.435559	3.371135	35.56155
3.269361	3.372411	3.376814	35.09152
3.338402	3.335884	3.360734	35.12746
3.251544	3.269434	3.394581	34.86621
3.306562	3.231356	3.302997	34.70298
3.20168	3.251844	3.376609	34.6794
3.24779	3.350239	3.373591	34.98881
3.20432	3.410219	3.461304	35.21673
3.329411	3.438592	3.371971	35.35697
3.18011	3.356911	3.434107	34.98773
3.411032	3.37814	3.34844	35.35181
3.293818	3.451599	3.460281	35.5007
3.38344	3.41797	3.360853	35.40572
3.224777	3.230143	3.468687	34.88381
3.265814	3.178318	3.374136	34.65345
3.209228	3.24625	3.383617	34.699
3.263917	3.254607	3.337783	34.73664
3.154127	3.26256	3.411633	34.67543
3.216858	3.330223	3.437295	35.01671
3.312914	3.350845	3.397753	35.18539
3.346197	3.428955	3.36411	35.35542
3.422003	3.445534	3.379627	35.59138
3.247708	3.438525	3.42611	35.29655
3.292375	3.448162	3.388545	35.33315
3.402824	3.34815	3.315752	35.19679
3.378615	3.264784	3.335412	35.00453
3.241604	3.235804	3.405017	34.79376
3.256617	3.293898	3.3622	34.85999
3.209846	3.296324	3.418743	34.88667
3.287632	3.397684	3.400294	35.23809
3.227746	3.404423	3.462822	35.2586
3.260823	3.368435	3.452864	35.23046
3.190462	3.383127	3.406961	35.00834
3.387812	3.436166	3.37579	35.48773
3.296086	3.435694	3.470563	35.49337
3.419569	3.346667	3.398623	35.41139
3.207413	3.203523	3.39216	34.62027
3.243212	3.228189	3.353623	34.66823
3.27299	3.232772	3.351304	34.7383
3.284002	3.30522	3.404898	35.03801
3.185637	3.337906	3.43218	34.95404
3.218384	3.385688	3.409143	35.07977
3.220735	3.365605	3.423927	35.07332
3.405423	3.437986	3.425087	35.63803

3.348136	3.456654	3.419033	35.54034
3.403154	3.371872	3.448942	35.54066
3.316419	3.311825	3.396304	35.10455
3.351064	3.278936	3.370931	35.05291
3.29122	3.279543	3.347672	34.8725
3.284291	3.290798	3.386772	34.96747
3.199824	3.33184	3.399168	34.89962
3.206877	3.410219	3.409416	35.10885
3.339227	3.419183	3.395553	35.38756
3.302438	3.368772	3.416083	35.24177
3.300788	3.390473	3.424149	35.30326
3.175202	3.366548	3.435198	35.00046
3.33733	3.427809	3.447816	35.51657
3.389791	3.418374	3.402715	35.51203
3.335763	3.264312	3.383344	35.01461
3.209682	3.154258	3.385612	34.50318
3.265814	3.215384	3.41431	34.82236
3.232324	3.238837	3.346752	34.65268
3.301737	3.326112	3.433629	35.18532
3.130494	3.373962	3.396048	34.83329
3.204815	3.348554	3.433135	35.02136
3.306191	3.421811	3.367299	35.25928
3.343104	3.430235	3.40089	35.43188
3.340382	3.466358	3.426246	35.56038
3.382863	3.396336	3.433271	35.51551
3.261153	3.294841	3.448055	35.05973
3.356591	3.247396	3.300695	34.84243
3.366901	3.250698	3.354203	34.98921
3.350445	3.298278	3.360427	35.07088
3.170294	3.343567	3.423808	34.91456
3.239665	3.453014	3.342045	35.12681
3.18873	3.385621	3.405716	35.00728
3.353827	3.393169	3.322573	35.20301
3.233355	3.41952	3.479788	35.34098
3.203536	3.423361	3.453801	35.22735
3.31345	3.403413	3.456052	35.42901
3.308459	3.312296	3.395553	35.08654
3.308418	3.189168	3.33485	34.68444
3.223704	3.202242	3.422699	34.71989
3.185018	3.264649	3.444611	34.81967
3.183863	3.351856	3.369413	34.84341
3.335391	3.378005	3.397122	35.29256
3.262556	3.348554	3.390847	35.05515
3.262391	3.351991	3.392007	35.06484
3.172686	3.421744	3.420517	35.08356
3.38245	3.495877	3.368339	35.59029
3.355518	3.422687	3.377479	35.39133
3.36022	3.376253	3.354629	35.2501
3.260947	3.245307	3.438199	34.9294
3.342197	3.308657	3.363019	35.08121

3.247502	3.320586	3.396321	34.97304
3.401628	3.354957	3.31154	35.19985
3.162334	3.338378	3.408188	34.85165
3.190792	3.365739	3.409007	34.97551
3.223746	3.385014	3.437841	35.15278
3.331638	3.442366	3.403414	35.43886
3.297118	3.436368	3.434499	35.41823
3.301159	3.378544	3.463299	35.3636
3.219003	3.313307	3.487563	35.09433
3.314481	3.278465	3.402357	35.0406
3.448357	3.236276	3.366702	35.16313
3.230633	3.238028	3.393388	34.7492
3.256039	3.301176	3.376797	34.90657
3.231747	3.342623	3.410627	35.01806
3.3297	3.417902	3.340511	35.24356
3.340176	3.380162	3.35792	35.22201
3.230468	3.424642	3.437978	35.25444
3.207166	3.413656	3.433663	35.17002
3.304665	3.403008	3.411837	35.31222
3.322853	3.30677	3.392007	35.09817
3.336464	3.229874	3.333366	34.83154
3.219126	3.245981	3.392518	34.73952
3.286683	3.295178	3.430083	35.07699
3.300252	3.394584	3.343955	35.1357
3.385378	3.3621	3.341994	35.24654
3.27233	3.316744	3.404301	35.03639
3.222137	3.395393	3.417379	35.12721
3.165757	3.415746	3.352328	34.90617
3.347435	3.45106	3.421267	35.53146
3.24779	3.430437	3.363991	35.1432
3.227705	3.281295	3.4434	34.94678
3.216652	3.272467	3.461372	34.9426
3.373665	3.235535	3.353351	34.96898
3.360467	3.250496	3.339829	34.94326
3.380553	3.30859	3.388392	35.22043
3.218673	3.33676	3.405665	34.9658
3.30252	3.421339	3.359336	35.23281
3.219992	3.397819	3.443588	35.18514
3.339681	3.401728	3.333162	35.21395
3.227829	3.404963	3.457843	35.24907
3.235789	3.46218	3.423603	35.31673
3.235995	3.311151	3.455643	35.05697
3.420023	3.248677	3.324482	35.03596
3.333577	3.223674	3.390506	34.93662
3.217683	3.285541	3.370794	34.77537
3.317121	3.388384	3.415538	35.31557
3.279424	3.38279	3.359506	35.09837
3.381337	3.31169	3.338959	35.12082
3.310315	3.371131	3.342983	35.1043
3.279383	3.396336	3.418675	35.2573

3.219539	3.471211	3.419357	35.29166
3.298149	3.472424	3.431225	35.49217
3.23187	3.315599	3.42599	34.99283
3.354611	3.258651	3.36174	34.99621
3.191204	3.217608	3.429469	34.69722
3.29852	3.240252	3.362814	34.83566
3.257936	3.263166	3.404369	34.88789
3.358941	3.339119	3.368885	35.19727
3.263298	3.362774	3.457979	35.23468
3.29456	3.405097	3.347553	35.15412
3.207496	3.321597	3.441729	34.98706
3.32871	3.474041	3.314541	35.30737
3.197308	3.471008	3.439973	35.28768
3.347764	3.44176	3.352975	35.3625
3.267257	3.237354	3.426928	34.90116
3.275588	3.247666	3.369993	34.81742
3.367355	3.279678	3.374699	35.0984
3.433881	3.404154	3.387999	35.54517
3.259297	3.393438	3.404488	35.17601
3.331515	3.34296	3.369174	35.14633
3.237191	3.267951	3.32791	34.68578
3.33667	3.403413	3.428838	35.42027
3.258679	3.493788	3.351049	35.27724
3.172934	3.457193	3.499159	35.3336
3.136722	3.339119	3.430611	34.8463
3.284002	3.214845	3.378706	34.7831
3.327143	3.245913	3.421744	35.0395
3.294932	3.293156	3.341193	34.89622
3.281816	3.288574	3.422853	35.0361
3.220199	3.313644	3.357426	34.81309
3.391977	3.359135	3.379542	35.33659
3.354858	3.425585	3.326119	35.28391
3.264412	3.368098	3.439427	35.20819
3.178666	3.406378	3.378178	34.97044
3.291261	3.432998	3.398299	35.31889
3.285157	3.450116	3.271895	35.06655
3.347352	3.329616	3.429793	35.28434
3.150085	3.220034	3.388988	34.52408
3.226303	3.260673	3.427764	34.86442
3.29753	3.426663	3.360904	35.23697
3.437716	3.454632	3.363786	35.611
3.260287	3.334199	3.410149	35.06101
3.288993	3.245846	3.392109	34.89112
3.22156	3.338243	3.43056	35.0298
3.346527	3.557138	3.264904	35.41951
3.17281	3.418104	3.473394	35.1915
3.233108	3.350643	3.340323	34.88484
3.149177	3.268221	3.399731	34.65096
3.254059	3.262627	3.343921	34.74604
3.326565	3.286889	3.473718	35.2415

3.397504	3.292415	3.385697	35.21623
3.233396	3.270108	3.478032	35.01049
3.363932	3.396336	3.332394	35.25351
3.369747	3.383599	3.409689	35.4074
3.445553	3.400986	3.386107	35.55963
3.197762	3.337367	3.427542	34.96924
3.070114	3.401728	3.415418	34.80433
3.279218	3.426394	3.49863	35.49752
3.255668	3.425248	3.295921	35.00022
3.363643	3.210869	3.28997	34.75452
3.194009	3.17023	3.416919	34.5723
3.221353	3.383329	3.395519	35.05131
3.26404	3.392832	3.423518	35.22667
3.459905	3.40813	3.322163	35.46681
3.253812	3.283789	3.425445	34.97006
3.263422	3.375916	3.415128	35.16998
3.280373	3.411432	3.405307	35.26324
3.370696	3.457934	3.343256	35.42676
3.246388	3.381779	3.355789	35.01579
3.258844	3.27368	3.355448	34.80588
3.120472	3.221315	3.428446	34.54841
3.31378	3.239646	3.377922	34.90074
3.278517	3.289248	3.407575	34.99694
3.308459	3.266603	3.430969	35.06406
3.290312	3.350576	3.404488	35.1501
3.392637	3.36365	3.402664	35.39847
3.332257	3.399436	3.385118	35.30632
3.333535	3.377803	3.341841	35.16717
3.167283	3.374501	3.402391	34.92879
3.337124	3.461236	3.46659	35.63028
3.163118	3.389395	3.363172	34.86649
3.283837	3.277454	3.32791	34.80857
3.260493	3.189909	3.363906	34.64479
3.273939	3.302389	3.369925	34.93334
3.333412	3.456586	3.446896	35.56892
3.360674	3.411769	3.36868	35.35949
3.282971	3.25946	3.391768	34.90697
3.370366	3.259662	3.283968	34.86279
3.258555	3.37969	3.425854	35.19105
3.247502	3.494124	3.354561	35.26122
3.288291	3.428753	3.465533	35.45014
3.180605	3.320114	3.435045	34.9104
3.218343	3.27651	3.346939	34.7049
3.227086	3.275499	3.416493	34.87391
3.204939	3.250564	3.436204	34.81405
3.268041	3.286282	3.419732	34.99414
3.339804	3.311757	3.396576	35.15614
3.298107	3.358933	3.352055	35.07076
3.366324	3.339052	3.350946	35.17404
3.298396	3.345724	3.396372	35.13942

3.294684	3.37012	3.346513	35.07562
3.245646	3.390203	3.35688	35.03497
3.347146	3.407389	3.310926	35.19402
3.222261	3.32928	3.413287	34.97395
3.19071	3.274893	3.367384	34.68564
3.177347	3.242207	3.423245	34.7071
3.309573	3.470335	3.443656	35.53977
3.334897	3.417767	3.371425	35.32224
3.315842	3.30859	3.392774	35.0885
3.259132	3.285608	3.411445	34.95506
3.386946	3.385014	3.387727	35.40008
3.312625	3.494866	3.371749	35.44284
3.287879	3.416622	3.339692	35.14752
3.096675	3.204399	3.43409	34.47172
3.177842	3.230278	3.341875	34.50415
3.237851	3.254607	3.417123	34.85314
3.262432	3.348487	3.391273	35.05567
3.211537	3.329953	3.497931	35.13708
3.185142	3.269973	3.403209	34.74105
3.358983	3.366279	3.418147	35.36448
3.358158	3.446477	3.379269	35.45304
3.367932	3.40476	3.39864	35.42555
3.265154	3.328471	3.419988	35.08064
3.24412	3.367087	3.34624	34.95782
3.36851	3.445534	3.441951	35.61069
3.366943	3.422283	3.303441	35.25352
3.187369	3.246924	3.379064	34.64272
3.195659	3.186607	3.375108	34.52029
3.195535	3.320788	3.419101	34.90966
3.324421	3.423429	3.42836	35.43621
3.327225	3.386092	3.391393	35.27986
3.321616	3.283924	3.452454	35.1777
3.248533	3.332582	3.411496	35.03471
3.368881	3.47613	3.348303	35.47362
3.298479	3.451936	3.436	35.45853
3.292333	3.333525	3.274453	34.83287
3.07696	3.212351	3.462515	34.50816
3.170665	3.239309	3.315888	34.45138
3.31576	3.329886	3.405051	35.16174
3.186338	3.334334	3.357511	34.78448
3.204774	3.286821	3.433987	34.88813
3.263875	3.301985	3.439887	35.06344
3.392431	3.390338	3.375705	35.39743
3.461266	3.392158	3.361126	35.52006
3.340836	3.352935	3.40899	35.27559
3.165881	3.324629	3.449658	34.92003
3.276908	3.412646	3.345353	35.12721
3.280208	3.383936	3.363991	35.1124
3.37878	3.328538	3.386004	35.25495
3.180894	3.251709	3.400447	34.68578

3.167407	3.314588	3.397258	34.78682
3.247914	3.377938	3.434857	35.18363
3.370407	3.440749	3.362831	35.43135
3.286518	3.343634	3.400959	35.11891
3.285157	3.277117	3.377632	34.91946
3.216074	3.367829	3.491059	35.2148
3.389627	3.546557	3.341756	35.65868
3.36752	3.392023	3.386209	35.36961
3.245522	3.223606	3.347826	34.65058
3.130577	3.182429	3.369311	34.35615
3.20865	3.334671	3.38609	34.89651
3.203289	3.467369	3.410303	35.22792
3.248203	3.377331	3.391444	35.088
3.200113	3.25764	3.41963	34.78273
3.280125	3.301042	3.488876	35.20405
3.25542	3.381847	3.427013	35.19144
3.334567	3.407254	3.416237	35.39652
3.278352	3.445197	3.391529	35.30253
3.285652	3.32281	3.4109	35.09321
3.207661	3.285608	3.363292	34.73719
3.298602	3.28945	3.293943	34.79281
3.310068	3.239983	3.335003	34.7995
3.182626	3.370794	3.383617	34.91318
3.204897	3.436772	3.415333	35.17553
3.333535	3.446005	3.372977	35.3844
3.407444	3.365403	3.416152	35.46418
3.384677	3.318564	3.326222	35.1153
3.304665	3.364324	3.425632	35.25779
3.249811	3.509895	3.423722	35.452
3.321492	3.406647	3.408325	35.3493
3.298437	3.217339	3.358995	34.77702
3.303222	3.145901	3.401351	34.72388
3.140434	3.24989	3.392092	34.57505
3.202588	3.441692	3.468602	35.29773
3.23253	3.414802	3.420193	35.19854
3.234345	3.28918	3.378706	34.83707
3.20531	3.288911	3.388613	34.79465
3.331308	3.39883	3.441661	35.42657
3.269814	3.393506	3.449453	35.29749
3.40134	3.475389	3.340306	35.52549
3.311017	3.360281	3.412144	35.23335
3.360426	3.270512	3.331832	34.96946
3.298025	3.262088	3.332889	34.81688
3.246388	3.245913	3.319571	34.63947
3.165675	3.310274	3.447748	34.88401
3.253606	3.429629	3.299655	35.01345
3.21991	3.433942	3.527601	35.44768
3.26404	3.40685	3.343631	35.08263
3.338691	3.322473	3.381111	35.14332
3.342485	3.400784	3.437347	35.44585

3.285116	3.465145	3.423381	35.4306
3.309243	3.462517	3.413031	35.45498
3.239583	3.264919	3.387368	34.81441
3.262349	3.121303	3.35272	34.47436
3.268866	3.211475	3.38655	34.75978
3.261442	3.377129	3.396048	35.12658
3.202588	3.487318	3.466641	35.39321
3.164602	3.3065	3.482789	34.95004
3.281693	3.285945	3.410917	35.00397
3.282724	3.290596	3.363974	34.91374
3.381295	3.407726	3.465806	35.60814
3.182873	3.472087	3.495083	35.37899
3.230179	3.451262	3.388016	35.20277
3.307965	3.311488	3.379405	35.04837
3.415321	3.268827	3.288128	34.99025
3.247007	3.203118	3.366651	34.65019
3.217353	3.277993	3.407847	34.83917
3.175326	3.415678	3.412196	35.05787
3.273073	3.425248	3.386209	35.23573
3.267092	3.429629	3.330143	35.10962
3.293034	3.347745	3.401027	35.1423
3.158581	3.301783	3.42495	34.80007
3.357292	3.422552	3.383498	35.40807
3.340341	3.542176	3.420261	35.713
3.379109	3.335817	3.368185	35.23262
3.286683	3.149136	3.344075	34.56954
3.29852	3.144688	3.349719	34.59804
3.232902	3.266064	3.359745	34.7419
3.377542	3.491227	3.385595	35.60713
3.167654	3.384812	3.41078	34.9705
3.09985	3.243824	3.382338	34.45171
3.206382	3.22273	3.393388	34.66271
3.338815	3.357517	3.374955	35.20677
3.333412	3.433807	3.377615	35.3676
3.243501	3.451599	3.43866	35.34338
3.192689	3.28325	3.471569	34.93608
3.382821	3.210667	3.272219	34.7572
3.366819	3.213295	3.294079	34.77575
3.286518	3.283721	3.32409	34.81979
3.214218	3.435559	3.447663	35.26396
3.22391	3.435088	3.405034	35.1909
3.356343	3.418172	3.400601	35.43382
3.396762	3.392023	3.354561	35.36435
3.236325	3.361292	3.468739	35.19598
3.217971	3.366076	3.402732	35.02196
3.290931	3.450386	3.444662	35.45758
3.300788	3.36055	3.357631	35.09235
3.417218	3.162076	3.342932	34.88079
3.193597	3.174476	3.412537	34.5711
3.270186	3.271321	3.409433	34.94359

3.254472	3.528967	3.435045	35.52866
3.325204	3.423024	3.32883	35.21939
3.251007	3.258044	3.422785	34.90181
3.210094	3.187551	3.44016	34.69618
3.162128	3.359202	3.498494	35.09422
3.364221	3.434144	3.35231	35.38037
3.293364	3.476198	3.388664	35.39689
3.320379	3.304007	3.392092	35.08691
3.317121	3.196514	3.391137	34.84262
3.441634	3.20049	3.339641	35.011
3.29254	3.275365	3.35347	34.87893
3.357539	3.485229	3.362831	35.50048
3.147816	3.398628	3.426178	34.991
3.316626	3.418509	3.286355	35.09787
3.229148	3.319103	3.341346	34.80944
3.301778	3.37194	3.373932	35.15507
3.271423	3.383936	3.392416	35.15535
3.279383	3.470604	3.504888	35.60824
3.236242	3.412106	3.428412	35.21873
3.345042	3.148058	3.34965	34.70699
3.372222	3.144351	3.347212	34.75299
3.228983	3.298346	3.468125	35.04093
3.276578	3.486374	3.418743	35.44821
3.281899	3.428618	3.449197	35.40014
3.306439	3.289719	3.364809	34.96551
3.302562	3.193751	3.313245	34.63441
3.249935	3.277184	3.484989	35.07735
3.120348	3.426124	3.412008	34.96008
3.252616	3.476063	3.444679	35.42998
3.288044	3.423833	3.374563	35.2399
3.349043	3.195436	3.301821	34.71475
3.285033	3.165378	3.347076	34.60801
3.282229	3.222663	3.430833	34.91031
3.222343	3.379151	3.380019	35.01045
3.335144	3.461573	3.398623	35.47805
3.300005	3.402671	3.336759	35.13711
3.299221	3.368772	3.394888	35.18838
3.151198	3.367627	3.383668	34.83764
3.262391	3.451329	3.357494	35.20661
3.234386	3.444927	3.430219	35.2904
3.262102	3.41743	3.459531	35.35498
3.227829	3.230952	3.46069	34.87477
3.253193	3.152304	3.294216	34.39419
3.408062	3.263166	3.401368	35.20963
3.423611	3.438053	3.280455	35.36166
3.246141	3.424102	3.490019	35.40134
3.203866	3.316475	3.325727	34.71425
3.199123	3.202377	3.372022	34.5556
3.317616	3.321327	3.345848	35.01761
3.237438	3.457867	3.439597	35.34588

3.191246	3.441423	3.48724	35.31309
3.226055	3.371872	3.450186	35.15609
3.356756	3.280284	3.370675	35.06774
3.357044	3.210127	3.356761	34.88453
3.287797	3.261549	3.358432	34.8492
3.289776	3.312094	3.401896	35.05911
3.20333	3.34633	3.426365	34.99844
3.395277	3.415813	3.304242	35.30308
3.315553	3.359741	3.386192	35.18533
3.353539	3.36237	3.325983	35.14248
3.131855	3.413656	3.453887	35.04956
3.264824	3.44176	3.348917	35.17224
3.297447	3.394449	3.49909	35.46853
3.275052	3.323888	3.307687	34.84668
3.181677	3.147047	3.455916	34.57991
3.335515	3.275432	3.377751	35.02616
3.306356	3.345117	3.442752	35.25693
3.411486	3.424439	3.410081	35.58885
3.260535	3.374029	3.410354	35.1491
3.175614	3.282845	3.429605	34.80609
3.206217	3.334604	3.393865	34.90804
3.293901	3.349835	3.403806	35.15484
3.218673	3.408332	3.390659	35.0895
3.224405	3.385958	3.46555	35.21688
3.150497	3.378207	3.464305	35.03559
3.379604	3.281632	3.391529	35.16626
3.428725	3.277049	3.343921	35.15955
3.267629	3.270647	3.374085	34.85922
3.297777	3.306029	3.396645	35.05186
3.235747	3.383936	3.33739	34.957
3.391194	3.395123	3.31611	35.27486
3.361581	3.405569	3.345763	35.29779
3.323513	3.409276	3.376592	35.29007
3.072506	3.411298	3.460844	34.92982
3.277733	3.410961	3.383771	35.20934
3.276496	3.382318	3.474962	35.34342
3.231252	3.301244	3.374256	34.84695
3.146497	3.160997	3.425581	34.46715
3.293076	3.281363	3.433629	35.06852
3.327638	3.400919	3.393729	35.31829
3.363066	3.394651	3.406961	35.411
3.312501	3.353137	3.392348	35.17768
3.26136	3.204803	3.404983	34.76909
3.18609	3.256966	3.383771	34.67217
3.26503	3.357113	3.364826	35.02238
3.215043	3.445399	3.455916	35.30533
3.322853	3.475254	3.454637	35.60358
3.215538	3.283115	3.437432	34.9111
3.327926	3.148664	3.365201	34.7049
3.373294	3.238163	3.411377	35.10081

3.359519	3.405569	3.425359	35.46735
3.307676	3.420463	3.406313	35.3449
3.316296	3.441288	3.332803	35.24853
3.372346	3.299761	3.273157	34.93117
3.412269	3.333256	3.325591	35.20639
3.222467	3.455643	3.427815	35.28251
3.085869	3.458204	3.436665	35.00875
3.290601	3.382588	3.393916	35.19762
3.278146	3.253596	3.432589	34.97287
3.209352	3.189303	3.393916	34.59726
3.218219	3.2879	3.407762	34.86254
3.269649	3.445803	3.445787	35.40348
3.289446	3.436638	3.47457	35.48967
3.392555	3.387575	3.373676	35.38722
3.244573	3.296054	3.409672	34.94219
3.282105	3.241735	3.376814	34.83362
3.224983	3.337906	3.382475	34.93139
3.24746	3.433672	3.461304	35.36236
3.138248	3.450251	3.400123	35.02599
3.310192	3.291943	3.364417	34.97773
3.25707	3.156482	3.422273	34.69185
3.350404	3.170298	3.342983	34.75277
3.23418	3.278465	3.42183	34.90758
3.420518	3.465213	3.451278	35.78786
3.350033	3.463258	3.389142	35.49356
3.294272	3.401728	3.386209	35.23065
3.370531	3.344982	3.320663	35.12998
3.29852	3.409141	3.418812	35.32745
3.236366	3.509625	3.391	35.35045
3.239913	3.437783	3.434431	35.29608
3.109707	3.220641	3.433681	34.53484
3.266474	3.13532	3.390046	34.59566
3.277898	3.169489	3.461935	34.85257
3.267793	3.43037	3.392978	35.25019
3.325658	3.464876	3.469762	35.6201
3.290395	3.38899	3.359302	35.13547
3.225436	3.339726	3.417601	35.01318
3.32772	3.343702	3.317355	35.02633
3.199989	3.32591	3.432129	34.95909
3.167737	3.36392	3.386004	34.87081
3.249234	3.464606	3.456649	35.4237
3.161963	3.363111	3.504223	35.11494
3.332504	3.273141	3.361263	34.9785
3.27365	3.187214	3.420449	34.79132
3.2501	3.191999	3.439154	34.79119
3.198793	3.353339	3.398674	34.94329
3.437634	3.461102	3.410303	35.72669
3.373418	3.42882	3.412008	35.51939
3.434829	3.378342	3.349224	35.40601
3.277774	3.34168	3.439205	35.17915

3.301984	3.411028	3.375978	35.24548
3.199329	3.488463	3.40633	35.2567
3.292746	3.384475	3.354988	35.12131
3.16287	3.215047	3.485449	34.75208
3.222756	3.180879	3.371647	34.55945
3.207248	3.272264	3.501154	35.00859
3.451863	3.50201	3.380156	35.78134
3.306851	3.369244	3.475985	35.38345
3.291715	3.298952	3.433987	35.10479
3.27134	3.340467	3.408717	35.09576
3.278764	3.297807	3.412281	35.02649
3.221271	3.356507	3.351015	34.89515
3.142372	3.397684	3.461747	35.05482
3.185059	3.375512	3.436	35.04337
3.235665	3.285204	3.418692	34.9187
3.343764	3.255416	3.340528	34.91902
3.232984	3.178722	3.465243	34.78178
3.298314	3.361224	3.400259	35.18164
3.220776	3.41925	3.484273	35.32269
3.471288	3.467706	3.295921	35.5646
3.452358	3.379623	3.379917	35.51426
3.354982	3.385755	3.354374	35.25887
3.241109	3.350845	3.433595	35.10675
3.30483	3.494124	3.326733	35.32573
3.213187	3.435222	3.328114	34.99953
3.308913	3.185798	3.339897	34.68919
3.091065	3.124335	3.403823	34.21818
3.251214	3.261886	3.43218	34.93121
3.205681	3.480107	3.401027	35.24072
3.353456	3.454834	3.481152	35.68384
3.273361	3.344174	3.423688	35.14102
3.386451	3.247801	3.426741	35.18425
3.257235	3.328943	3.420534	35.06555
3.360467	3.388519	3.36677	35.30401
3.152889	3.340736	3.388681	34.7935
3.16518	3.379825	3.399185	34.92882
3.11111	3.305894	3.405989	34.66379
3.278847	3.27496	3.439018	35.03518
3.331597	3.274084	3.375108	35.00886
3.28792	3.252451	3.444065	35.01684
3.196855	3.360618	3.418061	34.99737
3.337247	3.45908	3.454756	35.59995
3.335309	3.423159	3.414583	35.42931
3.444233	3.375916	3.24879	35.20163
3.255874	3.397482	3.382458	35.12919
3.218013	3.436772	3.384453	35.13668
3.282352	3.431651	3.450238	35.41004
3.230262	3.346128	3.323817	34.83264
3.21727	3.175554	3.394343	34.58544
3.206258	3.212958	3.435096	34.73228

3.224694	3.373018	3.47162	35.20249
3.350569	3.418374	3.414174	35.45132
3.420105	3.42262	3.400822	35.58347
3.25971	3.356641	3.457638	35.21267
3.363602	3.418846	3.347553	35.33516
3.291591	3.313712	3.410422	35.08526
3.312419	3.319979	3.363309	35.04148
3.23983	3.336423	3.39794	34.99444
3.280043	3.410624	3.389756	35.22674
3.12241	3.279139	3.373062	34.55798
3.302438	3.283856	3.33693	34.88298
3.186709	3.213901	3.463026	34.75267
3.286394	3.361561	3.406961	35.17097
3.228983	3.420463	3.457161	35.28401
3.430994	3.474648	3.406279	35.73299
3.314687	3.373153	3.440382	35.33127
3.406413	3.38771	3.328183	35.31833
3.264082	3.378881	3.423245	35.19566
3.296416	3.427809	3.330553	35.17066
3.19801	3.36055	3.423603	35.01187
3.19867	3.25299	3.385885	34.69561
3.272042	3.215452	3.320475	34.63093
3.241398	3.25946	3.426468	34.89194
3.29188	3.441962	3.379798	35.29938
3.206382	3.442905	3.458593	35.28679
3.296086	3.344106	3.376745	35.08791
3.403361	3.316946	3.366924	35.24163
3.365788	3.348689	3.377956	35.25301
3.22853	3.348285	3.454773	35.11995
3.264659	3.375916	3.404147	35.14867
3.17347	3.329886	3.417055	34.87682
3.314687	3.253866	3.338192	34.84694
3.276207	3.174207	3.38169	34.68371
3.198257	3.267816	3.403158	34.7649
3.149095	3.33211	3.462123	34.92694
3.289075	3.461978	3.374682	35.32583
3.272536	3.448095	3.404966	35.32553
3.450997	3.392764	3.314933	35.39791
3.262473	3.363313	3.42038	35.15183
3.282146	3.362168	3.358125	35.05621
3.33568	3.353204	3.363854	35.1662
3.38608	3.333458	3.346121	35.19446
3.147033	3.272534	3.362814	34.57497
3.178666	3.190112	3.350622	34.43725
3.154127	3.260471	3.393439	34.63108
3.324709	3.467841	3.429077	35.53554
3.32273	3.471345	3.482312	35.65529
3.274062	3.405569	3.454705	35.34465
3.272743	3.368233	3.403005	35.14705
3.361581	3.329414	3.479174	35.42301

3.381955	3.387103	3.338772	35.28668
3.267958	3.306029	3.405239	35.00544
3.126081	3.281969	3.397872	34.62646
3.13899	3.274354	3.376268	34.59079
3.365045	3.259864	3.357324	35.01202
3.283548	3.233715	3.391904	34.85224
3.203536	3.319979	3.369345	34.81658
3.121297	3.339928	3.428019	34.80867
3.33898	3.480376	3.444253	35.62734
3.446914	3.437177	3.304344	35.46295
3.390988	3.406984	3.446725	35.58599
3.20168	3.422889	3.398486	35.10129
3.351559	3.400515	3.394138	35.37061
3.276125	3.371603	3.358722	35.06498
3.376429	3.266334	3.393746	35.13071
3.218136	3.135186	3.374154	34.45491
3.111687	3.20386	3.435863	34.50725
3.193102	3.39701	3.376404	34.97765
3.328215	3.45389	3.430679	35.5162
3.257771	3.383262	3.428906	35.20382
3.315265	3.336086	3.464305	35.30379
3.197102	3.310948	3.416714	34.88635
3.3735	3.41952	3.381861	35.43331
3.388224	3.401997	3.422785	35.51668
3.354652	3.302322	3.381588	35.1352
3.197515	3.249351	3.436153	34.79505
3.25575	3.212149	3.300832	34.54513
3.185843	3.214036	3.404778	34.62369
3.307098	3.317081	3.282672	34.84717
3.151652	3.326988	3.535308	35.08137
3.172521	3.43839	3.447884	35.17945
3.305242	3.440614	3.44652	35.47157
3.453884	3.42754	3.367725	35.59572
3.367726	3.415611	3.382748	35.41407
3.251585	3.404828	3.458797	35.30282
3.245811	3.379892	3.399714	35.10645
3.256905	3.274556	3.373591	34.84324
3.372346	3.26074	3.278955	34.85852
3.222962	3.248542	3.444423	34.86702
3.228159	3.370592	3.370914	34.98453
3.168768	3.421676	3.457826	35.15643
3.347599	3.414802	3.382338	35.3674
3.258637	3.346802	3.429179	35.12658
3.287632	3.361022	3.396116	35.14878
3.19273	3.381577	3.456052	35.11726
3.389874	3.414465	3.391324	35.47876
3.270186	3.326988	3.41506	35.07763
3.354858	3.232839	3.342284	34.89775
3.239171	3.180205	3.438097	34.73919
3.214631	3.21033	3.398947	34.66578

3.251626	3.339658	3.394803	35.02045
3.204856	3.345993	3.394769	34.93195
3.219044	3.406243	3.33219	34.95788
3.217971	3.461371	3.481851	35.40338
3.259504	3.43219	3.392279	35.23451
3.376057	3.436907	3.381418	35.47596
3.377253	3.44304	3.390318	35.51145
3.332422	3.368503	3.403414	35.27904
3.284621	3.293426	3.348286	34.88977
3.272825	3.212419	3.343307	34.67594
3.319595	3.225358	3.332786	34.78351
3.303222	3.322271	3.331644	34.95714
3.178749	3.426259	3.409894	35.08346
3.234345	3.433133	3.406483	35.21261
3.297447	3.347948	3.504223	35.37806
3.296004	3.353946	3.407899	35.17738
3.317863	3.35435	3.403465	35.21637
3.213022	3.425113	3.412349	35.16127
3.246553	3.360752	3.468687	35.21706
3.249193	3.248879	3.323545	34.66078
3.384554	3.178655	3.386584	34.94108
3.254142	3.208308	3.368902	34.68206
3.193514	3.242342	3.415418	34.72563
3.211414	3.328673	3.397992	34.91546
3.328545	3.408871	3.371903	35.28994
3.186998	3.435155	3.429793	35.16447
3.337247	3.440075	3.378382	35.39137
3.21793	3.42909	3.403567	35.1615
3.318523	3.435088	3.386073	35.35634
3.332504	3.390136	3.43484	35.39526
3.425921	3.377129	3.361928	35.41165
3.250389	3.272264	3.4589	35.01053
3.280579	3.238433	3.328984	34.71846
3.184935	3.206084	3.406193	34.60741
3.328957	3.379353	3.330502	35.13575
3.276661	3.414937	3.45566	35.3729
3.176151	3.383734	3.372431	34.90286
3.168149	3.373759	3.476258	35.0906
3.296086	3.360955	3.37354	35.11775
3.299592	3.404423	3.511981	35.52322
3.273155	3.402199	3.380343	35.17267
3.199824	3.252248	3.455711	34.84921
3.254431	3.209993	3.341432	34.62631
3.410743	3.209723	3.34832	34.98261
3.273361	3.271725	3.354442	34.83116
3.20399	3.31142	3.385714	34.83465
3.217105	3.326112	3.447765	35.03115
3.305036	3.375714	3.330433	35.07533
3.292333	3.450386	3.386209	35.33282
3.349827	3.423563	3.380224	35.3868

3.167489	3.377129	3.458849	35.05845
3.310934	3.433335	3.371681	35.30444
3.283012	3.302255	3.38389	34.98342
3.443284	3.316946	3.319981	35.22628
3.234056	3.248272	3.426672	34.85187
3.306356	3.237557	3.380633	34.88587
3.21793	3.321394	3.356079	34.82214
3.314069	3.449645	3.367111	35.33696
3.160684	3.421002	3.389892	34.98872
3.297283	3.332312	3.374409	35.05963
3.135732	3.343837	3.455319	34.90848
3.278393	3.383397	3.4124	35.21311
3.394534	3.431246	3.412281	35.57147
3.335928	3.335412	3.446913	35.30947
3.209022	3.257505	3.411087	34.78323
3.261524	3.203388	3.415418	34.78918
3.325411	3.185529	3.335464	34.71498
3.364633	3.323888	3.329393	35.09005
3.153054	3.340062	3.374358	34.76106
3.173222	3.327325	3.391461	34.81471
3.317698	3.374838	3.320202	35.07873
3.313656	3.448971	3.398912	35.40413
3.295509	3.449645	3.3777	35.31953
3.283012	3.39418	3.406756	35.23445
3.220735	3.293022	3.4146	34.8942
3.398329	3.241331	3.376728	35.08671
3.416558	3.288641	3.357443	35.18786
3.215002	3.310409	3.393507	34.87356
3.226179	3.294976	3.377632	34.82954
3.27563	3.395595	3.42333	35.25765
3.364633	3.463123	3.331917	35.40005
3.284662	3.351048	3.36631	35.05529
3.20465	3.375309	3.458388	35.13473
3.128061	3.401256	3.449044	35.00355
3.275382	3.393708	3.427576	35.26227
3.263752	3.34505	3.418419	35.1104
3.326689	3.251709	3.388272	34.97799
3.215084	3.178452	3.413304	34.62846
3.300046	3.236276	3.421284	34.95816
3.306026	3.29228	3.368441	34.97815
3.33469	3.35718	3.378433	35.20462
3.197226	3.366413	3.365696	34.89634
3.310893	3.368705	3.316366	35.04205
3.153714	3.424709	3.406978	35.01895
3.297118	3.481926	3.413099	35.47106
3.316584	3.451869	3.431958	35.48914
3.31345	3.400245	3.37047	35.23493
3.31015	3.234254	3.39187	34.91152
3.287219	3.223943	3.372517	34.7965
3.34232	3.293224	3.394752	35.11712

3.33667	3.376051	3.309562	35.0996
3.202876	3.382992	3.395366	35.00983
3.248327	3.413791	3.366361	35.11315
3.320296	3.371266	3.376183	35.19902
3.248327	3.370794	3.340187	34.96188
3.2176	3.395528	3.420994	35.12549
3.160849	3.391888	3.487018	35.13781
3.174625	3.409276	3.403755	35.02388
3.238758	3.206219	3.408393	34.73022
3.31778	3.193818	3.341312	34.72921
3.206093	3.210532	3.403619	34.65777
3.305902	3.28116	3.405597	35.03482
3.227292	3.320451	3.463299	35.07502
3.418084	3.455441	3.311267	35.45498
3.257194	3.377736	3.440825	35.21654
3.353992	3.397212	3.330621	35.22981
3.144146	3.438457	3.408564	35.03156
3.301696	3.472963	3.346888	35.31668
3.193102	3.406176	3.397872	35.04464
3.336959	3.249283	3.319725	34.84524
3.199288	3.176228	3.436886	34.64063
3.338113	3.281093	3.371221	35.02994
3.307511	3.308724	3.372568	35.02639
3.331968	3.341006	3.376541	35.15915
3.28891	3.39856	3.388954	35.218
3.23253	3.346465	3.409467	35.02564
3.192524	3.339052	3.36822	34.83174
3.243996	3.44021	3.416851	35.27187
3.200608	3.468313	3.431515	35.27051
3.227292	3.406108	3.417106	35.16132
3.20465	3.283654	3.436954	34.88742
3.259132	3.1736	3.401214	34.68774
3.382285	3.235737	3.348985	34.97872
3.287013	3.273478	3.37117	34.90142
3.281404	3.268221	3.394411	34.92849
3.274805	3.352733	3.404215	35.12031
3.304913	3.38589	3.409058	35.26925
3.296169	3.374838	3.287907	34.96102
3.345042	3.409276	3.3895	35.36538
3.128514	3.456788	3.454023	35.13687
3.307057	3.436435	3.408734	35.38377
3.257441	3.371401	3.379133	35.06831
3.253482	3.254944	3.383089	34.81363
3.228159	3.295583	3.408308	34.90227
3.237232	3.299828	3.413236	34.94218
3.267299	3.418913	3.373045	35.18046
3.348466	3.388451	3.321754	35.17918
3.250512	3.33858	3.418215	35.06685
3.286683	3.365942	3.355584	35.06882
3.238346	3.359337	3.386925	35.01721

3.249275	3.463865	3.440979	35.3879
3.151116	3.429898	3.389346	34.98606
3.31877	3.404895	3.380156	35.27791
3.202546	3.273612	3.48313	34.96184
3.267505	3.223808	3.417447	34.85135
3.200525	3.252248	3.396389	34.72102
3.374861	3.274825	3.376336	35.10778
3.340547	3.305826	3.422256	35.20095
3.276537	3.349767	3.342028	34.98162
3.265319	3.371738	3.416152	35.16723
3.250306	3.372277	3.326324	34.93914
3.297942	3.404154	3.460639	35.40675
3.278434	3.466897	3.419442	35.41121
3.253812	3.323821	3.447884	35.10667
3.235871	3.275499	3.381724	34.81709
3.355394	3.260403	3.365355	35.00966
3.257648	3.367424	3.393643	35.0918
3.337206	3.395662	3.384708	35.30799
3.312584	3.362168	3.374273	35.15808
3.318894	3.352126	3.382594	35.16812
3.339681	3.337232	3.35729	35.12567
3.361334	3.405839	3.397668	35.41135
3.122905	3.435829	3.437159	35.04189
3.286765	3.422822	3.391	35.27084
3.137217	3.334536	3.508315	35.00728
3.230881	3.325438	3.410644	34.97862
3.206093	3.21808	3.407302	34.68234
3.290024	3.264379	3.400686	34.95266
3.143651	3.24652	3.499721	34.81008
3.405959	3.353272	3.374222	35.34271
3.329246	3.393708	3.450596	35.4304
3.388389	3.435088	3.320049	35.36474
3.236613	3.351519	3.43484	35.10111
3.16984	3.394854	3.3223	34.80375
3.338113	3.457799	3.40928	35.4996
3.377831	3.467908	3.277471	35.32031
3.21793	3.246992	3.505468	34.98612
3.202629	3.219023	3.415197	34.69409
3.263133	3.258584	3.419903	34.9232
3.438995	3.411635	3.394257	35.5864
3.390658	3.434885	3.412946	35.57241
3.27398	3.326179	3.435471	35.12879
3.241398	3.294235	3.362695	34.82853
3.317739	3.354957	3.415606	35.24397
3.299551	3.486442	3.308403	35.2573
3.282187	3.416015	3.457911	35.39227
3.133546	3.339321	3.473718	34.93406
3.170418	3.241196	3.4877	34.83069
3.341702	3.270243	3.368475	35.00805
3.218796	3.23048	3.398264	34.71747

3.286436	3.252518	3.406074	34.93066
3.18675	3.298885	3.45728	34.92604
3.326483	3.352935	3.401095	35.22694
3.454791	3.396404	3.294813	35.37017
3.343516	3.393101	3.43779	35.43227
3.187781	3.37039	3.452642	35.07452
3.271877	3.435425	3.430833	35.35295
3.220611	3.422822	3.34017	35.01501
3.328298	3.345791	3.374495	35.15712
3.173511	3.251103	3.379576	34.62267
3.132763	3.293561	3.458303	34.79857
3.222302	3.390743	3.399509	35.07833
3.447986	3.409141	3.357494	35.52021
3.304665	3.334806	3.440177	35.22505
3.375233	3.323551	3.331235	35.11652
3.220364	3.327392	3.412144	34.96318
3.326112	3.462719	3.342438	35.33794
3.303716	3.444725	3.441661	35.4666
3.284291	3.342489	3.333093	34.96312
3.122699	3.225224	3.443792	34.59539
3.188524	3.326314	3.411514	34.88981
3.293117	3.324427	3.477196	35.25805
3.238676	3.283924	3.379798	34.83743
3.25971	3.276712	3.4648	35.05354
3.191493	3.340062	3.432521	34.97231
3.308831	3.356035	3.399168	35.1909
3.391895	3.372614	3.34167	35.28307
3.254431	3.359607	3.413781	35.11171
3.244202	3.389125	3.366566	35.05064
3.231705	3.450656	3.376745	35.18013
3.268701	3.308118	3.405597	35.01242
3.30219	3.290596	3.286645	34.78721
3.177512	3.194964	3.402169	34.55806
3.255338	3.444792	3.398264	35.26605
3.241851	3.431314	3.473121	35.37078
3.33799	3.4053	3.298837	35.143
3.349208	3.296257	3.386397	35.12055
3.382038	3.319305	3.386414	35.24278
3.247089	3.421744	3.440177	35.28926
3.371356	3.429898	3.342557	35.36536
3.19867	3.317957	3.384112	34.8338
3.329411	3.245442	3.38684	34.9671
3.129298	3.314925	3.438625	34.79468
3.110326	3.343971	3.44369	34.82779
3.200525	3.272467	3.437108	34.85428
3.249894	3.281565	3.401095	34.90338
3.321864	3.339726	3.429742	35.2506
3.37515	3.398493	3.455916	35.55288
3.302892	3.41588	3.43397	35.3849
3.325287	3.381712	3.363258	35.20451

3.28957	3.353541	3.329683	34.99138
3.275836	3.429764	3.402988	35.28834
3.272413	3.317823	3.288043	34.78469
3.303098	3.226504	3.413764	34.92702
3.198793	3.240657	3.318003	34.52046
3.231994	3.298952	3.397395	34.89417
3.308583	3.478422	3.435676	35.53784
3.270763	3.407591	3.383617	35.18639
3.299386	3.341815	3.432197	35.21138
3.230674	3.295852	3.372022	34.82901
3.430581	3.484757	3.415606	35.77459
3.258225	3.456721	3.410303	35.32477
3.279589	3.380499	3.451619	35.29516
3.194999	3.277454	3.378263	34.72441
3.284002	3.267682	3.351782	34.83977
3.222343	3.291943	3.39291	34.84793
3.180234	3.323955	3.405239	34.85281
3.130123	3.260807	3.433408	34.66673
3.304005	3.270714	3.372841	34.93619
3.308088	3.363852	3.476309	35.37507
3.412805	3.401121	3.36752	35.44767
3.274104	3.3621	3.428531	35.19244
3.308831	3.337097	3.345831	35.03285
3.340093	3.34633	3.299536	35.02017
3.423487	3.443377	3.33369	35.48945
3.226839	3.280149	3.424422	34.90088
3.178955	3.243285	3.370573	34.59779
3.215456	3.258853	3.400038	34.77609
3.251255	3.406715	3.346752	35.0612
3.396679	3.382992	3.369106	35.37623
3.151652	3.36547	3.488467	35.06309
3.252698	3.253529	3.368799	34.77758
3.275052	3.357046	3.395127	35.11041
3.448522	3.51596	3.379116	35.80227
3.332381	3.454362	3.425496	35.515
3.233355	3.252518	3.468824	34.9518
3.208774	3.20979	3.379849	34.61003
3.349249	3.352059	3.369703	35.20616
3.262803	3.399302	3.414054	35.21742
3.207949	3.326786	3.425564	34.96405
3.117956	3.270445	3.394257	34.57558
3.309326	3.285204	3.423245	35.08974
3.38113	3.386699	3.37209	35.35685
3.357663	3.368031	3.406961	35.34097
3.250059	3.362033	3.463572	35.21634
3.331886	3.40321	3.401828	35.3503
3.267422	3.397954	3.438881	35.27887
3.428478	3.349767	3.340238	35.30998
3.285446	3.226437	3.350622	34.75019
3.143362	3.230346	3.384112	34.52126

3.141547	3.36857	3.417635	34.89288
3.278599	3.491833	3.389091	35.39972
3.314069	3.407861	3.399066	35.31547
3.290849	3.287563	3.376933	34.95322
3.166005	3.307983	3.433408	34.84836
3.34298	3.428011	3.378723	35.37827
3.451492	3.50302	3.420056	35.86999
3.276867	3.347611	3.316656	34.92214
3.193143	3.194627	3.452523	34.70162
3.246924	3.263773	3.380821	34.81364
3.260535	3.374029	3.435676	35.20447
3.312048	3.387575	3.349531	35.15836
3.241893	3.307444	3.46069	35.0728
3.076672	3.283317	3.460639	34.65861
3.31048	3.374635	3.35833	35.14588
3.317574	3.399032	3.426672	35.3642
3.351476	3.454969	3.400549	35.50354
3.207578	3.366481	3.465021	35.13633
3.322152	3.364459	3.436835	35.32083
3.269649	3.331638	3.3356	34.91286
3.424848	3.264177	3.322027	35.07504
3.161509	3.229267	3.429128	34.65703
3.218301	3.362707	3.361996	34.92623
3.129051	3.421339	3.426604	35.00056
3.306933	3.402806	3.284667	35.03864
3.305944	3.303468	3.393507	35.05725
3.226921	3.334334	3.344364	34.84448
3.206629	3.399706	3.453597	35.18193
3.305284	3.520206	3.433629	35.61752
3.357457	3.398695	3.408103	35.41007
3.359725	3.22583	3.32953	34.86518
3.201103	3.185259	3.384436	34.54964
3.187163	3.303603	3.4242	34.86491
3.187658	3.451936	3.387249	35.10957
3.213888	3.343365	3.440314	35.04555
3.183822	3.26849	3.384981	34.69506
3.216115	3.307848	3.488535	35.07821
3.234015	3.398628	3.429861	35.18756
3.420642	3.422148	3.399441	35.58059
3.391194	3.371333	3.337885	35.27046
3.250512	3.37841	3.427678	35.17465
3.349744	3.339321	3.307942	35.04433
3.325782	3.217541	3.365764	34.85206
3.374201	3.240589	3.323118	34.91509
3.278764	3.313981	3.381639	34.99486
3.234593	3.416824	3.402186	35.16809
3.190008	3.388316	3.351782	34.89803
3.334072	3.388384	3.397344	35.31285
3.259833	3.342826	3.353675	34.95538
3.250719	3.407995	3.395366	35.16914

3.191369	3.499314	3.43322	35.32183
3.390534	3.437109	3.451892	35.66217
3.309243	3.254809	3.37579	34.91932
3.285776	3.178587	3.393337	34.73968
3.232654	3.24989	3.43941	34.8802
3.289199	3.429561	3.425769	35.36693
3.17879	3.387777	3.405665	34.99015
3.238181	3.323888	3.322112	34.79759
3.191039	3.316812	3.449931	34.95855
3.298396	3.397954	3.363923	35.18268
3.279012	3.369851	3.425632	35.21378
3.296623	3.425855	3.36145	35.23441
3.274681	3.361965	3.445685	35.23092
3.374408	3.329212	3.291351	35.03987
3.381213	3.256629	3.362166	35.05089
3.231211	3.295246	3.320134	34.71539
3.291632	3.35408	3.449897	35.25995
3.162045	3.447892	3.374426	35.01668
3.27897	3.407658	3.428088	35.30174
3.301613	3.384273	3.407762	35.25567
3.217724	3.329145	3.419084	34.97642
3.184564	3.350104	3.444611	35.00556
3.27431	3.471345	3.358006	35.27756
3.30017	3.486239	3.441507	35.54929
3.348136	3.253799	3.371272	34.99228
3.198216	3.175015	3.443383	34.64984
3.332711	3.196514	3.352225	34.79162
3.297984	3.327392	3.41315	35.13512
3.367438	3.470874	3.433067	35.64433
3.163613	3.311218	3.460639	34.90975
3.288126	3.268962	3.335259	34.81545
3.127483	3.263773	3.445003	34.6928
3.340176	3.407119	3.369294	35.30583
3.174088	3.434953	3.52182	35.33705
3.26635	3.419385	3.43085	35.30582
3.263174	3.295583	3.41385	34.99097
3.389297	3.282037	3.329615	35.05295
3.358529	3.279543	3.374205	35.07772
3.323431	3.267749	3.410831	35.05527
3.161138	3.426326	3.444542	35.12087
3.310522	3.482263	3.414856	35.50495
3.268453	3.438996	3.320254	35.11146
3.348424	3.340669	3.342284	35.11949
3.164314	3.331908	3.461782	34.95903
3.216734	3.433672	3.400618	35.16245
3.318482	3.495809	3.460895	35.65266
3.372057	3.373894	3.377086	35.31994
3.328628	3.140308	3.360495	34.67786
3.239913	3.149204	3.427013	34.64878
3.264494	3.353272	3.411445	35.11475

3.254678	3.492372	3.423467	35.42377
3.377377	3.359404	3.347604	35.23541
3.126122	3.231356	3.414054	34.55125
3.184853	3.251035	3.369498	34.62528
3.227334	3.404423	3.409416	35.14091
3.283631	3.497899	3.392143	35.43067
3.251296	3.458675	3.377888	35.24301
3.301737	3.319103	3.430543	35.16324
3.235335	3.212756	3.339692	34.58679
3.406866	3.250429	3.319998	35.00122
3.389585	3.317351	3.345694	35.16597
3.239872	3.426933	3.422682	35.24657
3.235211	3.412848	3.389415	35.13282
3.38575	3.394382	3.325795	35.28252
3.289694	3.31142	3.35995	34.96572
3.314481	3.415004	3.398486	35.33073
3.211785	3.380499	3.425513	35.08979
3.277073	3.480511	3.522212	35.66274
3.18807	3.404963	3.429725	35.10064
3.351023	3.181822	3.351083	34.79704
3.278393	3.188696	3.420926	34.80598
3.387358	3.323147	3.392279	35.27564
3.232035	3.449308	3.483266	35.41085
3.340671	3.377601	3.283252	35.0542
3.346321	3.224348	3.338618	34.8525
3.275382	3.250564	3.324397	34.7236
3.163406	3.315868	3.505451	35.01747
3.25839	3.44641	3.380616	35.23766
3.243584	3.424844	3.422631	35.25
3.346527	3.365739	3.350009	35.18706
3.357168	3.233513	3.392961	35.0151
3.282765	3.230817	3.379866	34.81786
3.310769	3.295785	3.355516	34.96793
3.273444	3.358596	3.392211	35.1039
3.348424	3.392967	3.423177	35.41076
3.262886	3.382453	3.354783	35.05114
3.244532	3.30677	3.398435	34.94096
3.25608	3.356237	3.339556	34.94563
3.337536	3.425181	3.36382	35.32759
3.1778	3.453284	3.449385	35.22685
3.34166	3.397617	3.355857	35.25892
3.173965	3.197929	3.467664	34.70001
3.345785	3.191459	3.329615	34.75971
3.326772	3.257033	3.445071	35.11402
3.339516	3.435425	3.324499	35.26833
3.271299	3.429629	3.50748	35.50663
3.273609	3.350643	3.406466	35.11805
3.271505	3.194357	3.344859	34.63695
3.327266	3.258988	3.29824	34.79828
3.208403	3.41743	3.442275	35.19981

3.097582	3.520341	3.458525	35.21805
3.252657	3.428955	3.449999	35.33868
3.30615	3.22765	3.339078	34.77288
3.360055	3.170433	3.378706	34.85229
3.274062	3.299222	3.433817	35.0664
3.226921	3.375377	3.38227	35.01713
3.368056	3.417902	3.350213	35.34866
3.415239	3.413859	3.360239	35.46492
3.34397	3.309668	3.37475	35.11295
3.219126	3.318766	3.335276	34.77351
3.224942	3.426663	3.459906	35.29473
3.277898	3.474109	3.403209	35.39031
3.281569	3.449779	3.357307	35.24475
3.306521	3.228593	3.435454	34.98651
3.119647	3.164839	3.438063	34.44413
3.262473	3.271793	3.438745	34.99185
3.268742	3.461304	3.404556	35.34523
3.369665	3.451869	3.463555	35.67431
3.239294	3.330156	3.396986	34.97747
3.258967	3.153786	3.426877	34.70017
3.289034	3.287563	3.347587	34.88507
3.366365	3.446747	3.346803	35.40058
3.187534	3.481859	3.382799	35.16501
3.223746	3.422013	3.465465	35.2941
3.171119	3.215789	3.377018	34.53462
3.384182	3.180609	3.349292	34.86299
3.30615	3.24679	3.451414	35.06039
3.34562	3.477613	3.449538	35.64738
3.25839	3.462921	3.451704	35.42923
3.423199	3.44021	3.346581	35.51009
3.316914	3.336491	3.364946	35.091
3.426457	3.342623	3.252047	35.09708
3.149425	3.385755	3.463538	35.04807
3.134825	3.492642	3.363701	35.03156
3.249605	3.447151	3.382321	35.2238
3.231169	3.287832	3.358995	34.78407
3.222962	3.174207	3.433953	34.68156
3.2176	3.208443	3.440518	34.75906
3.25872	3.42727	3.452437	35.35359
3.366984	3.45079	3.415145	35.56023
3.35358	3.381173	3.381452	35.30499
3.284538	3.266738	3.407779	34.96133
3.285899	3.286282	3.366514	34.91681
3.202959	3.381712	3.365611	34.94215
3.314275	3.448634	3.354476	35.30757
3.258101	3.440681	3.413764	35.29699
3.275341	3.313981	3.382594	34.98946
3.2501	3.183911	3.393285	34.6732
3.334072	3.223404	3.335071	34.81589
3.278847	3.367694	3.453392	35.26941

3.26866	3.429022	3.379525	35.21971
3.240903	3.38926	3.469284	35.26835
3.419033	3.3652	3.368066	35.38393
3.320626	3.333795	3.343512	35.04635
3.33799	3.45234	3.374631	35.41162
3.259462	3.456654	3.387573	35.27762
3.200525	3.434885	3.481152	35.30578
3.099067	3.316812	3.333895	34.50367
3.293158	3.200153	3.393354	34.80303
3.222302	3.202647	3.385271	34.63585
3.236077	3.321934	3.457911	35.08569
3.258679	3.420733	3.408376	35.24285
3.372634	3.363044	3.446776	35.44987
3.388884	3.338175	3.371272	35.26591
3.373872	3.278599	3.349309	35.05477
3.255173	3.304142	3.38077	34.91985
3.20168	3.413319	3.411514	35.10885
3.257854	3.469391	3.435659	35.40712
3.240202	3.455643	3.312546	35.06922
3.325328	3.210936	3.393661	34.89763
3.190668	3.18627	3.419954	34.60671
3.234304	3.280756	3.448891	34.97204
3.269237	3.427203	3.443229	35.35631
3.353621	3.432392	3.373813	35.40038
3.317368	3.412713	3.374273	35.27908
3.351311	3.340939	3.385049	35.21991
3.285858	3.406108	3.376166	35.19987
3.389585	3.436233	3.335822	35.40435
3.279259	3.475793	3.393405	35.37552
3.276455	3.403278	3.387454	35.1978
3.15359	3.206151	3.398554	34.5223
3.272866	3.188157	3.403192	34.75393
3.225436	3.325842	3.372653	34.88452
3.276537	3.494798	3.446657	35.52759
3.251049	3.39054	3.438404	35.2258
3.396473	3.360348	3.403141	35.40068
3.280909	3.248946	3.377598	34.84849
3.386327	3.241937	3.371545	35.05045
3.252822	3.387912	3.39864	35.13698
3.218219	3.383599	3.379405	35.00981
3.166788	3.391484	3.428838	35.02268
3.294519	3.3065	3.365219	34.97704
3.281033	3.244431	3.43866	34.97242
3.200938	3.268423	3.372312	34.70464
3.164437	3.346937	3.538377	35.15967
3.340629	3.473232	3.414463	35.55018
3.509315	3.405502	3.352805	35.63612
3.37746	3.39364	3.343461	35.3014
3.335886	3.326921	3.451227	35.30025
3.275506	3.473165	3.36249	35.29396

3.296045	3.443579	3.392757	35.34037
3.362158	3.355698	3.307567	35.10647
3.33168	3.21316	3.408598	34.94905
3.071434	3.174409	3.428497	34.3387
3.286972	3.299424	3.411855	35.04705
3.271629	3.461169	3.440586	35.43004
3.270516	3.452475	3.446435	35.42138
3.21958	3.348487	3.440501	35.06961
3.274186	3.260605	3.398964	34.90601
3.30318	3.285743	3.394598	35.01484
3.406165	3.402671	3.329325	35.35301
3.206794	3.418778	3.452062	35.22065
3.278558	3.396067	3.336708	35.07566
3.190833	3.223202	3.389142	34.62045
3.268907	3.241735	3.320595	34.68181
3.203371	3.219091	3.399987	34.6626
3.223539	3.375983	3.327688	34.89169
3.174212	3.438794	3.513277	35.32703
3.385667	3.444658	3.350196	35.44564
3.346527	3.318025	3.41888	35.23332
3.489436	3.397482	3.405051	35.68936
3.306975	3.397482	3.420926	35.32506
3.350899	3.40375	3.396525	35.38146
3.258225	3.371064	3.368151	35.04527
3.267464	3.207432	3.414174	34.80828
3.225601	3.206556	3.357102	34.59002
3.201557	3.280082	3.454159	34.91047
3.233685	3.448229	3.421762	35.2776
3.330318	3.448432	3.507139	35.67607
3.303139	3.404019	3.406944	35.30039
3.27233	3.293291	3.414054	35.00643
3.352013	3.311622	3.331866	35.04103
3.267464	3.348554	3.36943	35.01905
3.31543	3.364661	3.384862	35.19291
3.342362	3.3683	3.383157	35.25604
3.294684	3.31607	3.365474	34.99889
3.200113	3.252248	3.387641	34.70098
3.287343	3.253394	3.374358	34.8652
3.187204	3.296863	3.424063	34.84997
3.231169	3.402065	3.397821	35.11878
3.212651	3.401593	3.389091	35.05816
3.358364	3.376051	3.416919	35.38181
3.327596	3.380094	3.288128	35.04173
3.430045	3.388856	3.420449	35.57429
3.319884	3.430437	3.376797	35.32886
3.380223	3.413589	3.436341	35.55418
3.245646	3.229537	3.315974	34.59416
3.24845	3.17542	3.398691	34.66284
3.251502	3.250361	3.426331	34.89385
3.246058	3.460899	3.415367	35.31838

3.232943	3.476535	3.456052	35.41286
3.257153	3.418778	3.362882	35.13575
3.288209	3.33703	3.491247	35.30561
3.30714	3.375983	3.354647	35.13347
3.308583	3.337973	3.451823	35.26601
3.277444	3.381577	3.365901	35.10537
3.223581	3.328471	3.386158	34.91575
3.224488	3.341613	3.349804	34.86697
3.334855	3.248946	3.350946	34.90818
3.1851	3.216665	3.35347	34.51561
3.168479	3.207971	3.410303	34.58454
3.176192	3.369581	3.383549	34.89631
3.289529	3.45106	3.422017	35.40647
3.314687	3.437446	3.355823	35.28696
3.379481	3.385486	3.440979	35.50124
3.221601	3.384003	3.393712	35.04938
3.414909	3.468178	3.36486	35.59309
3.285033	3.396269	3.419971	35.27234
3.385585	3.35927	3.333912	35.22312
3.287549	3.166928	3.403533	34.74036
3.247584	3.155269	3.301906	34.40523
3.139815	3.295785	3.412878	34.71952
3.311017	3.435627	3.315377	35.1865
3.175449	3.434885	3.519501	35.3348
3.242264	3.433942	3.351935	35.11241
3.216074	3.293426	3.451687	34.966
3.428437	3.406647	3.35422	35.46485
3.29254	3.336962	3.401436	35.11853
3.235995	3.358596	3.393746	35.02537
3.207826	3.322136	3.437023	34.97867
3.34496	3.277117	3.315667	34.91473
3.322606	3.18809	3.353334	34.75353
3.256204	3.224213	3.336879	34.65133
3.123277	3.335682	3.448686	34.84891
3.147445	3.406378	3.450817	35.06102
3.344547	3.487992	3.375108	35.50496
3.369665	3.400852	3.394598	35.41195
3.387482	3.392967	3.381912	35.40593
3.29885	3.413185	3.416987	35.33302
3.295427	3.445062	3.365781	35.28327
3.396514	3.40321	3.39673	35.48049
3.426127	3.259797	3.328711	35.08288
3.140269	3.087471	3.425905	34.29345
3.305696	3.235535	3.383072	34.88533
3.18242	3.435357	3.416373	35.12555
3.327431	3.445938	3.349855	35.32034
3.194586	3.404154	3.404625	35.05823
3.215208	3.337973	3.445361	35.04768
3.163778	3.319575	3.440927	34.88528
3.445676	3.421407	3.3799	35.59098

3.252121	3.340736	3.453477	35.1522
3.327349	3.31607	3.452642	35.26094
3.224612	3.309735	3.463555	35.04628
3.38146	3.202579	3.355772	34.91925
3.287962	3.216125	3.362286	34.75865
3.274392	3.291269	3.416578	35.01203
3.158705	3.343971	3.406466	34.85218
3.266927	3.451195	3.447254	35.41252
3.268742	3.474917	3.341056	35.23613
3.401216	3.418711	3.327296	35.37282
3.31477	3.379151	3.347348	35.14112
3.245976	3.385014	3.445361	35.21784
3.295344	3.435492	3.427525	35.39718
3.318358	3.271725	3.31507	34.84346
3.275753	3.139903	3.368066	34.57791
3.227952	3.178048	3.380002	34.58289
3.21261	3.345724	3.477213	35.12861
3.245275	3.502347	3.399253	35.37206
3.31048	3.428213	3.355312	35.25645
3.261442	3.335075	3.413969	35.0738
3.266721	3.37659	3.466829	35.29173
3.176027	3.362505	3.390404	34.89546
3.3191	3.370255	3.393013	35.231
3.338567	3.286282	3.408751	35.12435
3.277609	3.276173	3.44045	35.03826
3.226921	3.192403	3.371323	34.59305
3.397504	3.213295	3.381571	35.03419
3.236778	3.286552	3.351935	34.7781
3.286436	3.310409	3.437023	35.12493
3.206794	3.447758	3.474144	35.33231
3.265566	3.444388	3.452778	35.40674
3.228076	3.447825	3.388869	35.19251
3.328999	3.415678	3.38464	35.33367
3.251956	3.391214	3.444747	35.24313
3.388925	3.406715	3.350827	35.37117
3.272371	3.36237	3.457143	35.25181
3.330813	3.242072	3.321532	34.81998
3.380594	3.135253	3.331729	34.71755
3.231252	3.283991	3.387386	34.83794
3.170253	3.40321	3.450221	35.10266
3.258637	3.474041	3.408223	35.359
3.276537	3.386834	3.39482	35.17813
3.332876	3.347611	3.347962	35.11308
3.265195	3.366683	3.499312	35.33776
3.154869	3.389125	3.43484	35.00459
3.25938	3.344241	3.450067	35.16828
3.257111	3.290259	3.375518	34.88224
3.379274	3.310477	3.335412	35.1059
3.247254	3.188831	3.367248	34.62079
3.300747	3.244903	3.40367	34.94004

3.197597	3.302187	3.371579	34.76956
3.284992	3.30495	3.367333	34.95744
3.157302	3.41642	3.422972	35.04364
3.289735	3.499651	3.379474	35.42014
3.201392	3.396943	3.424677	35.1012
3.33535	3.435492	3.332667	35.27723
3.320338	3.358057	3.412997	35.25073
3.35956	3.3683	3.29176	35.09378
3.250595	3.29228	3.389892	34.90385
3.277114	3.212014	3.31657	34.62597
3.266061	3.231693	3.378433	34.78012
3.374779	3.385284	3.323118	35.23278
3.152064	3.424709	3.443212	35.09458
3.248038	3.430303	3.356676	35.12745
3.271217	3.296594	3.424882	35.03489
3.284208	3.390001	3.349258	35.10219
3.287425	3.406647	3.428872	35.31973
3.163283	3.386969	3.508162	35.17861
3.204774	3.263368	3.412264	34.78934
3.303428	3.21936	3.394513	34.87002
3.34166	3.313442	3.379116	35.1257
3.270763	3.243285	3.381298	34.82201
3.31576	3.311825	3.361706	35.02745
3.172562	3.341208	3.491264	35.06188
3.33535	3.400986	3.313723	35.16034
3.284208	3.452475	3.395553	35.34005
3.359354	3.420665	3.332718	35.29741
3.201722	3.410152	3.454483	35.19598
3.282146	3.393438	3.354357	35.11635
3.275011	3.343904	3.422972	35.14247
3.425426	3.362168	3.362746	35.37964
3.20898	3.2354	3.47133	34.86655
3.293777	3.226976	3.389551	34.85472
3.284745	3.255079	3.378757	34.87282
3.318399	3.434953	3.327586	35.22787
3.192895	3.434144	3.386499	35.08048
3.113543	3.374972	3.424302	34.86022
3.203701	3.371064	3.361399	34.91127
3.277156	3.408265	3.400413	35.23858
3.248574	3.439805	3.424353	35.2974
3.238717	3.332447	3.396798	34.98081
3.201515	3.231896	3.402988	34.6931
3.284002	3.238635	3.41315	34.91045
3.396927	3.236344	3.351356	35.01725
3.277238	3.254675	3.351441	34.79579
3.268123	3.278936	3.385118	34.90256
3.187781	3.331773	3.422222	34.92355
3.306727	3.406378	3.340937	35.16905
3.321327	3.416757	3.358534	35.26216
3.359065	3.455508	3.444133	35.61662

3.145548	3.398425	3.414634	34.96035
3.286683	3.375242	3.381094	35.14495
3.275052	3.384542	3.392501	35.1648
3.408145	3.314588	3.371016	35.25589
3.210053	3.210532	3.390847	34.6385
3.267835	3.182429	3.430492	34.7901
3.253358	3.364324	3.372909	35.0303
3.331721	3.41642	3.37504	35.32025
3.243542	3.393304	3.383208	35.09473
3.213146	3.374501	3.391529	35.00533
3.125792	3.354552	3.402732	34.79518
3.233149	3.445668	3.404983	35.23413
3.30681	3.46501	3.398077	35.42241
3.268948	3.32564	3.434431	35.11433
3.249481	3.23695	3.41992	34.84608
3.218961	3.197997	3.395911	34.64165
3.404062	3.261077	3.340255	35.06267
3.339103	3.259392	3.357614	34.95489
3.216528	3.290663	3.42183	34.89565
3.184688	3.354485	3.386141	34.88755
3.328792	3.392427	3.380565	35.27346
3.305242	3.42754	3.33132	35.19105
3.34562	3.434481	3.468739	35.59504
3.136474	3.431044	3.472626	35.13866
3.238263	3.374029	3.382202	35.03883
3.292168	3.312633	3.368185	34.9918
3.378326	3.265592	3.379337	35.10173
3.22853	3.230885	3.347707	34.62908
3.262514	3.225628	3.441541	34.8971
3.247708	3.407658	3.430151	35.23788
3.432478	3.418644	3.34358	35.47665
3.263834	3.355698	3.395434	35.0836
3.270639	3.375849	3.411104	35.17682
3.167489	3.362707	3.382901	34.86083
3.269649	3.454295	3.411991	35.34814
3.224117	3.450116	3.459616	35.34358
3.303675	3.305826	3.388937	35.04746
3.24346	3.25272	3.40974	34.84514
3.362076	3.29383	3.383003	35.13596
3.330112	3.255214	3.399322	35.0173
3.365087	3.296594	3.380838	35.14385
3.280703	3.332582	3.403977	35.08862
3.166995	3.344376	3.386908	34.82842
3.23748	3.435155	3.380292	35.16661
3.28029	3.463326	3.362405	35.28272
3.313409	3.430774	3.389619	35.34348
3.237974	3.399841	3.431379	35.20219
3.202794	3.272534	3.419596	34.82109
3.261318	3.264784	3.37903	34.84341
3.42489	3.280419	3.340579	35.15122

3.246512	3.283452	3.402664	34.90354
3.308501	3.336221	3.410013	35.17057
3.253936	3.448566	3.381076	35.23365
3.328916	3.408534	3.376609	35.3003
3.294066	3.369109	3.333571	35.04376
3.284868	3.400986	3.415265	35.272
3.093416	3.373894	3.397975	34.75627
3.299262	3.421609	3.401095	35.31759
3.176192	3.309668	3.42038	34.84583
3.262349	3.245981	3.402988	34.85694
3.268247	3.180811	3.445548	34.82039
3.295757	3.262762	3.42275	35.00991
3.283878	3.323753	3.456751	35.19167
3.325493	3.364257	3.381571	35.20684
3.281693	3.324629	3.394888	35.05352
3.315966	3.374433	3.310517	35.05288
3.165345	3.389529	3.392348	34.93546
3.284497	3.479904	3.313791	35.22187
3.288621	3.448364	3.39291	35.33493
3.283755	3.411028	3.371971	35.19685
3.202588	3.265458	3.459957	34.89342
3.278311	3.276645	3.344467	34.83093
3.29254	3.318497	3.400396	35.07587
3.412104	3.357315	3.386567	35.39199
3.337041	3.388384	3.400259	35.32572
3.298809	3.384542	3.349037	35.1217
3.292622	3.349363	3.412673	35.1704
3.212981	3.396943	3.349173	34.96142
3.338938	3.443781	3.39482	35.43912
3.214012	3.422148	3.439819	35.21703
3.203866	3.363381	3.427951	35.04037
3.224735	3.197592	3.462549	34.79912
3.272866	3.229739	3.391273	34.8188
3.201515	3.206421	3.43513	34.70768
3.407567	3.231963	3.426365	35.19498
3.238098	3.352059	3.517847	35.28706
3.397916	3.431246	3.322709	35.38299
3.354281	3.395123	3.403909	35.38614
3.312419	3.428213	3.329478	35.20419
3.194463	3.432527	3.461236	35.24381
3.25938	3.49709	3.327364	35.2342
3.188482	3.388653	3.370505	34.93637
3.301819	3.328673	3.334492	34.9743
3.189184	3.255281	3.440109	34.79845
3.251172	3.254675	3.4279	34.90599
3.283219	3.3034	3.423722	35.07349
3.363808	3.342084	3.393865	35.26903
3.337949	3.364526	3.390029	35.25316
3.278146	3.312903	3.409399	35.05185
3.245769	3.383531	3.43808	35.19822

3.256287	3.446208	3.402323	35.28009
3.310563	3.436772	3.37695	35.32266
3.262349	3.467841	3.405989	35.34867
3.264494	3.336086	3.460503	35.18445
3.221312	3.214643	3.455166	34.81277
3.359643	3.269906	3.363991	35.03674
3.284291	3.291809	3.407217	35.01439
3.301366	3.259797	3.3489	34.85419
3.275506	3.304816	3.442769	35.10137
3.33931	3.378881	3.395758	35.30006
3.333494	3.362909	3.361314	35.17709
3.35358	3.4084	3.321856	35.23421
3.179161	3.432459	3.391376	35.05742
3.248615	3.404693	3.416987	35.2046
3.242388	3.358663	3.399236	35.0515
3.256534	3.29383	3.34815	34.82894
3.19702	3.233715	3.389756	34.65831
3.306274	3.32719	3.458934	35.25293
3.208774	3.363448	3.437995	35.07321
3.427612	3.368031	3.372653	35.41891
3.324709	3.320923	3.381588	35.1104
3.334402	3.33703	3.404574	35.21708
3.289488	3.399773	3.377956	35.19786
3.217971	3.437851	3.388136	35.147
3.172934	3.418239	3.354033	34.93105
3.310604	3.352598	3.360922	35.10363
3.172232	3.290798	3.455438	34.87258
3.210011	3.234052	3.405597	34.7221
3.269361	3.240859	3.386703	34.82546
3.357003	3.304276	3.376831	35.13421
3.302232	3.343837	3.412537	35.17903
3.32339	3.372277	3.404437	35.26978
3.28891	3.351654	3.38285	35.10208
3.287219	3.366413	3.343188	35.04392
3.407567	3.417902	3.345882	35.42559
3.323101	3.394786	3.372312	35.24812
3.241315	3.377533	3.367862	35.02181
3.167489	3.297604	3.339147	34.62278
3.300128	3.234052	3.366685	34.83408
3.252286	3.336962	3.330143	34.8746
3.366984	3.389597	3.428719	35.45609
3.287302	3.41925	3.428002	35.34512
3.379852	3.375781	3.411889	35.41722
3.318729	3.328134	3.35156	35.04742
3.434664	3.38926	3.381025	35.49906
3.249151	3.448701	3.476378	35.43189
3.224117	3.382655	3.390029	35.04388
3.195246	3.300368	3.453801	34.94025
3.228653	3.287563	3.311796	34.67476
3.25674	3.24652	3.431242	34.90764

3.207578	3.24652	3.393507	34.71761
3.126246	3.215249	3.530056	34.76998
3.353868	3.344443	3.361263	35.18115
3.363478	3.381914	3.442002	35.46067
3.46436	3.412443	3.297234	35.43146
3.322111	3.339321	3.440092	35.27289
3.294519	3.37012	3.397531	35.18683
3.371438	3.464	3.381793	35.52592
3.354735	3.460428	3.326682	35.36106
3.239047	3.302389	3.3725	34.86266
3.177965	3.175487	3.430287	34.57795
3.249605	3.27341	3.376251	34.83058
3.254224	3.332312	3.428787	35.08438
3.368262	3.440479	3.415145	35.54048
3.207867	3.327325	3.469301	35.0607
3.261318	3.361965	3.408598	35.12059
3.302232	3.369379	3.422989	35.25775
3.455698	3.464606	3.368237	35.68187
3.291343	3.425585	3.517574	35.56369
3.145548	3.350374	3.39598	34.81448
3.186833	3.242409	3.452932	34.7932
3.319224	3.277723	3.351458	34.93804
3.20135	3.233985	3.402988	34.69731
3.221766	3.248677	3.408734	34.78665
3.150167	3.2941	3.403346	34.71763
3.307635	3.34505	3.418215	35.20592
3.44081	3.437446	3.343597	35.53603
3.413589	3.395797	3.420107	35.55274
3.270598	3.304344	3.405733	35.00861
3.278517	3.420665	3.422427	35.31681
3.246388	3.46865	3.349395	35.19178
3.43087	3.361089	3.320066	35.29585
3.281404	3.270377	3.387692	34.91851
3.153178	3.266536	3.447936	34.76144
3.181554	3.418711	3.445548	35.15106
3.326483	3.401593	3.443639	35.42638
3.348754	3.403884	3.400208	35.38512
3.273196	3.258988	3.42171	34.95005
3.222055	3.296998	3.432947	34.9459
3.374614	3.443849	3.323868	35.36213
3.431571	3.42909	3.410013	35.64279
3.28392	3.358731	3.331371	34.99406
3.205475	3.274893	3.409757	34.81059
3.208279	3.324495	3.390983	34.88414
3.318853	3.319575	3.358654	35.04449
3.233685	3.239646	3.323562	34.60671
3.214136	3.253596	3.421131	34.80783
3.160272	3.320721	3.403874	34.79909
3.347022	3.382992	3.365884	35.26059
3.412022	3.378005	3.33589	35.32623

3.412929	3.378949	3.380139	35.42704
3.236613	3.332514	3.412554	35.01081
3.30813	3.410691	3.470785	35.46551
3.238717	3.391484	3.311472	34.92332
3.338897	3.304209	3.416032	35.1802
3.186874	3.251912	3.387113	34.67014
3.163819	3.302861	3.390011	34.73748
3.15491	3.401728	3.431754	35.02549
3.390328	3.422485	3.331661	35.36681
3.341537	3.338715	3.475286	35.39101
3.244408	3.320855	3.353896	34.87409
3.220487	3.340939	3.468602	35.11654
3.382698	3.510434	3.349719	35.58195
3.443573	3.364998	3.383549	35.47101
3.316626	3.30367	3.378996	35.04932
3.111151	3.275769	3.405324	34.59654
3.262968	3.288776	3.413901	34.97574
3.28359	3.338512	3.373932	35.0422
3.247625	3.261616	3.409416	34.87299
3.288663	3.295044	3.38875	34.99064
3.158828	3.307309	3.439887	34.84536
3.319059	3.402334	3.3858	35.28529
3.312419	3.361494	3.364588	35.13507
3.38843	3.365739	3.432606	35.45932
3.242264	3.352126	3.392501	35.0222
3.422662	3.378814	3.376387	35.43983
3.314728	3.317216	3.375722	35.06764
3.341702	3.273343	3.271537	34.80284
3.121008	3.220304	3.459531	34.61535
3.233561	3.409006	3.396866	35.13711
3.200732	3.448836	3.448703	35.26577
3.278723	3.394719	3.375995	35.15898
3.349909	3.330021	3.417396	35.26371
3.320049	3.394314	3.421216	35.34736
3.263504	3.460832	3.410849	35.3465
3.397133	3.486981	3.418419	35.71246
3.31312	3.229335	3.373966	34.8681
3.350981	3.196716	3.293585	34.70378
3.162829	3.289585	3.380684	34.68588
3.163448	3.331571	3.353453	34.7195
3.263793	3.296796	3.420721	35.01
3.24379	3.279004	3.364366	34.80411
3.242965	3.338445	3.446026	35.11087
3.314357	3.370592	3.397275	35.23068
3.330195	3.392427	3.474621	35.48221
3.335928	3.387373	3.372227	35.25977
3.407031	3.378342	3.339556	35.32407
3.308171	3.408198	3.399185	35.30357
3.401092	3.314453	3.328933	35.14814
3.293694	3.244498	3.382202	34.87679

3.271959	3.261549	3.393916	34.89216
3.200443	3.387575	3.40205	35.02915
3.326813	3.462921	3.419647	35.50876
3.148394	3.390945	3.409058	34.93802
3.307717	3.338445	3.381435	35.11122
3.255214	3.326112	3.444952	35.10834
3.372222	3.503088	3.369226	35.58563
3.30351	3.500864	3.407012	35.51314
3.408598	3.353811	3.394479	35.39396
3.204402	3.163963	3.437926	34.62726
3.342444	3.227178	3.274811	34.71067
3.188235	3.297537	3.417277	34.83886
3.32306	3.398425	3.379269	35.27121
3.111605	3.242274	3.360154	34.42551
3.191081	3.287495	3.372534	34.72527
3.263793	3.391753	3.362422	35.09017
3.396968	3.400582	3.420278	35.52723
3.236737	3.412915	3.401487	35.16271
3.282971	3.387103	3.415998	35.2391
3.322194	3.408871	3.407694	35.35432
3.417053	3.422081	3.306544	35.36945
3.331267	3.255146	3.331064	34.87041
3.112759	3.164098	3.389142	34.32047
3.254142	3.225156	3.370607	34.72264
3.186668	3.392832	3.378928	34.95996
3.351765	3.46191	3.354783	35.41926
3.249358	3.386497	3.476514	35.2966
3.196979	3.321125	3.417328	34.90967
3.219126	3.400245	3.465686	35.23688
3.395442	3.578838	3.400601	35.87068
3.30813	3.467302	3.363701	35.35513
3.31213	3.21963	3.361177	34.81674
3.182832	3.16403	3.384231	34.46282
3.35692	3.242813	3.402561	35.05589
3.302273	3.378612	3.360632	35.14166
3.294354	3.289922	3.463981	35.1564
3.094035	3.270445	3.357443	34.44276
3.295138	3.305894	3.431396	35.12179
3.267546	3.421339	3.392075	35.22792
3.383935	3.395258	3.407285	35.45867
3.160973	3.368503	3.448157	35.00196
3.30747	3.372546	3.433834	35.29984
3.368427	3.422013	3.39823	35.46347
3.411568	3.379218	3.328916	35.31264
3.331597	3.221517	3.349002	34.83682
3.217518	3.180946	3.371272	34.54732
3.06104	3.340534	3.43438	34.69213
3.342939	3.482533	3.391819	35.52605
3.255255	3.448162	3.387368	35.24941
3.29687	3.332986	3.315343	34.93103

3.183616	3.32436	3.457007	34.97429
3.369541	3.520071	3.397668	35.67911
3.392307	3.497562	3.442002	35.77662
3.358034	3.396538	3.288691	35.14548
3.285198	3.164232	3.441047	34.81136
3.170335	3.23695	3.397548	34.62407
3.291508	3.360213	3.397071	35.15757
3.227251	3.404828	3.354425	35.02136
3.309491	3.285474	3.383907	35.00467
3.086157	3.263908	3.485807	34.69195
3.208156	3.326449	3.411991	34.93409
3.289199	3.40011	3.451022	35.35775
3.311924	3.456788	3.417465	35.45801
3.226096	3.369109	3.439597	35.12698
3.332092	3.348891	3.350674	35.1201
3.301696	3.259594	3.412008	34.99248
3.46007	3.302659	3.227834	35.03024
3.28425	3.260336	3.362507	34.8477
3.276867	3.440344	3.37076	35.24325
3.249069	3.405434	3.385612	35.1386
3.389132	3.428281	3.321225	35.35405
3.207949	3.359404	3.393388	34.96502
3.327431	3.32901	3.42877	35.23722
3.206052	3.451195	3.459821	35.30688
3.387482	3.463393	3.469233	35.7509
3.290065	3.332784	3.404028	35.10965
3.32904	3.211138	3.417993	34.9594
3.313986	3.270917	3.394411	35.00563
3.261689	3.309196	3.415469	35.02103
3.265566	3.397347	3.382321	35.14979
3.195947	3.324562	3.41385	34.90733
3.265525	3.275095	3.369857	34.8551
3.252245	3.315464	3.413577	35.00995
3.246141	3.418846	3.425905	35.24964
3.265154	3.388451	3.395843	35.15901
3.336505	3.389799	3.404232	35.33633
3.261772	3.300233	3.438063	35.05102
3.336175	3.345589	3.307925	35.02832
3.329164	3.288304	3.42333	35.14009
3.354363	3.25636	3.329666	34.92051
3.287797	3.304142	3.381162	34.99205
3.339887	3.424979	3.38285	35.3739
3.189101	3.417835	3.331115	34.9154
3.344382	3.373018	3.382679	35.26973
3.172026	3.377264	3.361723	34.85627
3.261195	3.44021	3.384453	35.23863
3.220694	3.48442	3.371357	35.2181
3.329411	3.40658	3.475235	35.51279
3.196442	3.249485	3.376455	34.66245
3.325493	3.199951	3.39453	34.87587

3.327596	3.23385	3.437654	35.0489
3.341784	3.426192	3.38285	35.3807
3.239171	3.385486	3.4663	35.24978
3.180605	3.300839	3.334116	34.64753
3.196566	3.27004	3.408529	34.77781
3.334195	3.358798	3.314183	35.06657
3.226715	3.387238	3.460486	35.21366
3.231417	3.472828	3.375842	35.22601
3.162499	3.411432	3.464254	35.13438
3.450172	3.344106	3.330689	35.32416
3.40468	3.323281	3.362013	35.24763
3.304005	3.216058	3.371715	34.81421
3.221642	3.328134	3.449794	35.04993
3.257524	3.39054	3.372022	35.0948
3.35325	3.381712	3.364417	35.2682
3.325163	3.36547	3.35613	35.15314
3.188565	3.301379	3.375927	34.75755
3.215744	3.342758	3.446043	35.06081
3.314357	3.502144	3.42067	35.56953
3.26037	3.469189	3.416851	35.37105
3.326607	3.263908	3.406688	35.04475
3.174996	3.176431	3.412826	34.53533
3.310398	3.295785	3.404369	35.07395
3.398246	3.430707	3.367742	35.48101
3.32673	3.462517	3.454091	35.58301
3.180894	3.335682	3.418965	34.90991
3.218384	3.280352	3.346377	34.71216
3.212074	3.328538	3.402169	34.92574
3.32042	3.432122	3.316775	35.20246
3.213105	3.355024	3.463555	35.12016
3.124596	3.410017	3.398623	34.90487
3.186255	3.272736	3.489081	34.93732
3.353456	3.274354	3.349207	35.00061
3.387358	3.255079	3.445003	35.24209
3.378037	3.27678	3.388272	35.1451
3.171696	3.328201	3.410507	34.85494
3.326442	3.415004	3.387045	35.33186
3.431736	3.422148	3.300423	35.38832
3.341578	3.378005	3.382543	35.27421
3.135031	3.417633	3.415947	34.98223
3.219456	3.48752	3.397412	35.27915
3.327679	3.470806	3.470069	35.63816
3.359766	3.336558	3.382065	35.2223
3.340547	3.160391	3.407234	34.85006
3.143156	3.188966	3.371613	34.40299
3.267422	3.291876	3.370266	34.89684
3.337701	3.418981	3.347195	35.27803
3.41495	3.409343	3.376046	35.48898
3.178955	3.213497	3.399731	34.59641
3.273774	3.293628	3.376609	34.92843

3.252698	3.35462	3.407234	35.08269
3.325534	3.418104	3.385049	35.33229
3.19603	3.389799	3.429929	35.08533
3.234922	3.369109	3.469165	35.21094
3.180935	3.304748	3.331832	34.65181
3.47863	3.321327	3.399032	35.48603
3.334319	3.290393	3.40754	35.1214
3.369129	3.311285	3.435249	35.30381
3.219044	3.364594	3.419289	35.05727
3.29188	3.388586	3.304566	35.01814
3.321822	3.355967	3.319162	35.04421
3.337	3.358394	3.283337	35.00436
3.166582	3.401256	3.447117	35.08358
3.210176	3.483274	3.384794	35.22198
3.321905	3.46218	3.44207	35.54544
3.354157	3.283182	3.378723	35.086
3.333494	3.172185	3.434226	34.91945
3.203866	3.256225	3.487376	34.93599
3.300417	3.365672	3.397122	35.1891
3.338361	3.44769	3.419835	35.50111
3.283837	3.37194	3.391052	35.15328
3.252822	3.269636	3.362797	34.79994
3.245068	3.293089	3.390182	34.89416
3.30017	3.37841	3.416834	35.25952
3.334154	3.439468	3.399322	35.42907
3.17446	3.441894	3.417737	35.12542
3.336959	3.329886	3.485944	35.385
3.214425	3.228189	3.399322	34.70521
3.409423	3.273612	3.355533	35.13522
3.371768	3.264447	3.397548	35.12471
3.308583	3.393236	3.364997	35.19699
3.24878	3.370861	3.453188	35.21014
3.387647	3.399908	3.350895	35.35364
3.357745	3.345656	3.312751	35.0862
3.324627	3.398425	3.337919	35.18421
3.174501	3.470941	3.401743	35.15406
3.104305	3.471413	3.472422	35.15614
3.182337	3.366616	3.414668	34.97132
3.299221	3.151091	3.404062	34.73241
3.329865	3.175015	3.391324	34.82389
3.351105	3.2879	3.443622	35.23156
3.247749	3.436031	3.402033	35.23853
3.361251	3.408804	3.368407	35.35367
3.280868	3.263705	3.423586	34.98124
3.32739	3.258718	3.344143	34.89834
3.182172	3.318497	3.42333	34.88467
3.243295	3.438457	3.398435	35.22623
3.279094	3.496348	3.480896	35.61144
3.313409	3.453688	3.410081	35.43833
3.327473	3.21626	3.41269	34.95558

3.198422	3.197323	3.408905	34.62367
3.258349	3.241533	3.398674	34.82903
3.31877	3.350172	3.400123	35.20191
3.350981	3.478961	3.378041	35.5057
3.272207	3.366683	3.376728	35.08503
3.320997	3.304613	3.396304	35.0988
3.273526	3.373288	3.331388	35.0032
3.331968	3.405704	3.3991	35.34997
3.180852	3.4084	3.428923	35.09062
3.217105	3.430033	3.42836	35.21598
3.1592	3.249148	3.42836	34.69378
3.299468	3.137342	3.385527	34.66235
3.286683	3.355293	3.45728	35.26793
3.359808	3.434616	3.37579	35.4231
3.306645	3.43064	3.468261	35.50037
3.317203	3.327932	3.400549	35.15078
3.313615	3.225493	3.388068	34.89162
3.412764	3.301446	3.320185	35.12609
3.158663	3.352193	3.443758	34.95162
3.214837	3.462315	3.352242	35.11515
3.160932	3.412039	3.490837	35.19041
3.338072	3.349363	3.325727	35.07966
3.344259	3.175891	3.410695	34.89964
3.138083	3.231693	3.438353	34.63128
3.20366	3.354687	3.464118	35.1
3.348919	3.411837	3.41622	35.43789
3.417672	3.420531	3.384436	35.53775
3.349868	3.307646	3.360035	35.08925
3.227705	3.348419	3.376677	34.94766
3.217105	3.418307	3.39748	35.1228
3.316584	3.490013	3.363496	35.42284
3.303469	3.400919	3.364247	35.20097
3.245893	3.277454	3.428036	34.94456
3.094984	3.149204	3.417447	34.31092
3.304954	3.263975	3.37782	34.93442
3.309284	3.445534	3.423194	35.44016
3.361251	3.44641	3.406534	35.51928
3.157509	3.332784	3.507548	35.04615
3.32838	3.277791	3.408257	35.08242
3.319595	3.249216	3.391273	34.96358
3.437015	3.349835	3.367913	35.38931
3.225643	3.442097	3.508315	35.43588
3.138743	3.471682	3.454569	35.193
3.207908	3.310679	3.388613	34.84793
3.362406	3.193009	3.360342	34.86665
3.245893	3.170837	3.342642	34.52466
3.292333	3.274488	3.444866	35.07643
3.191864	3.455912	3.45121	35.26734
3.444769	3.440681	3.33207	35.52655
3.438747	3.406984	3.341091	35.45942

3.430746	3.321799	3.348815	35.27253
3.266144	3.372681	3.439154	35.2214
3.246099	3.473637	3.358449	35.22185
3.31114	3.409074	3.448959	35.42083
3.346321	3.296796	3.338652	35.011
3.210011	3.138218	3.380701	34.45809
3.129711	3.140981	3.468278	34.48004
3.240367	3.428011	3.447867	35.30508
3.409588	3.515556	3.470921	35.917
3.33168	3.337569	3.372568	35.14232
3.268041	3.302592	3.423313	35.03763
3.254513	3.279139	3.437909	34.98868
3.293117	3.324158	3.37516	35.03433
3.36286	3.404154	3.365031	35.33963
3.234593	3.404154	3.346138	35.01782
3.234634	3.381577	3.488945	35.28083
3.209269	3.299828	3.362422	34.76991
3.332009	3.205208	3.35451	34.8141
3.25971	3.296661	3.359148	34.86613
3.261277	3.410422	3.486421	35.39665
3.210135	3.438457	3.422461	35.20625
3.454007	3.395662	3.386976	35.56838
3.365087	3.33521	3.398094	35.26604
3.383976	3.389193	3.372039	35.36842
3.267546	3.454362	3.4109	35.3413
3.222013	3.439266	3.405307	35.19649
3.187616	3.33083	3.375245	34.81839
3.29852	3.27988	3.394479	34.99156
3.126865	3.222191	3.431924	34.57191
3.229808	3.394854	3.404642	35.11495
3.176481	3.469728	3.520712	35.4159
3.415362	3.421878	3.440365	35.65795
3.397545	3.311083	3.469301	35.43998
3.342155	3.275567	3.362337	35.00726
3.266969	3.258044	3.472967	35.04646
3.281569	3.379555	3.392842	35.16889
3.259215	3.397482	3.414174	35.20585
3.327019	3.424439	3.373966	35.32516
3.224859	3.310544	3.4264	34.96734
3.212898	3.208308	3.411872	34.68584
3.266804	3.189235	3.451431	34.84852
3.236696	3.38461	3.464186	35.23783
3.356756	3.435492	3.41194	35.4974
3.319471	3.438525	3.364264	35.31823
3.357704	3.348285	3.429384	35.34691
3.327514	3.320047	3.361587	35.07088
3.405588	3.410758	3.315274	35.33871
3.230963	3.458204	3.429878	35.3112
3.294313	3.341747	3.325932	34.96776
3.184977	3.192133	3.448464	34.66943

3.286312	3.221652	3.321498	34.67793
3.243336	3.260605	3.375432	34.78708
3.290519	3.509288	3.393268	35.4731
3.140558	3.481859	3.556588	35.44233
3.289735	3.403278	3.401351	35.25723
3.299097	3.288641	3.405648	35.03641
3.430787	3.323079	3.416987	35.4245
3.259545	3.284867	3.429656	34.99416
3.276331	3.345521	3.378502	35.05165
3.224653	3.380768	3.411121	35.08705
3.311347	3.25117	3.347911	34.85499
3.239418	3.235872	3.354374	34.67837
3.201227	3.226706	3.391938	34.65696
3.159612	3.347678	3.421693	34.89557
3.308831	3.488666	3.487563	35.67425
3.391111	3.50949	3.41992	35.7518
3.392266	3.361089	3.38655	35.35682
3.373583	3.314116	3.369703	35.1764
3.346898	3.421811	3.413764	35.44991
3.372346	3.416554	3.34075	35.33439
3.37581	3.415341	3.299314	35.24871
3.270763	3.188831	3.349855	34.63417
3.170583	3.177778	3.408069	34.51822
3.234469	3.25299	3.429554	34.86939
3.247254	3.426596	3.354715	35.11334
3.32009	3.509288	3.477077	35.72104
3.212445	3.364459	3.42836	35.06238
3.229355	3.317553	3.468602	35.08479
3.296705	3.299155	3.355107	34.94364
3.433839	3.355428	3.457143	35.58973
3.212775	3.381307	3.374341	34.98182
3.312584	3.385755	3.398895	35.26351
3.266102	3.267479	3.3718	34.84396
3.321492	3.259662	3.332207	34.86141
3.236902	3.215317	3.324431	34.56244
3.255585	3.296931	3.391529	34.92851
3.17912	3.435492	3.475014	35.24687
3.326978	3.469728	3.399305	35.47952
3.359271	3.438188	3.462532	35.61943
3.45941	3.373085	3.295307	35.33036
3.313574	3.355361	3.481493	35.37983
3.357044	3.469256	3.318429	35.36737
3.333907	3.400919	3.459394	35.4756
3.350404	3.308859	3.310381	34.98449
3.193267	3.138421	3.376166	34.41199
3.131154	3.211677	3.38065	34.44617
3.120018	3.359674	3.409877	34.80938
3.257483	3.491698	3.402596	35.38278
3.273031	3.434548	3.410797	35.30974
3.305036	3.307646	3.434499	35.15405

3.33069	3.315464	3.398094	35.14763
3.231541	3.321125	3.447049	35.05025
3.385213	3.405232	3.357375	35.37413
3.310563	3.375849	3.4109	35.26368
3.296623	3.347408	3.349241	35.03616
3.264247	3.184113	3.39748	34.71375
3.297984	3.220641	3.299604	34.65337
3.176687	3.271658	3.463998	34.85918
3.223374	3.439603	3.386277	35.15858
3.156271	3.440479	3.462054	35.17947
3.344259	3.462854	3.345371	35.38433
3.358611	3.352867	3.356676	35.19992
3.416435	3.388586	3.398912	35.49684
3.305325	3.468987	3.399782	35.43159
3.375439	3.434144	3.386874	35.48049
3.289034	3.282306	3.38568	34.95688
3.327968	3.141184	3.381469	34.7242
3.132845	3.200086	3.344058	34.3445
3.264329	3.342489	3.428497	35.1281
3.210259	3.514949	3.380906	35.28293
3.233067	3.445871	3.430373	35.28992
3.225189	3.403076	3.453051	35.2287
3.34331	3.370457	3.372994	35.24061
3.307098	3.338243	3.461253	35.28398
3.291715	3.385149	3.435863	35.29739
3.297283	3.358326	3.443775	35.26821
3.380594	3.362774	3.397702	35.35937
3.320585	3.247329	3.326494	34.81996
3.258637	3.169017	3.297012	34.44876
3.173594	3.193683	3.366634	34.46898
3.16617	3.378477	3.415265	34.96321
3.229478	3.426663	3.477622	35.34339
3.316213	3.46683	3.367964	35.3811
3.289075	3.362437	3.476122	35.32999
3.273403	3.362033	3.363837	35.04928
3.382409	3.422215	3.436801	35.57883
3.359931	3.47458	3.369106	35.49615
3.363602	3.380836	3.390063	35.345
3.300788	3.209588	3.388733	34.83024
3.300706	3.130064	3.346939	34.56476
3.195906	3.288574	3.347758	34.684
3.333371	3.501538	3.385254	35.53233
3.153384	3.455306	3.454773	35.18966
3.234304	3.334401	3.351918	34.87729
3.219539	3.281767	3.405324	34.84669
3.308996	3.368974	3.42229	35.27013
3.313862	3.405367	3.450869	35.42285
3.331143	3.358798	3.393865	35.23414
3.239665	3.322203	3.48874	35.16155
3.370118	3.270984	3.341619	35.01309

3.359601	3.184922	3.391665	34.91133
3.291096	3.265929	3.2721	34.6772
3.114987	3.287226	3.469148	34.76956
3.215579	3.397078	3.384657	35.045
3.359478	3.452138	3.406142	35.52707
3.379027	3.464876	3.335549	35.4433
3.278929	3.377129	3.431975	35.24339
3.310439	3.440614	3.431992	35.45116
3.265525	3.425989	3.402527	35.25653
3.421013	3.386564	3.380548	35.46227
3.353868	3.230278	3.299348	34.79609
3.223746	3.126559	3.374767	34.44965
3.252245	3.260875	3.366054	34.78664
3.203206	3.419115	3.401095	35.10208
3.339516	3.424372	3.392484	35.39283
3.280373	3.355496	3.394667	35.11765
3.188647	3.336625	3.406824	34.90238
3.225065	3.378679	3.455507	35.18044
3.394493	3.47485	3.368629	35.57128
3.250018	3.373018	3.445565	35.20089
3.33003	3.322136	3.352566	35.06122
3.213517	3.299491	3.524531	35.13297
3.360962	3.257775	3.33057	34.94001
3.381625	3.1736	3.317935	34.77349
3.260617	3.266671	3.350264	34.7831
3.118245	3.311353	3.45728	34.80349
3.270021	3.474041	3.401641	35.3695
3.223168	3.459619	3.372329	35.1714
3.393957	3.397549	3.365082	35.39331
3.276125	3.449106	3.407489	35.34111
3.283012	3.365335	3.467033	35.30319
3.255915	3.400312	3.416629	35.2102
3.365912	3.304411	3.402988	35.21119
3.353002	3.239107	3.376046	34.98123
3.222838	3.179935	3.334475	34.47628
3.204939	3.33548	3.431788	34.99009
3.260535	3.486442	3.426178	35.42953
3.360591	3.429696	3.331456	35.31711
3.258143	3.350576	3.384436	35.0359
3.192937	3.336154	3.419442	34.93832
3.278599	3.460023	3.46335	35.49255
3.27996	3.392293	3.452386	35.32344
3.350404	3.327325	3.381025	35.17936
3.306232	3.216125	3.391614	34.86274
3.297736	3.181957	3.416032	34.82284
3.280125	3.248474	3.414463	34.92636
3.292086	3.337299	3.355601	35.01804
3.29221	3.402873	3.452727	35.37411
3.19669	3.37996	3.377871	34.95142
3.23715	3.395393	3.432385	35.19286

3.273774	3.42882	3.356079	35.17918
3.38311	3.440749	3.379678	35.49598
3.230592	3.337367	3.362132	34.89799
3.333041	3.355428	3.401624	35.24789
3.242553	3.283182	3.396542	34.8809
3.383935	3.242611	3.317321	34.92812
3.354528	3.224617	3.3644	34.92742
3.38476	3.320249	3.375773	35.22753
3.248285	3.438592	3.472353	35.39908
3.302355	3.423631	3.381298	35.28549
3.194091	3.362909	3.455251	35.07766
3.333948	3.362707	3.333758	35.11738
3.215249	3.350576	3.451636	35.08906
3.196071	3.428011	3.443246	35.19811
3.240449	3.327056	3.456206	35.10272
3.363932	3.211947	3.325676	34.83559
3.302768	3.187753	3.395025	34.80058
3.34298	3.249283	3.356198	34.93817
3.26136	3.264042	3.430543	34.95453
3.170335	3.413724	3.396576	35.00853
3.373954	3.371266	3.332343	35.22049
3.321121	3.385553	3.391342	35.26522
3.360137	3.496753	3.344211	35.49065
3.209434	3.425787	3.432657	35.19931
3.278805	3.426933	3.344262	35.16022
3.255379	3.353474	3.410201	35.09254
3.326854	3.290461	3.383975	35.05369
3.179326	3.233917	3.480078	34.81758
3.274764	3.236209	3.306425	34.65155
3.273238	3.277117	3.386209	34.91215
3.39041	3.461439	3.384657	35.56807
3.222137	3.384947	3.439904	35.15363
3.20931	3.369581	3.396917	34.99797
3.236902	3.375983	3.373949	35.02208
3.310522	3.441288	3.365901	35.30829
3.223292	3.455845	3.440109	35.31164
3.270392	3.413319	3.420721	35.27925
3.138042	3.236276	3.41876	34.59837
3.288333	3.185192	3.385254	34.74204
3.357622	3.202579	3.325727	34.80142
3.393751	3.266738	3.404147	35.19222
3.255214	3.332312	3.381213	34.98251
3.331185	3.337973	3.367759	35.1316
3.274227	3.374029	3.334458	35.01307
3.435365	3.397145	3.353419	35.45747
3.263463	3.484352	3.391324	35.35515
3.105748	3.44277	3.452369	35.05281
3.243831	3.385958	3.42437	35.16931
3.319925	3.339389	3.351577	35.07469
3.342485	3.273006	3.384521	35.0509

3.263215	3.248811	3.411735	34.88415
3.188647	3.258988	3.428224	34.7794
3.288951	3.389597	3.286014	34.97338
3.454832	3.39391	3.347331	35.47965
3.304748	3.37322	3.375381	35.16754
3.163076	3.365874	3.431992	34.96546
3.220075	3.347139	3.402169	34.98392
3.295097	3.486105	3.428514	35.50949
3.288745	3.473098	3.326273	35.24357
3.285239	3.281228	3.387914	34.95111
3.112388	3.194223	3.441541	34.50012
3.351023	3.263031	3.371545	35.01938
3.281528	3.276106	3.375091	34.90375
3.432355	3.355967	3.358722	35.37243
3.262968	3.354013	3.447748	35.19242
3.336752	3.326449	3.341329	35.06078
3.322111	3.390001	3.384384	35.2619
3.38641	3.458271	3.311046	35.39142
3.238304	3.47303	3.438608	35.37877
3.207537	3.444658	3.369516	35.09835
3.120554	3.276038	3.436409	34.68567
3.360715	3.259931	3.339897	34.96459
3.347063	3.315599	3.470461	35.34199
3.340629	3.329212	3.415418	35.23732
3.259669	3.367896	3.467238	35.25819
3.2899	3.441557	3.360171	35.25125
3.372263	3.383194	3.360427	35.30429
3.356261	3.356035	3.330621	35.14472
3.155199	3.35435	3.394854	34.84182
3.225065	3.400582	3.397531	35.10156
3.271505	3.465887	3.40205	35.35581
3.252987	3.310072	3.384299	34.93575
3.289158	3.211475	3.29442	34.60269
3.140351	3.126424	3.493037	34.52562
3.288786	3.212217	3.359762	34.74639
3.273361	3.309264	3.418147	35.05256
3.402659	3.412039	3.366173	35.44641
3.231334	3.380634	3.440842	35.16636
3.36983	3.386497	3.321822	35.22177
3.33667	3.432527	3.397258	35.41488
3.364674	3.4795	3.288299	35.34057
3.160602	3.405771	3.397804	34.97253
3.266927	3.380836	3.351304	35.04883
3.154787	3.343297	3.429196	34.89185
3.289982	3.249755	3.355005	34.82069
3.386822	3.279139	3.413611	35.22488
3.304706	3.298885	3.405938	35.07171
3.278228	3.344578	3.417396	35.13879
3.30417	3.396538	3.40662	35.28558
3.301366	3.331032	3.377223	35.07191

3.360426	3.377331	3.382594	35.31406
3.15656	3.442434	3.390216	35.02728
3.159653	3.421744	3.430014	35.07583
3.278805	3.423024	3.411241	35.29814
3.222137	3.310274	3.495629	35.11219
3.276496	3.260605	3.385254	34.88108
3.23517	3.196986	3.425922	34.74051
3.264494	3.244498	3.406415	34.86588
3.31312	3.269164	3.385408	34.98022
3.409217	3.371535	3.403533	35.45388
3.247749	3.320249	3.385834	34.94991
3.340423	3.40375	3.347655	35.25169
3.307841	3.450251	3.423859	35.44877
3.367355	3.481589	3.335617	35.45448
3.275671	3.444321	3.348491	35.20063
3.275795	3.294572	3.389432	34.96296
3.151033	3.300772	3.421489	34.77379
3.259792	3.312566	3.405784	35.00307
3.261236	3.307916	3.400822	34.98521
3.435077	3.332177	3.387232	35.38871
3.265195	3.28271	3.445685	35.03686
3.37284	3.351991	3.404164	35.33297
3.286023	3.34478	3.414583	35.15012
3.329287	3.409478	3.408086	35.37201
3.212486	3.43192	3.415128	35.18107
3.195081	3.386766	3.370675	34.94705
3.105913	3.289585	3.414651	34.6357
3.252946	3.239174	3.373369	34.75672
3.322111	3.257775	3.371681	34.94496
3.232283	3.261953	3.411326	34.84435
3.317409	3.30987	3.382424	35.07209
3.319801	3.338647	3.416373	35.2145
3.409176	3.44021	3.364468	35.51854
3.381543	3.370255	3.342148	35.25632
3.317863	3.405704	3.384453	35.28709
3.266804	3.428079	3.40928	35.27866
3.313367	3.398493	3.350656	35.18759
3.240779	3.325438	3.349241	34.86599
3.322482	3.222932	3.371886	34.87002
3.178295	3.229402	3.433544	34.70369
3.224653	3.331301	3.408461	34.97305
3.279259	3.40193	3.429656	35.29327
3.330937	3.363583	3.434976	35.33406
3.24379	3.37012	3.414446	35.11288
3.401463	3.373153	3.394973	35.42174
3.277073	3.393506	3.43143	35.27395
3.379934	3.446545	3.372755	35.48657
3.200608	3.349295	3.395297	34.93104
3.257483	3.331571	3.389159	35.00323
3.183574	3.270377	3.376524	34.68015

3.215249	3.288708	3.41431	34.87214
3.19306	3.25299	3.376387	34.66257
3.310645	3.254809	3.420449	35.02005
3.200938	3.33238	3.454074	35.0233
3.428931	3.458338	3.431634	35.74826
3.377501	3.363381	3.433612	35.43246
3.420724	3.409343	3.348423	35.4412
3.245357	3.424642	3.419494	35.24658
3.23715	3.456586	3.398043	35.25158
3.240449	3.370996	3.372841	35.01651
3.253111	3.331706	3.288299	34.7734
3.198257	3.242274	3.45479	34.82196
3.248244	3.29437	3.373659	34.86777
3.231747	3.330223	3.443383	35.06258
3.190008	3.37996	3.439001	35.07049
3.390286	3.396875	3.411258	35.48478
3.269361	3.374298	3.424933	35.20087
3.363643	3.404087	3.360632	35.33158
3.336629	3.418172	3.383702	35.35376
3.27963	3.39054	3.393916	35.19102
3.232365	3.31816	3.354493	34.84316
3.305366	3.337299	3.384112	35.10943
3.1015	3.203523	3.427559	34.46607
3.275217	3.198873	3.319947	34.60046
3.194834	3.210734	3.41622	34.66115
3.255462	3.296324	3.406517	34.95969
3.328916	3.354417	3.483812	35.41639
3.389255	3.393101	3.426672	35.50799
3.305572	3.374838	3.431498	35.2956
3.381543	3.384408	3.366855	35.3413
3.281528	3.400582	3.429793	35.29558
3.392802	3.406513	3.324823	35.32234
3.18972	3.298615	3.415128	34.83976
3.204114	3.31971	3.287122	34.63744
3.240119	3.285945	3.374136	34.83263
3.268412	3.356035	3.352583	35.00064
3.294519	3.408534	3.43779	35.35887
3.286106	3.378207	3.400959	35.19361
3.271505	3.331571	3.424012	35.11011
3.403361	3.348824	3.377359	35.33416
3.353374	3.450116	3.365423	35.42026
3.228818	3.384205	3.503336	35.30533
3.282394	3.356507	3.38771	35.10906
3.163613	3.2972	3.450476	34.85688
3.304624	3.339456	3.320492	34.9734
3.216775	3.282845	3.419357	34.87369
3.16584	3.271254	3.391069	34.67509
3.167531	3.275297	3.454228	34.82575
3.33069	3.364796	3.39644	35.2519
3.299221	3.382386	3.354084	35.12892

3.376924	3.362033	3.39216	35.3376
3.25707	3.370929	3.416646	35.1485
3.32574	3.409006	3.350554	35.23741
3.391029	3.415409	3.374205	35.44591
3.430952	3.369514	3.323084	35.32106
3.182791	3.261144	3.356284	34.61398
3.210053	3.241533	3.326051	34.5646
3.148188	3.351924	3.415401	34.86611
3.325204	3.437851	3.376592	35.35626
3.329576	3.416352	3.408359	35.38827
3.272248	3.297739	3.417379	35.02324
3.227292	3.358259	3.45375	35.13682
3.368757	3.466493	3.470512	35.71953
3.385956	3.472491	3.352856	35.51296
3.261236	3.374096	3.354834	35.02937
3.215992	3.211071	3.44664	34.77468
3.164314	3.267884	3.428565	34.74638
3.362777	3.306568	3.352259	35.09812
3.279424	3.321866	3.374068	34.99699
3.220281	3.248744	3.446913	34.86704
3.224199	3.346061	3.447407	35.08951
3.332752	3.370727	3.461986	35.41272
3.4604	3.393573	3.336674	35.46779
3.310315	3.321057	3.40101	35.12169
3.165345	3.357315	3.440331	34.96994
3.304046	3.394719	3.503882	35.49403
3.289116	3.354957	3.312717	34.95638
3.3841	3.258651	3.38036	35.10141
3.202629	3.216193	3.40488	34.66534
3.131113	3.275904	3.395553	34.61912
3.160973	3.419317	3.407558	35.0243
3.411444	3.461708	3.306118	35.44291
3.237562	3.357787	3.48047	35.21668
3.277816	3.280419	3.390523	34.93881
3.18675	3.439873	3.475559	35.27433
3.401051	3.521958	3.372636	35.6974
3.421879	3.409478	3.436699	35.63707
3.315347	3.313846	3.415487	35.14858
3.18176	3.16713	3.435471	34.5793
3.254431	3.257977	3.334833	34.71681
3.183863	3.294302	3.412656	34.81212
3.261648	3.287832	3.343393	34.8166
3.149136	3.281902	3.46098	34.81473
3.22721	3.370524	3.466505	35.19135
3.33102	3.374366	3.481425	35.4594
3.458421	3.423765	3.411053	35.69214
3.228324	3.33858	3.447339	35.08202
3.259174	3.43192	3.374665	35.19468
3.331515	3.412646	3.383754	35.3306
3.310315	3.272332	3.382441	34.97453

3.361787	3.183507	3.2913	34.69353
3.186008	3.188629	3.386908	34.52941
3.245563	3.415611	3.371749	35.12287
3.281899	3.409613	3.422239	35.29963
3.315141	3.397886	3.35729	35.20464
3.291467	3.305557	3.361604	34.9604
3.225973	3.418644	3.455643	35.27012
3.229932	3.545276	3.479669	35.60825
3.313367	3.483409	3.424746	35.53531
3.2866	3.321057	3.372636	35.00778
3.361705	3.204264	3.389193	34.95282
3.215249	3.232907	3.41772	34.75756
3.22589	3.326179	3.370487	34.88152
3.225065	3.279273	3.429469	34.90612
3.246512	3.27031	3.326153	34.70748
3.188689	3.307579	3.426195	34.88131
3.357003	3.384745	3.415401	35.39453
3.23418	3.407389	3.468005	35.29049
3.31576	3.425855	3.435676	35.43858
3.342527	3.404895	3.398827	35.3707
3.422662	3.437042	3.333162	35.47264
3.371974	3.310409	3.407694	35.24786
3.25641	3.251238	3.324482	34.68377
3.245151	3.218282	3.374597	34.69667
3.280826	3.380229	3.378792	35.13801
3.244945	3.430235	3.414225	35.24639
3.124308	3.42107	3.450545	35.04195
3.239047	3.262897	3.409604	34.85744
3.25971	3.312027	3.389619	34.96636
3.277609	3.510703	3.428514	35.52504
3.348548	3.491294	3.456325	35.69854
3.213393	3.309668	3.369021	34.81487
3.234716	3.200962	3.338925	34.55797
3.371314	3.258247	3.393098	35.10042
3.299551	3.353406	3.383685	35.131
3.263215	3.362909	3.384436	35.07397
3.201763	3.261414	3.378877	34.70547
3.246264	3.277454	3.433885	34.95816
3.30384	3.366346	3.322521	35.03493
3.322317	3.369851	3.403158	35.25934
3.177677	3.389462	3.492764	35.18187
3.294395	3.381577	3.419835	35.26039
3.292333	3.408602	3.439137	35.35719
3.476485	3.343971	3.392484	35.51654
3.356178	3.274354	3.370044	35.05213
3.272619	3.239915	3.419101	34.90137
3.237974	3.35563	3.38834	35.01139
3.388018	3.448229	3.346104	35.44965
3.267958	3.39856	3.399305	35.19482
3.244821	3.313981	3.376132	34.90858

3.141341	3.339389	3.434721	34.86598
3.275506	3.483813	3.395434	35.38929
3.364633	3.502077	3.528436	35.91499
3.369623	3.31169	3.327944	35.07112
3.223498	3.131142	3.514045	34.76371
3.247337	3.242476	3.38389	34.77468
3.278187	3.38899	3.417396	35.23582
3.327431	3.367896	3.314183	35.07167
3.162705	3.277791	3.3452	34.58222
3.109006	3.230076	3.449982	34.58959
3.306109	3.396067	3.368066	35.20448
3.29122	3.417767	3.427082	35.34843
3.314275	3.455238	3.433357	35.49452
3.185925	3.358191	3.458508	35.05661
3.309243	3.341882	3.409484	35.18342
3.308418	3.300233	3.349872	34.96017
3.510593	3.263031	3.296978	35.20527
3.230303	3.202647	3.457553	34.81142
3.254596	3.409815	3.369498	35.12503
3.301201	3.396875	3.429452	35.32976
3.39206	3.46346	3.299195	35.38921
3.315265	3.336895	3.405989	35.17803
3.303469	3.340399	3.36781	35.07641
3.119565	3.425585	3.495885	35.14061
3.332174	3.561181	3.407302	35.70836
3.223498	3.408939	3.358261	35.03053
3.338897	3.201703	3.451005	35.03251
3.190916	3.163895	3.34919	34.40357
3.388719	3.217002	3.418147	35.10307
3.271134	3.40968	3.341773	35.10027
3.332546	3.318429	3.389227	35.13879
3.153343	3.259797	3.342148	34.51572
3.280991	3.27806	3.327432	34.80263
3.161757	3.362774	3.412809	34.91385
3.253729	3.419924	3.432521	35.28306
3.259545	3.434279	3.411599	35.28141
3.323802	3.38279	3.415469	35.3178
3.376429	3.317823	3.428514	35.31934
3.386038	3.249822	3.405818	35.14202
3.284415	3.263301	3.391614	34.9182
3.336423	3.307444	3.303969	34.9368
3.331185	3.398021	3.383634	35.29764
3.282765	3.416554	3.347076	35.15233
3.308459	3.353946	3.426536	35.24537
3.269237	3.312364	3.341193	34.88203
3.255008	3.377533	3.457774	35.24838
3.295427	3.494124	3.394752	35.45391
3.274062	3.414196	3.512732	35.4904
3.160437	3.211004	3.458252	34.67844
3.367809	3.234726	3.383344	35.01999

3.250925	3.341613	3.442974	35.12853
3.383564	3.460899	3.353265	35.48327
3.303057	3.299155	3.465465	35.19887
3.284415	3.24342	3.374068	34.83635
3.260452	3.220371	3.426928	34.84914
3.341867	3.326853	3.368629	35.13255
3.225684	3.408332	3.484699	35.31048
3.244243	3.502144	3.371476	35.30862
3.226261	3.344443	3.488058	35.17938
3.325411	3.250496	3.384163	34.96355
3.373335	3.275432	3.391512	35.13896
3.367603	3.294841	3.407779	35.20444
3.197597	3.378207	3.497453	35.21108
3.346238	3.429831	3.341261	35.30746
3.342155	3.36392	3.30622	35.07776
3.425261	3.27806	3.345012	35.15657
3.173429	3.326314	3.416629	34.86799
3.1811	3.475659	3.404215	35.18421
3.256204	3.566236	3.435795	35.61559
3.281445	3.411163	3.417993	35.29274
3.314275	3.184316	3.383975	34.79406
3.135072	3.185461	3.4279	34.50074
3.2753	3.294572	3.408717	35.00405
3.386286	3.468717	3.462753	35.74575
3.309202	3.420531	3.388818	35.31012
3.225272	3.218619	3.386635	34.68026
3.263999	3.287765	3.395894	34.93641
3.245357	3.477276	3.392075	35.30172
3.416888	3.421946	3.388272	35.54752
3.181801	3.31452	3.442155	34.91633
3.114121	3.270647	3.455302	34.70113
3.228695	3.289248	3.435403	34.94885
3.29951	3.317081	3.345081	34.96705
3.235747	3.335817	3.367111	34.91677
3.32372	3.28763	3.450204	35.18548
3.212816	3.304816	3.387846	34.84417
3.368881	3.456317	3.372107	35.48235
3.357787	3.468178	3.320595	35.37137
3.426168	3.401391	3.397958	35.54404
3.169263	3.351452	3.397889	34.87287
3.229355	3.419452	3.376643	35.10653
3.235294	3.467976	3.427747	35.33738
3.312666	3.381981	3.280302	34.99609
3.22688	3.19584	3.499909	34.88167
3.110739	3.197457	3.385118	34.3802
3.301324	3.266401	3.464697	35.12178
3.364468	3.399369	3.320799	35.23596
3.301077	3.273275	3.407353	35.01086
3.231623	3.262358	3.436545	34.89894
3.287095	3.343432	3.434022	35.19203

3.264205	3.413319	3.370811	35.15658
3.358364	3.394651	3.331303	35.23526
3.216033	3.39546	3.434022	35.15041
3.178089	3.375983	3.445838	35.05068
3.222879	3.361696	3.371016	34.95376
3.423735	3.307511	3.383907	35.30269
3.219662	3.219226	3.371408	34.63602
3.323018	3.277589	3.411377	35.07708
3.246924	3.359472	3.396576	35.05737
3.3768	3.394382	3.345183	35.30534
3.375356	3.39054	3.352583	35.30997
3.414414	3.305355	3.336146	35.17315
3.214012	3.327932	3.408444	34.94238
3.261896	3.526002	3.366719	35.38899
3.15392	3.488935	3.465806	35.2885
3.331597	3.352261	3.36556	35.15894
3.254884	3.199277	3.471058	34.88734
3.259669	3.2323	3.443008	34.90868
3.302685	3.387103	3.413031	35.27572
3.370325	3.453823	3.424353	35.5943
3.319142	3.275028	3.35688	34.94383
3.291921	3.233311	3.389756	34.86496
3.183451	3.225561	3.403619	34.64112
3.245687	3.388721	3.367555	35.05516
3.347105	3.462382	3.415811	35.54356
3.276125	3.42727	3.444662	35.37465
3.276537	3.286956	3.454842	35.09097
3.186461	3.227245	3.383344	34.60706
3.316749	3.301311	3.437364	35.17208
3.256864	3.380701	3.402306	35.13806
3.317244	3.458743	3.429673	35.50062
3.291508	3.351452	3.344109	35.02259
3.355147	3.322742	3.369157	35.15376
3.309243	3.360752	3.310654	35.00855
3.363313	3.355833	3.396389	35.30353
3.163489	3.411972	3.452437	35.11188
3.226055	3.408467	3.343665	35.00317
3.209269	3.239444	3.404164	34.72914
3.270103	3.173196	3.371903	34.64675
3.299015	3.315733	3.474604	35.24627
3.303098	3.454699	3.455166	35.51659
3.218178	3.386766	3.487444	35.25291
3.359313	3.356844	3.386908	35.27626
3.358859	3.254607	3.403056	35.087
3.398989	3.362235	3.381639	35.36329
3.222096	3.369446	3.442206	35.12467
3.233768	3.344982	3.360853	34.91879
3.272866	3.384205	3.390455	35.15481
3.305737	3.363516	3.378024	35.15426
3.224117	3.256696	3.420875	34.83588

3.272083	3.242072	3.38834	34.83765
3.256658	3.283452	3.462822	35.05728
3.292704	3.364055	3.321652	35.00366
3.299386	3.44304	3.417192	35.39993
3.300128	3.41123	3.4028	35.30052
3.405423	3.383262	3.424012	35.51601
3.290931	3.461573	3.331456	35.23448
3.410949	3.409545	3.385527	35.50141
3.222879	3.384475	3.410235	35.08934
3.21327	3.387777	3.369993	34.98754
3.211909	3.231019	3.474945	34.87127
3.293736	3.258718	3.325403	34.78377
3.213228	3.225965	3.429401	34.76351
3.253565	3.361426	3.356744	34.98906
3.20832	3.355833	3.483403	35.15487
3.39305	3.346061	3.343188	35.23085
3.480115	3.287361	3.385067	35.38446
3.324916	3.344376	3.374955	35.14763
3.288333	3.362976	3.425632	35.21913
3.247502	3.326449	3.36087	34.90834
3.26767	3.425181	3.378246	35.20635
3.328834	3.421339	3.392842	35.36362
3.228571	3.29538	3.429315	34.94867
3.202051	3.207432	3.412008	34.6605
3.278187	3.219293	3.466846	34.97286
3.269938	3.359944	3.405989	35.12932
3.370984	3.441423	3.410166	35.53761
3.32108	3.427944	3.358142	35.28523
3.336423	3.338715	3.412076	35.24159
3.292704	3.353676	3.34699	35.03638
3.400639	3.392764	3.365815	35.39906
3.284373	3.443444	3.42611	35.38749
3.25307	3.43839	3.380019	35.20718
3.074692	3.230885	3.421148	34.45326
3.318152	3.257977	3.343938	34.87607
3.239336	3.315262	3.439751	35.03851
3.268742	3.38461	3.407779	35.18456
3.240985	3.409613	3.461782	35.29663
3.362983	3.352867	3.393183	35.28931
3.337041	3.242005	3.33751	34.86839
3.408681	3.330223	3.368339	35.2854
3.218879	3.345521	3.429537	35.03761
3.241439	3.437042	3.340852	35.09315
3.188936	3.380634	3.421148	35.03057
3.301036	3.268895	3.336435	34.84611
3.279672	3.193818	3.415879	34.80894
3.194586	3.304276	3.438796	34.91454
3.183203	3.357787	3.47515	35.08617
3.420312	3.489542	3.40159	35.73195
3.415775	3.414263	3.387846	35.52735

3.3118	3.332447	3.329189	34.99278
3.347723	3.340736	3.40355	35.25208
3.259174	3.426529	3.388664	35.2135
3.388059	3.454834	3.324499	35.41693
3.345826	3.380903	3.326256	35.16674
3.265855	3.242409	3.416152	34.88558
3.050152	3.174072	3.457433	34.35471
3.282806	3.33056	3.397207	35.07399
3.281198	3.463865	3.455796	35.49012
3.309985	3.445399	3.358534	35.29999
3.168314	3.328606	3.527703	35.10472
3.317987	3.272871	3.372176	34.97003
3.328298	3.259797	3.356062	34.92875
3.376965	3.349969	3.358398	35.23748
3.220776	3.387979	3.45213	35.18402
3.226303	3.405974	3.426041	35.1784
3.183368	3.294976	3.369737	34.71865
3.309326	3.208712	3.313774	34.68307
3.164685	3.179396	3.408444	34.50968
3.209682	3.318025	3.395093	34.88205
3.226055	3.424305	3.436085	35.23991
3.335268	3.489879	3.412059	35.5696
3.468608	3.452812	3.371204	35.69079
3.426622	3.363448	3.379746	35.42223
3.300293	3.374838	3.434704	35.29106
3.244862	3.402199	3.350691	35.04596
3.318482	3.329886	3.361996	35.07354
3.290477	3.29747	3.338891	34.89088
3.231376	3.189438	3.335498	34.51796
3.125586	3.237085	3.459258	34.66146
3.253358	3.394651	3.413372	35.1851
3.338567	3.483881	3.457979	35.66412
3.268825	3.381105	3.451892	35.27354
3.245728	3.280756	3.442701	34.98349
3.286147	3.287563	3.457433	35.11898
3.296334	3.316475	3.332633	34.93156
3.383481	3.423833	3.361569	35.42019
3.270268	3.38279	3.395502	35.15707
3.272784	3.347004	3.398895	35.09173
3.274392	3.281969	3.384777	34.92215
3.334484	3.195099	3.374188	34.84043
3.265855	3.283384	3.34196	34.81294
3.247254	3.40503	3.408342	35.18345
3.189431	3.382925	3.432197	35.06083
3.402659	3.401121	3.380292	35.45341
3.372098	3.346128	3.328046	35.15206
3.417466	3.413185	3.42599	35.6121
3.321039	3.456586	3.377496	35.3901
3.277568	3.401728	3.440791	35.31348
3.271629	3.295717	3.357631	34.88681

3.282064	3.213093	3.405392	34.83339
3.186709	3.217136	3.442922	34.71578
3.241356	3.404019	3.357682	35.05756
3.199742	3.458675	3.510754	35.42082
3.289694	3.39108	3.416919	35.26451
3.326277	3.334671	3.52182	35.45056
3.282187	3.302794	3.34919	34.90692
3.311759	3.269636	3.460008	35.14141
3.298891	3.400312	3.350554	35.15968
3.212816	3.401323	3.458525	35.20978
3.296004	3.407995	3.36099	35.19299
3.234758	3.256562	3.346922	34.69713
3.166912	3.188831	3.334099	34.37261
3.262184	3.231828	3.451295	34.93127
3.1234	3.368772	3.438319	34.89887
3.277816	3.49244	3.390063	35.40146
3.287467	3.455912	3.404556	35.37438
3.351311	3.38744	3.418419	35.39458
3.252616	3.334199	3.360495	34.93565
3.50317	3.442703	3.370112	35.74188
3.277279	3.425113	3.40101	35.277
3.381419	3.363852	3.34283	35.24354
3.207042	3.161671	3.434908	34.62142
3.293612	3.178048	3.278989	34.50558
3.176439	3.279476	3.408734	34.75488
3.333535	3.483611	3.391188	35.50647
3.163283	3.433605	3.517711	35.30148
3.235747	3.359404	3.34699	34.92435
3.170377	3.296931	3.462753	34.89793
3.407856	3.331571	3.351083	35.2488
3.313244	3.318025	3.405443	35.13115
3.30516	3.377803	3.349838	35.1226
3.230674	3.378477	3.443519	35.16605
3.273691	3.272601	3.389023	34.90942
3.327184	3.24679	3.317542	34.81364
3.246677	3.185596	3.358671	34.5937
3.143156	3.270243	3.401692	34.6465
3.182461	3.445736	3.48151	35.29078
3.357127	3.494259	3.318548	35.42249
3.374573	3.385284	3.402323	35.40553
3.361952	3.347678	3.312359	35.09896
3.324214	3.363516	3.458303	35.37022
3.276248	3.487857	3.353402	35.30784
3.40567	3.375849	3.318207	35.26896
3.390658	3.179463	3.379746	34.94124
3.154787	3.107419	3.373062	34.25326
3.287673	3.236546	3.420602	34.9302
3.227292	3.465415	3.388511	35.22848
3.307346	3.479972	3.433408	35.53356
3.216198	3.366683	3.42345	35.06472

3.184564	3.260134	3.504069	34.93883
3.207537	3.328403	3.402664	34.91661
3.445924	3.429427	3.418539	35.69356
3.255503	3.379151	3.404676	35.13688
3.373005	3.333795	3.397804	35.27963
3.248533	3.236141	3.377837	34.75021
3.368056	3.199412	3.397258	34.97373
3.303222	3.224617	3.311353	34.69921
3.25773	3.304613	3.423126	35.0191
3.124638	3.397954	3.399782	34.88112
3.284909	3.477883	3.420312	35.45129
3.25142	3.470065	3.44132	35.4069
3.433386	3.430977	3.324516	35.46392
3.300994	3.374029	3.468602	35.36496
3.331844	3.439873	3.38123	35.38534
3.309779	3.415274	3.482226	35.50416
3.340423	3.290932	3.336691	34.981
3.267257	3.129457	3.450937	34.71771
3.260122	3.199749	3.358313	34.65327
3.236077	3.347948	3.418061	35.05543
3.248327	3.485633	3.398213	35.33991
3.282146	3.38899	3.40101	35.20864
3.262721	3.334469	3.456342	35.16794
3.295427	3.332919	3.428002	35.1741
3.199123	3.361426	3.474741	35.12805
3.348878	3.407187	3.416782	35.42886
3.342032	3.349228	3.454671	35.37
3.330772	3.325708	3.373591	35.11663
3.223209	3.206016	3.413219	34.70632
3.381584	3.24342	3.34728	34.99026
3.217147	3.250294	3.378485	34.71394
3.305449	3.349295	3.330007	35.01753
3.131113	3.398088	3.483625	35.07892
3.297777	3.459282	3.328916	35.23889
3.254472	3.372344	3.418556	35.15009
3.288786	3.443242	3.357903	35.24754
3.283425	3.468987	3.46555	35.52752
3.393998	3.431718	3.357699	35.45197
3.215332	3.368233	3.359063	34.92541
3.342898	3.214778	3.351151	34.85149
3.268618	3.17178	3.393439	34.6875
3.282022	3.278936	3.363991	34.88675
3.229231	3.428685	3.369157	35.11008
3.208362	3.417093	3.470904	35.26159
3.271959	3.413522	3.386908	35.20918
3.30219	3.317418	3.398827	35.09119
3.319884	3.375444	3.439734	35.34623
3.146002	3.3621	3.426314	34.90745
3.267464	3.2817	3.392961	34.92431
3.303634	3.332582	3.405443	35.14197

3.419033	3.3528	3.303407	35.21541
3.241604	3.233378	3.418147	34.81716
3.259462	3.251912	3.342318	34.73092
3.177594	3.247666	3.417874	34.70783
3.285652	3.360752	3.381469	35.11183
3.212816	3.402671	3.438336	35.16857
3.279465	3.439805	3.392808	35.29597
3.198587	3.375377	3.375296	34.93991
3.331143	3.388856	3.412008	35.33955
3.276331	3.443579	3.3821	35.27395
3.401216	3.406715	3.409894	35.52722
3.262267	3.315666	3.393251	34.98786
3.298149	3.169287	3.362558	34.6791
3.284332	3.257236	3.354425	34.82342
3.385708	3.419385	3.368203	35.42984
3.214012	3.421744	3.429725	35.19407
3.229231	3.423024	3.368527	35.09632
3.271835	3.319238	3.413781	35.06149
3.331432	3.367627	3.350691	35.15967
3.306067	3.385351	3.409877	35.27239
3.186338	3.326921	3.491809	35.06195
3.18044	3.342286	3.424302	34.93503
3.335226	3.313172	3.412878	35.18487
3.338443	3.219226	3.363769	34.87907
3.308253	3.195773	3.386618	34.81173
3.188359	3.266738	3.438591	34.81839
3.132845	3.359202	3.457161	34.9398
3.331185	3.445534	3.39985	35.437
3.28924	3.458271	3.363292	35.29318
3.339969	3.434683	3.420107	35.47678
3.202175	3.392967	3.390932	35.02042
3.278682	3.386025	3.408308	35.21054
3.313821	3.370322	3.316707	35.05273
3.337619	3.30522	3.364315	35.06652
3.227622	3.178587	3.427559	34.68735
3.308624	3.178722	3.370607	34.74024
3.275176	3.342219	3.380752	35.04682
3.411898	3.453688	3.368424	35.56262
3.173429	3.432661	3.404846	35.07479
3.134247	3.372816	3.412809	34.87565
3.233025	3.372546	3.369226	34.99576
3.311058	3.407793	3.430287	35.37701
3.260576	3.383397	3.440365	35.2353
3.229107	3.317957	3.470376	35.08901
3.146909	3.241331	3.480743	34.76436
3.365499	3.294976	3.349787	35.07331
3.414496	3.211677	3.420039	35.15193
3.260205	3.299694	3.34121	34.83461
3.206258	3.313038	3.435744	34.95255
3.161179	3.38151	3.406466	34.93968

3.337495	3.472828	3.346189	35.39314
3.377253	3.459754	3.326341	35.40809
3.209682	3.46346	3.493054	35.41432
3.19669	3.423361	3.384453	35.06072
3.278723	3.435829	3.41801	35.34076
3.388142	3.407119	3.387505	35.45055
3.294602	3.288978	3.351577	34.90907
3.198834	3.178992	3.417754	34.60384
3.278104	3.240589	3.416356	34.90884
3.26004	3.399773	3.409604	35.20268
3.400762	3.388451	3.381997	35.42528
3.274516	3.342286	3.392484	35.07118
3.164355	3.352463	3.420755	34.91436
3.187864	3.38461	3.393047	34.97547
3.343805	3.474109	3.371425	35.46493
3.20832	3.418239	3.466778	35.25499
3.225601	3.283587	3.377223	34.80247
3.254802	3.256966	3.472695	35.01689
3.363767	3.312768	3.337237	35.08099
3.466545	3.233513	3.364758	35.19262
3.401463	3.296324	3.356266	35.16908
3.172068	3.263975	3.488723	34.88635
3.245151	3.380701	3.334696	34.9646
3.279919	3.412982	3.380343	35.21105
3.434128	3.455036	3.305333	35.47621
3.251172	3.452677	3.430611	35.34492
3.141424	3.419587	3.461713	35.10057
3.256245	3.379555	3.390506	35.1084
3.405505	3.324225	3.398571	35.33145
3.315883	3.285608	3.353538	34.95253
3.232365	3.239713	3.367589	34.70025
3.277816	3.289315	3.424149	35.0318
3.250801	3.417633	3.379269	35.15519
3.394947	3.407658	3.353896	35.39312
3.255915	3.324427	3.408529	35.02654
3.228695	3.369446	3.434874	35.12307
3.126329	3.390001	3.467255	35.01498
3.320296	3.467369	3.412127	35.48779
3.216528	3.325977	3.416646	34.96154
3.25674	3.202445	3.373949	34.68596
3.155158	3.220843	3.509389	34.80024
3.419033	3.268153	3.352856	35.13844
3.288951	3.294841	3.415009	35.04825
3.381378	3.365133	3.382458	35.33291
3.267711	3.384273	3.429196	35.2284
3.285528	3.4115	3.397122	35.25676
3.199453	3.43502	3.388119	35.10028
3.341743	3.451195	3.363036	35.39196
3.158292	3.412646	3.427542	35.04755
3.246594	3.365537	3.402868	35.08368

3.196649	3.28635	3.422921	34.84513
3.363643	3.233917	3.387914	35.01911
3.424065	3.242274	3.344603	35.0748
3.285693	3.257033	3.423995	34.9781
3.330236	3.41305	3.424473	35.41773
3.319678	3.425922	3.410149	35.39147
3.285239	3.351722	3.380701	35.0895
3.333206	3.368098	3.386703	35.24333
3.296499	3.451127	3.429588	35.43841
3.161757	3.444321	3.482653	35.24491
3.257029	3.351182	3.451704	35.1819
3.222921	3.201231	3.43438	34.7415
3.294148	3.218147	3.403943	34.8677
3.241893	3.236546	3.40089	34.78698
3.302067	3.26074	3.397872	34.96488
3.25208	3.387575	3.324687	34.97289
3.384842	3.351654	3.338345	35.21454
3.283672	3.376388	3.360529	35.0959
3.290271	3.419385	3.386635	35.26145
3.214713	3.428146	3.361331	35.06004
3.297571	3.46865	3.385885	35.3835
3.25175	3.377196	3.37354	35.05631
3.279177	3.318631	3.388681	35.02133
3.224777	3.273814	3.450886	34.94039
3.290477	3.317216	3.449027	35.17491
3.247584	3.283182	3.410644	34.92275
3.399195	3.383194	3.432811	35.52148
3.327679	3.397617	3.449624	35.43339
3.360096	3.344241	3.379525	35.23426
3.214301	3.358461	3.386772	34.96238
3.22853	3.399234	3.352464	35.00763
3.250183	3.41925	3.443707	35.29829
3.272536	3.460023	3.364792	35.26377
3.162788	3.288978	3.488604	34.92047
3.200031	3.200086	3.378297	34.5663
3.282311	3.24679	3.463964	35.03571
3.363231	3.29747	3.343734	35.06057
3.29918	3.297267	3.424404	35.09647
3.329576	3.402199	3.370453	35.27443
3.343558	3.352665	3.414174	35.29229
3.398453	3.390473	3.294864	35.23411
3.310728	3.484083	3.362985	35.39595
3.187658	3.413589	3.393183	35.03869
3.284827	3.376253	3.386721	35.1554
3.231128	3.293493	3.406552	34.90036
3.283961	3.296796	3.331456	34.8589
3.21195	3.303939	3.398861	34.86445
3.248533	3.360011	3.418283	35.10954
3.172109	3.348554	3.452164	34.99145
3.433386	3.40995	3.360853	35.4974

3.288044	3.355361	3.420483	35.19058
3.340753	3.33831	3.348713	35.11161
3.293983	3.390136	3.421216	35.28122
3.309614	3.403547	3.387164	35.27027
3.155818	3.394045	3.404846	34.95183
3.277857	3.284261	3.328165	34.81094
3.136474	3.207432	3.497743	34.70458
3.198546	3.216665	3.400959	34.64886
3.213105	3.285878	3.442837	34.92364
3.354858	3.346061	3.438472	35.3557
3.358282	3.412039	3.38493	35.39038
3.310934	3.373759	3.408598	35.25489
3.28693	3.360213	3.407097	35.16949
3.369046	3.397819	3.383907	35.38059
3.276455	3.488329	3.346018	35.29318
3.251997	3.40321	3.379047	35.12578
3.193927	3.327595	3.353044	34.77657
3.20135	3.236613	3.421744	34.74408
3.301572	3.304411	3.430151	35.12989
3.260122	3.324966	3.411718	35.04389
3.301036	3.322675	3.41888	35.14401
3.260246	3.346465	3.426468	35.12343
3.283425	3.390271	3.39702	35.20552
3.404845	3.351452	3.277761	35.12535
3.400597	3.382251	3.425172	35.50578
3.172397	3.416285	3.412809	35.05413
3.279424	3.406513	3.430765	35.30608
3.177223	3.382116	3.397071	34.95555
3.224488	3.254877	3.378382	34.73979
3.199866	3.208173	3.364877	34.55428
3.190132	3.213295	3.482516	34.80145
3.143527	3.36547	3.426451	34.90971
3.434417	3.36082	3.396099	35.46929
3.293241	3.373557	3.38343	35.16071
3.428437	3.363179	3.350759	35.36222
3.245233	3.367896	3.418829	35.12076
3.358694	3.474513	3.347519	35.44609
3.270227	3.478691	3.375313	35.32255
3.338237	3.397886	3.288128	35.10391
3.190215	3.253731	3.416782	34.74631
3.224735	3.281834	3.379661	34.80208
3.210754	3.305894	3.439649	34.9553
3.329164	3.354754	3.397054	35.22794
3.370902	3.36365	3.485858	35.53288
3.280661	3.358259	3.421813	35.18369
3.265896	3.364324	3.439086	35.20243
3.341702	3.362976	3.32259	35.1105
3.380264	3.426933	3.420056	35.54784
3.267257	3.35927	3.398844	35.10636
3.241274	3.32281	3.419272	35.01447

3.188606	3.266064	3.435181	34.81
3.309078	3.296324	3.339351	34.93006
3.177017	3.245913	3.411377	34.68853
3.27299	3.303737	3.419784	35.04324
3.17017	3.356304	3.449232	34.99775
3.327019	3.35408	3.391273	35.20914
3.385461	3.379083	3.392228	35.39371
3.370036	3.378005	3.350333	35.266
3.244079	3.393371	3.440791	35.22197
3.349208	3.430909	3.352447	35.34077
3.30714	3.324832	3.354323	35.0209
3.352714	3.283721	3.288879	34.88755
3.208733	3.278734	3.427337	34.86456
3.107192	3.322405	3.372977	34.61913
3.187286	3.374501	3.436733	35.04764
3.303345	3.416757	3.400123	35.31378
3.320874	3.343634	3.449982	35.30124
3.303428	3.337906	3.423808	35.19333
3.266309	3.368368	3.466164	35.27139
3.342114	3.444927	3.357972	35.36799
3.370613	3.422081	3.335481	35.33117
3.269897	3.347543	3.395485	35.07914
3.240408	3.255955	3.379934	34.78035
3.172026	3.252788	3.407916	34.68508
3.279218	3.248474	3.410934	34.91666
3.228612	3.311488	3.397361	34.91411
3.261937	3.34296	3.393047	35.04638
3.158498	3.348419	3.493446	35.05167
3.335639	3.468043	3.389346	35.47299
3.351518	3.382655	3.388187	35.31845
3.440892	3.38434	3.350674	35.43555
3.255874	3.379488	3.434039	35.20264
3.354363	3.37814	3.434226	35.41548
3.315718	3.270512	3.398384	35.01723
3.337495	3.248946	3.285417	34.77065
3.152312	3.19557	3.376745	34.44868
3.239212	3.349295	3.445702	35.12568
3.178955	3.420261	3.458712	35.17755
3.350115	3.401863	3.398026	35.37891
3.316048	3.361494	3.473513	35.38121
3.36917	3.334806	3.391	35.25857
3.321245	3.460562	3.413901	35.47885
3.310398	3.46346	3.40072	35.43265
3.308501	3.349026	3.389023	35.15267
3.291261	3.287563	3.357562	34.91176
3.168521	3.235063	3.369635	34.55494
3.132103	3.194088	3.400669	34.45356
3.297612	3.246318	3.408035	34.94583
3.181265	3.289787	3.419511	34.81155
3.277032	3.35098	3.377683	35.06333

3.266515	3.384475	3.462412	35.29887
3.258926	3.389193	3.431038	35.22397
3.281239	3.368503	3.349548	35.04932
3.449182	3.43374	3.404915	35.68032
3.317286	3.466089	3.446657	35.55391
3.357415	3.42909	3.380497	35.41608
3.302892	3.236209	3.332667	34.77045
3.316914	3.205477	3.370828	34.81736
3.246141	3.32409	3.30402	34.77588
3.283961	3.368772	3.477281	35.33519
3.205392	3.393371	3.380497	35.00552
3.278682	3.288978	3.3991	34.97818
3.255544	3.298346	3.393643	34.93614
3.403031	3.423429	3.455984	35.66853
3.333247	3.42855	3.419187	35.44666
3.357003	3.410826	3.416561	35.4541
3.254389	3.303872	3.465465	35.10276
3.311511	3.266873	3.298308	34.78122
3.308336	3.249822	3.404693	34.96963
3.216363	3.299694	3.372039	34.80616
3.057535	3.293561	3.44473	34.60438
3.205557	3.331369	3.37666	34.8619
3.239789	3.421272	3.442002	35.27625
3.383894	3.405637	3.354902	35.36672
3.273279	3.368435	3.448822	35.24886
3.308047	3.32928	3.367043	35.06043
3.390121	3.392158	3.427133	35.50882
3.452317	3.420126	3.249575	35.31771
3.31312	3.241735	3.347331	34.83697
3.211537	3.233446	3.320458	34.53793
3.258514	3.320923	3.385868	34.975
3.249729	3.42727	3.340357	35.08883
3.358034	3.446005	3.363189	35.41658
3.206382	3.319373	3.491178	35.0879
3.167861	3.310477	3.422103	34.83315
3.286106	3.406108	3.440791	35.34173
3.479413	3.51131	3.334799	35.76274
3.292622	3.395123	3.461099	35.37637
3.292003	3.261414	3.384248	34.91456
3.232283	3.169489	3.435403	34.6948
3.392802	3.292954	3.333008	35.09191
3.273774	3.280958	3.371903	34.89043
3.239047	3.303603	3.387965	34.89914
3.113213	3.262425	3.43409	34.63478
3.235871	3.370929	3.378638	35.01903
3.309573	3.446342	3.429349	35.45602
3.370448	3.425585	3.359626	35.39127
3.243914	3.334064	3.479055	35.17559
3.280167	3.368098	3.379405	35.11138
3.340629	3.483611	3.46705	35.68788

3.412723	3.348554	3.362678	35.32194
3.313079	3.224819	3.331047	34.76428
3.241356	3.199951	3.369004	34.63605
3.095808	3.369581	3.380752	34.71441
3.297406	3.436975	3.420449	35.38946
3.302397	3.429898	3.401828	35.34418
3.283878	3.345926	3.421267	35.16256
3.135773	3.359404	3.427013	34.88072
3.352879	3.457395	3.39557	35.50102
3.446254	3.432864	3.418215	35.70109
3.322977	3.314049	3.307311	34.92915
3.286642	3.219495	3.411991	34.87183
3.226426	3.300907	3.369379	34.825
3.310398	3.277184	3.424814	35.07798
3.244161	3.260201	3.332906	34.695
3.304706	3.246048	3.379525	34.8984
3.138578	3.315329	3.445207	34.83025
3.230509	3.401121	3.384913	35.08705
3.316667	3.393977	3.388323	35.2673
3.257359	3.402941	3.435914	35.26128
3.181595	3.404356	3.37088	34.95647
3.420642	3.411298	3.445838	35.65833
3.349744	3.326584	3.365219	35.14173
3.416641	3.275297	3.28864	35.0084
3.336216	3.21343	3.409194	34.96086
3.289982	3.369716	3.352328	35.07717
3.257813	3.466628	3.381724	35.28304
3.276537	3.437244	3.348985	35.18813
3.205392	3.32436	3.402357	34.90241
3.272536	3.346128	3.379167	35.04613
3.22156	3.445534	3.4973	35.41037
3.301696	3.493181	3.412895	35.50523
3.291632	3.304344	3.357255	34.9486
3.334608	3.235737	3.413628	35.01582
3.279259	3.264042	3.343682	34.80372
3.316172	3.312903	3.371374	35.05185
3.221766	3.267951	3.401845	34.81374
3.219332	3.271186	3.351748	34.70593
3.223333	3.279813	3.446503	34.94076
3.3151	3.363313	3.323101	35.05418
3.243171	3.345993	3.478015	35.19778
3.247584	3.420935	3.42599	35.25755
3.287095	3.377264	3.425564	35.24752
3.339433	3.439468	3.41356	35.47175
3.395442	3.309466	3.367452	35.20911
3.316873	3.299357	3.365884	35.01176
3.25872	3.246385	3.392501	34.82695
3.177677	3.370457	3.369925	34.87168
3.405629	3.463191	3.354834	35.53997
3.242882	3.403547	3.386772	35.12348

3.22985	3.321597	3.415589	34.97878
3.190833	3.319575	3.419852	34.89836
3.32805	3.456586	3.355516	35.35736
3.254472	3.44486	3.430697	35.33522
3.375563	3.38744	3.34121	35.27877
3.158663	3.166524	3.455166	34.57054
3.383976	3.220708	3.313706	34.8724
3.372882	3.292213	3.396508	35.18559
3.39672	3.362976	3.381025	35.35861
3.247048	3.294572	3.395417	34.91318
3.238717	3.27368	3.383089	34.82232
3.176522	3.332447	3.362217	34.76918
3.409712	3.388519	3.385902	35.45354
3.197102	3.415678	3.42142	35.12566
3.135815	3.342084	3.447663	34.88809
3.228571	3.339321	3.435471	35.05823
3.394617	3.354687	3.342301	35.2512
3.397628	3.280217	3.316144	35.03773
3.302067	3.209117	3.376046	34.80426
3.17945	3.309601	3.426229	34.8656
3.249234	3.438525	3.39303	35.22754
3.360302	3.425181	3.26782	35.16744
3.340299	3.334806	3.406671	35.2297
3.187781	3.316744	3.452523	34.95694
3.132845	3.46036	3.411786	35.06179
3.317904	3.54905	3.39835	35.63105
3.305861	3.42235	3.281171	35.07139
3.277857	3.216058	3.507019	35.05292
3.145177	3.227717	3.361263	34.46952
3.295055	3.321125	3.479924	35.26104
3.299881	3.448701	3.312086	35.18354
3.403732	3.308253	3.400345	35.29652
3.138867	3.265255	3.394939	34.61145
3.23286	3.266132	3.390438	34.80907
3.286353	3.391147	3.389756	35.19795
3.340918	3.470335	3.319162	35.33607
3.163283	3.382251	3.437517	35.01381
3.251709	3.342084	3.433425	35.11039
3.195453	3.373759	3.440041	35.07111
3.451698	3.311892	3.323937	35.24228
3.333494	3.238568	3.401504	34.99307
3.348713	3.331301	3.392978	35.2105
3.219085	3.39701	3.443178	35.18049
3.341784	3.400649	3.286304	35.11372
3.29489	3.322338	3.320629	34.91498
3.409547	3.348217	3.302707	35.18311
3.148683	3.372816	3.47324	35.03937
3.145383	3.539952	3.421216	35.28388
3.242346	3.464673	3.516483	35.53964
3.286807	3.259123	3.418658	34.97343

3.294189	3.162952	3.355397	34.64092
3.274887	3.214778	3.49007	35.00656
3.269484	3.439064	3.430765	35.35553
3.326689	3.376994	3.382799	35.23999
3.329246	3.209319	3.306817	34.71275
3.265401	3.263705	3.460622	35.02841
3.253152	3.347139	3.30402	34.84162
3.284208	3.361224	3.42258	35.1996
3.331391	3.486442	3.36793	35.4571
3.2501	3.410152	3.415009	35.21546
3.309862	3.375107	3.380752	35.19459
3.224323	3.32591	3.415879	34.97676
3.381667	3.270445	3.314456	34.97776
3.323307	3.234726	3.423313	35.01008
3.345496	3.358798	3.391393	35.26012
3.260328	3.397684	3.413372	35.20698
3.386863	3.397954	3.337936	35.31931
3.273609	3.329549	3.345899	34.93947
3.275382	3.386429	3.421352	35.23274
3.200855	3.470874	3.400055	35.20785
3.20898	3.4733	3.489422	35.42636
3.14031	3.285676	3.366634	34.59736
3.289075	3.217136	3.40546	34.85771
3.287343	3.224011	3.457143	34.98198
3.353374	3.384745	3.41373	35.38294
3.229148	3.401323	3.459599	35.24784
3.265401	3.292887	3.309409	34.76155
3.357415	3.287832	3.375722	35.09673
3.317987	3.369648	3.334781	35.09989
3.214053	3.352935	3.374631	34.9232
3.25076	3.410893	3.405614	35.19797
3.21958	3.375849	3.429384	35.10513
3.325081	3.338782	3.363752	35.11126
3.391689	3.359068	3.341739	35.25314
3.236448	3.266738	3.401896	34.8433
3.271959	3.284463	3.424575	35.00932
3.242305	3.382992	3.385817	35.07518
3.397133	3.429292	3.435676	35.62404
3.317368	3.424305	3.335651	35.21997
3.383894	3.331638	3.429605	35.36826
3.240862	3.294572	3.360563	34.82343
3.345785	3.41588	3.385459	35.37261
3.175573	3.503694	3.402578	35.22986
3.283054	3.402873	3.384981	35.20594
3.124101	3.177172	3.428633	34.46022
3.217353	3.18809	3.317065	34.44404
3.345744	3.319305	3.443434	35.28811
3.339887	3.439536	3.367606	35.3724
3.239418	3.33211	3.431242	35.05693
3.302232	3.271388	3.348286	34.88009

3.293529	3.19557	3.368748	34.74001
3.434829	3.3559	3.28124	35.20826
3.186709	3.411095	3.391802	35.02814
3.109996	3.472828	3.396662	35.00601
3.256452	3.343634	3.411582	35.07639
3.310109	3.274893	3.318497	34.83984
3.31081	3.259392	3.411582	35.01104
3.259462	3.251507	3.400447	34.85715
3.178172	3.371401	3.440297	35.02872
3.36917	3.36675	3.372994	35.28905
3.479867	3.395864	3.338345	35.51902
3.325658	3.36884	3.34312	35.13314
3.33135	3.334873	3.354288	35.09573
3.211909	3.350374	3.360444	34.88189
3.324462	3.464202	3.3991	35.46148
3.267711	3.384408	3.386703	35.13577
3.291343	3.184585	3.389551	34.7567
3.055967	3.136668	3.438404	34.24401
3.261566	3.271793	3.404147	34.91421
3.27431	3.445062	3.450869	35.42316
3.331432	3.43839	3.390574	35.40163
3.249358	3.244835	3.494179	35.02544
3.335226	3.207364	3.409689	34.94652
3.304871	3.335412	3.383259	35.10236
3.400226	3.388788	3.276823	35.19485
3.186874	3.429022	3.41315	35.11439
3.299798	3.434414	3.415145	35.37749
3.125916	3.213767	3.345746	34.36295
3.340011	3.223	3.337305	34.83288
3.247419	3.310544	3.383907	34.92375
3.285817	3.360618	3.392501	35.13602
3.251709	3.417363	3.386567	35.17255
3.421838	3.382857	3.352123	35.39381
3.41693	3.363381	3.402596	35.45086
3.413259	3.360618	3.314251	35.24359
3.24412	3.403413	3.408478	35.17336
3.157302	3.401391	3.409399	34.9811
3.320626	3.424911	3.412826	35.39719
3.216363	3.279206	3.393661	34.80863
3.216693	3.173802	3.337646	34.45636
3.209723	3.278465	3.467102	34.9531
3.201185	3.424102	3.47174	35.26306
3.290065	3.475119	3.475235	35.57663
3.33766	3.299087	3.369908	35.06542
3.266927	3.203455	3.409126	34.78738
3.366242	3.307916	3.405989	35.22614
3.303222	3.416554	3.395434	35.30282
3.307511	3.483409	3.357648	35.37577
3.275877	3.444927	3.353623	35.21363
3.294602	3.264312	3.36058	34.87482

3.167737	3.198266	3.34699	34.42324
3.311841	3.263571	3.353265	34.8949
3.232159	3.339254	3.449726	35.0971
3.246099	3.449645	3.406875	35.27528
3.310398	3.401054	3.453955	35.41259
3.347599	3.351452	3.379525	35.22271
3.367974	3.299155	3.339624	35.06564
3.341124	3.372007	3.462873	35.43577
3.232778	3.452475	3.357307	35.14394
3.346898	3.455171	3.380855	35.4509
3.124308	3.332447	3.390847	34.7176
3.194999	3.205612	3.350196	34.50593
3.177429	3.255888	3.376882	34.63581
3.221436	3.399908	3.43588	35.17601
3.226261	3.466156	3.536263	35.55096
3.290312	3.369109	3.400328	35.18153
3.345702	3.232839	3.427474	35.06403
3.413506	3.291202	3.319793	35.10445
3.305655	3.342758	3.453887	35.27459
3.239583	3.381914	3.341329	34.96958
3.246058	3.406715	3.45034	35.27636
3.218673	3.363179	3.383907	34.97599
3.332009	3.232839	3.354988	34.87557
3.285446	3.247194	3.354902	34.80494
3.201722	3.332245	3.397258	34.90047
3.192359	3.401863	3.494197	35.24423
3.328545	3.420328	3.414924	35.40907
3.329246	3.34323	3.347604	35.09479
3.379646	3.3034	3.34271	35.1072
3.242717	3.33184	3.456956	35.11979
3.350816	3.448634	3.281581	35.22807
3.362035	3.429898	3.416066	35.50573
3.291591	3.398628	3.293841	35.01601
3.158581	3.183979	3.370948	34.42436
3.201804	3.179396	3.307567	34.37026
3.107893	3.411702	3.484375	35.05955
3.307552	3.47458	3.376251	35.39723
3.222013	3.395056	3.492611	35.29072
3.263504	3.316273	3.464731	35.1482
3.232984	3.260942	3.417311	34.85676
3.38476	3.335345	3.351697	35.20789
3.31844	3.394112	3.423501	35.3484
3.267299	3.353137	3.390233	35.0742
3.196607	3.365807	3.415538	35.00266
3.255132	3.227178	3.329257	34.6388
3.377501	3.234524	3.317798	34.89741
3.244079	3.295178	3.355789	34.82135
3.185802	3.375916	3.404505	34.97701
3.231004	3.4084	3.453614	35.25429
3.406041	3.475254	3.327211	35.50684

3.383605	3.319912	3.42142	35.32409
3.331432	3.347139	3.337442	35.08589
3.262968	3.382723	3.392706	35.13484
3.301531	3.410826	3.383174	35.25978
3.283837	3.37659	3.359489	35.09443
3.379646	3.266334	3.35272	35.04803
3.086776	3.15628	3.348013	34.15661
3.187039	3.2941	3.407148	34.80657
3.244697	3.410017	3.373932	35.11352
3.252245	3.478691	3.432879	35.40911
3.197927	3.394517	3.394615	35.02257
3.313326	3.261347	3.430833	35.06291
3.236325	3.283924	3.383958	34.84139
3.402494	3.349902	3.40089	35.38609
3.321327	3.404356	3.415401	35.3594
3.340506	3.377736	3.395826	35.30032
3.237026	3.314049	3.38019	34.90056
3.224117	3.227582	3.398503	34.72329
3.32042	3.28143	3.338686	34.92083
3.273073	3.398358	3.36527	35.13113
3.175573	3.410152	3.454688	35.13925
3.292127	3.457597	3.374631	35.32282
3.331061	3.311757	3.409348	35.16495
3.459823	3.409411	3.30402	35.42975
3.309944	3.405974	3.395553	35.29464
3.226756	3.42909	3.357852	35.08083
3.261607	3.36675	3.414515	35.14463
3.266927	3.276915	3.38418	34.89347
3.253193	3.167669	3.353061	34.55648
3.270557	3.223943	3.381298	34.77926
3.119812	3.458136	3.484034	35.18642
3.241191	3.477006	3.46173	35.44435
3.328009	3.442097	3.404983	35.43376
3.264948	3.323281	3.345439	34.90581
3.324379	3.23412	3.40442	34.96978
3.249646	3.32281	3.425155	35.04565
3.302974	3.450925	3.405187	35.39877
3.354817	3.46555	3.404437	35.54248
3.413135	3.328471	3.340255	35.22989
3.227664	3.160054	3.419289	34.62883
3.271134	3.160997	3.34005	34.55267
3.158993	3.319305	3.41385	34.81502
3.261236	3.465887	3.360751	35.24304
3.254266	3.486576	3.445975	35.45941
3.226468	3.45234	3.419647	35.26618
3.264783	3.315599	3.405989	35.02107
3.421136	3.385149	3.399168	35.50017
3.265484	3.447623	3.401146	35.30073
3.351476	3.469121	3.393183	35.51838
3.247543	3.220573	3.414804	34.79484

3.230633	3.142936	3.348167	34.44235
3.326978	3.186877	3.368953	34.79459
3.256864	3.38097	3.336623	34.99501
3.262143	3.505244	3.452778	35.53234
3.182667	3.393843	3.40517	35.01081
3.267917	3.369514	3.460622	35.2653
3.268453	3.309061	3.360495	34.91531
3.309903	3.319238	3.478083	35.28535
3.175326	3.404423	3.45346	35.1235
3.271918	3.406243	3.384436	35.18776
3.311676	3.351519	3.383805	35.15366
3.458957	3.208847	3.34462	35.07804
3.265443	3.175285	3.399594	34.70168
3.15392	3.249148	3.366787	34.54758
3.173594	3.362707	3.454279	35.03027
3.329576	3.487924	3.356676	35.43177
3.292828	3.451195	3.380019	35.32213
3.387688	3.408063	3.389977	35.45703
3.182131	3.337165	3.492611	35.07691
3.350074	3.504571	3.40916	35.62777
3.332669	3.467234	3.460298	35.61989
3.419404	3.358124	3.363155	35.35852
3.248162	3.096906	3.444218	34.59007
3.236696	3.171241	3.357562	34.53806
3.243914	3.316003	3.372022	34.90203
3.356137	3.49682	3.394087	35.59112
3.218301	3.418509	3.435181	35.20831
3.077001	3.324629	3.452846	34.73264
3.201928	3.332986	3.441865	35.00009
3.305119	3.290259	3.377001	34.99047
3.304871	3.395258	3.399594	35.26895
3.21859	3.398762	3.474246	35.25118
3.218219	3.366885	3.378775	34.97188
3.298355	3.289989	3.319793	34.84998
3.49389	3.244431	3.338754	35.21942
3.250471	3.198401	3.405187	34.73172
3.204155	3.294437	3.43409	34.90366
3.188029	3.453149	3.418164	35.18064
3.363891	3.490553	3.310858	35.41236
3.351394	3.410489	3.355874	35.30839
3.305861	3.350239	3.415623	35.20772
3.177017	3.376522	3.417038	34.98653
3.297159	3.441692	3.392586	35.3383
3.311347	3.384812	3.408171	35.27903
3.420972	3.283856	3.404778	35.29056
3.2355	3.17825	3.407694	34.6604
3.257359	3.193751	3.445122	34.82395
3.298355	3.389327	3.364741	35.16551
3.298685	3.437581	3.40928	35.36916
3.20564	3.3435	3.382543	34.90147

3.176563	3.347476	3.436903	34.96546
3.127648	3.302052	3.436153	34.75752
3.304995	3.365403	3.365815	35.13006
3.325204	3.353811	3.419971	35.26734
3.341743	3.400649	3.407438	35.37853
3.242305	3.326045	3.433016	35.05386
3.363726	3.199075	3.335071	34.82753
3.349992	3.154056	3.289714	34.59986
3.312501	3.309735	3.341534	34.97164
3.103933	3.41278	3.460827	35.00176
3.285569	3.454295	3.341739	35.22932
3.308666	3.374972	3.422375	35.28271
3.327844	3.403682	3.328183	35.18145
3.309779	3.36082	3.464186	35.34563
3.166252	3.474985	3.401777	35.14494
3.267505	3.452677	3.455575	35.43522
3.301984	3.243892	3.360308	34.84571
3.345084	3.1798	3.333162	34.74044
3.190998	3.239983	3.418215	34.72109
3.266309	3.369716	3.414191	35.16068
3.300417	3.457799	3.433084	35.46921
3.362158	3.414465	3.318412	35.2587
3.171201	3.321597	3.423893	34.86869
3.185183	3.371266	3.383293	34.9191
3.193184	3.340804	3.469011	35.05743
3.221518	3.367559	3.380838	34.98508
3.245068	3.357383	3.435198	35.13321
3.325163	3.357652	3.379422	35.18698
3.277486	3.254136	3.450869	35.01258
3.36851	3.236815	3.300985	34.84599
3.295014	3.169287	3.426928	34.81301
3.297571	3.381038	3.320271	35.04842
3.227911	3.411972	3.394291	35.12561
3.229561	3.462112	3.316229	35.06815
3.31477	3.381914	3.423995	35.31478
3.281651	3.435222	3.368902	35.23845
3.29254	3.402469	3.471723	35.41549
3.279465	3.451195	3.392296	35.31976
3.323555	3.339793	3.368612	35.12076
3.298231	3.143812	3.409621	34.72649
3.393091	3.194896	3.352259	34.9202
3.266597	3.283115	3.406279	34.95463
3.346238	3.469391	3.360154	35.43528
3.28095	3.430168	3.446691	35.39598
3.298726	3.394921	3.366156	35.18165
3.256163	3.292348	3.38684	34.90949
3.31213	3.381307	3.387982	35.22892
3.10847	3.334604	3.482175	34.8874
3.241769	3.358461	3.427474	35.11146
3.18011	3.344106	3.460145	35.01667

3.35923	3.263166	3.353743	34.99869
3.261566	3.238231	3.411582	34.85707
3.375563	3.227313	3.401845	35.0612
3.197556	3.297672	3.4146	34.85368
3.332422	3.443107	3.362558	35.35285
3.230963	3.455238	3.407489	35.25576
3.410289	3.43502	3.346513	35.47036
3.185719	3.395595	3.367111	34.93809
3.251585	3.402334	3.414054	35.19952
3.29984	3.459012	3.348627	35.28591
3.362736	3.411769	3.451073	35.54418
3.327761	3.261077	3.431907	35.09624
3.219827	3.141858	3.429776	34.59483
3.298561	3.223606	3.39453	34.8687
3.359106	3.428753	3.348508	35.34909
3.258019	3.442366	3.390318	35.24923
3.210424	3.396201	3.406262	35.07905
3.259545	3.334401	3.396167	35.02925
3.235706	3.41433	3.400396	35.16116
3.229355	3.404828	3.423177	35.17631
3.220281	3.351789	3.45491	35.10987
3.169016	3.337704	3.506866	35.08058
3.206176	3.264986	3.431362	34.83771
3.419033	3.252653	3.381025	35.16614
3.257111	3.212891	3.326665	34.60622
3.308418	3.208173	3.41344	34.89786
3.210713	3.385486	3.413833	35.07281
3.391771	3.445062	3.413713	35.59878
3.339145	3.487789	3.290106	35.30682
3.393627	3.429764	3.354152	35.43913
3.07197	3.377129	3.369635	34.65447
3.257524	3.4733	3.413935	35.36744
3.213806	3.452408	3.455387	35.31679
3.355023	3.300772	3.386601	35.14359
3.160849	3.12164	3.443724	34.45214
3.253358	3.189101	3.436545	34.78627
3.282146	3.347745	3.43056	35.18307
3.379893	3.471345	3.403329	35.60757
3.323843	3.374635	3.395724	35.25688
3.297241	3.305557	3.415487	35.09086
3.117296	3.294302	3.432316	34.70954
3.263793	3.448297	3.367947	35.2259
3.193968	3.433605	3.412929	35.13944
3.2574	3.376725	3.384095	35.09072
3.218054	3.238433	3.48961	34.93299
3.311841	3.216799	3.33635	34.75563
3.374573	3.191864	3.401214	34.98013
3.34133	3.296324	3.339232	35.00033
3.271258	3.350509	3.483198	35.28041
3.24478	3.412106	3.422989	35.22555

3.360921	3.427944	3.423859	35.51607
3.277651	3.422081	3.33635	35.12978
3.36653	3.423159	3.388323	35.44016
3.150373	3.389193	3.412008	34.94497
3.194504	3.348621	3.363906	34.84756
3.287467	3.332986	3.464595	35.23686
3.305614	3.241398	3.375569	34.88157
3.173965	3.15601	3.39037	34.43931
3.293364	3.302996	3.430697	35.11004
3.268577	3.464673	3.501478	35.56419
3.401381	3.447016	3.345558	35.47502
3.282847	3.361426	3.412485	35.175
3.346651	3.335615	3.336759	35.09248
3.257318	3.372951	3.425939	35.17379
3.278476	3.449442	3.38123	35.28956
3.132061	3.359404	3.461458	34.94792
3.278888	3.336491	3.369243	35.01724
3.242676	3.274084	3.464663	35.01025
3.222591	3.226774	3.406023	34.73463
3.305737	3.275095	3.406398	35.02295
3.335556	3.300368	3.459275	35.25906
3.327102	3.346937	3.367538	35.14179
3.266845	3.392293	3.383225	35.14351
3.306933	3.405097	3.372534	35.2358
3.296416	3.382386	3.378365	35.17589
3.37049	3.428079	3.382679	35.44723
3.220199	3.407928	3.462464	35.24898
3.26767	3.39728	3.398486	35.1896
3.182543	3.284867	3.402323	34.766
3.364262	3.179868	3.348048	34.81508
3.234469	3.234254	3.415896	34.79855
3.275671	3.410758	3.42906	35.30343
3.286724	3.421946	3.399731	35.28793
3.352301	3.39081	3.38077	35.32178
3.314192	3.292483	3.354578	34.96614
3.387111	3.348217	3.33207	35.19826
3.166541	3.39081	3.39569	34.94818
3.246099	3.400986	3.429384	35.2181
3.11478	3.330156	3.417379	34.74978
3.236201	3.278465	3.432538	34.93542
3.255297	3.223134	3.364877	34.70821
3.329906	3.237085	3.432811	35.05044
3.180564	3.354822	3.449777	35.01843
3.335268	3.343567	3.425376	35.27877
3.365499	3.373962	3.384998	35.32304
3.313986	3.399773	3.358534	35.20897
3.320585	3.405434	3.343358	35.20259
3.266969	3.393169	3.386141	35.15208
3.313739	3.447084	3.3607	35.31662
3.311594	3.389462	3.342233	35.14554

3.217806	3.307444	3.427201	34.94689
3.247708	3.246857	3.354442	34.72067
3.255668	3.271321	3.447867	34.99589
3.382945	3.387036	3.382185	35.38363
3.340506	3.468717	3.398759	35.50569
3.292086	3.40375	3.370198	35.19528
3.285033	3.314992	3.446094	35.15173
3.27662	3.286619	3.316826	34.78859
3.320008	3.343028	3.394752	35.17724
3.276826	3.462921	3.402578	35.36212
3.209063	3.31324	3.450135	34.9906
3.140516	3.249216	3.395229	34.58062
3.312295	3.27058	3.401078	35.01578
3.236696	3.235063	3.387846	34.74385
3.330277	3.306568	3.402459	35.13682
3.192236	3.373625	3.46908	35.12728
3.317079	3.372479	3.362882	35.16555
3.418827	3.394382	3.340391	35.38677
3.477104	3.437177	3.357443	35.64509
3.259998	3.39108	3.418061	35.20207
3.296086	3.398965	3.392433	35.24219
3.197515	3.35125	3.443707	35.03441
3.276125	3.314655	3.34462	34.9096
3.209764	3.287024	3.421762	34.87275
3.181636	3.275971	3.444065	34.83584
3.13499	3.323753	3.447816	34.84653
3.389338	3.415206	3.354971	35.39971
3.325411	3.411837	3.462395	35.48746
3.340052	3.419857	3.374102	35.34393
3.30252	3.34815	3.444338	35.25864
3.2574	3.368637	3.405852	35.12061
3.39771	3.388451	3.373813	35.40071
3.2753	3.373625	3.406688	35.17249
3.244738	3.232772	3.407131	34.7986
3.157467	3.234457	3.420994	34.64175
3.281321	3.244363	3.401487	34.89161
3.247955	3.297133	3.424609	34.98461
3.291055	3.367963	3.406756	35.19471
3.219374	3.351924	3.503541	35.21453
3.311058	3.381105	3.345899	35.13411
3.417218	3.444051	3.345098	35.50216
3.415197	3.437109	3.33485	35.46015
3.254389	3.373153	3.429861	35.1764
3.213806	3.376253	3.426178	35.08638
3.223374	3.309264	3.386635	34.87434
3.352301	3.307174	3.30165	34.96586
3.153508	3.247666	3.413014	34.64453
3.257235	3.255281	3.446367	34.96096
3.182337	3.411972	3.469864	35.19121
3.367108	3.435896	3.429043	35.55832

3.404845	3.409006	3.366156	35.44452
3.439407	3.379757	3.437909	35.61305
3.288168	3.325438	3.433612	35.15413
3.333041	3.359068	3.359336	35.16337
3.245852	3.37996	3.374529	35.05162
3.340011	3.325573	3.360052	35.10693
3.232984	3.20851	3.360171	34.61715
3.097417	3.232839	3.481493	34.6392
3.259297	3.278599	3.4013	34.9179
3.310109	3.380229	3.392552	35.23214
3.282394	3.360752	3.444253	35.242
3.284126	3.336828	3.400191	35.09711
3.290684	3.352396	3.480197	35.32046
3.334195	3.438996	3.347263	35.31429
3.491044	3.455508	3.357221	35.71518
3.270103	3.402873	3.393302	35.19581
3.259545	3.316744	3.357409	34.90588
3.232283	3.270984	3.364178	34.761
3.321039	3.28608	3.38476	35.03311
3.235871	3.33521	3.381997	34.94826
3.263339	3.351924	3.426058	35.14124
3.160684	3.398358	3.448396	35.06714
3.380182	3.391753	3.400328	35.42758
3.31477	3.374905	3.412468	35.27424
3.442748	3.410422	3.4161	35.63972
3.293406	3.359404	3.437227	35.24777
3.260617	3.417835	3.413577	35.25212
3.29221	3.296931	3.395826	35.018
3.311223	3.21653	3.392961	34.87749
3.236985	3.234726	3.380429	34.72752
3.192565	3.258179	3.371272	34.66165
3.129834	3.299828	3.474553	34.84141
3.309738	3.412646	3.360171	35.23141
3.309573	3.395528	3.44652	35.38244
3.412599	3.394112	3.39985	35.50259
3.307057	3.406917	3.447322	35.4036
3.352714	3.453756	3.337032	35.36469
3.380347	3.396741	3.347348	35.32299
3.309573	3.354552	3.354766	35.09218
3.238841	3.279004	3.373523	34.81331
3.244532	3.223741	3.338874	34.62913
3.311429	3.297065	3.378485	35.0224
3.249894	3.351385	3.401607	35.05718
3.288745	3.399504	3.377632	35.19494
3.192937	3.428281	3.451499	35.20989
3.363025	3.351991	3.385135	35.26989
3.309367	3.348958	3.390847	35.1584
3.442212	3.414398	3.457553	35.7379
3.29885	3.432459	3.437517	35.42007
3.331844	3.407793	3.348013	35.24255

3.252203	3.191931	3.410763	34.73356
3.300293	3.20878	3.304651	34.64352
3.207248	3.202445	3.430611	34.70164
3.19735	3.292887	3.365508	34.73541
3.144146	3.310274	3.484767	34.91788
3.320214	3.402132	3.37516	35.2641
3.370201	3.360078	3.445838	35.43601
3.432107	3.40968	3.360342	35.4929
3.310893	3.434481	3.437926	35.45172
3.397669	3.469526	3.360768	35.54939
3.314935	3.398358	3.371903	35.23718
3.389338	3.304681	3.275596	34.98442
3.169634	3.262695	3.340204	34.55344
3.135732	3.262021	3.437722	34.69108
3.281693	3.337973	3.414515	35.12562
3.233314	3.328875	3.410627	34.99143
3.29456	3.419789	3.398674	35.29804
3.256946	3.372344	3.452079	35.22881
3.288539	3.35435	3.416987	35.18181
3.284167	3.358394	3.383907	35.10875
3.466587	3.460697	3.391819	35.7487
3.280249	3.383868	3.428429	35.25326
3.333865	3.330088	3.389074	35.16683
3.191864	3.215856	3.413577	34.66008
3.327638	3.238635	3.329819	34.82364
3.200196	3.228391	3.424694	34.73002
3.219745	3.29538	3.408871	34.88466
3.203619	3.317149	3.444389	34.97467
3.307181	3.400515	3.383395	35.25007
3.259091	3.350778	3.388323	35.04692
3.435283	3.381577	3.351509	35.41907
3.298149	3.424439	3.441933	35.41065
3.339227	3.407861	3.385203	35.34017
3.298726	3.404491	3.373096	35.21776
3.304418	3.246318	3.331013	34.79228
3.234593	3.25117	3.347741	34.68677
3.174047	3.30549	3.360205	34.70041
3.20498	3.326382	3.447339	35.00429
3.225643	3.402402	3.39262	35.09606
3.32009	3.348554	3.403278	35.20815
3.255503	3.337232	3.462344	35.17132
3.360632	3.382184	3.453597	35.4804
3.318069	3.443781	3.390455	35.38394
3.43186	3.392899	3.36868	35.47389
3.350775	3.317553	3.362627	35.11857
3.310769	3.274017	3.356539	34.92256
3.240078	3.217204	3.395536	34.72901
3.321204	3.254405	3.350333	34.88892
3.19669	3.280756	3.406483	34.79704
3.282311	3.349363	3.340255	34.98949

3.170088	3.361426	3.478151	35.07201
3.355931	3.433807	3.357716	35.37333
3.233025	3.364931	3.441064	35.1362
3.379233	3.43967	3.380957	35.48794
3.278599	3.407726	3.437415	35.32147
3.453018	3.39364	3.351577	35.48438
3.260287	3.293156	3.417652	34.98766
3.335061	3.201838	3.299792	34.69374
3.191163	3.229267	3.368373	34.58902
3.230922	3.319979	3.38771	34.91662
3.158333	3.390338	3.377922	34.89034
3.257689	3.3559	3.437841	35.16334
3.313697	3.373827	3.387505	35.21495
3.325287	3.347745	3.440177	35.29844
3.37581	3.475187	3.448908	35.70672
3.335969	3.423631	3.452727	35.5152
3.322482	3.42417	3.390728	35.3513
3.330855	3.344578	3.361587	35.13183
3.375315	3.249755	3.350469	34.99738
3.196112	3.172926	3.442769	34.63933
3.248698	3.237826	3.363974	34.72393
3.18275	3.298278	3.429605	34.85544
3.252368	3.369985	3.405665	35.11214
3.288003	3.444118	3.476463	35.50701
3.268041	3.408265	3.422563	35.26708
3.22787	3.346532	3.430151	35.06083
3.400721	3.459417	3.404096	35.62871
3.286765	3.477815	3.446162	35.51173
3.477599	3.465347	3.303066	35.58886
3.305944	3.233243	3.371817	34.85625
3.296293	3.238568	3.354647	34.80924
3.250842	3.285137	3.313314	34.7213
3.324998	3.344847	3.393797	35.19005
3.220735	3.358461	3.398503	35.0021
3.255462	3.379892	3.432675	35.19964
3.200113	3.336086	3.372312	34.8508
3.365375	3.398425	3.455063	35.52949
3.334278	3.46191	3.417601	35.51839
3.372758	3.403884	3.476037	35.60344
3.264329	3.324832	3.411735	35.05283
3.340052	3.286148	3.353487	35.00645
3.298932	3.302255	3.368953	34.98557
3.299221	3.212891	3.343989	34.73619
3.104923	3.222865	3.436272	34.53491
3.15359	3.356574	3.359285	34.76538
3.307016	3.356372	3.378655	35.14281
3.30384	3.389327	3.352805	35.15141
3.344094	3.381712	3.44918	35.43354
3.163613	3.337097	3.423859	34.88592
3.279342	3.448566	3.386686	35.30147

3.434953	3.406041	3.321754	35.40678
3.423982	3.313981	3.373045	35.29363
3.196566	3.233648	3.417192	34.71717
3.302727	3.254472	3.390114	34.93565
3.224694	3.416285	3.356812	35.04604
3.407114	3.424439	3.351168	35.45045
3.236861	3.336962	3.433817	35.06758
3.155034	3.278599	3.419101	34.72883
3.219951	3.339523	3.424353	35.01551
3.342197	3.515421	3.352208	35.50973
3.299138	3.43374	3.412622	35.36906
3.328133	3.311218	3.373881	35.07981
3.234139	3.188696	3.433067	34.73575
3.286436	3.276578	3.299331	34.74984
3.362158	3.264919	3.385322	35.07799
3.353374	3.23513	3.316928	34.84407
3.174418	3.256899	3.410763	34.70553
3.220776	3.370255	3.344416	34.9097
3.261195	3.418239	3.415555	35.2586
3.384884	3.414937	3.315701	35.3035
3.246553	3.338108	3.475968	35.18346
3.122328	3.392023	3.411172	34.888
3.373995	3.426394	3.443519	35.58426
3.4093	3.34013	3.289765	35.13659
3.395854	3.202377	3.347144	34.93142
3.208485	3.158369	3.373608	34.48331
3.181059	3.374231	3.430628	35.02008
3.247254	3.449914	3.381435	35.22276
3.409382	3.417093	3.342898	35.42127
3.288869	3.265997	3.368203	34.88263
3.221353	3.346263	3.411735	35.00572
3.193473	3.517038	3.417226	35.33021
3.385502	3.511242	3.434584	35.77544
3.288085	3.379757	3.3511	35.0923
3.284497	3.206286	3.395383	34.80194
3.174872	3.232974	3.433357	34.70361
3.398453	3.287226	3.400737	35.23985
3.236655	3.260538	3.356062	34.72996
3.376924	3.273747	3.309136	34.96298
3.115275	3.285069	3.482721	34.79516
3.27167	3.408804	3.363769	35.14763
3.284538	3.447488	3.390642	35.31913
3.330896	3.396808	3.425564	35.38604
3.244243	3.356035	3.428838	35.11454
3.316213	3.443377	3.506405	35.63256
3.250183	3.435425	3.414327	35.26941
3.456647	3.238365	3.318582	35.0806
3.395566	3.236411	3.364434	35.04302
3.313285	3.362842	3.393746	35.20367
3.280703	3.413454	3.393643	35.24288

3.297819	3.391147	3.327228	35.08628
3.298107	3.287832	3.405886	35.033
3.331061	3.290326	3.292903	34.86344
3.208362	3.417363	3.439018	35.19245
3.261236	3.504975	3.407643	35.43106
3.295344	3.407861	3.507633	35.51194
3.362901	3.260066	3.401641	35.10469
3.25208	3.245509	3.378246	34.77934
3.268825	3.297807	3.426314	35.03545
3.29918	3.296931	3.382475	35.00404
3.233314	3.27806	3.425769	34.91341
3.28062	3.27341	3.358807	34.86026
3.277651	3.340534	3.37105	35.02733
3.276702	3.384745	3.390677	35.16486
3.179409	3.373759	3.372363	34.88802
3.290601	3.373422	3.365952	35.11642
3.238882	3.389866	3.378229	35.06613
3.299881	3.398291	3.344092	35.1433
3.338155	3.307107	3.389892	35.12775
3.337577	3.257842	3.375228	34.98668
3.362901	3.228863	3.359762	34.94487
3.40167	3.420868	3.418948	35.57896
3.272495	3.450993	3.409297	35.34125
3.263092	3.403952	3.379252	35.15211
3.167572	3.245172	3.414242	34.67252
3.21628	3.39236	3.385322	35.03767
3.268247	3.532202	3.445156	35.58797
3.247296	3.456317	3.419852	35.32087
3.189472	3.290528	3.427491	34.84857
3.351229	3.192133	3.348985	34.81545
3.339804	3.281834	3.404369	35.10775
3.284085	3.368166	3.367879	35.09489
3.320956	3.223943	3.439632	35.01704
3.209063	3.264447	3.341585	34.64651
3.38113	3.336154	3.388545	35.2823
3.324049	3.436705	3.369839	35.33646
3.317987	3.394517	3.397855	35.29221
3.203701	3.375107	3.421182	35.05085
3.222508	3.331301	3.395775	34.94062
3.314316	3.454093	3.458951	35.54807
3.406701	3.365605	3.334099	35.28356
3.252245	3.262762	3.38077	34.82295
3.300211	3.236815	3.40824	34.93118
3.263628	3.348756	3.389295	35.05455
3.45079	3.461978	3.38152	35.69443
3.30252	3.401795	3.34844	35.16624
3.260576	3.275836	3.330706	34.76028
3.132804	3.315599	3.368134	34.64967
3.365128	3.537661	3.338669	35.5789
3.213435	3.540626	3.458115	35.51487

3.286807	3.424507	3.305197	35.08698
3.134907	3.13377	3.564517	34.6861
3.287013	3.235198	3.390591	34.86018
3.46238	3.325775	3.434925	35.53871
3.389627	3.384677	3.428633	35.49466
3.205557	3.276173	3.432726	34.8638
3.232654	3.267951	3.384316	34.79921
3.289652	3.300165	3.291556	34.79146
3.394411	3.422148	3.35538	35.42687
3.176151	3.421339	3.38302	35.00825
3.057493	3.402132	3.487103	34.93438
3.277609	3.388856	3.393234	35.18142
3.318523	3.291539	3.331866	34.92388
3.403608	3.298548	3.359421	35.18553
3.258349	3.247127	3.414958	34.87687
3.222426	3.334671	3.399748	34.9565
3.26037	3.377331	3.363582	35.05368
3.445016	3.446814	3.313279	35.49942
3.309119	3.34788	3.418283	35.2155
3.268825	3.292415	3.372602	34.90619
3.166293	3.418509	3.411923	35.04371
3.369747	3.503425	3.37047	35.58368
3.278929	3.393573	3.345712	35.0907
3.27864	3.244835	3.432504	34.95461
3.17248	3.227717	3.490718	34.81232
3.304005	3.327797	3.439069	35.20586
3.309367	3.464876	3.329359	35.27743
3.444233	3.294235	3.449095	35.46104
3.156766	3.200288	3.404267	34.52892
3.322441	3.225156	3.433135	35.00874
3.320049	3.342286	3.322249	35.01716
3.311347	3.431448	3.319589	35.18729
3.129752	3.427674	3.32421	34.79203
3.220694	3.377668	3.443485	35.14238
3.16287	3.324832	3.399577	34.80437
3.357622	3.262627	3.39835	35.09154
3.353415	3.254068	3.405409	35.07907
3.32009	3.329819	3.338465	35.02545
3.281239	3.347206	3.427457	35.17312
3.379192	3.404491	3.307993	35.25135
3.397875	3.398156	3.401368	35.48255
3.331927	3.405906	3.243607	35.01028
3.177058	3.375646	3.40957	34.96837
3.218631	3.543322	3.421011	35.45099
3.306109	3.454564	3.446367	35.50364
3.222838	3.294504	3.325829	34.70791
3.203619	3.207701	3.412963	34.66661
3.206918	3.219023	3.455643	34.79192
3.244532	3.414196	3.466505	35.32474
3.328916	3.322001	3.390438	35.14131

3.385708	3.24989	3.398878	35.12627
3.232365	3.297537	3.419033	34.9392
3.300664	3.410489	3.362951	35.21293
3.32075	3.421609	3.363377	35.2821
3.302149	3.41433	3.364792	35.2286
3.178172	3.420733	3.420193	35.09263
3.293736	3.378949	3.368373	35.14066
3.275918	3.325573	3.433288	35.12693
3.319554	3.257707	3.326477	34.84036
3.327431	3.295178	3.444389	35.19739
3.379522	3.328403	3.403874	35.29536
3.312749	3.386429	3.500403	35.48733
3.431035	3.432527	3.290635	35.38808
3.353126	3.339456	3.462412	35.38982
3.345001	3.329953	3.343768	35.09181
3.234964	3.461371	3.382543	35.22337
3.164355	3.514006	3.441746	35.31353
3.201144	3.38589	3.427013	35.08159
3.188482	3.237018	3.39794	34.66476
3.239789	3.278936	3.375637	34.81986
3.278558	3.289719	3.463981	35.12142
3.25707	3.416757	3.452284	35.32666
3.179326	3.303872	3.42258	34.84482
3.369252	3.280149	3.38834	35.13341
3.258349	3.280689	3.394394	34.9053
3.318647	3.368031	3.408734	35.25952
3.288539	3.424911	3.417925	35.33817
3.243914	3.424372	3.417788	35.2391
3.235087	3.388653	3.397071	35.09638
3.283136	3.333997	3.415384	35.12198
3.211909	3.291337	3.43218	34.90966
3.293323	3.235333	3.337169	34.75745
3.260988	3.354552	3.418317	35.12491
3.343599	3.398965	3.441882	35.45423
3.378367	3.377601	3.401146	35.39446
3.350858	3.357922	3.37163	35.22671
3.283383	3.34168	3.412963	35.13403
3.308047	3.331032	3.297422	34.91201
3.238882	3.49244	3.413355	35.36726
3.304376	3.433942	3.356215	35.2576
3.139114	3.236141	3.377206	34.50954
3.170871	3.212823	3.35139	34.47154
3.294189	3.27368	3.424694	35.03461
3.342073	3.437042	3.430407	35.50906
3.287219	3.396606	3.41963	35.27711
3.289735	3.289787	3.414583	35.03798
3.213682	3.240118	3.417584	34.76961
3.38575	3.331571	3.350725	35.19968
3.289859	3.352261	3.397395	35.13728
3.186255	3.433942	3.387999	35.0688

3.238016	3.385351	3.407694	35.1188
3.217641	3.274151	3.382475	34.77592
3.350363	3.237624	3.366429	34.95119
3.232613	3.304546	3.42154	34.96055
3.174171	3.35125	3.416032	34.92284
3.271588	3.402199	3.417788	35.25113
3.470959	3.426461	3.348644	35.58898
3.339516	3.399167	3.418419	35.39443
3.323678	3.362572	3.327944	35.08191
3.265855	3.348217	3.47058	35.23599
3.298396	3.425248	3.378365	35.27395
3.248822	3.426866	3.328336	35.05967
3.2607	3.254405	3.3777	34.81645
3.076259	3.171578	3.415333	34.31428
3.205268	3.2848	3.435607	34.88834
3.204155	3.416217	3.396457	35.08768
3.322111	3.456182	3.469421	35.59258
3.238758	3.349632	3.486353	35.21432
3.305696	3.249418	3.37782	34.90421
3.308418	3.32928	3.444133	35.22982
3.386575	3.449106	3.385749	35.5351
3.258431	3.33029	3.44918	35.13376
3.216899	3.353811	3.295137	34.7575
3.123029	3.270984	3.471757	34.75733
3.295097	3.286148	3.28685	34.76241
3.340836	3.299963	3.40928	35.16039
3.210548	3.362842	3.404505	35.00253
3.185884	3.382453	3.433016	35.05383
3.294973	3.392158	3.462753	35.37865
3.443738	3.4208	3.331508	35.47959
3.477599	3.398628	3.352873	35.55188
3.31609	3.373288	3.323834	35.07976
3.285281	3.343971	3.422682	35.16444
3.281693	3.445129	3.472643	35.48707
3.219992	3.357517	3.327023	34.8421
3.314357	3.21781	3.3371	34.76498
3.071887	3.186135	3.383771	34.26753
3.171036	3.301109	3.400072	34.77143
3.200732	3.419857	3.372977	35.0368
3.276991	3.413859	3.437637	35.33185
3.215992	3.304748	3.40604	34.89075
3.381584	3.212891	3.390029	35.01698
3.269155	3.29565	3.476241	35.14063
3.381584	3.413319	3.362337	35.39473
3.308047	3.422148	3.445804	35.43575
3.300046	3.379825	3.351321	35.11908
3.192277	3.225628	3.400345	34.65341
3.258101	3.26721	3.348713	34.77538
3.255544	3.27988	3.382338	34.87103
3.286559	3.38097	3.347485	35.08371

3.203289	3.402199	3.427423	35.12284
3.367396	3.371401	3.411991	35.38062
3.324586	3.33366	3.40546	35.19019
3.424848	3.445331	3.377632	35.5928
3.306191	3.41642	3.420926	35.36476
3.28062	3.358326	3.398452	35.13266
3.245811	3.416015	3.445361	35.28527
3.222055	3.250766	3.384896	34.73972
3.279465	3.188561	3.342762	34.6371
3.235665	3.283789	3.414395	34.90621
3.193349	3.440951	3.443281	35.22053
3.160643	3.415948	3.468926	35.15041
3.322317	3.440749	3.376183	35.35539
3.231747	3.236815	3.414583	34.79533
3.362035	3.234861	3.398009	35.03973
3.241398	3.368637	3.488928	35.26729
3.314811	3.438457	3.389756	35.36364
3.301036	3.376051	3.403261	35.22658
3.403443	3.317081	3.31287	35.1239
3.184358	3.20386	3.41668	34.62422
3.263752	3.300839	3.291829	34.73688
3.157962	3.301715	3.458866	34.87274
3.305036	3.459956	3.388937	35.38749
3.308088	3.450184	3.424473	35.45051
3.339227	3.410152	3.442496	35.47047
3.308748	3.347139	3.345166	35.05317
3.4093	3.439738	3.415879	35.6302
3.263752	3.443647	3.336009	35.1458
3.298314	3.353002	3.440245	35.2511
3.165881	3.300502	3.410695	34.78206
3.167489	3.163087	3.380105	34.41818
3.348507	3.246183	3.303952	34.82922
3.254554	3.392562	3.364315	35.07587
3.273691	3.455912	3.474741	35.49774
3.134701	3.369783	3.406824	34.85692
3.329411	3.298952	3.398486	35.10959
3.358034	3.288169	3.411445	35.17694
3.326112	3.338175	3.389483	35.16846
3.164685	3.43812	3.455507	35.17839
3.253688	3.422418	3.401607	35.22082
3.275094	3.381105	3.353811	35.07276
3.414414	3.25427	3.334372	35.05756
3.243377	3.184585	3.378638	34.62794
3.1194	3.305692	3.393507	34.65418
3.174955	3.384879	3.460434	35.0952
3.303139	3.503223	3.359557	35.41371
3.26668	3.39209	3.372431	35.11911
3.404392	3.291809	3.347229	35.14585
3.222467	3.356709	3.426638	35.06359
3.335639	3.510501	3.367742	35.5186

3.374696	3.487183	3.399594	35.62267
3.375934	3.382386	3.369464	35.33032
3.231334	3.190718	3.367469	34.59059
3.18741	3.144149	3.38846	34.4386
3.160726	3.325371	3.408393	34.82014
3.322276	3.502683	3.394547	35.5309
3.195411	3.449982	3.421131	35.19635
3.217641	3.358933	3.485262	35.1861
3.252781	3.278465	3.386874	34.87181
3.408434	3.26539	3.385561	35.18074
3.286642	3.40011	3.415418	35.2743
3.288291	3.38616	3.421574	35.26086
3.24779	3.373288	3.440126	35.18472
3.281198	3.310207	3.316025	34.84844
3.445181	3.177307	3.3142	34.91242
3.27897	3.25791	3.408461	34.93134
3.179368	3.393169	3.448004	35.09579
3.129133	3.43994	3.406534	34.99753
3.384017	3.392899	3.365491	35.3623
3.355394	3.391619	3.373676	35.3148
3.322359	3.370322	3.399918	35.25337
3.214095	3.402469	3.422904	35.13718
3.371314	3.477815	3.381366	35.55493
3.330277	3.365739	3.330161	35.10812
3.394864	3.230683	3.429452	35.17115
3.206712	3.135523	3.402237	34.49207
3.251915	3.241263	3.361808	34.73375
3.241109	3.45726	3.451073	35.37768
3.294395	3.470335	3.33606	35.27128
3.223292	3.326516	3.418061	34.98061
3.206052	3.343904	3.424268	34.9945
3.173346	3.252855	3.499977	34.88944
3.385007	3.377264	3.343665	35.28254
3.346073	3.449645	3.503882	35.70605
3.38047	3.422283	3.398759	35.49155
3.255091	3.28116	3.386823	34.88265
3.333618	3.180744	3.335822	34.72325
3.251502	3.14671	3.309017	34.41063
3.310109	3.319575	3.304668	34.90731
3.15722	3.471817	3.431856	35.18404
3.222632	3.460899	3.378996	35.18761
3.317162	3.375579	3.440109	35.34139
3.323225	3.344376	3.362831	35.11742
3.322771	3.359674	3.365986	35.15678
3.215662	3.45726	3.441132	35.30029
3.283713	3.397212	3.430458	35.29445
3.24845	3.274354	3.359489	34.79347
3.376387	3.180542	3.323323	34.789
3.248986	3.210127	3.421966	34.79081
3.222962	3.423429	3.355363	35.05471

3.201598	3.460765	3.45994	35.31833
3.360426	3.361359	3.265757	35.02363
3.284415	3.307107	3.392024	35.01489
3.264494	3.330493	3.372159	34.97902
3.193225	3.302861	3.414634	34.85563
3.309326	3.387036	3.380139	35.21817
3.311223	3.395999	3.429469	35.34979
3.410289	3.364796	3.37857	35.38689
3.315223	3.236546	3.399305	34.94388
3.294725	3.187416	3.335924	34.65301
3.220858	3.218956	3.354101	34.6002
3.224323	3.388316	3.438336	35.16235
3.281775	3.455238	3.347007	35.23461
3.24082	3.399099	3.407762	35.15514
3.215621	3.351789	3.38198	34.9402
3.336051	3.401458	3.454705	35.47121
3.321121	3.440277	3.398759	35.40111
3.252162	3.459215	3.45491	35.41451
3.207908	3.305153	3.362627	34.77902
3.251214	3.17697	3.430355	34.74152
3.348919	3.176768	3.315905	34.70446
3.322317	3.36055	3.338055	35.09663
3.180852	3.47148	3.405443	35.17722
3.244161	3.446073	3.317252	35.06724
3.249646	3.296661	3.444645	35.03109
3.285446	3.346937	3.300815	34.90479
3.312419	3.290865	3.441047	35.14782
3.175573	3.369042	3.382134	34.89068
3.187328	3.395393	3.480606	35.18936
3.288786	3.381577	3.333895	35.06018
3.444769	3.237422	3.391324	35.21164
3.263009	3.217676	3.380565	34.74745
3.310893	3.213295	3.419357	34.92741
3.213517	3.346869	3.393592	34.95023
3.366406	3.431651	3.371493	35.42165
3.270887	3.452273	3.366105	35.24608
3.334402	3.331773	3.342557	35.06997
3.133876	3.373827	3.441746	34.94032
3.24115	3.438323	3.437381	35.30641
3.316337	3.472356	3.417993	35.50286
3.416847	3.352665	3.316451	35.23886
3.192029	3.105195	3.441865	34.4803
3.307676	3.204803	3.352873	34.75642
3.276331	3.360752	3.469693	35.28438
3.326607	3.524452	3.325199	35.43632
3.232159	3.436301	3.396031	35.1919
3.139774	3.334536	3.396304	34.76793
3.156849	3.297065	3.376882	34.68085
3.267711	3.347139	3.327876	34.92562
3.243047	3.345724	3.445412	35.12563

3.214754	3.396269	3.44132	35.16534
3.291426	3.325977	3.418829	35.13011
3.301613	3.252451	3.373795	34.89311
3.503128	3.239242	3.342642	35.23678
3.289941	3.238635	3.415401	34.92836
3.258679	3.332043	3.363513	34.95079
3.270681	3.370861	3.422904	35.19181
3.363478	3.434548	3.324892	35.31967
3.332876	3.401121	3.363718	35.26456
3.332834	3.413185	3.339573	35.23805
3.149301	3.363313	3.497044	35.07199
3.247997	3.457732	3.357204	35.1885
3.296746	3.342219	3.368765	35.06778
3.358158	3.205679	3.379798	34.92761
3.190091	3.146643	3.392024	34.45771
3.347476	3.247194	3.459872	35.17015
3.282517	3.471143	3.393285	35.37222
3.399896	3.426798	3.383839	35.51127
3.187369	3.32564	3.369379	34.79367
3.283425	3.302659	3.387368	34.99282
3.184234	3.386969	3.343375	34.86407
3.234634	3.351385	3.389415	34.99715
3.195576	3.369918	3.388886	34.95111
3.300788	3.350037	3.449709	35.27072
3.284745	3.254001	3.40396	34.92558
3.391854	3.236344	3.438728	35.19723
3.360344	3.252585	3.371374	35.01655
3.345909	3.289382	3.398094	35.12388
3.264123	3.404828	3.349514	35.09125
3.243006	3.428281	3.374205	35.15036
3.240779	3.389058	3.442701	35.2095
3.286889	3.403076	3.278136	34.98111
3.277898	3.382857	3.406193	35.19728
3.255627	3.428955	3.334048	35.09161
3.241068	3.401795	3.499431	35.36205
3.170913	3.281093	3.404505	34.73708
3.361086	3.1643	3.363036	34.80687
3.267422	3.236344	3.342847	34.71544
3.337041	3.376388	3.451551	35.41166
3.324338	3.485835	3.450323	35.62054
3.296169	3.367155	3.398759	35.18664
3.260782	3.274151	3.341005	34.77957
3.318647	3.326247	3.433561	35.22244
3.165675	3.396673	3.473121	35.12844
3.264082	3.375849	3.396986	35.1316
3.205763	3.315396	3.407575	34.89503
3.265319	3.277521	3.380752	34.88378
3.350239	3.233917	3.436835	35.09678
3.27398	3.287698	3.347417	34.85208
3.246017	3.280486	3.450204	34.99993

3.309408	3.404019	3.31432	35.11155
3.337619	3.389529	3.416902	35.36589
3.42258	3.383397	3.245363	35.16314
3.246842	3.345858	3.411531	35.06012
3.248739	3.451936	3.322521	35.1016
3.264618	3.477478	3.409877	35.38321
3.311511	3.430774	3.411258	35.38665
3.21661	3.306568	3.39823	34.87901
3.259916	3.163963	3.4109	34.68956
3.202423	3.200692	3.472626	34.77914
3.395153	3.399234	3.364144	35.39756
3.391317	3.432055	3.427627	35.59977
3.248945	3.366413	3.413645	35.1143
3.234881	3.318699	3.408393	34.96771
3.168974	3.341815	3.368458	34.78681
3.379481	3.394247	3.391035	35.41118
3.259916	3.392967	3.425291	35.22183
3.14031	3.358865	3.415555	34.8644
3.136516	3.226908	3.494265	34.73966
3.362653	3.234254	3.347894	34.93016
3.303345	3.244633	3.446384	35.03854
3.288333	3.342219	3.372278	35.05706
3.221065	3.358057	3.505297	35.23548
3.314935	3.432324	3.355175	35.27488
3.398411	3.460832	3.324499	35.45269
3.431282	3.400852	3.292511	35.32345
3.176604	3.317351	3.352259	34.71457
3.247378	3.42909	3.438984	35.30335
3.130989	3.418374	3.402885	34.94645
3.327926	3.353137	3.349599	35.11793
3.285528	3.246924	3.421455	34.95007
3.214095	3.186674	3.469216	34.76655
3.221766	3.360955	3.439291	35.09901
3.369211	3.468717	3.467528	35.71886
3.377748	3.41433	3.416765	35.50758
3.280868	3.351182	3.365201	35.04486
3.203825	3.286552	3.41315	34.8399
3.261566	3.382857	3.404693	35.15828
3.345909	3.420261	3.370743	35.35028
3.280579	3.329953	3.391819	35.05601
3.120307	3.258651	3.451568	34.68026
3.259091	3.267614	3.350077	34.78141
3.298314	3.234524	3.439563	34.99052
3.383729	3.313644	3.355295	35.16605
3.283301	3.309331	3.413082	35.06337
3.249316	3.393977	3.370249	35.08049
3.312625	3.411028	3.435454	35.39881
3.333412	3.423159	3.325522	35.2304
3.400144	3.380701	3.386635	35.41712
3.212197	3.366346	3.380684	34.96171

3.187328	3.38771	3.415231	35.02959
3.223869	3.358528	3.387471	34.98498
3.331102	3.300772	3.360308	35.03378
3.152312	3.20204	3.4264	34.57141
3.288828	3.301648	3.384794	34.99679
3.241604	3.423092	3.4508	35.30344
3.450749	3.44277	3.368373	35.62359
3.417053	3.348621	3.416902	35.45014
3.35292	3.373355	3.378792	35.28064
3.208568	3.330493	3.454586	35.03698
3.246099	3.46191	3.355857	35.19054
3.185018	3.387036	3.428974	35.05312
3.299757	3.296998	3.316536	34.86125
3.170789	3.169017	3.513021	34.72903
3.217971	3.188427	3.406006	34.64063
3.279754	3.317553	3.467528	35.19266
3.354487	3.385418	3.380224	35.31358
3.333453	3.390877	3.409945	35.34451
3.326813	3.366818	3.401504	35.25892
3.335474	3.446612	3.434022	35.52346
3.370283	3.404963	3.375057	35.37956
3.303387	3.401997	3.353265	35.17913
3.231912	3.347745	3.409416	35.02698
3.222921	3.365942	3.391273	35.00743
3.228489	3.254338	3.413969	34.82518
3.33003	3.229874	3.307976	34.76195
3.187864	3.205545	3.433135	34.67155
3.267835	3.416285	3.405801	35.24751
3.255091	3.454901	3.492543	35.49378
3.37878	3.351452	3.317167	35.15453
3.360591	3.341141	3.41298	35.30173
3.395689	3.405906	3.380804	35.44975
3.199123	3.379892	3.452795	35.12044
3.283837	3.413859	3.307772	35.06283
3.17215	3.313577	3.392501	34.78458
3.2826	3.257707	3.331047	34.76955
3.220157	3.259257	3.439938	34.87451
3.22787	3.248407	3.431157	34.84844
3.274227	3.334536	3.402152	35.07474
3.28227	3.299222	3.405034	35.02141
3.321204	3.343769	3.383992	35.15795
3.321699	3.448364	3.374972	35.36804
3.321987	3.40166	3.366429	35.24786
3.379398	3.424102	3.344484	35.37449
3.340299	3.398695	3.297643	35.13099
3.255379	3.351722	3.361382	34.98195
3.286889	3.355226	3.391393	35.12415
3.211166	3.286215	3.413901	34.85686
3.273155	3.24652	3.356079	34.77917
3.228653	3.258044	3.388528	34.77801

3.315347	3.386766	3.416885	35.3111
3.232737	3.442703	3.439154	35.30147
3.28891	3.306366	3.3725	34.9804
3.332051	3.320384	3.376524	35.1142
3.422333	3.381307	3.407847	35.51336
3.206877	3.435896	3.461935	35.27985
3.228695	3.418239	3.340102	35.02252
3.195494	3.26074	3.379712	34.69211
3.288085	3.308927	3.352447	34.94035
3.170954	3.254203	3.399032	34.6664
3.273238	3.275634	3.445719	35.03904
3.154333	3.31169	3.445429	34.85723
3.389008	3.354013	3.41535	35.3972
3.334154	3.34296	3.424268	35.27258
3.447615	3.415409	3.362627	35.54433
3.35692	3.407928	3.33473	35.26864
3.315058	3.401458	3.368356	35.23648
3.277156	3.373557	3.340443	35.03153
3.33799	3.317553	3.309068	34.97348
3.214919	3.351924	3.406006	34.9915
3.141052	3.317283	3.403346	34.74839
3.267134	3.270377	3.398384	34.91068
3.220446	3.361359	3.368151	34.94143
3.313532	3.410624	3.426792	35.38097
3.250348	3.379757	3.484699	35.30193
3.245275	3.369311	3.40176	35.08662
3.317038	3.372681	3.366719	35.17429
3.414084	3.409006	3.383839	35.50339
3.265814	3.435425	3.442718	35.36568
3.198999	3.333458	3.371698	34.84128
3.168191	3.18135	3.459889	34.63413
3.301283	3.266334	3.355499	34.88274
3.216569	3.250025	3.437381	34.84088
3.281363	3.33083	3.41315	35.10629
3.230386	3.434616	3.460775	35.32593
3.293447	3.371333	3.370794	35.12867
3.40402	3.383666	3.371971	35.40002
3.479248	3.445399	3.418743	35.80181
3.31015	3.408871	3.427559	35.37142
3.341454	3.389799	3.325369	35.1747
3.24944	3.355765	3.351628	34.95647
3.341702	3.278195	3.347758	34.98014
3.269402	3.261818	3.366225	34.8266
3.084343	3.311353	3.45682	34.72835
3.165592	3.295448	3.435386	34.82437
3.324379	3.405434	3.391938	35.31712
3.327184	3.417363	3.456785	35.49115
3.402742	3.366279	3.38343	35.38426
3.253358	3.322944	3.423876	35.05126
3.282229	3.405569	3.379866	35.19884

3.409836	3.435357	3.412895	35.61527
3.291591	3.352126	3.425376	35.20197
3.197597	3.236276	3.384708	34.65414
3.22787	3.204601	3.414361	34.71592
3.286972	3.258112	3.415691	34.96509
3.275424	3.294302	3.370539	34.92024
3.303387	3.323281	3.437585	35.19138
3.188029	3.373759	3.45346	35.08422
3.371933	3.384812	3.371766	35.33191
3.338938	3.433268	3.373795	35.37015
3.454007	3.494327	3.421011	35.85857
3.308088	3.398088	3.390029	35.26126
3.300994	3.342758	3.395639	35.13701
3.300335	3.224078	3.306817	34.6818
3.361251	3.219563	3.33485	34.86644
3.217889	3.281295	3.406807	34.84529
3.208114	3.316138	3.38123	34.84418
3.135113	3.315599	3.475968	34.89053
3.314852	3.405906	3.356352	35.2195
3.381213	3.382521	3.430611	35.47587
3.393957	3.34013	3.371476	35.28172
3.286229	3.418711	3.472626	35.43918
3.365293	3.436031	3.372005	35.42992
3.288539	3.38744	3.449692	35.3257
3.380553	3.316475	3.264461	34.96665
3.145218	3.19557	3.419153	34.5259
3.142826	3.253394	3.404164	34.61435
3.258596	3.2261	3.375569	34.74529
3.209929	3.224078	3.395911	34.67893
3.284992	3.363313	3.422085	35.20481
3.289405	3.411837	3.457843	35.39876
3.2534	3.371468	3.404574	35.11526
3.332958	3.424035	3.356471	35.299
3.47962	3.457867	3.378843	35.74263
3.285693	3.437177	3.408529	35.33822
3.317822	3.34633	3.336009	35.05122
3.2793	3.201434	3.396389	34.78216
3.366077	3.281093	3.32334	34.98638
3.226138	3.298144	3.371323	34.82258
3.245357	3.334806	3.378314	34.96007
3.17281	3.323012	3.422802	34.87291
3.284332	3.369109	3.374154	35.11122
3.311182	3.407591	3.434567	35.3862
3.369458	3.412106	3.39141	35.42914
3.286353	3.441018	3.4412	35.41951
3.352631	3.417767	3.372107	35.36251
3.31147	3.36365	3.454228	35.33374
3.335598	3.274691	3.356352	34.97792
3.222467	3.227582	3.358654	34.63254
3.139939	3.238972	3.390625	34.54688

3.152271	3.288169	3.414787	34.73428
3.21294	3.34141	3.450664	35.06184
3.292375	3.422957	3.424149	35.35589
3.28891	3.368637	3.410763	35.20026
3.257318	3.37787	3.407489	35.1442
3.334938	3.426461	3.383617	35.368
3.474011	3.46501	3.372448	35.73201
3.307923	3.380027	3.44398	35.33938
3.31279	3.316677	3.326733	34.95509
3.277321	3.204264	3.371408	34.72939
3.294354	3.27159	3.36822	34.9069
3.232943	3.356035	3.37088	34.96309
3.2574	3.372142	3.375586	35.06209
3.164809	3.347408	3.424132	34.91168
3.261236	3.363583	3.453887	35.22299
3.223209	3.416689	3.450459	35.24847
3.394782	3.467774	3.385527	35.59339
3.305944	3.426394	3.462822	35.47765
3.367891	3.373288	3.420329	35.40406
3.260246	3.328673	3.439222	35.11241
3.276826	3.26721	3.411803	34.9543
3.214961	3.255753	3.386942	34.73959
3.186379	3.239781	3.372977	34.61162
3.051884	3.268221	3.441132	34.52874
3.262391	3.357922	3.406534	35.10958
3.342527	3.408265	3.417311	35.41849
3.296788	3.375444	3.426809	35.26746
3.266061	3.39236	3.420807	35.22413
3.330566	3.424305	3.409536	35.4104
3.42225	3.455104	3.38285	35.6199
3.420642	3.422552	3.322266	35.41271
3.333082	3.27651	3.337442	34.93504
3.25076	3.212621	3.383072	34.71509
3.266886	3.310274	3.393422	34.98654
3.251915	3.370457	3.370658	35.03563
3.317739	3.408871	3.350196	35.21884
3.143362	3.306029	3.440314	34.80967
3.248038	3.358191	3.405511	35.07655
3.226962	3.363785	3.426519	35.08863
3.345579	3.473367	3.369157	35.46222
3.264824	3.402132	3.437517	35.27934
3.402618	3.315599	3.402391	35.31462
3.177883	3.248946	3.430219	34.73826
3.317409	3.24342	3.32646	34.80439
3.206588	3.201905	3.384214	34.59756
3.263958	3.250901	3.382662	34.82677
3.133876	3.294033	3.39557	34.66485
3.22787	3.400649	3.441882	35.20483
3.339763	3.452947	3.437381	35.55404
3.356838	3.40685	3.362064	35.32587

3.290642	3.35462	3.419835	35.19323
3.322029	3.394045	3.399594	35.30382
3.359849	3.452475	3.398657	35.51225
3.391524	3.354754	3.314729	35.18428
3.395772	3.202512	3.33057	34.89529
3.201103	3.196716	3.417447	34.64689
3.242264	3.359404	3.375705	35.00139
3.265938	3.385688	3.386073	35.13331
3.310398	3.411432	3.394803	35.30593
3.259792	3.334132	3.40546	35.04953
3.211331	3.352261	3.469352	35.12292
3.216569	3.44331	3.414856	35.2143
3.445346	3.475456	3.460758	35.88529
3.258885	3.366481	3.381793	35.06653
3.359106	3.240185	3.376063	34.99698
3.268907	3.236344	3.46983	34.99638
3.352095	3.30549	3.46867	35.32697
3.2607	3.240118	3.350759	34.72629
3.317162	3.272601	3.352242	34.92405
3.131608	3.313779	3.398895	34.71034
3.204939	3.462449	3.421131	35.24445
3.284992	3.432998	3.409536	35.32975
3.330071	3.38589	3.3644	35.22661
3.294148	3.363179	3.376319	35.12445
3.238882	3.409545	3.510566	35.39857
3.281651	3.395325	3.325113	35.05544
3.421714	3.249081	3.341142	35.07698
3.27629	3.199344	3.367572	34.70799
3.232448	3.321125	3.386073	34.91889
3.17116	3.385284	3.391972	34.93807
3.274392	3.410422	3.328813	35.08067
3.29522	3.3652	3.481306	35.3608
3.264247	3.365739	3.363292	35.03617
3.274145	3.427674	3.482584	35.45413
3.320668	3.529304	3.395639	35.58798
3.30516	3.418239	3.462191	35.45673
3.296788	3.288574	3.382253	34.98005
3.31147	3.262897	3.375876	34.94206
3.240325	3.295044	3.437449	34.99143
3.32673	3.243353	3.375501	34.93187
3.199412	3.217945	3.383856	34.61616
3.262184	3.253125	3.36099	34.78036
3.251007	3.316542	3.453597	35.09712
3.190875	3.432594	3.440331	35.19039
3.258967	3.376725	3.381111	35.08762
3.326153	3.34013	3.357409	35.10268
3.26569	3.395528	3.435948	35.26336
3.429096	3.44122	3.33901	35.50864
3.379481	3.27806	3.447237	35.28
3.328091	3.225628	3.319981	34.77467

3.317616	3.180339	3.363428	34.74774
3.36088	3.386699	3.345933	35.25537
3.24214	3.442231	3.408529	35.25403
3.266886	3.422283	3.383771	35.21038
3.195081	3.289585	3.441456	34.88931
3.244408	3.422081	3.365219	35.12021
3.312501	3.51933	3.421489	35.60484
3.249481	3.426866	3.515664	35.47077
3.215909	3.295178	3.461219	34.99031
3.31411	3.226774	3.399185	34.91981
3.347806	3.32901	3.397736	35.2139
3.267917	3.330425	3.373523	34.98935
3.299798	3.238972	3.406807	34.93186
3.167283	3.261144	3.345234	34.55591
3.321451	3.347948	3.361876	35.11927
3.324916	3.38434	3.384043	35.2549
3.275753	3.429494	3.466846	35.42721
3.21991	3.378544	3.443997	35.14371
3.269443	3.353541	3.391188	35.08187
3.400721	3.409545	3.39482	35.49936
3.389915	3.364796	3.316502	35.2066
3.248285	3.240387	3.395127	34.79676
3.339681	3.208173	3.368748	34.86849
3.212156	3.338108	3.42877	35.00502
3.39672	3.462719	3.35422	35.51811
3.233149	3.441827	3.38609	35.18441
3.227292	3.308253	3.37117	34.84687
3.108346	3.287091	3.432657	34.67494
3.2866	3.501336	3.388392	35.43647
3.270268	3.497494	3.442428	35.51052
3.28924	3.342623	3.398264	35.11676
3.136186	3.188494	3.483488	34.63137
3.376511	3.258718	3.387027	35.09954
3.512697	3.302592	3.347195	35.4062
3.322565	3.315666	3.426314	35.19202
3.209846	3.267075	3.391478	34.76308
3.168892	3.31917	3.44427	34.90289
3.305985	3.397347	3.335344	35.13545
3.402412	3.407187	3.392842	35.49358
3.298479	3.420126	3.457075	35.43506
3.152106	3.342152	3.448891	34.92655
3.273774	3.379623	3.350009	35.05832
3.363107	3.443781	3.317457	35.3228
3.437221	3.240118	3.376882	35.16944
3.190256	3.206758	3.38273	34.56921
3.222261	3.160391	3.425376	34.63106
3.241191	3.394449	3.373062	35.0699
3.42757	3.413656	3.326187	35.41698
3.26103	3.37996	3.412895	35.16871
3.207784	3.267749	3.407847	34.79584

3.084302	3.44122	3.465362	35.03094
3.373294	3.585982	3.350298	35.72786
3.300541	3.431179	3.454347	35.45777
3.256575	3.285069	3.468142	35.07227
3.206176	3.228459	3.440791	34.77845
3.358488	3.308859	3.404437	35.20785
3.429138	3.339119	3.342557	35.2932
3.379522	3.306568	3.407762	35.25611
3.157632	3.259055	3.396576	34.64251
3.262556	3.344847	3.399373	35.06569
3.217105	3.41925	3.376114	35.07814
3.34364	3.395258	3.359421	35.26588
3.196731	3.387305	3.436136	35.09499
3.217353	3.324225	3.424336	34.97633
3.280579	3.419317	3.493838	35.47454
3.417383	3.3559	3.354715	35.33078
3.330855	3.250496	3.33248	34.86243
3.245646	3.224482	3.451363	34.87919
3.21096	3.279408	3.445548	34.91073
3.287425	3.433538	3.38077	35.27334
3.419569	3.378881	3.309869	35.28775
3.270804	3.316273	3.335805	34.88223
3.221353	3.341141	3.429298	35.03292
3.147486	3.518791	3.448873	35.30269
3.346197	3.524789	3.437841	35.72623
3.305737	3.34788	3.350196	35.05921
3.231458	3.213025	3.459855	34.84168
3.15058	3.275836	3.459463	34.80131
3.329906	3.381038	3.427269	35.35312
3.31345	3.350104	3.402374	35.19505
3.388389	3.281834	3.376404	35.15284
3.179203	3.303131	3.423927	34.84588
3.230881	3.312094	3.348559	34.81367
3.249935	3.322742	3.471893	35.14834
3.269072	3.485363	3.382679	35.35072
3.138413	3.388923	3.468142	35.04098
3.317863	3.385755	3.388664	35.25268
3.207496	3.322136	3.46173	35.03198
3.38608	3.32254	3.357119	35.19463
3.400556	3.263705	3.418624	35.23213
3.314935	3.367087	3.351645	35.1245
3.327514	3.437783	3.436886	35.49302
3.319471	3.413724	3.325659	35.17958
3.337825	3.279813	3.330894	34.93832
3.328586	3.330088	3.372551	35.11916
3.192813	3.464336	3.397003	35.16929
3.181182	3.480444	3.406193	35.19918
3.3151	3.359809	3.406142	35.22812
3.242635	3.194896	3.391938	34.67795
3.226303	3.251575	3.372551	34.72378

3.270763	3.293022	3.437654	35.05402
3.3118	3.456721	3.432385	35.49022
3.262721	3.268827	3.381315	34.86032
3.400309	3.252653	3.311063	34.9722
3.257524	3.304411	3.396986	34.96104
3.321657	3.41743	3.351987	35.25004
3.215579	3.418644	3.422853	35.17569
3.295014	3.461708	3.319248	35.21701
3.243584	3.350104	3.424353	35.09033
3.328628	3.294707	3.366531	35.02871
3.307222	3.260942	3.437722	35.06374
3.26503	3.236074	3.325369	34.6714
3.229685	3.288978	3.433749	34.9468
3.424436	3.437783	3.409689	35.64549
3.378285	3.42262	3.364877	35.41341
3.376635	3.332245	3.399782	35.2885
3.296829	3.329819	3.324772	34.94464
3.277981	3.419789	3.347707	35.15032
3.229231	3.489744	3.388477	35.28585
3.261937	3.470537	3.408734	35.35967
3.129999	3.191999	3.430543	34.50972
3.252616	3.142464	3.379184	34.55722
3.276661	3.253125	3.437705	34.97978
3.36554	3.466493	3.448498	35.66435
3.341495	3.364661	3.444952	35.38132
3.251255	3.258988	3.344484	34.73318
3.282229	3.27159	3.353265	34.84768
3.359395	3.353609	3.316042	35.11439
3.190751	3.426866	3.395229	35.07896
3.153467	3.426057	3.405989	35.01919
3.228324	3.378814	3.362576	34.98464
3.248986	3.284934	3.386703	34.87729
3.339928	3.325708	3.430424	35.26094
3.207826	3.260538	3.414907	34.7956
3.210218	3.276578	3.42877	34.86623
3.268041	3.324292	3.3681	34.96435
3.414744	3.391888	3.387863	35.4762
3.359395	3.399706	3.384777	35.36551
3.307305	3.354754	3.365764	35.11172
3.259916	3.372614	3.432879	35.19391
3.297118	3.490418	3.282126	35.20321
3.215621	3.462719	3.406978	35.23745
3.2793	3.330021	3.360086	34.98397
3.154457	3.189775	3.470785	34.64634
3.214672	3.24679	3.362763	34.66648
3.333535	3.37969	3.42906	35.36203
3.428973	3.437379	3.419357	35.67567
3.24544	3.271995	3.474178	35.03253
3.282806	3.256225	3.375006	34.86288
3.302892	3.350441	3.454842	35.28743

3.448893	3.467774	3.337373	35.60642
3.256864	3.375175	3.395894	35.11196
3.079146	3.353339	3.387982	34.65826
3.239047	3.368705	3.481152	35.24529
3.232819	3.406513	3.404898	35.14759
3.339804	3.392225	3.369362	35.2726
3.268206	3.222124	3.31773	34.63113
3.223457	3.264379	3.440416	34.89397
3.216982	3.369177	3.39291	35.0051
3.490838	3.390608	3.381588	35.62608
3.332504	3.389664	3.367111	35.24611
3.321822	3.357854	3.3511	35.11818
3.25674	3.330358	3.464731	35.16422
3.295757	3.463663	3.318378	35.221
3.237933	3.50484	3.406279	35.37683
3.242924	3.33676	3.348235	34.89324
3.130824	3.222056	3.490633	34.70866
3.242841	3.230278	3.380002	34.72967
3.28825	3.368974	3.425922	35.2327
3.400102	3.46218	3.442547	35.71749
3.282806	3.299626	3.427423	35.07242
3.336876	3.258449	3.411855	35.06657
3.30252	3.258112	3.408103	34.9825
3.344465	3.33366	3.390438	35.20081
3.235335	3.406311	3.432299	35.21258
3.21925	3.489744	3.382867	35.25176
3.153714	3.311488	3.399577	34.75517
3.256204	3.281228	3.410934	34.93796
3.325081	3.280958	3.398299	35.06036
3.2355	3.301513	3.410422	34.93592
3.274021	3.411028	3.451346	35.34915
3.356756	3.418846	3.391461	35.41621
3.386987	3.391416	3.384504	35.40712
3.434664	3.373355	3.269252	35.21985
3.212816	3.306029	3.418965	34.91487
3.225725	3.385081	3.399219	35.0728
3.340011	3.46373	3.424541	35.55009
3.216652	3.315531	3.367879	34.83232
3.244862	3.240859	3.355141	34.70286
3.102944	3.233513	3.484443	34.65921
3.241356	3.360078	3.421045	35.10003
3.293901	3.405434	3.455063	35.38852
3.336258	3.380768	3.356744	35.21219
3.261566	3.227043	3.404898	34.81799
3.384554	3.256562	3.353095	35.03821
3.279383	3.325505	3.427815	35.12239
3.378615	3.402267	3.336077	35.30664
3.227004	3.428685	3.391955	35.15506
3.270309	3.316677	3.341108	34.89363
3.253028	3.186	3.391717	34.68074

3.266226	3.256764	3.396764	34.87539
3.237108	3.330223	3.424285	35.03254
3.301943	3.450993	3.375023	35.3307
3.30252	3.455441	3.4508	35.5074
3.363066	3.391821	3.353692	35.28832
3.316378	3.345724	3.363309	35.10644
3.354198	3.39391	3.387454	35.34733
3.259586	3.3652	3.344671	34.98408
3.270763	3.368772	3.413764	35.16743
3.217435	3.368031	3.3858	34.98804
3.195989	3.155741	3.365764	34.43308
3.274227	3.21626	3.322112	34.64106
3.239995	3.375377	3.469557	35.2366
3.304995	3.443849	3.477077	35.54492
3.163035	3.375309	3.506661	35.14929
3.391153	3.338243	3.434567	35.40943
3.369582	3.240994	3.323459	34.90662
3.384182	3.302322	3.361808	35.15652
3.244079	3.381779	3.46023	35.23913
3.253688	3.472693	3.400669	35.32871
3.222756	3.384138	3.354237	34.96587
3.31345	3.221517	3.316724	34.72654
3.161262	3.158436	3.387317	34.41016
3.183904	3.254607	3.414498	34.72943
3.15491	3.387036	3.396713	34.91673
3.273073	3.460091	3.432044	35.41215
3.297736	3.434548	3.321311	35.16808
3.486507	3.308455	3.431737	35.54663
3.225189	3.339052	3.3954	34.96261
3.340299	3.41642	3.356744	35.299
3.330442	3.388249	3.338942	35.17691
3.358323	3.368031	3.287583	35.08135
3.181966	3.253057	3.4146	34.72202
3.151611	3.203051	3.340852	34.38501
3.165634	3.296998	3.43779	34.83311
3.321039	3.482061	3.369839	35.42906
3.280579	3.416622	3.479157	35.43654
3.281445	3.328673	3.480282	35.24856
3.272454	3.201973	3.406739	34.791
3.39705	3.253394	3.351441	35.055
3.317038	3.427405	3.404846	35.37734
3.271877	3.449038	3.380207	35.27201
3.210506	3.371401	3.464851	35.15312
3.219003	3.264379	3.317218	34.61482
3.359354	3.21006	3.328916	34.82854
3.246141	3.240252	3.390233	34.78107
3.195659	3.450656	3.463231	35.29043
3.159859	3.475726	3.429213	35.19258
3.422869	3.451734	3.389346	35.62809
3.37746	3.325303	3.365406	35.19995

3.338278	3.325708	3.3777	35.14203
3.186132	3.454093	3.440842	35.22815
3.293571	3.480039	3.329802	35.27702
3.261896	3.366279	3.330007	34.95942
3.348094	3.208308	3.366105	34.88141
3.114368	3.126896	3.403414	34.27384
3.148105	3.324562	3.39794	34.76791
3.237974	3.468043	3.46069	35.41544
3.312914	3.455845	3.368083	35.35012
3.230097	3.326988	3.463009	35.09482
3.310315	3.284732	3.423262	35.09091
3.243501	3.225426	3.394053	34.75123
3.349332	3.477613	3.3991	35.5452
3.302892	3.454025	3.425496	35.44978
3.363396	3.375916	3.45121	35.46751
3.265484	3.242476	3.313996	34.66152
3.293117	3.171848	3.347451	34.64066
3.158375	3.282306	3.34433	34.58073
3.264906	3.406378	3.425172	35.26181
3.188441	3.453823	3.454552	35.26259
3.264329	3.409748	3.375995	35.16037
3.296458	3.339321	3.440245	35.21713
3.419817	3.393304	3.312052	35.3246
3.301201	3.414735	3.404011	35.31318
3.207372	3.485768	3.350145	35.14553
3.264741	3.350711	3.446316	35.18595
3.175656	3.18654	3.364127	34.45238
3.361828	3.201771	3.314831	34.78502
3.292993	3.240859	3.38846	34.88098
3.243707	3.498438	3.405324	35.37336
3.17912	3.480646	3.480675	35.35799
3.272	3.360011	3.401845	35.12491
3.281816	3.289113	3.369567	34.92075
3.329741	3.267816	3.421949	35.09353
3.212981	3.351722	3.3718	34.91201
3.339969	3.446882	3.428105	35.52095
3.27963	3.38124	3.402135	35.18866
3.401505	3.25582	3.334918	35.03391
3.269856	3.175352	3.398929	34.71003
3.235582	3.159784	3.360222	34.51638
3.199618	3.340939	3.373267	34.86242
3.24911	3.47613	3.339522	35.1925
3.250925	3.45389	3.406006	35.29322
3.311841	3.431583	3.361808	35.281
3.215373	3.322203	3.490974	35.11331
3.375315	3.39701	3.365065	35.35132
3.410537	3.421137	3.395826	35.54838
3.401711	3.40968	3.440501	35.60172
3.232778	3.271119	3.398077	34.8365
3.241274	3.114496	3.336487	34.37789

3.278805	3.201636	3.370965	34.72592
3.36884	3.436975	3.339078	35.36773
3.211579	3.462652	3.435454	35.29073
3.130123	3.354889	3.417055	34.83671
3.262679	3.315464	3.467596	35.1509
3.309697	3.273208	3.41535	35.04706
3.340671	3.300502	3.428429	35.20308
3.225478	3.441692	3.477554	35.36736
3.221642	3.437042	3.43334	35.25211
3.29918	3.344174	3.366514	35.07245
3.477228	3.209588	3.336504	35.10187
3.255214	3.17825	3.380411	34.64385
3.203536	3.315127	3.374563	34.81737
3.217683	3.414533	3.493361	35.32549
3.377542	3.497427	3.318276	35.47347
3.324214	3.389934	3.368595	35.23182
3.304871	3.341882	3.376046	35.10073
3.123359	3.379286	3.41078	34.86155
3.303387	3.46063	3.373591	35.3518
3.300211	3.398021	3.419374	35.30806
3.411939	3.310072	3.375995	35.2652
3.198628	3.167063	3.364042	34.45984
3.274145	3.189909	3.411053	34.77775
3.25208	3.394786	3.3607	35.06742
3.299427	3.485431	3.44843	35.56104
3.157179	3.46191	3.390387	35.07159
3.20498	3.316407	3.455455	35.00023
3.125792	3.32746	3.409484	34.7507
3.308583	3.308994	3.44473	35.18713
3.292787	3.386969	3.455012	35.34559
3.286271	3.412578	3.457212	35.39215
3.210383	3.359202	3.440314	35.07252
3.331515	3.237287	3.382679	34.94477
3.380759	3.212891	3.332224	34.88877
3.307016	3.31971	3.357443	35.01625
3.111522	3.382992	3.454654	34.93971
3.261112	3.469189	3.427047	35.39497
3.361045	3.450386	3.373727	35.45578
3.329205	3.403278	3.266592	35.04885
3.315017	3.373692	3.473786	35.40622
3.189101	3.445871	3.430697	35.19448
3.275588	3.40321	3.458252	35.35058
3.296994	3.302052	3.326784	34.88868
3.276826	3.178924	3.421199	34.78178
3.191864	3.175689	3.369703	34.47629
3.28029	3.361426	3.395417	35.13208
3.295757	3.447488	3.444372	35.46116
3.367644	3.439738	3.339624	35.37235
3.141259	3.364459	3.428173	34.9063
3.271464	3.347408	3.354647	34.99296

3.216198	3.322944	3.465823	35.06173
3.242717	3.395056	3.374887	35.07856
3.248986	3.380229	3.477009	35.28317
3.396349	3.387575	3.380241	35.40988
3.293117	3.268962	3.45878	35.09649
3.345579	3.228189	3.331218	34.84309
3.321327	3.152034	3.420005	34.81768
3.284992	3.377062	3.336009	35.04664
3.154168	3.434414	3.433152	35.0984
3.205062	3.444725	3.379474	35.11486
3.352219	3.389866	3.391069	35.34206
3.277321	3.407321	3.374068	35.17926
3.267175	3.412982	3.395297	35.21588
3.2501	3.459686	3.373727	35.2335
3.25307	3.405232	3.418692	35.21925
3.175738	3.193953	3.398981	34.54499
3.365994	3.213632	3.338055	34.87085
3.217971	3.249351	3.426996	34.81976
3.303304	3.401458	3.356897	35.18571
3.29621	3.410152	3.491127	35.48275
3.320874	3.362909	3.319793	35.05869
3.256245	3.348958	3.412434	35.08944
3.278723	3.365066	3.443724	35.24225
3.198051	3.315194	3.47353	35.02195
3.257565	3.328606	3.354971	34.92216
3.220735	3.354552	3.544857	35.31361
3.385873	3.399975	3.394956	35.44626
3.346651	3.205679	3.391478	34.92799
3.356467	3.1767	3.31478	34.71836
3.271175	3.209723	3.375995	34.73792
3.256287	3.385149	3.329615	34.98756
3.216198	3.432392	3.353351	35.05512
3.310975	3.399167	3.331286	35.14147
3.203041	3.387777	3.345336	34.91125
3.222384	3.408198	3.413509	35.14729
3.271382	3.505649	3.399458	35.43682
3.323183	3.432594	3.444184	35.48816
3.314069	3.309735	3.381366	35.06218
3.214548	3.168209	3.430321	34.6421
3.363519	3.214373	3.396116	34.99403
3.327596	3.374029	3.31507	35.08738
3.266515	3.450116	3.378996	35.25999
3.211455	3.428281	3.422427	35.18681
3.296293	3.347948	3.429247	35.21158
3.210259	3.359674	3.344535	34.86383
3.323472	3.322675	3.387727	35.12495
3.143527	3.37167	3.488314	35.05855
3.136021	3.41588	3.454347	35.06454
3.277114	3.357585	3.344961	35.00639
3.433468	3.237422	3.384026	35.17097

3.272908	3.221652	3.350827	34.71276
3.307181	3.256427	3.403278	34.97846
3.199659	3.332245	3.413219	34.93086
3.362282	3.445129	3.396321	35.4964
3.324503	3.456317	3.344603	35.32515
3.399896	3.397684	3.36706	35.41091
3.232819	3.382925	3.394939	35.07424
3.246264	3.435222	3.391717	35.21096
3.212074	3.44122	3.4161	35.20263
3.335763	3.351654	3.366975	35.16982
3.276496	3.19402	3.434721	34.84364
3.200154	3.223808	3.41107	34.69012
3.282765	3.356304	3.467255	35.28339
3.361787	3.445399	3.364298	35.42588
3.340877	3.438053	3.418419	35.48244
3.228818	3.364729	3.402135	35.04143
3.110037	3.316003	3.418147	34.71013
3.267381	3.41487	3.354578	35.13141
3.271753	3.422822	3.447475	35.36151
3.246264	3.354552	3.409416	35.07325
3.203619	3.279071	3.48047	34.97031
3.23418	3.231087	3.38273	34.71847
3.419817	3.21498	3.379644	35.08245
3.404474	3.292011	3.36527	35.18592
3.244367	3.320653	3.432351	35.04512
3.245151	3.407389	3.392058	35.1484
3.374243	3.454699	3.419579	35.59435
3.33036	3.435425	3.300695	35.19625
3.37581	3.413589	3.351577	35.35916
3.123318	3.401458	3.455933	35.00869
3.215786	3.410489	3.38655	35.07892
3.271794	3.33986	3.439563	35.16288
3.225849	3.23567	3.392689	34.73205
3.21294	3.155943	3.393933	34.53219
3.364963	3.276443	3.428514	35.20378
3.263587	3.43347	3.486881	35.45312
3.425797	3.482735	3.327569	35.56719
3.379274	3.342286	3.455507	35.43809
3.315182	3.340062	3.35954	35.0832
3.209682	3.385688	3.479515	35.21463
3.244573	3.46036	3.3622	35.19768
3.221312	3.380431	3.420926	35.10045
3.281156	3.330223	3.333042	34.92933
3.206465	3.243892	3.481988	34.90292
3.268247	3.193212	3.364519	34.67031
3.297612	3.227043	3.410371	34.90879
3.338526	3.279341	3.3622	35.00728
3.305614	3.323821	3.366992	35.04306
3.270144	3.400515	3.42836	35.26741
3.348672	3.415611	3.376933	35.35969

3.321657	3.409545	3.365884	35.26318
3.300788	3.441018	3.353743	35.25982
3.242882	3.411635	3.422137	35.2185
3.296705	3.426798	3.411803	35.34677
3.213847	3.271254	3.398418	34.79615
3.362488	3.198805	3.324056	34.80015
3.252203	3.245037	3.397685	34.82109
3.278228	3.374433	3.402868	35.17231
3.308624	3.475726	3.482039	35.63342
3.369129	3.397415	3.372841	35.35568
3.308913	3.335951	3.362422	35.06681
3.386203	3.332447	3.387368	35.28272
3.167036	3.417093	3.442479	35.10906
3.243047	3.431853	3.447612	35.31879
3.162087	3.34296	3.397275	34.83727
3.223622	3.25454	3.417311	34.82229
3.214342	3.220641	3.392279	34.67312
3.333824	3.201838	3.460213	35.04185
3.195205	3.311555	3.404812	34.8575
3.392431	3.369042	3.44016	35.49181
3.320049	3.392967	3.349923	35.18851
3.418456	3.39728	3.352924	35.4197
3.3443	3.409208	3.399049	35.38449
3.25608	3.424574	3.413577	35.25694
3.294973	3.428281	3.388818	35.29596
3.332876	3.412039	3.306169	35.16258
3.262226	3.3034	3.452505	35.09052
3.234675	3.237691	3.379883	34.72777
3.224488	3.202175	3.463418	34.8105
3.299345	3.417767	3.334116	35.1629
3.364427	3.420328	3.419766	35.49813
3.310975	3.405771	3.447305	35.40963
3.220529	3.298009	3.478168	35.04367
3.282146	3.320923	3.371562	34.99539
3.263463	3.391012	3.428156	35.23157
3.257606	3.464673	3.390983	35.29856
3.203577	3.350374	3.459752	35.08084
3.072918	3.2292	3.419374	34.44182
3.336587	3.298885	3.377342	35.0789
3.261689	3.223067	3.356676	34.70411
3.312584	3.292213	3.374921	35.00652
3.25307	3.345656	3.456598	35.17186
3.276496	3.342691	3.390199	35.0714
3.413754	3.398358	3.383788	35.47927
3.489601	3.434953	3.285059	35.50926
3.294231	3.410758	3.501256	35.5019
3.216734	3.425652	3.342693	35.01824
3.209393	3.349632	3.402766	34.96732
3.341784	3.362168	3.297814	35.05473
3.278476	3.267143	3.399117	34.93002

3.179739	3.307511	3.465959	34.94855
3.124844	3.310274	3.417175	34.72786
3.415775	3.43994	3.37591	35.5574
3.331721	3.407658	3.401709	35.35941
3.405918	3.393775	3.406875	35.5026
3.270021	3.3034	3.360768	34.90695
3.298726	3.300705	3.3725	34.98949
3.265113	3.412713	3.337305	35.08396
3.332793	3.380094	3.419289	35.33992
3.132598	3.291269	3.381332	34.62487
3.128597	3.205882	3.425376	34.52571
3.293241	3.225089	3.329888	34.71895
3.27897	3.263031	3.426382	34.98173
3.300046	3.41487	3.416578	35.33843
3.187987	3.325034	3.439069	34.9461
3.276661	3.366481	3.366549	35.07206
3.374903	3.431785	3.379593	35.45824
3.486755	3.465347	3.39528	35.81054
3.276207	3.425518	3.412008	35.29959
3.216734	3.312364	3.375586	34.84243
3.224447	3.284867	3.345916	34.73428
3.364427	3.262762	3.363752	35.03106
3.277692	3.254472	3.317321	34.72172
3.245522	3.340736	3.429452	35.08523
3.204155	3.417498	3.436903	35.17893
3.32838	3.43219	3.415401	35.43569
3.341578	3.417296	3.371545	35.33608
3.441882	3.36857	3.442547	35.60414
3.268536	3.355698	3.460554	35.23629
3.312254	3.419857	3.404625	35.34989
3.253894	3.304074	3.401351	34.96191
3.294725	3.240926	3.361126	34.82514
3.223828	3.191662	3.417362	34.68535
3.198505	3.292348	3.406074	34.82547
3.10913	3.301042	3.488382	34.82903
3.348383	3.422013	3.383975	35.38846
3.349249	3.367761	3.492832	35.50977
3.294725	3.393101	3.367316	35.17146
3.251214	3.422081	3.485858	35.39891
3.411362	3.446342	3.306135	35.40916
3.396885	3.395999	3.36411	35.39419
3.347888	3.297065	3.316809	34.96725
3.156972	3.273208	3.367111	34.60758
3.22457	3.226976	3.341074	34.59736
3.265814	3.320384	3.397139	35.01443
3.193225	3.339186	3.416288	34.93869
3.329947	3.44176	3.364468	35.34867
3.19669	3.393708	3.484989	35.21573
3.309078	3.360348	3.364315	35.12466
3.273898	3.350845	3.409689	35.12617

3.411073	3.436166	3.374495	35.53577
3.252987	3.399436	3.460281	35.29734
3.390699	3.348285	3.363582	35.27517
3.205351	3.223539	3.489814	34.87309
3.331803	3.248811	3.285639	34.75839
3.173099	3.227785	3.375296	34.56141
3.261648	3.288237	3.363087	34.86056
3.158045	3.361696	3.451073	34.98705
3.225931	3.412646	3.379746	35.09094
3.356838	3.359674	3.369038	35.23796
3.454502	3.397819	3.404454	35.6124
3.309779	3.451666	3.392143	35.38675
3.373047	3.423765	3.357699	35.38876
3.319348	3.361763	3.384009	35.19328
3.28825	3.242342	3.323408	34.73159
3.266886	3.273882	3.320254	34.74695
3.153054	3.247598	3.427116	34.67423
3.230015	3.334536	3.36943	34.9065
3.215827	3.415813	3.440774	35.20923
3.304253	3.430909	3.439222	35.43222
3.274681	3.366279	3.382048	35.10119
3.331597	3.355496	3.456956	35.36588
3.208238	3.413993	3.399816	35.09909
3.453471	3.4115	3.394326	35.61791
3.328091	3.436975	3.365611	35.33664
3.332752	3.250631	3.379542	34.96979
3.248285	3.15318	3.339334	34.48404
3.251214	3.193549	3.404983	34.72229
3.246842	3.303131	3.357562	34.84867
3.312625	3.354822	3.439887	35.28559
3.13103	3.423429	3.448839	35.05809
3.282476	3.344376	3.436	35.18832
3.278888	3.338243	3.374358	35.03226
3.380759	3.477883	3.431907	35.68626
3.29786	3.452745	3.419306	35.42244
3.449223	3.370322	3.427627	35.5914
3.25274	3.301176	3.393166	34.93515
3.32937	3.278128	3.349378	34.95657
3.171738	3.306433	3.37458	34.72886
3.324214	3.346802	3.366242	35.13235
3.173594	3.361494	3.381094	34.86757
3.245522	3.373153	3.37238	35.03131
3.218714	3.423092	3.457774	35.26864
3.336381	3.378814	3.360836	35.21714
3.280496	3.359202	3.428838	35.20075
3.271052	3.421272	3.417942	35.29201
3.311388	3.377938	3.454688	35.36581
3.39437	3.332986	3.330962	35.1784
3.295798	3.256157	3.422699	34.99545
3.169717	3.183507	3.385953	34.48049

3.218508	3.224348	3.4301	34.77304
3.206052	3.335412	3.396321	34.91482
3.260988	3.425585	3.43131	35.30867
3.308418	3.413117	3.3725	35.25651
3.229066	3.355091	3.44814	35.1215
3.221395	3.411163	3.399083	35.12006
3.42555	3.463191	3.38996	35.66035
3.336134	3.445668	3.378092	35.40054
3.392761	3.294504	3.371357	35.17907
3.291838	3.158167	3.397872	34.7182
3.32673	3.240522	3.344416	34.8577
3.259462	3.307781	3.345029	34.85903
3.358117	3.394786	3.328779	35.2295
3.135567	3.365403	3.40621	34.84789
3.219704	3.393101	3.371272	35.01605
3.241439	3.402132	3.473513	35.30692
3.357333	3.423294	3.368134	35.37619
3.347228	3.420665	3.437909	35.50093
3.274681	3.390001	3.418999	35.23387
3.27464	3.306096	3.446503	35.11044
3.354116	3.226235	3.333486	34.86245
3.294189	3.209319	3.365082	34.7635
3.263999	3.167737	3.36336	34.60278
3.131195	3.27523	3.441405	34.7181
3.196154	3.373692	3.378314	34.93751
3.309284	3.497225	3.411411	35.52743
3.299303	3.423294	3.42449	35.37253
3.267546	3.41487	3.403567	35.2389
3.253235	3.397819	3.442479	35.25541
3.296829	3.468245	3.382321	35.3732
3.408269	3.39236	3.389091	35.46576
3.357457	3.290596	3.302042	34.94174
3.214878	3.183237	3.402937	34.61581
3.335309	3.275297	3.396252	35.06587
3.219126	3.33521	3.389227	34.92745
3.344919	3.403413	3.326972	35.21555
3.201185	3.33366	3.428241	34.97015
3.175862	3.345589	3.460093	35.01051
3.258514	3.404423	3.403602	35.19638
3.335061	3.456991	3.37562	35.41754
3.289488	3.425787	3.406091	35.31628
3.350651	3.352059	3.43027	35.34168
3.271918	3.307983	3.430697	35.07405
3.356673	3.219091	3.333076	34.85152
3.40035	3.201501	3.339351	34.92229
3.288374	3.24625	3.371562	34.84572
3.207661	3.299626	3.391256	34.829
3.2395	3.356911	3.347587	34.92841
3.256204	3.415611	3.374068	35.15121
3.364221	3.391214	3.359898	35.30309

3.23682	3.377601	3.434056	35.15688
3.236985	3.438794	3.442684	35.30993
3.339722	3.421205	3.43102	35.47063
3.389503	3.335075	3.378689	35.2767
3.349002	3.246318	3.320407	34.86658
3.254224	3.263166	3.357784	34.7779
3.283219	3.330425	3.385254	35.04846
3.271629	3.391214	3.372141	35.12738
3.353332	3.354754	3.334799	35.14466
3.2501	3.297672	3.366958	34.8644
3.220199	3.372546	3.472677	35.19394
3.257606	3.479028	3.408325	35.36788
3.365458	3.407995	3.421335	35.47684
3.345867	3.368435	3.403124	35.30767
3.267793	3.292078	3.43559	35.04095
3.246553	3.281902	3.451568	35.00718
3.365045	3.250833	3.413628	35.1154
3.256163	3.248003	3.378996	34.79537
3.343599	3.312633	3.345643	35.05497
3.150044	3.340804	3.397804	34.80737
3.277362	3.410961	3.331593	35.09442
3.185925	3.351182	3.404812	34.92386
3.254018	3.388788	3.369021	35.07674
3.190421	3.420531	3.448754	35.18144
3.384884	3.454969	3.341756	35.44802
3.24878	3.306837	3.411326	34.97858
3.408681	3.304546	3.324704	35.13382
3.419322	3.254338	3.33473	35.06922
3.307552	3.344376	3.370385	35.09967
3.292581	3.397415	3.435471	35.32525
3.221477	3.397212	3.365491	35.01628
3.287054	3.316812	3.385391	35.02738
3.284786	3.403817	3.327364	35.08579
3.223869	3.459552	3.455507	35.35468
3.269814	3.429629	3.447884	35.37305
3.246058	3.34296	3.396798	35.01986
3.265236	3.245577	3.418692	34.89671
3.312048	3.310207	3.424473	35.15306
3.292292	3.232367	3.434124	34.96074
3.28128	3.207769	3.414191	34.83927
3.208444	3.256966	3.396167	34.74816
3.342609	3.348621	3.358125	35.15881
3.316502	3.39728	3.412809	35.32771
3.295303	3.471682	3.373523	35.35814
3.122369	3.33238	3.439546	34.81971
3.291055	3.389732	3.310977	35.03286
3.254802	3.452947	3.499431	35.50394
3.342444	3.353676	3.351151	35.15425
3.300541	3.249081	3.412144	34.96726
3.294354	3.233041	3.328916	34.73665

3.320173	3.260538	3.368765	34.94038
3.428725	3.415543	3.406875	35.60008
3.315677	3.399706	3.331712	35.15386
3.276455	3.293898	3.436409	35.06566
3.141424	3.33211	3.377342	34.72476
3.286436	3.400043	3.424217	35.29295
3.26004	3.505447	3.381588	35.3725
3.22754	3.421474	3.51621	35.41219
3.137011	3.252653	3.465277	34.73365
3.33403	3.232367	3.389227	34.95383
3.399319	3.31479	3.407302	35.31638
3.386451	3.297672	3.374972	35.1801
3.267711	3.242072	3.41813	34.89323
3.2063	3.266132	3.370505	34.7074
3.314605	3.333727	3.36336	35.07644
3.371644	3.39883	3.339078	35.29045
3.220157	3.40658	3.394411	35.09712
3.106408	3.394314	3.423859	34.88594
3.295798	3.416554	3.456871	35.42094
3.340629	3.377399	3.388818	35.28453
3.360426	3.31607	3.360853	35.13255
3.195081	3.267884	3.463691	34.89048
3.165757	3.321597	3.419101	34.84631
3.319884	3.44149	3.384367	35.36959
3.473598	3.435627	3.372994	35.66804
3.320915	3.337434	3.418607	35.21916
3.19735	3.304344	3.41726	34.87364
3.200237	3.395595	3.413986	35.07234
3.352301	3.533819	3.375722	35.62348
3.276248	3.434414	3.374017	35.23605
3.249151	3.222932	3.42819	34.83279
3.17347	3.208712	3.478441	34.74608
3.3622	3.332582	3.351628	35.15236
3.324462	3.305759	3.443656	35.21243
3.417218	3.270984	3.369771	35.17765
3.213558	3.254944	3.45803	34.89021
3.276908	3.35253	3.286969	34.86807
3.349662	3.374164	3.397395	35.31596
3.376676	3.424574	3.349855	35.38132
3.205433	3.377938	3.384299	34.98017
3.152683	3.417633	3.393166	34.97101
3.196731	3.383127	3.494521	35.21353
3.392266	3.315666	3.355584	35.18977
3.313945	3.230009	3.414054	34.95904
3.219167	3.283047	3.450323	34.94708
3.290725	3.404087	3.452028	35.37199
3.371397	3.46555	3.325181	35.40542
3.403484	3.393573	3.366344	35.4082
3.308954	3.319912	3.280916	34.85358
3.144682	3.371131	3.376132	34.81457

3.20964	3.483881	3.405392	35.26718
3.354693	3.465887	3.396917	35.5265
3.185018	3.311622	3.382935	34.78752
3.253729	3.220843	3.389295	34.75317
3.136227	3.217406	3.503609	34.73868
3.32306	3.345117	3.405324	35.21161
3.270268	3.314453	3.450545	35.12799
3.371892	3.292752	3.388579	35.16726
3.235665	3.316273	3.419306	34.98799
3.37383	3.39108	3.350128	35.30244
3.341867	3.384138	3.425649	35.38252
3.325575	3.428281	3.364536	35.30978
3.124679	3.419452	3.312546	34.73745
3.261401	3.475119	3.489627	35.54542
3.272371	3.364526	3.370402	35.06684
3.304954	3.249688	3.383123	34.91477
3.294395	3.217204	3.453955	34.97554
3.321575	3.38461	3.424268	35.33615
3.318977	3.45106	3.435403	35.50014
3.384182	3.396538	3.354578	35.34675
3.303387	3.242005	3.400396	34.93232
3.250677	3.308455	3.381793	34.92168
3.228241	3.465347	3.449095	35.3629
3.19801	3.495068	3.390966	35.23466
3.21426	3.329819	3.358978	34.83887
3.230015	3.22738	3.470819	34.89389
3.251337	3.245913	3.325096	34.66237
3.27662	3.267143	3.433425	35.00098
3.363272	3.368435	3.453205	35.45524
3.285569	3.28143	3.422154	35.02715
3.322812	3.225628	3.367606	34.86728
3.375934	3.295448	3.303492	34.99592
3.339475	3.341276	3.386226	35.19734
3.264164	3.412309	3.407643	35.23482
3.261524	3.389934	3.385476	35.13164
3.260163	3.364392	3.307908	34.90318
3.326607	3.312903	3.355533	35.04003
3.163984	3.290932	3.409126	34.75356
3.203701	3.299761	3.437108	34.92091
3.177017	3.407658	3.446248	35.1185
3.392431	3.430235	3.417396	35.57585
3.313821	3.402469	3.358603	35.21465
3.446914	3.30832	3.327824	35.23251
3.17215	3.279476	3.441132	34.81635
3.367974	3.426731	3.307294	35.27393
3.262679	3.456114	3.401709	35.31439
3.29819	3.359539	3.31848	34.99885
3.120348	3.218012	3.443315	34.57343
3.151116	3.253327	3.392552	34.60693
3.282311	3.378073	3.40043	35.18386

3.375851	3.476737	3.364247	35.52506
3.314852	3.236344	3.459173	35.07355
3.295592	3.269838	3.432879	35.04717
3.31477	3.307511	3.385595	35.0681
3.422126	3.416083	3.333093	35.42548
3.257318	3.42545	3.379269	35.18654
3.17281	3.336625	3.424814	34.90709
3.248409	3.288169	3.409143	34.93217
3.227292	3.409748	3.35272	35.02848
3.308418	3.309196	3.382679	35.05151
3.226344	3.335547	3.401845	34.97157
3.226962	3.349902	3.410098	35.02236
3.140764	3.403008	3.488655	35.12178
3.467865	3.475389	3.320185	35.62697
3.356797	3.389395	3.45462	35.49001
3.369706	3.361292	3.318344	35.15877
3.252698	3.362033	3.434311	35.15812
3.316007	3.460023	3.354169	35.3356
3.276207	3.391686	3.430151	35.26528
3.296623	3.202782	3.358193	34.73946
3.091643	3.233378	3.369294	34.38239
3.227581	3.290191	3.459872	35.00198
3.27897	3.457395	3.38788	35.32258
3.280249	3.353946	3.471006	35.28093
3.310934	3.27523	3.388886	34.99631
3.323513	3.221517	3.503711	35.15746
3.273526	3.31479	3.411036	35.04946
3.387276	3.434481	3.318958	35.35859
3.334855	3.413724	3.357579	35.28302
3.331226	3.33548	3.410149	35.21894
3.207248	3.328875	3.354306	34.81126
3.252492	3.296459	3.306544	34.73486
3.220116	3.284328	3.338823	34.70812
3.253276	3.368637	3.42038	35.14336
3.197473	3.401997	3.453205	35.16606
3.292127	3.416622	3.372159	35.2278
3.372016	3.351789	3.407575	35.33818
3.438706	3.372411	3.353146	35.41009
3.28891	3.37942	3.420466	35.24506
3.195123	3.495068	3.447731	35.35248
3.262267	3.432324	3.457979	35.38452
3.219951	3.240185	3.353129	34.64251
3.285652	3.14051	3.379405	34.62568
3.168644	3.271051	3.374921	34.64547
3.185925	3.449173	3.482533	35.30811
3.156024	3.45598	3.437244	35.15857
3.354487	3.254607	3.33913	34.93765
3.249564	3.253799	3.425922	34.89623
3.360921	3.317957	3.41165	35.24884
3.318894	3.383666	3.428292	35.33703

3.351353	3.442231	3.389756	35.45181
3.238964	3.36675	3.400481	35.06442
3.421961	3.266873	3.293125	35.01142
3.193968	3.263099	3.41963	34.78123
3.25142	3.305287	3.293176	34.72259
3.137712	3.353946	3.484562	34.99887
3.264906	3.454093	3.394189	35.2984
3.328133	3.418981	3.404829	35.38315
3.328215	3.358798	3.403414	35.24862
3.270516	3.301715	3.405102	35.0013
3.360921	3.362572	3.391546	35.30244
3.322111	3.428955	3.421369	35.42796
3.285487	3.431988	3.37134	35.2451
3.19834	3.211341	3.37903	34.58882
3.148518	3.170096	3.425973	34.49232
3.26037	3.294639	3.352617	34.84887
3.290189	3.44331	3.424609	35.39663
3.222632	3.50147	3.4899	35.51886
3.207289	3.325438	3.452829	35.01929
3.264618	3.281093	3.429793	34.9973
3.402412	3.269164	3.383481	35.17127
3.338815	3.362033	3.364605	35.19401
3.198381	3.439064	3.493838	35.33797
3.288168	3.390877	3.393916	35.21043
3.256905	3.302389	3.397275	34.9559
3.368675	3.276847	3.306357	34.94564
3.248574	3.245846	3.373267	34.76153
3.182131	3.303737	3.366975	34.72906
3.155941	3.406647	3.430066	35.03481
3.307181	3.438659	3.387164	35.34173
3.273856	3.366279	3.397821	35.13388
3.341001	3.409613	3.43247	35.45125
3.223869	3.37167	3.436187	35.12025
3.361705	3.365066	3.341739	35.20069
3.438624	3.476467	3.393337	35.72535
3.368881	3.348285	3.374154	35.25057
3.210424	3.174476	3.388801	34.55599
3.176604	3.2199	3.324005	34.43967
3.238263	3.358124	3.380974	35.00137
3.310439	3.474985	3.366566	35.38325
3.157261	3.377803	3.441183	34.99893
3.200649	3.275499	3.394667	34.76837
3.268123	3.252518	3.428855	34.94043
3.379481	3.386025	3.402033	35.41725
3.330112	3.406176	3.375432	35.29519
3.239336	3.396943	3.411036	35.15434
3.254018	3.306568	3.44473	35.0625
3.293199	3.203927	3.328933	34.67049
3.462751	3.25144	3.324755	35.13604
3.227334	3.310477	3.393302	34.90023

3.262143	3.377803	3.399049	35.13615
3.191782	3.417363	3.419306	35.11309
3.356467	3.400312	3.34005	35.26262
3.371892	3.40166	3.354357	35.33059
3.303758	3.311757	3.361194	34.99994
3.189802	3.402132	3.463555	35.17222
3.330071	3.479702	3.439018	35.59494
3.353044	3.427337	3.350895	35.33795
3.400927	3.18964	3.373693	34.97271
3.17714	3.111867	3.42761	34.43116
3.187781	3.248946	3.43675	34.77419
3.209846	3.425046	3.37504	35.07259
3.263587	3.437649	3.422307	35.32104
3.176357	3.341343	3.431634	34.94007
3.184935	3.251979	3.385015	34.66146
3.200855	3.309196	3.443707	34.94975
3.390245	3.390271	3.370385	35.38087
3.325287	3.434279	3.43588	35.47828
3.252698	3.424439	3.394462	35.20745
3.226509	3.231626	3.415145	34.77376
3.293736	3.227178	3.401692	34.88162
3.390493	3.248205	3.349309	35.02465
3.30285	3.355698	3.335941	35.03882
3.197968	3.429292	3.447748	35.2149
3.237521	3.401256	3.384043	35.10078
3.313945	3.34013	3.45566	35.29084
3.333989	3.432459	3.318906	35.23753
3.332092	3.382453	3.455268	35.42223
3.189885	3.411972	3.411258	35.07955
3.29023	3.43994	3.442155	35.42772
3.290147	3.26074	3.390114	34.92185
3.344919	3.188292	3.371101	34.84162
3.21793	3.217069	3.421318	34.73666
3.250471	3.404087	3.394462	35.15807
3.243501	3.422215	3.495816	35.40412
3.33469	3.428753	3.314268	35.22081
3.175078	3.248272	3.408376	34.68289
3.295262	3.312835	3.364127	34.99013
3.146827	3.393236	3.50054	35.13966
3.279878	3.42235	3.398776	35.27175
3.357333	3.419924	3.422392	35.48747
3.442171	3.324697	3.38343	35.37955
3.280703	3.204669	3.422785	34.85002
3.285817	3.25144	3.300406	34.69587
3.255049	3.248879	3.409536	34.86163
3.290807	3.403547	3.360666	35.17119
3.246842	3.429561	3.403465	35.22553
3.196154	3.379488	3.399526	34.99657
3.24346	3.341006	3.38568	34.98559
3.338443	3.401526	3.403874	35.36543

3.276537	3.394247	3.367725	35.13509
3.283796	3.386295	3.413577	35.23384
3.232943	3.400919	3.426041	35.18187
3.225272	3.167737	3.351748	34.49269
3.384884	3.237489	3.330178	34.94711
3.252327	3.374366	3.444184	35.20587
3.269567	3.440142	3.40176	35.29464
3.22919	3.383262	3.450204	35.18789
3.25872	3.336086	3.403278	35.04668
3.253523	3.284665	3.411172	34.94013
3.295303	3.338782	3.426724	35.18385
3.185884	3.379286	3.503115	35.2002
3.246718	3.469526	3.353197	35.20273
3.261689	3.38616	3.388272	35.12986
3.434334	3.270108	3.369243	35.21201
3.362158	3.154393	3.353265	34.76618
3.284827	3.249553	3.377274	34.85767
3.261236	3.38124	3.356744	35.04917
3.301654	3.448903	3.374955	35.32535
3.219291	3.435896	3.341636	35.04393
3.320461	3.40995	3.405989	35.34916
3.159942	3.376253	3.441592	35.0023
3.301324	3.465617	3.401487	35.4192
3.337949	3.422957	3.4146	35.43467
3.373624	3.363785	3.395076	35.3406
3.28161	3.243757	3.432385	34.95848
3.238882	3.14051	3.327432	34.40974
3.320296	3.264379	3.372858	34.958
3.31081	3.438794	3.369021	35.31029
3.18543	3.437446	3.373608	35.04318
3.148146	3.398223	3.472285	35.09167
3.28858	3.282373	3.381844	34.94765
3.252533	3.265255	3.360291	34.78425
3.2793	3.404963	3.394684	35.22352
3.174253	3.4084	3.459053	35.14208
3.194091	3.389597	3.462225	35.15128
3.285982	3.343365	3.312069	34.92276
3.481022	3.206488	3.393234	35.22745
3.25274	3.211745	3.366225	34.68066
3.239336	3.329953	3.433578	35.05714
3.261813	3.431516	3.392296	35.23812
3.38641	3.413252	3.324073	35.32146
3.331762	3.364122	3.354033	35.16003
3.282847	3.324899	3.348355	34.95487
3.197144	3.389597	3.468125	35.17085
3.260328	3.46656	3.371476	35.26598
3.29819	3.358259	3.468346	35.32378
3.380553	3.308657	3.353385	35.14402
3.256617	3.155876	3.384845	34.60768
3.248945	3.161537	3.388681	34.61167

3.266267	3.394247	3.438319	35.267
3.351435	3.432661	3.446844	35.5559
3.237191	3.359068	3.346786	34.92632
3.199164	3.275095	3.364059	34.6973
3.153714	3.245307	3.420056	34.65522
3.268123	3.380499	3.416271	35.19278
3.289735	3.437918	3.413662	35.35991
3.255627	3.434211	3.374887	35.19242
3.350899	3.262088	3.39037	35.05821
3.294189	3.172994	3.335191	34.61869
3.350899	3.210464	3.349787	34.85657
3.368799	3.285945	3.338737	35.03662
3.264123	3.424709	3.438932	35.33027
3.325947	3.43994	3.332599	35.26624
3.374408	3.410893	3.406262	35.46979
3.276661	3.33676	3.317184	34.89912
3.297118	3.39546	3.362644	35.17163
3.164767	3.444253	3.442974	35.16458
3.269567	3.445264	3.425922	35.35868
3.180069	3.291067	3.439427	34.85529
3.324544	3.186877	3.384163	34.82253
3.223127	3.195368	3.457331	34.77932
3.395442	3.324764	3.363991	35.235
3.248533	3.438929	3.469847	35.39488
3.398783	3.419722	3.296398	35.30215
3.20399	3.309264	3.445412	34.96048
3.266391	3.369985	3.33132	34.98023
3.166871	3.297537	3.435249	34.83144
3.247419	3.377399	3.391972	35.08759
3.220611	3.413117	3.450545	35.23516
3.31213	3.374501	3.449112	35.34772
3.33865	3.301244	3.408717	35.15718
3.330896	3.238231	3.367589	34.91248
3.241356	3.232839	3.3762	34.72371
3.345207	3.310881	3.363888	35.09456
3.395731	3.366279	3.381793	35.36534
3.309697	3.424305	3.357102	35.2501
3.341578	3.401863	3.391324	35.34558
3.241439	3.405232	3.373608	35.09522
3.291014	3.453284	3.408871	35.38583
3.260452	3.459888	3.416578	35.35029
3.284538	3.406378	3.423194	35.30041
3.148559	3.255349	3.452642	34.73717
3.379646	3.216462	3.35301	34.9396
3.269113	3.178587	3.481698	34.89648
3.400061	3.349835	3.331388	35.22863
3.270598	3.37322	3.460963	35.28001
3.338443	3.367761	3.298359	35.06085
3.290519	3.359809	3.421011	35.20688
3.282476	3.384812	3.409382	35.21854

3.185678	3.328943	3.384726	34.83076
3.19636	3.338445	3.395229	34.89787
3.207991	3.374972	3.464731	35.15517
3.365912	3.42545	3.402664	35.47518
3.384017	3.269703	3.408325	35.18656
3.334278	3.157695	3.390114	34.79302
3.273691	3.186742	3.37088	34.68198
3.289158	3.362707	3.423381	35.21542
3.298231	3.4208	3.396662	35.30387
3.359643	3.45261	3.383566	35.4791
3.263752	3.37039	3.38152	35.08512
3.255255	3.35745	3.410695	35.10205
3.346445	3.465482	3.402851	35.52056
3.267299	3.457665	3.430407	35.39064
3.321245	3.383599	3.375927	35.2275
3.158375	3.2385	3.435676	34.68469
3.278269	3.23358	3.360444	34.7716
3.397298	3.322877	3.406807	35.32856
3.361169	3.399773	3.394138	35.39001
3.236119	3.374298	3.428514	35.13601
3.322688	3.33366	3.40974	35.1954
3.246429	3.310072	3.364741	34.87864
3.352837	3.343567	3.296654	35.03569
3.165963	3.376927	3.45375	35.04353
3.082817	3.40685	3.466232	34.95443
3.195782	3.377129	3.39349	34.9774
3.311058	3.283519	3.436886	35.11968
3.348878	3.208645	3.349804	34.84821
3.343846	3.226167	3.410235	35.00768
3.200196	3.320181	3.416765	34.91341
3.335763	3.417296	3.372295	35.325
3.420765	3.424642	3.323613	35.42049
3.43817	3.32901	3.305078	35.20889
3.22721	3.341276	3.323391	34.81442
3.223787	3.364931	3.395144	35.01558
3.281528	3.492305	3.458439	35.55881
3.323802	3.400178	3.322931	35.15346
3.255668	3.218484	3.431532	34.84462
3.091973	3.193616	3.425087	34.41817
3.279177	3.303468	3.415197	35.04615
3.359478	3.4888	3.386755	35.56485
3.365417	3.437177	3.407234	35.50973
3.229932	3.319305	3.420755	34.98525
3.21261	3.284934	3.422103	34.87515
3.255833	3.338175	3.362541	34.95585
3.397215	3.391282	3.42345	35.51437
3.203577	3.406984	3.426451	35.13181
3.241439	3.358394	3.490428	35.24826
3.159035	3.276241	3.385885	34.65978
3.396184	3.204669	3.370675	34.98861

3.320997	3.228054	3.370948	34.87592
3.30318	3.29073	3.419766	35.08078
3.218425	3.413319	3.452659	35.23545
3.429014	3.490687	3.344876	35.62946
3.342815	3.393304	3.327978	35.19104
3.454337	3.389597	3.274044	35.30887
3.139073	3.33366	3.517932	35.03046
3.134742	3.49399	3.349344	35.00293
3.255214	3.361292	3.447936	35.1918
3.326854	3.251507	3.312154	34.81145
3.267464	3.132355	3.43351	34.68639
3.219539	3.268086	3.452028	34.9189
3.212898	3.452677	3.504905	35.42369
3.417425	3.462449	3.409348	35.68335
3.401876	3.424102	3.361587	35.46105
3.271299	3.295178	3.354476	34.87801
3.278228	3.35563	3.42333	35.17593
3.257813	3.392764	3.342762	35.0363
3.274599	3.379757	3.378638	35.12303
3.274062	3.372411	3.374853	35.09751
3.181017	3.277993	3.395758	34.73327
3.189142	3.193212	3.370044	34.50941
3.334732	3.243353	3.421557	35.05009
3.224983	3.313577	3.437841	34.99926
3.304583	3.355293	3.35833	35.09069
3.260823	3.399571	3.449794	35.29184
3.376099	3.407052	3.33664	35.31284
3.358282	3.376253	3.379951	35.30123
3.403773	3.387171	3.326818	35.3084
3.25175	3.417767	3.434363	35.27804
3.313656	3.43994	3.435454	35.46429
3.124308	3.264582	3.331303	34.43898
3.296045	3.176768	3.330843	34.6215
3.214672	3.244498	3.412144	34.76946
3.22919	3.439131	3.457502	35.32603
3.289611	3.480309	3.428548	35.48489
3.380347	3.38461	3.402118	35.41624
3.298272	3.321799	3.342642	34.96933
3.387812	3.335075	3.40737	35.33572
3.246017	3.346128	3.40442	35.04336
3.21294	3.37039	3.427815	35.07524
3.129133	3.366885	3.384453	34.78948
3.214053	3.311488	3.419084	34.92978
3.251997	3.191459	3.371493	34.6462
3.250018	3.209251	3.415026	34.77598
3.204361	3.305961	3.502569	35.07906
3.33733	3.378275	3.40754	35.32017
3.394205	3.416554	3.361928	35.42851
3.359106	3.422957	3.3371	35.31146
3.414496	3.332312	3.418061	35.41142

3.318028	3.404828	3.345575	35.20052
3.320709	3.398088	3.39453	35.2987
3.34962	3.414061	3.294131	35.1773
3.322482	3.287361	3.356607	34.9775
3.17248	3.189303	3.374222	34.47356
3.229726	3.198064	3.419494	34.71691
3.240408	3.367829	3.43663	35.14899
3.310934	3.4795	3.434516	35.5428
3.264906	3.387777	3.412554	35.19354
3.281569	3.322405	3.438046	35.14276
3.321286	3.303603	3.398912	35.10292
3.380182	3.431516	3.361331	35.42926
3.264535	3.361157	3.440791	35.19626
3.270598	3.370929	3.416902	35.17865
3.138908	3.266132	3.432351	34.69527
3.248285	3.281565	3.290123	34.65718
3.254101	3.229132	3.409808	34.81697
3.256328	3.308657	3.391052	34.95473
3.167448	3.303333	3.556111	35.10968
3.276908	3.437109	3.360205	35.21319
3.434376	3.382049	3.413986	35.55474
3.522224	3.42572	3.413423	35.84112
3.333453	3.370255	3.445276	35.37668
3.316708	3.377129	3.374767	35.2009
3.258101	3.389597	3.387045	35.12685
3.321864	3.321799	3.33942	35.01388
3.295014	3.279139	3.357426	34.90125
3.100716	3.186607	3.420943	34.4129
3.191823	3.293156	3.430833	34.86677
3.310109	3.429427	3.438779	35.44082
3.308006	3.450656	3.401112	35.40027
3.260782	3.338445	3.392348	35.03245
3.314646	3.297739	3.35717	34.98429
3.248079	3.365537	3.426638	35.1389
3.429921	3.413791	3.322709	35.41481
3.321781	3.297807	3.423654	35.14544
3.23319	3.310342	3.319111	34.7505
3.154704	3.252585	3.420858	34.67506
3.305201	3.281767	3.379064	34.97659
3.278888	3.302389	3.419084	35.05166
3.255668	3.376186	3.462822	35.25791
3.151281	3.376522	3.420295	34.93737
3.307676	3.392158	3.421949	35.31719
3.362571	3.442097	3.346786	35.38208
3.453719	3.407456	3.409689	35.64321
3.333041	3.384677	3.348849	35.19644
3.319636	3.343634	3.411172	35.21367
3.290931	3.312903	3.343171	34.93498
3.292251	3.316677	3.317014	34.88892
3.266474	3.222663	3.335464	34.6673

3.254554	3.277993	3.371459	34.84095
3.19405	3.393169	3.454552	35.14222
3.279837	3.446949	3.42258	35.37751
3.325782	3.375916	3.438319	35.35707
3.340629	3.325775	3.372022	35.1349
3.303222	3.3435	3.443434	35.24803
3.309326	3.429494	3.364707	35.27727
3.291178	3.352935	3.433561	35.22073
3.30549	3.293965	3.377615	35.00073
3.224777	3.230952	3.408444	34.75384
3.124638	3.247935	3.405835	34.56629
3.283796	3.294033	3.408171	35.02026
3.239047	3.377062	3.39748	35.08059
3.233396	3.424709	3.430884	35.24548
3.220652	3.406984	3.375688	35.05814
3.303057	3.401863	3.422239	35.32895
3.331721	3.445331	3.351151	35.33123
3.470299	3.445197	3.365031	35.66434
3.277486	3.37787	3.386209	35.14177
3.349208	3.358394	3.406262	35.29987
3.313244	3.313981	3.360973	35.02506
3.272536	3.227043	3.336214	34.69178
3.159694	3.239444	3.436971	34.69247
3.242841	3.332784	3.368254	34.92814
3.234758	3.420328	3.439001	35.25662
3.294148	3.379757	3.362541	35.13058
3.266144	3.347745	3.501085	35.3023
3.417713	3.361965	3.369311	35.37669
3.295385	3.395191	3.43438	35.32413
3.322523	3.420328	3.420807	35.40877
3.267505	3.353878	3.445156	35.19638
3.219704	3.245577	3.33323	34.61025
3.240738	3.225561	3.330365	34.60621
3.238593	3.249216	3.359216	34.71634
3.17512	3.279206	3.427559	34.79257
3.217641	3.360955	3.413696	35.03402
3.285693	3.334738	3.382799	35.05794
3.288168	3.390473	3.400345	35.2236
3.355477	3.43064	3.425513	35.51367
3.287384	3.456654	3.390318	35.34469
3.348218	3.421878	3.377206	35.373
3.336876	3.379083	3.33751	35.1678
3.342403	3.311016	3.364673	35.09044
3.213352	3.251642	3.419715	34.79875
3.330154	3.237624	3.303543	34.76947
3.14229	3.26384	3.409689	34.6481
3.263793	3.385823	3.32259	34.99009
3.184399	3.386699	3.463077	35.12561
3.25175	3.410893	3.424831	35.24216
3.190586	3.344106	3.453529	35.02511

3.345744	3.421474	3.359711	35.32844
3.318152	3.403615	3.480214	35.49257
3.366489	3.431246	3.319094	35.30636
3.221518	3.244094	3.416237	34.7925
3.252121	3.192605	3.352174	34.60673
3.243914	3.25427	3.351424	34.72199
3.23286	3.225021	3.418812	34.78122
3.192359	3.391349	3.429486	35.07973
3.203289	3.387103	3.430032	35.09554
3.292168	3.373692	3.414395	35.22638
3.391565	3.432324	3.347485	35.42564
3.391565	3.439738	3.394138	35.54388
3.316997	3.42909	3.394735	35.35883
3.250183	3.415341	3.401811	35.19812
3.292003	3.306703	3.312205	34.85605
3.428272	3.284463	3.347962	35.18361
3.20234	3.25454	3.389909	34.71582
3.254472	3.260875	3.368936	34.79782
3.197432	3.32281	3.454006	34.99456
3.281363	3.465078	3.436477	35.45088
3.285198	3.390877	3.434516	35.29272
3.278352	3.381442	3.446981	35.28437
3.134082	3.362437	3.445156	34.92333
3.380429	3.421676	3.364025	35.41417
3.330978	3.44331	3.435198	35.50899
3.355807	3.306905	3.394189	35.17531
3.199866	3.168815	3.39187	34.52724
3.295674	3.197929	3.355107	34.72003
3.23352	3.230817	3.319913	34.57906
3.332422	3.329347	3.390029	35.16414
3.175779	3.38461	3.439956	35.05163
3.231334	3.416824	3.454552	35.27548
3.175738	3.42417	3.413372	35.07991
3.338361	3.412376	3.402937	35.38693
3.34727	3.41642	3.400805	35.41059
3.352672	3.409208	3.436409	35.4845
3.241027	3.32746	3.399936	34.98182
3.336134	3.285406	3.333213	34.95192
3.355065	3.250429	3.304447	34.85393
3.249481	3.245442	3.338959	34.6876
3.209145	3.304479	3.41049	34.88492
3.245316	3.381779	3.413918	35.14056
3.296416	3.408332	3.359421	35.1912
3.342197	3.415948	3.391751	35.37867
3.264247	3.401391	3.4995	35.412
3.165675	3.474985	3.476923	35.30801
3.270681	3.398291	3.438046	35.2849
3.353456	3.338108	3.417447	35.28926
3.408351	3.255686	3.396781	35.18387
3.200525	3.185529	3.400004	34.58302

3.271052	3.258651	3.387999	34.8709
3.25307	3.256292	3.300354	34.63475
3.299468	3.326449	3.422785	35.15738
3.181389	3.390406	3.363223	34.90877
3.221188	3.31789	3.416186	34.95304
3.168314	3.402065	3.411599	35.01146
3.341743	3.497157	3.38343	35.53707
3.351518	3.433403	3.409433	35.47589
3.349208	3.333727	3.381571	35.19194
3.246058	3.217204	3.442138	34.844
3.299345	3.320721	3.364997	35.01821
3.341248	3.264379	3.378775	35.01676
3.399154	3.373018	3.306698	35.22335
3.154498	3.357248	3.443792	34.95364
3.226385	3.429157	3.363633	35.0928
3.179656	3.392562	3.435113	35.0669
3.348672	3.469391	3.347587	35.41312
3.24313	3.434414	3.450323	35.3305
3.16617	3.404019	3.480214	35.1611
3.18774	3.309601	3.457143	34.95134
3.363478	3.257707	3.364519	35.01961
3.32673	3.210599	3.372602	34.85391
3.256287	3.191729	3.331047	34.56772
3.233479	3.273073	3.395297	34.83623
3.185802	3.33238	3.415538	34.90593
3.355477	3.446545	3.362166	35.40992
3.307965	3.396471	3.427286	35.33893
3.3005	3.406715	3.428565	35.3478
3.156642	3.443242	3.413696	35.08057
3.298809	3.409343	3.34283	35.16236
3.421343	3.355293	3.399441	35.43592
3.356549	3.293291	3.291129	34.92178
3.199659	3.174004	3.479464	34.72969
3.286106	3.290191	3.399799	34.9986
3.279713	3.33676	3.387215	35.05894
3.416311	3.383666	3.308283	35.28762
3.249605	3.344847	3.387454	35.0113
3.15491	3.36729	3.418539	34.92128
3.198051	3.417363	3.415214	35.11785
3.30648	3.453014	3.391188	35.38039
3.266804	3.415813	3.394121	35.21869
3.268907	3.353811	3.442957	35.19449
3.238222	3.258314	3.402493	34.83007
3.274186	3.177172	3.412963	34.75416
3.404433	3.213834	3.335464	34.94969
3.298314	3.269232	3.37932	34.93468
3.203248	3.309803	3.357511	34.76781
3.257813	3.35718	3.354169	34.98343
3.346898	3.406108	3.390745	35.36523
3.253441	3.364729	3.349787	34.9808

3.218301	3.432998	3.392484	35.14662
3.126122	3.462517	3.499431	35.24347
3.264618	3.413185	3.412468	35.24828
3.307263	3.317149	3.393967	35.09107
3.284662	3.218686	3.375023	34.78489
3.244738	3.270714	3.382679	34.8281
3.304871	3.319642	3.416493	35.14055
3.26305	3.431044	3.394189	35.24393
3.389338	3.368435	3.346939	35.27986
3.25307	3.304816	3.360376	34.87212
3.234551	3.384812	3.423739	35.14513
3.187204	3.444186	3.37209	35.05848
3.340794	3.478354	3.414719	35.5623
3.190173	3.420126	3.41049	35.09634
3.291178	3.289989	3.396167	35.00131
3.185637	3.193751	3.437159	34.64969
3.387647	3.231693	3.425291	35.14847
3.236902	3.248609	3.432214	34.87095
3.355065	3.337232	3.369362	35.18571
3.295509	3.333862	3.381793	35.07529
3.278723	3.41588	3.332428	35.10999
3.209022	3.38771	3.430833	35.11115
3.270474	3.411769	3.298905	35.00965
3.205722	3.399167	3.462327	35.19786
3.261689	3.446545	3.416373	35.32337
3.22523	3.350576	3.447867	35.10264
3.299716	3.254472	3.361177	34.86579
3.380759	3.283384	3.398026	35.18683
3.253606	3.348015	3.390642	35.03395
3.338485	3.431314	3.431242	35.49052
3.30516	3.325168	3.369891	35.05135
3.314275	3.269636	3.383839	34.98035
3.283466	3.347341	3.344893	34.99773
3.305036	3.427203	3.408188	35.35796
3.14262	3.477276	3.420483	35.13917
3.279919	3.448162	3.444133	35.42748
3.195246	3.274084	3.433766	34.83896
3.231169	3.277993	3.375705	34.7991
3.220075	3.199277	3.449777	34.76468
3.30648	3.24342	3.376916	34.89083
3.200237	3.243555	3.434226	34.78412
3.401958	3.349161	3.329751	35.22772
3.303098	3.382116	3.416032	35.27228
3.315182	3.416015	3.346666	35.22115
3.180729	3.306433	3.391069	34.78458
3.326194	3.433403	3.33901	35.26651
3.200938	3.496483	3.45034	35.374
3.312336	3.437109	3.381435	35.33709
3.256039	3.248137	3.445412	34.94063
3.298396	3.23722	3.43293	34.98209

3.276166	3.260066	3.385203	34.87906
3.4385	3.408332	3.390506	35.56989
3.332587	3.392764	3.345984	35.20687
3.283878	3.284126	3.404829	34.99146
3.197267	3.280082	3.339965	34.65137
3.245068	3.462517	3.47203	35.44366
3.266597	3.515421	3.34873	35.3368
3.207454	3.411972	3.454091	35.21164
3.193019	3.288911	3.523713	35.06321
3.232035	3.265255	3.426724	34.8847
3.387193	3.288237	3.389892	35.19372
3.256781	3.270175	3.399373	34.88977
3.308171	3.214508	3.471347	35.03781
3.306397	3.287024	3.403823	35.04485
3.347476	3.359607	3.368612	35.2164
3.413424	3.383397	3.262705	35.18104
3.311264	3.353137	3.365645	35.11658
3.121049	3.422687	3.395826	34.91871
3.262968	3.497292	3.482312	35.58134
3.25674	3.418509	3.315974	35.03168
3.338732	3.224415	3.381724	34.93032
3.14926	3.192066	3.416152	34.52051
3.239954	3.258314	3.470427	34.98241
3.258431	3.440951	3.389688	35.24565
3.420642	3.413117	3.343648	35.43883
3.267422	3.324225	3.434635	35.10835
3.331308	3.266064	3.356522	34.95005
3.226014	3.367087	3.467255	35.18286
3.274434	3.564147	3.345149	35.45266
3.231046	3.43064	3.459599	35.3161
3.185554	3.302592	3.357017	34.71227
3.151652	3.155134	3.436324	34.48909
3.231912	3.31971	3.398077	34.94087
3.320255	3.282373	3.445361	35.15582
3.406	3.330964	3.405733	35.36293
3.255957	3.226369	3.439819	34.88062
3.3443	3.351856	3.341056	35.13225
3.336587	3.406513	3.383958	35.32873
3.359725	3.426663	3.302861	35.24605
3.229808	3.341073	3.455643	35.10888
3.159076	3.406041	3.352924	34.87164
3.272	3.431179	3.535547	35.57293
3.275135	3.35435	3.362064	35.03239
3.35061	3.235063	3.350077	34.91037
3.182708	3.191459	3.424404	34.61038
3.299303	3.323753	3.392569	35.08504
3.241851	3.447286	3.446674	35.34787
3.446955	3.433942	3.322794	35.49631
3.32273	3.281565	3.396304	35.05218
3.310893	3.318564	3.403533	35.12302

3.29155	3.530382	3.449385	35.6442
3.336093	3.49035	3.376046	35.49368
3.203907	3.358596	3.428241	35.03063
3.254348	3.271456	3.391597	34.87024
3.116265	3.255281	3.4744	34.71398
3.31444	3.314655	3.421335	35.16115
3.198422	3.280486	3.453989	34.90413
3.321657	3.260538	3.414651	35.04398
3.300005	3.279678	3.422103	35.05478
3.433881	3.372209	3.358159	35.41006
3.288786	3.375175	3.40899	35.2104
3.319966	3.395797	3.315274	35.11875
3.193102	3.334671	3.347076	34.77719
3.314646	3.420665	3.411735	35.37244
3.152188	3.446073	3.339505	34.91478
3.274599	3.278802	3.394121	34.93611
3.297365	3.269771	3.375091	34.92453
3.24544	3.269973	3.410985	34.88992
3.361334	3.398156	3.399714	35.39902
3.308666	3.387979	3.357085	35.16837
3.394823	3.339254	3.436204	35.42325
3.320997	3.283452	3.381707	35.0206
3.308253	3.423968	3.398895	35.3376
3.233231	3.45881	3.420466	35.29691
3.296705	3.397145	3.405358	35.26782
3.159241	3.305557	3.422307	34.80399
3.239212	3.283654	3.363854	34.80315
3.152931	3.276578	3.445429	34.77738
3.244821	3.302794	3.422034	34.9845
3.213022	3.25299	3.430543	34.82465
3.300417	3.308859	3.409212	35.0913
3.268206	3.373759	3.452574	35.25761
3.455781	3.433605	3.388869	35.65937
3.319719	3.344713	3.397736	35.18682
3.309779	3.321597	3.372022	35.0583
3.159241	3.364189	3.349582	34.77317
3.351476	3.452003	3.446589	35.59773
3.168438	3.437446	3.329615	34.90982
3.253729	3.281767	3.384708	34.87637
3.310068	3.188224	3.384197	34.7939
3.302603	3.326988	3.384112	35.08084
3.356137	3.420665	3.442019	35.5294
3.271505	3.371805	3.328438	34.98909
3.359766	3.266334	3.405801	35.12064
3.325905	3.34943	3.350196	35.10671
3.306604	3.459012	3.378161	35.36529
3.210053	3.490148	3.420858	35.31561
3.262019	3.366346	3.393388	35.09844
3.17083	3.286484	3.421881	34.78669
3.257648	3.2848	3.357204	34.83143

3.23385	3.300502	3.465072	35.04961
3.193184	3.294572	3.433408	34.87847
3.221436	3.231828	3.41992	34.77355
3.335639	3.2879	3.433135	35.17481
3.276413	3.353878	3.356966	35.02301
3.441263	3.386564	3.356949	35.45495
3.235376	3.337502	3.390438	34.97065
3.289735	3.372614	3.364451	35.10948
3.231829	3.39054	3.385476	35.06803
3.344176	3.419857	3.333162	35.26342
3.253853	3.32564	3.363496	34.9262
3.214878	3.3003	3.395911	34.85644
3.227581	3.328943	3.41917	34.99772
3.322194	3.366144	3.348968	35.13245
3.348507	3.412106	3.412963	35.43046
3.271217	3.357248	3.422989	35.16339
3.308418	3.280554	3.42524	35.08195
3.390039	3.361089	3.345353	35.26186
3.287508	3.480511	3.37429	35.36208
3.219869	3.429898	3.453938	35.27766
3.223086	3.291809	3.387999	34.83852
3.18576	3.245644	3.36365	34.60269
3.282064	3.307174	3.412963	35.05569
3.296169	3.371333	3.427065	35.25767
3.174831	3.344241	3.485927	35.0618
3.159282	3.27058	3.392228	34.66181
3.418744	3.314588	3.406824	35.35737
3.347929	3.373894	3.375927	35.26464
3.410166	3.388856	3.429384	35.55036
3.255833	3.37012	3.417601	35.14612
3.264288	3.358798	3.354425	35.00169
3.304583	3.336086	3.368902	35.0718
3.373913	3.327864	3.30622	35.06836
3.207083	3.252316	3.358466	34.65257
3.199577	3.283317	3.380548	34.75224
3.207702	3.366548	3.464868	35.13642
3.346445	3.429629	3.415691	35.47023
3.379811	3.412713	3.404181	35.48103
3.251626	3.321394	3.43588	35.07034
3.256328	3.344241	3.459957	35.18323
3.325782	3.420261	3.288913	35.12731
3.323307	3.45598	3.393712	35.42919
3.282806	3.353878	3.345575	35.01208
3.082281	3.213093	3.401624	34.38825
3.115523	3.237354	3.396576	34.50297
3.267051	3.395595	3.39557	35.17818
3.260205	3.388316	3.474519	35.31994
3.262184	3.295448	3.408052	34.97583
3.174377	3.240792	3.378024	34.59862
3.323513	3.315329	3.404642	35.14597

3.421136	3.435694	3.336026	35.47262
3.340712	3.402065	3.340732	35.23349
3.181471	3.323416	3.377751	34.79422
3.325575	3.284867	3.44577	35.1738
3.234015	3.358933	3.321106	34.86292
3.371191	3.305759	3.351475	35.11303
3.157674	3.228863	3.397122	34.57777
3.128597	3.328471	3.456513	34.86189
3.237067	3.387845	3.456956	35.2299
3.347393	3.4053	3.357972	35.29288
3.364386	3.44304	3.419084	35.54621
3.405629	3.318631	3.342779	35.19748
3.246759	3.358259	3.498067	35.2763
3.326029	3.504638	3.355994	35.45907
3.394452	3.4826	3.376745	35.60589
3.270804	3.270175	3.337373	34.78485
3.129587	3.209117	3.394496	34.46742
3.201969	3.224684	3.377342	34.62224
3.233479	3.421744	3.417106	35.20904
3.332009	3.433942	3.408444	35.43225
3.211331	3.261414	3.412758	34.80049
3.230386	3.250766	3.42495	34.84553
3.28825	3.293493	3.444048	35.10727
3.420312	3.437109	3.427286	35.67348
3.304459	3.451869	3.408206	35.41068
3.195535	3.419722	3.349736	34.97432
3.217312	3.293965	3.460213	34.98852
3.226839	3.293359	3.295392	34.6476
3.40101	3.261347	3.33103	35.03641
3.259792	3.271725	3.431856	34.97078
3.175656	3.402671	3.392774	34.98767
3.183121	3.402402	3.408052	35.03682
3.404887	3.400178	3.37516	35.44499
3.381625	3.333458	3.326119	35.14098
3.307016	3.365335	3.421062	35.25515
3.198999	3.401121	3.447185	35.15432
3.386657	3.482937	3.421557	35.68757
3.264288	3.31324	3.32646	34.84091
3.373005	3.210869	3.329564	34.86157
3.110904	3.172657	3.410235	34.38125
3.219374	3.377668	3.387539	35.01715
3.191576	3.492372	3.491213	35.43392
3.251874	3.389395	3.365474	35.06562
3.276331	3.234187	3.426536	34.91322
3.295633	3.259123	3.371067	34.88866
3.25307	3.364189	3.459684	35.21913
3.366406	3.419385	3.359813	35.36929
3.363519	3.442703	3.386209	35.47169
3.237645	3.370592	3.412179	35.09552
3.237356	3.2354	3.356062	34.67652

3.301778	3.228054	3.376592	34.84624
3.285281	3.268625	3.351969	34.84504
3.286889	3.408063	3.36041	35.17194
3.185884	3.41642	3.425308	35.11126
3.297777	3.366683	3.363172	35.1113
3.346733	3.364055	3.466829	35.43929
3.370283	3.356304	3.332002	35.179
3.326112	3.376792	3.387454	35.24847
3.236902	3.491361	3.46879	35.4818
3.28392	3.450925	3.40899	35.36542
3.245893	3.178789	3.353947	34.56677
3.313285	3.114428	3.350963	34.56687
3.219745	3.254001	3.443229	34.86931
3.259462	3.418239	3.396951	35.21413
3.134247	3.389395	3.444952	34.98219
3.289116	3.327056	3.364332	35.00824
3.255585	3.247464	3.414515	34.8706
3.278104	3.272264	3.398128	34.93824
3.267917	3.394651	3.437926	35.27064
3.313326	3.480915	3.457416	35.60121
3.316749	3.41305	3.402459	35.3401
3.432396	3.279071	3.321771	35.12356
3.255792	3.177846	3.387539	34.65981
3.231293	3.206421	3.352856	34.59288
3.157715	3.315059	3.385476	34.74089
3.25142	3.483005	3.418078	35.38437
3.213765	3.439468	3.391989	35.14977
3.292622	3.364998	3.422358	35.22577
3.184853	3.316677	3.373523	34.77763
3.361004	3.36729	3.40355	35.33919
3.356549	3.471615	3.310636	35.35441
3.349703	3.486711	3.434175	35.64261
3.32937	3.253192	3.458371	35.14039
3.197597	3.145362	3.349105	34.37747
3.263298	3.2385	3.391188	34.81685
3.316502	3.452273	3.342574	35.29437
3.22424	3.428887	3.499431	35.38449
3.127442	3.369985	3.429196	34.89041
3.269361	3.162682	3.432606	34.75488
3.327266	3.264582	3.326153	34.87155
3.277197	3.456384	3.450306	35.453
3.230592	3.504773	3.496584	35.55811
3.216528	3.424507	3.466283	35.28556
3.302974	3.234052	3.321413	34.7413
3.446872	3.172994	3.367879	35.02407
3.21925	3.242813	3.351697	34.6436
3.233396	3.371872	3.366242	34.98857
3.162664	3.445668	3.463009	35.20689
3.393916	3.513803	3.342625	35.59834
3.341537	3.374029	3.404079	35.31252

3.35094	3.352463	3.366838	35.20448
3.234716	3.384273	3.532034	35.38114
3.278599	3.412982	3.347741	35.13686
3.294973	3.418576	3.446913	35.40178
3.472814	3.276241	3.394684	35.36521
3.26503	3.100747	3.419084	34.5804
3.240367	3.243285	3.395911	34.7875
3.312584	3.41487	3.425018	35.3843
3.307552	3.460158	3.410081	35.43967
3.233355	3.342691	3.391273	34.97941
3.145053	3.274017	3.459667	34.78569
3.146744	3.291472	3.478714	34.86921
3.317616	3.430505	3.376797	35.32405
3.313285	3.388451	3.444133	35.36987
3.304706	3.347408	3.444952	35.26314
3.269113	3.317823	3.416629	35.05867
3.279507	3.270782	3.340715	34.81252
3.369953	3.273545	3.300644	34.92872
3.314564	3.295717	3.369788	35.00729
3.206712	3.398088	3.382662	35.02346
3.238016	3.40166	3.428412	35.19977
3.346032	3.355765	3.345081	35.15339
3.316914	3.39391	3.387317	35.26549
3.319265	3.434683	3.416765	35.4242
3.173429	3.422283	3.46364	35.18066
3.235954	3.388653	3.445753	35.20474
3.281693	3.389597	3.36411	35.12829
3.388637	3.201636	3.428838	35.09266
3.268288	3.18317	3.332019	34.57737
3.231252	3.267951	3.474195	34.9927
3.268536	3.393977	3.367316	35.1161
3.345579	3.46218	3.299468	35.28536
3.151033	3.315464	3.378604	34.71213
3.244367	3.323349	3.384129	34.94556
3.226261	3.299155	3.44577	34.98786
3.273196	3.391484	3.344347	35.07061
3.308377	3.391282	3.494384	35.47521
3.362406	3.46191	3.415145	35.57454
3.290024	3.291876	3.428292	35.07316
3.342073	3.210195	3.317474	34.76602
3.293736	3.225493	3.42449	34.92779
3.33535	3.296324	3.348696	35.00794
3.187163	3.371131	3.356744	34.86507
3.149425	3.388182	3.404096	34.92339
3.31378	3.379218	3.437654	35.33659
3.276455	3.378612	3.38418	35.1367
3.30384	3.419722	3.395673	35.31162
3.245976	3.423294	3.403891	35.21086
3.30516	3.396471	3.489405	35.46864
3.224529	3.322675	3.390097	34.91376

3.433674	3.238298	3.392228	35.19127
3.263875	3.215317	3.376251	34.73475
3.310233	3.385553	3.38759	35.2332
3.290931	3.372209	3.370999	35.12553
3.339804	3.416891	3.395707	35.38415
3.211372	3.33238	3.39569	34.91844
3.264741	3.246453	3.347468	34.74179
3.149796	3.347543	3.468193	34.9755
3.279424	3.451532	3.35954	35.24877
3.180852	3.433875	3.478083	35.25383
3.40035	3.309196	3.374972	35.2357
3.30318	3.187618	3.417601	34.85056
3.322853	3.18782	3.346718	34.73901
3.218879	3.32591	3.437773	35.01274
3.318564	3.424642	3.369447	35.29723
3.279342	3.442636	3.358807	35.22754
3.28029	3.39391	3.307891	35.01171
3.202423	3.326247	3.426945	34.95381
3.258802	3.447218	3.413491	35.31223
3.277733	3.477613	3.386618	35.36133
3.275341	3.406445	3.400805	35.23149
3.323183	3.255618	3.425581	35.06046
3.195453	3.169691	3.399322	34.5358
3.364798	3.257842	3.325642	34.93777
3.388513	3.431246	3.341739	35.40404
3.270763	3.449038	3.404932	35.32364
3.239171	3.376186	3.410149	35.10665
3.278352	3.334401	3.391529	35.06024
3.227622	3.284395	3.393302	34.84382
3.284085	3.320316	3.362678	34.97888
3.122864	3.407726	3.475014	35.06313
3.234922	3.38434	3.384197	35.05844
3.199577	3.351789	3.455865	35.06668
3.376387	3.303468	3.377342	35.17595
3.254554	3.209251	3.383481	34.71691
3.384017	3.17697	3.373642	34.90792
3.178543	3.372681	3.487427	35.1354
3.429839	3.483005	3.358057	35.64329
3.257276	3.419924	3.395161	35.20912
3.335185	3.352193	3.277369	34.97378
3.155364	3.364324	3.414446	34.90684
3.240408	3.499044	3.455643	35.47751
3.318853	3.461641	3.397753	35.44067
3.375191	3.379151	3.367947	35.3183
3.296788	3.198468	3.469625	34.97407
3.215084	3.206219	3.370402	34.59536
3.28759	3.269838	3.428224	35.0195
3.387729	3.455171	3.364025	35.50338
3.284002	3.40995	3.378911	35.21021
3.206836	3.306096	3.428787	34.92342

3.254596	3.338175	3.362047	34.95207
3.240408	3.348419	3.419647	35.0694
3.272743	3.416824	3.400669	35.2482
3.228777	3.393034	3.471688	35.25534
3.246801	3.376118	3.467937	35.24956
3.175367	3.289989	3.40957	34.77735
3.464566	3.278465	3.386345	35.3338
3.209888	3.231019	3.430748	34.7702
3.362158	3.284934	3.378587	35.10703
3.219745	3.37194	3.422	35.0808
3.407815	3.471952	3.323681	35.49578
3.345909	3.394382	3.345763	35.23906
3.314481	3.36365	3.34196	35.09481
3.185183	3.422283	3.412963	35.09555
3.243047	3.497629	3.369174	35.2911
3.226674	3.401997	3.390847	35.09356
3.344877	3.295111	3.392774	35.12252
3.307965	3.139431	3.37811	34.66928
3.332174	3.204399	3.446384	35.0136
3.325328	3.352396	3.405273	35.23237
3.367768	3.451397	3.412554	35.5576
3.276702	3.339389	3.369089	35.01846
3.269361	3.306635	3.452932	35.11413
3.169222	3.31297	3.41946	34.8358
3.26767	3.351182	3.39985	35.09177
3.239088	3.399975	3.372977	35.0772
3.242388	3.38279	3.409911	35.1276
3.324132	3.346667	3.476446	35.37287
3.20997	3.215789	3.400856	34.67171
3.426086	3.204264	3.352941	35.01433
3.350651	3.258314	3.414242	35.10162
3.296416	3.36729	3.428088	35.25161
3.268412	3.440277	3.394138	35.27574
3.35358	3.419048	3.390114	35.40676
3.347847	3.373692	3.313808	35.12817
3.352136	3.359539	3.385339	35.26303
3.177759	3.431381	3.412878	35.09902
3.2647	3.45288	3.401692	35.3117
3.197845	3.334941	3.372397	34.84352
3.262927	3.189235	3.456666	34.85149
3.277362	3.206219	3.398571	34.79316
3.361828	3.316475	3.471552	35.37858
3.2501	3.429494	3.451482	35.33752
3.363808	3.430774	3.344603	35.35525
3.297283	3.311959	3.433425	35.14418
3.372016	3.383936	3.390728	35.37164
3.20498	3.377601	3.436971	35.09363
3.225354	3.387305	3.4242	35.13148
3.282352	3.39209	3.433749	35.28747
3.270227	3.348082	3.444883	35.18906

3.309738	3.301715	3.446708	35.17806
3.252492	3.239578	3.427013	34.87392
3.272413	3.177239	3.341687	34.59457
3.326895	3.250227	3.365338	34.92504
3.31778	3.365672	3.418061	35.27287
3.250348	3.431246	3.347229	35.1139
3.351559	3.437716	3.454159	35.58322
3.285899	3.430707	3.313399	35.11649
3.344382	3.392158	3.414583	35.38135
3.214507	3.451127	3.362456	35.1123
3.208527	3.390136	3.507616	35.28329
3.165056	3.283384	3.439956	34.80681
3.309532	3.211475	3.369584	34.81161
3.199577	3.175757	3.399987	34.55953
3.358983	3.417902	3.370317	35.37278
3.314935	3.409141	3.491519	35.52234
3.296664	3.397752	3.306936	35.05383
3.323472	3.304344	3.39823	35.10783
3.369335	3.411635	3.321822	35.27566
3.243501	3.3652	3.438932	35.15504
3.231293	3.389327	3.415265	35.12935
3.121586	3.372007	3.454432	34.93721
3.357704	3.39263	3.363326	35.29943
3.279094	3.25919	3.465345	35.05881
3.190916	3.223741	3.416169	34.68091
3.289364	3.215452	3.465874	34.98677
3.305407	3.336693	3.407489	35.15932
3.350487	3.433672	3.376797	35.40286
3.304748	3.402739	3.364997	35.20938
3.281693	3.387171	3.415879	35.23619
3.234304	3.416757	3.35451	35.06305
3.379151	3.454093	3.394615	35.54916
3.253482	3.451195	3.431583	35.34885
3.256864	3.405232	3.381929	35.14715
3.12472	3.183372	3.467647	34.56045
3.311965	3.2261	3.345626	34.79652
3.274764	3.329414	3.420721	35.10532
3.325988	3.443512	3.377939	35.3733
3.320791	3.404828	3.453955	35.44357
3.346527	3.351048	3.42495	35.31881
3.304418	3.32874	3.361263	35.03867
3.462916	3.378679	3.357443	35.48614
3.163736	3.355361	3.427968	34.93511
3.203536	3.351654	3.387863	34.92634
3.109749	3.318362	3.425104	34.72987
3.348424	3.353339	3.346939	35.15738
3.339268	3.268019	3.442888	35.1606
3.185595	3.218484	3.391802	34.6045
3.164644	3.212756	3.467374	34.71142
3.340671	3.360213	3.374648	35.21605

3.37016	3.453958	3.393678	35.52716
3.460318	3.451127	3.336725	35.59358
3.25938	3.351385	3.401913	35.0786
3.22655	3.400043	3.378775	35.06261
3.314069	3.48132	3.345029	35.35794
3.332216	3.42235	3.413781	35.41902
3.218508	3.367222	3.404147	35.02873
3.186626	3.219563	3.394803	34.61567
3.256163	3.216193	3.372994	34.71268
3.27497	3.384812	3.446725	35.28379
3.330896	3.44277	3.415231	35.46396
3.236366	3.362302	3.397872	35.04331
3.360962	3.358124	3.428378	35.37335
3.273031	3.348824	3.42258	35.14804
3.407196	3.361359	3.346189	35.3018
3.22556	3.313914	3.432862	34.99038
3.152477	3.342219	3.398895	34.81817
3.180935	3.362302	3.466147	35.07139
3.350074	3.351856	3.325488	35.11083
3.291302	3.227515	3.389722	34.85086
3.255792	3.236613	3.401913	34.81976
3.165097	3.289922	3.515937	34.98736
3.381584	3.416554	3.362354	35.40184
3.467577	3.433875	3.364264	35.63195
3.39771	3.416689	3.363445	35.43979
3.283425	3.378005	3.390165	35.1637
3.237232	3.369379	3.412008	35.09159
3.260988	3.403615	3.396662	35.18485
3.334979	3.37039	3.321856	35.11041
3.197226	3.193346	3.421011	34.63884
3.115358	3.157156	3.453597	34.45192
3.28029	3.327056	3.415401	35.10062
3.395442	3.437581	3.411514	35.58563
3.371809	3.446814	3.403124	35.5358
3.273114	3.376792	3.44161	35.251
3.300664	3.323416	3.42258	35.15291
3.284909	3.335615	3.398537	35.09256
3.425426	3.392764	3.307908	35.32663
3.243872	3.358528	3.439956	35.1435
3.221972	3.307781	3.423739	34.94917
3.190132	3.230413	3.380411	34.6156
3.334525	3.198131	3.312836	34.71299
3.269113	3.241668	3.402937	34.86219
3.228035	3.344039	3.415401	35.02348
3.200319	3.398223	3.504479	35.27616
3.403484	3.451127	3.419784	35.65093
3.371562	3.432122	3.292272	35.26071
3.477269	3.395999	3.359131	35.55909
3.243584	3.448769	3.39569	35.24341
3.222673	3.361494	3.408495	35.03483

3.149507	3.390338	3.394803	34.90796
3.299675	3.290326	3.337868	34.89313
3.186173	3.2199	3.362951	34.54576
3.200773	3.258584	3.452795	34.85876
3.204856	3.438323	3.486898	35.33533
3.443119	3.446477	3.413713	35.71416
3.461143	3.36729	3.437227	35.63183
3.308996	3.328943	3.388528	35.10875
3.314976	3.339456	3.42906	35.23345
3.314357	3.452947	3.437313	35.49834
3.322482	3.367694	3.406074	35.26136
3.320874	3.278802	3.29512	34.82081
3.151363	3.210464	3.44502	34.62848
3.144476	3.226571	3.389432	34.52708
3.300458	3.239242	3.486967	35.10919
3.272248	3.3528	3.360904	35.02015
3.326194	3.342354	3.470665	35.35531
3.257565	3.378477	3.390523	35.10897
3.332463	3.377129	3.448413	35.3964
3.389627	3.446814	3.310841	35.37295
3.426333	3.435492	3.356744	35.52885
3.245192	3.377264	3.41003	35.12191
3.280002	3.358124	3.332616	34.98689
3.223168	3.280217	3.36486	34.76275
3.327803	3.257505	3.332121	34.8703
3.197473	3.255753	3.406944	34.74509
3.260535	3.362437	3.4109	35.12494
3.217848	3.431381	3.515119	35.41027
3.325864	3.368907	3.34653	35.14119
3.299262	3.345656	3.414719	35.18129
3.392844	3.348824	3.387437	35.3332
3.290065	3.387238	3.500949	35.44068
3.343846	3.440614	3.35272	35.35086
3.22655	3.345858	3.416561	35.02675
3.270433	3.288574	3.346496	34.84422
3.219704	3.263368	3.427695	34.85574
3.253771	3.283317	3.352396	34.80919
3.140104	3.288708	3.438131	34.7599
3.267505	3.354283	3.391137	35.07914
3.319183	3.39081	3.411974	35.31759
3.396349	3.370457	3.393371	35.40115
3.399772	3.419183	3.384947	35.49677
3.341454	3.448566	3.420466	35.51117
3.302232	3.411567	3.362541	35.21782
3.334195	3.342219	3.353334	35.11593
3.263628	3.314251	3.31391	34.81423
3.133629	3.207364	3.417465	34.52266
3.269608	3.2972	3.388545	34.95324
3.1738	3.376118	3.463708	35.08067
3.288745	3.455306	3.451568	35.47866

3.278434	3.408063	3.385203	35.20767
3.238428	3.351924	3.410695	35.05316
3.219003	3.328134	3.42703	34.99438
3.403814	3.409613	3.407302	35.53357
3.317698	3.44176	3.415469	35.43341
3.305944	3.323686	3.416356	35.15144
3.274929	3.201299	3.380821	34.73826
3.286683	3.215384	3.441797	34.92811
3.165634	3.285878	3.364809	34.6492
3.304376	3.342691	3.415469	35.18763
3.153384	3.423631	3.404369	35.01016
3.286848	3.327527	3.404386	35.0919
3.238263	3.383801	3.396474	35.09141
3.453265	3.458069	3.422904	35.7818
3.369706	3.481926	3.41801	35.64054
3.317368	3.367694	3.337424	35.10005
3.283548	3.274488	3.411462	34.98417
3.355642	3.279813	3.319401	34.95215
3.284002	3.234726	3.406279	34.88687
3.251791	3.315599	3.361672	34.89574
3.080425	3.330493	3.47133	34.79337
3.215414	3.450588	3.406824	35.21013
3.294767	3.460967	3.465755	35.53523
3.328628	3.369446	3.389892	35.24324
3.281569	3.40193	3.467681	35.38148
3.297365	3.371131	3.424677	35.25463
3.277527	3.399639	3.427218	35.27914
3.354405	3.339658	3.346871	35.14039
3.213352	3.212823	3.361177	34.58585
3.169964	3.128985	3.454023	34.51066
3.307676	3.2261	3.329461	34.75179
3.217064	3.316205	3.39939	34.90361
3.279053	3.401458	3.364536	35.14938
3.177718	3.412713	3.484307	35.21431
3.278022	3.362707	3.353743	35.03878
3.327225	3.394247	3.444474	35.41377
3.464855	3.485902	3.290908	35.57935
3.298767	3.407389	3.433135	35.35548
3.392844	3.376994	3.32808	35.265
3.298025	3.279813	3.441115	35.09232
3.386698	3.237557	3.279603	34.84062
3.278311	3.2416	3.34861	34.76335
3.268618	3.318092	3.40101	35.02402
3.136186	3.320384	3.398486	34.7339
3.219374	3.428213	3.454552	35.27423
3.231376	3.375444	3.363428	34.98581
3.354281	3.37504	3.41315	35.36243
3.277981	3.425113	3.424336	35.32954
3.301861	3.41797	3.504769	35.54203
3.274104	3.456856	3.425632	35.39331

3.311058	3.251709	3.386618	34.94019
3.314605	3.161469	3.317696	34.59988
3.274764	3.172994	3.408103	34.73566
3.22688	3.269299	3.392842	34.80818
3.207248	3.37039	3.369294	34.93482
3.323101	3.378679	3.328575	35.11725
3.289488	3.314183	3.358671	34.96852
3.315141	3.390608	3.424763	35.33628
3.193803	3.491159	3.445685	35.33658
3.363561	3.461641	3.3991	35.54138
3.434623	3.384205	3.323681	35.36252
3.377418	3.258786	3.357102	35.03623
3.265855	3.225695	3.430151	34.87965
3.297984	3.310409	3.38152	35.02881
3.21925	3.324899	3.3873	34.90096
3.304376	3.349161	3.383447	35.13175
3.242759	3.36392	3.42836	35.1275
3.186255	3.331773	3.472763	35.03073
3.166376	3.325573	3.395775	34.80534
3.295385	3.432998	3.409672	35.35278
3.250801	3.386362	3.427832	35.19301
3.39272	3.376927	3.414804	35.45424
3.207826	3.31014	3.449521	34.97977
3.265319	3.185327	3.337305	34.58715
3.34133	3.204601	3.376387	34.881
3.313904	3.312296	3.408325	35.12637
3.175779	3.361157	3.416441	34.94892
3.241934	3.430842	3.36365	35.13053
3.210506	3.395191	3.423177	35.11401
3.357828	3.395123	3.29529	35.15637
3.297777	3.38124	3.484562	35.4086
3.184358	3.413454	3.435181	35.12302
3.326937	3.361224	3.376285	35.1918
3.369706	3.238635	3.320867	34.89606
3.326359	3.223471	3.395366	34.93103
3.251667	3.27961	3.380156	34.85719
3.276372	3.315396	3.414054	35.06361
3.255544	3.383936	3.365219	35.06115
3.368881	3.392832	3.358824	35.31447
3.170212	3.371468	3.410627	34.94657
3.292127	3.400986	3.41315	35.28325
3.152559	3.417026	3.457143	35.10932
3.339392	3.420665	3.349599	35.29067
3.282022	3.361696	3.449044	35.25373
3.313656	3.290865	3.379201	35.01528
3.23649	3.206825	3.449794	34.81712
3.34995	3.145025	3.368919	34.75323
3.259998	3.168546	3.42449	34.72948
3.385915	3.286013	3.268144	34.91982
3.110326	3.337232	3.456342	34.84072

3.216528	3.477748	3.37088	35.19336
3.213558	3.400784	3.411172	35.10667
3.349084	3.483948	3.347417	35.44549
3.255874	3.442434	3.411718	35.29148
3.215909	3.410893	3.441115	35.1994
3.207743	3.33083	3.433152	34.98904
3.356591	3.252383	3.378468	35.02341
3.346486	3.213901	3.327995	34.80678
3.314481	3.250766	3.370607	34.91059
3.266721	3.344915	3.319776	34.90088
3.172769	3.344645	3.429861	34.93557
3.300829	3.362235	3.359677	35.1006
3.274392	3.361022	3.378706	35.08175
3.291302	3.426057	3.404284	35.31689
3.188854	3.447084	3.442684	35.2228
3.309862	3.449645	3.397258	35.39369
3.370531	3.399639	3.414037	35.4537
3.291632	3.302322	3.374136	34.98109
3.163943	3.161739	3.450545	34.56151
3.311841	3.228459	3.387727	34.89348
3.224818	3.295246	3.427269	34.9357
3.354033	3.287563	3.335344	35.00044
3.244615	3.343837	3.373062	34.96671
3.176852	3.31452	3.409553	34.83421
3.236696	3.353137	3.392416	35.01205
3.298809	3.488329	3.370453	35.3955
3.282476	3.491159	3.414105	35.46143
3.293983	3.399032	3.39378	35.24068
3.254018	3.286282	3.410627	34.94356
3.275918	3.239848	3.366173	34.79269
3.403278	3.347004	3.377615	35.33056
3.331143	3.404491	3.350878	35.24006
3.269031	3.387777	3.384896	35.14207
3.238139	3.331773	3.35318	34.88269
3.2753	3.331975	3.39482	35.05545
3.239377	3.400986	3.359677	35.05096
3.189184	3.444388	3.436	35.20301
3.135691	3.42545	3.443383	35.06077
3.247749	3.339793	3.432453	35.0946
3.243336	3.277252	3.441627	34.96824
3.297159	3.27213	3.343	34.85906
3.24746	3.252653	3.4124	34.85955
3.255173	3.245846	3.449914	34.94357
3.235871	3.298144	3.359677	34.81839
3.431942	3.377533	3.357989	35.41709
3.323307	3.432931	3.384316	35.35824
3.287137	3.38151	3.39141	35.18221
3.131278	3.344982	3.398213	34.77637
3.303675	3.426057	3.36781	35.26418
3.238428	3.438457	3.399373	35.21764

3.29654	3.345791	3.40928	35.16374
3.272124	3.256562	3.390813	34.87483
3.357374	3.22091	3.409825	35.02487
3.332257	3.322742	3.37354	35.11328
3.450337	3.4053	3.392279	35.59302
3.259297	3.385284	3.383208	35.11164
3.201557	3.33858	3.421676	34.96736
3.227994	3.377331	3.382338	35.02389
3.273114	3.457193	3.399185	35.33405
3.257235	3.459484	3.44548	35.40558
3.176975	3.386969	3.467255	35.1191
3.152807	3.237759	3.507701	34.8284
3.356632	3.309129	3.436937	35.27546
3.337495	3.220371	3.382202	34.91981
3.259545	3.214845	3.379934	34.7323
3.278847	3.21808	3.453	34.94137
3.166788	3.327797	3.407694	34.83717
3.386492	3.432796	3.348303	35.41737
3.314522	3.434751	3.399322	35.37582
3.297035	3.434953	3.448891	35.44642
3.165881	3.376792	3.415896	34.96027
3.313656	3.434548	3.417055	35.41227
3.272289	3.385958	3.329274	35.02358
3.340836	3.321327	3.415401	35.22049
3.161385	3.173465	3.410849	34.49476
3.373129	3.229672	3.365679	34.98194
3.267587	3.34296	3.373062	35.01503
3.445223	3.383194	3.344416	35.42883
3.306439	3.342152	3.387931	35.13074
3.224075	3.288439	3.377155	34.8096
3.11111	3.370457	3.391342	34.77294
3.358694	3.550668	3.347076	35.61166
3.212692	3.456923	3.487222	35.39385
3.188276	3.406243	3.375773	34.98591
3.152724	3.206758	3.485057	34.7109
3.363148	3.286821	3.401709	35.16389
3.416682	3.28453	3.376592	35.22102
3.261566	3.237557	3.412741	34.85813
3.24115	3.231626	3.422239	34.82129
3.306232	3.302322	3.427099	35.12884
3.299015	3.382723	3.346803	35.11329
3.342403	3.401593	3.38198	35.32636
3.169634	3.403884	3.442888	35.08675
3.11907	3.429966	3.424967	34.99402
3.31877	3.448701	3.403755	35.42532
3.293199	3.304074	3.381247	35.0039
3.304748	3.248205	3.359233	34.85884
3.226468	3.202445	3.372585	34.61678
3.26734	3.360887	3.410371	35.13528
3.303716	3.419115	3.352242	35.21505

3.436438	3.410287	3.343853	35.46763
3.276331	3.288776	3.420721	35.01988
3.193597	3.309803	3.39424	34.82703
3.188524	3.453419	3.427423	35.20256
3.328669	3.527821	3.409212	35.63192
3.172232	3.363044	3.397668	34.90423
3.227127	3.250429	3.417652	34.82171
3.15124	3.257977	3.502501	34.85781
3.392679	3.343297	3.41003	35.37016
3.242759	3.268153	3.427934	34.91714
3.407815	3.292954	3.390983	35.25152
3.27365	3.277117	3.497317	35.15602
3.344589	3.373355	3.342216	35.18243
3.287755	3.427135	3.399032	35.3
3.277774	3.412982	3.368748	35.181
3.170789	3.366616	3.416646	34.95039
3.186296	3.420665	3.381247	35.02509
3.261813	3.407254	3.3873	35.17414
3.36286	3.271254	3.402732	35.13145
3.334525	3.222056	3.368646	34.88736
3.168933	3.237557	3.401283	34.6305
3.338113	3.364931	3.394667	35.26455
3.335556	3.407389	3.377547	35.31437
3.376511	3.37659	3.333861	35.24104
3.3443	3.320721	3.340443	35.06282
3.267464	3.402334	3.429725	35.26851
3.191782	3.500257	3.411445	35.27718
3.319018	3.461641	3.449249	35.55364
3.147981	3.319777	3.391	34.742
3.246966	3.258314	3.339113	34.71058
3.157055	3.262425	3.445975	34.75664
3.271918	3.289248	3.416152	35.00127
3.259009	3.2354	3.408853	34.83932
3.308336	3.255012	3.397719	34.96573
3.247955	3.358191	3.412264	35.09113
3.426704	3.388114	3.335685	35.38
3.278599	3.349902	3.393865	35.09978
3.240573	3.362707	3.395962	35.04921
3.190256	3.378612	3.342438	34.85691
3.311965	3.505649	3.442616	35.61995
3.258431	3.378949	3.406671	35.1472
3.296375	3.223269	3.399322	34.87366
3.284951	3.240455	3.444321	34.98467
3.339145	3.350172	3.400822	35.24799
3.345042	3.404693	3.429349	35.4425
3.347393	3.370524	3.2972	35.08393
3.307057	3.274556	3.398162	35.00664
3.29489	3.301311	3.312734	34.85173
3.271423	3.447758	3.40089	35.31345
3.197968	3.491901	3.424336	35.30062

3.200814	3.380094	3.409331	35.02953
3.186008	3.2385	3.415026	34.69996
3.231499	3.285204	3.435181	34.94565
3.271134	3.336491	3.424422	35.12095
3.266185	3.331369	3.417874	35.08461
3.18906	3.246048	3.341125	34.56153
3.361622	3.230278	3.385595	35.00166
3.329164	3.317081	3.369106	35.08444
3.365417	3.343634	3.357375	35.19613
3.256658	3.410422	3.372261	35.1369
3.230551	3.406041	3.432743	35.2025
3.259751	3.438457	3.360427	35.1791
3.39008	3.363987	3.335378	35.24648
3.202299	3.279813	3.413935	34.82354
3.179533	3.263908	3.452642	34.82362
3.238717	3.315329	3.401419	34.95348
3.423116	3.434414	3.411087	35.63829
3.403649	3.426731	3.399731	35.55409
3.312831	3.289854	3.38568	35.02543
3.220364	3.291269	3.449453	34.96577
3.324792	3.39856	3.322914	35.15205
3.269155	3.453217	3.41431	35.34977
3.313697	3.366481	3.312768	35.03545
3.056998	3.17542	3.499295	34.46417
3.160561	3.236141	3.403482	34.61391
3.284827	3.407726	3.461918	35.38868
3.360797	3.377062	3.385868	35.32144
3.3045	3.260134	3.452437	35.0882
3.203784	3.274017	3.433681	34.8573
3.287178	3.369918	3.383208	35.13901
3.435448	3.4301	3.339965	35.5003
3.289776	3.396201	3.409212	35.25904
3.139032	3.338984	3.380701	34.74191
3.260122	3.346128	3.378928	35.01846
3.278599	3.412174	3.373471	35.19136
3.334072	3.336086	3.319094	35.02737
3.228818	3.267345	3.411104	34.84808
3.152848	3.236344	3.450681	34.7007
3.204815	3.363313	3.419766	35.0244
3.455946	3.485633	3.34092	35.66865
3.350899	3.411702	3.405392	35.41825
3.390121	3.338243	3.312819	35.14093
3.231004	3.343702	3.48579	35.18317
3.296623	3.487857	3.356829	35.35989
3.301531	3.450453	3.441933	35.47494
3.278022	3.272197	3.301582	34.72678
3.143445	3.185664	3.444645	34.55611
3.172026	3.276038	3.382338	34.67999
3.276331	3.384879	3.453136	35.30093
3.328957	3.374096	3.420807	35.32174

3.27332	3.27159	3.417345	34.96833
3.183327	3.294504	3.464373	34.92448
3.300623	3.361898	3.381588	35.14733
3.417796	3.401256	3.378792	35.48352
3.281363	3.434481	3.40205	35.30869
3.222384	3.384071	3.358978	34.97528
3.22457	3.292011	3.370965	34.80495
3.247254	3.355293	3.347672	34.94201
3.336299	3.280958	3.317304	34.90777
3.222137	3.251035	3.453818	34.89121
3.242882	3.383531	3.407643	35.12535
3.209022	3.406041	3.436341	35.16328
3.40633	3.443242	3.349395	35.48598
3.426374	3.336491	3.338107	35.27168
3.280785	3.354957	3.393166	35.11409
3.236325	3.413319	3.4279	35.22045
3.382409	3.487318	3.418266	35.68067
3.215703	3.354148	3.371425	34.92245
3.291385	3.196851	3.389159	34.78275
3.070196	3.202647	3.431703	34.40476
3.134041	3.336221	3.442564	34.86024
3.199866	3.436368	3.443656	35.22558
3.22457	3.281026	3.480879	35.0213
3.303057	3.267749	3.37857	34.94016
3.329452	3.283654	3.443434	35.17452
3.295757	3.347274	3.419766	35.1882
3.369953	3.400649	3.365201	35.34785
3.286229	3.390136	3.39291	35.20237
3.247502	3.344915	3.419784	35.07755
3.17149	3.34505	3.364178	34.79002
3.279053	3.287293	3.37811	34.92941
3.245316	3.261953	3.384299	34.81375
3.286477	3.332717	3.39794	35.08834
3.213806	3.377533	3.472729	35.19098
3.305861	3.428416	3.393319	35.3299
3.321534	3.382857	3.431515	35.34808
3.38542	3.369177	3.321362	35.21698
3.292704	3.37504	3.39644	35.19123
3.228241	3.461708	3.418948	35.28901
3.304913	3.424439	3.419494	35.37637
3.207331	3.203927	3.369226	34.57082
3.264123	3.198131	3.373693	34.69212
3.182337	3.225358	3.397395	34.62464
3.174212	3.397819	3.447816	35.09428
3.096221	3.351789	3.395485	34.70862
3.305119	3.279745	3.338823	34.88399
3.270062	3.258718	3.417379	34.93313
3.38212	3.341006	3.391665	35.3019
3.272536	3.378544	3.472643	35.32144
3.328298	3.42909	3.437108	35.4762

3.348259	3.385284	3.367452	35.27173
3.40468	3.319103	3.397446	35.31598
3.166995	3.348285	3.424404	34.91897
3.251874	3.331032	3.393848	35.00004
3.171325	3.254675	3.451704	34.78343
3.250348	3.438794	3.326716	35.08555
3.279713	3.376994	3.466215	35.31968
3.244202	3.424305	3.39673	35.19353
3.326937	3.314453	3.379866	35.09735
3.299757	3.333525	3.431958	35.19354
3.308666	3.458136	3.372704	35.35595
3.293323	3.496483	3.442275	35.5584
3.290684	3.363179	3.393592	35.15465
3.196154	3.188494	3.400618	34.58128
3.281198	3.241802	3.343375	34.75866
3.248327	3.346532	3.438199	35.12317
3.263422	3.462854	3.505996	35.55881
3.164066	3.3528	3.409638	34.89015
3.348177	3.243622	3.392024	35.01549
3.406413	3.265997	3.383072	35.1722
3.296375	3.339591	3.395212	35.11905
3.250224	3.451195	3.490343	35.47022
3.322111	3.452812	3.437023	35.51436
3.280744	3.294235	3.423467	35.04747
3.404103	3.209521	3.297882	34.85735
3.237727	3.206758	3.351867	34.60553
3.193349	3.331908	3.383771	34.85193
3.148971	3.451599	3.447543	35.15609
3.287549	3.478152	3.38609	35.38281
3.27101	3.374231	3.371869	35.08829
3.380264	3.3652	3.382406	35.33051
3.160808	3.359741	3.495492	35.08595
3.353167	3.460428	3.347417	35.40298
3.468278	3.428955	3.444883	35.79903
3.297283	3.411163	3.342591	35.16248
3.200196	3.134714	3.399185	34.46938
3.192153	3.183844	3.393524	34.54685
3.253276	3.33831	3.386499	35.00295
3.324998	3.518993	3.398418	35.58098
3.254967	3.468043	3.444338	35.41683
3.101376	3.249216	3.44872	34.612
3.257318	3.228054	3.346325	34.68282
3.383358	3.337097	3.399322	35.3128
3.347599	3.416689	3.381315	35.36928
3.264082	3.432527	3.459207	35.39161
3.256823	3.361763	3.470307	35.24527
3.265608	3.154797	3.351032	34.55104
3.446419	3.246587	3.317611	35.07409
3.206671	3.24187	3.404966	34.73051
3.252616	3.400043	3.377922	35.11775

3.140887	3.440816	3.456052	35.13344
3.356879	3.395662	3.333145	35.23825
3.359313	3.400919	3.453136	35.51747
3.296746	3.43684	3.380377	35.30009
3.215332	3.409074	3.404574	35.11424
3.385007	3.421474	3.433544	35.57577
3.304706	3.35563	3.36261	35.10105
3.397669	3.193346	3.355942	34.93488
3.238016	3.142599	3.376046	34.51873
3.17512	3.298885	3.396031	34.76666
3.246512	3.435762	3.325864	35.06867
3.318523	3.410422	3.401709	35.33659
3.189596	3.317283	3.413219	34.87614
3.290931	3.264379	3.412417	34.9803
3.173841	3.291809	3.42906	34.82062
3.361622	3.431381	3.409194	35.49305
3.380718	3.453756	3.407762	35.5806
3.356467	3.414465	3.440518	35.51328
3.304913	3.248677	3.390318	34.92821
3.307882	3.135051	3.274231	34.43236
3.286353	3.230278	3.382406	34.83008
3.288126	3.422485	3.351731	35.1872
3.192937	3.471548	3.442547	35.28493
3.166541	3.410489	3.368117	34.93092
3.337412	3.313779	3.441405	35.25337
3.347146	3.388047	3.341091	35.21769
3.260947	3.407995	3.453341	35.31829
3.237315	3.444725	3.434243	35.30516
3.218961	3.422957	3.411786	35.16832
3.253688	3.237624	3.39262	34.79705
3.365045	3.159717	3.330911	34.73525
3.273733	3.270108	3.408785	34.94727
3.268577	3.472087	3.378843	35.31221
3.228612	3.435492	3.421813	35.23876
3.314399	3.380094	3.382389	35.219
3.147899	3.285541	3.396116	34.67814
3.299221	3.302457	3.362865	34.97333
3.134206	3.357113	3.502108	35.0365
3.348507	3.471278	3.398776	35.52883
3.245811	3.383464	3.440774	35.20406
3.454544	3.235467	3.356352	35.15226
3.351683	3.155134	3.393456	34.83278
3.314234	3.26876	3.3718	34.95201
3.245316	3.435762	3.456871	35.35254
3.33102	3.462382	3.346513	35.35684
3.253729	3.405771	3.370692	35.1169
3.278352	3.33184	3.402186	35.07794
3.176439	3.325842	3.405426	34.84904
3.277816	3.435829	3.494674	35.50643
3.316296	3.479298	3.383003	35.44144

3.358776	3.426057	3.448771	35.56173
3.274887	3.220034	3.403738	34.82926
3.276207	3.158639	3.418948	34.73114
3.258844	3.219091	3.39482	34.77261
3.344424	3.453351	3.36411	35.40489
3.20564	3.457125	3.400549	35.18933
3.164314	3.334469	3.399731	34.82894
3.282064	3.228054	3.393865	34.8409
3.227499	3.297402	3.395025	34.87576
3.284538	3.352328	3.437654	35.21384
3.244986	3.424372	3.430884	35.27009
3.271134	3.324225	3.408666	35.05967
3.222797	3.290461	3.32363	34.69417
3.472113	3.236141	3.389756	35.26521
3.291756	3.260336	3.346257	34.82858
3.277609	3.36702	3.399697	35.14781
3.265195	3.401121	3.384504	35.16201
3.336134	3.40348	3.346871	35.24
3.269237	3.386497	3.346939	35.05672
3.297819	3.351654	3.350554	35.05093
3.20997	3.412174	3.467306	35.24649
3.254761	3.497225	3.388272	35.35759
3.215249	3.390203	3.460775	35.1957
3.429674	3.357652	3.309545	35.26271
3.324792	3.149406	3.437227	34.85717
3.314522	3.181755	3.398111	34.81992
3.255173	3.299357	3.477418	35.12074
3.310274	3.476872	3.376592	35.40894
3.207619	3.36729	3.399441	34.99478
3.234469	3.356372	3.36839	34.96172
3.140063	3.279071	3.390387	34.63432
3.265938	3.308724	3.375569	34.94204
3.306274	3.439333	3.407029	35.38466
3.23682	3.451734	3.439683	35.3313
3.33799	3.368233	3.389705	35.26065
3.240532	3.198603	3.372176	34.63824
3.378697	3.217406	3.341824	34.91513
3.356219	3.25636	3.395076	35.06761
3.305036	3.411028	3.397668	35.29959
3.232077	3.397752	3.380838	35.0742
3.322565	3.361426	3.339437	35.1021
3.285899	3.32126	3.356761	34.97197
3.312625	3.402132	3.372568	35.24184
3.214425	3.466763	3.476582	35.39589
3.250059	3.456788	3.441678	35.37567
3.207661	3.364729	3.434584	35.06612
3.358158	3.249283	3.377138	35.01715
3.272784	3.195503	3.441695	34.85402
3.410331	3.266199	3.378928	35.17215
3.236943	3.443579	3.49568	35.4362

3.307593	3.440412	3.337834	35.23859
3.315553	3.352396	3.392365	35.18277
3.313739	3.279139	3.343887	34.91259
3.178378	3.259999	3.387795	34.67074
3.213682	3.383194	3.430697	35.11117
3.244326	3.463393	3.490854	35.48512
3.33469	3.407456	3.438523	35.44597
3.351394	3.255483	3.403209	35.07293
3.254843	3.181957	3.369925	34.62821
3.30285	3.220776	3.399236	34.88218
3.287343	3.38279	3.381298	35.16334
3.36587	3.447084	3.346803	35.40023
3.242965	3.418037	3.372227	35.12354
3.329411	3.378612	3.393575	35.27305
3.245687	3.407321	3.419903	35.21032
3.295633	3.481926	3.405324	35.45081
3.220652	3.445601	3.431686	35.26505
3.233974	3.324292	3.449232	35.06727
3.154704	3.146912	3.442564	34.49144
3.365788	3.234389	3.361723	34.96755
3.240037	3.319642	3.428838	35.02576
3.359436	3.437514	3.393848	35.46812
3.302809	3.450925	3.435062	35.46374
3.299386	3.335749	3.397463	35.12216
3.263422	3.338378	3.381025	35.01331
3.322111	3.341073	3.374631	35.13357
3.19735	3.372816	3.393183	34.97072
3.202876	3.410287	3.407916	35.09697
3.194174	3.383868	3.453068	35.11891
3.370737	3.308927	3.358313	35.13392
3.375892	3.226235	3.470597	35.20991
3.215126	3.206758	3.437278	34.74288
3.291343	3.30185	3.404215	35.04521
3.319348	3.365133	3.40812	35.25338
3.320173	3.410287	3.349787	35.22636
3.342733	3.389732	3.392433	35.324
3.165097	3.354485	3.333622	34.72985
3.251667	3.440816	3.370078	35.18768
3.315141	3.507603	3.403346	35.54529
3.295385	3.42262	3.409348	35.32937
3.29885	3.272197	3.346036	34.86954
3.144682	3.190381	3.431293	34.53993
3.254018	3.222528	3.417465	34.81909
3.354652	3.361628	3.369567	35.23861
3.358158	3.404154	3.397054	35.39938
3.230344	3.351654	3.370129	34.94618
3.31015	3.317351	3.428036	35.17232
3.243955	3.335143	3.327006	34.84554
3.340217	3.309061	3.336623	35.02004
3.173758	3.362168	3.429742	34.97579

3.089045	3.413656	3.500813	35.05856
3.259586	3.373827	3.409877	35.14554
3.3808	3.261616	3.394581	35.13178
3.361045	3.221921	3.39141	34.99484
3.312378	3.231828	3.41535	34.96243
3.22189	3.3559	3.461099	35.13592
3.346568	3.450049	3.319503	35.30481
3.42159	3.436233	3.347127	35.49907
3.379563	3.384205	3.305112	35.2015
3.219332	3.333795	3.411326	34.97314
3.254719	3.464	3.331934	35.16164
3.276166	3.420059	3.439956	35.34868
3.345744	3.347408	3.297422	35.03026
3.231829	3.209319	3.454296	34.82222
3.10084	3.180474	3.410627	34.3772
3.282971	3.328336	3.454893	35.19564
3.368716	3.447353	3.392961	35.50799
3.33469	3.473772	3.429196	35.57059
3.21793	3.337299	3.46983	35.10567
3.24379	3.358259	3.475389	35.22022
3.23418	3.380768	3.413372	35.1128
3.348177	3.365874	3.351901	35.1951
3.220776	3.377938	3.431958	35.11795
3.218425	3.354013	3.493105	35.19421
3.149219	3.253664	3.3895	34.59685
3.430581	3.223808	3.341244	35.04132
3.300293	3.194492	3.397258	34.81479
3.312501	3.352598	3.404642	35.20338
3.261153	3.408063	3.43588	35.2807
3.425385	3.462517	3.345336	35.56093
3.371851	3.399436	3.365952	35.35099
3.464896	3.453688	3.296262	35.52071
3.137794	3.354215	3.455421	34.93591
3.216074	3.433605	3.340818	35.03009
3.205351	3.415139	3.461526	35.23023
3.287054	3.272534	3.354851	34.86376
3.261071	3.140105	3.418419	34.65636
3.179574	3.222595	3.471603	34.77483
3.229066	3.41588	3.445941	35.24963
3.39639	3.457328	3.413355	35.63492
3.353044	3.316677	3.3378	35.06732
3.267134	3.31297	3.399612	35.00651
3.269526	3.352396	3.396917	35.09207
3.240243	3.384542	3.388238	35.07935
3.309367	3.410556	3.341841	35.18594
3.268453	3.376859	3.392774	35.13416
3.251997	3.371535	3.410678	35.12569
3.176109	3.217069	3.443775	34.69432
3.321699	3.226774	3.345285	34.81854
3.335639	3.224348	3.453989	35.08143

3.298396	3.339254	3.36631	35.05953
3.237067	3.398021	3.472302	35.28572
3.385296	3.40321	3.31096	35.26839
3.347311	3.351115	3.370794	35.20225
3.32306	3.372411	3.303134	35.04782
3.268742	3.429427	3.41876	35.30658
3.203454	3.501538	3.376404	35.22887
3.09651	3.377062	3.315616	34.58986
3.264288	3.205882	3.372568	34.70696
3.185307	3.211543	3.433476	34.67982
3.259215	3.34195	3.444133	35.14993
3.280208	3.431111	3.428974	35.35767
3.299015	3.382318	3.426331	35.28632
3.423941	3.303131	3.391955	35.31117
3.357787	3.300031	3.379184	35.13179
3.246429	3.370659	3.395144	35.07762
3.228653	3.411635	3.343375	35.01515
3.139485	3.360213	3.357307	34.73817
3.22952	3.335075	3.416237	35.00895
3.302314	3.292685	3.350759	34.93225
3.23748	3.248677	3.461799	34.93706
3.232572	3.269164	3.423808	34.88805
3.298685	3.322742	3.436545	35.17765
3.286106	3.425652	3.401351	35.29822
3.311594	3.428213	3.398981	35.35438
3.397916	3.407928	3.370181	35.43581
3.305366	3.388519	3.340937	35.12702
3.358446	3.397886	3.343597	35.2694
3.256781	3.452003	3.351697	35.18314
3.31444	3.33393	3.345575	35.03763
3.153549	3.262425	3.407353	34.66452
3.248986	3.157291	3.356079	34.53118
3.27629	3.293628	3.391529	34.96656
3.282971	3.458945	3.453614	35.47846
3.232242	3.427135	3.437978	35.26377
3.345785	3.358865	3.404232	35.28898
3.326277	3.296459	3.403278	35.10776
3.351311	3.391753	3.427678	35.42426
3.320791	3.418576	3.404352	35.36516
3.191947	3.287428	3.391342	34.76815
3.119276	3.272601	3.396389	34.58784
3.2428	3.316609	3.294335	34.73104
3.247749	3.287091	3.464101	35.04856
3.305036	3.331975	3.349258	35.02085
3.133257	3.333593	3.502859	34.98463
3.290725	3.411904	3.346104	35.15744
3.390616	3.376725	3.462481	35.55346
3.425921	3.467032	3.256617	35.37796
3.374325	3.350104	3.405358	35.3347
3.243625	3.417161	3.332633	35.03648

3.259669	3.360618	3.348099	34.98174
3.275547	3.39209	3.332326	35.05079
3.283755	3.316138	3.366924	34.9783
3.11874	3.172926	3.396849	34.3697
3.208857	3.265323	3.378502	34.72871
3.275671	3.413589	3.47544	35.41104
3.288291	3.462652	3.421676	35.42836
3.272413	3.359068	3.440638	35.20858
3.256905	3.322001	3.479242	35.17803
3.239748	3.378612	3.42229	35.13977
3.468979	3.46191	3.305231	35.56723
3.281486	3.38306	3.447765	35.29648
3.305201	3.291472	3.350759	34.93591
3.105501	3.255079	3.464595	34.66856
3.296953	3.249822	3.30518	34.72712
3.183904	3.294504	3.418624	34.8257
3.319224	3.322473	3.372721	35.0824
3.180522	3.302929	3.533313	35.08753
3.359395	3.373827	3.340562	35.21223
3.313202	3.400784	3.391665	35.28192
3.481352	3.429561	3.347894	35.61684
3.322895	3.381442	3.364826	35.20212
3.240367	3.39108	3.383447	35.08344
3.214548	3.362639	3.357767	34.90863
3.21859	3.33986	3.346513	34.84304
3.247749	3.240994	3.347059	34.6918
3.202216	3.190853	3.410866	34.62211
3.178955	3.331571	3.446299	34.95646
3.298644	3.4671	3.434022	35.48773
3.32937	3.484083	3.371954	35.45633
3.266267	3.352126	3.425666	35.14722
3.276991	3.332986	3.392331	35.05592
3.276331	3.376859	3.42906	35.23074
3.292086	3.43839	3.39569	35.32678
3.301036	3.301918	3.401692	35.06103
3.337949	3.245779	3.355124	34.91715
3.096345	3.246385	3.42345	34.53954
3.322688	3.27988	3.334816	34.91394
3.19273	3.296122	3.43397	34.8821
3.222879	3.345791	3.412485	35.00966
3.220776	3.336019	3.380122	34.91292
3.316667	3.395123	3.423927	35.34766
3.303634	3.455238	3.307209	35.19538
3.372593	3.455845	3.411445	35.57546
3.292498	3.417633	3.396048	35.28307
3.322482	3.358798	3.378365	35.18131
3.24977	3.297672	3.331815	34.78683
3.200773	3.248879	3.360768	34.63629
3.180316	3.266266	3.322385	34.54564
3.219992	3.337569	3.392143	34.94088

3.206341	3.444792	3.442769	35.25622
3.312625	3.456451	3.396593	35.41317
3.239129	3.430572	3.422392	35.25227
3.394081	3.376051	3.373795	35.36562
3.343723	3.367357	3.465396	35.4368
3.328256	3.387979	3.385544	35.27345
3.269361	3.310544	3.414856	35.03941
3.243295	3.221854	3.32733	34.59706
3.309202	3.174139	3.38568	34.76444
3.254307	3.256629	3.342762	34.73093
3.194793	3.260807	3.415265	34.76847
3.241398	3.428213	3.388937	35.17891
3.297324	3.418509	3.418266	35.34412
3.293034	3.423765	3.34196	35.17937
3.380429	3.39728	3.413901	35.46989
3.298437	3.454295	3.403244	35.39197
3.360137	3.47175	3.40824	35.57599
3.381749	3.358865	3.348286	35.24528
3.35028	3.34168	3.316178	35.06867
3.216775	3.211677	3.419238	34.7178
3.336299	3.234928	3.340579	34.85801
3.173387	3.249957	3.448174	34.7699
3.240614	3.411837	3.34208	35.03891
3.212074	3.427944	3.496993	35.35049
3.300376	3.428079	3.384657	35.29823
3.171284	3.374838	3.445224	35.03195
3.309037	3.417161	3.366361	35.25328
3.273155	3.439401	3.473445	35.45763
3.390451	3.415004	3.357648	35.40755
3.203289	3.234389	3.420329	34.74036
3.244079	3.187146	3.330058	34.52884
3.232613	3.244296	3.326051	34.61998
3.278146	3.268019	3.34121	34.80458
3.203577	3.374501	3.403141	35.0098
3.196814	3.385284	3.470444	35.16577
3.295179	3.368031	3.39158	35.17069
3.40633	3.423833	3.362064	35.47124
3.392967	3.433807	3.395621	35.53722
3.228942	3.449779	3.416032	35.25808
3.272495	3.415948	3.349787	35.13447
3.3264	3.339052	3.366497	35.12074
3.417136	3.292146	3.331013	35.13899
3.193555	3.215249	3.390642	34.6123
3.223952	3.264245	3.404011	34.81515
3.193638	3.313644	3.403585	34.85595
3.308831	3.446208	3.415606	35.42404
3.269897	3.376522	3.401214	35.15504
3.238593	3.355967	3.44531	35.13806
3.156271	3.356507	3.420738	34.90548
3.379646	3.400447	3.403397	35.45214

3.363891	3.472559	3.393507	35.55375
3.331721	3.318227	3.428565	35.22257
3.232324	3.236815	3.370163	34.69945
3.203124	3.248137	3.394206	34.71293
3.279919	3.231693	3.327296	34.69859
3.339186	3.289045	3.341244	34.98412
3.177965	3.35772	3.43733	34.99186
3.123854	3.361022	3.406739	34.81385
3.22024	3.378949	3.422427	35.09814
3.304913	3.452206	3.405852	35.40726
3.333123	3.444118	3.390387	35.41745
3.254348	3.370929	3.432044	35.17622
3.220776	3.285743	3.397037	34.83996
3.378615	3.305085	3.303969	35.02391
3.32042	3.310005	3.395707	35.10802
3.303881	3.302726	3.325505	34.90241
3.158375	3.328673	3.387778	34.77714
3.228983	3.381375	3.41119	35.09799
3.334567	3.405771	3.366975	35.28555
3.310357	3.358798	3.400208	35.20256
3.311347	3.350037	3.449351	35.29303
3.168108	3.39391	3.38418	34.93322
3.266804	3.441288	3.428838	35.35031
3.379316	3.367829	3.362422	35.29048
3.405588	3.233109	3.405307	35.1471
3.205103	3.207971	3.420858	34.68771
3.262349	3.237422	3.388596	34.80675
3.233438	3.26411	3.372789	34.76732
3.283177	3.353339	3.371562	35.06854
3.192483	3.406243	3.430219	35.11417
3.148023	3.353069	3.420943	34.88038
3.245192	3.40476	3.371545	35.09788
3.371727	3.47795	3.377342	35.54733
3.321534	3.425585	3.44855	35.47877
3.368221	3.377331	3.353385	35.26723
3.224859	3.244835	3.423467	34.81723
3.292333	3.233243	3.304924	34.68021
3.357787	3.245846	3.342438	34.93294
3.34562	3.334806	3.326392	35.06578
3.183368	3.339321	3.397668	34.87671
3.225601	3.405637	3.378178	35.07146
3.282476	3.409074	3.422103	35.29941
3.347517	3.430505	3.338942	35.30665
3.25175	3.446342	3.490309	35.46288
3.144764	3.428281	3.46098	35.12528
3.275712	3.390608	3.395536	35.18614
3.323885	3.264582	3.400191	35.02607
3.295344	3.20231	3.371886	34.76558
3.214796	3.208847	3.420397	34.70981
3.238923	3.231019	3.394752	34.75498

3.208403	3.313105	3.386772	34.85029
3.346073	3.440749	3.378297	35.41196
3.259297	3.370524	3.40708	35.13157
3.290519	3.365537	3.371766	35.11172
3.176481	3.430909	3.464254	35.20755
3.356673	3.485161	3.37134	35.51705
3.348136	3.411904	3.408683	35.41984
3.342155	3.331638	3.346803	35.09591
3.253977	3.239309	3.452437	34.93218
3.355518	3.275567	3.375245	35.06471
3.266102	3.321529	3.371391	34.96126
3.411197	3.348217	3.30963	35.20186
3.17479	3.336625	3.404215	34.86637
3.168768	3.35563	3.386686	34.85643
3.216775	3.373759	3.39262	35.01404
3.32673	3.416757	3.389534	35.34177
3.274186	3.431785	3.422103	35.33095
3.241604	3.375983	3.496976	35.3014
3.20333	3.306568	3.482738	35.03477
3.338691	3.258584	3.37579	34.99197
3.419652	3.21808	3.35659	35.03846
3.246718	3.292011	3.427934	34.97797
3.22721	3.308118	3.416851	34.94629
3.208279	3.303737	3.402101	34.86306
3.336629	3.393371	3.358858	35.24519
3.328751	3.398695	3.370675	35.26545
3.201433	3.436233	3.428292	35.19511
3.2136	3.479028	3.41813	35.29308
3.321451	3.394854	3.392637	35.28911
3.304871	3.34451	3.386465	35.12926
3.318193	3.25427	3.330536	34.83875
3.207166	3.205949	3.465141	34.78464
3.295344	3.369311	3.444883	35.29042
3.262143	3.406108	3.400737	35.20174
3.377955	3.376792	3.368066	35.31944
3.261566	3.34323	3.39025	35.04004
3.190751	3.345993	3.417482	34.95077
3.136103	3.407928	3.431157	34.99661
3.315058	3.492642	3.385459	35.47328
3.238263	3.388316	3.387727	35.08216
3.249275	3.323686	3.44398	35.08792
3.204815	3.297537	3.414651	34.86937
3.385007	3.243824	3.455524	35.23534
3.395689	3.211408	3.359677	34.97821
3.390946	3.254877	3.416322	35.18677
3.230839	3.283317	3.37782	34.81464
3.251214	3.410017	3.381844	35.14507
3.219662	3.406715	3.373045	35.04961
3.256946	3.401593	3.357204	35.0853
3.218549	3.438457	3.418334	35.21563

3.204856	3.44021	3.431225	35.21771
3.250389	3.389664	3.452454	35.25317
3.448893	3.303063	3.330143	35.23041
3.320296	3.209656	3.348337	34.78471
3.213022	3.318227	3.397139	34.89427
3.332422	3.356978	3.426741	35.30485
3.286477	3.405906	3.350418	35.14447
3.377336	3.32928	3.342301	35.15785
3.287054	3.336895	3.379457	35.05832
3.230303	3.418172	3.411599	35.18224
3.229396	3.461236	3.382509	35.21082
3.299015	3.42882	3.437926	35.41337
3.239006	3.328606	3.410985	35.00407
3.241686	3.280284	3.367572	34.80932
3.180935	3.176835	3.434721	34.59708
3.354735	3.234659	3.400481	35.02873
3.24346	3.243959	3.455984	34.9271
3.307965	3.318699	3.369567	35.04263
3.284909	3.367761	3.413509	35.1956
3.28062	3.439333	3.340033	35.18205
3.147033	3.370659	3.421608	34.91813
3.301448	3.434211	3.321004	35.17479
3.176522	3.425316	3.472695	35.21386
3.29522	3.443107	3.323476	35.18603
3.21063	3.245981	3.438472	34.82144
3.285404	3.233513	3.337237	34.7363
3.406371	3.239915	3.389841	35.12988
3.337289	3.374501	3.404556	35.3053
3.351353	3.438053	3.431106	35.5331
3.28858	3.334941	3.404778	35.11276
3.26437	3.287563	3.34474	34.82491
3.313244	3.367424	3.377786	35.1787
3.367149	3.525597	3.385203	35.6587
3.150703	3.472491	3.490172	35.29879
3.219869	3.34788	3.433698	35.05404
3.219456	3.231693	3.435079	34.80207
3.326318	3.234861	3.367998	34.89599
3.270639	3.249418	3.397958	34.87158
3.279094	3.25272	3.435948	34.98037
3.221477	3.281228	3.334577	34.69503
3.412352	3.361696	3.345746	35.31284
3.279713	3.407119	3.412076	35.26717
3.342444	3.404626	3.36972	35.30627
3.199453	3.316812	3.388937	34.84356
3.277527	3.381847	3.37516	35.12639
3.272124	3.455238	3.472285	35.48747
3.308171	3.418441	3.36943	35.2609
3.248038	3.21006	3.396849	34.73366
3.305366	3.197794	3.394343	34.82673
3.245646	3.307377	3.342131	34.82159

3.459947	3.40011	3.396235	35.61134
3.377336	3.347813	3.355584	35.22742
3.338773	3.243892	3.435266	35.09009
3.207991	3.375107	3.332838	34.86704
3.277403	3.488126	3.36336	35.33273
3.190503	3.505379	3.365611	35.18535
3.232324	3.384408	3.401419	35.09056
3.118245	3.29073	3.457962	34.75989
3.245893	3.265727	3.366361	34.78404
3.394411	3.291472	3.423722	35.29056
3.269361	3.304479	3.391	34.97398
3.289611	3.230683	3.446043	34.97726
3.21661	3.303198	3.359353	34.78662
3.351146	3.380836	3.396031	35.33082
3.418332	3.418104	3.328489	35.41154
3.24779	3.374501	3.379337	35.05443
3.104222	3.358865	3.372551	34.69144
3.278063	3.472356	3.475372	35.54464
3.277527	3.446275	3.392637	35.30551
3.358323	3.336221	3.35081	35.15005
3.181842	3.189572	3.438779	34.6358
3.186461	3.301244	3.455745	34.92721
3.304706	3.395528	3.365474	35.19457
3.510923	3.418037	3.35671	35.67559
3.264247	3.298683	3.433067	35.04212
3.29885	3.280554	3.370931	34.94226
3.230839	3.337502	3.470256	35.13528
3.305284	3.499314	3.316025	35.31465
3.236943	3.394045	3.413423	35.14799
3.243996	3.316812	3.385339	34.9331
3.143238	3.195705	3.408649	34.4989
3.265319	3.311757	3.354783	34.90186
3.317244	3.346937	3.513533	35.4395
3.399772	3.354215	3.425854	35.44416
3.223498	3.263773	3.430492	34.87103
3.286518	3.354889	3.376473	35.08997
3.353456	3.37504	3.412127	35.35839
3.353332	3.381173	3.339488	35.21268
3.187328	3.368435	3.376046	34.90175
3.160726	3.393843	3.349872	34.8419
3.185884	3.414533	3.400294	35.05243
3.27299	3.346263	3.377768	35.04436
3.276083	3.258988	3.398759	34.90617
3.215579	3.248542	3.440365	34.842
3.308047	3.38097	3.40633	35.25938
3.256287	3.464673	3.476821	35.48339
3.41429	3.42417	3.374154	35.51582
3.370201	3.249957	3.357102	35.00114
3.293282	3.373085	3.403158	35.20291
3.257029	3.467908	3.396662	35.31679

3.362323	3.479298	3.439086	35.66473
3.114162	3.322877	3.381486	34.65401
3.230839	3.221045	3.373795	34.66966
3.117915	3.239107	3.499858	34.73789
3.211043	3.34195	3.391188	34.9288
3.302603	3.285945	3.461918	35.16123
3.310934	3.272669	3.387846	34.98844
3.2972	3.315868	3.446299	35.1807
3.423652	3.440344	3.418147	35.66787
3.317492	3.415611	3.457007	35.46661
3.33931	3.376455	3.341943	35.17707
3.157137	3.319373	3.38389	34.74559
3.263752	3.453082	3.444457	35.40359
3.18741	3.396336	3.364161	34.93695
3.291261	3.220573	3.36793	34.78794
3.224158	3.220776	3.419698	34.75484
3.291014	3.327527	3.437654	35.17376
3.339021	3.454025	3.446401	35.5745
3.342444	3.425518	3.307635	35.21619
3.295633	3.28298	3.446248	35.10524
3.353085	3.351654	3.319094	35.10299
3.281321	3.410017	3.407319	35.26662
3.222261	3.514208	3.402868	35.35558
3.260782	3.371333	3.368629	35.0525
3.169139	3.247261	3.42287	34.69938
3.24148	3.229806	3.408802	34.78865
3.262597	3.330762	3.453614	35.15359
3.259998	3.330762	3.411514	35.05585
3.171614	3.256494	3.462753	34.8122
3.349249	3.256966	3.419221	35.1065
3.333865	3.374298	3.397685	35.28235
3.354858	3.410893	3.387437	35.38587
3.290642	3.358124	3.433885	35.23162
3.268	3.377062	3.390438	35.1285
3.274557	3.350845	3.36706	35.03439
3.379769	3.350374	3.349326	35.22466
3.192071	3.275162	3.366156	34.68652
3.20432	3.245105	3.421284	34.76813
3.142455	3.321529	3.432197	34.82384
3.308171	3.445938	3.422444	35.43696
3.402989	3.422552	3.318344	35.36553
3.379563	3.274556	3.482175	35.34893
3.232572	3.298278	3.465737	35.04341
3.344877	3.422013	3.382662	35.37792
3.307098	3.526945	3.380906	35.52093
3.305944	3.415072	3.304941	35.10764
3.192565	3.185057	3.4648	34.70627
3.174501	3.200153	3.362252	34.47553
3.290931	3.335008	3.391273	35.08851
3.318564	3.463326	3.383839	35.41329

3.251131	3.342017	3.505314	35.26619
3.221353	3.250968	3.382202	34.73274
3.280455	3.266671	3.447765	35.0397
3.453801	3.422957	3.371698	35.59421
3.34562	3.384475	3.372022	35.27419
3.194091	3.340669	3.420875	34.95385
3.243707	3.311488	3.421676	35.00029
3.251874	3.343971	3.339778	34.91009
3.3914	3.326786	3.352515	35.20548
3.232902	3.243689	3.330365	34.62872
3.150703	3.281228	3.418078	34.72287
3.047265	3.41588	3.40367	34.75962
3.401628	3.48907	3.38771	35.65971
3.293983	3.420059	3.402391	35.30549
3.408434	3.30677	3.351577	35.19691
3.160602	3.339928	3.526373	35.1097
3.309367	3.555183	3.361859	35.54599
3.376552	3.447016	3.423671	35.59155
3.333577	3.208308	3.350759	34.8161
3.107068	3.163558	3.457007	34.45525
3.188194	3.272264	3.398094	34.74155
3.164767	3.371064	3.398009	34.90619
3.319513	3.364122	3.41344	35.26316
3.2063	3.307781	3.426996	34.92202
3.24016	3.281295	3.479924	35.05389
3.296293	3.397954	3.397003	35.25042
3.430375	3.422418	3.433322	35.67656
3.306439	3.407389	3.388681	35.27504
3.219291	3.326314	3.423262	34.98279
3.275259	3.327056	3.445361	35.15513
3.23385	3.35462	3.343529	34.90216
3.403361	3.306433	3.319742	35.11546
3.209517	3.244296	3.40089	34.73313
3.211744	3.381375	3.401777	35.03971
3.302685	3.394921	3.447663	35.36855
3.394287	3.402132	3.316639	35.29811
3.409753	3.31607	3.41078	35.3496
3.284208	3.327392	3.408188	35.09415
3.210548	3.444051	3.473786	35.33163
3.338237	3.522565	3.38684	35.59243
3.324297	3.358528	3.344961	35.11164
3.335185	3.15446	3.392552	34.79326
3.113172	3.198064	3.356266	34.32375
3.175408	3.279004	3.424694	34.7865
3.229932	3.438457	3.390591	35.17985
3.273361	3.3528	3.442223	35.20042
3.177388	3.27341	3.415265	34.75797
3.348631	3.245037	3.422427	35.08607
3.240449	3.352261	3.443042	35.12906
3.373253	3.490418	3.386073	35.59702

3.356096	3.4733	3.403823	35.56088
3.346898	3.351385	3.382458	35.22744
3.262556	3.293628	3.355721	34.85822
3.245481	3.278465	3.328063	34.72724
3.202546	3.304883	3.35156	34.74251
3.2534	3.433268	3.3275	35.08186
3.222921	3.387373	3.47781	35.24354
3.30219	3.388923	3.354033	35.1496
3.343104	3.321327	3.377325	35.14219
3.364551	3.342489	3.379133	35.23932
3.280167	3.396269	3.43438	35.29321
3.313821	3.4764	3.454006	35.58496
3.254307	3.360348	3.455711	35.20475
3.257689	3.174341	3.35642	34.58825
3.329906	3.207701	3.334833	34.77192
3.226509	3.279678	3.403891	34.85423
3.237315	3.459417	3.442275	35.35486
3.142249	3.410152	3.443536	35.04199
3.306892	3.275769	3.324789	34.84848
3.2826	3.275432	3.413986	34.98968
3.33733	3.386429	3.367265	35.24993
3.332257	3.390945	3.394155	35.30751
3.286064	3.396336	3.379525	35.18629
3.298396	3.365133	3.423518	35.24123
3.478919	3.30832	3.263796	35.16248
3.286188	3.285204	3.490087	35.18532
3.210795	3.249957	3.271299	34.46491
3.187163	3.273275	3.417618	34.7842
3.253276	3.400312	3.397139	35.1618
3.279754	3.425922	3.400737	35.28358
3.236655	3.445197	3.405341	35.24155
3.229932	3.317823	3.429588	35.00132
3.404433	3.343769	3.42981	35.44016
3.340877	3.469189	3.373182	35.4516
3.390534	3.484218	3.452096	35.76564
3.283301	3.283182	3.358517	34.88686
3.181059	3.130401	3.431174	34.48805
3.291014	3.249957	3.370675	34.85766
3.29555	3.386497	3.367469	35.15916
3.252368	3.513803	3.434414	35.48952
3.147239	3.309398	3.411514	34.76254
3.284621	3.221854	3.422972	34.89658
3.280496	3.277117	3.380224	34.91493
3.312089	3.387238	3.431583	35.33715
3.159942	3.45389	3.476719	35.24889
3.219786	3.472559	3.426809	35.31144
3.328298	3.323686	3.39187	35.14677
3.440191	3.200692	3.338192	35.00511
3.307593	3.211408	3.401112	34.87617
3.169675	3.329886	3.35347	34.72948

3.16419	3.44331	3.439904	35.15454
3.350816	3.404019	3.343512	35.26594
3.285569	3.382925	3.414327	35.23199
3.356302	3.369716	3.339078	35.19323
3.223746	3.358528	3.471211	35.16784
3.337165	3.470537	3.364178	35.42675
3.370696	3.513399	3.458218	35.79946
3.483661	3.313375	3.405597	35.494
3.243212	3.139364	3.413014	34.60386
3.238511	3.225224	3.390455	34.73201
3.283177	3.382925	3.378229	35.14782
3.343516	3.515286	3.444048	35.71316
3.223374	3.409411	3.402868	35.12884
3.149714	3.260942	3.434363	34.71195
3.232737	3.291539	3.454688	35.00487
3.317368	3.423496	3.35272	35.25552
3.298561	3.410961	3.461423	35.4247
3.205846	3.392764	3.445156	35.14658
3.208114	3.300772	3.458115	34.97871
3.267876	3.188427	3.314661	34.55001
3.447738	3.230548	3.32941	35.0677
3.272	3.330358	3.430986	35.12379
3.203536	3.397886	3.366855	34.9815
3.268412	3.408871	3.422785	35.26971
3.352837	3.406108	3.316775	35.21646
3.31213	3.40166	3.384777	35.26642
3.279754	3.423226	3.363087	35.19535
3.162293	3.392764	3.49522	35.16082
3.323761	3.444725	3.487649	35.611
3.259751	3.388586	3.369038	35.08887
3.378037	3.17542	3.411377	34.97397
3.274722	3.111867	3.410235	34.60656
3.253235	3.260066	3.466266	35.00619
3.290147	3.42909	3.319179	35.13488
3.340382	3.379825	3.37487	35.25879
3.155653	3.296863	3.45682	34.8526
3.201144	3.294235	3.357784	34.72976
3.196442	3.354754	3.44427	35.04096
3.351806	3.389058	3.363394	35.27887
3.331721	3.397482	3.455251	35.45424
3.227622	3.440344	3.38007	35.15592
3.247378	3.269636	3.441183	34.95946
3.377212	3.214778	3.314507	34.8464
3.321905	3.253933	3.343239	34.87391
3.355889	3.341208	3.343307	35.13923
3.132556	3.387036	3.371869	34.81351
3.255338	3.345454	3.376933	35.00216
3.332174	3.42235	3.4161	35.424
3.268082	3.369514	3.328507	34.97674
3.30483	3.408265	3.439291	35.38412

3.209846	3.458338	3.387113	35.1718
3.321657	3.450993	3.464783	35.5701
3.267629	3.263234	3.366446	34.8263
3.402783	3.213834	3.399577	35.08629
3.200443	3.201838	3.399049	34.61641
3.362488	3.367155	3.41049	35.35732
3.27101	3.427809	3.47203	35.42449
3.354693	3.452273	3.369055	35.4358
3.196607	3.283991	3.391955	34.77217
3.243047	3.247059	3.329717	34.65686
3.124555	3.3559	3.499431	35.00689
3.229231	3.433268	3.395911	35.17861
3.26569	3.423968	3.411479	35.27204
3.415651	3.345387	3.324346	35.23759
3.278929	3.194357	3.43102	34.84161
3.342692	3.192875	3.324056	34.74389
3.264082	3.32874	3.425121	35.09011
3.288085	3.438996	3.379064	35.283
3.280909	3.41797	3.400805	35.26886
3.228695	3.36702	3.334594	34.89847
3.311635	3.351182	3.371681	35.12632
3.265113	3.432594	3.404096	35.2735
3.229396	3.413589	3.391546	35.12639
3.297489	3.410691	3.381162	35.24625
3.311635	3.325842	3.423211	35.18359
3.18077	3.145295	3.375859	34.39903
3.422168	3.267479	3.381707	35.20691
3.273073	3.377399	3.399936	35.1611
3.322853	3.414802	3.420329	35.39636
3.276331	3.327527	3.40292	35.06569
3.296334	3.328606	3.389892	35.08331
3.2793	3.278936	3.398759	34.95683
3.310274	3.403143	3.394002	35.28578
3.108883	3.381914	3.484972	34.99788
3.243666	3.386092	3.378178	35.06823
3.235541	3.326584	3.476974	35.13638
3.401298	3.237826	3.360887	35.0509
3.314935	3.25582	3.423739	35.03883
3.369252	3.253799	3.394274	35.08876
3.224364	3.301446	3.388886	34.86433
3.304583	3.417093	3.359131	35.22758
3.235376	3.449847	3.355516	35.13996
3.333164	3.357113	3.334918	35.10597
3.157962	3.373827	3.376779	34.85093
3.235541	3.451127	3.469762	35.39296
3.287755	3.477411	3.4124	35.43918
3.323348	3.361965	3.447919	35.34223
3.29951	3.305894	3.418147	35.10237
3.194174	3.131479	3.332326	34.30293
3.330731	3.255146	3.389091	34.99613

3.328834	3.433335	3.346939	35.28947
3.295921	3.405839	3.395843	35.26432
3.227499	3.342826	3.402578	34.99162
3.273155	3.3003	3.365679	34.91777
3.261648	3.260066	3.358671	34.78929
3.292416	3.355226	3.38273	35.11729
3.188482	3.419048	3.474331	35.22989
3.194999	3.460495	3.467238	35.31927
3.23319	3.323821	3.403005	34.96343
3.463122	3.202175	3.347945	35.07983
3.194215	3.168006	3.389449	34.50782
3.33135	3.296054	3.361723	35.02709
3.232365	3.389058	3.417737	35.13651
3.356838	3.438862	3.341397	35.35068
3.337577	3.427	3.328984	35.25548
3.34265	3.313981	3.349599	35.0645
3.228983	3.39054	3.387846	35.06699
3.241398	3.469121	3.414157	35.32352
3.249234	3.413791	3.45462	35.30814
3.329329	3.295717	3.404846	35.11624
3.284126	3.166052	3.410576	34.74636
3.320833	3.217743	3.450681	35.02738
3.345702	3.385553	3.436068	35.41678
3.361828	3.509221	3.39187	35.62583
3.291261	3.366953	3.366992	35.10599
3.27266	3.302052	3.402459	35.00095
3.12373	3.26411	3.413833	34.61716
3.250801	3.346128	3.379457	34.99923
3.318977	3.459956	3.421011	35.48812
3.267917	3.421946	3.408035	35.26496
3.292663	3.338243	3.433084	35.19081
3.204567	3.230278	3.368339	34.62047
3.320049	3.137881	3.377751	34.69153
3.303015	3.301379	3.383208	35.02376
3.280991	3.448836	3.402442	35.34012
3.248368	3.448229	3.347417	35.14712
3.431159	3.420194	3.378996	35.55461
3.301943	3.345589	3.302725	34.94209
3.362076	3.343971	3.440791	35.37198
3.185389	3.47458	3.418812	35.22315
3.21096	3.404693	3.479328	35.25858
3.23385	3.312633	3.36527	34.85789
3.261607	3.178587	3.352839	34.59827
3.241563	3.181418	3.405869	34.67659
3.351105	3.408467	3.394598	35.38802
3.294519	3.483342	3.511981	35.68471
3.350775	3.431314	3.31212	35.25689
3.342444	3.287091	3.454108	35.23379
3.36653	3.285271	3.313996	34.97608
3.25109	3.29282	3.433834	35.0022

3.273196	3.427337	3.356829	35.17632
3.183492	3.459686	3.475167	35.30967
3.339681	3.402941	3.379388	35.31769
3.274434	3.242139	3.427491	34.92855
3.225065	3.171713	3.350759	34.49878
3.235252	3.201299	3.472098	34.8511
3.291302	3.381847	3.394274	35.19832
3.340258	3.478961	3.367401	35.45898
3.348177	3.397819	3.345064	35.25
3.29188	3.324427	3.35879	34.99641
3.226715	3.393438	3.379764	35.05069
3.336587	3.415543	3.32334	35.21591
3.283961	3.468245	3.440842	35.47304
3.261442	3.377331	3.353197	35.03331
3.149425	3.179126	3.464783	34.59893
3.273526	3.189033	3.379661	34.70583
3.350404	3.338378	3.416629	35.28139
3.345249	3.46838	3.363428	35.43807
3.277114	3.437918	3.508724	35.54019
3.350487	3.36547	3.440297	35.39257
3.261071	3.351385	3.371749	35.01633
3.40897	3.322608	3.345371	35.21914
3.164602	3.354889	3.364383	34.79692
3.180275	3.377803	3.455507	35.08058
3.134536	3.35745	3.360274	34.72779
3.306067	3.300435	3.354578	34.96576
3.343186	3.247868	3.384112	34.99656
3.259669	3.211677	3.439529	34.85597
3.167118	3.309196	3.435335	34.85766
3.344053	3.402132	3.4412	35.46065
3.432066	3.432459	3.352396	35.52524
3.332257	3.377466	3.406279	35.30455
3.221147	3.362235	3.395962	35.0057
3.239954	3.434211	3.438489	35.29723
3.354817	3.519869	3.304703	35.44317
3.33469	3.421811	3.365884	35.31851
3.158787	3.238365	3.39141	34.58849
3.197597	3.15446	3.369857	34.44274
3.226303	3.225358	3.435198	34.80345
3.414579	3.395191	3.308676	35.30989
3.346445	3.414263	3.381707	35.36231
3.26767	3.369244	3.393507	35.1174
3.271794	3.305624	3.461526	35.13604
3.273898	3.361561	3.343103	35.00399
3.426374	3.330021	3.412161	35.41948
3.249151	3.351654	3.416902	35.0896
3.148806	3.338647	3.442189	34.89701
3.122452	3.376859	3.440859	34.92003
3.350816	3.374905	3.35272	35.22241
3.264123	3.255349	3.368748	34.80643

3.271918	3.229402	3.412554	34.86253
3.186668	3.348285	3.44236	35.00126
3.387647	3.426326	3.400259	35.51937
3.507294	3.417228	3.318821	35.58303
3.392637	3.365605	3.396986	35.39033
3.263257	3.382386	3.393456	35.13637
3.25109	3.421676	3.347809	35.09587
3.228241	3.449982	3.432265	35.29249
3.31279	3.366009	3.335668	35.08251
3.186874	3.212149	3.436971	34.69222
3.146043	3.189775	3.429793	34.5383
3.272743	3.311083	3.442547	35.10855
3.326772	3.446679	3.35081	35.32261
3.317451	3.421676	3.401641	35.35871
3.282064	3.346667	3.386226	35.08358
3.251007	3.344847	3.435863	35.12023
3.272536	3.306163	3.332326	34.8563
3.368757	3.300165	3.360308	35.11479
3.224282	3.336895	3.362763	34.88454
3.276042	3.391821	3.430407	35.26577
3.134124	3.292078	3.386703	34.64173
3.364014	3.248609	3.367674	35.00779
3.273939	3.222056	3.392007	34.80595
3.243914	3.319103	3.401419	34.9731
3.214878	3.421541	3.493924	35.33591
3.388183	3.480039	3.363394	35.55738
3.452193	3.412511	3.341943	35.50278
3.439943	3.389799	3.335464	35.41215
3.216074	3.351991	3.431498	35.04992
3.272949	3.395662	3.305589	34.99445
3.205846	3.317957	3.41078	34.90782
3.285941	3.265255	3.352805	34.84094
3.133464	3.225021	3.439273	34.6086
3.202299	3.302592	3.444235	34.93962
3.177759	3.399032	3.493924	35.20551
3.431942	3.454093	3.438728	35.76108
3.314935	3.380499	3.422222	35.30817
3.310233	3.357922	3.413423	35.22927
3.326442	3.359068	3.437313	35.31946
3.283631	3.35718	3.387573	35.11294
3.322565	3.296796	3.366173	35.01924
3.303634	3.336625	3.323118	34.97079
3.258225	3.315666	3.421199	35.04013
3.088838	3.216665	3.441882	34.49844
3.266927	3.23695	3.330024	34.68764
3.215951	3.267345	3.423603	34.84727
3.233768	3.391753	3.410729	35.13015
3.200897	3.422552	3.493992	35.3077
3.360055	3.468178	3.377973	35.50181
3.414496	3.392023	3.373182	35.44385

3.425715	3.406715	3.379064	35.51338
3.311635	3.420733	3.424422	35.39375
3.259421	3.457395	3.398776	35.30365
3.193968	3.296122	3.380906	34.76876
3.305242	3.19557	3.323715	34.66714
3.118946	3.183035	3.427389	34.45905
3.280373	3.367357	3.394002	35.14213
3.2501	3.416689	3.47856	35.36873
3.369129	3.413185	3.364144	35.37115
3.366571	3.322877	3.429997	35.31208
3.453224	3.355159	3.353419	35.40471
3.307552	3.425922	3.437432	35.42462
3.268948	3.414735	3.366838	35.16135
3.272083	3.298615	3.414378	35.01824
3.309697	3.267547	3.376404	34.94951
3.273238	3.272399	3.338192	34.79682
3.159694	3.246992	3.486881	34.81812
3.258266	3.266334	3.389977	34.86407
3.263463	3.351452	3.506951	35.31737
3.251626	3.428618	3.365849	35.15167
3.299633	3.387912	3.385476	35.21056
3.334979	3.404423	3.367197	35.28399
3.317987	3.406176	3.426058	35.37938
3.420435	3.443242	3.364468	35.54979
3.288333	3.386025	3.376336	35.16173
3.313367	3.313375	3.346939	34.99332
3.199247	3.215519	3.374546	34.59013
3.288415	3.197053	3.386891	34.77174
3.149177	3.328066	3.397344	34.77662
3.269402	3.443107	3.418488	35.33734
3.219497	3.446208	3.464731	35.33612
3.392307	3.394719	3.396457	35.45212
3.244285	3.324966	3.401913	34.98781
3.399401	3.378342	3.426536	35.4976
3.349827	3.398897	3.356539	35.28107
3.245893	3.303063	3.420858	34.98486
3.16254	3.218552	3.329495	34.41797
3.322565	3.301783	3.413901	35.13452
3.276413	3.25117	3.371289	34.82972
3.238139	3.27368	3.387317	34.8303
3.139155	3.237759	3.456598	34.68679
3.269278	3.357046	3.486404	35.29739
3.316626	3.477276	3.450476	35.58529
3.322441	3.461708	3.361928	35.37032
3.3549	3.41952	3.404932	35.44308
3.362736	3.417296	3.384163	35.40994
3.266309	3.402604	3.40737	35.21769
3.334649	3.360078	3.32571	35.09557
3.398576	3.327392	3.341824	35.19912
3.160891	3.173196	3.353641	34.36798

3.284291	3.193953	3.407899	34.80188
3.162664	3.3652	3.430748	34.96036
3.307511	3.459888	3.37088	35.35327
3.256699	3.412241	3.375825	35.14877
3.281198	3.348352	3.475235	35.28002
3.231087	3.377533	3.388835	35.04531
3.477929	3.420733	3.420193	35.74816
3.273526	3.396201	3.377479	35.15411
3.361787	3.308724	3.386703	35.17599
3.178831	3.152775	3.406688	34.47856
3.332958	3.286889	3.358381	35.00326
3.242223	3.25946	3.303304	34.62441
3.23484	3.309398	3.419221	34.97096
3.150786	3.338917	3.475099	34.9739
3.282105	3.382723	3.397685	35.18758
3.25542	3.410152	3.445821	35.29447
3.483661	3.459619	3.288265	35.55722
3.388472	3.392495	3.432794	35.51833
3.312089	3.404828	3.384981	35.27371
3.269237	3.354687	3.398691	35.10033
3.289611	3.309533	3.331047	34.89821
3.33634	3.260134	3.361723	34.95945
3.197473	3.229132	3.339982	34.54044
3.169552	3.31944	3.426314	34.86566
3.219415	3.416959	3.426741	35.18889
3.330071	3.404087	3.394053	35.33125
3.285569	3.359	3.385783	35.11725
3.301324	3.357517	3.439836	35.26667
3.212156	3.3683	3.374205	34.95172
3.386492	3.442231	3.375364	35.49718
3.352054	3.397482	3.385936	35.34713
3.278929	3.245442	3.367265	34.8139
3.170913	3.196042	3.406603	34.55568
3.232365	3.201771	3.33074	34.53669
3.228942	3.290326	3.392842	34.85867
3.303552	3.361292	3.344109	35.07045
3.145424	3.386766	3.501631	35.12483
3.211249	3.438188	3.3895	35.13602
3.259875	3.39209	3.457843	35.291
3.387647	3.429966	3.321311	35.35468
3.31877	3.410691	3.444457	35.4312
3.289529	3.380903	3.354374	35.10512
3.267299	3.311353	3.465396	35.1472
3.317863	3.266064	3.312955	34.82537
3.350239	3.179868	3.308335	34.69757
3.22057	3.231289	3.346735	34.61043
3.166912	3.344915	3.419425	34.90053
3.216198	3.451329	3.465192	35.34111
3.320214	3.405232	3.347809	35.21107
3.361993	3.389462	3.420909	35.42781

3.313285	3.361696	3.427815	35.27567
3.211043	3.390877	3.47	35.20815
3.301984	3.424507	3.401555	35.33089
3.32871	3.264919	3.403823	35.0453
3.357085	3.211138	3.348968	34.86978
3.172975	3.164637	3.407234	34.49289
3.230592	3.265525	3.376387	34.77206
3.233355	3.319912	3.406006	34.96181
3.24779	3.379218	3.35983	35.02209
3.204444	3.349767	3.386686	34.92162
3.291137	3.408804	3.435352	35.34674
3.239913	3.460023	3.459957	35.40053
3.439985	3.482465	3.359029	35.66642
3.342733	3.430303	3.381145	35.38804
3.350322	3.313509	3.377513	35.14128
3.284703	3.254607	3.377717	34.86943
3.287879	3.249822	3.377751	34.86598
3.269278	3.240859	3.315104	34.6687
3.317574	3.295111	3.349531	34.96825
3.167819	3.39546	3.411599	34.99594
3.152353	3.407524	3.440245	35.05114
3.310027	3.383801	3.404488	35.26587
3.287962	3.387845	3.383072	35.17963
3.31877	3.409748	3.356539	35.23688
3.204691	3.423361	3.480197	35.2876
3.216569	3.362168	3.436545	35.08429
3.326112	3.260942	3.383208	34.98584
3.302809	3.222326	3.348968	34.77556
3.260741	3.267075	3.330109	34.74018
3.218673	3.277049	3.398895	34.82042
3.194586	3.368705	3.392893	34.95505
3.326813	3.358461	3.419119	35.27916
3.272825	3.33521	3.380087	35.0249
3.322276	3.408063	3.40824	35.35392
3.203	3.45726	3.376541	35.13135
3.318729	3.442299	3.435863	35.48144
3.340258	3.371535	3.37915	35.24975
3.392637	3.305287	3.390864	35.24504
3.223539	3.266132	3.392296	34.79275
3.305284	3.29875	3.391307	35.04068
3.261854	3.350172	3.390591	35.0566
3.314893	3.387238	3.376882	35.22366
3.224983	3.402199	3.377632	35.0614
3.156684	3.41487	3.441286	35.07895
3.152848	3.342152	3.399936	34.82111
3.334154	3.408467	3.377768	35.31415
3.264412	3.392225	3.453904	35.29261
3.343269	3.388047	3.386021	35.30747
3.208527	3.228661	3.471126	34.85037
3.274887	3.200086	3.31923	34.60083

3.335309	3.189033	3.324039	34.71931
3.320956	3.237691	3.327569	34.80205
3.15124	3.327932	3.422733	34.83635
3.256699	3.442299	3.390455	35.24649
3.300953	3.386295	3.447936	35.3465
3.322482	3.437918	3.283968	35.1479
3.292663	3.437177	3.415282	35.36823
3.184193	3.41278	3.417072	35.08159
3.279754	3.367963	3.40355	35.16299
3.304748	3.257033	3.372073	34.90622
3.336917	3.231356	3.323817	34.81489
3.257235	3.256899	3.37961	34.81851
3.240573	3.317014	3.420449	35.00284
3.274681	3.403076	3.378314	35.17349
3.371479	3.399167	3.313996	35.23597
3.23517	3.366548	3.446111	35.15547
3.231252	3.373355	3.432862	35.13281
3.180399	3.392697	3.443843	35.08792
3.315636	3.440816	3.363923	35.31411
3.308047	3.399369	3.460622	35.41834
3.30252	3.366211	3.390915	35.18131
3.226633	3.171713	3.431157	34.67802
3.379563	3.220439	3.351219	34.9442
3.245275	3.219023	3.381247	34.7131
3.332669	3.318968	3.320867	34.99075
3.210218	3.339793	3.444031	35.03784
3.194916	3.373018	3.383157	34.94392
3.221312	3.363313	3.34965	34.90714
3.279754	3.473098	3.369089	35.31754
3.290024	3.458608	3.434243	35.45079
3.29621	3.389732	3.415282	35.27223
3.222632	3.330021	3.424012	34.99984
3.303057	3.277319	3.362558	34.92608
3.414249	3.318833	3.290106	35.10158
3.266804	3.341006	3.364929	34.99126
3.252245	3.353339	3.388664	35.03829
3.285899	3.330156	3.411053	35.11015
3.280661	3.373422	3.409825	35.19063
3.279919	3.377736	3.417925	35.21615
3.288993	3.459888	3.405648	35.3888
3.156725	3.458204	3.462123	35.21937
3.253729	3.398965	3.449914	35.27526
3.275259	3.269097	3.431992	34.99915
3.327803	3.272332	3.405102	35.06232
3.211867	3.247194	3.37059	34.67835
3.249358	3.168074	3.380019	34.60793
3.224818	3.276308	3.417857	34.8737
3.348631	3.328471	3.367214	35.14778
3.214796	3.394045	3.397548	35.06484
3.294643	3.373355	3.386482	35.17001

3.107233	3.413589	3.39835	34.87411
3.233603	3.42262	3.40471	35.18412
3.313945	3.380701	3.40251	35.26334
3.282765	3.339523	3.418624	35.14034
3.234015	3.226639	3.375228	34.69197
3.250677	3.223539	3.394684	34.76418
3.301159	3.358596	3.415435	35.2153
3.4312	3.411163	3.334781	35.43826
3.271959	3.41642	3.395485	35.23427
3.194999	3.330425	3.326614	34.7273
3.259916	3.3652	3.394684	35.09417
3.300128	3.448634	3.361945	35.29297
3.197638	3.478152	3.470853	35.37156
3.282847	3.435425	3.403414	35.31698
3.154663	3.286215	3.525435	34.9772
3.343063	3.267075	3.434039	35.14748
3.381419	3.259257	3.388613	35.11492
3.242924	3.203455	3.345592	34.59595
3.278434	3.228795	3.401078	34.85036
3.235995	3.343095	3.382202	34.96622
3.354611	3.432594	3.357102	35.36645
3.31015	3.431381	3.318003	35.18106
3.309614	3.366144	3.391819	35.19865
3.047677	3.43192	3.441337	34.87797
3.280125	3.456317	3.366855	35.27677
3.267134	3.391147	3.43588	35.25679
3.323307	3.260134	3.353828	34.91369
3.14229	3.13714	3.424899	34.40429
3.309119	3.242342	3.352242	34.84029
3.313615	3.376051	3.413491	35.27646
3.358488	3.434077	3.351304	35.36549
3.268041	3.367963	3.42495	35.18417
3.251379	3.349767	3.323903	34.88697
3.140516	3.418104	3.47012	35.11372
3.312171	3.484689	3.381162	35.44018
3.213352	3.500729	3.430407	35.36684
3.239665	3.328336	3.352174	34.87631
3.153013	3.258516	3.482977	34.82017
3.259751	3.226369	3.429043	34.86535
3.388802	3.240994	3.362627	35.0343
3.295674	3.203253	3.378911	34.78372
3.292911	3.257101	3.400208	34.94201
3.242635	3.369042	3.464186	35.21677
3.338526	3.43839	3.331047	35.28697
3.310274	3.373894	3.379883	35.19094
3.298479	3.37322	3.370658	35.1435
3.067227	3.372748	3.478441	34.87246
3.293818	3.506592	3.413168	35.51793
3.260782	3.338512	3.379951	35.00549
3.299716	3.263503	3.363701	34.89106

3.168851	3.178655	3.41992	34.54227
3.308212	3.346398	3.445259	35.26927
3.275506	3.409343	3.437585	35.31862
3.427653	3.383329	3.327961	35.35472
3.245811	3.302389	3.416356	34.97336
3.347558	3.318092	3.353487	35.09272
3.196442	3.428348	3.480214	35.2805
3.271918	3.532135	3.36261	35.41534
3.200525	3.451195	3.401709	35.16771
3.243377	3.325371	3.413918	35.01297
3.187286	3.283317	3.433067	34.84021
3.297489	3.269569	3.403414	34.9863
3.322936	3.223404	3.358432	34.84262
3.309037	3.259257	3.412196	35.00821
3.255462	3.32126	3.388937	34.97577
3.279053	3.361696	3.340579	35.01004
3.329865	3.383531	3.325642	35.13624
3.252698	3.362639	3.365969	35.00999
3.215497	3.402469	3.460196	35.2218
3.183038	3.463326	3.422631	35.20175
3.247419	3.462719	3.381247	35.25072
3.323266	3.294572	3.390711	35.06957
3.24181	3.236546	3.38904	34.76089
3.196319	3.223404	3.413168	34.68543
3.333412	3.414263	3.421966	35.42185
3.298314	3.379892	3.421267	35.2684
3.386203	3.387036	3.359131	35.34035
3.260988	3.292685	3.384998	34.91676
3.330483	3.385755	3.369106	35.23751
3.230509	3.468852	3.427832	35.32902
3.281156	3.476737	3.407148	35.41179
3.125545	3.37841	3.429111	34.9045
3.309078	3.223	3.330826	34.75106
3.204031	3.288776	3.438728	34.90115
3.335433	3.326584	3.424081	35.23915
3.286064	3.252518	3.47353	35.07736
3.335763	3.281834	3.385612	35.05789
3.319843	3.350306	3.390438	35.18337
3.269979	3.406445	3.38904	35.19403
3.241893	3.361359	3.396849	35.05109
3.222384	3.407254	3.384777	35.0824
3.200278	3.379825	3.381793	34.96754
3.270681	3.4115	3.47174	35.38747
3.244986	3.395393	3.410593	35.16234
3.211909	3.269164	3.386908	34.76217
3.316626	3.236141	3.379661	34.9031
3.221766	3.346128	3.390779	34.9605
3.343599	3.43684	3.43646	35.5252
3.318028	3.390271	3.373386	35.2295
3.289116	3.328269	3.458508	35.21684

3.265938	3.383801	3.342369	35.03361
3.41297	3.446005	3.395229	35.60678
3.188895	3.436166	3.423245	35.15651
3.267381	3.376186	3.345439	35.02683
3.103438	3.257505	3.497198	34.74065
3.254142	3.294841	3.409092	34.95919
3.244243	3.294235	3.446128	35.01721
3.277816	3.317081	3.414446	35.0713
3.187328	3.235535	3.424677	34.71747
3.320585	3.304748	3.412417	35.13343
3.30813	3.337367	3.417601	35.18886
3.413424	3.447421	3.385544	35.58968
3.22391	3.381038	3.359881	34.97396
3.212734	3.357113	3.377888	34.93658
3.152477	3.440142	3.326546	34.8741
3.347929	3.435829	3.329802	35.29921
3.256204	3.338647	3.411326	35.06438
3.240325	3.187011	3.363155	34.59271
3.184069	3.223202	3.398827	34.62684
3.367726	3.392562	3.393661	35.38753
3.412228	3.389664	3.369447	35.42556
3.254183	3.360618	3.423518	35.13467
3.285693	3.294235	3.402237	35.01187
3.231004	3.366076	3.369976	34.97883
3.408062	3.468717	3.372022	35.59496
3.312501	3.449914	3.446316	35.50733
3.104181	3.323281	3.354919	34.57498
3.151033	3.246857	3.399458	34.60771
3.308418	3.295785	3.390438	35.03915
3.287178	3.318025	3.383498	35.02616
3.30714	3.254472	3.418266	35.00687
3.1811	3.243285	3.404352	34.67635
3.322482	3.275836	3.401913	35.05138
3.399195	3.436031	3.311591	35.37194
3.371851	3.40348	3.380821	35.39235
3.208815	3.381981	3.398094	35.02658
3.196566	3.367963	3.372039	34.91216
3.20865	3.425248	3.411377	35.14988
3.367644	3.394314	3.328438	35.24856
3.284951	3.22455	3.409058	34.87277
3.198876	3.216934	3.416169	34.68344
3.137588	3.308185	3.407643	34.73032
3.381873	3.40813	3.412622	35.49398
3.388967	3.383531	3.438608	35.51253
3.424065	3.313712	3.336896	35.21417
3.201268	3.326651	3.486421	35.08223
3.313904	3.446275	3.357409	35.30802
3.414455	3.47795	3.37163	35.62827
3.245687	3.368368	3.344757	34.9608
3.122699	3.25272	3.424149	34.61256

3.18077	3.264312	3.393985	34.69894
3.277486	3.32591	3.350469	34.94998
3.311759	3.3034	3.387062	35.05573
3.314357	3.242813	3.408257	34.97527
3.191369	3.272332	3.418829	34.79398
3.319884	3.444051	3.363411	35.32936
3.422044	3.418509	3.37209	35.51589
3.43219	3.376051	3.36972	35.44004
3.112182	3.307714	3.424473	34.71053
3.257235	3.390743	3.367657	35.08506
3.256328	3.448701	3.353078	35.17794
3.303057	3.27678	3.300491	34.78917
3.173429	3.249418	3.391239	34.64431
3.204526	3.239511	3.463708	34.84913
3.113502	3.362235	3.406671	34.79372
3.46968	3.439468	3.303986	35.51696
3.312501	3.359202	3.445958	35.30818
3.384306	3.316542	3.323186	35.10343
3.179285	3.307309	3.483079	34.98455
3.291385	3.507199	3.367947	35.41505
3.300005	3.410893	3.38568	35.26207
3.272866	3.335682	3.313109	34.87955
3.139444	3.212284	3.424285	34.56105
3.12307	3.29255	3.489814	34.84407
3.220941	3.435492	3.422921	35.22441
3.319554	3.32719	3.443229	35.24763
3.315388	3.266064	3.397412	35.00465
3.262184	3.245981	3.529647	35.13356
3.304913	3.372277	3.364673	35.14242
3.386822	3.389395	3.373795	35.37892
3.352219	3.407658	3.364178	35.32216
3.257111	3.376522	3.420039	35.16825
3.264783	3.367087	3.328848	34.96497
3.269155	3.334199	3.371033	34.99486
3.38443	3.270377	3.281632	34.91187
3.213847	3.279273	3.430697	34.88427
3.268948	3.362976	3.392211	35.10365
3.20201	3.426259	3.480555	35.28885
3.350528	3.448297	3.374136	35.42911
3.413713	3.314925	3.362388	35.24993
3.327638	3.301581	3.398571	35.11164
3.249976	3.396269	3.449862	35.26104
3.355312	3.476063	3.399987	35.55682
3.22688	3.30057	3.352788	34.78897
3.255627	3.174476	3.358261	34.58806
3.170377	3.278465	3.395826	34.71118
3.096056	3.442636	3.472081	35.07443
3.176893	3.431583	3.407626	35.08608
3.256287	3.294033	3.461134	35.07592
3.260163	3.277049	3.45641	35.03693

3.340011	3.302052	3.391819	35.12497
3.228447	3.314049	3.435386	35.0025
3.339021	3.42909	3.391393	35.39968
3.312295	3.419924	3.369652	35.27365
3.283095	3.34013	3.344654	34.98063
3.21129	3.268221	3.363019	34.70651
3.234758	3.269501	3.336248	34.70209
3.290642	3.284126	3.375296	34.94167
3.26503	3.432392	3.362217	35.18129
3.317368	3.434009	3.498408	35.59711
3.255379	3.414802	3.419101	35.24612
3.366448	3.348689	3.361075	35.21753
3.345042	3.302457	3.420994	35.20066
3.311264	3.420935	3.403994	35.3487
3.301737	3.502414	3.444389	35.59439
3.276455	3.300368	3.415623	35.03435
3.154333	3.174341	3.415299	34.49098
3.28326	3.229065	3.354834	34.76037
3.148765	3.332919	3.386021	34.76156
3.253565	3.477343	3.440297	35.42527
3.137464	3.308118	3.413696	34.74314
3.334814	3.268423	3.390796	35.03782
3.304005	3.287226	3.365491	34.95623
3.365087	3.400717	3.354629	35.31424
3.249729	3.414061	3.42142	35.23721
3.368139	3.444455	3.388272	35.49013
3.277032	3.245711	3.458917	35.01077
3.392349	3.257303	3.259345	34.85186
3.204485	3.230817	3.440791	34.77991
3.098819	3.265188	3.318207	34.35593
3.167943	3.346532	3.370129	34.79852
3.301036	3.433335	3.365884	35.27011
3.329906	3.428887	3.385203	35.36577
3.373377	3.372277	3.459053	35.49853
3.165551	3.371333	3.466368	35.05799
3.336258	3.366616	3.369533	35.20921
3.398205	3.499786	3.416919	35.73953
3.299262	3.345791	3.40089	35.15134
3.22919	3.160054	3.375279	34.53592
3.168108	3.17825	3.378433	34.44904
3.272454	3.274893	3.386414	34.90602
3.265113	3.473974	3.34965	35.24493
3.211826	3.384677	3.449232	35.15089
3.140599	3.250294	3.49065	34.79183
3.269814	3.246453	3.380565	34.82526
3.279218	3.377196	3.436767	35.25464
3.318193	3.429292	3.391546	35.35491
3.235087	3.453351	3.442633	35.3375
3.269773	3.341276	3.453205	35.19138
3.271258	3.231424	3.347059	34.72228

3.471371	3.256899	3.270531	35.04825
3.239253	3.248137	3.372159	34.74373
3.223539	3.378949	3.42865	35.11897
3.141341	3.398628	3.379133	34.87396
3.370613	3.394314	3.35272	35.30815
3.310975	3.376522	3.372636	35.18238
3.351848	3.35435	3.400379	35.28394
3.198092	3.388586	3.475235	35.18627
3.360756	3.466089	3.374495	35.49117
3.331721	3.410287	3.389005	35.33738
3.398741	3.184046	3.396048	35.00459
3.232984	3.137881	3.362473	34.46773
3.130123	3.256629	3.404591	34.59457
3.182213	3.407389	3.427286	35.0878
3.270887	3.464741	3.440706	35.43649
3.233149	3.323349	3.495885	35.16542
3.176027	3.223943	3.402442	34.61878
3.214961	3.262897	3.461031	34.91723
3.389255	3.426124	3.423927	35.5742
3.389874	3.503155	3.43779	35.77432
3.261195	3.42754	3.399322	35.24344
3.315306	3.242881	3.394939	34.94837
3.303964	3.20514	3.352515	34.74826
3.290972	3.205545	3.306271	34.6196
3.311017	3.285069	3.363445	34.96237
3.28759	3.409074	3.454893	35.3823
3.159035	3.399302	3.378246	34.91219
3.33469	3.410152	3.414975	35.40037
3.274269	3.369783	3.340801	35.01775
3.266309	3.35253	3.45803	35.21897
3.221807	3.429427	3.373079	35.10404
3.299757	3.470065	3.472558	35.58092
3.266927	3.339052	3.337169	34.92655
3.363643	3.182496	3.325181	34.76947
3.206465	3.193212	3.434908	34.68913
3.296169	3.37659	3.326273	35.04875
3.202794	3.44277	3.54958	35.47762
3.216569	3.426596	3.343802	35.02237
3.203248	3.238231	3.427337	34.76399
3.267505	3.275162	3.372602	34.86558
3.193968	3.411904	3.553195	35.39873
3.328009	3.503897	3.406142	35.57144
3.237603	3.433201	3.479805	35.38023
3.400556	3.337299	3.381946	35.31286
3.370531	3.177037	3.378655	34.88953
3.29254	3.188292	3.348713	34.67811
3.245852	3.354754	3.386635	35.02297
3.254802	3.489205	3.433067	35.4381
3.303799	3.462382	3.360836	35.32864
3.266639	3.391416	3.393933	35.16456

3.232654	3.355428	3.394257	35.01225
3.307387	3.402065	3.471535	35.44666
3.340134	3.450453	3.429861	35.53296
3.272908	3.465887	3.464237	35.49487
3.308542	3.317418	3.396576	35.10016
3.224818	3.12629	3.394735	34.49507
3.325452	3.24962	3.337578	34.85985
3.321864	3.412106	3.391683	35.32565
3.296416	3.429561	3.383054	35.28931
3.153178	3.369379	3.373659	34.82391
3.264618	3.290191	3.42345	35.00333
3.219786	3.254742	3.41477	34.80879
3.291261	3.37841	3.438063	35.28647
3.244532	3.405569	3.409126	35.18039
3.232654	3.400717	3.483062	35.30549
3.24845	3.349161	3.374546	34.98998
3.43652	3.193886	3.349923	35.00786
3.364427	3.280015	3.356556	35.05305
3.311553	3.328066	3.398964	35.13525
3.23983	3.391686	3.365696	35.04478
3.26767	3.41588	3.360291	35.14675
3.271258	3.364055	3.323749	34.96135
3.319059	3.363516	3.338618	35.09722
3.215332	3.403682	3.403687	35.10052
3.254142	3.426124	3.396031	35.21772
3.261978	3.42882	3.51355	35.49775
3.351724	3.385216	3.293244	35.11688
3.304913	3.178655	3.389074	34.77236
3.240325	3.165513	3.408171	34.64414
3.264205	3.267884	3.461389	35.03661
3.280002	3.45389	3.337237	35.20642
3.290065	3.429427	3.45878	35.44072
3.206093	3.35125	3.365082	34.88123
3.144599	3.210599	3.420858	34.56114
3.265855	3.363448	3.383634	35.07916
3.319554	3.482533	3.399117	35.49087
3.272207	3.453621	3.423774	35.37803
3.313491	3.425787	3.37313	35.29669
3.283507	3.196447	3.418266	34.8283
3.36187	3.21316	3.316298	34.81322
3.334154	3.289315	3.43247	35.1732
3.318647	3.438929	3.421403	35.44227
3.21426	3.412309	3.473377	35.26944
3.378078	3.39883	3.342966	35.31302
3.253276	3.314453	3.349122	34.86904
3.374119	3.391282	3.309017	35.21361
3.179326	3.416554	3.482448	35.22216
3.210795	3.44304	3.385817	35.13758
3.223498	3.374164	3.434908	35.1221
3.374696	3.226032	3.357255	34.95899

3.281074	3.13869	3.391938	34.6391
3.358158	3.288372	3.413645	35.18246
3.248492	3.461506	3.463742	35.43081
3.328957	3.432931	3.383481	35.36877
3.285817	3.382857	3.344603	35.07991
3.267752	3.260336	3.33427	34.74987
3.183863	3.33676	3.380821	34.83535
3.224859	3.395325	3.445889	35.19537
3.251874	3.490081	3.443093	35.45554
3.3191	3.387912	3.390574	35.26428
3.406289	3.169017	3.333469	34.85138
3.214342	3.152708	3.378706	34.49488
3.282889	3.286417	3.386414	34.95404
3.238428	3.418644	3.392774	35.15988
3.384182	3.495607	3.361655	35.57887
3.309202	3.394247	3.382424	35.23866
3.280043	3.329212	3.358347	34.98002
3.277156	3.363852	3.37504	35.08597
3.359313	3.481791	3.394326	35.56572
3.262968	3.453553	3.387062	35.27739
3.311347	3.356776	3.376404	35.14825
3.144599	3.210734	3.40442	34.52549
3.279713	3.206084	3.404932	34.81191
3.385543	3.288641	3.400123	35.21337
3.353126	3.477343	3.426945	35.6138
3.246677	3.410961	3.399032	35.1748
3.349909	3.341006	3.426809	35.30832
3.22391	3.283587	3.343648	34.72535
3.378615	3.307242	3.342011	35.11181
3.205021	3.357315	3.441234	35.05868
3.153879	3.429898	3.415674	35.04967
3.166995	3.389664	3.439632	35.04276
3.311058	3.294033	3.38996	35.04005
3.372717	3.17333	3.412554	34.96034
3.357333	3.168546	3.365287	34.81287
3.206217	3.284463	3.489081	35.00661
3.345207	3.40348	3.297899	35.15275
3.398288	3.379151	3.317883	35.25933
3.335474	3.406311	3.341602	35.23323
3.22057	3.33703	3.392092	34.94086
3.293901	3.448769	3.352532	35.25906
3.264412	3.481724	3.360035	35.28305
3.372675	3.39054	3.378911	35.36168
3.264865	3.281767	3.439273	35.02005
3.104305	3.176835	3.419835	34.39695
3.275094	3.231491	3.427661	34.90708
3.298561	3.361763	3.375654	35.12955
3.445223	3.464336	3.356147	35.63193
3.222591	3.296526	3.415282	34.90741
3.262679	3.313914	3.420329	35.04414

3.252038	3.251777	3.364673	34.76328
3.352507	3.329414	3.386567	35.20065
3.134948	3.429764	3.420312	35.01812
3.272289	3.444455	3.374239	35.24984
3.14563	3.317957	3.439137	34.83815
3.351971	3.247531	3.312018	34.85738
3.33003	3.191325	3.382884	34.84146
3.288209	3.315868	3.367265	34.9882
3.222096	3.404221	3.452233	35.22265
3.373418	3.452947	3.369874	35.48001
3.362777	3.35098	3.365406	35.22399
3.420765	3.346061	3.292357	35.1803
3.221436	3.368907	3.401624	35.0333
3.161179	3.463797	3.367197	35.03376
3.182296	3.446073	3.409962	35.13469
3.293818	3.312768	3.413474	35.09474
3.211043	3.200153	3.379593	34.59336
3.249894	3.263234	3.462958	34.99858
3.255297	3.382723	3.423927	35.18634
3.396225	3.494529	3.42478	35.74089
3.389585	3.367087	3.383873	35.35822
3.267051	3.312431	3.421625	35.05329
3.270804	3.263031	3.358944	34.8164
3.234015	3.26694	3.425104	34.88918
3.325658	3.448364	3.353282	35.32927
3.293323	3.411972	3.440655	35.37004
3.265731	3.354485	3.392007	35.0776
3.190503	3.190179	3.380411	34.52842
3.297159	3.19247	3.392689	34.79352
3.334154	3.309129	3.417294	35.18334
3.312749	3.444253	3.427968	35.45537
3.215621	3.45106	3.388562	35.17168
3.411321	3.396134	3.425854	35.56108
3.335144	3.331773	3.308249	34.99657
3.372717	3.361763	3.385749	35.31379
3.228447	3.469728	3.333213	35.11951
3.193019	3.444994	3.445736	35.23402
3.174831	3.269164	3.347485	34.59487
3.256617	3.134714	3.351577	34.48865
3.246842	3.205814	3.46867	34.87883
3.333371	3.372546	3.400072	35.28265
3.263504	3.458945	3.450596	35.42929
3.306686	3.434077	3.355397	35.26116
3.395236	3.315127	3.406603	35.30666
3.36121	3.364189	3.321822	35.15414
3.257235	3.357854	3.404232	35.09313
3.248285	3.372883	3.362405	35.01495
3.230674	3.353002	3.447475	35.119
3.28759	3.314857	3.321294	34.88411
3.257565	3.296391	3.410627	34.97342

3.202711	3.245172	3.431242	34.78654
3.279053	3.235535	3.360717	34.77818
3.301036	3.303872	3.396576	35.05412
3.280414	3.415813	3.443315	35.35603
3.282806	3.4239	3.380053	35.2406
3.380718	3.348285	3.455643	35.45466
3.291426	3.420059	3.370658	35.23051
3.452275	3.450656	3.357631	35.62068
3.237397	3.429898	3.3873	35.17026
3.314852	3.421002	3.364605	35.27056
3.109171	3.271119	3.382816	34.53282
3.241728	3.238298	3.38701	34.7601
3.173882	3.255888	3.444952	34.77691
3.316378	3.34633	3.333554	35.0427
3.29089	3.37012	3.458115	35.31138
3.354075	3.362976	3.362678	35.22523
3.356591	3.301446	3.428633	35.24041
3.417672	3.379151	3.259362	35.17374
3.278929	3.277117	3.396457	34.947
3.208114	3.332582	3.376217	34.86917
3.242016	3.405906	3.406807	35.17056
3.215373	3.407254	3.366975	35.02813
3.308542	3.311083	3.361859	35.01038
3.20267	3.221247	3.422989	34.71608
3.176646	3.224415	3.440979	34.70544
3.33568	3.312162	3.437637	35.2378
3.33535	3.400447	3.370811	35.28401
3.340134	3.436031	3.35804	35.34436
3.382533	3.345454	3.386959	35.30224
3.270928	3.378207	3.397889	35.15371
3.356632	3.41797	3.346104	35.31484
3.29155	3.416083	3.416697	35.32276
3.309037	3.342354	3.338209	35.02813
3.127978	3.209453	3.363684	34.39726
3.282146	3.238433	3.32542	34.7141
3.200402	3.359741	3.411036	34.98785
3.331803	3.453284	3.430543	35.52242
3.193638	3.426866	3.435505	35.17335
3.368221	3.336221	3.384777	35.24598
3.270103	3.297874	3.431515	35.04976
3.406619	3.311218	3.331047	35.15777
3.288993	3.335008	3.427883	35.16433
3.161633	3.367155	3.358841	34.80513
3.101954	3.312498	3.422955	34.69531
3.23517	3.282306	3.327091	34.71097
3.231334	3.231963	3.406125	34.76532
3.226674	3.256831	3.377069	34.74597
3.173222	3.273949	3.498903	34.93295
3.344259	3.434077	3.425786	35.49725
3.450378	3.500662	3.371084	35.75531

3.38311	3.423092	3.350111	35.3927
3.357869	3.371872	3.362132	35.25179
3.282064	3.361022	3.372994	35.08604
3.283012	3.373962	3.394667	35.1638
3.302438	3.351115	3.318634	34.99005
3.317822	3.2354	3.365201	34.87248
3.070526	3.19712	3.436767	34.40447
3.27897	3.288102	3.369908	34.91306
3.22391	3.420261	3.46347	35.28627
3.287879	3.45726	3.396679	35.36101
3.254389	3.364661	3.423057	35.14296
3.278805	3.311959	3.406841	35.04564
3.271299	3.363852	3.393132	35.11272
3.349125	3.450319	3.312035	35.29466
3.234799	3.29255	3.454824	35.01189
3.248162	3.305826	3.347059	34.83448
3.146043	3.254338	3.40396	34.623
3.243749	3.231087	3.342233	34.65083
3.214466	3.27523	3.373813	34.75239
3.214796	3.289719	3.454824	34.96195
3.198793	3.424102	3.466505	35.24638
3.396679	3.44304	3.468687	35.72531
3.333989	3.477074	3.422495	35.56163
3.503541	3.477411	3.377274	35.83425
3.372634	3.394112	3.366821	35.34296
3.258225	3.365807	3.407319	35.11943
3.265113	3.401728	3.37429	35.14082
3.227045	3.305422	3.340119	34.77224
3.317451	3.169556	3.34994	34.6943
3.12637	3.182361	3.425172	34.46896
3.213393	3.354552	3.38759	34.95364
3.290436	3.431785	3.403857	35.32659
3.322029	3.382386	3.379405	35.23417
3.233809	3.345724	3.425359	35.06157
3.313574	3.36729	3.381503	35.18726
3.286972	3.423429	3.410644	35.31558
3.361622	3.361696	3.346036	35.20254
3.294808	3.287967	3.367742	34.94266
3.242511	3.296863	3.372789	34.85879
3.179656	3.237557	3.326068	34.48947
3.253235	3.249822	3.380343	34.79589
3.170995	3.306837	3.366702	34.71089
3.271959	3.299828	3.424217	35.04213
3.200319	3.345454	3.430884	34.99983
3.351476	3.421339	3.421557	35.47593
3.321864	3.449712	3.36943	35.35923
3.358446	3.393438	3.407302	35.39899
3.315553	3.399908	3.414736	35.33559
3.346197	3.381981	3.358534	35.2405
3.228653	3.345387	3.415333	35.02763

3.258926	3.271119	3.342898	34.77302
3.310728	3.209588	3.36631	34.80294
3.200113	3.280823	3.372107	34.7295
3.194091	3.396741	3.473342	35.19121
3.262019	3.414465	3.415538	35.25211
3.36752	3.379151	3.392842	35.35596
3.39969	3.340736	3.392518	35.3416
3.288044	3.316138	3.467357	35.20732
3.281486	3.444118	3.35526	35.22771
3.286064	3.35927	3.424541	35.20368
3.277403	3.275702	3.343853	34.82554
3.361705	3.279341	3.377786	35.09205
3.166912	3.244229	3.332803	34.49092
3.277774	3.259662	3.426945	34.97298
3.202093	3.300907	3.379678	34.79431
3.240202	3.353069	3.445071	35.13472
3.214589	3.41743	3.406739	35.13563
3.391482	3.426596	3.426041	35.58472
3.268082	3.410758	3.437926	35.30622
3.437964	3.412578	3.284906	35.34707
3.361127	3.361022	3.445071	35.41656
3.383234	3.339389	3.336811	35.18084
3.282435	3.274354	3.411718	34.982
3.182915	3.267951	3.347331	34.60956
3.179368	3.280621	3.388255	34.71901
3.29023	3.443714	3.328455	35.18733
3.210506	3.451464	3.453733	35.3039
3.261937	3.424574	3.39141	35.22127
3.287013	3.333727	3.444798	35.19419
3.362695	3.37969	3.382389	35.32373
3.284332	3.413319	3.417038	35.30168
3.269773	3.441557	3.397804	35.28953
3.21727	3.212756	3.452557	34.7941
3.255915	3.204871	3.361024	34.6612
3.284951	3.267816	3.352873	34.84452
3.280661	3.254742	3.329001	34.75435
3.210754	3.332312	3.39748	34.92086
3.189596	3.360955	3.440126	35.03048
3.318234	3.365335	3.406688	35.24825
3.321657	3.405569	3.36423	35.25087
3.406206	3.418981	3.384384	35.50917
3.259627	3.442366	3.426246	35.33131
3.277692	3.409613	3.369891	35.17595
3.321327	3.304074	3.386021	35.07585
3.403031	3.29565	3.361689	35.18289
3.169923	3.246857	3.449521	34.75849
3.223044	3.270445	3.358176	34.72649
3.206341	3.329347	3.388289	34.88462
3.264082	3.462719	3.372176	35.26732
3.224323	3.43684	3.369839	35.11867

3.231004	3.382116	3.456137	35.20233
3.159777	3.369042	3.423006	34.94552
3.388389	3.426192	3.418965	35.5616
3.348342	3.461304	3.368339	35.4401
3.36851	3.296257	3.406944	35.20769
3.276537	3.178587	3.345967	34.61589
3.273815	3.166187	3.361058	34.61582
3.257606	3.225426	3.338891	34.66145
3.344547	3.304546	3.359609	35.0699
3.176563	3.386227	3.388204	34.9437
3.208774	3.313307	3.416646	34.91688
3.302355	3.410287	3.424149	35.35001
3.335268	3.505312	3.365491	35.50152
3.379563	3.429966	3.376387	35.45744
3.207867	3.391484	3.39303	35.03421
3.2209	3.357113	3.429929	35.06824
3.292127	3.303939	3.269593	34.75709
3.306356	3.234052	3.364877	34.84375
3.27464	3.266199	3.396986	34.9149
3.179656	3.281902	3.42865	34.81078
3.20531	3.422889	3.415674	35.14682
3.332216	3.435492	3.383361	35.38124
3.31778	3.388316	3.395894	35.27391
3.293901	3.347948	3.473598	35.30334
3.18143	3.391484	3.399663	34.9909
3.281528	3.410085	3.435744	35.32938
3.306892	3.402199	3.394002	35.27632
3.40666	3.270714	3.358875	35.13014
3.230262	3.183103	3.379133	34.5971
3.346115	3.223808	3.369567	34.91855
3.266391	3.24315	3.425376	34.90855
3.288209	3.423159	3.337118	35.1569
3.158457	3.379825	3.438932	35.00104
3.225024	3.368166	3.37886	34.98975
3.185637	3.397212	3.456922	35.13784
3.349827	3.484622	3.318054	35.38437
3.305655	3.374029	3.440586	35.31388
3.361581	3.39883	3.34138	35.27347
3.231994	3.297335	3.440859	34.98568
3.324586	3.228795	3.308744	34.74936
3.315718	3.272534	3.3238	34.85855
3.278476	3.28271	3.359506	34.87744
3.120431	3.322742	3.439819	34.79499
3.230468	3.418037	3.407251	35.1728
3.296334	3.395056	3.366702	35.17791
3.309532	3.389529	3.368544	35.19871
3.293653	3.385553	3.429878	35.28942
3.141506	3.40193	3.487768	35.11911
3.280208	3.421272	3.433408	35.34585
3.266185	3.290528	3.426041	35.01316

3.371892	3.248003	3.324943	34.93024
3.198546	3.205342	3.41801	34.66139
3.284002	3.226639	3.357221	34.76191
3.23451	3.296459	3.381929	34.86039
3.341743	3.400245	3.334185	35.21745
3.162499	3.332986	3.382458	34.78395
3.248079	3.412982	3.373506	35.12647
3.153302	3.467437	3.413918	35.12666
3.277816	3.42855	3.331235	35.1331
3.373088	3.33548	3.377615	35.23934
3.311965	3.33393	3.369362	35.08424
3.245605	3.254068	3.423228	34.88227
3.312955	3.303063	3.380565	35.0434
3.243542	3.292954	3.372312	34.85145
3.364798	3.314722	3.340545	35.09475
3.167325	3.388316	3.390711	34.93355
3.207454	3.342826	3.373199	34.88353
3.162994	3.375175	3.403499	34.92331
3.243089	3.369648	3.451568	35.1915
3.264824	3.45598	3.420585	35.36007
3.267092	3.39856	3.485875	35.38224
3.232654	3.318497	3.449641	35.0526
3.316502	3.223674	3.409194	34.94015
3.407402	3.252518	3.362405	35.09969
3.208362	3.266199	3.42079	34.82202
3.25043	3.299289	3.361859	34.85751
3.262679	3.358798	3.385953	35.06712
3.337165	3.385216	3.346274	35.20101
3.309697	3.369716	3.346871	35.10835
3.250348	3.426259	3.383702	35.18276
3.147899	3.446208	3.445821	35.13819
3.297942	3.423563	3.412213	35.34329
3.255049	3.308994	3.394326	34.95983
3.359601	3.237624	3.399373	35.04343
3.239377	3.252248	3.387965	34.78756
3.333865	3.314049	3.430833	35.22308
3.265278	3.378207	3.398077	35.14176
3.308418	3.350778	3.352328	35.07607
3.216198	3.40038	3.43825	35.17077
3.18906	3.395932	3.392825	35.00236
3.128803	3.403682	3.40633	34.91707
3.268866	3.489677	3.430901	35.46515
3.309326	3.432594	3.461935	35.49667
3.29221	3.320249	3.378348	35.03077
3.187534	3.226369	3.434976	34.7204
3.243831	3.238365	3.407762	34.81023
3.387111	3.226369	3.379883	35.03636
3.298107	3.281093	3.350196	34.89647
3.224364	3.30495	3.420875	34.94194
3.274929	3.421137	3.372056	35.19984

3.292045	3.391012	3.411599	35.25787
3.273238	3.424035	3.344126	35.1414
3.296045	3.472559	3.428378	35.48164
3.155034	3.445466	3.481919	35.23111
3.25773	3.360685	3.395093	35.08041
3.278847	3.297807	3.375432	34.94609
3.305572	3.26539	3.422409	35.03638
3.190008	3.274556	3.422307	34.80348
3.368881	3.289989	3.418897	35.22094
3.227746	3.365807	3.433902	35.11091
3.399442	3.418778	3.35405	35.4276
3.16419	3.352598	3.397003	34.86235
3.339557	3.406378	3.369925	35.30424
3.16353	3.401189	3.412417	35.00087
3.253977	3.430033	3.404932	35.24537
3.234345	3.436099	3.424012	35.25743
3.341124	3.329684	3.385817	35.1747
3.181471	3.2385	3.431242	34.7255
3.333288	3.231154	3.399697	34.97245
3.283301	3.234389	3.431498	34.93975
3.362406	3.319507	3.341739	35.1026
3.246677	3.310274	3.396576	34.94924
3.236572	3.38744	3.396866	35.09653
3.1957	3.352328	3.378024	34.88916
3.24478	3.40011	3.345234	35.02928
3.169222	3.4301	3.420738	35.09474
3.301531	3.44924	3.364059	35.30199
3.224199	3.374366	3.426758	35.10625
3.206753	3.208038	3.368066	34.57602
3.382904	3.302929	3.41049	35.26151
3.241975	3.364324	3.363582	34.98501
3.344341	3.383599	3.370146	35.26537
3.320915	3.359876	3.37608	35.17524
3.280249	3.328066	3.406552	35.08338
3.231705	3.341343	3.375569	34.93851
3.330731	3.401795	3.411582	35.36601
3.133546	3.464134	3.484358	35.23028
3.273279	3.391214	3.36793	35.12178
3.205351	3.284261	3.430748	34.87671
3.280579	3.270782	3.384384	34.91036
3.218301	3.24032	3.456001	34.86416
3.29221	3.276578	3.401487	34.98587
3.240119	3.274084	3.408922	34.88276
3.343104	3.377736	3.338601	35.18086
3.276413	3.371401	3.420449	35.20015
3.347641	3.411635	3.320015	35.22427
3.207949	3.348756	3.376319	34.90441
3.219374	3.412915	3.364946	35.04482
3.164108	3.494933	3.45612	35.30271
3.308501	3.363583	3.348218	35.09527

3.270268	3.266064	3.360768	34.82585
3.199206	3.175083	3.388579	34.5323
3.269113	3.273612	3.376797	34.87488
3.40167	3.387508	3.401231	35.46727
3.388183	3.414937	3.310977	35.30039
3.204732	3.315194	3.432657	34.94718
3.226509	3.354687	3.435386	35.08713
3.197597	3.442905	3.391938	35.12182
3.375439	3.498303	3.359012	35.55987
3.205268	3.394651	3.478219	35.22175
3.140887	3.321394	3.454159	34.86814
3.167489	3.243959	3.423057	34.68896
3.357415	3.239578	3.372926	34.98509
3.279259	3.220776	3.360563	34.74602
3.273733	3.252585	3.469352	35.0414
3.222962	3.335008	3.413236	34.9879
3.381832	3.384205	3.335054	35.27194
3.370943	3.445803	3.31565	35.3404
3.352219	3.391214	3.401504	35.36783
3.160561	3.344106	3.361945	34.75917
3.284373	3.443781	3.421881	35.37898
3.18906	3.478152	3.412537	35.22527
3.367974	3.360752	3.389023	35.30837
3.284827	3.20669	3.430134	34.87954
3.241356	3.219091	3.496447	34.95661
3.228117	3.38589	3.389892	35.0594
3.348796	3.416757	3.396627	35.40553
3.375645	3.312903	3.388119	35.21853
3.316914	3.268693	3.3526	34.91574
3.169222	3.323349	3.430816	34.88333
3.291797	3.54777	3.319452	35.39862
3.233726	3.445871	3.43218	35.29531
3.204072	3.418981	3.341039	34.97235
3.167778	3.271523	3.464936	34.84145
3.216322	3.252585	3.354391	34.66445
3.287425	3.229739	3.41165	34.8952
3.366819	3.267614	3.365952	35.05171
3.255132	3.226908	3.436306	34.87231
3.236778	3.322001	3.418488	35.00116
3.323637	3.369042	3.413031	35.28204
3.290601	3.365066	3.374767	35.11743
3.353827	3.361763	3.420039	35.34747
3.149301	3.429966	3.434976	35.08202
3.247378	3.478961	3.434908	35.40349
3.24746	3.408871	3.435454	35.25159
3.360591	3.245981	3.290993	34.82686
3.228818	3.209251	3.419101	34.73853
3.296375	3.35253	3.405784	35.17047
3.249523	3.428416	3.386482	35.19175
3.434046	3.425855	3.294335	35.38816

3.314069	3.262223	3.355243	34.90115
3.261195	3.288708	3.34474	34.82047
3.268206	3.407321	3.438455	35.30013
3.286972	3.489205	3.41697	35.47325
3.17846	3.324764	3.365969	34.76482
3.266474	3.288843	3.320816	34.78
3.164644	3.246048	3.484716	34.82215
3.223416	3.33703	3.453068	35.08042
3.186791	3.341815	3.443656	34.99021
3.301984	3.297133	3.384316	35.01464
3.266226	3.331975	3.427491	35.10706
3.349538	3.348689	3.366941	35.19338
3.288168	3.338647	3.431907	35.17929
3.318275	3.361628	3.366787	35.15298
3.263092	3.367559	3.394888	35.10672
3.250306	3.415004	3.444406	35.2908
3.211372	3.381307	3.347059	34.91909
3.259998	3.258584	3.398094	34.86866
3.29254	3.257168	3.353248	34.83865
3.215786	3.341208	3.419084	34.99856
3.381708	3.431044	3.37209	35.45509
3.302974	3.420059	3.410593	35.34309
3.285198	3.360685	3.41963	35.19414
3.32607	3.305624	3.335651	34.97946
3.405588	3.395595	3.383293	35.45429
3.242841	3.509153	3.44207	35.47526
3.227829	3.397549	3.440416	35.19475
3.180069	3.24497	3.397122	34.66197
3.165716	3.254405	3.339778	34.52581
3.198133	3.303063	3.374733	34.77955
3.27563	3.305287	3.46451	35.15021
3.128514	3.269771	3.48226	34.78965
3.344547	3.328403	3.428633	35.27302
3.357292	3.381712	3.350213	35.24597
3.462215	3.362976	3.358449	35.45246
3.281816	3.365537	3.387437	35.12695
3.22057	3.300772	3.412895	34.90706
3.225519	3.347274	3.396645	34.98404
3.379976	3.430235	3.27048	35.22733
3.259833	3.307983	3.392774	34.96469
3.087312	3.215789	3.349412	34.29097
3.253565	3.214778	3.405307	34.77456
3.238016	3.392899	3.362132	35.03567
3.417837	3.462449	3.422802	35.71368
3.228653	3.405097	3.476599	35.29219
3.229602	3.239376	3.401777	34.76824
3.268288	3.423968	3.386482	35.22306
3.333247	3.535841	3.387914	35.61289
3.323266	3.486239	3.448959	35.61609
3.195205	3.32901	3.436426	34.9648

3.139197	3.196447	3.434516	34.54825
3.292416	3.30549	3.383907	35.0111
3.247832	3.362707	3.379661	35.02944
3.296911	3.339793	3.404625	35.14125
3.209475	3.255618	3.427474	34.81593
3.262597	3.331975	3.42686	35.09774
3.415734	3.413319	3.364434	35.474
3.51645	3.423833	3.39644	35.78723
3.282847	3.339389	3.486421	35.28849
3.265278	3.334199	3.380207	35.00644
3.269484	3.348352	3.483744	35.27301
3.406165	3.378679	3.273515	35.1785
3.339763	3.286215	3.379422	35.06268
3.037449	3.245913	3.410661	34.38175
3.091065	3.296594	3.435744	34.66468
3.342279	3.477546	3.410286	35.55409
3.335639	3.432392	3.414225	35.44944
3.365417	3.335412	3.471603	35.42795
3.188152	3.289989	3.440245	34.8724
3.294478	3.462652	3.378775	35.34807
3.400391	3.498303	3.343989	35.58158
3.277444	3.376455	3.401879	35.17285
3.17978	3.242476	3.389023	34.63817
3.187823	3.242274	3.370828	34.61553
3.302397	3.361157	3.426195	35.24714
3.287054	3.364931	3.369481	35.09782
3.291962	3.307174	3.49377	35.25404
3.100758	3.313307	3.470802	34.79909
3.351641	3.361696	3.378365	35.25141
3.339021	3.382049	3.37475	35.26042
3.497066	3.417093	3.372039	35.67674
3.275341	3.335817	3.481101	35.25263
3.27398	3.370659	3.423876	35.2007
3.333041	3.358663	3.354016	35.15085
3.414538	3.339928	3.276482	35.11855
3.224529	3.207095	3.411309	34.70739
3.213105	3.341141	3.38198	34.91141
3.128226	3.378207	3.481306	35.02406
3.378656	3.47485	3.302605	35.39226
3.401835	3.331706	3.414856	35.37539
3.395359	3.309129	3.359592	35.191
3.235459	3.347071	3.465209	35.15527
3.383976	3.555385	3.330945	35.64199
3.319719	3.406715	3.434652	35.40314
3.340671	3.27004	3.301377	34.85862
3.101129	3.185259	3.377547	34.31595
3.14662	3.305961	3.39823	34.72462
3.288003	3.410556	3.403619	35.27432
3.227664	3.327729	3.460162	35.08489
3.265154	3.315262	3.351424	34.90182

3.234716	3.250833	3.470512	34.95479
3.275052	3.366076	3.382304	35.10212
3.352384	3.411567	3.444542	35.50681
3.458998	3.451532	3.326	35.56812
3.239665	3.362842	3.478287	35.22756
3.301778	3.326719	3.2876	34.86739
3.333494	3.251844	3.421744	35.06636
3.326235	3.288911	3.306698	34.87996
3.205805	3.326045	3.389739	34.8794
3.186461	3.426326	3.404147	35.08791
3.214589	3.433201	3.355721	35.05855
3.328009	3.352733	3.385271	35.19523
3.261937	3.30859	3.370624	34.92218
3.349662	3.383397	3.443178	35.43627
3.285446	3.483746	3.422222	35.46946
3.412888	3.424439	3.419579	35.61268
3.261071	3.226571	3.366412	34.73171
3.257276	3.141049	3.435727	34.68797
3.168232	3.249351	3.398691	34.64909
3.260452	3.474041	3.391069	35.32546
3.083188	3.442434	3.406006	34.90135
3.24911	3.321394	3.447049	35.08926
3.289652	3.305287	3.463794	35.17931
3.313161	3.349498	3.419903	35.23142
3.311017	3.400447	3.446674	35.39669
3.293076	3.437716	3.39129	35.31785
3.299221	3.466224	3.394462	35.40056
3.4093	3.317553	3.286082	35.07916
3.284497	3.269636	3.346871	34.83438
3.252863	3.263908	3.293994	34.63705
3.258844	3.312768	3.416356	35.02456
3.173882	3.448229	3.370863	35.03551
3.300293	3.420665	3.448226	35.42085
3.2866	3.417835	3.42524	35.33445
3.270804	3.285676	3.426127	35.01284
3.184193	3.351991	3.475355	35.07611
3.377295	3.475322	3.359063	35.51378
3.277939	3.561653	3.413918	35.60526
3.397174	3.260605	3.343103	35.0528
3.239789	3.111059	3.426911	34.56487
3.291178	3.206286	3.351628	34.72086
3.268371	3.330762	3.394036	35.03593
3.315595	3.519397	3.40546	35.5767
3.089787	3.323416	3.442888	34.73617
3.228076	3.247531	3.381025	34.73735
3.231004	3.242072	3.390523	34.75259
3.38509	3.40321	3.365508	35.38723
3.202959	3.44614	3.49302	35.36166
3.294519	3.483207	3.399526	35.43849
3.299056	3.318362	3.423808	35.14103

3.360179	3.232435	3.296126	34.80756
3.277692	3.250698	3.330314	34.74188
3.308418	3.217473	3.357443	34.79574
3.078115	3.42545	3.449436	34.94809
3.271464	3.460967	3.395008	35.32956
3.346857	3.441894	3.348508	35.35104
3.350775	3.367155	3.347348	35.19363
3.283383	3.345454	3.480419	35.2898
3.265443	3.454295	3.40442	35.32238
3.401958	3.453082	3.411121	35.63292
3.391482	3.40375	3.372926	35.41861
3.340506	3.1736	3.354016	34.76247
3.200196	3.174341	3.417788	34.59672
3.280084	3.310409	3.39985	35.02976
3.22189	3.467504	3.456206	35.36927
3.317657	3.373018	3.375893	35.19644
3.136433	3.231491	3.455166	34.664
3.191741	3.304344	3.398571	34.8205
3.293076	3.41123	3.419766	35.3222
3.326813	3.401054	3.358125	35.23892
3.196277	3.368503	3.424763	35.028
3.289735	3.364324	3.479191	35.34227
3.266969	3.310274	3.358449	34.91024
3.480486	3.284463	3.350435	35.3032
3.325823	3.281632	3.274896	34.79359
3.356714	3.276038	3.422904	35.17258
3.163365	3.36055	3.364929	34.80779
3.328999	3.344915	3.364877	35.1357
3.221436	3.426731	3.413577	35.1859
3.365045	3.366211	3.326614	35.17743
3.229066	3.341006	3.480487	35.16144
3.296375	3.456047	3.393183	35.36929
3.352466	3.486374	3.429997	35.63878
3.359354	3.286552	3.353368	35.04928
3.268866	3.190718	3.372841	34.68441
3.1957	3.216058	3.398299	34.6355
3.215744	3.292617	3.419033	34.8921
3.229891	3.446477	3.433476	35.29108
3.193679	3.331234	3.384862	34.85356
3.275506	3.249688	3.427133	34.94662
3.22222	3.29228	3.475014	35.02794
3.197803	3.383464	3.434687	35.08576
3.309903	3.461708	3.407012	35.44149
3.282889	3.438188	3.42229	35.36439
3.336752	3.277521	3.409058	35.1019
3.319843	3.25299	3.371357	34.92882
3.308006	3.255888	3.359898	34.88422
3.265896	3.295717	3.353061	34.86428
3.356673	3.428416	3.389483	35.43263
3.168768	3.378679	3.37666	34.88491

3.325947	3.339389	3.362763	35.11232
3.278311	3.336221	3.383856	35.04735
3.308377	3.378612	3.413509	35.27064
3.238841	3.420733	3.373386	35.12295
3.270392	3.526406	3.469966	35.63424
3.229891	3.347274	3.441678	35.09208
3.335309	3.211677	3.327006	34.77531
3.26371	3.209386	3.388204	34.74756
3.332422	3.390675	3.369925	35.2543
3.215291	3.468447	3.464442	35.37492
3.238057	3.375849	3.345643	34.96241
3.194504	3.210464	3.347348	34.50923
3.340959	3.274556	3.342335	34.9587
3.166995	3.397212	3.449061	35.07989
3.271794	3.470604	3.408171	35.38014
3.242429	3.454295	3.452523	35.37725
3.427158	3.310814	3.372176	35.29175
3.37449	3.145901	3.376592	34.82559
3.228035	3.210464	3.377444	34.64837
3.279012	3.353137	3.386158	35.09091
3.241934	3.418981	3.401368	35.18707
3.403526	3.459147	3.319384	35.449
3.254884	3.307107	3.341756	34.84038
3.21261	3.347611	3.324568	34.79892
3.227045	3.448701	3.455455	35.33779
3.324132	3.504301	3.30518	35.34306
3.297324	3.4115	3.474877	35.4526
3.304335	3.34195	3.342062	35.02539
3.18543	3.139971	3.421693	34.49781
3.357168	3.24962	3.349804	34.95595
3.356013	3.375849	3.39569	35.32981
3.363437	3.427068	3.486268	35.65613
3.265071	3.363516	3.374154	35.05687
3.184647	3.312633	3.367674	34.75555
3.13466	3.327797	3.345882	34.63174
3.399236	3.432594	3.388272	35.5322
3.235087	3.364526	3.415214	35.0833
3.201268	3.305153	3.382338	34.80761
3.1851	3.317486	3.488126	35.03056
3.371521	3.284058	3.359677	35.08423
3.387853	3.27058	3.336555	35.03991
3.290477	3.250361	3.342932	34.7967
3.221807	3.232435	3.481306	34.90992
3.230097	3.382925	3.351492	34.97327
3.38344	3.389799	3.31819	35.25081
3.299138	3.443444	3.40014	35.36298
3.233891	3.427135	3.346854	35.06811
3.179863	3.421744	3.460162	35.18595
3.279012	3.420935	3.280881	35.00895
3.328463	3.392832	3.457553	35.44198

3.292911	3.347408	3.428378	35.2011
3.211084	3.189977	3.414446	34.64742
3.238016	3.230211	3.427968	34.82387
3.349868	3.333997	3.379133	35.18864
3.395153	3.378746	3.465482	35.57436
3.232365	3.358326	3.400549	35.03172
3.259669	3.262223	3.438472	34.96419
3.208774	3.306298	3.344416	34.7436
3.261236	3.334469	3.417533	35.07982
3.196236	3.439873	3.386686	35.10072
3.214095	3.447488	3.491264	35.38512
3.194298	3.369716	3.437926	35.05511
3.420807	3.24679	3.333281	35.05279
3.363726	3.148732	3.401572	34.86287
3.363602	3.285406	3.354169	35.05782
3.241604	3.344713	3.459207	35.15042
3.28359	3.477478	3.33444	35.25973
3.339681	3.336491	3.257299	34.90538
3.38047	3.339254	3.325505	35.14978
3.2103	3.402604	3.428446	35.1413
3.204485	3.42235	3.411991	35.13578
3.238387	3.416622	3.392433	35.15462
3.323266	3.326382	3.396252	35.15125
3.37317	3.181216	3.404232	34.96038
3.206753	3.197188	3.375501	34.56855
3.294189	3.396943	3.374546	35.1945
3.308212	3.437109	3.44693	35.47129
3.355312	3.412713	3.412213	35.44502
3.251007	3.315194	3.385817	34.94594
3.230633	3.208173	3.378928	34.65229
3.245481	3.32719	3.402783	34.99719
3.328628	3.458069	3.398418	35.45569
3.159488	3.444658	3.365423	34.98432
3.26767	3.316946	3.392706	35.00128
3.184111	3.209117	3.445071	34.69726
3.30483	3.14826	3.333008	34.5831
3.382079	3.302389	3.396184	35.22725
3.30714	3.469189	3.43779	35.51912
3.278599	3.458001	3.373727	35.29214
3.394947	3.40321	3.371613	35.42213
3.281115	3.274758	3.296484	34.728
3.341578	3.343365	3.340852	35.10728
3.232118	3.489205	3.441268	35.40643
3.156684	3.460967	3.454978	35.2097
3.163819	3.319238	3.372022	34.73395
3.265319	3.155808	3.412878	34.68787
3.2793	3.186742	3.4626	34.89482
3.282559	3.39209	3.386362	35.18429
3.235789	3.497562	3.525162	35.6162
3.333618	3.411095	3.358603	35.27681

3.347187	3.313846	3.426263	35.24178
3.274392	3.30987	3.338192	34.88129
3.201062	3.264851	3.396713	34.75046
3.216693	3.40348	3.38638	35.0652
3.238098	3.381847	3.422427	35.14353
3.234799	3.390271	3.389892	35.08359
3.343764	3.287226	3.342097	34.99202
3.223374	3.177307	3.44664	34.71699
3.260741	3.222056	3.403124	34.8014
3.235789	3.379353	3.447884	35.1887
3.366571	3.440007	3.431157	35.57076
3.331927	3.477074	3.358807	35.41784
3.366571	3.337367	3.382611	35.24014
3.21925	3.364526	3.40754	35.03188
3.386368	3.436772	3.312069	35.34655
3.174955	3.489205	3.402868	35.19745
3.283095	3.4084	3.354629	35.15174
3.110037	3.240522	3.475832	34.67122
3.247708	3.15911	3.365901	34.55385
3.289859	3.290798	3.453887	35.12641
3.309161	3.474378	3.385135	35.41973
3.239789	3.412106	3.51268	35.41077
3.334938	3.353272	3.440927	35.33327
3.296788	3.25737	3.3378	34.8146
3.38641	3.259999	3.313586	34.96339
3.228489	3.373692	3.363667	34.97619
3.078734	3.453149	3.481783	35.08076
3.174501	3.409411	3.369771	34.94958
3.331597	3.293763	3.395417	35.09631
3.306397	3.207566	3.428565	34.92519
3.264535	3.139162	3.42952	34.68614
3.204196	3.402873	3.439273	35.15222
3.33436	3.4146	3.404556	35.38659
3.475495	3.48132	3.380343	35.78818
3.352714	3.383801	3.342421	35.22349
3.242965	3.281228	3.357699	34.79259
3.229437	3.357585	3.367998	34.95251
3.270928	3.477546	3.397344	35.36975
3.293241	3.461843	3.322521	35.22058
3.29819	3.236209	3.436051	34.98625
3.088962	3.13222	3.393047	34.20725
3.273444	3.234457	3.460315	34.98136
3.322853	3.486239	3.411786	35.5339
3.337577	3.493653	3.484358	35.74101
3.25971	3.398695	3.376302	35.12677
3.282022	3.272062	3.448754	35.05708
3.286106	3.267951	3.374819	34.89533
3.400639	3.337839	3.376882	35.30314
3.210713	3.376994	3.406944	35.03917
3.238469	3.424776	3.39557	35.17949

3.196401	3.234591	3.403874	34.68975
3.383729	3.198873	3.331729	34.86353
3.212445	3.229132	3.420517	34.74929
3.249564	3.349093	3.348303	34.93488
3.15491	3.414196	3.525418	35.25758
3.484445	3.497696	3.308539	35.68654
3.356797	3.361224	3.396917	35.30222
3.44081	3.348891	3.284411	35.21294
3.279135	3.34141	3.442411	35.18855
3.23352	3.470065	3.300883	35.06064
3.172274	3.376994	3.411241	34.96451
3.242305	3.226302	3.360666	34.67752
3.160602	3.094817	3.351083	34.19035
3.218631	3.256629	3.414191	34.80912
3.229726	3.509558	3.388051	35.32933
3.334484	3.472087	3.484426	35.68724
3.418621	3.367896	3.305862	35.25289
3.353868	3.28635	3.452164	35.2529
3.318358	3.27833	3.337118	34.90612
3.309367	3.30859	3.436187	35.16928
3.275052	3.356237	3.336026	34.9794
3.273444	3.400245	3.413901	35.24242
3.343063	3.300772	3.376728	35.09584
3.200649	3.203186	3.388954	34.59774
3.273526	3.164098	3.342642	34.57035
3.225601	3.242679	3.407711	34.77968
3.200402	3.423294	3.472575	35.2614
3.335515	3.447825	3.343222	35.32764
3.368056	3.357113	3.433016	35.3968
3.323266	3.347543	3.295205	34.97655
3.391936	3.398695	3.402391	35.47298
3.282889	3.456249	3.328455	35.19869
3.286518	3.352059	3.400686	35.13673
3.190833	3.284934	3.390983	34.75948
3.168314	3.141723	3.404812	34.42729
3.188812	3.301176	3.403943	34.81892
3.283054	3.444792	3.368492	35.26155
3.245481	3.472626	3.572224	35.68578
3.39672	3.412848	3.407097	35.52468
3.22853	3.303805	3.501631	35.12515
3.417507	3.362842	3.351083	35.33829
3.315595	3.298346	3.483676	35.26434
3.302768	3.34141	3.361928	35.06422
3.142578	3.318766	3.48683	34.93754
3.238016	3.340332	3.308897	34.8043
3.29522	3.258449	3.387641	34.92253
3.254926	3.303872	3.32039	34.78667
3.147156	3.339591	3.493361	35.00737
3.246801	3.407658	3.411326	35.19473
3.357085	3.453621	3.428821	35.57468

3.356219	3.40503	3.341056	35.2746
3.393875	3.353204	3.373045	35.31356
3.251667	3.383936	3.40205	35.13321
3.334567	3.431651	3.333093	35.26805
3.379976	3.397886	3.39291	35.42432
3.348548	3.363246	3.256924	34.98246
3.072753	3.143205	3.427559	34.2713
3.207867	3.255618	3.387744	34.72553
3.197803	3.382588	3.498084	35.22249
3.303758	3.505312	3.36752	35.43705
3.205433	3.390877	3.459463	35.17284
3.303304	3.318699	3.432453	35.16996
3.258307	3.336154	3.435539	35.11648
3.437551	3.362437	3.411582	35.51354
3.276083	3.39108	3.421489	35.24474
3.305737	3.3559	3.419477	35.22826
3.202258	3.325977	3.457348	35.01934
3.247914	3.250294	3.345285	34.70862
3.361993	3.253125	3.338601	34.94966
3.235954	3.276375	3.359148	34.76981
3.156313	3.3497	3.394888	34.83416
3.21661	3.401323	3.445838	35.19033
3.359065	3.449308	3.374085	35.44988
3.472938	3.366548	3.41078	35.59817
3.337082	3.381577	3.342438	35.18448
3.253482	3.392158	3.418215	35.19051
3.304005	3.424979	3.377155	35.28298
3.325246	3.350711	3.375978	35.16444
3.310893	3.21963	3.316178	34.71563
3.181801	3.127705	3.411718	34.44123
3.11078	3.302726	3.443911	34.73907
3.259545	3.465819	3.412264	35.35184
3.334897	3.492979	3.453409	35.666
3.26503	3.363044	3.388477	35.08707
3.339846	3.272197	3.415862	35.11189
3.236448	3.356911	3.402851	35.04259
3.364344	3.412106	3.420738	35.48209
3.326483	3.378544	3.392757	35.26471
3.284868	3.329212	3.424285	35.13477
3.250471	3.231963	3.343051	34.66924
3.329617	3.203995	3.382952	34.86841
3.134041	3.310611	3.350759	34.60347
3.25076	3.359202	3.401794	35.07658
3.141506	3.390608	3.411923	34.92849
3.218136	3.435492	3.443519	35.26332
3.299262	3.363044	3.355636	35.09011
3.422044	3.338445	3.355141	35.30374
3.324503	3.396067	3.372568	35.25455
3.349125	3.387642	3.383208	35.31324
3.242841	3.284328	3.433288	34.9644

3.312295	3.21653	3.304089	34.68548
3.268288	3.197727	3.284241	34.50472
3.24445	3.261212	3.366412	34.77112
3.201268	3.470739	3.438608	35.29277
3.255214	3.486509	3.389636	35.33813
3.268825	3.383801	3.49522	35.37419
3.314564	3.322136	3.360836	35.04548
3.270928	3.341747	3.471876	35.23577
3.239294	3.424237	3.414702	35.22196
3.271134	3.364257	3.413918	35.1587
3.24445	3.292213	3.330774	34.76098
3.336258	3.18627	3.359148	34.79212
3.242717	3.208375	3.35538	34.62766
3.20168	3.224617	3.410968	34.695
3.205392	3.337299	3.429742	34.99059
3.303015	3.415274	3.393388	35.29509
3.31543	3.40658	3.395621	35.30811
3.350816	3.459552	3.37516	35.45659
3.261442	3.397347	3.496567	35.39061
3.382079	3.41278	3.358449	35.38613
3.334814	3.379757	3.440297	35.38954
3.358488	3.358731	3.336009	35.16727
3.255627	3.222595	3.408615	34.8034
3.254926	3.204938	3.289373	34.50249
3.181677	3.234659	3.379252	34.60386
3.31976	3.412174	3.361808	35.25587
3.231994	3.467976	3.457092	35.39434
3.174047	3.398628	3.383839	34.95577
3.199041	3.319912	3.452233	34.98786
3.295097	3.354889	3.466573	35.30577
3.328834	3.406513	3.466761	35.49285
3.35692	3.373692	3.445156	35.43525
3.189926	3.284261	3.466829	34.92188
3.272743	3.213497	3.421557	34.84924
3.432025	3.219832	3.305129	34.95681
3.262638	3.248205	3.450749	34.96688
3.172934	3.355967	3.332156	34.74703
3.190668	3.355698	3.48533	35.12019
3.303015	3.437379	3.346854	35.24167
3.394081	3.423226	3.38626	35.49604
3.383729	3.434077	3.347059	35.4114
3.158746	3.369177	3.436068	34.97212
3.281115	3.371738	3.412895	35.19465
3.288415	3.371064	3.386431	35.15127
3.421838	3.33056	3.401146	35.38665
3.228695	3.209858	3.330979	34.54687
3.280373	3.177778	3.431038	34.80855
3.216775	3.340265	3.36469	34.87971
3.399071	3.446208	3.394462	35.57515
3.207331	3.431448	3.424063	35.1883

3.193679	3.362774	3.46867	35.10581
3.110532	3.382588	3.447595	34.92123
3.259339	3.391619	3.41622	35.19778
3.349125	3.41487	3.425649	35.46559
3.300252	3.343432	3.435181	35.22334
3.162169	3.235804	3.419784	34.65234
3.294024	3.202579	3.375432	34.77104
3.344795	3.235198	3.406483	35.0213
3.387152	3.40476	3.308539	35.27054
3.132061	3.377196	3.456888	34.97684
3.149837	3.389934	3.333844	34.77449
3.328463	3.395999	3.41269	35.3508
3.356054	3.472963	3.324175	35.38588
3.284868	3.438457	3.463828	35.46015
3.232118	3.414398	3.38464	35.11901
3.202876	3.314318	3.487581	35.06131
3.365169	3.298346	3.354101	35.0894
3.409795	3.262088	3.314592	35.0213
3.229148	3.249822	3.337373	34.64925
3.239542	3.368974	3.422154	35.11794
3.268618	3.388721	3.43247	35.24727
3.365128	3.403817	3.300661	35.20309
3.263752	3.373355	3.402101	35.13661
3.238841	3.406513	3.396917	35.14331
3.106161	3.383531	3.456683	34.9336
3.291797	3.4084	3.355653	35.17301
3.233149	3.299222	3.439614	34.98961
3.374037	3.239781	3.333145	34.93489
3.183739	3.191796	3.475235	34.72453
3.346651	3.202714	3.367316	34.86867
3.332051	3.269838	3.395246	35.04461
3.31609	3.389058	3.346462	35.16373
3.153096	3.392562	3.450869	35.04328
3.192194	3.410085	3.333366	34.91014
3.185595	3.409141	3.420517	35.08423
3.319554	3.478287	3.38759	35.45638
3.264247	3.413387	3.411514	35.24582
3.26037	3.383734	3.396389	35.13942
3.301737	3.337704	3.425172	35.19217
3.281404	3.25946	3.390574	34.90094
3.389503	3.247261	3.347468	35.01639
3.270021	3.241129	3.399918	34.85639
3.245275	3.368705	3.370726	35.01743
3.257483	3.393708	3.442479	35.25571
3.329865	3.386227	3.34624	35.18718
3.254059	3.365066	3.407677	35.10948
3.282229	3.420194	3.418436	35.31517
3.159571	3.402604	3.493975	35.17366
3.279342	3.393708	3.348303	35.09757
3.272289	3.24807	3.487785	35.06868

3.352342	3.246857	3.378365	35.00181
3.231293	3.278667	3.434431	34.92926
3.324421	3.229874	3.432879	35.02282
3.255998	3.352463	3.405938	35.08236
3.356219	3.324562	3.347007	35.11164
3.207166	3.344241	3.378502	34.89759
3.352136	3.425585	3.382969	35.40228
3.212816	3.420463	3.337049	34.98599
3.280455	3.418711	3.377069	35.21759
3.226303	3.414061	3.402749	35.14515
3.334608	3.291809	3.384095	35.07386
3.298272	3.279745	3.382185	34.96384
3.353497	3.280486	3.374136	35.06863
3.307057	3.278869	3.430338	35.08644
3.350528	3.329549	3.359881	35.13825
3.264082	3.382251	3.387522	35.12491
3.212321	3.388182	3.418829	35.09315
3.236613	3.391956	3.420943	35.15915
3.212486	3.418711	3.348031	35.00545
3.284126	3.442164	3.406603	35.34149
3.223209	3.431314	3.482516	35.35055
3.151446	3.301446	3.473649	34.89023
3.19471	3.209588	3.398571	34.61978
3.334113	3.230009	3.389159	34.9487
3.268742	3.277589	3.420755	34.97889
3.32838	3.297267	3.364741	35.02985
3.257235	3.407861	3.423739	35.24514
3.337289	3.402806	3.388886	35.33293
3.29254	3.378881	3.326563	35.04646
3.332298	3.412982	3.44045	35.45704
3.174295	3.455104	3.427883	35.17614
3.319719	3.452543	3.405511	35.43963
3.18609	3.352665	3.399185	34.91516
3.30747	3.258786	3.352873	34.87402
3.306191	3.249148	3.398281	34.94945
3.312419	3.273343	3.401368	35.02273
3.28891	3.352463	3.387727	35.11451
3.316791	3.399369	3.35526	35.20706
3.29654	3.379083	3.38048	35.17356
3.287467	3.366953	3.342165	35.0434
3.135155	3.345993	3.399441	34.78974
3.258967	3.443849	3.39806	35.27147
3.30285	3.424439	3.456189	35.45211
3.229808	3.420059	3.434976	35.23641
3.216487	3.261616	3.493361	34.98847
3.240367	3.21963	3.407302	34.76068
3.347352	3.256494	3.404403	35.06891
3.340836	3.28635	3.379064	35.06454
3.307552	3.299491	3.398623	35.06326
3.284167	3.408602	3.393081	35.23861

3.307181	3.385688	3.422392	35.30293
3.29056	3.406647	3.326938	35.10367
3.365334	3.453621	3.406773	35.5445
3.171284	3.407726	3.395485	34.9951
3.219126	3.380836	3.40708	35.06627
3.258679	3.268423	3.36527	34.81551
3.266721	3.211071	3.403943	34.79225
3.22222	3.285137	3.394104	34.83538
3.282146	3.321462	3.410286	35.08126
3.246553	3.322608	3.444525	35.0808
3.408475	3.385823	3.321089	35.3032
3.315883	3.363852	3.449999	35.33458
3.295757	3.409748	3.359881	35.19386
3.208279	3.415611	3.434635	35.17886
3.248822	3.461506	3.371204	35.22917
3.252245	3.401054	3.428514	35.22978
3.297819	3.324764	3.358756	35.01006
3.210754	3.241061	3.455779	34.8488
3.254761	3.200962	3.381554	34.69502
3.236943	3.22091	3.445037	34.83851
3.37482	3.27341	3.354084	35.05593
3.347022	3.328538	3.400123	35.21638
3.282187	3.354283	3.329478	34.97641
3.272495	3.316475	3.340886	34.89748
3.257153	3.45234	3.324022	35.12416
3.320709	3.4733	3.423398	35.52631
3.284456	3.488059	3.397412	35.42247
3.226839	3.346869	3.412161	35.01997
3.214631	3.221787	3.389892	34.67104
3.354941	3.26101	3.342898	34.96088
3.248945	3.27833	3.393507	34.87764
3.335763	3.337771	3.391188	35.19241
3.290684	3.346869	3.448362	35.23875
3.318564	3.356978	3.418283	35.25605
3.316626	3.36237	3.347621	35.10908
3.363891	3.390338	3.353214	35.28583
3.128185	3.417093	3.49834	35.14626
3.274516	3.454969	3.390711	35.31372
3.171655	3.358596	3.463674	35.03759
3.263174	3.298346	3.386482	34.93716
3.222797	3.218147	3.445702	34.80299
3.271258	3.278869	3.429605	35.00655
3.176109	3.295246	3.434226	34.84439
3.376635	3.325168	3.395792	35.2643
3.346692	3.354957	3.397804	35.26836
3.373294	3.358124	3.327773	35.18031
3.272289	3.311285	3.42183	35.06269
3.201639	3.403952	3.422375	35.11203
3.372387	3.501942	3.356966	35.55668
3.366943	3.422957	3.368748	35.39781

3.298809	3.288169	3.435539	35.10011
3.184606	3.2447	3.430287	34.74383
3.243336	3.286215	3.445514	34.99634
3.360261	3.420665	3.390983	35.42681
3.363272	3.404626	3.400942	35.4201
3.275424	3.337569	3.51812	35.33759
3.240779	3.363852	3.352652	34.95746
3.290106	3.398897	3.409143	35.2655
3.316007	3.427674	3.339846	35.23353
3.239913	3.400986	3.442053	35.23228
3.125504	3.314588	3.455626	34.82282
3.150662	3.297739	3.443298	34.81404
3.326607	3.282441	3.32762	34.91237
3.197845	3.253933	3.413372	34.75598
3.278104	3.225763	3.471893	34.99786
3.233149	3.331975	3.419408	35.01705
3.363973	3.4146	3.429588	35.50609
3.446089	3.423024	3.306271	35.43441
3.414166	3.330964	3.396099	35.35972
3.242058	3.378814	3.410354	35.11916
3.254101	3.415139	3.427406	35.26222
3.256905	3.444658	3.362968	35.19199
3.376676	3.335615	3.316724	35.11432
3.291632	3.238702	3.4087	34.91755
3.129504	3.230009	3.457689	34.65112
3.25872	3.343702	3.439887	35.1434
3.417466	3.420194	3.421472	35.61755
3.31048	3.344308	3.412008	35.19695
3.303015	3.362572	3.395349	35.18413
3.197102	3.346937	3.445821	35.0287
3.277156	3.478152	3.364127	35.31206
3.339598	3.423765	3.43218	35.4785
3.242882	3.369177	3.372039	35.0161
3.216157	3.3003	3.469693	35.02059
3.12274	3.304546	3.393899	34.65983
3.280414	3.280419	3.451823	35.07855
3.22688	3.22738	3.372619	34.67228
3.231788	3.223404	3.442428	34.82698
3.185637	3.34323	3.380514	34.8527
3.333371	3.416083	3.401555	35.3811
3.39173	3.435155	3.338891	35.4134
3.446707	3.344645	3.37134	35.40666
3.176646	3.381914	3.458303	35.08775
3.237603	3.461506	3.403346	35.27493
3.283177	3.398762	3.369908	35.16425
3.351765	3.268221	3.320236	34.92015
3.152972	3.159178	3.437739	34.50392
3.226096	3.332312	3.415555	34.99393
3.180192	3.389395	3.460298	35.11623
3.458049	3.419452	3.317184	35.47662

3.278063	3.295313	3.490206	35.18992
3.372758	3.335817	3.358466	35.19748
3.316791	3.387777	3.486063	35.46775
3.346403	3.427203	3.321754	35.25941
3.273155	3.405434	3.390029	35.20093
3.306232	3.338849	3.324363	34.98405
3.150291	3.311555	3.379167	34.70319
3.192607	3.221584	3.389824	34.62228
3.282806	3.221989	3.356062	34.74659
3.211001	3.210734	3.414327	34.69237
3.254967	3.334064	3.370522	34.96242
3.242553	3.371064	3.450954	35.19208
3.295427	3.393573	3.373813	35.18823
3.350693	3.343702	3.366753	35.18459
3.344589	3.341815	3.384998	35.20702
3.274351	3.456384	3.406415	35.3508
3.247296	3.44122	3.349531	35.13408
3.248203	3.310881	3.430526	35.02815
3.34628	3.287293	3.290976	34.88587
3.198381	3.248003	3.411514	34.74012
3.273361	3.401323	3.373199	35.15559
3.275135	3.37659	3.470921	35.31908
3.345537	3.383666	3.390097	35.31176
3.30252	3.292213	3.399663	35.03862
3.380182	3.369514	3.412008	35.40449
3.31147	3.468987	3.439222	35.53128
3.314027	3.406445	3.409757	35.33566
3.159241	3.302052	3.377768	34.69893
3.3297	3.220708	3.354919	34.84384
3.246017	3.30704	3.386567	34.91884
3.090736	3.327123	3.429588	34.71726
3.193968	3.278869	3.399305	34.77127
3.255173	3.25946	3.420653	34.90935
3.299922	3.357113	3.422716	35.22528
3.402165	3.407321	3.408785	35.5282
3.258596	3.365874	3.457638	35.23043
3.262514	3.357854	3.390881	35.07547
3.380842	3.374635	3.325727	35.22845
3.32904	3.409882	3.469404	35.50645
3.264164	3.361494	3.315138	34.9214
3.276785	3.248811	3.345763	34.76955
3.280538	3.230548	3.390029	34.83462
3.193679	3.386295	3.400004	35.00709
3.335928	3.488329	3.423671	35.59305
3.203206	3.464067	3.388528	35.1729
3.324297	3.277117	3.370675	34.98983
3.245316	3.341006	3.399236	35.01929
3.448275	3.464876	3.441115	35.82559
3.331473	3.450993	3.440927	35.5394
3.346156	3.295583	3.396645	35.13481

3.209434	3.218282	3.465942	34.81832
3.267299	3.263908	3.325181	34.73682
3.224488	3.289652	3.418539	34.90365
3.24911	3.314588	3.382799	34.93387
3.081043	3.226032	3.445497	34.50979
3.267216	3.277521	3.416322	34.96572
3.409011	3.39573	3.39939	35.49727
3.422291	3.430437	3.429179	35.66736
3.290931	3.411972	3.40517	35.28721
3.308624	3.391012	3.40176	35.27261
3.316254	3.368705	3.360495	35.15027
3.426869	3.42909	3.294335	35.37954
3.215662	3.2879	3.380343	34.79699
3.161262	3.20359	3.375279	34.48258
3.253812	3.280149	3.430697	34.97358
3.246099	3.400582	3.407012	35.16829
3.408475	3.468178	3.328592	35.49971
3.203289	3.374164	3.492065	35.2029
3.189802	3.324697	3.424234	34.91689
3.325122	3.369311	3.409672	35.27853
3.47133	3.538267	3.407745	35.96353
3.322276	3.416554	3.420653	35.39964
3.230262	3.253866	3.403806	34.8058
3.239088	3.153382	3.327432	34.43834
3.354033	3.292954	3.380343	35.11064
3.251461	3.34943	3.393985	35.03967
3.176687	3.294572	3.450545	34.87987
3.128886	3.279004	3.395639	34.62122
3.234881	3.383194	3.438404	35.17438
3.405217	3.413859	3.397958	35.52549
3.395689	3.395123	3.41772	35.5069
3.241975	3.34195	3.499176	35.2326
3.324751	3.403076	3.357665	35.23783
3.266515	3.368907	3.447254	35.23167
3.385956	3.366279	3.282672	35.12721
3.284126	3.255551	3.356744	34.82436
3.144558	3.230211	3.398776	34.55565
3.078734	3.339523	3.404164	34.66254
3.305779	3.461169	3.393848	35.40251
3.323431	3.428146	3.447254	35.48568
3.280785	3.284328	3.381571	34.93428
3.129834	3.362505	3.45491	34.93551
3.317492	3.484892	3.402442	35.49879
3.463741	3.519397	3.414924	35.92137
3.358282	3.355698	3.323545	35.13293
3.199536	3.215452	3.453136	34.76248
3.203083	3.255618	3.399731	34.74128
3.317739	3.384879	3.375228	35.22111
3.308501	3.350913	3.352703	35.07737
3.298437	3.261279	3.397872	34.95813

3.098695	3.263638	3.504342	34.75931
3.309243	3.414398	3.351833	35.21592
3.338608	3.363246	3.410166	35.29584
3.336959	3.440142	3.390711	35.41785
3.188276	3.397886	3.428701	35.08338
3.383564	3.339591	3.363241	35.2398
3.307511	3.319036	3.389346	35.08563
3.431282	3.276982	3.283507	35.03288
3.130247	3.286215	3.442837	34.74318
3.28425	3.350037	3.35879	35.03573
3.156642	3.374366	3.414054	34.93073
3.304706	3.4733	3.30431	35.23089
3.307387	3.350845	3.428565	35.24069
3.326442	3.335615	3.36058	35.10038
3.215744	3.39573	3.457024	35.20067
3.310109	3.469189	3.416646	35.47937
3.385585	3.319305	3.358244	35.18894
3.374738	3.232974	3.314303	34.88033
3.187946	3.197727	3.370931	34.5186
3.179698	3.257977	3.39158	34.67748
3.313079	3.379825	3.336913	35.11608
3.230179	3.311488	3.396184	34.91496
3.229272	3.303535	3.419374	34.9463
3.20036	3.325303	3.465755	35.03211
3.237892	3.380364	3.442275	35.18324
3.438005	3.353474	3.423791	35.52163
3.367932	3.397347	3.390711	35.392
3.211867	3.379286	3.4375	35.11353
3.425838	3.422013	3.345149	35.47293
3.309779	3.309061	3.460076	35.22345
3.35758	3.271658	3.288606	34.87121
3.226014	3.22893	3.381213	34.69257
3.232902	3.351924	3.393166	35.00274
3.168809	3.44277	3.430679	35.14329
3.322152	3.455575	3.409194	35.45964
3.238593	3.350172	3.384589	34.9926
3.257441	3.366683	3.426945	35.16255
3.192112	3.490687	3.457399	35.35746
3.368716	3.486374	3.478151	35.77962
3.349579	3.30549	3.368561	35.10255
3.283755	3.180676	3.413901	34.7848
3.227416	3.224482	3.424456	34.78048
3.271175	3.4053	3.377905	35.16979
3.164726	3.380499	3.392978	34.91573
3.260865	3.272264	3.374205	34.84823
3.191617	3.298615	3.506593	35.04393
3.291343	3.364796	3.379678	35.1292
3.321245	3.346061	3.456052	35.32064
3.300293	3.444118	3.449828	35.47564
3.345166	3.45571	3.452233	35.60438

3.384884	3.36857	3.309358	35.18823
3.348507	3.282037	3.376063	35.06532
3.247378	3.259931	3.363428	34.7682
3.285074	3.241466	3.427013	34.9493
3.141836	3.444253	3.346325	34.90307
3.302397	3.458608	3.438046	35.48616
3.247213	3.408332	3.376882	35.12179
3.211125	3.31014	3.417465	34.91688
3.222302	3.337906	3.471313	35.1198
3.282064	3.453688	3.334867	35.2053
3.269608	3.508547	3.437449	35.52236
3.346981	3.251103	3.416561	35.0829
3.224529	3.160189	3.435573	34.65788
3.382945	3.236007	3.349872	34.9827
3.313821	3.32254	3.384453	35.09639
3.373624	3.470537	3.418965	35.62629
3.145507	3.305287	3.438319	34.80838
3.26668	3.230278	3.359967	34.73799
3.109048	3.253192	3.431771	34.60041
3.387317	3.440007	3.384589	35.51429
3.190132	3.473165	3.500318	35.40867
3.261937	3.453823	3.41119	35.32849
3.247213	3.268423	3.422648	34.91591
3.442129	3.231963	3.31686	35.03109
3.341372	3.232637	3.351066	34.88702
3.27996	3.29228	3.398094	34.986
3.172068	3.395595	3.434908	35.05649
3.337825	3.447623	3.388886	35.43211
3.285487	3.433807	3.355192	35.21376
3.349785	3.341006	3.354084	35.14901
3.172645	3.329953	3.467664	34.98584
3.253812	3.431111	3.419391	35.27899
3.330648	3.452071	3.474366	35.61308
3.35956	3.301311	3.364127	35.10554
3.231252	3.160391	3.409877	34.61683
3.258266	3.208577	3.406398	34.77367
3.2899	3.322136	3.400277	35.0778
3.234675	3.477546	3.417243	35.33399
3.222921	3.361224	3.34769	34.9018
3.137093	3.195638	3.438881	34.55142
3.192813	3.259864	3.405307	34.74031
3.261896	3.416352	3.410235	35.24437
3.310893	3.427337	3.336947	35.21527
3.217559	3.397212	3.450476	35.19356
3.326607	3.276847	3.46364	35.1976
3.192071	3.278667	3.398503	34.76492
3.439284	3.311083	3.362661	35.29805
3.360632	3.285002	3.349923	35.04116
3.278022	3.405569	3.398486	35.23036
3.303098	3.398291	3.392706	35.25664

3.400803	3.362639	3.305197	35.20098
3.252286	3.340197	3.369021	34.96669
3.343186	3.409074	3.334134	35.2398
3.127195	3.368637	3.438949	34.90825
3.22391	3.434144	3.435198	35.2548
3.203	3.413993	3.46185	35.22329
3.358611	3.175487	3.388477	34.88156
3.287219	3.168006	3.408802	34.75352
3.304211	3.272736	3.410218	35.0228
3.25773	3.439401	3.479413	35.43695
3.364179	3.391753	3.314609	35.20514
3.219827	3.278667	3.410542	34.85195
3.241274	3.272736	3.38036	34.81988
3.154085	3.328943	3.431634	34.86425
3.273403	3.400582	3.390506	35.1919
3.300335	3.383397	3.377206	35.18413
3.306191	3.415476	3.423398	35.36811
3.344382	3.282306	3.423876	35.16145
3.375851	3.281363	3.382338	35.13737
3.25773	3.293965	3.360973	34.85989
3.320503	3.292213	3.354766	34.97976
3.390328	3.376118	3.396167	35.40648
3.249151	3.352867	3.342693	34.92997
3.331515	3.363313	3.357477	35.16525
3.255255	3.324966	3.394803	34.99626
3.263834	3.409074	3.397344	35.2045
3.263999	3.426596	3.365065	35.17259
3.284167	3.464404	3.45034	35.48586
3.145589	3.285676	3.438097	34.76519
3.348259	3.164165	3.338891	34.72572
3.260906	3.239578	3.458388	34.96093
3.405175	3.390675	3.321703	35.30794
3.274104	3.451599	3.504342	35.55394
3.237892	3.361628	3.358875	34.95989
3.255503	3.207297	3.346325	34.63346
3.348919	3.294437	3.394752	35.13421
3.198051	3.406445	3.451295	35.17288
3.240903	3.499583	3.410627	35.38133
3.165922	3.416352	3.429179	35.07592
3.319224	3.264784	3.385527	34.98425
3.390328	3.173398	3.365952	34.89709
3.333041	3.25272	3.432521	35.09085
3.226756	3.338378	3.403994	34.98336
3.32904	3.432459	3.391205	35.38481
3.314522	3.428887	3.357085	35.27064
3.360302	3.319373	3.33427	35.08137
3.225931	3.294302	3.450596	34.98708
3.244532	3.491159	3.511316	35.59104
3.308831	3.505312	3.376592	35.46798
3.321492	3.458271	3.456632	35.56783

3.302644	3.326247	3.429588	35.17876
3.170459	3.184653	3.427559	34.57561
3.231953	3.253125	3.408802	34.8188
3.417177	3.415543	3.462071	35.69554
3.391936	3.495338	3.443383	35.77397
3.223663	3.383194	3.40662	35.08034
3.273568	3.204466	3.419357	34.82648
3.245522	3.245981	3.318702	34.63582
3.30219	3.342893	3.443042	35.24359
3.181512	3.494259	3.356846	35.1222
3.212734	3.431785	3.464714	35.28975
3.159983	3.283317	3.411462	34.73326
3.374366	3.191662	3.364178	34.89824
3.237521	3.277049	3.412196	34.89072
3.30017	3.355496	3.404284	35.18197
3.236201	3.45571	3.457007	35.37653
3.322235	3.424035	3.362337	35.28838
3.402329	3.341545	3.34578	35.24693
3.413795	3.34505	3.267325	35.1081
3.175078	3.331571	3.503387	35.07282
3.190792	3.446073	3.357204	35.0379
3.19005	3.412915	3.430833	35.12478
3.321327	3.327932	3.377086	35.10849
3.250348	3.139836	3.450886	34.70332
3.257648	3.223808	3.448754	34.89825
3.230674	3.402739	3.483471	35.30648
3.385667	3.463528	3.403943	35.60444
3.332009	3.349161	3.417874	35.26747
3.297612	3.230143	3.357102	34.79907
3.234758	3.18809	3.41078	34.68704
3.224282	3.342017	3.381707	34.93717
3.362901	3.487116	3.356011	35.50142
3.301861	3.454227	3.420107	35.43618
3.272083	3.309466	3.40355	35.01829
3.189472	3.136736	3.44959	34.56058
3.277857	3.167804	3.305538	34.50678
3.297984	3.324832	3.397889	35.09615
3.335845	3.457462	3.377206	35.42376
3.244079	3.415072	3.442752	35.27372
3.362076	3.429359	3.361604	35.38554
3.350157	3.299828	3.3873	35.13241
3.42522	3.335817	3.387846	35.37645
3.238181	3.447623	3.36411	35.16003
3.181925	3.415746	3.464663	35.18719
3.176852	3.31762	3.369567	34.75355
3.298066	3.210801	3.347331	34.7364
3.240408	3.191527	3.400277	34.68395
3.304995	3.419048	3.485944	35.51008
3.252245	3.543255	3.503728	35.70524
3.349703	3.361224	3.371135	35.23033

3.429715	3.3034	3.386533	35.31252
3.374037	3.220102	3.383293	35.00152
3.257524	3.335143	3.440382	35.12314
3.210919	3.41588	3.366446	35.0361
3.280043	3.459888	3.358313	35.26572
3.346651	3.351519	3.339556	35.13337
3.274186	3.240387	3.344194	34.74202
3.155405	3.211677	3.423944	34.59388
3.26833	3.277319	3.454705	35.05165
3.340217	3.450588	3.466761	35.61413
3.365087	3.471548	3.381912	35.5288
3.330236	3.419317	3.352873	35.27486
3.294148	3.313509	3.411326	35.09239
3.278022	3.392225	3.370061	35.13902
3.412022	3.472895	3.369976	35.60828
3.266185	3.435964	3.416782	35.31096
3.240243	3.340736	3.401504	35.01257
3.107274	3.177239	3.372636	34.30111
3.265938	3.201703	3.383617	34.7256
3.204485	3.359944	3.399799	34.97264
3.348301	3.467639	3.408871	35.5425
3.223581	3.415948	3.467937	35.28588
3.394122	3.365874	3.411991	35.42698
3.281816	3.265525	3.393592	34.9217
3.465308	3.298615	3.339096	35.27616
3.256988	3.373894	3.431379	35.18703
3.182461	3.401458	3.383208	34.97898
3.169263	3.327864	3.460639	34.95851
3.337577	3.281026	3.311506	34.89803
3.269319	3.255753	3.377547	34.83792
3.21162	3.259999	3.422853	34.8201
3.153384	3.411567	3.486472	35.16333
3.399979	3.421541	3.387778	35.50857
3.507088	3.423631	3.391	35.75442
3.335391	3.37814	3.372585	35.23919
3.301118	3.378881	3.379167	35.18026
3.35226	3.409748	3.345149	35.28521
3.284951	3.43037	3.392126	35.28584
3.375109	3.383329	3.324704	35.23269
3.177883	3.232435	3.435454	34.7136
3.08888	3.204871	3.359574	34.29275
3.280331	3.262155	3.433749	34.9989
3.257111	3.440344	3.452233	35.37822
3.327473	3.432998	3.424882	35.45621
3.242882	3.31297	3.526987	35.23203
3.285569	3.268558	3.399509	34.94948
3.288003	3.21781	3.344194	34.72286
3.451698	3.376657	3.315309	35.36504
3.235252	3.424102	3.463418	35.31936
3.28227	3.413859	3.350759	35.15341

3.210795	3.24834	3.356642	34.648
3.364386	3.226167	3.305794	34.8242
3.195741	3.213565	3.384316	34.59956
3.209434	3.409411	3.405597	35.10432
3.202505	3.444927	3.480385	35.33039
3.393256	3.453823	3.360291	35.50436
3.330277	3.382857	3.355874	35.20178
3.460235	3.314116	3.386294	35.40218
3.278269	3.373894	3.423944	35.21731
3.325039	3.39856	3.378161	35.27341
3.259998	3.350509	3.349889	34.96427
3.280661	3.222393	3.389892	34.81676
3.18906	3.191055	3.346257	34.45249
3.212692	3.307848	3.454688	34.9967
3.208485	3.476737	3.465345	35.38014
3.356549	3.459686	3.455387	35.64487
3.363478	3.372277	3.365184	35.27161
3.276991	3.30805	3.426724	35.0766
3.341495	3.267951	3.426502	35.12949
3.356178	3.330425	3.429043	35.30377
3.326153	3.440142	3.371749	35.35275
3.329617	3.404693	3.33456	35.20148
3.307882	3.241668	3.386635	34.91132
3.271794	3.187685	3.371545	34.68135
3.27464	3.218754	3.450186	34.92749
3.186173	3.452677	3.372772	35.07629
3.342238	3.479365	3.449487	35.6437
3.289446	3.435964	3.325096	35.16132
3.364592	3.330695	3.440092	35.34692
3.335556	3.365133	3.325164	35.10741
3.372222	3.40375	3.429469	35.50014
3.25608	3.440614	3.369976	35.19667
3.37878	3.395123	3.377138	35.38117
3.231829	3.199749	3.418965	34.72403
3.316048	3.167669	3.319947	34.62152
3.203742	3.230413	3.435266	34.76532
3.290642	3.455845	3.378809	35.32487
3.173057	3.454969	3.516074	35.366
3.239954	3.397415	3.359148	35.04326
3.304418	3.297065	3.429469	35.11856
3.441758	3.376455	3.351424	35.42184
3.263628	3.341073	3.456768	35.1853
3.352342	3.353541	3.334048	35.1382
3.284786	3.355967	3.436392	35.21958
3.316213	3.279813	3.35908	34.9527
3.252863	3.226639	3.396269	34.7792
3.204279	3.225358	3.31657	34.49587
3.120637	3.22091	3.414753	34.51794
3.156972	3.403884	3.396252	34.95708
3.335763	3.464336	3.405938	35.50144

3.368386	3.451734	3.415879	35.56696
3.407402	3.359674	3.348866	35.30442
3.241398	3.379623	3.411445	35.12187
3.453348	3.475591	3.351697	35.66458
3.382945	3.439266	3.407847	35.55397
3.350157	3.294033	3.333571	35.00224
3.183863	3.142262	3.447884	34.55667
3.240325	3.247598	3.31698	34.62423
3.198422	3.382386	3.406824	35.02383
3.335103	3.44951	3.441746	35.54588
3.164231	3.392023	3.512186	35.20054
3.232077	3.339793	3.475985	35.15552
3.271175	3.332245	3.367725	34.98777
3.386162	3.336423	3.420176	35.36307
3.227334	3.391888	3.395025	35.08203
3.359106	3.387103	3.503592	35.59715
3.239294	3.32409	3.37504	34.91622
3.349002	3.216395	3.344995	34.85491
3.226921	3.23513	3.312887	34.5587
3.304541	3.276106	3.428565	35.07102
3.125875	3.365739	3.389415	34.7907
3.266721	3.429224	3.402186	35.26547
3.306026	3.486374	3.400942	35.47368
3.405918	3.393371	3.373096	35.42785
3.301984	3.369783	3.426451	35.26566
3.259462	3.414465	3.416834	35.24935
3.362365	3.424642	3.432726	35.53139
3.404598	3.407254	3.255816	35.19885
3.275506	3.199142	3.355124	34.67861
3.138207	3.181148	3.364519	34.35956
3.207496	3.349498	3.452642	35.07194
3.254884	3.531798	3.335736	35.31858
3.291508	3.429629	3.434124	35.3904
3.265896	3.350037	3.401283	35.08852
3.168892	3.2972	3.483284	34.94016
3.260163	3.316677	3.450613	35.11091
3.410454	3.390945	3.417601	35.52979
3.268123	3.416352	3.392552	35.21932
3.301737	3.304681	3.45184	35.17827
3.309573	3.206151	3.334236	34.72276
3.377212	3.207634	3.397685	35.01267
3.227911	3.279273	3.341483	34.71993
3.281033	3.3621	3.397736	35.14025
3.102902	3.410556	3.393558	34.84753
3.277279	3.473098	3.345371	35.26026
3.36554	3.42855	3.384384	35.44117
3.362035	3.390945	3.371971	35.32412
3.316626	3.391686	3.513004	35.53486
3.326937	3.430774	3.358381	35.30475
3.338443	3.399302	3.486216	35.54064

3.350569	3.249553	3.304396	34.84207
3.289199	3.123122	3.392348	34.62371
3.235706	3.25636	3.373932	34.75783
3.131237	3.432055	3.432044	35.04067
3.254678	3.477815	3.363496	35.26079
3.254018	3.391551	3.415947	35.1854
3.300706	3.322001	3.357903	35.00847
3.237974	3.353609	3.47249	35.19099
3.300829	3.401728	3.402783	35.28123
3.282889	3.357585	3.449573	35.24779
3.3264	3.331369	3.408035	35.19478
3.343929	3.327056	3.372431	35.14582
3.262556	3.173533	3.378297	34.64496
3.326607	3.173196	3.399526	34.83072
3.190462	3.344713	3.408649	34.92802
3.290725	3.35462	3.362234	35.06745
3.267299	3.429696	3.457161	35.38799
3.271423	3.452475	3.317372	35.14112
3.237851	3.385351	3.434652	35.17739
3.371727	3.452475	3.325062	35.37729
3.265896	3.387642	3.387522	35.14067
3.38942	3.410758	3.403482	35.49625
3.218508	3.29538	3.448618	34.96888
3.33502	3.181755	3.366514	34.79564
3.347641	3.195975	3.368407	34.85848
3.346115	3.420194	3.420346	35.45906
3.18011	3.464067	3.408717	35.16654
3.278599	3.425383	3.387863	35.25172
3.174418	3.330627	3.404488	34.85304
3.30648	3.369311	3.38418	35.18202
3.292168	3.418778	3.403005	35.30007
3.236036	3.33184	3.477963	35.15112
3.232159	3.3065	3.436358	34.99624
3.268948	3.27806	3.438114	35.01834
3.34727	3.240522	3.341875	34.89706
3.302149	3.230548	3.39187	34.88591
3.299592	3.289854	3.438387	35.11174
3.143238	3.363111	3.449573	34.95448
3.338856	3.422283	3.366583	35.33018
3.319307	3.423024	3.42304	35.41251
3.311058	3.452273	3.412144	35.43461
3.201268	3.39081	3.393797	35.01998
3.288415	3.359539	3.409621	35.17678
3.382491	3.435559	3.407762	35.54469
3.37317	3.360752	3.374767	35.28856
3.2176	3.190381	3.444031	34.72725
3.273733	3.167602	3.390455	34.68302
3.258926	3.290528	3.382117	34.90123
3.410083	3.419587	3.383344	35.5167
3.179863	3.39856	3.409212	35.02383

3.208156	3.39263	3.411445	35.07762
3.168314	3.371468	3.397804	34.91438
3.266721	3.394517	3.407353	35.20087
3.266927	3.402536	3.444747	35.30063
3.308295	3.329751	3.442957	35.22801
3.132969	3.276645	3.423484	34.68588
3.296911	3.242342	3.362064	34.83507
3.418167	3.224617	3.352242	35.04
3.34727	3.282037	3.362013	35.03189
3.213063	3.329212	3.440416	35.01302
3.185513	3.39108	3.314541	34.8128
3.281486	3.413656	3.400873	35.26084
3.394163	3.450319	3.297609	35.3616
3.253523	3.441692	3.487154	35.44969
3.131979	3.425855	3.38701	34.93026
3.248327	3.386969	3.377837	35.07959
3.369953	3.369109	3.366719	35.2822
3.314069	3.299896	3.383685	35.04573
3.248615	3.205882	3.376541	34.68138
3.222879	3.217541	3.419221	34.74393
3.279094	3.387508	3.411445	35.22155
3.399484	3.479365	3.350742	35.55295
3.26536	3.362909	3.39037	35.09163
3.227292	3.34195	3.40089	34.98556
3.111027	3.351722	3.399373	34.74935
3.299922	3.469661	3.352873	35.31866
3.224447	3.36857	3.41801	35.07499
3.260947	3.283115	3.402323	34.93362
3.197473	3.198334	3.441865	34.69589
3.369541	3.25737	3.328183	34.95267
3.306232	3.313779	3.412332	35.1216
3.426251	3.395999	3.330484	35.38488
3.183863	3.351587	3.448839	35.01652
3.172892	3.404019	3.323527	34.83315
3.271175	3.385621	3.391188	35.15581
3.35061	3.450858	3.327978	35.33395
3.220034	3.460832	3.370112	35.16235
3.183698	3.373018	3.434925	35.03259
3.167489	3.279947	3.45121	34.82923
3.404969	3.269164	3.373744	35.15557
3.340093	3.266401	3.329547	34.911
3.224117	3.225898	3.401061	34.7252
3.313697	3.341882	3.357034	35.07846
3.230963	3.434616	3.392978	35.17893
3.40864	3.399302	3.319657	35.32991
3.2866	3.399436	3.40621	35.2526
3.229231	3.415746	3.441729	35.24048
3.134948	3.405771	3.46792	35.06977
3.333412	3.376118	3.358517	35.19968
3.247914	3.267816	3.426877	34.92536

3.231582	3.261481	3.390233	34.79566
3.147321	3.187685	3.445565	34.57102
3.3516	3.267682	3.381383	35.05233
3.320956	3.294167	3.427832	35.14481
3.351023	3.345724	3.408666	35.28139
3.32042	3.357854	3.368339	35.15281
3.265278	3.417161	3.413355	35.26036
3.324833	3.445871	3.388272	35.39853
3.326359	3.475928	3.382543	35.45507
3.198917	3.47458	3.350946	35.10433
3.204856	3.318294	3.393746	34.86914
3.193927	3.266806	3.380224	34.70307
3.312625	3.231626	3.36972	34.86274
3.352631	3.263975	3.38036	35.04424
3.356797	3.320047	3.422495	35.26811
3.265608	3.410758	3.392501	35.20147
3.26569	3.386901	3.378382	35.11861
3.314728	3.306366	3.416714	35.13355
3.374614	3.441827	3.377666	35.47535
3.217229	3.462719	3.410763	35.24924
3.120637	3.421474	3.453188	35.04059
3.265979	3.341343	3.45092	35.17824
3.279218	3.214508	3.401913	34.82265
3.228241	3.207903	3.396713	34.68536
3.228406	3.239309	3.400532	34.76275
3.310315	3.252046	3.373727	34.91111
3.294024	3.374568	3.368561	35.13212
3.418951	3.383262	3.376251	35.44114
3.289446	3.360348	3.381707	35.11976
3.339433	3.444792	3.419101	35.49552
3.266144	3.420935	3.361791	35.15774
3.354858	3.444994	3.363496	35.40809
3.190297	3.410489	3.360666	34.96657
3.294231	3.339321	3.41119	35.14871
3.224034	3.267749	3.421949	34.86222
3.356591	3.304411	3.434721	35.2602
3.225024	3.274893	3.409553	34.8529
3.39272	3.336086	3.425018	35.38726
3.30318	3.395999	3.423791	35.31979
3.285281	3.336019	3.401419	35.10055
3.249275	3.351722	3.40812	35.07081
3.226509	3.378073	3.373335	35.00258
3.215786	3.46528	3.436835	35.3087
3.207619	3.471548	3.375415	35.17024
3.199659	3.262829	3.512186	34.99549
3.289116	3.237624	3.401283	34.89347
3.278929	3.228728	3.420397	34.89354
3.246594	3.284665	3.398503	34.89727
3.326277	3.2879	3.434567	35.15747
3.275259	3.359944	3.393916	35.11455

3.333907	3.373692	3.36706	35.21414
3.379893	3.368637	3.329069	35.22057
3.309367	3.403008	3.355175	35.19859
3.266474	3.443242	3.377206	35.24096
3.322688	3.432864	3.376251	35.3391
3.190462	3.362505	3.355584	34.85089
3.280414	3.311151	3.354152	34.93216
3.155983	3.255888	3.472848	34.79877
3.328463	3.268962	3.41344	35.07463
3.230015	3.308657	3.423637	34.96845
3.342485	3.357854	3.414157	35.30126
3.305531	3.3652	3.408171	35.22342
3.336134	3.345387	3.381571	35.18884
3.199742	3.349767	3.417447	34.97861
3.248657	3.47768	3.370965	35.26366
3.144558	3.431314	3.445565	35.09775
3.222055	3.413319	3.383566	35.09229
3.178749	3.184181	3.434789	34.60852
3.258349	3.183237	3.394343	34.69207
3.290354	3.218552	3.394598	34.83985
3.34166	3.237422	3.391273	34.98604
3.317327	3.3497	3.461782	35.33256
3.310687	3.383464	3.374358	35.20069
3.310398	3.351048	3.363309	35.10501
3.375975	3.433942	3.338209	35.3748
3.3808	3.454969	3.382748	35.52873
3.179038	3.418644	3.370385	34.98104
3.279218	3.375444	3.418948	35.21185
3.198298	3.26384	3.389551	34.72654
3.335226	3.285406	3.381025	35.0545
3.258472	3.265929	3.383225	34.84887
3.283012	3.267614	3.389568	34.92009
3.214053	3.344713	3.399594	34.95981
3.409465	3.435694	3.402237	35.59189
3.270887	3.351182	3.427747	35.15981
3.329081	3.388856	3.370965	35.24529
3.223374	3.368772	3.440382	35.12201
3.331143	3.431044	3.348082	35.29201
3.207702	3.414398	3.404147	35.10827
3.262514	3.332649	3.39719	35.03415
3.17512	3.192201	3.470938	34.69717
3.257895	3.234524	3.380292	34.77251
3.19867	3.24989	3.415401	34.75338
3.400144	3.31762	3.38759	35.28127
3.295674	3.363179	3.435045	35.25621
3.404887	3.34168	3.374495	35.31561
3.285941	3.345319	3.391393	35.10041
3.332587	3.395797	3.370163	35.26638
3.224034	3.443579	3.389653	35.17611
3.269897	3.493788	3.3858	35.37777

3.161509	3.361965	3.377751	34.83487
3.204279	3.233176	3.381435	34.65481
3.330978	3.272804	3.345712	34.94042
3.277527	3.297604	3.382816	34.95891
3.356673	3.327325	3.411445	35.25959
3.234716	3.380768	3.407268	35.10063
3.340506	3.354417	3.378843	35.21219
3.34529	3.341478	3.332292	35.09255
3.316626	3.385216	3.380599	35.23116
3.194958	3.38616	3.405784	35.02223
3.264989	3.375377	3.399987	35.13912
3.222178	3.360146	3.368066	34.94238
3.25938	3.238568	3.366105	34.75358
3.243542	3.221113	3.4493	34.86271
3.193803	3.231154	3.461577	34.80274
3.210383	3.266334	3.406466	34.79541
3.395194	3.390945	3.419903	35.50145
3.285899	3.442029	3.410081	35.35268
3.431819	3.384071	3.339829	35.3914
3.250595	3.39081	3.408717	35.16048
3.272743	3.413387	3.343051	35.11469
3.311388	3.394314	3.349514	35.17162
3.354858	3.329212	3.373727	35.17727
3.199783	3.260605	3.345643	34.62669
3.256658	3.258381	3.37047	34.8005
3.207001	3.353272	3.437671	35.04637
3.336752	3.398156	3.398009	35.34154
3.351353	3.369851	3.434022	35.39032
3.345455	3.301176	3.458712	35.28124
3.226344	3.303872	3.419647	34.94123
3.383646	3.446747	3.317935	35.37524
3.286724	3.449442	3.423535	35.40011
3.318729	3.362572	3.332343	35.08071
3.114162	3.242813	3.479174	34.69256
3.169593	3.303468	3.343665	34.65008
3.245769	3.264649	3.408188	34.87288
3.252616	3.289922	3.370027	34.85967
3.260205	3.309196	3.441865	35.07551
3.218054	3.33548	3.42142	34.9961
3.342815	3.427	3.409877	35.44383
3.422168	3.442838	3.385544	35.59878
3.393338	3.35125	3.353675	35.26576
3.1884	3.356844	3.421284	34.97767
3.313161	3.435357	3.410849	35.39938
3.259297	3.338849	3.345984	34.9287
3.376882	3.237287	3.288691	34.83844
3.180027	3.267951	3.436017	34.79719
3.121751	3.348756	3.359745	34.67966
3.233231	3.407995	3.474775	35.30455
3.375398	3.441086	3.370385	35.45952

3.253523	3.272736	3.426058	34.9466
3.317698	3.341545	3.33352	35.03505
3.292663	3.416015	3.452966	35.40436
3.340836	3.46683	3.393456	35.49069
3.298437	3.334671	3.33248	34.97562
3.331432	3.247666	3.369516	34.9385
3.168273	3.231222	3.386482	34.58284
3.237191	3.26721	3.408853	34.86117
3.161509	3.26411	3.382594	34.63147
3.2501	3.29902	3.410098	34.96169
3.245151	3.358865	3.43663	35.13976
3.22189	3.388249	3.498067	35.2875
3.344424	3.463123	3.36145	35.42044
3.389874	3.384542	3.371374	35.36969
3.319801	3.381914	3.397275	35.26735
3.263628	3.455104	3.391393	35.2917
3.255379	3.413185	3.386635	35.17158
3.244408	3.283654	3.336555	34.75481
3.294602	3.223876	3.330826	34.72132
3.205557	3.249351	3.403124	34.74041
3.269691	3.340602	3.380616	35.03099
3.237768	3.424979	3.375296	35.13407
3.35193	3.344915	3.445276	35.36167
3.291178	3.341815	3.360154	35.03589
3.329659	3.388721	3.418539	35.35029
3.243501	3.480713	3.401572	35.32595
3.373624	3.475524	3.520302	35.8588
3.176274	3.325573	3.406756	34.851
3.348672	3.197255	3.395928	34.92372
3.167077	3.240185	3.40621	34.64297
3.201969	3.264649	3.406841	34.77415
3.164767	3.269164	3.422938	34.73787
3.233809	3.332784	3.364195	34.89952
3.240573	3.381981	3.400004	35.1002
3.360055	3.422957	3.381383	35.41038
3.231705	3.37504	3.441405	35.15617
3.349992	3.360213	3.354459	35.19228
3.399525	3.452475	3.449368	35.70992
3.432685	3.473165	3.345984	35.60159
3.293694	3.314049	3.411172	35.09224
3.22853	3.238028	3.330604	34.6073
3.255998	3.222932	3.41755	34.82449
3.338155	3.417161	3.341056	35.26162
3.227375	3.454362	3.527004	35.50736
3.270144	3.382184	3.390199	35.14387
3.279672	3.256764	3.454876	35.03187
3.381007	3.374366	3.396951	35.38398
3.36055	3.45389	3.435266	35.59694
3.270969	3.447421	3.464987	35.45189
3.263834	3.337839	3.351782	34.94908

3.222261	3.214643	3.398776	34.69153
3.361334	3.279947	3.365133	35.0649
3.26136	3.287091	3.419221	34.98017
3.164314	3.277521	3.356403	34.60965
3.074197	3.233446	3.453341	34.52818
3.307057	3.358124	3.368936	35.12548
3.28029	3.349565	3.417311	35.15402
3.309779	3.381375	3.406347	35.26409
3.199453	3.342017	3.456666	35.0468
3.303716	3.382992	3.392689	35.2245
3.386327	3.454632	3.380429	35.53501
3.46007	3.319844	3.299723	35.22503
3.239583	3.194627	3.342148	34.5618
3.215992	3.209858	3.402732	34.67601
3.130618	3.402065	3.402664	34.90949
3.328504	3.453958	3.3777	35.40112
3.27266	3.431314	3.353214	35.17593
3.243707	3.27186	3.436204	34.9454
3.136186	3.278128	3.436102	34.72375
3.371232	3.44331	3.429656	35.58489
3.411733	3.450184	3.47706	35.79216
3.26305	3.356978	3.361246	35.00992
3.286972	3.210464	3.435812	34.9049
3.184564	3.232233	3.405375	34.66199
3.406825	3.302389	3.35729	35.19631
3.329081	3.290326	3.339982	34.96206
3.157715	3.286619	3.449419	34.81853
3.151198	3.316407	3.461679	34.89623
3.289735	3.374164	3.39569	35.18118
3.322523	3.380027	3.363172	35.1946
3.342939	3.390069	3.402749	35.34775
3.161839	3.347004	3.462617	34.98846
3.259792	3.383464	3.453955	35.26346
3.368304	3.338715	3.299928	35.06606
3.416146	3.272467	3.339743	35.11288
3.192483	3.202445	3.402596	34.60809
3.17116	3.387036	3.459957	35.09057
3.285487	3.484824	3.411718	35.44894
3.415899	3.487453	3.291692	35.4774
3.202093	3.359404	3.403857	34.97511
3.27864	3.314385	3.359864	34.94785
3.176687	3.39081	3.446776	35.08208
3.325947	3.538604	3.415282	35.66282
3.366035	3.396269	3.377206	35.35596
3.400267	3.227448	3.422444	35.16056
3.155488	3.160728	3.407626	34.44696
3.351229	3.268153	3.382117	35.05415
3.300912	3.308185	3.415265	35.10415
3.282394	3.303468	3.367623	34.94915
3.154663	3.30832	3.381332	34.71041

3.144764	3.398088	3.357818	34.83366
3.211537	3.422687	3.437023	35.20668
3.340918	3.43347	3.358944	35.34245
3.256575	3.358663	3.480947	35.26122
3.267876	3.375916	3.368441	35.07762
3.31576	3.352935	3.347826	35.087
3.313945	3.24524	3.328097	34.80438
3.373707	3.194357	3.265586	34.68709
3.255049	3.219765	3.363428	34.69713
3.212445	3.428753	3.390642	35.1205
3.309326	3.460428	3.418351	35.46223
3.301242	3.416015	3.389074	35.2834
3.271258	3.346398	3.323766	34.92277
3.263587	3.378881	3.466437	35.28903
3.150126	3.499044	3.476173	35.32498
3.300458	3.458001	3.413389	35.42668
3.258926	3.277723	3.407217	34.92812
3.361828	3.231424	3.365628	34.96095
3.231705	3.258314	3.439973	34.89778
3.315718	3.380768	3.419784	35.30514
3.261772	3.316879	3.436	35.08291
3.304871	3.291876	3.314217	34.85617
3.161922	3.303737	3.462549	34.89387
3.294602	3.382925	3.39407	35.20744
3.243872	3.351519	3.435539	35.11851
3.285157	3.38097	3.419238	35.23755
3.258596	3.401593	3.452574	35.29747
3.431365	3.446814	3.419579	35.70202
3.345909	3.368637	3.404556	35.31133
3.371809	3.224684	3.292374	34.80784
3.274269	3.204264	3.448839	34.89205
3.261648	3.360078	3.348269	34.98526
3.223746	3.401391	3.397327	35.1
3.162623	3.398493	3.352072	34.86103
3.270392	3.349295	3.437909	35.17683
3.17413	3.327999	3.389551	34.81399
3.296416	3.517038	3.40708	35.53315
3.267216	3.443107	3.449982	35.40143
3.196896	3.337839	3.351475	34.80203
3.185059	3.195031	3.407575	34.58653
3.383646	3.258044	3.371152	35.07896
3.28392	3.350037	3.430202	35.19118
3.297695	3.373153	3.318156	35.02683
3.25608	3.276443	3.392825	34.88762
3.238346	3.318631	3.415487	34.99065
3.278723	3.369648	3.293585	34.92394
3.334154	3.391619	3.370198	35.26074
3.17281	3.377062	3.434994	35.01777
3.218714	3.333323	3.453324	35.0626
3.28825	3.345926	3.462191	35.26161

3.420147	3.367424	3.409808	35.48251
3.394452	3.26694	3.370573	35.12077
3.297984	3.227852	3.421557	34.93583
3.226138	3.340197	3.382065	34.93803
3.353085	3.434548	3.379644	35.41668
3.330566	3.437244	3.34578	35.29928
3.226096	3.318025	3.36486	34.85183
3.092385	3.27651	3.344484	34.42408
3.223993	3.416487	3.344552	35.01814
3.322895	3.510973	3.457212	35.68742
3.309656	3.367963	3.38655	35.1912
3.220487	3.157897	3.430952	34.63392
3.237438	3.190314	3.327398	34.51542
3.283507	3.313846	3.429043	35.1086
3.408434	3.431314	3.315292	35.38992
3.239583	3.27213	3.462958	34.99548
3.208279	3.267075	3.353282	34.67613
3.28128	3.294302	3.41344	35.02687
3.255627	3.404828	3.362013	35.1
3.284621	3.426057	3.423296	35.34385
3.163035	3.409343	3.464527	35.13158
3.183574	3.353204	3.485466	35.09952
3.278888	3.34013	3.371766	35.03072
3.471288	3.299626	3.341466	35.29663
3.231541	3.261953	3.365304	34.74209
3.294395	3.300165	3.35983	34.95113
3.243171	3.341478	3.465465	35.16046
3.455698	3.464876	3.233973	35.38884
3.341949	3.302457	3.365219	35.07192
3.26536	3.343634	3.32305	34.90226
3.098572	3.381779	3.437773	34.87182
3.258637	3.551948	3.376592	35.4602
3.270186	3.398156	3.484835	35.38585
3.277651	3.240455	3.332872	34.72498
3.208815	3.130603	3.474962	34.64495
3.280208	3.198873	3.451943	34.90003
3.300994	3.446342	3.440552	35.46176
3.341867	3.416757	3.384367	35.36357
3.248615	3.258584	3.390711	34.82762
3.243749	3.282576	3.372312	34.82921
3.232613	3.382184	3.407643	35.09994
3.300912	3.463191	3.42154	35.45684
3.232984	3.459888	3.36793	35.18384
3.218178	3.378544	3.46289	35.18123
3.340629	3.293224	3.407899	35.14218
3.315883	3.267682	3.40442	35.0246
3.40567	3.301311	3.285059	35.03347
3.348466	3.295583	3.418453	35.18755
3.289817	3.399975	3.348508	35.13463
3.318729	3.409545	3.426468	35.38927

3.376346	3.419452	3.354152	35.37879
3.298809	3.347071	3.300695	34.93404
3.336175	3.393236	3.395656	35.32437
3.060504	3.441153	3.449521	34.94411
3.269361	3.533617	3.429878	35.56009
3.210011	3.254809	3.377837	34.70679
3.198463	3.195233	3.459036	34.72882
3.163324	3.263975	3.381912	34.63365
3.348424	3.374501	3.442121	35.4118
3.263463	3.457665	3.460145	35.44729
3.362695	3.308859	3.410286	35.22984
3.257194	3.268558	3.428719	34.95131
3.358034	3.350306	3.353419	35.18593
3.242264	3.402604	3.423416	35.2002
3.216198	3.421541	3.415401	35.16708
3.198463	3.439738	3.412554	35.16186
3.275836	3.373018	3.424149	35.21052
3.258637	3.306096	3.379252	34.92838
3.36818	3.25946	3.398759	35.1086
3.287838	3.257168	3.39569	34.92118
3.329287	3.420328	3.407916	35.39537
3.334855	3.352598	3.402613	35.24783
3.310315	3.355091	3.369635	35.1275
3.338361	3.354013	3.424132	35.30565
3.263215	3.368705	3.368833	35.05252
3.218879	3.397415	3.416152	35.12182
3.275588	3.509962	3.394479	35.44457
3.218384	3.429427	3.460434	35.28759
3.140104	3.144621	3.368885	34.29337
3.30483	3.200827	3.397753	34.83965
3.203083	3.268693	3.382816	34.73289
3.362035	3.456047	3.382816	35.4902
3.264082	3.367087	3.41726	35.15678
3.343351	3.257977	3.384998	35.02097
3.321905	3.226167	3.355192	34.83933
3.395153	3.34195	3.401214	35.35335
3.157096	3.376455	3.378314	34.85814
3.238428	3.494664	3.38597	35.31124
3.12703	3.429898	3.462327	35.09298
3.358034	3.257842	3.313109	34.89557
3.2753	3.226841	3.430151	34.90281
3.261195	3.257033	3.422239	34.92068
3.243707	3.413791	3.382458	35.13825
3.329452	3.426798	3.391052	35.373
3.371521	3.367357	3.32733	35.19566
3.398329	3.367627	3.346053	35.29582
3.219786	3.337771	3.366378	34.88453
3.2534	3.391147	3.335822	35.00794
3.289116	3.496753	3.400464	35.45835
3.291673	3.45106	3.362524	35.28105

3.262391	3.16558	3.404369	34.68423
3.207991	3.123931	3.381929	34.42511
3.281775	3.306837	3.433987	35.10029
3.335763	3.442097	3.379729	35.39549
3.347558	3.375309	3.414856	35.35205
3.245811	3.261414	3.428582	34.91049
3.267257	3.228661	3.419153	34.86515
3.305242	3.320114	3.356266	35.01068
3.405959	3.402941	3.398094	35.50353
3.166541	3.461304	3.446248	35.2129
3.124968	3.433268	3.446503	35.06124
3.241439	3.220708	3.380821	34.70747
3.318894	3.214845	3.34844	34.79322
3.170459	3.253799	3.432896	34.73849
3.288209	3.397145	3.364997	35.16098
3.191452	3.437986	3.444883	35.2134
3.498715	3.432864	3.280165	35.51392
3.413053	3.308253	3.431771	35.38563
3.428107	3.348352	3.331235	35.28638
3.248945	3.356237	3.500403	35.28177
3.271217	3.437851	3.325386	35.12622
3.190998	3.48725	3.367248	35.15037
3.362158	3.223202	3.329342	34.86434
3.111481	3.133838	3.408376	34.29356
3.100015	3.284934	3.482943	34.76197
3.245522	3.4115	3.396645	35.16823
3.372057	3.397617	3.4508	35.53301
3.284415	3.304411	3.357716	34.93397
3.32805	3.209656	3.411872	34.94061
3.259215	3.213632	3.408768	34.79198
3.27101	3.447151	3.382168	35.27028
3.288456	3.482196	3.335975	35.28405
3.240408	3.436301	3.371135	35.1555
3.259009	3.371401	3.401913	35.12156
3.188565	3.193683	3.383225	34.538
3.266762	3.234996	3.338686	34.70195
3.2395	3.328606	3.405511	34.99318
3.281651	3.401256	3.443315	35.3269
3.227375	3.432122	3.370794	35.11712
3.327968	3.333525	3.360137	35.09817
3.403855	3.363044	3.261818	35.11367
3.439943	3.441962	3.426877	35.72613
3.224777	3.416015	3.413304	35.16917
3.247007	3.414196	3.450596	35.29536
3.218549	3.330897	3.378024	34.89226
3.263174	3.18654	3.354629	34.623
3.214177	3.167467	3.349002	34.46184
3.223292	3.408332	3.480606	35.2963
3.140846	3.465684	3.492202	35.26678
3.42423	3.352126	3.352669	35.33303

3.295097	3.230683	3.426519	34.94656
3.359766	3.345319	3.404966	35.29154
3.295179	3.341545	3.417294	35.169
3.311347	3.361359	3.328677	35.0539
3.215414	3.368098	3.36868	34.94633
3.330937	3.401997	3.38036	35.29863
3.194793	3.278599	3.420176	34.81812
3.222714	3.252383	3.351833	34.6724
3.248285	3.27341	3.428224	34.94135
3.246017	3.329819	3.34017	34.86719
3.353415	3.402334	3.401828	35.39547
3.269361	3.410219	3.374256	35.1686
3.295138	3.323349	3.401896	35.09545
3.34133	3.375175	3.297063	35.08054
3.421673	3.393371	3.434158	35.59584
3.229107	3.413859	3.405528	35.15692
3.204237	3.420126	3.417447	35.14231
3.190132	3.278262	3.401163	34.76562
3.31609	3.218417	3.317338	34.72688
3.242388	3.245172	3.447748	34.9094
3.276207	3.433335	3.411241	35.31501
3.164932	3.437649	3.477333	35.22563
3.295427	3.358394	3.348252	35.0554
3.367025	3.249485	3.412673	35.11469
3.397834	3.288506	3.388204	35.21389
3.239212	3.329751	3.459207	35.11248
3.313821	3.426461	3.371835	35.29605
3.196319	3.409748	3.409808	35.08559
3.349373	3.29437	3.352856	35.04343
3.278599	3.232098	3.344484	34.73418
3.182007	3.275095	3.417413	34.77646
3.074486	3.402671	3.485671	34.96958
3.321121	3.480039	3.402459	35.49616
3.357787	3.434683	3.426246	35.52917
3.341908	3.383464	3.360376	35.23839
3.22853	3.291135	3.404693	34.88545
3.334608	3.417026	3.371545	35.32024
3.371191	3.455508	3.346598	35.42984
3.362158	3.402267	3.346803	35.29411
3.133381	3.220169	3.362473	34.42986
3.185637	3.152304	3.421864	34.5256
3.296705	3.272467	3.391614	34.96512
3.316502	3.483746	3.434516	35.56426
3.348136	3.499786	3.444389	35.69011
3.169345	3.377736	3.523576	35.20539
3.285404	3.309601	3.400686	35.04145
3.304335	3.256225	3.405051	34.97567
3.404268	3.352935	3.374904	35.33977
3.228117	3.402065	3.469728	35.26936
3.306356	3.399975	3.46983	35.43611

3.250842	3.25454	3.387164	34.81589
3.405835	3.188359	3.321294	34.86606
3.148559	3.176094	3.356488	34.35358
3.17813	3.350037	3.376797	34.84304
3.174831	3.495877	3.430219	35.27158
3.319307	3.497292	3.342693	35.39922
3.408681	3.408669	3.387539	35.49893
3.30582	3.353002	3.375262	35.12541
3.238841	3.38926	3.418692	35.1532
3.341949	3.474715	3.384657	35.49113
3.37284	3.327392	3.418061	35.30956
3.343558	3.209386	3.324397	34.78264
3.166087	3.142599	3.397685	34.40875
3.121833	3.251709	3.394479	34.54357
3.174088	3.466897	3.405801	35.15319
3.272825	3.501538	3.46746	35.5797
3.303881	3.418846	3.444338	35.41622
3.259009	3.331099	3.429179	35.09305
3.217435	3.318766	3.440501	34.99992
3.424601	3.395864	3.401828	35.53699
3.405464	3.385486	3.398981	35.46622
3.239418	3.313644	3.31802	34.76895
3.227829	3.266738	3.42154	34.86741
3.266391	3.256629	3.387931	34.85614
3.332257	3.242948	3.311267	34.80261
3.262597	3.279543	3.3238	34.7577
3.184358	3.388788	3.457672	35.11827
3.170913	3.448903	3.432606	35.16551
3.345001	3.43347	3.379405	35.39612
3.346898	3.394045	3.37961	35.3145
3.338155	3.449375	3.423109	35.5115
3.231169	3.49682	3.418351	35.3709
3.356714	3.380499	3.3607	35.26499
3.254637	3.236411	3.339556	34.68043
3.263999	3.169826	3.336333	34.54825
3.129133	3.192605	3.427201	34.50184
3.147404	3.295785	3.360171	34.62085
3.133175	3.413117	3.43866	35.01796
3.249605	3.421811	3.38759	35.17991
3.248574	3.327527	3.546477	35.31893
3.347558	3.40968	3.408922	35.41424
3.203907	3.326651	3.45878	35.02756
3.29918	3.341208	3.406193	35.15274
3.389627	3.352193	3.405511	35.37306
3.353415	3.412174	3.365542	35.33763
3.236737	3.338917	3.37475	34.94241
3.2534	3.223674	3.379252	34.73668
3.273774	3.21936	3.390728	34.7969
3.245646	3.321327	3.371954	34.91731
3.254307	3.401593	3.470512	35.32731

3.167283	3.446477	3.431651	35.15018
3.325947	3.426326	3.385186	35.35147
3.421714	3.318227	3.345899	35.23859
3.361581	3.463258	3.394598	35.53075
3.261483	3.491901	3.40355	35.39406
3.380718	3.342489	3.400004	35.32031
3.230674	3.248474	3.395707	34.7772
3.365499	3.221652	3.299212	34.80237
3.136846	3.219765	3.406552	34.53295
3.281569	3.374568	3.381588	35.13337
3.133835	3.43347	3.403448	34.98691
3.285899	3.422755	3.382662	35.25057
3.194215	3.367559	3.465141	35.10973
3.241975	3.273612	3.395928	34.85737
3.231211	3.28945	3.453733	34.99488
3.33634	3.359741	3.383771	35.2255
3.309532	3.398695	3.437637	35.36985
3.427241	3.312296	3.362064	35.27306
3.290972	3.238837	3.364792	34.82038
3.129339	3.170702	3.39482	34.38358
3.227292	3.276578	3.387471	34.81325
3.241398	3.447758	3.429725	35.31084
3.253441	3.48442	3.385697	35.32107
3.339186	3.399032	3.432453	35.4241
3.243831	3.36547	3.419016	35.1128
3.396927	3.342017	3.430083	35.42051
3.3914	3.442029	3.364741	35.48424
3.297654	3.410826	3.416441	35.32405
3.363066	3.260268	3.355056	35.00361
3.189926	3.174948	3.414651	34.56873
3.268247	3.215317	3.351032	34.68916
3.24445	3.379016	3.39349	35.08795
3.263752	3.478826	3.467784	35.5109
3.149507	3.410826	3.426041	35.02108
3.295385	3.327595	3.410627	35.12437
3.251709	3.390608	3.436341	35.22288
3.260782	3.39883	3.41431	35.21253
3.191081	3.362033	3.469387	35.10008
3.261195	3.355293	3.449232	35.19459
3.31312	3.307377	3.393933	35.08243
3.455162	3.21963	3.365696	35.13941
3.305449	3.185664	3.337322	34.67569
3.262679	3.21808	3.446606	34.89203
3.06104	3.387305	3.406552	34.73356
3.316914	3.512523	3.400515	35.55374
3.276125	3.491698	3.326136	35.25635
3.385997	3.400178	3.364042	35.37937
3.274351	3.40375	3.437739	35.30419
3.348136	3.414465	3.474553	35.56949
3.308666	3.454699	3.37782	35.35962

3.437139	3.377668	3.326853	35.36066
3.334649	3.107015	3.464356	34.84535
3.143321	3.148934	3.365287	34.30197
3.264288	3.297267	3.430151	35.03274
3.210424	3.496079	3.372789	35.22427
3.273361	3.428011	3.420466	35.31731
3.136227	3.358663	3.415316	34.85451
3.188276	3.302052	3.462259	34.94719
3.277733	3.410085	3.403414	35.25038
3.272495	3.373288	3.404215	35.16021
3.218714	3.364189	3.406347	35.02736
3.279589	3.302861	3.482925	35.19384
3.276785	3.242476	3.369362	34.80731
3.403237	3.218012	3.367589	35.02646
3.330195	3.250631	3.31241	34.8174
3.248739	3.24989	3.425445	34.88483
3.159612	3.41642	3.415265	35.03184
3.403031	3.413791	3.363667	35.44557
3.251997	3.404828	3.342165	35.04866
3.395607	3.452138	3.313484	35.40345
3.247832	3.385081	3.500932	35.34357
3.31081	3.434751	3.357494	35.27624
3.278022	3.422013	3.494197	35.47563
3.3337	3.347206	3.39673	35.22065
3.322688	3.117731	3.396321	34.69385
3.291756	3.16248	3.343512	34.60858
3.195989	3.352935	3.441729	35.03043
3.303057	3.467774	3.407558	35.44098
3.323555	3.39081	3.37724	35.2512
3.256039	3.349161	3.377206	35.0124
3.170583	3.401728	3.476719	35.15809
3.300293	3.387373	3.428241	35.30434
3.355518	3.34013	3.42079	35.3055
3.29819	3.342152	3.371886	35.07761
3.331267	3.338243	3.426417	35.26065
3.268165	3.259257	3.375654	34.83892
3.380883	3.256966	3.366429	35.06023
3.26602	3.231896	3.375961	34.77506
3.326813	3.313172	3.392689	35.12232
3.145301	3.366211	3.427201	34.91685
3.359973	3.403278	3.332599	35.26048
3.273155	3.4177	3.440024	35.33708
3.31844	3.454227	3.380872	35.38664
3.196112	3.378073	3.452915	35.11013
3.346321	3.437312	3.357562	35.35964
3.252863	3.343567	3.430918	35.11068
3.441346	3.242342	3.352396	35.12978
3.348383	3.235737	3.374512	34.96041
3.27897	3.325371	3.344126	34.93818
3.279713	3.401997	3.403619	35.23747

3.262968	3.475187	3.375637	35.29972
3.18176	3.316879	3.368475	34.76027
3.293571	3.310409	3.341653	34.93198
3.226633	3.35098	3.439768	35.08888
3.216198	3.385823	3.459411	35.18521
3.249028	3.328538	3.478969	35.17451
3.331473	3.275162	3.396576	35.0579
3.33069	3.279206	3.429588	35.13722
3.321534	3.205208	3.391393	34.87184
3.260865	3.295583	3.398537	34.95243
3.28858	3.386699	3.411872	35.24146
3.282889	3.402065	3.350418	35.12822
3.340217	3.413926	3.376592	35.33677
3.320173	3.408669	3.339096	35.19944
3.287054	3.379892	3.446111	35.29811
3.290849	3.414802	3.405494	35.29393
3.250966	3.40476	3.376217	35.12073
3.366654	3.294639	3.380343	35.14192
3.266474	3.182766	3.378723	34.67465
3.255957	3.211812	3.349838	34.65201
3.29555	3.305085	3.331678	34.90285
3.402453	3.483274	3.414242	35.70686
3.222096	3.444792	3.415742	35.23157
3.246636	3.376118	3.467801	35.2489
3.264783	3.371401	3.375381	35.07616
3.309078	3.421137	3.410081	35.35768
3.22886	3.421205	3.388886	35.13605
3.238676	3.395325	3.469847	35.27797
3.13037	3.20878	3.434226	34.55528
3.404557	3.247464	3.377768	35.11602
3.361416	3.231559	3.381571	34.99521
3.374614	3.271725	3.357579	35.05944
3.246842	3.336828	3.410439	35.03799
3.310315	3.376253	3.331047	35.08939
3.32174	3.396673	3.354152	35.20956
3.456152	3.434077	3.290618	35.44636
3.234304	3.412578	3.449641	35.26195
3.111357	3.462247	3.405392	35.00494
3.248244	3.377129	3.463231	35.24464
3.3118	3.313981	3.339556	34.97507
3.340464	3.229941	3.388664	34.96136
3.220941	3.216867	3.389926	34.67415
3.192442	3.24524	3.463077	34.83385
3.273609	3.456317	3.369652	35.26863
3.35692	3.410826	3.386209	35.38755
3.286064	3.357517	3.387778	35.11945
3.227952	3.336019	3.392348	34.95535
3.18044	3.377399	3.429588	35.02338
3.319884	3.519195	3.379815	35.52956
3.272949	3.414937	3.398691	35.2402

3.285817	3.295785	3.413082	35.03925
3.106161	3.177037	3.467784	34.50631
3.296416	3.253462	3.343512	34.81773
3.272165	3.257438	3.433016	34.96913
3.416888	3.382049	3.326887	35.32603
3.242841	3.350037	3.503115	35.26079
3.351188	3.375916	3.361041	35.24363
3.324173	3.434751	3.420994	35.44432
3.371892	3.417026	3.30564	35.25766
3.22391	3.388249	3.362831	34.99618
3.181347	3.401526	3.404625	35.02353
3.161509	3.333256	3.347741	34.70646
3.376759	3.261347	3.364332	35.0562
3.271464	3.292483	3.38996	34.95007
3.31411	3.265053	3.459752	35.13597
3.230674	3.380364	3.419511	35.11768
3.300912	3.41588	3.451005	35.41782
3.352507	3.37322	3.3356	35.18499
3.418992	3.366481	3.364843	35.37959
3.163035	3.362572	3.427883	34.94916
3.183822	3.45571	3.397275	35.13136
3.222096	3.416217	3.382185	35.0957
3.227375	3.276982	3.413423	34.87107
3.283425	3.223943	3.435812	34.92662
3.22523	3.207162	3.417413	34.72242
3.249481	3.240859	3.429725	34.87607
3.317904	3.342826	3.354084	35.08327
3.337825	3.349632	3.398895	35.23971
3.28062	3.404491	3.377513	35.18782
3.297076	3.388586	3.397685	35.23314
3.362117	3.413589	3.298342	35.2128
3.361334	3.428618	3.383157	35.42943
3.307717	3.39209	3.321959	35.09847
3.236737	3.38097	3.403533	35.09732
3.195411	3.303737	3.360922	34.74487
3.262803	3.266266	3.353146	34.7933
3.284497	3.252653	3.393882	34.90005
3.335268	3.378477	3.396781	35.29257
3.260163	3.39728	3.442752	35.26998
3.411156	3.359472	3.374699	35.36868
3.259875	3.34943	3.389346	35.04792
3.302232	3.346061	3.322777	34.98761
3.16419	3.380229	3.462771	35.0666
3.284538	3.438727	3.325591	35.15771
3.094654	3.289113	3.454961	34.69819
3.209599	3.249418	3.337305	34.60546
3.284662	3.254675	3.443656	35.01368
3.286353	3.302322	3.3895	35.00314
3.265608	3.35098	3.447612	35.19127
3.293571	3.368705	3.370948	35.12353

3.319636	3.348352	3.379388	35.15448
3.388595	3.37167	3.345984	35.28322
3.26899	3.424439	3.398981	35.25296
3.245563	3.454295	3.396235	35.26101
3.279342	3.402604	3.321191	35.05773
3.221725	3.338782	3.35347	34.86275
3.274929	3.307579	3.340033	34.88148
3.227581	3.259123	3.447663	34.90734
3.210053	3.341141	3.400055	34.94426
3.239913	3.367963	3.451755	35.18128
3.344094	3.423765	3.385936	35.3872
3.249316	3.383329	3.431992	35.19223
3.395277	3.399436	3.390677	35.45629
3.259421	3.352126	3.454824	35.19602
3.330318	3.398897	3.335344	35.19205
3.294932	3.400717	3.396986	35.25345
3.260617	3.320518	3.398145	35.00556
3.167654	3.214306	3.400618	34.5754
3.224694	3.238905	3.414174	34.78358
3.220322	3.267614	3.348423	34.69302
3.325782	3.351654	3.421131	35.26642
3.313532	3.400649	3.410763	35.32411
3.336381	3.381105	3.47856	35.4796
3.359766	3.348419	3.349241	35.17646
3.401051	3.399302	3.366037	35.41474
3.31811	3.403952	3.305743	35.11168
3.269897	3.367222	3.364622	35.05468
3.138867	3.324292	3.4109	34.77546
3.197638	3.301715	3.339505	34.69848
3.263463	3.27058	3.388954	34.88248
3.272248	3.2817	3.385663	34.91881
3.264494	3.342623	3.479532	35.24036
3.166128	3.411028	3.405239	35.01237
3.348589	3.393034	3.423927	35.4129
3.383069	3.370592	3.353589	35.28541
3.341537	3.365403	3.438455	35.36883
3.233273	3.406041	3.377274	35.08715
3.317451	3.40476	3.493293	35.52214
3.187204	3.384273	3.384879	34.95543
3.316048	3.244363	3.395894	34.95532
3.13833	3.210195	3.475559	34.66617
3.162334	3.266806	3.45907	34.80641
3.244037	3.305826	3.424132	34.994
3.265484	3.317823	3.414736	35.04659
3.322029	3.366413	3.424677	35.29825
3.321905	3.34505	3.437568	35.27945
3.298272	3.427203	3.455796	35.44728
3.390781	3.499112	3.295512	35.45632
3.416311	3.38926	3.402715	35.50636
3.369623	3.395932	3.319657	35.23722

3.197968	3.24187	3.382202	34.6617
3.220735	3.284732	3.335822	34.7038
3.146167	3.320855	3.413099	34.78872
3.311305	3.395258	3.3622	35.20125
3.218879	3.364324	3.477691	35.18404
3.31811	3.416689	3.420056	35.38952
3.300912	3.368705	3.486609	35.39251
3.403361	3.379083	3.359404	35.36107
3.312048	3.386092	3.39187	35.24771
3.233685	3.341343	3.41992	35.03982
3.215827	3.343904	3.441524	35.05362
3.17314	3.312768	3.368902	34.73336
3.307923	3.228661	3.332002	34.76349
3.213063	3.223202	3.385749	34.66165
3.158127	3.290259	3.407489	34.73569
3.150992	3.327258	3.433016	34.85682
3.388843	3.433201	3.399799	35.53601
3.313615	3.326921	3.417925	35.17872
3.332298	3.360281	3.37313	35.19457
3.277692	3.419452	3.427047	35.32246
3.350734	3.42855	3.406534	35.45723
3.33436	3.360415	3.347502	35.14333
3.401133	3.250361	3.259277	34.85575
3.119688	3.20696	3.398964	34.45083
3.227622	3.310544	3.365201	34.83955
3.172727	3.359	3.419766	34.9448
3.305036	3.392967	3.402732	35.27116
3.298272	3.364931	3.44502	35.28754
3.291467	3.380027	3.481033	35.38442
3.251214	3.4053	3.466147	35.31911
3.363602	3.459012	3.421489	35.58469
3.347929	3.438794	3.411855	35.48513
3.276496	3.386497	3.374853	35.13363
3.209929	3.224348	3.399867	34.68817
3.197226	3.220573	3.388681	34.62768
3.330195	3.287428	3.331934	34.94056
3.228983	3.264582	3.374273	34.76185
3.174955	3.316542	3.36411	34.73511
3.280538	3.34013	3.447748	35.20049
3.296128	3.382184	3.447595	35.32621
3.455162	3.348419	3.360291	35.40923
3.331226	3.395325	3.386823	35.2988
3.245811	3.455238	3.432333	35.34256
3.327679	3.428146	3.390779	35.37147
3.281775	3.299828	3.374887	34.95572
3.344465	3.200221	3.317252	34.74895
3.224199	3.296594	3.389074	34.85376
3.147486	3.299222	3.404983	34.72654
3.249028	3.398897	3.39112	35.13626
3.309326	3.398358	3.429452	35.35077

3.222137	3.346734	3.400669	34.98426
3.309821	3.426394	3.373659	35.29115
3.238139	3.473839	3.416765	35.33241
3.384347	3.432122	3.415759	35.55872
3.426746	3.356372	3.367742	35.38078
3.331185	3.299087	3.403278	35.12424
3.209063	3.230885	3.405733	34.7134
3.185348	3.219226	3.354237	34.52343
3.133959	3.206286	3.38759	34.45569
3.253688	3.291	3.331115	34.77927
3.188235	3.351856	3.498562	35.1354
3.236861	3.472963	3.406142	35.30447
3.232489	3.393371	3.433016	35.17962
3.403526	3.366548	3.33398	35.27842
3.295509	3.389664	3.520473	35.50059
3.284909	3.437986	3.326256	35.15836
3.273444	3.308792	3.394053	34.99902
3.304253	3.246655	3.297132	34.71856
3.323926	3.186742	3.383344	34.81909
3.211744	3.301446	3.364571	34.78355
3.207537	3.380499	3.438932	35.10985
3.251997	3.393506	3.446776	35.25267
3.296623	3.420059	3.364997	35.22949
3.287013	3.377938	3.386959	35.16439
3.359148	3.392562	3.413509	35.41218
3.240202	3.402941	3.457962	35.27197
3.328339	3.460562	3.41801	35.50335
3.319513	3.336423	3.385732	35.14199
3.398494	3.311016	3.36515	35.21414
3.209682	3.197525	3.434584	34.70489
3.248038	3.214912	3.355243	34.65329
3.145589	3.221787	3.439001	34.62745
3.230262	3.312027	3.324227	34.75896
3.201433	3.385149	3.428378	35.08359
3.250265	3.418037	3.387215	35.17228
3.202794	3.367627	3.449999	35.09553
3.336794	3.386227	3.357153	35.2262
3.290024	3.433942	3.434158	35.39666
3.324214	3.434953	3.300951	35.18234
3.251585	3.288372	3.412298	34.94646
3.232489	3.241668	3.337987	34.64006
3.351806	3.185933	3.32733	34.7558
3.276331	3.382453	3.359148	35.09009
3.191039	3.397549	3.444099	35.12236
3.215249	3.350576	3.402732	34.98211
3.271588	3.274151	3.467306	35.0794
3.257771	3.422283	3.448567	35.33215
3.396803	3.492237	3.400481	35.68401
3.232984	3.444927	3.503251	35.44704
3.263298	3.390406	3.401351	35.17127

3.327761	3.278532	3.380036	35.02098
3.387936	3.300502	3.373267	35.18581
3.248285	3.247127	3.33485	34.67968
3.199659	3.21498	3.402186	34.6503
3.185554	3.239915	3.393643	34.6553
3.329576	3.353137	3.323596	35.06467
3.209228	3.371468	3.377922	34.96038
3.363726	3.394314	3.439683	35.48326
3.132061	3.376522	3.447322	34.95445
3.323472	3.410691	3.403158	35.35117
3.390451	3.424305	3.435471	35.59808
3.450007	3.336625	3.345149	35.33906
3.258885	3.221584	3.357238	34.69596
3.262226	3.217743	3.373403	34.73022
3.235747	3.341613	3.379593	34.95674
3.344754	3.430168	3.353402	35.33149
3.203825	3.404693	3.40176	35.07335
3.140558	3.332312	3.410985	34.79688
3.223581	3.306231	3.400805	34.89914
3.299468	3.45881	3.432675	35.46846
3.329576	3.483476	3.378877	35.47059
3.237892	3.357248	3.434277	35.1152
3.178749	3.246857	3.444201	34.76616
3.368799	3.284193	3.34653	35.04983
3.382986	3.268962	3.400805	35.16624
3.243047	3.234794	3.35526	34.68589
3.128556	3.235467	3.384726	34.50143
3.186791	3.331032	3.455541	34.99262
3.377212	3.430303	3.352686	35.40121
3.323678	3.424305	3.382048	35.33523
3.326359	3.365807	3.401385	35.25545
3.112347	3.329751	3.507753	34.9412
3.279177	3.380634	3.396662	35.17437
3.375274	3.41487	3.316263	35.28357
3.445099	3.305355	3.374801	35.32479
3.172892	3.177307	3.396576	34.49711
3.246264	3.244498	3.419971	34.85566
3.244491	3.397752	3.298308	34.92087
3.354281	3.40011	3.350622	35.28052
3.197102	3.385351	3.429452	35.07691
3.126122	3.335278	3.425922	34.80446
3.179161	3.413926	3.435744	35.11392
3.347806	3.507064	3.417328	35.64613
3.301448	3.440479	3.451295	35.47342
3.303881	3.288372	3.371681	34.972
3.183038	3.205747	3.446776	34.69127
3.289735	3.283587	3.360069	34.90521
3.377913	3.276712	3.37741	35.12093
3.334402	3.224145	3.314933	34.77419
3.110945	3.199816	3.365662	34.34326

3.219704	3.367694	3.413628	35.05311
3.347723	3.430505	3.393916	35.42732
3.371644	3.421474	3.383003	35.43602
3.276661	3.38461	3.432351	35.25561
3.102366	3.315059	3.447526	34.75554
3.301201	3.429022	3.447612	35.43977
3.364798	3.360011	3.330007	35.17075
3.377955	3.284867	3.367947	35.11816
3.204237	3.21033	3.397446	34.63977
3.224364	3.337502	3.418743	35.00847
3.254967	3.434751	3.382713	35.20927
3.402783	3.432729	3.34578	35.44733
3.210713	3.245037	3.408785	34.75463
3.19405	3.337636	3.42715	34.96085
3.168809	3.458001	3.493787	35.3146
3.367685	3.510569	3.411531	35.68458
3.296705	3.412578	3.407643	35.30657
3.317451	3.27523	3.437671	35.11724
3.225189	3.218821	3.431106	34.77777
3.37482	3.314116	3.438523	35.32961
3.26437	3.273208	3.385732	34.88316
3.384059	3.263031	3.321549	34.98229
3.096468	3.265929	3.450954	34.6427
3.20201	3.395595	3.33751	34.90898
3.193019	3.355563	3.375518	34.88489
3.306769	3.400784	3.336009	35.14614
3.201515	3.367896	3.464237	35.12446
3.211125	3.393438	3.459957	35.19197
3.227581	3.344847	3.420056	35.03444
3.428148	3.252316	3.353078	35.12422
3.348754	3.249957	3.417737	35.08684
3.22556	3.26721	3.336094	34.67663
3.271712	3.410489	3.404096	35.23959
3.272743	3.427944	3.349258	35.16009
3.3118	3.330425	3.383617	35.10739
3.262391	3.315396	3.319367	34.82596
3.227169	3.391619	3.451909	35.20547
3.211414	3.519667	3.392484	35.32109
3.340712	3.367829	3.353675	35.18693
3.245481	3.232637	3.430151	34.85027
3.296416	3.262829	3.366924	34.88942
3.193102	3.294302	3.471961	34.96201
3.352507	3.331167	3.381247	35.19284
3.270474	3.258247	3.437654	34.97734
3.319678	3.235198	3.338635	34.818
3.236943	3.297874	3.42524	34.96352
3.229808	3.434481	3.358449	35.1006
3.240325	3.396943	3.406671	35.14696
3.291756	3.395258	3.435454	35.31869
3.217064	3.35098	3.364417	34.90318

3.280496	3.375646	3.43484	35.24984
3.318193	3.334469	3.423603	35.21765
3.331391	3.282239	3.395911	35.07174
3.361787	3.252248	3.374392	35.02557
3.291014	3.360685	3.329922	35.01068
3.259833	3.443377	3.411036	35.30071
3.249069	3.433335	3.313586	35.0421
3.298891	3.347476	3.37475	35.09705
3.215703	3.433403	3.396389	35.15036
3.22886	3.492911	3.421284	35.36371
3.195824	3.478961	3.482516	35.39486
3.256534	3.293224	3.40783	34.95812
3.215332	3.189977	3.516892	34.88074
3.262886	3.291337	3.394803	34.9394
3.270227	3.275702	3.412809	34.96064
3.311017	3.326584	3.463299	35.27152
3.181801	3.273612	3.43293	34.8067
3.359395	3.281363	3.347724	35.02568
3.262226	3.364122	3.366514	35.03526
3.304046	3.427203	3.401777	35.34178
3.151941	3.417093	3.358517	34.89244
3.248409	3.358124	3.381793	35.02534
3.241356	3.354552	3.416714	35.07848
3.318688	3.351789	3.3873	35.17722
3.307841	3.256225	3.41448	35.00396
3.36818	3.231693	3.349991	34.94123
3.247543	3.221652	3.423876	34.81703
3.339186	3.392427	3.417806	35.37763
3.337	3.445062	3.330689	35.29744
3.27695	3.334267	3.354698	34.97633
3.173594	3.281026	3.412315	34.75988
3.25109	3.374635	3.415691	35.14144
3.247955	3.518993	3.402988	35.42249
3.273444	3.44769	3.430697	35.3829
3.136722	3.216328	3.491997	34.71201
3.316584	3.198334	3.365287	34.7889
3.39837	3.269164	3.365014	35.12205
3.293364	3.450723	3.392279	35.34908
3.295592	3.25892	3.452795	35.06685
3.287095	3.258044	3.344569	34.80968
3.269196	3.275634	3.425274	34.98549
3.367438	3.368233	3.304106	35.13786
3.356756	3.41433	3.430066	35.49076
3.056668	3.425787	3.373727	34.73637
3.240985	3.385216	3.519637	35.3698
3.277568	3.307781	3.357119	34.92506
3.329535	3.241668	3.369464	34.92112
3.242264	3.208982	3.321498	34.5539
3.233891	3.299087	3.432726	34.97587
3.239253	3.403008	3.370539	35.07886

3.468566	3.439131	3.32675	35.56357
3.334237	3.376792	3.415811	35.32825
3.304046	3.336356	3.329495	34.98504
3.154003	3.33393	3.44016	34.89362
3.318853	3.571695	3.353999	35.58566
3.225478	3.459619	3.425359	35.29242
3.262102	3.261347	3.398009	34.87911
3.146084	3.137949	3.504632	34.58872
3.336216	3.275904	3.410507	35.10035
3.280249	3.394921	3.431481	35.2841
3.466587	3.44021	3.377018	35.67153
3.301242	3.236209	3.419852	34.9575
3.319636	3.290124	3.397531	35.06682
3.267051	3.3003	3.380633	34.93712
3.332133	3.427405	3.375569	35.34633
3.207207	3.439738	3.423791	35.20556
3.174047	3.401728	3.384606	34.96423
3.227499	3.32746	3.414924	34.98501
3.334567	3.304681	3.371084	35.07347
3.369953	3.312835	3.338192	35.09675
3.194421	3.304142	3.416373	34.86485
3.282682	3.379892	3.41726	35.22546
3.265484	3.406108	3.460758	35.3403
3.447862	3.4084	3.295665	35.38311
3.422827	3.354148	3.401828	35.44189
3.251007	3.332851	3.436341	35.09505
3.126494	3.444388	3.391239	34.96804
3.318688	3.505312	3.433271	35.61348
3.204361	3.386969	3.44386	35.12783
3.229643	3.218956	3.418982	34.76129
3.155611	3.240522	3.426706	34.66345
3.267711	3.333727	3.432606	35.12532
3.280373	3.410489	3.425462	35.30525
3.363354	3.352665	3.439904	35.39185
3.286023	3.229537	3.406415	34.88024
3.341619	3.233041	3.368083	34.92566
3.260741	3.366144	3.381503	35.06921
3.266927	3.41305	3.373659	35.16817
3.227705	3.448701	3.404863	35.2286
3.270021	3.360415	3.392126	35.10021
3.233355	3.270849	3.369788	34.77531
3.30615	3.229806	3.375381	34.85698
3.276661	3.230683	3.388392	34.82286
3.358735	3.366279	3.383702	35.28862
3.303675	3.397482	3.424422	35.32549
3.332669	3.362774	3.320714	35.08621
3.367932	3.346128	3.377939	35.25206
3.289529	3.342489	3.299996	34.9022
3.22057	3.380162	3.374648	34.99703
3.144187	3.483678	3.423603	35.16343

3.166376	3.416824	3.424694	35.06814
3.253894	3.195031	3.386345	34.69064
3.240284	3.179868	3.366225	34.58371
3.191163	3.251238	3.383634	34.67044
[truncated: 352,709 more chars]
